# iRNA-AI: identifying the adenosine to inosine editing sites in RNA sequences

**DOI:** 10.18632/oncotarget.13758

**Published:** 2016-12-01

**Authors:** Wei Chen, Pengmian Feng, Hui Yang, Hui Ding, Hao Lin, Kuo-Chen Chou

**Affiliations:** ^1^ Department of Physics, School of Sciences, and Center for Genomics and Computational Biology, North China University of Science and Technology, Tangshan, Tangshan, China; ^2^ Hebei Province Key Laboratory of Occupational Health and Safety for Coal Industry, School of Public Health, North China University of Science and Technology, Tangshan, China; ^3^ Key Laboratory for Neuro-Information of Ministry of Education, School of Life Science and Technology, Center for Informational Biology, University of Electronic Science and Technology of China, Chengdu, China; ^4^ Gordon Life Science Institute, Belmont, Massachusetts, United States of America

**Keywords:** A-to-I editing, nucleotide chemical properties, nucleotide density distribution, PseKNC, web-server

## Abstract

Catalyzed by adenosine deaminase (ADAR), the adenosine to inosine (A-to-I) editing in RNA is not only involved in various important biological processes, but also closely associated with a series of major diseases. Therefore, knowledge about the A-to-I editing sites in RNA is crucially important for both basic research and drug development. Given an uncharacterized RNA sequence that contains many adenosine (A) residues, can we identify which one of them can be of A-to-I editing, and which one cannot? Unfortunately, so far no computational method whatsoever has been developed to address such an important problem based on the RNA sequence information alone. To fill this empty area, we have proposed a predictor called iRNA-AI by incorporating the chemical properties of nucleotides and their sliding occurrence density distribution along a RNA sequence into the general form of pseudo nucleotide composition (PseKNC). It has been shown by the rigorous jackknife test and independent dataset test that the performance of the proposed predictor is quite promising. For the convenience of most experimental scientists, a user-friendly web-server for iRNA-AI has been established at http://lin.uestc.edu.cn/server/iRNA-AI/, by which users can easily get their desired results without the need to go through the mathematical details.

## INTRODUCTION

**Figure 1:**
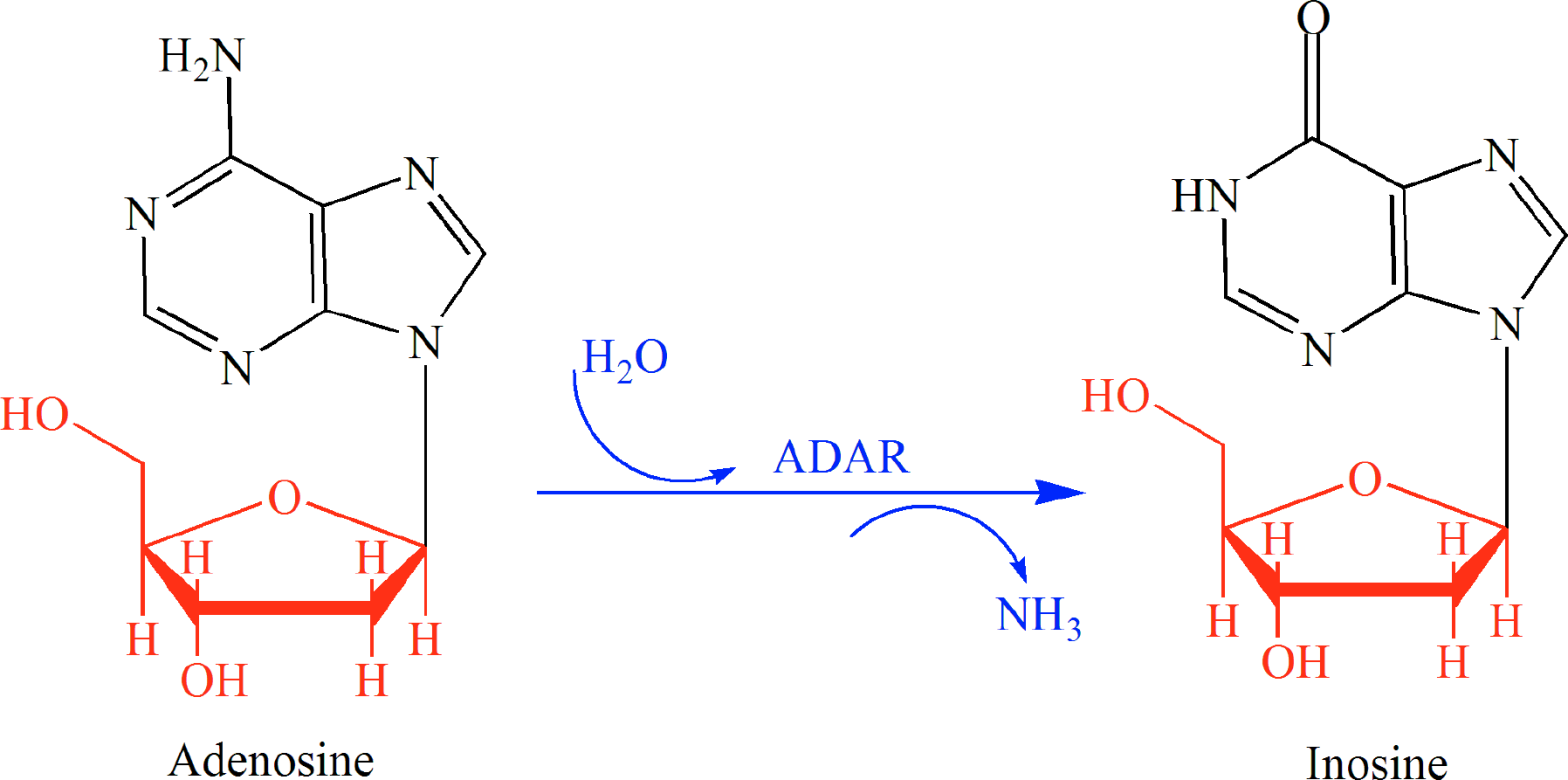
An illustration to show the most common type of RNA editing, a modification from adenosine (A) to inosine (I) or the “A-to-I editing” that is catalyzed by the adenosine deaminase (ADAR) See the text for further explanation.

RNA editing is a post-transcriptional modification that changes the genomic template through the insertion, deletion, deamination or substitution of nucleotides within the edited RNA molecule. Among the five types of RNA editing reported so far, the modification from adenosine to inosine, the so-called “A-to-I” editing, is the most common one [1, 2]. This type of editing is catalyzed by the enzyme called “adenosine deaminase” (ADAR) [3] as shown in Figure 1. The concrete process is: adenosine is deaminated to inosine, followed by decoding to become guanosine (G) due to the polymerase enzyme and translational machinery [4].

Besides altering the genetic code that can expand the transcriptome and proteome, the A-to-I editing may also involve in various important biological processes ranging from alternative splicing [5], nonsense-mediated mRNA decay [6] to gene expression and translation [7]. RNA secondary and teritary structures may also be affected by A-to-I editing [8]. In addition, RNA A-to-I editing was also found to be closely associated with the formation of cancers [9–11] and a series of major diseases by editing of glutamate receptors, editing of serotonin receptors and by other mechanisms [12]. Therefore, knowledge about the A-to-I editing sites in RNA is crucially important for both basic research and drug development.

Although the experimental method called “RNA-Seq” is a powerful tool for determining the RNA editing candidates [13, 14], it is time-consuming. Besides, since the A-to-I editing sites determined by the RNA-Seq tool are derived indirectly from the A-to-G sites rather than directly from the original A-to-I sites themselves, it is very difficult to discriminate RNA editing events from the case of A-to-G mutations in the single-nucleotide polymorphism (SNP) [15] that simply does not exist in the reference genome. And hence the A-to-I editing sites obtained by RNA-Seq often include many false positive ones.

Therefore, it would be very useful for in-depth genome analysis or drug development to develop a sequence-based computational method that can effectively predict which adenosine sites in a RNA sequence can be “A-to-I” edited, and which ones cannot.

The present study was devoted to address this problem.

## RESULTS AND DISCUSSION

A computational method called “iRNA-AI” has been developed. It is the first predictor ever established by using the computational approach and sequence information alone to identify human A-to-I editing sites.

Rigorous cross-validations on a well-established benchmark dataset (Supporting Information S1) have shown that the iRNA-AI predictor can achieve very high scores in sensitivity (Sn), specificity (Sp), overall accuracy (Acc), and stability (MCC); i.e.,

$$\{\begin{array}{l} S_{n}=86.18\%\\ \begin{array}{c} S_{p}=95.23\%\\ \text{Acc}=90.71 \end{array}\\ \text{MCC}=0.82 \end{array}$$ (1)

For the rigorous but intuitive definitions about S_n_, S_p_, Acc, and MCC, see Eq.16 given later.

Since so far there is no other existing computational method whatsoever for predicting the A-to-I editing sites in RNA of human transcriptome, it is not possible to show the predictor's power by the comparison manner between counterparts. Nevertheless, the power of iRNA-AI and its quality can be examined via a practical application on a set of experiment-confirmed independent dataset (Supporting Information S2), which contains 3,243 true A-to-I editing sites and 3,243 false A-to-I editing sites. The corresponding success rates thus obtained are given by

$$\{\begin{array}{l} S_{n}=84.19\%\\ \begin{array}{c} S_{p}=89.36\%\\ \text{Acc}=93.81 \end{array}\\ \text{MCC}=0.80 \end{array}$$ (2)

The above results indicate that the success rates achieved by the predictor iRNA-AI on the independent dataset of Supporting Information S2 are quite consistent with those via the jackknife test on the benchmark dataset of Supporting Information S1.

For the convenience of most experimental scientists, the web-server of iRNA-AI has been established at http://lin.uestc.edu.cn/server/iRNA-AI/.

Moreover, to maximize the users’ convenience, a step-by-step guide has been provided in Supporting Information S3, by which users can easily get their desired results.

Because knowledge about the positions of A-to-I editing sites would be of great help for in-depth understanding the biological functions and processes concerned. It is anticipated that iRNA-AI will become a useful high throughput tool for understanding the biological significance of A-to-I RNA editing, or at the very least, a complementary tool to the existing experimental methods in this regard.

Although the detailed ADAR's action mechanism is not well understood yet, the crystal structure of human ADAR has been solved (PDB code: 3IAR) that provides structural basis for the elucidation of its catalytic mechanisms and its specific recognition of the target sequence. Since both computational biology and structural biology have made great contributions to understand enzyme activities and catalytic mechanisms [16, 17], it is anticipated that the current iRNA-AI predictor will become a useful tool for revealing the catalytic mechanism of ADAR.

## MATERIALS AND METHODS

Prediction is always difficult, particularly in dealing with a complicated biological system as studied here. Nevertheless, a prediction method would be deemed rewarding or successful if it could timely help getting some useful information or stimulate and inspire some other relevant methods. To realize this, we should make the following five procedures very clear as done in a series of recent publications [18–29] according to the Chou's 5-step rules [30]: (1) how to construct or select a valid benchmark dataset to train and test the model; (2) how to formulate the biological sequence samples with an effective mathematical expression that can truly reflect their essential correlation with the target concerned; (3) how to introduce or develop a powerful algorithm (or engine) to run the prediction; (4) how to properly conduct cross-validation tests to objectively evaluate the anticipated accuracy; (5) how to provide a web-server and user guide to make people very easily to get their desired results. Since the content about the web-server has already been described in the RESULTS AND DISCUSSION section, below let us address the other four procedures one-by-one.

### Benchmark dataset

For facilitating formulation, the Chou's sequential scheme [31] was adopted. It was successfully used to study signal peptide cleavage sites [32, 33], hydroxyproline and hydroxylysine sites [22, 34], methylation sites [35–37], nitrotyrosine sites [38, 39], carbonylation sites [19], phosphorylation sites [24], sumoylation sites [29], and protein-protein binding sites [40, 41]. According to Chou's scheme, a potential RNA A-I editing site sample can be generally expressed by

$$R_{\xi }\left(A\right)=N_{-\xi }N_{-\left(\xi -1\right)}\cdots N_{-2}N_{-1}AN_{+1}N_{+2}\cdots N_{+\left(\xi -1\right)}N_{+\xi }$$ (3)

where the symbol A denotes the single nucleic acid code A (adenine), the subscript ξ is an integer, N_– ξ_ represents the ξ-th upstream nucleotide from the center, the N_+_ ξ the ξ-th downstream nucleotide, and so forth. The (2ξ + 1)-tuple RNA sample $R_{\xi }\left(A\right)$ can be further classified into the following two categories:

$$R_{\xi }\left(A\right)\in \{\begin{array}{c} R_{\xi }^{+}\left(A\right),\text{ if its center can be of A-to-I editing}\\ R_{\xi }^{-}\left(A\right),\text{ otherwise} \end{array}$$ (4)

where $R_{\xi }^{+}\left(A\right)$ denotes a true A-to-I editing segment with A at its center, $P_{\xi }^{-}\left(A\right)$ a false one with A at its center, and the symbol ∈ means “a member of” in the set theory.

**Figure 2:**
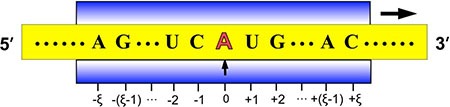
Illustration to show the sequence segments highlighted by sliding the scaled window [−ξ,+ξ] along a RNA sequence During the sliding process, the scales on the window are aligned with different nucleotides so as to define different (2ξ + 1) -nt RNA samples. Adapted from Chou [43] with permission. See the text for further explanation.

The benchmark dataset is derived from DARNED database [42] that contains 333,216 A-to-I editing sites confirmed by experiments. The detailed procedures to construct the benchmark dataset are as follows. (1) As done in [43], by sliding the (2ξ + 1)-tuple nucleotide window (Figure 2) along each of the RNA sequences taken from DARNED database, collected were only those RNA segments with A=A at the center. (2) The RNA segment samples thus obtained were marked with a positive label if their centers were experimentally annotated as the A-to-I editing sites, while those with a negative label if their centered adenosine could not be edited to inosine as confirmed by experiments. (3) To reduce redundancy or homology bias, we used the CD-HIT program [44] to remove those RNA segments that had 60% pairwise sequence identity with any other in a same-labeled group. By strictly following the above procedures, we obtained an array of benchmark datasets with different ξ values, and hence different lengths of RNA samples as well (see Eq.3), as illustrated below

$$S_{\xi }⋉\{\begin{array}{l} 37\text{ nucleotides},\text{ when }\xi =18\\ \begin{array}{c} 39\text{ nucleotides},\text{ when }\xi =19\\ \begin{array}{c} 41\text{ nucleotides},\text{ when }\xi =20\\ \vdots \\ 49\text{ nucleotides},\text{ when }\xi =24 \end{array}\\ 51\text{ nucleotides},\text{ when }\xi =25 \end{array}\\ 43\text{ nucleotides},\text{ when }\xi =26 \end{array}$$ (5)

In Eq.5 the symbol ⋉ means “formed by”. But it was observed via preliminary tests that when ξ=25 (i.e., the RNA samples formed by 51 nucleotides), the corresponding successful scores (see Eq.16 later) were most promising. Accordingly, hereafter we only consider the 51-tuple nucleotide samples.

After going through the above procedures, we obtained 6,243 positive label samples, from which we randomly picked out 3,000 to form the positive subset for the benchmark dataset. But keep it in mind that the remaining 3,243 samples would be used later for other purpose.

The corresponding negative label samples were substantially more than the positive ones. Although this reflects the fact that in the real world most adenosine nucleotides in RNA cannot be edited to inosine, a machine learning predictor trained by an imbalanced or highly skewing benchmark dataset may negatively affect its performance [36, 45]. To balance out the numbers between positive and negative samples for model training, we also randomly picked out 3,000 negative label samples to form the negative subset for the benchmark dataset. Thus, the benchmark dataset S can be formulated as

$$S=S^{+}\cup S^{-}$$ (6)

where the positive subset $S^{+}$ contains 3,000 true A-to-I editing site-containing RNA sequences, while the negative subset $S^{-}$ contains 3,000 false A-to-I editing site-containing RNA sequences, and $\cup^{\text{​}}$ denotes the symbol of “union” in the set theory [46].

The detailed sequences for the samples in the benchmark dataset are given in Supporting Information S1.

In literature the benchmark dataset usually consists of a training dataset and a testing dataset: the former is for the usage of training a model, while the latter for testing the model. But as elucidated in a comprehensive review [46], there is no need to artificially separate a benchmark dataset into the two parts if the prediction model is examined by the jackknife test or subsampling (K-fold) cross-validation since the outcome thus obtained is actually from a combination of many different independent dataset tests. According to such a point of view, it is enough to use the benchmark dataset of Eq.6 alone for the current study. It is instructive, however, to use the proposed predictor on an independent dataset for demonstrating its practical application. The independent dataset $S_{\text{Ind}}$ can be formulated as

$$S_{\text{Ind}}=S_{\text{Ind}}^{+}\cup S_{\text{Ind}}^{-}$$ (7)

where the positive independent subset $S_{\text{Ind}}^{+}$ contains the remaining 3,243 samples mentioned above, while the negative independent subset $S_{\text{Ind}}^{-}$ also contains 3,243 samples constructed in a way similar to that of $S^{-}$ in Eq.6. None of the samples in the independent dataset $S_{\text{Ind}}$ occurs in the benchmark dataset S.

The detailed sequences for the samples in the independent dataset are given in Supporting Information S2.

### Formulation of RNA samples

With the avalanche of biological sequences emerging in the post-genomic era, one of the most challenging problems in computational biology is how to formulate a biological sequence with a discrete model or vector that can, however, reflect its essential sequence pattern or feature. This is indeed indispensible since nearly all the existing machine-learning algorithms were devised to handle vectors but not sequences, as elucidated in a recent review [47]. Unfortunately, a biological sequence expressed with a vector might totally lose its sequence-order information [48] and sequence pattern features as well. To deal with such a problem for protein/peptide sequences, the pseudo amino acid composition (PseAAC) [49–51] was proposed. Ever since the concept of PseAAC was proposed in 2001 [48], it has been widely used in nearly all the areas of computational proteomics (see the long lists of papers cited in two review papers [51, 52]. Inspired by its great successes, the concept of PseAAC has been extended to cover DNA/RNA sequences as well by introducing the pseudo K-tuple nucleotide composition (PseKNC) [53, 54], which has been proved very useful in computational genetics/genomics [55, 56] as well as conducting various genome analyses (see, e.g., [26, 28, 57–64] and a review article [65]). Also, because the approach of pseudo components has been increasingly used in both computational proteomics and genomics, a very powerful web-server called “Pse-in-One” [66] has been established that can be used to generate various modes of pseudo components for both protein/peptide and DNA/RNA sequences.

According to [65], the general form of PseKNC for an RNA sequence sample can be expressed as

$$R={\left[\phi_{1}\phi_{2}\cdots \phi_{u}\cdots \phi_{Z}\right]}^{T}$$ (8)

where **T** is a transpose operator, while the subscript *Z* is an integer and its value as well as the components $\phi_{u}$ (u=1,2,⋯,Z) will depend on how to extract the desired information from the RNA sequence sample concerned. To enable Eq.8 to reflect both the short- and long-range sequence coupling information within the RNA sample, we are to use the nucleotide chemical property and nucleotide density to define its components as described below.

### Physicochemical properties of nucleotides

**Figure 3:**
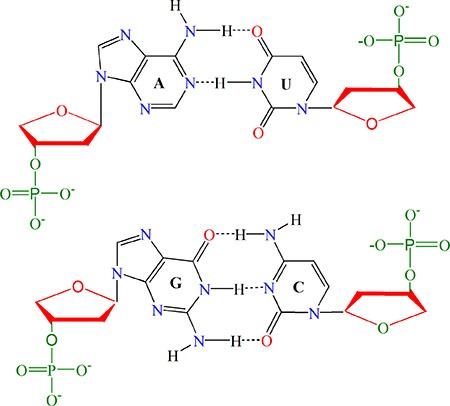
Illustration to show the structure of paired nucleic acid residues The upper panel is the A-U pair bonded to each other with two hydrogen bonds; the lower panel is the G-C pair with three hydrogen bonds. It can also be seen from the figure that A and G have two rings, while C and U have one ring. Also, according to chemical functionality, A and C can be classified into the amino group, while G and U into the keto group. See the main text for further explanation.

RNA is formed by four types of nucleotides: A (adenosine), C (cytidine), G (guanosine), and U (uridine). Among the four types: (1) A and G have two rings, whereas C and U only one; (2) from the angle of chemical functionality, A and C can be categorized as amino group, while G and U as keto group; (3) in forming the secondary or tertiary structure, there are three hydrogen bonds between C and G but only two between A and U (Figure 3), and hence, the former is stronger than the latter in hydrogen bonding, which would play different roles for the low-frequency vibration [67, 68] and its biological function accordingly [69, 70]. Therefore, the four types of nucleotides can be classified into three different groups as shown in Table 1.

**Table 1:** Nucleotide chemical property^a^

Physicochemical property	Classification	Nucleotides
Ring structure	Purine	A, G
	Pyrimidine	C, U
Functional group	Amino	A, C
	Keto	G, U
Hydrogen bonding	Stronger	C, G
	Weaker	A, U

a See the section of “Physicochemical Properties of Nucleotides” for further explanation.

Similar to the approach used in analyzing the codon usage for HIV proteins [71] and *E. Coli* proteins [72], to reflect the aforementioned features, let us formulate the *i*-th nucleotide of Eq.3 by

$${Ν}_{i}=\left(x_{i},y_{i},z_{i}\right)$$ (9)

where *x_i,_ y_i,_* and *z_i_* refer to the “ring structure”, “functional group”, and “hydrogen bonding” in Table 1, respectively; i.e.,

$$\begin{array}{ccc} x_{i}=\{\begin{array}{c} 1,\text{ if }{Ν}_{i}\in \left\{A,G\right\}\\ 0,\text{ if }{Ν}_{i}\in \left\{C,U\right\} \end{array}; & y_{i}=\{\begin{array}{c} 1,\text{ if }{Ν}_{i}\in \left\{A,C\right\}\\ 0,\text{ if }{Ν}_{i}\in \left\{G,U\right\} \end{array}; & z_{i}=\{\begin{array}{c} 1,\text{ if }{Ν}_{i}\in \left\{A,U\right\}\\ 0,\text{ if }{Ν}_{i}\in \left\{C,G\right\} \end{array} \end{array}$$ (10)

Thus, the nucleotide A can be formulated as (1, 1, 1), C as (0, 1, 0), G as (1, 0, 0), and U as (0, 0, 1).

### Distribution density of nucleotides

To reflect the occurrence frequency of a nucleotide and its distribution along the sequence of a RNA sample, we use the following equations

$$D_{i}=\frac{1}{\left\|L_{i}\right\|}\overset{@}{\underset{j=1}{\sum }}f\left(N_{j}\right)$$ (11)

where *D_i_* is the density of the nucleotide N_*i*_ at the site *i* of a RNA sequence, L_*i*_ the length of the sliding substring concerned, @ denotes each of the site locations counted in the substring, and

$$f(N_{j})=\{\begin{array}{c} 1,\text{ if }N_{j}=\text{the nucleotide concerned}\\ 0,\text{ otherwise} \end{array}$$ (12)

For instance, suppose a RNA sequence “ACGUA”. The density of “A” at the sequence position 1, 2, 3, 4, or 5 is 1=1/1, 0.5=1/2, 0.33≈1/3, 0.25=1/4, or 0.4=2/5 respectively; that of “C” is 0=0/1, 0.5=1/2, 0.33≈1/3, 0.25=1/5, respectively; and so forth.

By combing Eq.9 and Eq.11, the *i*-th nucleotide of Eq.3 can be uniquely defined by a set of four variables; i.e.,

$${Ν}_{i}=\left(x_{i},y_{i},z_{i},D_{i}\right)$$ (13)

Accordingly, the RNA sequence “ACGUA” can be successively expressed by the following five sets of digital numbers: (1, 1, 1, 1), (0, 1, 0, 0.5), (1, 0, 0, 0.33), (0, 0, 1, 0.25) and (1, 1, 1, 0.4). Or, according to the general form of PseKNC (Eq.8), it can be expressed as

$$R\left(\text{ACGUA}\right)={\left[1\;1\;1\;1\;0\;1\;0\;0.5\;\cdots \;1\;1\;1\;0.4\right]}^{T}$$ (14)

meaning that the 5-tuple nucleotide example can be defined by a 5 × 4 = 20-D (dimensional) PseKNC vector.

Consequently, a 51-nt RNA sample in the current benchmark dataset can be formulated with a 51 × 4 = 204- D vector.

### Support vector machine (SVM) operation engine

Being a machine learning algorithm based on statistical learning theory, SVM has been widely and successfully used in the realm of bioinformatics [58, 62, 73–75] and computational biology [18, 36, 59–61, 76]. The basic idea of SVM is to transform the input data into a high dimensional feature space and then determine the optimal separating hyperplane. For a brief formulation of SVM and how it works, see the papers [77, 78]; for more details about SVM, see a monograph [79].

In the current study, the LibSVM package 3.18 was used to implement SVM, which can be freely downloaded from http://www.csie.ntu.edu.tw/~cjlin/libsvm/. Because of its effectiveness and fast speed in training process, the radial basis kernel function (RBF) was used to obtain the best classification hyperplane here. In the SVM operation engine, the regularization parameter *C* and the kernel width parameter γ were optimized via an optimization procedure using the grid search approach as described by

$$\{\begin{array}{l} 2^{-5}\leq C\leq 2^{15}\text{ with step }\Delta C=2\;\\ 2^{-15}\leq \gamma \leq 2^{-5}\text{ with step }\Delta \gamma =2^{-1} \end{array}$$ (15)

where ΔC and Δγ represent the step gaps for *C* and γ, respectively.

The predictor obtained via the above process is called iRNA-AI, where “i” stands for “identify”, and “AI” for “A-to-I editing” sites in RNA sequence.

### Prediction quality examination

How to objectively evaluate the anticipated success rates is an indispensible step in developing a new predictor [30]. To address this, we need to consider two issues: one is what metrics should be used to reflect the predictor's success rates; the other is what test method should be adopted to derive the metrics rates.

To quantitatively evaluate the quality of a binary classification predictor, four metrics are generally needed [80]. They are: (1) Acc for the predictor's overall accuracy; (2) MCC for its stability; (3) Sn for its sensitivity; and (4) Sp for its specificity. Unfortunately, the conventional formulations for the four metrics are not quite intuitive and most experimental scientists feel difficult to understand them, particularly the stability of MCC. Fortunately, as elaborated in [59, 81], by using the Chou's symbols and derivation in studying signal peptides [82], the conventional metrics can be converted into a set of four intuitive equations, as formulated below:

$$\left\{ \begin{array}{l} \text{Sn}=1-\frac{N_{-}^{+}}{N^{+}}\;0\;\leq \text{Sn}\leq 1\\ \begin{array}{cc} \text{Sp}=1-\frac{N_{+}^{-}}{N^{-}} & \;0\leq \text{Sp}\leq 1 \end{array}\\ \begin{array}{ll} Acc=\;\Lambda =1-\frac{N_{-}^{+}+N_{+}^{-}}{N^{+}+N^{-}} & \;0\leq \text{Acc}\leq 1\; \end{array}\\ \begin{array}{ll} \text{MCC}=\;\frac{1-\left(\frac{N_{-}^{+}}{N^{+}}+\frac{N_{+}^{-}}{N^{-}}\right)}{\sqrt{\left(1+\frac{N_{+}^{-}-N_{-}^{+}}{N^{+}}\right)\left(1+\frac{N_{-}^{+}-N_{+}^{-}}{N^{-}}\right)}}\; & -1\leq \text{MCC}\leq 1\; \end{array} \end{array}\right.$$ (16)

where $N^{+}$ represents the total number of A-to-I editing samples investigated, while $N_{-}^{+}$ is the number of true A-to-I eting samples incorrectly predicted to be of non-A-to-I editing sample; $N^{-}$ the total number of the non-A-to-I editing samples investigated, while $N_{+}^{-}$ the number of the non-A-to-I editing samples incorrectly predicted to be of true A-to-I editing sample.

Now it is crystal clear to see the following from Eq.16. When $N_{-}^{+}=0$ meaning none of the true A-to-I editing samples are incorrectly predicted to be of non-A-to-I editing sample, we have the sensitivity Sn = 1. When $N_{-}^{+}=N^{+}$ meaning that all the true A-to-I editing samples are incorrectly predicted to be of non-A-to-I editing sample, we have the sensitivity Sn = 0. Likewise, when $N_{+}^{-}=0$ meaning none of the non-A-to-I editing samples are incorrectly predicted to be of true-A-to-I editing sample, we have the specificity Sp = 1; whereas $N_{+}^{-}=N^{-}$ meaning that all the non-A-to-I editing samples are incorrectly predicted to be of true A-to-I editing samples, we have the specificity Sn = 0. When $N_{-}^{+}=N_{+}^{-}=0$ meaning that none of true A-to-I editingsamples in the positive dataset and none of the non-A-to-I editing samples in the negative dataset are incorrectly predicted, we have the overall accuracy Acc = 1 and MCC = 1; when $N_{-}^{+}=N^{+}$ and $N_{+}^{-}=N^{-}$ meaning that all the true A-to-I editing samples in the positive dataset and all the non-A-to-I editing samples in the negative dataset are incorrectly predicted, we have the overall accuracy Acc = 0 and MCC = −1; whereas when $N_{-}^{+}=N^{+}/2$ and $N_{+}^{-}=N^{-}/2$ we have Acc = 0.5 and MCC = 0 meaning no better than random guess.

Accordingly, it has rendered the meanings of sensitivity, specificity, overall accuracy, and stability much more intuitive and easier-to-understand by using Eq.16, particularly for the meaning of MCC, as concurred recently by many investigators (see, e.g., [36, 38, 40, 45, 61, 62, 75, 83–88]).

Note that, however, the set of metrics as defined in Eq.16 is valid only for the single-label systems. As for the multi-label systems whose emergence has become more frequent in system biology [89–91] and system medicine [92] or biomedicine [25], a completely different set of metrics are needed as elucidated in [93].

With a set of good metrics to measure the quality of a predictor, the next thing we need to consider is what validation approach should be adopted to score these metrics. In statistical prediction, the following three cross-validation methods are usually applied: (1) independent dataset test, (2) subsampling (or K-fold cross-validation) test, and (3) jackknife test [94]. Of these three, however, the jackknife test is deemed the least arbitrary that can always yield a unique outcome for a given benchmark dataset as elucidated in [30]. Accordingly, the jackknife test has been widely recognized and increasingly used by investigators to examine the quality of various predictors (see, e.g., [63, 76, 78, 95–107]). In view of this, here we also used the jackknife test to examine the quality of iRNA-AI predictor. During the jackknifing process, both the training dataset and testing dataset are actually open, and each sample will be in turn moved between the two. The jackknife test can exclude the “memory” effect. Also, the arbitrariness problem as mentioned in [30] with the independent dataset and subsampling tests can be totally avoided since the outcome obtained by the jackknife cross-validation is always unique for a given benchmark dataset.

Even though, however, in order to reduce the computational time, the K-fold cross-validation approach has still often been used, as done by many investigators with SVM as the prediction engine (see, e.g., [25, 28, 102, 107]). Also, for demonstrating the practical application of a predictor, the independent dataset test has often been used as well [18].

## SUPPLEMENTARY FIGURES AND TABLES






