# Supplementary material for: iRNA-AI: identifying the adenosine to inosine editing sites in RNA sequences
Source: Oncotarget. 2016 Dec 1;8(3):4208–17. doi: 10.18632/oncotarget.13758 (PMC5354824; doi:10.18632/oncotarget.13758)
Supplement: Supplementary file 1 [file oncotarget-08-4208-s001.pdf]

iRNA-AI: identifying the adenosine to inosine editing sites in RNA sequences

Supplementary Materials

Supplementary Information S1: The benchmark dataset constructed for training and analyzing the model. It consists of (1) a positive subset and (2) a negative subset. The former contains 3,000 experiment-confirmed A-to-I editing RNA samples, and the latter contains 3,000 experiment-confirmed non-A-to-I editing RNA samples. Each of these samples is 51-bp long with the adenosine (A) at the center. None of the included samples has ≥60% pairwise sequence identity to any other in a same subset. See the main text for further explanation. See Supplementary_Information_S1

Supplementary Information S2: The independent dataset constructed for further testing the model and demonstrating its practical application. It contains 3,243 experiment-confirmed A-to-I editing-site RNA samples and 3,243 experiment-confirmed non-A-to-I editing-site RNA samples, none of which occurs in the benchmark dataset of Supporting Information S1. See the main text for further explanation. See Supplementary_Information_S2

Supplementary Information S3: User Guide for the Web-Server iRNA-AI

To maximize the users' convenience, a step-by-step guide has been provided below for how to use iRNA-AI.

Step 1. Open the web server at http://lin.uestc.edu.cn/server/iRNA-AI/ and you will see the top page of the iRNA-AI predictor on your computer screen, as shown in the figure bellow. Click on the Read Me button to see a brief introduction about the predictor and the caveat when using it.

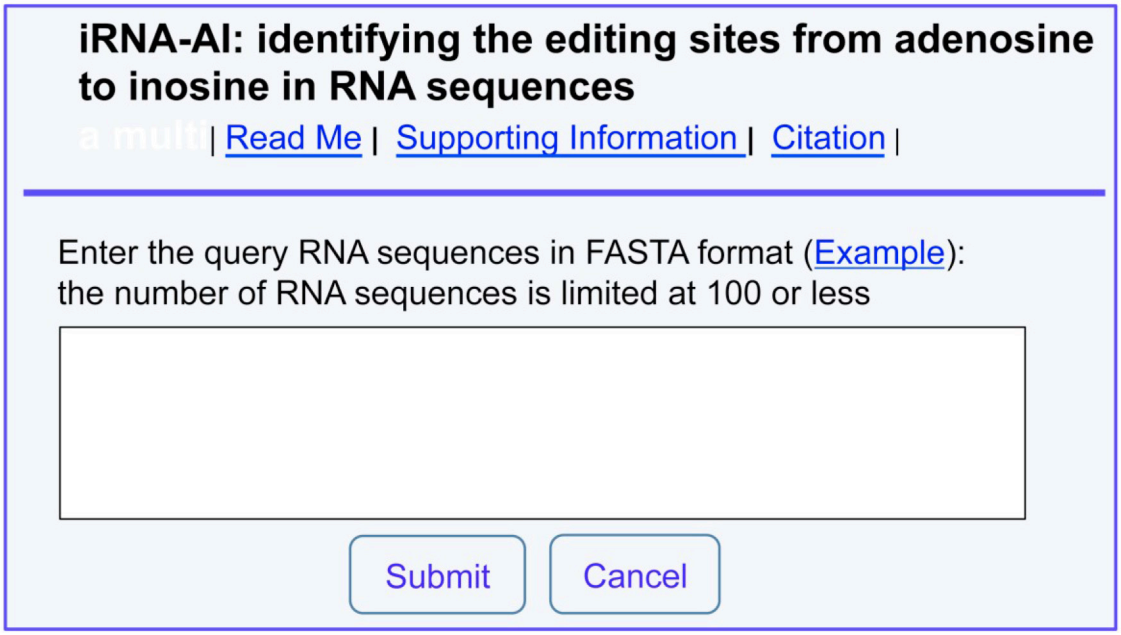

Step 2. Either type or copy/paste the query RNA sequences into the input box at the center of **Figure S1.** The input sequence should be in FASTA format. For the example of RNA sequences in FASTA format, click the Example button right above the input box.

Step 3. Click on the Submit button to see the predicted result. If you use the two query RNA sequences in the Example window as the input, you will see the following shown on the screen of your computer: the 1st query sequence is 102-nt long with 25 A (adenosine) residues of which the ones at position 26, 65, 69, 73, and 77 can be edited to I (inosine), while all the other cannot; the 2nd query sequence is 197-nt long with 15 A (adenosine) residues of which the ones at position 49, and 90 can be edited to I (inosine), while all the other cannot.

Step 4. Click on the Data button to download the datasets used to train and test the model.

Step 5. Click on the Citation button to find the relevant publications that have played the key role in developing the **iRNA-AI** predictor.
