# Supplementary material for: iRNA-AI: identifying the adenosine to inosine editing sites in RNA sequences
Source: Oncotarget. 2016 Dec 1;8(3):4208–17. doi: 10.18632/oncotarget.13758 (PMC5354824; doi:10.18632/oncotarget.13758)
Supplement: Supplementary file 2 [file oncotarget-08-4208-s002.docx]

**Supporting Information S1. The benchmark dataset constructed for training and analyzing the model.** It consists of (**1**) a positive subset and (**2**) a negative subset. The former contains 3,000 experiment-confirmed A-to-I editing RNA samples, and the latter contains 3,000 experiment-confirmed non-A-to-I editing RNA samples. Each of these samples is 51-bp long with the adenosine (A) at the center. None of the included samples has $\geq60\%$ pairwise sequence identity to any other in a same subset. See the main text for further explanation.

**(1) List of 3,000 A-to-I editing site samples**

>P1

GUCGGGCGUGGUGGCAGGUGCCUGUAAUCCCAGCUACUCGGGAGGCUGAGG

>P2

ACCACCGCCGGCCGAUGGGCGUCUUACCAGACAUGGUUAGACCUGGCCCUC

>P3

AGACGCAGGCUGGGCGCGGUGGCUCAGGCCUGUAAUCCCAGCACUUUGGGA

>P4

AGGCCUGUAAUCCCAGCACUUUGGGAGGCCGAGGCUGGUGGAUCACCUGAG

>P5

CGAGGCUGGUGGAUCACCUGAGGUCAGGAGUUCGAGACCAGCAUGGCCAAC

>P6

GGAGUUCGAGACCAGCAUGGCCAACAUGGUGAAACCCCGUCUCUACUAAAA

>P7

AACAUGGUGAAACCCCGUCUCUACUAAAACUACAAAAAUUAGCCUAGCGUG

>P8

CCCAGCUACUCGGGAGGCUGAGGCAAGAGAAUCGCUUGCGCCCAGGAGGCA

>P9

CGCCCAGGAGGCAGAGGUUGCGGUGAGCCGAGAUCGCACCACUGCACUCCA

>P10

GUCUCUACUAAAAAAUACAGAAAUUAGCUGGGCUUGGUGGCGGGCACCUGU

>P11

GUGGCGGGCACCUGUAGUACCAGCUACUCGGGAGGCUGAGGCAGGAGAAUG

>P12

GGGAGGCAAAGCUUGCAGUGAGCCAAGAUCACGCCACUGCACUCUAGCUUG

>P13

UUUUUUGUAGAGACGGGGUCUCAUCAUGUUGCCCAGGCUGGUCUCGAACUC

>P14

UUGCCCAGGCUGGUCUCGAACUCCUAGGCACAAGCAAUCCUCUCGCCUCAG

>P15

UAGGCACAAGCAAUCCUCUCGCCUCAGCCUCCUAAAGUGUUGGGAUUACAG

>P16

UUGUUUUUUUUUUUUUUUUUGAGACAGAGUCUCAUUCGUCGUCGAGGCUGG

>P17

AUUCUCCUGCCUCAGCCUCCUGCAUAGCUGGGAUUGCAGGCAUCCACCACC

>P18

AGUAGAGACAGGGUUUCACCAUGUUAACCAGGAUGAUCUCCUGACCUCCUG

>P19

AUUUUCUUGCCCGCACGUUCUUGAUAGCAGAUCACUGACAACAGCCAUUGG

>P20

GCCGGGCGCGGUGGCUCACACCUGUAAUCCCAGCACUUUGGGAGGCUGAGG

>P21

CUCGGGAGGCUGAGGCAGGAGAGUGACGUGAACCCGAGAGGUAGAGCUUGC

>P22

GGAGAGUGACGUGAACCCGAGAGGUAGAGCUUGCAGUAAGCAGAGAUCAUG

>P23

GCCACUGCACUCCAGCCUGGGCCAGAAUGAGACUCCGUCUCAAAAAAAAAA

>P24

UAAAAAAAAGAAAAAAAAAGAAAAGAAUUAGCUGGGUGUGGUGACAUGUGC

>P25

ACUUUCAAGUGUGCUGCUCAAGUGCAGUGGCGCGAUCUCGACUCACUGUCA

>P26

AUUCUCCUGUCUCAGCCUCCGGAGUAGCUGGGACUACAGGUGUCUGUCGCC

>P27

GCCCGGCUAAUUUUUUUGUAUUUUUAGUAGAGAGAGGGUUUCAUCCUGUUA

>P28

GUGGGCCUCCCAAAGUGCUGGGAUUACGGGCGUGAGCCACCGUGCUCAGCC

>P29

AGCCAGCUAAUUUUUUCAUGUUUUUAGUAGAGACGAGGUUUUUCCAGGUUG

>P30

CUGCAUUUUGACAACUGUGUUCUUAAGCCGGCCACAGAAGGAAAACGGUGA

>P31

ACUACAGCCUCCGCCUCCUGGGUUCAAGUGAUUCUCCUGCCUCAGCCUCCC

>P32

UGAGGCGGGAGAAUCACUUGAACCCAGGAGGUGGAGGUUGUAGUGAGACAA

>P33

AGGAGGUGGAGGUUGUAGUGAGACAAGAUUGCGCUACUGCAAUCCCACCUG

>P34

GUACUCCUUUUUUUUUUGAGAUGGAAUUUCGCUCUCAUCGCCCAGGUUGGA

>P35

AUCGCCCAGGUUGGAGUGCAGUGGCACGAUCUCAGCUCACUGCAACCUCCG

>P36

AGUGGCACGAUCUCAGCUCACUGCAACCUCCGCCUCCCGGGUUCAAGUGAU

>P37

CAUCCGCGGGAGUGGGGCCGAGGACAUGGGAGUGGCAGGUGCAGCCCCCGG

>P38

GGGAGUGGCAGGUGCAGCCCCCGGUACUCACUCAGCCCCAGGGAGUGUCCC

>P39

GGCAGGUCCCGGGGGAGGCUGGGUUAGUGGCAGCUCCGGGAUGAGACCUCA

>P40

GGAUGAGACCUCAGAGGUCUGUCUGACUUGUCCAAGCCCGGCUAUGGGGAG

>P41

GCCCGGCUAUGGGGAGGUGGGGGGAAGGAAGGAAGAGGAGAGAAAUAAGGA

>P42

CCAGGGUGGAUGUCUCAGUGCACACACAGGAACGUGAAAUGGCUGAGCCGG

>P43

AGCGUGUGCGGGGUGGCCGGCGGAGAAGCAGCAGGAUCCCAGGCGGGAGGG

>P44

CCAGGCGGGAGGGCACGGCCUUCUGAGAGGCAGGUUGCAGGACAAUACUCA

>P45

GUGUUUUGAUCCAGGAGGUCCUAUUAGGGCGUUUCAUUGAUAAACGCACAU

>P46

CGCACAUUUGAGGUGGAGGGUGGCCAGUGUAGAAUGUGCCUGGCAGCUUUC

>P47

GGACCUGGCAGGCAGAGCUCCGGAAAGGAGGGAACUGCACAGACAGAAAGC

>P48

CUCACCAUGAAAAGUCACCUAGGCCAGGCAUGGUGGCUCACGCCUGUAAUC

>P49

AUCCUGAGUAACAUGGUGAAACCCCAUCUCUACGAAAAAUACAAAAAUUAA

>P50

UGGGCAGGAGACCCCCGUUCUCAGGAGGCUGUGCUGCCAGCAGGGGAUGCC

>P51

AGGAUUGGAAAGCAGCCGGGGCCACAGUUCAGGCAGGAAACUAUUCUUAUU

>P52

GUGUGUGGUGUGCGCCUAUAAUCCCAGCCACCAGGGAGGCUGAGGCAGGAG

>P53

UAUCUCACUGCAACCUCCAACUCCCAGGCUCAGGCGAUUCUCCUGCCUCGG

>P54

GCACGCAGGUCCUUCCGAAACUGUCAUCUUCCAUUAUCCACGAGAUUGAUA

>P55

UUGGGUCCUUGGGGCGGGCAGGGCCAGCCUCUCCUCUGCUGAGAAUCCCCA

>P56

AGAGGGCAGGUGGCCCACCCUCGCCAUCAGGGAGGGUGGCUGGCCCCAUCC

>P57

GGGCCAGGUCCCCCCUCUCGGGAGGAGGUAUUGGGUAGGACCAUCCAAGAA

>P58

GAAAGGAGGGCGCUGGCCCUGCUGGACGCUUCGGAGCCCCCACUGUUUCCC

>P59

UCUGGCUUGGGGAGGAGAACCUUAAAGCCCUCACUGCUCUUCCCCUCCCCC

>P60

CCCGGCUAAUUUUUAUAGUUUUGGUAGAGAUGGGGUUGUGCUAUGUUGCCA

>P61

UGCUAUGUUGCCAAAGAUGGUCUCCAACUCCUGGGCUAAGUGAUCCUCCCA

>P62

UGGUCUCCAACUCCUGGGCUAAGUGAUCCUCCCACCUCAGCCUUCCAAAAU

>P63

AGUGAUCCUCCCACCUCAGCCUUCCAAAAUGCUGGGAUUAUAGACGUGACC

>P64

AACACGCACGACGACAGCAGGAACAACGCGGCCUCAGCCAGGUAAAGCAAG

>P65

AAAAUAUGGAUCAUCACAGAUCCAAACAGAAAAAUGGGGGCGAUGUCCCCA

>P66

UAGUCAGGCAUGGUGGUUCGUGCCUACAGUUCCAGCUACUUGGGAGGCAGA

>P67

AGAGGUUGCAGUGAACCAAGAUCGCACCACUGCACUCUGGCCUGGUCAACA

>P68

GAGCAGCUGCUAGAACGGGACCACAAAGGAAAGCUGAUGAAAAAGUCUAUG

>P69

GAGAUGGAGUCCCCCUCUGUCACCCAGGCUGGAGUACAGUGGCGGGAUCUC

>P70

CGGGUUCAAGUGAUUCUUCUGUCUCAGCCUCCUGAGUAGCUGGGAUUACAG

>P71

GAGACGGGGUUUCAGCAUGUUGGUCAGGCUGGUCUUGAACUCCUGACCUCG

>P72

UUAUUUCUAGUAGAAAAAGAGUUUCACCAUAGUGACCAGGCUGGUCUCAAA

>P73

UCACCAUAGUGACCAGGCUGGUCUCAAACUCUUGACCACAGGUGAUCCACC

>P74

CCUUGGCCUCAAACAAUCCUCUCAUACCUCAGCCUCCAGCGUGGCUGAGAC

>P75

CAUUCUGGGCUGGGCAUGAUGGCUCACGUCUGUAACCCCAGCACUUCGGGA

>P76

UCCAUGCAGGUGUUAUGGACAUUCCAGGCUGGAUAAUUCUUCGUUGUUAGG

>P77

UUGACGGCACAGCCAAUUCCUCGCUAUGCCCAAGGGCUUUUCCCAGGCAUC

>P78

AGGCUGGUCUUGAAUUCUUGAGCUCAAGUGAUCCUCCUACCUCAGCCUCCC

>P79

UGAGCAAGGCCGCCCCUCUGUUAGAAGUCCCCUUCUCUAUGCCUCAUGGUU

>P80

UUUGGGAUGCCGAGAUGGGAGGAUCACCUGAGGUCAGGAGUUUGAGACCAG

>P81

GAUCACCUGAGGUCAGGAGUUUGAGACCAGCCUGGCCAACAUGGAGACAUG

>P82

UUAGUCCAUUCUUACGCUGCUAUAAAGAACUAUCUGAGACUGGGUAAUUAA

>P83

CACACACCACCAUGCCCAGCUAAUUAUUUAAUUUUCGUAGAGAUGGAGUUU

>P84

AGCUUGAGACCAGCCAGGGCACCAUAGUGAGACCCCAUCUCUACAAAAAAU

>P85

UCUGCAACCUCCUCUUCCAGAGCUUAAGCGAUCCUCCCAUCUCAGCCUCCU

>P86

UGGGCUCAGGCCAUCGUCCUGCCUCAGCCUCCGGGGUAGCUGGGACCACAG

>P87

CGGCUAAUUUUAUGUAUUUUUUAGUAGAAACAGGGUUUCACUAUGUUAUCC

>P88

CUCCCAAAGUGCUGGGAUUACAGGCAUGAACCACCGCGCCCAGCUGGUUAG

>P89

UUCUUAAAAUUCACUAAAUUGGGCUAGGUGUGGCUCAUGCCUGUAAUCCCA

>P90

GUAAUCCCAGCACUAUGGGAGGCUGAGGUGAGAGGAUCACUUGAGCCCAGA

>P91

GGAUCACUUGAGCCCAGAAGGUUGAAACCAGCCUGGGCAACAUAGUGAGAC

>P92

GAGCCCAGGAGACUGAGGCUGCAGUAAGGUGUGAUUGCACUAUUGCUCUCU

>P93

UCCUGUAAUAAUCCUGCCUGCUUUUACCUCUCGUCCACUGACCAGCAAGUG

>P94

UCCCAGCAUUUUGGGAGGCCAAGGCAGGCAGAUCACUUGAGGCCAGGAGUU

>P95

AGUAUGAGACCAGCCUGGCCAGCAUAGUGAAACCCCCUCUCUUAGCCAGGC

>P96

UCUGUUGCCCAGGCUGGAGUGCAGCAGUGCAAUCUUGGCUCACUUCAGCCU

>P97

AUGAUCUGCCUGUCUUGGCCUCCCAAAGUGCUGGGAUUACAGGUAUGAGCU

>P98

GAGCCCAGGAGUUUGAGACCAGCCCAGGCAACAUAGUGAAACUCCAUCUCU

>P99

AAAUUAGCUAGGCAUGGUGGUGUGCACCUACAGUCCCAGCUACUCCACAGG

>P100

AUCGAGGCUGCACUGAGCCAUGAUCACACCACUACACUCCAGCCUGGGUGA

>P101

CUCAGCCUCCCGAGUAGCUGGGAUUACAGGCACCCACCAGUAUGCUUGGCU

>P102

UUAUUUUUAGUGGAGACGAGGGCUCACUAUGUUUCCUAGGCUAGUCUUGAA

>P103

UCCUGCCUCAUCCUCCCAAAGUGCUAGGAUUACAGGUGGGAGCCACCGUGC

>P104

UGAGGUUUCACCUGUUGGCCAGUCUAGUCGCAAACUCCUGACUUCAGGCGA

>P105

CUUUUUCUUUUUUUUUCAAAGCAGAAUCUCACUCCAUCACCCAGGCUGGAG

>P106

AGAGAUGGAGUCUUGCUAUGUUGCCAAGGCUGGUUUCAAACUCUUGGUGUC

>P107

CCGAAGAUCCCUGUUUUGCAUCUCAAAACCGUGUGUACAAUGACAUUGGCA

>P108

AAAUGCAAUGCUGUUAUCAAUGAGGACCCCAAUGCCAAACUGGUUCGUGAA

>P109

CCUGACCUCAUGGUCUGCCCGCCUCAGCCUCCCAAAGUGCUGGAAUUACAG

>P110

AGGUGAUCCUCCUACCUCAGCCCCCAAAGUAGCUGAGACCACAGGUAUGUG

>P111

UAGUCUCCUGAGUAUCUGGGACUAUAGGCGUGCACCACCACGGCCAGCUAA

>P112

UGGUCCACCUGCCUCAGCCUCCUAAAGCUCUAGGAUUACAGGCGUGAGCCA

>P113

UCCAGCUUCAGCCUCCCAAGUAGCUAGGACAGAUGCAUGCCACUACGCCCA

>P114

GACACUUGUCCCUAGAGAUCCUUAAAGCCCCUCUGAAGGACAGGCCACUCA

>P115

GCUGUGACUACAGGCAUGUGCCACCACACCUGGUUAAUUUUUGUAUUUUUA

>P116

GGCCAGGAGCCCCCAUGGGCCGCCCAGUACCAUGCACACUCCUGUCCCGAA

>P117

ACUCUGUCUUCCAGGCUGGAGUGCAAUGACGCUAUCCCCGCUUUCUGCAAC

>P118

CAGGCACCCAUCACCACGCCUGGCUAAUUUUUGUUUUUUUAUUAAAGAAAG

>P119

GUUUUUUUAUUAAAGAAAGGGUUUCACCAGGUUGGUCUGGCUGGUCUUGAA

>P120

UACUGGAUUACAGGCGUGAGCCACCAUCCCAGGCCUGAAAGUCUUUUAAAA

>P121

GUGGUCUUGCUCUGUCUCCCAGGCUAGAGUGCAGUGGACUGAUCAUAGGUC

>P122

GGGGCUGCUUCCCCGAUAUUCUGAGAGCCAGGCGGAAGGACAGGAGCAGCU

>P123

AGUUAAAUGGGUGUGUAAAGACAGGACUGUUGAAGUCAACACUGAAUAUGA

>P124

CACUAAGAUGAUGGCAUGUGCCUGUAGUUCUAGCUACUUGACAGGCUGAGG

>P125

AGUAAAAGAAAUAGAUAGGCAUAAGAAUCAAAAAGGCAAACAACAAUUUUA

>P126

AAGAAGGAACACAGAGGCCAGGAGCAGUGGCUCACUCCUGUAAUCCCAGCA

>P127

AGCAGUGGCUCACUCCUGUAAUCCCAGCAUUUUGGGAGGCCGAGGAUGAUG

>P128

CCCAAGUGUCAGUAUUGCCAAGGCUAGCCGAGUACAGUGGCUUAUGCCUGU

>P129

CAAGGCUAGCCGAGUACAGUGGCUUAUGCCUGUAAUCCUAGUAUUUUGGGA

>P130

GCCGAGAUAGGAGGGUCAUGGAGCCAAGGAGUACGAGACUAGCCUACACAA

>P131

GAGCCAAGGAGUACGAGACUAGCCUACACAACAAAACGAGACCUCAUCUCU

>P132

ACUAAAAAUACAAAACAAAUUAGCCAGGUGUUGUGGCAGGCACCUGUAAUC

>P133

GGUGGAGGUUGCAGUGAGUGGAGAUAGCUCCACUGCACUCCAGCCAGGGUG

>P134

UGGCCUGUCCAGAAUGUGGCCGAUGACCCAGCAUGGUGGCUCAUGCCUGUA

>P135

UACACUCCAGCUUGGGUGACAGAGCAAGACUCCAUCUCAAAAAAGUUAAAA

>P136

GAAACUGUGCCUAAUCAAAUCAGCUAUUGAGUAGGUAAACAGUCUGUGCUG

>P137

UUUAAAAGAAAUGUUCAGGUCCAGUAGCUCAUGCCUGGUAAUCCCAGCACU

>P138

GUAGCUCAUGCCUGGUAAUCCCAGCACUUUGGGAGGCUGAGGCAGGCAGAU

>P139

GCAGGCAGAUUGCUUGAGCCCAGGAAAUCGAGACCAGCCUGGGCAACAGAG

>P140

CAGGAAAUCGAGACCAGCCUGGGCAACAGAGCGAGACCCUUUCUCUACAAA

>P141

CAGAGCGAGACCCUUUCUCUACAAAAGACGCAAAAAAUAGCUGGACAUGGU

>P142

CAUGGUGGUGUGUGCCUGUAGUCCCAGCUCCUCGGGGGCCGAGGUGGGAGG

>P143

AGGAUUGCUUAAGCCUGGGAGGUCAAGGGUACAGUAAGCCAUGAUCAUGCC

>P144

UGUGGUGGCACAUGCCUGUAGUCCCAGCUACACAGGAGGCUGAGGUGGGAG

>P145

GGAGAAUCACUUGAACCUAGGAGGCAGAGGUUGCAGUGAGCCAAGACCACG

>P146

AGCUGGCAUGGUGGUGUGUGCCAGUAGUCACCACUAUUUGGGAGGCUGUGC

>P147

UGCCACCAUGUAAUUAGCCCGGCUAAUUUUGUAUUUUUAGUAGAGACGGGA

>P148

GCCCGGCUAAUUUUGUAUUUUUAGUAGAGACGGGAUUUCUCCAUGUUGAUC

>P149

GCUGGGAUUACAGGCGUGAGCCACCACUCCCGGCUAAGUUAGUAUUUCUUU

>P150

ACCAGGAGCCUCAGGGAACCCAGCCAGCACAGCAGCAGCAGCCUUGCUUCU

>P151

CGACUUGCAAACGGAUUGGCCUAAUAAAGAAGUUCAACCUGGAGAGAUGGA

>P152

CAGCCUCUCAAGUAGCUGGAACCACAGGCAUGUGCAACCAUACCUGGCUGA

>P153

AAUGUACUUACUUGUUUACAUAGUGAGACGGGAUAUUGCUAUGUUGCCCAA

>P154

AAUCCACCCGCCUCAGCCUCUCAAAAUGACAGGAUUAUAGGAACGAGACAC

>P155

AUUAGCCAGCCAUGGUGGCGCGCCUAUGGUCUCAGCUGUUUGAGAGGCUGA

>P156

AAAAAAAAAACAUGUUUUUGAUAGUAGGGAUGGUGUCUCACUAUGUUGCCC

>P157

GGAUUCUCCCUCCUGGACCUCCCAAAGUGUUGGGACUGCAGGCCAGAGUCA

>P158

AAAAGAAAGCUCUCUCUAUAUACACACACACAUACACACUCACCACAUAUA

>P159

CACUGGGUUUUUCUGUAUUUAUUUUAGAGACAGAGUCUCACUCUAUCACCC

>P160

UAUUUUAGAGACAGAGUCUCACUCUAUCACCCAGGCUGGAGUGCAGUGGAA

>P161

GGCUGGAGUGCAGUGGAACAAUCACAGCUCACUGCAGCCUCAAACUCCUGG

>P162

CACUACGCCCAGCUUGACUUCUUUUAGUAGAGAAGAGGUCUCACUGUGCUG

>P163

CACCUAUAAUCCCAGCUACUUGGGAAGCUGAGGUGAGAGAAUUGCUUGAAC

>P164

GAAGCUGAGGUGAGAGAAUUGCUUGAACCUAGAAGGCAGAGGUUGCAGUGA

>P165

GUGGUGGUGCAUGCUUGGAAUCCUAACUACUUGCGAGGCUGAGGUGGGAGA

>P166

CAAUCCCAGCACUUUGGGAGGCCGAAGUGGGUGGCUUACUUGAGGUUAGGA

>P167

UGGCUUACUUGAGGUUAGGAGUUCGAGACCAGCCUGGCGAACAUGGUGAAA

>P168

AACAGAGCAAGACUGUCUCAAAAGGAAAAAAAAAAAAAGUAUGUACCAUUG

>P169

GUCUCGCUCUGUGGCCCAGGCUGGAAUGCAGUGGCAUGAUCUCAGCUCACU

>P170

AGUUCUUCUGCCUCAGCCUCCGAGUAGUUGUGAUUACAGACGUGUGCCAGU

>P171

UUACAGGCGUGAGCCAAGGCACCUGACCUAGGGUUGUUAUAUGUUUGACAU

>P172

GUAUGCUGGAGAAGAUGCGGGUGCUAGGGCUGGGGCUUGGGAGGACAAAGG

>P173

UCAGUUAAUAGGAUCCCUCUGAGACAGGGUCUCGCUCUGUUGCCCAGGCUG

>P174

AUCCUCCCACCUCAACCUCCCGAGUAGCUGAGACUACAGGCGCACGCCACC

>P175

UGAGACUACAGGCGCACGCCACCACACCCAGCUAGGUUUUUUUUGUUUAUU

>P176

AUAGAGAAGACUCAGUGUGUUGCCAAGCUGGUCUCGAACACCUGGGCUCCA

>P177

GGUCAAGAGCAGCAAGGGGCCAGGCAUGGUGGCUCACACCUAUGAUUCCAA

>P178

CGCCUGGAAUCCCAGCACUUUGGGAAGCCGAGGUGGGCAGAUCAUAAGGUC

>P179

GGGCAGAUCAUAAGGUCGGGAGAUUAAGACCAUCCUGGCUAACACGGUGAA

>P180

CUCAGCCUCCCGAGGAGCUGGGACUACAGAUGAGCACCACCAUGCCUGGCU

>P181

UCCCAACACUUUGGGAGGCUGAGGCAGGUGGAUUGCGUAAGCUGAGUUCAA

>P182

UGGCUGACACCUGUAAUCCCAACACAUUGGGAGGCUUGCUUGAGGAUUGCU

>P183

AGCCUGGGAAACAGAGUGAGAUCCCAUCUCUACAAAAAAAAAAUAUGUUUU

>P184

CGUCCUCCUCAGGUCUCUUCGAGCCAGGGGACAUGCAGUACGAGCUGAACA

>P185

GAGCGUGGGAGGCAGACGUUGCAGUAAGCUGAAAUUGCGCCACUGUGCUGC

>P186

ACCCAGGGCCCAGCGGCCCCCUCGGAGCCCGUGGCAUCCCGGGAAUUAAAG

>P187

AGGAUGACAAUUAACACCUUAAUUUAUAACAAUCUAGUUCAGAUUAACACC

>P188

AAUUUAUAACAAUCUAGUUCAGAUUAACACCAACUUAAUUUUAGUAGUACA

>P189

AGGCAUGGACUAUGGUGCCCAGCCUAGGAGCCAACAUUAUUAUUAUUAUAG

>P190

GGGGUCAGAAAUCCCUUCUGAGGCCAGGUGUCGUGGCUCAUGCCUGUAAUC

>P191

AGUAUCGUAUUUUAGAAGGGGAGAUAGAGGCCGGGUGCGGUGGCUCAUGCC

>P192

AUGAAAUUAAGGUUUUACCAUCUUUAUUUCAAUAAUUACUGUGGUGAGUGC

>P193

AUUAAAAAUAGCGGGGCAUGGUGGCACAUGCCUGUGGUCUCAGCUACUAGU

>P194

GAGAAGCCCUGUCUCUACUAAAAAUACAAAACUAGCUGGGCACGGUGGCGC

>P195

GCAGGAAAAUCGCUUGAACCUGGGAAGUGGAGGUUGUGGUGAGCUGAGAUC

>P196

AAAAAAUAAAUGUAUUUUUUGAGACAGGGUCUCGCUCUGCUGCCCAGGCUA

>P197

UACACUACUAAAGUGUAUUUCUACCACCACCCCCGGCUAAUUUUUGUACUU

>P198

AGUGAGCUAUGUUGGCCAGGCUGGUAUGGAACGCCUGGCCUCAAGUGACCC

>P199

CGGCGAGCCCGGCCACAUGUCGUUCACGUAGUUGGACAUCUUCACCUGCGG

>P200

UCACACCUCAGCUUCCCAGGUAGCUAGGGCCACAAGCACACACAACCACAC

>P201

CUGUAGUCCCAGCUACUCAGGAGGCAGAGGCAGGAGAAUGGCAUGAACCCG

>P202

AUUUUAUUUUUUAUUUUGUAGAGACAGGGUCUUACCAUGUUGCCCAGGGCU

>P203

CUCAGCCUCCCAAAGUGUUGGUAUUACAGGUGUGAGCCACUGCAUCCGGCC

>P204

UCACUACGUUGCCUAGGCUGGUCUCAAAUUCCUGGGCUCAAACGAUCCUCU

>P205

AGUUCAAGACCAGCCUGGGUAACAUAGCAAAACCCCAUCUGUACAAAAAAA

>P206

UGGGCUUGAACUCCCAACCUCAGGUAAUCUGCCCACCUUGGCCUCCCAAAG

>P207

UAGGACAUUUCCUGCCGGGCUCAGUAGCUCAUGCCUGUAAUCCCAGCACUU

>P208

CUCACGCCUGUAAUCCUAGCACAUUAGGAGGCCGAGGUGGGCAGUCCACUU

>P209

UCCACUUGAGGCAGGCCAGGAGUUCAAGACCAGCCUGGCCAACAUGGCAAA

>P210

AACCUCCACCUCCCAGGUUCAAGCAAUUCUUCCGCCUCAGCCUCCCAAGCA

>P211

CUUGGGAGGCUGAGUGAGGAGGAUUACUUGAGCCUAGCAGGUCGAGGCUGC

>P212

UCUGGGUAUGCUGGUAUGCACACCUAUAGUCCCAGCUACUCAGGAGGUUGA

>P213

AAGUAGGCCGAGAGCAGUGGCUCACACUUGUAAUCCCCGUGCUUUAUGAGG

>P214

GCUUUGAAAACCUUGGAAACAGGCCAGGCACGGUGGCUUAUGCCUGUAAUC

>P215

GCCAGGGGACAUGGACAUAUGUGAGAGAGAAUGAGUGGGACGUGGUGACUG

>P216

AGCUUCGUGCUGCCUGAUGACAGCCAGGCCAGCCGCCAGCGUACAAGGGUU

>P217

AAGCCCUCCUGGAGCAGCCGUGCGAAUGGGUGGAUGGCCUUCUACCCCUCA

>P218

GAAGCCAGGAGUUCAAGACUAGCCUAGGCAACAUAGUAAGACCCUGUCUUU

>P219

UACCAAAAAAUUAGCCAGGCAUAGUAACAUGCACCUGUAGUCCUAGCUACU

>P220

CAUGCACCUGUAGUCCUAGCUACUCAGGAGGCUGAGGUGGGAAGAUCACUU

>P221

CACUUGAACCCAGGAGUUGGAGGCUACGGUGAACUAUUAUUGUACCACUGC

>P222

AAUACAAAAUUUAGCCGGGCAUGGUAGCAGGCGCUUGUAAUCCCAGCUACU

>P223

AGGCUUUUGAAGCUGUGGACAAAGCAUAAAAGUUGCUACUGGAUCAGGACC

>P224

CAUAAAAGUUGCUACUGGAUCAGGAACAAAAGAAGAGGACCCUGGAUGUAA

>P225

AUCAGUUCAAAACAAAACCUAGGCCAGGCAUGGUGGUUCACGCCUAUAAUC

>P226

AGGCCAGGCAUGGUGGUUCACGCCUAUAAUCUCAGCACUUUGGGAAGCCAA

>P227

AGCACUUUGGGAAGCCAAGGCGGUCAGGAGUUUGAGACCAGCCUGACCAAC

>P228

UUUGUUUUGUUUUGUUUUUGGAGAUAGGGUCUCACUCUGUUACCCAUGCUG

>P229

UGGCUCAGAGCAACCUCUGCCUCCCAGGCUCAAGCAAUCCUCCCACUUCAG

>P230

CACUUCAGUCUAAGUAGCUGGGACUACAGGCACGUGCCACCAGGCCCAGCU

>P231

UUUGUUAGAGAUGAGGUUUUGCCAUAUUGCCCAGGCUCGUCUUGAACACCG

>P232

UACUAAAAAUACAAAAUAACUGGGCAUGGUGGUGUGCACCUGUGGGAGGCU

>P233

GUGUGCACCUGUGGGAGGCUGAGGCAGAAUUGCUGGAACCCGGGAGAUGGA

>P234

AGCCUGGUCAACAUAGCCAGACCCCAUCUCUAUAAAAAAAUGUAAAAAUUA

>P235

GGGAGGCUGAGGCGGGCAGAUCACAAGGUCAGGAGAUCAAGACCAUCCUGG

>P236

GAGUGCAGUGCAGUGGCAUGAUCUCAACUCACUGUGUCCUCCACCUCCCGG

>P237

CACCUCCCGGGUUCAGGGCUGCCUCAGCCUCCCAAGUAGCUGGGACUACAG

>P238

UUGCAGGGGUAGUGGUCAAUUCCAGACACCAGAGCAUGGCUGUAGGGGCAA

>P239

GCUGAGUGUGGUGGCUCAUGCCUGUAAAUCCAGCACUCUGGGAGACUGAGG

>P240

GGGAGACUGAGGCAGGAGGAUCACUAGAGCCCCGGAGUUCGAGACCAACCU

>P241

GGCAACAUGGAGAAACACCAUCUCUACAAAAAAGUAAAAAACUAGCUAGGC

>P242

UUCACCAUGUUGGCAGGAUGGUCUCAAACUCCUGAUCUCAGGUAAUCUGCC

>P243

UAUGUGAUCCUGGGCAAGUCACUUAACCUCUUUAGGCCUCCAUUUCCUUGU

>P244

CCUGUUCUUGACGUUCUCAUGAAAUACUUCAGGAGAGAGAACACACCCUAC

>P245

GAACCCCUUCCCUACAAAAAUACAAAAAUUAGCCGGGCAUGGUGGCAUACG

>P246

AUGGCAACAACAUGUUUGGUGGAUAAAACAGCACUGGCCAACUAAGGUAAA

>P247

UACCUUAGUUGGCUAGUGCUGUUUUAUCCACCAAACAUGUUGUUGCCAUUG

>P248

AACAUGUUGUUGCCAUUGAACAAUUAUAGCAGAAGUGCAAUUUAGGUACAG

>P249

CCUCAUUUCUUUUCUUUUCUUUUCUAUUUUUUUUUUUUUUUUUUUGAGAUG

>P250

CUCAGGAGGCAGGGGAAGGAGGAUCACUUGAGUCGAGGAGUUCAAGGUUAC

>P251

GAGUCGAGGAGUUCAAGGUUACAGUAUGCUAUGAUCACAACACUGUACUCC

>P252

GCCAGGUGUGGUGGCUUGCACCUAUAAUCACAGCAUUUUGGGAGGCCAAAG

>P253

GAGAUGAGGUCUCACUAUGUUGCCCAGGCUAGUCUGGAACUUCUGAGUUUG

>P254

AAAUGUUUAAAAAUUAGCUGGGCAUAGUGGUGUAUGCCUGUAGUCCCAGCU

>P255

ACAUUUAUAAAAACCUAGACGGGGCAGUGUCCUCCCCAGCCCAGGUGCCAC

>P256

ACAAGAGACUAAAAACAACAGGGGAAGGCUGGACACUCAAGGUUUGGGAGU

>P257

GAAGGCUGGACACUCAAGGUUUGGGAGUAUAAGCACCCCACUUCUGGCUCA

>P258

AAACCUACUUGGAAAAGAAUUGGGGAAGAAAACCAACAACUGCCUUAUGCA

>P259

AAGAAAACCAACAACUGCCUUAUGCAGGGGUGGGGACAGGGAAGGAGGUAG

>P260

GCCUUAUGCAGGGGUGGGGACAGGGAAGGAGGUAGGGCCAGGGACAGGAGC

>P261

GACAGGGAAGGAGGUAGGGCCAGGGACAGGAGCAUUUCACAUCACUAACCU

>P262

GGGACAGGAGCAUUUCACAUCACUAACCUAACUUGGGAAGCUGUAAGGGAC

>P263

CACUAACCUAACUUGGGAAGCUGUAAGGGACCAUCUUCAACUGGCCUUAAG

>P264

AAGGGACCAUCUUCAACUGGCCUUAAGAGGAGAACCAGAUGGCUGAUGGGA

>P265

CCUUAAGAGGAGAACCAGAUGGCUGAUGGGAGAAUCCACAGGAGGGAGAGG

>P266

GAUGGCUGAUGGGAGAAUCCACAGGAGGGAGAGGAGGAAAGGGAACGUGGC

>P267

CACAGGAGGGAGAGGAGGAAAGGGAACGUGGCUGGGAGGAGGCAAUAGCCC

>P268

AAAGGGAACGUGGCUGGGAGGAGGCAAUAGCCCCUUCCUUUCUGGGCACAG

>P269

UUUGAACCUGGGAGGCAGAGGUUGCAGUGAACAGAGAUCAUGCCACUGCAG

>P270

AAAAAAAUUAGCCAGGCUUGGUGGUACGUGCCUGUGAUCCCAGCUACUUGG

>P271

AAGCAACAAUAGUUACUUGUGGGCCAGGCGCGGUGGUUCACGCCUGUAAUC

>P272

GUUUAGGCCAAGCAUGGUGGCUCACACCUGAAAUCCCAGCACUCUGGGAGG

>P273

UCCUUCUGGAGUACAAACUUUUUUUAAGGCGGAGUCUUUGUCGCCCAGGCU

>P274

GUGCAGUGGCGCGAUCUCAACCUCCACCUUGCGGGUUCAGGCAAUUCUGCC

>P275

AGCCUGAGUGACAGAGCAAGACUCCAUCUCCAAAGAAAAAAAAAAAAAAAA

>P276

UUUUUAAUUGUGGUAAAAAAAAUACAUCUAACAUAAAAUUACCAUCUUAAC

>P277

GGUUAGGCUCAAACACCUAGGCUUAAGCAGUCCUCCUCCUCAGCCUCCUGA

>P278

UAUUUUUAUUUUUGACAUGGAGUCUAGCUCUGUCACCCAGGCUGGAGUGCA

>P279

UCAGUCUCCUGAAUAAGGUUAAACUACAGGUGCACACCACCAUGCCUGGCU

>P280

GCCUGGCUAAUUUCUUUUUAUUUUUAGAGACGGGGUCUCACUGUGUUGCUC

>P281

CAGGUGGGAAGAUUGCUUGAGCCCAAGAGUUUGAGACCAGCCUGGGCAAUG

>P282

CCCUUGAACCCAGGAGGCUGAGUGAACCAAUGUCGUGUCACGGCACUCCAG

>P283

AUGUCGUGUCACGGCACUCCAGCCUAGGUGACGGAGUGAGACCCUGUCUCA

>P284

AGUUCAAGAUGAGCCUAGGCAACAUAGCGAGACCCUGUCUCUGCAAAAUUU

>P285

GCCAGUGAGAAAUAGAAGGUGUAUUAAUUUGCUAGGACUGCUGCCACAACA

>P286

AAGUCCAAGAUCAAGAGGUCGACACAGUUGGUUCCCUCUGAGGCUUCUCUC

>P287

UCAGGACACCAGUCAUACUGGAUUAAGGCCCAACCCAAUGACCUCAUUUUG

>P288

CUCAUUUUGACUUAACUGACUUUUUAAAGACUCUACCUCCAAAUAAGGUCA

>P289

GAUUAAAAAAAAGAAAAAAGCCAUUAGGGUGUGUCCUAAUCUAAUAUGACU

>P290

AGCCAUUAGGGUGUGUCCUAAUCUAAUAUGACUGGUGUCCUUUAAAGGAGU

>P291

ACUGGUGUCCUUUAAAGGAGUGAUUAGGACAGGGACACAUAUACAGGGAAG

>P292

CAUAGGGAGAACACAGCCAAGUGCAAGCCAGCGAGAGAGACUUCAGAGUGA

>P293

GAGAGACUUCAGAGUGAAACUAACAAUGCCAACAUCUUGGUCUUGGCCUUC

>P294

AGCCACACAGUCUGUGGACUUCGUUAUGGUAGCCCUACCAAACUAAUACAC

>P295

ACCCUGGAAAAAUUCAGAAAGCAUGAAAAAAGUGAAAUGCAUUUGAAGUCA

>P296

UUUUACUUUCUUUCUUUCAUUUUUUAAGACGGAGUCUCGCUCUGUUGCCCA

>P297

AAACCCCAUCUCUACUAAAAAUACAAAAAAAAUGAGCCAGGUGUGGUGGUG

>P298

UGGCCUGACAAAUUAUGCUGCAGCGAAUUGUACUGGCCUGCUGCUGGCCCG

>P299

GCUGGGCUCAGUGGCUCAUGCCUGUAAUCUUAGCACUUUGAGAGGUCGAGG

>P300

AUUUUUUAUUUAUGUAUUUUUUUUUAAGAUGGAGUCUCACUCUGUCACCCA

>P301

AAUCCUACCUUUAAGAAUUUAUCCCAGCUAUCUGGAAGGCUGAAGUGGGAG

>P302

UACUCGGGAGGCAGAGUCAGGAGAAAUGCUGGAACCUGGGAAGCAGAGGUU

>P303

UUGUUUUGUUUUGUGGCACUAUCAUAGUUCACUAUAACUUCAAACUCCUGG

>P304

AGAGAUGGGGGUCACUAUACUGCCCAGGCUGGUCUUGAACUCCUAAGCUCA

>P305

GAGGACGGCUGGUGGCUGGGGAAGAAGAACGGGCAGCUGGGAGCCUUCCCA

>P306

UAUAGUUCCUGCUACUCAGGAGGCUAAGGUGGGAGGAUCGCUUGAGCCCAG

>P307

CUAUAAUUUAUUUUAUGAAUAAAUAAGAAAAAAGUCCCUGGCUAUAAAGGA

>P308

UGAGACUUUUUAUUUAUUAAUUUUUAUGAGACGGAGUAUUGCUAUGUUGCC

>P309

AGGCUGAUUCUGAACUCCUGGGCUCAAGCAGUUCUCCCGCCUGGGCCUCCC

>P310

AGCAGUUCUCCCGCCUGGGCCUCCCAGAAUGUUGGGAUUACAGGCAUGAGC

>P311

CGCCCUCGCGGCACCGGAGUCAAGGAGAAGGGAACGAAUCCCCAAGGAAGA

>P312

CUGCCGGGACCCCGGCCUUCCCUUCAGCACCCAUCAAAGUUAACACAGGCA

>P313

CUCCCUCUCUCACCCGGGCUGGCGUACAGUGAUGUGGUCAUAGGUCACUGC

>P314

UGGGACCACAGGCAUGCAUCACCACACCCAGCUAAUUUUUUAAAAAUGUUU

>P315

GCUAAUUUUUUAAAAAUGUUUUUGUAGAGAGGGGGUCUCCCUGUGUUGCCC

>P316

CUCAGUCUCCCAAAGUGCCGAGAUUACAGGCGUGAGCCACUGUGCCUGGUC

>P317

AUGAUGCCCAAUGCCAAGCAACCAAAGACGCUGGAACUAUUGCUGACCUAA

>P318

UUAUGAGGAUCAUCAAUAAGCCUACAGCAGCUGCUAUUGCUUAUGGCCUGG

>P319

CAGGCGCAUGCCACCAUUCUCAGCUAAUUUUUGUAUUUUUAGUGGAGAUAG

>P320

UUUAGUGGAGAUAGGGUUUCAUCAUACUGGUCAGGCUGGUCUCAAACACCU

>P321

UUCUUUGAGAAAAAAAAUUUUUUUGAGACGAGGUCUUACUCUGUUGCCCAG

>P322

GAGCUGGGCAUAGUCCCAGUUUCUAAGAAGGCUGAAGUAGGAGGAUCGCUU

>P323

UCCGAGUAGCUGGGAUUACAGGUGCAUGCUACCAUGCCUGACUAAUUUUUG

>P324

ACCACCACGCCUGGCUGAUUUUUGUAUUUUUGUUAGAAACAGGGUUUUGCC

>P325

UUUUUUUUAAUUUUUUUUAGAGACAAGGUCUUGGCUGUUGCACAGGCUGCA

>P326

CAGUGGUGUAAACAUGGCUCACAGCAGCCUCAAACUCCUGGGCUCAAGAGA

>P327

ACUCCUGGGCUCAAGAGAUUGUCCCACUCCAGCCUCCUGAGUAGCUAGGAC

>P328

ACUAUAGGUGUAUGCCACUACACCCAGCUAAAUUUUUGUGUUUUUUGUAGA

>P329

GCUGGUCUUGAACUCCUGACCUGAAAUGAUCCUUCUGCGUUAGUCCUCCAA

>P330

UGAUCCUUCUGCGUUAGUCCUCCAAAGUGCUGGGAUUACGGGCAUGAGCCA

>P331

CAGUGAGCCGAGAUUGCACCACUGCACUCCAGCCUGGGUGACAGAGUGAGA

>P332

GGUGACAGAGUGAGACUCUGUCUCAAAAAAAAAAAAAAAAAAAAAAAAAAA

>P333

UAUAUAAUUUUUUUUUUUUUUUUUUAGUAGAGACGGGUUUCACUGUGUUAG

>P334

UGAAAUAGCCUGACACUUGGGGGCAAGUGGGUCAUUAUUCGGGAGACCCAG

>P335

AGUUGCAGUGAACUGAGAUUGUGCCAUUGCACUGCAGCAUGGGCAACAAGA

>P336

GUGCCUGACUUAAUUCUACUUCAUAAGGUUGAUGUAAUUUUGAGACUUAAA

>P337

UUUGAGACUUAAAUAAGUUGAUACUAAAAUACCAAGCAGUGUCUGGGACAC

>P338

AAUACCAAGCAGUGUCUGGGACACUAAGUGCUAUGAUGACCACUUCAGGGA

>P339

UGGGACACUAAGUGCUAUGAUGACCACUUCAGGGAAGCUCAGCAGGAGAGC

>P340

GUACAGGCCACACCAGGGGAACUGCAGAAUCCUGGGCUGGGAAUUAGACUU

>P341

GGACCUGAGGUCUCCUUCCCAGCCCAGGGCUUUCUACAGCUCCCCUGGUGU

>P342

CCCUGAUGUGAUCAUUAUAGCACUUAGUGUCCCAAACACUGUUUGACAUUU

>P343

CCCAAAGUGAAAGUAUGUACCCAUUAAAUACUAACUCCCCAUUCCCUUUUU

>P344

UGUUUUAAAAAUUAUUUGAGUGGCCAGGCAUGGUGGUUUACACCUGUAAUC

>P345

CCACCACCACACUCAGCUAAGUUUUAUAUUUGUAGUAGAGAUGAGGUUUCA

>P346

AGGGUCUCACUAUGUUUCUCAGGCUAGUUCUAACUCCUGGGCUGAAGCAGU

>P347

ACCGAGAUCGCUCCACUGCACUCCAACCUGGGCAACAAGAGCAAGACUCCA

>P348

CCUUGUUAACAAGAUGUUUACGAGCAGUAUACUUGGUAAAAGUCAUUGCCA

>P349

CUGACUGUCCCCCAGCCCAACACCCAUAAAGAGUCUGUGCUGAGGUGGAUU

>P350

GAACGUUUGCCAAUUGUGGUCGCUCAUGCUUAUAAUCCCAGCACUUUGGGA

>P351

AGAAUUGCUUGAGCCCAGGAUUUCAAGACCAGCCUGAGCAACGUUGUGAGA

>P352

AUGUAAAAUUAAUGCAGUUCCCGGUAAGUUUAAUUCUUUGUUUUGUGUUGA

>P353

CAAUCAUGGCUCAUUGCAGUCUCCCAGCUCCUCGGCUCAAGUGAUCCUCCU

>P354

ACAGGAUACUGCCAGAAACUGGGCAAGAGGCUGUUUGUGAAUCUCAGAAGG

>P355

CAGUGGCGCAAUCAUGGCUCACUGCAGGCUUGACCACCCAGGCUCAAGCAG

>P356

CCUUAUACAAUAGCUAUAAGUAGCUAGGACUACAGGUGCAUGCCACCACGC

>P357

CACUGUGUUACUCAAGCAGUCUUGAACUCCUGCCCUCAAGUGAUCCUCCCC

>P358

AAACACUAUAUUUUGAAGGCUUAAUAUCAAAUGCAAAAUAUGGAUUAGACU

>P359

CAACAUAGAGAAAGGUCAGCUUGUGAAAACCCAAAGACUAAAGAUUAUGGA

>P360

CCAGACCUUAUUUAAAAAAAAAAAAAGGAUAUCUGUGAUUACAAGAUUACC

>P361

AAAUUAGCUGGGUGUUGUGGUGCGCACCUGUAAUUCCAGCUACUCGGGAGG

>P362

UCCUUUAGAAAAGUAUCCAGUAAUGAGAUUGCAGGAUUGUAUGGUAGUUCU

>P363

CCAAUGUGGCAAAACCCUGUCUACUAAAAAUACAACAACAACAACAAAAAA

>P364

CCUGCCUCAGACUCCUGAGUAGCUGAGACCACAGGUGCAUUUCACCACUCA

>P365

UUUUGUAGAGAUGAGGUCUCUACCAAGCUGGUCUCUAACUCCUGGGCUCAA

>P366

ACUCCUGGGCUCAAGCGAUCUUCCCAUCUUGGCCUCCCAAAGUGCUGGGAU

>P367

GCUGGGAUUACACAUGUGAACCACUAUGCUUGGCUCAAAGAUUUAUUGAUA

>P368

GUCAGAAUUUUUGGAAGAAAAUUUUACUGGAUCUUUCUGCUGCUUCUCACA

>P369

AGUAGGCAACUGGAUUUUCCAGCUAAUAUUUAUAGCUCAAUUAACAUGUGC

>P370

AGGACCUUUCUCUUUCACCCAGACUAGAGUGCAGUGGCUCGAUCACGGCUC

>P371

UGAACUCGAGUGAUUCUUCCACCUCAGCCCCCCGAGUGGCUGGGACCACAG

>P372

UCUUGUAAAGACAUUGUCUCACUAUAUUGCCCAGGCUGGUAUUGAAUUCCU

>P373

CUCUUAGGAGGUAGCCACAUUUGGUAUCAGGCUGGCUGGGAGAAGGGCCUA

>P374

AGGGCCUAAAGGGUGUGUGAAAUGUAAACAAGGGCUUCCUGACACUGCUCU

>P375

GAAAUGUAAACAAGGGCUUCCUGACACUGCUCUGACUUGUGCUAAACUUUC

>P376

UCCUGACACUGCUCUGACUUGUGCUAAACUUUCUGGGGGUGGGAGACCCAG

>P377

UUGGAUUCAUGGAUUGGGUAUGACUACUCUGGGUCUGCCACCCCCAGAAAG

>P378

UGGGUCUGCCACCCCCAGAAAGUUUAGCACAAGUCACAGAGCAGCGUCAGG

>P379

GAGCAGCGUCAGGAAGCCCUUGUUUACAUUUCACACACCCUUUAGGCCCUU

>P380

CUUGUUUACAUUUCACACACCCUUUAGGCCCUUCUCCCAGCCAGCCUGAUA

>P381

AGGGAUUGAUUGAGCCCUGGAAGGUAAGGCUACAGUGAGUCAUGAUGGGUG

>P382

GUGAGUCAUGAUGGGUGGCAGAGCAAGACCCUGUUUCUUUCUUUCUUUCUU

>P383

CAGCCUCCCAAAGUGCUGGGAUUACAGGCAUGAGCCACCUCGCCUGGCCGA

>P384

AAGGGGAGGAGUCAAGGGGAAAGAUAGGAAUUAAGAUGCCUCCCAGGUUUC

>P385

GUCCCUCCUGUUGUCUGCUUCCAUCAAGAGGGAAGGAGAGUCUCCAACGGC

>P386

GUCUCACUUGGUUGUCCAUGCUGGAAUACCAUGAUAUGAUCGUAGCUCACU

>P387

AGCAAGUAGUCUGUGGCUUAGACCAAGGCAUUUGAAGUUUCUCCUUGCUGA

>P388

CAGCCUGCUGAAUAGCUGGGACUACAGGCAUGUUCCAUCACAUUCGGCUAA

>P389

GGCUAACUUUUUUUUUUCUUUUUGUAGAGAUGAGGUCUCCCUACGUUGCCC

>P390

GCAGCAUCCUGUUCAUCAUGAGCAGAGGGGUGACUGAAUAGAAUGGGAGGC

>P391

GGGGUGACUGAAUAGAAUGGGAGGCAGGUGUGGCCUGAGCAGUUCCCAACC

>P392

CCCACAAAUCACUAAGCUGAAGGGAAAAGUCAAGCUGGGAACUACUCAGGG

>P393

GAAGGGAAAAGUCAAGCUGGGAACUACUCAGGGCAAACCUGCCUCCCAUUU

>P394

UCAAAUGAGUUUUUUUUUUUUUUUUAGACAGAGUCUUACUCUGUCACCUAG

>P395

AAAAAGACUAUCUCUAAUCAAGGCUAGAACCAAGGGAAGGCUAAGAAUUGC

>P396

AAUUUUGUCGUGGGCUGGGCGCAGUAGCUUCACACCUGUAUCCCAGCACUU

>P397

GGGGUGCAGUGGUGCGGUCUCGCUCACUGCAACCUGCAUCUCCCAGGUUCA

>P398

UCGAACUCCUGACCUCAGGUGAUCCACUAGCCUCAGCCUCCCAGAGUGCUG

>P399

UAGGAAAAAUUAAAUUGAUAUUGUUAUCUUCUAUUCUUACCUGGUAUCUCU

>P400

CAGCCUUUUAAUUGCUGAAGGUCUGACCCAUAGACUAAGAUUGCACAUUCU

>P401

UUUUACUUGUAGAAACUGAAGGCUCAGAAGAGGAUGAUAAAGAAAAUGAUA

>P402

AAAAAUACAAAAAUUGGGGCCAGGCACGGUGCCUAAUGCCUGUAAUCCCAG

>P403

UGCACUCCACCCUGGGCGACAGAGCAAGACUCCGUCUCAAAAAAAAAUUGG

>P404

AGUGAGGAAGUGGGCCAGGUGCAGUAGCUCACAACUGUAAUCCCAGCACUC

>P405

GGUGGCAGGUCCAGACCCCAUGUCUAGAGAAUGUCAAAUGCCUCCUGUACC

>P406

UCUACCCUCCCCUCCCAGUGCUUUGAGAGGCUGGGGUGGGAAGAUCACUUG

>P407

AAACUGAGGUCUGCUGUUUUGGGUUAUGCCCACAAGAAGCACCAGUCAGGG

>P408

UUGCCUCAGCCUCCUGAGUAGCUGAAAUUACAGGCGCACGCCACCUAAUUU

>P409

AAAAAUGCAAAAAUUAGCCGGGUAUAGUGGUGCACACCCAUAGUCCCAGCU

>P410

UGAUCCCAGGGAGUUUGAGGCUGCAAUGACCUAUGAUCACAUCACUGUACU

>P411

CAUCAAGAUGUAGAAAAUAGAAAAUAGGCUAGGCACAGUGGCUCACGCCUG

>P412

UAAAAGUACAAAAGGGCAUGAUGGCAGGUGCCUGUAAUCUCAGCUACUCAG

>P413

CAGGUGCCUGUAAUCUCAGCUACUCAGUAGGCUGGAGCGGGAGAACUGCUU

>P414

UGCCUGUAAUCCCAGCACUUUGGGAAGCCAGGGUGCAAGAAUUACUUGAGC

>P415

AAGAAUUACUUGAGCCAAGGAGUUCAAGACCAGCCUGGGAAACAUCAAAAG

>P416

CACCACACCUAGCUAAUUUUUUUCUAUUUUUUGUGGAGACAGGGUCUCACU

>P417

UCAAUGAGCAAAGAUUAAGACUUCAAGCCUGGCAUUGUGGCUCAUGCCUGU

>P418

CAAAGCGGGAGGAUUGCUUGAGUCCAGGAGUUCAAGGUUACAGUGAGCUAU

>P419

GGAGGCGGGAGGAUUGCUUGAGCCUAGAAAUUGGAGACUAGCCUGGACAAC

>P420

ACCAGACCAGGUGGCUCACAUUUGUAAUUUUAGCACUCUGGAAGGCCGAGG

>P421

GAUAUAUUUGUUUGUUUAUCUCUUUAUUGACUGUCUACCCUAGAGUACAAU

>P422

UCUUCAAAGUCAUGGAUGUUCUUUAAGCCACCCAGUCUAUGGUAUUUUGUU

>P423

CACCAACUUUGGGACACUUUCUACAAUCUUCCAUCCUCCAAAGGCAGUGCC

>P424

AUUACAGGCACGAAACACCAUGCCCAGCUAGUUUUUGUAUUUUUAGUAGAG

>P425

AUUUUGUAUCCUACAACUUUACCGAAUUUGUUCAUUAGCUCUAACAGGUUU

>P426

ACCAUAUCUGGACAGCCAACGAGCCACAUGCAGGUGUCAUUAUGAAGAAAA

>P427

ACUAAUGUUUUUACAACAUAAAACUAUCUGAUUUUUGUGAAAAAAGAAAAA

>P428

AAUUUGGGGCCAAGCACGGUGUCUCACACCUCUAAUCCCAGCACUUUAGGA

>P429

GUAUGGUGAAACCCUAUAUCUACUAAAACUACAGUAAUUAGCAGGGUGUGG

>P430

CUGGGACAGGCCCUUCUCAGGAGCAAGCAUACGACCCAUGAUGGCGGCGAU

>P431

UAUUCAGAAGCAUUUUAAUUUAGAAAGGAGCUGGUUUGUUAGUUCACUGAU

>P432

CUACUUUUUUUCUUUUUGUAGAGACAGAGUUUCACUCUGUCGCCCAAGCUG

>P433

CAGUGGCACAAUCAUACCUCACUGCAGCCUCUUGGGCUUAUGUGAUCUUAC

>P434

GUGACUUUCCCACCUCAGCUUCCCAAAGUGCUGGAAUCACAGGCAUGAGCC

>P435

UUUCAUAGACUUUGCAUGAUAUGGUACACUCCUAAUUAUGCAUUCUUUGGU

>P436

UCCUUUCUAAGGAUAUAAAAAAUUCAUUGGAAAGUGUGUAUAUUUCAAAGA

>P437

GUUUUGGGAUUUUUUUUGCCUAAAUAAAUGUUAUAAAUUUUAUGUAAAGUG

>P438

UAUCAGUUAUCUAUUGUUGUGAACAAACUAUAGCUUAGUGGCUUAAAACAG

>P439

AGAUCUCAGAGAAGAGGGUCUAGCUAAGUCAUGCUGAGACUCCUGCCCCAC

>P440

UGAAAUUCUUGGUAAUUAUUUAAUCAUUUAUUCACACUUUUUUGUUUCUCC

>P441

UUAGAAGUAAAAAUUGUUUCUAUAUAAUGAUUACAACUGUAUGCAUGCCUA

>P442

AGUGAUUCUCUUGCCUCAGCCUCCCAAGUAGCUGGGAUUACAGGUGCUCAC

>P443

GGUGCCAAGAUAAUUGAAAGGGAAAAGAAUAGUCUUUUCUGUAUUUUUUCU

>P444

AAAUAAACAAAACACAAUAGACAAAAAUAAAGAUAAGUUGAAAUUUAUUUU

>P445

AAUUUAUCCUUUUUGUCAAUUAGAAAAAUAAAUUUUAAUUUAUCUUUUUAU

>P446

AUACACAUAAACAUCUCAAUGCCUUAAAGAGCAGUAUUGCUGCCCGCAUGU

>P447

UUAGUGACCGAGCUGGUCUCGGCAGAGGUGGGCGGGUCUUUUGAGUUCAGG

>P448

UGUGCCUCUGUACUUUCAGCUACUCAGGAAGCUGAGGCACAAGAAUUGCUG

>P449

AGAUCAUGCCACUGCACUCCAGCCCAGGCAAUAGAGUAAGACUCUGUCUCA

>P450

CAACUAAGAAACUCUUUUGUGAGACAGCCUUGCUCUGUUGCCCAGGCUGGA

>P451

UUGGAGGUUUUAUUUGUUUGUUUUUAGAGACACUUUCACUGUGUUGCCCAG

>P452

AUGCUUGUAAUUUUAGCACUUUGGGAGGCUGAAGCGGGAGGAUCGCUUGGG

>P453

AAAAAGGAGCUAUAUGAGAGCAACAAGCAUUUCCUGGGGCCCUACAACCCU

>P454

CACUGUGAGGAUUAAGGGAGAUGAUAUAGGGGAAAGGCCUGUCAUAUGGUA

>P455

AAAGGCCUGUCAUAUGGUAAAAGCUAAGUAAAUGUGCCUCACGUCCUAGCU

>P456

CUAGGUCUUAAGCCCUACAUGCAUUAGGUAUUUCUCCUAAUGCUAUCCCUC

>P457

AAAAACAGAAACGUAGGCUGGGUGCAGUGGCUCACACCUGUAAUCCCAGCA

>P458

GUCAGGGAUUCCAUAUACAAAUUUGAAAGGGGAAGCAUUUCAUUCCAGAGC

>P459

UGGGCCCACACAAUCCUCCUACUUCAGCCUCCUAAGUAGCUGGGACUAAAG

>P460

GAGAUAGAGUCUCACUACAUUGCCCAGGCUGGUCACUGAACUCCCAGCUUC

>P461

AAAAAUUUUUUUUUUUUAGUCAGCCAGGUGUGGUGGCCUUUGCCUGUAGUC

>P462

GGAGAUUGAGGCUUCAGUGAGCCUCAGUUGUACCACUGCACUCCAGCCUGG

>P463

CUGCAACCUACACCUCCCGGGUUCAAACUAUUCUCUUUGCUCAGCUUCCUG

>P464

UUGUAGUCUAUGUAUAAGUUUCUGAAGUUGCAUGCAGAAUUUAGCCUAUAU

>P465

AAAAAUGAUGAUCUUCAGGCCUGUAAUCCCAGAACUUUGGGAGCCGAGGCG

>P466

GAGGCUGAGGUGGGAAGAUGGUUUGAGUCCGGGAGGUGGAGGUUACAGUGA

>P467

CACUGCAGCCUGAAACUUGAGCUCAAGCAAUCCUCCCAUGUCAGCCUCUCA

>P468

AGCAAUCCUCCCAUGUCAGCCUCUCAAGUAGCUGGGACUACUGGCAUGUGC

>P469

CUUUCUCUGUCACCCAUGCUGGAGUACAUUGGUGAGAUAAUGGCUCGCUGC

>P470

UUAUUUAUUAUUGAGACAAGAUCUUACUCUGUUUCCCAGGCUGGAGUGCGG

>P471

UAUUAUUAUUAUUAUUUUUUGAGACAGGAUCUCACUCUCUUGCCCAGGCUG

>P472

ACUCUGAAACAAGGUUUUGAGUGCAAGUAUUAUAGUUUAUUUGGGAGGUGA

>P473

CUAGACUAAAGGUCCAUCAGCCUUUAGAAGAAGCCACAGUGGUUUUCAUUU

>P474

GAGUUCAGUGGUGCAGUCUUGGCUCACUGCAACCUCUGCCUCAUGGGCUCG

>P475

AUCUCUACAAAAACUUUUUAAAAUUAGGAGAGCAUAGUGGUAGAGACCUGU

>P476

AGACUAGGAAUUCUCAACAUAGCGAAACCUCGUCUCUACUAAAAAUACAAA

>P477

CCUUCAGAAGAUCAGAGAAUGAAAUAACACUUUAUAUGCUCUUGGUACUCC

>P478

CAAUCCUCCCACCUCAACCCCCCAAAAUGCUGGGUUUAUAGGUGUGAGCGA

>P479

CUGAGCCUCCCAAGUAGCUGAGACCACUGACGCGCACCACCACGCCCAGCU

>P480

UAGAUAUACUAAGAACUGCCUUUUUAUUCCUAAAAUCUUGAAUUUUCUAAA

>P481

CACUUAAACUCAGGGUCUCUACAAAAAAUUUAAAAAUUAGCCAGGCAUGGU

>P482

AGCUGGGUCUGGAAGAGAUAUUUCUAUUUCCGUGUUCAUAGCAACAUUAUU

>P483

CAGUGGUUCUUAACCCUGUUUUAUAAUAGGAACACCUAGAGAGCUUUAAAA

>P484

GGGGAUCCUAGCAUCUGUAAUUUUUAAAAGCUCCGUAGGUGUUCCUAUUAU

>P485

GAUGAUCAGUUGAGGCCCAGAGUUCAAGACCAGUUCCGGCAACAUAGUAAG

>P486

AAUAUAAAGUGGUUUAUAGGUGGCUAGGCGCGGUGGCCCACGCCUGUAAUC

>P487

GACAGUUUUACUCUGGAGUGCAGUGAUACGAUCUCUGCUUAUUGCAACCUC

>P488

AGAAUUUUACUGGCUGCAGUGGCUCACGCCUGUAAUCCCUGCACUCUGGGA

>P489

GAGCACAUGCCUAUCUAUAAUCCCAACACUUUGGGAUAUCGAGACGGGCGG

>P490

GCUACUCCGGAGGUUGAAGCAGGAAAAUUGCUUGAACCUGGGAGGUGGAGG

>P491

GAUCAUUAAAAAGUCAGGAAACAACAGAUACUGGAGAGUUUGUGGAGAAAU

>P492

CUCCUAAUGCCCUUGAGAUCCAGGUACACUCCUGGGAGUUUUGUUCACCUC

>P493

GUCCUAGUAAGUUGAAGUCCUAGUAAGAAAUACUCAGUAUAUUGGCCAGGC

>P494

UUUCUCCAAAUGCAAUCUGUUGGCUAGGCACAGUGGCUCACGCCUGUAAUU

>P495

AUCUACCUUUCUGGCUGGAUGCGGUAGCUCACACCUGUAAUCCCAGUACUU

>P496

CUGUGGAUGACAGCUCUGACAGCUGAUGGUGAAAGUUCCCACGGAAAUGAG

>P497

AUUGGUUGAGUUUGUCUAAAGACCUAGAAUUAACAGAAAGGAAUGUCUGGC

>P498

GAAACAGUCUCUGCAGUUAAAUUUUAAAAGAGCCGUGAAUGAGGAGGAAGU

>P499

ACACUACUAUCAUUGAAACUGUAUUAGCCCAUUCUCACACUACUAUGAAGA

>P500

CAUUUCAUAUGGCUGGAGAGGCCUCAGAAAACUUACAGUCAUGGCAGGAGG

>P501

AGAAAGACUGGAUGUGAGUAUGUAUACAUAUGUGUGUGUGUAUAUAUAUGU

>P502

UGUGUGUAUAUAUAUGUAUACACAUAUAUACACACACAUAUAUGUAUAUAU

>P503

UAUAUAUGUAUGUGUAUAUGUGUGUAUAUGUAUACACAUGUGUACAUAUAU

>P504

UAUAUAUGGGUGCGUAUAUAUGUGUAUACACAUAUGUAUGUAUACAUACAC

>P505

UACACAUAUACACACAUAUAUACACACGUAUAUACAUAUAUACAUAUAUGU

>P506

AAAGUCAGUUACUACUAGGUAAGGUAAGUGGGUACACACAGUUUUCUUUAA

>P507

AUCCCUAUAUACCACCUCUUUGGUUAUAUCAUCAAUCACUGCGUUUACAAC

>P508

GCCUCUUUGUGCCGAGCCCUCAUCUACUGGAUGUUACUCACAUCAAUCUCA

>P509

CAGGGAUUCAGUUUCUUCCUGGUUCAGUCUUGGGAGGGUGUAUAUGUCCAG

>P510

CUGGUUCAGUCUUGGGAGGGUGUAUAUGUCCAGAAAUGUAUCAAUUUCUUC

>P511

GGUGUAUAUGUCCAGAAAUGUAUCAAUUUCUUCUAGAUUUUCUAGUUUAUG

>P512

CUAGUUUAUGUGCAUAGAGGUGAUCAUAAUAUUCUCUGAUGGUUGGUUGUA

>P513

AUAAUAUUCUCUGAUGGUUGGUUGUAUUUCUGUGGGGUCAGUGGUAAUAUU

>P514

GUUGUAUUUCUGUGGGGUCAGUGGUAAUAUUCCCCUUGCCAUUUCUGAUUG

>P515

AAUCUUCUCUCUUUUCUUCUUUAUUAGUUUAGCUAGUGGUAUGUCUGUUUU

>P516

UUGCUCUUGUCCAUUGUUCCCUGUCACCCAUUUAGAAGAGAACGCAAGGGU

>P517

UGUCACCCAUUUAGAAGAGAACGCAAGGGUCCAUAAAGUAAUAGCAGAAGG

>P518

UCCAAGUUUUGUCAAUUAUGCAUGAAGUUGCUGUCAACAUCCAUAUGCAGG

>P519

UGACAGUAUUUAUGAUGAAAUGCAGAAAAAAAAGGAGGAAAACAAUACUAA

>P520

AAAUGCAGGAAAAAAAGGAGGAAAAAAAUACUAAAUUGCUUUUGGGGAAAG

>P521

UAGCACAGAAGAGGGAGGAUACUGUAUCCAAAGAAGUGACUAGAAAACUUU

>P522

UGAUUGAGCCUUGGAGGUGGAGGCUACAGUGAGCUGAGAUUGUGCCACUGU

>P523

ACCUCGCCAUUGAGUAUGAGGUGUUAGUGGUGCCCACUGUGCUGGCCAUGA

>P524

UAUGUACAUAUACAUACAUAUAUGUAUGUGUAUGUAUAUGUAUGUAUAUGU

>P525

CAGAAUAUCCUGGCUAACAUGGUGAAACACUGUCUCUACUAAAAAUACAAA

>P526

CUUGCUCUUGAGCUGUGGAGGUGGAAGGCUGACCUCCAUCAUUACUGCCAU

>P527

UUAAAAUUAAUCAUUUGAUUAAUCAAAUUUGAUAAAUUCUUGCCAGUUAGA

>P528

GAAAUACUACAUGUUCUGUCUUAUAAGUGGGAGCUAAAUGAUGAGAACACG

>P529

CUAAUUUUUGUAUUUUUUGUAGAGAAGGGUUUUUGGCAUGUUGCCCAAGAU

>P530

GUAAAGCAAUGUAGAUUAUCUUAUCAGCAUGGAAAAAAUGCAAUUAUUAUA

>P531

CCCCCUGAUGGGGGAUGAUAGACCCAGACACAUAGAGGCCUGGUGUUGACC

>P532

UGCCUUUACUAAAAAUACAAAAAUUAACCAAGUGUGGUGGCACACGCCUGU

>P533

UGUUGUGAAUUCUAAUGCUUUCAGAAAAUAUUACGGAGAAUCCCACAAUGA

>P534

AGCACAGUGACAGUCAUAGCUCACUAUAGCCUCAGACUCCUGGCCUCAAGC

>P535

UUUUAAUUUUAUAUUUUUUAGAGACAGGGUGUCACUAUGUUACCCAGGUCU

>P536

CACUAUGUUACUCCUUGCCUCAAGCAAUCCUCUCACCUUGGCCUCCCAAAG

>P537

CAGCCUCAACUUCCCUGGGCUCAGGAGAUUUUCCCACUUCUGCCUUCCAAG

>P538

AGGCCACAAAAUAAGUCUCAUCAAAAUUUUAAAGGUAGAAAUCCUAUCAAA

>P539

GUAUUCUGGAUAAUAUUCCAUGUGCAGAUAAGAAUGUGAGCCUGGGCAACA

>P540

UGUGAGUCAAUUAAACCUCUUUUCCAUAUAAAUUACACAGUCUUAGGGCUG

>P541

GGGUAAUUUAUAAAGAAAAGAGGUUAAAUUGACUCACAGUUCCACAUGGCU

>P542

AUGCUGUACUGGCCUUGGAGCCAGUAGACUUGGGGGUAUGCAACCCAGCGA

>P543

CCCCAACCCAAGGCAGUGCAGCUCUAGGAGAGGCUUCUUCUGCUUGAGAGG

>P544

GGCUGCCCAUAACUUGGAGGAACUUAGAAGGCAAAACCUACUGCGCCCCAA

>P545

CAAAACCUACUGCGCCCCAACCCUUAGAGGGGCCUCAACCCCGAAGGCGAG

>P546

AACCCCGAAGGCGAGGGGCGAGAUCAGGGACUCGGCGACGAGGGCGAGCGC

>P547

AUUGGGUGACCAGCUGCAGAGAGGAACUCUCUGCUGAGAACUUCAGAGACC

>P548

GAGCUACCGUCUCCAGGGCUUCUCUACUGAGAACUGGACACUCGUUGGGGU

>P549

AUGAUCAUGGCUCACUGCAAGCUCGACCACUCGGGCUCAAACAAUCCUCCC

>P550

CAGCUCAUUGCUCCUGGGCUCAAGCAAUCCUCCAGCCUCAGCUUCUCGAGU

>P551

GAAUUUCUUGAACACACCAGGUGGAAGUUGCAGUGAGCCAAGAUCGUGCCA

>P552

UCUACAGAAAUUUGUUGGGCACGAUAGCUUGUGCAUGUAGUCCCAGCUAUU

>P553

GUCUGAGUCUGCAGUCAACUGGGAUAGCACCACUGCACUGCAGCCUGGGUG

>P554

AAUUUUUUAAAUUUUCUUGUAGAGAAGGGGUAACACUAUGUUGCCUCGACU

>P555

AACUGUAGAUCCAGGGGGGUUCAAUAUUUUGAUUAUCCAAACCUAGAUCAC

>P556

UUCAAUAUUUUGAUUAUCCAAACCUAGAUCACAUGCCUGUCCCUGAAAUCG

>P557

AGCUUAUGCUUUUUUAGUCAUUUUGAACCCAAAACAUCUCCUUAUCUUUUU

>P558

GGGCAACAUAGUUAGACCCUAUGUCAAAAAAAUAUAUAAAAAUAAAAUCUG

>P559

UCCUCCCUCAACCUUCCAAAGUGCUAGGACUGUAGGCAUGAGCCACUGCAC

>P560

AGGCAUGAGCCACUGCACCUGGCCUAAAAUUACAUUGUUACAGGCACGUAA

>P561

AAUCCCUGCUCCACCAAUUAGUAGCAGUGCCACCUUGGGCAAAUUACUUAU

>P562

GUCACCUAGGCUGGAGUGCAGUGGCAUAAUCACUGUUCAGUGCAGCCUCAA

>P563

UCCCAUCUCAACCUCUCAAGUAGCUAGGACUACAAGUGUGCACCACCAGGC

>P564

GCCCAGCAUGGUGGCUCACACCUGUAAUCCCAAGGCAGAAUGGUGGCUUGA

>P565

CCCAAAGUGCUGGGAUUACAGGUGUAAGCCACCGUGCCUGGCCUACAUGUU

>P566

GUGUAAGCCACCGUGCCUGGCCUACAUGUUCAAUUUUCUAUGAACAAAGGC

>P567

UCUUGAGACAGAGUCUCAUUCUGUUACCAGGCUGGAAUGCAGUGGCGUGAC

>P568

CUGUGCUCUGGGGGCUGUGCCGGGUAGAGAGGGCAGUGGGAGGUAAGAGCU

>P569

UGUGGGUGGCAGGUGGGGAGACAGAAGAGGAGAAGAUUCGUGUGGACAUUU

>P570

GGCCCAGACCAGGGAGGGACUUCCAAAGUCAGGUCUGUAGCAGACUCCGGC

>P571

GACGUCUAUAACCUGGUUCUCCAAAAUGUCCACACGAAUCUUCUCCUCUUC

>P572

CCACACGAAUCUUCUCCUCUUCUGAAGAUUCUUGUGGACAUUUUGGAGAAC

>P573

GGGUGAUUACCCUGUGUAUAAGAGUAUGUGUCUCACUGCACCUUCAAUGGC

>P574

AGCCUUCCAAAGUGCUGGGCGUACAAGCGCAAGCCACUGUGCCCAGCUGUC

>P575

UGCAGAUAAAAGCUAACUUUUAUUAAUACCAGCCCUGAAUAAUGGCACUAA

>P576

AGUGGCAAAUGCUACUGAGAUAGAAAGAUUAGGACGGAGAAAACAGCACUC

>P577

UCCCACCUCAUCCUCCCAAGCACCUAGUGCUACAAGCACAUACCACUGUAC

>P578

CCCCACCCCCAGUAGCCUCUCUCUUAAGGUUCUGGUCUCAACACUGGCUGC

>P579

CCCUUCCCUGCCAAACUGGACCUGCAACAGGGACAGAAUUUACUGCAAGGU

>P580

CACACUGGGGAAAAGAUAGUCUCUUAAAUAAACGUUGCUGGGAAAAUUGGA

>P581

GGAAAAUUGGAAAGUCACAUGCAGAAGAAUAAGACUAGACCCUGUCUCCAC

>P582

UAAGUCUUUAAUCCAUUUUGAGUUGAUUUGUGUAUAUAGUGGAGACAGGGU

>P583

UGUGUAUAUAGUGGAGACAGGGUCUAGUCUUAGUCUUCUGCAUGUGACUUU

>P584

GGCUUGAACCUGGGAGGGAAGGUUUACAGUGAGCUGAGACCGCACCAUCAC

>P585

AGAGCGACACUCUGUCUCAAAAAAAAAAAAAGGAAAAAAAGGACUAGGCAG

>P586

UCCCACUUCAACCUUCUGCGGGGCUAGGACUACAGGAAGACACCACCAUGC

>P587

AAUUUUUUUUUUGAGAUGGAGUUUCACUCUGGUUGCCAAGGCUGGAGUGCA

>P588

GGUAGAAACAGGGUCUCACCUUAUUACCCACUUUGGUCUCCAACUCCUGGC

>P589

AAAGCAGUCUAAUUCAGUCCGGUCAAUAUGGUGAAACCCUGUCUCUACUAA

>P590

CUCUUGUAACCCAGGCUGGAGUGCAAUGCCACAAUCUUGGCUCCUUGCAAC

>P591

AAAUGUUAGAGUUAUGGUCGGGCACAGUGGCUCAUGCCUGUAAUCCCAGCA

>P592

CUUUUAUUUACUCACUUACUUUUUUAGAGAUGGGGUCUCCCUGUGUCACCU

>P593

AAAAGUUGUUGUUGUUGUUUGAGACAGAGUUUCGUCUUGUUGCCCAGGCUG

>P594

AUUAGGUUUACAGCCUGGGCAACAUAGGGAGACCCCAUUUCUACAAAACAG

>P595

AGCUGCAGUGAGCUAUGAUUGCGCCAGUGUACUCCCGCCUGGACAGCAGAG

>P596

AAAAUUUUUUUAAUGAGGCAGGCAAAGUGGCACAUACCUGUAGUCCUAGCU

>P597

ACUGACAGAAAUUGAUUACCUUGCCACUCCAACCUACGUACUGAGUGAGUG

>P598

UAUGUUUUUAAAAUGUAGUAACUUUAGCCAGGCACAGUGGUAUGCACCUGU

>P599

UUGAGGGAGGAGAGGCAGGAGAAUCACUUGAGCCUGGGAGCUUGAGGCCAA

>P600

GGCUGAGGCAGGAGAAUCGCUCGAAACUGGAAGGCGGCAGUUGCAGUGAGC

>P601

AAACUGGAAGGCGGCAGUUGCAGUGAGCCAGGAUUGUGCCACUGCACUCCA

>P602

GGGCCGAGGCAGGAGGAUGGCUUGAACAUUGGAGGUCGAGGCUGCAGUGAA

>P603

GAGGUAAUCACCAGACAACUGCAGAAUGUAGAACACUGAGCAGGACAACUG

>P604

AGAAUGUAGAACACUGAGCAGGACAACUGACCUGUCUCCUUCACAUAGUCC

>P605

UCCUUAUCACCACAAAUCACACAACAAAAAGGAGAAGAGAUAUUUUGGGUU

>P606

GAGGACAGGUCAGCUGUCUGGCUCAAUGAUCUACAUUCUGAAGUUGUCUGA

>P607

UGUCUUCAUGAUUAAAUUCAGCCUAAACGUUUUGCCGGGAACACUGCAGAG

>P608

UGCAUGACUGAUUUUAUUUAACGUAAUGUCCUCCAGAUUUAUCCAUGUUGC

>P609

UCUUGAUUAUCUAUCCAUAGAUAUUAUGAAUGGUGCUGCCGUAAACAUGGG

>P610

AGAAAUACCCAGCAGUGGGAUUGCUAGAUUGUAUGGUAGUUCUGUUUUUAG

>P611

UGGUAGUUCUGUUUUUAGUUUUUUGAGAAACCUCCAUACUGUUUUCCAUAG

>P612

GUUCUCUUUUCUCUUCAUCCUCAACAGCAUUUAUUGACUUUUGUCUUUUUA

>P613

AACUAAUGAAAAAAUGCACAUCACUAAUUAUUGGAGAAAUGCAAAUCAAAA

>P614

CAUCACUAAUUAUUGGAGAAAUGCAAAUCAAAACCACAAUAAGAUACCAUC

>P615

CAGAUGCUGAUGAGGCUGUGGAGAAAAAGGAAUUGCUUAUACACUGUUCGU

>P616

AUUCAGCCACUGUGGAAAGCAGUUUAGAGAUUUUUCAAAGAACUUAAAACA

>P617

UCUACCAAAAAUCAUGCACUUGCAUAUUCAUUGCAGCACUAUUCACAAUAG

>P618

UAGUGGUGGAUUGGAUAAAGAAAAUAUAGUAUAUAUUCAGUAUGGAAUACU

>P619

UAUGGAAUACUAUGCAGUCAUAAAAAGAAAUGAAAUCAUGUCCUUUGCAGC

>P620

AAAUGAAAUCAUGUCCUUUGCAGCAACAUGGAUGGAGCUGGAAGUCAUUAC

>P621

UUGCAGCAACAUGGAUGGAGCUGGAAGUCAUUACCUAAGUGAAUUAAUGCA

>P622

CUAAGUGAAUUAAUGCAGGAACAGAAAACUAAAUACUCUAUAUUCUCAUUU

>P623

AGGAACAGAAAACUAAAUACUCUAUAUUCUCAUUUAUAAGUUUAUAAGCUA

>P624

GGUGGUGACAUGGACUUGUUUAUAGAAGACAGGUCAGCUGUCUGGCUCAAU

>P625

GCAGUCUUGGCUCACUGCAGCCUCCACUUCCCAGGUUCAAGCAAUUCUCGU

>P626

GACUCGGCUCCUACCUGUAAUUCCAACACUGGAAGGCUGAGAUGGGAAGAU

>P627

UUUUGAUACAGGGUCUCGCUGUGUUACCCAGACUGCAGUGCAGUUGCACCA

>P628

AAGAAGUGUGUGUGUGUGUGUUUUUAAGAGAUGGGGCCUUAUUGCCCAGGC

>P629

GGCUGGACUCGAACUUCUGAGCUUAAGUGAUCCUCCCACCUCAGGUUCCCU

>P630

UGAGCUUAAGUGAUCCUCCCACCUCAGGUUCCCUAGUAGCUGGGAUUACAG

>P631

ACAAUAAGAUAUUUUAAAGUGGGGCACAGAGGCUCAUGGUUCAAUCCCAGU

>P632

GCACAGAGGCUCAUGGUUCAAUCCCAGUACUUUGGGAGGCCGAGGCAGGAG

>P633

AGGCCGAGGCAGGAGGAUUACUUUAAGCCAGAUGUUUGAGACCAGCCACAA

>P634

UUAGGGUUCUGUAUAGCUCACUGUAACCUUGACCUCAUGAGCUUGAGCUCA

>P635

UGGGUUCAAGUGAUCCUCCCCACUCAGCCUCCCGAGUAGCUGGAACAUCAG

>P636

AUCUCUACUGCAAAUAUAAAAAAUUAGCGGGGCGUGGUGUCACAUGCCUGU

>P637

GACCUGUCUCCUUCACAUAGUCCAUAUCACCACAAAUCACACAACAAAAAG

>P638

AUAAUGUAGCUGCAUUUCUUUAGUUAUUUUGAACCCCAAAUAUUUCCUCAU

>P639

AGCAGCCGGGGCCACCCUAUUACACAGACCCAGGAGGACCGGGGAUGAACC

>P640

AAACCUUCCAAGUGCUCUAUGCCUCACGGUUUAGCAGAAAAUAUCAAGCAA

>P641

UUGAGAUAUUUCAAAUAUUUGGUGGAGCUUUUAAUGAGACGGAGAGACACU

>P642

ACACUCUCGAGUGUGGAAGAAAAACAUGAGGGGGUGUGAGGAUAAGGCGAC

>P643

CAAACACCGUUUCCCACAUUGAAGAAAUCACAGAGAUCAGCAACUCUAGAG

>P644

UCAGCAACUCUAGAGUGCGAUGAAGAAGCUUCACUCUGGGAGAACCCCCUU

>P645

AGAACCUGCAGGCGACACACCAAGGACUCCACGAGGGAGUCCUGAGUACUG

>P646

AGCGGGGAGGCGCCCGAGGUGAGACAGGGGCACCCUCUGCAUCAUAAAGGA

>P647

GAAUCAGACGGAUGCGGAAACCGAGACGGGCUGGAUAGGAAACUCUUUCCA

>P648

GGUCUCAGGUCAGCCUGAGCUCCUGAGACGCCCAGGCCCGGAAAGACACGU

>P649

UUCCCCUGCUAAGUCUGAAACAUUUAUUUCUUUUGUACUAAAGAAACAAGU

>P650

GCAGAUCAGAGGUCGGGAGUUCAAAACUAGCCUGGCCAACAUAGUGAAACC

>P651

AACUCUGUCUCAAAAAAAAAAAAAAAGACACGUGCACACAAAUGUUCAUUG

>P652

UGUUCAUUGCAGCACUUUUCACAAUAGUUAAGACACGGAAUCAACCUAAAU

>P653

ACACCAUGGAAUAUUAGGCAGCCAUAAAAAAAUGAGAUUAUGCCUUUUGUG

>P654

AACUAAUGCAGGAAGAGAAAACCAAAUACUGCAUGUUCUCACUUACAAGUU

>P655

CUCUUGCUCCAUCCAUGUUGCUGCAAAGGACAUGAUAUCAUUCUUUUUUAU

>P656

AAAGGACAUGAUAUCAUUCUUUUUUAUGCCUGCUUAGUAUUCCAUGGUGUA

>P657

CCUUUCCUUUGAGUAGAUACCCAGUAGUGGGAUUUCUGGGUCAAAUGGUAA

>P658

CUGGAUGGAGUGUAGUGGCAGUCACAGCUCACUGCAGCCUUCCCAGGCUCA

>P659

ACAUUCCAACCAACAGUAUAUGAGCAUUCCCUUUUCUCCGCAUCCAUGCCA

>P660

CCAGGCAUCAUGGUACGCACCUGUAAUCAUAGCUACAGGUAAGACAAAGCA

>P661

AAAUAAAAUUUAAAAAUUAUCAGCUAGGCACAGUGGCACAUGCGUAUAAUC

>P662

GUUGUUGUUGUUGAGACAGGGUUUCACUCCUGUCGCCCAGGUUGGAGUGCA

>P663

GGGGUUUAUCUGUGUUAGCCAGGAUAGUCUCGAUCUCCUGGUCUCGUGAUC

>P664

AUUUAUUGUGUACUUUAUUAUUAUUAUUUUUUUCGAGAUGGAGUGUCGCUC

>P665

UUGAAAAUUCAGCAAGCAGCUAGGCAUGGUGGCUCAUGCCUGUAAUCCUAG

>P666

CAGGGUGGCUCACUGAGUGCCUGUAAUCCCAGCAACUUGAGAGGCCAAGGC

>P667

AGACCCCCAUCUCUACAAAAAAAAAAAAAUAGGUAGACAUGGUGGCGCGUA

>P668

GCCAGGCGUGGUAGCUCAUGCCUGUAAUGCCGGCAUUUUGGGAGGCCGAGG

>P669

AAAAGAGAGAAGAAUAGAGUGAUUUAGGGGUGGGUGCAGUGGCUCACGCCU

>P670

GGCGAUUGUGGCUCAUUGCACCCUCAGCCUCGCAGGCUCAAGUGAUCCUCC

>P671

ACGUCAGCCUCUGAGUAACAGGACUACAGUUAUGCAAUUACGCCUGGCUAA

>P672

ACCUCAUUUCUAUAAAAAAAAUACAAAUUAGGCAGGUGUGGUGGUGUGUAC

>P673

AUGACAAGGCUUAGAGAGGAUGUGGAAAAAUUGGAACCCAUUUACAUUUCU

>P674

CUGUGUGCAGUGGCCCCCUCUUGUAAUCCCAGCACUUAGGGAGGCUGAGCU

>P675

AGACCAGCCUGGCCAGUAUAGGGAAACCCUAUCUCUACUGAAAAUACAAAA

>P676

GGCGACAUAGCAAGAACUCGUCUCUACAAAAAUAAGAAGAAAAUUUGCCAG

>P677

UGGGUGACAGGGCAAGACUCCAUCUAAAAAAAGAAAACCCAGGAGUCUUUG

>P678

UGUUGGAAAAACCAAUUGGAAGGGAAGUUAGACAAGCUUUGGGGAAGAGAA

>P679

UGAAAUGGGAGAAUUGCUUGAGUCCAGGAGUUUGAGAUGCGCCUGGGCAAC

>P680

AAAAAUUAGUUGGGCAUGAUGGCAUACGCCUGUAGUCCCAGCUACUCUGGA

>P681

UGCUCAGCCUCCCAAGUAACAGAUUACAGGCACCUGCCACCACAUCUGACU

>P682

CGGCUCACUGUGACCUCUGUCUUCCAGGUUCAAGUGAUUCUGCUUCCUCAG

>P683

UCUUAAGUAGAGACAGUCUCAUCAUAUUGCUCAGUCGAUCUCAAACUCCUG

>P684

CCUCCCAGGCCCAGGCCAUCUUCCCACCUCAGCCUCACAAGUAGCUGGGAC

>P685

AAGACAGGAUUUCACUCUUUCGCCCAGGCAGGAGUGCGAUGGUGCGAUCUC

>P686

UGGCAAGGCUGGUCUCGAACUCCUGACCUCACGUGAUCCACCUGCCUUGGC

>P687

UGCUCUUGCGUCACACGUAAUGGCCAGGCAUGGUGGCUCAUGCCUGUAAUC

>P688

AGCAUCAUGAUGAGACCUCAUCACUACAAAAACUCAAAAAUUACCUGAGUG

>P689

GACCACAGGCAUACUCAACAUGUUUAGCUAAUUUUUCAUUUUUCGUAGAUA

>P690

UCACCUGGGUUGGAGUGCAUGGCACAGUCUUGGCUCACUGCCGCCUUGACC

>P691

GGGUUUAUUCAUUCAUUUUCUAAUUAGCCAGGCACGGUGGUGCACGCCUGU

>P692

ACGCCUGUAGUCCAGCUGCUUAGGAAGCUGAGAUGAGAGGAUCAAUUGAGC

>P693

GAAGCUGAGAUGAGAGGAUCAAUUGAGCCUGGGAGGUCAAGGCUGCAGUGA

>P694

AGCUAGCUACAGCCUCAACCUCCCCAGCUCAAGUAAUCCUCCCACCUCAAC

>P695

CUCAACCUCCUGAGUUUCUGGGACAACAGGCACACAACACCAUGCCUGGAU

>P696

GAAGAGAGGGGCAACUUUGUUGCCCAGUCUGGUCUCAAGCUCCUGGCCUCA

>P697

UUCAGGUUUUUAAUUUUCCCUGCAAAGUAUUGUGUGUGUGUGUGUGUGUGU

>P698

GGCUGGUCUCAAACUCCAAGGCUCAAGCAGUCUGCGUGCCUUAGCCUCCCA

>P699

GGCCUCAGGAGGCCUCAGGAGGCCAAGGCAGGAGGAUCCUUGAGCCCAGGA

>P700

AUCUCAUCUCUACAAAAAUAAAAAAAUUAGCCUGGUGUGGUGGAGCAUGCC

>P701

CAGGAGGUCAACCUGGCAGUGAGCCAUGUUUGCAUUAUUGCACUCCAGCCU

>P702

UUCAAGCGAUUCUCCUGUCUCAGCUACAGGCGCCCGCCACCACACCCGGCU

>P703

ACCCCGUCUCCACCAAAAAUAAGCCAGGCGUGAUGACAGCUCCUGUAAUCC

>P704

AAAGACAGCUUCAAAUUGUGAUCUGAGUAUUUAUAAUUAAAUGACUCAAUU

>P705

AACCAGAAAGACCAAGAGCCCUACUAGGAAGUACUUUAAUAGUUUUUCUUA

>P706

UACUUUAAUAGUUUUUCUUAGAAAAAAAAAUUUCCAGACACUUAACAUUUC

>P707

UAACAUUUCACAACAUUUCAACAGCAAAGUAUUAGUUGAGAGAGGGGUUUU

>P708

UCAACAGCAAAGUAUUAGUUGAGAGAGGGGUUUUCAGGAGUUGGAGAUUAU

>P709

UGAGAGAGGGGUUUUCAGGAGUUGGAGAUUAUAGAAUAUUAGGAAGAAAUG

>P710

GAGUUGGAGAUUAUAGAAUAUUAGGAAGAAAUGUUGGUAUCCUCCAUUAUA

>P711

AAGAAAUGUUGGUAUCCUCCAUUAUAGAUGGAUGGCAUAGGUCACAAAUGG

>P712

CCAUUAUAGAUGGAUGGCAUAGGUCACAAAUGGGAGACUGGCAGCUAAGCC

>P713

CAUAGGUCACAAAUGGGAGACUGGCAGCUAAGCCAAUAUCAAAACCCAGUG

>P714

GAGACUGGCAGCUAAGCCAAUAUCAAAACCCAGUGGAAUGACACUUCUAUG

>P715

CAAUAUCAAAACCCAGUGGAAUGACACUUCUAUGGAGUUUACUUUUCUUCC

>P716

UCCUGCUAUCUUCCCUAUCCCACGGAAAUGUCUGUCACCAUGUAAAGCCCA

>P717

CCCACGGAAAUGUCUGUCACCAUGUAAAGCCCAGUAGCAGGCAGCUUAGGC

>P718

CACCAUGUAAAGCCCAGUAGCAGGCAGCUUAGGCUCCAGUCUUCCCCCUUG

>P719

UAGGCUCCAGUCUUCCCCCUUGGGUAGGAAAAGGAGUGAAGGGAAUGUCAC

>P720

CUUGGGUAGGAAAAGGAGUGAAGGGAAUGUCACUCCUGAGUUUCCAUGCUU

>P721

UUGCCCAGGUGUGUCUCUUGGGCUCAGGCGAUCUGCUGGUCUUGGCCUCCC

>P722

AUAUGCCCCUUAGGGGCUGGGUGUGAUGGCUGACACUUAUAAUCCCAGCAC

>P723

AAAAAAAAUUUUUUUUUAAACACAGAGUUUCACUCUGUUGCUUAGGCUGGA

>P724

CCACACUGGGCUUUUUUUAUUUUUUAGAGACAGGAUCUUGUUAUGUUGCCC

>P725

AUGUCAACUCUGCCGGGUGCGGCGCACCCACAGCGGGCACAAGAUCACACC

>P726

UUUGGAUAGACACCAAGAACUACGUAGAAGUGACCCGGAAGUGGUAUGCAG

>P727

CCCGGAGCGAUCUCGGUUCACCACAACCUCUGCCUCCCGGGUUCAAGCGAU

>P728

GUGAUCCAUCUGCCUCGCCCUCCUAAAGCGCUGGGAUUACAGGCGUUACAG

>P729

UAAGGUAUAACAGGCCAGGCAAGGUAGUUUAUGCCUGUAGUCUCAGCACUU

>P730

AGAUGGACCUGACAAGGCCAGGCAUAGUGGUUCAACAGCACUUUGAGAGGC

>P731

AUGAUUGCCUGAGCCUAGGAGUUCAAGGUUACAGUGAACUGUGAUCACAUC

>P732

AUAAUAUUCAUAUAUGUAUAUAUGUAUAUAUAAUAUUCAUAUAUGUAUAUA

>P733

CGUAUAUGCAUAUGCACACAUACAUAUUCGUAUAUAUAUGUGAAGAUAAAC

>P734

CAAUUCCUUCAUGGCCAGGCUUGGUAGCUCACAGCUGUAAUCACAGCACUU

>P735

CUCCCGAGUACCUGGGGCUAGAGGCACGCAGCACCACUCCUGGCUAAUUUU

>P736

GAGACAGGGUUUUACCACAUGGCCCAGGCUCGUCUGGAACUCCUGGGCUCA

>P737

GGUGCCCAGAGGCUGUGGGAAGCUGAGCACUAAUUAGCUCUGGCUUUCUAA

>P738

AAAGAAUUGCUUGAGCUCAGGAGGCAGAGGCUGCAGUGAGCCGAGAUGGUG

>P739

AAAAAAUUAGCCGGUCAACACCUGUAGUCCAGCUGCUUGGGAAGCUGAGGC

>P740

GGCAUAUAUCUUUUCUUUUUGAGACAGAGUCUAGCUGUGUCACCCAGGCUG

>P741

AGGAGAGGAGUGCUCCUUCCUCCCUACCGCUACUCUCCCCAAGCCUGUGUU

>P742

UUGGCCAGAACAGGUUCUGAAAACCACUUCUCUACCUUCACCACCACCACU

>P743

GUUCCUCUUUUUUUUUUUUUUUUUGAGAUGGAGUUUUGCUCUUGUCGGCCA

>P744

GAAUAUUGCUGCAGCAAAACCAGCUACACCAAAAAGCCAGGUAACCUUAAC

>P745

AACAUCCUGUGUCUGCUUUGAUGGAAAUCUGAAAUAAGAGAAGGUGGCAAC

>P746

AUAAAUGGAAGUGCUUACCAGCCCAACUUUGCCAGCCUUAAUAAGAAGCAU

>P747

GCCACCUGCUUCAGGAGUGCCUCACAUAGAUAGAUUGAGGUUUUAUAAUAA

>P748

AAUGCAGUCCUCAUAUACUAUCAGCAGCUGCCUUUUCUCUAUCCUCUUUCC

>P749

UCCUGCCUCCAUGUUCUGGUCUCUAAGACUCCCUUGAUCCCUUCUUGCCUC

>P750

UCAAAGUGCUCUCCUACAACUCAGAAACAGUGCAGGCCACUGUGGAGACAG

>P751

UUAUUUGGUCACUGUCAUAUUAGGCAGGAGGGAACUUUUCUUUAACCUCCU

>P752

AUUAGGCAGGAGGGAACUUUUCUUUAACCUCCUCUCUCCACAGUGGCCUGC

>P753

UCCUCUCUCCACAGUGGCCUGCACUAUUUCUGAGUUGUAGGAGAGCACUUU

>P754

AGAUAUCCGUGCCCGGAUAUUUCUCAUCGCCAGCAAAGAGCUUGAGGUGAG

>P755

AGCUCACUGCAACCUCCACCUUCUGAGUCCAAACAAUUCACCCGUCUUGGC

>P756

GGGCGCCUGUAAUCCAAGCUACUCUAGAGGCUGAGGCAGGAAAAUCGCUCG

>P757

UGUGUGUGUGUGUGUGUGUGUGUGUAUGUUCAUGUACACUUGGCUGUCAGG

>P758

CUGUCUCUUCUCUUCUGUUACUGUCACUACGCUCACCCAAGAUCCUACACU

>P759

GCCCGCCCUGGCCUUCCAAAGUGCUAGGAUUACAGGAGUGAACCACUGCAC

>P760

UAUUUUUUCCUUAAAAUUAUUCAGCAUAGUAACUUUUUUUUUUUUUUUGAU

>P761

UGUAACUUCUGCCUCUCAGGUCCAAACAAUCCUCCCACCUCAGCCUCCCGA

>P762

CAAAUAUGUUGCAAAUAUUCCCUCCAAUUUGUCACUUACUUUCAUUUUGUU

>P763

CUGCAGUGAUAUCAGAAAGGAAAAAAGCAAACUUCCCUGACCGGGAAUCGA

>P764

GCAGCACCUUCUGAGAAAAGAUCUUAGGACAGACGCCUCAACCACCUCAGG

>P765

UAUUUCUUUUUUUUUUCCCCCAAGUAGCUGGGACUCCAGGCACACACCACC

>P766

AUGUAUUAGUCUGUCUCUAUGUAUUAGUGUGUUCUCAUGCUGCUAUGAAGA

>P767

UGAGACUGGGUUAUUCAUGAAGAAAAGAGGGUUAAUUGGCUCACAGUUCCU

>P768

AAGAAAAGAGGGUUAAUUGGCUCACAGUUCCUCAUCGCUGGGGAGGCCUCA

>P769

AAAACCCUAAGCUCUCGUGAGACUCACUCACUAUUAUGAGAACAGCAUGGG

>P770

GAUUAUACUAUAACUUUUGUUCUAUAGUGAAAUGGUUUGGCUAUGUCCCCA

>P771

CCAUGCUGUUCUUGUGAUAGUGAAUAAGUCUCAUGAGAUCUGAUGGUUUUA

>P772

ACUCCCCGUGGUCAAAUCUGGGAAAAUUUGGCAUCAGAAUAAAAUGAGGGU

>P773

GUUGGUAAACCGCUACUCUCAUUUUAUUCUGAUGCUCAGAUUUUCCCAGAU

>P774

UAUUCUGAUGCUCAGAUUUUCCCAGAUUUGACCACUGAGAAUUCAUUCAUA

>P775

UCCCAGAUUUGACCACUGAGAAUUCAUUCAUAUUGGGGCCUUUGUUCUUUU

>P776

CUGGUGGAUAAAAUACAGGAACACAAAUUUUAAGCUAGUUGAAGGCAGAGU

>P777

CCUGAACUCUGCUUUCAACUAGCUAAGAAUUUGUGUGUUUCGUAUUUUAUC

>P778

UGAGCUCAAACAGUUCUCCUGUGUCAGUCUCCAGAGUAUCUGGGACUACAG

>P779

AGAGCGAAACUCUGUCAAAAAAAAAAGAAAGACUUCCUCAAACUAAUUACA

>P780

AGGAUGAUUCUGUAGGCAUUGUCCUACUUAAAACCAUGUGGUGAUUCCAGG

>P781

ACAACUGCACCCUGCUGGGGGUGCUAAUGAGGAGUCAGUAGCUGAAUUGUU

>P782

UUAAGAGAUAUAGGGGAGCAGGAAUAGGUUAGUUUAAAAACAAGGAGAGGG

>P783

GAGCUUCAUUUAUACAUUUCAUAUUACCAUUAGCUCAUCUUUUUUAUUACU

>P784

GCUUUUUGUUUUUCAGGUGUUUUUAAGGCAGGGUCUCACUCUGUCGCCCAG

>P785

CUAUUCCAGCAGCUUAGAGGGGAGAAUCACUGGAGAACAGGAGUUCAAGGC

>P786

GAGAAAACAAAUUCUCUGGGAUAUAAGGGUUCACCUUUUGGAGGUGGAUUA

>P787

UGGGAUAUAAGGGUUCACCUUUUGGAGGUGGAUUACACUUUUCAGGACACC

>P788

UAAAUGAUCCCCUGGUGCCCUGGAAAGAGACCGUUUACCUCCAAAAGGUGA

>P789

CGUAAAAAGGCUGAUAAGAAAGGCCAGGUGCGGUGGCUCAUGUCUUUCAUC

>P790

AGAGAAUAGGACAUGUCUUUCCCUUACCAUUCACUUAAGAAGUAUUAUAGG

>P791

CUCCUGAGUAGCUGGGAAUAUAGGCAUGUGCCAACACACCAGGCUAAUUUU

>P792

CUGUAAUCCUAGCAUGGGAGGCCGAAGCAGAAGGGUUGCUUGAGCUUAGAA

>P793

AAUCCUCUAGCUCACUGUAGUCUCAAAAUCCUGGGCUCAAGCUAUACUGCU

>P794

UGCUGCCUCAGCCUCCCAAGUAGAUAGGAUUAUAGGCAUGUGCCACUAUAC

>P795

UGCAGUUAUUUUAAAACAACAGAAAACUACUUUGCGAGGUUACAUGUCACA

>P796

UGUGGGUGUGUGUGUUGUCAUUUUGAGGUGGGGUUUCACCAUGUUGCCCAG

>P797

UGCACAAAAACAACAGACAGCCCUAAGUUGUCAGUGAAUUAGCAGGUCGUC

>P798

UUAAGAAGCAGAAGAAGCUUCUGGUAGAAAAAAGGGGCAGCAGCUACCAAG

>P799

CAGCCCCUGGAAAGAAGCCAGCAGAAAAGAAACCUACUACAGAGGAAAAGA

>P800

CCUCACUACAGUACACUUAAAAAGAAAACUCAAGGCCGGGUGCGGUGGCUC

>P801

AAAGGAAGGAUACAUUGUCUAAUUUAAUCACAAACGCAGCUAUGUGAGGAA

>P802

UGCUUAUUGUAUUAGGCAUUGUUUUAGGCACUUAAGAAAAUCUAGGCUCUG

>P803

CUAGGCUCUGAGAAGUUAACUGUAAAAGCCCUUCCUCAUGUAGCUGUGUUU

>P804

UGUAAUAAAACACUGUCUUUUCAGGAUUGCUUCAUGGAUUGGAGAACUUUC

>P805

UUCAGGAUUGCUUCAUGGAUUGGAGAACUUUCUAACCAAAAAUUAAAAAAA

>P806

UGAAGUGUUUUUUGUUUUUUCCUUGAGACAGGGUCUUACUCUGUCACCCAG

>P807

GAAUAUAUAACAAUUCUAAAUAUAUAUUUGCACCCAACACUGGAGUACUGA

>P808

UAUAAAAAGAAACCUCAGCGGGCACAGUGGCCCAUACCUGUAAUCCCAACA

>P809

GCGGGCACAGUGGCCCAUACCUGUAAUCCCAACACUUUGGAGUGCUGAGGA

>P810

UUUAUAGAUCAGUAAGGAAUUGAAUACUUAUUUGUAUUCAAUACCUAUUUG

>P811

CAUCCUCUCCUCCAGAUCAUGAAUUAAUGCUGUCCUUUCCCCUUAAGCCUG

>P812

AAAUAACUUCCACAAAUUCACACCUAACAGCUGAUCUGGGAUGCUGAGGCA

>P813

GGAUUAGAGGCAUGAGCCACCACACAUGGCCUACUUAUAUUACAUCUAAGC

>P814

AAGAGGGGGAGAGAGAGAAGGGGAAAGGGGGGAACCCACCAGCACCCUCCG

>P815

GGUAGAAUAUUCCCUCAUCUGUAAAAUGGUGAUGAUAAUAGGACUUCCCUC

>P816

UCUGUAAAAUGGUGAUGAUAAUAGGACUUCCCUCAGAGGCUUUUAUAGGAG

>P817

AGUAAAAAAAGAUAAUGGAUAAUGUAUGUAAAGGGCUUAUUACUAUGCUUA

>P818

GGAUAAUGUAUGUAAAGGGCUUAUUACUAUGCUUAGUGCAUAGUAAAAGGU

>P819

CCUGGCUAUUUUUUUGGUAGAGACAAGGUCUCUCCAUGUUGCUCAGGCUGG

>P820

UAUUAUUAUUAUUAUUUUUGUUUGUAGAAAUGGGUUUUCGCCAUGUUGUCC

>P821

CAGCAGCAUGAUCACCACUCACCGCAGCCUUGACCUCCCUGGGAACUGGUG

>P822

GCCAGACAUUUUGGUGCUUGCAUGUAGUCCCAGCUAUUCUGGAGGCUGAGG

>P823

UUUUUCAAACUAAAAAUGUUCUGUGAAAACAAGCAGCUAGUUCAGCUUGUA

>P824

UUGUAACUACUUUUUCUAGGGAAAAAAAAAUCUAGACAUCCAUACUUUAUG

>P825

GCCCAGGCUGGACGCAGUGGCUCACACUGGUAGUCCCAGUACUUUGGGAGG

>P826

AAUAAACUUUGAGGCCAGAUGCAGUAGCUCACGCCUGUAAUUUUAGCACUU

>P827

GAAUAAAAUUGCUGAGGCAGGAGGAACGCUUGAGGCUAGGAGAUUGAGACC

>P828

GAGGCUGAGGUAGAAAGAUCACUUGAGCCCAAGAGGUUGAGGCUGCAGUGA

>P829

UUGCAAGUACAUGGAUGAAGGUGGAAACCAUCAUUCUCAGCAAACUAACAC

>P830

UGGAAACCAUCAUUCUCAGCAAACUAACACAAGAACAGAAAACCAAACACC

>P831

CCAAAAAUUAUCUGGGCAUGGUGGCACAUGCCGGGAGUCCUAGCUACUUGA

>P832

UCUUUUGAAAAAUAAGAUCCAGGCUAGGUGCGGUGGUUCAUGCCUAUAAUC

>P833

GCCAGGCAUAGUGGCGCAUGCCCAUAAUCUCAGCUACUCAGGAGACUGAGG

>P834

AACUAGGCUCACGCCUGUCAUUCCAACACUUUGGGGAGGCUGAGGUGGGCG

>P835

UCCAUGUCACUGAUCUUUCUGGCAAAGAAACCAUCUGCCGUGUGACUGGUG

>P836

AGAAAAUGUUCAUUGGAGCAGUUUGAGUUUUAAAUAUUUGGAUUUGGGAUG

>P837

ACAAAAAUCAAUGAAAAUUGAUUUUAUUGAUGUUGCCAUGGAACUAAUUUA

>P838

ACCAUGAGAUAUAUCCAAAUGUAUUAAGAAAAGUUAAUAUUUAUCAAGACU

>P839

GUUCCUGUCUCCAGUAAAUUGUGUUAUGACGUUAAAAUGUGAUCUCUCACU

>P840

AAAUUCUCUUCCUUAUGAUUUUCUUAAUGACAUUUUAUUUUCUCUAGCUUA

>P841

UAACAUAAACAGUCGAUUAACACAUAGUUUGUAUGUUAUAUAUAUUAUAUA

>P842

AACAGUAUGUACUAUGGUUAAUUUUAUGCAGUUAUGAUUUAAUAUUGCACC

>P843

GGUUUCGGGUUUCACCAUGUUGGCCAGGUUGAUCUUGGACUCCUGACCCCA

>P844

UUGUAUUAUGCUUCUUUCUCCUCUUAGCACUCUCAAAUUUCAGGUUUGUAA

>P845

AAACUUACAGCUCUAAAAUUCAGAAAGCCCUAAAAUUUCAAACACUGUUUG

>P846

CACUUCCAUAGCCUCUGCUUCCUCCAGAGUAACCAGGGCCACCUCCUCCAU

>P847

CAUAUGAUAGUUAUUCUGAGUCUUCAGGAUACAAGAAUCAUGAUAGAAGGU

>P848

GGUUGGAUUCAAACUCCUCAGUACAAGUGAUCCUCCCCACUCAGCCUCCUG

>P849

GAAUAUAAAAAUCAAAUUUGAGGCCAGGCACAGUAGCUCAUGCCUGUAAUC

>P850

CUAGAGACUGAAACCACCUUUGCAAAAAUCAUAACUGAGAAAAUUAUGACA

>P851

GAAAAUUAUGACAGUGAAAGAUACCAGACAUAACUGACCCCAUCUUGCUUC

>P852

UUCAUUCCUGGGUAUAGGCUGAACUAGCUUUGAGAAGGAAUUUAGUGUAUA

>P853

AGCAAAGCAUUCAACAGGUGACUUGAGUGCUGUUAAAGGCAUUCAGUUUUA

>P854

GCACUAAACAUGGAAAGAAUCAACCAGUACCAGCCACUGCAAAAACAUGCC

>P855

GAUCAAAUUCACCGAUAACAAUAUUAACCUUAAAUGUAAAUGGGCUAAAUG

>P856

UGUAAAUGGGCUAAAUGCCCCAAUUAAAAGACACAGACUGGCAAAUUGGAU

>P857

CUCAUUCCUCUAAUUCCGUUACUUUAGGAGGCUGAAGUGGAAGGAUCAUUU

>P858

AUCAUAACAACAUCCCUAAUGAGGUAGGAGCUAUUAUUAUUCUCAUUUUAC

>P859

UACACAUAUAUGUGUGUGUAUAUAUAUACAUAUAUAUGUGUGUGUAUAUAU

>P860

UCCUUUGUUAGUGCCGCCAAUUAUCAAGGGAGCAAAUAGUGAUCUCCCUGA

>P861

CUACCUGACUUCAAACUAUACUACAAGGCUACAGUAACCAAAACAGCAUGA

>P862

UUUUUUUAUUUUGUGGGUACAUAGUAGACACAUAUUUUUGGGGUGCAUGAG

>P863

CAGGCAUGAGUCAACAUGACCAGCUAAUUUUUUUUAUUUUUUGUAGAGACA

>P864

ACCUCAGCUGGGACCACAGGCAUGCACUGCAAUGCCCAGCUAAUUUUGGUA

>P865

AACAACAUGCUACAUGGGAAAAUGGAGUUCUCCACCUCAGUGUGAAGGUUA

>P866

UAUACAUAUAAACAUACUAUAUAUUAUGUUACUUAACCAUCAUAAUAAAAC

>P867

GUUACUUAACCAUCAUAAUAAAACUAUAUGACAGGUAUUACUGACCCAAUU

>P868

AUUGGGUCAGUAAUACCUGUCUUACAGUGUUGUUAUGAUGGUUAAGUAAUA

>P869

UAGUAUGUUUAUAUAUAUACUUUUUAAAGACAUAUGUACAAAUGUAUGUGC

>P870

AGUUUUCUUCUCAGAGUUGGCACUGAAAAGUAAUUGAAAACGUGGUGACCU

>P871

UAUUUGUAUCUUUUCAUUCCAUAAUAAAUCAACAUACAAUAAAACCUGUAG

>P872

GUAGUCUGUCUUCUGUAGCAAAUAAAGUUGUAUAGGUGGCCACAUUUCCAA

>P873

CAAAUAAAGUUGUAUAGGUGGCCACAUUUCCAAUUAGUUUUCAGAACCAAU

>P874

GGUAUGUGUGUAUAUAUAUAUUUGUAUAUACUGUUAAGUAUAUAUAAAGUA

>P875

GGCUGGUCCCCAACUCCUGGCUUUAAGUGAUUGUCCUGCCUCAACCUCCAG

>P876

GCCCAGGAAUGAACAAGGACAGCUUAAAGGUUAGAAGCAAAAUGGUUAGGU

>P877

CUGAAACUGUCUUUGCAAAAAUUACAGCAAUGAGAAAAUUAUCACAGUGAA

>P878

ACCUCCCCCAUUGCUCCGAAAGAUAACAUCACUAUUGUAAAACCUAAGAUC

>P879

UUCCCCACUUCCUGGCCCCCUACACACCAAAUUAUCCUUAAAAAUUCCAGU

>P880

AUUAUCCUUAAAAAUUCCAGUCUCCAAAUUUUCCAGGAGACUGGUUUGAGU

>P881

UCAUUGCAAUUCCCCUGUCUUGAUAAAUCUGCUCUGUCUGCGUAUCAGGCA

>P882

AUCUGCUCUGUCUGCGUAUCAGGCAAGGAGAAGCCAUUGGGUGGUUACACA

>P883

AGUACCCUCUACCCCCUUGCCCAGUAGCUGUGACUACUACAGGUGCAGGCC

>P884

GUUAGGUAGAUGAAAUCUCACAGGUAGCAGCCCUCAGAAAGAAUUGAUGGU

>P885

GUAAAUACUUCUUUUCGACCUUUAAAGGUGUCAGAGCCUCAGUUAAUUGUU

>P886

UAAGGAGGGAGUCAUGGUGGCCAAGAAGGAUGUCCACAUGCCUAAGCACCC

>P887

UUAAGAUCAGAAAUUACUCCAGGCCAGGCACAGCGGCCCACGCUAUAAUCC

>P888

ACCAGGCACAUUGGCUCAUGACUAUAAUUCCAUCACUUGGGAGGCUGAGGC

>P889

UGACAGUUUGUUUGUUUUUUGAGACAGGGUCUUGCUCUGUCAUCCAGGCCG

>P890

CACAAUAACAGCCCAUUGAACCUCAAGCUACCAGGCUCAAGCAGUCCUCCC

>P891

GAACCUCAAGCUACCAGGCUCAAGCAGUCCUCCCACUUCAGUCUCCAGAGU

>P892

CCACAGCUACUCUGUCACCAUGCCCAGUUAAUUUUAUUUAUUUUCUGCAGG

>P893

UCCUGCCACAGCCUCCCACAUAGCUAGAACUAUAGCCAUGCACCACCACAC

>P894

UAGAACUAUAGCCAUGCACCACCACACACGGCUACAUUUUUUAUUUUUUAU

>P895

UAUUACUACAAAUCUUAGUCACCAUACUCUCUCGCCCAGGCUGGAGUGCAG

>P896

GCAGCUAGGACUGCAGGUGUGCACCACCAUGCCCAGCUAAUUUUUGAAUUU

>P897

UUGCCAUGUUGCCCAGGCUGGUCUGAAGCUGCUGGGCUCAAGCAAUCCACC

>P898

ACCUGGCUGGGCCUCCCAAAGCGAUAGGAUUACAGGCGUAAGGGCCCACGC

>P899

AUUGUCCCUCUCUGUCCAGCAGAUAAGCAGGUACUGCUCCCUGUGGAGUCU

>P900

CUGCUCCCUGUGGAGUCUUUUCAUCAUUCUUUUGUUUGGUGUUUCUCUUUU

>P901

GAGUACUUCAAAGAGAAGUAUUCAGAGUUCUCAGGAGGUGAUGACCCUUUA

>P902

AAAACUUUUUUUUUCUGGAGGCAGAAUCUUGCUCUGUUGCCUAGUCUGGAG

>P903

UUACAAAAAUUUUUUUUAAAAAAUUAGCUGGGUAUAGUGGUAUGUGCCUGU

>P904

GUGUGUGUAUAUUUUGAGUUGAGACAGAGUCUCGCGCUGUUACCCAGGCUG

>P905

UUUUUUGUGUUUUUUAGUAGACACGAGGUUUCGCGAUGUUGGCCAGGCUGG

>P906

GACACGAGGUUUCGCGAUGUUGGCCAGGCUGGUCUCGAAUUGUUGGCCUCA

>P907

UAAGUUUCCUGAAGCCUCCCCAGCCAUGCAGAACUGUGAGUUAGUUAAACC

>P908

UGCUUGCUUAUUUAUUUAUUUAUUUAGAGACAAGAGUCUUGUUUUGUUACC

>P909

UCCUCCUGCCCCAGCCUCUCAAGUAACUGGGACCAUAGGCCUACACCACCA

>P910

GCCUCCCAGGAUAGUGCUGACCCCCAAGCUCCAGCCCAGGGGAAUUUCAGG

>P911

AUAGACAAAAAGAAGGAUAAGAAUUAGAGGGCAGGCUAGGAGUGGUGGCCC

>P912

UAUAGAUUUAGCUGAUUAAAUUUAUAGAAAAAGUCCUGUCAUAUAAACUGG

>P913

ACACUAACAAUAGCUGAUGAGCUAAAAAAAAAAAAAAAAAAAAUCGUGGAC

>P914

ACCCCAGCCUGGGCAACAAUAGCGAAACUGUCUCAGAAAAAAGAAAAAAAA

>P915

UCAGAAAAAAGAAAAAAAAAAUCGCAAAAAGAAAAAUCUCAUAAUGUCGUU

>P916

AUUUUUAAUAAAACUUAAGAAGUAAACAUUUUACUUAUGUUUAUAGGUAUU

>P917

AGCACUAAGAAAUUGAGGUCAGGCCAGGCGCGGUGGUUCACUCCUGUUAUU

>P918

GGUGGUUCACUCCUGUUAUUCCAGCACUGGGGUGGCCAAAGUGGGCAGAUU

>P919

CAGCACUGGGGUGGCCAAAGUGGGCAGAUUGCUUGCGCUCUGGAGCUCGAG

>P920

AUAGGUCUUUUACUAUAUGAACACAAACUUCUUUGUUAAAAAUGGAUACCU

>P921

AUCCAUGUGAAGAGACCACCAAACAAGCUUUGUGUGAGCAACAAGGCUGUU

>P922

CCAAACAAGCUUUGUGUGAGCAACAAGGCUGUUUAUUUCACCUGGGUGCAG

>P923

GGACAUGGCACACCACAGCCACCCCAUAAAUCUGUCUUCUCAGCAGUUUCA

>P924

UCGAGAGCGGCUGAGUCACAAGCCUAAAACUGCCGAGUGGACAGACUCACG

>P925

UCCCCCUUCCCCUUCUACCAUGAGUAAAAGCUUCCUGAGGCCUCCCCAGAA

>P926

AAAAGCUUCCUGAGGCCUCCCCAGAAGCCAAGCAGAUGCUGGUGUAAUGCU

>P927

CUGGUGUAAUGCUUGUACAGCCUGAAGAACCCCAAGCCAAAUAAACCUUUU

>P928

CCAGCCUCAGGUAUUCCUAUAUAGCAACACAAAAUGGACUAAGACAGUUCC

>P929

CAUCCUACCAGUCACACGGCAGAUGAUUUCUUUGCCAGAAAGAUCAGUGAC

>P930

ACGUGUUCAGGGCAGAAAGUGCGGAAGUUGCAGUGAGCGCUUGGAAUUCAG

>P931

GAGAAAGCUCUUAGAGCUAGCCAAUAUGACUUAUCCUACACUUAAAUUAAU

>P932

GGACUCUUUCAUUUCCUGGAAUGCAACUAGUUUGAGUCUUCUGAUGUAAAA

>P933

UCUGGCAAUAGCAACUCAAUUUUAUAGCAACUGAAAGGCAGGAAAAGUCCU

>P934

AAAAAAAAUAGUGGCGGGCGCCUGUAGUCCCAGCUACUCUGGGAGGCUGAG

>P935

CUUUAAUGUAUAAAAUAAAUAUUAUACAAUAUACUUGUAUAGCAGUUUCUG

>P936

GUAUAACAAACGCAGCAUGAAAAGAAAAGAGAUGAUAGGCUGGAUUUCUUU

>P937

AAUCAGCUUUAUUUUCCUGACCUCCAGACCCUGUUCUCCUGCCCCAGUUGG

>P938

UGGCCAGGGGUCAUAGCUCAUGAGUAUAAUCCCAGCUCUUUGGGAGGCUGA

>P939

AAUCUCCAGUUGGUUUGUAUUCUAGAACUUGCCUCAGCACCAGAGCUUGGG

>P940

CAUGGCAUGGUUUGCUUAGGAGUUCAGAGUUCCUUCAUCAUCGAAAUAGUG

>P941

UGAAAGAAGUGUUCGACUACAGUUAAAGAGAUUGCAUUCUGUCCUGGUAUG

>P942

CUCACUCAUUUCUGUGACCUGAAAGAUGCUAAUGCCAGACAUGAACAGCCA

>P943

UGCAGAAAUUCUCACAACAAAAGUCAUGAAGCCGGUAGUGGAGUUACUGAG

>P944

AUCAAAUUGUAGUGGAAAUAAUCCAAGCGACUACAAUUAGCAGCUUUCCCC

>P945

CAUAGUAUAUAUGUGUAACAUAUAUAUGUAACGUAGUAUAUAUGUAACAUA

>P946

UCAGUCUAAAUAUUUACCUAGUAUUAGGUUGGUACAAAAGUGAUUGCGGCU

>P947

UUUUGCCAUUACUUUUAAUGGCAAAAACCACAAUCACUUUAGCAGCAACCU

>P948

CACAUCACCCAGGAUGGAGUGCAGUAGUACGAUUGUGGCUGACGGCAGCCC

>P949

UAGAAUAACAGAGGGAGAUGAAUUUAGCAAAUACAUCACUGAAGCUUCUCC

>P950

GCCAGGCACAGUGGCAUUCACCUGUAGUGCCAACCACUCAGGAGGCUGAGA

>P951

AAAAAAAAAAAAAAAAAAAAAAAAAAAAGGCCAGGCGCAGUGCUGUGGCUC

>P952

UUCCUGUGAAAACAUAAGUAGGUAUAGGCCGGGAGCUGUGGCUCAUGCCUG

>P953

UUUUCUUACAAUCUGUUUCAAGUGAAAUGGGUUCCUGGAGCACAAUGGGUU

>P954

AUGGAAACAAAAUGUGUGAAUUCCAAUUCUGCCACCUAGUUGCAGAAUGAC

>P955

AUAUUGAAGUAGCACAGUCAUACAGAAUAUUGGCCUAAAGAAAUGACAGAC

>P956

AUUGUAGCAAGUUGGUAACCAUCAAAAGUAACAGCAUCUCCACUGGAAAUU

>P957

UUUGCACAUCAAAUCUGGGGCUGAUAUCUCCACACUUGUUUAGCCUGCCUG

>P958

CAAUGUAACCAUGCUUCAUCAUCACAGUGAGAAACAGGAUGAUGACUUUGG

>P959

UUGACUUUCCACUCAUGUGCUUUUUACUCUAGCAUUAUGGAAUCUGGGCUG

>P960

AUUAGUAGUAGUAGUAGUAUACUUUAAGUUCUACAGUAUAUGUUCACAAUG

>P961

CAUCCUCCAUGCCACGACAGGCCCUAGUGUAUGAUAUUUCCCGCCCUGUGU

>P962

CAGUGGCAUGACCACAGUUCAUUGUAGUCGUGACCUCCUAGACUCAAGACA

>P963

CUCCCAAAGUGCUGGCACCGUAGGUAUGAGCCACCACGCCCAGCCAAAAAA

>P964

UGGUAGAAAUAGAAUCUGUUUGCCCAGGCUGGUCUUGAACACUGGGCCUCA

>P965

AACAAAAGGAUUAAAAAUAGACAAAAGAUAUGAACAGACAUUUCUCAAAAG

>P966

AGUAGAAAAUAGUACUAAUUCUUUUAUCUAAAAAUUUUUUACGGAACACCC

>P967

CAGCAAUACACAAUUUAACCGUGUAACAAACCUGCAUAUGUACCCUCAGAA

>P968

AAUUUUUAAAUUUUUUUGUCAAGACAGGGUCUCAUUAUAUUGCCCAGGUUG

>P969

CAGAAAUUUCAGUGAGAAGGUAUGUACAUUCUCUCAUAGGACAUGUGCUAA

>P970

AGUAAUAUCUUCAAGCCUGGGCAACAUGGAGAAACCCCGUCUCUACUAAAA

>P971

UACUCAGGAAGCUGAGGAUCGCUUGAGCCCAGGACGCAGGUUGCAGUGAGC

>P972

UGCCCGACACCCCCUGAGUGAUGACAGGUCUCAGGAGGGGGCCGCCCCCAC

>P973

GUCUCAGGAGGGGGCCGCCCCCACAAAUGGGAGGCGGAAGCUUAUUUUCCC

>P974

CCCCACAAAUGGGAGGCGGAAGCUUAUUUUCCCCGCUUGGGCUGGGGGCGG

>P975

GCUGCUGAGGGGGUGAAGAAGAGAUAGCAAUUUAUGUCGCAUGUUUAGUGA

>P976

AGAUAGCAAUUUAUGUCGCAUGUUUAGUGACACAAUCCAGAUGCAGGUGGG

>P977

UCAGAGGCAAUGAGAUGCUCCCAGUACCAAACUGUGACACUGGGAUCAGGG

>P978

UGGGUCAGAGUGGGACCCUAGAGCCAGAGUGAGCUGUGGGUGGCCUGGGUG

>P979

UCUGGAUUGUGUCACUAAACAUGCAACAUAAAUUACCAUCUCUUCUCCAUC

>P980

ACCAUCUCUUCUCCAUCCCCUCAGCAGCCGCCCCCACCCCAAGCUGGGAAA

>P981

AGACCAUGACCUUGUAUGUGGCUGGAGGCCUCAGGCUUAGGGAGCAGCCUG

>P982

AAAUGGGGACACUGUCUCAGGUUGGAACCUGUAAGGGGCUGACUUGUGUCC

>P983

UUGAAGCCUGAACCCUCAGGACCUCAGAAUGUGACUGUGUUUGGAGAUAGC

>P984

UCAGAAUGUGACUGUGUUUGGAGAUAGCAUCUUUAAGGAGGGAGUUGAGGU

>P985

GUUGAGGUCACUGGGGUGGGCCCUAAUCCAGUCUGACUGGUGUCUUUAUAC

>P986

GGACAGAGACGUGCAUAGAGAGAUGAUGCCAUGAGGACGCGGGGAGGAGAC

>P987

CCUCCUGGCCUCAAGUGACCCUCCCACCUCAGCCUUCCAAGGAAGUGGGAC

>P988

UAAAUCUUUUCUCUUAGCUGGGUGCAGUGGCUAACGCCUGUAAUCCCAGUG

>P989

CUUUGGGAGGCUGAGGCGGGAAGAUAGUUUGAGCUCAGGAGUUCAAGGCCA

>P990

CAGGAAUUUGAGGCUGCAGUGAGCUAUGAUUGCAUCACUGUGCUCCAGCCU

>P991

UUCCCCUUCACCUUCCACCAUGAGCAGAAGCAGCCAGAGGCCCUCACCAGA

>P992

AUGACUGGGAGAGCGGGCUGAAUGCAAUGGAGUGUGCAUUACAUUUGGAAA

>P993

AAGGUCUUGCUCUGUCUCCCAGGCUAGAAUGCGAUGGUGCAAUCAUGAUUC

>P994

UUUUUUAAAGUCUUCAAAUUAUCAUAGGUGCCCUGGCCAUGAAUCUUAUAA

>P995

UAGGUGCCCUGGCCAUGAAUCUUAUAAUGGGUGAGAAAUCUUUUCUUCCCU

>P996

CAAAGACUCUGAAUUAUUUCCAGCAAACAAUUUAACAUCUCUUUAUUUCCC

>P997

UCUUUAUUUCCCUUUGUAGGAAAGAAAAGAUUUCUCACCCAUCAUAAGGUU

>P998

GGAAAGAAAAGAUUUCUCACCCAUCAUAAGGUUCAUGGCCGGGGAACUUAU

>P999

UCUCAAGGUGAUGGUAUCAGGAGGUAGAGCCUUUGGGAGGUGAUUAGGUUA

>P1000

AUGCUUUCUUCUGAAUUCCUCCUGAAGGACUUUCCUGAAGCUGGUUUACAU

>P1001

UUUAUUUUAAAUAUUUUUUAUUAAAAAAAAUGUCAAAUGUAAACCAGCUUC

>P1002

AAAAUACAAAAAUUAGCUGAAAAUCACUUGAACCUGGGAGGCGGAGGUUGC

>P1003

GAGACAGAGUAAGAUUCCGUCUCAAAAAAAAAAGAAAAAUAUUUGCACAAA

>P1004

CAGCAAACAAAAAUACCAAAUGCAUAGUGAACUUUGGAUUUCAGAUAAACA

>P1005

CAAGCUGGCCAUGCAGGAGUUCAUGAUCCUCCCAGUCGGUGCAGCAAACUU

>P1006

ACCAUGUUCAAAAAUAAAAAUAAAAAGUCAGAGUCCGGGUGCUGCGGCUCA

>P1007

GGUGGAUCUGCAGAAUGGCAGCAGCAUGAAACCUCGAGCCGAUGUGGCCUU

>P1008

AUUGGUGUGGUACACCUAUUACAAUAUGAACCAAUAUCAAUACAUUAUUAA

>P1009

AUUUAAAAGUAGCUGGCCUGGUGGUAUGCACCUGUAGUCCCAGCUACUCAG

>P1010

UUGUAUGUGUCUGGAACCCUGUGAUAGUCUUAGGCUUGCCAGCCUGGCCCA

>P1011

AUCAAACGUAACCAGCAUUCGGGGCAGCAUGGCAGCCACCAGCCCAUACAA

>P1012

GCUAAAGGUACAGCCCUCCGGAGUGAAGGCUCCCCAUAUCUCAGCAAGUGG

>P1013

CUCCCAUGUCAGCAGGGGUUUCCUUAUUGUCCUUGUCCUUCUUUUCCUUCU

>P1014

CCUUCAGCACAUUCCCAGAGCCUGCAUAUGCACACACAUCUACCAGUGUGU

>P1015

ACCAGCCUGGUCAACCAGCCUGGCCAACAUGGCAAAACCCUGUCUCUACUA

>P1016

UAAGCAGUCAUGUGCAUAGGUUCCUAUGUGUGUACAUGGUAAUUGUUCAAA

>P1017

GUUGCGGAACUUCUCUUUCCUUUAAAGCGCUGGGAUCCUUCUUUUCUUUCU

>P1018

GGCUUUGACCUCCCAACCUCCAACAAACCUCCCACGUCAGCCCCCUGAGUA

>P1019

GUAUAAGGACAGGAAGUGGGCAACUAUAGAAGCAGCAGAUCAUCAGCGGGC

>P1020

AGGCUGUUUUUUCUGGACCAGGAUGAGCCCCUUGAAACAAGUGGGUCAGGG

>P1021

AGAAAUGGAGUGAUAGACACUGGAGAAAAGGGGAAAGUCAAAAAGAAUCUG

>P1022

GAAAGUCAAAAAGAAUCUGUGAAAAAGAAGAAUUCACAAAUAGGAAAAUAU

>P1023

CAGUAUGUAGUUUCUCCCCCCGCCCAGACGGAGUUUCGCUCUUUCUCCCAG

>P1024

GAUGCCAAGAUCGGAGAGAAUUGCAAGGAGGAGGCAGUGGCCAUGCCAGAU

>P1025

GGUUGAAUUACAGAUUACUUGUACAAGAAUAUUCAUAGCAGCACCACAGCA

>P1026

UUCAGUCACUUAUAAGCUAUUAAAUAGUCUCAUUUUGCCGGGCGCGAUGGC

>P1027

GAACCCCCCCCUUUUUUUUUGAUACAGGGUCUCUCUCUCUCACCCAGGCUG

>P1028

GAGGCAGCAGGAUCUCUCGAGGCCAAGAGUUCAGGACCAGCCUGGGCAACA

>P1029

UCCCUCCACAACCUCCUAAGUAGCUAGGACUGCAGGCUUAUGCCACCAUAC

>P1030

CAACUCCUCUUAUCUUGCCACCCACAACAAUAGCAGGGUCAGGAAAUUGGG

>P1031

UCGUUCUUUAAUGCAGAUAUUUACUAGUUAUAAGACCUUAAGGCUGGGUGC

>P1032

UCUGUGAUAGAUUUGCAAACAGAGGAAAUAACGCAUCCUCGUGUCCCUCUU

>P1033

CUAAGGAGCUGGUGGAAACAGGAAUAACAGUUCGCUCCUACCCCAAGUGCC

>P1034

GAGAGAACAUACAUAGUGGGAGUUUAUCCUGUCCCUUUGAGACAGGAUAGC

>P1035

UGUGGGAAUUAAGAGAGCUAGAACUAGGUAGGCACGGUGGCUCACGCCUGU

>P1036

GUUAGGCUGUUUCCACUCAUGACAGAAGGGGAAGGGAAGCUGGCAUGUGCA

>P1037

ACCAGAGGCUGAAUAAUUUUAAAGAAGAGAGGUUUAUUUGGCUCACAGUUC

>P1038

UGUGUGUGUGUGUGUGUUGUUAUAAAGGAAUACCAGAGGCUGAAUAAUUUU

>P1039

AAGAGAUGAAUUGUGUAAUGCCUAGAUGUCAGUAGCGGAGAAGGUAUCUGA

>P1040

GCACUCGGGCUGCUGGGAGGUAGGCAGGGACUUGGGCCUGGGAGGUCGCGG

>P1041

CAACAGGCCACCGUGAGGGAGGAACAGGAUCGCACUCGGGCUGCUGGGAGG

>P1042

UGCAGUGAGCCAAGAUCGCACCCUUACACUUCAGCCUGGGCGACCGAGACU

>P1043

CUGGAGUGCAGUCUCCGUUCACUGCAGCCUCCACCUUCUGGGCUCAGGUGA

>P1044

CUGUCCGUCUAUGUAUUUGUUUUUUAAGAGAUGGGGUCUUGCCUUUUUGCC

>P1045

UAAAGGUGUAAGAUACGUUCCUGCCAGGAACGGCCCAUAUCAUGUGACCAG

>P1046

GCCCGUGUCGCCCACGGGACUGGUCACGUGAUACGGGGCUGCUCCUGGCAG

>P1047

GCAGAGUUUCCUGGGGAGAGGGCACAGCCUGUGCCUGAGGGCCUGGCCCUG

>P1048

CAUGUAACGGUGACCUCCUGGGCUCAAGGGAUCCUCCAAACUCAGCCCCCU

>P1049

AGGACCACUGUGUGCAGCACCUCCCAGACGGCUCUGUGACUGUGGAGUCCG

>P1050

CUGGCCAAAAUUUUGUUUUUAAAUUAGCCUGGCGUGGUGGGUGCGUGCCUA

>P1051

AAAAAAAAAAUUUAUUUGCCUGCAAAUGGAUUUACUUUAUCCUCGUUCUUA

>P1052

UUUUUUUUUUUUUUGACAGUGUCUCACUUUGUUACCCAGGCUGGAGUGUGA

>P1053

AGGAAUCGGAGGUUACAGUGAGCCAAGAUCAUGCCACCGUACGCCAGCCUG

>P1054

CAAUCCUUCCUACUCAGCCUCCUGUAGUGUCGAGAAUAUAGGCGUGGGCUA

>P1055

UGAGUUUGAUGGCUGCUGGAUCCUCACUCAACGAAAACUCGGUUGGAAACU

>P1056

CUUUCAAGGAAACUUCCCCUACUGAAAGGCAUAAAAAGGUUAAAAAAGAAA

>P1057

CCGGCUGCGGUAGCUCAGGCCUCUAAUCCCAGCACUUUGGGAGGCUGCAGA

>P1058

AGUGCUGGGAUUACAGGCAUGCGCCACGGCGCCCGGCCUCCUACAGUGCUG

>P1059

UAUUUGUAUUUUUAACAGAGAUGGGAUUUCACCGUGUUAGCCAGGAUGGUC

>P1060

GAGGCUGAGGCAUCAGAAUCACUUCAGCCCGGGAGGCGGGUGUUGCAGUGA

>P1061

AUUACAGGUGCCCGCCACCACGCUUAAUUUUUGUAUUUUUAGUAGAGACCA

>P1062

AUGGUGGCUCAUGCCUGUAACUGCAACACUCUGGGAGGCCAAGGUAGGAGG

>P1063

GCUGAGGCAGAAGGAGUUUGAGGUUACACUGAGUUAUGAUAUGCCACUGUA

>P1064

AGCUUUUUUUGUGAGACAGAGUCCCACUCAGCCACCCAGUCUGGAGUGCAG

>P1065

UUUAUGCUAAGGCAGAACUUUCUUUACUUUUUGAGAUGGAGUUUCGCUCUU

>P1066

AUAGUGAAACCCUGUCCCUACAAAAAUACAAAAAAAAAAAUCAGCCGGGAG

>P1067

ACAUUAUAAAAGUACAUUCAGGGCCAGGCGUGGUGGCUCUCACAUAUAAUC

>P1068

AGGCUGGUGUGGAACUCCUGAACUCAGGCUGUCUGCCCACCUCAGCCUCCC

>P1069

GACACAGGGUUUCGCCAUGUUUCCCAGGCUGGUGUGGAACUCCUGAACUCA

>P1070

GGCGGGUCUGGGGAGUGGAGCUGCCAGGAGGCCUCCCAUUUCUCACAGCCU

>P1071

AGCACGUUCCCUCACCAACUCCCCAAGAACGCCCAACCUCCAAGAGCAGAC

>P1072

ACAGGGUAAACUUCCCUUCCGAAUAAAACCACCAGAAUGCGGUCUGGCCAA

>P1073

AUCUCAGCUUCUGCUGAACAGGGUAAACUUCCCUUCCGAAUAAAACCACCA

>P1074

CGCUGACUGACAUUUUUGCCGAAUAAAGGGGCCCUGUGGGUUUCCUGUUCC

>P1075

GUUUCCCCACCUCACUCACACACUCAGGGACCCUCGGCUGCUCCCCGGGGC

>P1076

UCCUGUAGAAGGGGGAGCCCCCCAAAGUCCAUCUGUGUUUCCCCACCUCAC

>P1077

CAGUCCACUCCGGGUUUCCUGUAGAAGGGGGAGCCCCCCAAAGUCCAUCUG

>P1078

GGCAAAGCCGAGGGGAAGGGCCGGUAUGAAGGAGGCGCCCGAUGGGAGGGG

>P1079

CGGGAGGCAGGAGCUGUCCAGGCAAAGCCGAGGGGAAGGGCCGGUAUGAAG

>P1080

GACACAGGUGGGCCCUGCGGGAGGCAGGAGCUGUCCAGGCAAAGCCGAGGG

>P1081

UGGGUGGAGCGGCGCUGGGGGACACAGGUGGGCCCUGCGGGAGGCAGGAGC

>P1082

GGUGUCUGGCAGCAGAGAGGGGCUCAGGCCUGCUGGGUGGAGCGGCGCUGG

>P1083

CCCGAGCAGCAGGAGAGUGGGCAGCAGGGGGUGUCUGGCAGCAGAGAGGGG

>P1084

ACAAGCUGUGCCUCAGAACCCGAGCAGCAGGAGAGUGGGCAGCAGGGGGUG

>P1085

AAAGAGAAUCCGCCUCGGUGUGACAAGCUGUGCCUCAGAACCCGAGCAGCA

>P1086

GGCCAGAAGAGAAAGAGAAAGAGAAAGAGAAUCCGCCUCGGUGUGACAAGC

>P1087

UAGCAGGAUGAACUCCAGCAGAGGAACUCAGCGCCAUUGCUGCGGCUGUGG

>P1088

AGACCGGCAGCUCGGGAACCCAGCUAGCAGGAUGAACUCCAGCAGAGGAAC

>P1089

UGCUUUGGAGAGGAAUUCCAGGGGUAGGGAUGGUGGGAGAACAUUCCCACC

>P1090

UAAUUUUCAAAGUAUCCGGGAUUACAGGCGCCCGCCACUACACUAGCUAAU

>P1091

AGAUGGGGGUUUUGUCAUGUUGUCCAGGCUGUCCUUGAACUCCUGGGGUGA

>P1092

GGAGCCGAUGUGACUCACACCUGCAAUCCCAGGGCUUUGGGAGGCCAAGGU

>P1093

UCUGUUGCCUAGGCUAGAGUGCAAUAGUCCGAUCAUAGUCCACUGCAGCCU

>P1094

CCAUUGUGUUGCCCAGGCUGGACUCAAACUCUUGGGCUCAAGCUAUCCUUC

>P1095

UUGUACAGGCUGGUCUCAAACUCCUAGGUUCACUUUGGCCUCCCAAAGUGC

>P1096

UAACUUUUUUGUUUUUUGUUUGGUAAAGAUGGCAUUUCACCAUGUUGUACA

>P1097

CACGUGCCUAUAGUUCCAGCUGCUCAGGAGGUUGAGGGAGAAGAAUCACUU

>P1098

ACAAAAAUUAGCCAGGUGCGGUGGCACGUGCCUAUAGUUCCAGCUGCUCAG

>P1099

UUAGUGCAUACGUGUAUGUGCAGGAACACUCCCUGUAAGACUCUUGUUCUC

>P1100

CCAGCUAUGUCAAAGUGAAAUUUCCAUAAGAGUGUGAAUCUGUUUCUAAGC

>P1101

AAUAUAUGGCCAGGUGAGGUGACUCACUCCUAUAAUCUCAGUGCUUUGGGA

>P1102

GAGCCAGGGGAGGUGGAGGAGCUUUAGUGGUGCCAGAUCUCUGCAGCUUUG

>P1103

AUCCAUCAACAGUCAUAGGAAGCUUACAAAGCUUCGGAGAUCUGGCACCAC

>P1104

CUGGAUGUGGUGGCUCACAACUGUAAUCCUAGUGCUUUGGGAGGUCGAGGC

>P1105

UCCAGGGACCAAGUCGAGAUGGUGAAAACAAGACCAAGAUUGGAAAGCCAG

>P1106

CUCCCAAAAUGCUUCGAUUGCAGGCAUGAGCCACUGUGCCUGUCCAAGAAA

>P1107

UUGAAAUGGGCAGUGGUACCAUCUCAGCUCACUGCAACCUCUGCCUUGGGG

>P1108

GACAGGUCUUGAACUCCUGUCCUCAAGCAGUCCUCCCUCCUCAGGCCCUCA

>P1109

GCAGUGGAUCACGCCCAUAAUCCUAACACUUUGGGAGGCUGAAGCAGGAGG

>P1110

AGAACAGUAAACAUUUUUUAAGGCUAGGUGCAGUGGAUCACGCCCAUAAUC

>P1111

AAAUUUCCUUCUGCCUCUGUUUCUAAGGACACCUGUCUUCGGGCUCACCCA

>P1112

GUCUUCUCCUCUUCUGACUGUCUUAAAUUUCCUUCUGCCUCUGUUUCUAAG

>P1113

CAUCACUCCAGUCUCUGCCUCUUUCAGUGGCCUUCUCUGUGUGUCUGUGUC

>P1114

GAAGAAGCCUCCCUUGUCUCUCUUUAGCUUUGGUGACUGUCCCAGCAUUCC

>P1115

UGCUGGGACAGCCACCAAAGGUAAAAAGAGACAAGGGAGGCUUCUUCAGGC

>P1116

AUUUAAGACAGUCAGAAGAGGAGAAAACACAGACACACAGAGAAGGCCACU

>P1117

GGUGAGCCCUAAGACAGGUGUCCUUAGAAACAGAGGCAGAAGGAAAUUUAA

>P1118

CCUAUUUAGGUUAAGAUUUUGAAAUAAGACCAUCUUGGAUGAUCUGGGUGA

>P1119

GUCCUGCCUACCUCCCAGAGUCUCUAGGAGGAUCACACAAGAUGGGUCCUU

>P1120

UCCCGCCUCAGCAUCCUGAGUAGCUAGCACUACAGGCAUAUGUCACCAUGC

>P1121

AGCUCACUGGAACCUGGAACUCCUGAGCUCAAGCGAUCAUCCCGCCUCAGC

>P1122

AUCUCUACAAAAAUUAGCCAGGCAUAGUGGCAGGUGCCUGUAGUCUCAGCU

>P1123

CCAGAGUGCUGGGAUGAUUACAGGCAUGAGCCACCAUACCCGGCCUGAGCC

>P1124

GUUUUUACUAAAAGUACAAAAAACUAGCUGGGCGUGGUGGCAGGAGCCUGU

>P1125

CAGCAUCGUGGUGUCUCGCGAUAGGAUGACCAACCACCCCAGUUUGCCCAG

>P1126

CAGCUGUCCCCAGGCUCCCUGGCUUAGAUGCCCAGCUGUGGCUGGGUACAG

>P1127

CAGCCGGUACCCCGCUGGCUUGGACAGCCCCAGUGACCCUCAGUGACCCAG

>P1128

UCUGGGUUCCAUGGCAGCAGCCGGUACCCCGCUGGCUUGGACAGCCCCAGU

>P1129

GUAGCCUCGCAUCUGCUAGGCUCCCACCUUAGCCUCCUGAGCAGCUGGGAC

>P1130

CUUGAUUAGAACAUUGGCUUCAGCCAGGUGAGGUGGCUCAGGCCUGUAAUC

>P1131

AGCUAGCUUUUUACAAAACAGAGACAGAGUCUUGCUAUGUUGCCCAGGCUG

>P1132

UGUGGAUCUUCUACUGCAAAGGGGGAUCUAUUUCUGUUGCUGUGCUAAUAA

>P1133

AGAUUCUGUGGUUUUGCCCCAGUCAAGGCUGUGCCUAGGGUAGUCAGAUGG

>P1134

GUCUCCUCCCCUAGAAACAGCCUUGAGUGGGACAAAACCACAGAAUCGCAC

>P1135

GAUAUAGAUCCCCCUUGCUGAUGGAAGAUCCAGAACCCUGCUGGGGUAAUG

>P1136

AAUGCUAGCUGUUACUAUUUUUAUUAGUACAGCAACAGAUAUAGAUCCCCC

>P1137

GUAAUAUACAUGGCUGUGGCACUGUACAUGACUGUGACAAUGUGUGUGACG

>P1138

GCCAUUGGAAAUAAGAUCAUUGGCCAGGGGCGGUGGCUCACGCCUGUCAUC

>P1139

UUCCUUUUAAACAUUUUUUUUUUUGAGAUAGAGUUUUGCUUGUCACCCAGG

>P1140

GCUGACAAGUUCUCAAGAAGAACUUAUGAGUAAGCAGUCUGAGAACUAAAG

>P1141

CGCUGUUGCUGCUCGUGUUAAAUCUAGAACCGUAGCCAGACAUGGGACUGG

>P1142

CUCUCUGUCACCCAGACUGGAGUACAGUGACAUGAUCUCUGCUCACUGUGA

>P1143

CCCACAGAUAUAAGCCCAGACGCCUAGGCGAGAGUGAGACUGUGUCUCAAA

>P1144

UAAAACUUAAGUUCCUGUUCUUUCCACCUACAUUAGGCAAAUAUAGGAGGA

>P1145

UGUAUUUUCUACUGAAAAUACAAAAAUUAGCUGGGCACGGUGGCGGGCGCC

>P1146

ACCCUGUCUCAAUAAAUACAUAAAAAGGCCAGGCGCCGUGGCUCACACCUG

>P1147

UUUUGAGAUGGAAUCUUGCUCUGUCACCCUUGCGGGAGUGCAGUGGCACAA

>P1148

AGCAGCACUGAAUAGAAGGGGUCAUAACUAUAAAAAGGCAACAUGAUGAAU

>P1149

CAAAAUUAGAAAUGUCCUGUAUUCCAAUGAUCAUCCUGUAAACAUUUUAUC

>P1150

UCUUUAGUUAUUUUGAACCCCAAAUAUUUCCUCAUCUUUUUGUUGUUGUCA

>P1151

GGUUCAAAAAAAGUAAAAAGAUAAUAUAGCUGCAUUUCUUUAGUUAUUUUG

>P1152

CGCCAUGUUCUUGCAGAAAACGCUUAGCCUGAGUUUCAUAGGAGGUAAUUA

>P1153

UGUCUGCUUUUCCAGGCUCAGCGGCAUGCUGAUGGAAGUGGAAGAGCCUGA

>P1154

AACGAAGAAAAGAAGGAGAAGGGGAAGAAAAGAAGGGGAAGAAGAUCAAAA

>P1155

GAGGUCAGGAGAUCGAGACCAUCCUAGCUAACACAGUGAAACCCUAUCUCU

>P1156

GCAUGCUCUCCAGAGUGGCCCUCAGAACAGGGGCCCUCAGCCACCAAAUCC

>P1157

CAGUGACAGGUGGAGACGCUGGGGCAAGUGGCCCGGGAGAAGGAGGCGCUA

>P1158

ACUGGGUUAGGCCCAGAGUUGGGGUACUGCACGAUGAACAAGCUGGGGGAU

>P1159

GUUCGCCUGCCUCAGCGUCCCAAGUAGCAGGGACUACAGGCGUGCACCACC

>P1160

UGAAACCCUAUCUCUACAAAAAAUUAGCUGGGUGUGGUGGCACGCGCCUGU

>P1161

GGAGCAGCUAGCACAGGUAGGCCAAACUGUGUGUGGGGUGGUCUGGAGAGC

>P1162

CUUGGCCUCCCAAAGUGUUGGGAUUAGAUGUGAACCACCGUGCCCGGCUGG

>P1163

GGCGCAGUGGUUCAUGUUUGUAAUCAGAGGACUUUGGGAGGCCAAGGUGGG

>P1164

UGUGGGUGAGGGAAGUCUAAAGAAAAAUCCAGGCCGGGCGCAGUGGUUCAU

>P1165

AUUCUCCAGAAGGGGAUCACCCAGAAUGCACUUGACUACAUGAAAAAGCAC

>P1166

ACAAAGUAUCAUCAGAAUCAUCCCAAGACUCAUUUCCUGAUUCCUAAUUAU

>P1167

AAGACUCUAUCUACAAAAAAUAAAAAAAAAUUAGCCGGGCCUGGUGGCAUG

>P1168

AAGCCAACCUUGUAUUCCUGGGAUGAAACUAACUUGGUCAUGGUGCAUUAU

>P1169

CUCCUGAGUAGCUGGGACUACAGGCACGUGCCACCACACCCAGCUUGCUCC

>P1170

CCUCAAACCUUAAAAUGCUUGGCUUAGGGUUGAGCUCGGGGAACAGAACCC

>P1171

UGCUUUAUACAAGAAUUUUAAAAACACUGUCAUAUGGUUUAGUGUAGUUAA

>P1172

UCAGAGCAUGGAAGCACAUGCCUGUAGUCCCAACUGCUCAGGAGGCUGAGG

>P1173

UCACUCUGUUUCCCAGGCUGGUCUCAAACUGCUGGCCUCAAGCAAUCCUCA

>P1174

AAAACAUAUUUUUUUUUUGUAGAGAAGGGAUCUCACUCUGUUUCCCAGGCU

>P1175

AAAAAAAAAAAAAAAGCCAACUCCCAGCUGGGCGUGGUGGCUCAUACCUGU

>P1176

UUUUUUUUUUUUUUUUUUUGAGACAAAGACUUGCACUGUCACCUAGGCCAG

>P1177

AGACCAGCUUGGCCAUUCACUUUUCAGAUGUCUGUGAGCAGAGGACUGCAG

>P1178

AAUAUUCUCACCUUCAGAGUAUAAGAAUCAUUCUUAAAGCCAAAUAUUCAG

>P1179

GAGGCAGGAGAAAGGCAUGAAUCCAAGAGGCAGAGCUUGCAGUGAGCUGAG

>P1180

CUUUGUUACCACACCUUUCAUUUUCAAAGUAUGCUAUGUAAUUUUCUUUUG

>P1181

CUUGAACUCCUGGGUUCAGUGAAGUAGUCUGCCUGCCUCAGCUUCCCAAAG

>P1182

AGAUUGCACCACUGUGCUCUAGGCUAGGUGACAGAGCGAGAUGCCAUCUCA

>P1183

GGGAGGUGGAGGUUGCAUUGAGCCAAGAUUGCACCACUGUGCUCUAGGCUA

>P1184

GCCGGACGCAAUGGUGCACAGCUGUAGCCCUAGCUACUUAGGAAGCUGAGG

>P1185

AAACUUUACCAAAAACAAAGACACAAGCCGGACGCAAUGGUGCACAGCUGU

>P1186

UUGGGCCAAAAAUCAAGGUGUCAUCAGGUCUGUGACUCCUCUGGAGGCUCU

>P1187

CUGUUUGUCUCUUGUUUUCCCCACCACCCCGCCAACAUUUGAAAUUGUAGA

>P1188

GGAACCUUCAGUUCAUUGAAAAAUAACAUUUUGAAUAACACUUUGAAAAAU

>P1189

GUAACAAGAUGAGACUCCUGUCUCUACAAAAAAUUUAAAAAAAAAUUAGCU

>P1190

AAAAGCUAUGAAAGCUGGGCAUGGUAGCUCAUGCCUGUGCUUCUAGCAUUU

>P1191

AUUUGAUAAAACACGUAGCAUAGCUAGGUGGGGUGACUCAAACCAGUAAUC

>P1192

UAUGUGCCUCUGGUCCCAGCCACUCAGGAGGCUGAGGUGGGUGGAUUGCUU

>P1193

CUCACCGUUGGACUCAAACUCAGGUAAUUCUACUGCCUCAGCUUCUUGAGU

>P1194

UGGGAGGCUACAAGGGAGCCCAGCUAAUUUUUGCACUUUAGGGUUUUGCCG

>P1195

AUAAGUACUGCUUUUUUCUUUUGGUAGUCUUCAUCCUACCAAACAGGUUGA

>P1196

AGUUUGAAACCAGCCUAGGCAACAUAGUGAUAGACCGUCUUUGCAAAAAAU

>P1197

GAUAAAAUUUGCAGGCCGGAGCCAGAUGUGGUGGCUCAUGUCUAUAAUCCC

>P1198

GCUGAGCAUGGUGGCACACACCUGUAGUCCCAGCCACCGUGGAGGCUGAGG

>P1199

UGAGGUGGGAGAAUCGCUUAACCCCAGAAGUUUGAGUCCAGCCCAGGCAAC

>P1200

CCAGCCUGGGUGAAAGGGCAAGACUAUGUCUCAAAAAAAUAAAAAUAAAUA

>P1201

UUCAAGGAUGCAGUAAGCCAUGAUUAGGCCUCUGUACUGCAGUCGAAGCAA

>P1202

UCCCAGCUACUUGGGAGGAUCACUUAAGCCCAGGAGUUCAAGGAUGCAGUA

>P1203

GGUCUCAAACUCUUGAACUCCAGCAAUCCACCCUCCUUGGCCUCCCAAAGU

>P1204

ACCAAAGUAGGUAAGGGCAGGAAAUAUGGGCAUUUGGAUGCAGGUCUUAAC

>P1205

GGCAUGGCCACCAACAUGCGUGGUUAUUAACCAAAGUAGGUAAGGGCAGGA

>P1206

CUUUCCCUUAUCUACUUUGGAUAAUAACCACACAUGUUGGUGGCCAUGCCA

>P1207

GCCACUGUAUUCCAGCCUCCAGCCUAGGUGACAGAGCAAGACUCUAGAAGA

>P1208

CUAGGCCCAGCUAAUUAUUUUUUGUAGAAAUGGCAUAUUGCUAUGUUGCCC

>P1209

CGUGUCUCAGCCUCCUGAGUCGCUAAGAUGACAGGUGCCCGCCACCAUGCC

>P1210

CCUGGAAUGGAGAAAACAUAGAAUCAGUGAAACAAAGCCGUAGUCCAGUUU

>P1211

CCAGGCUGGAUCGCACAAUGGCGCAAUCUUGGCUCUGUGUAACCUCUGCCU

>P1212

GGAUAAAGCCAAUGGAUACUGUUUCAGAGCCCAAGAGCAGCGAAGCUUGGA

>P1213

GUCCCUACUAAAAAUUUUAAAAAUUAGCCAGGUAUGAUGGCACAUACCCAU

>P1214

GGAGACCAGCCUGGAUAACAUAGCCAGACCUUGUCCCUACUAAAAAUUUUA

>P1215

GAGGAGAUUCUUAAAGUUUGGGACUAUUGGUCUCUGUAGAUUAGAUCCUUU

>P1216

AACAAAUUCAAGAACUUUUUGGACCAGGUGCGGUGGCUCACACCUGUAAUC

>P1217

CAGCUACUCGGGAGGUUGAGGUGGGAGGAUCACCUGAGCCUGGGAGGUCUA

>P1218

CACUUGAGCCCAGGAGUUCAAGGUCAACCUAGGUAACGUGGUGAAACACCA

>P1219

CAUCCAACAGCGGAGUGGGGCAAGCAGUCAUCCAGAUCGCCGCAGCCCUGG

>P1220

GCUCUGUGGUCAGUGUGGCUGCAUAACAGUGGAUUCCAUGAAAGGAGUCAU

>P1221

UAUGCCUUCAGUAUUUAUUAGGAACAUCGAUAUGCUUGAAGUAGAUGAUAC

>P1222

AUUAAUGAAAUAAACUCAGGGUCUCACUUUGUCCCCAGACUGGAGUGCAUU

>P1223

AACAUAGUGAGACCUUGCCUCUACAAAUGAUUUUUAAAAAUUAACUGAGCA

>P1224

UUGGUGAAAUGAAUAUAUGGUUUUAAGGACUGACAAAGACCCGUGGAAGCA

>P1225

UAGAGACAACAUCUCUACAAAAAAUACAAAAACUAGCCAGGCAUAGUGGUA

>P1226

AGAAAAUACAAAAGUUAGGCGUGGUAGUGCGUGCCUGUAGUUCCAGCUAUC

>P1227

CGGCUAAUUUUUAUACUUUUAGUAUAGAUGGAGUUUCACCAUAUUGGCCAG

>P1228

GUGACUGGCGGAGGCACAGAGACCAAGGCCGCUUAACCUGGCUGGUUGCAC

>P1229

GUAUAUACUUGGCCUUGGAGACAAAAGACCAGUGUGAUCAAGGGCAAGGGC

>P1230

ACAGGAAGCCUGUGGUGACAGCCAGAUGAAUGUCACCUAAGUGUCCUGAAG

>P1231

CUACAAAGGGUGAUCAGAAAAAAUCAGUAGUGGUUGGAGACUGGAUGGAAU

>P1232

AUGAAGAGGACAGUGUACAUAUAAAAGACACAAGUUAGCCAGGCAUGAUGG

>P1233

AAUUUUUUUUUUUUUUUUCUAAAAGAGACAGAGUCUGGCUCUGUUGCCCAG

>P1234

GUAACAAAGCCAACACUGGCCCGGCACGUUGGCCCAUGCCUGUAAUCCUAG

>P1235

GCUCAAGCGACUCUCCAGCCUCCCAAAGUGAUAAGACUACAGGUGUGAGCC

>P1236

UGGACAUUUGGGUUAUUCCAUUUACAACUUAGUCUUAACCAAAGGUUUUUG

>P1237

CUACCCCUCCUUGCUACCAGCUCUAACUAGAGUCAGAUCCCAGUAUGCUUC

>P1238

UACACCUGUGGUCACCUGAAAUGAAAUGUCUUUUGGAGAAAAGACGUUGGC

>P1239

AUUUUUAGUAGAGACGGGAUUUCACAGUGUUGGCAACGCUGGUCUCAAACU

>P1240

CUUGGUCUGCCAAAGCACUGGGAUUACAGAUGUGAGCCACUGCACCCAGCC

>P1241

AGGGGUGCACCACUGCACCCAGCUAAUUUUGUCAAAAUUUUUUGUAGAGAC

>P1242

CCCAGCCUCCCAACUAGCUGGGACUAGAGGGGUGCACCACUGCACCCAGCU

>P1243

CUUGCUCCAUCACCUAGGCUGGAGUACAGUGGCACGAUCGUAGCUUACUGC

>P1244

UAGUUUUUGUGUUUUUCAUAGAGACAGGGUCUCACCAUGUCAUUCAGGUUG

>P1245

GCCUUUAGGAGUGAGGAUUUGGGCCAGGCAUGGUGGCUCACGCCUGUAAUC

>P1246

GAGGAAGAACUAGAGGAAGAGGAGGAGAAAGAAGAGGAGGAGGAAGAAGAA

>P1247

GCCCAAAAACAAGCAAAGCGGAGGAACCCAGACAGCCCUGCCAAAGCCAUA

>P1248

GCCACCAUGCCUAGCUAGUUUCUGUAUUGUUUUGUAGAGAUGGGGUUUUGC

>P1249

CGGCUGGCUGCAGUGGCUUACCUGUAAUACUAGUUUUUUGGGAGGCUGAGG

>P1250

GGUGAUAAAGUGAAACUCUGUCUCCAAAAAAAAAAAAAAGAAUUUUGGGCC

>P1251

GUAUGUGCCUGAAGUCUGUAAUCCCAGCUACUCGGGAGGGUGAAGCUGGAG

>P1252

UCUACAAAAAAUACAAAAAUUAGCCAGGCAUGGUGGUAUGUGCCUGAAGUC

>P1253

AACACAGGGAGACCCCCUCUCUACAAAAACAUUUUUAAAAAAUUAGCUGGG

>P1254

GGUGGGAGCAUCACUUGAGACCAGGAGUUUGGGACCAGGCUGGGCAACACA

>P1255

AGGCAUUUAAAAAAUUAAAAUGACCAGGCACAGUGGCUCACGCCUGUAAUC

>P1256

GCCUCCCCAAGUGCUAGGAUUACAGAUGUGAGCCACCACGCCCGGCCUUUU

>P1257

CGCAAGCAAGCCAGGACAGGAGAAGAGCGAGAAGAAGAGGAAGAAGAGCAG

>P1258

CAGACUUGGUGGAGUUCACAGAUGAAGAGGGAUAUGGUCGUUAUCUCGAUC

>P1259

CAAGCCGGCCCCAGCAAGCCGCAGGAAGCCGUCCGCUGAACCUCAGGUUCU

>P1260

AAACAGGCCAGGCAUGGUGGUUCACACUCAUAAUCCCAGCACGUUGGGAGA

>P1261

GGGAUGGGGUCUUGCUAUGUUAUCUAGGCUGGUCUCGAACUCCAGGGCUCA

>P1262

UCUGGGAAUCGAGUGGAAUGGGGCCAGGUGCGGUGGUUCGUGCCUGUAAUC

>P1263

UAGACGGGUAUAGUGCGAUGUGCCUAUAGUUCCAGCUAUUUGGGAGGCUGA

>P1264

CAGCUCACUGCAGCUUUGACCUUCCAGGCUCAAGUGGUCUUCCUGCCCCAG

>P1265

CACUACUAAUUCUUUCUUAACCUUAAGAUUCAACCUAUUGGCCAGGCGUGG

>P1266

UCUUGAUCCUGGGAUUAUGCUUUAUAGGAUGUGGUCCCACAGGCCUGUCAC

>P1267

AAAUUUGCAUACAGUGGCCAGCUGCAGUGGUUCACACCUCUAAUCCAAGCA

>P1268

UUCUGCUUCAGCCUUCUGCACAGCUAGGACUACAGGCGCAUGCUACCAUGC

>P1269

AUUGCAACCUUGAACUCCUGGGCUCAGGUGAUCCUUCUGCUUCAGCCUUCU

>P1270

UCCCCAUGUGUCAUGGGAGGGACCCAGUGGUAGGCAAUUGAAUCAUGGGGA

>P1271

CACUCUGGACCUAGCCUGUCCCAGAAGAGGAGAAUGCACAAGUGUAACUCC

>P1272

GCAGAGCAUGUUGACUGUAGUUAAUAAUAAUGUAUUAUGUAUUUCGAAAUA

>P1273

GGUCAACAGGUAGAAACUUAUAGUUAGAUAGGAGGAGUAGUUCUGGUGUUC

>P1274

UUAAAAAUAAGCAAAGGACUUGAAUAGACAUUUUUCAAAAGAAAACAUAUA

>P1275

AAGUAAAAUACUUAGAAGAAAAUUUAACCAAGGAAGUGAAAGAUCUAUACA

>P1276

UGGUUGAUCCCAUAUCUUGACUAUUAUGAAUAGUGCUGCAAUGAACAUGGG

>P1277

UUUUUGUCAUUGGCAUGAAUUUUCCAGAUUGGGAGAGGAGGUCUGAAGUUU

>P1278

GAGGCUAGAAGUCUAAUAUUAAGGUAUUAACUAGGCCAUGCUCCCUCAGAG

>P1279

GCCUCACAUAGUGCCACCAUCCCCUAGCUAAGUUCUAGUUACAUUGGCUCC

>P1280

AGGUGCCUGCUUCCCCUUCCGCCAUAAUUGUAAGUUUCCUGAGACUUCCCC

>P1281

UGGAUCAUGGGGGUGGUUCCCCCAUACUGUUCUCAUGAUAGUGAGUGAAUU

>P1282

CUCCAUGAUCCAAUCACCUCCCACCAGGCUCCACCUCCAACACUGGGGAUU

>P1283

UAGUGCUUGGGAUGCCUAGGUGGGAAGAUACUUAGAGGCCGGAAGUUCAAG

>P1284

GAGUGCAGUGAUGUUAUCAUGGCUCACCGCAGCCUCAACACCCUGGGCUUA

>P1285

AAUAGAGCUACCAUACAAUCCAGCAAUCCCACUCCUAGGUAUAUACCCACA

>P1286

UCUUGGCCUACAAAGUAAAUGGACAAUAUAUUAUGGAAGGACUUGCAUCCA

>P1287

UUAUUUUAUAUUUUUUAUUUUUGGUAGUGACAUGGGUCUUGCUGUGUUGCC

>P1288

AUUAGGGAAGAAAAGAAAGAAGACAAGGUGGAAAAGUUGCAGUUUGAAGAG

>P1289

GAGGCAGGAGGAUCCUUUAAGCUCAAGGGUAAGCAGCUGUAGUGAGCUUUG

>P1290

AAAAUUUAGAAAAUUAGCCAGUCUUAGUGGCAUGGGCCAAUAGUUCUAGCU

>P1291

CAAGGUGGUCUAGAACUGGCCUCAAACGAUCCUGUCACCUCAGCCUCCAAA

>P1292

GGGUCUCUGUCACCGAGGCUGGAGUACAGUGGCAUGGUCAUAAUUCAUUAC

>P1293

UUUGGAAGGCUGAGGCAGAAGGAUCACUUGAGCCCAGGAAUUUGAGGUUGU

>P1294

AAAUGGCCAAUAGUAUCUUAGUGAUACUAUGAAAAUAGUUCUGACCUCUUG

>P1295

CCUGAGCCUGGGAAGUCGAGGCUGCAGUGAGCCAAGAUCGCACCACUGUAC

>P1296

CACGGUGAGAGGGUCAGUUGAGCCCAGGAAGUGGAGGCUGCAAUGAGCUGU

>P1297

CUUAUGUGUUUAAAAAUUGUGGUCUAUGUGUGGUGGCUCAUGCCUAUAAUC

>P1298

UUUUUUUAAUUUUUAUUUUUUUGAGAUGGAGUCUCUGUCUCUCACCCAGAC

>P1299

GAUGUUGCCACUGCACUCCCAACUUAGGCAACAGAGCAAGACCCUGUCUUU

>P1300

UCAUUCCCUUUUUAAAAAAUUAGCCAGGCAUGGUGACAUGUACCUGUAAGU

>P1301

CAUGUGGGAAGGCAAAGGGGAAACAAGAUACAUCUUACAUAGCUGAAGGGG

>P1302

CAAAAAACCUACUUGAGACUGAGUAAUUUAUAAACACAAGAGGCUUAAUUA

>P1303

UGCCUGCAGAACUGUGAGCCAAUUAAACCUCUUUUCUUUAUAAAUUACCCA

>P1304

UCUCUCUAUCCCUCCUGCUCCAGCCAUGUAAGAUGAGCCUGCUUCCCCUUC

>P1305

ACUCGGAAGGCUGAGGCUUGAACCCAGGAGGUGGAGACUGCAGUGAGCCGU

>P1306

AUCUUCUCACCUAAGUCUCACGAAUAGCUGGGUCCACAGGCAUGUACCACC

>P1307

UGAGUCAGAGUCUCGCUCUGUUGCCAGACUAGAGUGUAGUGGCACAGAUCU

>P1308

GUAGCAUGUUGUAUAAACUCAUGCCACAAAAAAAAAGAAAAAAAAAAACAG

>P1309

UAUUUGAUGAGAAUGAAUGGGGGAAAGCUAUUAGGUCAGAGUAAGGAAUAG

>P1310

UUUUUUAUAUUAUAUGUUAAGUUCUAGGAUACAUGUGCACAACGUGCAGGU

>P1311

UUUUGUGGAGAUGGGGUCUCGAUAUAUUCCCCAGUCUGAUCUUGAACUUCU

>P1312

AGGUGCAUGGUGUCACACCCAGCUAAUUAAAACAAAUUUUUUGUGGAGAUG

>P1313

UAUAAAUGGAACCAUACAACAUGUGACCUAUUGUAUGUGGCUUCUUUCACU

>P1314

UCCCAAAAGAAAUCCUGUAACCAUUAGUGAUCACUUCCCACUACCUCCUCC

>P1315

UUCACUCUGAAAAAGUAUACAAUUAAGUGGUUUUUACUAUAUUCACAGAGU

>P1316

GACUGAAGUUUGGAAAUACUACCAUAUAAGGUAUUACUGAAUGUAACAACU

>P1317

CUUUAAGGAAAAGAGCAUAAGAUAUAUGCAAAUACUGCAAAGAUUAUGAGG

>P1318

CAUGAUAAAUAAAUAAAGAAGAGAAAGUGGCUUUAAGGAAAAGAGCAUAAG

>P1319

UAAGGCUAUCAUACACGUAAAGCAUAUGAUACUGCAAUUUCAUUUAUCAUG

>P1320

CUCCUGUCAGAUCAGUGGCAGCAUUAGAUUCUCAUAGGAGCACGAACCCUA

>P1321

UUUAAAAUCUGUUUUUAGGCUGGGCAUGGUGACUUACACCUGUAAUCCCAA

>P1322

ACAUCCCCAUAUUAGUAUAGUAAAUAAGAAAAAGCAUUUGACAAUAUUCAA

>P1323

GAACCUUUUUUCUUUUUUUAGAGACAGGGUCUUGCUAUGUUGGUCAGGCUA

>P1324

CUUCAUGGCUUCAUCCCCUGCCCAUAGCAGACAUUGCUAAUCAAUCCUCUG

>P1325

GGAGGAUCACUUAAGCCCCAGAGGCAGAGGUUGCGGGAGCUGAUAUAGUGC

>P1326

CUAGGGAGGCUGAGGUGGGAGGAUCACUUAAGCCCCAGAGGCAGAGGUUGC

>P1327

UGUCUGUGGGGCCUUGUACCUGACUAAGGCCUUUUCCUCAGUCAGAUGCUU

>P1328

CUCAGCCUCCUGAGUAGCUGGGACUACAGACUUUUUUUUUUUUUUUUUUUU

>P1329

GUUAAUUUAUUUAUUUUUAGUUUUUAGAGACAGAGUUUCGCCACUUCGUCC

>P1330

UUUAGAACAUUUUCAUCUUCUCUAAAAUAAACCCUUGUCCUCAUUAGCAGU

>P1331

GAGUAUUGCUUGAGGCAAGGAGUUCAAGACUGUAGUGUAUAAUGAUUGUGC

>P1332

CAUACCAUGCCUGGCUGAUUUUUGUAUUUUUAAUAGAGACGGGGUUUCACC

>P1333

UGUCUCCAUAAAAUUUUUUAAAAAAAUGCCAGGUGUGGUCGUGUGUGCCUG

>P1334

GAACCACAUCUUUUGCCUCUGUAUCAGAUGCCACUGUAAAGUUCAGGAUGG

>P1335

CAGGUUUCUCAGCUGUAGAAUAGGGAUACUAACCCUUGCUGUGAAAAUUAA

>P1336

AAAACAUUUAUAAACCUUCGUUUAUAAAACAAAACAAAGGUUUGGUUUUGU

>P1337

AUAUCCCAGUAACCCUUCAGUCACAAGCAAACCCAAACAGCCCUUCAUUUC

>P1338

UGGUCUCAAACUCUUUGGGCAAGCAAUCCUCCUGCCUCAGCCUCUCAAAGC

>P1339

CGUAUUUUUUGUACAGAUGAGGUUUAGCCAAGUUGCCCAGACAGGUCUUAA

>P1340

CUAGUAGCUGGGACCACGGAUGCACACCACCACACCCAGCUAGUUUUCGUA

>P1341

CAUACGGACAGGCCUAGUGGCUCACACAUGUAUCCCAACACUUUGGGAGGC

>P1342

GCUUGCAGGUGGCCACCUUCUUGCUAUGUUCUCACAUGGUGCAUGCAUUCA

>P1343

ACAGAGUGAGACCCUAUAUCAAAAAACAAACAAACAAACAAAUGAAAGAGA

>P1344

AAAAUAUUUCCAGAUGAGUGCCUAUAGUUUCAGCACUUUGGGAGGCCGAGG

>P1345

CUAUGAAAUUACUGUCUUUAAAAGAAGUCAGUUGUAGGCCGGGCACAGUGG

>P1346

ACUGCAUCCUUGACCUCCUGGGCUCAAGCAGUCCCUCUGCCUUGGCCUAGG

>P1347

GAUUAGUUUCUCCUUUGAUAUACACAUUGAAAGCCCUAAAUAAAAUGCUAU

>P1348

UACGGUAUGACUCCAUUUACAUGCAAUCCCAGAGCAGGCAAACUAAUCUAU

>P1349

AAUUGUAUAGUAGACUGUACACCUAAGCUAUAGUAGGCUAUACAUCUAGGU

>P1350

GUGUACACCACCACACCCAAGAAUCACUUGAACCCGGGAGGUGGGGGUUGC

>P1351

UUUUCUUUUUUUAGAUAAUAAAGAUAGGGUCUCACUAUAUUGCCCAGGCUG

>P1352

UUACAUUUGGAGGGAAUAUUAAAAAAAAAAAAAAAGAAAAAAAAACUGGCU

>P1353

GCAAAAUCCUGUCCCUACGAGAAAUACAAAAAUUAGCCAGGUGUCAUGGUG

>P1354

UUGGGACCAGCCUGGGCAACAUGGCAAAAUCCUGUCCCUACGAGAAAUACA

>P1355

UGUGGUGGCAGUGGCUCAUGCCUAUAACCCCAGCAUUUAUGGAGGCCAAGG

>P1356

CAGCCUCCUGAGUAGCUGGGACUAUAGGUGCACACCACCAUAUCUGGCUUU

>P1357

AGGAAGGCUGCCUCCGCCAUGGGAAAGGAAUGAGGCUGAAAUGGGCUUCAU

>P1358

ACAAAAAAAGAGAAAAAUGUCAGCUAGGCAUGGUGGGCCUGUAGUCCCAGC

>P1359

GCAUCUGACAAACUACUCCGUGAACAAGCAUAAUGAGCAUUUUGAACGGGA

>P1360

AAAUCACCCUUAAAUCAACAAAAAGACCUCAUGCUCAUGCAGUGGACAUUU

>P1361

CCUGUCUCAAAAAAAAAAAAAAAUCACCCUUAAAUCAACAAAAAGACCUCA

>P1362

CUAUUUGGGAGGCCGACACAUGAGAAUCGCUUGAACACUGGAGGCAGAGGU

>P1363

GAUUAAUUGUAAGUCUUGGGGGAAGAAAUUCAAGGUAAUGUAAAAAAAUCA

>P1364

UAGCCAUUUCUCAACAGUGAGAUAUAGCUAUUCUUCUGUAGGGCGAAUGGC

>P1365

GAGAUUACCCAAGUUCUCGUGAUACAAGAGAUUAUGCACCACCACCACGAG

>P1366

GAGGUCCACCUCGAAGGGAACCGCUACCCUCUCGUAGAGAUGUUUAUUUGU

>P1367

GGCUCAGUUUUGUAUUUUUAGUGUAAAUGGGGUUUCUCCAUAUUUGCCAGG

>P1368

GUUAAAAAUCAACUCAUUGCCUGUAAUCCCAGCACUUUGGGAGGCCGAGAC

>P1369

AGGCUCAAGCAGUCCUUUCACCUCAACCCCCCACAUAGCUGGGACUACAGG

>P1370

UGGGUCAUUGUAGCCUCAACCUCCCAGGCUCAAGCAGUCCUUUCACCUCAA

>P1371

AUUCUGCUUUGGAUUCUUUGUGUAUAUGCCCAGAAGUGGGAUUGCUGGAUC

>P1372

AAGGGUAUCUUAGGCUGGGCACGGUAGCUCCCGCUUGUAGGCCCAGGCCUU

>P1373

UAAUUUUUUAUAUCAUCACAUUAAAACUACCAUGAAAAAUAAAAUGGAGUG

>P1374

CUACAGGCUUUAACGCCUAGCCUCAAGUGGUCCUCCCACCUCAGUCUCCCA

>P1375

AAUUUUAUUUUAGAGAUGGGUUUUCACUGUAUUGCCCAGGCUGGAGUGCAG

>P1376

AGUACCAUCCCCCUAGUGCUGCCUCAUGAUAGAGUUCUCACAAGAUCUGGU

>P1377

GUAUUGCUUUUUCAUCCUGACUGGAAUGCAGUGGCACAAUCAAAACUCACU

>P1378

AUCCUCGUGUAUCUCCCAAUGUAUUAGGAUUACAGGUGUGAGCCACCACAU

>P1379

AUAGUGCUUUCACUUAGAAAAGGCUAGACGUGGUGGUUCAUGCCUGUAAUC

>P1380

CCUCCAGGACUGUACGAAAAUAAAUAUUUGUUGUGUAAGCACCCCCAGUGU

>P1381

CAGAAACCAAUCCUGGUGCACCUUAAUCUCAAACUUGAGCCUCCAGGACUG

>P1382

CUCUCUCUUUGUAUACACAGAGAAAAAGUCAUGUGAUGACACACCAAGAAG

>P1383

UAAACAACAGAAAUUUGUUGCUCACAGUUCUGAAGACUGGUAAGUUCAAGA

>P1384

AAUGUAGAGUUGCUCUGUUUUCUUCAACUGUAUUUAUUGCUGCAUUUCUCA

>P1385

UUGGUUUCCAAUAGAGCGCUCUCUUAUUGCUGUAUGCUUUGUAGUAAUUCU

>P1386

UGCAAUGACGUGAUCUUGACUCACCACAGCCUCUGCAUCCAGGAUUCAAGC

>P1387

GGAUACCCUUGAGGAUAUGGCUUGGAGGUUGGCGGACGGUGUUAUGCAGUG

>P1388

UUGGUAGGAUAGCGGAUGACGGUCCAGCUGUGGAUGUUGUAGACCCUGGGA

>P1389

CUGUUUCUAUAAAUUUGACUUUCCUAGGUACCUCAUAAUAAAUGGAAUCAU

>P1390

AUUGAAUCAAGGCGUUAAAGAAGUUAGGUUCUGUGUGGGAGAAAACUAAAA

>P1391

AGUCUGGGAGGUUGAGUCUGCAAGUAAGCUGGUAUUGUGCCACUGCACUCC

>P1392

CUGCAGCCACAACCUCCCAGGGUUAAGCAAUUCUCCCACCUCAGCCUCCUG

>P1393

AUUCUUGUGCCUCAGCUGGGCCUACAGGCGUGUGCCACCAUGCCCGACUAA

>P1394

UGGAUUUUUUUCGGUUUUUUUUUUAAGAGAUGAGGUCUGGCUAUAUUGCCU

>P1395

UGCUUGAACCCAGGAGGCGGAGGUUACAGUGAGCUGAGAUCGCACCCGCCU

>P1396

GAUUGAUCAGAAUUCUGGAAUCUGAAUAUUAAAACCUUACUUAGUGACUGG

>P1397

AGGCUGGAGUGCAGUGGUGCAAUCUAGGCUCACUUAUCCAGGUUCAAGUGA

>P1398

UUUCACUUAUCAUAGACCAGUGUUGAGUCCAAAUUUUAGUCAAUCAAGCAA

>P1399

CGGUACCUCAGAUGGAAAUGCAGAAAUCACCCGUCUUCUGCGUCGCUCACA

>P1400

CACUCCCUAGUGAAAUGAAUCCGGUACCUCAGAUGGAAAUGCAGAAAUCAC

>P1401

GGACCCUCGGAGCCACGUGUGGGAUAUAAUGUCGUGGUGCGCCGUUUUUUA

>P1402

GCUUCCCGGCUGCUUUGUUUACCUAAGCAAGCCUGGGCAAUGGCGGGCGCC

>P1403

ACUGUGGUGGGCUCCACCCAGUUUGAGCUUCCCGGCUGCUUUGUUUACCUA

>P1404

AGAGGUGGAGCCUACAGAGGCAGGCAGGCCUCCUUGAACUGUGGUGGGCUC

>P1405

AGGGACAUUUAAGUCUGCAGAGGUUACUGCUGUCUUUUUGUUUGUCUGUGC

>P1406

ACUGCUCUCUUCAAAGCUGUCAGACAGGGACAUUUAAGUCUGCAGAGGUUA

>P1407

UCUCCAGCUGCAUGCUGGGAGAACCACUGCUCUCUUCAAAGCUGUCAGACA

>P1408

UGUGUGUGUGUGUGUGUGUGUGUGUAGAGAGAGAGAGAGAGAGAGAAAAUG

>P1409

CUUGGCCUCCCAAACUGCUGGGAUUACAGGCAUAAGCCACUGUGCCUGGCU

>P1410

AGUAUAAGAGACAGGGUUUUACCAUAUUGCCCAGGCUGGUCGCCCUGAGGU

>P1411

UUGCUAUAAAAAAUAAGGAUUGGGAAGCUGAGGUGAGAGGAGGACUUGAAC

>P1412

GGAGUGUGGAGUGUGCAGUGGUGCAAUCUUGGCUUACUGCAACCUGAGCCU

>P1413

CAAGAGAACUUUGAGCCUGGCCGACAUGGCAAAAACCUGUCUCUACUAAAA

>P1414

GCAGAUGGCUUGAGUCCAGGAGUUCAAGACAAGCCUGGGCAACAUGAUGAA

>P1415

UGUUGGACUAAUGGCCUGUAAUCCCAGCUCUUUGGGAGGCCGAGGUGGGCA

>P1416

UGUUCUUUCUAGAUCAACAGUUCUCAGAGUGUGGUUCCUGGAUCAACAGCA

>P1417

GGAAGAAACACUGCUUCCUGCCACCAGGGCUGAAGACGCGAGCCUGUCCUC

>P1418

GAAGGAAGGAAGGGGAGGGAAGGAGAGAGAGAGAGAGGGAAAGAAAGAGAA

>P1419

CGUUAAGCAGAUGAUGAUAUGUAUUAGGCCGUUCUUGCACUGCUAUAAAGA

>P1420

GGAGUACUGCUUAAGGCCAGGGUUCAAGACCAACCUGGGCAACAAAAAGAG

>P1421

UGCCUAGCCACUUUAUUUAUUAUAGAGACAGAGUCUCCCUAUGUUGUCCAG

>P1422

CAGUCCAGGAAUUCGAGACCAGAUUAGGCAACAUGCAGAAACCUUGUCUCU

>P1423

UGGGCUCAAGAGAUCCUCCCACCUCAGCCUCCCUAGACUACAGGUGCACGU

>P1424

AGCCCCAGCGAGAGCAUGAGAGGAAAAGAGAGGCCCUCAGCUGCUGGGGAG

>P1425

ACAGUUUUGAAAGACAUUAAUUCUCAUUUUCUUCUAGUCAGGAGUAAUACA

>P1426

UCUCUACCAAAAAUACAAAUAAGUUAGCUGGGCAUGGUGAUGCACUCCUAU

>P1427

UGAGACCAGCUGAAGCAACAUAUUAAGACUCUGUCUCUACCAAAAAUACAA

>P1428

GAGGCCAUGGUGGGAGGAUCACUUGAGUCCAGGAGUUUGAGACCAGCUGAA

>P1429

AUAAUAUAUGGCCUCCUCCCAAGUAACUGGGAUUACAGGUGCGUGCCACCA

>P1430

UUUAGAAAAUAGAGAUAAGCCAGGCAUGGUGAUGUGUACCUAUAGUCCCAG

>P1431

CUUGCCUUGGCCUCCCAAGUAGCUGAGAUCACAGUUGUGUACCACCAUUUC

>P1432

AUCAUUAUUAUUUUUUGAAUAAUCUAUAAUCAUAAUUGUUCUUUUCUUUAG

>P1433

GGCGAGGGGUCAGGAGCAGUGAUGAACAUACUGAUGCUUAUGCUGUUUCCU

>P1434

GCAUCAUUUCUACCAUAGUACUUCAAGGCUCUACAGACAAUCUGAUGGAUG

>P1435

AGUGAAGCUAAACUCAAAAUGGGAUAUCUGAAGACUCUGUAAAACAGUUGG

>P1436

UUCAUUAUGCAAACAAAUAUAAUAUAAUGUUAGUGAAGCUAAACUCAAAAU

>P1437

CAUGCGUAUCUAUUUUUCCUGAUUCAGGCCAUUUCAAAGUUGAUAACAUCA

>P1438

AGUCUCAUCUCUACUUCGUACCUCUAUAAUGUGUAAACAAUAUGGUAAUGA

>P1439

UCAUGAGAUUCUUCCUAAUUUGGUAAGUUGUUCUGCAAAAAACCUUCGAGA

>P1440

UCUCUUACUCUCUUGGAGCUUCUCAAGCCACAGGUGUCAGUCAUAUUGGUU

>P1441

CUCUACGCUGCAAAUUUUGGGUCUCAAUUUUUACUGUGCCUUUGUUUUUAC

>P1442

AAGAGCCUGAAGUCUUGCAGGACUCACUGGAUAGAUGUUAUUCAACUCCUU

>P1443

AUACAUUGUCCAUCAUAGACUUUUUACAUUAAUUGUGAUUUUUUAAAAAUG

>P1444

CCCUAAGUUGCAAAAUUUAAGGGUUACUAUAAAACUCAUUAAUCAAGAUAC

>P1445

CACAGCCUGAGCUCCGCCUCCUGUCAGAUUAGCAGACAUUAGAUUCUCAUA

>P1446

AAUUUUUUUCCUCUCUUGUCCUUUUAGGAACUCCUGACUUUCUUCCCAAUU

>P1447

GACCCAUUUAAACUGCAUCUUAGCUAGGUGCAGUGGGGUAUGCCUAUAGUC

>P1448

GGGCCCACUUAGUCCUCAUGGCAGUAUCUAUUCUUAUUCCUGCAUCUCCAU

>P1449

GUUGGGAUUACAGGCGUGAGCCACCAUGCCCGGCCCCCCUCAAAUGGUUUU

>P1450

AACUGUAGUAAGCAUCCAAGGGGCAAGGCCAAGCUGGGUGUAGUGGCAGGU

>P1451

GAGCAAAACUCUGUCUCCAAAAAAAAAAUAAAACAUGGUUCCUGCCCUCAA

>P1452

UGUAAUCAUGGCUCACUACAGUCUCAGCUCCUCACUACGAGGUCAGGAGAU

>P1453

UUUUUUUAGAGGCAGGAUCUUGCUCAGUCACCCAGAAUGGAGUGCAGGGUG

>P1454

UAAGACAGGAGGAUCACAAGAGCCCAGGAGGUUGAGAAUGCAGUGAGCUUG

>P1455

GUGGUCCCUGCUAUGCAGAUGUCUAAGACAGGAGGAUCACAAGAGCCCAGG

>P1456

CACAAAAAUAAAAAAGAAACUAUCCAGGAGUGGUGGUGUGUGCCUGUGGUC

>P1457

GACAACAUAGUGAGACCUUGUCUCCACAAAAAUAAAAAAGAAACUAUCCAG

>P1458

GUGACACUCACUUGUAGUCUCAGCUACUUGGGAGGGUGAGGCAGGAGGACU

>P1459

AGUAAGUGAGAGAGAGUGUGUGUGUAUGUGUGUGUGUGUGUGUGUGCGCAC

>P1460

UUACCUCAUUUUUAUCUCUUCACUCACAGGUAACUAGUUCUCCCAACUGUA

>P1461

AAAGACUCCUGGGUUUUCUUUUUUAAGAUGGAGUCUUGCCCUGUCACCCAG

>P1462

UAAACACCUUCAGUGCCCCUCAGCCAAGUCCCUUUUGACCAUGGAAUACCA

>P1463

GAACCUUUGACAUCUGAAAACAACCAAGGAUCCAUCUGGGCUUCUCUUCCC

>P1464

AGAAGCCAGACAUACAAAAUUAGCCAGGUGUGGUGGUGCAUGCCUGCAAUC

>P1465

AGCUCUUUGUCCCAAGAGUUACUCAAGAGGUUGAGGUGGGAGGAUUUCUUG

>P1466

AAAAAAAAAAAAAAAAAAAUUAGCCAGACAUGGUGGCUCUUGGUUAUCCCA

>P1467

AUAUGAGGUCUCUCUCUGUUGCACAAGCUGGUCUCGAACUCCUGAACUCAG

>P1468

CACAUCUCUGGUCAUGGUCAUGGCCAGGCUGGUUUUGAACUCCUGACCUCA

>P1469

GCAUGCCUGUAAUUACAGCUCCUUGAGAGGCUGAAGCAAGAGAAUCGCUUG

>P1470

ACUAAAAGUACAAAAUGAGCCAGGCAUGGUAACGCAUGCCUGUAAUUACAG

>P1471

GAUUGAAUUUGCCAGGAAGAGUGUGAAUAGUGCCAAUCAGAAAAUUCAGGA

>P1472

UUCUCAGCUCCGUUCUUCUGUUCACAUGAUUGAAUUUGCCAGGAAGAGUGU

>P1473

CUCACCAAAUCAGAGCUCUUGGUAGAACAGUACCUCCCUCUCACUGGGGAA

>P1474

CUCAGGUUGUUGCCAAUGCCAAAGGAGCUGUGACUGGGGCAAAAGAUGCUG

>P1475

UAUUCUGAAUCAGCCAUCAACUCAGAUUGUUGCCAAUGCCAAAGGUGCUGU

>P1476

GACUCGGGUGGUCAACCUGCCCUUGAUGAGCUCCACAUAUGACCCCAUGUC

>P1477

UGCAGUUGAUCCACAACUGAGUGUGAUGACUCGGGUGGUCAACCUGCCCUU

>P1478

AUUUUUAGAAUUUUGCAUUUUUAGUAGAGACAGUUCAAGACCAUUUUGCCA

>P1479

CGUUCUACUUUGCAUGAAGAAAAACAGGAUUUUUAGGCCGGGCGUGGUGGC

>P1480

AAAAACAAAAAACACCAGGCGUGGUAGCACCCAUCUGUAAUCCCAGCUACU

>P1481

CUUGCUGGGCCUGGCUUGAUGGCACACGCCUGUAGUUGCAACUACUUGGGA

>P1482

AGUUCGAGACCAGAGUGGGCAACAAAGUGAGACCCCAUUUACAAAAAAUAU

>P1483

GGCAUAUCUAUUUAUUUUUAAGACAAGGUCUCAUGCUGUUGCCAACGCUGG

>P1484

CAGAUCACAGGAGGCCAGGAGUUCAAGACCAACCUAGCCAACAAGGUGAAA

>P1485

UUUUUUUUUAAAGUAGCCUCUAGCUAGGUGCAUACCUGUAAUCCUAGCUGC

>P1486

CCACACACAAACACCACACACACACACACCCCGCACACACCACACAUCACA

>P1487

CUAAAUACAAAAAUUUUUUGGCGACAGAGUCUCUCUAUGUUGCCCAGGCUG

>P1488

CCUUUCUUCCUUUCUUUCUUGUCUCACUUUGUCACCCAGACUGGAAUGCAA

>P1489

UGGGCUGCCUUCAUGUCCAAUUCAAACACAUCCAUGUAGUGGAUGACAUGU

>P1490

CUUGUUUAGCCAGGUGUGGUGGUUCAUGCCUGUAAUCCCAACAUUUUGCGA

>P1491

CUUUGAGUUUAGGAGUUUGAGACCAACCUGCGCAACACAGUAAGACCCCGU

>P1492

ACUGGGCGCAGUGCCUCACCCUGUAAUCCCAGCACUUUGCAAAGCCAAGGC

>P1493

UCCCUACCCUCCGCUUGAACCUAGGAGGCGGAGGUUGCAGUGAACCGAGAU

>P1494

UAGAAAAAAAUUAGCCAGGAGUGAUAGUGGAUGCCUGUAAUCCCAGUUACU

>P1495

AUAAAAUGUCCCAUCCCUUCGUGGUAGCAUGCACCUGUAGUUCCAGCUACU

>P1496

UUAAGUGCCAAAUUUUAUCCUGAAAAGUUUGUAGGCUGGGCACAAUGGCUC

>P1497

ACAAUAGAAUAAAAAUAAAGGGGCCAGGUGCGAUUGCUCACGCCUGUAAUC

>P1498

UUAAGAGCUUGCUGGCCACACCAAUAGUCUCAGCUACUCUGGAGGCUGAGG

>P1499

AGGCUAGAGUUCAGUGGCGUAAUCUAGGCUCACUGCAAAACCCGCCUCCUG

>P1500

UGAUGCACCUGCCUUGGCCCCCGCAAGGUGCUGAGAGUACAGGCAUGAGAC

>P1501

GGCUAGACUCGAACUGGUGACCUCAAGUGAUGCACCUGCCUUGGCCCCCGC

>P1502

CAUUCCAGCCUUGGCAACAGAGCAAAACCCAGUCUUAAAAAAAAAAAAAAU

>P1503

AUGCCACCACGCCUGGCUAAUUUUUAAUUUUUUUGUAGCGAUGGAGUCUCA

>P1504

GAAAAAGGGGCCUAGGGCCUGGCAUAGUGGGUUAUCCCUAUGAUCCCAGUG

>P1505

AUUUCUACAGUGCGUUUAUGUAAAAAGGUUUUGAUGUCGCUGGGCAUGGUG

>P1506

UCGGGUGAGGUGACUCAUCCCUGUAAUCCCAGCACUAUGAGAGGCCGAGGC

>P1507

AGACUGGCCUGGCCGACGUGGUAAAACCCCAUCUAUAUUAAAAAUAACAAA

>P1508

UUUAAAUGCCUUAAAAAAUAGUAAUAGGCUGGGCUUGGUGGCUCACUCCUG

>P1509

AUUUUAUUUGUAGAGACAGGGUCUUACCACAUUGCUCAGGUUGUUCUGGAA

>P1510

CAGCCUUGACCUUCUAGGCUCAAGCAGUCCUUCGUCUAAGCCUCCUGAGUA

>P1511

UUUUGUUUGAGGGGGUUCUUGCUCCAUCACCCAAGCCAGAGUGCAGUGGUG

>P1512

UCAUGCCUUGUUGAAAACAUUAUACAUUGUCUUCAUGGAAUUCCUCCUUAU

>P1513

CUCUCUCUCUUUUUUUUUUAAGACAAUAUCUUGCUCUGUCGCCCAGGCUGG

>P1514

UUUUUUUUUUUUUUUUUUUUUUAAGAAAAAAAAAAGCCCAUAGGCAGAGCA

>P1515

AAAAAAAGCAUAUGAAUGUGUGCAUAUGUAUAUAUAAAUUAUAUACACAAG

>P1516

UGCAAUCCUGUAAUCGAAGCACUUUAGGAGGCCAAGGCACCUGAGGUCAGG

>P1517

UGAGAUGGAAGGGUCCCUUGAGCCUAGGAGGUCAUGGAUGCAGUGAACCAA

>P1518

CACAUACCUGUAUUUCAGCUACUUAAGAGGCUGAGAUGGAAGGGUCCCUUG

>P1519

CCAUAAUGGUUUGUUGGGGGUGAGGAAAAAAACCCACAGGGACCAGAAUGU

>P1520

CUGUUUGAAGUACCCUUUUCCCUCCACUCCAUUAUAUCUGAAGCCUUCUCA

>P1521

AGACCAGCCUGGCCGCCUUGGCGAAACCCCGUCUCCACUAAAAAUACGAAA

>P1522

AACAUACCAGGAGGAGGGGACAGGUAGGGUGCAGCUCUGCCACAUUGCCCC

>P1523

GAGGGAGGGAGGGCCGGCCGGGCGCAGUGGCCCAAGCCUGUAAUCUCAGCA

>P1524

UCCGCCCGCCUCGGCCCCCCAAAGUACUGGGAUUACAGGCAUGAGCCACCG

>P1525

UCUCCUCAGCCCAGCACUCUGGUGAAACUGCAUCCUCUCAGAGGCCACUCA

>P1526

AGAAAUACAAUUUUUCAGUCCAAGUAUGGUGGCUCAUGCCUAUAAUCCCAG

>P1527

AGGGAUUGCUUCCUCCUGGUAUGACAAUAGAUUUGUUUUGAGGCAAAGCAG

>P1528

CUGUUGAAGGCCUGAAUGUAACAAAAGGGCUUAUUCUUCCUCAAGUAAGGG

>P1529

ACACCUGGCUAAUUUUUGUAUUCUUAGCAGAGAUGGGAUUUCCUCAUGCUG

>P1530

GGUAUCCCUAGUAAGUAGGACUACAAGAGCCCGCCAUCACACCUGGCUAAU

>P1531

AUGCUCAAAGUGUGCAUGCUGGCAUAGAACAAGUCACUGUUUACAAGCCUU

>P1532

CACAAUCUUCAUAGAAUUGUUGGUAAGGAUUAAGUGAGUUGAUAUGCUCAA

>P1533

GUUGUACCAAGUCACUGUCAUCCCCAAACUUUUCAAUGAUUCCAGGCCUUU

>P1534

CCCCCAGUUCCACAAGAUCCAACCAAGAAUUCUGUAUCCUGGGACAGUCAG

>P1535

UGAGUAGCUGGGACCGCAGGGGCAUAUCACCAUACCUAGCUAAUUUUUGGU

>P1536

GAAACCUGAAUGAAUUCAUUGGAACACCCUGUCUUUGACAGUAUGAUUCUU

>P1537

CUCUGACCCUCAAAGAGAAACCUGAAUGAAUUCAUUGGAACGCCCUGUCUU

>P1538

GAACUUAAAAUGACAUGACAGUUUUAAUUAUCUUGUUCUUCGUCCAAGAGU

>P1539

UCUUGGACAGAGGACAAGAUAAUUAAAAUUGUUGUGGCCUUCUAAGCUCUU

>P1540

UACUGGCUAAAUGCUCAUCUUGGACAGAGGACAAGAUAAUUAAAAUUGUUG

>P1541

UAAAACAUCAGCUUUAUUUCUCUCUACCUACUGGCUAAAUGCUCAUCUUGG

>P1542

GAUAAAGUAUUUAUGAGCCUUUGGGAGAGUAUAUUGUAAAACAUCAGCUUU

>P1543

GGACCAGACUGUGCGCGUUUGGGAUAUUUCUGGUGAGCUGCCCAAGUUCAG

>P1544

AGAUACUAUCUCUAUUGAAAAUAGAAAUACCAUCUAUUUUCAGUGUUUAAC

>P1545

CUCGCCAUUGCAUUCCAGCUUGGGCAACAGAGUGAGAUUACAUCUCAAAAA

>P1546

AUUGAUUUUCUCUCUGUAGAAAAAAAGAUGAUCCCAGCCAGGCGUGGUGGC

>P1547

AAAAGAAAGCUGGAGCUGGUGGCUUAGGCCAUCACCCUUCCCUUGGCUGGA

>P1548

GAAACCCUACUAAAAAUACAAAGUUAGCCAGGCAUAGUGGUGCAUGCCUGU

>P1549

AGCCUGACCAACAUGGAGAAACCCUACUAAAAAUACAAAGUUAGCCAGGCA

>P1550

AGGCUGGGCGCAGUGGCUCACGCCUAUAAUCCCAGAGGCUGAGGCAGGCGG

>P1551

GUCCUUCGGCGGCUGUUGUGUCGGGAGCCUGAUCGCGAUGGGGACAAAGGC

>P1552

ACAGGAUUACAGGUUACACCACCAUACCCAGCUAAUUUUUUAUUAAAAAAA

>P1553

GUCUCAGCCUCCGGAGUACAGGAUUACAGGUUACACCACCAUACCCAGCUA

>P1554

AGGUUCGAAUUCCUGGGCACAAGUGAUUCUCUUGUCUCAGCCUCCGGAGUA

>P1555

AUCUUUCUUGUUCCUUUGCCCAGGCAGGAGUGCAGUGGUGCAAUCAUAGCU

>P1556

ACCCAGGCUGGAGUGUAGUGGUGCUAUCUCUGCUUACUGUAACCUCUGUCU

>P1557

GGGACACCGUUUCCCCAUUUUGGCCAGACUGGUCUCAAACUCCUGACCUCA

>P1558

GCCAGCCUUGGCCUCCCAAAGUGCUAGGAUUGGACACAUGAGCCACCAUGC

>P1559

UCUUGGGUAUGUCUUUAUCAGCAGCAUAAAAACGGACUAAUACAUUUACCA

>P1560

UUAAACCUCUUUUUCUUUAUAAAUUACCCAGUCUUGGGUAUGUCUUUAUCA

>P1561

UGUCAUGAGAUCUGACGGUUUUAUAAGGGGCUCUUCCCCCUUUGCUCGGCA

>P1562

AGCCAAGGGGGAAGAGCCCCUUAUAAAACCAUCAGAUCUCAUGAGAACUCA

>P1563

CAUGACAUAAGAUCAAUAAAGAGGCAAAAUUUUUAGCUGUUUUAUGUACAA

>P1564

UAUCCAGCUUAUGGGCAGCAGCCAGAAGCCACUGCACCUACAAGACUGCAG

>P1565

ACACAUCCAUGCACUGCACUGCACUACACCACACCCACCCACACUUAUACU

>P1566

GUAUGCUGGCUUUUAGACAGGUUAUACAGGUGAAUGUAGGGGUCUAUCCUU

>P1567

AAAGAAAGUUACUAUUGGUCUCAGAAUGUAAAAGUCUGGGAUUAGGUUGCU

>P1568

CUUGUUUUUUCCAUGUUGUAGACAAAUGAAAUUUGUCUAAAUGCUUUUGUC

>P1569

UAAGAAGUGUUUGGGGUAGAUUUUGAGGAGAGUGGGCAUCCUCUCUUUUGU

>P1570

UGGGCUCAGUGCCACAGUUAUACUCAGAGACCUGCUGCCACUUCCAGGCUG

>P1571

CCAAACUCAGCCUUCCAAGUAGCUGAGACCACAGGCAUAUUGCCACCAAGC

>P1572

CGAUUCUCUUCAGCCUCUUGUAGCUAGGACUAUAGGCACCUACCAUCACAA

>P1573

GGUUUUUUUAUUUUUUAUUUUUUGUAGAGGCGAGGUCUUGAUAUAUUGCCC

>P1574

UAUAAACAACCUGAAUACAAAAAUUAGCCGGGCAUGAUGGUGGGCGCCUGU

>P1575

CAAGCAAUUCUUGUCCCGAGUAGCUAGGAUUACAGGCUUGUACCACCACAU

>P1576

CAGCCUCCCAAGUAGCUGGGAUUACAGGCACGCACUACCACACCCUGUACC

>P1577

GCCAGGUGUGGUGGUAGGCGCCUAUAGUCUCAGCUACCUGGGAGGCUAUGG

>P1578

AUGUUACUCUGAAUGGUAAGACCCUAUCUUCCCUGGCAGCUUCCCAUUAGC

>P1579

CCCUCUAAAAAAAAGCUUUUGGGCUAGUGGGAAACUGUUGGGGAAGAAACC

>P1580

AUCCAUCAAGCUGGAGUAUUCACGUAUUCUCUAAUAGAAAGUCAACUGACU

>P1581

CUUAGGAAGCUGUCCUAUAUAUGUUAGCAAAUCCAUCAAGCUGGAGUAUUC

>P1582

AGCCACUAUGACUCCUAUACAGCCUACAGAACUAUGAGCCAAUUAAACCUC

>P1583

GCUCAUUGCAGCCUUGACCACCCAUAUUGGUCAGACUGGUCUCAAACUCCU

>P1584

AGGAAUAUAGAGAAAAGGGUUGGCCAGGUGCAGUGACUCACACCUGUAAUC

>P1585

CUGUACAAUGUAUGUGUUUUAAGCUAAGUGUUAUUACAAGAGUCAAAAAGC

>P1586

UACAGUGAGCUAUGAUGGCACUACUACACUCCAGUCUGGAUGACAGAGGUU

>P1587

AGCCAGGCCUGUGGUGUGCACCUGUAGUCCUAAUGAGGAGGGUGAGGUGGA

>P1588

CAUUGCUUUGAGAGGCAGAGACAGGAGGAUUGCUUGAGGCCAAGAGUUCAA

>P1589

UUUUAAAGUUCUACCAGCCAAGCGCAGUGGCUCAUCCCUGUAUUCUCAGCA

>P1590

ACUACAGGCACGCGCCACCACGCCUAGCUGAUUUUAUUCAUUUUUUGUAGA

>P1591

CGCAAGGCCACACAGCCAAGGAAGUAGUAAAGAGAAGGGUCAAAGAGCCAG

>P1592

GGUAUAGACUUGAACUAAGCUGCCUAGCUUUGAGUCUUGUCUUUAUCACUU

>P1593

UGUAUACAUUAUAAUGUAUAAUGGUAGUUAAGGGUAUAGACUUGAACUAAG

>P1594

UAUUUCUAGAUAAUUUUUUUCUUUUAAGAGAUAGGGUCUUACUCUGUCACC

>P1595

UCACCUGGAGUUGGCACAUCUCUGAAACUCCAAACACAUAACAAUAAUAGG

>P1596

CAGUGGUGAUAACCCCACACUGCUUAUAAGUGCAUCUUUAUAGUAUUUGGG

>P1597

CCAGUCAUGAUGGCUCAUGCCUGUAAUCCUAGGACUUUGGGAGGCCAAAGC

>P1598

AUCAGAAUCAACAGAUACACUUGUUAGAAGUGUUGGCUUCUGAGUCCUGCU

>P1599

GCAGGAGGAUUGUUUGAGCGAGGGAAGUUGAGGCUGCAGUGAACUAUGAUU

>P1600

CUAGACAAGCCUGGGAAACAUAGCAAGAUCUCAUCCUUACAAAAAAUAAUA

>P1601

GGUAGCUUACACCUAUAAUCCCAGCACUUUGGGAGGCUGAGGUGAGAGGGU

>P1602

GAGAUUCUCCUCUGUCAGCGUCCCAAGUAGCUGGAAUUACAGCCAUGCGGC

>P1603

UAGAGUUAAAAUAAAGCCAAAUUUUAUGAGCAACAAUGGCAAAUGUGUAAU

>P1604

UUUAAGCAUGUAUAUGUCAGAUAUUACACAUUUGCCAUAGUUGCUUAUAAA

>P1605

ACAGUACAUUUCAAUUAUUGUUAUUAAUUUAAUUCUUCUCUCUAAAAAAUC

>P1606

AUUCUAAGAUCUAAAAUUCUUAUUUAAAUUUAAACAGUACAUUUCAAUUAU

>P1607

GGUGGCUCUGAUCCAUAUGGUCAAAAAGGACUGAAAAAUUGUGAUGUAUUU

>P1608

AUUAAUAUUUGGCUUCUUGUUACUUAUGCAAAUUUUUUGCUGCUGGCUUAU

>P1609

UAUUUUCCCCAUUGUCUUGGAGAUUAAUAUUUGGCUUCUUGUUACUUAUGC

>P1610

GCUACCUUGUGAAGACCUCUGAAAUACCCUGGAGAUAUUUUCCCCAUUGUC

>P1611

CCCUGGGCCCAGCCCUAGGCCUCCUAGGCAUCCAGGCCUGUGAUGGGAGGG

>P1612

GAAACAAAAAUAGAUCACAGUGGCCAGAUGCAGUGGCUCACACCUGUAAUA

>P1613

UGGGACUUGAGCCCUGAAGAACAUCAAAAUUCACAGACAUUGACAAGAGAA

>P1614

UGGCGACAGAGCAAGACCCUGUCUCAAAAAAAAAAAAAAAAAAAAUGCUUU

>P1615

GGAAACACAGCUCACUGCAGCCUCAACCUCCCACACUCAAGUGAUCCACCC

>P1616

AAUAGUCAGUGUCAAGUAAUGAGACAGGGUCUUGCUCUGUUGCUUAGGUCA

>P1617

CCUGAGACGAAGGCUGAGCAGGGUGAGCCUCGAAAACCCCAUAGCACUGGC

>P1618

CCUACAGUGUAUUAGGCAAUGUUCUAGGUGCUGGAGCCACAGGGGUAAAAA

>P1619

CCUUAUAUUCUUAGAAAUUCUGGAUAUUGGCUGGGCAUGGUGGCUCACGCC

>P1620

UUAGGAGUAUCUUGAUAGUGGCUGGAAUUUGAACAACAUGCCAGUGAUGGA

>P1621

UGGUCUUGAGCUCCCAGGCUCAAGCAGUCCUCCCCCUUUUGGCCUCGCAAA

>P1622

GAGCCCAAAAGUUUGAGACCAACUUAGUCAACAUAGCAAGACUGACUCUAC

>P1623

CCUGCAGCUUCCACUUCUUGGGCUCAAGUGCUCCUCCCUCCUCAGCCUCUA

>P1624

CCCAUCUCUAUUUAAAAAAUGAACAAAAUUACCUGGGCAUAGUGGUGUAUA

>P1625

CUAAUCUGAAAAUCUUGAAAUUCAAAAUGCUCCAAGAUCUAAAACUUUGAG

>P1626

ACGUUCUCCAACCCAAGGAGGCCAGACAGAGGGACGUGGUCACUCUCUGAA

>P1627

AUGGCAAUUCCUCAAAGAAUUAAAAAUAGAAUUACCAUGUGAUCCAGUAUA

>P1628

GUAGUGUGAAUUUCCUAGGGCUGCCAGGAGUUAACAAAUUACCACAAACCA

>P1629

GAUCCUCCCGCGUAGCUAGGUCUACAGAUGUGCGCCACCAUGCCUGGCUAA

>P1630

GUGUACACCUGUAGUCCCAGUGCCCAGGAGGCUGAGGCAGGAGGAUCACUU

>P1631

CACCCCACCCCACUACUUUAUCCACAGGGAAGCAGCUGUAUCAAAUGGCCA

>P1632

CAUCCCUGACUUCACAGUCUCAGAAAGUGACUUCCAUCAGCAUAAGGACCA

>P1633

UUGUAUCUCAGCAUUGUAUGAUUCCAAAAAUGAGGGGUAAACUUGGUAUUU

>P1634

UGAAGAUGAUAUUGAUGAAGAUGGGAAAGAAUAUUUGGAGAUUCUGGCUAA

>P1635

ACACAACAGUUGGCAAUGACAUUUAACCAAGUAAUCCAGACAGGGCCAGAU

>P1636

CCUGCUGAACAAAUGUAUUUUUAAAAGGAAAAAAAUUCUCUUUUGGCUCUA

>P1637

UUGCUGCUUUUGUCCCCCGUGAGCUAAAAAGAUGGAGUGACAUCAAAUCAA

>P1638

AAAUUUAAUUGCAGUUUUUUUUCUUAGAAUUGACUGCUGUGAGAGUUCCAU

>P1639

CUUUUCAGCUUGGGAAAAAGGCGGCAGUGGAGGAAGGCAUGGAAUGCCCAC

>P1640

GGGAAGUCACAGGCGUGCUCUUUCUAGUUAAUUUGAUGCCACAUCUUCCUU

>P1641

UGUUGUUACGUGGUGAUGGGAAGUCACAGGCGUGCUCUUUCUAGUUAAUUU

>P1642

ACCUUUUCUUGAGUACAGCUUUGAUAUGCACCUGUUGUUACGUGGUGAUGG

>P1643

ACUACAGGCAUGAGCCACCGUCCCCAGCACAUGUUUGUUGAUGUCCACAAG

>P1644

UGAGACACACUCUUUCGCCCAGGCUAGAGUGCAGUGGUGCCACUUCGGCUC

>P1645

GUGCACGCCUGUGAUUCCAGCACUUAGGGAGGUCAGUGUGGGUGGAUUGCU

>P1646

UCUUUAUUUUGGGACACAGCCUGGUAAAACUUGUAAGAAAUUAUGACAGCU

>P1647

AAAAAAUUAUAUAAAUUGGCUGGACAUGGUGGCUCAUUCCUGUAAUUCUAG

>P1648

AGAAAUUGUCUGUGGAUCUAAAAAAAUUAUAUAAAUUGGCUGGACAUGGUG

>P1649

UAAUCUCAUCUGGAAACACCCUCACAGACAUAGACACAAUAAUAUUUGACC

>P1650

GAUUAGAAGAGGCUCACCCACAUUUAGGAGAUAAAUCUACUCAGUCUACCA

>P1651

UAUAUAUCUAUAUACACAUAUACAUAUAUAUCUAUAUGGGAUAUAGAUAUA

>P1652

AGCACUCCUGUUUCUUGGGCCCACAAACUCAGAUGAAGUCUUACCCCAUUA

>P1653

CGUAAGGGUGUUUCUGGAAGAGACUAGCAUUUGAAUCGGUAGACUGAGUAA

>P1654

AUGCCCAGAUAGCUGCUCAAUAAAUAUUUCUGGGUUUGUCCGUAAGGGUGU

>P1655

AUGGUUAAUUUUAUGUGUCAAUUUGACUGGGCUAAGAGAUGCCCAGAUAGC

>P1656

UGGUUGGCUGCUCGAAGAGGCCCACACCUGUUAAGCCACAGCACUUUGAGA

>P1657

UAAUUAAUUUUCUUUAUUUUCAGAAACAAUUUAGUGUUCAUGGUACGAAGU

>P1658

UGAGCAUGGGUGGGUCAUGUCUGUAAUCCCCACACUUCGGGAGGCCAAGGC

>P1659

GGAUUCGCCCACCUCAGCUUCCCAAAACGCUGUGAUUUUACAAGCGUGAGU

>P1660

AUCACCUCAGCCUCCGAGUGCGACUAUAGCCAUGUGCCACCAUGCCUAAUU

>P1661

CAAUAAUCUUGAACUCCUGAGCUCAAGCUAUGCUAUCACCUCAGCCUCCGA

>P1662

UGGAGUAUAGUGGCACACAUAGCUCACAAUAAUCUUGAACUCCUGAGCUCA

>P1663

AUGCCACAGCUCAAUGAGUAGUGACAGGGUCUUGCUUUGUUACCCCAGUUG

>P1664

GUUCAUAGACAGUUCACAGUAGCCAAGAGGUGGAAACAACCCAAAUGCCCA

>P1665

UUCCUGAAGUACUGGGAUUAUAGGCAUGAGCUACCAGGUCUGACUAAUUUC

>P1666

UUUAAUAGAGGCAGGGUUUUGCUAUAUUACCCAGGCUGGUCUCUAACUCUU

>P1667

AUGCCCAGCUAAUUUUUGAUUUUUAAUAGAGGCAGGGUUUUGCUAUAUUAC

>P1668

ACCUUCCCACCUCAGCCCUACUAGUAGCUGGGACUAGAGGUGCACACCAUC

>P1669

AUCACACCUGAUCCAACCAAUCUGCAGGCCCUACAUAAAUCAGACACCGCC

>P1670

GGAUCCUUUAUGAGCAUCAAGUUUUAAGUAGUCAGUGAUUUCUUAAAACAC

>P1671

UGGGUCAGAAGGAUUCCUAUGUGGGAGACGAGGCCCAGAGCAAGAGAGGCA

>P1672

AUUUUUGUAGUUUUAGGGUCUCGCCAUAUUGCCCAGUCUUGUCAUGAACUC

>P1673

UAUUAUGUAGAUGAAUAUGGUCUUUAUCUUUGAGACUCAGUCUCACUCUGU

>P1674

UUUACCAUUUUAGCCAUUUUUAAGUAUAUAUUUCUGUGGCAUUAAGUACAU

>P1675

GAGGAUCUCUUAAGGCUAGGAGUUCAAGAUUAGCCUGGGCAACAAAGCAAG

>P1676

AUUUUAAGUUUGUUUAUUUUAUUUUAUUAUUUAUUUAUUUAUUUUGAGACA

>P1677

AAGGCUGAGGUGAGAGGAUUGCCUGAACCUGGGAAGUCAAGACUGCAGUGA

>P1678

UAUAUUUACAUAAGCAUGUUUAUGUAAACAAAUGUUCACAUAAAUAUUUUG

>P1679

AAGUCACUUUUUUUGGCCGGGCAUGAUGGCUUACACCUGUAAUCUCAACAC

>P1680

CUCUUGACCCCAGGCGGUCAGGGCUACAGUGAUCUAUGAUUGUGCUACUGU

>P1681

UAGGCAUGCACUACCAUGCCUGGCUAAUUCUUAAGUUUUUUUGUAGAGAUG

>P1682

AUUUUUGACGGAGUGUUGCUCUGUCACUCAGGUUGGAGUGCAGUGGUGCGA

>P1683

AAGUUUUGAGAUCAGCCUGGGUAACAGGGAGACUCAUUUCUACAAAAAAAU

>P1684

AAAUUGAAAUAAUUUACCCUGGCGCAAUGGCUCAUACCUGUAUAAUCCCUG

>P1685

AGAGGCUGAGGUGGAAGAAUCACUUAAACCUGGAAGGUGGAGACUGUAGUA

>P1686

GAAGCUCACUUAGGUCGUUGCUUUUAGGCCUUUGUAUUAGGUUGGUGGAAA

>P1687

UAGAGGCCUGGGACAUUUUUGAGACAGUUUUGCUCUUAUUACCCAGGCUGG

>P1688

AAAAAUAGAGAUGGGGUCUUGCCAUAUUGCCCAGUCUGGUCUCUAACUCCU

>P1689

CAAGUAGCUGGUAUUACAGGCGCCUACCACCCCACCCAGCUAAUUCUUAUA

>P1690

GCUUGCUUUUUCUUGUUUUGAGACAAGGCCUGACUGUUACACAGGCUGAAA

>P1691

CAAAACAAAAUUUAUUUUUUAAAAAAGAACCUGGGGCUGGGCACGAUGGCU

>P1692

GGCUGGAGUGGCAUGAUCUUGGCUUACUGCAACCCCCACCUCCUGGGCUCA

>P1693

CACGGAUGUUACCUGCCCAAAACCAAACUCUCGACUUUUACCCUCAAAUCU

>P1694

CCAGGUGUGGUGGCUCAUGCCUGUAAUCCAGCAUGAGAUUACAUGCUGAGA

>P1695

UUUAAAAGAAUGCGUAUUUUAGGCCAGGUGUGGUGGCUCAUGCCUGUAAUC

>P1696

CCCGACUACCAGCAGCAGUGCCAUGAGAUGGCUGAGCGUCUGGAGGAUUCC

>P1697

CUCAGCUCCCUGAGUAGCUGGGAUUACAGAUGUGCUGUACCACCACACCCA

>P1698

CUUUGGAAGCCUAGGGCGAUUGAUUACUUGAAGCCAGGAGCUCGAGACCAA

>P1699

CAUCCAAAAGUACUGAAACUCGACCAGGCACAGUGGCUCACACCUGUAAUC

>P1700

GGCUAGAUUCUAACUUGUGGAUUCAAAUGAUCUUCCUGUCUCAGCCUCCCA

>P1701

AGAAUCUUAAUGGAUUGCCCAGGCUAGAUUCUAACUUGUGGAUUCAAAUGA

>P1702

AAAUUUUCUUUAAUUAGCCAGGUGUAGUGGUUGCAUGCCUGUUGUCCCAAC

>P1703

AAUUUAAGCAUCUUGUCAGUACUCAAAAAGUUCCAGAUUUGGAGGCAUUUC

>P1704

UUAAAAAAUAAAAAAAUAAAAAAUUAGCUGGAUACUAAUUUUUUUUAGCUG

>P1705

UCUUAAGGUGGACGUGGUGGUUCAUACCUAUAAUCCCAGCACUUUGGGAGU

>P1706

AGUCUGUGACUUUGCUAUUGGGAAUAGUGCUGUGAUAAACAUAUAAGUACA

>P1707

GAAAAGGUUUGCCAACCCCUGAAUUAGUCUCUUUUCUAAUACCAGACUUUU

>P1708

AACAUAGCCCCACCUAUUUGUUUACAUACUGUUUUUGUUUUGAUUAUGCAA

>P1709

UUAACAAAGUUAGAAUUUUGAUUUUAGAAUUUAGUGGAAAGCAAUGAUUAU

>P1710

UAUUCCUCAUAAUAAUAAUUCAAUUAAAAUACCUCUUACCCUGAUAAUUCU

>P1711

AGCUUACCAAGUAGCUGGAGCUACAAGCAUGUGCCACCAUGCCCAGCUAAU

>P1712

AAAUCCAAACCAUGUCAAGGGUCUUACUGUGUUACCUAGGCUGGUCUUGAA

>P1713

CAGCCUGGGCAACUGAGCGAGACCCAGUCUCAAACAAAACAAAACAAAAAA

>P1714

GAUUCCUCUCUGAAAAACGAGGAUAAGAUGAGGGUGAAAGGGAGGAUUUAA

>P1715

GGAAGAAGGAACCAAAAUCAUGAACACAACUGGCUCUCUCAGGGGGAGGCU

>P1716

UGUGUACACAUAUACUUAUGUAUGUAUACACAUACAUAUAUGUGUACACAU

>P1717

AUAAUUGGGCAGAUGAGGAUUUUUCACUUUUUGACUAGUUCUUUCUCUAGA

>P1718

UCUCAGGGGUGGAGGUUGCUUCCCGAGUUCGCUUCUGGGCUUGUCUAGAAU

>P1719

GCCAGAGGAUUGCUUGACUUCAGGAAGUCGAGACUGCAGUGGGCUGUGAUU

>P1720

GUAAUCCGAGCACUUUGGGAUGCCAAGGCCAGAGGAUUGCUUGACUUCAGG

>P1721

GCCCAGGCUGGAGUGCGAUGGUGCUAUCAUGCCUCAUUGUAGCCUCAACCU

>P1722

GCAGGCUAAGGAGAGUGGAUUGCUUAAGCCCAGGAGUUCGAGACCAGCCUG

>P1723

AAUACCUGUUCAAUUUAUUUCUUUUAGGUUUUCCGGUUUUAAUUAAACAAC

>P1724

UGCUUAUAUCAUGAGGUAAUUCAUUAGCACAAAAAAUUGUAAAGAUAGGCA

>P1725

UAUAGCUCUGAUCUGCCACAUCCUAAUGGAAAACUUUUGAUGGCCCCCUAU

>P1726

AUGUGGCUACCUUUCUUCUACUAGAAGAAUCUUCCUAAAAUAUAGCUCUGA

>P1727

UGGCAGCUACAAGUUGACUGAUGUAACCAUCAGUCUCUAUCCUCAUCAGCC

>P1728

GCCCUUGAUUCAGGAGGCAGUGGGGAGCCAUUGAAGGUUUUCCAUUAGGAU

>P1729

UUCAGCCUCCCAAGUAGCUGAGAUUAUAGGAGUGUGCCACCACACAUGGCU

>P1730

CUAAAGAAAACUGGGUAAAAUACAAAGAAAGGGGCUAGGCGCAGUGGCUCA

>P1731

AGACAGGGUCUUGCUCUGUUGCCCAAGGUGGUCUCAAACUCCUGGCCUCAA

>P1732

AUAGUUAAAUGCCUAGUAAAUAAUGAAUGGAAAUCCAAAUGAACAGAAAUG

>P1733

UAAUGACAGGAUGUAGGCCGGGUGCAGUGGUUGAUGCCUGUAACCCAGCAC

>P1734

CAAUUUCUCUUGAGUAGCUGGGACUACACCUGGCUAUUUUUGUAUUUUUUG

>P1735

GGCUAGUCUUAAACUCCUGGAUUCAAGUGACCUUCCCACCUUGGCUUCCCA

>P1736

GAGGUCUCCCUAUGCUGCCCAGGCUAGUCUUAAACUCCUGGAUUCAAGUGA

>P1737

UUUUUUUCCCCGGAGACAGAGGCUUACUCCGUUACCCAGGUUAGAGUGCAG

>P1738

AUUAAACCUUCAGAUAAUUUCAAAAACACAGUUUAGGCCAGGCACAGUGGC

>P1739

GCUGGCAUUACAGACAUGUGCCACCAUGCCUGGCUGUUUCUCGUUUUUUUU

>P1740

UCCCAAGUAUUAAAUAGCUGGCAUUACAGACAUGUGCCACCAUGCCUGGCU

>P1741

AUAUCUGUCCACCGCAGCCUUCCAAAGUGCUGUGAUAAUAGGCGUGAGCCA

>P1742

GCCUAGCUCUGUCACUUAGACUGGAAUUUAGCGGUGUGAUCAUAAUUCACU

>P1743

GAGGCUCCCAGAAACUUGGGAGGCAAGAGGAUCUCUUGAGCCCAGGAUUUA

>P1744

CAUAGUCAUGGCUCACUAUAACCUCAAACUCCGGGGCUCAAGCGAUCCUCC

>P1745

UGCUCAGCUUCCUGCAUGCCAAAACAUGAUUGCUGGACACCUUUCCUUUGC

>P1746

GUAACCUCAGCCUCCAGGGUUCUAGAGGUUGUCAUGUCUCAGCCACCUGAG

>P1747

GAGUUCAAGACCAGCCUGGGAGUUCAAGACCAGCCUGGGUAACAUAGUGAG

>P1748

CAGCAAGACUCCCUCUCAAAAAAAAAAAAAAAAAAAAAAAAAAAAAUGUUG

>P1749

GUGGUAUGAGUUUGUAGUCCCAGCUACUUGGGAGGCGGAGGCAGGAGAAUC

>P1750

AUGGCACAUGUAUACCUAUGCAACAAACCUGCAUGUUGCGCACAUGUACCC

>P1751

AGGAGAAAUACCUAAUGUAGGUGACAGGUUGAUGGAUGCAGCAAACCAACA

>P1752

GGGGAGCUGGGGGAGGGAUAGCAUUAGGAGAAAUACCUAAUGUAGGUGACA

>P1753

AUGGACACAGGGAGGGGACCAUCACACACUGGGGCCUGUUGCGGGGUGGGG

>P1754

UUGAACAAUGAGAACACAUGGACACAGGGAGGGGACCAUCACACACUGGGG

>P1755

CAGCAAACUAUCACAAGGACAGAAAACCAAACACCGCAUGUUUUCACUCAU

>P1756

UCCAUCAAUAAUAGACUGGAUAAAGAAAAUGUGGCACAUAUAUACCAUAGA

>P1757

ACCAUGUUGGCCGGGCCAGUCUCGAACUCCUGACGUCAAGUGAUCUUCCCG

>P1758

AUCCCUGCACUCUAGCCUGGGUGACAGAGCCAGAUUUUGUCUCUAAAAAAC

>P1759

AGUUCGAGACUAGUCUGCACAACAUAGUGAGAUCCCAUCUCUAAAUAACAA

>P1760

ACUAGGACUGGGCACAGUAGCUCACAACUAUAAUCUCAGUACUUUUGAAGG

>P1761

AGUAGAAAUGGGUUUUCACCAUGUUAGCCAGGCUGAUGGACGCUACCUUAU

>P1762

UGGUUGGCCAAACACAGUGGCUUACACCUGUAAUUCAGCACUUUGGAGGCU

>P1763

UAUAUGUAUACGUAUACAUAUAUGUAGAGUAUAUAUACAUCUACGUAUAUG

>P1764

UGCCAACAUGCUGGGUUAAUUUUUUAAGUUUUUCUAGAGUUGAGGUCUUGC

>P1765

CCUGGUACUCCAGCCUGGUCAACAGAGAAAGACCCUGUCUCGAAAAAUAAU

>P1766

UUCAUCACUAAUGGGAAGAGCUAAAAGCUCCAGUCCAUCAGGAUUGAAUGG

>P1767

UUUGCUCCUCCAGUUAGGUGCUAGUAUCUUAAGCUGGCCAUUUCAUCACUA

>P1768

AAUAUAAGUGGGUAAGAGAAAGGGCAGCAACUUCCAUUAUUUUGCUCCUCC

>P1769

ACAGGAAUGGGAAAGCAGAAGUGUAAAAUAUAAGUGGGUAAGAGAAAGGGC

>P1770

UACUACAUUUUGAGCUGGACACGGUAUCACACACCUGUAAUCCCAGACACU

>P1771

UUUUUUUUGCGGGGGACAGAGUCUCACUGUGUCGUCAGGCUGGAGUGCAGU

>P1772

UCAGCGAAAAACUGAAGUUAAAAUAAGUGAACAAGUAUUUUCAAGGUGACA

>P1773

AAUGAAAUAUAUGACUAGAUAUCUAAUAUUUUUCUGAUAUACACCUCUUUC

>P1774

ACACCCACCUCAAAAGUGGAAUAUAAAGCUACGCAGCCCUCAGUCCUCCUU

>P1775

UUUGUGUAGGGGUGAGAUCUCACUUAUGUUGCCCUGGAUGGUCUCGAACUC

>P1776

GGCCUCCCAAAGUGCUGGGAUUAUAAGUCUGAGCCACCACACCCAGCCUCC

>P1777

GGAGUCUAGCUCUGUCACCCAGGUUAGAGAACAGUGGCGCGAUCUUGGCUU

>P1778

CUUGUGAGGUUUCUGUAGCUUACUUAAUCCGCUGUCUUCUUGAUGUGUGUC

>P1779

UAUUAUAUGGAUAUACCAUCAUUUUAAAUUGAAAUUUUAAAAUGAAAAUUU

>P1780

UGAAUGCCUAGAGUCCCAGCUACUUAGGGGGCUGAGGCCCAAAAAUCAUUU

>P1781

CAAAAAAAAAGUAGCUGGUCAUGGUAGUGAAUGCCUAGAGUCCCAGCUACU

>P1782

AAUGUUCUCGUCACAAAAAUAUAUGAGGUAUAGAUUUGUUAAUUAGCUUAA

>P1783

ACUGUAAUGGGAGCUGCAGGUGGAAAUGGGGAGAGGCCAGGCAUGGUGGCU

>P1784

GAGGAUCACUUGAGUCCAGAAGGCCAAGUCUGCAGUGAGCUAUGAUUGCAC

>P1785

AUUGCACCACUGCCUUCCAGCCUGGAUGACAGUCAAAAAAAAAGAGAGCGC

>P1786

UUAAUUCUUGUUUCUCAGGACCCACAAUUUGCCAGGUUGCUUCAUAAAUAU

>P1787

UGUAGUGUGGGAAGUGUAUUAGUCCAUUUUCAUGCUGCUGAUUAAGACAUC

>P1788

UAAGACAUCUGAGACUGGGAAGAAAAAGAGGCUUAAUUGGACUUACAGUUC

>P1789

UCCAUCCAUCCAUCCAUCCAUCCAUACAUCAUGCACACAUCCAUCUACCCA

>P1790

UUUUUAUGGCUCUUUUCAGUUUAUUACAUGAAGGAGUUACACUAGUCCAAG

>P1791

UGUGUGUGUGGCUGUGUUGGAUAGAAGUGUUUUUAAAUAGUGUAUGUAUUU

>P1792

UGGAUCACCUGGGAUCAGGAGUCCAAGAUCAGCCUGGCCAACAUGUCGAAG

>P1793

CUGGGCUUGGUGGUGUGCACUUGUAAUCCCAGCUACAUGGGAAGCGGAGGU

>P1794

AGCCUGCGGGACAGAGUGAGACUCCAUAUCAAAAAAAAAAAAAAAGAUAUA

>P1795

GAUAGUUUGUGGGGCCAGACACAGUAGCUCAUGUCUGUAAUCCCAGCACUG

>P1796

UCCACUGUAACCUAACUAGGCAUGUAAACAGUCUGUAACCUACUGUUGUAC

>P1797

UAAACAGUCUGUAACCUACUGUUGUACCAAUCACUGUGUUUUGGCUGCUCA

>P1798

AGCUGUAACCAAUCCAGCUGUGGCUAUACCUCACUUCUGUUUUCUGUAUGU

>P1799

UUAACUAUAUCCCACAAUGUUAUGAAAUGUUUUAUUUUCAUUUUUAUUCAU

>P1800

AGCUACCCAGUCUAUAGUUUUUGCCACCCAGUCUGUGGUUUCGUUACGGCA

>P1801

ACAUGCAAAUAGAGUUAUUUCAGUCAUGACCCUGGCUCAUAAGUAAAGAAA

>P1802

UUUUUCUACACUUUACUAAUUUUGGAUUCUUUUCAAAAUUUCUAUAAUAAC

>P1803

CUAGAAGGCUGUUCUUCAGAGCAUGAUUGUCUGCUACACUGUCUCUGUCAU

>P1804

GAUAGAGUGAGACUCCAUCUCAAAAAAGAAAAAAAAAAAUCACUUAUGUGG

>P1805

AGGCACAUGCCACCUUAGUAGAGAUAGGGUUUCACUAUGUUGCUUAGGCUA

>P1806

UCCUACACUGUAGCCUUGAACUCCUAGACUCAAGGAAUUCUCUCUCUUGCC

>P1807

UGAGUUAUGUUUCAUCAAUCUGACCAAAAAAGCAAUGGGGAAAGGAUUCCC

>P1808

AAUCUGACCAAAAAAGCAAUGGGGAAAGGAUUCCCUAUUUAAUAAAUGCUG

>P1809

AUGGGGAAAGGAUUCCCUAUUUAAUAAAUGCUGUUGGGAAAACUGGCUAUC

>P1810

GCUGUUGGGAAAACUGGCUAUCCAUAUGCAGAUAACUGAAACUGGACCCUU

>P1811

GGACCCUUUCCUUACACCUUAUACAAAAAUUAACUUAAGAUGAAUUAAAGA

>P1812

UUAAAGAUGUAAACGUUAAGACCUAAGACCAUAAAAACCCUAGAAGAAAAC

>P1813

UAAGACCUAAGACCAUAAAAACCCUAGAAGAAAACCUAGGCAGUACCAUUC

>P1814

GAAGAAAACCUAGGCAGUACCAUUCAGGACAUAGGCAUGGGCAAAGACUUC

>P1815

CCAUUCAGGACAUAGGCAUGGGCAAAGACUUCAGGAGGAAAACACCAAAAG

>P1816

CAUGGGCAAAGACUUCAGGAGGAAAACACCAAAAGCAAUGGCAACAAAAGC

>P1817

GAAAACACCAAAAGCAAUGGCAACAAAAGCCAAAAUUGACAAAUGGGAUCU

>P1818

AAGCCAAAAUUGACAAAUGGGAUCUAAUUAAGCUAAAGAGCUUCAGCACAA

>P1819

AAUUAAGCUAAAGAGCUUCAGCACAACAAAAGAAACUACCAUUAGAGUGAA

>P1820

UCUCAUGAGACUUAUUCACUAUCACAAGAAAAGCACAGGAAAGACCUGCCC

>P1821

GUGGGAAUUGUGAGAGCUACAAUUCAAGAUGACAUUUGGGUGGAGACACAG

>P1822

CCCAAAGUGAGGACCUGAUAUGGUUAGGCUGUGUCCCCACCCAAAAUCUCA

>P1823

UGUAGGUUGCAGUGAGCUGAGAUUGAGCAACUGUACUCCAGCUUGGCGACA

>P1824

UGGGGCAUACAAUUUGACCUAAGCAAUUGUUUCAAAACUGUCUUCAGUUUC

>P1825

CAAUACAUCUAAAUACACUUAUUUAACGUUCUUAUUACAAAGAAUGCUAAA

>P1826

ACGAACAAUAAUAGCCAGGUGUGGUAGCAUACACCUAUAGUCCCAGCUGCU

>P1827

CAGGUGCCUGCCACCAUGCCCAGCUAAUUUUUAAACGUUUUGUAGAGACGC

>P1828

CAUUGCCAGGGAACAUCAAGUAAAGAGACAAAUGCUCAGGUAAGCUUUUAA

>P1829

UUAAAACCAUUCCUCUUACGUUGUCACCCUCAAUCUUACAGGUAAAUGGCA

>P1830

UAGGUUAGCUGUUAUUAAAAAUGCAAGAGAUAAUAAGUGUUGGUAAGGGUG

>P1831

AAGGGAACCUUUGUACACUGUUGGUAGGAAUGUAGAUUAGUGCAGCCAUUA

>P1832

AGGAAUGUAGAUUAGUGCAGCCAUUAUGUAAAACAGUAUGGAGGUUCCUAA

>P1833

ACAAUUCACAAUUGGAGAGAUAUGAAACCAACCAAAUUGCCCAUCAGCCAA

>P1834

GGAUAUGUAGGUCAAAGGAUACAAAAUAGCUGAUGUAUAGGAUGAACAAGU

>P1835

UGCAAUGGCAUGAUUGCCGCUCGCUACAACCUCUGCCUCUCAAGUUCAAGC

>P1836

CGCUCGCUACAACCUCUGCCUCUCAAGUUCAAGCAAUUCUCGUGCCUCAGC

>P1837

CGCAGGGAAAUUCAGUCUCUAGUUCAUAUGGAAACUUGGGACCUCACUGUG

>P1838

AGUAGUCGAACCUCUCUGGGCCUCAAUUUCCCAGAUGUAAAAUGGGAAUAG

>P1839

CUGUUUGGUGAUACAGGUUGGGAGCAUGGGUGGGGAAGCCUACCCCAGGCC

>P1840

GGCUUUGUGGCCCUGCUGGGGCCAAAGGCAGUUUUCUGUGCUCCCUUUAGC

>P1841

UUCAGCUGCCCAAGCAGCUGGGACUACAGGUGUGUGCCAUUAUGCCUAGCU

>P1842

GUCGGAUAUGGCGGUGAGUACCUGUAGUCCCAGCUAUUUGGUAGGCUGAGG

>P1843

UCCCAGCCUAGCCUCCCAGGUACCUAGGACCACAAGCGUGCUAUAUCAUGU

>P1844

CUGGAGGUUGAGGCUAUAGUUAGCCAAGAUUACACCACUGCACUCCACUCC

>P1845

CUUGGCCUUCCAAAAUUUUAGGAUUACAGGGGUGAGCCAGUGUACCCAGCC

>P1846

CCGGUAGAAAGAAGCCCCUUAGGCCAGACGCAGUGGCUUACACCUGUAAUC

>P1847

CCUGCCACAGCCCCCUACAGCUUAGAGACCCUGAAGGGCGGCACUAUCCUU

>P1848

GGCCUUGAAUUCCUAGGCUCAAGCAAUCCUCCUGCCUCUUCUGAGUAGCUA

>P1849

GCCUGGUGCACUAGUUCAUGCUUGUAAUUCCAACAGUUUGGGAGGCCAAGG

>P1850

UUUAGUUCAGUAUAGGCAAUCAACCAGUAAUCUAGACAGCUGUGUGGAUGC

>P1851

GACAGCUGUGUGGAUGCUGCCUCCCAUGACACACCCAGACAAAAGAACACA

>P1852

CUGCCUCCCAUGACACACCCAGACAAAAGAACACAGGGCUAGGUGUGGAGU

>P1853

AAUGUCAUUAAUGCAACUCACUGCUAAAAACCAAUAAAUUAACUCAUCUGC

>P1854

GUGUUCUCCUGUGUGAGUGUGUUACAGGAAGCAGCUUCCUGCCAGCUGUCG

>P1855

CUGGAGCUGGGAGCUGUCAGAGCUGAGCCUGGGAAGGAAAAGUCAGAUAAC

>P1856

CCAAAAUAGCAUGGUACUGUUACAAAAACAGACACAUAGACCAAUGGAACA

>P1857

ACAGACAUUCUACAGAAAGGGAGAAAAUUUUUGCAAUCUAUCCAUCUGACA

>P1858

UUUUUUUUUUUUUUACUUUUAGUGAAAACAGGGUUUCACCAUGUUGGCCAG

>P1859

AACUCCUCUGGGGGUACAUUAUGGAACUGGAAGCACCCUUGGAGGAGUCCG

>P1860

GCUGGAGUGACAGAAGGAAGUCAAGACACCAUUAUGACUCAGAUGAGAAAU

>P1861

GCUUCUGUGGAACAUUUGGCCAUCCAGUGUCAUUGGUCUCAGAGGCCAGCA

>P1862

ACAAAAAGUAGGAAUUACUUUUAAAAGUGUAGGUAUUCCUUCUACAAUGGA

>P1863

CCCAGCACUUUAGGCUGAGGUGGGCAGUUGGCUUAAGCUCGAGAGUUUGAU

>P1864

CGCACCUGCAAUCCCAAUCCCAGCUACCUAGGAGGCUGAGGCAGGAGAAUC

>P1865

UUCCAGGAAUUCCCCAAAUACUCAUACAGCCAUUGAGCACCCCAAUCCAAA

>P1866

AUUUUGCAUUUCAGAUUUUUGGAUUAGGAAAACUCAACCACUGUAUUUCUG

>P1867

AGGACUGUUUUGUGGCCAGGAGUUCAAAAUCAGCCUGGUCGACGUAGUGAG

>P1868

UGAGCCACUGUGUCAUUAUGGCAUUAAUUACCUCUUGAUAUACUAUAGUAA

>P1869

AAAUACUCAUUUGCUUUUUUUUUUGAGGCAGGGUCUCACUCUGUUGCCCAG

>P1870

CUAAAUGACAGAUACCUGUAUUUUUAAAAGUACUAUGCAGAGAAAAUAAAU

>P1871

UUUAAAAGUACUAUGCAGAGAAAAUAAAUUAAGCUUAAUUUCCAUCUUAUA

>P1872

UGGCUCACUGAAACUUUCGGGCUCAAGAAGUCCUGCCUCAGCCUCCCAAGU

>P1873

AAUCAUCCUGAUGUUUGAAGUGAAUAGUGAGAUAACUCUGUGAUUAGCUCA

>P1874

UAUCUUAUUUUUAAUUUUAUUUGACAGGGUCUUGCUCCGUCGCCCAGGCUA

>P1875

CACGUUGGUAUUGGCACCAAGUACUAAACAGAAGUCAAUAACCAGCUCAGA

>P1876

UCUUAUCUUGAAUUGUAGUUCCCAUAAUCCCCAUGUGUCAUGGGAGGGACC

>P1877

AUGAGGGUAACUGCCCCCAUGAUUCAAUUAUCUCCUGCUGGGUCCCUCCCA

>P1878

UAGGGCAAACUGUGGUAGGAAAAACAUACGGCCUUUGGAAAUGUGUGCUUC

>P1879

GGAUUCAGAUUGCAUCUAUUCUUCAACUCCUGAUGUUAAGGCCUGAGGGUC

>P1880

AAAUGAGGUACAAAUACACCAUGGAAUACUAUGCAGCCAUAAAAAAGAAUG

>P1881

CCAAUGGAACAGAACAGAGCCCUCAAAAAUAAAGCCACAUAUCUACAACUA

>P1882

CAAUCUAUCCAUCUGACAAAGGGCUAAUAUCCAGAAUCUACAAUGAACUCA

>P1883

AAUAUCCAGAAUCUACAAUGAACUCAAACAAAUUUAUCAGAAAAAAACAAA

>P1884

UGAAGGACAUGAACAGCCACUUCUCAAAAGAAGAUAUUUAUGUAGCCAAAA

>P1885

AGACACAUGCACACGUAUGUUUAUUACGGCACUAUUCACAAUAGCAAAGAC

>P1886

ACCUAAUGUUAAAUGAUGAAUUAAUAGGUGCAGCACACCAACAUGGCACAU

>P1887

CAAAGUGCCAGUUUUCCUGCUUAUUAGCAUUUAAACUCAAGUAUUCACAUA

>P1888

AGAUAUUUACCUUACCUGAGUAGAUAACAAAAUUCGGUUAGCUUCCUGGAA

>P1889

CAAGUUGGGAGGAUUGCUUGAGGGUAGGAGUUCGAGAUCAGCCUGGCCAAC

>P1890

GAGGAUUACAUGAACCUUGUAGUUAAAGACCAGCCUGGGCAACAUUGGGAC

>P1891

ACCCUGUCUCUAUAAAUAAUAAAAAAAUUAGCAUGGCAUGGCAGCGUAUGC

>P1892

AAUAAAAAAAUUAGCAUGGCAUGGCAGCGUAUGCCUGUAUUCCCAGAAGUA

>P1893

AGCGUAUGCCUGUAUUCCCAGAAGUAUUUGGGAAGUUGAGGUAGGAGGAUU

>P1894

CCAGAAGUAUUUGGGAAGUUGAGGUAGGAGGAUUGCUUGAGCCCAGGAGUU

>P1895

AGCCAUGAUUGUGACACUGUACUUUAGCCUGGGUGACAGAGCAAGACUCUG

>P1896

UGCUCUGUCACCGUGGUUUAAGUGCAGUGGUGCAGUCAUGGCUCACUGCAG

>P1897

CUUCCCCUCCCUGCUCCCCAAAAGAAAGGGUCUCCCUCUGUUGCCCAGGCU

>P1898

AUUUUAUGGUAGUCAUUAACAAUACACAUUCAAAGGACAAACCAACAGAGA

>P1899

CCAAAGUGCUGGGAUUACAGGCAUGAGCCAGCACAGCCAGCCUAAUAAUUU

>P1900

GAGCUACAAAAAAGUUUUAAAGAUUAGCUGAGCAUGGUGGUGCACACCUGU

>P1901

GAGACAGGGUCUCAUUCUUUGUCCCAGGCUGGAGUGCUAUGGCACCAUCAU

>P1902

AUGAAUGAAUGAAUGAAUGAAUGACAGGGUCUUCCUCCAUUGCCCAGGCUG

>P1903

UUUUGUUUGUUUGUUUUUGUUUUUGAGACAGAGUCUGGCUCUGUCGCCUAG

>P1904

UUAGUUGUUUUUUAAUUGGUCACGCAAAAGAAAGGCUUACAUUGAUUUUGA

>P1905

UUAGAUCUGGGAAGAGAUUUAGCACAUUUCUGUAUUAGUAGAGCAUUUGAG

>P1906

AGACCCUCUGUCAAGAGGCUCUGUUAGGAGCCAGCCUGCUGCCCUGCCUUU

>P1907

CCCGGGUUCGGGUUCAAGAGAUCCUAGUGUGUCAGCCUCCCAAGUAGCUGG

>P1908

ACGGCAGGCGGAGUGUUUUGGGCCUAUAAUCCCAGCACUUUGGGAGGCUGA

>P1909

UACAGUUUUGGUGCAUAUUUACUUUAGGAUGUUACUGGAGCUCCCAUCUUC

>P1910

UCUGGAGAUUUGGUUCUUCCCAGAUAUGCUGCCUCAGUUUAUAGAUGGAGC

>P1911

UAAACAAAAAUGUACCUUCACAAAAACUUGUACAUGAAUAUUCAUAGCAGC

>P1912

AAGUUGAUUGUGGUGAUGAUUGCACAGCUCUGUAAAUAUACUAAAAAGCAU

>P1913

AUUGCACAGCUCUGUAAAUAUACUAAAAAGCAUUGAAUUGUACACUUUAAA

>P1914

GCCUGGCCAAGAGUGUCUUUAUUUUAAAUGCUAAUCCACCAUGUAACUUCU

>P1915

CUAAUCCACCAUGUAACUUCUGACUAACCCUGAGUCUGGGAAUGCCUCCAA

>P1916

UUGAAAUAUUCUAAGAUGUAACUUUAUUUAUUUAUUUAUUUAGAGACAGAG

>P1917

AGGAUGUGGUGUUAUGAUAUAUACUAGUUUUUAUCCAUGGUUCCUGGCUCA

>P1918

UUUUUUCUUUUUUAGUCAGGGUUUCACUCUCUUACAGGCUGGAGUACAAUG

>P1919

AAUUUCUUGUAGAGAUAGAGUCUCAAUGUGUUGCCCGGGCAGGUCUUGAAC

>P1920

UUUUUUUUCACAGACAAGGAUUCCCAGGCUCAAGCAAUCUUCCUGCCUCGG

>P1921

UUGGCCAGGUUCAAGUGAUUCUCCCACCUCAGACUCCCAAGUAGCUGGGAC

>P1922

GGUUCAGUAAAGGUCAAUAGUUUUCAUUUUCUGGUAAGAUUUCAUCUUCAA

>P1923

AAGGAUUGUCAAUGUCACCAGAAUCAGAAGCACCGUUUGUCAUGAGGCUGC

>P1924

CUUAAGUAGAUAGAAACUUUUAAAAACAGGAGGCCGGGCAUGGUGGCUCAC

>P1925

UCCACAGAGGAGGAGCAUGGUUGGUAGCUGUGGUGGCAUCGUCAGCUGUGC

>P1926

CCAGACUCCGUCUCAAAAAAAAAAAAAAAAAGAAAGCUUAGGGACACACUA

>P1927

CAUUGUACUAUUAAAUUUACAGGCCAGGUACAGUGGUUCACGCCUGUUAAU

>P1928

GGUGGCUCAGGCAUGAGAAUCAUUUAAAUCCAGGAGCCGGAGGUUGCAGUG

>P1929

AUAGUCUCUCUCUGCUUCCCAGGCUAGAGUGCAAUGGCGCUAUCUCGACUC

>P1930

UGCACAACUGUGGUCUUAGCUACUCAGGAGGCCGAGGUAGGAAAAUUGCUU

>P1931

CUCACGCUUGUAAUCCCAGCACUUCAGGAGGUCAAGGCCUGAGGAUGGUUU

>P1932

AAAAAUGUAUAUGUAUUAUAUAUAUAAUAUAUAAAAAUAUAUAUGUAUAUA

>P1933

UGGCUUAGGGAAUUUUUUAUUUUUUAGAGACAGGGUCACACUCUGUCGCCC

>P1934

UGAUCAUAGCUUAAGUGAUCCUUCUACCUUGGCCUCCUGAGUAGCUGGGAC

>P1935

AAGCGUUUUGUAAAGAUGAGAUCUUACUAUAUUGCCCAGGCUGGUCUUGAG

>P1936

CCUGUGCUUUUUUUUUUUUUCCCAUAGCAUUUAUCACAAGUUAUUUCUGGA

>P1937

CUUUAGACCUAAUUUCUAGUUUACAAGAAAUGCAGGGAUAGAAGAACAUAU

>P1938

CUAUUUUAUUUUAUUUUAAUUUUUGAAACAGAGUCUUACUCUGCCAUCCCA

>P1939

AAAUGUUUAAUAUCUGUACUGUCCAAUAUGGUAGACACUAGCCACAUGUGA

>P1940

GCUGGGUGUAGUGGCAUGUGCUUGUAGUACCAGCUACUUGGGAGGCUACAG

>P1941

GCAAGAUGUGCAGUUUUGUUACAUAAGUAAACUUGCGUCAUGGGGGUUUGU

>P1942

AGCUAAUGCAUGCUGGGCUUUACCUAGGUAGUUAGAUGAUCUGUGCAACAA

>P1943

CUUAUAUAACAAACCUGCACAUCCUACACAUGUGCCCCUGAACUUAAAAUA

>P1944

AAGAUCUGCCCACUGCGGCCUCCUAAAGUGCUAGGAUUACAGGCUUGAGCC

>P1945

UGAGCCACUGCACCCUGCUGUCAGAAGCUAAUGUUAUGUUACUAUUCCAUU

>P1946

CCCACUUAUGAACUUCUCCUUUAGGAGGCAGGACUCAGAAUAACAAUAGUA

>P1947

AGGAAUGCAUGUGCCAGGGAGAAGAAGGACAGGGAGGGUUUUUUGAUUGUU

>P1948

GUUAUUAACUUUCUGUGGAUCGAUCACAUCUGUCUAGUUCUGUCUAUAAGA

>P1949

UGACAUACAUAUACAUAAAUCCAUUAUACCAGGAGUGUUUUGUCAUCUUAU

>P1950

CAAAGAUUAUUUAUUUUUAUUUUUUAGAGAUGGGGGUCUUGGUAUGUUACC

>P1951

CACUUUGGGAGGUGGAGGCAGGCUGACCACUUGAGCCCAAGAGUUCAGGCC

>P1952

GGAGGGUGAGGCAGACGAUCGCUUGAGCCUAGGCAUUCAAGGCCAGCCUAG

>P1953

GAGCCUAGGCAUUCAAGGCCAGCCUAGGCAAUGCAGCGAGGCCUUGUCUAG

>P1954

GGAAUAGGAGGGGACCAGGAAGAGAAAUCACUUGAUGAGCAGUUGUCAUAG

>P1955

UGGAGAGGUAGCACUGAGCUGAGAUAGCACCAUGGCACCCCAGCCUGGGCA

>P1956

UUAAAAUAUGGAAUGCCUAACGAAUAUGCGUGUCAUCCUUGCGCAGGGGCC

>P1957

GAAAGAAGAGAGCUGUGGACACAAAAAAGACAAAACUUGAAUCCUUGUCCC

>P1958

GUAAAAGAUAAUUUCACUGCAUGUGAUAAGGACAAUGAUGGAGAAAUGCAU

>P1959

GAGAUGCAGUUUCGCUCUGUUGCCAAGACUGGAAUGCAAUGAUGUGAUCUC

>P1960

GACUGGAAUGCAAUGAUGUGAUCUCAGCACACCACAACCUCCACCUCCCGG

>P1961

CUCCCGAAUAGCUGGAAUUACAUGCACCUGCCUCUGCACCCGGCUAAUUUU

>P1962

UUUGGAAAAAUUAGAAAAUAGAAAUAGGGCUGGGUGUGGUGGCUCAUGCCA

>P1963

UUAUCCAAAAAUGUAGGCCAGGCACAGUGGCUCUUACACCUGUAAUACUAG

>P1964

CUAUUUGAAAAUAUACAAUAAACCAAGCACAGUGGCACACUCCUAUAAUCU

>P1965

ACCAUUUGAAAGUAAGUAGCAGGCCAGGCGCCGUGGUGGUGGCUCACGCCU

>P1966

GCCAGGCAUGGUGGCGUGUGUCUGUAGUCCCGGGUACUCAAGAAGGCUGAA

>P1967

UGUAGAAAUAAAAUCUAACAUUUUAACCAUUUUUCAGUGUACAGGUUGGUG

>P1968

UUUCAGUGUACAGGUUGGUGGCAUUAAGUACAUUCACGUUGUUGUACAAGC

>P1969

ACAAGCAUCAACCUAGUCUAUUUCCAGAACUUUUUCAACAUCCUGUACUGA

>P1970

UAGUUUAGUUUAGUUUAGUUUUUUGAGAUGCAGUCUUUCUCUGUCGCCCAG

>P1971

GUGACCAGUGAAUGACACCGCAUAUAUACCUGGCUUAAAGUUACUGACUCG

>P1972

AACUCAAAGUUCUAAGCUUUUUUCUAGUGCUUGUAUAUAUUUAAAGCUCCG

>P1973

UUGUAUAUAUUUAAAGCUCCGUGCUACGUGUGGGAUUGUAACUGCAAGUGG

>P1974

UGCUACGUGUGGGAUUGUAACUGCAAGUGGGAGUGUUGCAUUCAAUCUCUA

>P1975

UUCAAUCUCUAUCUCAUCUUUAGCUAGGGACUAGGGUCUGGAAAGCUUUCU

>P1976

CUUUAGCUAGGGACUAGGGUCUGGAAAGCUUUCUCGAGAGUCUUGGAAAGU

>P1977

GAGUCUUGGAAAGUUUCUUAAUCUUAAGACAGAAUGAGGUGUAUGUGUAAG

>P1978

UUAAGACAGAAUGAGGUGUAUGUGUAAGAAUGCUUUUAUUAUUCCAUCAGA

>P1979

GUAAGAAUGCUUUUAUUAUUCCAUCAGAUUUCAGGGGCUGAGAAAACCCAG

>P1980

UUCCAUCAGAUUUCAGGGGCUGAGAAAACCCAGGCAGGUUCUUAAUGGGUU

>P1981

GCUGAGAAAACCCAGGCAGGUUCUUAAUGGGUUGGUUUUUGCAUUCCAGUU

>P1982

CUUAAUGGGUUGGUUUUUGCAUUCCAGUUUUGUACUCAGGCACCAGUUUCU

>P1983

CAGGCACCAGUUUCUCCAGUUUUUUAAUGUUUAGCUUACACUUUCAUUAAC

>P1984

UAGCUUACACUUUCAUUAACGUUAUAGUAACGGGUUCUUGGAAACUGAUCG

>P1985

UUUUAUGGCAGACUAGGUUUCCAUUAGCAACCAGAUGAAUCAAUUUCCACU

>P1986

UUUCCAUUAGCAACCAGAUGAAUCAAUUUCCACUAACCCUUUUCUAUAAUU

>P1987

UCAAUUUCCACUAACCCUUUUCUAUAAUUUUGAUGAAUGUGUAAGUUAAAC

>P1988

UUUCUAUAAUUUUGAUGAAUGUGUAAGUUAAACAUUAAAGAGCUAGAGAAA

>P1989

UGUGUAAGUUAAACAUUAAAGAGCUAGAGAAACUGGUGCCUGAGUACAAGA

>P1990

GAGCUAGAGAAACUGGUGCCUGAGUACAAGAACUGGAAUGCGAAAACAAAC

>P1991

CUGAGUACAAGAACUGGAAUGCGAAAACAAACCCAUUAAGACCCUGCCUGA

>P1992

AACAAACCCAUUAAGACCCUGCCUGAGUUUUCCCAGAUCCUAAAGUCUGAU

>P1993

CCCUGCCUGAGUUUUCCCAGAUCCUAAAGUCUGAUCAAAUAAUGAAAGCAU

>P1994

CCAGAUCCUAAAGUCUGAUCAAAUAAUGAAAGCAUUCUUACACAUACUCUU

>P1995

AUACUCUUUGUACCAGGGCCCACUUAAGAUUAAAAAACUUUCCAAGACUUU

>P1996

CCACUUAAGAUUAAAAAACUUUCCAAGACUUUAGAGAAAGCUUUACAGACC

>P1997

GACGGUAGUUAGAUUGUACAGACUGAAUGAAACACUCUCACUUGUAGAUGC

>P1998

CACUUGUAGAUGCACUCUCCCAUGUAGCAUGGAGCUUAGAAUGCAUAUAAG

>P1999

GAGCUUAGAAUGCAUAUAAGUGCUAAAGAAAACAAAACAAAACAAAACAAA

>P2000

CAAAACAAAACAAAACAAAACAAAAAACCAAAUUGUAACUUUCAGUUGGUC

>P2001

UAGAGAUUGUUUAUUUUAUUUUAUAAAUAAAAUAUAUAUUUAUUUAUAAAA

>P2002

CAUGUCCUAGAAUACCUGCAUGGCCAGUUGAUUCGUCUCAGAAAUCCCACA

>P2003

AUGCUAAGCUACAAAAUUUUAAAGCACAGAUCCCUUGGGAAUGUUACAUGG

>P2004

CUAAUGCAUACUGUCUAUAAAAACAAGCCAGGCUCGGUGGUUCACACCUGU

>P2005

UUUAACUGUUCAGAUGGUUUGGACUACAAGGUACAGUUCUUGCAUCUGAAU

>P2006

AACCUCUUUCCACUGUUACUUAUCUAUAUCAGUCCCUAAAUCUGGGUGCAG

>P2007

CCAUACAGGCACACUGGAAUCUCCAAGUUCCCCAAGGACCUACCCAUGACA

>P2008

UUUUUAUUGCCCAGGCUAGAGUGCAAUGGCCCAGUCUCAGCUCACCACAAC

>P2009

GAUUGUUGGCUGGUUAUGGUGGCUAACGCCUGUAAUCCCAGCCCUUUGGGA

>P2010

AUUUUGAUUUUUUGUUUUGGAGACAAGAUCUCGCUGUGUCGACCUGGCUGG

>P2011

AUGUCAUGGUUAAAUCUCCAUAAGUACACAUAAAAAAAUGUCCAAAGCACA

>P2012

AAAUGUCCAAAGCACAUUUGAAAUUAAGGUUUUAUAAGGUAUAGCUAACAU

>P2013

UUUAUAAGGUAUAGCUAACAUUUUUAAGAACAUGUUAUGUGUAAAAUUCUG

>P2014

UGUCACAGAUUUAUGCCUAUCUCAGAUGUUGUGCAAGGCGCCUCAUGGCUU

>P2015

GGGAAGUGUGCACACAUUUAAAGCAACAAUUCUGAAAUGUGUCCAUGUGGU

>P2016

UGUGGUGAAGUGUGCACCUACCUGGAAGCCAUGAGCACCCUGCAGAACACC

>P2017

CUGCAGAACACCUGAAACAUUGAUAAGUGUAAAUCUGUAAUAGAAACUGAG

>P2018

CAAUUUUAUACCUAUCAUGUUAUUUAAAAGUUAGAUAUACCAUAUAAAAUU

>P2019

AUUUAAAAGUUAGAUAUACCAUAUAAAAUUUUAAUUCCAAAUGUGCUUUGG

>P2020

CUCCAAAAGUGCUAGGAUUACAGGCAUGAGCCAUCAUGCCCAACCUCCCCA

>P2021

UGUUGUAGAGAUGGGGUUCCACUAUAUGGCCCAAGCUGGUCUUGACCUCCC

>P2022

AAGAUCGAGCCACUGCCUGAGUGACAGAGCGAGACUCCAUCUCAAAAAAAA

>P2023

CUCAUACCCAUAAUCCUAGCACUUUAGGAGGCUGAGGCGGGAGGAUCUUUU

>P2024

UUGAGACCAGGAGUUAGCCUGGGCAACACAGUGGGACCCCAUUGCUAUUAA

>P2025

ACAGUGGGACCCCAUUGCUAUUAAAAAAAAAAUUAGCCAGUUUUAGUGGCA

>P2026

AUUAAAAAAAAAAUUAGCCAGUUUUAGUGGCACACACCUGUAGUUCCAGCU

>P2027

GGGAGGCUGAGGGGAGGAUCGCUUGAAUCCAGAGGGUCAAGGCUGCAGUGA

>P2028

CUAAGUGGGAAGAUCACUGGAGCCCAGGAGGCAGAGAUUGCAGUGAGCCGA

>P2029

AUUUCAAUUAUAUCUGAGACAGCGUAUAUAAGUUUAUGUAUAAUCAUUGUC

>P2030

ACAUCGUGAUGACCCAGUCUCCAGAAUCCCUGGCUGUGUCUCUGGGCGAGA

>P2031

ACUGCAAGUCCAGCCAGAGUGUUUUAUACAGCUCCAACAAUAAGAACUACU

>P2032

AGUGUUUUAUACAGCUCCAACAAUAAGAACUACUUAGCUUGGUACCAGCAG

>P2033

UACUUAGCUUGGUACCAGCAGAAACAAGGACAGCCUCCUAAGCUGCUCAUU

>P2034

UGGCAGCGGGUCUGGGACAGAUUUCACUCUCACCAUCAGCAGCCUGCAGGC

>P2035

UUAUUACUGUCAGCAAUAUUAUAGUACUCCUCCCACAGUGCUUCAGCCUCG

>P2036

AAAGUGGGGUCCCAUCAAGGUUCAGAGGCAGUGGAUCUGGGACAGAUUUCA

>P2037

GAGGCGGGAGGCCAGGUAGCUCAGGAACCCAAACCUGUCGGGCAGGUUUUG

>P2038

GGCAACGUGCAGACGCUGGUGUGCCAGAGCUGGGAGAACGGCGAGCUCUUU

>P2039

CCAUUAUUGCAUUGGGGGAUGAAGAACAAUGACUUUUCUUUUCUUUUGUGA

>P2040

UUUUCUGAAGUUGCAAGACUUAAAGAAAUAAUCCAUCUGCAUCCCAAGUCC

>P2041

GAAAGACAGGAAUCCUAAAAGGGAGAGUUAUUACUGGCCACAAGCCCUGUA

>P2042

AGCCCGUCCAAGGUGCUGGGAUUACAGAUGUGAGCCAUUGCUCCCAGCAUU

>P2043

AAAAAGUAAGUAAUUUGGGAGGCUGAGAUGGGAGGAUCACUUGACCCCAGG

>P2044

UGUGUGACAUGCAAAAGUAUUUAUUAAAUGAAAAUAUUUUUUAUCUUUUAU

>P2045

CUUGGGAGGUCUCACCCAGUGAGGAACGGAUCGGGGAUCUACUUAAAGAAU

>P2046

CCCUGUCCCCUAGGAAGUCAGUCUCAGGUAGGUAAAACACUGUUGCUGGUG

>P2047

CCUUGAUUAUGUGAGUAAUGCGAGUACCUGGUUGUUUCAGUUGAAGGUGCU

>P2048

GCGAGUACCUGGUUGUUUCAGUUGAAGGUGCUGUAUUGACUUGCCCUUUUC

>P2049

CUGUUUUUGCAACUUAGCCACUUCAACCUUCAGUCUUUGGAGUGUCCAAGG

>P2050

CAGCAUACCACAGCUGCUCUAUAAAAAUGAGGCCAGACUGCGUUUUUAAGC

>P2051

AAAAAUGAGGCCAGACUGCGUUUUUAAGCACUUUCCCAAUGCCAUUCCUCC

>P2052

AGAAAAUAUUUGCAAACUAUACAUUAGACAAGGGGUUAACAUCAGAAACAU

>P2053

AUAAAUCCAAAGCCUUCAUGCCUGUAAUCCGAGUGCUUUAGGAGGCCAAGG

>P2054

GAAUACAGUGGCAUGAUUUCAGUUCACUGCAGCCUCGACCUCCCAGGCUCA

>P2055

UUAAGUGAAUUCAACAAUUUCUGUGAGUGGCUCUUUGCAUGAACACUGUGC

>P2056

AACAUGGGAAAACUUCAUCUCAACAAAAAAUACAAAAAUUAGCUGGGCGUG

>P2057

CCUUAAAUACAAGUUGAGCCUCUGAAAUUCAAAAAUCUGAAAUCUGAAAUG

>P2058

AAAAAACAGCAUAAGAAUUAAAAACAGUUAUAUUCCAAUGUUUUUAAUACU

>P2059

ACCACGAAAGCUGGAAAAUCUUGCCAAUAUGUGUUGCAGAGAAGCUGAGAA

>P2060

AACAUUGUUUUAUUAUAACAUUGAUAAAAAUAGUUGUUUUGUUACACGUCA

>P2061

CUCCUCAUCCAGUGAGGAGUCGGGGACGACGGAUGAGGAGGACGACGAUGU

>P2062

AAAUGGGCCACAACAAACAAGACUGAGAGCAUGUACUUAUCUUGCUUUUUC

>P2063

UCCCUGAGCAGAGGUCACUGAACUGAGCACUGGGUGUCCAGAGGGUGAACC

>P2064

GCUGAGAAAAGAGACUAGAGGCAGGAGAGCUUUGCAGUUCUUCUAAGGAAU

>P2065

AAGGAUGGUGGGAGAUGAAAGACAGACACCAGAUUCUCUUCCUCUUCUAUG

>P2066

CCCACACCUGAAAUCCCAACUACUCAGGAGGCUGAGGCACGAGGAUUGCUU

>P2067

UGUGUCCCUGCCCAAAUUUCAUGUCAAAUUGUAAUCUUCAGUGUUGGAGGU

>P2068

GAGAUGUGGUUGUUUAAAAGUGUGUAGCAUUUUCGCCUUCACUCUCUUCCU

>P2069

CCACUCACUGCAGCCUUGACCUCCCAGGCUGAAGCAAUCCUCCCAAGUAGC

>P2070

CAUAAGACAUGUAGACCAAUGGGAUAGAAUGGAGAGCCCAAUAUAAGCCCU

>P2071

AGGGGGAUAUGAUAGUCUGCGCAGCAUACGCACACGAACUGCCAAAAUAUG

>P2072

UGAAAUAAGAUUCAUCCAUUUUGGAAGCCAGGCAUGGUGCUGUGCAUCUAU

>P2073

GCGGCUGCUUCCUGGGAAGGUCGUGAGUCCCGCUGAGCUGUCCCCGGUGCC

>P2074

GACAAAGCGGGAGUGGAUGUCUUAUAUGGCAGGAGCAGGACUGAGAGAGAG

>P2075

AUAUUCAGUUAACAAGGAGCAGCUUACAAGAGCUGGAUUUUAUGCUUUAGG

>P2076

UCACAAUCAGGUAUUCUAGUUAUUUAAGCAUUCAUUAUCCAAGUGACACAG

>P2077

UCCAAGUGACACAGGAAGUUGAACCAGUAAGCAGGAGUCAUCAUAAUAUGA

>P2078

CCCUGAUAAACAGACGAAUAACAUUAGGUUUCACUUAAGCACCCUCUCCAC

>P2079

CCAUUAACCCUGACCCAGCUGUGUUAGAUAGUGAGGGUGUUUAAGUGAAUU

>P2080

UACUAAACAAGAGGAUAAUGGGGCAAGGAAAAUGGGGGCAGUGUAGUGCAG

>P2081

GUCUUAUAUGGCUGGAGUAGGAGGGAGAGAGCAAUGGAGGAGUUGCUACCC

>P2082

CUACAAAAAACGCAAAAAUUACCCAAGCAUGGUAGCACAUGCCUGUAGUCC

>P2083

CAGGCCUGUAAUGAGAGAGGCUGCCAUGAAGGUAUCUGAAAUGCCUUCAAG

>P2084

CAUGAAGGUAUCUGAAAUGCCUUCAAGGCAUUUUCCCCAUUGUCUUGGCUA

>P2085

UUUCUCGUAAUUUGCACUGAGGGAAACCAAAAUAUUUCACCCCAAAACAGA

>P2086

UAACUCAGUGUAACCUCGAAUUCCUAGGGUCAAGCAAUCCUUUUUCCUCAG

>P2087

UUAAAAUGAAAUUAUCAUUUGACCCAGCAUUCUCAUUACUGGGUAUAUACU

>P2088

GAGCUCAACCUAGAUUCCCAUCAACAGUAGACUAGAUAAAGAAAAUGUGGU

>P2089

AGUGACAUGGGUGAAACUGGAGGUCAUUAUCCCAGGCAAACAAACAAAGGA

>P2090

GGUCAUUAUCCCAGGCAAACAAACAAAGGAACAAAAAACCAAAUACUGCAU

>P2091

UGCCAAAAGCAACCAGAUGAUGAUGAUGAUGAUGAUGAAGGAGAGUUCUGU

>P2092

UAGUAGGCUGAGGAGGAGGAGGAGGAGGGGUUGGUCUUGCUGUUGCGGGGG

>P2093

UUCAUGGACUUACCCAAUUAGCAGAAGAGCCAGGUUUGGGCAGGAUCUUGG

>P2094

CCCCACCCAGCAGCAGCCAUGUCUUACUCAUUUCUGUAUCCCCAGCAUGCA

>P2095

ACCUGCCUCUACAAAAAACACAAAAAUUGGCCGGGCAUGGUGGCCCAUGCC

>P2096

GGUGGAAGGAUCACUUGAGCCUGAGAGGUCAAGGCUGCAGUAAGCCGACAU

>P2097

UAUGCCACCACACUCCAGCCUGGGCAACAGAGCAAGACCCUGUCUCAAUUU

>P2098

UCUUCAAUUAAAAUUAUUUUAGGCCAGGUACAGUGGCUCACGCCUGCAAUC

>P2099

ACAUCUUUAAACUUUUUUUAGAGACAGUGUCUCUCUCCAUUACCUGGGCUG

>P2100

GUCCUGCUUGGCCACCCACGGUGGAAUGCAGUAGUGCAAUCAUGACUCACU

>P2101

UCUGUCACCCAGGCUAGCAUGCAGUAGCACAAUUACAGCUCACCACAGCUU

>P2102

GCAGGGCAUGGUGAUGAAUGCCUGUAAUCCCAACACUUUGGAAGGCCAAGA

>P2103

AGUAUUAUGCUGUGUUAGUUACUAUAGCUUUGUACUAUGUUUUUAAGUCAG

>P2104

AGGUUAAGUUGUUGUAUCACUCAGUAAAGACCCUAGACUAUCAAACCCAGA

>P2105

AGCCUCCCAAAUAGCUGGUACUACAAGUGUGCACCACCAUGCCUGGCUAGU

>P2106

UCAUCCCCGUUUCUUCACUGUAAAAAUAACACGUUUUUUGUAGAUGACUUG

>P2107

UGAAUUUCAUGUUGUCUAUUUCAGUAUGUAUUAAAUUAUCUGUGACGGUGG

>P2108

AAGAAAUUUUUCACUAUUAAAAGAUAGUAAAAAAAAACAAAAUAUCAAAGA

>P2109

GUAUGUUAAGAAACAGUUUCCGGUCAGGCACAGUGGCUUACACCUGUAAUC

>P2110

AGAAAAAGAGUUACAAGGGAAUUGUAGUCUUUUUCUGAAUAGAAUAUUAGU

>P2111

UUGCUCUUGUUGCCCAGGGUGGAGUACAAUGGUGUGACCUUGGCUCACUGC

>P2112

UCGGCAACGAGCGGUUCCGCUGCCCAGAGGCGCUCUUCCAGCCUUGCUUCC

>P2113

UGCCCCGAGGCGCUCUUCCAGCCUUACUUCCUGGGCAUGGAAUCCUGUGGC

>P2114

UCCAGCCUUGCUUCCUGGGCAUGGAAUCCUGUGGCAUCCACAAAACUACCU

>P2115

AUGCCAGGCAAGGCCAAGCUGGCUCAAAGAGCAACCAGCCACCUCUGCAAG

>P2116

UCAAAGAGCAACCAGCCACCUCUGCAAGGGUGUGCCAGGAGCAGGUGGAGC

>P2117

ACCUCUGCAAGGGUGUGCCAGGAGCAGGUGGAGCAGCCACCAACCUCACCC

>P2118

CUUCAGGAGCUCUUGUAACGCAAGAAUGUGGAUUUAUUUCUGGUAAGGUAA

>P2119

UAUCAGAUCAUUCACAAAUACAGAAAUACCAUCUCAAUAAUUAGAAGAAGU

>P2120

GAAUGGGGUAUCCCUCCCCUCAAGCAUUUAUCCUUCAAGUUAUAAAAAAUU

>P2121

GAAUGACCUGAGCGUUGAGACUGCCACUGUUGUCCAUGUCACCCACCAAUA

>P2122

CCGCUGUUGUCCAUGUCACCCACCAAUACAAGGAACGCCUCUGUGGGACUC

>P2123

CCCAGGUGAUCACCAAGCCCCAGGUAGACACCAGCCCAUAGGUGAGCAACA

>P2124

GGGCUGGCUGCCUUUCUUUCCUCUAAGAAAAUGAAGUGAGGGGCAUGCAAA

>P2125

CUAUAGAGCUUUCAAGGGUCUUGGAAGCUCUUUGGAAGGGAACUAGGGAGU

>P2126

CUGUCUCUACAAAAAAAAAGUUAAAAAUUGACUGGGUGUGGUGGUGCGUGC

>P2127

UUUUAUCUUGAGAACAUGAUGUCUGAAGUUAAAGGUAUUGGCGUAUUCCAC

>P2128

UCCUGGCCCAGUUUCCCUAGUAGCUAGGACUACAGGUGAACACCAUGAUGC

>P2129

ACAUGCUAAGUAAAAGAAGACAGUUACAAAGGACCAUGUACUGUAUGAUUC

>P2130

GCCCACCUCGGCUUCCCAUAAUGCUAGGAUUACAGGCGCAAGCCAUCGCAC

>P2131

CUGGAUACACAAGCACAUAUACAUUAGCAGGCAUUUUGACUAACAUUUUGA

>P2132

UCUUGUUUAAAAUGUCAUUUAGCCAAGCAUGGUGGCUUGUACCUGUAAUCC

>P2133

UUUUAAAUCUUCUUCUACAGAAAGUAAUUUGAAGGCCUUCCCUGACUUAAA

>P2134

GCAUGCCUAUAAUCCCAGCACUUGAAGCCAGGAGUUCGAGACCAGCCUGGG

>P2135

UUAAAAAUAAGCUGGGCAUGGUGGCACGUGCCUAUAGUCCCAGCUACUCAG

>P2136

UAACUAAAUAAAUAACAAAUAAGCUAGACAUGGUGGCACGAGCCUGUAGUC

>P2137

GGUGGCACGAGCCUGUAGUCCCAGAAGGAAGAGGUGGGAAGACCCCUUGGG

>P2138

UUUAAAUGCCAUUGUUGGGCCAGGCACGGUGGUUUCCACCUGUAAUCCCAG

>P2139

GGCUAAUUUAUUUUUAUUUUUUUGUAUUUUUAGUAGAGACGGGGUUUUACC

>P2140

AAAAAUAUUAAGGGCAGCGAGAGAGAAGGGUCGGGUUACCCACCAAAGGGA

>P2141

UGUCACCUCAGGCUCCCAAGUGGCUAAGACUAUAGGUAUGUGUACCACACU

>P2142

AGUUCCAGACCCAUCUGGGCAACAUAGUGAAGACCCAUCUCUACAAAAUAU

>P2143

GCCUGUAGUCUCAGAUGCUUGGGAGACUAAGGCGGGAGGGUUGCUUGACCA

>P2144

UGGACCUGGUGGUCCCUUUCCGUGUAAACUUCCGGCUGAAAGGGAAGGACA

>P2145

GGGAGAGCUCGUGGCUCAUUGAGGAAGGCAAGGUGGUGACUGUGCAUCUUG

>P2146

UGAGCAGAGAUCACCACUACACUCGAGCCUCAGUGACAGAGACUGUCCUCC

>P2147

UUAUGAGUAAAUACAAAAUUGGCUGAGCGCAGUGGCUCACACCUGUAAUCC

>P2148

CAAAACUUUUGGAAAGACUCAGGCCAGGUGUGGUGACUAACACCUGUAAUC

>P2149

UUUCUGUGCCUGGCUUAUUUCGCUUAACAUAAUGAUCGCCAGUUCCAUUCA

>P2150

UAAAGCAACUUAUGACACUACUCUUAUGGAGAAAGAAGGUACCCAUGUUCU

>P2151

UUUUUUUGAGAUGGAGUCUUGCUCUAUUGCCCAGGCUGGAGGGCAGUGGGG

>P2152

GCGCAGAGAUCCUACUCUGAUGACAACAAGAACAAAAAACACAACACAAUC

>P2153

AGAGUUGAAAUAUCAUAAACAACUAACCUGAAAUCUAAGGAAGGAAAGGCA

>P2154

ACUAACCUGAAAUCUAAGGAAGGAAAGGCAUCUCCAAGGUUGGACUUGCUU

>P2155

GGCAUCUCCAAGGUUGGACUUGCUUACCUGGGACAGAUGAUGCUGGACACU

>P2156

GGGACAGAUGAUGCUGGACACUAGUAAGAAGAACUGAGUUAAAAAUGUUUA

>P2157

CACUAGUAAGAAGAACUGAGUUAAAAAUGUUUAGCAAAUUGCUAAGUGUUG

>P2158

AGUUAAAAAUGUUUAGCAAAUUGCUAAGUGUUGAAUGUGGGCAGGCAAGAG

>P2159

GCUAAGUGUUGAAUGUGGGCAGGCAAGAGAAUACAGAACCCCACAAGGCAU

>P2160

GGCAGGCAAGAGAAUACAGAACCCCACAAGGCAUCAGUCAGGUGGUGAAUU

>P2161

UCAGGUGGUGAAUUCAUACCCCUUGAGGGCUCUUCUACAUGGACCUCCCCU

>P2162

AUGGACCUCCCCUGAUGCUAAUUUUAAAAAGAUUGGCCAGAGUCCUGAGAA

>P2163

AAAAAGAUUGGCCAGAGUCCUGAGAAGGCCUCCCACUGAGUGCGGGGCUGG

>P2164

AGCAGCAACCCGAGGAUGGAAAGGCAGAAAACCCCAGUCUUCCUUAGGGCA

>P2165

UUAAUUUUUACAGUCAAGAACAAUUAGAUUGUCUGCUCGUAUUUUCUGGAU

>P2166

CAAUUUUAUCUUUUAGUUUCUAAAAAAUUUAAAAUGAAUUUUAAAAAGUAG

>P2167

AGAAUUUCGCUGUGUUGCCAAGGUUAGUCUUGAACUUCUGACCUCAAGUGA

>P2168

UUCCUUUCCAAGACAUGGUCUUGCUAUGUUGCCCAGGCUGGACUCAAACUC

>P2169

CACGAUCAGCUUACUUGAGCCUCCUAGGCUCAAGCGAUCCUUCCACCUCAG

>P2170

GCUGAAGAAGAGCCGAGAGGGAGGAAAGGGAGGCAAGAAGGAAGCAGGUAU

>P2171

UGGAUCAUUGCAACCUCAGCCUCCUAGGUUCAAGCGAUUUUCCUGCCCAAG

>P2172

UACACUGUUCCUGGGAAUGUAAAUUAGUUGAACUAUCAUGGAAGACAGUGU

>P2173

AAUAACUUGUUUCUUAAAGCUUGCAAGCUGGGGACUGUAGCUCACACCUGU

>P2174

GUAGCAAGGCCUCAUAUCAACAAAAAGUCAAAAAAUUAGCCAGAUAUAGCA

>P2175

ACAAUACUGUGUCUUUAUUUUUUCUAUGUUGCUACUUUUUCUAUGUUUUGU

>P2176

UACUUUUUCUAUGUUUUGUUACACAAGUACCACCGUGUUACAGCUGCCUAC

>P2177

GUUACAGCUGCCUACAGUAUCCACUACAGUAACAGGCUGUACAGCUUAGCA

>P2178

ACUACAGUAACAGGCUGUACAGCUUAGCAGUUUAGGAGCAAAAGGCUGUAC

>P2179

UACAGCUUAGCAGUUUAGGAGCAAAAGGCUGUACCAUAUAGUCUAGGCAUG

>P2180

AGGCUACACCAUCGAAGUUUGUGUAAGUCCACUAUGUGGUAUUAGCACAAU

>P2181

UAUUGCUCCCAGGCUCCAAGCCUGUACAGCAUAUUGCUGUACUGAAUACUG

>P2182

AAAAUAUUGUAGCAUCAGGUGUCAGAGCAUCUUGACAGCUGGCAGAUGCCU

>P2183

GCCUGCUCCCUCUGGUGCUCAGAUAAGGCCUGGGGAGCUGGGAAUCAGCUC

>P2184

UCAGAUAAGGCCUGGGGAGCUGGGAAUCAGCUCCUGACUUGUUACCUCCUC

>P2185

GCAGCCUUGUUCUCCUGGUCUCAAGAGGUCCUCCCACCUCAGCCCCCUGAG

>P2186

UGAUCACCCCACCUUGGCUUCUGAAAAUGGUAGGAUUACAGGCAUGAGCCA

>P2187

ACUUUCUGUACUGUACUAGAAAAUUAGUGUCUGGGUGUGGUGGCUCACUCC

>P2188

CUACAGCCUAGACCUCCCAGGCUCAAGUGGUCCUCUCACCUCAGCCUCUCC

>P2189

AUGGGUAUGAAGUGGUAUGUAUCUCAUUGUGGUUUUGAUUUGGAUUUCCUU

>P2190

UACACUUGUCUUGUUACUCAGUUGUAAGUGUUCUUUAUAUAUUCUAGAUAC

>P2191

CUUUAAUAAACAUUUUUCCAAAGAUAUAUUAAUGGCUGAUAAGCACAUGAA

>P2192

AAAUAUUAGUCAUUAGGGAAAUGCAAGUCAGAACUACAGUGAGAUACUUAA

>P2193

AACCUAAGUGGCCACCUGAAAUGACAACCCUGCUAUCCCCUGAAUUUAAAA

>P2194

AGAGGCUUUUGAGUGGUUUCACCACAAAGAAACAAUUACAUGCAUGAGGUG

>P2195

CCAUCUAUCACCUCAGACAUGUAUUAUUUAUUUGUGUUGGGAGCAUUCAAA

>P2196

AUAGUUCCAGAGGCUGGGAAGUCCAAGAUCAAGGCUCUGGCAGAUUUGGUA

>P2197

UGUUUUCAAGAUUUAUCCAUGUUGUAGCUUGUAUCAGUACUUCAUUCUUUC

>P2198

AAAAGCUUACAGAAGUGGUUCCCAAACUUGUUUACACAUUGAAAUCAUCUG

>P2199

CUCCACCCCAGAAUAAUUAAAUUUUAAUCUCUGGAGAUAUGUGGCAAGCGG

>P2200

GAGACAGGCCUUUGCUCUGUUGCCCAGAUGGAAGUGCAGUGUAGGCUUAUA

>P2201

GUGGACCCGUGUUCAUAGUAGCAUUAUUUGCAAUGGCCAAAAGGUAGAAGC

>P2202

UAAUUCAGCCUUAAAAAGGAAGGAAAUUCUGACACAUGCCACAACAUGGAU

>P2203

GAAGAUGAAACAAGCCUGUCACAAAAGGACAAAUACUAUAUGAAUCUACUU

>P2204

AACAAGGUUAGGCAUGGUAGCCCACACCUGUAAUCCCAACACUUACGGAGG

>P2205

AGCUAAUGUUUUGUUAUUUUUUGUUAGAGAUGAGGUCUCACUACUUUGCAC

>P2206

CAGUUCGUAGCAAACUACUGGAACAAGAAUCUGUUUUCUUGCUGAGUGAAU

>P2207

AAUGGCGCACACCUGUAAUCCCAGCAGUUUGGGAAGCCAAAGUAGGAGGAU

>P2208

GAUUCAUGGUCAAGAAAAAUCUUAUAUAUAUAUAUAUAUACACACACACAU

>P2209

AAUCUCUGGUUUUCAAAGGUAAUAUAGAAUAUUUGACACUUGGUAAAAGGU

>P2210

UUGGGAUGUGUUGCUUAACUCCUUUACACCAUUAGUUAUCUGUGAGACCAU

>P2211

CGUGGUAUUAAUUCUGCAGGCCCACAGAAUGCAAGAGCUAUGGAGAUGUGG

>P2212

AAACAACAAAAUGGUCAGAAACAGAAGAAUGGGCCAGACGUCGUGGCUCAC

>P2213

GAGUGCAGUGGUGCGACCUGGACUCAUUGCAGCCUCAAUCUCCUGGGUGCA

>P2214

GCUCUCUGGGCUGGAGCACAAUCAUAGUUCACUGCAGCCUUUAACUCCUGG

>P2215

AAAUCUUUUUUUGCAUAUUUUAAUUAGAUAUUACAGUAAAGAGAACAAUCU

>P2216

UGGAGUGCAGUGGCUCACUGGGACUACAGGUGUGAGCCACCAUGCCCGGCU

>P2217

GAUUCUUGCUGAAGCAUCUAUGAGAAGUGUGAUUUUAGCCAAGUCUGUGCC

>P2218

AAUUUUCUUUCAGCUCUACUCAAUUAAGGGGUAUCAGUAUGUGAAAUGGAA

>P2219

CCAAAAUUAAAAUCCAAAAUAGCCAAGCAUGGUGGCUCACGCCUAUAAUCC

>P2220

UAGCCAAGCAUGGUGGCUCACGCCUAUAAUCCCAACUACUCCAGAGGCUGA

>P2221

AAGCAAACAAGAGGCCGGGGGUGGUAGCUCUCACCUGCAAUCCCAGCACUU

>P2222

GGCAACGUGGUGAGGCCCUGUCACUACAAAAAAUACAAAAAUUAACCAGGU

>P2223

CAGGAAAGUGAAGAGGAAGACAGGAAUAUCUGUCCACAAAGUAGGAAAUGA

>P2224

CAAGGUGGGCAACUCACUUGAAACCAGGAGUUCAAGACCAGACUGGGCAAC

>P2225

ACCACUGCACUCCAGCCUGGGUGACAAAAAAAAAAAAAAAAAAAAGAAUCU

>P2226

CUUAGGAUGUUCACCCAAUGACUAUAAUCUAAAUUCACUACACAAAACUAU

>P2227

CAUCUUGGGGAAAAAAAAAAAAAUUAGCCAGGCUUGGUGGUGGAUGCCUGU

>P2228

UAGCCAGAAUGGUUUCUAUCUCCUGACCUCAUGAUCCGCCCACCUCGGCCU

>P2229

AUACCAUGUACUUGCCAUGUGUAUUAGUCUGUUCUCAUGCUGCUAAUAAAG

>P2230

ACAUACCUGAGACUGGGUAAUUUAUAAAGGGAAGAAGUUUAAUGGACUCAU

>P2231

AUGGCUGGGAAGGCCUCACAAUCAUAGCAGAAGAGGAAGGGAGAGCAAAGA

>P2232

AGAAGUCCCCUUAAUAAAAUCAUCAAAUCUCAUGAGACUUAUUCACUAUCA

>P2233

GUAUUUGAAGAACUGAGCUCUGUACAUUAUAAAUUUGCUUAAAGAUUUUGA

>P2234

CUUAUUUGCUGUCAAAUUCUGAAUUAAAAAAUCUGUUGGUGGCCAGGCGCA

>P2235

UGAAUUAAAAAAUCUGUUGGUGGCCAGGCGCAGUGGCUCAUGCCUGUAAUC

>P2236

UUAAUAUAUUUAUUUUCAUUUUUUUAAGAGACAAGGUCUCGCUAUGUUGCC

>P2237

GCUACCUCCCUGAAGGCAGUUUCUUACAAAAGCUGCUUAGGAAAGCAAGUG

>P2238

GAUUGAGGGAGGUUAGGGCUUCAGUAGGCCGUGAUCAUGCUAUCACACUCC

>P2239

AAAAGCAAAAUUACUUGGGGAAAUUAAGUAACUUGACCGUGGCUAACCAGC

>P2240

ACAUGUGUCUACACCACUACACUGCACUAUGUCAAAUCAACAAUAUACUUA

>P2241

UGGCUUUUCCUUUCCUUUUGUCAUUACUCAGGGAAUGAUAAAGAUAUUAUU

>P2242

UUACUCAGGGAAUGAUAAAGAUAUUAUUGAUUUGAAACAGUACAGUGUAGU

>P2243

AAAGAUAUUAUUGAUUUGAAACAGUACAGUGUAGUGUUAUAGACACACCUG

>P2244

AGAGGUUUAAUGAACUCACAGUUCCACAUGGCUGGGGAGGCCUCACAAUCA

>P2245

GCAAUGUGACUUUUUAUUUUUUGAGACGGAGUCUCGCUCUGUCGCCCAGGC

>P2246

AGCAAGGCAUGUCUUACACAGCAGUAGCAGGAGAGAGAGAGAGCACAGGGG

>P2247

AUAAGUCAGACACCAUGGGACAAAUAUGGUAUGAUUUCACUUACAGAAGCU

>P2248

AUGAUUUCACUUACAGAAGCUACUUAGAAUAGUCAGAAUCAUAGGGACAGA

>P2249

UUUAUAAAGAAAAAAUGUUUAAUUGACUCACAGUUCUGCAGGCUGUACAAA

>P2250

AAAUUUUAAAUGAUGUAUGUACAUUAUAGUUCUGUUGAACAGUGCUGGAUA

>P2251

CUUUGACAUACUGAUUUUCAACCUAAAUGCCCAUCAAAUGAUGAAUGGAUA

>P2252

UCCUGCCUUGGCCUCCUGAAUAGCUAGAACUACAGGUAUGAACCACCAUGC

>P2253

ACAUACAUCCACACAAUUCUACCUUAGAUACCCAGUUCAUAGUGUGAACAC

>P2254

GCAGAAUUGGGUAGUUGUGACAGAGACCAUCUAGCCUAUAAGACCAAAAAU

>P2255

AGCAUUUUGCUGGGAUUACAGGAGUAAGCCACCACACCCGGCCAAUUUUUA

>P2256

UAUCAGUUUGAUGAGACAGGGUCUUACAUUGUUGUCCAGGCAGGUCUUGAA

>P2257

CUUUUUUUUGGCACAAUCUCGGCCCACUGCAGCCUCAACCUCCUGGGCUCA

>P2258

UGCAGCCUCAACCUCCUGGGCUCAAACAAUCCACCAGGCUCAGCCUGCAGG

>P2259

CUCAGCCUGCAGGGUAGCUGGGACUACAGGCACGUGCCACCACGCCUGACU

>P2260

UUGGGGGUUGGUCUGCCCUAAACCUAUACCAUCCAGUCACAGUGCACAAAU

>P2261

AUUGCCAUUGAAAGUGGCUCUCAGCAGGAGGGAGAGAGCUGGAAAAUGGGG

>P2262

AAUUGGAACGAUACAGAGAAGAUUAACAUGGCCCCUGUGCAAGGAUGACAC

>P2263

GGCAACAUGGUGAAACCCCAUCUCUACAGGAAAAAAAAAAAAAAAAAAAGC

>P2264

UGUGUCCAUGCACAGCUAACUUUUUAUUUUUUUUGUGGAGAUGGGGUUUCA

>P2265

AGCCAUGUUCACACCGCUGCACUCAAGCUUGGGUGACAGAACAAGACCCCG

>P2266

CUUAGAGUUUGAUGAGACCAGGCACAGUGACUAACACCUGUAAUCCCAACA

>P2267

UGAAACCCGAUCUUUAGUAAAAAAUAAAAAAAUUAUCUGAGCAUGAUGGUA

>P2268

ACAGUGAGACUCCCAUCUCAAAAAAAAAAAAAAAAAAUGCGGGAGAGGGGC

>P2269

UGGUCUCAGCUACUCGAGAGACUGAAGUGGGAGGAUCCCUUAAACCCAGGA

>P2270

CAGACAUUAUUACUGUUAUUAUUUUAAAAUUCAGACAAGGUCUUGUUCUGU

>P2271

AGAUCGCGCCAUCACACUCCAGCCUAGGCGACAAGAGAGAAACUCCGUCUA

>P2272

GGAAGCUGAGGUGAGAGGAUUGCUUAGGCCCGGGAGAUUGAGACCUCAGUG

>P2273

CUUGUCUCUGAAAAAUAAAAAGAAAAGUUAGCUGGGCAUAGUGGUGCACAC

>P2274

UGGCUGGGCGCAGGGCAGUGGCUUAAGCCUAUAAUCCCAGCACUUUGGGAG

>P2275

CAGGGAGACCUCAUCUUUACAAAAAAUAAUAAAUUACCUGGACAUGGUGGC

>P2276

AGACUCUGUCUCAAGAAAAACAAAAAAAAAGAUAAAUGGACAAAAGACAUG

>P2277

AUAUUUUAAGUAGGCUGGGCACGGUAGCUCAUGCCUGUUAUCCUAACACUU

>P2278

UUCUCUUGCCUCAGCCUUUUGAGUAACUGGGGUUACAGGCGCGUGCCAUCA

>P2279

AUUUUUUUGUGUUUUUAGUGGAGACAGGUUUUCACCAUGUUGGCCAGUAGU

>P2280

GUGUGCUAUUCAUAAUUAUUGCAAAAAUAGGCCGGGCCUGUAAUUCCAGCA

>P2281

GGGUGACGAGCGAAAAUCCGUCUCAACCAAGAAAAAAGAAAAAAAGAAAAA

>P2282

GUCUCAACCAAGAAAAAAGAAAAAAAGAAAAAGAAAACUUAACUGGCCUUU

>P2283

AUUUUAAAAAAUUACAGGAAGGGCUAAGCACAGUGGCUCAUGCCUGUAAUU

>P2284

CAGAGUGUAAAUAUUAGCCAAGCAUAGUGGCACACACUGUAGUCCUAGCUA

>P2285

GAGGCCAGGAGUUCAAGGUUGUAGUAAACUGUGAUUGUGCCACUGUACUCC

>P2286

UAUACAGUGCGGCUACUGUGCACAAAGGGGAUGAUUAAUGGUUUGAGUGCA

>P2287

CAUUGCACUACAGCCUGGGCAACAAAAGCUAAAAAACUCCGUUUCAAAAAA

>P2288

GAGUGCAUUGGUGGGAACACAGCUCACUACCUACUCAGCCUCCUGAGCUCA

>P2289

AGCUCACUACCUACUCAGCCUCCUGAGCUCAAGCGAUCCUCCCACCUCAGC

>P2290

CUUGCUGCUAUAAACAAUAUAUUGCACAUGGGAAAGAUAGUUACGUUAUGC

>P2291

UGCUUCUUAUCACUUUUGACUCUUCAGUGUGUUAUCCAGGAAACAAUAUAU

>P2292

UGAAGAAGAGAAGUCUGAAGAGGAGACUUCAGCACCUGCCAUCACCACUGU

>P2293

GGAGACUUCAGCACCUGCCAUCACCACUGUAACGGUGCCAACUCCAAUUUA

>P2294

GUAACGGUGCCAACUCCAAUUUACCAAACUAGCAGUGGACAGUAUAGUGAG

>P2295

CAGGGAGGAGCAAUACAGCUGGCUAACAAUGGUACCGAUGGGGUACAGGGC

>P2296

ACAGGGCCUGCAAACAUUAACCAUGACCAAUGCAGCAGCCACUCAGCCGGG

>P2297

UAAAUUUUCACCUGUUAAGGUGGAAAAUGGACUGGCUUGGCCACAACCUGA

>P2298

AGAUUGGAGAACAGUGGCCGAUCUCAGGUCAAUGCAACCUCUGCCUUCCAG

>P2299

UAGCUAUGUUGCCCAGGCUGGUCUCAGAACUGACCUCAGGUGAUCCUGCCA

>P2300

AUGAGUAUAAAUGCUAUAAACCUUUAAAAAACAUGAUUUGAAAGUUUUUUG

>P2301

AGGCUGGAGUGCAGUGGCUUUUCACAGGUGCAAUCAUUGUGCACUCACUGC

>P2302

CUGCAGCCUAGAACUUCCUGGUCUCAAGCCAUCCUCUAGCCUCAGCCACAC

>P2303

AUCCUCUAGCCUCAGCCACACAAGUAGCUGGGACUACAAGUGCAACUGGCA

>P2304

AACUGGCACCUGUCUCCUGAAUAUUAAGCUUUUAAUUUUUUGUUUCGGUCA

>P2305

CUCUUGAUAGCAGACAUUGACUGAAACAAAAAAUUAAAAGCUUUAUAUUCA

>P2306

GUACCAUGAUGGUGCCUAUAGGAAUAGCUACUACACUCCAACCUGGACAAC

>P2307

AAAGCAGAAGAAAAAUGUUCAGACAAGGUUUUGUAAAGGUUUGUAGCAUUU

>P2308

AAUGCAAUGAAGCUAACAUAACUUGACUUAGGUAGUCCCUUACCUUGGAAA

>P2309

UCAACAGUGAACUAAAAAUUCUAAAAGAGGCAGUUCAUAGUGGAUCAGGUG

>P2310

AGUGGAUCUUUUCCUUUUGUUUUUUAGAGAUGGAAUCUUGCUAUGUUGCCC

>P2311

GGAGUAUUGAUGUAAAUUACCUAGAAUUCUUCUGCAUGGGAGUUGUAUAUU

>P2312

AAACAUUUAUUGAACCUUAUGCUGAAGAUAAUGGGAAGCCAUUAAAGCAAG

>P2313

GUUUGGAUAUAAUUUCUGGUAGUUCACAGAUUCCUUGAUGUCAUCUUUGGA

>P2314

AGUAUACCAGUCUUGGCCCACUUCAACCUCCACUUCUUAGGUUCAAGCGAU

>P2315

UAUUUUGAGUAGAGAUGGAGUUUUCACCACAUUGGCCAGGCUGUUCUUGAA

>P2316

CAUUUAAUAUUUUUAUAAUGUGUCUAGGCUGGGUGCGGUGACUCACGCCUG

>P2317

GACCAGUGUGGCCAACAUAGCAAAAACCCGUCUCGACGAAAAAUACAAAAA

>P2318

AGAGUCCAACUUAGCCAGGCACAGUAGCUCAUACCUGUAGUCCCAGCUACU

>P2319

CUGGCCUGUCCAUUGGUGAUGUUGGAAAGAAACUGGGAGAGAUGUGGAAUG

>P2320

AGCAAAGCCUGAUGCAGCAAAAAAAAGGAGUUGUCCAGGCUGAAAAGGCAA

>P2321

AAUAUUAGGAAAUUAUUGUUCAGCCAGGCGUGGGGGCUCAUUCCUGUAAUC

>P2322

CAGUGGUACAGUCAUAGCUCACUGCAGCCUCCUCCAAAUCUUGGGCUCAAG

>P2323

CAGAGUAAGGAGGUCUCAAACUCCUAGCCUUGAGCAAUCUACCCACCUCAG

>P2324

UAGCCUUGAGCAAUCUACCCACCUCAGCCUCCGCCUCCCAGAGAGUUGGGA

>P2325

AAUAGAAUGAUGAUUGAUAUUAUUUAAUACUGGAGUGCCUCUCCAGUAUUA

>P2326

UUUAAUACUGGAGUGCCUCUCCAGUAUUAAAUAAAAUGUGCUAAGCAUUAU

>P2327

GGUGGCUUAUGCCUGUAAUCCCAGCACUAUCGGAGGCCAGGGCGGGCGGAU

>P2328

GGGAUUUUGCCACAUUGCUCAGGCUAGUCUCUAACUCCUGGGCUCAAGUGG

>P2329

UUUAGCCUCCCAAAGCACUGAGAUUAGUGGUCUGAGCCAUUGCACCUGACC

>P2330

UGGUGCUGGCUCCUGGGGACUACAAAUCCCAGAGUGCGGUGUGCCCGGCCU

>P2331

CCAGGAGGACCUGGGGUGUAGCCCCAUGUCCCCUCCCUCUGCCACUGGUGA

>P2332

UCGAGGUGAAAUCCACAAAUGGGGAAACUUUCUUAGGUGGGGAAGACUUUG

>P2333

UUGUGAAGGAGUUCAAGAGAGAGACAGGGGUUGAUUUGACCAAAGACAACG

>P2334

GUAAAGAAAAAGUAGAAGAUGAAGCAGCAAAGGAAGAGACUAAGGAAUAAA

>P2335

AAAAGACAUGCACGCCCGGGGCAGAAGGGCAUGUGAACAGAUUUGGGCACC

>P2336

UUGGGCACCAAGAAGAGAGGCGCACAGAAAGGGAUUAGUCUUUACUGGAGA

>P2337

GGCGCACAGAAAGGGAUUAGUCUUUACUGGAGAUGUUUAAGAAUGCUAACA

>P2338

UUUACUGGAGAUGUUUAAGAAUGCUAACAGGACUUGAUCCUGCCAGAACUU

>P2339

AAUGCUAACAGGACUUGAUCCUGCCAGAACUUUUGGAAUCUUCCUCAUGGU

>P2340

GAACUUUUGGAAUCUUCCUCAUGGUAUCCUCUGAGGUGUCUUCAGGUCUUU

>P2341

UCAUGGUAUCCUCUGAGGUGUCUUCAGGUCUUUGGGUGUAGCUCAACUCCU

>P2342

AGAAUUCCACAGUGCAAGAGCUUCCAAAUCUAGCCCCUAACUCUCAUCAGG

>P2343

AAGAGAUGAUCCUAAAAUUGUUUUUAUUUUUCUGUUUUUUUGAGAGGGAGU

>P2344

AUGCCGAGGUAGGUGGGUUGCUUGAAGCCCAGGAGUUUUGAGAGCAGCCUG

>P2345

UUCUACAAAAAAUAAAUGGUGGCAGACACCUGGAGUCCUUCCUAGCUACUC

>P2346

UACCUGAGCCUAGGAGGUCGAAAUUACAGUGAACUGUGAUUGCGCCACCAC

>P2347

AGGCUACAGUAACCAAAACAGCAUGAUACUGGUACCAAAACAGAGAUAUAG

>P2348

AGACAGAGACAGAUCAUGAGGCAUUAGAUUCUCAAAAGAAGCACGCAACCU

>P2349

AAGAAAAUAAACUAUGUUUAUUAUUAGGUUGAGGCAAAAAAGUAGUUGCGG

>P2350

GGUUAAAAUCUAAUAGAAGUACCCAAUUGACAAGGAAAUUUAGAUAUUUUG

>P2351

CAUUUUACUUUAGAACAAAAAACUUACAAGACUUUUUUCUCAUGCAAAAUU

>P2352

AUCAUUUCAAGUUAUUUUCCUGUCAACCAAUUUUAUAGCCUGUGAAUAUCA

>P2353

AAAGAAUCUUAAAGUUAAAUAUAUGAGUAUUUUGCUGAUAACUUAGAAGAU

>P2354

UGCAGUUAAUUUAAAGUUCUUACUUAGGUGAACAUUUGAUAUUCACAGGUU

>P2355

UGAUAUGGGGAAACAUGUUUCUAAAAAUUGCAGAAUGUUUCUCAUCUAUAA

>P2356

UAACAUUACUAAGGACAAGAAUUCUAGUUAAUAUGUAAUUCUGUAUAUAAA

>P2357

UAACCUCUACGUUCCAUGUUCGAGCAGUUCUCCUGUCCCAGACUCCAGAGU

>P2358

UAGAAAGUAGUUUGAGGCUGGACAUAGUGGCUCAUGCCUGUCAUCUUAGCA

>P2359

AUGCACCACCACGCCCAGCUAAUUUAUGUUUAUUUUUUUGUACAGACCGAG

>P2360

GGAAGUCCCUCCCAGGUUUGAAAUAAACCUGUUGACUGUCGAGCCACCCUU

>P2361

GACUCCAGAGUUAAAGUUGAAGUUGAUAAAGUUUCUAGCUUUAAUAUCUAA

>P2362

CUUUUUUUUUGUCCGCCACACGGAAAUGAGAUGCUCCUUUAAAUAAAGCGU

>P2363

AAGACAGGUACAGUGACUCACGCCUAUAACCCUAGCUAUUCAGAGGCUGAG

>P2364

AAGAGCCUCGCCCAGCCCCCACGGGACCCCCCGCCAGCCAAGGGAGGCGCC

>P2365

AAAAUACACUGAGAUUAGGCCGGGCACAGUGGCUCACACCUGUAAUCCCAG

>P2366

CAUGGUGGUGCGCAUCUGUAGUCCCAACUGCUUUGGAGGCUGAGGCACAAG

>P2367

UCUGUUCCCAGGUGAUCCUAGGUGGAGGCCGAAAGUACAUGUUUCGCAUGG

>P2368

GUGUGGAACCGCACUGAGCUCAUGCAGGCUUCCCUGGACCCGUCUGUGACC

>P2369

GCAGGCUUCCCUGGACCCGUCUGUGACCCAUCUCAUGGGUAAUGACCCCCU

>P2370

UCCCACGUCUUCUCCUUCGGAGGCUACCCCCUGCGAGGGAGCUCCAUCUUC

>P2371

CCAUCCCGGAGUUCUCCUGCUCCCCACCUCCUGUCGUCCUGCCUGGCCUCC

>P2372

UUUUCAAAUACCUAACUCCAAUACAAAAAAUUAGCUAGGCGUGGUGGCAGG

>P2373

GGGUGGUUGGUUUUUUCUUUUUUUGAGACGGAGUUUCACACUUUGCCCAGG

>P2374

GCCACCGUGUGUGGCCUUUAUUUUUAUUUUUUGAGAUGGAGUUUCACUCUU

>P2375

GUGAUGGGAGGAUAGCUUGAGCCCAAGAAGUCGAGGCUGCAGUGAGCCGUG

>P2376

AGCUGCAGUAAUGGAGCUCGCAUGUAUAUGGCAGCUAUUCCAUGAGGCCCU

>P2377

GGCCAGGUGUAAGAGAUUCCAUGUGAUCUACAAGGUGAAUAGUCUGGGGGG

>P2378

GGUCAGGCUGGGUUCCUGACACCUCAGCUCAUUUGAAUUAUAAAUCUACUG

>P2379

GACAUGGAAGGAGAAAUACUUUGAAAGGCGAAGCAACUGGCUGGGCAGGGA

>P2380

GUGCUAAACAAGGCCAGAGCCAGGAAGGACACGCGGGGCCAUGGAUUGAGU

>P2381

ACACACACACACACACCCCUUGCUUAUGUCCUCCACCCGUCCCUGAAAACA

>P2382

UCAUGUCUUAAAAAAAAAAAAAAAAAGAUGGGGUCUCACUUUGCUGCCCAG

>P2383

UUUCUUAUCGCUGUUGUAACAAAUUACCACACACUAAGUGGCUUAAAACAG

>P2384

UUCUACAUGGCUGGGGAGGCCUCACAGUCAUGGCGGAAGGUGAAUGAGGAG

>P2385

AGCUUGCUCAGGCGAACUCCCGUUUAUAAAACCAUCAGAUCUUGUGAGACU

>P2386

AAAACCAUCAGAUCUUGUGAGACUUAUUUUCUACCACAGAACAGAAUAGGG

>P2387

CUUAUUUUCUACCACAGAACAGAAUAGGGGAACCCACCCCCAAGAUUCAGU

>P2388

AACCAUAUCACCAGUCCGUGGAGCCAGGUGUGGUGGCUCACACUUGUAAUC

>P2389

GCCAGCUAAUUUUUAUACUUUUUGUAGAGAUGGACUCUUGCAGUGUUGCCU

>P2390

UACUUUUAUUUAAAGACAGAUUCUCACUCUACUGCUCAGGCUGGGGUGUAG

>P2391

UGGACUCAAGCAACCUUCCUGCCUCAGCCUCCCAGAGUGCUAGGAAUACAG

>P2392

GCAUCCCUCCUGUCCCUGAGCUCGAAGGCUGAAUGGUCGAUGGGAGCUCCU

>P2393

AAUGUAGGAUUCUGGCUUUGGUCUAAGGUGACAAUGUUCUACCGCUUCUUA

>P2394

GUAGAGAAAUAGGGUCUGAGGGACAAGGAGCCUGUGUGCCCGUGUCGGCAG

>P2395

CUCAGUCUCCUGAGUAGCUAGCGCUACUGGUGCAUGCCACCAUGCCCAGGU

>P2396

UACCCUGUCUUAUUGGUUUAAAUCAAAUUGCAGAAAAAUAAAAGCUCAAAU

>P2397

GCAUUUCUUAUUUCUCUGUCCUUUAAUUUUGAAGGGUCAGUAUAAAAAUAC

>P2398

GCGGGUUGAGAUGGGGUCUCGCCCUAUAGCCCAGGAUGGAGUGCAGCAGAA

>P2399

AUCUCUAUAAAAAAUAAAAAACAUUAGCCAGGUAUGGUGACGUGCACCUGU

>P2400

CACACAACCAUAGUCCCAGCUACUCAGAAGGCCACGUCAGGAGGAUUGCUC

>P2401

UCUCAUAUUGACCAGGCUGAUUUCAAACUCUUGGCUUCGAGUGAUCUUGCC

>P2402

UGGGUGGAUGGGUAGGCAGCUGGGUAGAUGUAUGAAGGAGUGAGGGAUGAG

>P2403

AUGCAGGAUGUACGCAACAUGGAAUAUUAUUCACCCCUAAAAAGGAAAGAA

>P2404

ACCUGAGGUUCUUAGUCAAAUUAAUAGAGACAGAAACUAGAAUAGUAGUCA

>P2405

AAUUAAUAGAGACAGAAACUAGAAUAGUAGUCACCAGGGGCUGGGGUCAGG

>P2406

UCACGAGCUCCUUUCACCGAGCAUAAUGCUUCUGAGGUCCAUCCAUGCUGU

>P2407

GAUUUGUUUUCUUUUCACAAUCUGAAAUUCAAAGUUCCCUGGGUAUCCUGU

>P2408

CUGUCAGGAAUAUUCUCUUUUCUAGAAUGUUAAACUCAUAGGCAGGAUUAC

>P2409

UGCCAUUUUUUUUUUUUUUUUGGCCAAGUAUAGUGGCUCACACCUGUAAUC

>P2410

GACAACAAGAGCGAAACUCUGUCUAAAAAAAAAAAAAAAAACACACACACA

>P2411

AUCCAAUGGGUCUGAAAAGGUCAACAAACCCAAAGCACAAGAGCAUGAAGA

>P2412

UGUUUGAGCCCAGCCUGUGCAGCAUAGAGAGACCCCCAACUCUACAAAAAU

>P2413

CAAAGCUGCAGUGAACUGUGAUUGCACCGCUGCAUUCCAGCUUGGGGGACA

>P2414

GCCCGCCUCGGCCUCUCAAAGCGCUAGGAUUAAUAUAAAUCUUACACAGAC

>P2415

AGUACUUUUGUAAAGUACUGAAAUUAGCCGGCCGUGAUGGUGGGUGCCUGU

>P2416

ACAGUCUUGCUCUGUCAUCCAGGCUAGAGUGCAAUGGUAUGAUUUUGGCAA

>P2417

AUGAUUUUGGCAACCUCCACCUCCCAGGUUUAAGUGAUUCUCGUUUCUCAG

>P2418

UAGCCUGAAGUGAUCCACCCUUCUCAGCCUCCCAAAGUACAAGGAUUACUA

>P2419

GGAUACAGCAGGGGGUUCAGGCAGGAGGAUCACUUGAGCCCAGAAGUUGGA

>P2420

AGUUGGAGGCCAACCUCAGCAACAUAGCAAUACGCUGUCUCUAAAAAAAUA

>P2421

GAGACCCCCCUCGUUCUCUAGAAAAAAAUUAAAAAAUAAUUAGCCAUGCAU

>P2422

AGGUGUGAGCCACCGUGCCUGGCCUAGAAUUUGUUUUGAUAGAUAUUAGAU

>P2423

CCCCAGAGCAGGGUCCCUGCCGAGGACUGGCCUAGAGCAAGCACUGGAAAA

>P2424

CUGCUCUCUUGGCCGCCUUGGAUGCAGGUUUGUCCCCAGCCUCCAUCUCCU

>P2425

UGACCUCGAUGGGAAGGAGCGUUCUACCCAUUUUCCUUGUUUUCAGUGCUG

>P2426

UGUCUUUAAACAUAGUUUGACGUCUAUAAUCCCAGCACUUUGGGAGGCCAA

>P2427

GGUAAUUAAAAAAAAUUUUGUAUGUAGAAACAGGGUCUCAUGUAACUCCUG

>P2428

AGGGUCUCAUGUAACUCCUGGGCUAAACGAUCCUCCCACUGUGGCCUCCUA

>P2429

CCGGGAAGCGGCCUCGCUGGCUCAGACGUGGCUGUGGUGCCGGGCCUCCUG

>P2430

GUGAGCAGUCCUGGGGGCCCAGGGUAUGACCCCCGUCGUCCAAACCCCGGC

>P2431

CCCGGAGUUCGAGGCCAGCCUGGUUAACACGCUGAAAUCCCAUCUCUACAA

>P2432

AACACGCUGAAAUCCCAUCUCUACAAAGAAUACGAACAUUAGCUGGGUCUG

>P2433

AACAAAGAUUAUUGAGGUUCAGGAGAAUGGCAAAACUCAAUCCAAGGAAAC

>P2434

CCAGGGAGCUGUGUUGGUCAGCGAAAUAUCGCAUCCUGGGUGGCUUAAACA

>P2435

GAAAUCAAGAGUGAGUUGCAAAUACAGGCCAUUCUUUUGGUUUUGUCCACA

>P2436

AGGCCAUUCUUUUGGUUUUGUCCACAGAUAGCUGAGUUACAGAGUAACUGU

>P2437

CCUGUCAUUGCACUCUAGCCUGGGCAACAAGAGCAAAACUCCAUCUGAAAA

>P2438

UUUGUUUUGUUUUGUUAUUUAAAAUAGGAACAGUGUCUCACUGUGUUGCCC

>P2439

ACGAGUUACUGGGUGCAGCACAGCAACAUGGCACAUGUAUACAUAUGUAAA

>P2440

GUUCUCACUCAUAGGUGGGAAUUGAACAAUGAGAACACUUGGACACAGGAA

>P2441

AGACUGGAUUAAGAAAAUGUGGCACAUAUACGCCAUGGAACACUAUGCAGC

>P2442

GUGGAAGUCAGUGUGGCAAUUCCUCAGGGAUCUAGAACUAGAAAUACCAUU

>P2443

ACUGUUUUCCACAGAGGCUACACCAAUUUGCCUUCCUGCCUACAGUAACAA

>P2444

UCCGCUUACUGAGUUAUCAUUGACAAAUAUUGUAUAAAUUUCAGGUGCACA

>P2445

CUUUUCCAUUUAACAACACCUUAGAAACAUGUUUUCUUAUCAAACCUUCGG

>P2446

UGUGGAUCCUCAGCCCUGUCUAAGGAGGACACAGAUGAGACCGCACGGGAG

>P2447

UGAGAUAGGAGAGCCAGGAGUAGGAACAGAGGAGGAUGUGUCCAGAGCCUG

>P2448

AAAAAUUAGCCAGGCAGGGCAUGGUAGCGCACACCUGAAGUCCCCCUACUU

>P2449

UGGCCCAUGCAGUAAGCCUGCACUUAGGAAGGCUGAGGCAGGAGGAUGACU

>P2450

GCUGGUUGUAGGGGUACAUGCCUGUAGUCCCAGUUGCUUGGGAGGCUGAGG

>P2451

GGCAGGUGGUGAGGAAGAGUGGGGUAGAGCCAGGCGGCACAGGCAGCAGCC

>P2452

GAAGAUCCUGCUCCUGGAAGGAGAAAGAUGUGGAGUGGAGAAAAUCUCAGC

>P2453

CUGGACUUGGAGGCAGGGUUAGGGAAGAUCCUGCUCCUGGAAGGAGAAAGA

>P2454

GAGGUCAGGGCCAUGACCGUGAGCUACACGUCCUUGGCGCCCAGUGUGAGC

>P2455

AUUGAGAAGAGUUAAAGGCUAAAGAAAACAUCUUUUCUAGCUUCAACAGAG

>P2456

CUCUUCCAAAGAACAGCAGCAACUCAAAGAAACUCACAGCAAGGCUACUCA

>P2457

UCUUUAACAUUCUGCUUUGCAUUUCACUGACUUUUUGCUCAUCUCCAACAC

>P2458

CCUUGCUCAGGAAAUAAGCCACUACAGCUUCUGCCCCUUAUGCCAUAGUCC

>P2459

UAUUAUUAAUAUUUUAAAAAGAGUUAAGUUGUAUUUUGAUAAUUCACAAAC

>P2460

AGAUGGGGUUUUGCCAUGUUGCCCAAGCUGGUCUCAGACUCAUGACCUCGA

>P2461

UAAAACUGAUCAUGUACAGUGGCUCAUGCUUGUAAUCCCAGCAUUUUGGGA

>P2462

UAGAAACUUUUUUUUAAUUUGAGGCAGGGUCUCUGUCAUCCAGGCUGGAGU

>P2463

UUCAGCCUCCUGAGGAGCUGGCAUUACAGGCGCACAACAUCAUACCCAACU

>P2464

GAAAGACUAUGAGGCUGGGCGCCUAACGCUUUGGGAGGCCGUGGCAGGUGG

>P2465

UGAGGUGGGAAGGUCACUUGAAUCCAGGAGACUGAGGCUGCAGUGAGUUGU

>P2466

CAUGGUGGCACGCACUUACAGUCCAAGCUAUUUGGGAGGCUGAGGUGGGAA

>P2467

CUCUCACCAUACGCUUCUCCACUCAACCUUUGGAGAGACACGGACCACCAA

>P2468

AUCUCAACUGCAGACAUAUAUGAUAAAAUUUGAUCAGUAUAUUAAAGAUAG

>P2469

AUGAGUUUGAACCCAGCACGCCUUUAUUCCCAGCAUUUUGGGAGGCUGAGG

>P2470

GCCAGUGUGAAACCUGGGCAUGGCAAGACACCAUCUCUACAAAAAGUACAA

>P2471

AAAACAGUAUAUUUAAUAGUACAGUAUAUUUCAUGUGUAUAUGGUUUAUAA

>P2472

ACCAUGGAUGAAUCUUGAAGACAUUAUGCUAUGUGGAAUAAGCCAGACACA

>P2473

UUUGUGCUAUUGUCAUACAUUUUUUAUUUUUUGAGACAGAGUCUCACUCUG

>P2474

ACCUCUAUAAGAAAUUUUAAAAAUUAGCCGGGCUUGGCAGCGUGCUCUUGU

>P2475

ACUAGUUACUCUGCUUUUGCAGAGUAACCUCUGCCUAGAAUGUUUUCCUUC

>P2476

GUAACAUUUGUUAAAAUGAACAUGAAGGGAAACAUUCUAGGCAGUGGUUAC

>P2477

AGCCUGGGCAACAUGGCAAAAUUUCAUCUCUACAAAAAAUACAGUAAUUAG

>P2478

GUUCUGUCGCCCAGGCAGCAGUGUAAUGGCGUGAUCACUGCAGCCUGGACC

>P2479

CAUUUGAAGUCUUGGCUAUUUGGGAAGCUGGGGAGGGAGGACUGUUUGAAC

>P2480

AAAACGUAUCUGGGCGUAUUGGUGCACAUUUGAAGUCUUGGCUAUUUGGGA

>P2481

GUUGAGACCCUGUCUCUACAAAAAAAAUAAAAAUUAGCUAGGCCUGCUGGU

>P2482

AGAAAAUCCUUUAAAAAAAACCGGAAUCUUCCUCUGUCACUCAGGCUGGAG

>P2483

CUCCAGCCUGGGCAACAAGAGUGAAACUCCGUCUCAAAAAUAAAUAAAUAA

>P2484

AAAAAUAUGACCAGAGGCAGAGUGCAGUGGCUCACACUUGUAAUCUCAAUA

>P2485

UAGUUUCAGUCUUUUUUUUAAGACAAGUUCUCACUCUGUCAUCCAGGCUGG

>P2486

UGCUGCACCCAUCAACCCAUCAUCUACAUUAGGUAUUUGUCCUAAUGCUAU

>P2487

GUGUUUUCGCUUUUUUUUUAUUAUUAUGCUUUAAGUUCUAAGAUACAUGUG

>P2488

AUAAUUCCAGCUCUUUGGCAGACCAAGGUAGGAGGAUUGCUUGAGGUCAAG

>P2489

CACGUUGAAGCUAUUCUCAUGCCUCAGCACACAGAGUAGCUGGGAUUACAG

>P2490

UUGGGUGCAGUGGUGCUUGUCUGUAAUUCCAGCACUUUGGGAGGCGGAGGU

>P2491

CACUGAGUUAAUUUUUUGUAUUUUUAGUGGAGACACGCUUUCGCCUGGCUU

>P2492

GGGCUGAGGUGGGAGGAUUGAGGCCAGGCCUUUGAGACCAGCCAGGACAAC

>P2493

AAAGAAAAAAAAUUUAGAUGGCUUGAGCCUGGUAGGCAGAGUUUGUAGUGA

>P2494

AGUACUGGGAUUACAGAUGUGAGCCACUGUGCCUGGCCCCGAGUAGUUUUU

>P2495

UGAGUCAGUUCUUACUCUAUUAGUUACCGUGAGAUCUGAUUGUUAAAAAGA

>P2496

UUCAGGCUGCAGCAAGAUAUGAUCAAAUCUGUGCCUGGGCAACACAGCAAG

>P2497

GAUCUGAAGCAGAAGGGAUUAUGUUAGUUGGGAAGGUUUCCAACAGAGGCC

>P2498

UGGGAGGCUGAAACUGGAGAAUUGCAUGAACCUGGGAGGCGGAGACUGGAG

>P2499

AAUCUGUGUGGCUCUUAUGGGGUUAAUUUGAUUUGGACCUGUAUUAAUUUC

>P2500

UUAUUGGCUUAAAUUCUAAAUAACUAGAAACUGUAUAAUAGGCAAAACUGU

>P2501

AUAGAUGAAGGGAAUCACAGUUGAUAGUUAUAUGGUGACAUUAUUGGCUUA

>P2502

UUUUACCUGUUACAUGGUUUUCAGUAAUUUAGAAUUUAAGCCAGUAGUGGG

>P2503

GAGGGGUACUUUGUCUCACACUUUUACCUGUUACAUGGUUUUCAGUAAUUU

>P2504

ACAAGUCUCAGUCAGAUGAACCCCAAGAGCCACAUGUAUUUGAGGGGUACU

>P2505

UUCUGAUGCCAUUCAAGUAAAUACAAGUCUCAGUCAGAUGAACCCCAAGAG

>P2506

CUCACUCUGUCACCCAGGCCAGAGUACAGUGGUACGAUCACAGCUCCUUGU

>P2507

ACCAUGUUGACCCAGCUGGUUUUGAACUCCUGACCUCAGGUGAUUCACUUG

>P2508

AGCCAUGAUCAUGUUACUGCAUUCCAGCCUGGGUGACAGAGUGAGAUCAUA

>P2509

UAAAAAGACAAAAGCUGGACGCAGUAGCUAAUGCCUGUUAUCCCAGCAUUU

>P2510

GCAAAACUGAAACUUUACACCCGUUAAAGAACUCCUAUUCUCCUCUCCCUA

>P2511

AUAUCUCUUUAUUUUUAAUUGUGGUAAAAUAUACAAACAUAAAAUUUACCA

>P2512

GGCAUAAAAAUGGUUAAAAGGGUAAAUUUUAUGUUAUGUCUAUUUUACCAC

>P2513

AUGCCACUGAAUUGUAGGCAUAAAAAUGGUUAAAAGGGUAAAUUUUAUGUU

>P2514

GUUAUACAACAUUGUGAAGGUAGUUAAUGCCACUGAAUUGUAGGCAUAAAA

>P2515

AAUAGAUAGUGGUGAUGGUUAUACAACAUUGUGAAGGUAGUUAAUGCCACU

>P2516

GUGAUGAAAAGUUCUGGAAAUAGAUAGUGGUGAUGGUUAUACAACAUUGUG

>P2517

AUAGCUUCUGUUUGGGGUGAUGAAAAGUUCUGGAAAUAGAUAGUGGUGAUG

>P2518

UGGGAACUUCUUGUUUAAUGGGUAUAGCUUCUGUUUGGGGUGAUGAAAAGU

>P2519

GGACAAAUACUGUUUGAUUCCACUUAUAUGAGGUACCUAGAACAGGCAAAU

>P2520

AACGGAAUAUUAUUCAGCCUUAAAAAGGAAAGGAAUUCUGACAUAUGCUAC

>P2521

UCAUAAAUGAAUGCAUGAACAAAAUAUGGUAUAUAUACACAACGGAAUAUU

>P2522

UUUUUCACUUAGCAACAUGCAUUUAAGAUUCAUCUAUGUCUUCCUGUGGUU

>P2523

UAGCCUUUUAAGACUGGCUUUUUUCACUUAGCAACAUGCAUUUAAGAUUCA

>P2524

AUAAGAGUUUUGCCUUUUCCAGGAUAUUACUUAAAUGGAAUUAUACCUUAU

>P2525

CCCAUGUCUAUACACAUAUAUCUACAAAUCCUUCUCUUUGUAACUAUCUGU

>P2526

ACCCUGCAAGCACAUUAUCAAGUUAAAAGGUUUUGCACAUCAAAAAAGUAG

>P2527

UGGUUGCCUGUGCUUGUAGGGUAUUACUGAAGAAAUCUUUUCCCAGACCAG

>P2528

AAGUUUGUUUUCUGGUCUGUGAAUAAGUACUUCACAGGUGUUUGAAAAGUG

>P2529

AAAAACAAAAAAAUUAGCCCCAGCUACUCAGGAGGCUGAGGCAGGAGGAUC

>P2530

ACCACCACAGUGGAGUUUAGGUUUCAGCAUGAAUUUGGGAGGGGCACAAAC

>P2531

CCAAGCCUGGGAGGCAGAGGUUGCAAUGAGCUGAGAUGGCGCUGCUGUACU

>P2532

AGUUUGAGAACAGCCUAGGCCACAUAGUGAGACCCUGUCUCUACAAGGGAA

>P2533

UAUUUUGUAGAAACAAGGUCUCACUACGUUGCCCAGGAUGAUCUCAAACUC

>P2534

GAGUUCUGAUCAGGCAUACUGGCUCACGCCAGUAAUCCCAGAACAUUGGGA

>P2535

CAAAGUGUAUAAUUCAGUGGUUUUUAGUAUAGGCACAGUUGUACAGCCAUU

>P2536

GAGAAAGCUGCCGUCUAGUGAACAGAGCAGCAUUGGAGGGUGUGCCUGCUC

>P2537

GCCAGGUGUGGUGGUUCACAUCUGCAGUUCCAGCUGUUCGAGAGGCCAAGG

>P2538

AGUUAUCUUGAAUCUGUGUGUCCAAAUAAUAUUGUCUCAAAGCACAUAAAG

>P2539

GAGGUGAGAGGAUCCUUUAAGACCAAGAGUGCAAGACCAGCCUACCAACAU

>P2540

UGUGAAAGCACAGUAAGGAAAGUGAAUAACAAAACACUGUAAGUAUUUGCA

>P2541

AUUCUCCUGCCUCAGCCUCCUGAGUAGAUGGAAUUAUAGGCAUGCACCUGG

>P2542

UAAUUUUUAGUAAGGCUAGCUCUAAAUGUAGUUAUUUUCGGCUCUUCUAUC

>P2543

GUAAUGAUGUCAGUCAAAUUCAAUGAGUGGAAAUUUAAGCACAUUAUCUUC

>P2544

GGAAUAAUUGAAAACAUACUGAGUUAAGAGUCUGCAAGAAAAGCUAAUGCG

>P2545

UAACUUCUUUCUUCGUAAGUUAUAUAUGUCUACUUCUACCUGUGUUAUCAU

>P2546

UCAGGGCAUAGUGGCACACACCUAUAGUCCAUGCUCCUUGGAAGGCUGAAG

>P2547

CAGUUUAAGAGUUUGGGUCCAGCCUAGGAAACACAGUGAGACUCUUGUCUU

>P2548

GAUGACUUACUGGGCUAGGUGUAGUAGCUCAUGUCUGUAAUCCUAGAACUU

>P2549

UAUUGUUGAGACAGAGUCUCAGGCCAGAGUGCAGUGGCGCGAUCUCUGCUC

>P2550

AUUUAUUAUAAUGUUCUAGGAUUACAGGUGUGCGACACCAUGCCUGGCUAA

>P2551

UUACUCUGUUUUACUUUUAACAAAUACAUUCUGAUCUGCCACAGUGCAUAU

>P2552

CCUGGGCAAUAUAACUGUCUCUACUAAAACUACAAAAAUUAGCCAGGCAUG

>P2553

CUUUCAAAUUAGUUACCAAAUCAUAAACAGAGUGGAAUAAAUAUAAAUGAG

>P2554

AUGUACAUGGAGAGCAGGGCAUGGUAGCUCAUACCUGUAAUCCCAGCACUU

>P2555

CAGCAAGAUAAUGAACAGGCCAGGCAUGGUAGCUCACUCUUGUAAUUCCAG

>P2556

AGAAAACCAAAUACCACGUGUUCUCACGUAUAAGAGAGAGCUAAAUAAACA

>P2557

UUUCGUAUGUUUUUGCAGUGGCUGGAACUGGUUUUUCCUUUGCAUAUUUCA

>P2558

AACCAUAUACUUUAGAAGAACAGAAAAACCUCACAGUCUGUCCUGAUGGAG

>P2559

CGGCAGAACCCUGUCUCUACAAAAAAUAGAAAAAUCAGCUGGGCAUUGGAA

>P2560

AGCAAACAAUUUGAGACAGAGUGUCACCCUGUCGCCCAGGCUGGAGUGCAG

>P2561

UUUUUUUACCCCCAGAGUGGGUGUCACUCUGUCAUCCAGGCUGUUGAAUGC

>P2562

GUCUAGCUUUUGUGACAUUUUCUUAAGUAAUACAGUGGAACCCCUUAUCCU

>P2563

AUUACAGGCGUGUGUCACCACACCCAGCUAAAUUUUUGUUGUUGUUGUUUU

>P2564

CGAUCCUCCUGCGUUGCACUCCCAAAGUGCAGGGGUUAUAGCUGUGAGCCA

>P2565

GACAAAAGAUGACAGCUAUCAGCAUAGUUUGGAUGACAGAAAAGGGACAAA

>P2566

AGAUGGAGCCUCCCGCAGUAGAGAUAGAGUUUCACUAUGUUGGCCAGGCUG

>P2567

UUAUUGUGUAAUGACUAACCAUUUUAAAGAUGAACUCUUAUUCUCAGCCAA

>P2568

UGUCAUCCCCAUCCUGCUACCAAUGACUUUCUUCACAGAAUUGGAAAAAAC

>P2569

GAAAAUGGCCAUAGUGCCCAAGGUAAUUUAUAGAUUCAAUGUCAUCCCCAU

>P2570

GGUAGGAAGAAUCAAUAUCGUGAAAAUGGCCAUAGUGCCCAAGGUAAUUUA

>P2571

AUAAAAGAGGAUACAAACAAAUGGAAGAAUGUUCCGUGCUCAUGGGUAGGA

>P2572

GAACUACGAACCACUGCUCAAUGAAAUAAAAGAGGAUACAAACAAAUGGAA

>P2573

ACAAGGGACGUGAAGGACCUCUUCAAGGAGAACUACGAACCACUGCUCAAU

>P2574

AAAAUACCUAGGAAUCCAACUUACAAGGGACGUGAAGGACCUCUUCAAGGA

>P2575

UUGCUUCAAAGAGAAUAAAAUACCUAGGAAUCCAACUUACAAGGGACGUGA

>P2576

GGAACUCCCAUUCACAAUUGCUUCAAAGAGAAUAAAAUACCUAGGAAUCCA

>P2577

AAACAGAGAGCCAAAUCAUGAGGGAACUCCCAUUCACAAUUGCUUCAAAGA

>P2578

GAGUUGAGUGUUUGUUUUCAAAAUAAAGUUUUAUUAUUUCAAAAACAUGCA

>P2579

UAAAUAAUGUGUACAUAUGGACAUAAAGUAUGGAAUAAUGGACACUGGAGA

>P2580

UGAAAUACCCGGCCCAAUAAACAAGACAACAGAGCGACAUCUAGCUAUCAA

>P2581

CCUGGUUUUAGCUCCUUUUUGGUUCAGGUGUGCUGGUGUCUCUAAGCCUUU

>P2582

CUGCAUAUGUGCUUUCUAAGUAUUUAGAAGAUUAGAUUUCUAAUAAACAUG

>P2583

GCAUGCAUACAUUGGUUAGAUAACUAAUCUAUGCCUGGAGGCAGGAGCAGU

>P2584

ACGUCAUGGAGGCAAGAGCAUGCAUACAUUGGUUAGAUAACUAAUCUAUGC

>P2585

GUUAUUUGAUAUAGGAUUCAAAGGGAUGAACGUCAUGGAGGCAAGAGCAUG

>P2586

CUGCUCCUGCCUCCAUGCAUAGAUCAGUUAUCUAACCAGUGUAUGCAUGCU

>P2587

CAUUUUUGUGCAUCCUACAGUGAGGAGUAACUGAUCAGGUGUCUAUAACAU

>P2588

AGCAGAAGAGGUGAUGUCAUCCUGGAAAACUGGGUAGUUCAUCUGACCAUG

>P2589

GUUUGUAAAUAGGUUUCAUGAAGAGAGAAAAACCAAGCUCAGGUAUCAUCA

>P2590

GAAGAAAAAGCACGUCAUUACUUGGAGCACUUCAGAGCCAAGCUAUUAAGU

>P2591

UUCAUAACACUUUCUAGAUUAAUGGAAACAUUCAUUUUAGACACCGAACAG

>P2592

GGAAAGCUGGCAAAUAAUAUCCAGGAAUUAUUAUAUAGUGCCUCAGAUAUA

>P2593

UCAUUAUAGUGAAACAUUUCCUUCAAUGUUUUUUUCAAUAACACUGAAGGA

>P2594

ACAGUAAGUCUUCAAUGUUAUGGAUAGGUCUUGGAAACUGUGACUUUAAGU

>P2595

AUACAGGCUGGGCACGGUGGAUUACACCUGUAAUCCUAUCACUUUGGGAGG

>P2596

GAAAUAUGUAUAUAAGUUCACCAAAACACAGCAGAAGAAUGUUCCUGGCAG

>P2597

ACAGUGACUUAAAUAGACCACUGGAAGAUGACUGUCAAGUUUUAGAAGAGG

>P2598

GUGUGCUUAAUGAUGUAGUUGAUUUACUUUCUAGCAAAAUUCUUUAUUUUA

>P2599

UUUGUCUUUUUAUUAGAGAGAGAAGAUUCAUGAGAGAUGCAAGAAUAUUUG

>P2600

CAUCUCUAGUUACAGAUCAACAUAGAUGGACUGUAUAUCAUUCCAAAGUAA

>P2601

AUCCAUGUUUUCUCAAAUUAUAAAUAGACUUUUUUUAAAAAAGUUCAUUUA

>P2602

AAUUGUUAACAAUGUGUACAAGGCCAGGCAUGGCAGCAUGCACCUGUAGUC

>P2603

AGAUUGGUUAAACUUCUUUUUUUCAAAGAGACAGGGUCUCACUUUGUUGCC

>P2604

AAAAAUACAAAAAAUUAUUGGUCGUAGUGGCACGUGCCUGUAGUCUCAGCU

>P2605

AGAUAAACAUCUACCGUAAGAUCCAACUAUUCCACUCCUAGGUAUUUACCC

>P2606

GCCAACAUGGCAAAACCCUGUCUCUACUAAGCAUAGAAAAAUUAAUUGGGU

>P2607

CACCUGAGAUCAGGAGUUCAAGACAAGGCUGACCAACAUGGCAAAACCCCG

>P2608

UAAAAAUAAAGCUGCCAUUGUUGCAAUUAGAAGUUUGAGAGGUUAGAAUAG

>P2609

AAAAAUAAUAGGCGCAGUGGUUCACACCUAUAAUCCUAGCACUUUGGGAGG

>P2610

CAGCUGCUAUUCAUUGAAAUGUUACAAUUUUCCUGCCUUAGUAUUUCACAC

>P2611

UUGCCCAGGAUGGCCUUCAGCUCCUAGCCUUAAGCAGUCCUCCUGUCUCGA

>P2612

UCUACCAAAAAAAUAAAAAAUAAAAAUUAGCCGCGUGUGGUAGCGCACACC

>P2613

UUGAUUUAUUCCUCAGAAAAUCCCCACACUACUGGAGACCCAGGCCUGGAG

>P2614

GAGUUGGUUAUUAUUGGAAUGUUAGAUCUAUGUUAACUGAAAUAACAGUUG

>P2615

UGAAUAGCCACUGCACUCCAGCCUGAGCAACAUGGUGAAAUCCCAUCUCAA

>P2616

GGUCAAAGAAAGAUUCUGAGGGAAUAGAAAUGGGAGUCGCAGGUUCUGUAA

>P2617

CAGUAACCAACUGAGAAAUAGAGUCAUUGAAGUGUAUGUAUAAAAAUAAAA

>P2618

UUAUGGCAAAACCGGGUCUCUACUAAAAAUACAAGAACUAGCCGGGCGUGG

>P2619

UUGUGCAAUGGCCUAGCCUAGUAGAAAUGGGGGAAAAGCAUUGCUGUGGAC

>P2620

AGUUAUUUUUAUUAAAAGCAAUAUGAUAGCGAGUGGCACGUUUGCAAUGGG

>P2621

UAGCAUCCUGCCUCAGCCUGGGAUUACAGGGGUGAACCACUGCACCUGGCC

>P2622

GAACAUGGGGGUUUGUUUUAGAGACAGAAUCUUGCUUUGUUGUUCAGGCUA

>P2623

UUUGCUUAGCAUGUCUUUGAGAUCCAUCCCUGUUGCUGCUUGAAUCAGCAU

>P2624

AUAAGAAUAUAGAAGAAAAUCUGCUAGGUGCAGUGGUUCAUGCCUGUAAUC

>P2625

CACUAUACUCCAGCCUGGGCCACAGAGUGAUCCUGCCUCAACAAACAAGGA

>P2626

UCUCAGCAACUUGGGAGGCUUAAGUAGUAGGAUCGCUUGAGCCUGGGAGGU

>P2627

GUGGCUUGUGCCUGUAGUCUCAGCAACUUGGGAGGCUUAAGUAGUAGGAUC

>P2628

AAAGAACGAUCAAGGUGCCCCACUCAGUGGACUGCUUGCAGGGCAUUCUCA

>P2629

CUUAAGAACUGUUUAUUGGGAGUUUAGGCCGGGUGCAGUGGCUUAUGCCUG

>P2630

GAGGCAGGGUCUCCCUGUAUUGCCCAGGCUGAUCUCAAACUCCUGCCCAUG

>P2631

GAUCAUGCCACUGCCCUCCAGCUUAAGUGACAGAGACCCAUCUUAAACAAA

>P2632

UAGUUAGCCAGGUGUGGGGGUGCACACCUGUGCUACCAGCUCUUUGGGAGG

>P2633

CCCUACAAAAAAAAUGUUGUUAGUUAGCCAGGUGUGGGGGUGCACACCUGU

>P2634

GCAGUGGGACGAGCCUGGGAAGCAUAGGAAGACCCCAUCCCUACAAAAAAA

>P2635

CCAUGCCCAGCCCAGAUUCUUCUUUAGAAGGCCCAUGUACAUCCAGCUGCA

>P2636

CUUGCUCUAUUAUCACUGGAGUACAAUGGCGUGAAUGUGGCUCACUGCAGU

>P2637

UUUUUUUGAGACAAGGUCUUGCUCUAUUAUCACUGGAGUACAAUGGCGUGA

>P2638

AUACUGUUUUCAAUAGAAGUGUCUGAAAUAAGCAUUCUUGUCUUAUUACUA

>P2639

UCUGUCUCCUGGGCUUGAGUGCAAUAGCAUGAUUUCGGCUCAUGCAACCUC

>P2640

AUCCUCCCUCCCCUCCUCAGUGGCUAGUACUACAGGUACCCACCACCAUGC

>P2641

AAUAUUUGUGACAUACUUUGAUACUAAGACAUUAUUUGUUGUUUAUCUGAA

>P2642

UACAGAACGAAUAAUUUUUAGUAUAAGUAUUUCCAUGCAAUAUUUGUGACA

>P2643

UUUUUUCCUUCAGAGAGAGGGUCUCACUGUGUCACCCAUGCUGGAGUGCAG

>P2644

CUGCAAAGUCCAAUAUUAAGAUGUCAGCAGAUUCAGUGUCUGAUAAAGUCC

>P2645

UUUCUCACAGUUCUGGAGGCUGCAAAGUCCAAUAUUAAGAUGUCAGCAGAU

>P2646

GAUACUGUAGACUGAGAAAUUUAUAAACAACAGAAAUUUGUUUCUCACAGU

>P2647

UUAGUCCAUUCCUGCUCCCAUAACAAGAUACUGUAGACUGAGAAAUUUAUA

>P2648

UGCAGAAAAUUAUGUCAUUUUGUCUAGCUUUAUCCUGAUACCAAAGCCAGA

>P2649

CAGAACUGUGAGAAUUCUUUUGUUUAUAAGCUACCAGUCUAUGGGAUUUUG

>P2650

UCGCACUAGGAAAUAGGCCUUCAUCAGACACCGAAUCUGCUGAUACCUUGA

>P2651

AAUGGGAUUAGUAUACUUAUAAAAGAGACUUCAGAGAGCUGCCUUGUUCCU

>P2652

CACCCAGCUUGUCCUGCAGCCCAGCAGGAGACUUCAUUUGGGAGUCAGACA

>P2653

AGGCUACAAACCUGUACAGCAUGGUACUCUACUGAAUACUGUAGGCAAUUG

>P2654

UGGUAUAGCCUAUUGCUCCUAGGCUACAAACCUGUACAGCAUGGUACUCUA

>P2655

UACACAAACCUAGAUGGUAGAGUCUACUCCACACCUAGGCUGUAUGGUAUA

>P2656

AACAUCAUAGAGUGCACUUACACAAACCUAGAUGGUAGAGUCUACUCCACA

>P2657

GAUUUUGUCAUUACGGGAACAUCAUAGAGUGCACUUACACAAACCUAGAUG

>P2658

GGAUAUGUCCGAGAAAUACGACAUUAGGUGAUUUUGUCAUUACGGGAACAU

>P2659

UUUGGCCUCUCAAAUCUCUGGGAUUACAGGUGUGAGCCACUGUGUGUGGCC

>P2660

AGGCUGGUCUCAAACUCCUGGCCUCAAGCAAUUCUCUUACUUUGGCCUCUC

>P2661

GCGCUGGUAGGGAGUCAGAAGGAGCACAGCGAAGUGCCAACACCUAAGAGA

>P2662

GAAGGUUGAAGUAGGCUGAGGUCACACCACUGCACUCCAGCCUGAGCAAGA

>P2663

UGCCAUUCAUCCAGACAGCCCAUCCACCAGCCACACACAAUUGUAGGCAAA

>P2664

AUCAACUGAAUUAGAAUACACCAUUAUUACCAAUGGCCUACUGGGGAAACA

>P2665

UUCCUUUGGUUAGACAUCCAGCAGUAGGAUUGCUGCAUCCAAUGGUAGUUC

>P2666

UGAUAAAUUUUAAGGUGAUUUAUAUACUAAUUACCCUGAUUUGGUCAUUAU

>P2667

AAAAAUCUAAAAAUGGUACUACUAUAGGAUCCAGCAAUCCCACGACUGGGU

>P2668

UGAAAUUAGUAUAGCCAUUAUGGAAAGCAGUAUGGAGGUACCUCAAAGAUC

>P2669

GUAUACAAUGUGUAAUGAUUGAAUCAGGGUAAUUAGCAUACCUAUCACCUC

>P2670

CAUUGCCCAGGCUGGUCUCGAACUCAUGAGCUCAAGCAGUCUACCCACCUU

>P2671

CUUUCUCUAAAGUCUUGGAAAGUUUAUUAAUCUUAAGUGGGCCCUGGUACA

>P2672

UAAGUGACCUGAAUCCUGAGAUACUAUAGCACUGGGAAGUCAAAGAGAUGA

>P2673

UAAGCAAAGCUGCUAUGCACAGGGAAAAAAUCUUUUCAUCAGGCAAUCCUC

>P2674

UGUGCCCUAGUCUGGCAAAAGAGCAAGACUCCGUCAGAAAAAAAAAAAAAA

>P2675

GAGGCGGAGGUUGCAGUGAGCAGAAAUCACAGCACUGUGCCCUAGUCUGGC

>P2676

GACCUGGUCACCUGUCUGGAGCAAAAAAAAGAGCCCUACAAUGUGAAGAUA

>P2677

CUACAGAAACCUGGUCUCCCUGGGUAUUGCUAUCUCUAACCCAGACCUGGU

>P2678

UUUAUUUAUUUAUUUAUUUUAUUUUAUUUUUGAGACAGAGUCUUGCUCUGU

>P2679

AAAUCCAUGUUUUGUUUUUUGAGACAAGAUCUUGCUCUGUUGCUCAGUCUA

>P2680

GCAUGCCUUGGCCUCCCAAAGUGCUAGGAUUAUAGGCUCACGCCUAUAUUA

>P2681

AAAAAAAAAAAAAGAGAGAGAGAGAAGGGGUUUCUCCACGUUGUCCAGGCU

>P2682

CAAUUUCCUUAGAAAAGUUGAUGAAACUAAAGACUGAAGUUCAAAGAAAAA

>P2683

AUGUGCAAGAUACUAUACAUUUGAUAGAAUCUGGAUUUGUUCCAAAAUCAA

>P2684

GAGAGGCUAAGGCAGGAAGAUCACUAUGAUUGUGCCACUGCACUCCAGCCU

>P2685

AUGGUAGCACAUGCCUUGAGAGGCUAAGGCAGGAAGAUCACUAUGAUUGUG

>P2686

AAUUUAAAAAUUAGCAAGGCAUGGUAGCACAUGCCUUGAGAGGCUAAGGCA

>P2687

UUAAAAGCAUCCUUUCAACCAGGCAAGGUGGCUCACACUUGUAAUCCUACC

>P2688

CUCAUCAAAUGUAUUUAAAUAACAAAAGCUCAACCAAAAAGAAAGAAAUAU

>P2689

UAAUAAGUAAUAAACUCAAAAAGACACAUUCUAUAGGAAUAAGGGCUUCAC

>P2690

GUUGUUUUUAGAGUCUGUAAUAAGUAAUAAACUCAAAAAGACACAUUCUAU

>P2691

GUUGUUGCUGAUUAUGCAUGAUACAAAAAAGUGGAAAAAUACAUUUUUUAG

>P2692

AUCUCAAUUGCAAGAUUUUCUCUGCAUCGGUCAGGUUAGUGAUAUUAACAG

>P2693

UUCAAAAAGUCAGAUGAGUAAGGGAAAGAAAAUUAGUUCAUCUUAAGGAAC

>P2694

UAAAUUUGGAGCGUUUUUGUGUUUGAGAUAUUAGCUCAGGUCAAUUCCAAA

>P2695

UAAUGCUUUUUCUUAUAAGGUUUUAAAUUUGGAGCGUUUUUGUGUUUGAGA

>P2696

GGGUUUGAGAGGAGAAUAGGAUUCAAGGGGGAAAUGGGGAAGAAAUAGCUA

>P2697

UUAAAAAGCUGAGCAAAUAGACGAAACAUUGGGUUUGAGAGGAGAAUAGGA

>P2698

AGAAGUAAAUAGCAGGACACCAGAAAAUGAACUUAAAAAGCUGAGCAAAUA

>P2699

CAUCUGUCUCAAUUAUUCGUGAGAUAUUAGUGCCAUUGUAUCAUUUGUGCA

>P2700

UAAUUUUUAAGAUUUCUAAAUCAAAAUAACUUCAUUGGGGGAAAGAGGCUU

>P2701

GGAGUUUUUGUAUAGGAGGGAAGUUAAGAGGAACCAUUGUGUGUACACUUU

>P2702

GUGAGAAUAACUUGUCUAAGUAAUAACUUCAGAAAUGUCCUGGGGAACAUA

>P2703

CCUCCUCUAUCUCCCUACUUCCUGGAUAAUGGGGCAGCCUUGAUCCUUGGG

>P2704

GCAACUUACUAUUGUCAACAGGCUAACAGUUUCCCUCCCACAGUGUUACCA

>P2705

CAGCAGAAACCAGGGAAAGCCCCUAAGCUCCUGAUCUAUGCUGCAUCCAGU

>P2706

CACUUGUCGGGCGAGUCAGGGUAUUAGCAGCUGGUUAGCCUGGUAUCAGCA

>P2707

CUGAUCUAUGCUGCAUCCAGUUUGCAAAGUGGGGUCCCAUCAAGGUUCAGU

>P2708

CAUCCUCCCUGUCUGCAUCUGUAGGAGACAGAGUCACCAUCACUUGCCGGG

>P2709

AUGUUGUUUUACUCACUGGUUAUACAAUGGGAGGGAGACAGAGAAAGAGAG

>P2710

AGGCUCAGUCUGCGGCCAAGACUCCAUCCUGCAAGGUAAGGUCCCCUCCAG

>P2711

GCACAGCGCGGGCGCAGGCGCCGAGAGGCGCACGGGAGACCUCAGGCCCAG

>P2712

UUGCUACAAAGAGCCUGUUUUUGUCAGUCUUAAGGUCUCUGUUUUAAUGUU

>P2713

AUCAGACUUAAGAGAAAUUUAUUUUAGGAUAAAAUACUUAGAUUUCUCUCA

>P2714

GAGGAUUUAUCUUGUAGGAAGUGCAAAGGAUUUUUGGAGGGCAGGACACUG

>P2715

GGCAGCCUAAAGAGGACAUUACCAAACACGGGAGUGAGGAUUUAUCUUGUA

>P2716

CAAGGUGGCUCUUGUCAGGAGCAGCAGCAGGUUGGGGCAGCCUAAAGAGGA

>P2717

CCAAAAAUCCUUUGCACGGCCUACAAGAUAAAUCCUCACCCCCCGUGUGUG

>P2718

UCCUCUUCUCAUGUAUCCUUGCCAUAUUAACCAUACUUACCAGUUUGAACC

>P2719

GGUGAUCUAUAUAAAAUAAAAGGCCAGGCACAGUGGCUUAUGCCUGUAAUC

>P2720

GGUAGAGGGAGGAAUGAUGGAGGGUAGGAGAGCCUUGAGCCUGCGAGAAGA

>P2721

UAUCAGCAGAAGGAGCUUUGGGGCUAAGACUAUGGGGUUUUCUAGAUAUAG

>P2722

UUUUUGCACAUUGAUUUUGUAUGCUAAGACUUUGCUAAAGUUUAUCAGCAG

>P2723

UUUAUUUUUUCUGUGUGUAGCAAUUAUGAAUGGGAUUAUGUUCUUCAUUUG

>P2724

GCAGUAUAGCCAUUUUAAUGAUGUUAAUUCUUCCUAUCCAUGAGCAUGAAA

>P2725

AUCUGUAAAUUACUUUGGGCAGUAUAGCCAUUUUAAUGAUGUUAAUUCUUC

>P2726

AUGCCAUUGGUAAUUUAAUAGGGAUAACUUGCAUCUGUAAAUUACUUUGGG

>P2727

UGAAAUAGUUUGCUCUAGUUCUGUGAGGAAUGCCAUUGGUAAUUUAAUAGG

>P2728

UUUGGUUCCAUAUGAAUUUUGAAAUAGUUUGCUCUAGUUCUGUGAGGAAUG

>P2729

UUUGGUUACUGUAGCCCUGUAGUAUAGUUUGAAGUUGGGUAACAUGAUGCC

>P2730

GUACCAGUAUCAUGCUGUUUUGGUUACUGUAGCCCUGUAGUAUAGUUUGAA

>P2731

CAGCCAUAUUCUACUAGAAGUACAAAGAAGAGCUGGUACCAUUUUUACAGG

>P2732

CAUUUGACCCAGCAAUCCCAUUACUAGGUUUAUAUCCAAAGAAAUCUAAAU

>P2733

GAACAUGCUCCUUUAGCUCUGUGAAAUUUGUUAUUACCCACCUUCUGAACC

>P2734

GGUGGAGAAAUCAAUGCAUUGGGUUAGAACAUGCUCCUUUAGCUCUGUGAA

>P2735

CUAAAGGAGCACGUUCUAACCCAACACAUUGGAACGAAUCCCAGAACUUAA

>P2736

GUAGUAGCAAACUUCACUGAGCUAAAGGAGCACGUUCUAACCCAACACAUU

>P2737

AAAGCAUAGCCAGACUGCUUCUUUAAGCAGGUCCCGGAUUCAUUCCUCCUC

>P2738

CAGCACAGCUGCUGUACAAAAGCAUAGCCAGACUGCUUCUUUAAGCAGGUC

>P2739

UAGCCAUUCCAACCUUUGGGCUUUAAGGAGUCCCAGCUGACUUAGGGUGGA

>P2740

GGACAGAGGGAUCCAUUAGCACAGCACAGUGCUACUCUACCAAAAUAUGGC

>P2741

AGAGCCGUGCACCCUACCUGCUUCUAGUCAGUCAUCUUGGCCACACACCCC

>P2742

UGACUUGCUCCUUUCAUUCCUCUCCAUGAGAGCCGUGCACCCUACCUGCUU

>P2743

CCCAACAGGCUGGAAUAGCUAAGUUACCCAAGCAACAAAGAUGGUGGCCUG

>P2744

CUGUUCUGGGGAUCCCCUCCACCCUAGUCACUUUGGACUCUCCAAAGCCCA

>P2745

GUCAGCUCUAGAACUAAGAGGUGGAAGCCCCACUUGGGAGGUCUCACCCAA

>P2746

UGGAAUCCAUACCUACCGGGGCAGAAGGGAUCUCCCAGCACAACAAAGCUA

>P2747

AGCUGCCAUCUUUGCUGUUUGUGCAACUUAGCCACUCCAGCCUGAGGGCUU

>P2748

GGAAGGGAUCCCCCAGCACAUCACAACUGCUCUACCAAAUAGUGGCCAGGC

>P2749

UCACAGCUUAGUUUACUGUUUAAUGAAAGUUGCCUAGCUUUCAGCCUAUCU

>P2750

GAAACAGGGUCUCCCACUGUUACCCAGGCUAGUGUGCAGUGGUACAAUCAC

>P2751

UAGUGCCUUAAGAAGCUCUUUUCUCACGCCUGUAAUCCCAGUACUUUGGGA

>P2752

CUGCAAUGGACUGAGAUGGGCCAGAAAUCGUGAUAUCUUGUAUAGGGCAGG

>P2753

AAGACCAGCUGCAGGCUGCCACUGCAAUGGACUGAGAUGGGCCAGAAAUCG

>P2754

GUUGAGUAUAUUAUAGUGCAGGAGUAGAACUUACAAGACCAGCUGCAGGCU

>P2755

GAUCAUUUUGGAUUUUUUGUUGAGUAUAUUAUAGUGCAGGAGUAGAACUUA

>P2756

UAGAGAUUCAAUUUAUACUUUAAAAAGAUCAUUUUGGAUUUUUUGUUGAGU

>P2757

GGUGGACACAGUGGUUCACGCCUGUAAUCCCAACACCUUGGGAGGCUGAGA

>P2758

CAGCUCACUACAGCCUUAACCUCUAAGGCUCAAGCAAUUUUCCCACCUGAG

>P2759

UUGAGAGAAGCUCUCACUCUGUCACAGCUCACUACAGCCUUAACCUCUAAG

>P2760

AAGACUCUGUCUCUAAAAAAAUACAAAAACAUGCUGGGUAUGGUGGCACAU

>P2761

UUUGCCAUGAGAGUACACCGAACAAAGGAGACCGGGUCAUUUAUAACCUGA

>P2762

AGGCCAUUUUUAUACUUUCUGCAGAAAGGGUACACUCGCCAGCAGUUUUGC

>P2763

CUGUAAAGAAGUAGCACUUGAACAUAAAUUUAAUUUCCUCAGCAAGGCCAU

>P2764

GGAGUGAGACUCUAUCCAAAAAAAAAAAAAAAAAAAAAGAUGUGGAGGAGA

>P2765

AGGGGAGGCCAGAUGUAGUGGCUCAAGCCUGUGAUUGCAGCACUUUGGGAG

>P2766

AAGACCUUCCAGUGGGAAAAGAUGUAGGGGAGGCCAGAUGUAGUGGCUCAA

>P2767

CCUAGGAGAUGACAACUUCAUGCAUAUAAUUGCCCUUGAAGACCUUCCAGU

>P2768

CAGAAGAUGGCAUUGUUGUCCUAGGAGAUGACAACUUCAUGCAUAUAAUUG

>P2769

CAGAUCCUUCCAGAGGUGUUCCAGAAGAUGGCAUUGUUGUCCUAGGAGAUG

>P2770

UUACCUGUAAAACAGCCUCAGGCAGAUCCUUCCAGAGGUGUUCCAGAAGAU

>P2771

AUAUUCCUUCUGCUUAUAUAUUAAAAAGUUACCUGUAAAACAGCCUCAGGC

>P2772

GGACUAUACUAUAAUUUUUAUUGUUAUUUUAGAGCAUAUUCCUUCUGCUUA

>P2773

UCACGAGGUCAUAGCACAAGGCAUUACUCACUGCCUGUGGUGAUGUUAGUG

>P2774

UGUCUCUAAAAAAGAAAAAAAAAGGAAAUUCUAGAGAUAAAAAUUAUCAAU

>P2775

GGAGUGUCUCUCUGUCCCCUAGGCUAGGCAAUUCUCCUGCCUCAGCCUCCC

>P2776

UUAAAGCCAUUCUUUAAAUCACAUAAAUUCAGGUCUGUGAUAUCUCCUUUU

>P2777

UUUAUCCUUCCUAUGAAGGACCAACAGUGUUGGUUGGUACUUGAGGAAAUU

>P2778

GACACUUGGGUUAUUUCUGCCUUUAAGUUAUCAUAAAUGAUGCUGCUAAGA

>P2779

AUAUUGAGUGAAAUAAGUUGUGAAAAGACAAAUACUGAUAUUCAAAUGUGA

>P2780

CCGUGUUUGCAGCAGCAUUAUUCAUAGUCACCAAAAGGUAGAAGCAAUCUG

>P2781

GGCUUGCAGGUCUGCAGGCUGUACAAGAAUCAUGGUCCUGGCGUCUGCUUC

>P2782

CAGGCUCAAGAGAUGCUCCCACCUCAGCCUCGCAAAGUGCUAUGGAAUUAU

>P2783

UUGUUUUUCAUUGAGAUACAGUCAUACUUUGUCGCCCAAGCUGGAAUGCAG

>P2784

UAGAGUGGCUCAUGCCAGAAUCCCAACACUGUGAGAGGUCGAGGUGGGAGA

>P2785

GCAUACCUGGAGUCCCAGCUACUUGAGAGUCUGAGGCAAGAGGAUCACUGG

>P2786

GCAAGGAUUUCUUCAGGCCGGGUGCAGUGGCUCACACCUUGAAUCUCGGCA

>P2787

GGUGGAGAGGCAACAUAGAUUAAUUAAAUUCCAUUUGUACGUUUAGGGGAA

>P2788

AAAUUCCUCCCUUGACUAUGAGACUAUUUCUUCCCCUCACUUUCAACCAGA

>P2789

GCCCGUUGGAGAAAACGGGAAUCCCAAAGAAAUGGUGGGUCCUGGCCAUCC

>P2790

GGCUCUCUUGCUUACAGUGGUCGCCAGCGCCCCCUGCUGGCGCCGGGGCAC

>P2791

GCUCUCCGGGUCUGUGCUGAGGAGAACGCAACUCCGCCGUCGCAAAGGCAC

>P2792

ACCACUGAAAUCUGUGCAGAGGACAACGCAGGUCCGCCCUCGCGGUGCUCU

>P2793

GCUCCGCCUUCGCAGUACCACUGAAAUCUGUGCAGAGGACAACGCAGGUCC

>P2794

CGCCCGCCCGCCCGGGUCUGACCUGAGGAGAACUCUGCUCCGCCUUCGCAG

>P2795

GACCCCCAGGUCUGUGCUGAGCAGAACGCAGCUCCGCCCUCGCGGUAUCCU

>P2796

CUGCUCCACCUUUACGGUGACCCCCAGGUCUGUGCUGAGCAGAACGCAGCU

>P2797

GUCACAAAGCCAGGCAGUGGCCCCAACCUCUGUGCUUAGGGGAAUGCUGCU

>P2798

UGUAUUUUAAACUAUAUAAUUUGGGAACAUUAUUUUAUAGACUGUGGAUAA

>P2799

CCUGUUUCAAAAAAAUAAGAAAUAAAAAGUGAGUCUUCAUAUUCAAAUUAG

>P2800

UAAUUUAAAAUGAGUCUUUAUAUUCAGGCCAGGUGCGGUGGCUCACACCUG

>P2801

UGCAGUAUUUUAUUUUUUAUUUUUUAUUUUUUUUGAGACAGAGUCUCACUC

>P2802

UAUGGAGGCCAAAUAUAUCAGAUGAAUAGGCUAAUUUCCCUGAGUUGCUGG

>P2803

AACAGGCAUGGUAGUGCAUACCUGUAGUCUGAGCUGCUUGGGAGUCUGAGG

>P2804

UGUCUCUACAAAAACAAAAACAAAAAACAAACAGGCAUGGUAGUGCAUACC

>P2805

GUAACCUUGAAUCCUGGGCUCAAGCAGUCUACUUGCCUCAGUCUCUCAAGU

>P2806

GACAGCUACCUUAGGAAGGUGCUGCAGGACUGGACCGCUUUCCAAAGCCCA

>P2807

CUUCAUCCACUUUCCCCCAUUGGCAACACCUUGCUUGAGUAUAGUCCAAUU

>P2808

UUGUCUCAAGAUGUUUUCUAAGGCUAGGUGCGGUGGCUCACGCUAGUAAUC

>P2809

CAUUAUUCACAAGAGCCAAAAGGCAAAAGCAAUCCAAGUGCCCAUCAGCAG

>P2810

AGCUGCUCCCAGCUACUAGAAGGCUAAGGUGGGAGGAUAGCUUGAACCUGG

>P2811

ACUGACACAGACGAGCUGCAGCUCAAGGCUGGUGAUGUGGUGCUGGUGAUC

>P2812

CUCAGGGAUGCCUCUCUAGGUAGCUAGGAGAGCUCUUCCUUUCAUCAGCUG

>P2813

GUUUUCCAAAGCAGCCGCAUCAUUUACAUUCUCAACAGCAAUGCAUGCGGG

>P2814

GAAAGAAAUGCAUAAAGUAGGCCAGACAUGGUUGCUCACGCUUGUAAUCCC

>P2815

CUACAGAAAAUUAGCUGGGCGUGGUAGCAAAUGCUAUAGGCUGAGGUGGAA

>P2816

GAACAGUUUAACCACUCAUGGUGGCACAUGCCUGUGGGCUUAGCUACUCAG

>P2817

AGAGCCCAAGAAAUGAUUUUUUUGUAGAGUCAGGGUCUCAUUUUGUUGCUC

>P2818

UGGUCUGGAAUUUCUGACCUCUAGCAGUCCACCUGUUUCCGCCUCCCAAAU

>P2819

CACUCUCCAGCGAUCAUCCCACCUCAGCCUCUUGGAUAGCUGGGACCACAG

>P2820

CCAGGGGUUCUGUGAGCUACCUGAGAAUACUGAAGUAUAUAAUGCUCUGGC

>P2821

UGCAACCUUAACCUCCUGAGUAGUUAGGACUACUUAAGAGCCACCAUACCC

>P2822

UCCCACCCUAGCCUCCUGAGUCAUUAGGACUAUAGGUGUGCACCACCACAC

>P2823

GCGUGUGGCCCCUGAGGAGCACCCCAUCCUGCUGACCGAGGCCCCCCUGAA

>P2824

GGGAGGAGGAUCAUGUACGCCCGGAAGUAGGACCUCGUCCAGUCGUGCUUG

>P2825

GUUGAUGACCGAGCUGAGGUAGAAAAACGUCUCCGAGAAGGGGAGGAGGAU

>P2826

CACCGUGUACAGGAGCGGGUUGAUGACCGAGCUGAGGUAGAAAAACGUCUC

>P2827

GACUGGCGCCGGGACGCGAAGAGCAACGGGCGCUGCACAAAGCGGGCGCUG

>P2828

UCGGCCUCGCUCUGAAAAGUGCUUAAGAAAAUCUUCUCAGUUCUCCUUGCA

>P2829

UGGUUUCGCGCCUGAGUUGGGCUCUAGUGACUCGAGACUCAAUGACUGGGA

>P2830

ACAUUCAAACUUCAUGCUCCUGAAAACCAUUCUCUGCAGCAGAAUUGGCUG

>P2831

CCAUCCCUGAUGGGGGCAUAGUUUGAGACUGCAGAGUGAGAGUGACGUUUU

>P2832

GGCCAGGCUGGCAGAGUCAGUCCUUAGAAGUCACUGAGGUGGGCAUCUGCC

>P2833

GAAUUUUCAGUAUGUACUUGAAGGAAGGAGGUGGAGUGAAAGUUCACCCCC

>P2834

UUCUGAAUAAAUUCAGCCUGACUGAAUUUUCAGUAUGUACUUGAAGGAAGG

>P2835

GUGCAAAUAAUGAAAGAGCUCGGUAAAGCAUUCUGAAUAAAUUCAGCCUGA

>P2836

CCGUGUUCUCUUUUGUUCCUGUGCAAAUAAUGAAAGAGCUCGGUAAAGCAU

>P2837

UUCUGGGUGUCCUUUUAUUCUGGGUAGGGAGCGGGAGUCCGUGUUCUCUUU

>P2838

CUUCUCGGCACACUGCUGAAGCUGAAGGAGAUGCCACCCCCUCCUGCAUUG

>P2839

CUCCGCACCACCAUCCUGUUCCUCAAAUUAGCCCUCUUCUCGGCACACUGC

>P2840

AGCUGCUGCAACACCCCUCUUUGUAACGGGCCAAGGCCCAAGAAAAGGGGA

>P2841

ACCAGUCCUUCUGCUCCCCAGGGAAACUGAACUCAGUUUGCAUCAGCUGCU

>P2842

GCCCAUCAUGUUCAGAGGUGAGGUCAGUCUGAAUGUGGAAGAGAAGAAGAC

>P2843

UUCUGAGCCUGUUCAGAAGGUCCUUAGCAACAGGCCCAUCAUGUUCAGAGG

>P2844

GCAAGGUGACAUUGAACCCCAAAGAAUGGUGAGACACCCUGACAGUCACCA

>P2845

GUACCUGAUGAUUCUGGAACUUUCUAUGAUCAGGCAGUUGUCAGUAAUGAC

>P2846

AAGAGAUUUGUGCACUCUGAUGUUCAAUGCAGCAUUAUUUACAAUAGCCAA

>P2847

UAACUCAUGUACUUGCCUUGACUUAAGUUACCAGUCUGUCCAAGAGAAAAG

>P2848

UGAGAAUGUGAUUGGAGCUAGAAGAACCUCCUGGAGAAUAAUCAGCAGCAU

>P2849

UGGUUUUGAUUUGCAUUUCCCUGAUAAUUAGUGAUACUGAGCAUUUUAAAA

>P2850

UCUGUAUUACUAGGUUCACGACAAUAGCCAAGAAAUGGAAACAACCUAAAU

>P2851

UUUCAGUGAGAAAUGACUGAUUGAUAAUUAGGUGAAAGCAUGUUCUUAUGA

>P2852

AUGGCAGAAGGGCAAAAAGGAAGCAAACAUGUCUUCACAUGGCAGCAGGAG

>P2853

UCACCUUCUUUUAUAUAUUUUUGGAAGGAGGAAGCUUUGUCAUAUCACUCC

>P2854

AGAUAGUCAACUAAUACUAGUGUGUAUGGAAUAUGAUGCAGCCAUAACAAA

>P2855

AUGAGUCCUAAAAAUCAUGGACAUUAGCUGGGUGCAAAGGCUCAUGCCUGG

>P2856

ACAUAUCAGCCCACACUGCCAUCCAAGCUUCCAAUGAAAAAUCUGUUGAUA

>P2857

ACAUAUAUAUGUGUGUGUGUGUAUCAGCAGAUUUCUCACCAGAAACUUGGA

>P2858

UGUCACAAAAUCACAGCCUUAUAUUAGGUUGGUGCAAAAGUAAUUGGAGGU

>P2859

AUUUAAUUCAUUCAACCUCAUUCUUAGGCAUUAUUCUUUUUUAAAUUUUAU

>P2860

GCUAUCCCUCUCCCCACCCCAUGACAGGCCCUGGUGUGUGAUGUUCCCCUU

>P2861

CUAAUGACUGGCCAUCAUUCUCAGCAAGCUAACACAGGCAUAGAAAACCAA

>P2862

GGUAGUUUACUGCGCUAUGUGAGAAAUAGAUAUGUAAAGCUAUAUAAAUAU

>P2863

UAGAAGGAUAUGUGUUUUGGUUGAUAGGUGCAGCAAACCACCAUGGCACAU

>P2864

UGUACUCCAGACUGGGAGGCAGAGCAAGACUUCAUCACAAAAAAAAAAAGA

>P2865

CCUCCCACAGCACUUGGGAAUUCAAAAUGACAUUUGGCUGGGGACACAACC

>P2866

CCCCAUGAUUCAUUUACUUCCCACCAGGUCCCUCCCACAGCACUUGGGAAU

>P2867

AUCAUACAUGGAUGGCAGCAGGUGAAGAGAGAGCUUGUGCAGGGAAAUUUC

>P2868

ACUUAGAGUUCCAUGUGGCUGGGGAAGCCUCACAAUCAUGGUGGAAGGCAA

>P2869

GCAUGAUUGUGUGGCCUCCCCAGCCACGUGGAACUGUAAGUCCAUUAAACC

>P2870

GGUCUCAUGAAAUCUGAUGGUUUUAAAAACGGGAGUUUCCCUGCACAAACU

>P2871

UUGUGGGAGGCACUCAGUGGGAGGUAAUUGAAUCACGGGGGUGAGUCUUCC

>P2872

AUCUUGAUUUCCCACAUGUUGUGGGAGGCACUCAGUGGGAGGUAAUUGAAU

>P2873

UUCAAAAUAUGAACAGUCAAAAAGCAUGACAAAUAUGUACUAUAGUGUGCA

>P2874

GUGUAGACUGAGGAAGGCCCUGAUAAUUUGGUAAUUUAGGUAAUUCCUGGA

>P2875

GGUCAAGUGAAUAGUUGGAAUUUCCAGGCUGUGUAGACUGAGGAAGGCCCU

>P2876

UAUUUUGAACACUUUUCCUCUAUUCAGAAAUUACCUGAGUUAUCAUAUUAU

>P2877

AUACUCUGUAGUGUGUAUUUGCCAUACUUUUUUGACUGUCCAUAUUUUGAA

>P2878

AAUAGAGAAAGAAAUAUGUUCUAAUAAAAUAUUCUAAAGCUCAGCUUAGAA

>P2879

GCAGCUGGGAGCCUGAGGUGGGAGAAUCAUUUGACUCUGGGAGAUUGAAGC

>P2880

AAUAGCUGUCUACUUUGGGAGGCCAAGGUGAGUGGAUCAUUUGAGGUCAGG

>P2881

CAGCUACUACUAUGGAAAAAGGAAAAGAAGAAAGGAGGAAAGGAAGGGAGA

>P2882

UUGUAUAUGAAAAAGUGAUAUGAAAAGAAAGUAGGCCGGGUGCAGUGGCUC

>P2883

AGAGAGAGACAGGGUCUUGCUCCUUACUCCGGUGGAAUACUUCAUUGUACU

>P2884

GAUCUUGCUCUUUCACCCAGGGUGAAGUGCGGUGGUGUGAUUGUAGUUCAC

>P2885

CAGCAAGACUCCAUCUCUACAAAAAAAUUUAAAAAAAAAAAAGAAAAGACC

>P2886

UAAUCCCAGCAACUUGGCAGACUGAAGCAGGAGGAUGGCUUGAGUCCAGGA

>P2887

CCAGGCAUUGUGGUUCACAUCUGUAAUCCCAGCAACUUGGCAGACUGAAGC

>P2888

CAUUUUAAAGUUAUUUCUUCUAGCCAGGCAUUGUGGUUCACAUCUGUAAUC

>P2889

GUGUGGAGUGCAAUGGCUGGAUCUCAGCUCAUGACAAUCUCCGCCUCCUGG

>P2890

AUUAAGGCAGAGCCCUCAUGAUCCAAUCACCUCUGAAAGGCUUCACCUCUG

>P2891

GCCAUAUGAAUUGAGUAGUUUGCCCAGGUUGCCUAGAAACAAAUAAACAAA

>P2892

UUGGGGAGGAAAUAGAAGCCAUAUGAAUUGAGUAGUUUGCCCAGGUUGCCU

>P2893

GGAGUGCAGAAGACGUUUAGGUUGAAGAUGGAGUUUGGGGAGGAAAUAGAA

>P2894

UGGAGAGGUACAGGGGCAGAUGGAUAAAGGAUUGUGGAGUGCAGAAGACGU

>P2895

UCUAUUUCCUCCCCAGACUCCAUCUACAACCUGAAUGUCUUCUGUACUCUA

>P2896

CUUUGUUUGUGUUUUUAGGCAACCUAGACAAACUUCUCAAUUCAUGUGGCU

>P2897

GCUGGAAGUCCAAGAUCAAGGUAUUAGCAGGUUUGUUUUCUUCUGAGGCCU

>P2898

CAGAAAUUUAUUCUCUCACAAGUCUAGGGGCUGGAAGUCCAAGAUCAAGGU

>P2899

ACAGACUGGGUACCAAGCAACAGAAAUUUAUUCUCUCACAAGUCUAGGGGC

>P2900

UCUUGAUCUUGCACUUCCAGCCUCCAGAUCUGUGAGAAAUAACUUUCUGUU

>P2901

GCCCUCACAAGACACUGACUCUGCCAGUAUCUUGAUCUUGCACUUCCAGCC

>P2902

AUGAAUCAGGAAACAGGCCCUCACAAGACACUGACUCUGCCAGUAUCUUGA

>P2903

CCAUGUGAGGACACCAAGAAAACACAGCUGUCAAUGAAUCAGGAAACAGGC

>P2904

UGGUAAAGCUGUCAGGGGAAGCUGUAGGUAAGCACUUGAAAAACAUGGUAA

>P2905

AACUCCAAAACCAGAACAUUUUAUUACGGCUUUAGUGUUGUUGCUUGCUAU

>P2906

GAUCUAUGAAAGGUUACUGCCUGGUAGCAGAGGUUGGAUGGACUAAUGUCU

>P2907

AAUGCUAGUACCAUUUGUUGACAUGAGUUCACUCCACUCUGCCACCAGGUA

>P2908

AUGUAUACAUUACACUGUGUAUAAUAUAUACAUCACAAUAUAUAUGAUAUA

>P2909

CCAGGAGUUUGAGACCCGCCUGGGCAACAUGGAAAGGCCCCAUCUCUUAUA

>P2910

GUGGCCUGAUAACAGGUUCAUGCCUAUUAUCCCAACACUGGGAGGCCAAGG

>P2911

AAAAAAAACAGAAUGUGUGCCUAUAACCCCUGCACUUUGAGAGGCUGAGGU

>P2912

UGGGUCUCAUUCUGUCACCCAAGCUAAAGUGCAGCAGCACAGUCAUGGCUG

>P2913

CUAUUAUUUUUAUUUUUUUGUAGAGACAGAAUCUAUCUCUGUUGUCCAGGC

>P2914

AUUGCAGGUUGAAAAAUGUACAAUUAGCUGUAGGUAGGCAUAUUCUACAAG

>P2915

UAUUGAAUGUGCCUACAUAUAGCUAAUUGUACAUUUUUGAACCGAGUGGUC

>P2916

AACACAAUUUUCCGUGUUAAUCCUUAUUGAAUGUGCCUACAUAUAGCUAAU

>P2917

UUCCAAGGGCUUGUUAGCUUCCUAUAUAAUUUGAAAAACACAAUUUUCCGU

>P2918

GUGUCUGUAAAAAGGAAAAGAAAUUAGAUUCUUCUAUCCCUGGAUAAAUUC

>P2919

UGAUUCAGAAUUAGAAAUUCCAAGUAGAAAUUCACCCACGGAUAGAAGAAU

>P2920

ACCUCCUCCUCCCAGGCUCUUGAGUAGCUGGGACUACAGGCAUAUGCAACC

>P2921

ACAGGGUUUCAAGCUAUGGACAAAGAUAGUCAAAAAAAGCAUUUAUUCCAU

>P2922

UUGUGUUUACUCCGUGUAGAAUUAGAAUACUGGCAAAAAUCACUGGGGAAA

>P2923

UUUUCUGAAGUUAUCCAGUUGACAAAGAAUGAGGCUGUGGCUUAGCCCAGU

>P2924

AAUCCUCCAGCCUCAGCCACCAGAAAAGCUAGGACUACAGGUGUAUAUGCC

>P2925

GCUGGAGUGCAGUAGCAUGAUCGACAGAGUGAGUUUGUCAAAAGUAUCUCA

>P2926

AUUAAGUAGCCUUAUGACAAACAGUAUGAGAUACUUAUGACAAACUCGCUC

>P2927

GAAAUUAUGAAAAUGCAUAUUCUCAAACUCCACGUCAGACUUACAGAAUCA

>P2928

GGUGGAGUCUGAAAAUUUCAUUUCUAAAUUUCCAGGUGAUAUUGAUACUGU

>P2929

CAUUAUUAAAAUUUUUUUCAUGUUUACUACUGUGAAAGGUUCAUGGAAUUC

>P2930

GUUAUAAGUAACGUACAUUUAAGAAAGAUAUAUCUGUAGAACAGUUUCUGC

>P2931

AAGAAGUGGUGAGAGGAAGCGGCAAAAUCAAUGACAGAACACAAAUACCCU

>P2932

AAAGAAUGAAGUAGUAAUACAUGCUACAGUGUGAAUAAUUCUUGAAAACGU

>P2933

CUCGUUGCUCACACAAAGCCUGUUGAUGGACUCUCUUCACACAGACGUGCA

>P2934

AGUGAAAAUAGUCAGUUCCUGCCUUAACUGAUGACAUUCCACCAUUGUGAU

>P2935

GGUGCAGGCAGUCUGAAUCCAAAAAAGGAGUCAGCAAAGGGAGAUAGGAGU

>P2936

CUCAGCCUUUAAAUUCAAGGCUUUAAGGCCUGGGUAUGUUGGCUCACACCU

>P2937

GCCAGUUAUGGUGGUGCAUGCUGAUAGUCCCAGCUACUCCAGAGGCCAAGG

>P2938

UGAUGCUGAGCAGCAGCAGUGAGCCACAGCUCCUGGUCAGCCGCAUGAUAA

>P2939

ACAUUCAGUAGAAAUUGGUACAAUAAUCCCUUGAUGCUGAGCAGCAGCAGU

>P2940

UUUUCUAAAUUGUUUACUCGGGAAUAUUACUGUGCAAAGGCACACUAAAGU

>P2941

GCCCAGGUCUGAUGCUGACAACACUACUGAUUUUAAUUUCAAGGCAAUCAU

>P2942

AAAUUAUUUAUACAAGAAUUUUACUAUUAGAGAAAGAUAGAGCUUCUAUAU

>P2943

GGAAUCUGCAGAAUCUCUCUUUAUCACUAGAUUUGGGGAUUGACACCCUGG

>P2944

AACUGCAAACAAACAAAAAACAAAAAACAAAAAACUAUUCCACAGGCAGCC

>P2945

CACAGGAUUGCAAGAUAAAAUACAGAAUGCCCAGUUAAAUUUGAACUUCGG

>P2946

AUAUAUACUGAGGGGUCACUUUGAAAACACAAUUGAGGCUGGGCAUGGUGG

>P2947

CCAGGCAUGGUGGUGGGCACCUGCAAUCCCAACUGAUCAGGAAGCUGAGGC

>P2948

AGUACUGCUGCUUUUUUUAUAUUUUAGUUUGUGUGUGUGUUUUUUUUUUUA

>P2949

UGUUGAGAGCAUGGGCUUUGAGUCUAGACCAGGGGUGAGUCUGCAGCCUCC

>P2950

GCAGACUCCCCCCUGGUCUAGCCUCAGUGCCCACGCUCUCAACAGCCAUUC

>P2951

CGAUCACACAGAUCCUGAGUCUGGAAGCUGCAGACUCCCCCCUGGUCUAGC

>P2952

GCUGGCCUAAGCCCAGAAAAAAUUAAGGCAGCUUGAAAGCUAAAGGCAAGA

>P2953

AGGCUUGUAGGCUAGGGGUUUUUCAAAGACAGUUUGGAGGAAGGGAUGUGG

>P2954

GCAUACAAUGGAAUACUACUUAGCAAUAAAAAGGAAUGAAUUAUCGAUCAU

>P2955

AACUAUUAUGUAAUAGAGAAAAAAAAAGAGUCCACCAUGGUUGUUUAGGUU

>P2956

AUUCCUGUAUGUUGAAAUGUUUUUUAUAAUUUUUGAAACUAUUAUGUAAUA

>P2957

CUGGAUGGUAGGCACAUGUGUAUUCAUUGUAUUACUCUUUGCACAUUCCUG

>P2958

GGAACCCUCCCAUGCUGGUGGGAGUAUAAAAUGGUGCAGCCACUUUGGAAG

>P2959

UGAAAAAUAAUUAUUGGCAAGGCACAGUGACUCAUGCCUUUAGUCCUAGCA

>P2960

CAACAGUUUGAGAACACCUUGGGCAACAUAGAGAGACCCCAUAUCUACAAA

>P2961

UUCACGUAAUUUGGUGUUUAUGUUUAUCCAGGUAUAGUCCAAAUGAGGGCA

>P2962

GUUAUUAAAAAUUCCAAGCUUAUUUAAUUAAUUUAUUUAUUUGAGAUGGAG

>P2963

UGGGAAACAAAGUUACAAUAAAAUAAGGCAUGAGAAUGGAGAAGGAGGUAA

>P2964

UCAGAUCACUUGAGCCUGAGAGAUGAAGAUCAGCCUGAGCAACAUGGCAAA

>P2965

GCCCCACCAGCUUUCUUAUGCAGUGAUUGCAUAGUAUACCUUUUUUUACGU

>P2966

AAGUUUUUCCUCAAAUCAGUUGCUUAGCUGUAUCUCAUGCUCAUGCACAGA

>P2967

AGACAGCUUUGCAGGGCCAUUUCAAAAUAUGUCAAGAAAUAUAUUUUGGGA

>P2968

UGUCCUACCUUUCAGGACUAAACCAAUGUAUUUCUUAAAUGUAUUUGAUUG

>P2969

ACCAAUUGUAAAUCAGAAAAUGUUUAAAUUUACCUAUAGCCUGGAAGCCCC

>P2970

AGAACCUUGGUCUCCACAAUCCUUUAUCUUAACCUGAACAUUUCCUUUCUG

>P2971

UCUAGCACCUUUUAAAGAUCUGAAUAGGAAACGUUUGUCAUCUGUUGUCUC

>P2972

UGAAAGGAGUGUCUGGAUUAAGACAAGGGGUUGUGGAGACCAAGAUUCUUA

>P2973

AAGAGGUCCUGAAAACAUGUGCCCAAGGUGGUCGGGUCAUAGCUUGAUUGU

>P2974

GACCAAGGCCUGAGGCACAGUCCCAAGAGGUCCUGAAAACAUGUGCCCAAG

>P2975

GCAACUGAGCAAGAUUCCAUCUCAAAAGAAAAAAAAAAAUUGACAUUGGUA

>P2976

AGACUCCAACUCCUGGCCUCAAACUAUCCUCCCACCUUAGCCUCCCGCACA

>P2977

GUUCCACCUCAGAUCAUCAGGCAUUAGAUUCUUGUAAGGAGCGCACAACCU

>P2978

AAGAGAAAUGCAAAUCAAAACCACAAUGAGACAUCAUCCCACACCCAUUGG

>P2979

UUAAAAAAAAAUUAUUUAUUUAUUUAGAGACAUGGUUUUGCCAUGUUGGCC

>P2980

UGUGCCUGUAAUCUCUCAGGAGUGUAAUUAUUUCAUGAUAACCACACCCCC

>P2981

AGACCAGCCCGGGCAACGUGGUGAAACCCGGUCUGUACUAAAAAAAAAAAA

>P2982

CUCUCCAACAGGAAAGAUAUAAAGAAAUUGAGCAUUCAUCCCCACCCUCAC

>P2983

AAGCAGAGGCUGGCACCAUGCUUCUAGUACAGCCUGCAGAACUGUAAGCCA

>P2984

CUCCCCUGGAAGCUGAUUGUUUAAAAGAGCCUGGUAUCCUCCCUCCCUUUC

>P2985

CAGUUCUGGAACCUGGGAAAUCCAAAGGCAUGAUGCUAGCAUCUGGUGAGG

>P2986

CAACUAAUUUGUUUUGAUUUUGGGUAGAAACAGGUGUCACUAUGGUGCCCA

>P2987

GGAUCACUUGAGCCUGGGAAUUAGAAGCUGCAGUGAGCUAGGAAGGUGCCA

>P2988

GGUGAUGCAUGCUACUUGGGAGGCCAAGGUGGGAGGAUCACUUGAGCCUGG

>P2989

AAGGCAGGAGGAUCAGUUGGGGCCAAGAAUUUGAGACCAGCAUGAGUAACA

>P2990

AAAAAGUAAAAUAUUAGCCAGGCAUAGUGGCACAUGCCUGUAAUCUCAGCU

>P2991

CAACCUUCUGAGUAGCCGGGGCCACAGGUACGCGCCACCACGCCUGGCUAU

>P2992

UUUAAGGCAUAAAAUUCGGCUGGGUAUGGUGGCUCACACCUGUAAUCCCAG

>P2993

ACCCCAGCUGAUCUUGUCUCACACAAACACACCACCCCAGCUGAUCUUGUC

>P2994

AGGGAGGUAGAUGGAAACUCUAUGUAUGUCCACUGUGUAAUUUUUAUACCU

>P2995

AGUCAUUCUAUGACCCAGCAAUUUUACAUAUACAUGUACAUAUUCAUAUAC

>P2996

UGAACAAGUCACUUCACCUUCCUUCACCUUGAGUUUCCUCACCUAUAAAAU

>P2997

CCACCUGGGCAACAUAGUGAGAGCCAGUCUCAAAAUUUUAAAAUUAAAAAA

>P2998

GCCUCCUGGGUUCAAGCGAUCCUCCAGCUUCAGUCUCCCAAGAAGCUGGGA

>P2999

GCUGGCAGGCUGGGUGCCAUGGCUCACACCCGCAAUUUCAGCACCAUGGGA

>P3000

AGAGAAAUGAAAAUAUGUCUACCCAAAGACAUGUUCAUAGCAGAAUUUGUU

(**2) List of 3,000 non-A-to-I editing-site samples**

>N1

UUCCACCCUAGCUCCUGUCCCAGGGAAGCAGGCCAUGGGCUCCAGUUUCUU

>N2

GAACCAGAAAAAUCACAAAUGAAUGAAGAGUCGAUUGAGACUCCAACAUAA

>N3

AUUAUGGGUUCUCUUGAAACAAAUGAAAAAAAUAGAAAAUUUCAGCAAAAA

>N4

AAUUAAAAAUGAAAAUCAUGAGAAUACCUCAAAUAAAAGGCUACGCAUGGG

>N5

CAUCUCAAUGAAAUGGAGGUGACAGAGGAUGGAAUCCGUGACCUUGAGGAU

>N6

AAUUUGUUCCAUCUGAAAAACAGAGAAAACAGUUAUAGGGCAGCAGAGUCG

>N7

UUCAUCUGAAACCAUGGAGACCAGAAAUGAAUGAAGUGCUUUUCCAAGUGU

>N8

AGCGAUAUUUGAUGUUUGAAGCAAAAGUUACUAUGCCAUCUGAUGGGAUGA

>N9

UAGAAGAAAUACCUGAGACAUGGAUAUUUAAAAGAUGGGAAGUGGAAAGAG

>N10

ACUAGAGACGGGGUUUCACCGUGUUAGCCAGGAUGGUCUUGACCUCCUGAU

>N11

AUGGUGGAAAAGAGGGAUCUUUAGGAUGGCUCCCGGAAGUUUGGCUUCAGG

>N12

GUGGAAAUAUUCUCAUUUCUAAUUCAAUGGUCGUUUAUUGUCCCUAACUCA

>N13

UAGAUAUUAAAAGCCUAAAAACCUAAAGAUUGAUGAUGGUUUGUGUCUGAU

>N14

AUAAUAAGGAAAAUAGGUAUAGGGAAUGUAUUGAUAAUAGAAUGUACAUUA

>N15

AAAAGCCAUUAGGUUUUUGUUUAUUAUCCUUUUUCUGACUUUUCUCCAUAU

>N16

CAAUUAUCACUUGAAUCAACAAUAUAUAUUAAUUUUUAAAAAUAGAAAUAG

>N17

UUAAUCCCUCCGUGUGUGUUUGUGUAUGUGUCUGUGUGUGUGUGUGUGUGU

>N18

GUGUGUGUGUGUGUGCAAUGAAAGAAAAUACAUACACUAAUUCUCCAAAAU

>N19

GAGAACAUGGACAUAUAAAGUAUUCAUUGCCCUGGCUCCAAAAUGUGCAAG

>N20

AUACAUUGCAAACAAAUUUAUAAGGAACAUCAUAUUGUAACUAAUAAGGGC

>N21

AUUGUAUUAAAAAUAACAUACUGGGAAUAGGAUGUAUCCUCGAAUUAAAUA

>N22

AUAUCUGGGCCCAACAGCUAUCUCAAUUGUGGAUGAUAUUUAUAUAAGAGG

>N23

CAGAUGACAACUCUGCAGAUUCUGCAGCGGUAAGCCCUUCUUGACUCUUUG

>N24

GAGCUCUAGCAUCCUGACUUCUUUAACUAAAUUAAUACCUUUAAUCUGAUA

>N25

GGCAAGCGGCCCAUCGGUAUUGGCUAUUGAUAAUAAUUGAAACAAUAUGCU

>N26

GAGAACCAGUGGGGUGUUUGGAAUGAUGGCUUUUGUCCAUUUCAACAUUGG

>N27

CUAUAUUAUAAUAAGAAAGGUAGACACAUGGCAGAAUCAAGUAUAAAUUGC

>N28

GUUUGUAGUUCUUUGAGAUAUAAAAAAGCCUCCCAAUUGUCCUAUAGAACU

>N29

GAUUACCUAAACCUGCUUCCUGUUGACCAGUUCUUCUUCUUUACCCCUCCU

>N30

CAGGUGGAUUUAAGACUGAUCUGCCAUCUGCUCUCCUACAGCACCCAAUUG

>N31

CUGAACCCCUGACAUUCAGGUAACAAAACCAAUUUCCAGAGGACAUAGGCA

>N32

UUUUGAACUAGAAAAAAGAAAAUAAAAUGCUCCAAAAGUCACAGCGUAAGA

>N33

CUAGGGCAAUCAGAAAUCUGCCAAAAGUUUUGUAGGGGAUUUUUUUUUUUC

>N34

UGCUGUGUAUACCUGGACUCAAUACACUUACCUAGUCAGAUGAAUAUAAAC

>N35

CCAGGGAGCCCAGUGCCCUUUAAAAAAUGAACAGUGAAGAGAUGGCUCUAC

>N36

CCAACAGCAAGAGAGAAGUAUUUUCACCUUAUAGUAUAAUUUACCCCAAGG

>N37

AUUAAGAAAUAUUGCAGGUUCUCUAAAGCUUGUAUAUCUUGGGCUAUUACG

>N38

GACUGGGAAAAUAAUAUUUUUAGGAACUCACUCAAAAAUGGACACAUGCUU

>N39

AUUUAAGUGGUUUCUCCAAGUAACAACAAUCAUGUGUUCCUUUCUUCCCAA

>N40

UUAAGACCCUACAGAAGCUGCAUCCAUGUGGCUGCAAAGGACAUGAUUUCU

>N41

ACAUCUAGGUUGAGUCCAUGUCUUUACUAUUGUGAAUAGUUCUGCAAUGAG

>N42

CUGGGCCGAAUGGUAGUUCUGUUUUAAGUUCUUUGAGAAAUCUCCAAACUG

>N43

UGUCAGAUGUUAACACAGGAACAGAAAACCAAAUACCGUUUAUUGUCACUA

>N44

AACUGUGCAUGUACCCCCAAACCUAAAAUGAAAGUUGUAAAAAAAAAAAUA

>N45

AUGUGGGAGCAGAAAGCAAAAACAAAAUUAGUUGGCUUAACUGACAUGUAG

>N46

ACUUUGAGGAAAAAUUAUAGCAUUAAAAAAAGACCUUGCAGAAGGAUAAUG

>N47

UUUAGAACUGCAGAGCUCCAGGGACAUAUGAAUGCAUGGCUGAGGUCACUU

>N48

UCCUGCCAGGCCAAUACCAAGAAGGAAAUGCUAAUAUAGCCCAGUUUGGAU

>N49

CUGUCAGAAGCCAGAGGAUAUGCUGAAGGAAGCAGAGAUUGUAUAUUAUGC

>N50

CUACCAUACCAUGGCAUAUGCUGUGAAAACGCCCUCAAUCUUCCUCAAAGC

>N51

CAUUUACUUAGGCUUCUUUAUACUGAGGAAAAAAGAAUAACAAGCCAUUUC

>N52

UAAUAAAGGGCCCGAAUUGACAUUGAUUGACCCGAAUAAAAAUCCAAAGAU

>N53

CACCUUUCAGUGGUCUGCUAGGUCCAUGAAUCUACUCAAAGGUCAUUUCUC

>N54

GUACAUAAUUGGAACUGACGUCCUUAGUAAUUGUAGUAACCCCCUUAUUGA

>N55

UGUGAGAAAAAAACUGUCAUCAUGGAGAAAGGCCAAGUGGAAACCUCUGAA

>N56

ACCCCUCUGCCAAAAUAGUAAGUAAAAAACAAUAUUGUAUCCUGGGAGAGC

>N57

UUCUAAUUAGCAAAGAUGAUUAGAAAUAAUUCAUAUUCACUUGGAAGGGGA

>N58

AAGAAAGAAAAGACAGAAAGAAAAGAAAGAGAAAGAAGAAAGAAAAAGAAA

>N59

AAAAGAAAGAAAGAAAGAAAAAGAGAGGAAAGAAAGGAAAGAAAGAAAGAA

>N60

AGAAAGAAAGAAAUCUGAAUUCAGGACUAGAUCUAAAAUAAUAAAAAUCAA

>N61

AAAUAAAUUGAAUAAGUUAAAAGUCAGCAUGACCUAGUGAAGACUGUCAAA

>N62

CUCCAUUUUCUCCUGUCAGAGAGAAAUAAUAUUUUUUAUCCUAGAUGGUCU

>N63

CUAAGCAGCUGGAAUACUCUUGCCCAUGGCUUGCUUGCUCAUUUCUGCCAU

>N64

AUCACCCAUUGUGUAUAUAUUGCCUAUAACUGUUUUUGUACUGCCCUCAGA

>N65

GCUGCCUUGUGGCCUGCAAAAUCGAAAAUAUUUAUUAUCUGGCCCUGUACG

>N66

UUUAUUCAACAGGGUGUCUAGCACAAAAUUAGCACACAAAAAUGGUCAAUA

>N67

GUUUGUUUUGUAGAGGGAAAGGGAAAUGAUUAGUAAAAGGGAGGUUUCAGU

>N68

GAGAUAUGGACUUCUUUGCAGUCUGACAAACUUUGUAGACACUUCUCAAAU

>N69

AAAUGCUAAAGGAAGAAGAAGGAGAAAGCCAUGAGAUGACAAAGGCAAUAU

>N70

UAAUAACUUCUGUGAUUCUGUAUUUAAUGUGGUGGAUAUAAUAACAAUUAA

>N71

UCAUGCUGAGAGUAAACUUUUCAAGAUAUUUGGAGCCACUGUGAAUGUGAG

>N72

UAAUUUUUCCCUUGUAUUCUUUCUAACUCUUUUUCUACUUGAAGCAUUAAG

>N73

GACAAAUUUGAGUCCAUACAUUUUUAUUACUCCAAAAGAGGUACCUUUUAU

>N74

AAAAAUUAGCAGGGCGUGGUGGCACAUGCCUAUAAUCCCAGCUACUUGGGA

>N75

GCAGAAUUGCUUGAACCGGGGAGGCAGAGGUUGCGGUGAGCAGAGAUCACA

>N76

ACCUUUAGAGUCCCAGUGAGAUCUUAUUUUCCUUCUAAAGAAAUUGCUGAU

>N77

UUAUGUUUAUAAUUAGUCUCGAUCCACAUUAGUCAAUUUGGCAUAGCUAGC

>N78

GCUACAGUGGGCCCUAUGCAAAACAAUUCAUGUAGCAUUAUGGGAAUUUGG

>N79

UCCUGGCUAUCUUGCUUUUAUGUGCAUUUAUUACUAAGAAGUUGUACUCAU

>N80

AUGUCUGUAACUUUUUUUUUCUCUAAACCGAGCCUUUUCUGGGUGGGAAGA

>N81

ACAUCUAACAUUUUAUGAUUCUAAUAUAAUCUUACAAAAUAAUAAUAAAUU

>N82

UUUCUAGCCAUGAUGCUAUUAAGAGAAUUGUUGGCCUGGAAAAUAGAUCUC

>N83

AAUGUUGCCGCUUAGAGAACUACCAAGUUUAAGACAGGUAAAGAAACAGAC

>N84

ACUCAGGAAGACAGCUGCAGGUGGAAGCUAUGACUAUGCUGAGCUCUCAGU

>N85

ACCGUGAAACGUCAUGUGAGCUCCAAGUGUCCAGCUCAGCACGGAAUAGAC

>N86

UCAAGGUGCUUCUGGAGAAGUUAGAAAACUCAGAAAGGAACAUCAUGAGCA

>N87

GCAGCAAAUGAGAAGAAUGUGGAACAGGAGAGACAGAAAAAGGGCAAAAAC

>N88

GGCAAGUGGACAAGAUGGUCUCACCAACCAGGUCGACCACUGUAUGAGAGU

>N89

CAGUAACUCCACCUUCUUAAGACAUACAUGGGCUCUUGUGCAUAAGGCUCA

>N90

AAACUUCAAAAAGGCCAGUUUACCCAGCCAAACUGAGAGCUGGAUUUCAGA

>N91

CAUCUAGAAUGGCCAUUUAGUUUUCAGAUAAAUUUCUAUUUCAAAUAAACA

>N92

AACCUAGUUUUUUUAAAUCACUAUUAUGGCUAAUUUAAUACAUUUAAAUUC

>N93

UAAAAGGCAAAUAUUAAGCCAACAAAUUAAUGUAAUAGAUUUUUUUAAAAG

>N94

GUGAAACCCCAUCUCUACUAAAAAUACACAAAUUAGCUGGGCAUGGUGGCG

>N95

AUAAAUAAUUUUUUAAUUUAGAAAGAAUUUCAAGCUCAGCAAAAGUGAUAA

>N96

AUUCUGUCCCCUCCCACACGCACACACAUCUAGGAGCGGGGAGCAUCUGAU

>N97

AUACAUCAUGGCCCUUUACUACUAAAUACUUUAGUGUGUAUUUACUAAAAU

>N98

UUCUCUCCAGUCCAGAGUCCAGUGCAGGGUAAGACUGUGCAAUUACUUGUU

>N99

CUUGAGAAGUAUUACAUGAUCUUGAAUUUCCUUCAAAGGCAUUAUAUAAAA

>N100

AUCAGGAUACUUCAAUAGGGAAAUAACAGUCUUCUUCAACAAAUGAUGUGC

>N101

CAUGAACCAUAAAAGAAAAAAGUUUAUAAAUGGGGUUCCAUACAUUUCUGG

>N102

CCACCACUAAGAAAAUGAAAAGACAAGCCACAGACUAGAAAAAAAAUCUGA

>N103

UAAACAAAUCAAUGAAAAAUGGGCAAAAGAUUCAAAUGGACAUUUCACAGA

>N104

UGUUCAUAGCAGGGUUUUUUGUAAUAGCUAAGACUGGAAACAACUCAAAUA

>N105

UGGUCAGCCACAGUCUGAAAAGAUUAAAUGGAAAAUUCCAGAAAUAAACAG

>N106

CAGGUGACCUUCCCUUUAUUUAAUAAUAGCCCCACAGCACGAGAGUGGUGU

>N107

GGACUCCUGUAUAUGAAACUUCGGGAAAUCAAAUCCCAUCUACAGUGACAG

>N108

AACUGUGGUGAUUAUCACGUUAAGUAUGUAAACUUGUCACAUCUUUUCAAU

>N109

ACAAUGGUUCAUUUGACUCUAUAUAAAUUAUAUCUCAAAGUUGAACCUUUU

>N110

AGAUUGAGCCAAGUCACCCAAUUUUAUUUUAACAUUUUUAACAUUGAAUCA

>N111

CUCAACAAACCCUUUACUAUUUAAGAAGGAACAACCCUUGAUCUCUACUCA

>N112

GCUCCUGCCUGAGCUUCGGAACACGACAACUACCAGCAGAUACAUCCCCAU

>N113

AUACUGUUUCUACAUUAAUGCUGCAAAGUGCUCUCAUUUUCAUCAUGUUGG

>N114

CCGCCGACAGCAGAAGUUUCAGGGGACAAGGGGGACCUGCCAGAGCGAGAG

>N115

GAGCCCAGCAGAAGACAAGCGUUUAAGAAGACAAAGCAGUAGAAACCCCGA

>N116

CCCACCCGUGGGCUCCCACGCCCUCAUCUGUGAAGACAUCACCAGGCCGCC

>N117

GGAAUAAAAGGGGGUCAGUAAGGGAAAAAAGACUCCAGCAAGUAACUCAAU

>N118

GUGCGCGGGUUCUCAAUCAGGGACAAUUGUGCCUCCAGGGGACAUUAAGCA

>N119

AGCGGCUGCUCAGGGCAAAAAAGCAAAGGAAACCAGGCAAAAUCCCUGGGC

>N120

GCGCUGCCUGACACAGCUGAAGGCAAUUCCACACACCAACUCCUUUCACCC

>N121

CAUGCAGGAUCCACGCACUCAACACACAUGGGCGCCACCCCCCCGCCCUGC

>N122

GAACCCUGCCCUUGAGGGCCAAUCAACGUCAAAUGACUUGCCAUCUCCUAG

>N123

CUUUGCUAUUAUGAACAGUGCUGCAAUAAACAUACGUGUGCAUGUGUCUUU

>N124

UGGUGCUUGAAAACAUAAGAAAUAAAUUAGAGAGCAAACCACAACAAAGUC

>N125

CUCUCAGCCAUGCCUACGACAAACCAUUUCUCAGUGUGACAGGUACUGUUC

>N126

UAUUCAGCAGGAGUCUUGAUGGGAGAUUAAACACAAGGAGACGUCCCCCGG

>N127

UAGCUAGCUUGCUUGCAUCAAAGUAAAACUGGUUUUCUAACUAGUCUUUUU

>N128

CAGAGUGAGGAAAAAGCAGAAAAAAAGAGAAACGUGAAAUUACCAAUAAAU

>N129

AUUCAUCUAAUACUGUUUUCAAAGUAAUGACAGAAAUAGCAUACAUCACUU

>N130

AUCCUCCCACCUCAGCCUCCCAAGUAGCUGGGACCACAGGUGCGUUCCACG

>N131

AAGCAAUUCUGCCUUGGCCUCCCGAAGUGAUGGGAUUACAGAGGCGUGAGC

>N132

CUUCAAAUCUAAAGUCAAACCCCCCAGACAGCAGACAAAGGCCCCUCAGGU

>N133

CCUGUACCCCCGCCCUCCGGCCUCCACAUCUCUGUGGUCUUGCAGACUCUA

>N134

AUGCCUGUAGUAAUAUCUAUCACAUAUCAAGUACUACACUUCAGCCUGUGU

>N135

GAAUGAGUGCAGUCACGAUGCUAGGAAGCGGCUCAGCCCACGUGGAGACCG

>N136

GAAAAAAGAGUGGGUAUCGAAACAGACACAGCAUGGAGCAUCAGGCCUCAA

>N137

GUUUUUAAAGAAUUUCAGUCGCUUAAGAAAGGGCUCACAACAUAAUGCUGU

>N138

AAGGGAAAGAUGAAGUAAUAACAUAAACAGCGACAACCAGGCGCGGUGGCA

>N139

AAAACUCUGUCUCAAAAAAAAAACAAAACAAAAACAGCGACUACAAAGGAG

>N140

UUUGUAAAAGUGUUUUUUACAUCUCAAAUUUUCUAAAUUGAACAUGAAUUC

>N141

UUAUUCAGUUCACUAUAUCGACUGAAUUUCUCAAUAGCCCGCAACCUUACU

>N142

AAUGUAUAUUUUAUGAUGCCAAAUAAAAAUCUCCAUUUUAAACCACCUCUA

>N143

GGCCCUGUCUUUAUGCUGGUAAAAGAAUGUCACAGCACAUACCAUUAUAAA

>N144

GAGAUUGCAAUCCCAGCGAAGUCUGAACAAAUCUAUGUCUAAAAUACGGGA

>N145

CCCAACAGUGUCCUAGGAGAAAUCCAAGCCAAUCUCCAGGUCCAGCCCUCA

>N146

AGGCGACUCCUUCUUGACUUCCGUGACAGUUAAUUUUGUGUGUCAACUUGC

>N147

GCACCCAGUGAUUUAACCAGACACUAACCGAGGUGUUGCUGUCAGGGUGUA

>N148

AGCCAAUUCCUUACAUUUUAUAGAUAAUGUAGAGAUAGAAAUAGUGGCCAA

>N149

CAACACAGCAAGACCCCAUCUCUUAAAAGAAAAAGAGAUCGUGAGAUACCU

>N150

CUGUUUCUAAAGAGCCCUGACUAACAAUCUCUCUGCCCUUCUUUCUCUGCU

>N151

GGUGACAUCCAUUUGCAGACCUUUCAUUUCUGUGAGCACACCUUUGGUCCU

>N152

UAGAUCUAGUCUUCCGCUGCCUCAGAAGGGUGCCCCGCUCCUGUAUCUCCC

>N153

GGGUCACAUUACACUCCUGCAGAAAAUUGUCAACGCCCUCAGGAUAAAAUU

>N154

UUGUUAACAUCAGCCUUCCCUUCCCACUAAGACAAAAUCAUGCCAUUUGGG

>N155

CAGGAAGCAUCAAAUAGAAGUUAUGAGGGAUAUUAGUGUGGAACUGGGGCA

>N156

AGCUCUGGAACCUUCUCUGCCCACUAGCCUGACCAAACCAGAGCAGCUACG

>N157

AUACUCUUAAACAUUAAAAAUUAAAAAUAGAGCCGGCGCAGUGGUUCACGC

>N158

AGUGCUUUGGGGCUGAGGCAGGAUGAUCACUUGAGGCCAGGCGUUUAAGAC

>N159

AAGAGAGCAAGACCUCAACUCUACAAAAAAAAAUUUUUAAUUAGCUGCAAC

>N160

CUUCUCUAGUUUAAAAUGCAUUCACAUAAUUAUAUAUAUUCAAUUCAAGCG

>N161

GAAAAAUCAAUCCCUUUGCUAUAGCAGGAUGUAUUUACAUCUGCUUCACAA

>N162

AUCUCGUACAUACUUAAAUCCCCUAAUUUAACAACAGUGUCUUGUACCUAC

>N163

GGACAGAUGAUGGCUACUUUCAUCUAAAAAAACUGUUUUGCUUGUUUAAAC

>N164

UUUAGUCAAACUCACAGAAGCAGAGAGUGGAAUGGCAGUUACCAGGUGCUU

>N165

UCCAGCAUGGUGAGUACAGUUAGGAAGAGGGUACGGUGUAGUGGAAAUCUU

>N166

AAUCUUACAUAUUCUCACUGCACACAUUGAAAAAGGUAGCUGUGUGAGGUG

>N167

AUAUAUACACACACACACAAUUUUUACUUGUCAAUAUACCUCAAAAAUAUU

>N168

GAGAGAGGGCGUCUUACCCGGCAAGAGUGACCCAGCAGGUUGGAAGCACUG

>N169

CGUGCUCUGCAGAAGUAUUUGCAGCAUGUCUUCUAACCAGAACAGUGUUUG

>N170

GAGCGAACCUCGUCAGUGCAGCUUCACUGCUCAGACACUGCUUCUGGAGGG

>N171

GAACUGAAUAAAUGCAGCACAGACCAUACGGCCUGCAGAGCCAAAAUGAUA

>N172

ACAGCAGCUGUUACCUGGCAGGCUAACGCACAGAAACGGGCAUUCUUAGGU

>N173

AACUUCAACUUAGUCAUCAAGACCCAUACUUUGGAAAGAAGUCCCUUUCUA

>N174

UGAGAGGCAGUGGUGCCCACCAAACAUUCUAUCACCCUCCCGCUUCCAGUA

>N175

GAGAUUUUGGAUUAAUUUUUAUCGUAACAUAUUCUGGUCUACUCUAACUUU

>N176

AUUCUCUAUCACUUGCAAGGCACUCAUAACACUGGAGAGGUCCUGUUCAAA

>N177

UUCUGAAAGCCACAGAGAAGAAAUGAUUAUCCUAGUGACAAAGUGGUCAGC

>N178

CUGCACUCCAGCACGGGCAACAAAAAGAAGAAUCCUCUUCCCUCCGCAUUA

>N179

GGUCUCCAAGACCAUCUCUGCUCCAAAACUUCUAUAAAGUCCAGGGAGCUU

>N180

AAUGUCAGGCGGGAUUGAAGUGGGAAGGACAUGACCCGCUAGCUUUCUUCU

>N181

UUAAUUAUAAAAAAAUCUUAACAGUAAAAUUUCAAACAACUUUAAAACACU

>N182

CAAGUUCCUUUUUCACUUCAUAUAUACUAUAGCUAUCUUUUCAUCUGAAUA

>N183

UAAGAAUUUUGAAAAAAAUCAUAAUAAUACAUGAGCAUAUUCUAUCUGACC

>N184

GAGGCCAAGGCGGGUGGAUUGCCUGAGCUCAGGAGUUCGAGACCAGCCUGG

>N185

GUAGUCCCAGCUACUCAGGAGGCUGAGGCAGGAGAAUUGCUUGAACCCAGU

>N186

CCAAAAUAAAAUAAAAUAAAAUAAAAAUUCUAGUUAACUGAGUCAUAUUUU

>N187

GUAAUAGACAGUAAUACCGUCUUCUAAGAAUUAUGGGCCUUUAAUUGUGGA

>N188

CCUUUAAAGUAACUUUAACAAAUAUACAAUUUAUUAUAACUUAAAAGAAUU

>N189

UCUCCUCAUGAGGUAAAAUGUAUGGAUUUAGUGAAGAAAAAAUACCAACAU

>N190

UGCACGAUCUACGUGACACGCAGUGAGAAACAUGACUUUGACAUAUCAUUG

>N191

AGUAAGCUUGAAUGUGCACUCUCAGAGGAAGGAGGAGGAUAUUCAGUUGCA

>N192

ACUAGAUUAUCAUUGUUAUUAUUUUAUUCUUCAGAUUACUAGCGAGGAACC

>N193

CUACUGAAUAUAAACUGAAUUAGCCAGGAAUAUAUACCAAAAUAUAAAUAC

>N194

AUGUAAAUCUUAAAAUGUGCCUCCUAAUUACUGCUUUCUGCAAAAGUACUU

>N195

CUGGCCUUAGACAGAAGCUUUACCUAACUUGCAGCAGGGAACAGAGCAGGG

>N196

AGUCUUCCUAACUAUUUGUGGUAUAACCCAUCCUUAACUAUUAAAACAGAA

>N197

GCAAAUUCCUUGACUAUAUCGGGGCAAAUGUAACAGUAUUUCUCCUGCAUG

>N198

GGUAAGUCUGCUUUCUCUGCAACUCAGCUAUUAAUCUCAGCCUUGGUCUAA

>N199

CGCUAUCAUAUCUGAAAUGAUGUCUACAGGUGUCUAGAAAGCACCUCGUCU

>N200

ACCCGUGCUGCGAGCUCAACUACCGAGUGCGGCACUGUCUACACCCUAAAG

>N201

AUUACACUCUAGCUGGGAGUCAGAAAACAAUAAGCAACCAUAAUAAGUAAG

>N202

GGUUAUAAAGUGAUAGGUCCCGGGGAAGAAAGGAAAAGCAGAGUCAGGUGA

>N203

ACCGACAGGCAGCCUGAGGGAUGCGAAGUGUUUCAUGGGUAUCUGGUAAGC

>N204

CGGAAUUUUAAGAAGACUGACAGGCAAGAAGUGCUACACAACCCUGUGUUG

>N205

CAGAGCAACAGGGUCAGCUGAUUACAACCUCAAAUCUACCAAGACUGGAGA

>N206

UUCCUUCAAAGAGAGAAGUCAGGUGAGAACUAACAGGGCUACCCCACAGAG

>N207

AGCAAUCCUGGCGGAAAUAGGGAGGACUCAGACUCACUGGAGUGGACCCUC

>N208

AGCCCUAGGAUUAUAGAUGUGAGCCACUAUGAAGGGGCUCAAUUCUGACUA

>N209

AUACCUCAGGAAACCUUCCCCUGAGAUGGAAGGGACCACGUCUUCCCCACG

>N210

AGCACUGAGAGCCCCCUUCCCAGGAAUCUCUGGUCCCAGGUAAAGCAGGGC

>N211

UCCAGCUGAACAAUGCUGCCUCUGCACGCUGGUACGCUGUACCAGCUACCC

>N212

AGGGAUUCCCACCUCCCUCUCCCUUAGCAGCUGUUGAAUGAAAUACGUAAG

>N213

GGAAUAUGAAAAGAAAAACCACAUAAGAUAGUUCAUUUCCAAAAAGAAAUA

>N214

CCUCAGCGCGUUUGUCCAUCGUAUGAGGCAAACAAUACCCUGCUGGGGCCG

>N215

UCAGAUGAAACUGCGUGUGUCGAGCACACAGCACUGGUUUUGGGGGCUGGU

>N216

AACGCCCACCGCUGGAUCAUCCGGUAAAGACACUCAGGUUUCACGUUACAC

>N217

UACUAAAACUCAACUUAAACCCAUAAAAAUAAUAUUAGCUUUCUAACACAU

>N218

GCUCAUGACAGUGGUAAAAAUAUUAAAACAAGAUACAUGUCCAACAACAGA

>N219

AUGUACUUCUUAAAUGUAUUUUCACAGAAUAUUUAACCACAAGAGAAAAAU

>N220

CUACUCAGGAGGCUGAGGCAGGAGAAUCGCUUGAACCUGGGAGGCAGAGGG

>N221

UUAGUGGGCCUGGACACACUGUGAAAUGUAUUUUCUUUCCUUUUCCUAAAC

>N222

AAGUCUAGAGCACACUUCACAGGGCAGGGCAGCCACCUCUCCUCCCCACAC

>N223

AUCGACCAUUAAGCCUUUCCUCUGCAGUGAUACCUAACCUUCAAGGAAUUC

>N224

CACAAACACACCCCCCCAUAUACAUAUGCAGAAACAUUAUCUGUAACAUAA

>N225

UAGGAAUCACUUCUCUGAAACCAAAAAGUGGUAUUGCAGAGCCUUCUGCAU

>N226

ACAAUCUCCCCUUCUUGGCUUUUAAAUUUUUUUUUUUUUUUUUUGAGACGU

>N227

AGCCACCGCGCCCAGCCCUUAAAUAAAUUUUUAAAACAAAAAGCAAUUUUG

>N228

UGCACCUACAAAUUCAACAUCCAAAACGGUGUCUAUCUCCCAACAUGUCGC

>N229

UGUCCAAGAUGCCGCCAAGGCCAGCACUGCCUGGAGAAAAGACAUGAAAAA

>N230

AGUUUUCAAUAAAGAAACGUGGCUGACUGUGGUGACUCAUGCCUGUAAUCA

>N231

AAUAAAAAAAAGAAAGAAAGAAAAAAAACUAAGGUUUUACAAAGAAAAGCA

>N232

CCAGAAAUUCUAAUCUGGAAGGUACAGGGUAAUUUUUCAACCAAAGAAAAC

>N233

AGCUGCAGGAUGCAGCAUCGCCCAAAUUCAUCUUUCUAUGGAACUUUUCCA

>N234

CCCUAUAGUCCUGAAUGGCAUGGGGACGGGGGAGGGAGUGGAAAAAGGAAU

>N235

UAAAUGAUUCGAACAUAAUUUCUGCAAGAUACUCUCUGUUUUCUGGUGUAU

>N236

UUCUGUCUGUAAGAAAACAUUCACUAUAUUUAGUGUUUACCCAAAUUUCAG

>N237

CAGAACCAUAAACAGGUUAACCUGAAAGUAUGUUAGCGAGGACAUUAUACU

>N238

ACAAGUUACAGAAAAAGAAACUCUUAUCUUCAAUAACAUAACACCUAAACA

>N239

AGAAAAGGGACAGAGAAAAGAGGAAAAACAAAUGGCAGACAAAAACACAUA

>N240

UUAAAAGUCAGCCAAGUGUGGUGGCACAUGCCUGUGAUCCCAGCUACUCGU

>N241

AGGACCACUUGAGCCUCGGAGGUGAAGACUACAUGAACCAAGAUUGUGCCU

>N242

UUCUAAGAAAAGGAAGAGACACCAGAAAUCAGUCUCUCUCUCCUAUUCUCC

>N243

CGCUCACACAGGGGAAAGGCAUGUGAGGACCCAGCAGGAAAGGUGACCACA

>N244

AAUUACAGCAGCCGAGUAGACUAAUACACCCCUACGACAGGGAAAGUACAA

>N245

GAAGAAGGUACGACCCAAGAAUUCUAUAUCUGGCCACAGUGUCAAUCAAGG

>N246

AUCAAAAUAUCUAAUAUUUGAGAUGAUGGAUAUGCUAAUUACCCUGAUCUA

>N247

AAAAAAAACAAUAAAAUAAAAUACUAAGGAGAAAAAAUAUAUAUCCUUAAA

>N248

CUUUAUCCACUCAAGAAGAAUGCAGAAAGUCCAGCAAAAGACUUACAGUGA

>N249

GCGGAGGUUGCAGUGAGCUGAGAUCACACCACUGCAUUCCAGCCUGGGCGG

>N250

CUCCGUCUCAAAAAAAAAAAAAAAAAAAAAGACUUUUAUUUUCUGGGAAUA

>N251

UGUAAAUGUUAUAAAUUUUAAUGGAAAAAAAUGAUAUCAAGUUGAGCAGUA

>N252

UGAUAAAGUAUCAGAGGCAUUAGUAAACAUAAAGUUAUAAAUGUUAACACC

>N253

GGGUAAACAUAUCAAAUUAUACUAAAAUAUGCAAACAUGAGUCCUUUUAAA

>N254

AACACACACCCAAUACAAAGCAAAGAGAAAAAUAAGUCUUCAGUUAAAAAG

>N255

UAAAUAAAGUACAUUAAAAACAUGAAGCAUAACAUUUUAAAAAAAUAGUUA

>N256

CUGAGUCUCCUCAUGGGUAAAGUGCAGGUAAUUAGUACCCCACUCAGAGAC

>N257

UAAAGGCAGUCUAAAUAAUGUGCUUAGAGCAGUACUGGAUACACAGAUGUU

>N258

GUGAGUUGCUAUUACUAAAAUUAUCAGCAAAUACAAAUGGGAAAUUCAACA

>N259

AAAUACCCAUCACUAAACACUUUGGAACAAUUUAAAAAAAUGAACUCUAUU

>N260

CUCUAUGAUGAAUUGUUAAGUGAAAAAGAGAAAGGUGUCUCCAUUCCGUAU

>N261

AUAUCCUUCUGCUUCAACAAUUUGUAAUGAAAACUUUUUUUUUUUUUUUUA

>N262

AGGCACAUGCCGUCACGCUCAGUUAAGUUUUGUAUUUUUAGUAGAGACAGC

>N263

AGGGGCAGGUGGAUCACCUGAGGUCAGGAGUUCAAGACCAGCCUGGCCAAC

>N264

CCAGUCGGGUGGCUGAGGCAGCAGAAUUGCUUAAACCCGGGAGGCAGAGGG

>N265

AGAAUUGUACAGUUAAAAAUGGCUAAUAGGGCACAAAUAGUAUUAGGGUUC

>N266

ACCAACAUAGUGAAACCCCGUCUCUACUAAAAAUACAAAAAUUAGCCAGGG

>N267

AUACCUGUAGUCCUAGCUACUCAGGAGGCUUGAGGCAGGGGAAUUGCUUGC

>N268

UCCAUCUCAAAAAAAACAACAACAAAAAAAGAAAGAACAAAAACAAGUGUC

>N269

UAUGCCUACACACAAUCACCCCUGGAACAUGAACAAUCAGCUGGCUAAGAA

>N270

UUACUACUUUAGAUUUCCUUCAUUUAAAGCACGUGAGUAAGAGAUGCAGAA

>N271

GUGUGUACAAUCUCUCUCUUAAGCUACCUCCUAUCGAUCUGAAAAGCGGGA

>N272

UAUUUAAACUGAGUAGAGGUGGGGAAGUAUCAGGAGAGAAAGGUUAAGAAA

>N273

AGCAAAAACAGACCUGUAGGAAGACAUCACCAACAAGAAUCAGAUUAACCA

>N274

AGAUUAAAUACAAACAUCAAAGGAAAAUGGGUUUUACCAACAUACAAAUUA

>N275

GCACAUGCUUCAACAUGGAUGAAGCAUGAAGACGCUAAGUGAAGUAAGCUG

>N276

GACAGAGUUUUAGUUUGGGAAGAUGAAAAGGCUUCUGGAGACAGACGGUGG

>N277

AAUGUAAAGCAUAUGGCAAAUUUUAAUCCAUUAAAUGAUCAAAAAUAAUAA

>N278

UGCAGAGACUAAUAAAAUUACUAACACUGCUGAACUCCAUAAUCAAACACG

>N279

UUUAAGUAAAAAAGACUCGAAAUACAGGAAAAACUGUAUGCACAAAGAUUU

>N280

UAUAAAACCAAGAAAAUGACUAAAAAUAGUGGAACUGCUAAGCAGGAAAUU

>N281

AGAUUAUCAUAUAGCUGUUAAAAUAAGGGUUAUGAAAACUGUAAGGAAAUC

>N282

UAUUGGGUGUAAAAUCAUUUUAAAAAUGGGUAAAAAGAUUAAAUGAAAAUA

>N283

CAACAGUACCUAUGUUAAUGUGAUAAGACUAUAAAAAUCCCACAGAUAUCU

>N284

AACUCCGAGUUAUGGCUGUGGGGCAAGAUCACACAAACUCCUCAUCUAACA

>N285

AGAGAGAUUGUUAGCCACACCAGAGAGCUGCCAUAUCAGCACUGGACUCUU

>N286

UGCCAAAAACAUUUCACUGUCUUUGAAGAAUUAAAAUCUAUGUAUUUCCAC

>N287

AACUAUUAUUUAUAACAAGAUCUAAAUUGGAGAUGUCCAGUUAUUCUGGGA

>N288

ACCCAUAUUUCUGGAUAAAGAUAGAAAAUUGAACACAUGUAUCUAAUAUUA

>N289

AUGCCCCUCUAAAACUACAGUAAAGAAACAUUUUUAAAAAUACAAAUCCUC

>N290

CAAAUCCAAGAAAGCUGACCUCUACACUGGCUAGGUCUACACUGGCUAGGG

>N291

UAGUGAAGUGCACAAGGCAGGUCUAACCAUUCUCAAGAAUUCAGGAAGGAU

>N292

CAUGUUUCCCAUCUUAACCAAUCACAUUAUCAUCCAUGCACUGCCCCAUCU

>N293

CCUCCUGUAUAUCUCUGCUAACUAUAUGUAACUUACCCCAACCAACUGUAA

>N294

AGCAGGCGAAAUAACUCCAUGAGUGAUACAAUGUCUGCCCGGGCUCCUUAG

>N295

CUCUGGAAGCCUGAUUGCUCAAUACAAUGUCUGCCCGGGCUCCUUAAUGAG

>N296

UGGACUGAGGGCAACCUAACAAACAAUGUGUGCUUCCACACACCAUACUGC

>N297

GUAAGUCAACCACCCCAGCUGGUGCAGAAGAAAGUUACUCCACACUGAGGU

>N298

UUCUCCCACUGACUCAGAGCUGCAUAAACGGGCCCUUCCUGAAGGAUUUUU

>N299

UUGACUAUACAUAUCUCCUUACAUAAUUUUAAGAACCUCGCAAAUGACAGU

>N300

UCAUCUUCAAGACUCUGUGUGCCAUAGGUGUCUGCCCACAAAGUCUCCCUG

>N301

CUCCCCCGCUCUCGUCACCCUAACCAAGUCAGGUCCUCAGAGUCACCACGA

>N302

CGGUUAAGGACAAAUACCUGAAGGAAAUUUACCAUUCUCAUCACUUCCUCA

>N303

GCAACAGAAUGACUUGCAGAAUUCAAAGCACACCACAUGUAAUACGAUAUU

>N304

AUCUCAUGUAUGCCAGCUAAAUCAGAAGAGUCCCCUUUAAUAAAAAUUCCU

>N305

UUGGCCCCAUUUGCUCCACAUAAACAACUGUGGGCCGGGUAUGGUGGCUCC

>N306

UACUAUAAGAUAAUUAAAAUGAUGAACUGGAACUACAUAUGCCAACAGUGA

>N307

UGUGAAAUUCUUCUUCUGGACAAUAAGACUUAUGAUCUCCCCACCAUGUAU

>N308

CCUCCUCUGCUAACAAUAAAUAACCACCUUUUACUGUAAUUUUUCAUUACC

>N309

CUGUCCUCCUGCUCUUUGCUCCAUGAGAAAAUCCACUUACAACCUCAGGUC

>N310

UGGACUCCAAACUCUGGAGCUGGUCACAGACUCGGGAAGACAGUCUUUCCG

>N311

UCACUGCGGGGACGCCUGCCUGAUUAUUCACCCGCAUUCCAGAAGUGUUUA

>N312

CCUCUUAAACUCUCACCUGACCUAAAACCUAAGAGUCUUAUUUUCUUCUGA

>N313

CACCUGCCCAACAGUUUCCUCUUAGAGAGGUGGCUGGAGCUGAGGGCACAC

>N314

AGGGCAGUUCCCCGAGAUGAUCCCCAAAGGGAAUACCAGACAGGCUUCCCC

>N315

GGUGAGAUUACAUGGUCUCUUGCCUAGUUGAAGGGCUAAAAAAGGCAGCAA

>N316

UAGUUAUGACAAGCUUAAAGAAGCCACUCAAGGUAAAGAUGAAAACCCAGU

>N317

GACCCCACUGGAAAUCGGACUGUCCAACUAAUAUCACAGCCACUUCUAGGG

>N318

ACCUCUACUUGUUCUCUGGCAACCGACCAUGCACCCCUUACCAUUCCAUUU

>N319

CUUCAACACCUCUACUCAAAAGGGUACCGAGUAUCCCCUUCAAGGCUCAAU

>N320

UUUCAUUCUCUCAAAAAGGCCCUAGAAACAGCUCCCACACUGGCACUCCUC

>N321

AACCUUUUUCCGUCCACACAGCUGAAAUACAAGGCUGUGCUGUUGGAGUGC

>N322

UCAUAUUUCUAUUUUUCCAAUCCCAAACCCAGACCACACUUAGUUUAUUGC

>N323

GGCCAAGGCUGAGGUCAUUCACUGUAAAGGACACCAGAAACCAACGGAUCU

>N324

GGAAAAUUCAUUCUUCCUUAGCUUAAUCCAGGAGACAAAGAUUAUUUUACU

>N325

CAUCGCCACCUACAUUAAAGCUAAUAUGCCUGAUUACUGUUUUUAGAGAAU

>N326

CUUAGACCCGAAACUUACUAGACUAACUCAUUAUAAAAUUUUCUUUAAGGC

>N327

AUGUCAGGCCUCUGAGCUGAAGCUCAGCCAUUGUAACCCCUGUGACCUGCA

>N328

AGCCAGCUCCCGCCUUAACUGAUUGACCAACCUUAUGACAUUCCACCAUUC

>N329

UGGCCCUGCCCCAACUGAUUAAUUGACCUUGUGACAUUCUUCUUUUGGACC

>N330

UGAUCUCCCCACCAUGCACCCCGUAACCCCCUCCUCUGCUAACAAUAUAUA

>N331

ACUGUAACUUUCCACUACCUACCCAAGUCCUAUAAAGCUGCCUCUCUCCUA

>N332

CUGACUCUCUUUUCAGACUCAGCCCACUUGCACCCAAGUGAAUAAAUAGCG

>N333

GGCAAUCGGUUGAAAGGGACAAGUUAUUAUCCAAAGACCUGGAAUCAAUAU

>N334

UGUUAAUUCCGGUUAGCUCUUCCUGAAAUCAAGAAGGGAGGGGGGUACAGA

>N335

GACUUGGGGACUGUGAAGAAGAUGCACCUGGGUAAGUGUUAGUGGCAGAAG

>N336

GAGGCAGAGGCCAUGCACAGGACGGAGCUCUUCUGGCAAGCACAGUACUGC

>N337

UUAUAGAAGAGAGUGAUCCGCCUCUACCUCAGGGUACUGAUUGGCUCAGAC

>N338

AAAGACAGGGAGAUGUGUGAAGCAAAUGUGGCAAACAUAUAGUUACUCUUA

>N339

CUCCAAAAAAAAAAAAAAAACAACAACAACAAAAAUUAGUCAGGCACAGUU

>N340

GUAGUCCCAGCUACUCACAAGGCGGAGGCGAGCGGCUCACUUGAACCCCGU

>N341

AAUAAUAUUUAUUAUUAUUUUAAAAAUACAUACAAGUGAUGAAGUAGGCAA

>N342

AGCCGAAGAUGCUAGCAACGCCUACAAAUCAAUCAGGAAAAGACAACCCAC

>N343

GUGAUCUGCCCACUUCGGCCUUCCGAAGUGCUGGGAUUACAGGCAUGAGCC

>N344

CCGGCUAAUUUUUUGUAUUUUUAGUAGAGACAGGGUUUCUCCAUGUUGGCC

>N345

UGGCGGCUUUUGAUUCCCAUUUAUUAAUAUUAAACCCACACACCUUACUGA

>N346

UAGCUGUAGUAAAUUUUGUCAGUUAAUUAUUUUGUGUUUCUCACUUAUGCU

>N347

CUAGUGGUGUUGACUAAACCACCAGAACAACGUUAAAUGAUGAUGGUAAUA

>N348

UUAUCUUAUUCUUGAAUUCAACAGGAAUACAUUUCAUGUUUUCCAUUUAAA

>N349

GGGAUGUCUCACAAGAGGGUGUUAAAAAGGACUCUUUAUAGGACAGGGCUU

>N350

GUGAGGCUAAAGCUAUGACGGUGAAAAGCAUUAGUCAUUUGUAUCAGCCAA

>N351

CUUACUGCAGUCCUGGAAAACUAAUAAAGAUGCCAACUUUAAAGUAUACAU

>N352

UGCGGUGGCUCACGCCUGUAAUCCCAGCACUUUGGGAGGCCAAGGUGGGCU

>N353

UCAGGAGUUCAAGACCAGCCUGGCCAAUAUGGUGAAAUCCCAUCUGUACUA

>N354

GAGACUCCAUCUCAACAACAAUGGCAACAACAACAACAAAACAAAGAACAU

>N355

AGGCAAUUUAAGAGUUAAAGUGUACACUCUACUGGGAAAAUCCCCGGUUCU

>N356

ACCACAAGCUCCGCCUCUCAGGUUCAAGCGAUUCUCCUGCCUUGGCCUCCC

>N357

UCCAUGUUUCUCAGGCUGGUCUUGAACUCCUGACCUCAGGUGAUUUGCCUC

>N358

CCAAAGUGCUGGGAUUAUAGGCAUGAGCCACCUCGCCUGGCCCAGAACAAC

>N359

GUCUGUCUAGCUCUGACAUUCCACAAGGCUAUGAAAUGGUCCAGGAAGAGG

>N360

UGUAUCUGGAAGCUCUAGGGGCAGGAGCCAGUCCUAUUUAUCCUUUGUAAC

>N361

AGUCGCAAAGUACAGUAAAGUUUAAAUGGUUUAUUAGAAACUAAUCUAUUG

>N362

UGUAUUGUUAUUCUCUUGUGCAAUUAAUUUCAGAAAGAAUUUAUGAGUGGC

>N363

GAGUGAGACUCCAUCUCCAAAACAAAAACAAAAACAAGAAUGCCUUUUUCA

>N364

AUACAACUCAAAUCAUUGGUAGCUCAUGAAAAGAACUCACUCUUUGACUAA

>N365

UGAUCACCUCGGCUUGUGUUCAGCAAAAAUCUCCCUAACCUUUAUGUGUCC

>N366

GAGGCAUGGAGAUCAACUGAAAUUAAAAAUAACUUGCAUAUGGCUGGGCAC

>N367

AGACCACCCUGGCCAAUAUGGCGAAACCCCGUCUCUACUAAAAAUACAAAG

>N368

UGUGGUGGUUCACGCCUGUAGUCUUAGCUACUUGGGAGACUGAGGCAGGAG

>N369

GGCUUGGCAUUAAUUUGCUUGGGGCAGGAGAAGGGUCAUCUGAAAGAUAUU

>N370

AGGUUGCCCCUGGGAAGACUAGGGAAGCCUGCCCCCGGGCCAGCCCCUUGG

>N371

CAUGUGGAACCAACAUGGGACAAACAGGCAUUUUGUGCUCGUCCAGGGAAA

>N372

CGGCUCCCACACAAUUUGUAGCUGCAGGAGUGGCAGAGCACCAGAGUGGAU

>N373

AUGUUCUCUGGGUUAGGUGUUAAGGACUUGUGGGAGUCAUCAGGCAGCAGG

>N374

UACAGGUCAUACAGACCUUGCUGAUAAAACAGGUUGCAGUAAAGAAGCUGA

>N375

UUAUAAUGCAUUAGCAUGCUAAAAGACACUCCCACCAGCAAGCACCAUGAA

>N376

GGGAACUGCCCACCCCUUUCUCUGAAACUCAUGAAUAAUUCAACCCUUGUC

>N377

UCAAGAAAUAACCAAAAAAUGGGUAACCAGUAGCCCUGGAGCUGCUCUGCA

>N378

AGGUCCGGGUCGCACUCGGGCGGUCAGCCUGGCACGCACAGCGCCGGCGUG

>N379

UGGGUGCCACCGAGUCCGGGAGGAAAUCUAGGCCGCCAUCCGUCCGGAGAU

>N380

GACCACCACCCAACAUGUAUUGAACAAGGGCUUGGAGGUCCUUUCUCUAAU

>N381

UAAACGAUUACCGACUGUUGACCUCACGGCCCCUUCGCUUCCGGCCGCACA

>N382

CCACGCCUGCGCACUCAGGCACCGCAGGUAGGAGGAACGUACCAACUGCGC

>N383

AGAGCGCCCACUACGGCCUUUGGCCAGGCCCCCUGGGCGCGUUCCGGGAGC

>N384

GGCGCUGGGAGAAGCACGCUGGCCAACAAGUUGGGUGGUGGGCACGCGGCC

>N385

UGCUGUUACUAUUCCCGUAAUGUAGACGAGGAAACCGAGGCGUGUGCUGGG

>N386

GGCCGUGCUCAGAGACGGGCUCUGGAUGCCAUGCUCUGACUCCAUUGUUAC

>N387

GGGAAAAGGCUUGGUGAGAGCAGCGAGAAGAAUAGGUCUAAUUUCUUGAGU

>N388

UUCUCUCUGUUUAGGGACAGAGCCAAGCCUUUUCCCUCCCAUCCCCACCUC

>N389

GUGCUUCAGCUUAGAAGCCCUUGGAAUCUUGUCUUGUUGUGGUUGUAGGCC

>N390

UGUCAGCUGUGGUGAUGUGUGUCACAAGUAACUUCUUUUUUUUGAGACAGU

>N391

CAGUGCCAGCUGCACUGUCAUCCUAAAGUACUUCUGUGGAAGAGAACAUGA

>N392

UGUGAAGCAAUAGCCUAAGGUAAACAACACCUUGCAAGACGGGUCUAAGCU

>N393

ACACCUGUAAUCCCAAUACUUUGGGAGGCUGAGGCAGGGGGAUUGCUUGAC

>N394

CAAGACCAGCUUGGGCAACAUGGUGAAAUCCCGUCUCUACAGAAAACACAU

>N395

ACAGAGCAAGACCCAGUCUGAAAAAAAAAAAAAAAAAAUACUUUGGAGGGG

>N396

AAGGAGAAGGGAAGUGAAAAGGGUCAGCAUGUGUGUUGCAAAGUGAUUAGA

>N397

UCAUAUUCCUUUGCCUGGUCUCCUGACCCCACUUCUACGUGGAAGGCCCCC

>N398

UGUUUGGGGUGAACCACAUUGACUCAGGUUUGGAACAAGAGAUGGAGGGUG

>N399

UCAGGCUGUUCUCAUACAUCAUUUAAGAGUUGCAGAGACAGCCUGAGAAUU

>N400

AAGUUUGUAAACAAUAAAACCCCAAAAAAACAUGGAGAAAGACCAACACAA

>N401

CAAACUAGGCCACCUAGCAGUCACCAACUGCUACAAAGUCCAGACAGAUAA

>N402

CGUGCUUGUCCCAUUCUAGCCCAGCAAGGGAGGUUUGUGGUGAUCCUAUAU

>N403

UACAGGGGCACUGGUCUUAAAAUUUAGCGUUCCCAAGUCACGUCUUUUACG

>N404

GAAGCAAGUCCCAUGCCCUAGGAUUAGGAAACCCUCACCUAACCGUGGUGU

>N405

CAGUUGAAACCUAGUCUCCCGUGAAACAUUUUCAAGGUCGGGGGGUUUCAC

>N406

UGGCAGAAAAGCCAAGUCUCUGGCAAACAUGAGGUAGUUCAAUAGGCUGGG

>N407

AGUUGCUCUUCGUGGUUAGAAAAACAACCAGAAGUAUUCCUGCUAUGCAGU

>N408

CUGCCCAUGAGGAAUUUCCUCCCUCAGAAGGUAGUGGCUGUGGGUUGGGUC

>N409

CAGCUCUGCACCGACUGUCAGCUCAACAAGCUCUUCUACCGCGAGGAGUUG

>N410

UCUUUGAACUCCCCAAGUCCCCUGCAAAGCCCCAGGGGUCUGUGCGUGGAC

>N411

CUCUGGGGCCUUCCUGCUGGCCCAGAGGGAGAAGGGCUUGCUGGAGAGCAA

>N412

GCUUUGUACAUGUGAUGCUCUGUUAAAACAUAAGUGGGUAUGAAGGUCCUG

>N413

GGAUGUGGAGUGGGCAUGUGCUGGCAGGUGGGAUAUGUCCUCAGCCUUUGA

>N414

UAGCCAUUUCCAGAGGGAAGUACACACAUACCCCCACACACCAGUCUUUUA

>N415

GAGCCUGUUCCCUUGCCUCUCCUGGAUGGGUUGUCAGCCCUGUGUUUCACG

>N416

CAUUAUAACAUUUGGAUAUUACUGAAAAUGUAAGGAAGCAGGAAAAAUUAU

>N417

UGUCUUUAGUUGUUCCAAGUGGGGUAAGGGGAGUGGUUCUGUUGGUGUCUG

>N418

GUCCACCCGAGGCUGCUCUGGGUUGAAGCGUUCCUCCCCCUAGACUUAGCA

>N419

CCUGUUUGCAGUGUGCAGGUGAUGGACCAGAAACACAGCAGAGGUCACUGA

>N420

GCCUCACCUGUGCCCAGGACUGCUCAUCCAGUGGCUGUGUCUCCAGCCUCG

>N421

CACCUCUGCUGCUCGUGCAUAGGACAUCUUCCACAUUCACUCCUGAAAUAC

>N422

AACUCUGUCUCAAAAAAAAAAAAAAAAAAGAAAAAAAGGAGCCCCUGGCAA

>N423

UAGGGCCUGUAUCUUUUGUUUUCACAGCAGACAGGUUUCUAGUGCCUGGUU

>N424

UUCCAGUCUCUUUUGGAAAGUAUAUAUAAAUAUUUGAGGUAUAAAUAAAAC

>N425

GGCUCUGGGCCUGGUUUCCUUGGAGAGGGGAAAAUGAAAAGUGUUGAUAUA

>N426

CACCGACACCCUGCAUCAGAUCUGCAGGGCAGGCAUCACCCCGCCGGCCUU

>N427

UAUUUUCCUCACUGCUUAAAUCUUGAGGUCCCUCCACCAGGCCACCCCUGU

>N428

UGUCCAGCUGAGUCCAGAGCAAAAGAGCUAGCUUUGGCGGGCAGUGAAGUC

>N429

GUUUUCUCCUUCAGGACUAAAGGCCAACUUCUUAUGCUCUACAGCUCCAAA

>N430

AGCUGACCCAUAUAAUCUGGAAACAAGUUUGAAUUCAUUGCUGUUAGAAAC

>N431

AUACUGAGUGAAUCAAACAAGGCACAUGUGCUCUCGUUUGCAUUGCAUAUA

>N432

UGCCUGGCUGAGAUUGUUGGUUUUCAUGGUAAAAGCAGGGUCAGACUCCCU

>N433

CACCCCAGCUGCUGAAGUCACGCACAGGCGAGUUCUCCCUGGAGUCCAUCA

>N434

CUUGCAGAGUCUCAAUGCCGCAGGCAACCUACUGGCCACCCCGGGCCAGCA

>N435

CCCCCACACUCGUCUUAGUUGCUUCACACUGGUCACUGGCCCUGCACACUG

>N436

AUUAAAUGCUUCUGUUUUCAUUUGCAUCCCUGCCUGGCAUUUCAUGGGGGU

>N437

UGCCAGCAUCCUCCACAUCCCCUCCACAUCCCUUGGAGAAUGUCCAAACAC

>N438

AUGGAACUUUCAUGUGUGUGCAGGCAGGAGUCCUUCCCAGAUGGGUUGUGU

>N439

GGGGCCUUUGCAGAACCUGUAAUACAGAGCCCAGGCCGUGCUCACAGAGCU

>N440

GGGGUCUCUAACCUCAGGGCUUCUUAAUGGACCUCCCAAUUCAUGCAUGCU

>N441

UCCGGCAGCUUGUCUGCUGCAGCUUAAAUUCCAGCCUCACAAAUAUUCCAU

>N442

CUGCGGACAGACAGGCAGGCAGAGGAUGGCCGGUAGCCAGCUGAGGGUGCG

>N443

UCCAAAGGGCUGGGGCUCAGAGCCCAAGCACUUCUGUCCCCCUUCGUGGGC

>N444

ACCCAGCCACAGGGCCAGAGGGAUCAGCAGCCGUCGCAUGCUUCCGUGUCC

>N445

CCUUUCCCCGCUCAGCCUUCUCCCCAACCGCUACUCCCUCCCCGUCCAGAU

>N446

UGGUCUUGGGACCGGAGAGCGACAGAUGUGGAAACCGAGGCCCCUCAGUGG

>N447

GGAUGAAGAAGCUCUGAGACUUUGCACGUCAGUAACCACCAACACCAUGCA

>N448

ACACCGCAGGGAUCACCUGAAGUAGAGGAUCUCAUGUAACUUCAUGGCUCC

>N449

CUGUUUCUUUAUCUGUAAGAUGGGGAUCAGAAACUCUGUCUUAAGGAGUUU

>N450

CACUACUUAGGGUACUUCACUCCCCAGCUGUGAGCUACUCAGCCGCCCUUC

>N451

UUUGUGGAGAGAUUUGCAUCUGUAGAGGAAAUCUGCAUUGAUGUAGCCAGU

>N452

GGCUCUCCCUUGUCUAACUCUAGGAAAGAUUAACUGAGAGGCUGACACCUA

>N453

UAUCUGAGCCCCCAUAUUUGUAUAAACUCAAGAUGGUGGAUAAGCUUCUGC

>N454

AAACUGUACUUCAGCCUCUCUGGACAGAGCAAGACCUUGUCUCUAAAAUAG

>N455

AUACUUAAAUGGCCACACAAAAUUCACUUUGGGUGUAGGUAUGUGAGGUGU

>N456

AUAUUCAUGAGCUCAGCUCCAGGUAACAUGUCCCAGUCCAGGGGAGCAACC

>N457

UAAUAUUUGCAACCACAUUGACAAAAAUAGUUUUUUUUAGAAGAAGUGAUU

>N458

GUAAGAGCAUAUCAGUUGCAAAACUACACCUAUUGAAGUUAAGUACAUUUA

>N459

UGUAAUUAUUUGUCAGUAUUAUGAAAAUAUUUAAUUUGAAAUGCUUAUAUU

>N460

GGAGUUCAAGACCAGCCUGGGCAAUAUAGAGAGAGGCUAUCUUUACAAAAA

>N461

ACGAUCACUUGAGCCUGGGAAGUCAAGGUUGCAGUGAGCUAUGAUCAUGCG

>N462

GGCCACAGAGCAAAACCUUGUCCCCAAAACAAAUAAUAAUAAUACUUAUUU

>N463

GGUAAUCAUGGCUUCUGUCUCAAGAAAAUCUUGGAGUUAGUUAAAAGUACU

>N464

GGAGAACUCUUCUACAACCCUGACAAUGAGCUUUGGUAAAGAAAAGAUCAA

>N465

AUCUUUCAUUGCUUUCAACAGUUCCAUAAAACCAGCAGUGAUUCAUAGCAU

>N466

AUACGGAAUUAGUUUUAAUGGCUGUAGCCAUGAGACUUGCAUAGAAUAAAA

>N467

UCUAGAAGGGAAAAACAAGAAACUUAUAACAGUGUUUGCCUGUGAGGAGAU

>N468

GUGGAUAAUUUGUCUAUUAAAAUAUAAAAAAACUUUAAAAACUAUUUUUUU

>N469

GGCUGGAGUGCAAUGGCAUAAUCAGAGCUCACUGCAGCCUCCAUGUUCUAA

>N470

AUGUGGCAGGGACAAAACUGGAGGCAGUGAAGAUGGGAAAACAAAGUCCAA

>N471

AGACUCAGGAGGGCAGUGAGGAGAGAAGUGUGAGGUGUCUGUGGAACAUGG

>N472

AGAUCGAGAUCAGAGCAGAGAUGAGACUGGGGAUUGGAGAAGGAAGUAGCC

>N473

UGCUCCCCAGAAAACUUGUUUUGGAAACUCUUUUUUCCUUCCUUCCUUCCG

>N474

CAACAGUGUUGGGAGGUGGGGCCUAAUGAGAGGUAUUUAGAGCACGAGGGG

>N475

CCCCAUCUCUACAAAAAAAAUACAAAAAAAAAAAAAAAAUUAGCUGGGCAA

>N476

AAAUAAAUAAAUAAAUAAAUAAAAUAAAUAAAAAAUAAAAGUGUUUGAGGA

>N477

AACUCCUGCUCAUUAUAAAUUACCCAGUCUCAGGUAUUCUGUUGUGGCAAA

>N478

UUCUGAUGUGAAAUCCUGAGGUUCCACUUUUAAUCACAGCAUGUGAUUGUU

>N479

UAAACAUGAUCAUAAACAAGAAAUAAAACAUGUUGCAGAAUUGUUAAAAAA

>N480

CGAGACCAUCCUGGCUAACAUGGUGAAACCCCGUCUCUACUAAAAAUACAU

>N481

GACAGAGCAAGACUCCGUCUCAAAAAAAAAAAAAAAGAAAAAUAGUGAGAA

>N482

UGGAAACAAUAAAAUAAUAAGUGAUAGGAAAUGGGAUUAAUAUACAAAAUC

>N483

CUAACAUGAAUAUAUUUGGAAAAAAAAUAUAUGUAUAUAUAUAUAUAUAUU

>N484

UAUAGUUUUUUAAAACCCCAUUUACAAUAAUAAAAAAAGGUUAAGUUCUUA

>N485

UAGUAAAAAAAUACAAAGACUAUUUAAAAAAACUAACAACAACAAAAAGAU

>N486

CUUAACAUAAUAUUAAGGUAUAUGAAAGAAACCUUGACUAAAUAGAUAGAA

>N487

AAUCAUAAAAAUGUAAAUCAUUCCUAAACUAAUAUAUACAUUUAACAAGAA

>N488

ACUCCGGUCUGGGCAACAAGAGUGAAACUCUGUCUGAAAAGGAAAAAAUAC

>N489

CAGUGACAUUAGCAAAUGAAUAGACAAAAAAAUGGAACAGAAUAGAAACCA

>N490

AACGUUUCUCCAUGAUUAAAAAAAAAAAAAUCAGCUUAGGCUGGGCAUGGA

>N491

GUUGUGUGCUAUGACCGCCCCUGUGAAUAGCUGCUGCACUCUAGUCUGGGU

>N492

UGUUUCAAUUUGUGUAAAAAAUAGGAAUGUAUGCACAUGCUUGCACAUCGA

>N493

GCCCUCCCAGUUUCUCUUAUUAUUAACAUUUUGCAUUAGUUUGGUAAAUUU

>N494

UGUAGCACAUUCCACAAUGAGGUGUAUCCACCAUUACAGUAUCAUAGGAAC

>N495

GAGACGGAGUCUCGCUCCGUCACCCAGGCUGGAGUGCCGUGGGGCGAUCUU

>N496

ACAUGCUCACGUUUGCAUGUCUGAAAAUAUCUUUAUUCUACCUUAUGUUUU

>N497

GUAGCUGGGAUUACAGGCCUGCGCCACCAUGCCUGGCUAAUUUUUGUAUUA

>N498

CUCGGCCUCCCAAAGGGCUGGGAUUACAGGCGUGAGCCACUGCGCCUGGCU

>N499

AAAGUUCCAUUCAUCUCCUUAUUUCAGCUGUGGUUUUGGUCUCUCACGUUU

>N500

UAAAAAGCCUGAUUGAAAGCUUCAUAGGAAGGUGGGGCCCUGUUAAUUGGC

>N501

GCAGGGUGAUCAUGCAGCGAGGCUGAUUUUUUGUUUAUUUAAAUGAUCCCU

>N502

CAUUUAGCACAGAGACUUUGACUUUACCUCCCCCUAUGACCCUUUCGCCCC

>N503

GGCACGUGCCUCAGGAGGCUGAGGCAGGAGAAUCGCUUGAACCCAAGGUGU

>N504

AAAUUUUUUUUUUCAAAACUAGCUGAGCGUGGUGGCGCAUGCUUGUAGUUC

>N505

GGAGGCUGAGGUGGGAGGAUCACUUAAGGUCGGGAGAUUGAGGCUGCAGUA

>N506

ACUGGAGCUGCCAUGUGGCCAAAGAAGAUUUAUGGACAGAAAAAGGAAAGG

>N507

AUGCGACAGGAGUGGGUGGGGAUAAACAUAUGUAUCUAAUCCACCAUGUUA

>N508

UAUGAGUUACUUAGAAAGACAAAUUACUACAUUUAUGUAUAUAUAUACAUC

>N509

AACCCAGCUAUGGUCUGUGUCCUCUAAAGUUCAUGUGUUGGAAAUUUAAUA

>N510

AGGCCAAGGCGGGAGUAUUACUUGAAACCAGGAGUUCGAGACCAGUCUCAG

>N511

AAGACCUCCAUCUCUGAAAAAAAAAAAGCUUAGCUGGGCAUAGUGGUGCAU

>N512

UCAAAAAAAAAAACAACAAAAACAAAACAACCAACAAAAAAACUACCACUC

>N513

GUUGGUAUACAAUCUUCCACUGCUAAAUUUGACUGCCUUUAAAAAAUAUUA

>N514

CAUGUGCCUAUUUGUUAGAACCGUAAACCAGCUCAGGUCAUAGAAAAUGAC

>N515

UACACAUAAUAUAGUAUAUUUAAUAAUAUACUAAGAUUUAGUAUAUUAAAU

>N516

UCUCCUGUUUUAAGAAAGUGAAGGCAACCCAGUUAGGAGGCAUCUGGGACU

>N517

UCAUCUUCUGACUGAAGUUGAAGAUAAAACCUGAUACCAGUUUGGAGUAGU

>N518

GUAUUGUAAUCAUAAAUGACAUUUGACCUUAAAACAGUAACACAGCAAAUA

>N519

UAUAUUUAAGGCCCUGCUAGCUAUAAGCAAUCCUUCAAGGAAGAGAAUUAG

>N520

GCCAAGGGAAAACAGUUUGAAAUUUACUGCUAACAAUUGGUUCACAUACAA

>N521

CAGGAGUUCAAGUCCAGCCCAGGUAACAUUGUGAGACGCUGUCUCUUAAAC

>N522

AAAAAAUUGGUUCACCCUGCUGUAUAAUGCAUUGGCAAUUCAGUGUGGUCA

>N523

ACCACACACACAAUCCACCAUACACACAUACCACACACACACCCCACCACC

>N524

ACAAAAUCCACCACACACACAACCCACCACACACACAAAAUCCACCCCACC

>N525

AAUCCGCCAAACACACACACAAUCCACCAUACACACACAACCCACCACACC

>N526

ACACAAUCUACCAUACACACAUACCACACACACACACCCCACCACAUACAC

>N527

CACACAGAAUCCACUACACACACACAGUCCACCACGCACACAACCCACCAA

>N528

CACACACACACAAAAUCCACCACACACACACAACCCACCACACACACACAC

>N529

ACACACAACCCACCACACACACACAAAAUCCACCACACACACACAACCCAC

>N530

ACCACACACACACAAUCCACCAUACACACGCAAUCCAUCACACACACACAC

>N531

ACACAAUCCACCAUACACACACACCACACACACACAACCCACCACACACAC

>N532

CAAACCCAUCACACACACACACCACACACAAUCCACCACACACACAAACAA

>N533

AUAUCUUAUGAUAGAGUAUAAGAUUACAAUUUAGAGCCAAAGUUAGGAGAC

>N534

UGCUUAGGCUAGAGUGUAGUGACAUAGUUAUGGCUCUCUGUAACCUCAAAU

>N535

GGUGGCUCACGCCUAUAAUCCCAGCACUUUGGGAGGUCAAGGCAGGAGGAU

>N536

CAGGAGUUUGAGACCAUCCUAGGCAACAUAACGAGACCUUGUGUCUACAAC

>N537

UGACAGAGUAAGACUCUGCCUCUAAAAAAUAAUAAAAAUAUAAAAUAAAAG

>N538

GGCCGGCAAAGUGACAAGUGUAGCAAGAUGCUGCGCUGAGGGCUUGGGAGA

>N539

GGCCUCGAGCCCAAGAAUGCCUGGGAAGCAGCUCUUCCAGCCAGAAAAGGG

>N540

GUGUCUAAGGGGAGAACUUGUAGAAAGGUAAGUAACUGGCAGAUAAAUAGA

>N541

AGUGGUUAACUUUUCCCUGGUCGAGAGAGGGAGCAGACCCCUUUUAUCCUC

>N542

AUCCUGCAUUUAGGCAGUGGAGGGCAGAGAGCUUUCUUCCAUUUACUUUUU

>N543

CACAGUCAUCCAUCCAGAAAUUAUCAACAAGCGCUUGUUUUUAUUUUGUAC

>N544

UUCUGAACAAUACUUGGGUGUCUUCACAGAGGAUUUCUGGAGACACUGUUC

>N545

GCCCUUUCUCCGGCUAGCUUGUCAAAAGCUGAGACCGUCUCCACUGGGCUA

>N546

AAGGCCUUAAGCUUCCAGCUGGCAUAAAGGCUCAGGAAUCCCUUCGCAACC

>N547

UCACUCGUUAACCCUGGGGGAUCCCACGCUUACCCUGUGCCCAGGCCAGCC

>N548

CUGAGUGGCCUUGAGUAAGUUCCUGAACAUUUCAGAGUCUCGGUUGUUACG

>N549

AAAGUUGGGCUGGGUGCGGUAGCUCACACCUGUAAUCCCAGCACUUUGGGA

>N550

CCCCAUCUCUACUAAAAAUACAAAAAUUAGCCAGGUGUAGUGGUGCGUGCA

>N551

GCUACUAGGGAGGCUGAGGCAGGAGAAUCACUUGAACCUGGGAGGCGGAGA

>N552

ACAUUGACAAGAAAUUCUAAAGUUCAUUUUGUUACAAAACUGAACUGGGGA

>N553

ACAAGCAAGGUUUUUUUUUUCCCCCAGUCGGAGUCUAGCUCUGCCGCCCAU

>N554

AGUGGCAUGAUCUUGGCUCACUGCAACCUCCGCCUCCUGGGUUCAAGUGAC

>N555

CAGCCUCCCGAGUAGCUGGGAUUACAGGCUCCUGCCACCACGUCCAGCUAU

>N556

GUGAAUUGAUACAUGGAGGUUAUGCACUGGUUUAGCCUCAAAGGGCAGGGC

>N557

AAGCUUUGCCUAAAACCUUGGGGUUAGCAGAAAGGAAUGUUCAGGUCUGGA

>N558

UCAGGAUCCUCAGGAAGAAAUUUAGAACAAAGAACAGCAGAGCUUAGUCCA

>N559

UUGUCUGAGGUCUGCAGGCCAGGGCAUCCACUUGGGAGGGGGGCCCAGGUC

>N560

UUUUAGGUUGCGGGUAGCCUCUCUAAACAUAACUCUAAAGGCAGAAACCAG

>N561

UGCUAAGUUUUGGCACAUUAAAACAAAACUUUCUCGCCGGGCACGGUGGCC

>N562

AUCUCUACAGAAGAAAAACAUUAAAAUAAAAUAAUUAGCUGGAUGUGGUGC

>N563

CAGUGAGCCAUGAUAGUGCUACUGCACUCCAGCUCUGGUGACAGAGUGAAU

>N564

AAAAAAAAAAAAAAAAAAGUUAACAAGUACGAGGAAACACGUCUGCAACAA

>N565

AAUGGGCAAAUAGAAAAGUGGGCAAAUGGACAUAAACAAAUUCACAAGAGA

>N566

CAAAAGAUGAGAAAUAACCAUUUCAAACUAAAUUUUUUUUUUAAAAAAGUA

>N567

AGUAUAUGGAAAAUGGGCAUUUCUCAGGGAAAACUAAUACGUUUUCCUGGA

>N568

UCUAAUAAAAUGGAAGAAAAUUCACAAGAUCGUGUUAAGUGAAGAAGGCAA

>N569

CGCAAGGCAUUAUUCCAAUUAUGCCAGACAUGCCAUGGAUAUACGUAGAGU

>N570

GGCAACAUACCAAAAUAUUAAAUGUAAUUGACUCUACGAGGUAAAACUGAA

>N571

AAAAUUGCCUGUGUUGAAAAGCAUGAAAGUACAUGAAAUUGUACUACCCUA

>N572

GCACCUGCCACCGCAGACGCCACACAGGCUGCUGAGCCUUUCCAAGCGAAC

>N573

ACUUCUGCUCUUUCUUAAUUUCUAGAAAAGAGUGAAGAGAGGCCUUUUCAA

>N574

UCCUGCCAUAGGCCGGCCUUUGGCCAUCAGGGAAAGCCCAGACUCUGGCCU

>N575

AAUUCGUUCCGUGACCUUACCAGGGAAGAAAGUGACCCCGAAAGACAGCAG

>N576

UGUUGAAGGGGCACAUUCAUUUAUUAAUCCAAUGUCCAUUAAGGUCACUCU

>N577

UGCAGCCGCGGAAGUAUCCCCAGGAACAUCCCCAAGAGGAGCUGGAAAAAC

>N578

GGCAGGGGACUGAGAUUGCAGUCUCAUCUUCCCUCCCCUUCUAUUGCCCCG

>N579

GCAGACACAGGGAGAGGGCAGCAUAAGGCACUGUAGGGAGCAGUGGCCACA

>N580

AGUCCUGUGGCAGGAAUGUUUGUGCAUUCAGCACUGGAGCAUUUACUCCCG

>N581

UACAUACCACACAAGGAAACAGGAGAGUAGUCUAGCUCCAUGUCCGGGGCA

>N582

CAGUGGGACCAGCUAGUUGCCCACAAGCGGGUGCACGUAGCUGAGGCCCUC

>N583

CACAGCAAGAUUCACAAGCGAUCCGAGGGGUCGGCCCAGGCCGCCCCCGGU

>N584

CCCCCCUCCCUCUACAGCUGCGACGACUGCGGCAGGAGCUUCCGGCUGGAC

>N585

GGCAAAGCCUUCAGCCAGAAGUCCAACCUGGUGUCGCACCGGCGCAUCCAC

>N586

GACGAGGAGAGACUCCUGGCCCACCAGAAGAAGCACGAUGUCUGAGACGGC

>N587

AAAGGAAGACCCUCCAUCCUCUGGUAUUAACGCCUUAAUGCCCCUGUCUUG

>N588

GCCUCUGCUUGAGCACUUAGGUGACAGGAACUUCCGCACCUCCUGAGGCCG

>N589

CCCUCCUUUUCAAACUAGAGAAUAAAGAUUUGGUUUUAGAACUGGUGGCUC

>N590

CCUGCCUCAGUUCUUCCCUGAGCAUACCCAGGCAGUUGGGCUAAUUCUCUC

>N591

UUAUGAAACUACAGCAAGCACUAUGAGCUCUUUUGAGGGGUGCUGGGUGUU

>N592

CACCAGGCGAGGAGAAUGGGCAGCUAAGGCUCAAACUCCCUGAUGGCUUUG

>N593

ACAUUGGUGGGGGUCCUGGCUCCUGAAAAACAACUCAAGGAUAUAUGUCAG

>N594

GAUCACCUGAGGUCAGGAGUUCAAGACCAGCCUGGCCAACAUGGUGAAACG

>N595

GGCAGGUGGAUCACCUGAGGUUAGGAGUUUGAGACCAGCCUGACCAACAUA

>N596

AAUUAAAAAAGAGGGUCUCUGCUCCAUCUCAAGGUCAGGUCAGACUUUUCA

>N597

UCUGCAUAUAUCUCCAGCAUUUAGCACAGAGCACAUAAGAAUAACCACAAU

>N598

GAGUGGCACCUGGUGAAGUUUACCUAAAUUAUCUCAUGUGUCCUUGUGUGU

>N599

CAAAUUCAAGUCUGUCUGACUCCUGAAACCUGUGCUCUUGACAACCGCUGU

>N600

UUAAAGUGUGUAGGAUGGAAAGAACAGCUUCAAAGCUUCAUAUUGGUUUAC

>N601

AGCCACUUAAAAACCUGUGUAGUUGAUGGCAAAUUAUCUGUCAAAGCCUGG

>N602

GAAGGGGAGAUGGCCAGGAAAGAGUAGGUCGAUGUCUGGAAAUGAUGGGCG

>N603

AGAGGGGAUCUGACCAGGGGUCCUAACCUUUUGAAAAGUCCCGUUUAAAGG

>N604

CUUUGUUUUCUUCUACAAUUACAAAAGCAUUAAAUUAUCUUUAAAAAUAAU

>N605

AGAAGGAAACAGGCCCAGAGAGUGGAAGUGACCCCCACCGGUGGGGGUCCA

>N606

AAACAACUCAAGGACAUAUGUCAAGAUGUCAUCUUCAGUUUCUAUAAGGAA

>N607

CUCAGCAACAGGAAUGUAUUCCACAAGCCUUCGUAGAACCUCUUCUAGGUU

>N608

CCUUGAGGCCAGGAGUUUGAGGCUGAAGUGCGUUAUGAUUGUGCCUGUGAC

>N609

ACUUUAACCUGGGAAACAUAGCAAGACCCCGUCUUUAAAAAAAUAAACAGU

>N610

UUACAAAUCCUUCUAUAGAUAAUAUAACCAAAUUAACGGUAAAAACUCAGU

>N611

GCCUGGGCAACAUGGCGAGACCCCCAUCUCUACAAAAUAUUUAAAAAGUUA

>N612

AGCGAGACCACCUCCCUGAAACAGAAAACGAAGGGGUCGGGUAUAAUGAUG

>N613

CUCGUAGUGAACCCUAAAAGUUAUCACACCCCACGGUGGGGCUAUCGCGGC

>N614

GUCCGGCGCGCUGUGGCGCUCGGCGAACGUGCGCUGAGUUCAGGCGUUACC

>N615

GGAGGCUAGUGAGAAUGGAGGGAGGACGCGGUGGCGAGUAGAGGGGUCGCG

>N616

AUCUGAGCUGGGGCUGGGUGUGGUCAGAGCCCCAGGGGUUGGAGGCGGACG

>N617

GGGCUGGGAAGGGGAGAACUGAGCAAGGGCCUUCCAAGGUUGGUCACAGGU

>N618

CGGUGAGUUCAAGAGCCACGAGAGGAUGGGGCGUGUCGGGGUGCAAUGGGA

>N619

AGAGUUGAGUUCUCAGUUCCCUCUUAUCCGAGGUGUGCGUGCCAGCAAGUC

>N620

GAACUCAAGAUUUUCCUUUAUUUUCAUGAUUGGAGGGCACAGCAGGUGCCA

>N621

CUGCUCCAUCUCACUUUGCUUUCCCAAGGUCCUUUGAAUGCAGCAAGGGAC

>N622

UUGCAUGCAGAUCAGGUCUCCUGGGAAUGUCGAGGGGAGCAAACUGAAGCC

>N623

GCUGGCUGUUCAUACCCUGAGGUACAGUUCUUAUAACCCUGAGUGGGGAGG

>N624

ACACUGUUUGCGUCCCGCCAUCCCCACCCCCCACCGCCCCCGCCCCACUUC

>N625

UGAGGUCUGAAGAUGGUGGAGUAGGAAGAGGGGAAGGGUCAGGGCUGGGAC

>N626

GGGGGUGGGACAGGGGAGUUGUGGGAGGCUUCAGGGUGCCUGAUGCUGGGG

>N627

CAGGUGGCGGCUGAGGGUCACCCAAAGAUGGCUGAAGGGGAGAGGGCCCGC

>N628

CUGCUGACUUAUCCCACCCCAGCGCAGGUCAUCCAACGGUGACUUAUGGCC

>N629

AGGCUAUAGAAAGGGGCAGCAUGGCAGGGAGGCAUUGGCUGGGCAUGGGCG

>N630

AGGCCAGUUUCUGUGGGGGUCUCCUAUGGAAGACCAGCUGAGCUGUGCCCG

>N631

ACCUGCUGGGACCUCAGGGCUGAGAAGGGCCUGAUACUGGGCCUUUGGCUG

>N632

AGCUGCCCCCACCCCCAAAAGCCUUAGCGCCUAGUGGCGGAUCCUGCUCCG

>N633

CUGUGUUUCCCCCUUUGAAUUUCUCACUUCUUCCUCUUGGGUCCUCUUCCU

>N634

CUACCUGGAGUGGCUGUUAGGUCCUAAUACCUCUUGGGAGGCAGGGUGGGA

>N635

GGGAGGAGCCCCCAGCACCCAUCAGAGCCCCACCAGGGGCUCCUCCCGCCA

>N636

GGAGCUCUGAGCCUGCAAUACCCCGAAUGACUGCAAAGACGGGAGCUCUGG

>N637

AGCUAAUUGCCUUUACCCACAGGAAAAAUAAAGUUCUUAUAUUUUUAUGAU

>N638

AGGCAGGGGUGAUGUCUUCCUUGAUACUUAAAUUUCAAAGAAAUGGCCCCG

>N639

GCCCAGCCUUAUUUAACUUUUAAAAAGAUUUAUUUCUACUUGAGGAGCAUU

>N640

UUUCUAAACAAAAUGCUCUUGAAAAAAGUGGGUGUCUCUUCUUCUCUUUGG

>N641

AUGACUUUCUGCAUUUUCUAGAUUCAUAUUCACCCCUCCCCCGACGUCUCA

>N642

GCUCCUGACCUUGCUGUGGUGGGGAACAGAAAAGUUAAGAUCAUGUCAGGA

>N643

AAAUUAGCUGGGUGUGGUGGCGCACACCUGUAAUCCCAGCUACUUAGGAAA

>N644

AGGCGGGGAUCUGCGGUGAGGGGACAGCCAGGGCAAAGGCGUGGGGCAUCC

>N645

GUGAAACCCCGUCUCUACUAAAAAUACAAAAAAAUAGCCAGGCGUGGUGGG

>N646

AAAAAAGAUGAAUCCUACAUCAGUGAACUGGGCAUGGGCUCAGCAAUGCCA

>N647

UGGAUAGAAAAGCUCUUUGGAAAAGAGAGUGCUUUAUGGGAUGGGAGGGGA

>N648

UUUUGGAAAUUCCAGUGGAACUCAGAGCACAUGUGCCCAGCGUGUUAUAGG

>N649

UCUGUGCUUUGCACCUUCUAAUUUUAUCUGAACCAUUAAAACCUAAAACAA

>N650

AUGAUGACUGAGGAGCGGUGCCAUUAUUUCGGGACUCUCCACGGGCAGGCC

>N651

ACUCCACCUCAGCUCCAGGGCCUCCAGGGAUGCGUGCUGGACUUAGCACCC

>N652

CAUGCCUGUGUGUAUACAUAGAGACAUAUGGUUUAAUUUUUUAUGAAUUCU

>N653

GACUAGUGGGGUUGAACCUCUUUUUAUCACGUAUAGGAGUCUGUUUUAUAU

>N654

AAGCGUUUGUCUAUUCUAUAUCUGCACAUAUUUUUUCCUGGACUUGCACUU

>N655

UUAAUUAAUUUAAAUACAAUUAAAAAUGUCAUUUCUUAGUUGCACUAGUCG

>N656

GCUCAGUAACCACAUGUGUUUCAAGAGCUCAGAUGUAAACUGUUUUUGCUU

>N657

AUUUUUAUAAACAAUCUUCACUAUGAAUCUAACUUAAAUAUUUUCCCCAAG

>N658

UAAGUCACCAGGCCCACCAUGGAAGAGCCAUGCCAAUAACAAUAACAACUU

>N659

AAGCAGUCCUCCCACCUCAGCCUCCAGAGUAGCUGGGACUACAGGUGUGCC

>N660

UUCUCUCUGUAGUUUUGCCCUUUCCAGAAUGUCAUAUAGUUGAAGUCAUAA

>N661

UAGCCAUUCAUAUGUGUACAGUGGUAGCAUUUUUGCUUUUAAUGUCGUAUC

>N662

CUUAAAUAGGAUUACACUGAAUUUAAACAUUAUUUCAGAGUGAAGAACAUA

>N663

AUCCUUCCCUCCAGAAAUAAUGUUGAAUUUUAUCAUAUGACUUUUUCACAG

>N664

UGUUUGUGUCUAUGUUCAAAGAAUGAUAAACCUUUUGCCCACUUCCAUUCA

>N665

AGCCUGUUACCCAUUUUUGAGGUACACUGAGAUGUUCUUCUCAGUCUCUUG

>N666

UCCUUUUUGCGGCUACAUGUCCCUUAUCUAUAAUCUGUUUUUAUCUUUAUA

>N667

AGGUGCAUGGAUCACAAGCUCUGUAAAUUCUUAUAAAGCAAACAUUCCCCA

>N668

CCCUCUGUAAUAGGGCAUUAUCCUGACUUCUAACUCGUGGGUUCAUUUUGG

>N669

CCUUGUUGUCCUUUCUGCCUUCCCGACUCUUUUUUCUUCUUUUGCCCCUGC

>N670

CCUCGAGCCCUGGGGGGACAGGAGGAGUCUGGGAGUCCAAGGUGGGCCCCG

>N671

UCCCUGAAGAUCCACCAGCGCACCCACACCGGGGAGAAGCCGUACCUCUGG

>N672

UGACGCGGCACCUGCGCAACCACACAGGCGAGCGCCCGCACCCCUGCCCGU

>N673

GCUGCGCCCUGCCCCAGCUGCGGUAAGAGCUGCCGCAGCCGCGCCGCGCUG

>N674

CGGGACACGCUGUGGGGCCGGGGACAAGCGGGCCUCGCUGGGCCUGGCGAC

>N675

CCCAACCUGACGCGGCACCGGCGCAACCACACAGGCGAGCGGCCCUACCUG

>N676

UAGAAGCGUCCCAAAGGGUGCUGGGAAAGGUCCCAGCGUGGGUUGAGGGAC

>N677

CUGCCCCUGUGGGAGAGUUGCCAAGACUCGGCGAUACCAAGGAUGGAAGCG

>N678

GCGAGGGAGAGCCAAGCAUAUUCCCAGCACCAGGGCCACUGCUGGCACGUG

>N679

GUGGUCUCACUUUCCUUUCUGAACCACCCUUGCUGAAGCCCGGGUCUGUGC

>N680

GAAGAAAGGACCCCAACAUGGCUCUAGAGAAGGAAGAAGUGCACUUGCCCG

>N681

CCCAGAUCCUUCUCUGUAUGCAGAGAAGUGUUACCUUCUGGGAGCUGCCAG

>N682

UUUGAUCACAAAACAUGCAGUGUGAAAUCACACGGGGUGUUGUACGCCGGC

>N683

CCUACUUGAGCAUUCAGCGCUUAGGACCACGGGAAACCAAACCAGGAGGGA

>N684

CUUGCCAAAUUCUGAAAUCAAGUUGAGCAAGCCCACCCACUUCAGGCCUCA

>N685

UCUUUCAUUCCUGGGUUAGAGAAACACUGCCCGCAAGGACAGAUCUGUGGG

>N686

AAUAAAUCAAGACUUUAAAGGAUUUAGAUCGGGUCUAUGGCCAGGUAAGUA

>N687

AGAUGUGGAAACAUCCUGCAAAUGCAGCCACGCUGCUUCGUACAGGCGGUC

>N688

CAUGGGGAGCCAGGUGAGCUUGGUGAUGUUGGGGCUUCUGGAACUGCCUGG

>N689

ACCAGAGGCAGCCGCUCAGCCAUGCAAUAGCACAGGCCGAGGGAGAGGGCG

>N690

GGAAAUGAAUGGACUCCCUUAAGUUAUCCCUUAGGCUAAAAAGAGAACAGG

>N691

UAUUUAAAUGUGGCUUGUACGGCUGAGGAACUGAACUUUAUUCAAUUUUAG

>N692

UAGCCCCACGUAGCUACUGGGCAUAACAGCCCCAGGGAUGAUAGGAUGACA

>N693

GGAGAGCCAGAUAGUUCUGGGGUUCAAUUCUGGCUUUGUCAUUUAAACCAG

>N694

GCACCUGGGGUGCUGGUGUCACUGCAUGAGAACUCAACAUCUGUCCUAUGA

>N695

CAGUCCCAGGGCGGUGCAGUGGAACACAAGCUGGGCCACGGUGCUGGGUCU

>N696

UUCCCGUCUGUGCUCUGCUACUUAGAGCUGUGAGUGUUGGAGGAAGCCACG

>N697

CGCCCGCUGCUACUCCAGACGUCGUAAGUUGCUCUUUUAUGGGAUAUCUCA

>N698

AAUAGGGAUGGUGCUAUAACGGCCGAAUGCAGGCCUCAGGGAAUCCCGAAA

>N699

CUGGGUCGGCGGAUCGGGGGGCUUGAGAGGGCCCGCUCCGUGGGACGUUCU

>N700

ACGACGCCGGCGACUGCGGCGCUGCAGCGGAGACGGAAGGCUCGGGAGGCG

>N701

GGGGUUGCGACGCAGAGUUUACCAUAUUUCUCCAGGGUUGCUCCGGCCGCU

>N702

GGGAGAGUGAGGAGGCUGCCCAGUCACAGGGCCGGGCUGAGAUUGGCCAAG

>N703

CUCACCUUGCUCUGUGACCUUGGCAAGUAUGUUGACCUCACUGAGCCUCAG

>N704

CUUUGCUGUGAGGAUACCAAGAGGAAUCACGCUGAGACCAGUCCCCCUGGG

>N705

UUAAGCUUCACAAAAAUAUCUUUAAAUCACUGAUAAUGCCACCCUUUUCUU

>N706

CGAAGGUAACUGAAAAUCCUAGCUGAAACUGAAACUGAAAAUCCUAACUGA

>N707

GCAGCUCUGUCCUUAGGGUGCUGGGAGUCGGGGGACAGAGGUGGCAUGACG

>N708

GCUUAAAAGGCAGUGGAGGAAGAGGAACACCAAUUCCCAGCAGGGGAACUU

>N709

GGGUCCCCUGCGGGGGACGGGGAGGAGGGUCUGGGGGACGAGCGAGGCCUC

>N710

AGCUGCUUACCGGAUCUUCAUCCCCAGAAUGUCAGAGUUGAAAGAGACUUG

>N711

UCACUGGUGCACAGAGCCUUAGUUGAUUCUGCCACACCAUGUGCCACUGAG

>N712

UGUGUGACGCGGGGCCAACCUACAAAGUGCUCUGGGGUGAGACCGAGGAGG

>N713

UCUCUUCGUCACCCAGGGGAGUUUGAUGGCUUUGAACCAGGUCAUGGGGAU

>N714

GUUCUAGGAAGAGCAAGUGUGUAGAAGGGUGUUUUCAAGGGAGGGGGGGUU

>N715

GGUGGGGACACUGAGAUUUUUAAUGAGAUGAGUGCUAUGAUCUCAGACUUG

>N716

GGAGAAUCAGAGGCACUGUGAGGACAGACUGAUGUGGGAAGAGCUGGAGCA

>N717

UGGGUCAUCUGGGCAAGAGAACAUAAGGCCCCCAGCUAAGAGGCCUGGCCU

>N718

CACAGUUGGUACUUAGGGGUACAACAGGUGGAAGCCAGGGCUUUGUUUUGC

>N719

CCAGAACCUUUUAGCUUCACUGCUUAAAAAGUGGACGAUGGAGCAGGGAGC

>N720

ACUCUCUGCCACUGGUGGUAGGACGAUGCCUGUGUGGAGAGCUGGCUUCUC

>N721

CUGUCAUUUCCUCUUGAGUCCUACCAGGGUCUAGCACAGGACUGGGCUCCA

>N722

AGUGCACGUGGGGAGAGGGUAUGUGAGUUAACUCAAGGGUGCCUUUUCUUC

>N723

GGGAGCAUCAGGGACCCCCACCCCCACCCCGGCUCUGCAGGAGUGCACGGU

>N724

UCUCUCAACAUCAGCUUGUGUAAGGAGUGGUCAUUUGGGGCUUAGCGGGGU

>N725

AAAGACCCUAGAAGACACACAUCAGAGCCUCAGUGACGAGCUAUUGUAGGC

>N726

CCUUAUGGCGUUGUGAGAAGCAAACAAGUAGAUAUGGAAAGGACGCGAGUU

>N727

GCCAGGAGGGUCCUGUGAGUAAGGAACUUAGGGACUGCACCUUUGAAACGA

>N728

CGCACAAUGGCAAUUCCCACCCCAGACUCCAGUCAGAAACUGCAUGUGCUA

>N729

GCGGACAGCAUCUGCUGUCAGACCCAGCGCCACUUUGCCAUUGCCCUCCAG

>N730

CACCCCCACCCUCCAGGACUGGCGCACACAUCAGCGGGGCUCAUCCUGACC

>N731

AAGGGCCAGUCCUGGGCACAGAAGCAGGUGGCUCACACAGCAUGGCUCCUG

>N732

UUUUUUUUUUUUGAGAUGGAGUCUCACUUUGUCACCCAGGCUGGAGUGCAU

>N733

CUCUGCUUCUAGGAUCCCCACCCUAAGGCAAGGUCUUUGAAGAACACAAGG

>N734

ACUGGAAACAAGGGACAAGAGUCAAAUUCACAGGUCAAGGGUUGGGGUCUC

>N735

UUUUUUCUUUAAGCAGGAGAACUGCAGUUGCUAGCAGUUUCUAGCAGCAGU

>N736

GUAUUUCAGGCUGCCAAGGCGAUGCACUGGCUGCUGCUUUUCCCACCUCUC

>N737

UUGCUAAGUCCCCCAGUUUAUUAGCAUAAGUCACACCCUCUUGUCCUCCAU

>N738

UUUUUUUUUUUUGAGACGGAGUUUCACUGUUGUUGCCCAGGUUGGAGUGCU

>N739

GUAGCUGGGAUUACAGGCGUGCGCCACCACGCCUGGCUACUUUUUUGUGUA

>N740

UUUCUGAAGUUCCUGUGUCACAUGAAAUUUAUAUUAAAUACAUUUGUAUGA

>N741

GCCUGGUUGCAUGAAGGUUGUCUUGAUGGGAUUCUUGUUCUGGACAUAGAG

>N742

AGGUAUUUAUAGUGAGUUCUCCCUCAUGCCUGGAGAGUAUUAUAAAAUGGU

>N743

UGGCCAUUGUCGGAGCAUAUUCAGGACAUUUUACAAUAAGAGAAAAUCAAU

>N744

UGGUAGGUCACAUUCUGUGAACGCAAUGCAAUAGAAGUCAUUCACAAAAGC

>N745

CUGCAGCCCCAAACUCCUGGGCUCAAGGGAUCCUCCUACCUCAGCCUCCCA

>N746

AAUUCAAAUCAAAGAAAAAAUUGUGAUGGAAAUAAUGAAAUAUUUAUGAAU

>N747

GCUUUAAAUGUAUUCAUCAGAAAAGAAGACGUUUUGAAAAUCAAAAAGUCA

>N748

CUCUAAAAAAAUUUUUUUUAAAAUUAGCUGGGCAUGGUGGCAUGCACCUGU

>N749

ACUUGGAAGAUCCCUUGAGCCCAGGAGUUCGAGGCUAUGUUUGCACCACCU

>N750

UAAUGGAAUAUAUAGCCAAAGAAGGAAAAAAAUAAGGAUAAAAAUUAAUGA

>N751

AAAAAUAAUAUUAAAGAGACUCAGUAAAACCAAAACCCGUUCUUUGAAAAC

>N752

CAGACAAACCUCUGCAAAAAGUAAAAAAAAGAGAGAAGUCAGUCAUAAACA

>N753

UGAAAAGAAGAUGAUUACCAUGGAUACAAUAGAGAUUUUGAAAAAUCAUGA

>N754

CUAAAAAUACAAAAAUUAGCUGGGCAUGGUGGUGCGUGCUUGUAGUCCUAA

>N755

UCCAAAACAAAGAAAACAAAACAAAAACCAAACCACCCUCCUUUCCAUGAA

>N756

UUCAGGAUCUCCCGCUUCAAACGUGAGUUGUCCAGGACAGUGGGACCCUGA

>N757

CCCAGAACAAAACUGGGCAACUUUCAGGUGCCAAGGAAAUGUUUGCUCUAG

>N758

AUUAAUUUUUCAAAUGUACUGUAAUAUUUGGGACUACAUUUAACAGCAAAG

>N759

UUCUCUUUGUUUGUGCUUCCAAUAAAUGGAAUUUAUUUCAUUUUAAAAGUU

>N760

ACAUUUUGAGAUCUGACGUUGAGUGAAUGGAUGGAUCCGCAGCCAGGGCUC

>N761

UGCCCUUAGGGGCCGCAUUGAUGUUAGAAUACAAGUCAGGGACCAUGUAGG

>N762

GUAUUUGUCGAAUGAAUUAAUGUCUAGAACCAUGAACUUUUCGGACUGCCA

>N763

AAUGUGUUUCUUUUCCGGUUUAAAGAGGACAUAAGAAGAAAACGUAGGGUA

>N764

GUACGUGUUUCAAAUGUAGAUGUUUACUGGGGAAAUUUGAACGCCAAUGGA

>N765

UUACAGGAUUGUUAAUUUUUAUUUUAUUUAUUUAUUUUUUUUGAGACAGAA

>N766

UGAGCCACUGUGCCUGGCCAGGAUUAUUAAUUUUUCUUAGUUAUGAUUAUG

>N767

UCAUGUGCUAGAAUUUUCCCGUGACAAGUUAAUAUAUGUUUGUAUAUGAUA

>N768

CAAAAUAGUCCCUUCAAGACUGAGAAAGGCCUUAGCUGGGUGCGGUGGUGU

>N769

UGAAGCAACUUUUGUUAAAAAAUAGACUUUAUUUUUAGAGCAGUUUUAGGA

>N770

GCCGGGCGUGGUGGCGGGCGCCUGUAGUCCCAGCUACUUGGGAGGCUGAGA

>N771

GGCGACAGAGCGAGACUCCGUCUCAAAAAAAAAAAAAAAAAAAAAAUCCCG

>N772

CACAGCUUGACAUGUGCAGUGUUUGACUCUCCAGAAUGACAGUUCCAAAUU

>N773

UACUAAAUUGGGGGUGCAGGUGGGGAAUGCCCAGCUCAUGCCAGCAUUUCA

>N774

UUUUAUGAAGACAUGCAACAUUAACAACAACUUCCAGUCGAGACUGGUCGG

>N775

AACAGUAUUAAGAAGUGGGGACUUUAGGAGGUGAUUAAGUCCUGAGGGCAC

>N776

GUUAAAUAUACUCAUAUUGUGGUGCAACCAAUCUCCAGGACAUUUUCAUUU

>N777

ACCAUUCUACUUUCUAUUUCUAUGAAUUUGACUACUCUAGAUACCUCAUAC

>N778

UGCGUAUAUAUACCAUAUUCUGUUUAUCCAUUCAUCUGUCAAUGGACAUUU

>N779

UAUGUUUUGGCUAUUGUGAAUAAUGAUGUUGUGGAUAUGAGUGUACAAAUC

>N780

UCCUUCUUUCAGUUAUUUUAGAUGUAUACCCUUCAUUACACUCCUCAUUCA

>N781

UUCACAUUCUCCAACAUAUAGAUGCAGAGAUGAGGCUAAAGAACAUGGAGU

>N782

AUUUUGGCCACAUCCAUUCAAUGGGAAGGCAGGGAACCUCUUUUUGGGAAA

>N783

GUACCCGGAGGCACAGAGGUUGUGAAAGGUGCCAAUGUCAUCUGUUUUGUC

>N784

UUCUUCAUGUAUUCUGCAUAUUAUGAUAUGUUUGGUGAAUCCCAAUCUUAA

>N785

CAAAGUGACUAAAAUAGGAGGACUCAAUUUGGAAAAUUUGAGACGUGAAGA

>N786

GGGGCCUCCUCCUGCCCCGUCAGGGAGAACGUGGUGGUGGUGGGGGGAGGU

>N787

GCUGUCCCAUGUCCCUGUAGGGCUAAUGAGAACUUCUCCAGGCCAAGCAGC

>N788

AGGGAGGCCUUGCCUUGUGUUCCUCAGAACCGGGACACUUGGUCCUCACCG

>N789

GUCCCGUAGAGUCCCACCCUCUAGGAUUCUCUAAGGAAUUCACAGGUACCG

>N790

ACUCCCACCUCCUGUGGAGUAGACCAGAUGGCCGCAUCUGCCCAAGGAAGC

>N791

GAAAUGGCAAAGUGCAUUCCAGCCAAGGUAGAAAUCACUGGCAGGCAAGCG

>N792

UUAUGCAGCAAUGGAGAACAAAUACACCCGGCAUGUCCUGGGACAAAGCCA

>N793

UCGAAGAGGCCGACUAGUUCUCAAAAGGGAGAAGAGGUAUGGCCUGAAAGC

>N794

GGGAGAUGCUAACUCUAACCGCAUUAUUUUUUUGGAGACAGGGUCUCCCUA

>N795

AGAGAGAUUAUUUAAUCCUCAUCCUACUCCACAAGACCAUUAUCGUUACGG

>N796

ACCAGUGUUUUGAAUUAACGAACUCAAAUGUCAUCUCCUCCCACACAAACC

>N797

GACAUACAAGGAUGAAGAUGAAGAGAAAAAGCCAGGCCAUAUGCUGGGCUA

>N798

UAAGCAGAGAACCCAGACACAGAUGACUUGGGAGGGGGCUCAGGAUGCUUA

>N799

GAACCACAUCAGCAGAGAAGCCAAAAAGGAGAUACGACCCUUGUUUCAUCA

>N800

UUUUUUAAAUGACGUGCAUUCCAAAACAGUUUCACAGGGCUAUAAGGAAAU

>N801

GUCAAGUACCUCACAUUCUAGAGAGAACAUCAACAGUCCUCUUGGCAGUUA

>N802

CUGGAGUGCAUACUUGGAAGACUUGAAGGGGGAAAAGGAGUGAGAAGGAGU

>N803

ACACACAAGUCCUAGAACAUUUAAAAACAUAAGUAAAUCCCCCUCCUUCUG

>N804

UUCUCCAGCUAUGAACUUGAACUCAAUGGGAGAAAUCUCCCAGUAAGAUGA

>N805

GGAGAAAUGGAGACUUGAAGAGGUGAAGCAAAUUUCCCUAGAUGACUUGGU

>N806

UGUGGGGAGUUGGGUUUGAACUUGGACAUUCAGUUUUGAAUCCACGUGCUU

>N807

CACACAUUCCUGCCCUUCUGAGUUGAUUUCAAUCCUAUCUGAGUAAUAGAU

>N808

ACUGGCCUUUCAGUGGCCCUCGUGGAACGGUAGGAAGCUGAGCUGGACUCG

>N809

ACUGUAGGUUGUUCUAGAGAAAAACAAAAUUUUAAGGACUUUUUCUUUGUG

>N810

AGGUAGGCAUCAUGUGGGCCUAGGCAAUAGGACCUAGGAAGGAGACUCCCC

>N811

CCUGCCGCUCCUACUCAGGAGGCUGAAGGGUCCCCACCCCAACUAACCACG

>N812

AGACUUUUUUUUUUGAAGAAUUAUCAACCUAAAUAACAAACAGAAGCGAUU

>N813

UGUGGUGACUCACGCCUAUAAUCCCAGCACUUUGGGAGGCUGAGUCGGGUG

>N814

AGAAUCGCUUGAACUUGGGAGGCAGAGGUUGCAAUGAGUCAAGAUCGUACG

>N815

AGGAGUUGUUGAGUUGUUUUUGAACAAAGAGAACAUUGGUUACUUGGGCAC

>N816

AUCUUGAAAUGGACAACUGGCACAGAAUAGAGCAUUUCAAGGGCAGAUUAC

>N817

AGGAACUUCACGAAGGAAGGGAAACAUGAGACAAAGAAAGAGAAGUGGUUU

>N818

UGGCUCUGAGCCAUUCUUUGGGCUGAGGUGGGUGUCUGGGGUGCUGGUGGG

>N819

UUGGGCGGACUGUAUUUCCCUGCCCAUUGAGUUUCGGUUUGGUCUUGUGAC

>N820

GAGAUCAACAAGAUGAAGCCAGUAGACCCACAGAUGACUGAGAAAUGUGUA

>N821

AAGGGUGGAAUCCCCAUAAUUCCAUACCCUUCUGUGAAUACACUGGUGUCG

>N822

UUAAAAGCAAAUUGUUUCAUUUAACAAUACAUGGUAAAUAUACUUGGUGUU

>N823

UAUUAUAACCAAUCCCUUAUUUGAAAAUUUAGGUUGUUUUGGGUUUUAUAU

>N824

AUGCUUAUGGCAUAUAUUUGUGUAUACUCUUAAUUAUUUCCUUACAAUAAC

>N825

UUACUCUUCCACUGGCAGUGAUGCCAGGUAGAACCUCUGACUGUUUGGCAG

>N826

UUGACUAGGAUGUCAUCUUUUUCUCAUUGAUUUAUAAUACCACUUAUAUAU

>N827

GAGAGUAGAGGAUGAAAUAUCCAAUAAGCAGUGCUAGGACAACUAAUUAUA

>N828

CACCAUGCCCCCACUAAUUUUUUGUAUAGAGAUGGGGUUUCACCCUGUUGC

>N829

GCGUGAGCCUCCGGGCCGGUCAUAAAAUUACAUUUUUAUCUCACUCUAUUG

>N830

GUACACAAAAAAAGCACAAAUCAUAAGAGAAAAUAUUGAUAAUUUUGACCG

>N831

CCUGCAAAGCAUAAAAUAGUGAAUUAUUAGUACAGAUGCUCCUUGAUUUAA

>N832

CCUAUGGAACAUCAUAGCUUAGCCUAGCCUACCUUAAACAUGCUCAGAACA

>N833

CCUAUAGUUGGGCAAUAGUCAUCUAACACAAAGCCUAUUUUAUAAUAAAGG

>N834

GUCUGGAAAAAGAUCAAAAUUCAAAAUGCAAAGUACAGUUUCUACUAAAUA

>N835

AAGAUCCGAGAUUGCACUACUGCACACUAGCCUGGGCAACAGAAUGAGAUA

>N836

GCAUAUCAAAUGCCUAUAACUUAAUAAGGAAUGUGCAGACAACCCAAUAAA

>N837

AAGGAUAUGAACAGGCAAGACACAGAAGACAUUUAAACAUUUAAAGCAUGA

>N838

ACCAGCUAUUGGUGAAGAUGUAGAGAAAUGAUAACUAUCAACCACUGUCAU

>N839

CUAGGACAAAGAAAUUUAACUUUACAGUAUAGACUCCAGGGGAGCUCUCAC

>N840

AUACCCUGAUCCAGCUCCAGACCACAGUCAAUCCUCCUUAUUUUGUCUAGA

>N841

GGCUGGAAUCUGUGGCACCUUCAAAAAGAGUGAUGUGGAGGGUAGAAAGAG

>N842

UGGCAGGAAUUGCAGAGCAGUAAGGAAAAUGGGCAUAGGGAAAUCUCAUUG

>N843

AGUUUUGAGAUAUAAUUCACAUGCCACAAAAUUCAACCAUUUAAAGUGUAC

>N844

AGUACAAAAGUUCUGAUUUUGAUAAAGCCCAAUUUAUCUAUUUUUUCUUUA

>N845

CUUUUGAUGUCAUAUCUAAGAACCUAUUGUCAAAUCCAAUCAUGAAGAUUG

>N846

UGAUUUAUAUGUUUAUCCCUAUCUCAGUAUCACACUAUCUAGAUUACUGCU

>N847

UUUAAUUCUUUCAACUACGUUUUAUAGUCUUUAAAGUAUAAAUUUUAAACC

>N848

UUUUGUUGAGGACUUUUGCAUCCACACAUCUAUAUUCAUAAGAGAUAUUUA

>N849

CGACAGUGUUGUGUGUGCGGGUGGAAGCACAGGGAGCUAUCUAUUGUGAUG

>N850

CCUACUUCUCGCCUGCAGAUGAAGCAGCAGCAGAAAAAAUGGAAGCCAUUU

>N851

GUCACAUCUUAGCAGUUGGCAGCGUAAGAAGCGGAAGGAUCCACUGGUCAG

>N852

UUUUCUGCCGGUCAUGCUGGGAGCCAACAGCACCGGCUCCUGACGUGGGGC

>N853

CACUGGAUCAUCUGUGGGUGUUGGCAGGAACCUGAAGGUGUUGGAAGGGGC

>N854

AGAGGACGCCCCUGCUUCCAGGGGCAAGUGGGUACAACCAGAGGACGGGAA

>N855

AAGGGGCCUCAGCUGAGAAACGGCUAGAAUUUCCUGUGUGGGUUGUGGGGG

>N856

ACGGGGCUGGGAUUCAGAUGCAGUUAGGACACUGCCUUAUCUCAGGCUCUG

>N857

GGAGGCCAGGGCUGCAUGCUGAAUGAGAACUUUACCUGGGCCACCAAGUCA

>N858

AGGUGCUGCUCCCUGCAGUCAGGACAUGCUGGCCGGGGUCUCUGACCUUCC

>N859

UCAUUUCCCACUAUAGGACCAGGGAACCAGGCUGACCAGAAGACCUACGAG

>N860

AGUGUUGGGAUUACAGGUGUGAGCCAACACAUCCAGCUGUCCCCACUUAAA

>N861

GUACCUGGGACUACAGGAGUGCCCCAUUAAGCCAGGCUAAUUUUCUAUUUA

>N862

CCAGCUAAGAAGGUGAAGAAAAGUCACCACUGCAAUAAAAAGAGGGCACUG

>N863

GCAGGCUGGAAGAAAGUCAUUCUUUAUCAUCUCAUUGGCCCUUGGAUGGUU

>N864

AUCCUUGGGACACAGGAAUCAAUCAAGACCUUUGGAUGCCCCUGCCUGCUA

>N865

GCCCUCUCUCCAUAAAGGCCACUAGAAAGUACUGUGAUGCCAGGCUGCUCA

>N866

CAUUUUCCAUCUUUGAAAUGAAAUAACGUAUAAUAAUGUUUUCCAAAAUAA

>N867

CAGCUAAUUUUUUUGUAUUUUUAGUAGAGACGGGGUUUGACCAUGUUGGUC

>N868

CCUGGCUUCGGGUUUUUGUCUUUUCAUUAUAAUGAGUUGGUUUUAUGUGCA

>N869

GUAAAGGAAGGGUGGGGUUCUUCCAAAAUAGAUCCUCUUUCAAUGAGGAGU

>N870

CUUAGCACCACCUCAGACAUUUGGAAAACUAACUUGAGCUCCAGUCCCUCG

>N871

GAGGUGGAGGUUGCAGUGAGCCGAGAUCACACCAUUGCACUCCAGCCUGGG

>N872

AGACUCUGUCUCAAAAAAAAAAAAAAAAUCAUAGAAACGGAAAGUAGAACA

>N873

AAAAGUCCUGCAGAUCUGGCACACAACCAUGUGAAUGUACUUUCUACUGUG

>N874

UUCUGAGCCUGUGGCCUGCACAGGGAACUUCCUCUCCGACUGCAUUUAUGG

>N875

CUCACAUCCUGUGCCCUGGGGGACCAGCAGCUGCUUCCACCCAGGUGAGAC

>N876

GGGUUGAAUGGCAGGGUUGAAUGGCAGAGUUGAAUGGCAGGGGCCCUGGGU

>N877

AGCCGGUCAGAUGCCCCAUGAAGCUAGAUGGUGCCGCAGAGCAGCCCCCAC

>N878

GAUCUUUCCUGCCCCUUGCACAGUUAGGAUUCUGUGAACUGGGCAUUGGAU

>N879

UGUAUAUAUGUAUGUAUAUAUGUAUAUAUGUGUAUAUAUGUAUAUAUAUGA

>N880

GUGUGUGUGUGUGUGUGAGAUAUAUAUAUAUAUAUAUAUAUAUAUAUAUAU

>N881

ACAAAACAGGCAUGUUUGGAGUUUCAGCACAUGCUGUUAUGGAGCUUUGUA

>N882

UACAGAGAACAUUUAGACAUUUAAAAAUAAAAUUAAUAAAUAAUUAUAAAG

>N883

AUCAGAUAGAAUCAAUGUUAUGAAGAAAAUUGAGGAGAGAGAGGAGAUAGU

>N884

GAUUAGGUUUGAACCCACCGGAAAUAUUUUUCCCCUGGUGGACCGGUUCUU

>N885

CAACUAAAUGCAGAUGUGAAGGGUGACAGUAGUGGGUGUUGGGUACGGGGG

>N886

GAGGAGUCACGACUCUUAGGGUGGCAGUGGUGGGUGCUGGGUAUGGGGCGA

>N887

CCUCCUGUACUAAGGGGACAGUGUGAUAGAGACCUAAGGGAAGUGCUCAGG

>N888

GCGCAGGCUGAUGUGACUAUGGCACACAAUGUGAGGGGAAGGGGGUGGGAA

>N889

GUGGAAACCGGGGAGCAGUGUUAAUAUUACUGCAAAAGUCCAAAGAGGAGG

>N890

CUGAGCCAAAAAGCAAAUCUCAACCAAUGACAAAUAGUUUAUACAGACAUA

>N891

AUCACUUACUUUUGGGUCAAAAAGAAAUCAUACUUGAAAUUAGAACAUAUA

>N892

AAAAAACAAAAAACUGGUCAACAAAAAAGAAUGGAGGAAAAAACAACAAAC

>N893

AAACAACAACAAAGCUAUAAAUGCAAUGCAAUGGCAUUCAGCAUUUCAACA

>N894

AGUUAUACUAAAGUUACAGGAGUUAAGUAAUAUUGGCAUUUAGAAAUGAAA

>N895

CUUAAACCAGAGCUUGUUAAAUUUGAAUGUGCACGAGGAUAACCUGGGUCG

>N896

UUCUUGGCGAUGUUGCUACUCUGUGAAUUGCACUUUGAUUAACAAGCUCUG

>N897

AAUAUUAAGUGCUCAUUUAACAAAGAGUUGGUCUCUGGGAUAUUCUGGGAG

>N898

CCCAUGCAAGAAGUGGGAGGAGAUGACAACAGGAACUGUCUACUUGAGUCU

>N899

CCAAGGAAAUAGUACUAAUAUUCCCAGAAGUUCCAAGCAGACUUUCAUGUA

>N900

AAGAUUAUCUGUCUCAAAAUUCUUAAUAACAUUGGCAAGGCCCCUUUUACC

>N901

GGAUUAGUUUGUUUAGCCAAUCAACAGUGUCAUUUUUUUCAAUAUGGAAAG

>N902

UAGUUGCCUACCUCAUACUAUACACAAAAGCAAAUUCCAGAGAGAUUAAAU

>N903

UGUAAGAAAUAUUUAGAAGAAAAUAAAGAAUAUCUCUUUGAUGUCAUGAUG

>N904

UGGUGCUAGAGGAAUGAAGACACAGACACAGAAACAGAGUGCAGAGUGGGG

>N905

AGGCUUGGGGCCUGUUCCCAGCAACAAUGCUUAAAUUAUGAAAGAAAGGGC

>N906

GCCACAGACUGUGAAAAGAUCUAUGAGAUGUGUAGAAUCAACAAAGGUUUG

>N907

AAGAAUAUUAACUAAAAAUUCACAGAAGGGCCAGUCCAAAUAACCAACAAA

>N908

CUCGGCCACUCUCCACUCCAGCUGCACUGCCUUCCCUGCUCCUUCCCCAAA

>N909

GUCCACUGAUUUUAAUGUAAAUCUUAUCGCAAAACACCCUCACAGAAACAA

>N910

AAUUAAAGACAAUAACAAGGUCAUAAAUCCAUCUAACAUGAUACAACUGUC

>N911

GGGUGACCCAGACCUUUAUUCCUUAAAAAUCUGAGUUAUUAGUAGCCCUGA

>N912

UUGUUGUAAUUUUCCAUUGACUUUAAUCACAGGACGUGGCUACACUAAGAG

>N913

UUUCUAUCAAUUCCGCUCCUUCAGGAUGGCAGGGAACAUGGUAAGACCAGU

>N914

UCUGCUGCUAGUAGACUGGGCACUCAGUGGUGGCCAUAGCCAGAUUGGCCC

>N915

GGGUCAUUUUAUCCAUUUGAUGAUUAUUUUAAUAAAAUCUUCCUCUGCUGU

>N916

UUAUACUUGCCAUAUCUCCUUUUAAACAGAAUGAACAACCAGGUACACUAA

>N917

CUCUGAGUUUUCUCUUCCUCUGUCAAUUCAUUGUAGGAAACUCCCCUAUGG

>N918

UGGAUGAUGGAGUCCUUCCGUGCACACCUGACUUUACUGUAUACUCAGGUU

>N919

AUUUCCUAUCUAGAUUAUGUGUCCAAUGUUGAAACUGGGGUAUUAAAGUCA

>N920

UUUAUAUUCCAUUCAGCCAGUCUAUACCUUUAAAUUAGUGAAUUUAAUCCU

>N921

CUUUGUGUGAGCAAGAAGGCUGUUUAUUUCACCUGGGUGCAGGUGGGCUGG

>N922

GGAGUCAGCAAAGGGUGGUGGGAUUAUCAUUAGUUCUUAUAGGUUCUGGGA

>N923

GGGGAGAUAACAAAGUACAUUGAUUAAUUAGGGUGGGGCAGAAACAAAUCG

>N924

GGCUUUAUUUCUUCCUCAUUUCUGAAGGAUAGUGUUGCUGUGUAUAGUAUA

>N925

UUCAGAGAAGAUGUUUUUGGAUUGAAUCUAUUUGAGAAUCUUGAAUUUCCU

>N926

GUCUACAUCUCUCACAAUACUUUGGAAGUUUUCAGCUAUUAUUUAUUACAU

>N927

AAUUAUUUUAAAAGACCUGCCUUCAAGUUCAGAAAUUCUUUCUUCUGCUUG

>N928

GAAUUGUUUUUUCUGAUUUCUUUGUAUUGUCAAUCUGUGUUCUCUUUUAUU

>N929

CUGUGGUAAGAUUUCCUGGGGAUAGAGAUACCAAGUGGGCUCACCCUUGGG

>N930

CCAGGUGGUGUGUGUUGGCCACAGCAACAGGUUUGGUGGGCCAGUCUUUGC

>N931

GAUUGCUUGAGGCCAGGAGUUUGAGACCCACCUGGGCAACAUAGAGAGACG

>N932

AAGAAAUUAUAGGCAUAUUUUAACAAUUUAUGUUAAAUGUGCUAUUUAUGA

>N933

AACACUAAACUUCAUAAUCAGAAAAAUAUAAAGAAUGAAGAGGUUAUGGCA

>N934

AGAUAAUGAGUGUAAGCACAUUGUGAAUUGGCAGCUAAGGGAUCUUUGCCC

>N935

GGUACAGCAAAUCAGCUCAAAACAUAUGUCAAACAAGGUGGGGCCUGAAUA

>N936

GAUGAGUAGUUCACUUGGCUGGAGCAUACAGUGUGUUGAGGAGAGUGGCUA

>N937

UUUCUAGUUUGUCUUCUGUGAUAUUAUUUUCAGAGUAUCAUGUAUACCAUU

>N938

UCUAGGUUUGUCUCAGAGAUGAGGGAACAGGAAGAUCCUGGGAAAGUGUUC

>N939

UCUAGUCUGGGUCUAGAACUUCAGUAGCUCAAAUUCAUUGGCGGCAAUUUA

>N940

AACAACAAGACCAAUAGUAAGGAUGAGCAGAUCACCCAGGUGUUGGAGCUC

>N941

UUGAGUCUUUGGUGAAUACGAACGGAGGACCCUAUCAUGUGAACUUCAAAG

>N942

CAGGUUUCAAGUAAGAAUUUUGUUAAUAUCUUGAAAGAUCUCUAUGUUGCG

>N943

GAUGAAAGUGGGUAUAAAUAAAUGAAAUAUUGCAUUGUGUGAUGGAACAUG

>N944

AAUAAGAUAUUUAUAUAUAAUUUGCAGGAAUAAGAGGUAUCAGAUUUUAGU

>N945

UAUCAGCCUAAGGACUUUAGGCUUUAUUAGCAAAUUAAUGGACAAAUUUAU

>N946

UACUUGGUCCCUCAUAUUCUUCAACACACAAUGAUUUGUUUGACUCUCUCU

>N947

GUUCUGGCCUUCCUCAAUUCUUUGUACUCUCUUCUCAGAUGAUCCCACACG

>N948

AGGAAAAUGUAUUUGUAACUAAGGAAGAUACAAAAAUGCAGUCUUUUGCCA

>N949

GGAUGCUAGCACAGAGGAUUUGGCAAGGGGAAGCCCCAGAGGGUGUACAGG

>N950

AGAUUGUAGAACAUAUAACUUAAUAAGUCUCCAUAUGUUCAUGUAUGCUUC

>N951

UAUGAACAUAGAAAAAGUUAGAAGGAUAUAUACCAGUUAACAACUGUUACG

>N952

AGAGAGACUGCUCAGAGGGAAGAGUAAUAAGCCCAAGCAGCAUGGAAAUAG

>N953

AUUUACUGAAUGCCCACUAAAUUCCAGACAAAGACAUAAACUAAUUAAGUC

>N954

CUGCAUGGAACAGGGAUGUUAAUAAAGAGAAGAAUGGGUGUGUAGUAAGUG

>N955

CACUGGUUGUCUACCUACCCACUCCAUGAGGGCAGAAGAAAUCAUAUCUGU

>N956

AUAGCAUCUCAGUUUUGUCUUCUGUAAAGCAGAGAUAAUAAUCACAUGUAU

>N957

GCUGUGAGAAUUAAAUGAAAUGCUUACAAAGUCGUUAACAAAUGCUUGAAU

>N958

GUUCAGUAAUGCUGUUAUUAUGAUGAUGAUGAAAAUGCCUGAGGAUUAAAU

>N959

AUCAUUAAGAACUUUAACAGCCCAGAGACUGAGUUAGAGCCAUACUUUUAA

>N960

CCUGGAAAUAAAAAAAAAAAGGUAUACCUAAAGAUAUAUGUACAAGAAUAC

>N961

AUCAUUUGAAACAGUAAAACCAGAAAUAAACCGAAGAUUGAUCAAUGAAAC

>N962

UCAGUUACGCAUGUCCGUACUAUGGAAUAAAUUCUAUCUACUUAAAGAAUA

>N963

GUAUAUAUACCAGUUAACAACUGUUACUUUUCUGGUGGAGUGGGAAUGCAA

>N964

UUUUUAUAUUAUGGACAUUUACCUCAUAACAAUCUUUUGAAUUUAGAGAAA

>N965

GGGAGAGGGAUGGUUGAGGGUGUCAAUGAUAUUGGGCAAAGCCAGGCAAGA

>N966

CACAACAAUAAAUAUUUUGGAACUAAGUAUCAGGAUUCCCCAGGCAGGAGC

>N967

AUGACCAGGGCCUUGAUUUGACCACAUCUGACUCAGCAGAUCAUUCUGAAC

>N968

GCACUAUUUUGUUCCUUUAUUUUCUAGGAUUGUGUGAAUGAAGCUGCAUCU

>N969

AUGAUAGAUCCUGCACAUCUGCAUAAGUCAAUAUGUAUAGAUCUACCUCCA

>N970

AGAUGCAGUUUGUUUCACAAUGUGGACACACAAAACAAAUGCAACCAUGCU

>N971

AUAGACAAGAUCCUUUUCCUCAUGGAAAUCUUCCAAAUAAUAAAAUCAUAG

>N972

GCAAAACCCUGUCUCUACUAAAAAUACAAAAAUUAGCCAGGCAUGGUAGUG

>N973

CAAUCUUCAUAGCAUAUUCAUAUAUAAUCCUCUUAAAAUACACCAGUUCAU

>N974

CCAGGAACACACGUGUGACUGAACAAAAGUGCGUUUAUUGACCUGUGGCUC

>N975

UGGAUGCGCUUAGGAAGUGGGGGGUAGUUCCACAAUGAAGUGUUUGAAUAA

>N976

GUGCCAUGGCCUUGCUGUGGAGGCCAGACCAGCUCCUGGCUCUCAAGGGCG

>N977

UAUUCGAAAUAAAUAUAAAAUUAUUAUUUUGGUAAGAAUAGAGGAAAUACA

>N978

GAUUUGUAUAGUUUUGCGGUGAGCAAUUUAGGUGAAGUUCUCUUGAAGAAU

>N979

GCAAAAGUCCUGAAGUCUGUAAAGGAGAGUAGGGGGGAGUGUGAGGGAGGG

>N980

CCAGGCCUGACUCAAGUUCUUCCUUAACUGUGAAGCCUUCCAUGCACACUU

>N981

UAGAUUCCCAUUCUACCAACAUGGAAACAGAUUCGCAAAGCGGUAAGUUUU

>N982

UAAAAUGCACAAAUCUUAAGCAAACAGCUUGAUGCCUUUUUCUAUAUCUAG

>N983

UUUUUGAGAUCCAUCUAUACUGCAUAUCGCUAGUUCACUUUUUUUUUAAUU

>N984

AAAAUGAGGGGAACCCUGAGGACCUAGACCAACGAAGGCCAAAGUUGGCAC

>N985

CUGGGGAAUCAUAAGCUCCAUGAAUAUCUCUUAGUUCACUCCUGCCCCUGC

>N986

CACCUUGAUGGAGAGCUUCUGGCUGAGAGUAGAGUUCUACUGGUUUACCGU

>N987

UUCUCUAUACAAGUUUGCAGGGAUGAGAAAUCAUAAAACCUGCCCACAUCA

>N988

GCAGGAAGAGACUCUGAACCUGUCCAUUUGUCCCACAGGCCCAAGGGAAAU

>N989

GCGGUGCUGGAAAAAGUGCAGCAGGAAACAGCAUUCUGGGGAGGCAGGCCC

>N990

AUUUAGUGAGCAGUCAGUAACCCAGAGCUUCUUGUCUGAGAGCAGAAGCUG

>N991

AAAGUUUCGAUCAUUGAUGCUCCGGACAUCUCAUCUUUAAAGAACAUUGAG

>N992

UAAGAAUGAUGAGGCAGUGCUGAGCACCAUCCAAAACAAUUUUGGAGAAAC

>N993

UACAUGAUCAUACUUCUUACCAGGAAAGAAGAUUUAGGGGAUCAGGAUCUG

>N994

CUGUGAAUAGGAUAUAUAUUUUUACAUAUAUCAUUUAAAAAUAGGUAUAAA

>N995

UACCUGUACCUAAAAUAAAAGUUGAAAUGAAACAAAAAAGAAGUUGAAACG

>N996

CUUAAGAAGGGCUCCUCAUCCACUUAGUGCUACACGUUCAGUUGACCUAUA

>N997

AAAACCCAAAGGAGCAUAUUUUCUUAUUGAACCCUCCUUUAGAAGUAUAGU

>N998

AUUAAAUGGCUGAGCAGAAAGCUGGAAGAUAUUCCAGAGAGUACACCAGCG

>N999

CAGGUUAAUAAUUUCCCCUUGAAGGAAGAGCAUGGGAUGUGCUUUCUUCCA

>N1000

AAUGAGCACUGGACUGUCACAGUGCAAGUGCAAAAGCAUAAGAACGAUCUG

>N1001

UCUUCUGACUCAUCAGUGAAAGGCAAUUAAGUUCAAUGUGGUUUUAAUGAU

>N1002

GGGAGUCUGUCUGGAAGGUGCUGCAAAAAGGGAGCAGAGCCGAUUCAGUGA

>N1003

UCCUUCAACCAGAUGCUGGAUGUCGAAAAGGACCCAUCCCGGUUAGAAGAU

>N1004

ACUUCAUGAAGAACUCAGAUAACAAAGCCCUUCGGCGCAUUUUUAAAAAGG

>N1005

AGUUUGUGCUUUUAACAACAAAGAAACAGGCCAGGCCCAGGAAACCCAGGG

>N1006

UUAACAAAGGUCAAUGAUCUGAGAAAAGAAAGUGGGUGGUCCGGGUAUCCU

>N1007

CAAGAAUCACCUCACUUGUGUAGGUAGGUUGGAAUCAGGAUAGACACUGUA

>N1008

UUAGGGCUAGAGUGUUCUUUUCUUCACACGUUAGCCAUUCCUUACGGCUCC

>N1009

AAAUUAGAAGCAUAGUAAGAUUGCCAAAAUCAGAGAAUCUUGCAAAGUUCG

>N1010

GUGUUGUUCUAGAUUUCCUCUAGAGAAGGUUAUUAGAAUCUCCAUUGCGUA

>N1011

CUUCUCUUUCCCUUGAGGUUAGGAAACAGGUUAAAACUCAGAGAACUCCAC

>N1012

CAGAUUAUUUUGAGAGGUGGGUGAUAUGUGAGAGAGAAAUCUGGAACCUUG

>N1013

AUACAGGAUAAGAUAGAUACAGGGUAAAAUGUUGAGCACUUUGUACAUGCG

>N1014

AACUUCUAGAAAAUGUGUGUGCCGUAGACACAAAGAAGGGAACAAUAGACU

>N1015

CAUCUAAGUAUAGCAAGGACAAAUAAAUGUAACCAGUAUUAGAACCAGAAA

>N1016

UGUGACAUCAUUGAGGUAAUGUGCUAGUUCUCAACUGGAAACAAUUUUGCA

>N1017

GUGCUACUGGUAUGUGGUAAAUAGAAUCCAGGGAUGCUACUGAACAUCCUG

>N1018

UAGUUUCGUGAUUCCAUGACGACAGAAAGUCCCAUAAAAUUUGGCUAAAUA

>N1019

CUCUUGCACCUCUGUAUUUCUGUGAAUACACACAGGAUGUGCUCAACAGAA

>N1020

UCUAGUUGUGAACAGCAUGAUCAGCAUGAUACAUUUUCCUCUUCAGCUACA

>N1021

AUUUAUUUCUGAGGUUUCCCAAUGGAUGAGUUACAUUAGCUACCGAAAAUA

>N1022

GUCACGUGGUGUUUAUCUUGGUUUUACUUUUGUCCUGAUCCAAAUCCUUCU

>N1023

CUGCUUACUAGGCACUGUUAACUACAAAGCAUGCCUUUCAAUCCUGUUGGG

>N1024

CUUCUAUUUGUGUUUUCACUGGGUAAUGAGGUUCUCACGGGCACCACUUUU

>N1025

GCUUGCUUCUCUCAAAUUACCUUUCACACUGCAACCAUAAUGCUAUAUAUG

>N1026

AAGAUAGAGAACACUGUCAAAAAAUAGUCAGAAUCAACUCUUUUUUUUUUA

>N1027

GGUAACCUUGAAAACCAAAAAACUAAAAUCACUAUGAAAACUAGCAGCCUU

>N1028

GUGUAUUUUCUCAUUUCUUAGUGGGAGCUAAGCUAUGAGGACGCAAAGGCA

>N1029

AGAUAACUUAUCCAUGUAACCAAAAACCACCUGUACCCCAAAAACCAUUGU

>N1030

CCUGAGAUCCUCCCCACUGUUUCUAAGAGUCCUCUUUUUCCUCAGCCUUUU

>N1031

UGGUGUUUUUACUGUUACUUUAAAAAAAAAAAAAAAACACCCUGGUAUUCG

>N1032

UCUGCAGAGAACAUGCAGAGUCAGCAUGCAGCCUUCCUUCCUGAUUUUGAU

>N1033

CUACCCCCUUCUACAUUACAUUGAAAGGUAGGAAAAGUCCAUUUAAUAAAC

>N1034

CCCACAUAUUCAGAAUAUUGGAUACAUUAUAAAUGAUCAUCAAGUAUGGGC

>N1035

UUAGAGUACCCCAUCAGGUUUAACAAGAAGAUAUAUUUAUACUGAUUUCUU

>N1036

AUCUCUUCAUGUUGGCUGAGAGAAGAUAUUUUGGGGUGUGUUUCCUGUUUA

>N1037

UAAUAAGUUAUUGAUUUUAUUGGAAAUACUAUGUCCAGAAUUUUUCGCUAU

>N1038

AUUUGAUGGCAUGAUAUUUUUGUGCAGGAUAGAGCUAGUAUAAUGGAAAAC

>N1039

UUAUUAACACAUAACCUGGGGGCACAUGUAGUAGUUGUUGCAAGACAGGUA

>N1040

ACUGGAUGCAGAAAAAACAUUGUACAGCUCUUUAUCUGGGCACCAAGCAAC

>N1041

UUCAGUAUUUACAUUAUAGCAUUUUAUUUGUUGCAAUAUAGAAACAGGUUC

>N1042

AGAUUGUAUUUUAGUUCUCACUACUAAUGUUACUUUGCCUCAAACCAGGAU

>N1043

UUAGAAAGAAAAUUUGGGAAGCCUCAUUUUGAACUUUCAUUCUUGUUGUGU

>N1044

GCUGAAUCGAAACAGUAACACCUGGAAUGUGAACGGUGGUUCGAAAAGUAC

>N1045

ACUUUUUGGAAUUUCUAAGCACAUGAACAUGGCUAAAUUCUGUUGGAGGCU

>N1046

UAUUCAGAUUCAGAUAUUUUAAGAAAAUGGCUUUGCAUAAAUCCAACAACA

>N1047

UACUAACUCUUAGUACACAAUUUUUAAAAACAGUCUCUGGGGCUUCUGAUC

>N1048

UAAAAUAAAAGAGACCGUAAACGUCAGAUAAGGUUCAGCCAGAGAAUUAUA

>N1049

AAAAAACAAAAGAAUAUGAAAAGGCAAAUUUAAGAUGUCUUGAUUCCACCC

>N1050

UAAAAAUGUAAACAAAUUGCAGUGCACGUUCUUAAUUCCUUUCUUUCUUUC

>N1051

CACUAUACUGCCCAAGCUGGUCUUGAGCUCCUGUCUUCAAGCAAUCCUUCU

>N1052

UUUAAUAUCAGUUUUUUUCUGUUAAAAUUUUUUCAUAUAUUUUGUCCAUUG

>N1053

ACAUCCCCAACUAUUGACAUCCAGCACCACAGUGAUACGCUGGUAACAAGA

>N1054

GGCAUCAAGUGCCCCCGAUGGAAAUAUACAACAUCAUCUAUGAGUAAUAUA

>N1055

AAUCUUUUGUUUCAUGCCAUGAUAGAGUAAUGGGAAUUGUUAUUUACCCUA

>N1056

CAACCAGAAAAAUGGUAAAAUAUAUAAAGGAACUGUUUCCUGACAUUGGAA

>N1057

UCAGGACUGAUUCAUGAGAUAGGUCAAGCGGGUGGAAAAUGAAAAUAGAUC

>N1058

UCUCCACAAAAGAAUAAGAGAUCAAAAAUAGUAAAUACUUGGAUAAAUAUA

>N1059

UGAAAUUUUAAAAAGUUGCCAUAAAAGGCCAGGCAUGGUGGCUCAUACCUA

>N1060

GCCUGGUGUGGUGGCAUGCUCCUGUAGUCCUAGCUACUUGGGAGGCUGAGA

>N1061

GGUGACAGAGCAAGACUCCACCUUAAAAUAAACAAAAAAGUUGGCAUCAAG

>N1062

GAACUGAAAAAGAUAUACAUCAUGCAUACAUUACACAUACAAAAACGACAA

>N1063

AGUUAUCAGACAAAGUGGGCUUCAGAACAGUGCUAUCUCUUCAUCAGGCAU

>N1064

UCAUAAUAAUAAAUAGCAUAAUAAUAAUGCUAAAAAAUUAGUUCAUCAACU

>N1065

CUUAAGAUUGAGCACAGAUGACCAAAGAGAACUGUGUUUCAUAAUUCAGAU

>N1066

CAAACUGAAUCCAGCAGCACAUCAAAAAGCUUAUCCACUAUAAUCAAGUGG

>N1067

AAUAAGAGCUAUCUAUGACAAACCCACAGCCAACAUCAUACUGAAUGGGCU

>N1068

UAGUAUUGGAAGUUCUGGCCAGGGGAAUUAGGCAGGAGAAGGAAAUAAAGA

>N1069

ACAAAAUCAAUGUGCAAAAAUCACAAGCAUUCCUAUACACCAAUAACAGAU

>N1070

CCAAAUCAUGAGUGAAAUCCCAUUCACAAUUGCUUCAAAGAGAGUAAAAUG

>N1071

CCAACAACUUUCUUCACAGAAUUGGAAAAAACUACUUAAGUUCAUAUGGAA

>N1072

GAUACAGACCAAUGGAACAGAACAGAGUCCUCAGAAAUAAUACCACAGAUA

>N1073

CAAACAAAUUUACAAGAAAAAAACAAACAGUCCCAUCAAAAAGUGGGUGAU

>N1074

AGACACUUCUCAAGAGAAGACAUUUAUUCAGCCAACAGGCACAUGAAAAAC

>N1075

AAUGGCGAUCAUUAAAAAGUCAGGAAACAACAGGUGCUGGAGAGGAUGUGG

>N1076

AUUCCUCAAGGAUCUGGAACAAGAAAUACCAUUUGACCCAGCCAUUCCAUG

>N1077

AUAAUAAAAAAAAUUAAAAAAGAGAAAUACAAUUUAUUUUUGCAUUUUUAU

>N1078

UGUUUUCCUUUUAUUUCCUUUUCUUAUUUUAUUGCACUGACUGAAACUUAC

>N1079

GAAUUUUUUUUAUGCUUAAGUGUUGAAUUUUAUCAAAUGCUUUUUCUGCAA

>N1080

UGAAUAAGCCUUGAAUCCCUAAAAUAAACUCCACUUGUUCAUGAUGUAUAU

>N1081

GUUGCCAUUGCUUUUGGUGUUUUAGACAUGAAGUUCUUGCCCAUGCCUAUU

>N1082

AUGGCUAGCCAGUUUUCCCAGAACCAUUUAUUAAAUAGGGAAUCCUUUCCU

>N1083

UUUUCUCAGGUUUGUCAAAGAUCAGAUAGUUGUAGAUAUGCGGCGUUAUUU

>N1084

UAAAUGUAAUCCAGCAUAUAAACAGAACCAAAGACAAAAACCACAUGAUUA

>N1085

UCAUACUGAAUGGGCAAAAACUGGAAGCAUUCCCUUUGAAAACUGGCACAA

>N1086

CCCUCUCUCACCACUCCUAUUCAACAUAGUGUUGGAAGUUCUGGCCAGGGG

>N1087

GAAGUACAAACCACUGGUCAAUGAAAUAAAAGAGGAUACAAACAAAUGGAA

>N1088

GAUUCAAUGCCAUUCCCAUCAAGCUACCAAUGACUUUCUUCACAGAAUUGA

>N1089

UUUAAAGUUCAUAUGGAACCAAAAAAGAUCCCACAUCGCCAAGUCAAUUCC

>N1090

UCUAUGUGUUAUAUGUCUAUAAUGUACUAUGGAAGUAGAAAGCCUGUCAGA

>N1091

GGUUUAUUUUUCCCUGUUUAUUGUUAUUUUUUUGUAUUACAAUAAGAAAUA

>N1092

AUUUAUUUAUAUUAUAAAUAUAUAUAAAUAUUUAUAUAUAUUUAUAUAUAA

>N1093

AUAUAUUAUAUUAAUAUUAUAAUAUACAUAAAUUAUAUUUAUAUUAUAUUA

>N1094

UCAGUUCCAAGAUGGCCGAAUAGGAACAGCUCCGGUCUGCAGCUCCCAGCA

>N1095

UGGACAGUGGGUGUAGCCCACAGAGAAUGAGCUGAAGCAGGGCAGGGCCUU

>N1096

ACUGCUAGCACAGCAGUCUGAGAUCAAACUGCAAGGUGGCAGCCUCCUGAC

>N1097

UAACAAACAGAAAGGAAUAGCAUCAACAUCAAUAAAAAGGACAUCCACACC

>N1098

CUGUAGGUCACCAUCAUCAAAGACCAAAGGUAGAUAAAACCACAAAGAUGU

>N1099

GAGGAGAAAAGCCGAAAAUUCUAAAAACCAGAGGGCCUCUUCUCCUCCAAA

>N1100

CAAGGAAGCUAAAAACCUUGAAAAAAGAGUAGAUGAAUGGCCAACUAGAAA

>N1101

GAGAAGACCUUCAGUUACCUGACAGAGCUGAAAACCAUGGCAUGAGAACUA

>N1102

AAUUGAUGAAAUGAAGUGAGAAGACAAGUUUAGACAAAAAAGAGUAAAAAA

>N1103

UCCAAGAAAUAUGGGACUAUGUGAAAAGACCAAAUCUACGUUUGAUUGGUC

>N1104

AUUCCUUGAGAAGAGCAACCACAAGACACAUAAAUGUCAGAUUCACUAAGU

>N1105

AGACUAACAGUGGAUCUCUCAGCAGAAACCCUAUGAGCCAGAAGAGAGUGC

>N1106

ACCAGCCACUGCAAAAACAUGCCAAAUUGUAAAGACGAUCUAUGCUAGGAU

>N1107

CAACUAACUGGCAAAAUAACCAGCUAACAUCAUAAUGUCAGGAUCAAAUGU

>N1108

UACAAAAAGAUUUAGACUCCCACACAAUAAUAAUGGGAGACUUUAACAUCU

>N1109

CACCAAGCAGACCUAAUAGACAUCUACAGAACUCUCCAACCCAAAUCAACG

>N1110

CAUCAGCAAAUGUAAAAGAACAGAAAUUAUAACAAACUGUCUCUCAGACCU

>N1111

ACCUGCUCCUUAAUAACUGCUGGGUACAUAACGAAAUGAAGGCAGAAAUAA

>N1112

UGAAACCAAUGAGAACAAAGACACAACGUACCAGAAUCUCUGGGACACGUU

>N1113

UGUAGAGGGAAAUUCAUAGCACUAAAUGCCCACAAGAGAAAGCAGGAAACG

>N1114

ACACCCUAACAUCACAAUUGAAAGAACUAGAGAAGCAAGAGCAAACACAUG

>N1115

ACUAGCAAGACUAAUAAAGAAGAAAAGAGAGAAGAAUCAAAUAGAUGCAAC

>N1116

CCUGGCAGAAACACAACAAAAAAAGAAAAUUUUAGACCAAUAUCCCUGAUG

>N1117

CAAAAAUCCUCAAUAAAAUACUGGCAAAAUGAAUCCAGCAGCACAUGAAAG

>N1118

UUGAUGGAACAUAUCUCAAAAUAAUAAGAGCUAUUUAUAUCAAACUCACAA

>N1119

AAGAAAUAAAGGGUAUUCAGUUAGGAGAAGAGGAAGUCAAAGUGUCCCUGA

>N1120

CAUGAUUGUAUACUUAGAAAACCCCAUCGUCUCAGCCCAAAAUCUCCUCAA

>N1121

AACUUCAGUGAAGUCUCAGGAUACAAAAUCAAUGUGCAAAAAUCGCAAGCC

>N1122

CCAAUAACAGACAGAAAGCCAAAUCAUGAGUGAACUCCCAUUCACAAUUGC

>N1123

AACCACUGCUCAAUGAAAUAAAAGAAGACACAAACAAAUGGAAGAACAUUA

>N1124

UGUAGGAAGAAUUCAUAUCGUGAAAAUGGCCAUACUGCCCAAGGUAAUUUG

>N1125

UGGAGACAUCACGCUACCUGACUUCAAACUAUACUACAAAGCUUCAGUAAC

>N1126

UUAGAUCUAAAACCAUAAAAACCCUAGAAGAAAACCUAGGCAAUACCAUUG

>N1127

CAUGGGCAAGGACUUCAUGACUAAAACACCAAAAACAAUGGCAACAAAAGG

>N1128

AAAUGGGAUCUAAUUAAACUAAAGAACUUCUGCACAGCAAAAGAAACUACC

>N1129

ACAGGCAACCUAUAGAAUGGGAGAAAAUUUUUGCAAUCUACCCAUCUGACA

>N1130

AAAGUGGGCAAAGAUAUGAACAGACACUUCUCAAAAGAAGACAUCUAUGCA

>N1131

UGUCCAUCAAUGAUAGACUGGAUUAAGAAAAUGUGGCACAUAUAUACCACG

>N1132

AUGUGUCAUUUAAAUACUAUUUUCAAAUUAUCUUUCUUCAUAACAAACAAA

>N1133

CACAUCAGAGUCCAAAAUGAUAAUGAUUGCACCUCUGAGUAAGGCACUGGU

>N1134

AAGGAGGAGACAGAGCACAGUUGAAAGCAAUCUCCAUGGUGAUAUUUCUUA

>N1135

GGAAACAGAAAAAGAAAGGGAGUUAAAUUGAAAACAAAAUGAGGAAAUUAG

>N1136

CACCAUUAGUAUUAUGGAAUGGUGAAGAAUUCAGGAGCGUAUAAGCAAAUC

>N1137

AGGGACUCGAUAGCACUGGGAGAAGAGGGCUUGUGCUAAUAUUGCUCCCUC

>N1138

AACUACGUUCUGACCAACAUCCGAGAAGUACUGUGGGUAGGUGGGCCAGGU

>N1139

UUAUGUUUUCCCUUCAAAGCCGUAGAUAUUACUAGUGGCAGAAUCAGACGA

>N1140

CCGCUGACACUGCAUUUUGUAUAGUAAUGGGAGACAUCCAGUACUGAUAUU

>N1141

AAUCUACUACUGAAGGUCAUGGAACAAAACAGGUGUCAUGGGGCUGACAGG

>N1142

AGGCCCCUGUAUAUUCAAUUGCAUUAAAGCUUGUUUUGUUUUACUUUAAGA

>N1143

CUUAAUUUCCUAUGUCUGUCUUUAAAGGUAACACUUUUUUUGGUCUUGGUC

>N1144

GAUGACAAAAGUUAAAUACUUCUGCAAAAGGAUCUGAGUUUGGAAAGGGUU

>N1145

GAGACUUUCAUGUUCCAUCUGGGCCAGAUGUGUGGGAUGAGGAAUGAGGAU

>N1146

UUUGGGGUGCCACAAUCUGCGCCUUACAAGAUGAUGAACUAGUUGAUCAUU

>N1147

UGGGGCAGGUAUGUGAAAAGCUGAGACAGGCCAAAAGCUAGACCUCUUGCG

>N1148

UAUAUGAAUGAUAAGAAAGCUAAACAGCCUUACUGCUGAUAUGAAGAAAGA

>N1149

UGGAUAGAAGAUUAAGCCAGCCGCAACAUUCCCUUAAGCCACAGUCUAAUC

>N1150

ACCCCACAAUUUUAUAAAGUCUGACAGAGGUGAGGAAGCUGCAGGAAAUAG

>N1151

AGCAGAUGUUGGUUCAUGAGGUUUAAGGAAAUGACCUGUCCCCAUUACAUU

>N1152

GCGAAGCAGCAAGUGCUGAUGGAGAAGCUGCAGCAAGUUAUCCAGGGGAUG

>N1153

AAGUCAAUGCUCACUUACCAUUCCAAAAAACCUUAGAGCUUUUAAGAAUUG

>N1154

AUUCUUCCUGUGCUCUAUAAAUGGAACAACAAAGCCUGAUGACAGCAUAUU

>N1155

GCAGAGGGGGAUUUGGCAGGGUCAUAGGACAAUAGUGGAGGGAAGGUCAGC

>N1156

GUGAACAAAGGUCUCUGGUUUUCCUAAGCAGAGGACCCUGCGGCCUUCCGA

>N1157

AUCUGUUUAACAAACCACAUCUGGCACCGCCCUUAAUCCAUUUAACCCUGC

>N1158

GAAGAAUUUUUCUUAGUACAGAACAAAAUGAAAAGUCUCCCAUGUCUACCA

>N1159

AGACACAGCAACCAUCCGAUUUCUCAAUCUUUUCCCCACCUUUCCCCCUUC

>N1160

UGGUGGCCGGGCAGAAGGGCUCCUCACUUCCCAGUAGGGGUGGCCGGGCAG

>N1161

UGUUGAUACUUCUGUUUAGAAAAAAAGAUUCAAAAUAUUACUGCUCAUUGC

>N1162

UAUUUUAUAAGGCUAUAGUUGCCAUAAAUAGUGAUUCUUCUGAUAAACCUA

>N1163

AUUGAAAACUUUCUGGGAAGGAUUCACCAUUCUAGAUACCAUUAACAACAA

>N1164

CACUUUGGGAGGCCAAGGCAGGAGGAUCACUUGAAACUAGGAGUUUGAGAG

>N1165

AACACAGUGAGAUCCUGUCUCAACAAAAAAAAAAAUAAUUAGUAAGACAUC

>N1166

GGCUGCAGUGAGCUAUGAUCACACUACUGUACUCAAGCCUGGGUGACAGAA

>N1167

GAAACAUUUUGUGAGAGGAAGAGUCAUUCUAUGUGACAAACCUCAUUGUUA

>N1168

UGUUAGCAUCUUUUAGCAAUAAAGUAUUUUUAAAUUAAAGCGCAUAUAUUU

>N1169

ACUCAGCAUACAGUAAGUGAUCAGCAAGUGUCAGCUAGUAGCAGUGGUGUU

>N1170

CAGCCUCAGCCUCCAAUUCCAAGAAAGGACUGUGCCUUAUUAGCAGAUAUA

>N1171

CGGAUAGAUGAUGGGUGAUGAAGGCACCCAGGGACACCGGCACUAGGGCCU

>N1172

CAUUCUUUGGCAUUGGUAGUUAUUUAAUGCUGUUAGGCUCUGGAUUUCCUU

>N1173

UAAUAAUGGCAUUGAGGGAUCAAGAAUAAUUGUUGGAGAUUAUGUAUGAUA

>N1174

AAUUUUGCUUAUAGACAAGUAAAUAAUUUUUUUGUCUGGCAGAACGUAUAC

>N1175

AUUGAAACACUGUAACAAUAGCUGAAUUCCAGGCUAAGCAAUAAGGAUGCG

>N1176

CAGUAAGAACUUCCCAACUACCAUGACAUGGACUGUCCGCAUGGGCUUCAG

>N1177

UUUCUUAUUUAACCACCAAUUUUCUAUCACAAUUUAGAAUUGUAUCCACUC

>N1178

AAAUGAGUGAAAUUUGAAUAAGCACACAGGGGAGAAAACCGUGUAACUCUA

>N1179

UUGGAAGGAUGCAUCUACAAGUCAAAGAGUAGCAAGGAUUGCCAGAAACCA

>N1180

GUAAUUUGUUACAGCAGCCCCGGAAACUAAUACAGACUUGAUAUUGGGAGG

>N1181

GAAACAAGAAAGUCAAAGAAGGAAGACAAUGUGAAGAUGGCAGGAGAGAUG

>N1182

UUUUUGGUAAUUUGUCAUGAUAGCAAUAGGAAGCUGAUACUCCAAGGUUAA

>N1183

CACACACACAUACACAUACACACAAAAUCAAAAACAUAAUGUUCUUUUUUA

>N1184

GUUAGAUGUUACCAUAAGUAUUAACAGAAGAAAAAGGGAAAGUAAAGACAA

>N1185

CCAUAAAAGGGUCUGAUGCAAGAUAAACUAGCCUGUUGGUUUAACAAUAGA

>N1186

CAAGAGGACAGCAAAAUAUUGAAAAAAUUAACUGGCUCAAAAAUUAUAUUC

>N1187

GUUCUGAUGUUUCCUUGGAGUUGUCAUAGCACUCUGUGUGAACCAAAGGGA

>N1188

AUGCCCCUUUGAACAGAGUGAUUUGAAUAGAUUCUCCAGUGCACCAACUGG

>N1189

AUAAUUCUGGUAUUUGUACCUAGCAAUACAGAAGAAAAAGCAGCAAGGAGU

>N1190

UAUAUAGGACAUAAGAUGAUUUGCCAGCCCUAGCUAGAAUUCCCAAUAUGG

>N1191

UGUUAUAAGCAGGAGGCAACAGUGAAAGUACCGAAUCCUGGCUUUCUUCUU

>N1192

GGUCACUAUUUACUGUGUGAGUUCCACUGGAGACAAGCACACUUGCUCCAC

>N1193

UGUGCUGAACUGUGGGAAAUCAUACACAGCCCUAUGCCUCUGUCCAUGCUA

>N1194

CUUUAAUUCUCCUGUGAAUACAGCCAUAUUGGUAGAGAUAGUUCAAUCCUG

>N1195

UGUCUUGCUGAACCAUAGGCCAUAAAUGGAGAAAUAACCCAAAGCCAGCUU

>N1196

CUAUUUCUACUUGGAGCUCGUAGUGAGAAACGUUGGCGGAACAUACUACUC

>N1197

AGUGACAAAAAUCCAAAUAUUCCAAAGAAGGCUUUUGUUUUAUCUUCCUUA

>N1198

UGGCGUGGGGCGCUGGAGGGUGGCAAGGCCGCGACAGGGGCUGUGGGCUUC

>N1199

AUCUGUCUCUGCCUGAGACUGGGGUAGGAAGAUGUGGGGUAGAGAGGAAGA

>N1200

GAAGAAUUAUACGAAAAGCAACUAGAGAAACUAUGUGUUCAUGGGCAGAUG

>N1201

UAUUAUACAAUCACUAAGAACCCCCAGCUCUACGCUUGGAAAUGGCAAGGA

>N1202

UCCUUGGAGAAAACCUGAAAAUUCAACUUGUUCAAGAGAAGGUGAGCCUGC

>N1203

AUCACUCUAUGAAAGCCUAAGGACAACCCUGCAAGAUUGGUUUUAUUUUCA

>N1204

UUUAGAACUGUUCACCUCUGUUGUAAAUGCUUUUGUCAUUUCUCAGCCUUG

>N1205

AGUGAUAUUUCUGACCAUCUCUCUCACGUUUGGCUUCUCAAUAUGAGAGAA

>N1206

CCACGAGAAAUGCGGAGCCCUCCUCACGGUUCCCUUAUAACAGGCUGGCUU

>N1207

AAUGAAAAUUUAGAAUCAUGGCCUGAGAGUAGCCAAGAACAGGCAGUAGAC

>N1208

UAAGGAACUUCCAUGUACCUAUAUUAAAAUCUCACAGUUAUCAAUUUAUGA

>N1209

ACAUCAUUUUAUCAGUAAAUAUUUGAGUAUGUGUCUCUGAAAGAUAAAGAU

>N1210

GUAUUCAGAUUUCCCUGAAGAUCUCAUAAUUUUUAAAAUUGUUUGAGGUAU

>N1211

GAACUGGGCAGUAUUCAUACUGCUGAUUGCAUCCUAACAAUGUCAUUUAAA

>N1212

UUUGUUUUUUUAGUGGGAAUACUUCAUAGAUGCUAUCCUAAACUUCUAUUC

>N1213

AUGUUUGAAUGCCUCUCUUUUUGUGAUGUUAGCAGAUGUUAAUGAUUGUCU

>N1214

AAUAGUCAUACACAAAGGAACUUUCAUUCAGUGAUUCUUUCAUUUUUGUGG

>N1215

AAAGAUGAGAUGUAUGAAAGACAAAAUAAAUGCUUCUUUCCCUUUAGUUGA

>N1216

GUGUUGUUAUCAACUACUGGAUUUCAAUAUAUUUGGUAUAUUUUAAUCCAG

>N1217

CCAAGCCCUUCUGAUAAAACUAAGUAAUUUUUUAUAGCAUCCCUACUUUCU

>N1218

CACACACACAUACACACACACACACAGUGAAAUUAUUAAUACUACAUUUUA

>N1219

UCUUUUAACUUUUACUGGUGUUUUAAAAUAUAAUUUUUAUUGUAUAAUUAU

>N1220

UAAAUGUUCUCAAGAUCAAAUUUGCAAAAACAAAGGAUAUUCAAAGAAGUU

>N1221

AAACUAAUAUUCCUUCUUCUUAUAUAAAUGAUAGUAAACUAUACAUUAUUA

>N1222

GCUUUUUUUCACUUAGCAGUAUAUCACAGAGAUCUCUUCAUACUGGUAUAG

>N1223

UCCUAAAAUCGGGAUUGAUAAUCAAAGAGUAAAUGCAUGUGUAAUUUUUCA

>N1224

CUGUUUUCCCCAUACCUUUACCACCAGAUUAUGCUGGGAAAUGGAUUUUUU

>N1225

AGUAGAGAAAUAGUGAUCUCUGAGAAUUUUUUAUUUUUUCUAUGUUUUUAU

>N1226

AGUUAAUGUAAUUGAAUGGAAUAUAAAUGAACAUAAAAUGAGCAAUGGCCA

>N1227

AGGAAGAAAGAGAAAGCAGAAAAGAAGGUUGGGAAUGGAUCUCACUUACUC

>N1228

UUUUUCCCCUAUCUUCCCCUCCUUAAGGUCUUGUACGUGCCUAAGUUCUAU

>N1229

GAAGUCAGCCAUGAAGCACAUGGUCAUCUUGUUCACUCGCAAAGAAGAGUG

>N1230

UCUGAUGACAUAUACAAGGACACAGAGGAAAGGCUGAAACAACGGGAAGAU

>N1231

AAAUCUACACUGACCAAUUAAAUGAAGAAAUUAAACUAGUAGAAGAGGAUA

>N1232

AGAGGAAGAAAAGGAGAAAGAAAUUAAAUUACUAAAAUUAAAAUAUGAUGC

>N1233

AAUAUAAGGGAAGAAGCUGAGAGAAAUAUAUUUAAAGAUGUUUUUAAUAGA

>N1234

UGUGAUUUGUUAAUGGAUGAAUUGUAUUUUGCAAAGAUAGUUAGAGAAAUC

>N1235

GAUUUUUAAAUAUAUAUAUAUAUAUACACACAUUGUGAAAUAAUGAAAUAU

>N1236

UUUACUUCUUUUUCAAGGUUGAAUAAUGUUCCUUUAUUAAUGGAAUGUUAA

>N1237

AUAUGAAUUUUGGGGGGACAUAAUCAUUUAGACCAUAACAUUCUGUUCCUC

>N1238

AUGGGUGAGACUUAAGGUACAAUUCAUUCUGAGGCAAAUUGCUUACCAGUU

>N1239

GAAAUCAAGUAUAUUAUGAGCGUCAAACAUACAAUGGUGGGACAAGAAUCU

>N1240

CGACACACACACCCUCCCAAGAUCAAGAUUUCUUUUUUUUUUUUUUUUUUU

>N1241

UGGCCAGGAUGGUCUGGAUCUCCUGACCUCGUAAUCCGCCCACCUCGGCCU

>N1242

GCAAGUUUUUAUUAGGGAUUUUCAAAAGGGGAGGGAGUGUGCAAAUAGGUA

>N1243

GUCAGAGUUUAAGGUUAUCUCUCUUAUUCCCUGAACAAUUGCUGUUAUCCG

>N1244

AAUUAUUACAGGGUCCUGAGGCAACAUACAUCCUCCUCGGCUGACAGGAUC

>N1245

AGUAAAGACAGGCAUAGGAAAUCACAAGGGUAUUGAUUGGGGUAGUGAUAC

>N1246

UGGAAAUACUGACUCAGUUAUGCAGAUAAUCAAAUAAUGACAGAUUUCAUU

>N1247

UAAAUACUACCACUAUUUUCCCUAAAGCAAAGGCUCACUGUUUUCAAGAAA

>N1248

CAACAGAGUGUGGGGAGCAGAGUCCACAAUGUGAGGUAAUGCUGAGCAGCG

>N1249

UAAUUUUCUUUAUCAAAUGAUCAAUAGGCCACAUCAUUUUCUCUUCUGAAC

>N1250

AUCUUUUUCCAAUAUGGAUAGACUAAGAAUUUUCUAAAUGUUUAAGUUCUU

>N1251

GUGAGACCUCAUCAGAAUGGUCUUUACUAGCCAUAUUUCUACCAAUAACCG

>N1252

UAUAUCUACUUAGGUAUUCACUAAGAAAAUUGAGGAUUUCUGAACUUCAGG

>N1253

CAUGGCCUAUAAGCAACAGAAAUUUAUUUCCUACAGUUCUGGAGGCUGAGG

>N1254

UAUGAGGUCACUACCCCCAUGAGCUAGUUACAUUCCAAGUGCCCCAUCUCU

>N1255

ACACUAAGCAUGAAAAUUUAUCAUCACAUUAAACAUAUCAAUUUUGUGGGC

>N1256

AUUACUGAUGUUGGGCAUUUUUUUCAUAUUCUUGUCAUAAAGAUAUUUGUG

>N1257

UGAAAAAUGGAAUUAGAAUUUUGACAGAAUUUCAUCGACUAUAUAGAUUGG

>N1258

AAAGACAUUUAACAAUAUUACUUUAAUCCAUAAACAUUACACACACAAACU

>N1259

CUUCAAUUUCUUUUAUCAACAUUUUAUAGUUUUCAGCAUAUAGGUCUUUCU

>N1260

ACUUCAUCACCCAGGUAUUAAGCCCACUACCCAAUAGUGAUCUUUUCUGCU

>N1261

CUCCCACUAUCCACCCUAAAGUAGAACCCAGUGUCUGCUGUUUCCUUCUUA

>N1262

ACUGCUUUCCACAAUGAUUGAACAAAUUUACACUCCCACCAACAGUGUAUU

>N1263

GUACCAGUACUAUGCUGUUUUGGAGAUUGUACCUCUGUAGUAUAGUUUGAU

>N1264

UGAUAGCAAUAGCAUUUAAUCUGUAAAUUGCUUUGGGCAGUAUGGCCAUUC

>N1265

GAUCUUGUUGGUGUAUAGGAAUGCUAGUGACUUUUGUACAUUGAUUUUUGG

>N1266

UUUGCUGAAGUUGUUUAUCAGCUCAAAGAGAUUUUGGCCCAAGACUAUGGC

>N1267

ACAUUUAUUGAUUUGUGUAUGUUGAACCAACCUUGCGUCCCAGGAAUAAAC

>N1268

UUUUGCAUCAAAGUUCAUAAAGUAUAUUGGCCUGAAGUUUUCCUUUUUUGU

>N1269

GAAUUUGGCUAUGAAUCCAUCAUGUACUGGGCUUUUUUUUUUUUUUUUUUA

>N1270

CUAUUUAUUACUGAUUCAACUUCAAAGCUCAUUAUUGGUCUGUUCAGGGAG

>N1271

CUUGGCCUCCCAAAGUGCUGAAAUUACAGGCAUGAGGCACAGUGCCCAGCU

>N1272

AGCAAUUUUCUUGGUCUUGACAUCUAUUCCUAUUGUGCUGUGGUCCAUGAA

>N1273

GUCCUCCUGUGCUGGGUGCAUAUAUAUUUCAGAUAGUUAGGUAUUCUUAUG

>N1274

UUGUCUUAAAGCAAAAAAAAAAAAAAAUACAUAUUUGGUUAGUUUGUACCU

>N1275

AUUGAUGCUAGAGAGGCAAAAUAAAAAAAACAAACCUGGACUCAAAUAAAA

>N1276

GUGUGUUUAAGUGUGUUUUUGUAUUACCUGGUAGCAGUCUUUCUUUUCUAU

>N1277

CUGAAAAGGAUCUUAUUUCUCCUUCACUUAGGAAGCUUAGUUUGGCUGGAU

>N1278

UAUUGGGGAUGAUCCUCUUAUGUAGAUUCUUGCAGGAGUUCUCUGUAUUUG

>N1279

UGGUGUGCCAUUUGGAGGACAUAUGACAAUCUGGCCACUUGAGUUACUGGC

>N1280

GCCCCACCCCAGAAAGAUGCAGGUCAGCAGUUGCUCAGUGCAAUUAGCCCG

>N1281

GGAGGUGGCUGGAGACCCCGAUAGAAAGGUCCCACCCAGUAAGGAGAAAUA

>N1282

ACUUCAGCUCCUGGUUGCCUUGGACACUUCAAAGCCUGAACGCUGGAAUAC

>N1283

CCAAGCUAAUGGGUCUUAUCCUGUGAGGUGCCAUGGAAGUGGGGCCUGCAU

>N1284

CUCAGUCCCUUGGAUUCAGCCUCUUACCUAGGGGUAUGUACAGGGAUCUAG

>N1285

GCAUGCCUGAGCAGCUGCUCCGCUGAGACUCCAUAGCUCUGUUUGUCCACU

>N1286

UCAGUAUGCUUGUAUUUUAUUAAAGAGUUUAACAUCUAUGUUCAUCAGAGA

>N1287

GUGUAUACCUGCAGUAUCAGUUGUAAUGUUUCUGCUUUUAUUAAUAAUAUA

>N1288

UCUGGUGCCAAGCUAGCCACUAUAGACCUGAGAUUCAGGCAUAAACCCAAC

>N1289

CUAAGCUCCAGUGCCACCCUAGUGGAUCCAGGCUCCAAGCUGGUCAUUGUC

>N1290

AGAAAAGAGUUAUAAUUGCCUCAUCAACCUGUAGGCUGUACCAACAUGGCA

>N1291

GUCACAUGAUAAGAAUGGGAGUAAGAGCGGGAGGUAGAGGCCACGCACUUG

>N1292

GUUCUCACAUGAACUCAGAGCAAGAACUCAUUAUUACCAAGGAGAUGGUUA

>N1293

AUGAGGGAACUGCCCCCAUGAUCCAAUGACCUCCCACCAGGCCCCACUUCC

>N1294

AUAAACCCCAUUAGCUGGCUUACCUAGAAUCUUAGGGCUGACUGGUGGAGG

>N1295

CUGAAUCCAGUUUGUAAAGACCGAAAAAGCUGCCUACUUCUUUAAAUAUGG

>N1296

UCUGAAAAAAGUUUAGUGAGCCACAAGAAAACACAAACUGAACAAUAAUCA

>N1297

ACAAAGAGUUGUUCAACAAAUAGAAACCAUUAAAAUGAACGAAAUUCAGAA

>N1298

ACAAUAACUGAACUGAAAAUUUCAGAGAGCUUCAAUAGCAGAUUUGAAUUC

>N1299

UGAGUUCAAAGGCACAUCGUUUGAAAUUACCCAGUACGAGGAACAAAAAGG

>N1300

AAUUUCAGAAGCAGAGAAAGAUAAAAAAGCAGAACAUUUAUUUAAAGAAAA

>N1301

UCCCCAAAUCUGGAGAGGGAAAUGAACAUCUAGGUCUAUAAAGCCUAAAGU

>N1302

UCAUAGCCCCAGGAUCUAAGUUAUAACCGUGGCCCCUGCCUUCAGGCUUCA

>N1303

AGGGAUCUUCACUGAGAUGCAUUAUACUCAGUUCUCAAAAGUCAGUAACAA

>N1304

CAAAUCAGUAAGAUAAAUACAGCUUAACAGGAAGAAAUCUUUUUUUUUUCU

>N1305

UCGGUUCAAGUGAUUUUCCUGCCUCAGCCUUCCAAGUAGCUGGGAUUACAC

>N1306

CAGGCGUGAGCCACCGCACCUGGCCAAGGAAUCUUCAUAAGACUGUUAGCA

>N1307

GAAACUUUACAGGCUGAGAGAGUGAAAUGAUAUAUUCAAAGUGCUAAAAGA

>N1308

CAACCAAGAAUCUUAUACCCAGCAAAGAUGUUCUUUACCAAUGGAGGAGAC

>N1309

UUCCCAGACAAAUAAAAGCUGAAGGAAUUCAUCAUCAUCUGCCUUACCAUU

>N1310

AGAAUAAAAGUUUCAUUUUGAGUUUACUGGCAAGGAGACAGGAGGAAAUGG

>N1311

CAGCAUCAACUCAAGGAAAAGUUCCAAGUCCAAAGUCCAAAGUCUCAUCUU

>N1312

AUAGGCAUUGGGUAAACCACUUCAAAACAAAGAAAUCAGCCACAAGAAAGU

>N1313

AGAUGGGGAUCUCAUUUUGUUGCCCAGGUUGGUCUCAAACUCCUGGCCUCA

>N1314

CUACCUCGGCCUCCCAAAUGCUGGGAUUACACGCAUGAACCACCAUGCUCC

>N1315

AAUUAAAUUUUAAAGUGCCAAAAUAAUCUCCUUUGACUCCAUGUCCCAUAU

>N1316

CCUGCGAUGGGAAGGGCUGCCUCAAAAAUCUCUGAAAUGUCUUUAAGGCCG

>N1317

AACUCUCAUGGCAUAAACACAAUGCAGCCAAGUUCUUUGCUAAGGCAUAAC

>N1318

AUGGUUCUGCAGGCUGUACAAGCAUAGUGCUGGCAUCAGCUCAGCUUCUAC

>N1319

UUUCCCCUCCAAACCCUGCUAUUAAAGGUUCUCUGUGAGGGCUCUGCCCCA

>N1320

UCUUUCUGGGCACCCAGGCUUUCUCAUACAUCCUCUGAAAUCUUGGUGGAU

>N1321

CUCUUCCAACCUCUGCCUGUUACCCAGUUCCAAAGCUGCUUCCACAUUUUA

>N1322

GUCAUAAUGAUGCAUUUAUUCAGGGAAAAUUUUAAUUUUUUUUUUCUUCUA

>N1323

UUCACCAGAGGAAGAUCCUUACCAUAGUUGAUGAUACAAGUAAACCUUCUU

>N1324

UGCAAUCUCAGCUCCGCUCAUUGGAAUAUUGACAUUUACAAUGCCACAGUC

>N1325

AAUUGGAGCAAAAGUCUCUGUGUAUAGAAGCAGGAACAGACACAUCACAUG

>N1326

AACAAGAGAGAGAGAAUGGGUAGGGAAGGUUCCACACAUUUAAACAACUGG

>N1327

CCAACACUGGGGAUCACAAUUCAACAUGAAAUUUGGCAGCAACACAUAUUU

>N1328

CAAAGUCCAUCAAUUUACCCCCUGUAUAAGCAUGUGUGUGUGCCUGCCCUA

>N1329

CACAAUAUGAACAAAGCCACUGGGAAGUUUGGACUGGGCAGAGCCCACUGU

>N1330

CUUGCGGGGAGGGGUGGCUGUGUGUACAGCUUCAGCAGACUUAAAUAUUCA

>N1331

CUCUAAAGAGAGCAGCAGAUCUCCCAGCACAGUGCUCGAGCUCUGCUAAGG

>N1332

CCUCACUGUUAGAAGGAAAACUAACAAACAGUAAGCAAUAGCAUCAACAUG

>N1333

AAACAACCAUGCAAAAACCCCAUCCAAAGGUUACCAACAGCAAAGACCAAA

>N1334

AAGGAGCAUGUUCUAACCCAAUGCAAGGAAGCUAAGGACCUUGAUAAAAGA

>N1335

AGCGCAAGAACCUCGUGAAGCAUACACAAGUAUCAGUAGCUGAAUCGAUCC

>N1336

CCUAUGUUUGAUUGGUGUACCUGAAAGUGAUGGGGAGAAUGGAACCAAGCA

>N1337

AGGAGCUACAGAGAACACCACAAAGACACUCCUCAAGAAGAGCAACCCCAC

>N1338

AAACCAGAAGAGAGUGGGGGCCAAUAUUCAACAUUCUUAAAGGAAAGAAUC

>N1339

ACCAUUAACACUAUGAAGAAACUGCAUCAACUAAUGGGGAAAAUAACCAGG

>N1340

CAGGAGACCCAUCUCACAUGCAAAGACACACAUAGGCUCAAAAUAAAGGGU

>N1341

UCUACCAAGCAAAUGAAAAGCAAAAAACAAAACAAAACAAAACAAAAAGCA

>N1342

UAGAGGAGGAAGAGCAAACAAAUUCAAAAGCUAGCAGAAGAUGAGAAAUAC

>N1343

GAGGGAAGAAUCAAACAGAGAAAAUAAAAAAUGAUAAAAAGGAUAUCACCA

>N1344

CUAGAAGAAAUGGAUAAAUUCAUGGACACUUACACACUCCCAAGACAAAAU

>N1345

UCAAAUCCCUGAAUAAACCAAUAACAAGUUCUGAAGUUGAGGCAGUAAUUG

>N1346

AGAAAAUUUCAGGCCAAUAUCAUUGAUGAACAUCGAUGCAAAAAUCCUCAA

>N1347

GCAUAUCAAAUUUAGCAGCACAUUAAAAAGCUUAUCCACCACGAUCACAUG

>N1348

CUGGGAUGCAAGGCUGGUUCAACAUAUGCAAAGCAAUAAAUGUAAUCCAUC

>N1349

AACCAAUGACAAAAACCACGUGAUUAUCUCAAUAGAUGCAGAAAAAGCCUA

>N1350

AAUGGGAGCUAUUUAUGACAAACGCACAUCCAAAAUCAUACUGAAUGGGCU

>N1351

UAGUAUUGGAAGUUCUGGUCAGGGCAAUCAGGUAAGAGAAGGAUAUAAAGA

>N1352

AGAAAAUCAAUGUGCAAAAAUUACAAGUAUUCCUAUACACCAAUAAUAGAU

>N1353

AGAGAUGUGAAGGACCUUUUCAAGGAGAACUACAAACCAUUGCUGGAGGAC

>N1354

ACACAAACAAAUGGAAAAACAUUCCAUGCUCAUGGAUAGGAAGAAUCAAUC

>N1355

GGCCAUACUGCCCAAAGUAAUUUAUAGAUUCAAUGCUAUUCCCAUGAAGCU

>N1356

UUCUUCACAGAAUUAGAAAAAAACUACUUUAAAUUUCAUAUGGAACCAAAU

>N1357

AUAGACAAAGCAAUCCUAAGCGAAAAGAACAAAGCUUAAGGCAUCAUGCUU

>N1358

UUCAACAAACCUGACAAAAACAAGCAAUGGGGAAAGGAUUCUCUAUUUAAC

>N1359

GGGAAAACUGGUUAGCCAUAUGCAGAAAACAGAAACUGGACCCCUUCCUUU

>N1360

GAAGAUAACCUAAGCAAUACCAUUCAGGACAUAGGCAUGGGAAAAGACUUA

>N1361

CUUCUGCACAGCAAAAAAAAAAAAAAAAAAAAAAAAAAAAACUAUUGUCAG

>N1362

AAGAUAUGCACAGACAUUUUUCAAAAGAAGACAUUUAUGCAGCGAACAAAA

>N1363

GAUCUAGAACUAGAAAUCCCAUUUAACCCAACGAUCCCAUUACUGGGUAUG

>N1364

AACAGCAAAGACUUGGAACAAACCCAAGGGCCCAUCAAUGAUAGACUGGAC

>N1365

AGUUGUCAAAUAAUAAAAACCAAAAACAGUGUUCAAGGCUAGAAUCUAAUG

>N1366

UCCCUUUUUGUACCAGAUACAAAUUAUAAUAAAACUAAUUUGUGUGCAAAA

>N1367

AAGAGGCUUCCUUUCGGGUCUAAAAAGUCAACCUUAAUUCCUCAAAGUAAC

>N1368

UUAUAAAAGAAAACAGAUUAUUAUUAAACUUAUAUAAAUAAUUAUAUUGCG

>N1369

GAAAAAAGUUUUCUUGACUCUGGAAAACAGAACACAAAAAGAAUUAGCAAA

>N1370

AAUUUGACAUAAUAAUUACUAGAACAUAUUAAGACAUCAGAAUUUUAGAAC

>N1371

GAUUUCCAGGGGCUCUAAUAUCCAAAAAGUUAGUUUGAGGUCAAAAAGGCU

>N1372

AUGGAGGGAAGACACAGGUGCUGAGAUGAAGGGGGAGUAAGCUCAGAACCG

>N1373

UCUGUAGUGGAGUGUGGCCAGGGACACCCAUUCCCCAACGCUCCACUUGCG

>N1374

ACUUUAACCCUAGGGAAACUGUCAGACCUGAAAUCUGACAGGAUGGUCUUG

>N1375

UGUGUCUGCUUGCAGUGCAGCCCUCAGGUACCUCCUGGGGGCCUGCAUCAC

>N1376

CUGGCAGACUCUGCCUGACAGGCAGAGUGCUCCAGUAGAGUGGCCCCUAUG

>N1377

CCCAUCCCUGUCAGCACAUGUGUGCACAUGCAUUCUGCUAUUCUAUGGCUU

>N1378

CACUACCACCAGUGUGAACCCUUAUACAGUGGCUUUCAGAGGCCUCCUGGA

>N1379

AACCUGCCACAGCCAAAGAGUGUGCACACCACUGUUCUGCUGCUGCUGCUG

>N1380

GUGUUGUUACCAGCAGUUCAGGAGGAACUAGGCCCCUCCUGCACAACAGGA

>N1381

UAGCAUAAGAACUCUGACAACUCAAAAAUCCAGAGUGUUUUAUUUUCUCCC

>N1382

AAGUUUAAACAGCAGAAUAGACCUAAUUGAGGAAAGAAUCUCAGAGUCUGC

>N1383

UCUGAAAGGAGGCAGUCAGACAAAAAUAAAGACAAAAGAAUGAAAAGGAAU

>N1384

AUGAGGAAAGUAUAAGCAACUUGGAAAACAUAUUUCAGGAUAUCAUUUAUG

>N1385

ACAAAUGUUAAAGGCAGCUAGAGAGAAAGGGCAGGUCACCUAAAAAGGAAA

>N1386

ACAAAGGAGAAAUAAGAUCUUUUUCAGAGAAGCAAAUGCUGAGGGCAUUCA

>N1387

GUUAGCAUAACAACCAGCUAAUGACACAAUAACAGGAUCAAAUCCACAUGA

>N1388

UAGGCUUAAAAUAAAGAAGUGGAGAAAAAUCUACUAAAAGCAAAUAGAAAA

>N1389

GAGGGGUUGCAACCCUAAUUUCAGAAAAAAAGAACAUUAAACUAACAAAGG

>N1390

ACAAAGAAGGGAAUUACAUAAUGGUAAAGACUUCAAUUCAAAAAGAAAAUG

>N1391

CCAAAUGAGCCUGAUAAACAUCUACAGAACUCUCCACAAAAACACAAUUGA

>N1392

GAAUGGCUUUUAGAUAAACAAUGAAAUUAAGGCAGAAAUCAAGAAGUUCUU

>N1393

GAAAACAAAGAUAUAGCAUGGUGGAAUCUCUGGGACACAGCUAAGGUAAUU

>N1394

CCACAACUAAAAGAACUAGAGAAGCAAGAGCAAACCAACCACAGUGCUAGA

>N1395

ACCCAAGAGCUGAUGUUUUGAUACAAUUAAUAAGAUAGAUAGACCACUAGA

>N1396

AAGAAAAGAGAGAAGAUUCAAAUAAACACAAUUAGAAACAAAAAAAGGGGG

>N1397

UAGAAAAUCUAGAAGAAUUGAAUAAAUUCCUGCACACAUACACCCUCCCAC

>N1398

UCCCCAGUUCUUUCUAUGAGACCAGAAUCAUCCUGAUACCAAAAACUGGCC

>N1399

AUACUGGUAAGCUAAAUCCAACAGCACAUCAAAAAGCUAAUCCACCAUGGA

>N1400

AAACAGAACUAAAGACAAAAACCACAUGAUUAUCUCAAUAGACACAGAAAU

>N1401

AAAAUGCAACAUUCCUUCAUGUCAAAAACUGUGAAUAAACUGGGUAUUGAU

>N1402

AAAGAAAAUGUGUUACAUAUACAUCAUGGAAUACUAUGCAGCCAUAAAAAU

>N1403

AUGUCCUUUGCAACAACGUGAAUGGAAUUGGAGGCCAUUAUCCUUAGCAAC

>N1404

UGUCUGAUCUGGAAUCCUUAUGAGGAUAGAGUUACUUUAAUAUCAUAGGUA

>N1405

AAUAGUCAUUUAUUUAGCCAAAGUGAUAACCAAAAUAUUUCAAAAAGCAAU

>N1406

ACAAUUUAAAAACUAUAUUUAUCAAAGACUGCUAAAAUCACAUAAACUUGA

>N1407

UACAUAAAAACAUACUCACAUGUGCACAUAAAAAUACAGAGAGAAGCAAAA

>N1408

UUGAUUUUAAAACUCUAGCCAUGAAACUGGCAGAACUCACUCAUCCUAAAC

>N1409

UUAAGCUUAUGUCCCUGCAAAUUGAAUAAGUCAAUGCCCACUUGUCCCUGA

>N1410

AGAGAAAGUCCACGCUCCUUUAAGAAGGAAUUUGUGUGUGUGUGUGGUAGG

>N1411

UGCACAAAAGCUUCUGCACAGCGAAAGAAACUACCAUCAGAGUGAACAGGC

>N1412

GAACUCAAACAAAUUUACAAGAAAAAAACAAACAACCCCAUCAAAAAGUGU

>N1413

GCAAGAACAAAAAACCAAACACCGCAUAGUCUCACUCGUAGGUGGGAAUUU

>N1414

AGCAUGGCACAUGAAUACAUAUGUAACUAACCUGCACAUUGUGCACAUGUC

>N1415

UAAAGUAUAAUAAUAAUAAAAUAAAAUAAAUGAAAUCAUAAACAUAUUCAU

>N1416

UAGAUUUAAACCCAACUAUGUCAGUAAUCACACAGCAUGCAAAGGAUCUAA

>N1417

UAGAAGCAGAUUUUCAGGUUGGAUUAAAAAAGCAAGACACCAACUGUAUCU

>N1418

AAACCCACUCUUAAUAUAAAAACACAAAUAAGUUAAAAGUAAAAGAAUGGG

>N1419

AAAAGUAGACAGAAAAUUAGUUUAUAGGAGACUUGAUCACACUAUCAACCA

>N1420

CAGAAAUAUAUCUGGAAAAUCCCCAAAUAUUUGGAAACUAAGUUACAUUUA

>N1421

AUGCUUGUAAUAGAAAAAAAAGAAAAAAUUUAUAUCAAUGACAUCAGUUUA

>N1422

UGAAACUACCAAAGUUCACUUAAGAAGAAAUACAUAAUCUGAACAUUCCUU

>N1423

UGUUGCAGGAAGUCAGGGACCCCGAAUGGAGGGACCGGCUGGAGCUGCAGG

>N1424

CAAUAUGAAAUCAGUCCACCUUGAAAAAGAACAGAAUAAUGGCAAUUUUUA

>N1425

AAGGAAUAUUAAUAUUAAUAUUAAUACCCUGGGAAAGGAAUGCAUUCCUGC

>N1426

UGAUCUUUGUUGGACCCUUAUCAGUAGUUCUGCUUUUGCCCUUUGUCCUGG

>N1427

AUCCUUUUCUGAAUUCUCUUUAAUCAGUGUUAAUAUGAGGUACACAUGUCU

>N1428

AAAGUCUGGUGGCAACAAAGGAAUGAGAAAAGACAAGUUAAGAUUUAAAGC

>N1429

GGCCAGUUGCUGGAGGGAAGGCUGCAAAAGGCCCAGAGCUCUGGCUGCCUG

>N1430

UGUUAGAUUUUCCUCUCUUCCUUCUAGAUAGUGUUUAACCUUUUCCCAAUG

>N1431

GCAUUAUAAGAUUGAGGAGUAAUACAAAAAUCAGAAGUAUUCCAAUCACAG

>N1432

UUCUAUGUUCCAGGUUUAUAAUUCAAUCCCCCAUUCAUGGUACUGUUUGAA

>N1433

AAUCUGAUUAGCUAAUUUUUGAUCAAUUUGUUUCUGGGUAUUCCAAAGCUU

>N1434

CUUUCCAAGGUCUAUUAAGGGAAACAGGUAUCCAAACUCCCUCUCAGGCUG

>N1435

AGGAACCAUAUCACUGCCUUCCCAGAAGACAGGAAAGUCGGCUUCUAUUCG

>N1436

CAGCUUUGUUUGUCAUAAUCUAAACAUCCUGCUGCAGGCCCCAGACAUAUC

>N1437

AAUACCAAUGGACACAUUUAUUAACAUACCUUCCUCCUCUGGAUGAGUAGC

>N1438

UCUUAAUCUGCCCCCAGGUCGGUGGAGUUGACUGGUGAGUCUUGCUGGUCC

>N1439

AGCAGAAAUCUUCAUUUGAGCCAUCAGGCAAGUUGGGAGUGCAAAGUUCCC

>N1440

AGCUGAUUUUCUCCUGGUGAAAUACAAGAAAACCCUCUACCCCAUGUAAUA

>N1441

AACCAGAGACAAUAUUUACAGGCUGAUUAAAAUCUUUCAACACAGUUAUAG

>N1442

UGGAUAAUGAUUGUUAAGGUUUCCUAUGAAUUCAGCUUGCCAAUUAACUUU

>N1443

UACUUGCCAAUUAAAGGAGUUAAUAAAAACCUGUCGAAUCUGGUUUUUGUU

>N1444

UUGUUGUAUUUUUUCCUCAAUCAUUAGUGCUGGUUGUAAGCCACAGAUUGC

>N1445

UUGUCCCAUAUUUAACGUUGGGAGGAGCCUGAAGCAUCUCUUUGGAAUAAA

>N1446

ACUGGAGCUAGAUGUUGAGGUGAUCAGGCCCAACCCCUUUAAUGCCUGCAA

>N1447

UAGAUGAAACGUGGUGCUCACUGCAAAUGUAGUCCCUAACAGAGAACUUUU

>N1448

CAGGUUUAGGCUUCAGAAACAAAAUAUAAUCCUAGUCUUUUAAGGUAUCUC

>N1449

AACGGUGAGCUGGGACCCCAUUACAAUACUCUCAAAGUUUCCAUUUUAGUU

>N1450

GGCCCAUCCUAGUCAGUCCAGUGAAAGUCAUUUACUCUCUUUUGCUUCUAG

>N1451

AUGGACAUACCCUGGAAAUAAGGACACACUGUGGACUCAGAGGACAUACCC

>N1452

CAAGGGUCUUUCAGAAUAACUGAUAACUCUCUAGUUUUGGCCCAGAAGUUU

>N1453

GUUGUUCUGCCUGACUUUGCCUUUUAGAAUUCUGACAAUGGAGUAGCUAUA

>N1454

UAAAUUGAAACAAAAUAAAAUUUAAAAUUCAGAACUGCAAUCACACUCGUA

>N1455

CGUCUGGUAAACAUGUUUUCAGACUAUAUUCUCGCUGGUUCCCGUUUCCUU

>N1456

GCUGCUCAGAGGAUACUUGUUUUACAAAUAAAUGUUUCCUCUCGCUGGUUU

>N1457

AGCUGGUGGGAAGAGGAACAUUGGGAUGUGUAAGGGGCACAGCUCCAUAUG

>N1458

AAGACUGCACCCAGGUCAGGAUUGGAGGCUCUGCAGUCUGAGGUCUUGUUA

>N1459

GGCAUUGAGCUCCCCUUUAGCGGGUAGGAGGAGGGAGAUGCCACCUGGUGG

>N1460

GCCACCUGAAGUCAGCACCUACAGAAGCACAGUCUCCUGGCUUUGCCUCUA

>N1461

AGCAGAGCAGCAUUAAAGAGCCCACACACUAGAAGGAGGAUAUGAAGAAAC

>N1462

AGCUGUUUCCUAUACCCUAUUCUUGAAGGAUAAAGAAAUAGUCAUUCAAAA

>N1463

CAUUGCAAAAUGUUUGUAGGUAGCAAUGAAAUAAAACAUUUAAAAUUAAUC

>N1464

UGGACAUAAUGAUCAAUCAAAGGCAAGAAGCAGGGAGAUUAUCCAUUUAUC

>N1465

AGACAUAUGUGGUCUUCUCAUCACAAAGCUUGUAGUAUAAGAGGAGACAGA

>N1466

AGCAAUUAACUUCCUAAAGUAAUACAUAUUCUGUAAGAAGCCUCUGGGGUA

>N1467

GUGUGGCUCACACAUAGGAGAGAGGAGAGAGCUGAUCACACAGGGCCUAUA

>N1468

GAAAUCAAUGUAGCAAGCUCAUGGCAAAAAGAAAGUUGUUGUUGACUACAA

>N1469

AUUCUAGUUGCUUCCAUUUCCCUAAAAUGACAUGGUUAUGUUUCUUGAUCG

>N1470

AGAUUAAAUAUCCUCUGAUUGACCCAGGGCUCGGCACAGUUUCUUACCUCG

>N1471

UUUGCUGGUUUAAUAAACUAUCCCAAAGUUUCAUAGCUUAAAACAUCAGCU

>N1472

AGCCUUCUGCUGUGAUGCUAACUAAAAAUGUGAACGCCAGCCCACGUUUUG

>N1473

GAGGCCGAGGCGGGUGGAUCAUCUGAGGUCAGCAGUUUGAGACCGGCCUGG

>N1474

GAAACCCCGUCUCUACUAAAAAUACAAAAAUUAUCCGGGCAUGGUGGUGCC

>N1475

GUAAGGCAGCAAAGGGCAAGAACGGAAGUGGAGAGUAAGGAUGGGAUACUU

>N1476

AUGAAAAUCUGUUUGUAUUUUCUGCAUAUUCUCUGGCAACCUUGCCCCAUA

>N1477

UCUUGCAUUCAGAGUCACAAUGAUCAUCUUACCCAUGUGGUUUUUGAGAAU

>N1478

UUCUUUGUUUGCAGUAGGUAAUCUUAGAGAUGGAGAUGAUUGUAGAAUUAA

>N1479

AUUGAAGAACAGAGACCUGUCUGGAAAAUCGAUCUCUACAAAUUCAAUUAC

>N1480

GUUCUCUUCUUUUCUAGAGAAAGAUAGUUGCAACCUCACCUCCCUCACUCA

>N1481

AUGUUGUUUAUAAUAAAACUUUUCAAUUAUCUCAUAUUUGUGCAGAAUUGU

>N1482

GGAUCUUCAUUAUGUGCUGAUGGAAAUUAUGAGUAACCAAGGUUUCUGAGU

>N1483

CUUUUUUUUAAAUUUUAUUAUUAUUAUACUUUAAGUUUUAGGGUACAUGUC

>N1484

GUUCCAAGUCUUUGCUAUUGUGAACAGUGCUGCAAUAAACAUACGUGUGCG

>N1485

UUUACAGUCCCACCAACAGUGUAAAAGUGUUCCUAUUUCUCCACAUCCUCG

>N1486

UUGUUGCCAUUGCUUUUGGUAUUUUAAACAUGAAGUCCUUGACCAUGACUU

>N1487

GUUUUUGUCAGGUUUGUCAAAGAUCAGAUGGUUGUAGAUAUGCAGCAUUAU

>N1488

GCUGAGGAUUGACUUGGUGAUGCAGACUCUUUUUUGGUUCCGUAUGAAGUG

>N1489

UGUGAUUUUUGCUCAUUGAUUUGGUAUCCUGAGACUUUGCUGAAGUUGCCU

>N1490

GGACAAUUUGACUUCCUCUUUUCCUAACUGAAUACCCUUUAUUUCCGUCUG

>N1491

GGGUUUGUCAUAGAUAGCUCUUAUUACUUUGAGAUACGUCCCAUCAAUAUU

>N1492

AGAGUUUUUAGCAUGAAGGUUGUUGAAUUUUGUCAAAGGCCUUUUCUGCAG

>N1493

GUUUCCAUGUAUUUGAGCAGUUUUGAGUGAGUUUCUUAAUCCUGAGUUCUA

>N1494

ACUGUGGUCUGAGAGACAGUUUGUUAUAAUUUCUGUUCUUUUACAUUUGCU

>N1495

UUCAACUUUGUUGAAUCUGACAAUUAUGUGUCUUGGAGUUGCUCUUCUUGU

>N1496

CAUAUCUUCCUUAUUUCCCCAAAGCACAAAUAAGUUUAGACUGUACAUUUA

>N1497

GCAAAGGCUUAAAUAUAAGAGAAGCACUACACAAUGAAAUGUGCGAUUCCA

>N1498

UUCAGGUUAAUGCUGCAGAUGAGGGAAAGAAAAAGGUUCUGACUUAAGAAA

>N1499

AUGAGAAGCCCAAGUGAAAAACUCUAAGAAGAGUGUUGCCUGUUUGCCUCG

>N1500

GGCAGACAGAAGGUCCUUGGCAAAUACUAUGCAUUGUGAUUAAUACAAGAA

>N1501

UUUCUUUACUUAUUCUUGUGCACCUAAUUGAUUUAUCUUGUUUGAUUGCAC

>N1502

GCCUCCAAUGUUUCCCAGUUAAACAAGUAAGGUUCUGGGUUACAGUAGUAU

>N1503

AGACUGGAUAUUUAAAUGUUUGCCAACAUGAAAGGUCUUCCACUGAGGCAA

>N1504

UAAAAUCAUUUACACUUGUCAAGUAAGAAUUCUAGCUAGAUAUUUGACACU

>N1505

UGGAACCCAACAUGAUUUUGCCCUGAUUGCCCCCCAAAAGGAUUUCAUCUU

>N1506

UACAGCCACUUAUACAGAAUAUUUGAUCAGGAAAAUUGAAGCAUAUGUAGA

>N1507

AGGUCUUGGAAGCUUGCUGAUGCACAGAGCUGGGUGGUUUUGAGGCGCUCG

>N1508

UUGUCCACCAUCCAAAUCUCAUGUGAAUGUCUCAUCUUGAUUUAUUCUCAC

>N1509

AGUAGGCAGCAAACUGUUUCUCAUAAAUGCAGAAUUUUGGCAGACUGACAG

>N1510

ACAUCUGAAGCUUGUUAGUAAUCAUAUGAACCUGUGAUCAAUUAAGAAGCU

>N1511

AAGGGACCUGAAGAGGCCUGCAGGGACAAAUAGAGAUAAGGAGGGAUAAAG

>N1512

AACAGAAAAAACCAAAGCCCAGAAAAAAUUAAAACCAACCAGACGAAUGAG

>N1513

UCAUUGCUCUGCAGUAUUGACUCAGACAAUGGCUAAUUUGGUAGCUGACAU

>N1514

UUGUGGACCUUUGCCAACAGGAUACAUGGGAUUAAUUUUGGGCAAAAGCUU

>N1515

UUGUACCCCUCUCUACAUAAGAAGAAGUGAGGAGGUCAGGGAUUUGGAAGA

>N1516

AGGAACAGCUCAAAGUGUUCAACAGAGUGCUGAGAUUCUGUCAUGUCUCAU

>N1517

GGGGGUGGAGGGCAGGAGAGGGAGGAUGAGAAUUGGCCUGUACUGCCCUUC

>N1518

AGACACCUGUACUAUCUUCUGAAGCAGAAACAGAGACCCUAAUAGUUAGGG

>N1519

CCUCUCAUUAUCAAUGGAAAGUUUUACCCUGCGGUAAUUAACCAAAGAGAG

>N1520

UUACAGGUUGCAGAGCAGACGCUUCAGCAACGGCAUGCCUCCCGGCUACAG

>N1521

UUUUUGCUUCUGCUUUGCUAGAUUUACUAACGUGGGGGCGAGGGUAUGCUC

>N1522

UGCCUUAUUCAAUUAAUUCAAAAACAAAAAGGGAGAAAUGUUGGAGGACAU

>N1523

AUGCAGAAUGUUGGCAGACCGACAAACUGUGUCUUCCACCCAGAAGGGAUA

>N1524

GAACCUGUGUUCAAUUAAGCAGCUGACCAAUCAUUACCUUCUGCUCCCUGU

>N1525

UGUCAUAGUGACAGUCUCAAGUAAUAACCAUGGCAGUCAGCCACAAGAUGC

>N1526

CUGAAGCAUUCAUAGAGCAGCAUUCAUCAUCUAUCUGACCUUUUUGUGGGG

>N1527

CUUGCAGUUCAUUCACUUGAAGUUCAGUUUUCUGCCUAGUUUCCACCAUCA

>N1528

AGACAGCACUGGGCAUUUCUAUACCAAGCUCACUCUCCUCCCUCUGGCUUA

>N1529

UCUCAGGAAUGCAGAUGACUGAUCAAUUCCAGGAUGUUAGAGAGCCUGACU

>N1530

CCUGUGGUGACCAGACAUGACCUUCAUUUUUGCUUUCUCUUUCCACUCUAC

>N1531

AAAGUCAUAUCCUUCUGGUAAAAAGACGGUAUUUUGCCUGAUUUGAUUGAG

>N1532

UCCACUCUACACCUACUUCCUCAGAAAGUUUGUAUUUUAACAAGCUUAAAU

>N1533

GCUAGAGGUUCUUUGAGAAUGCAAUAUUUGCUUCAUAGACAUAUAGAAAAU

>N1534

UGUGCAGAACGUACAGGUUUGUUACAUAGGUAUACACGUGCCAUGGUGGUG

>N1535

CUCUCACUUAUGAGUGAGAACAUGCAGUGUUUGGUUUUCUGUUCCUGUGUA

>N1536

GGCUGCAUAGUAUUCCAUGGUAUGUAUGUGCCACAUUUUCUUUAUCCAGUU

>N1537

AAUGGUUGAACUAAUUUACACUCCCACUCUUGGGUAUACACCCAAGGGAUC

>N1538

AAUUAGUUUGACCAUUGUGGAAGACAGUGUGGCAAUUCCUCAAGGAUCUAA

>N1539

GAACCAACCCAAAUGUCCAUCAGUGAUAGACUGGAUUAAGAAAAUGUGGCG

>N1540

AUGGAAUACUAUGCAGCCAUAAAAAAGGAUGAGUUCAUGUCCUUUGUAGGC

>N1541

AGCUGGAAACCAUCAUUCUCAGCAAACUAUCACCAGGACAGAAAACCAAAA

>N1542

CUCACUCAUAGGUGGGAAUUGAACAAUGAGAACACAUGGACACAGGGUGGU

>N1543

UUUUAAAAAGUGUUCCUAUUUCUCCACAUCCUCUCCAGCAUCUGUUGUUUU

>N1544

AAUGAUGGCCAUUCUAAGUAUUAUGAGAUGGUAUCUCAUUGUGGAUUUGAU

>N1545

AAGUGUCUGUGGAUGUUGUUUACCCACUUUUUGAUGGGGUUGUUUGUUUUU

>N1546

UGUUUUAGUCAUGAAGUCUUUGCCCAUGCCUACGUCUUGAAUGGUAUUGCG

>N1547

UGAUUUUGUAACCUGAGACUUUGCUAAAGUUGCUUAUCAGCUUAAGGAGAU

>N1548

GUCAUUGGUUCUGUUUAUGUGAUGGAUUAUGUUUAUUGAUUUGCACAUGUU

>N1549

UUUUUUCCUGUAGGCAUUUAGUGCUAUAAUUUUUCCUCUAAACACUGCUUU

>N1550

CGUGCAAUUUUGAGUGAGUUUCUGAAUCUUGAGUUCUAAUUUGAUUGCACU

>N1551

UACCACUACUAUUGUGUGGGAGUCUAAGUCUCUUCAUAGGUCUCUAAGAAC

>N1552

GUUUUUAUCAGAGACUAGGAUUACAACCCCUGCUUUUUUUCUCUCUCUAUU

>N1553

AGCUCAGAGGUGUUUGUUAUUACCCACCUUCUGAAGCCUACCUCUGUCGAU

>N1554

UCACCAGCCGAGGCUGCAGAAUAGCAAAGAUUGCUGCCUGUUCCUUCCUCA

>N1555

UCUGUCCCAGGAAGAUGGGAGUUUUAUCUAUAAGCCCCUGACUGCGGCUGC

>N1556

AGCUCAAGCAUCUCAGGUUGACUUCAGACUGCUGUGCUGGCAGUGAGAACA

>N1557

CAGUCUCUCACGGCUACCCUUGGUUAGGGGAGGGAGUUCCCUGACCCCUUA

>N1558

AGCCUCUGUGUUAUUUUGGAACCAGAGUCAGACUCUGAGUUUUGAUUUUCA

>N1559

UUGGGAUAUGUUUAGAAUGUACAAGAUUUGGUUGUGAGAUUGUCAAAAGAC

>N1560

AGAAAGGGAGGGAGAGAGAGUCUGAAUGUCUGGGUCCUUCCAGAAUUGUUG

>N1561

UAUUUUGUUAUAGCACUACAAACACACACAUAUCACACACACACAUAUACG

>N1562

CAAACUAUUGUAAGUGAAAGAUAGAAAAAGGUAAUAUCUUAAAAGCCACUU

>N1563

ACACAUACAUGGGCAAAGAACGAUAAAAAUGAAGGCUGAUUUCUCAUUAGG

>N1564

AAGUACAGAGAAGGUGAUGGCAAUAAAGCUAUAUUGGAGUAAGAAAAUGAA

>N1565

GAAAACGGGUGAUUUCUGAAUUUCCAACUGAGGUACCGGGUUUAUCUCACA

>N1566

UGGAAAAUCGGGUCACUCCCAACCUAAUACUGCACUUUUACAACAGGCUUC

>N1567

CGAGGCUUGGGGAGGGGAGCCUGCCAUUGCCAAGGCUUGAGUAGGUAAACG

>N1568

CACCUCUGGGGGCAGGGCAAAGAAAAACAAAAGGCGGCAGUAAAGUCUGCC

>N1569

GCUGAGGGUCCUGACUGUUAGAAGGAAAACUAACAAACAGAAAGGACAUCA

>N1570

CCCAUCUGUAUGUCACCAUCAUCAAAGAUAAAAGGUAGAUAAAACCAUAAA

>N1571

ACAGCUCCUCACCAGCAAUGGAACAAAGCUGGAUGGAGAAUGACUUUGAUC

>N1572

AGGCUUCAGAUGAUAAAACUACUCCAAGCUAAAGGAGGAAGUUUGAACCCA

>N1573

CUUAAAAGACCUGAUGGAGCUGCAAACCAAGGCACCAGAACUACAUGAUGC

>N1574

CUCAGCAGCCGUUUCAAUCAACUGAAAGAAAGGGUAUCAGUGAUGGAAGAC

>N1575

AAAUGAAGUGAGAACAGAAGUUUAGAGAAAAAAGCAUAAAAAGAAAUGAAG

>N1576

GGGGAGAAUGGAACCAAGUUGGAAAACACUCCACAGGAUAUUAUGCAGGAU

>N1577

CUCGAGAAGAGCAACUCCAAGACACAUAAUUGUCAGAUUCAUCAAAGUUGC

>N1578

AAAAUGUUAAGGACAGCCAGACAGAAAGGUCGGGUUACCCACAAAGGGAAA

>N1579

AGUGAAGGAGAAAUAAAAUACAGACAAGCAAAUGCUGAGAGAUUUUGUCAA

>N1580

UAAAUGUAAAUGGGCUAAAUGCUCCAAUUAAAAGUCACAGACUGGCAAAUU

>N1581

UUGCAAUCCUAGUAUCUGAUAAAACAGGCUUUAAACCAACAAAGAUCAAAU

>N1582

AACCUAAUAGACAUCUACAGAAAUCACCCCAAAUCAACAGAAUAUACAUUG

>N1583

CACAAUUAAAAGAUCUAGAGAAGCAAGAGCAAACACAUUCAAAAGCUAGCU

>N1584

AAUAACUAAGAUCAGAGCAGAACUGAAGGAAAUAAAGACACAAAAAACCUA

>N1585

AUGAAUCCAGUGGCUGGUUUUUUGAAAAGAUCAACAAAAUUGAUAGACCGA

>N1586

AUAAACUAGAAAAUAUAGAAGAAAUAGACAACUUCCUCAACACAUACACCA

>N1587

AAACCAGGAAGAAGUUGAAUCUCUCAAUAGACCAAUAACAGGCUCUGAAAU

>N1588

AGAGGUACAAGGAGGAGCUGGUACCAUUCCUUCUGAAACUAUUCCAAUCAC

>N1589

GACACAACAAAAAAAAGAGAAUUUUAGACCAAUAUCCUUGAUGAACAUUGA

>N1590

AAGUGGGUUUCAUCACUGGGAUGCAAGGCUGGUUCAACCUAUGAAAAUCAC

>N1591

CCAGCAUAUAAACAGAACCAAAGACAAAGCCACAUGAUUAUCUCAAUAGAU

>N1592

GACGUAUCUCAAAAUAAUAAGAGCUAUCUAUGACAAAUCCACAGCCAAUAG

>N1593

CACUCCUAUUCAACAUAGUGUUGGAAGUUCUGGCCAGGGCAAUCAGGCAGC

>N1594

CAGACAAACAGAGGGUGAAAUCAUGAGUGAACUCCCAUUCACAAUUGCCUA

>N1595

UACUGGAACCAAAAUGGAGAUAUAGACCUAUGGAAGAGAACAGAGCCUUCG

>N1596

GAUCCCUUCCUUACACCUUAUAAAAAAUUAAUUCAAGAUGGAUUAAAGACG

>N1597

GGCAAGGACUUCAUGUCUAAAACACAAAAGCAAUGGCAAUGACAGCCAAAG

>N1598

GAUUAAGAAAAUGUGGCACAUAUACACCAUGGAAUACUAUGCAGCCAUAAG

>N1599

ACAUGGACACAGUAAGGGGAACAUCACACACCGGGGACUGUUGUACAUUGC

>N1600

GGGAUGUUUUGGGAAAUGGCUUAUUAUUUGCCCUUGUAGUUUCAUAUUCUG

>N1601

AAUACUAUGUCCAGAAUUUUUUGCUAAGGAUUUCUGCAUUUGAUGGCAUGA

>N1602

CAGGAUAGAGCUAGUAUAAUUGAAAAUCUCACACACAUUAUUAACACAUAG

>N1603

CAGCUCUUUAUCUGGGCACUAAGCAAAGUAUGUAAAAUUUGAAGCCAUAGA

>N1604

AGUUUGUGGUUUUUAACACCCCACAAUUUGUCCCCUUGAUUUCCAAACCCA

>N1605

GUUCCAAAGUCAAGUUUACAAUCACACAGCUAGUAGAGAAUUGCUGCUUUA

>N1606

UCCUAGAAGGCCCAACUCAUUAUAAACAGGAUCACUCUUCAGUAUUUACAU

>N1607

CAUCUGCACUACCUAUUACCAACUCACCAGUAGCUUUUAGAUUGUAUUUUA

>N1608

ACACACACAUAUCACACACACACAUAUACACAUAUACAUACACACAUGAAA

>N1609

UAACUGAAAAUAUCUCAUAUUUGGUAAAAAUUUUCAACCUACUUAUUCAAU

>N1610

AUAUUUCAAAAUGAAGGUAAGAUAAAGACAUUAAGAUUGAAAAAGAAGGAU

>N1611

UACAGUCACACUUAAAGAUAAGAAAAUUUUCCUUCCUUCCUUCCUUCCUUA

>N1612

GAGAAAUAUAAUGUUUACUAAUAAAAGCACCUAGAAAAUGGUGGGAAUAAA

>N1613

ACAGCUCCCACUGUGAGUGACACAGAAGAUGGGUGAUUUCUGCAUUUCCAU

>N1614

GGUAAACAAGGCAGCUGGGAAGCUCAAACUGUGUGGAGCCCACCACAGCUA

>N1615

UGCAGCUUGAGAUCUAAGAAUGGGCAGACAGCCUCCUCAAGUGGGUCCCUA

>N1616

AACCAUAAAGAUGGGGAAAAAACAGAGCAGAAAAACUGGAAACUCUAAAAA

>N1617

AGUUCCAACACAUGGCAAAAAAGUUAAAAACCUUGAAAAAAAAUUAGGUGA

>N1618

GGUGAUGGAAGAUGAAAUGAAUGAAAUGAAGUGAGAAGGGAAGUUUAGAGC

>N1619

AAAAAAAAUGAACAAAGCCCCCAAGAAGUAUGGGACUAUGUGAAAAGACCU

>N1620

UCAUCAAAGUUGAAAUGAAGGAAAAAAUGUUAAGGACAGCCAGACAGAAAU

>N1621

CCACAAAGGGAAGCUCAUCAGACUAACAGCUGAUCUCUCGGCAGAAACUCC

>N1622

GGCUAGGAAAAAACUGCAUCAACUAACGUGCAAAAUAACCAGCUAACAUCA

>N1623

AUCAAAUUCACACAUAACAAUAUUAACCUUAAAUGUAAAUGGGCUAAAUGG

>N1624

AGUCACAGACUGGCAAAUUGGAUAUAGAGUCAAGACCCAUCAGUGUGCUGA

>N1625

AAACCAACACAGAUCAAAAGUGACAAAGAAGAUCAUUACAUAAGGGUAAAU

>N1626

AAAGCAAGUCCUUAGUGACCUACAAAGAGACUUAGACUCCCACACAAUAAU

>N1627

UUCAACACCCCACUGUCAACACUAGACAGAUCAAUGAGACAGAAAGUUAAC

>N1628

AGGAAUUGAACUCAGUUCUGCACCAAGUGGACCUAAUAGACAUCUACAGAC

>N1629

UCUCAGACCACAGUACAAUCAAACUAGAACUCAGGAUUAAGAAUCUCACUC

>N1630

GGGACACAUUCAAAGCACUGUGUAGAGGGAAAUUUAUAGCACUAAAUGCCU

>N1631

AGAUCAAGAAAAUUGAUAGACCGCUAGCAAGACUAAUAAAGAAGAAAAGAA

>N1632

CCAGGACCGGAUGGAUUCACAGCGGAAUUCUACCAGAGGUAAAAGAAGGAU

>N1633

UCCUUCUGAAACUAUUCCAAUCAAUAGAAAAAGAGGGAAUCCUCCCUAACU

>N1634

CUGGUUCAGCAUAUGAAAAUCUAUAAACAUAAUCCAGCAUAUAAACAGAAG

>N1635

CUAUGACAAACCCACAGCCAACAUCAUACUGAGUGGGCAAACUGGAAGCAU

>N1636

CUGGCCAGGGCAAUCAGGCAGGAGAAGAAAAUAAAGGGUAUUCAAUUAGGU

>N1637

CCAAAAUCUCCUUAAGCUGAUAGGCAACUUCAGCAAAGUCUCAGGAUACAC

>N1638

UAUGUUGUUGGAAAUCCCUAAACUCAUGAACAUAUAUUAGUCAGCUGCUUU

>N1639

AGACUAAGUUUCAUUUCUGUUCAACAUAAGCAUUUACCAGAAAUGACCCAG

>N1640

UGUUGAUGUAGUCCUUGGAAAGAAAAGUCAGGAAGAUGUUGUUGUCAUCAA

>N1641

CCUUGAUAGAAUGUAAUGCACCAGGAGGUAAACACAAUAACUAUAAGGAGC

>N1642

AGAAUUGAAAAAUUCCUCUGAGCAAAGAUUCCCAGGAGUCAAAUUUAAUCA

>N1643

AGACAUCAUGCUGCUAUAAAGACACAUGCACACAUAUGUUUAUUGUGGCAA

>N1644

AGCAAAGACUUUGGAACCAACUCAAAUGUCCAACAAUGAUAGACUGAUUAU

>N1645

UAUCUUUUCACUCUUUUCUACUUCCAUGUGAUCGAAUCUUUCAACUCCCAC

>N1646

UAUUUCACUUAAGAUAAAGACCUCCAGGGAGAUCUGACAAGAUGGCCGAAU

>N1647

CUGGUCUGCAGCUCCCAGCAAGACCAAUGCAGAAGACCAGUGAUUUCUGCU

>N1648

GCCCAGACUAUUUAUUGGUGAUCAAACAAAGAAACAGGUGGUGAGAAUGUA

>N1649

GAGAUAAUGGAGAGAAGGUUCUUCUAACUCAAGAUACAAUCGAUCCUGGGU

>N1650

UUUUCCCAACACUCAGCUUUUCCCAACAUACCCCCCUUUUCUUUUUCAUAU

>N1651

GCUAUCAUUGCUUGUUCUUGAUGGCAGCUUUCUCUUCAGAGGCGGUUUCCA

>N1652

GAAUUAAUGCAGGUAAACAAGCAACAAUCAGGGCAUGUAAUAAAGUGUGUU

>N1653

AGUAACAUUUCCUAUUGCUAGCACGACUUUGAAUCCAAAAGGUCCUGCUGG

>N1654

UUGAUUGGACUUUGAGCCAUAGCUAAUUUCCAUAAUUCAGAAUGUUCUUGU

>N1655

UUUACCUCUAUCUCUAUCUCUAGUUAUGUCUAUCUCUGUCUCUAUUUAUCA

>N1656

CAAUGUUUUCAAUGAAUUUGUGGUUAUAGUUGAUGAUCACUGAUUUCACUC

>N1657

AAAUAUAGCCCAGAACAUUGUCUUAAAACUCUCUUCAACGUUGUGAAGGCC

>N1658

AAUGAAUAUGACCAUGAACCUGAAGAUGUCUUUGACUGCUCCUCCAAGUGU

>N1659

GGUCCAAAGCUUUCAUUUGCUGGCAAAAUACAAGCUAUCCCAGAGAAACUA

>N1660

ACAAUGCUGUGAAAAUCACUAACAUACCAAAAUCAAGCAUAUUCCACAACA

>N1661

AUUAAAUCUGUUUUUCUCCAGCAGAAGCAGGUUAACUGUGAAACUGAAGCC

>N1662

UCUGAAUAAACAAGCCUCAGAUUCCACUGGGUCUUACCCCAUGUCUCACCU

>N1663

GCCAAUGUGAGUAAUGGAGGCUGUGAAGGCCCUGAGCUCUGGAAGCCCAGC

>N1664

ACAUGAUUUACAGCUAUGACAGUUUAGCAUAUGCUCUGCUACUUGAGAUAC

>N1665

GUUCUUUUAACUCAAGAUACAAUCGAUCCUGGCAGAGCAAGGAGCAAGGAG

>N1666

GGUAGGCUUCCACCCUUUAGCACAGAGCUUGGUGUUCCAAAGGCCAAGAGG

>N1667

UCCCUUAACAGGGAUACCUUCUGAGAAAUGCAUUAUUAAGUGGUUUCAUUA

>N1668

UCAUAAAGCAUACAUACACAAAUGUACAUGGUACUGCCUACUACACACCGA

>N1669

UGAAUACUGCAGACAAUUGUAACACAGUGGUAAGUAUUUGUAUAUCUAAAC

>N1670

UACAUGUAGACUAUAUUAAAUUUAUAAAAGAUAUUUUCUUUUUUCAAUUUG

>N1671

UCUUGCAUUUGCUGAGGAGUGUUUUACUUCCAACUAUGUGGUCGAUUUUAC

>N1672

GCAUUGAUCCAUCUACCAUUAUGUAAUGCCCUUCUUUGUCUUUAUUUAUCU

>N1673

AAAGUCUGUUUCAUCAGAGAAUAGAAUCGCAACCCCUGCUUUUCUUUUUUU

>N1674

UUUUUUGCUUUUCAUUGGCUUGGGAAAUAUUCCUCCAUCCCUUUAUUUUGU

>N1675

UGUCUUUAUAGUAGAAUGAUUUAUAAUCCUUUGGAUAUAUACCCAGUAAUG

>N1676

UGAACUAAUUUAUACUCCCACCAACAGUGUAAAAGCAUUCUUAUUUCUUCU

>N1677

AGCAUCUGUUGUUUCCUGACUUUUUAAUGAUCACCAUUCUAACUGGUGUAC

>N1678

ACUCUGAUGAUAGUUUCUUUUGUUGAGUAGAAGCUCUUUAGUUUAAUUAGC

>N1679

AAACUAAGGCACAAACACACACAUUAGCUUAGGCCUACACAUGGUCAGGAA

>N1680

UUAAAAUAUAGUAAAUACAUAAACCAGUAACAUAGUCAUUUAUUAUUACAU

>N1681

UGUACUGUAAAUAAUUGUAUGUUCUAUAUGACUGGCAGCUUUUCACACCAA

>N1682

AGUCUCAUGAUAACCUUAUGAAGCCACUGUAGUAUAUGCAGCCCUUAAUUU

>N1683

CACGAUGCAGUGCAUGACUAUACCCAAUUAAAUGUAGUAAUUUUAAUAUUA

>N1684

UAUUAACCAUAGGUAGAAUAAAAUUACAUACAAAUAACAUACACAUAUGUA

>N1685

AUUUUACGUGAGUGACUGAGGAGACAUCCAGUGUAUUUCCAAUUAGUCUUC

>N1686

GAGGUUCAGAUGGAAAAGACAAGAGAUCAAAGAGGUUAGUAGGUUCAGAAU

>N1687

UAGCACACUGCAAAGAUUUCCUUAAAGGGCAAUUUUCUGAGUCAUUCUGUU

>N1688

CCGCCCUUUUUUUCCUAAUGUACUCAUAGAUAAAAAAUUUCAUCUACAUUC

>N1689

AUUGUGGCCACUGUAACUCUAAGUUAUCUUCCUGAGAUGCACUCAUUUCUC

>N1690

CAGGUUCUGGGUCCGUAAUUUUUAAACCUAAAAUGCAUUGGAGUUACAGAA

>N1691

CAGAUUUUGGUUAGACCUAGUCUGGAAGCUAGUGUACACUACACUGGACCC

>N1692

ACAAGCAAUUCAUGAAUUGGUAAACACACAAACCAAAAGAGGUUCAGCACA

>N1693

AAUGCAGAAGCAGAAUUUAAAAAUUAUUUGAUUGGUUGCUGUUAUGAAUUG

>N1694

CUGAGAUUAGGUUUUGUUUCUGUCUAAGAAAGACAGAAAUGACUCAAGUUA

>N1695

GACAUUUCUUGUCAUCCCUUUCCCCAUAUGGACCAUAUGACAGUCAGCCAU

>N1696

UCAGGUGAGAUUUGGGAGGUGGAUGAGAAAGAAGGAGAAUCAUUCUCUGGC

>N1697

GUCUCUAGUUCCCUGGAUGGAAAGUACAUCCUACUUUUAAUACCUAAAUGA

>N1698

UCCUAAUUGACUCCUCUGUAUCAGCAGAGGAACAUGAAAUGCUCUUUGGAU

>N1699

CACAAAUUUUGAAUAUUUUGAGACUAAACAUUGCCAAAGUCUUUGUAAGAU

>N1700

AAAAUUUUCUGUAACUGAAGUAAUAAUUUAUUGUACCUUCUCUCCAACUAC

>N1701

CCAUUCAAUUCUGCAUCAGCAUUAUACCUUCACUUCAAAAUUAAAACUUGU

>N1702

AAUAAAGAUAUUGUGUCCUGAUAAAAAAUUAUUCAAAUUGAAUCAAUCCAA

>N1703

AGUGUUAGCCUCCCAAAGUGCUGGAAUUACAAGCGUGAGCCACCAUGCCUC

>N1704

GCUGAGCUUCGUGAGAGGGUGUCACAUGGCUUUCUACUGCAUGCACAGAAA

>N1705

UCAUCAAGUCAAGAAAUCCUAAGUCAAACCCUCAUAAGUCAGGGAUGAUCA

>N1706

GUAAAAUAUUUUACACUGUUUCAAAAUUUAAAAGUAAUGACAACUGAAUAG

>N1707

UGCCUGUGGUCCCAGGUACACAGGAAACUCAGGUGGGGGAAUUGCUUGAGA

>N1708

UGUCUCAAAAAAAAAAAAGAUAAAAAUGUGUGAGAUACAGAUAAACAUGCC

>N1709

AAGGUCUUCUAACUUGAUGGACUGGAACUUGCAAUUCUCUUUGCUACUGUC

>N1710

CCCUGUGCUUGUACAGUUCAGGGUCAGCCAAGGAUUUGAAGGGAGAAAUGG

>N1711

CAUAUUUGAGGUAAAUAAAAACAACAUUUAAUGAAUGACAUGUUUUGAGUC

>N1712

UGCAAAUGAUUCUAACUUUUACUUCAGUAGCUCUGGAGUUUCAAGUCUGUA

>N1713

UGUGAAUGAGAACUGAAUGUAUGAUAAAUGUUGCAUGUUCAGUUCAAUGGG

>N1714

CUUAUUUAAUAAGUGAUUGUGGCACAAAGGCUGUCUUCAGCAAACACCAAG

>N1715

GAGCAUCUGUGUCUAGCUGUUGAGCAUCUCCAACAGUGGAUGAGUGUCCAU

>N1716

AAUAUAUUGGGGAGCAGUUUCCCCAAUGCCUCCUAUUAUCUUCCCAAAUGG

>N1717

CAGAAGGCAAAUAUCAUGAGGGGCCAUGAAACCCAUGGAAAAAGGGGAAGG

>N1718

CGGGGCUUGUUUGUUACAGCUCAAGAGUGAAUAAAAGGGCUGCUCCUCCUA

>N1719

AAACGUGACUGCAUGACUCCAAUGCAGUCAAAAGCUGUGGAAGUUACACAC

>N1720

GCAAUGCAAUGAGAUAGAAGGUCCAAUAGGAAGACAGCGUGCUGUUGGGGG

>N1721

AAGCACUCCAUGGGAUGGAAAGAGAAACAGAGGGCUGGACACAGGGGGGUG

>N1722

GUUGGUCUCUCGCACAUAGUCUUCCAGGGAGCCGCCAGCCAGGUCUUCCUU

>N1723

GGCGGAUAAGACGAUGGCUUGGCAGAUAGCGUCUGCCACCUCUGGCGAGAG

>N1724

GAAAGAAGAGCGAGACUUGGCCAAUAGAACACCAAGAAACCCAAUACCAAA

>N1725

CAGAGCACAUAAAGAAGUGACAAGUAAUAGCUGGUUUCCCAGUCUUGUCCC

>N1726

AUUGAUGUAUCUCAGAUGACAAAGCAUGAAGCUGGCAUGUGGCAGCAUGGC

>N1727

CCCACGUGCCAAUUCUAACUGAGUGACAUUGGGCCCCCUCCAGCUUCCCCA

>N1728

CCAGUGCAGAGCCAGAGGAGACAGGAACCCCCAUCUGAGCAGCCUGGAGCC

>N1729

ACUGGACUUUACUAAAGCCAAAUGCAUCACUUGAGCUCUGGAGAGGAUUCC

>N1730

AGAGCCCAGAUCUGAGGCAGCAGCCAAGUACAUUCUGCUUGUGCACAAGGC

>N1731

GGAAGAACAUCUACCAUGUGAAAGCAUUUCCUUUGGGACUGGCAUGGUGAA

>N1732

UCUUCUUCCUCCAUCUACAAAAAAAAAAAAAAAAAAAAAAUCAUAUGUUCU

>N1733

CUCCAUCAUAAGUUUAAGAGAUUUUACAGAUAAAGGUGCAACUGGCCUGGU

>N1734

GAAGGGAAGAAGGAGGAGGUGGAGGAAGGGAAGCACAGAAGCAGGUGUGAG

>N1735

UGGUCCAUGACUGUUUUUCCAGAUCAUGUAGGACAUUCACAACCAGAUGAA

>N1736

CCAGAUCCCAGAAGUAUAUAUGCGGAUAUUGCUUUAUGAUUGGUAAAGAUC

>N1737

UGGCUUCCUUACAUCCCUGGUGGAAAUUGAGGCAGCACUUUACCAAUGUAC

>N1738

CAUUGUCUCCAACACAGAAGAUGGAACAGAUAGUACCAUCCUUGUCAUAUG

>N1739

CAGGUGAACACACAAACACAUACACAAACACAAAUAUACAUACACAACACG

>N1740

AUGACACGCACAAAUACUCAGACUUACCUCUGCACCAGCUCACAGGCAAGU

>N1741

GCUGGGCUGGAGACCUGCCCUUGAAAGAGCAGUGUCCGCUGGAGAUGGAGA

>N1742

CUUCUAGAAGAUCUGCACUAUAAGAAAAGGGAAAAUACAAAAGAAUGUCUG

>N1743

UUAAAGAGAGCACUUGUUAUGGGGGAAGAACAAUAUUUUAAACACAGGCCA

>N1744

CUAAAGGGAAGCCAUUGAAGGAAGAAAUUCAGAACAUAGGAGCAUAGGUGG

>N1745

AUAGUAUGUGUGUGUGUACAUAUAUAUAUCUUUUAAUGUUCUACUUAAAUC

>N1746

UGCCUCAGCCUCCCAAAUAGCUGGAAUUACAGGCGUCCGCACACCCGGCUA

>N1747

UUUUUAAUACAGACGGGGUUUUACCAUGUUGGCCAGGUUGGUCUCAAACUA

>N1748

CCUAAGUGUUUAGAAAGUAUUUAGAAUGUUAUUACUCCACCCAUAAUUGAG

>N1749

UGUUACUGCAAGGUAAUCUGGCCUAAUCUAAUUGAUACAGCAUAUAAAUCU

>N1750

GCUUUACUCCAGAGAAGAUUUGUGUACAUUUUUAUCAGGAACUGACAAGGG

>N1751

CAAUCUACAACUCACUUUACAACUGAUUCCAAAUUUAGGCUCCAGAGAGUU

>N1752

UGUUAUCCCUCACUUCCUGCAAGCCAAAUAAUGUAGUCCAUUGUUUUGUUG

>N1753

UCUUUUCUAUUGUGAACAGUGCCACAAUAAACAUAGGUGUGCAUGUGUCUG

>N1754

AGCUCUAGAUCCUUGAGGAAUCGCCACACUGUCUUCCACAAUGGUUGAACU

>N1755

UGACCUUUUAAUGAUCACCAUUCUAACUGGCGUAAGAUGGUAUCUCAUUGC

>N1756

UGCAUUUCUCUAAUGAUCAGUGAUGAUGACCUUGUUUUCAUAUGUUUUUUU

>N1757

UAAAGUAGUUUUUUCUAGGUCGGUGAAGAAAGUCAAUGGUAGCUUAAUGGU

>N1758

GUUGUUCUCCCUGAAAAGGUCAUUCACAUCCCUUGUAACUUGUUUCCUAGA

>N1759

GUAUCCUUGUCUUGUGCCAGUUUUCAAAGAGAAUGCUUCCAGCUUUCGCCG

>N1760

UGGUAGUUUGUAUUUCUGUGGGAUCAGUGAUGAUAUCCCCUUUAUCAUUUG

>N1761

UAUUUCUGCCUUAAUUUCGUUAUUUACCCAGUAGUCAUUCAGGAGCAGGUU

>N1762

UAAUUAGGUGGUCAAUUUUAGAAUAAGUGCUAUGUGGUGCUGAGAAGAAUC

>N1763

AGAUUUGGGGUGGGGAGUUCUUUAGAUGUCUAUUAAGUCCUCUUUGUCCAU

>N1764

UUCUUGUUGCAUUGACCCCUUUACCAUUACGUAAUGCCCUUCUUUGUCUUC

>N1765

GUUGGUUUAAAGUCUGUUUUAUCAAAGACUAGGAUUGCAACCCCUGCUUUU

>N1766

UCGUUUGCUUGGUAAAUCUUUCUCCAUCCCUUUACUUUGAGCCUAUGUGUU

>N1767

AGUGCAUCCUUAAAUAGCUCUUGUAAGUCAGACCUGGUGGGAACAAAAUCU

>N1768

GCUUGUAGGGUUUCUGCAGAGAUCCACUUUUAGUCCAAUGGGCUUCCCUUG

>N1769

UGACCUUCCUCUCUGGCUGCCCUUAACAUUUUUUCCUUCAUUUUAACCUUC

>N1770

CAGCUAAUCAAUCCCUUUUUAAUUGAGUGUUUUUAUAUGAACAGUGCCUUU

>N1771

UCCCCUAAUAUUAAUUCAUAUUUCCAUUACCAGAAUGAUCUUACUUCAUCA

>N1772

AAGUUAAAUUAACUACAUUUUAUUUAAUGUGCAUUGUUUAUCUGGUUUGCU

>N1773

AGAAACCUGUAAUGAAAAAAAAAACAUAGGUUUUAAUUUCAUACAUGCCUU

>N1774

AUUUUACCCUGUAUGCAAGCUAACAAGUUAGUCUGCUGACAGUUUCCUGGG

>N1775

CAUGUAUGCCAAGGUGGGUUUAUAUAGGAGUAGACUUCCCCAAUAACCUCU

>N1776

AAUAUCUGCGAUGUAGAUGGAUUCUACUUAAAUGUGGUGCUUCAUUCAUAA

>N1777

CAUCAUUUUAUUGUGGCCCUACUAAAGUAUAGUCAAACUGAAGUCUUUAUA

>N1778

ACUUGUUCAGUUGUUUCUGGCAAGCAUUGGCCCUGAAAGUUACUGUGCAGG

>N1779

UCUUCUGGGUCUAGUCACAAUCUGUAAACCAUGGUAAGAGUCCUGCAGAGA

>N1780

CCCCUAGAGACACUAUCAGACUCCCAUCAUAUCUCUGUUCCCCACAAUGGU

>N1781

GGGUCUAAUGGGAGGUGUUGCAGUCAUGGGCACAGAUCCCUCAUGAGUGGG

>N1782

GAACUGGGACAGGGUGCAGUGGUUCAAUGCCUGUAAUCCCACCACUUUGGA

>N1783

GAGGCUGAGGCAGGAGGAUCACUUGAGCCCAGGAAGUGGAGGUUGAAGUGG

>N1784

ACAGCCUGAAGAACUGUGAGUGAAAAUAAAUCUCUUUGCUUUAUGUCAGUU

>N1785

UUCUACAUCAGUACAAAAUGGACUAAUACACUGACCUUGGCAUGAAGCCGC

>N1786

AUAUGCUGUGGAUUUAUUACUUACAAAUGGGCAGAAGGGACAAUGGAAGCU

>N1787

UGGGGUAACCUGAAUCACUGGAAUAAAGCAUUGAAGGACAUUCUGAUUCUC

>N1788

ACCUGUCACCAUGGCUAUUCCAUGCACUUGCCCAUUGUGCCAGCAUCGACC

>N1789

GUGAACAUUCUCUGGUAGGAGUAAAACGAGAUCUUCACACUUUCUGUGCCG

>N1790

AAAAUGUAAAUCAUUCAAAACAUUGAUCAUUGAGAGGAUUUUCCUGCACCC

>N1791

CAGUGGUCUUAGGUGUGACUGAGGCAGAGGCACUGCUACAAGAGUGAUGGC

>N1792

UCUGAAGGAGUUCUUCUGGCAUGACAAAGUCCACUGGGAACCUAAAAGUUU

>N1793

UACCAAUUAUGCUUAUUUUUAAACAAAUGUUUUUACUUGUUGCCCUAGAGA

>N1794

GUAUCUUAUAACAGGAAUAUUCCUAAUUCCUCUCUCCUAUUUAUUGUCACG

>N1795

UCAUUUCAUUAAUCUGCAUGCUAUGAUUGCACAACGCUUAGUUACUAUUAC

>N1796

UUCUUCUCUCUGAAGAACUUCUUGUAAUUUUUUUUCAGGGCCCAUUGGCUU

>N1797

UCCUUCAGUUUUUGUUUGUUUUGAGAAAUUCUUUAUUUCUCCUUUACUCUU

>N1798

CUUUUGUACAGUUUGAAUAUUAUAUACCUAUGUGUAUAUUUUUUGGUAUUG

>N1799

CCUCCUUUUGGUAUUUCAAUUAUGCAUGUUAUGUCUUUUGAAACUAUCACU

>N1800

AUAUUCUGGUCUGUCUUUUUCUUUCAUUUUUUGGGUUAUAUUUCAGUUUGG

>N1801

UUCAAAGGCAUUCUUUAUUUCUUUAACGGUGUUUUUGGUUUUUCAUCCUUC

>N1802

UUAAAGUUCCCAUCUCUCUGCUGACAUUACCCAUCUUUUCUUGAAUGUUGC

>N1803

AAUGACAACCUUUAACAUGUUAAUGAAAUUAUUUUAAAUUCUGCAUCUGAU

>N1804

CUUAUGAUGCAGUUACAUCCCAAUAAACCCAUCAUAAGUCAAAAAUAUCAA

>N1805

GUGGUGGUAAGAAGCGAGGCAAGGAAAGCAUUCUAUACAUUUAGUAUUAAU

>N1806

AAUAUCUUGGUAAAUAUUUUAUGUGAAGGUGGAGUCCUGAGAACGUGGGUA

>N1807

AGUUCAACCAGUAUUUGAUUUUUAAAGAUAAUUCCUUAUAAUACAAAAUAA

>N1808

CUACCUCAACAUCAAAUCCAGCAUCAUGCUUAGUCGGGAAUUUGACAACCU

>N1809

UGAAUGCUACAACGAUAAAACAGAAAGUAAUUGCAUGCAUAAAAAUUGUAG

>N1810

CUACUUAAAAAUAGGAGAAUUUGGUAAGUUGGUAGGUGCAAACUAAAUACA

>N1811

UGUCUCCACUGAAAUCUCAUGUUGAAUUGUAAUCCCCAGUGUUGGAGGAGG

>N1812

CCCCCUCAGAAGCAGAAGCCAGUAUAGCCCACAGAACCAUGAGCCAAUUAC

>N1813

ACAUUUUUAAAAAUAACAAAAAGAUAAAAUACCUAGGACAAACUUCUAAAC

>N1814

CUGUGUAAAGAAAACUUUAAGAAACAUCUGAGGGGCUGGGCAUAGUGGCUU

>N1815

AAGACCAGCCUGGCCAACAUGGUGAAAUCCCCCUUCUACUAAAAAUACAAC

>N1816

AACCUAGGAGGCAGAGGUUGUAGUGAGCCAAGAUCGCACCCCUGCACUCCG

>N1817

CAGAAUGAGACUGUCUCAAAAAAAAAUUAUCUGGUAGUAUCUAUGAAAGCA

>N1818

AAAAGAUAAUGCCUAUAAUAUUUAUAGCAGUGUACUCAUAAAAAAACUGAC

>N1819

AGCAAACAAAUAUGACAAUUCCCUAAGCUAUAAACUUAUAUGAGCCUUUUC

>N1820

GUUAUCAUAAAAAAGUCAGUAAAUUAUUUCUACAUUACCUGUAAAUUUAAU

>N1821

GUGUACCCACCCAAAUCUCUCGCACACUCACCCUUCAACUUCCCCUCAUUU

>N1822

UAAGAUGGCCCUGCACAGCUACCCAAAAUUGGGGCCAGAGGCCAGGCCUCC

>N1823

GCUGUCAGCUCUCAACACUCCCGUCAAGUCAGCAGGGAGACUCGGGACUUA

>N1824

GGUGAGGCUGGCUGGGAAGGAGAGCAGGGAGCUGUGUGUGAGUGGGGGUCA

>N1825

AGAUUAGCUGCUCUAAAAAACGACUAUAAUUUGUAAUUCCAGCUGAACCCA

>N1826

GCAUCUUGAGUGGCUGCUGUGCUCUAGGCAAUGUGUAAGACUUUUAGAAAG

>N1827

CAUGUCUGAAGCACACGCUUGUUUCACUUCUCAGCCCUCCUCUUAGCUGGU

>N1828

UUCAUUCUUUUUAUGGCUGCAUAGUAUUCCAUCAUACACACACACGCGCGA

>N1829

UUUUGCUACUGUGAAUUGGGCUAUUAUAAACAUGCAUGUGCAAGUAUCUUA

>N1830

AUCUUCUUUUGAGAAUUGUCUGUUCAUGUCCUUAGCGCACUUUUAGAUGGU

>N1831

UUACCGAUUUGUUUGAGUUUGUUGUAGAUUCUUAAUAUUAGUCCUUUGACC

>N1832

UUUUCUCCCACUCUGUGGGUUGUCUAUUUACUCUGCUUACUGUUCCUUUUA

>N1833

UUUGUAGUUUCAGGUCUUAGGCUUAAGUUCUUACUCCAUCUUGAGUUGAUU

>N1834

AGAAUUGUUUUUUCUAAUCUUGUGAAGAAUGAUGGUGGUAUUUUGAUGGGU

>N1835

UUCUCUGCUUGGUUGCUGUUGGUGUAUAGAAGAGCUACUGAUUUAUGUACA

>N1836

AUCCAGAACUUUGCUGAAUUCUUUUAUCAGUUCUAGGAGAUUUCUGGAGGU

>N1837

UUGAAGAGGAGCAGUGAGAGUGGGCAUCCUUGUCUGGUUCUAGUUCUCAGG

>N1838

AUUAAGGUAUGGUCCUUUUAUGCUGAUUUUGAUGAGAGUUUUAAUCAUAAC

>N1839

AGGAUUUUAGCAUCAAUGUUCAUCAAGGAUAUCAGUCUGUAGUUUUCUUUA

>N1840

UUCUUCCUCUAUCUUGUAGAACAGCAUCAAAAGGAUUGGUACCAAUUCUUC

>N1841

AAACCCCGUCUCUACUAAAAAUACAAAAAAAUUAGCUGGACGCCAUGGCGG

>N1842

UCCCAGCUACUUGGGAGGCUGAAGCAGGAGAAUCGCUUGAACCUGGGAGGA

>N1843

AGCUCACCUUCUGAGCAAAGUGUGGAUCAUUUCCUUUGCCCUCCCCUGACC

>N1844

AUGAUGUCAAUGAAAAUGUACAAUCAUGGUUUGAGAGUAACCAAGGACAGC

>N1845

AUCAAAUCUUCAUAACAGUCAAUUCAAAGAAGUCCAGCUUCUGCUCUCAUA

>N1846

UAUAUAACAUAUAAAUAUAAUAAAUAUAUAUGCAAAAGUGUAUCUUGUGCA

>N1847

AAGAAGUACUUCAUUGCAUAGAUGUAUCUAAGUUCAUCCCGCCAGUCUCUG

>N1848

UCAAAGGGUAAAUCCUUCUUUAUUUACCCCCAUAGGGCUGAACUCUUUUGG

>N1849

UUAAAAAGCAGUAAGUUCUAUUCUUAUUUGUAACUUGUUUGUGCAACAUUU

>N1850

AACAAAGAAUUGAACAAAAUGCACAAAGUAACAAAAGAAUGAAGCAAUGAG

>N1851

UUGGCAACACCUCUAGGUGCCAUUUAUUUAUUUAUGACUGAGUCUUGUCUC

>N1852

CCUUAAUUCCCUAUUCUCCUGUCUCAUUUCCUGCUGAGAGACAUGAUCCCA

>N1853

AUGGGAGGCAGAGGCACUGAAGAUCAGCUUUCUGUAGCUGCUUCCUGCUGU

>N1854

GCUGCAAUGAAACUUGUACUUGGUCAUAAAAGGUGCAGCUAGCUUGAUCCG

>N1855

UUGAGUAUCUUUGGCUCGAUUUUGGAUUUUGUUCCAAAACUGACUGAAACG

>N1856

UUUACUUAUCUUUUGGCUUUCUUUUAAACAGGUACUUCAGGUCUUCCACAA

>N1857

CUGGAAAGAUGUAUCCAACAAUCUGAUCCUAGUGCUUUGACUGUGGAAGGC

>N1858

CAUAUAAUAUUUCAUAUGGGCUCAUAUUAAUUUUUGCUCUUGGGGAAUUAC

>N1859

AAUGUAAAUAGUAUUUGAUUCCAAGAGCCUUAGUUAUGUGAGAGUUAUUUG

>N1860

AUAACCCUUGAGUUAUUUGGGAGACAAAAGAAGGCUGUGAUCACUUUGGAA

>N1861

AAUUCUCCAUUGGGCCGUUUGAGAGAGAUACAUUUUUGAUCCCAUACACCU

>N1862

UUUAUUAAGCAAAAGGAAAACUCCCAGCAAAGAGAGUGGUCCUGAGAGCAA

>N1863

UUCCAGUGCACAUGUGGGCCCUUAGACUGAGUGACUCCAAAUUGAUUUAUC

>N1864

CACAUUUAGAAUCCAUAUAGAUGUUAAGCUUUUUCCCUUUUCCCAAUAUUG

>N1865

CAUGAAAGUAUUUCUUCUUAUGGGAACAAUAGUGCCAUUAAUUUCCUUGAG

>N1866

UGGAAUGGAGUAGGGUCAUUGGUCUAUUUACCAACGUCACUGACUUUGACA

>N1867

UGAGUUGAAAUGAGAUCAAAUGGGAAGGGCAGUGAGGGAAGGGCCGACAGC

>N1868

UUUUUAUGUCGGACUCCGUGGCCUCAGUUUCCUUUGUGAGUUUAUACUCAC

>N1869

CGGUCUAGCCAAGACUCAUUCCUGGACACAUGGUCACUUGGGCCAGCUCUA

>N1870

AGCACAAAAAGACAAUGAAAGAUGAAUGGGAUAUUCAAGACAAUUAUCUCA

>N1871

CAGGAGAUCGAGACCAUCCUGGCUAACACGGUGGAACCCCAUCUCUACUAU

>N1872

UGUGUUACAAUUUUUUUGAAUGGAAAUUGCUUCUCUGGGGUUUGGAAUCCG

>N1873

UGUUGCACAUGUGGCGAUAAUAAUGAUCAACAGUGUCAUCAUCACCUUUAG

>N1874

CUCUAACAUCCCUAUGAUAUCGUCUAAAUAGAUAUUAAAAUCUGCUUUUAC

>N1875

AACUGAGCAUGACAGGAUGCACUGAAGCCAUGCAGUUAGUUAUGAAGCCAA

>N1876

UGAAGGGAGUUUGUAAAAUAAUUAUAUAUUAUUAUACAUUAUAUAGAAAAA

>N1877

UCAGUUGAUAUGCAAAGCAGAAAACAGACAAAUGGGUACCUGUUUCAGAAA

>N1878

GAGAAGACUAGUGUUUGAAAAGGGAACUUAGAAAGCUUGAUGACUUUAGCA

>N1879

ACAAUUUCAACCAUGAGUAAAACAGAAGUUAAAGUUCACGCAGGAGAGUGG

>N1880

UCUGCAAGAUUGGGACCACAUAAAAACUUCCAUGUGGUUUUUGAGAGCAAA

>N1881

UCCAGGCAACAGAGACAGUACAGCCAUCUCUCCCACCCUCUCCCCAAGGAC

>N1882

AUAAAAUUAUUUAAAUGAUUAAAUAAUAGAAAGAAUAAUGCAAAGACAUGA

>N1883

AGCAGAAUAAACACAAAAAAUUAUUAAGUAAGUGAAUUUUGUUACAUACCC

>N1884

AGAAAAAUAACACAUAUUGCACUGCAUAAGUUUAUAAAACUGGUGCAACAC

>N1885

AGACUGGGAGAGAACCUAUGUCCACAUGUGAAACUGUAAAAGCUAUCUGAG

>N1886

UUGCAUGUAGAUUGUAAAUUCACACAAACCAAAAUUCAGAUACUUUCUAAA

>N1887

GAAUACCAUGCAGGCAUAACAAGGAACAAGUUCAUGUCCUUUGCAGGAAUG

>N1888

UGGAGGCCAUUAUCAUCAUCAAACUAACACAGGAACAGAAAACCAAACACC

>N1889

AUUUAUAAAUGGGAGCUGAACAAUGAGAACACAUGGACACAAGGAGGGGAC

>N1890

UGGGGCCUGUUGGUUGGGCAGUGGGAGAGAGAACACCAGGAUAAAUAGCUC

>N1891

GGCUUAAUACCUAAGUGAUGGGCUGAUAGGUGCUGCAAACCACCACGGCAG

>N1892

AGAAUGUAGGUUAUCAUUGCUUCCAAAUAGGCAGCAAAUCUAAAACUAAAA

>N1893

UUUAGGCUUUCAGAACUCAGUUGUCAGAUUCCUGCAUGGCAGGUUAGGCCG

>N1894

CUUCAACUAAGUGGUCAUCUUGCCUACUCCUGAGGAAGUCAGAAAUGGUCG

>N1895

GAGACAUUGUCCAAACCUAAGUGCAAAGCCCUUGGGAUGGAGGCGAUCCUG

>N1896

UUGAUGUGGGAAGAGAACUUAAGUCACCUCCCGCAAUCCUCACUUUAACCG

>N1897

ACAAUGCUGGGGGAGGCGGGCGGAGAAAGUCCCUACGUAAUUUAAUGCAGG

>N1898

UCCCAUAGAGAUGCUAAACUCAGAAAGGAUAAGACCUAAAAGUUGGAGAAU

>N1899

UGCAAGAAAGAGAGAGGAAAGAGGGAAGAGCAGUGAGACAGAAAGAGGCAA

>N1900

UAAGCGGUAGCUGUAUCUGCUCUAUAAGGGGUACCCAUUUACUUUUUCUUG

>N1901

GCUUGUUUUUACCUUAAAGCCAUUCACACUAUUUCCUUCCACCUGUUUAUU

>N1902

CCAUUUCUCCAUGUGUGCUGGUUUCAUUUCCUCAGCAACAUGGGAAGACAU

>N1903

AUGGGCUCCAUCCUUAACUCAUGGAAUCAGAAACUCUGGGGGCGGAGUCCU

>N1904

CUCAUCUAAGAUCAUCCUAGAAGAAACAAAAUGGAGAGGUGCUGUGGGCCU

>N1905

AUGUUGCCUAGCCAUUUGUAUUUUAAUAAACUCUUUAGGUGAUUCUGAUGG

>N1906

AAAUAGAGCAGUCUCUGCUAUUUUAAUGGAUGAUAUUCAAGCCUGGGAUCC

>N1907

AAACCUAUAGAGGUCAUCUUUUUACAAGAUGUGGCCAAGAUAACAGGCAAU

>N1908

AUUAUGUAAAGCCUGAUGAGGGAGAAGGUUUUGAAUUCAUAAUUCACACAG

>N1909

UGAUUUUUCUAAGAAGUCUUUUCCAACUACUCCUACUAUUAGUUUAGCAGU

>N1910

AGAGGGAGGUUUUAUAGGAUGGCACAGAAGAAAAUAAAAUAUUUGAUUGGA

>N1911

AUGGUAAACAUUGAGAUAUUGCCACAAAAGUUAAGAACAAGCUAAGGAAGA

>N1912

UUAGAAGUUGUUUUAUAAAAAAUUCAAAAAAAGAAGAUAAAUUAUCAUUUA

>N1913

AAGAACUUUUACAAAUCAAUAUCAAAAUUAUUCCCAUCAGAAUUGAAACAA

>N1914

UAUGUAAGAGAUAUCAAUAAAAAAUAUAGUUCUUUCUUUAAUAAAUAGACA

>N1915

GACUCAAUCAUAUUCCCAUUGAGUAAUGUAUAUGAAAAUCACACUAACAUU

>N1916

CUGUUGCUGCAUAAGAAACUACUCCAAAACUCAGUGGCUUAAAACAACGUU

>N1917

CAGUUGGAGUGGCUCAAAUGGCUCUAGAUGGGGUGGGGGACAGAUCAAUGU

>N1918

UUUUACUUUCCCAGGUUUAUCAUAGAAUUGUAAAGUCAACAGAACUUAAAG

>N1919

GGCCUGCUGAGUCUUUUACAAUGCCACCCCAUUACAUCUCACAGUAGAGGU

>N1920

GCCAGUGAUUUAACACUACACUCAUAUCUGUCUGCAGUAAGCAAUGAUAUU

>N1921

AUUGCCUAAAUGAGGUUUAGAGGAGAGGAGAGGGAGACCGGAGUCCUCACA

>N1922

GGAGAUGACUGUGGCCUGUUGUCUGAAAUCAUAGUCAAAGCAAUGUGAUGA

>N1923

AACACUGUGCUUGAGAUUGCAUCAUAUAGUGGAAUGCUUCUGUUGAUGACU

>N1924

AUUUACACUUGCACCAACAGUGUAAAAGCGUUCCUAUUUCUCCACAUCCUA

>N1925

GUUGUUUCCUGACUUUUUAAUGAUCAUCAUUCUAACUGGCGUGAGAUGGCU

>N1926

UGUUUUUUUCUUGUAAAUUUGUUUAAGUUCUUUGUAGAUUCUGGGUAUUAU

>N1927

GAUGGAUAGAUUGCAAAAAUUUUUAAAUUCAAAAAUGUACUCAAUUAAUCA

>N1928

UCUGCAAGAUUGACUCCUUCUUGCAACUCUGAGCAUUGGUUUGAACUGAAU

>N1929

UCAGACCUCUUCAAAAAGAUCCAAAAAAAAAAAAGAUCCAAGAAGGGUUUU

>N1930

AUCAGCAAUAAAUUGUCCCUUUCUCAUAGAAGAACUUUCACUUAAUAAUGA

>N1931

UUUUUUUUUUACGAAAUUUUCCUGGACUAAAUUCUUUUUCUUUUCGUUUUU

>N1932

UAUGCCAUGUUGGUGUGCUGCACCCAUUAACUCAUCAUUUAGCAUUAAGUA

>N1933

UGUGUCCAUGUGUUCUCAUUCUUCAAUUCCCACCUCUAAGUGAGAACAUGC

>N1934

UUUUUGUCCUUGCAAUAGUUUGCUGAGAAUGAUGGUUUCCAGCUUCAUCCU

>N1935

UGUAAAAGGGUUCCUAUUUCUCCACAUCCUCUCCAGCACCUGUUGUUUCCG

>N1936

UGAUCGCCAUUGUAACUGGUGUGAGAUGGUAUCUCAUUGUGGUUUUGAUUA

>N1937

AUUUUUUAUAAGGUGUAAGGAAGGGAUCCAGUUUCAGCUUUCUACAUAUGA

>N1938

CUUCUAUUUGUGUUUUCACUGGGUAAUGAGGUUCUCACGGGCACCACCUUU

>N1939

GCUUGCUUCUCUCAAAUUACCUUUCACACUGCAACCAUAAUGCUAUAUAUG

>N1940

AAAAUAGAGAACACUGUCAAAAAAUAGUCAGAAUCAACUCUUUGUUUUUUA

>N1941

UACCCCCACUCUGCAGUAACCUUGAAAACCAAAAAACUAAAAUCACUAUGU

>N1942

CCUGGAAGCCACUGAAGGCAGCUGAAUGGAGUUAGAGCCCCUUAAAAGUCG

>N1943

GGCAUGAGACCGAUAUAAUGGACAGAGGAACUGGGAGGGGGGAAGAUUGGA

>N1944

CUCAGAGAACACCACUACAUAACUAAUCCAUGUAACCAAAAACCACCUGUU

>N1945

UAUUGAAAUAAAAAUAAAAAUUUAAAAAGGAACAAGGUAAUGAAAAAGAAC

>N1946

GGAACUUUUACUGAAGAUUCUGCAGAGAACAUGCAGAGUCAGCAUGCAGCA

>N1947

GCUUGUUAUUUGCCCUUGUAGUUUCAUAUUCUCUGCGGCCCCUCCUCAUGG

>N1948

AUGGAAAAUCUCGCACCCAUUAUUAACACAUAACCUGGGGGCACAUGUAGA

>N1949

GUAAUAUGUAUGAAGGCUAUAGUAGACUGUAUUACAAGAGCCAGUUCCAAG

>N1950

AUUAUAAAUGGGAUCACUCUUCAGUAUUUACAUUAUAGCAUUUUAUUUGUC

>N1951

AACAUGUUUGUUAUUGACAGUAGAAAACUAUGGUUUUGUCAACAUCUGCAA

>N1952

AACCAGGAAACAGGCACCAUCUUGGAAGCAAGGAGUAAGCCUUUACCAGGA

>N1953

AAGGUUGAAACAGACAUUAAGGUUGAAAAAGAAGGAGUUGCUGUGAAUACU

>N1954

GGGUCCCUGACCCCCGAGUAGCCUAACUGGGAGGCAUACCCCAGGAGGGGU

>N1955

CUUACACGGCCGGGUACUCCUCUGCAACAAAACAUCCAGAGGAACGAUCAU

>N1956

AAAGACCAAAGGUAGAUAAAACCACAAAGAUGGGGAAAAAAACAGAGCAGC

>N1957

AAUUAGAUGAAUGGCUAACUAGAAUAACCAAUGCAGAGAAAUCCUUAAAAA

>N1958

AGUUUAGAGAAAAAAGAAUAAAAAGAAAUGAACAAAGCCUCCAAGAAAUAA

>N1959

CAUUCAAAUUCAGGAAAUACAGAGAAUGCCACAAAGAUACCCCUCAAAAAA

>N1960

AGACACAUAAUUGUCAGGUUCACCAAAGUUGAAAUGAAGGAAAAAAUGUUA

>N1961

GAGAGAAAGAUCCGGUUACCCACAAAGGGAGGCCCAUCAGACUAACAGUUA

>N1962

UUAUUCACCGUGAAAUUAUUUAUUUAUUUAUUUAUUUAUUUUUUAUUAAAU

>N1963

UCAACCCAGAAGUUCAUAUCCAGCCAAAAUAAGCUUCAUAAGUGAAGGAGU

>N1964

AAGGAAGCCUGAAACAUGGAAAGGAACAACCAGUACCAACCACUGCAAAAG

>N1965

UGUAAAGACCAUCAAGGCUAGGAAGAAACUGCAUCAACUAACGAGCAAAAU

>N1966

GAGGAAGAUCUACCAAGCAAAUGGAAAACAAAAAAAGGCAGGGGUUGCAAG

>N1967

GAUAAAACAGACUUUAAGCCAACAAAGAUCAAAAGAGACAAAGAAGGCUAU

>N1968

CACUCAAAACUGCUCAAAUACAUGGAAACUGAACAACCUGCUCCUGAAUGU

>N1969

CAUAACAAAAUGAAGGCAGAAAUAAAGAUGUUCUUUGAAACCAAUGAGAAA

>N1970

UAGCAGAACUGAAGGAAAUAGAGACACAAAAAACCCUUCAAAAAAAUCAAA

>N1971

GCUGGUUUUUUGAAAAGAUCAAGAAAAUGAUAGACCACUAGCAAGACUAAA

>N1972

CAGAAACACAAACUACCAUCAGAGAAUACUACAAACACCUCUACGCAAAUA

>N1973

CAACCAAAAAAAAGUCCAGGACCAGAUGGAUUCACAGCCGAAUUCUACCAC

>N1974

AGGAGCUGGUACCAUUCCUUCUGAAACUAUUUCAAUCAAUAGAAAAAGAAG

>N1975

UAACUCAUUUUUUGAGGCCAGCAUCAUCCUGAUGCCAAAGCCUGGCAGAGC

>N1976

CAAACCGAAUCCAGCAGCACAUCAAAAAGCUUAUCCACCAUGAUCAAGUGG

>N1977

AAUAAGAGCUACCUAUGACAAACCCACAGCCAAUAUCAUACUGAAUGGGCU

>N1978

UGGUGUUAGAAGUUCUGGUCAGGGCAAUCAGGCAGGAGAAGGAAAUAAAGA

>N1979

AAAUCAUGAGUGAACUCCCAUUCACAAUUGCUUCAAAGAGAAUAAAAUACA

>N1980

AAAGAGGAUACAAACAAAUGGAAGAACAAUCCAUGUUCAUGGGUAGGAAGA

>N1981

UGAAAAUGUCCAUACUGCCCAAGGUAAUUUAUAGAUUCAAUGCCAUCCCCA

>N1982

GCCAAGUCAAUCCUAAGCCAAAAGAACAAAGCUGGAGGCACCACGCUACCC

>N1983

UAUACUACAAGGCUGCAGUAACCAAAACAGCAUGGUACUGGUACCAAAACC

>N1984

AAGCCUCCCCUCCCCAUGUUUUGGAAUUUUGGGAUGUUAAAUCCACUUGUC

>N1985

AUUGGCCACCUUGCUAGUGCUCAACAGCCACAUGUGCUAGUGGCUGCCAUC

>N1986

CAGACAGUAAACAUGUCCAUCACAGAAUUCUCUAUUGAACAGUACUGCUUG

>N1987

AAUUUUAAAAUGUCAUAGUACUCUAAAACAUGCACCUCUACUCAGCCCCUG

>N1988

CAGAUGAUCUAAAAGAUGAAGCAGGAGAAGCAAUAUUUGCAUCUCCAAACA

>N1989

CUGUUGAGCCGUCAGACCUGUCCAGAAAUUAGCUUAUGCUGAAGAGAGAUG

>N1990

UCCUUCAUUCUGAAAGUCCAACCAAACUUUAGAACUCCCUGUAGAGUCUAC

>N1991

CAUUUCCUGAGAAGAUUCCCUAAUAAACUUCCUUCAUCCAUUUUAUCUUCA

>N1992

ACGCAGUGAUGUAAUUUGCAAUAUUAGUAGGUCAAAAGGAAAAAGACAUGA

>N1993

GUCAACGUCAAAAUAAAAUUUUCUAAAUUUAUACCACAGAUAAAAGACUUA

>N1994

GACUAAGCAAAGUAUAGCCACACUGAUUUUAAAAUAAACUAAUUAAUUUCG

>N1995

ACUCACUUUUGAAAAGGUAGCAAGCAAUUGUAAUUAUACAAGAGAAAGCAA

>N1996

UCAGGCAUCUUGAACUAGAAAUACCAUUUGACCCAGCCAACACUCCAGAGC

>N1997

UGAUCCUUACAAUCAGACUCAGACAAAAAUGUCCCCUCUCAGGUUCCCCUA

>N1998

UCUUCCCCUGGGCAGUGCCUAUCAGAGGCUCCUCUUCUCUCCCAGUGGCAU

>N1999

ACAAUCCCUGCUGCUGGUGUCAAUGAGGUUUAAGCCCUUCUCAGGGGGCCA

>N2000

GACAAACUUCUAACUGUUGGGACAUAUCCCAGCUGGGAAGGACUGAAAUCA

>N2001

UCAUUAGCAGUCAGGAAGCUCAGGAAGAUUCAAGAGAGGAAGAAGACCGAU

>N2002

UUUCUAGGUCCCAAGCCAUGCACAAACAUUUUCAUUUUUCUUAGAACAAAA

>N2003

CGCUUGGUCUAUGCCUGCCAGAUUUAAGCAAGAGAAACUAGCUGAUGAAAA

>N2004

UUUCUCACUCCAUAGUAGUUCCUACAUUGCAAGGGCUUCAUAACCAGGGGA

>N2005

UGAGAAAAAUUCAAGCCAUUAAUGGAUCAAGUGAAGUGUAUUUCCAGCUUA

>N2006

GAUGGGCAUGGUGGUGCACACCCAUAAUCCCAGCUACUCGGGAGGCUGAGA

>N2007

GGUGACAGAGUGAAACUGUGUCUCAAAAAAAAAAAAAAAAAAAAGAAAGAG

>N2008

AAGAAAAGAAAUAUAAGAAUUUUUCAUGAUUUAGAACUGAAAAAUAAGUUA

>N2009

UGUUUCUCAGGAACACCAAUGGACCAAAAUGAACACAGUCACUGGGGUAAC

>N2010

ACUGCAACCUCCACCUCCCAGGUUCAAGUGAUUCUCCUGCCUCAGCCUCCC

>N2011

CUCCCAAAGUGCUGGAAUUACAGGCAUGAACCACCAAGUCUGGCUCGAAAC

>N2012

UCUGCCCCUCGACAGCCCUGUCUUCAGUCUCUUACAUCAAGAAGAGCCCAC

>N2013

UGUGUAUGGAACUUGUUUAUUGAUCAAGCUGAACUCUGAGAUCCAAAUGAU

>N2014

AACUUUGGUGACAUGCCCAAUAUUUAUGAGUGGAUGAUUUAGAGACCCUCC

>N2015

ACAGAAAAUAAGAUUAAAUGUCCAUAAAUUUUGGCCAAUUUGUCUGGAAGU

>N2016

AAAUAUGUGCAACAAGUGUUGGCGUAGCAGCAUGGCAAAGCACUAAUACUU

>N2017

GGCAGUCCUGUGAGCCCGAGAGGUCAUUUGAACCAGCUAAGAACAAGACCU

>N2018

AACCACUUGGAACAGAUCCUGGCACAUUAAAAGUGCUCAAUAAAGGGUAGA

>N2019

AGGUUGCUAUAAACAAUCCUAGCCUAAUUUUCACCAAGUGCUUAAGUGCAG

>N2020

UUAAGUUCUUUACAUAUAUUAACUUAUUUAGUCAUUAGAACACCACCACAU

>N2021

AAAGACAAUCUAAACUAAGCAAUCCAGCUCCAGAUUCUUUUUUUCUUCACG

>N2022

UGGUCAAUGUAGACUUUCCUUCUCCAACUUUCUCUCUGCAACAUGAGAGUG

>N2023

AGUCAUUCUUAUCACUGCCAUGUGGACCAGCGAAAUGGCACCAUGGCUCCA

>N2024

AUGGAACCCAUGGCCUUGCCUACGCAUCUCUGAUCUCCAGAAAACUUUUCG

>N2025

CCCAAGUAUGUUUUCCUAGGCUCCAACAUGGCAAUACCCUGCAGUCCUACC

>N2026

UGUCUCUACUAAAACUACCAAAAAAAAAAAAAAUUAGCGGGAUGCGGUGGC

>N2027

AAAAAAAAGAAAAAGAAAUGAAAGGAAAAAAAAAUAUUCCACUGCCGUGAA

>N2028

AUAACACAGUAAACUUGCUCCUCACAGGACCACAUGCAAAGGGCCAAUGUG

>N2029

UGAGCUGAGAAUCAUCCUGGUGGGCAAAACAGGAACUGGCAAAAGUGCUGC

>N2030

CCUCUUUACCCACAAGGAAGACCUCAAUGGUGGCUCCCUGAUGGAUUACAU

>N2031

UCUAAAUGUGGACCUGUGGGAUCAGAUGAAAGAGUAAAGGAAUUCAAACAG

>N2032

UAUUUGAGAUUCUAUGAAAUGUUUAAAUCUGAACAUCACUCCAAUUAUUAA

>N2033

AUCACUGAAACAUAAGUAAAGACAAAUGUCAGAAAAGAUAGCAAAUUGGAU

>N2034

UAAUUGAUGAGUUCACCAGAAAAAUAAAGAAAAUUCCAAAGGUAGAGCAAA

>N2035

CCCAAAUCAUGUCCCUAUGAACAAGAUCAAAAAGAUUAAAAAACCCAACAA

>N2036

AUCUAAUAACUUCAAAAAAAGAAAUAAAAAUAAAAGGUAUGAGUACUGAUU

>N2037

AUAAUAAUUUUAGAUAGAUAAUUAAAGGUAAUAAAAGUAGAGCAAUGAAUA

>N2038

CUUCCUGCCCCAUGUGAAACAAUCAAAAAAUGGACAAAUGAUAUAAAAUGC

>N2039

GACACUGAAAAUCAGGUAACAAAAGAGAGUAAUCACUGAGAAUUGGAAAAA

>N2040

AACAGACAGACCCUAUGUUGACUUCAAAUGAUUCCUGGCCCCUGGUGUUCU

>N2041

AGCCACAAAGAAACAAAUUGUGUCAACAUCCUGAGGGUUCAGAUCUUUCCC

>N2042

AGCUAUGUCUACACUCCCUGCCCACACUAAUCUUGAGAUAAUAAAUCUAUA

>N2043

UCACCACGGACCAUAAAAACUAAAAAAGCCUGUGAUUUGUGGGGCAUUGGC

>N2044

AGAAGAGCCUUGCCUCACUAGUGGGAAAUAAUUGGUUCUAGACUGACAACC

>N2045

AAGGUGGAAGACAUUAAAAAGAUCCAAAUUUAACCUCUAGAGAUAAAAACU

>N2046

AACUUAUGAUCAUCUAAAUAGAUGCAGAAAAGGCUUUCAAUCAAAUUCAAG

>N2047

GUCCUGGCCAGAGCAAUCAGAUAAGAGAGAGAAAUAAAAGAGAGUAACAAA

>N2048

AGCAAAGCCUCAGGAUACAAAAUCAAUGUACAAAAGUCACUAGCAUUCCUC

>N2049

AAGAAGAAUCAAUAUUGUUAAAAUGACCAUACUGCCCAAUGUAAUUUAUAU

>N2050

CAUAUGGAAUCAAAACAGCCUUAAUAGCCAAGGUGAUCCUAAGCAAAAAGU

>N2051

ACACCAACUACAACCAUCUGCCAAAAAUAAACAAUGGGGAAAGGACUCCCC

>N2052

CCAAUUAAAGAGCUUCUGCACAGCAAAAGAAACUAUCAACAGAGUAAACAU

>N2053

GAAUGGGAGAAAAUUUUUACAAAUUAUGCACCUGACAAAGGUUUAAUAUCA

>N2054

AGAAACUCAAGCAAAUUUAUAAGAAAAAAAAGCAACCCCAUUAAAAAGUGA

>N2055

CAAUAUCACUGAUAAUUAGAGAAAUACAAAAUAAACUACAAUGAGAUACUU

>N2056

AGGGAACACUGAUACACUGUUGGUGAGAGUGUAAAUUAGUUCAACCAUUGA

>N2057

CAUUGCAGCACUAUUCACAAUAGCAAAAACAUGGAAUCAACCUAAAUGCCU

>N2058

GACUGGAUAAAGAAAAUGUACAUAUACACUAUGGAAUACUACACAGCCAUA

>N2059

AGAUCAUGUCCUUUGCAGGAACAUUAAUGGAACCGGAGGCCAUUAUCCUUG

>N2060

GCAGGAACAGAAAACCAAAUAGUACAUGUUCUCAUGUGGGAGCUAAAUGAU

>N2061

GGACACAAAGAGGAGAACAACAGACACUGGGGCCUAUUUGAGGGUGCAGGU

>N2062

AACCUAAAAAUUUAGAAAAGAGAGAAGAUUAAUGAACUUGAAGACACAGCG

>N2063

CCCAACAUAGAAAACACAAAAGAAAAAAAGAAUAGUCACUUUCAAAGAACU

>N2064

GAGCUCUGGGACAACCUCAAGUGGAAUGUAUAUGUAACUGGAUUCACUAAU

>N2065

AGAAACAUGAUAAUCAGGUUGAUCAAAAUGAAUGAUAAAAGGACAAUUCUA

>N2066

AGGCAACUGUAACACACUGGUAAGUAUUUGUGUAUCUAAACAUACUAAACU

>N2067

UGACCAAAACAUCAUUAUGUGACACAUGACUGUAUUAGAAGAAAAACUGUU

>N2068

UAUUUUUCAAAAUUACCUUUCAAAAAUGAAGGCAAAAUAAAAGCUUUUCCU

>N2069

CUGCUUAAACAAAAAUAAUAACAAUAUAGAUUGAGUUUUGCAAUAUAUGCA

>N2070

GGGAAACAAAGAACAAAUGCGACAAAUUUGAAAAAUAGUAAGAUGAUAGAA

>N2071

AUUGUCAGAGUGGAUUUAAAAAGCAAAGCUUAUCUAUAUUUUGCCUACAUG

>N2072

UAAUCAAAAGAUACUUGGAAUGACAAUAUUAAUAUCAGACAAAGUAGACUC

>N2073

AAUAUUAUCAGGAAUGAAGAAGGACAUUCUACAAUGAUAAAGCCAUCAAUA

>N2074

GACAAAAAAAUCAGUAAAGAUAGAGAAGAUUUGAACACCAUUGUAUUAGUA

>N2075

CUCUUAAAAUUUAUAUUUAGAGUCCAUCUGGACAAUCAAAAAUAAUCUCUU

>N2076

UUGGUGAGGAUGAGUUCUUUUCUGGAGGCUGUACUGGAGAAUCAAUUUCCG

>N2077

CUCCCACUUGAGAGACUAGCUCCUAAAUCACUUAUCUCUGGGAACUGACAG

>N2078

UUACAAGUCCCCCCAGACCACAGAGAACAAAGAAUUGAUUUGAUUUUAAAU

>N2079

CCAGUUUCUCCCUGGAAGGGGUUCAACUGCAAACCUAACAUCUUAACUUUU

>N2080

UCUCUGGGAGCCUGAAGGGAACUAUAGGCACUUCCCACAAAUCCUCUUCUU

>N2081

AGAGAUAGUACCAAGCCUCCAUCUGACUCACCUGUCUCUGACAGCCAAUGU

>N2082

ACGAGAGUGCAGGAAUCUGACACAGAUAGUCUCCAUGCCUUAUUCCAGAAA

>N2083

ACCAUAAAUUUUCCAGCUUCUUCUCAAAGUAUUCACUUCUUUUUGAAUUGC

>N2084

UUUACCCAGCAGGGAAUUCAAAAUAAUGGUCUUAAAAAUAUUCACUGAGGA

>N2085

AGGCAUGAACAAAGUGAGACUUUCAACACACAGAUAGAAAAUGUUUAAACA

>N2086

CAGACUAAACCAAGCAGAAGAAAGCAUCAGUGAACUUGAAGACAGGCCAUG

>N2087

UGAAAGAAAGUAUUAAGUUGGUAAAAGAGUAAUUGCAGUUUUUGCAAUACG

>N2088

AACGCAAUUACUCUUGCACCAACCUAAUAUAUUCCAAGAGAUAAUAGCUAA

>N2089

AGCCUGGGGGAGUAAAUAGAAAUCCAGAUCCAGGAAGCCCAAAGGAUACUA

>N2090

AUCCAAAAAGACCCAUUAUAAUUAAAUUGUCAGAAGUUAAAGACAGAGAAA

>N2091

GAAAGAGAAAAGCAACCUACUACAUACAAGGGAAUCUUCAUAAGACUAUUA

>N2092

AUAAGAAGGAAUGAAGCUCACUGGUAAAGGUAAAUAUAGACAAAAAUAGAC

>N2093

UAAAAAUGAAACAGAGCAAGAGAUAAAAAUAGGGACAAAAGGGCUGUAAGU

>N2094

ACAGUUAAUCAAAUGGUAAUAAUAAAUCCCUCCUUAUCAAUAAUUAUUUUA

>N2095

GGAUGAGAAAGGAUAUUUCAUAUAAACAGUAACCAAAGGAGAGCAGGAAUG

>N2096

UAUAAGAUAAAGCAGAGUUUCAGUUAAAAAAAAAAACUGUUUUUAGAUACA

>N2097

UAGAUUUAUACAGAACUUUUUAUCCAACAGGAGAAGAAUAUAUAUUCUUCA

>N2098

UGGAACAUUCUCCAGAAUAGAUCACAUUUCAGGUCACAAAGCAAAUCUUAA

>N2099

CUGGGUAAAAGAAGAAAUCAGAGAAAUUUUAAAACUAUCUUGAGAGAAGUA

>N2100

UAUAAUUUAUCAAAAGUUACAGGAUACAGCAAAAGGAGUUCCAAGAACGAA

>N2101

ACCAUGUGUUCUCACUUAUAAUGGGAGCUAAACAUUGAAUACACAUGGACU

>N2102

AACAAUAGACACUGGGGAUUACUUGAGUGGGGAGGGUGGGAAGGGGGAGAG

>N2103

UGACACACACUGUACUCAUGUAACAAACCUGUACAUAUACCCCCUUAACCG

>N2104

UAGAAAAAAAAGAAGAGCAAUCUCAAAUAAGCAACCUAACUUUACACGUCC

>N2105

AAGACAACAAAUAGAUAUGGAUAGCAUUGAAUCUAUAAAUUACUUUGGGCA

>N2106

ACAAGGCUACAGUAACCAAAACAGCAUGGUACUCGUACCAAAACAGAUAAU

>N2107

GAACAGAACAGAGGCCUCAGAAAUAACACCACACAUCUACAACUAUCUGAG

>N2108

CCUGACAAAAACAAGAAAUGGGGAAAGGAUUCCCUAUUUAAUAAAUGGUGC

>N2109

GACUAGCCAUAUGCAGAAAACUGAAAUUGGACCACUUCCUUACACCUUAUU

>N2110

AGCAAAAGAAACUAUUUUCAGAGUGAACAGACAACCUACAGAAUGGGAGAU

>N2111

AGAAAAAAACAAACAACCCCAUCAAAAAGUGGGCAAAGUAUAUGAACAGAA

>N2112

AAAAGACAUUUAUGCAGUCGCAAACAUAUGCAAAAAAGCUCAUCAUCACUA

>N2113

AAAUGCAAAUCAAAACCACAAUGAGAUACCAUCUCACGCCAGUUAGAAUGG

>N2114

CUGUGCUUUUCCAACAGGCUUAAAAAACAGCGCACCAGGAGAUUAUAUCCA

>N2115

CUCUGUAGGCUCUACCUCUGGGGGCAGGGCACAGACAAACAAAAAGACAGC

>N2116

GCAGACUUAAAUGUCCCUGUCUGACAGCUUUGAAGAGAGCAGUGGUUCUCU

>N2117

GCAGCCUAACUGGGAGGCACCCCCCAGCAGGGGCAGACUGACACCUCACAA

>N2118

CUCCUCCAAAGGAACGCAGUUCCUCACCAGCAAUGGAACAAAGCUGGACAC

>N2119

UGACGAGCUGAGAGAAGAAGGCUUCAGACGAUCACAUUACUCUGAGCUACU

>N2120

CAAACCGAAGGCAGAGAAGUUGAAAACUUUGAAAAAAGUUUAGAAGAAUGU

>N2121

ACGUGAAGAAUGCAGAAGCCUCAGGAGCCAAUGCGAUCAACUGGAAGAAAU

>N2122

AUGGAAGAUGAAAUGAAUGAAAUGAAGCGAGAAGGGAAGUUUAAAGAAAAA

>N2123

CAGCCAAAAUAAGCUUCAUAAGUGAAGGAGAAAUAAAAUACUUUACAGACC

>N2124

UAGGAAGAAACUGCAUGAACUAACGAGCAAAAUAACCAGCUAACAUCAUAC

>N2125

AAAUUCACACAUAACAAUAUUAACUAUAAAUGAAAAUGGACUAAAUUCUCC

>N2126

CCAACAAAGAUCAAAAGAGACAAAGAAGGCCAUUAUAUAAUGGUAAAGGGA

>N2127

AAGAAGAGCUAACUAUCCUAAAUAUAUAUGCACCAAAUGCAGGAGCACCCC

>N2128

AACACCCCACUGUCAACAUUAGACAAUUCAAUGAGACAGAAAGUCAACAAU

>N2129

AAUUGAACUCAGCUCUGCACCAAGCAGACCUAAUAGACAUCUACAGAACAG

>N2130

CUUGGAAGUAAAGCUCUCCUCAGCAAAUGUAAAAGAACAGAAAUUAUAACA

>N2131

CAGACCACAGUGCAAUCAAACUAGAACUCAGGAUUAAGAAUCUCAUUCAAU

>N2132

GAAAUAAAGAUAUUCUUUGAAACCAACGAGAACAAAGACACAACAUACCAA

>N2133

GGAAAGAUCCAAAAUUGACACCCUAACAUCACAAUUAAAAGAACUAGAGAA

>N2134

ACACAUUCAAAAGCUAGCAGAAGGCAAGAAAUAACUAAAAUCAGAGUGGAA

>N2135

UAGAGACACAAAAAACUCUUCAAAAAAUUAAUGAAUCCAGGAGCUGGUUUA

>N2136

CAACAAAAUUGAUAAACCGCUAUCAAGACUAAUAAAAAAAGAGAGAAGAAU

>N2137

AGAAUACUACAAACACCUCUACGCAAAUAAACUAGAAAAUCUAGAAGAAAG

>N2138

UAGCAGGCUCUGAAAUUGUGGCAAUAAUCAAUAGCUUACCAACCAAAAAGA

>N2139

AAACUAUUCCAAUCAACAGAAAAAGAGGGAAUCCUCCCUAACUCAUUUUAG

>N2140

UCAUCCUGAUACCAAAGCCGGGCAGAGACACAACCAAAAAAGAGAAUUUUA

>N2141

AUCAAAAACCUUCUCCACCAUGAUCAAGUGGGCUUCAUCCCUGGGAUGCAC

>N2142

GGGCAAUUAGGCAGGAGAAGGAAAUAAAGGGUACUCAAUUAGGAAAAGAGA

>N2143

AUUCACAAUUGCUUCCAAGAGAAUAAAAUACCUAGGAAUCCGACUUACAAC

>N2144

GGAAGAACAUUCCAUGCUCAUGGGUAGGAAGAAUCAAUAUCGUGAAAAUGU

>N2145

CAAGGUAAUUUACAGAUUCAAUGCCAUCCCCAUCAAGCUACCAAUGACUUC

>N2146

UCCUAAGCCAAAAGAACUAAGCUGGAGGCAUCACACUACCUGACUUCAAAA

>N2147

AUCAUGCUGCUAUAAAGACACAUGUACACGUAUGUUUAUUGCGGCAUUAUA

>N2148

CGCAUAUUCUCACUCAUAGGUGGGAAUUGAACAAUGAGAACACAUGGACAC

>N2149

GCAAAAGCUGGAAGCACUCCCUUUGAAAACUGGCACAAGACAAAGAUGCCU

>N2150

AGGAUAUACAAAUAGGAAGUGAGGAAGUCAAAUUGUCUCUGUUUGCAGGUA

>N2151

AUGGUACUGGUACCAAAACAGAUAUAUAGACCAAUGGAACAAAACAGAGGC

>N2152

CUUAAAUGUCAAACCUAAAGCCAUAAAAACCGUAGAAGAAAACCUAGGCAA

>N2153

UAUCAAAAAUUGGGUGAAGGAUAUGAACAGACACUUCUCAAAAGAAGACAC

>N2154

AACAUAUAAAAAAAACCUCAUCAUCACUGGUCAUUAGAGAAAUGCAAAUCA

>N2155

UCCCAUUCUCUCUUGCUCCUACUCCAGCCAUGAGAUGUGUGUGCUUCCCCU

>N2156

AGCCCAGAGACAGUGAGACAUAAUCAAGAGCUAAUAGAAAUGUAGAGGAAA

>N2157

AAACGGUUACUUUUCAAUAGGAAGAAGAUCCCAGAAAGCACCAGUAGGAAG

>N2158

GGUGAUGAGGAUGUUCCCACUGCAGACAACUGGGUCUCAGUCCUGCUGGGC

>N2159

UAUCCAUAAGUCCUAACUGACAUUGAUUGAAAUCUGCUCCUAGGAUAUCAU

>N2160

GAGUUAUGAGUGGCUGAAGUAAGACAUCAUACACAUGCAUGGAAACAAUAA

>N2161

GCUGGGUCUCUUCCCUACCAGAAAAAAAGUGGUCUGGCAUGAAAGAGGUUA

>N2162

GCUCUUGAGAGAAGAUAGAAUAACUAGAACUGGUGAUACCAACAUAGACGU

>N2163

GCCUCCUAAUAAGGCUAAAUUGAAGAUAGUGCGAAGGUGUUCAUCCUCAAA

>N2164

ACCCUCAAGUCCUUCAAUAGCUGGGAAAGCCCUCCUCUCUCAGAGAAUGAC

>N2165

CUAUUUUAUAUGGGGAAAUGUCCCUAUAGGGCAAAGGCAACUGAAGAUUAC

>N2166

AAUUUUAGAAGUACAGUCAGGCCUCAGAGACUACAACAUCCCUCCCUUCUC

>N2167

GGGCUGGGGGUGCUCUGAGAACCAGAAAGAACAUGAUUCCUAGAGUGUUUG

>N2168

ACUUGCAUUCUACAAGGCAUCAUGCAGGCGAGCAAGAUCUGGAACAAUCAC

>N2169

CCAGGUUUUUAAGUAGGCAAAGUUCAGAGGAAAUUUUAGGAUCAUCUUCUU

>N2170

GCACACACGUUCUUGGUGGGGAGUGAAAUCUGUCUCCUCCCACAUUCCCUA

>N2171

CCUCAAAUUCCCAUCUGUACAAUAAAGGGGGUUGCAAAAAUGACUGAUGUA

>N2172

UACAUUUUUAAAGAACAUUCAAGUAAUUCUGAGAAGCAGCACAUUUGAGCG

>N2173

UUUAUGAUCAUCUGUUCAUUCCUACAUGCAAAAUAAUUAAUCACAUAAGGA

>N2174

GGAGGAUCAAUAGUCCUAGGAGGGGAAGCCGAGUGGGUUGGAAAGGCCCUG

>N2175

AAACCAAAUUAUUUGAUUUUAAGAAAACAGAGUUAGGGGCCCUCCAGAUCC

>N2176

AAGGUGGGUGCCUAAGUCACUGGGCACAUUUUCUGGUUCUGAGGGUUUACG

>N2177

GACUAGCCUGCUCCCUUGGUUCCACAGUAUAAAAUAAGGAUAAUAAACCUU

>N2178

CGCAGAAGAAGUGCAGAGGGGAUUAAAUCAUGCACUACAGUGAACAUGAUC

>N2179

GAGAGAGAUAAGAGAGGAAGGAGAGAAAGAGGGAGAGAAAGAGAGAAGAAA

>N2180

GGUGCAGGCAGACAACCCAAAUACAAAAGGAAAAGGUGGAAAUGAACGCCU

>N2181

AUCUCUAUUCUUAACCACUUAUGCUAAAGGCAAGAAGAUUCCAGUUCCCAA

>N2182

CUGGGAACAGCAUCCUGGGCCAGAGACGGUUCUUCUCCAGGCUGGGGGCCA

>N2183

CAGGGCCUGCACCACGGGCAGCCGCAGGUGGGACAAGUGCCACGUGGAAGC

>N2184

CACCGCCCAGGACCAGCAGGCGGUGAGGCAGGUGAGGGACAUGUUCGGGGU

>N2185

GGGAUGGUCGAGGGCUUGGUGCUGGAGCACAAGGGCGCCCAUUACUCCAAG

>N2186

AGCUGGCGCAGGUGCUGCGCUGGGCAGGCCCUGAGGAGCGGCUCCGGCGGG

>N2187

GAAAACUUUGCUGCAUCACCUUUGCAACUUUGCCAAAGCUCAGAGUUCACA

>N2188

AAUUUUCUAGUUCUUAUACAUGGGUAGAUAGGUUCCUUUGUGAUCCUUGUU

>N2189

AGUUUCCACUUUUCUAGAACAUAUUAUGGUUCAUGGCAUUCCAAAAUGAAC

>N2190

AGGUCAGGAGUUCGAGACCAGCCUGACCAACAUGGCGAAACCCCAUCUCUG

>N2191

GCAAGAAUCUGUCUCAAAAAAAAAAAAAGAAAAGAAAAGAAAAAGAAAAAA

>N2192

ACACACACACACACACACAGUCUGUAUCUCGAGCUCCUCUCUGGUUAUGGC

>N2193

UGGGCCCAGGCAGGGGCCUCGAGCCAGGGAAGGAUCCUUUGAUGACCAGAC

>N2194

AGGGCUCCCAAUAGGCAGUGUGCCCAGAGUAGCAGCUCGGAAGCAGUACUC

>N2195

UAUACCCACUUUCAACUAUAUGCAAAUUUAGGGGUGGAUUAUGCCAAAAUU

>N2196

ACCUCUUCCCUUUCAGCUUGUCUUCAGUGUGGUCUGGAGCCCAAUCCCCAC

>N2197

UAGUCCUGCCUCCUACCUCAGUAAUAAAAAGUAGUAGUAUAUGGUUAAUUC

>N2198

AAUUUGAAUAAUGUAUUAUUAAAGAAGUUACAGUUUUCCUUUACAUUCAAG

>N2199

CAAUUUUAUACUUAAAUUACAGCAUAAAACAGCACUGCCUCCUGUUGGAAU

>N2200

CUAAUUUUAACCAGACACAGUAAAAAUACUGUCCAAUACCUCAAAAAUUGG

>N2201

AGUUAUAAUUAUUGGUUUGAGUCUUAUUUUUAUACUUUCAAUCACAUUCUU

>N2202

GGCACUUGGACUUUUUUUACUUUCAAAAUGGUGUUAUAUUGUGCAUAUCCA

>N2203

AACGUGCGCGCGCGCACACACACACACACACACACACCAAGGAUAGACAUA

>N2204

AGCUUGCACAUGAAGACCUAGAUCAAGAUUGUCUCUGAUGCUCAGUUAGGC

>N2205

CCCCAUCUCUACUAAAAAUACCAAAAAAGAAAAAAUAAUGAGCCGGGUGUA

>N2206

UAGUACAAUACUCCCAUAGAGAAUAAUGUUAACACUCUGGAUAAAAUAAAU

>N2207

GCUUCAAAGACCUAACAAAAGAGUGAGGAAUUUCUAGAACAAACCUAAGAA

>N2208

AAAGUAAAAUCCACAAUCCAUCCAAAGUGAAGAAUGUCAUCCUCGCCUUAA

>N2209

GGGUGACAGAGUGAGACUCCAUCCCAAAAAAAAGAAAAAAGAAAAAAAGGU

>N2210

AUGUCUAGCACUCAGAGAAAACCAAACACCAAAAGAAACAUGAUGCAAUGA

>N2211

CAAAAAGUAACAGAAACAAAUAAGCAAAAACUUGAGAUACUAUAAUGAUCA

>N2212

UAAAAUAAGUAUACUUACAAUAUCUAAGACAAUUUUUAAGCAGUCUUUAAG

>N2213

AAUUGACAAACGGAAUGUGGCAGCCAUAAAACAUGCCACCCAUGUCACCAG

>N2214

CUUGGCUCUCUGAAUCUUUCCUGGAACUGCACUGCCCACGACUCUUCCUAU

>N2215

AAAAGGAAUAGAAAAACAGGAGUUAAGACAAUAUCUGAAAACAUGAGAAUA

>N2216

ACCUGUUCUGCACAAGACUUCUAUUACACAUAUACAAAGAACACCCUGGAA

>N2217

UUAAUUUUAUGUGUCAGCUUGAUUGAACUAAAGGAUGCCUAGAUAGCUAGG

>N2218

UACAUCAUCAGCUUCCCUGGUUCUCAGGCCUUCAAACUUAGAUUGGAUUAU

>N2219

AAGUAUAAUAAUCCAUAAUAAAAGGACAAAACAAUCCACAGAACCAGACUA

>N2220

CAAAUGUAAAUGUUAGAAAUUAAAAACAUUCUAUGAGAAAUGAAGUAUUCU

>N2221

AUUGUUCAAACUGAGCAAAAGGAGAAAAACAAAUGAGGGGAAAACAAUAUA

>N2222

GAAAUUAUAUCAAGCAGUUUAACAUACCUGUCAAAAAGGGAGAAUGGACAU

>N2223

GAAACAGAGAGCUCCAAGCAGGGUAAACACAUAACAAAGCAAAACAGAAAA

>N2224

AGGACAUUAUAUUAGAAGAAUAAAUAUUAGAAUUGCAGCUGAUUUUUUGUA

>N2225

AGAAGGAAUGAAUAUGACACCAGAUAGAAAUUUGAAUCUACACAAAGAAAC

>N2226

AGAAAUGGAAAAAAUAAAUACAAAUAUAAAAGAAAUUUCUUUUACAUUUUU

>N2227

AAAAAUAGUAAUAGUGUAUUAGGGAAGUUAUAAAAUAUGUAAAAGUUUACC

>N2228

AACCACAAGAAAACCAUAAGAUUCAACCAUAGGCUAUCUACUAAAAAAUUA

>N2229

CUGCACAGCCUGCAAACUGUGAGCCAAAAUAAAUGUAUUUUCUUUAUAAAC

>N2230

CAGUAUUUCUCUAUAGCAAUGCAAAAACAGCCUAGCACAGGAAUCCAGACU

>N2231

GGAUCCUCGACUCAGGGUUUUGUGAAGCUGAAAUCGAAGUCUUACCCAUGU

>N2232

UAAAGCAUGAAUGUUUUCUUUCUUAAAAGCUGGAAGUAUCUCUUGCUCUUU

>N2233

UAAUUAGGUGAGACCUACCCAGAAUAAUCUCCCCUUUAACUAAUGCAAAAU

>N2234

AUUUGCACCUAAAAACAGAGCUUCAAAAUACAUGAAGCAAAACUAAAGAAU

>N2235

AACAUGUAGACAGAAAACCGUAAGAAUACGGAAGGUACAAGACUGGAUACA

>N2236

AAAACUGUUUUUAUUUGCAGAUGACAUAAUUUUCUAUGAAGAAAAUCUCAA

>N2237

AAACAUUGAUGAGGAAAAUCAAACAAGACCUAAAUAACUGGAACAAUAUUA

>N2238

AGAUCAAUGGAACAGAAUACAGUUCAGAAAUAGACAUAUAUAUAUGAUCAA

>N2239

AAAGGGUACCAAAGUAAUUUAAUAAAAAAGGAUACUCCCUGCAAGAUAAGG

>N2240

AAAAACUGGACAUAUAUUUGCAAAAAAAAUCUUGAUUUAUAUCUCACGCCG

>N2241

AAAGCCACAGGAGGUAUGACUUCUAAUGUUGUCACUGAACUGUUUCUAACA

>N2242

AUGUUAAAAGACAAUGUGCACCCCAAGAAGCCUGGGCCAGUCCUACAGAUU

>N2243

UAACUCUGAUACCCCAUGAUGUCAAAUGUCAAUAGUAAUUUCAGACAGAAG

>N2244

CCCAGACAGCAGGACUUUGUAUAUUAUAUUUUUUUCAUCAAAAGUAGAGCA

>N2245

UUCAUGUCGAUGUCCACAUGGUAACAUUUGUCACAACAUAGUGUGUAAACU

>N2246

UGCUCACCAGGAGACAUUCAGUUGCAGGUGAUAAUGUCAGGGUAGAGGAGA

>N2247

ACUAAAUGGGUGAAGGUUCUACUCUAAAUGCAAUGCUAUGGAAUUAGACAA

>N2248

UCCACCUCCAGUCAACCUUGAUCACAUGUCCAAGUUCUACUCCACACCUCC

>N2249

CAUACACUCAUAUUUUGUGUGACUAAUUUUGAUAGUUUCUAUUCUACUCUA

>N2250

UGAUGCUCUACAAUGGGGUGUAUGAAAUACACUUGUCCAAAACAUUCAUUU

>N2251

UAGAGUGUACUUCAUGGCAUCGUUAAUCCUUCCUCCAUGUUGUAUGUCAAU

>N2252

UUCGUAAGAACACAAUGUUCUAAAAAAAAGUUCCAAGAAAGAACUUUUUUU

>N2253

GGGUUUCAUUUCCACAUUUCCCACUAGUCAUGUUCAUAGGAGACCUUCAUG

>N2254

GGGCCUUGUAGUACUAAAUCCAGUCAAAUUCAGGGCAAGUUGGCGGUUGGU

>N2255

CUUCCUCCUUCAUCAGAAGGCAAUAAUUGUCUGUCUCCCAUUUCAAACAGC

>N2256

UAUUUUGUACUAUGUCAGGUUUGGUAGAGAACUGUCUCUGUCCAGGCUGAA

>N2257

GGGGUUUGUUUGUUUUGCAAAUCUGAACAGAGGAAUAGAGAGGGUGUGGAG

>N2258

AGAGUCUAGUUUUGGAAAUCCUUUCAUAUCUGGAUAUAUACGAAUUUCUAA

>N2259

CCCUGGGGUCUCUUUGAGCCAUAAGAGGAGCCUGGGAACUGCUAGAGAAGU

>N2260

GUGGAUAAAAAUAAUGUUUGCAAAUAGUUCAAACCAAAAGGAAAGAAGAGA

>N2261

UCACUGUUUAUAAGAUUUUGAUGUGAACUCUGUUGGUCAAGUUUUUGAAAC

>N2262

GGUGCUGGGGGAGUACCUGGUCCCUAUCUAGCAAGGGUGAAAUCCUGGUCA

>N2263

UGAUUACUGAGUGAAUUUAGGAUGAACUAUGCAUGUCAAAGGGUGCUGUCA

>N2264

CUUAUGUCCACUGCCCUAACCUUAAAUGGCAAUCAGGUUUAUGUCCACUGC

>N2265

GACCUUUUCUGAACUUGGAAGUGGGAGAGUCUGUAAUUCCCUUCAUAUCUG

>N2266

AUACUGCUUGCUUGAAACAGUCAAAAAAAAAAAAAAAAGGAAAUGGGAUGA

>N2267

AGAGGUGUGGGGGACACACUCCAUAAUCUCUACUUUUCUUUUUGUGCAGCC

>N2268

UAGGCUUUCCCCCAUCUCCCCUAGCAUGCCGGGGCAUUCUUCUCAAUUUUA

>N2269

UUCUCCUGUCCCAGACCAGCCAGACACGUACCAUGUCUCAGACAAGCCUGU

>N2270

UGGAUUCUUAGGGCUCCCCAGGGCAAAUGUGGGAUAUUUUCCCACCCCAGA

>N2271

AAACACAUGAUUCCAAUACCUGGAAAUACAUGGGCUGGGGCAGAUGGGUGA

>N2272

GUCUCUUUUGUGGGACGAUUAGUAAAAUUUUUCCUUUGGAGAAGACGCCUG

>N2273

UAAAAGAAAAGACCUCAAUUCAGAGAAACUCCUGCCACCAGCAAUUUUAUU

>N2274

CCAAGAGGUUUGUUGACUUUUUUGGAAAAGUCUCUUUUUCCACCUGUGGAU

>N2275

AAUGUAUUCACAGAGGCUUAUUACAAUUAUGAUGUUCAUAAUCAAUAUCAA

>N2276

GAGAGGAUGUCAGGGAACUGGUGCUAUUUUAGCAAAAGCCAGAAGCAUAAA

>N2277

CGAGAACAGGGUGUGGGAGAGAGGAAAAUGUUCUUCUCUGGGCUUAAGAAA

>N2278

GGAGAGAGGGGAGGGUAAAGAGGAAAGGAGGAAGAAAAGAAAGAAGGGAAA

>N2279

CAGGAAGCUGUCUUAAUACAUUCUGAUAAUAUAAAAAUAGAUUUCUUGACG

>N2280

CAAGAAAAUCCACUGAAUGAAUAGAAAAAUGUGGAAAAGAUAGAAAUCACC

>N2281

AAAAUUGAAGUUUAUAAAUUACCAAAAUAGCAAAUAAACCAUGAAAACUGA

>N2282

AUGAUAAUCAGUAAAAUGUAAGUUUAAGCCAAUUAAGACAUCCUUAUUUAA

>N2283

AAAUGGGUGGUGCAAAUAGGAGUUAACUCAACUCCCUGGGAGUCAUAUUUU

>N2284

GUCACGUGGAAAAUGUACAUCCCCAAUACUGUUGCACAAUGCUGGAAACCA

>N2285

AUGAGUUGUUCUUGAAUUCGGCCUGAAGUUGAUUCUUUCUCUUCAAUCACA

>N2286

CUCUCCACCAACCGUCAGGGUGGGAAAGCGGUCAUACCGGUCCCCCUAUAC

>N2287

AUCUAACAUCACCUGCUUUGUUUAAAGUUAUUUACAGAUGUUUCAAAGUAC

>N2288

AGAAAAUUAGGGAUGGAAAUGGCUCAUUUGAGAAUGAUUAUUCUUAGAUGU

>N2289

GUGCCCUCAGGCUCUACCAGCUCCCAAACGUACAUCUGCUUUUUAUCCUUG

>N2290

ACCCCCAUCUAAAACACAGUGGCAGAAACCCAUUCAUUUUAAAUGAAGAAU

>N2291

UUGAAGGGUCUGGAAGCAAAUGGUGAAUAUGUUUUCUCUUUCUUUGUCUGG

>N2292

UGUGCUUCAAUCAGCCACAUAGAUGAGCUUUUCAACACACAUGCUUCUAUG

>N2293

UCCACCUUUUUGAGCCAUCCACCCUAGGGUCAUCUCCCAGACGCUGCCCUU

>N2294

GAAAGAGAAGUAAUUGGAUUUUCUCACUUGUAACUACUAAUCCCCUGCCUU

>N2295

GAAAGCUCUAUUUGCCACAAUCCAGAUGAAGUGCUUAAAGCAUCCCCUUAG

>N2296

CUAAUUCCUGGCUGUGGAUCCAAGCAUGGGCUUCCAAUGGGGCUUUCUACU

>N2297

AGCAUCCUUGGCCAGCCCGUGUUUGAGUCCAAGCUGAGGGCCCAGUCAGUC

>N2298

ACACAGUGGCCAUCAGGAAGGUGAAAGAGGUCUUUGGGACAGGGGCCAUGG

>N2299

CUGGACCCUGGAGCACUUCUAAUGUAUCACCCCAUGGAGUCAUUGUUCUAU

>N2300

UAAACACUAUUCCACUCUGUCUGCCAACAACUGCUUCAGGAAUGGGCCUGG

>N2301

CUGAGCCCUGAGCCACCAGCCCUGCAGCCUGCCCUAUCUCCGCAUUUCCAC

>N2302

ACAUUCCCUGAACUCUGAGUAAAGCAAAACACUUUCAGAUGGAUGGAUUGG

>N2303

UUCUCAGUUUGGCCAGGGGCCCAAAAGAAACACAGCUCACAUUUCAAUGCC

>N2304

UCACUUUGGCAAAUGUUCACACAUGAGAAAGGUUAAGCAAACUCUUUCUCA

>N2305

UAAAAUGUCCUUGGAUCAACUCUCCAGCUCUAUUUCUUUGGAAGUCAAUGG

>N2306

CCUGUCCCCAGGCUUAUACUUAUACAACCCAGCUGCCACCUGAUUUUCCCC

>N2307

ACCUGCUCAGAAGCCUCCAGGGAGGAUUCCAGAAGGCACCCUUGAAUCCUA

>N2308

UCCUUCUUGCUGACAUUGCUUCCAAAAGAAACCUUCUCCUACAGCCCCACC

>N2309

CUCCUGGUGCCAGGCAUUGACCCUGACCCUCAGUACUCACAUCAUAAAUGA

>N2310

GGACUGAAUUAAGUAAGUCAAGCUCAGUUGGAGCAGUUUGGGAACAAUGAU

>N2311

CUGCAGGUCUCCCUUACCGAACACAAAGAAGAAGGUGUGGUGGAGGGACCG

>N2312

UGCCUUCUUCUCUUUGCUGCUUGAGAUGGGGAACACCACCCUUUCCUCAAC

>N2313

CUGCUUGGUUCAGCAGAUGGAUCUCACCAUCCUUGCUCUCUGAGGUGGGGC

>N2314

CCUUUUCAGCCUGCAAAUAAAUCUGAGCAUAUCUAUGAAUAAAGUAAGGAA

>N2315

GUAAAAAGCAAGCAAAGUGCUGUACACACAUAAACAGAGAUGGCAACUCUU

>N2316

AAGGCAGGCUGUAGAAAUCACCUAAAAUGCUCAGUGUCCACAAGUGAUACA

>N2317

AAAAUGGUAAGAACAAUCAAACCACAUGUAAUAAAAAGGCAGACAUGACAU

>N2318

CAUCUAGUGUUUGACGCUGAGGAGCACCUUGCAUGGCCAGUUACUCUCAUG

>N2319

GAAGGAGCCCUCUCGCUCCCUCCCCAGCCUCUCGAUCGCAGCCAGGAGCUA

>N2320

CCCUAAGUCCCCUUUGUGGGUGAAGAGGAUGACCACAUGUCUCAUGACCCU

>N2321

GCUCUUGAGUCUGGACCUUGGACUCAAAGAUGGAGAGCGUGUCGACCACCA

>N2322

ACAGAAGGCAAAGGCAUCCAGAGGCACUGGCACCCUGGGUCAGUGAGGACG

>N2323

GCUCCCAUCACCUGACCCCAAGUACAGAGAAUAGAGGGACUCACACAGUGC

>N2324

AUAUAGUCUUGUGCCUUUCACUGCCAGGUGGGAAGUUUGCCCCCGAGUUCA

>N2325

ACGUUUCCAUGGUGAUGGUGGUACAAAGGGUGACCAACCAUCCUGGUUGGU

>N2326

UUCUCAGGCUUCGCUACUAUGAAAAACCCAUACAACCCAUUCCAGAGCAAC

>N2327

GACUGAUGUCAUGGUGCUUUAGGGGAAGAAGUUGGUGUUCCACGGGGCGUG

>N2328

UCCGGGAAUGAUGGCUAUGCUAAUAAUGGGUAUAGUGCCCGUGAAGAAGAC

>N2329

UCACUGAAAAUCUGAGAAGCAAAGUAACUGCUAUAAAAUCUUUUUCCAUUC

>N2330

GGAUUUCUAGGUAAAACUAUGGGCAAACUGGAGAUUUUAUCCAUAAGGAGU

>N2331

ACAACUUUGAUAAAAAUCGGCACCAAAACAUUCCUAGUUUUCAAACGCUAU

>N2332

UUUGUUACAUGGGUAAAUGUGUGCCAUGGUGGUUUGCUGAACCUAUCAACG

>N2333

GCUGAAGAUAAUGGCUUCCAGCUUCAUCCAUAUACCUGCAAAGGACACGAU

>N2334

AAGUAGUCAACCAGUCUGUGAUAGAAUGGUAACUGAAUUUUCCUAACUGCG

>N2335

AAGUUAAGUAAUUACUAAACUCUAAAUGGAAAUAGUAUUCAAGAAACUCAG

>N2336

UGAAUAUAAGAGUAUUGUUGCUUUAAUCCAGUGUAUUUGUUUAUGGAAAGU

>N2337

UGGAAUUUACCCGUUUAUAGGCUCCAAAAAUAAAUUUUCAAAUAAAAUAUU

>N2338

UAUCUAGCUCCCUGCCCCCCAACAGAGACACAGUUAAAUUGGCCAGUGAUG

>N2339

UGAUGGUCAUUCUUAUGUGUCACUUAGCAAGGCCAUAGUACCCAAUGAUCG

>N2340

AAAUCUCAUCUUUUUGGCCAGGCGCAGUGGCUCAUGCCUGUAAUCCCAGCU

>N2341

CCCUUUCUCUACUAAAAAUACAAAAAUUACCCAGGUAUGGUGGCGCAUGCA

>N2342

GCUACUCAGGAGGCUGAGGUGGGAGAAUCGCUUGAACCCAGGAGGCGGAGA

>N2343

AAAAAAAAAAAAACUCAUCUUUCCAAGGAUGAUUCUUUUCUUUUCAAGAAA

>N2344

GCAAACUGAGUGCCUGUAGGGCCUGAAUCUGUGGCUGCCCCUGUGUGCUGG

>N2345

CGGGCCAUUUCCUCCGAGAAGGAGAACCUGAAAUUGCUACUGUCUCGGAAG

>N2346

CUCCAAAAAAAAAAAAAAAAAGCCUAUUCUGGGCUGGCCAUCAACUCACCC

>N2347

ACAUGGUAAAACCCCAUCUCUACCAAAAAAAAAAAAAAAAAUUAGCCUGGC

>N2348

AUGCCUAUAGUCUCAGCGACUAAGGAGGCUGAUGUAGGAGGAUCACUUGAC

>N2349

CUGUCUCAAAAAGAAAGAAAGAAAAAGAAAGGAAGGAAGGAAGGAAGGAGC

>N2350

AAGGAAGGAAGGAAGGAAGGAAGGAAGGAAGGAAGGAAGGAAGGAAGGAAG

>N2351

UUAACUACUUCUAUAUAUAUAUAUAAACCACACUAAGCCAAGAAUUUACUU

>N2352

UGUAACUCUAAAACUGCUUUGAAAAAUAUAUACAUAAACGUAACUGUAUAC

>N2353

AGUUAUAUAUAUAUUUAUAUAUUAUAUAUUUAUAUAUAUUAUAUAUACAUC

>N2354

GACUAUCCCCUACCUUCUGUGGAAGACCAGGGUGGGGGAAGGAUGUUCAGA

>N2355

UUGCCCUGCAAUUUGUGGGGAAGCCAUAUGUGUACCCACAUAGAGGGUGCC

>N2356

UGUGCAUAUAUGAAAAUGCUCUUGCACAAACACAAGAAAUGAGCUCAUCAA

>N2357

AAUAAAGAAACUGAGGUUUAGAAAGAUCAAGUGUCUUGUCCACGCUGGCAG

>N2358

UGAGACAUGAUGAACCUGGCCACAAAUUUGGUCUGCUAGAGAGAGAGAGAA

>N2359

AGGGACCAUAUCCCUGGGGACAGGAAAAUAACAAUGUGUGUGGGUUUCCGG

>N2360

CCUCAGAGGGUGACUCAAGGCCCUGAGAGACAGCCAGGGUAAGAAGCUGUC

>N2361

CAGGGAAGGCCAACAGCUGCCUGUUACUGGCCCAGGCUGGACAUAUACACC

>N2362

ACCAUCUGCAGUGCGCCAUAAAGAGAGUUUCUUAUUUUUGUUGUUUUUUUC

>N2363

GGGGUCUCCCUAUGUUGCCCAGGCUAGUCUUGAACUCCUGAGCUCAAGUGU

>N2364

AAUUCUAGAUGGCAGUGAGGACUGAACCCAAGGGCACUCUCCAGGUUCCAG

>N2365

GCAGGCAUCUUUCUUGAAACAUUUUAUGGCUCCCUCUUACUUCUGCCAAAA

>N2366

GGAUUUCAUUUCCGCAGGGACCAGGAAGACAUCUGACCCCAUUCAUGAUGA

>N2367

GCAAUGAAAGCCAGAUUUGACUUCCAAAAUAUUCUCUCAUAUUAAUAUUGU

>N2368

ACUAAAUUCUUAUAAGAUUUUUUGGACAAAUCUAAAGGGAAUCACUGAUAG

>N2369

AAAGUGUAGCACAUCUAGACCAUGGAAUCUUAUUCAUUCAAAAAGUAACUU

>N2370

UCAUGAGAAAGCAUAAAGAAAACUUACAUGCAUAUUGCUAAGUGAACUAAG

>N2371

AAUAUAGGGAAUUGUUAGGGUAGUGAAACUAUUCUGUGUGAUACUAUAAUG

>N2372

CACACUAAUGCAAGAUGUUACUAAUAGGGGGAAUUGCUAGGGCAGACGGUU

>N2373

AAACCUAUUAAUUUUAAAAAGGGGGAGAUUAAAGAAGAUAAGAUGAGGAAA

>N2374

AGGGGAAGAGGAAAACAGGGAGGAGAGGGGUAGGAGGGAAGAACAAGGGUG

>N2375

AACAAAAGCCUUAUAUUAACCCAAGAUGUAGAUCAUCUUUUAUACUGCUAU

>N2376

AGGAUGGAUGGAUAGAUAGAUAGAUAGAUAGAUAGAUAGAUAGAUAGAUAC

>N2377

AGAUUCAUAGAUAGAUAGAUAGAUGAUAGAAUACAGAUAUUAAUAUCUAAU

>N2378

AAUAAUAAGUCAAUAGAGGAGAUGAAAUAGAAUCAUAAUUCCAGUAAAUGA

>N2379

GUAGAUUUUAAUUCAGUUAUAUAAUAACACGAAAUGUGAAUGAUCAAAAUG

>N2380

AAGUCACUUUAAAAACAAAGACAUAAAUAUGUUACAAAUAAAAGAAUAUUA

>N2381

CUUUAUCAAGUUGAAUGAUGAAGAAAAAGGUGAAAUUUUAAAAAAAUAAAG

>N2382

AAUAAAAAUUUUUUAAAACUUCAGAACAAAGAGUAUUAUCAAAGGUAAAAA

>N2383

AUAAUGAUAAAAAUGCCAAUUUACCAAAGUAACAUAACAAUCCUAAAUGUC

>N2384

UAUCUACAUUACAGUUGGAGAUUUUAACACAUCUCUCUCAGUAAUGGAUAA

>N2385

UAGAAGAUCAUUAAGUAUGUAGAUGACUUGACCACUAUCAACCAAUUUGAC

>N2386

CCAAAAUGGAAUUAAAUUCAAAAUAAAUAACAGAAAGAAUCUAGAAAUUGA

>N2387

GAAAAUUAAACACAUACUUGAAAAUAACCCUUGGGUACAAGAAACCUCAAU

>N2388

UACAUGUGCCAUGUUGGUGUGCUACACCCAUUAACUCGUCAUUUACAUUAA

>N2389

ACAUUUUCUUAAUCCAGUCUAUCAUAGAUGAACAUUCCGGUUGGUUCCAAC

>N2390

UGAGAAGUGUCUGUUCAUAUCCUUCACCCACUUUUUGAUGGGGUUGUUUGU

>N2391

UUUCUUCUAGGGUUUUUAUGGUUUUAGGUCUAACAUGUAAGUCUUUAAUCU

>N2392

GAAAGUCAUUGGUAGCUUGAUGGAGAUGGCAUUGAAUCUAUAAAUUACCUA

>N2393

CAUUGAGUACCAAAGCCUGGCAGAGACACAACAAAAAAAGAGAAUUUUAGU

>N2394

AAUAAAUUUGGUAUUGAUGAGAUGUAUCUCAAAAUAAUAAGAGCUAUUUAC

>N2395

CAGCCAAUAUCAUACUGAAUGGGCAAAAACUGGAAGCAUUCCCUUUGAAAA

>N2396

CCAGGGCAAUCAGGCAGGAGAAAGAAAUAAAGGGUAUUCAAUUAGGAAAAG

>N2397

AUUGUCCCUGUUUGCAGAUGACAUGAUUGUACAUUUAGAAAACCCCAUCGA

>N2398

UCCAUUCACAAUUGCUUCAAAGAGAAUAAAAUACCUAGGAAUCCAACUUAU

>N2399

AAGGACCUCUUCAAGGAGAACUUCAAGGCAAAUUUUAAAAAUAUGUUUAAG

>N2400

AUAACAUUAAAUGCUUAUACCAGAAAAGAAGAAAAACCUCAAAAAAUUAAU

>N2401

AGAUAAGAACAAAGUUAUGAAAUUAAAAACAGAAAAAAAUAGUAGAGAAAA

>N2402

UGAUGGGAUCCAUACCCUAAAUCUCAGCAUCAUGCAAUAUACCCAUGUAAG

>N2403

AUGUACCUCCUGUAUCUAAAAUAAAAGUUAAAAAAAUUAAUAAACCUCAAC

>N2404

CAUUAGGUUGGUAUUAUCAACACACAUGCACACACAAAAAAAUAAAAAAAC

>N2405

UCAGCAAGGAUGUGAGAGAAAUUGGAGCUCUUGUGCAUUGCUGGUGGGAGG

>N2406

CCAGCAAUUCCACUUCUGGGUAUACACCCAAAAGAAGUGAAAGCAGAGACU

>N2407

UGUCCAUUGACACAUGAAUGAAUAAACAAACAUGAUAUAUUCAUACAAUGG

>N2408

AGCCUUUUAAAAUAAGGAAAUCCUGACUCAUGCCAUCACCUUCAAUGGCAC

>N2409

AGUAUAGCAGUUUCUUAUAAAUGGAAAAUAGUUUUGCAAUUUCUUAUAAAC

>N2410

ACUUACCAUGUGACCCAACAAUCCCACUCCAAGGUAUUGACCUAAGUUAAC

>N2411

GUCUUUCACAGAUGAAAGGGUAAGUAAGUCUGUGUAUAUCCAGACAAUGGU

>N2412

GCAAUAAAAAGUAUCAACCCACAACACAUGAAUGAAUCUGAAAUUCUUUUA

>N2413

AGAAAAUCCAAAAUGUUACAUACUGAAUGAUUCUAUUUAUAUGGCAUUCUG

>N2414

UGCCCCACUAAAGGGCCUCAUUAGGAAACUUUUGGGCAGUCCAAUUGUUCA

>N2415

AAGUUAGGAGGGAAGAAACGAGCUGACCUAAGUGCCUUUGUAAAGCAAUGG

>N2416

GAGUGUCAGGCCAAAGACAAGAAGAACAGUACAGAAGCAACAAACUCUCAA

>N2417

GUUUUUCACAAACAACCCUAAACCUAUGGUAUACUUGGGCUCAAAAAUAAC

>N2418

GUAAGCUAUGGGAUCCACUUUUCUCAUUGUCAGAAAGAAGUUACAAAUAAU

>N2419

UUAUUUUUAGUAGAUACAAAAAUAUAAUAUGUGUGCAUAUAUGGGUCAGUU

>N2420

GAAUUACCUAGCUCUGCCCACUGAGAGAGAGGAGACUAUGAGCAGUGACAU

>N2421

GAGCACAGCCAGCACUCAGAUCUUGAUUUCGAAAUGCCGUUCCCUGAUGAU

>N2422

UGAAAAAUGCAGAAGAGGAGGCAAAAAUUUGCCCCUCCUCUUUUAUGGUUG

>N2423

GGAGAAUUAAAUAGACAUAAGACAAAUCAACAGGAGAAAAGUAUAUAAAUU

>N2424

CAAACAUUUAUGCCCUGAAUUGUGAAAAUGUAACAAGGCAAAGGGGCUUAU

>N2425

AUAACUGGGUAAAUAAGUAAUUAGGAACAUAAGGGAUGCUUGUAUAGAUUA

>N2426

CAACUUCCUAUCCUUUGUGAUGAGAAUGUUACUUUCUUCUUGGUUUAGGGU

>N2427

UUUGGGUUGGAAGUUGUGAAACAAGAGAUCAACGAAGGGAAAGAAAAACAA

>N2428

ACACAGAAGAACAAAUAUUCCCCGUAUCUUAUUGACACAGCCUUUUAGUCC

>N2429

GAGGCAUUUCUAUGGAAACAAAAGAAAAAUAAAGGUUAAUGUUUAGGGCAA

>N2430

CAGCUUUUGAGUCCAGAGGGCAAUCAGUUGGUAAAGGGUACUAUAGGUUGC

>N2431

UCCACAGUCACUCCCACUUUUACCAAACAUAAUCACAGUAAGACUAAUUUC

>N2432

AUGUUUUCCAUUGUGUCCUGUUACAAACAGAUAGAUUCUUAUUGGACCUAA

>N2433

CAGAUAGCGUAGGAUAAAAGAGAAAACAUUUCCUCAGAUCUGGAAAACAAA

>N2434

ACGAGCAAUGUUUUGAAAAAAGGUAAUUUUUUAAAGCAUCAUUUUUUUUCA

>N2435

CAGUUUCUUCAUUAGAGUUCUGGAAAUUCUUACCCAGGCCAAUGGUGGGAU

>N2436

AGUGGCUUGCAAAAGCUUUUAGGAAAAUAUCACAGUAAAAUAACAUGUAAU

>N2437

AAAGUUAGUUCAAGAUCAAUAAAAAAGGCUUAAUUUAGAAAUUAAUCUUGC

>N2438

ACAAUAAUAAAAACUAGGAGUGGAAAGGAUAGGGGUUUAGAUGAAACAAGA

>N2439

UGUAUUUAGAAUUUUCCACUUAAAAAGUUUUUUUAAAUAGAUUGACAGAGA

>N2440

UCAGCUUAAAAUCAAGAGAAGACCUAAAUUUUUAAAAAUCCCUUUAGUGAU

>N2441

AAAAAGUUUGAAGACCAUUGCCUUAAAGAAAAACUUACAUGAAUGCAUAAA

>N2442

CACAAUUACCUACAUCAACAUGAAUAAAUAUCAUAUACAUAAUGUUUAAUA

>N2443

CGUUCUUUUCCCAGAAGAAUACAGAAUCUGAUUCCAUUUUUGACAAGGUUC

>N2444

AAAACUAAAAAUAUAUAUUGUUUAGAGCAACAGACAUAGGUGAUACAAAAA

>N2445

GAAAGAAAGUGAAUGAUUAACACAAAAGAGAGGAUAGUAAUAAUACCCUAA

>N2446

AGGAACUCAUGGAGGUGGGAUGGGAAGCUUCGAAGGGCAAUGCUCUACUCG

>N2447

UUAACUAUAAAUAUAGCUCAACCAUAUUGAAAAACAUGAAACAAGACCUGC

>N2448

AAACAGACCAUGUUCUGGUAUGAAAAUACUUACCACUAUGAAGAUAUACAA

>N2449

UGGAUAUAUACAUAAUUAAAUUCCAAUUGAAAUCUGUUGUUUUUGUGUGUA

>N2450

AAUAAUGGGAAUAAAUGUAUGAAAAAGCAGAGGAAAAUUGGUUAUUUAGUU

>N2451

GAAAAAACAGCUUUCCAAUUAGAAGAAAGUAAAGCUGGGCUGCUACCUCAG

>N2452

GAGGCUGAGACAGGAGAAUCGCUUUAUCCCGGGAGACGGAGGUUGUAGUGG

>N2453

CACCACUGCACUCCAGCCUGGCAACAGAGCAAGACUCCAUCUAAAAAAAUG

>N2454

AAAAAUUCACUUCAUAAUGAAGGAAAUGAAAAUCAAAACAACUUGAAAGCA

>N2455

CUCAAAAAAAAAAAAAAAAAAAAAAAUAGUGGUGAGGUUGGGAAGAAACGU

>N2456

CAUGGCAAUAAAAGCUCCAGUUAGUAUAGUCUGGUUUUGCAGAAUUGUUUU

>N2457

AACAUAGGAAGAGAGGUAAAAGUGCAUCAGUAGGGAUACUCAUAUUGGUAA

>N2458

ACACUGAGUGUUGGUGAAGAUGUGGAGGGACUGGUGGUAUAUAAAAUGGUC

>N2459

AGUACACUCCAGCCUGGGCAACAAGAGCAAAACUGCAUCUCAAAAAAAAAC

>N2460

UGACUCAGGCAUUCCACUCCUAGGUAUAUUCCUAAGAGAAAAGAAAUCAUA

>N2461

ACUUCUCAAUAAAAAGAUAUGAGCUACUGAUUCACACAACAUGGGUGAACG

>N2462

UAUGCUCAGUGGAAAAAGUCAAACAAAAGGAGUACAUAUAGUAUAUAUGGU

>N2463

AUGGGGGUGAGAAGUGUCAGGAGGAAGGACUGACAAGGGUCAUGAGGAUAG

>N2464

CAGAUUCAAGACAAACAACUUUAAGAAAGUUUCAGGAUAAAAACUCAAUGA

>N2465

UCGUAAUAGCCACGCACACAGAAAAAAAUAAAAUACCUAGGCAUUUUCAAU

>N2466

UAAAAUAUCUUUCACCUCCCUAACCAAGGAGAGUUUUGUAAUUCUCAACCG

>N2467

AAAGAUCUCUACAAUGAGAAUUACAAAACAAUGCUCAAAGAAUUUAGAGAG

>N2468

CAAGGUGGAUUGAAGACUUAAAUGUAAAACCAAAACUACAAAAUCCUAAAU

>N2469

AAAGAAACUACCAACACAGUAAACAAACAACCUACAGAAUGAGAGAAAAUA

>N2470

CCACAAUGAGAUACCAUCUCACACCAGUCAGAAUGGCUAUCAUUAAAAGUC

>N2471

AAACCCCAUCUCUACUAAAAAUACAAAAAAUCAGCUGGGCAUGGUGGCAGG

>N2472

AAAAAAAAAAAAAAAAAAAAAAAUCAAAAAGUAACAGAUGCUUGAGGUUGA

>N2473

GAAUGCUUAUACACUGCUGAUGGAAAUGUAAAUUAGCUCAGCCACUGUGGG

>N2474

GUGAUUUCUCAAAGAACUUAGAACUACAAUUUGAACCAGCAAUCCCAUUAG

>N2475

AUAUCUGUGAUAGCAAAGACCUGGGAUCUACCUAGAUGGCCAUCAAUGGUA

>N2476

AGAAAAUUGCUACAUAUAUACCUGUAUAUGGAAUACUAUGCAGUCAUAAAA

>N2477

AGGAUCAAAGAACUACCUAUUGAGUACUAUGCUAAUUGCAUAGGUGAUAAG

>N2478

CUAAAAUAAAAGUUGGGGGAAAAGAAACAAUAGAUGUUGGCAUGGAUGUGC

>N2479

AAUUCGUAUAAUCUCUAUGGAAAACAAUGUGGAAAUUUCUGAAAGAACUAA

>N2480

AUUUUAUAUAUACACAUAUAUACAUAUACAUAUAUUAUAUAUAUAUAUAUG

>N2481

GAGACAAGCCAGGCACAAAAAGACAAACAUCACAUGUUCUUUUUUUUGAGU

>N2482

AAACCCAAGGACGGAGGAGGGAGGAAUAAUGUUUUCAAAGAAAAAUCAGGA

>N2483

AAAGAUUAAUCAUUUAUUUAUUGAUAAUCUUGGUGCACACUAGAGCAAUGG

>N2484

UCUGGAACUUCAUAUAUAUGAGUCUAAGUGAGUAUUUUUUUCCUACUUAUC

>N2485

CAGACUGGAGUACAGUGGUGCAAUCACAGCUCACUGCAGCCUCACACUUCC

>N2486

GGCUGGUCUUGAACUCCUGGGCUCAAGUGACCUGCCUGCCUCAGCCUCCCA

>N2487

UGAUAGAUGCACGAGACUGCCAUAAACUAUAGUCUUAAUCUCACUGAAGAU

>N2488

ACACCUGCUGCCUCUGCCCUCGUGGAAACCCUCCUGGUACCCUUCUCCCCG

>N2489

CCAGGGGAAGUCUUCAGUUUUAAAAAAUUUUAAUGAUUCUAUUUAAAUUUU

>N2490

UCUGAAACUAUCCUACGCGUAUACAAACAAAUAAACAUAUAUUUACAUAUA

>N2491

UGCAUACAUUGCAAAGAAUAUAUACAAUGUUAUGUUACACAAGUUUUUGUU

>N2492

UUACAGUGUUUCUACAUUUAUAAGAAAUUUAACUUGUCUUCUCAUUGUACA

>N2493

UUAAGACUGGUUUUUUGCUUUUGUUAGCGCUCUUCACAAAGCACUAAUUUA

>N2494

GAUUUUUCAGCAAUAUAUAAAUGUUACAGCAGAAAUGUCCAUACUGUUCCA

>N2495

GUAUUAAGACGUAAAUGGGAUCUAGAAUUCUGAUUUGAUUCUGUUUCUCUG

>N2496

ACAGAGUGAAUUAUCUAAGACAAAUAAACUAUACAUUUUAUGUAUAUGUAU

>N2497

ACAUAUACAUUUUAUGUAUAUGUAUAUAUAAAUGUACAUAUACAUUUUAUU

>N2498

UUAUAUGCAGCUUUUUAAGGAACAGAACACUUGCUUGAAGUGAGUUUCCCU

>N2499

AAUAUCUGGGGCCUACAGCCUAGACAACCCACUUAAGUGCUAGUUACUAUA

>N2500

AGGCCAUGUGCCCACAUUUGAAACAAUCUCCAUGGCCUGGGGAAAAUGAUC

>N2501

AUGGAAAUCUGGGGCUGUUAUUAGAAGAAAAGAGAAUGGAUGCAGGGCCAA

>N2502

ACCAAGAAUGGUGGUGUAACUUGAGAAAGUCCCUAGAGGAUUUCACAACAA

>N2503

AAAAGUUCUCCCAGCUCACACCAAGAACCACUGGCCUACUCGAUUAUUCCC

>N2504

CUGUUGAUCACAUUGCAAACUGUUCAAUACAAACGGCUUAAACAAACUCCC

>N2505

ACUCCCCCUAAUAUCUCUCUUGUGUAUACAAGGCAGGAGGAUUUGUUUGCA

>N2506

AUUGCUGACUUCCAAGUUUAUAUAUACCAAAUGCUCCUAAACUCCAGGCUU

>N2507

UUGAACUCCUGUUUUCUGUCAUUUAAAAAUGUUCCCUCCAUAGUCUUCCAA

>N2508

AUCACUUCUUGCUACUUCCACUGCCACCAUCAUGGUCCAAAGCACAUCAGA

>N2509

CAUCCUUCUCACCCACCCACCCCCAAUUCCACACAUUCCCAGUUACAAAAU

>N2510

ACAAACACUGACAUACAACUGGUUCAGGGUUAGGAUGUGGGAGGCCCCCUC

>N2511

CAUGCACCCUCCCAUCCUCAUCUCCACAACCACCAGCCAGUGUGCAUGUCU

>N2512

GAGCUUCUCUCUGUUCCUGGGCUUCAGGAACAGGCAUGUGAUGAGGGCACG

>N2513

AUUUAGGUGCUUAUAUGAAACCAGGAUGUUCAAAUUAUUCUAUUUUCUGGG

>N2514

ACUCUGUCUCAAAAAAAAAAAAAUUAUAUUUUCAAGAGAAAAAUAAACCCG

>N2515

GCCCAUAACAAACUGCGGCAAGGGCACUACCUUGGUUCUGGAUAGAAAGGU

>N2516

UGAGAAACUGUCGGCAUUAUAGCUCACAUGUAGAUUUGAGGCCUGCCUUAC

>N2517

CAAGUGAGAGAGUUAGGAGAGUGAGAAAGAAAAGGAGAAAAAAAGAGCAUA

>N2518

GCUUAAUCCACUAGAAAUCUUUUGAAAAAUAUGUAGAAUUUAUAGCCUCAG

>N2519

CAGAAACAAACUAAAAUCUUCUCUGAAAAAAGUACAUUCAUCAACCUAGGG

>N2520

UCCCACAGAUAAAGUUCCAACAAACAUGAGGUCAAAAUUUUUUUUUAAAUA

>N2521

UAAUUAAAUAAAAUAUUUUUAUAUAAAAAUCCCACAGCUAACACCAACGGA

>N2522

UUUGAACUAAUAAGUAAAUUCAGCAAAGUUGCAGGAUACAGAAUCAACAUG

>N2523

AAGGACCACUUCAGUCCAGGAAUUCAAGGCUGUGGUGAGCUAUGAUUACAA

>N2524

UCGGGGAGGCAUGGCUUUGGAGAAUACCCUCAGUGCUCUCCUCACUUGUAG

>N2525

CAGGGGAGGGAGGUAAAUGAGAUAAAUCCUGAAGUUCACAGCAGCACCUAU

>N2526

CACAGGGCCUCAUGCCAUCCGCUAGAGAACCCUAAAGAUUUCUUUAGAGUC

>N2527

GUAAAAGCAGACUCUGGGUUUUUGCAGAGGCAAGAGGUGGUCAAAAGAAUU

>N2528

CCCACACUGAGCAACAGAAUUAGACACCACCCAUCACCCACAUCACAUGAU

>N2529

AGGCUUGAGCCACUGCACCCAGCCGAUAGCUGCAGUUUUUAACACGAAAAC

>N2530

AACCCAGGGGGAACAAAGAAAAAGAAAAAAGAAAACCACCAAGUUGACAUC

>N2531

AGGAUGGUCUCUAUAUCUUGACCUCAUGAUCCGCUCGCCUUGGCCUCCCAC

>N2532

GAAGUGAAAAAAAAAAUCAACCUUUACCCCCUAGUCUCUAAAAAAAAAAAA

>N2533

CUUAUUGACUGAAUAUUUAUUAAUAACCCGUAGAAGGCUGAAUACAAGAAG

>N2534

CAGUUUGAAACAGGGCAGCUCCUGCAAAGCCCCACUCUGGGGUAGCAGAGC

>N2535

AGACUUAAGUGGCCCCAGAUUUCCAAGUAUUUCUACUUCCUCUUUUUGACC

>N2536

GCCCUUGAUAGAAGGACAUUUCAGAAGCCUCUCUCGGCCUCAGACAGUUUG

>N2537

UAUACCAGAAAAGCCUAGGAGAUUUACUGGAAAUUAUACUAGAGGAGACUU

>N2538

ACAAAGGGCUCCUCAAUAGUAAACAAGGAAGGGAUUUUGAAAACGCAGUGC

>N2539

CUGAUCCCCCUCUCAGGCUGUCCCAAGCUGUCUCCCGUCUCCCAGUGUCUC

>N2540

GGCUGCCGUAACAAAACACCGACUGAGGGGCUUAAACAGGAGAAAUUUCUG

>N2541

CUCCCACCCUACCCCAGAAGCAAAAAAGGUUUCCUUCCUGCCUCAAGGACC

>N2542

AGUUCUGAAUAUCAACAUCUGUGACAACUAAAAGACAACCCCCCUGCCCAG

>N2543

GUUCCUUGUCUUCCUACAGUUCUUCAGCAAGCACACCACCUUCCUGCCUGU

>N2544

AGACUGUAAACUUCUUGGUGUCAGAAGCUCUGUCUUAAGUCUGUGUGUAUC

>N2545

ACACAGCAGGACAUGGUAGUCGGGAACGGUGAAGGGUCUGAGAUCUAACUC

>N2546

UGGGUUCUAGGACCCCAGUUCCCACAGACAAUGCAAAGAGGCAUUGCAGGG

>N2547

CAGCCGGUGGGCUGGGUUCUAGAAAAGGAACCCUGAGCUUAAGGAAUCUGA

>N2548

AGCACUCCACACCCCUCAACAGCGAAACAGAACGUAACAUUCUACACUGCG

>N2549

UCGGGGCCAGCAAGCUUUGUGUGUAAAAAGCCUAAUAGUAAAUAUUUUAUA

>N2550

CAUUCAGUCUCUGUUAAGACUCCUCAACUCUGCCCUUGUGGCACAAAAACC

>N2551

AGCACCGCCCUCGGCACAGGCCCCUACUCCAAGUCCUGUGCAGGAGCAGGC

>N2552

UUAUGUUAUAUCUUCAUAUAUAAAGAAAAUGGAAAAAGUCAAAAUCAGUUU

>N2553

ACAAUGAAAGGAAGGAAGACUAAGGAUGUGUUUAUGUGUGUGUGGAGACAU

>N2554

GGGCAAAAAUCUCAUUCCAUGCUCCACACAGUUCUCUUAAGUCUACAGGCG

>N2555

GCAACAAUAUCUGUUCAUGGACUUGAGAACCCCAGAGUGGGAACAGCAGGU

>N2556

AGGGGAAAAGGAUGCAGGCAGAGUUAGUAUCAUAGAACUCAGAAAGCAGAU

>N2557

AGGAGGGAAAGGAAAAGGUGACAGGAAAAGCAGAUCAGGCAAAAAAAAAAG

>N2558

UGACUCUAUCCAGCUUGCUGCUCCCACCACAGCUGACUGUGUUUCCAGCCC

>N2559

GAGAAGCCAUUGCCGGCUCUUGCUGACAGCCCCCGCUGAGCUCCGGACCAG

>N2560

GAAAACUGUGAUACCAACACACCAGAAAAGCCAGAAGGGUGACCCACUAUG

>N2561

AAAAAUGUCUGUAGCUUACAUUAAAACCUCCUGAAUGAAAGACCCAGCAUG

>N2562

AGACAGGGUCUGAGCGAUCACACACAGUGUCGAUGUAUAAAAAGGGUUCAA

>N2563

GAAGAGUCAUGAGGUCCCCGCCUCAAAUCCCUCUCCUCUUCCCCAGCCCUG

>N2564

CAGCAAGUUUGCCCGGGUGCUUCUCAUGGACAAUGGCCCCAGCUCCUGCUA

>N2565

CCACCAGCAGUUCCCAGAAUAGCCAAUGUGAAGCAGGAUCCCAGGGACAGC

>N2566

UGACACACAGACCAGGAUUCCACCUAUACCUGCCCCACCCCUCCCAUCUGC

>N2567

AGAUUCUGUCCCCUUAAAAUCCAUGAAGCAGCACUGGCCUGGGUCUCCCCA

>N2568

UGAAGAUUCCCACUUUUCCCCCCCCAUUCUCCUUCAGAGGUCCCAUUGUCC

>N2569

CAUGCCAUGGAGGUCUGUGAAGGACAGGGACUCCUUUGUCUCCUCACCAUC

>N2570

GAUAUAGCCCAGGGAUUCUCUCUGUAGCUUGGCCUGAAGUGCUGAACACAA

>N2571

UCCUGUUGCCCUACCUCUGGCUGGGACCGACUCCUCUGUGCCAAAGAGGAU

>N2572

CCUUUCCAUCCAAAUUUCUGUGGGGACACUUACAACUUUUUCCUACACAGU

>N2573

AAUAGUGAUUUCUCAUGUCCCUCUCAUGGGAAUUCAACCCACUUUCCUCUA

>N2574

UUGAAGAAGGAGAGUUUCCACUGUGAAAGCUGAGCCUACAAAUGGGGUCAA

>N2575

CCCAAAAUGUAAUUCUCUACAAAUCAGGCUGCUGAAGCUGCCUGCUGCAAU

>N2576

CAUUUCUCCUUUUUAUAAGCCUCUAACCUUCUCUUUGUUCUAAAGACAUAA

>N2577

CAAAGCUGACUCCUGGUGGAUGUGGACGUUGACGUGGGUGGGCUGGGAUGU

>N2578

UGGCAAAACUCCCUCUCUACUAAAAAUACAAAAAUCCGCACGGUGGUGUGG

>N2579

CUAUUUGCCUUGCAGAUGCAAAGUAAUAUUAUCAGUCUCUUAUAAAUCCUC

>N2580

UUUGCAUACAAAAGUGUGAAAAAGUAUUUCUGCCCCCUUUUCCCACAAAUU

>N2581

AUGAGUCCAUAAAAAGUGUCAACCUAAAUAACAGAGAGAGGCUCUCCAAAC

>N2582

UUUGAGGUGAUCAUCCUUGGCUACAAGGAUCAAUAACAAGGGUGACACAAC

>N2583

GUCAGUUAUCCUCCACGGAGGUGGUAGCAAUUCACAUGCAGGAGGCAAACU

>N2584

UUUCCUUUUAUAUGGGGAAAAAUGCAUUGAUUUUAAUCAAUUUUAUGUAAU

>N2585

CAAUCAAGAUAAAGGACAUUCCUACACAGCAAUACAUUCCCUAAAGCCCCA

>N2586

UACCCCCAACCACUGGCCAUGAGCAACCACUGACCUGCUUUCUGUAAUGAA

>N2587

UGAGUGAGGUUGGUUGUCUUUUCAUAUGUUCAGGGCCAUUUUUAUUUCUUA

>N2588

CUAUUCUAGUCCUUAUAUUCUGCCCAUUGGCAUGUCUCAUGGGCUAGGCAC

>N2589

UGCCUGGCUCUAAAAUAAAUCCUGCAUAGGCAAUUCUAAUGGACAUCCUGU

>N2590

CAGUUUAAAUCAAAUAUUAUUAACAAUUCUAAUAACACCUCCCAUUCGUAA

>N2591

CCGCCUGAAGAGUCGGAUCUGAACCAGGCCGGCACCACGGAGAUUCCCCUC

>N2592

CUGCACCCAGCUGUCCCUCCCUCCUACGUGAGUCCUCCUGGAAACCAGGUC

>N2593

CCGGGCGGGGCUGGAGGCGAGAACGACCCCACGGGCUUUCCCAGGGCCGUG

>N2594

CCCGCUCGCAGGUCCCGAGGAGCGCAGGUGAGGCGGCACCCCACUCCCGGG

>N2595

GCAGCCGGGGCGCAGCUGCUGGCACAGGAGCUCCACAGGCAGCCAAGGACC

>N2596

CCAGAGCCUGCGGACUGUGGAGGGGAGGCCGCAGGAAGAGCCCGGGAGGAC

>N2597

CAGAGCAGAUCUUGGGCGAAAGGGGAGGUUCCCGCAGGCUCUGUAGUCGCA

>N2598

AGACUGUGUCCCUGACAAUGGGAACAGCCGACAGUGAUGAGAUGGCCCCGU

>N2599

UCCUUUCCUCUGGGCCAAACUCAGGAUGGGCCGGCUCUCCUCUCCUUCCUA

>N2600

GCCUUCCUCCAACAUGGCGCCACAGAAUGCUGGCAGUCGGAGAGAUCUGAU

>N2601

CAUUCCCUGCCUGUUCAGAUGAGCAAACAGCUGAGGGCGGUGGUGGCAUGC

>N2602

CUGUCUGCUCUCCCAUACCCUUCCCAGUGCUGUUCCCACCAGGCUGGCUGA

>N2603

CUUCAACAGUUAAUGUCAACUAAGGAUCUAAGGACCUGCUCAUCUCCAGAC

>N2604

AGAUGGGGAAGGCAGAGCGUUCCAGAUAGUAUGUGGUCCCCCAGGAAGCAG

>N2605

GAUGGGUGUGUGCAGUUUGCACAGGAGCUGGGUAGGCUGGUCAUGGUACCA

>N2606

ACAAGCUCUUAGGUGUCACUAAAGCAGGGCCUUUUGCAGCCUCAGGAGUUC

>N2607

CCACCCUCUCAGCAAUUAUCUUACCAUGGAUAAUAAAGCUUAGCAUGAAAU

>N2608

AGCUGUGUGCUGGGGAAGGGCCUGCAUGGAGGCUCUCUCGUGGGAGAGGAA

>N2609

UCAUUUACGAGAAACGGGGUGGUACAUACUGGGUAAGUUCAGGGAAGGGCU

>N2610

CCAGGUUCAGGCUGGAGGUCACCCCAAUCUCCUGCCCUGUUGCAGGCUUCA

>N2611

CCGCAUCUCCAGCUCGAGUGACUGGAACACUCCAGCCCCCACUCAGAGUCG

>N2612

UCCCAGGUCAAUAAGGAGCAUGGGGACAGAGCAGGAAGGCAGCUGUCAUCU

>N2613

AAGGAGACAGGAAUGGACAGUGGACAGUUUGGGGAGGAGAAAGGGGCCCAA

>N2614

AAAGAGACCAGAAGGAAAUGUUGGAAGUGAGUGGAAUCUAGCCAUGCCUCA

>N2615

CCUCCUGAGUAGUCAUGUGAUAAUAAACUCUCAUGUUAUUGUUCCCAGGUC

>N2616

CCACAGCUCCCCAACCUCCUGCCUCAGGCUAGAGUUGCUGGCCCUGGAGCC

>N2617

ACUCUGAGCCCACUCCCUACCACUCACAUUUAAGAAGGUGAAUGCUUGGCG

>N2618

AAAAGGAUACACAAGGUGGGGAAAGAACACAUUAGGCGGACUCUGGAAGAU

>N2619

GCUUCCUAUAUCUUCCCACGGUGAGAGGCCACACAGUAACACAGCAGUGUG

>N2620

GAAACCCACCUAAGACUCAAAGACCAGGGUUUUUAUUGAGAGCUGGCCACG

>N2621

CACCUUAUCAGCCAAGGAACAUUCCAGAAGCCCAAUUCCUAGAAGACAGCC

>N2622

CUAGAGCCACUUAAGCUGGAGAGUUACAGUGAAGGACCCAGUAUAAAUCUC

>N2623

AGUCAGAUGAAGAUUCGUGAAUACAAGCCGGCCCUAACAUGGACUUAUGGG

>N2624

CAAAACACAACUAAAGCAAAGAUACAGAAAGAUAGUAAAAGGAUGGGGAAA

>N2625

CAACCAAAUUAGUAUGGCUGUAUUCAAGAGCAUUGCCAGAGACAAAGAGGA

>N2626

GAAGUCAAGAGAAUUCACUGGGAACAGAAAGAAUUGGUUGGUUUCCCUACA

>N2627

UCAACAACAUUAAUUGAAUAUGCCCAACGAAUCCUGGAUACUUACCUUUGC

>N2628

GUAGCAACAAAACUAUAAGAUACUUAGAAAUAAAUCUAGACACUCAAGACA

>N2629

UAAAUUCAGUGCAAUCCAAAUCAAAAUCUCACCAGAGUUUUCUUAGUAUGA

>N2630

UUCUCAACUUCAUACAAAAGAUCAAAAAAUCAAGAAUGUUGGUGUGUUCCA

>N2631

CAUUUCCAACUGAGGUACCUGCCUCAUGUCAUUGGGACUGGUUGGACAGUG

>N2632

GGAUUUUCCUUUCCUAGCCAAGGGAAGCCAUGACAGACUAUACCUGGAAAG

>N2633

CCCGCCUAAAUACUGUGCUUUUCCAACAGUCUUAGCAAACAGCACAGCAGU

>N2634

AAGGCCUGCUUUCUCUGUUGACUCCACCUCUGGGGGCAGGGCAUAGCUGAC

>N2635

CCAGCACAGCGUUUGAGCUCUGAGAACUGACAGACUGCUUCCUCAAGUGGC

>N2636

ACAGAAAGGAAUAGCAUCAACAUCAAGAAAAAGGACAUCCACACCAGAACA

>N2637

GUCACCAACAUCAAAGACCAAAGGUAGAUAAAACCACAAAAAUGGAGAGAG

>N2638

AGUAAUAACAAACUUCUCUGAGCUAAAGGAGGAUGUUCGAAUUCAUCGCAC

>N2639

UAGCCGAUUUGAUCAAGUGGAAGAAAGGGUAUCAGUGAUUGAAGAUCAAAG

>N2640

AAGUGAGAAGAAAAGUUUAGAGGGAAAAGAGUAAAAGGAAACAAACAAAGA

>N2641

GCAAAGCAGGCCAACAUUCAAAUUCAGGAAAUACAGAGAACACCACAAAGA

>N2642

GUGGAUCUCUCGGCAGAAACUCUACAGGCCAGAAGAGAGUGGGGGACUAUA

>N2643

UUAAAGAAAAGAAUUUUCAACCCAGAAUUUCAUAUCUUGCCAAACUAAGCC

>N2644

AGGAGAAAUAAAAUCCUUUACAGAAAAGCAAAUGCUGAGAGAUUUUGUCAA

>N2645

UGCAAAAACAUGCCAAAUUGUAAAGACCAUUGAUGCUAUGAAGAAACUGCC

>N2646

GGCAAAAUAACCAGAUAACAUCAUAAUGUCAGGAUCAAAUUCAUACAUAAU

>N2647

GCUCAAAAUAAAGAGAUGAAGGAAGAUCUACCAAGCAAAGGGAAAGAAAAG

>N2648

CAAAGAAGGCCAUUACAUAACGGUAAAGGGAGCAACUCAACAAGAAGAGCA

>N2649

AAAGAGACUUAGACGCCUACACAAUAAUAAUGGGAGACUUUAACACCCUAC

>N2650

AGACAGAUCAACAAGACAGAAGGUUAACAAGGAUAUCCAGGACUUGAACUU

>N2651

CACCAAAUGUAAAAGAACAGAAAUCACAGCAAACUGUCUCUCAGACCACAU

>N2652

CCCUAACAUCACAAUUAAAAGAACCAGAGAAGCAAGAGCAAACGAAUUCAA

>N2653

GCUAGUAAGACUAAUAAAGAAGAAAAGAGAGAAGAAUCAAAUAGACGCAAC

>N2654

AAUAGAAAAAGAGGGAAUCCUCCCUAACUCAUGUUACGAGGCCAGCAUCAC

>N2655

AAGCCUGACAGAGACACAACAAACAAAGAGAAUUUUAGACCAAUAUCCUUA

>N2656

GCAGAAAAGGCCUUCAACAAAAUUCAACGGCCCUUCAUGCUAAAGACUCUU

>N2657

GUAUUGAUGGAACGUAUCUCAAAAUAAUAAGAGCUAUUUAUGACAAACCCC

>N2658

CUCUCUCACCACUCCUAUUCAACAUAGUGUUGGAAAUUCUGGCCAGGGCAC

>N2659

AAGAGAAUAAAAUACCUAGGAAUGCAACUUACAAGGGAUGUGAAGGACCUA

>N2660

ACUACAAACCACUGCUCAACAAAAUAAAGGAGGUCACAAACAAAUGGAAGA

>N2661

UAAAGUUCAUAUGGAACCAAAAAAGAGCCCACAUAACCAAGACAAUCCUAU

>N2662

UAAAUGAUAGACCUAAAACCAUAAAAGCCCUAGAAGAAAACCUAGGCAAUU

>N2663

CAUAGGCAUGGGCAAGGACUUCAAGACUAAAGCACCAAAAGCAAUGGCAAA

>N2664

AUUGACAAAUGGGAUCUAAUUAAACAAAAGAGCUUCUGCACAGCAAAAGAA

>N2665

AAAAGUGGGCAAAGGAUAUGAACAGACACUUCUCAAAAGAAGACAUUUAUA

>N2666

GGAGAGGGUGUGGAGAAACAGAAACACUUUUACACUGUUGGUGGGAGUGUU

>N2667

CCAGCGAUCCCAUUACUGGGUAUAUACCCAAAGGAUUAUAAAUCAUGCUAC

>N2668

UAUGCACACGUAUGUUUACUGCGGCACUAUUCACAAUAGCAAACACUUGGA

>N2669

AUGUCCAUCAAUGAUAGACUGGGUUAAGAAAAUGUGGCACAUAUACACCAA

>N2670

UCAUUCUGAGAAAACUAUCACAAGGACAGAAAACCAAACACCGCAUGUUCA

>N2671

GUCUUUGUAUUUGUAAAAUGUGGAUAAUUGUAUCUAAAACCCAAGGCUAAA

>N2672

AAUAAAUGUGGAUUACUAACAAAAAAAAUUGGGGAGAACAGAAAGUACUCA

>N2673

GGACUGACUAUGCAGACACCACAGAAGGUGCAUGAAGUGGCCUUUGGCAGG

>N2674

UAAGCAAUAAGAUUUAAGAGGAAAUAGUGUGUGUGUGUCCUGGGAACAUCC

>N2675

GUUCUCCUGUGUGGCCAAUCCAGAUAGCUGCCUUAUACAACUGCCUCCUGA

>N2676

GCCCCCAGGGUCUGCAAGUACUAAAAUCUCUUAAAUUGUCACAUUGUGGUA

>N2677

AGAAUGGCGUGAACCCGGGAGGUGGAGCUUGCAGUGAGCCAAGAUCGCACG

>N2678

CGCCUUGGUGACAGAGCGAGACUCCAUCUCAAAAAAAAAGAAAAAAAAAAC

>N2679

GCCAGGCGUGGUGGCUCAUGCCUAUAAUCCCAGCUACUCGGGAGGCUGAGA

>N2680

CUUGAACCCAGGAGGCGGAGGUUGCAGUGAGCCGAGAUCACACCAUUGCAG

>N2681

GCAACAAGAGCGAAACUCCGUCUAAAAAACAAAACAAAACAAAACAAAACG

>N2682

AACAGAGCUUCCAAGUACAUGGAACAACUGUGGAUAGAACUGAAAGGAGAC

>N2683

CCACAGUUAUAGCUGGAGACACAACAUUCCUCGCUCAGUAAUAGAUAGAAU

>N2684

AUAAUACAAUCCACAACAAUAUCAGAAUACACAUUCUUUUCAAGUGCUCAU

>N2685

CCAAGAUAGACCAUAUCUUCGGUGUAUCCCUUAACCAUAAUGCACUAUAAA

>N2686

AAAAGUAAUGGGAAAAUCUCCAAACACUUGAAAACUAAACAACAUAUUUCG

>N2687

AGUACUUGUAUGAGAAAAGAAUAAGAAAGUUCUUAGCUUAAGAAACUAAAA

>N2688

AUAAACUCAAAAUUGACUCAAAGCAAAAUAGAAGAAAGAAAAUUAUAAAGA

>N2689

AUUAAACAGAAAAAUUAGGGAAAUCAAUGAAACUAAAUGCAGGUUUGUUGG

>N2690

ACACCUCUAGUAAGAGUGUCAAAAAAUUAUAAUAAUAAGAGAAACUGGCCU

>N2691

CCUGAGCCCAGGAGGUUGAGGCUGCAAUAAGUCCCACAAUCGCACCACUGA

>N2692

GGCAACAGAGUGAGACUCUGUCCAAAAAGAGAAAAAAAGAAAAUAAGAAAU

>N2693

UACAUAAUCUCUUCCAGAAAAUAGAAGAGGAGAACUUCCAAACUUAUUUUC

>N2694

AUAUAGAUUAAACAGGGAAAAGAAAAACUGUCCCUAUUUUAGGUGACAAUC

>N2695

GCUAAUAAGUGACUUUAUCAAAGUCACAGGAUACAAGUCAACACACAAAAG

>N2696

ACAAAAUGUUAAGGACAGGUAUCAAAUAAGACCUUACUAAAUGAGAUACAG

>N2697

UGGGUUGGGAGACUAAACAUAGUAAAGAUGUCAAUUCUCCCCAAAGUUUUA

>N2698

GCACAAUUCAAACCAAAAUCCCAGCAGAAAUUUUAUGUAUAUAUAAAUAUA

>N2699

UAUAGAGAAAGAGAUAGAAAGAGAGAGAGGGGGGAGCUGAUUUUGAAAUGA

>N2700

GCAAAUAAACCACAACAGCUGAAACAAUCUUGAAGACAAAGAAUAAAAUUA

>N2701

GAGUUGCUAUAAAGCUACAGCAAUCAACACAUUGUGGUACUGGUGGAGAGG

>N2702

CAUCAGUAGAACAGGGUAGAAAGAAAGUCUUGAAAUAGUCACACAAACAUC

>N2703

CUGAGGUUAGGCAAAAAGUUCUCAGACAUGACACUAAAAUACAGUUCACAC

>N2704

AAUAUAUAAAGAACUCUCAAAACUCAACAGAAAGCAAGAAACCCAAGUUUG

>N2705

AAUGGAAAUUCCAUCAAAGUGGUGCAAUGACCUGAGUAGGAGUUUACAGAG

>N2706

GAUAUGAUACUCUCAAAAUGGAAAAAUUAUAGUGAUGGAAAACAACUCAAU

>N2707

GACAAUACUGUUCUUUAUCCUGAUCAUGGUGUUGGUUACAUGAAUCUGUAU

>N2708

AGAAUCUAAAAUAAUAAUGAGUUUGAGAUGAAUUUGCCUGUCUCUAUCUCA

>N2709

UGGCACGACAUCUGAUUUCCUACAGAAGAGAAAACCCGAGACAUGGGACUG

>N2710

GGUUGCCAGAUUCUGAGAGUAAAAGACCAUUUUCCAGCAGAAGGAAAAACA

>N2711

UCAUUUAUCUGACUGAAGAAUGGUAAGAAUUUACAUCCAACCUAACCAUUU

>N2712

UUAGAAGCACUUAGAAGAAAAAUUCAUACCUUCUUUAGCCUGAGAAAGUAU

>N2713

ACAGAAUUAGAACAAGAUUAAUUUUAAAUUCAAAAGCCUGGUUCCGUAUCA

>N2714

AAAGAAAACAUUAACUCUUUCAUGAAAUUAUGUGAAAAAUAUUAAUUCAUA

>N2715

GGUUGGACAGUCCCCUGCCUCCCCCAACCCACAGUGACGUUGAGGAGUGAA

>N2716

CAAUGUGACCCAGGGCCCAAAGUAGAAUGCCCUGAGAAUGCAGCCCCAGUG

>N2717

CAUAAAGAAAAGGACUGAUAAGUCCAAAUCCACUAAUGUUAAAACUUUCUC

>N2718

AACGAAGGAAGGAAAUGAAAAGAUAAGCCACAUAAUCUGAUAAAAUAUUAA

>N2719

CCAAGUGUUGGUUAGGAUGUGGAGCAAUGGGAACUCUUGUACACUGCUGGA

>N2720

ACUCCACCAUUGCAUUUCAAGGUAUACACCCAGAUACACGAAUUACCAAGG

>N2721

AGAAAGGAUUAUAAAUACAUUGUUCAUAAUAUCCCCAAACUAGAAACAAGC

>N2722

UAAGACCCUGUUUAUAGAAAGUUCAAAACAGACGAAACCAACUUAUAUCCG

>N2723

AUGGCUCAUUUCUGGAGAGAGGGAGAAAGGGAGGGGUUGUUAUUAGGGGAU

>N2724

AGAACCCAAAAUUCUGUCAAAGCCAAAUAUCUUUAGACAAAUCUUGAGGAA

>N2725

GAAAUUCCAUCAAAGUGGUGCAGUGACCUGAGUAGGAGUUUACAGAUUAUG

>N2726

UAACAAUGUCCCUCUUUUAGACUCUAAAUAGGAUUCAAGGGAUCCCAGUAG

>N2727

CCCAAAAUCAAGAACCACAACUUACAAAGAGAAAUUAUAUGCUGAGCAGCU

>N2728

CAGAAGCAAGGUGAAGGGGCGAGUGAGCAAGAAGUGAGAUAUGAAGCAGAG

>N2729

CAAAACAAAAGUGAGAUUUUAAAAAAGUGAGUAAGGAGCCCACAUGGCCGC

>N2730

CUUAAAAUGUUCCCGUAUGGUAAUUAGCUACUUAAGGUUCAUGCAUAUGGC

>N2731

CAGGCAGUCUUGGCCACAGAAGAGAAAAAAUAUAUAAAAGAAUUCAAAGUU

>N2732

ACGUUGAUCUCAUUUCGCAGAGGUCAGUCCACUCUCCCCUCUCUGGGAGUG

>N2733

CGUCAUACACUUUUGCUGCUUUGCUAUCUGUGUGUAUCUCGUCCAAUUCUG

>N2734

CUUGGUUCUUUAACUUGACCUUGGGAAGACAGGGCCAGCCAGCAAGGUGGC

>N2735

UGCCUGGUAGGCAUGGAAGGAGGGCAAGUGUUGGUAGCCAUGGUAACUCGC

>N2736

UGCCCUAGCAAAUGGGUCUUUUCGAACAUUACCCACCUCAACGUAAGUCCA

>N2737

AAUUGCCGGACGGCAGGUGGCACAGAAUUCCUUUGGAACUCCAAAUUCUUG

>N2738

UCUCAGAAUAGAGGGGUGGAAACCAAAGCGGGUCUGCUUGAGAGGAUUCAU

>N2739

CAAAAGCCACCGAUUUGGUCAGGUGACUGUGUCCUGCAAGUCUCCCCCGAC

>N2740

GCAUGGCGGAGAGAGGAAGAUGAUCAAGGGAGGCAUCAGAGAGUCAGCAGG

>N2741

UCCUGGAAUUUGUUGAGUUUGAGGUACCUGUGGUCUAGGCAAGGAAAAAUU

>N2742

AAAUGGGGAUAUGGGUCUGAAGCUCAGAAUUGUCUAGACUAAAGGCAUGCA

>N2743

AUUUUCAUGAUAGGGGAGGAAUGGUAUAAGCAAGGAGUAUGCUAAGCUUCC

>N2744

UUUCGGUUGAGAAGUAGGUCAUUGAACAAUAAUGAAAAUGCUGAACAUUUC

>N2745

AGAAUUAACUGCCCUUUUCCUACAAACUUUCAGCUUUCAAAUCUGUACCUC

>N2746

GACUAAAGCUGAAUUGUAAAGGACUAAAGCCCUAACUUCUCUUCGAAUGAU

>N2747

UGAAAUUUUUUCCCAUUGUAUGUAAAAUUAACAUACAACGAAAUGCACAAA

>N2748

AGUGUAUUAGUAUCCUUGCUAUGGUAACAAAUCACCACAUACUUAGUGUCC

>N2749

UACAUUUAUUCUCUUGGAAGUCCAAAAUCAGUCUGGCUGGGCCAAAGUCAA

>N2750

GGGCUGGCUUCUUCUGGUAUCUCUGAAGGGACAAUCUGUUUCCUUGCCUUA

>N2751

UCCUUUUUGCCAUGUGAAACAACAUAUUCACAGCUUCCAGGAAUCAGGAUG

>N2752

GGAGAGCAAUUAUUCAGCCUACCACAUGCACAUCUAAUGAGUUUUUACAAU

>N2753

CUGAAGACUCAUCCAUGCUGCGUGUAUCAGUCUCAGUCUGUUCCUUUUGAC

>N2754

CACCCAAGUACAAUACAUUUUGUUAAGUAUGGUCACCCUCCUCUGCUAUCU

>N2755

CUACAGGGGAAAUUCCCUACUGGAGACAGCUUUGCUGAAUGAGCUUGACUU

>N2756

GCUGGGGCUUAUUGUGGUGACUAUGAGGUCACCAAGGUUGCCAAAUCCCAU

>N2757

CCCAGUCUCUGUUCUUUAUCUUUCCACUCUCUACCUCCAUCUCUGUUCUCU

>N2758

UGCCUUUUUGUGCCUGACUUAUUUCACUUAAGAUAAUGGCCUCCAGUUCCU

>N2759

UGCAAAUGACAUGUUUCAUUCAUAUAGGCUUGUCAUAUAUGGUCUUUGUUC

>N2760

AUUUUUAUCAGAAAUUUUAUCAAAUACUGUUUCUGCAUUUAUUGAGAUGAA

>N2761

ACUGCAACUUCCACCUCCCUGGUUCAAGCAAUUCCCCUGCCUCAGCCUCCC

>N2762

AGUUGUUGGAUAGAAUGUUCUGCAAAUUGUUAGGUCCCUUUGUCUAUAGUU

>N2763

ACUAGCAUCUGUUCCCCUCACAAAGAAUCAGUCACCCUUGUCAGCCUCAUC

>N2764

UACUUGUUUACUUGACCCUUACUUGAGGACAUCUGACUCUACUCACUCUAU

>N2765

CCAGGGCACAGCCAGGGCAUUUCAUAUCUGUGUCUGCACAGCAAGGGCAGC

>N2766

UCACUUCUUCAUAUGUAAUCUGAUGACCCUCACAGGGUCAUUGUGAGGAGC

>N2767

UUUGCUUCACUGUGAAACUGGCUCCAACAUAGACCUUGAGAUUGCUAAAUC

>N2768

AGAACUUGAGCUUCUCGAACACUAUAUUUUGGUUAUAGACUUUUUUAUUGA

>N2769

AGACAAAAAUUACCCAUAAUUUCACACCCCAGAGAUAACCACUGUUAAUAA

>N2770

UCUUUUCACUAGUGAUAGAUAGAUAAACAAAUAAGUAGACUGACAUACAAA

>N2771

ACAUUUUUCCCACAAGUGGAAAAACACCAAUAAUUAGAUUUCUUGGUCCAC

>N2772

UGAUCCGCCUGCCUCAGUCUCCCAAAGUGCUAGGAUUACAGGCAUGAGCCG

>N2773

UUGAAGAAAACUCCUGAACCUGCCAACUGGGAAGGUCCCAAGAGUCCAGAA

>N2774

UCAGAGGCAGCAAAGGCAGCAAAUGAGAUGGUGAAUUACAACCCAAAGCCC

>N2775

CUGGGAGGCUGAAACGCACACAUUUAUUUAUCACAGUUCUGGACGCUGGAA

>N2776

CUUCAACAUAUGCAUUUUGGGGGACACAGUUCAGCCCAUGACAGCGCCUCA

>N2777

AACUUUUAAAAGUAAGGCAUAUCACAACACCAAUGACAUUCUUCGCAGAAU

>N2778

AUUCCUGGAAUUUGUAUGGAAACACACACAAAAAAGAAACCCAAAAAGCUU

>N2779

ACUAAAACAGCGUGGUACUGGCAUAAAAACAGAUGCAUAAACCAAUGGAAA

>N2780

ACAGUGGAAAAAGGACAAUUGCUUCAAUAGAAUGGUGUUAGAAAAACUGGC

>N2781

CAGAAAAAUGAAAUUACCCCCUUAUAUCUCACCAUGUUCAACUCAAAAUAA

>N2782

UAAAUCCAAGACCCAAAACUAUUAAACUACUAGAAGAAGACAUAGGGGGAU

>N2783

CACUGGUCUGGGCAAUAAUUUUUUUAUAAAACGUUAAAAGCAUGGAUAACA

>N2784

ACAUCUGACAAGCAGUUAAUAUCCAAAAUAUAUAAGGAAACCAAACAACUU

>N2785

UAAUAAUAAUAAUAAUAAUAAUAAUAAUAAUAACCCAGUGAAAAAUUGGCA

>N2786

GUAGACAUUUUGCAAAAGAAGACAGACAAAUGGCCAACAGGUGUAUGAAGA

>N2787

AUCACUAAUCAUCAGGAAAAUGCAAAUAAAAAACCACAAGGAGAUAUCACC

>N2788

GGCUAUUAUCAAAAAGACAAAAGAUAACACUUGCCAUCAAAGCUGUGGAGU

>N2789

UUUAUAUAUUGUUGCUGGGAAUGCAAAUUAGUACAGCCAUUAUGGAAAACA

>N2790

UCCUCAAAAAAUUAAAAACAGAACUACAAUAUGAUCCAGCAACCCCACUAU

>N2791

CUAUGCACAAUAACCAAAAUAUGGUAUCAACCUAAAUGUCCAUCAAAGGAA

>N2792

AAAAAAAUGAGUUGUAUAUAUACACAUGGAAUACCACUUAUCCAUAAAAAU

>N2793

AGAAAGAUAAAUACCGCAUGAUCUCACUCAUCAAGAUUCAUGUGGAAUCUC

>N2794

AAGUUAUGUCAAGUAAAAAAAAAAAAAAGAUGAAAAAAAAAAAAGCAAAGC

>N2795

CACUGCCUGGGUGAUUCCAACAUGCAGCCAGCGGGGAGAACACCCCACAAA

>N2796

CAGGCUGUGGCUGCCUCCCUUAGAAAACAAAACCUUUAUAGAACAGGGACC

>N2797

ACGUCCAUAACAAUGACAACCUGCUAAGGCACUUAGAUGCUCAUGCUGGGC

>N2798

CAUUUAAAACGGGGUCUGUAUCUGGAGCAGUGCAGUGUAUGUUGGCAUGGG

>N2799

ACUCCUCUGUAUGAGCUCCAUAAGUACAAUCAGAUACACUAUCUCAGUUUC

>N2800

UAAGAAGAGACUUCCAGUGCAUAGAAUUGGCAUUUAUACAGGACAGGUUCU

>N2801

UACUCAGGGUCUAAUAGUUGUUUGAAGAUUUUUAAAGUUAUGUAAAAAUAA

>N2802

UUGAGGCCAGGAGUUCGAGACCAGCAUGGCCAACAUGGUGAAACCCCAUCC

>N2803

CAUCUCUAGGAUUGAACAAGUCCCCAGCACAGGAGUGCCAGGGCCAUCAGU

>N2804

CAGUUCACACAGACUGAAACUUGUAAAGCCAAACUUUACCAAAUCCUAGUA

>N2805

UCCAAGCCCAGCCCCUCCCAGCCCUAAGACCCCAAUUCUGGGGCUGGCUAC

>N2806

CCUGAUGCCUGCUGAUACCUGGCCUAGCAGCUCUCAUCCUAUCAACCUAUU

>N2807

CCUUGUCCAUGUCAUCUGUUUAUUAAUUCACUCCAAAACCCAAAAAUGAGU

>N2808

UAGUUUAUAUUUUAUCCAAUAGGCAACUGGAACCCACCAGAGGGAGGAAGA

>N2809

AGAUCUAUCUUCCAGCAAAAGCAUGAAGGGCAGAUUGGAGGUGCUCAGGAC

>N2810

GCGUCUGGAGGCAAUUGCAGUUUUCAGGCCAAAUCACAAGGGCUUAAAUGG

>N2811

AAAUCCAGAGCUAUUUAGGAAUUAGAACAGGAAGAACUUCAAAACCAGUUG

>N2812

UAAUUUCUUACCAGUGGUAUCUGUGAUAAAUUACAGAUUGACAGGGCCACU

>N2813

GAAAAUAACACUUAGAAAGAAAAAUAAGAGUUUUGAAAAGAGAGGGGGGUG

>N2814

CUUUAGGGAAACUGGCUUGUUACUAAGACAGAUGUGAACUAAACAAGACAC

>N2815

UCAUAUCCAUUAUUGUGCCUGUUUCACAAAUGAGAAAACUGAGUGAGGUUU

>N2816

CUUUUUUUUUUUUUUUUUUUUUGAGACAGAGUCUUGCUCUGUCGCCCAGGA

>N2817

AACAUCCCCACCCCUACACAAGAACACCAAGUUUGACAACUAUCUACUCAA

>N2818

UCCCCUGGCAGUGGCUACAUGGCACAGAGAAUCUGUAUAUACACUUGAGAA

>N2819

CCAUGACUGUGAGGCUUUGCAUUGAACUCAGUGCUGCUAUGUCACAGCGGG

>N2820

GAGCUAUACUCAGCUGACACCACGGAGGGAACAUGUGGACCAGCCCUGGCU

>N2821

CACCCAGCCCAGCAGUCAGAGCAUGAGUUCCAGCAAGCCUCACCCCCAUUU

>N2822

UUUGGGACCCUAAGUGAACUUGAGGAGCAGUCUACGCAACAAGGACUGCAC

>N2823

AGACCUAGUGCUGAGCUGGGCUUGGAGUUAGUGAACUGGUGGGGCACAUGA

>N2824

GCAUUUUGGAUACCAGCUCAGCCACAGUAUGUUAGGGCACUGAUAAAGGCC

>N2825

CAUUCCAGGCCCUAGCUCCCAGACAACAUUUCUGGACACACCAUGGGCCAC

>N2826

UGGUCCUUAACUGAGCAUCAGUGGUAGCCUGGCAGUACUCCCCACGGGCCU

>N2827

GGAUGUAAGCCUGGCUGGCGUCACCACCUUUGCUGAUUGUAGAGCCCUAGA

>N2828

AAUAGGCAGUAGCCAGGUAGUGGUUACAGCAGGCCUUGGGCAAGACCCAGA

>N2829

UUCCCAAGGAGGACAGGCUCAAACAAACUCAGACUGCAAACAUGGCAAUAG

>N2830

UUUUAAUGCUCGAACACUGACGAACAUCCACAAGCAUCAAAGCCAUCCAGU

>N2831

UUAAUAGCAGAACUGCUCAAGCAGAAGAAAGAAUUAGUGAGAAUAGACAGC

>N2832

UUAUAUCGCCAGAAGAGACAAAAAAAAUUUUUUUUUAAUGAAGCACACCUA

>N2833

AAAAUAGCCUCAAAAGGGCAGAUCUAAGAGUUGUUGGCCUUAAAGAGGAGG

>N2834

UAAAAGCAGCAAGAGAAAAAAAAAAAAAAAACAAGUAACAUACAAUGGAGC

>N2835

UGUCCUACAAAAAAAUGCUAAAGGGAGUACUUCAAUCAGAAAGAAAAGGAC

>N2836

AAUAAGAAAUUAUCUAAAGAUACAAAACUCACGGGUAUUAGUAAGUACACC

>N2837

CUUGAGCCCCAGAGGCGGAGGUGGCAGUGAGCUGAGAUCACGCCAUGCACG

>N2838

AGUAGUGUUAAGUUGUUAUCAACUUAAUAGAUUGUAAGAGUAUUUGCAAGA

>N2839

UCUCAAAUCAAAAAACAUACAACAGAUGCACAAAAAUUAAAAAGCAAGAAA

>N2840

CCACCAGAGAAAAUCACCGUCUCUAAAAGGAAAAGUUAACCGCUUUUCCCA

>N2841

UAACCAUGCUGCUAUAUUUCCUUCCAAAAUUGUGCUCAGCAAAGGGCUGCG

>N2842

ACACAAAUUCUUUAUUUCAAAGAUCAGCAACAUAUUUUAACUUCUGCUUCA

>N2843

UCAAAGUCCAGAAGUGGGCUCAGCCAGCAGCCAGCCAUGCUCACACACUGA

>N2844

CUUGCCCCCCAAGACUAUUGCAACCACCCCCCGGCCUGCUCUCUGCUCCUC

>N2845

GCCUGAAAUGCCCCCGCCACCAGGCAUCCUUGUGGCCCACUCCUUCACCAA

>N2846

AGAGGAGUCCCCUUUCUGAAAGGUCAGGCCCUGCCCCCACCACAUGCCCCA

>N2847

CCCUGCGCCCUGCAUCUCCCCAGGGAAGUAGAAGCUCAGAGCUGGGGCAGA

>N2848

GGUCACGUAGGCACACUCUCUGCCCACCACAACUAACAAGAUUCCAGAUUU

>N2849

UGGCAGAAGCAGGGCUAGCACCUGGAGAUGAACGUUCUCCCCUCUCUGGGG

>N2850

AUUCACAUAAAGAUCUAAUCGAAUGAGCUUCCCUGGUGUACCCCCAGUAGC

>N2851

CCUUAGGCCUGGACCUCCUUAAGAAAGGCAAGCAGCGAACAACUGACAGGU

>N2852

CAACACAGCAUGACCCCGUCUCUACAAAUAUAUAUAUGUAUAUAACAACAG

>N2853

CAGAGAUUGGAGUGACAUGGCCACAAGCCAAGGCACUGAGGGACACCAAGA

>N2854

ACACAGAAGCUGGAAGAGGAGAGGAAGGACCCUCUUCUUGAGCCUCCAAAA

>N2855

AUCAAUAACCCCUUCCCACAACCACACAUAAGGUCCCCGUUUCCCCACAUC

>N2856

CUUGGCAUCAUCCAGCUUUCCCAUGAAAAAUUAUAUCUCGUUGUUGAAUGA

>N2857

UUUAUUGAGACACACCUUACAAUUCACCCAUUUAAAGUGUAUAAUUCAAUC

>N2858

CUAAUGUGCUUCUCAUCUGCUUGAGAGAUUUACCUACCCCACACGUUUCAA

>N2859

UAGACCACAUUUUGUUGACUCAUUUAUCAAUGCAGAGACAUAUGAGUCGUA

>N2860

GGCUAUUGUGAAUAACACUGCAGUAAACACUCUGGUACAGGUUUCCCCAUU

>N2861

GAGGUCACAUCCAAGAAUUAGAUGUAAAGUCAAGCAGAUUUACUCCUAUGU

>N2862

AGAGUUUUACAGUUUUAGCUCUUACAUGUAUGUCUUUGGUCCCUUUCGAGA

>N2863

UGACUAAGUGUGUUCACCUUCCUUCAUCUAGCUGCCAGUGCACACUGAAAU

>N2864

GCGGGCAGCAGGAAGGAUGAUGAUGAGAAAUACAAAACCCCUAUAACCACU

>N2865

CUGCCCUGGUCCAUCUGUCAGCUUCAGGUAAGACUACCGGCUAGGAAAUGC

>N2866

GGGCGGGGUCUGAUUGACUUCAAGAAGGAGUCAGAGAUCAGCUUAAGGGCA

>N2867

GCAGAGCGAACUGGGAGCAGAGCACACAGGUGCGUUCUGAUGGGUAUUCGA

>N2868

AAGGGGCUGACCUGGAGGACAGAAAAGCAGUGGAAGGAGGGCACAGAGUGG

>N2869

GGUGCCCGGGUGCUUGGUGGCAGGCAACAGCCGGCCAGGGUGGAGUGGGGA

>N2870

ACCCCCACUGCCACUGCACAUGGGGACAGAGGUUUGUCCUGGGGAAAGCAC

>N2871

GAGACCCAGCCGUGAAAGGACAUGGAGGUACCCUAAAUGCACACUGCCAAG

>N2872

CAAUCUGAAAAGGCUGCAGACUGUAAGAUUCCAAAUACAGGACGUUCUGGC

>N2873

UAGACUUUAUAAAGUUUGUUAAAAGACUUUUAAUAGACUUUUUAAAGACUA

>N2874

UCAGGAAUUCAAGACCAGCCUGACCAACACGGUGAAACACCAUCUCUACUG

>N2875

GCAACUAGAGGGACAUCCCCAUGAGAGGCCAUGACCAUGGAGCAAAACGAG

>N2876

GAGCCCUCGGGGAGAGGGGCAUGGGAAAGGGAGAGAAGGACAUCUCAGAUA

>N2877

GUGGAGAAGUAUGUUAGUUGGGGCCAAAAACAGCAUUCCUCGGCAGUGCGA

>N2878

GCAGCUGAGCUUCUCUGGAGGAAAAAAGCCACGGGAAAACAGGGCCUCUAU

>N2879

AUCUGUAGCAACAGCGCAACAGGCAAGAGUAACAGUCUCCCAGAGACUCCC

>N2880

AUGGGAAGACAGAGCUGUGGCCACCACGUCUCCCAGCCCUGCAGGAGGCUC

>N2881

AUUUCUCUCCACAACCCUAGGAGCUAAGCACUGUUGUCAUUAUUUUCAUUG

>N2882

CUAAAUUUCAAAAUCUGUCUACCUAAAAGGUAUUUCGUUGUGUCAGAAAUA

>N2883

CAGCCUGGCCUCUGUGACUCACGGCACCCCUGGCUGAGAUAAUACACAAAC

>N2884

UCCCCUGAGUAAGGAAGGCUGCAGGACUGAAGGACAUUUACAGAGGAGUGU

>N2885

GAAAUAAAUGGAUGGAUGCACAGGCAGCGGCCCGACGGCGCUGGGGACUAA

>N2886

UUUCUCAUCGAGAUGCUGCUGCCCAAGAAGUACCAUGUGCUGAGGUUUCUG

>N2887

CUUCUCAUUCCAAGACUGCCAUGACAGAUGCCUGGCCUUCACCGAUGUGGG

>N2888

CGUGGAGCAGGUGUGGUACAACGGGAAGUUCUAUGGGAGCCCAGAGGAACC

>N2889

GGCAGUGGCGCUGUAUGGAGGACACACACCUGCAGGCAUGCAGACCAAGUA

>N2890

GGCUGGGGCCUGGGCAGCGUCACUCAUGAGUUAGCCCCCGGCAUCGACUGC

>N2891

UGUCUAGCGCUUUGAGAUGGAGUGUAGUAUUGUCUGGGAAUAUUCAGGGUC

>N2892

CCCCCAGGAGAACCCCUGGGGCCACAAGCGCACGUACCGCCUGCAGAUCCG

>N2893

GACCAGGUGCUGCCCCCAGGCUGGCAGGAGGAGCAGGCCAUCACCUGGGCC

>N2894

GACCCAGGGGGCCUGGGGGAGGGUCAGUGGCUUCCCUCAGUGUGUGUGUCA

>N2895

GAUACUGUGUUCUUACAAUAAAGUAAGCUAGAGAAAAGAAAAUGUUAUUAA

>N2896

GCCGGGCACGGUGGCUCACGCCUGUAAUCCCAGCACUUUGGGAAGCUGAGG

>N2897

GGCAGGAGAAUGGCGUGAACCCGGGAGGCGGAGCUUGCAGUGAGCCGAGAA

>N2898

GGAAAAGAAAAUAUAUCUACAAUCCAUUAAGUGGGAGUGGCUCAUCCCAAA

>N2899

AGUUCGAGACCAGCCUGGCCAACACAGCGAAACCCUGUCUCUACUAAAGAG

>N2900

GCCGGGUGUGGUCGUGGGCGCCUGUAAUCCCAGCUACUCGGGAGGCUGAGA

>N2901

GGUGACAGAGCAAAACUCCAUCAAAAAAAAAGAAAAAGAAAAGAAAAGAAG

>N2902

GAGAGAAAGAGGACCCAUGCAGCUCACACCUGUGUUGUGCAAGGGUUGCCA

>N2903

CGGUUAACAGCAGCCCGUCCUGCUGACUCCCAGGACAGAUGAUCUGUCAUC

>N2904

GCCUUGCUGUGUGGACGGCAAGUUCAGAGGUCACAACAGAGCUGCUCAUCU

>N2905

UGCUCCGGCCAUUCAACUUCUUCCCAGAGGACCCCUCCCUGGCAUCCAGAC

>N2906

CGUGUGGCCUCGGGACAACGGCCCCAACUACGUCCAGCGCUGGAUCCCUGU

>N2907

UGCUCGAUGCCUCCCCCUUUUAGCUACAAUGGGACCUAUAGACCUGUGUGC

>N2908

GUUCCUCCCCCAGUUCCUCCCAGGAAGCCCAGGAGCCUCACUGGGGCAGAA

>N2909

GAAGAUCUCUUGAGCUGAGAAGCCGAGAUCACGCCACUGCACUCCAGCCUG

>N2910

ACACAGUGAGACCCUGUCUCAAAAAAAAAAAAAAAAAAAUCUGAGAGUGAG

>N2911

UGGGGAGGCUGCAAGGGAAUUUCCCAGUGUCAGUGACAGGCUUGCAAAAUU

>N2912

CCCAACCUCAGGGGGUUUAUCCCCAAGAUCCACCUGUAAACACAGGAACGG

>N2913

ACAGGGAUAGGAGCAGGCUUUCUUCAUGGAAACCUUUCUAUUGAGUUGUUA

>N2914

CCCGGCCAUGGCACAUGGCCCAGGAAAGCACUAACUGCAGAAACCCAGGUU

>N2915

GUGGAUCUGUUACCUUACAUGCACUAGGAAGGAAUUCUGUGGGCAUGAUUU

>N2916

UACAAUGGUGUUGUUUGAGGCCACUAAGUUUGUGGUACUGUGUUUAUAGCA

>N2917

GGGGCAGGGUCCUGGAUCUUUUCAAACUGGACAAUUCAAUAGAACAGAUUU

>N2918

CACCCACCAUACCAAGGGCAGCUAGAAGACCAAAGGCAUUGCUUACUUUAU

>N2919

UGAGUCUUUGCACUUUGCCCCACAUAAUUAUUCUGGCCAUUCCUGUGGCCU

>N2920

ACUGUUCCUCCUUCCCUUAAGCAGAAUGUAACUGGCCUGGGACACAAAGAC

>N2921

AAGUCCCCGACCUCAAGAUGUCCACAGUCACAGGGGUGACAGCCCAGAGCG

>N2922

UCCUCUGUUUCCUAAUAAAAACUGGAAGUACUCAGAAGGCUGAAGCAGGAA

>N2923

UGCAAGCCUGGUCUAACCCUCAAGUAGGCAGAGCUUGGUCCCCGUGGACUA

>N2924

CUGCCUGGGUGGUGAAUUCUCCCUGAGCACCACGUACACUCACGUGAGAGU

>N2925

GCCUGGCUCACCUCUGAAAACUCGGAUGCCCCAAGAAUCCCUGAGUGGAGA

>N2926

GUAAUGCAUUGCAGCAAGAGAGCACAUAAUCUUCUCCUCAAAGCAUGUUCA

>N2927

AUUCAAGCUUAUGCAAAUUAGAGCAAUUGCACACUUAACCCUAAGUUAUCA

>N2928

CACUUCAAGGUGGCUACAUCCCAUAACCUGGAAUUUCUAAUGGCUAAGGCC

>N2929

CAUUACUGGGUGUAUGCCCAAAGGAAUAUAAAUCAUUCUAUUAUAAAGACU

>N2930

UAUGUUCACUGCAGCACUAUUCACAAUAGAAAAGACAUGGAAUUGGCCAGC

>N2931

CACACCUGUAAUCCCAGUACUUUGGAGGGGCCAAGGCGGGCAGAUCACAAU

>N2932

UGAGACCAGCCUGGCCAAUAUGGUAAAACCCCAUCUCUACUAAAAAUAUAU

>N2933

AUGUUCUCACUUAUAAGUGGGAGCUAAAUGAUGAGAACGCAUAGAGGAGAU

>N2934

UACUAAGCUUAAUACGUGGGUGAUGAAAUAAUCUGUGUAACAAACCCCCAG

>N2935

UGCCUGAGAGCUGAUUCCACGUUACAGUUAUCCAUUGCUACAUAACAAGCC

>N2936

AGUGGCACAAAACAACAACUAUUUUAUUAUGCUCAUGAACUCAAUGGGACC

>N2937

AUGCCUUUUAUGACCUAACUUCAGAAAUCCCAUGACUAUUUCUGCCAUACC

>N2938

GUGGUCACCAUUGCACCCAAAUGGAAAGGAAGAAGACAUAGACCCCACAUA

>N2939

GACCAGCAAAAAAGUGAGAGGGGCCAUGGUUCAGAACUGCCACUCCAUGAG

>N2940

CUCCUUAUAUUCUGCUGUCAAGGUAAAAAUGUUCUCCUUUCACCACAAUAA

>N2941

AUAACAACGCCAUAGUGAAUUCGAAACCUCUGCUGUGGUGUGGGAGGAGAA

>N2942

GUAUGUCCUGUUCUUGUCAUUUCAGAAAGGCACCAGCCUUAUUAUCAAAUU

>N2943

CUCCCUGUGGCCCGACCCUGUUGGGAAGGGGGAAAUUGUCAAAAACCACAU

>N2944

CCACACUGCAACAGGGCUCCUUCACAACUAGGAGGAAAAGCCCCAGGCCAA

>N2945

CCAGCAGUGUCAACCCUGGCUGUGCACCAGAGUCACCUAGAGAGGCUUCUC

>N2946

GUGUUGUGCUGCUUCGUGCCACACCAGUCAUAUCAGAAUAUCCAAGGGUGA

>N2947

AGGGGGGAACUGAGAGCACAGCCUGAAUAUUCUGAGUUCCCUGUAGCUGCU

>N2948

GAUUAGGACUCAAAUAUAUCUUUUGAGGGGGUCACAAUUUAGCUCACAAUG

>N2949

CUAUAUCUCUACAUUUCAGGGAAAGAAAUGGACACAGAAUUCUAGACAGUU

>N2950

CUCUAUUAUAUUUCCCUCCUAAUGUAUCUUCCUGUUCUGCAGCAACUAGAC

>N2951

GCCUAGAAAGGCAGGGACUGGCAGAAUGAUCAUGCACUUGUGUGUGCUGCG

>N2952

UCGCUUAGCCCAGGAGUUUGAGAUCAACCUGAUCAAAAUAAAGAGAACUUA

>N2953

AAAAAAAAUAAAAAGGUUUUUUUUAAUUAGCCAGGCAUGGUUGUGCACACA

>N2954

ACUGCUUGGGAGGCUGAGGCAGAGAAUGGCUUGAGCCCAGAAGUUCAAGGA

>N2955

UCAAUAGCAAAAUAGACUUUCAACAAACAGUGCUGGGACAACUGGAUAUAU

>N2956

GAAUGAAACCGGAACCCUAGUAUUUAUCAUAUAUAAAAAUUAUCUCAAAAA

>N2957

CCUAAAUGUAACAGCUAAAACUCUUAGAAUAAAAUGUAAGCAUAAAUCUUA

>N2958

AACCCAAUUUAAAAAUGGGCAAAGGAUCUGAACAGACAUUUCUCCAAGGAU

>N2959

CAAGUGUUGGUGAGGAUGUGGUGAAAUUAUAAACUUCAUACAUUGCUGGUA

>N2960

AGAAUACAGAAAUUUCACCCCAGGUAUGUGUCCAAGAAAUAUGAAAAUGUU

>N2961

AUUCUGCAAUAAGGAAAAAUGAAAUAUUGAUACAUGCUACAACAUGGAUGC

>N2962

GGGAGGAUUGGGGAGAAUUGGAUAUAGAGUUUCUUUUAGAGGUAAUGAAAU

>N2963

UUGGUUGUGACAAUAGAUUUAAUAAAUCUAUAAAUUGUAUAGUAUGUGAAA

>N2964

GUCACAGAAUAAUAAACAUUGUAGAAUUCUAUUUAUAUAAACAAAAAACAA

>N2965

GUGUUUUAAUGUUUUAUGAACAUUUACUCAUAUAUUGUUUUUGUAAUCUUU

>N2966

AUUACAAAAAUGUAGCCAUCCUAAUAUAUAACAAAAUAGAAUUCAUAGCCA

>N2967

AAAGAGAUAGAGAUAUGUUAUAUCUAUAGAUGAAAAGAAACUCAAGAAGCA

>N2968

GACAAUUGGAAAAUUAGAACACUGGAUAUUUGAUGGUAUUAAGAAAUUAUA

>N2969

CUGGAUGAUGGAUUUGAGAGGGUUCAUUAUAUCCUUCUCUCUACUUCUGUG

>N2970

UAUAUGCAUCUAACAACAUAAUCUCAAGCAAGAAGAAGAAAACCAACAACA

>N2971

UCUCAAAUUCUAAAGAAUUGAAAGCAUACAGACCAUGUUCUGAGACCAUAG

>N2972

UAUCCAUCCUCCUCAAAUGUUUUUUAUAAUUCCAAUCCCAAUCAAAAUCCA

>N2973

UUUUAGGGGUUAAUGAAUUUAUUCUAAAACAUAUGAAAAAUGGGUUCACAU

>N2974

CAUUUUUCAGAAAGAAGGACAAAGAAGUGAGACUGGUCCCAUCAAAUAUUU

>N2975

UAAAGCCAUGGAAAUUUUUUUAAUCAUGGUAUUGGCUCAAGAGUAGACCAA

>N2976

CUAAAAAUCAUAAACCACAAGGUAAAAAAAUAUGUAGAUUUGAUUUCAUUC

>N2977

UCCUAUGACUCAGAAAGCCCACUACAAAUUAUACCUAAUUGAAAGAAAUUA

>N2978

AAGCCACAUUGAGAUGUCUCUACACACAAAUUAGGAAAGUUAAAUUUAAAA

>N2979

UGUUCAUAGUAGCUUUACUUAUAACAGCAUAGGAAACAGCCCAGAUGUCUA

>N2980

AACUCCAAAUGUCACAUGCAGUAUGAUUUCACUCGGAUAGCAUUCCCAAAA

>N2981

UCUUGACUGAGGCGCUGCUUACACAAAUCCACACCUGACAAAAACACCUGA

>N2982

AGAGAGAGAGAUGAAUGCAGGUUUAAAAGCAGUGAACACCGAAUAACUUCG

>N2983

CCACUAUGGAGAUUAAGAUUUCAACACAUGAAUUUGAGGGAGACAUACUCA

>N2984

CUCUUGUGAGUUUAUAAUUACGCCAAAACUAAAAAGCUUUCUUAAAAAUAC

>N2985

UUCUUUCAAAGGAAAUAUGGCAUGAACAAAUAUUGAGACAGGGAUGUUUCA

>N2986

ACUCCCCUUAUUCAAGGGUUGGAUCAUAAUAACUACAUCUAAGUAAUCCAA

>N2987

UCUGCAGCUUUAUAUCAAUAUACAUAAAUACAUACCAGAUAACCAUAUUUA

>N2988

AUAUAUAUUCAAGGGUAUAUGUCAAAUUUUAAAAAGAAAGGGGAUUUGGCC

>N2989

GCACUGAUUUGUUGAAGAACUAGGGAAUUGUUCUAAUUCUGCAGAUUAGUA

>N2990

GUAACAAUACAAGGCUGCCGUAACUAUGUAAUUGGCCAUAAUUGAAGACAU

>N2991

AAGUAUGGGUUUGCAAUUACCUAAUAUUCCUGUCCUAACCCAUUUCAGGAG

>N2992

GAGGUGCAUGUUACCCAUAUAACAGACCCUGGGAGUAUACCCAGGAGCAGC

>N2993

AACAGGGGGACGUGCAAGUCAGAGGAGAUGCCUGCUUCCUCCUAGAUCCCA

>N2994

UCCACACAUGUUUUAGCUUUAGUGGAAGCUCACAGAAUAACCCUCAAUUAU

>N2995

CUGUGGCAUUACCUUCUUAAAGGAAAGCUGAUGGGUUCUUUCAACAGGUUU

>N2996

CCCAGCUAACAAUUAGUGUGACCUUAGCCGAGCACAGUGUCUCACGCCUGU

>N2997

GUACCCUUAGCAUUGUUUUAUUUUUAUUUUUUAAAAUUAUCUAAACAUUGU

>N2998

UAAAUUAUUAUAAAGAAGAGCCAAGAAAAGUAUUGCUUUGAAACUUUUUAA

>N2999

ACUUAUAUCUAGUUAAAUGAUUUUUAAGCAUUUAAAGUCUCUGGAAAUUGC

>N3000

UAGAUCAUAAGAACAGAGAGCACUGAAGCUCUCCCCCGACCCCCCAAGAAG
