# Supplementary material for: iRNA-AI: identifying the adenosine to inosine editing sites in RNA sequences
Source: Oncotarget. 2016 Dec 1;8(3):4208–17. doi: 10.18632/oncotarget.13758 (PMC5354824; doi:10.18632/oncotarget.13758)
Supplement: Supplementary file 3 [file oncotarget-08-4208-s003.docx]

**Supporting Information S2. The independent dataset constructed for further testing the model and demonstrating its practical application.** It contains 3,243 experiment-confirmed A-to-I editing-site RNA samples and 3,243 experiment-confirmed non-A-to-I editing-site RNA samples, none of which occurs in the benchmark dataset of Supporting Information S1. See the main text for further explanation.

**(1) List of 3,243 A-to-I editing RNA sequences observed by experiments**

>TP1

CAGAAAGGGAGAACUCCACCUCCAUAAUCCAAUUGCCUCCCACCAGAACCC

>TP2

AAAGAUCUCAUGAGAACUCACUCAUAAUCACAAGAACAGAAAGGGAGAACU

>TP3

CUCACACUGCUAUAAAGAACUACCUAAGCCUGGGUAAUUUAUAAAGAAAAG

>TP4

UGUAUUAGAAGUAUAUAUUUAUAUUAGUCCAUUCUCACACUGCUAUAAAGA

>TP5

UUCUGUUUCUCUGGAGACCCCUAAUAUGUUGCUCAAUGGGUAUAAAGUUUC

>TP6

CUCCCAGAGAAACAGAACCAAUAGUACACACGUAUACACACACACAUUGAC

>TP7

CUUUAUACCCAUUGAGCAACAUAUUAGGGUUCUCCCAGAGAAACAGAACCA

>TP8

AGAACUGUUUCAUCUUGCAUAACUGAAACUUUAUACCCAUUGAGCAACAUA

>TP9

UAUCAUUUGAGCUAAAUGGGGCACAAAUGUCCAUCAGUAUCCCAGGAGGCC

>TP10

UUAACAGAUGCAAUGGUUUUUUAUCAUUUGAGCUAAAUGGGGCACAAAUGU

>TP11

UCAGCCUCCCAAAGUGUUAAGAUUUACAGGCACAAGCCACCAUAACUGGCA

>TP12

GUUAUGUUAUUUGCAUAUUACCAUUAUUAAAAAUAAAAAAAUUUUUAAAAG

>TP13

UUUUGUAUUUUUAGUUUUUGGGUUUAGCCACAUUGGCCAGGCUGGUCUCGA

>TP14

UGAGGCAGGAAGAUGGUUUGAGCCUAGGAGUUAAGAGGCUGCAGUGAGCUG

>TP15

AGCUGCACUACAACAGAUUCUUACCAUAUCCACAGAGGUCAGAGAUUGUAA

>TP16

GCCUCCAUCCUUUUCUCACAGUAAUAAAUUUGCAAUCUGAACCCAAGUGAA

>TP17

ACAGAGGAGCCAAUGGCAUCCUAGUAGUGUAUGAUGUGACAGAUCAGGAGU

>TP18

UCAAAUUACUUCUGAUUGGCGACUCAGGGGUUGGAAAGUCUUGCCUCCUUC

>TP19

UCUGUAAAGGCACACAUGGACUAAAAGUGAAGAUAUACCAUGCAAAUUCUA

>TP20

GACUCCCUUGCUUUAUGCACACCAAAAUUUCCAGGGUCAUCUUGUUAUCUU

>TP21

ACCGUGUUGCCCAGGCUGGACAAGAACUCCUGAGCUCAGGCAAUCCGCCCU

>TP22

GAGAUCUCAUCUCAUUGCAACCUCCACCCUCUGAGUUCAAGUAAUUCUCCU

>TP23

CUCCUGAGUAGCUGUAAUUGCAGGCAUGAGCUGCUGCACCUGGCCAGGGUG

>TP24

CACACAAGGAUUGGGAGUAUCCAGAACUGACCAUAUGUGUGGAGCUUUCCU

>TP25

GCUUAGUAAAACCCAGGGGCAGGACACCAGGAAGAAUGGGAAGGGGCUGGG

>TP26

UGUUUUCUUAUUUUUAUAUUUAUUUAUCUAUGCUUCGUUUUUCCAAAAUGU

>TP27

CCAAAAAAAAAAGAGAAAAGAGACAAAGUCCCAGUCUGUCACCCAGGCUGG

>TP28

UGCUGGAGUGCGGUGGCAUGAUUUCAGCCCCCUGCAACUUCUACCUCCCAG

>TP29

UCACUUUAGUGAAAUCCAUUUGCAAAGAACAUUGAAGAACUGCACAAGGAG

>TP30

GACAUUAAUAGACAGAACUCACUUUAGUGAAAUCCAUUUGCAAAGAACAUU

>TP31

GGCAUUUAUACCUACUGACAUUAAUAGACAGAACUCACUUUAGUGAAAUCC

>TP32

AGGGUCUCACUCUGUUACCCAGCCUAGAGUGCAGUGGUGUGAUUAUAGCUU

>TP33

AAGUUGGUUAUGAUGCUAUGGCUGGAGAUUUUGUGAAUAUGGUGGAAAAAG

>TP34

GUUGCCUCAAUAUGUUGCCUAGGCUAGUCUUAAACUGUUGGUGAAUCUUAA

>TP35

AUUUUUUUAUUUCUUUUUAUUUUUAAUAGGGAUGAAUUCUCACUCUGUUGC

>TP36

CUGGUGCAAACAUAAUUCAUCUAUCAGGUUAUUUUUAUGAUAAACAGUGUU

>TP37

UGAAACUUCAUCUUGAAUUGCCUUGAUGUUUUGUCAUGUCUUUUUAAACUU

>TP38

AUCAUGCUAAAGAAGCCAGACACAAAAGGUUGCAUUUUGUAUGAUCCCAUU

>TP39

GAGGCCAAGGUGGGAGAAUUGCUUGAGCCUAGGAGGUCAAGACUGUAGUGA

>TP40

AGUUCUAGGCUUACCUAGGCAAUAUAGUGAGACCCCCAUCUCUACAAAACA

>TP41

UUUGGGAGGCUAAGGUGAGCUCAGAAGUUCUAGGCUUACCUAGGCAAUAUA

>TP42

UUCCAAAAAUCCUUGGAAUUUUUAAAUAAUUUUUCUUGUGGCAUAUAAUUA

>TP43

UUUGAGACAGGGUGUCUGUCACUCAAGUACAGCCUCAAGCUCCCAGGCUCA

>TP44

UCAGUGUCCCUUUAGCUCGGGCUAAAGUCACACGCCACCACACCUGGCUAA

>TP45

GUCUCACUGUAUUGCCCCGGCUGGAACGUGUGGUGUAAUCUUGGCUUGCUG

>TP46

UAUACUAAAAUUAGCUGGGUGUGGUAGCGCACCCUUGUGAUCCUAGCUAUC

>TP47

UGGGAUAGAUAUAAUCUGUGAUAAUAGGCCGGGCACAGUGGCUCAUGCCUG

>TP48

GGUCUUUAUCUGGCCCGGGGUGAUAAUCCAAAUGGGAUAGAUAUAAUCUGU

>TP49

UGUGAAAUAACUGGCAGCAUAGUACAGAAAUGUAAGGUAUAUGUUUUGCUU

>TP50

UUUGAAUUUUUUGAAAUAGGGUCUCACUCUGUCCUCUAGGCUAGAGUGCAG

>TP51

GAGAAAAUACUUGUUUUCUGUAAUAAAACAAUUAGGUUGGUGCCAUGUAUG

>TP52

AUAAUACCAAAUCACAGCAAAUGUUAGAAUUAAUACAACAAAAUUUUCCUU

>TP53

GGACAGACAAUUGGCUGAAACACUGAUACUUCAAUGUGACACAUUUUUAUA

>TP54

UUCAUACUGUAUUUUGUAUCAAAACAGCAAUAAGGUUUUCUUUAUCCCUCU

>TP55

UCCCAGCUAUUUGAGAGGCUGAGGCAUGUGGAUCACUUGAACCUGGUAGGC

>TP56

CCAGGGUCAGUGGCCCAUGCCUGUAAUCCCAGCAUGAUGGGAGGCCAAGGC

>TP57

CCUGGCGUUAAAUUUCUACCAUAGAAAAAAUGUAGGCCAGGGUCAGUGGCC

>TP58

UUUUUAAAAGAGACCAGGUUUCACAAUGUUGCACAGGCAGGUCACAAACUC

>TP59

CACGACUCUCAGCUAAUUUUUGUAGAGAAAGGGUCUUGCUAUGUUGCCCAG

>TP60

CUGCUGGAACCGCCAUAGCCACCUUAGUUUCGUGGUUUUGCAAAGUAUUGG

>TP61

GUAGGGCAGGCUGUGUCAAAGUCAUACAGCCAAGGUCAGAAGUCAUGCAUC

>TP62

AAUCUGCAUUAGAACUCCUGACCUCAGGUGAUCCACUCGCCUUGGCCUCCC

>TP63

UGCCUCAAUCUACUAGGAUCUAGCUAGGAUUACAGGUGUGCGCCACCAUGC

>TP64

CACCAUGCCCCACUAAUUUUUAUUUAUUUAUUUUUUUUUUCUGAGACGGAG

>TP65

AGUACAUAACACAGUGGGCAUGUCUAGUUGUCUUUGUUUCUGCUGUUAAAA

>TP66

UUUGUUUUAUCUUAGAUUGAGUCUCACUGUAUUGCCAGUCUGGAGUGCAGU

>TP67

UACAGGUUUAUUUCUUUGUAAAAAAAAAUAAUAAUAAUUUGAAUCAUGUAA

>TP68

AUUUCAGGUAUAUAUAUAUAUAUAAAUAAAGGAAUUAAGUAAAAAUAAAAU

>TP69

GACAAUAGGUGUAUCACAUGGCAAGAGCGGGAACAAGAGAAAGAGAAGGGG

>TP70

AGAGUUCAAGACCAGUCUGGGAACAAAAUAAGACUUUGUCUCUAAAGAAAA

>TP71

CACUGUUGCACAGGCUGGAGUAUAGAGGCGUGAACAUAGCUCACUGCAGCC

>TP72

AUGGCCUCUCAAAAUGCUGGGAUUAAAGGUGUGAACCACGCACCUGCCACU

>TP73

UUUGCCAUAAAAUCCUCUUUAAAGUAAAAUUUGGCCAGGCUGUAGCCUAGA

>TP74

CCUUCCAUGGGGAAUAAAGCACUAAAAUUAGUUGUCAAUCAUGCCAAUACC

>TP75

UGAUUAGAAUGUACUUUUUAAAAAAAGUUCCUAACCCAAGAAAAAGCUAAA

>TP76

AUUCAAUAUUUACUAAGUCCCUAUUAUGUGUCAGAUACAGUUCUAGGCCCC

>TP77

AUCCCAGUGCAUGGAUAUAUAAUUUAUUUUAAUCAAUCUCACCAAUGAGCA

>TP78

UCUAUAUCCUCACCUUUAGGAUGUUAAAAAAAAUUAUCAUAAAAACAAUCU

>TP79

ACACUGAGAGGCAGGAUGUACAGUUAUAGAACAACCCUAUAUCCCCACACA

>TP80

UCCCAGAGUGGUCAGAGAGAAGGGGACCAAAAAUGCUUUGGAGAACAGUUU

>TP81

UUAUCAAAUUGGAGAUUCAUGGGAGAAGUAUGUGCAUGGUGUCAGAUACCA

>TP82

UCUCUACUGAAAAACAACAAAAACAAAAACACAAAUUAGUCGGGUGUGGUG

>TP83

UUAUUUAAUUGUAAAGGUAGAGGCCAGACGCAGUGGCUCACGCCUGUAAUU

>TP84

UCCUGGGCAGCUGGUCACUCCCCAGAGAAGCUGGGCCUUCAUGGACACAUG

>TP85

GCUCUCCCUGACUAGGGGUAGCUAGAGGCCUCCCUCCUUUGGGUUCGGUAU

>TP86

UAUUUUUUUGGUCCCCAGGGAAGCAAGGGCAGAAUUUCGUUUUGAUUUGUU

>TP87

UUCCAGUCUGCAGCAUCUCUGUCACAUGGAAACCUGAUGGGUGCCACUGUG

>TP88

AGCGCAGAUAGGAGUGAAGCCUCCUAGGCUUCCAGUCUGCAGCAUCUCUGU

>TP89

AGGGAUCCUCUCACCACAGCCUCCCAAAUAGCUGAGACUACAGGAAUGCAC

>TP90

UUCUUGUCACCCAGGCCAGAGUGCAAUGGUGUGAUCUCAGGUCACUGCAGC

>TP91

CCCACCCAAAUCUCAUCUUGAUUGUAAUUCCCACGAUUCCCACAUGUUGUG

>TP92

UCUAUCAGUACCAAUUUACUGUAUUAGUCUGUUCUCACAUUGCUAAUAAAG

>TP93

AUUUGAAAAAACUUUUUCGAGAUAGAGUCUCACUGUGUUACCCAGGCUAGA

>TP94

ACUCUCUCAUUAUAUAUACAACCUUAUGACUUUAGGGGCAUUUCAUUCUGU

>TP95

CUAAUAGAAAGAUGUCUAGUAUACUAACUACUGUUAUGCUUAACUCUCUCA

>TP96

AUUACCAGAGUGCAGGUAAAAUAGUAAUAGUAAAGAUAGGCAUCCUUAUCU

>TP97

GUGGGGGACUCAUCGCCACCUUAGAAUGGAUUGCCCUUAAUGCCAUAAGGU

>TP98

CACAUGAAAAUUUAUAUAAAUUUUCAGGGUCCAUGAAGUUUUAUUUGAAAC

>TP99

AGUACACUAUUUGUUUUCUUUUUGAAAUGGGGUCUUGCUGUGUCAUCCAGG

>TP100

AUGCUUAUUAAAAAUAUGCUAUUUUAGGCCAGGCACAGUGGCUCACCCCUG

>TP101

AAUUUGAGGGUAGGGGUAUGAGACCAUCUUGGCCAAUAUAGCAAGACCCUG

>TP102

GAAAACUAUUGUCAUUCAACUGAAGAAGAGGAAGAUAAAAGAUUGUCUUGU

>TP103

AAAUAGUGAUAAUAAUGAUGACCAUAGUAAUAAAAAAAAAUAGCUGGGCGC

>TP104

GUUGGGAUGUUGGGAAGUGGUCAGGAGUCUCUGGGAAGAGGUAAUAUGUGG

>TP105

UAGCUGGGACUACAAGUCUCACUAUAUUGCCCGGGUUGGUCUUGAACCCCU

>TP106

CAGCCUCUACCUCCUGGGCCCAAGCAGUGCUCUCGACUCAGGCAUGCCCAA

>TP107

AGGCGGAAGUUGCAGUGAGCUGUAAAUUGCACCAUUGCACUUCAGCUUGGG

>TP108

UACUGAUAAUCAGACUUCUGGCAGUAGCUCACACCUGUAACUCCAACAUUC

>TP109

UGGGCUGGUCAGGAAGUCAAGAAGCAGUAGAGCCCUAUUUACCUGGCAUCU

>TP110

GCCCAGCAAAAUGCUCAGGCCAUGUAUGCCCUUUCGUGACAAGUGCCAAGA

>TP111

UAAAAAAUUGUAGUUCAGGCGGCUAAAGUGGGAGGAUUGCUUGAGCCCAGG

>TP112

UGUAGACGGUCUCACUUUGUUGCCCAGGCUGGUCUUGAAUUCCUGAGCUCA

>TP113

AGCCUCUCGAGUAGUUGGGACUACAAGCACGCAUCACCACACCCAUCUAGU

>TP114

GAAUGCAUCUCAAAGUCAUUAUGCCAAGAGAAAGAAAGUAGAUGCAAAAGA

>TP115

GUUAGCAAGGAUGUGGAGCAACUGGAACUCCCAUAUAUUGCUGAUGGGAAG

>TP116

UAAAAUGUUAAGUCAACCAAGUGUUAGCAAGGAUGUGGAGCAACUGGAACU

>TP117

AAGAAACUCUCGCACUAUUAUCCAAAGUGACUGAACCAUUUAUACUCCCAC

>TP118

AAGUGAUUCUCUUCCCUUAGUAGCUAGGCCUACAAGUACAUGCUGCCACAC

>TP119

GAAGCUGCAUAGUUAGGGAAGCAAUAGUCCCCACAUUCCCUGGUGCGAUCA

>TP120

UUUAUUGAAGCACUUUGAUCUCUUUAUGAGGUUUUUAGCAGAAUACCACGA

>TP121

AGAAAUUCUUGGAAAAAUGUCCUUUACUGAGAAGAAUCUGAAGGCUUUAUU

>TP122

GAUUGUUUGUCAAACUUCCAGAAAUACUUGGAAAAAUGUCCUUUACUGAGA

>TP123

CGCAACAUUUGCUGCGAUUGUUUGUAAAACUUCCAGAAAUUCUUGGAAAAA

>TP124

AUGAGGUCCUCUCCUGGAAGCUCGUACCUGACAAUUACCCACCAGGUGACC

>TP125

AAGGGAGAAGAACUAAUGAAAUAAAAGAGGUCCUCUCCUGGAAGCUCGUAC

>TP126

UCUGACUCCUAGCCAGGAAGGGAGUACUGUGUUUGCUGGCUUUGAAGGGAG

>TP127

GGAGGAUGAUUGUUACUACAUUAAUAGGAGGAAACGGUUAGUGCAACUUCC

>TP128

AAAAGACUGAAGUGAAAGAAGAACCAGAGCUUCAAACAAAAAGGGAAAUGG

>TP129

CAAGGAUGAAAAAAUAAGUGAAGAAAGUGAUAUUGAAGAAAAGACUGAAGU

>TP130

UGACAGUAGUGAAGACAAGGAUGAAAAAAUAAGUGAAGAAUGUGAUAUUGA

>TP131

CCAGACACAAUGGCACAUGCCUGUAAUCCUAGCAGGUGUAUCACCUAAGGU

>TP132

AGAAAGCAGAUCUCGGCCAGACACAAUGGCACAUGCCUGUAAUCCUAGCAG

>TP133

CCUCUUCAUUUACCAACUCCUACCAAGCCACUUUUCUUGUGAAAUGUCCCA

>TP134

UUUUACUCUCUGCCCCUGUGAAAAUACACCUGCUCUCGUGGCUUCACUGGC

>TP135

GAGUAAAUCCUACAGACAAUAGACAAAUGUUUGAAAUACUUAGAACAUGGU

>TP136

UGAGUUUCAAAGAUAUCAGCUUUAUAUAAAGCUCUCCGUUCCAACCCUACC

>TP137

UGAUGCGCUUCGGGAGGCUGUUGCCAUCCUUCGUCAGCAGUAAGUCCUGCG

>TP138

AAACAAACAAACAAACAAACAAACAAACAAACAGUGAAAGGGACCAGUAGA

>TP139

UUCACUGAUCUUAAGCUCCUGAGCUAGAUUGAUCUUCCCUCCUUGGCCUCC

>TP140

UUAAAUUAAAAAAAUAAAAUUUAGUAGAGGAACUUUUUUUUUUUUUUUUAA

>TP141

ACGGCAGUUUCGAUUUACUAGACUAAAACUAGCAUAUAAAGAAGCUAAGAA

>TP142

GACUAAUAAACACCCCCAUGAUUUAAUUACCUCCCACCAGGUCCCUCCCAU

>TP143

GUUACCUCCAUGCUGUUUUCAUGACAGUGAGUGAGUUUUCAGAUCUGAUGG

>TP144

AUGGUUUGGUCAUGUCCCCACCCAAAUCUCAUCUUGAAUUGUAGCUCCCAU

>TP145

GACGUGGAUUUGCUGGGACGUGUCUAUGGUCACCGGGAUGAUGCUCUCGAU

>TP146

GGACGUGUCUGUGGUCACCGGGAUGAUGCUCUCGAUAGGAAGCAGUGGAUU

>TP147

CUCAGCAAAAUGGAGUUGCCUGGGUAGAUAGCAGCAGCAGCUCACCAGCCC

>TP148

UUAAUGAAGUAGGGCCAGGUGUGGUAGCUUAUGCCUAUAAUACCAGUGCUU

>TP149

CCCACAGUCCAGUGGACAGAGCGGGAGUGUGAGCUGGCGCUCUGGGCCUUC

>TP150

CAGGUGCAUGCUACCGCACCAGGCUAAUUUUUUUCUUCUUUUAGUUAGUAG

>TP151

AGCAGUCCUCCCACCUUAGCCUUCCAAGUAGCUGGGACCACAGGUGCAUGC

>TP152

CAGGCUGGAGUGCAGUGGUGCAGUCAUGGCUCACUACAGCCUUGGCUUUCC

>TP153

UCUGCAAACCCAUGGCUGUUCUCCCAUUUUCUUAGGCCAAUAGCUUCACCG

>TP154

CGGCUCACCGGAGCCUCAACCUCCUAGGCUCAGGCAGUUCUCCUGCCUCAG

>TP155

CCAGAAAAGGAAAAUCAUGAGUUCAAAAUCACUUGAAGUAAUUCCUUUUUU

>TP156

UGCUCACCUGUAAUCCCAGCUACUUAGAAGUCUGAGGAGAAAGGAUUGUUU

>TP157

UACAAAAAAUAGUGGUGGCGUGCUCACCUGUAAUCCCAGCUACUUAGAAGU

>TP158

CUAUUAAUAUCAAUACCUUCUGGCCAGGUGCAGUGGCUCACGCCUAUAAUC

>TP159

UGAUCCACUCAUUUCAGCCUACCAAAGUGUUGGAAUUACAGGUGUGAGCCA

>TP160

GUAGUUGAGACUGCAGGCAUGCACCAUCAGGCCCGGCUUUUUUUUUGUUUG

>TP161

ACCCCUGACACUCUCCUACUCCUCAAUUAUCUUCAGACAAAUCCCAGAUAC

>TP162

CUCAAGUAGCUCGGAUCACAGGCAUACGCAACCUCGCCCGGCUAAUUUUUU

>TP163

UUCUCUUACAGAGCAAUAACAUCGCAAAGACUGUGGAUGAGCUGCAGCAGC

>TP164

AUCAACCCAAACGCCCAUCAAUGAUAGACUGGAUAAAGAAAUUAUAGUACA

>TP165

UAGCCUCCCAAGUAGUAGGGACCACAGGCGUAUGUCACUACGCCUGGCUAA

>TP166

CCCCACCCCCCCCCUUGGAGAUAGGAUCUUGCUGUCUUCCUCCAGGCUGGG

>TP167

CACUGCCACCUCCAACUCCUGGACUAAAACAAUCCUCCCACCUCAGCCAUU

>TP168

GCUCAAAUUUUGUAUUUUUGGUACAAAAUACAAAAGUGAGCCAGACGUGGU

>TP169

CUGUUGGUUAGUAAAUAACCUCCACACAUUGGCUGUUUAAAAUUCAGCUAU

>TP170

GCUUAAUCUUUUUUGUGUAUUUAGUAGAGACAGGGUUUUGCCAUAUUGGCC

>TP171

AAUUUGGCAAGGUGCAGUGGCUCAUACCCUUAAUACCAGCACUUUGGAAGG

>TP172

AGAGAAGGGGCAAUCAGAGAUGGUUAGAGGCUGGUAGAACCUUCUGCCGCU

>TP173

CACCACUUCCCCAGCCUCCACACCAAGGCCCAGCUGCCUUCUCCCCAUGUA

>TP174

UCCCUCCCACCGAACCUUGAUUUCAAGGUGGCACCCAGCAUCCUGAAGCCA

>TP175

CCUUACAGACCAAACAGAGGAAUCGAGCUGACUUUUGUCCCUAAGCACGGG

>TP176

GGACACAGAAUUGCCUUGUUUUCUAAGAAACAUCUCCAUCUAAUGUGAACU

>TP177

CAUUCACUGAAAAAGCCAGGCUGUAAUCCCAGCACUUUGGGAGGCCAAGGC

>TP178

AGCGUGGAAGGUCGAAGCUACAGUGAGCUGUGAUGACACCACUGUAACUGU

>TP179

CUGGGUUCACGUAAUCCUCCUGAGUAGCUGGGAUUACAGGCGCCUGCCACC

>TP180

UUAGUAUAAGCUUUUCACAAACAUUAGUAUAGUCUCCCUUUUAUAAUUAAU

>TP181

AAUUAGCAUGGCGGCACACAUCUGUAAUCCUAGCUACUUGGCAGGCUGAGG

>TP182

CUCAAAAAAAAAAACAAAAAACAAAAAUUAUCCAGGUGUGGCGGUGGGCGC

>TP183

AGAAAUAUAUAAAUUAUUUCUAGCUAGGUGGCAAAACUCCCCCAGCCAUCU

>TP184

AUGCUGUACUGCCUGAUGGUAUGGAAGAGGUAACAUAGAUGUUUGUAAACA

>TP185

AGGUAACAUAGAUGUUUGUAAACAUACAGUCAUUUGAUGGCUAAGGAAGUU

>TP186

UAAACAUACAGUCAUUUGAUGGCUAAGGAAGUUUUGCCACCCAGCUAGAAG

>TP187

AGUAUGGAUGAACAUUGAAAAUGUUACACUAAUUGAAGAAGCCAGUCACAA

>TP188

UGGGCGACAGAUUGAUUGGGAUUCCAUCUCAAAAAAAAAAAAAAAGUUUGG

>TP189

UAUGUUGAGACAAUAUGUGAGACAGAGUCUCACUCUGCCCCCCAGGCUGGG

>TP190

UGGGGGUGUUAGGGAACAGGGUCUCACUCUGUCACCUAGGCUGAAGAGCAG

>TP191

AAAAAAAUGUUUUUUUGGUAGAGACAGGAACUCACUAUGUUGUACAGGCUG

>TP192

AACUGAGUAGAAGUUAGCCAGGGCAAGGCUGGAAGCGGCCUUAGCUGGACC

>TP193

GAGUGAGACCAUUUAAAAAAAAAAAAAGGCCGGGCGCGAUGGUUCACGCCU

>TP194

UAGAUGUACUUUUUUUUGGUAUUUAAGAGAUGGGAUCUUGCUGUCGCCUAG

>TP195

UACCUUGAUAAUUGAGGGUAGAAUUAAGAAAGUUUUAGGAUUAGGAAUCAU

>TP196

AUUAAAUAUCUAGGCUGGGCGUAAUAGCUGAUGCCUGUAAUCACAGCACUU

>TP197

AUCUUUUGAAAGAAGCCAGGAAACCAACACAGAAAAAGUAUUUCGUUCUCC

>TP198

CUCCCCCCACCCCACAACAGUCCCCAGAGUGUGAUGUUCCCCUUCCUGUGU

>TP199

AACCCUUAAAGUGGACAGGUAUCCAAAGUUCAUUUUCUGUGACUCAUCAAA

>TP200

UUCAGUGACAUGAUCGCCCACCGUAACCUUGAACUGCUGAACUCAAGUGAU

>TP201

AGAUGUGCCUACACCAAAACAAUUCAUAGCAGGUGUAAAGCACCUACAUCC

>TP202

AUUCAUAGCAGGUGUAAAGCACCUAAAUCCAUCAUCAUCUCCUUAUACAAG

>TP203

UGAAUAGAAGUGGUAAGAAUGGGCCAGGCAUGGUGGCUCAUGCCUCUAAUC

>TP204

UAUAUAUAGAGCAUAUAUAUGAUAUAUAUAGAGCAUAUAUAUAUAUAGAGA

>TP205

UGUGUACUUUAUUUCUGUUAUUAUUACAUUGUAAUACGUAAUGAAGUAAUU

>TP206

GUCCCUUCUGAGGGUGAUGGGAGACAGUGACAGAUCAUCAGGCAUUACAUU

>TP207

AUCAUCAGGCAUUACAUUCUCAUUAAGACCACGCAACCUAGAUCCUUCAUA

>TP208

GAGUUCGCACUUCUAUGAAUAUCUAAUGUCACCACUGAUCUGACAGUAGAC

>TP209

AAUGCGAGUGACGGGGAGCAGGUGUAAAUGCACAUGAAGUUUCGCUUGCUC

>TP210

UCUAAAACAGUGCUUCCCAACCUCCAGGCACAGGUCCGUGGCCCUUUAGGA

>TP211

UCCAGGCACAGGUCCGUGGCCCUUUAGGAACUGGGCUGCACAGCAGGAGGU

>TP212

GGCUGCACAGCAGGAGGUAAGAGGCAGGCAAGCAAGUGAAACUUCAUCUGC

>TP213

GAGGCAGGCAAGCAAGUGAAACUUCAUCUGCAUUUACAGCUGGUCUCCAUC

>TP214

UCAUCUGCAUUUACAGCUGGUCUCCAUCACCCGCAUUACCACUUGAGCUCU

>TP215

UCUCCAUCACCCGCAUUACCACUUGAGCUCUGCCUCCUGUUAGAUCAGUGG

>TP216

AUCAGUGGUGACAUUAGAUUGUCAUAGGAGCGUGAACCCUGUGGAGAACUG

>TP217

CAUAGGAGCGUGAACCCUGUGGAGAACUGUGCAUACGGGGGAUCUAGGUUG

>TP218

UGUGCAUACGGGGGAUCUAGGUUGCAUGCUCCUUAUGAGAAUAUAAUGCCU

>TP219

UAGGUUGCAUGCUCCUUAUGAGAAUAUAAUGCCUGAUGAUCUGUCACUGUC

>TP220

AGAAUAUAAUGCCUGAUGAUCUGUCACUGUCUCCCAUCACCCCCAGAUGGG

>TP221

UCUGUCACUGUCUCCCAUCACCCCCAGAUGGGACUGUCUAGUUGUAGGAAA

>TP222

CCAGAUGGGACUGUCUAGUUGUAGGAAAACAAGCUCAGGGCUCCCACUGAU

>TP223

UCAGGGCUCCCACUGAUUCUAUAUUAUGGUGAGUUGAAUAAUUAUUUAUUA

>TP224

AUCCCUGCACCCCCGGUCUGUAGAAAAAUUGUCUUUCAUGAAACCCGUCCC

>TP225

CUCUGUCUCUACUAAAAAUAGAAAAAGUCAGCCAGGAGUGGCGCAUGCCUG

>TP226

ACAAAAGAUUAAGUUUUACAGUAGUAGUAAUAGUCAUAGUGGUAACAGCAG

>TP227

AGCACUUUGGAAGGCCAAGGUGGGCAGAUCACCUGAUGUCAGGUGGGCAGA

>TP228

CUUGCAGGUGCCCAUCAGAAGCGACAGAUCCCCAUCAAGUACAAGGCAAUG

>TP229

CACAUGAAAUAGUAUCAAACUAUAUAUGCUUUGACAAGACAGAAGUAACAA

>TP230

UAGAGCUUCAGUUAUCUGGUUUGUCAACUUUAAGUGAUAUUCUUUGGCUCC

>TP231

AAAAGACAGUUUCUAGCCCAGGCAUAGUAGCUCAUGCCUGUAAUCGCAGCA

>TP232

AAAAAAGUUCAGCAUGAAGGAUGCCAUAUAUGUUGUUGCCAACACUUGGAA

>TP233

UGCCAACACUUGGAACACAAUGACUAAAGACAUAGUUGUGCAUGCCUGGCA

>TP234

UUAUUGAUGAUGUUGAACAAGUGGUAACUUUAAAGGAUUCUGUAUGCCAAG

>TP235

UUUAAAGGAUUCUGUAUGCCAAGUGAAAAAAAAAGAUGUCUGACCUCCUUA

>TP236

AGGCUAAGGUGGGAAGAUUGCUAGAACACAGGAGGUUGAGGCAGCAGGGAG

>TP237

GACUCACUGCAACCUCAGGCUCCCAAGUAGCUGGGAUUAUAGGCACCUGCA

>TP238

UCCAGCAAAAAAGAUCACCGCCGCGAGUAAAAAGGCUCCAGCCCAGAAGGU

>TP239

ACACAUGAACGAUAAUAAAGUGGCCAGGUGCAGUGGCUGACACCUAGAAUC

>TP240

AAAGUGGCCAGGUGCAGUGGCUGACACCUAGAAUCCCAGCACUUCGGGGAG

>TP241

AAAGAGCUUUGGGCCGGGCAUGGUGACUUGUGCGUGUAUUCCCAGCAUUUU

>TP242

CUGGGCUGAACACAGACUUUUUUUGAGGCUGAGUCUCACUCUGUGGCCUAG

>TP243

AAAACCCCGUCUCUAUUGAAAAAUUACAAAAAUUAGCCGGGCGUGAUGGUG

>TP244

GGAAGCCAGGUGCCGGGCCAAGCCCAGCCACCGUGGUUCUGGGCCGGGAGG

>TP245

CCUGUGACAGGCCCAGCCCAGCCUGAGAUCCCUUUGCAGAAUGACACAGCA

>TP246

GAAGCCUGGGUUCAUUAAUGAUUUAAGGUUUUACAGUAUUGGAAAUGGCAG

>TP247

AGCACUCAAGCCCACAUUCAGGGUUACAGAAAACCAUCGUCCUUCCCAUGU

>TP248

CUCUUGUUUCUAAUACUGUAAUCUUAAAUCAUUAAUGAACCCAAGCUUUUC

>TP249

UUAAUGAACCCAAGCUUUUCUUGGAAUGUGUGAAGUCGGUGAGAACGGCUU

>TP250

UCAUUCUGCAAAGGGUUCUCAGUCUAGGAUGGGGCUGUCACAGACCUGGUC

>TP251

UCCGUUCCUUGCAGGCCCUUGGGUCAGGACCCACCACCUGCCGGACCAUCA

>TP252

UCUGAUUUAGAUAUUUUUGAGACAAAGUCUCACUGUCGCCCAGGCUGGAGU

>TP253

CUCUCCACCCUCACUCAUUCCUAGAAGGCCCUCAGCAUCCUCUCCUCACAC

>TP254

AUCCUCCCACCUCAGCCUCCCGAGUAGCCGGGACCACAGGCGUGUAUGACU

>TP255

UGGGAUUAUGGCCAUUAGCCACUGUACCUGGCACAAUUUUUUUUUGUACCC

>TP256

UGAAUGGGGGUGGGGGGGGCGCCUUAGGUACUUAUUCCAGAUGCCUUCUCC

>TP257

CCAGACAAACCAGAAGCAACAGAAAAAAUCGUCUCUCCCUCCCUUUGAAAU

>TP258

AAUAUACCCCUUAGUGUUUGGGUAUAUUCAUUUCAAAGGGAGAGAGAGAGG

>TP259

UUUCUUUUUGUUUUUGUUUUUGUAGAGAUGGCGUUUUGCCAUGUUGCCCAG

>TP260

UGUUGUUGACUUUAGACAUCUAGAAAUAUAAAGCAUAAUCAAUUUAAGAAU

>TP261

UGCCCCAGUAAUUGUAUUUUUGGUAAAGACAGGGUUUCACCAUGUUGGCCA

>TP262

AGAUAAAUUAGCCAAGUGUGAUGGCAAGUGGCUGUAAUCCUAGCUAUUCAG

>TP263

AAGUGAUUCAAAUUCUGGUGUGGCCAGACAUGAUGGCCCAUGCCUGUAAUC

>TP264

AUGCCUGUAGUUGCAGCUACUCAGAAGACUGAGGCAGGAGGAUUGAUUGAG

>TP265

UCUUGAACUCCAGAGCUCCUGCCUCAGCUUCCAGGUAGCUGGGAUUACAGG

>TP266

CUUUGGAACAACUCCUUCAGGAAGCAGAGGCCUGUGAUUUGGUAACAGACC

>TP267

GAAGCUGAGGCCUGUGAUUUGGUAAAAGACCUGCUCGGUAGGCCAUCUUCA

>TP268

CUGCUGCAGCCAGUGUCUUGGCAGUAGCCACUCUAGAUGGACCACUGCUAC

>TP269

CCCUGUCUCACAACUGGAGUGCAGUAGCAUGAUCAUAGCUCACUGUACCCU

>TP270

CAACCUCUGCCUCCUGGAUUCAAGCAAUUCUGCCUGCCUUAACUUCCUGAG

>TP271

GGGUGAAUAAAGUAAAUAUUUUGGGAGGCUGAGGCGAGCGGAUCAUGAGGU

>TP272

AACCUCCUGAGUAGCUGGAACUAUAAGUUGGAACCAUCGUGCCCAGCUAAU

>TP273

CACGUAUUGUCCCAGCUUGUCAGGAAGCUGAGGUGGGAUGAUUGCUUGAGC

>TP274

UCCCACCUCAGCCCCUGGAGUAGUUAGGAUAACAGGCGCACACUACCAUUU

>TP275

AAAGGGGUCAGAGGCCGAGUAUGGUAACUCAUGCCUGUAAUCCCAGCACUU

>TP276

GAGACAAGGUCUCUCUUUAUUGCCUAGACUGGUCUUGAACUCUUGUCUUCA

>TP277

UCCCGCCUGGGUCUCCCAAAGUGCUAGGAUUAUAGGCGUGGAAAACUAAGG

>TP278

UUGCUUUUUUUAUUUUUUUUGUGGCACGAUCUUGGCUCACUGCAGCCUCCA

>TP279

CCUCACAGCAAGAACGCCCAUGGCGAGGAGAAGGAGAACCUCACGGCCCGG

>TP280

AGAGGUGGAAAACAGUUCUUUCCAAAGACAAGCUUAAUGACUGCUGUGCCA

>TP281

UUGUCUCUACAAAAAUAAAAUAAUUAGCAGAGCAUGGUGGUGGUGUGGCUG

>TP282

UAGAAAGCUUACCAGCAGUUGGCCAACUGCGGUGGGCUCAUGCCUGUAAUC

>TP283

AAAAUAUUCUCAGCAUAAUUUUUUUAAGAGAUAGAGUCUCACUGUUACCCA

>TP284

AUCCAAUCUUGGCAUAGCUGUCUCAAACUUCUGGGCUCAAGUGAUCCUCCC

>TP285

CGACGACGCCCGCCGCCUCACUGUUAUGAGCCUUCAGGUGAGAUGCAAGGA

>TP286

AAGCGGCCUCAUCCGGAGAAUACCAAGGGUACUAACUGGUUACUGUGGUGU

>TP287

AGAGAGCAGCUGAACAGACAACUAGAAAGAAUUAUUCUAGGCCAGGCGUGG

>TP288

AGGUUUCCAUGUUCUUCCGAUCAGAACCAAAGUGGGAGGUGGUGGAACCUU

>TP289

CGCUGGGGCUUGCCCAUGGUGCUCUAGAUAUAUAAGGCCCGGACAUUCUGC

>TP290

CUGCUGGUCCCCCAGGACACACACAAACAACUUAGGGCGGGGAGUGGACUU

>TP291

GUAUACUUUGGCCCAGUCAUAGCCCACUAAAGCCUUGACCUCCUGGGCUCG

>TP292

UCCUGAACCAUAGUAGGCUUUGGUGAGCAGAUAUGAAAAUUGUGAUGACCA

>TP293

AAGCCUGCAAGGACAGGAUGCUGAUAUCCGUGUUGAGGGUGGUCAGCGGCA

>TP294

GUCCGUGUUGAGGGUGGUCAGCGGCAUCCUGGAUGCCUGGUUCUGCCCGGG

>TP295

CCAACCUAUUUGCAUCAACAAUUUUACCUAACUGGUCCAUGUUAAGAAAUG

>TP296

GUGAAAGUGGAAAAGAUUCAUCCGAAAAUGGAUGGCACACUACUGAAAUCU

>TP297

AACAACUAAUGAUCCUUGUGAAUCUAGAUCUGUAUUUUAAUAAUCACACAU

>TP298

UGCCUGUGCCUAUCUACUCCAUGGAAUUUCAAAAGGGGCCACUUAUGGAGA

>TP299

GAUGGAGUGUGCAUUACAUUUGGGGAAAAAAAAAUAUGAGUCAGUCACUAC

>TP300

UGGGAGCACCCGAAUCUGGCUCGGCAGAAUAUCUCUUUGACAAGCACACGC

>TP301

GCCUGUAAUUCUAGCUGAUGCGGGCAGAUCACUUGAGCUCAGGAGUUCAAG

>TP302

UUGAGGCUCAGAUGAGUCCGCUUCCAGCUCAGCCCUGUUUGCGCAGAUUAA

>TP303

CACUGCAACCUCCCUGUCCGGGGCUAAAGCGCUUCUCAGCACCCAUCAGCC

>TP304

GGAUACCUGAACAAAGUCUGUCUCAAAACCAGUGUUAAAUCACUCUCAGGG

>TP305

AGGAGGCAAGAAUUGAAGUUGCUGCAGACCCAUAUGGAUUUACCUCCCACU

>TP306

GGAUUUACCUCCCACUAACACUUUUAGUACAUAGGACAGUAUCAUUAUUCC

>TP307

AAGUGUUAGUGGGAGGUAAAUCCAUAUGGGUCUGCAGCAACUUCAAUUCUU

>TP308

UGACAGAAGAGGGAGACCUGGAGACAGUUACCAUGGCAUGGUUGGUUUCAG

>TP309

AUUGUGUCAACAGAUGGAUCACUGGAAUGUGGGGAUUCUGAAACAGAAAUG

>TP310

UAUUGAUACAUGCUACAUUAUGGAUAGAUGUUGAAAUGUUAUGCUAAAUGA

>TP311

CUCUGUUUUAUGGGCAGUGGCAGGGAGGAAGAAGACAUGUAUAUGAACAUG

>TP312

AGUUCAAGACCAUUCUGUGCAGCAUAGCAAGAUCCUGUCUCUGAAAAAAUA

>TP313

UGCUAAUUUAAAAAAAAAUUUUUUUAGAGAUGGGGGACUUGCUGUGUUGAC

>TP314

CGGUGGAGAGAUCACAGGACACUGCAGCCUCAAAUUCUUGGGCUCAAGUGA

>TP315

AAAGAGACCUGGCGUGGUGGUUGACAACUGUAAUCCCAGUGCUUUGGGAGG

>TP316

UUCCUCGGGAGUUAAGAUGGUAAAAAGAAAAAAAUAGAAUGAAUAUGAUCA

>TP317

GCUUUUUAUCUGUGGAUGAAUGGAAAUCACACUGAAUUUUAAUUCUUAUUU

>TP318

AUCACAAUUGCACUACAGCCUGGACAACAGAGUGAGACCCUGUCUCAACAA

>TP319

GGGUUGGUUUGUUUGUUUGUUUUGCAACAGGGUCUCACUUUGUCAUCCAGG

>TP320

UAAUUGAAAAGAGAAAAAGAAUUAUAUCUUUCAGUUCCAAAAUGUCAAUUU

>TP321

CCCUGUGGGGUUGGCCAAGGCUGUCAUUGGACCUACAUUAUAACUCAUUUU

>TP322

UCACGGAGAGCUAUGAGGGGAUAAAAGGGUAUGAUCUAAUGGUAAGAAACU

>TP323

AGGACCUUACACUGUGCUGAGAGUCACAGAAGUAAGUGGUUGUAAUAUGAU

>TP324

AGUCCACCAAAAGGUUUCAGCAACUAAUAAACAACAUCAGUAAAGUUUCAG

>TP325

GUAUAUACAGGAAUAUAAAAGAAUGAAUCUGAGACCUUCAUGCAUCACGUU

>TP326

GACGCAGUGUCUGCUGGGACUGCGCACGUUCGUGGCCUUCGCUGCCAAGCU

>TP327

UAUGAAUUUUAGGAUUAUUUUUUCUAUUUCUAUAAAAAUGACAUUAGAAUC

>TP328

UUGAGGGCAGGCCUUAGUGAGAAUAACGGAAUGUUCUGGCAUAUUUCAAAA

>TP329

AAAAAAUCCAUAAAAGAUUGGGGAAAAUAUAGAAGUAAAUGUGUCUCUGAU

>TP330

GUCUCUGAUUUCUGAGUAGGAAGAUAUAUAAAUGUGUACAUAUACAAGUUG

>TP331

CAUAAUAAUGUCCUUGAAGUUUAUCAAUGUUGUAACCUGUUGAACGGAUUG

>TP332

UCUUUAACCAUCUGUCAGUGAACAUAUAUGUUGCUUCCACUUAGCAAAAAU

>TP333

AAUAUUUAUUUAUUUAUUUAGAGACAGGUUCUCACUCUAUCACCCAGGCUA

>TP334

CCACUUAUUAAUGAGAACAUGCAGUAUUUGGUUUUCUGUUCCUGUGUUAGU

>TP335

UACACUUUGUGCUAAAUCAAGAGUCAAUGACUUUGACUGGUUUGCCAAAUC

>TP336

GCUAUAAAGUACUAUGGAGUUAUUUAAAUUUUGCACCAAAGGACAGGGGGC

>TP337

AAAGUAAAUCAACGUAUUACUAUUCAUCUUGGAUUCUCAAACCCCCUGCCC

>TP338

UCAUUGCUUUUUCCCUUAAGAGACAAGUCCUUACUAUAUUGCCCUGUCUCU

>TP339

UGAGCCACCACACCUGGCCAUUUUAAUUACUAUUCUUAUAUUACUAGUAAG

>TP340

UGUCUCUACAAAAAACUAAAAAAUUAGCCAGGCGUGACAUAGUCCCAGCUA

>TP341

ACAAAGAUAAAUCUUUCUGACAUGCAGACGGAAAUCAAGCUGAGGCCUCCU

>TP342

GGGAUAGCAUUAGGAGAAAUACCUAAUAUAGAUGAUGGGUUGAUGGAUGUG

>TP343

AGAUGAUGGGUUGAUGGAUGUGGCAAACCACCAUGGCACAUGUAUACUUAU

>TP344

GAUUCUGUUGCCUUGUUUUCCUGAAAUUUUUUUUUUUUGGAGACGAAGUCU

>TP345

GGCUUCCCUUUCCACCAUGAUUGUAAGUUUCCUGAGGCCUCCCCAGCCAUG

>TP346

UUUUAUUUUUUAUUUCCCAUUUCUUAGAGACAGGGUCUUUCUCUGUUGCCC

>TP347

GAGGAUUGCUUGAGCCUAUACGUUCAAUAUCAGCCUAUUCAACAUGACAAA

>TP348

AUAUGGUUUGACUAUGUCCCCAUCCAUAUCUCAUCUUGAAUUCCCAUCGGU

>TP349

GGAGGGACCCAGUGGGAGGUAAUUGAAUCAUGGGUGCAGGUCUUUUCCAUG

>TP350

GCAGGUCUUUUCCAUGCUGUUCUCGAGUUAGGGAAUAAGUCUUACGAGAUC

>TP351

UAGGGAAUAAGUCUUACGAGAUCUGAUGGUUACAAAAAGGGGAGCUUCCCU

>TP352

UACAAAAAGGGGAGCUUCCCUGCACAAGCAUUCCUCUCUUUGCCUGCCACC

>TP353

CUCCUUGCCCUCUGCCAUGAUUGUGAGGCUUCCCCAGCCAUGUAGAAUUGU

>TP354

AGCCCGGAUGCAGUGGCUCAUGCCUAAAAUCACAGCACUUUGGGAGGCUGA

>TP355

AAAAAAAAAAUAGCUCUAUUGAGAUAUAAUUCACAUACCAUACAAUUUACC

>TP356

GAUUGUUUUAUCCACUGAAUUCAGUAGAUAAAAGCAUAGACCCCUCUUCCU

>TP357

CUACUUGCUAAGGAGCUCUGCUCCAAAAAUCCUCCCCGUUCCUAUAUCAAA

>TP358

GUUUUUCUUUCUUUUUUUAUAUGGAAGGGACAACAUGUUUUUGUAUACUGA

>TP359

UUAAAGUUUUGCUAUUGUAUUCUUUAUAGUUUUUUGACAUUAAUUUUGAUU

>TP360

ACAAUAAUGUUCAUUAAAAUUAUUUAUCUUGGCUACUGAGUUUUUUGGUGC

>TP361

CCCUGUAUACCCUGCGGGAUGGUGUAGGAGGCCUGCCUACCUUCCUGGAGA

>TP362

UUUUUUUCAUAUUUCCCUUUCUGUAAUCUCAGCACUUUGGGAGGCUGAGGU

>TP363

UCCAGCUAUUUUUAUCUUCUUAAGUAUGCAUUCUACAUGAGAACGUUUGGG

>TP364

GAAUGAAGAUGGAAAUCUCCCAUCUAACUCAGGAGUUACAUCAGCGAGAUA

>TP365

AUGGUCUCGAUCUCUUGAUUUCGUGAUCUGCCCGCCUCAGCCUCCCAAAGU

>TP366

UGGUCUCCUGAGUAACUGGGAUUACAGAUGUGAGCCACCACACCUGGAGGC

>TP367

AAAAUUUAUUCAAGAAAAUCUGGCCAGGCACCGUGGCUCACGCCUGUAAAU

>TP368

GGUUGCAGUAAGCCAAGACCGUGCCACUGCACUCCAGCCUGGGUGACAGUC

>TP369

GGCUGUUGCUUUCCUCUCGCCCAGUAGCCAACCCAAGCAAGGGGUGAGUCA

>TP370

ACUGGCUCGAUAAGAAUCAAGCUGCAGAGAAGGAAGAAUUUGAACAUCAGC

>TP371

UGUUAGCAAAUGUCACUUAGAGGCAAGUGACUUUGAGAUCCCUGUGACCAC

>TP372

UGGCUGGGCCUAGUGGUUCAUGCUUAUAAUCCCCGUACUUUGGAAGCCUGA

>TP373

AAUCUCCUAAAAGUGUGUUUUUAUUAGUAAAUCUUUAAGAGAUUGCAGAGA

>TP374

UCUUACCUUCUGUUUGAGUCACAAUAGCAAAUUAUUAUUUGUCUUUGAGGG

>TP375

CUCCACUAUAUUAGAAGUUACUAUAAGCUCACUGGGCAUGCUCAUGGGCAG

>TP376

CACUGGGCAUGCUCAUGGGCAGUUUAUUUUCAAGGCACCCUUUUAAAUAAC

>TP377

UUAAAUAACCCAUACAUAUAAUGUUAGUAUUUGUUUAUAUAUACAUCAAGA

>TP378

AAAUGCUAACAUAUAUAUAUGGUUUAUUUAAAGGGUGUCUUGAAAAUAAAC

>TP379

UAAACUGUCCAUGAGCAUGCCCAGUAAGCGUAUGGUAACUUUGAAUAUAGU

>TP380

GCUUCCCAGGUGGCAUCACUCCGACACUCGAUGGGGCCAGGGGGCUAUGGG

>TP381

AAAACUAAAAAUAAGAUUUACUUGGAGAACUGAUGAGGCACAGUGGCUCAC

>TP382

CUUUCUUUGAGACAGUGUUAGUUUUACUCUGUCACUUAGGCGAGAGUGCAG

>TP383

AUAUGGCUAAAGCAGUGGUUCUCAUACUUUAGUUUCUAUCUGAAUCGCCUA

>TP384

ACUUUAGUUUCUAUCUGAAUCGCCUAGAGAGUUUGUUAAAAUAGAUUACGG

>TP385

AUUUGUUCUUGUCAUUCUUCACUGAAUAGAUGAAAUAUGUUAAGGUGUCUU

>TP386

UUUUUCUGUAUAUCUUUCAGGCAUUAAGGGAUCAUUGCAAGAGCAGCGUGA

>TP387

CUUCCUUCCUUCCUUCCUUCCUUCCAUCCUUCCUUCCUUCCCUCCUUCCUU

>TP388

AGCGAUCCUUCUGCCUUACUUUCCCAAGUAUCUGGGACUACAGAUGCACAC

>TP389

CAGGAAAACAAACAAUCCCAUUAAAAAGUGGGCAAAGGACAUGAAUAGACA

>TP390

CACUCCAGCCUCAGUGACAAGAGCAAAACUCCUUCUCAAAAAAAAAAAGUA

>TP391

ACCAGAUGUCCAAUUAAAUAAAAUUAGAAUCUGGAGGAGGUAGAACUCAAG

>TP392

GGAUGAAGGUGAAGAAGAUGAAGAUAAUGAUGAAGGGGAGGAAGGAGAGGA

>TP393

CGCAUAGGGGCACCAGACCAUGAACAGUGAGAUUCUCUCUAGCACGCUCAG

>TP394

CAGCUGAGCGUGCUGGAGAGAAUCUAACAAUCCAUUGUCCAGCGCCUCUAU

>TP395

AAAUUUAAAAAGGAGGGAGGUGGGUAUUUAGAGACUCUUAUUGUACAGCCU

>TP396

AAACACCAGAAUAGACGUUCCUGAAAAGAAGACGACAUAUAAACGGCCAAC

>TP397

GUUCCUGAAAAGAAGACGACAUAUAAACGGCCAACAGUAUAUGAAAAAAUG

>TP398

AUAUAAACGGCCAACAGUAUAUGAAAAAAUGCUCACCAUCACUAAUUAUCA

>TP399

GAAAAAAUGCUCACCAUCACUAAUUAUCAGGGAAGUGCAAAUUAAAACCAC

>TP400

AAGACAAAAAUUAGUGGUUGCUGGCAAGGAUGUAGACAAAAGGAAACCCAC

>TP401

GCUGGCAAGGAUGUAGACAAAAGGAAACCCACAUACACUGUUGGUGGGAAU

>TP402

CCACAUACACUGUUGGUGGGAAUGUAAAGUAUAUAGCCACUAUGGAAACCA

>TP403

GUAUAUAGCCACUAUGGAAACCAGUAUGGAGGUUCCUUAAAAAACUAAAAG

>TP404

GAAACCAGUAUGGAGGUUCCUUAAAAAACUAAAAGUAGAACUACCAUAUGA

>TP405

CAGGAAAGCAAUGAGAGAUUCUGACAGUGGACUAGAAAAAAUGGCUAUUGG

>TP406

UUUAAAACUAAAAGUAAUUUAGAUUACAUGGAUUUGAGAAGCCCAUGUUGA

>TP407

AAUAAUAGAAUCCAGUGUUUCCCAAAGUGUGUUCCAAAGAACACAAGUUCC

>TP408

AAAUGAAUGAGUUCAUUUCCUUUGCAGGGAUGUGGAUGAAGCUGGAAACCG

>TP409

GGUGCAGCAAACCACCAUGGCACAUAUAUACCUACGUAACAAACCUGCACA

>TP410

AAAGUACCACAGGACUUCCUGUAUUAGCUUGCCACAUGGAACAUAUAAUCC

>TP411

AGCCCACUCCUGUAAUAAUGGCAUUAUUCCAUUCAUGAAGGCAGAGAUCUU

>TP412

GUCUUAACAUGAAAGCAGAGUGGUGAUCCUACAGCUGACUGAUCUCACCCA

>TP413

UUGUAUAGUGUUAUUUGUGUAGCACAUAUUGUUACUAAUGAUUAAGGUCCC

>TP414

GUCUCACUGUGGUGCCCAGGCUGGAAUGCAGUGAUGGCAUCAUAGCUCACU

>TP415

CUGUUAACUGUAGCAUCCAAAGAUGAUAGGGUGAGUACUCUGUAAGUCUGG

>TP416

UUCAGUGACUCACAACUGCGAUCCCAACUACUCGGGAGGCUGAGGUGAGAG

>TP417

AUAAGGAAAUCAGCCUGGGCAACAUAGUGAGACCUUGACUCCACAAAAAAU

>TP418

UUUUGUUCGGCAUGUGAUAUGGUUUAGAUAUGUGUCCCCUCCAAAUCUCAU

>TP419

CUCUGGCUCCUGCUCUCGCCAUGUGAGAUGCCUGUUCCCCUUUUGCCUUCC

>TP420

CAGACUGUACAGGAAGCAGGGCACCAGCAUCUGCUUCUGUUGAGGCCUCAG

>TP421

CAGGGAGCAAUGGGAAGAGAAGGGAAGUCCCAGACUCUUUUUAACCAGCCA

>TP422

ACUCAUUACUGUGGGGAGGGCACCAAGUCAUUCAUGAGGGAUCCACCCCCA

>TP423

GCAGUCUGCCCACUUUGGCCUCCCAAAGUGCUAGGUAAUAUCACAGAUGUG

>TP424

GCCUCCCAAAGUGCUAGGUAAUAUCACAGAUGUGAGCCGUUGCGCCUGGCC

>TP425

UAUAUAGGAAUGCUUGUGAUUUUUGAACAUUGAUUUUGUAUCCUGAGACUU

>TP426

GCUGGGCAUGGUGGCGACUGCCUGUAGACUGAGCUACUUGGGAUGCUGAGG

>TP427

AACUGGAUUUUUUUUUUUAAAAAAAAGAAAAAAAAAGAAGAAAAGUAUACC

>TP428

AGUGUCCGUGGGUGAAUAAAAAAGGAGGGGGGGGUGCAAAACUGGGGUUGC

>TP429

CAAUGUGGCCUGCAGAUAUGGCAACAAGAAACUGUCAUGUAUUCUCUUUGA

>TP430

UAUGUAUAUUUUAUAUAUUGUGUAUAUAGUUCUAUUGUUUUAGAUUGCUAU

>TP431

GUGGUAUACAAAAACCACAUACAUUAUACUUUAUGGAUGACUUGAGAUUUU

>TP432

CCUCACCUCUUUUUUCUCUUAAAGAAAUGGGAUCUCCCUCUGUUAUCCAGG

>TP433

UCACAGCUACCCCAGAGGCUGAGGUAGAAGGCCAAGAAGUUUGAGGCUGUG

>TP434

CAACUGGCCUCAUUUAAUUCCUGGGAGGAGAUAGUUUGGGAUGCAGUGCUU

>TP435

UGCUGAGUUGGUGGCCGCCUAAAUAAAUCCUCCUGUUUCACCCAGUGACCU

>TP436

UCUACGUCAGCCUCCCAAGUAGCCAAGGCCACAGGUGUACACCACAAUGCC

>TP437

UCAGAUCUGGAACAUUUUGGAUUUCAGAUUUUUUAAGAUUUGGAAAGUUCA

>TP438

CUUCCCAUCUCUGUACUCCUGGCAUAUUUGACAGUCUGAGGGUCCUCAAAC

>TP439

CAUAUUUGACAGUCUGAGGGUCCUCAAACUUUUCCACUCCCCUUCCUAGCC

>TP440

CUUUUCCACUCCCCUUCCUAGCCCCAAACUGUAGUUACUGCCUGACUUCUU

>TP441

CUCACCACAUUCCCCCGGCCAAACUACAUGCUCCGUGACCACAUUCCACAC

>TP442

GCACCCACUCUCCACAUAGACCCACAUUACCUCUGUUAUUAUAUAGGAAAG

>TP443

ACCCACAUUACCUCUGUUAUUAUAUAGGAAAGAAUGUGAAAUACAGUGGAA

>TP444

UAUUAUAUAGGAAAGAAUGUGAAAUACAGUGGAAAGAGUAUGGAUUUUGGA

>TP445

GGAAAGAGUAUGGAUUUUGGAGUCAAGCCUGGGGUGAAAUUCCAGCUCUGA

>TP446

AGCUCUGACAUCUACCAGUCCUGUGACCUUGGACAAGUUAACUGAAUACAU

>TP447

AUUCGAAUCACCCUAACAAGCCGCAACGUAAAAUCCUUGGAAAAGGUGGGU

>TP448

AUGUGUUCACAGUGCCCAGCACAGAAGCAUGCACAUAGUAGGUGUUUAUUU

>TP449

UGAAAGAAAACGGGACAUGUGCCUUACCCAAGUUCAGUCAGCGAAGAGUGU

>TP450

UAUGUGUGUGUGCAUAAUUACCCUUACCCAGAAAAUUCUCCAUUAUAAUUC

>TP451

AAAUUCUCCAUUAUAAUUCUUUAAAAGCACAGGUUCACAAAUUAUUAGAAA

>TP452

UAAAAGCACAGGUUCACAAAUUAUUAGAAAGUAUAUUUUGUAUACUAUUUA

>TP453

AAUUAUUAGAAAGUAUAUUUUGUAUACUAUUUACGGUAGCAAAAUAUCAAA

>TP454

UUUGUAUACUAUUUACGGUAGCAAAAUAUCAAAUGCAAGAAGCAAGUUUAA

>TP455

AGCAAAAUAUCAAAUGCAAGAAGCAAGUUUAAUUAAAGAUAGGCAAGACAU

>TP456

UAUCCUCCAUUUAUUUAGAUCUUUUAUUGCUCUCACUAGUGUUUUGUAGUU

>TP457

CAUUGGAUACUUUGCUACUGUAAACAGUAUAUAAGAUAUAUUUUCCAAUGA

>TP458

AUACUAAAAUUUCAGUGGUCCUUAGAGUUUCCUUAGAUACAAAUUUUAUUG

>TP459

GUUGAGACUUAUUUUUUACCAGUCUAGGCAACAUGGGGAGACCCUGUCUCC

>TP460

AUUGUCUUCAUUGUAUUUGCUUAAUAGUGGUCCAGACAAGUUCAAAACUUG

>TP461

AAUAGAAUCAUGCUUUGGGAGGCCAAGGCGGGCCGAUCACGAGGUCAGGAG

>TP462

GUCUCUACUAAAAAAAAAAAAAAAUACAAAAAAUUAGCCUGGCUCAGUGGC

>TP463

CUCCGUCUCAAAAAAAAAAAAAAAAAAAUAGGAUCAUGGCCCUUUUAAAGA

>TP464

ACACAUAUAUAUUUCUUUUCUUUUUAGAGAUGAGGUUUCUCCAUGUUGCCC

>TP465

UCCACCAGCUCUGCCUGACUCCACUAACACAAAACCACACAUCACUGAGGU

>TP466

GGAACCCUGGGGUGGUGAUGUAGUCAUGGGCCAAGUGUCCUGGAACCCUGG

>TP467

CUGAAAGCUUGCAUUCUUACAGGUAAUACACCCAGCACCCAGAUCAAGAAA

>TP468

ACUCAUGUCAGUUACUGGGCCUGCCAGGUGUGGUGACUCAUACCUGUCAUC

>TP469

UCUACCAAAACAAAUUGUUUGAAUUAGCAGGGUGUUGUGGCAUGUUCCUGU

>TP470

AGAUAAUGAACGAUGGGCCGGGCACAGUGGCUCACAUCAGUAAUCCCAGCA

>TP471

UAAUCCCAGGUAUGCAGAACGCUGAAGCACAAGAAUCGCUUGAACCUGGGA

>TP472

CACAUGUUUUUGUUUUUGUUGGAGAAGGAGUUCUGCUCAGUCGCCCAGGCU

>TP473

GGCAAGAGACUUCAUUGCUGAAAUCAGCAAGGAUGAUCAGGAGCAGCAAUU

>TP474

AGAUUAUCAUUUGCUAACAGAUAUUAUGGGAAUUGAAGCUUACAAUGGUGA

>TP475

AAAGCUUUGUAUCCUGUUAUUCCCAAAGAUUUUCCUUUUGCCAUAAGAGUU

>TP476

ACAUUUUACAGCAGGACUUUUGCCAAGCUAUCAAAGUGGGAGUGAAAUAUA

>TP477

GUUGCUGGAGCACCUAAAAGUCAGAAUGUCAUGUUGGAAGCCUCUGCAGAG

>TP478

CUCUGGAAAGCUGGCCAGAUUUGCAAAUGGCUCUGCUGUAAUACAGUCAGG

>TP479

AGGCUUAUAAAUCAUUCUACUGUAAAGACACAUGCACAUGUAUGUUUACUG

>TP480

CUGGGAAUAUACCCAAAGGCUUAUAAAUCAUUCUACUGUAAAGACACAUGC

>TP481

AAUUCCUCAAGGAUCUAGAACCAGAAAUACCAUUUGACCCAGCAAUCCCAU

>TP482

AUUAGUUCAACCAUUGUGGAGGACAAUGUGGCAAUUCCUCAAGGAUCUAGA

>TP483

CAUCUAGCAUUAGGUAUAUCUCCCAAUGCUAUCCCUCCCCUCUCCCCCAAC

>TP484

CGCUUAUCUUGCCUGCUGCCAGGUAAGAUGUACCUUUGCUCCUCCUUCACC

>TP485

CAGGCCUCAGGAAGCUUUGAGUCAUAGUAGAAGGUGAAGAGGAAGCAGGCA

>TP486

UUGCUGUAAAGAAAUACUUGAGGCUAGGUAAUUUAUAAAGAAAAGAGGUUU

>TP487

UUGGAAUCUCAGAAGCAGAGGAGAAAGAGAGUGAUGCUGAAUGAUUGUUCA

>TP488

UCUGUACAUUAAAAACAAAAGAUCUAAUAUUUUAUCAUUGGAAUCUCAGAA

>TP489

UGAAAAAAAGUAUGAAUAGAGCUUUAGGAAUCUGUACAUUAAAAACAAAAG

>TP490

AAAAAGAGAAAACAGACUGAAAAAAAGUAUGAAUAGAGCUUUAGGAAUCUG

>TP491

AGCACAGAUCCACUCAAUCUGAACAAAAAGAGAAAACAGACUGAAAAAAAG

>TP492

GGAUAGAAUCAGUUAAGUUGAGCACAGAUCCACUCAAUCUGAACAAAAAGA

>TP493

GCAUAAUGUCUCCAGGUUCAUCCAUAUUGUUGCAGGUGGCAGGAUUUCCUU

>TP494

GUGAGUACAGUGCACAGCCAAGAUUAAGAACCCCUGAACUAACUUGUAUAA

>TP495

CUUUUUUUUUAAGAGGCGGUAUCUCACUAUGUUGCCCAGGCUGCAGAGCAG

>TP496

CCUAAGUAGCUACAACUACAGGCACAUGCUGCUAUGCCUGGCUAUUUAAAU

>TP497

UCAUGGCAGAAGGCAAGGAGGAACAAGUCACAGCUUACGUGGAUGGCAGCA

>TP498

UUUUUUUUGUAAACUGCCCAGUCUCAGGUAUGUCUUUAUCAGCAGCGUGAA

>TP499

UCAGAAUUACUUUAGAGUAAAACUGAUGCUUGAAGAAGUGUCAGCAAUGGU

>TP500

AGGCACAUGCUACCGUGCCCAGCUAAUUUUCGUGUAUUUUGUAGAGUCGGA

>TP501

CUCAAAAAAAAAAAAAGAGAAGUUCAGGUUCCUCCUUGGGACUGAACUUCC

>TP502

GCUUAGAUCACGUUACUGCACUCCAACCUUGGCUGCAGAAGCGAGACUCUG

>TP503

AGGGAGAAUCGCUUGAACAUUGGAAAGCAGAGGUUGCGGGGAGCUUAGAUC

>TP504

UGCCUAUAAUCCCAGCUACUCAAGAACUGGGACAGGGAGAAUCGCUUGAAC

>TP505

AGUAUAAUUAAAAAAAAAAUACGAAAAUUAGCUGGGCAUGGUGGUGUGUGC

>TP506

AAAAAUUGGGGGAAGGGAUAGCAUUAGGAGAGACACCUAAUGUUAAAUGAC

>TP507

GCAAAGCUUCAUGUUGGGAAACAAAACCACUCAACCUACCCAGAGAUCAGC

>TP508

AGUGCAGGGGGCGGGACGGAGGCCAAGGCUAAUCUCUGGGUGGGGUGAGCG

>TP509

CAAUGGGAACUGAGAGUUGCCUGCUAUACUUCCUGCUUUGUGGACAGCUAU

>TP510

UGACCGAUGGUGAACUGCAGAGACAAUGGGAACUGAGAGUUGCCUGCUAUA

>TP511

AGGGAGUCACAGAAGGUUCUGGGUAAUGACCGAUGGUGAACUGCAGAGACA

>TP512

AAGCUCAGGGCAAGUGACUUGUCCAAGUCCCCACAGUUAGUAAAAUGGCAG

>TP513

CAGUGAGAGUGAGACUCCGUCUCAAAAAAAAAUAAGAGUGGUUAACUUUUU

>TP514

UGGAGCAGGACAGAGGGCAGGCAGGAUGGAUGGGGAAGGUGGCAGGGUGAG

>TP515

CCAGAGAUCCACAAGCUAUGGCUAUAACAGUGACAAGGAGGCCUAGCGUGA

>TP516

UAGCACAUAGAUAGAUGAUUUAAGCAGAAAUAGAUAAGCAUAAUUGUCCAC

>TP517

UUUGCCCUCUGAAGAAUAACUAAAAAAUCACUCACCAAAUGCAGAUUAAUA

>TP518

CUUAUAAAGCAGGUUGUAAUAAAGAAGCCAGCCAAAUCCCAGCAAAACCAA

>TP519

AGAGUGAGACCCUGUUUCAUAAAUAAAUAAAAUCACUCUUUUUACUUUAUA

>TP520

CAGCCACCGCAUCCAGCCUAUUUUUAUUUUUUAUUUUUUGAGAUAGGGUCU

>TP521

AUGAGAAGAUGUGCAUAGGUUAUACACAAAUAAACCAUUUUGUACAAGGGA

>TP522

CUUAACACCCAAAGAGGCCAGGUCAAGAGUUCAAGACCAGCCUGGCCAACA

>TP523

GCUGGAUCAUAUUGUAAAGUAUGUCAAGUUUUGUAAGAAACUGCCAAACUG

>TP524

AAAAGUUUUUCAUGGAAUCACUUGAACCCAGGAGGUAGAGUUUGCAGUGAG

>TP525

UGCUUGAGGCCAGGAGUUCAAGACCAGCUGGGACAACAUAGCAAGACCCCA

>TP526

AGAACUGUUCUUUUUUUCUUGAGACAGGGACUCAUGCUGUCACCCAGGCUG

>TP527

CACAGCCAUUUGGGAGACUGAGGCUAGAGGAUUGCUUGAGCCCAGUGAGGC

>TP528

AAUCUAGCACACCACCUGCUUCUGGAUGGUCUUCAACCUAAGAAUAGUUUU

>TP529

ACAUUUCCAUCAUCACAGAAAGUACAGUUGGACUAUGGGCCAAAUCUAGCA

>TP530

CAUCAUCCAGAAUUGUAGUCACAUUACGGAAAGCAAAUUCCAAAAUUUGAU

>TP531

UGAUCCUGUGAGAAAUACAGACUCUAGGGCCAAAGAAGUAAAAGAGAUACU

>TP532

AUUCCAGCCAUUUAUAUUCAGAACCAUUGAUCCUGUGAGAAAUACAGACUC

>TP533

UCUCACAGGAUCAACUGUUCUGAAUAUAAAUGGCUGGAAUAUUAGGGGACA

>TP534

GUAUCUCUUUUACUUCUUUGGCCCAAGAGUCUGUAUUUCUCACAGGAUCAA

>TP535

UUAAAUUAGUGUAUAUAAAGCAGUUAUUAGGGACUUUAGACGUCUUCUAAA

>TP536

ACUUUCAAGAACAUUAGAAAAGCUCAAGAUAUAGGUGCCAGACAUUGCUAC

>TP537

CCUUUUGCCUCCCACCAUGAUUCUGAGGCCUCCUCCAGCCUCAGGGAACUG

>TP538

UUUCUCUUGCCACUUCCACGUAAGAAGUGCCUUUUGCCUCCCACCAUGAUU

>TP539

CAUACUGCUGUCAUGGUAGUGCAUAAGUCUCACAAGCUCUGAUGGUUUUAU

>TP540

GGAGGGGCAAGGGGCAGAAUGACAUAGUUUGGCUCUGUGUCCUCACCCAAA

>TP541

UGACAAAAAUGCUGAUAGUGAUACGAACAAUAAGGUCCAGGCUGAGGUAGU

>TP542

AAAUGUGUUGCAAGUACAUGUUUUCAUUUGUGCAUUAUUUCAUCCAAUACA

>TP543

GAAGAGGUGGUUUUGAAAGCUACAGAUGUCAAUGAGAAGGUAAAAAUUAAA

>TP544

UAGUUCUAGUUCAAGGAAGAAUGUGAUAUCAACAAUAAGUUAAAAAAAACC

>TP545

CUGGGCAGGUAAAAGCAAUAGAUAAAUGCUAAUUCCUUUAAGACUGGGUUC

>TP546

AUACUCCCACUAACAGUGUACAAGCAUUCCUUUUUCUUUACAACCUCACUG

>TP547

AAGAAUCACCACACUGUCUUCCACAAUGGUUGAACUAAUUUAUACUCCCAC

>TP548

CCUCUAUGUGUCCUUGUGAUCUCAUAAUUUAGCUCCCAUUUAUAAGUGAGA

>TP549

UUUCUUUCUGCAUCUGUUGAUUUUCAGUUGCCUUCAGCUCAAAACAAUUCA

>TP550

GAAUAUACCACUAUGGGAUAAAAUUAUUUUGAGCUCAAGACAUUUGAGAAU

>TP551

UAAUGAGUGAAGGGAUCUGGAAUAUACCACUAUGGGAUAAAAUUAUUUUGA

>TP552

GUGGGAAAAACCUGCCCCAUGAUUCAACUGCCUCCCACCGGGUCCCUCCAG

>TP553

UGCACUCCAGCCAGGCAACAGAGUGAGGCUCUGUCAACAACAACAACAACA

>TP554

UAAAUAACUUUUAAAUGGACAAAGGACUUGAAUAGCUAUUUCUUCAAAGAA

>TP555

UAACAUAUAGAGCAGAAUGGCUCACACUUGCAAUCCUAGCACUUUGGGAGG

>TP556

CAGAUUGUCUGAGCUCAGGAGCUUGAGCCCAGCCUGAGCAACGUGGAGAAA

>TP557

UAAUCUCAACAUGGUAGGCUGAGGCAGGCAGAUUGUCUGAGCUCAGGAGCU

>TP558

GUGUAGACCUUUUAGUCGAUGCUGUAGCCAUUACAAUGGGGCCAAAGGGAA

>TP559

AAUUGUGGCUUGCCAAUGAAACAUAACAUCCUCAACUGGACCUGCAGAACA

>TP560

UGCAACCUUAGGUGGUUUGAAGGAGAAGUCUGUAGGAAAAUGAAUUGCCAA

>TP561

CUCAGCCUCCCAAGUAGCUGAGACUACAUGCAUACAUCAUCACACAGGGCC

>TP562

ACUGCAACUUCAAAUUCUUAGGCUCAAGUAAACCUCUGGCCUUGGCCUCCC

>TP563

CCUUUUAGAGACAGGGUCUUGCUCUACCACCUGGUGCAGUGGUAUGAUCAU

>TP564

UCCUCUAAGCACCACUCACUCAGAAAGCCCAACACCAAGCUCCUCCUCUUG

>TP565

CCAGCCUUGUCUCCUAGCCUCCUCUAAGCACCACUCACUCAGAAAGCCCAA

>TP566

GGCUGAUCAUUCUGCGUCUGUGGCUAGUAGUCCUUGAGAUGCAAGUUUGGA

>TP567

CCUGUCUGAAUCAAUGGCAUAAGCAACUACACCAUUGCUCAAAUCAGAACC

>TP568

AACUGCAGUUGGUAGCCUAUCAGGCAUCUCAAAUUUGACCUAGCCAAAGCA

>TP569

ACCCCCGGUCACCAGAGCAGCAGGAACACCUCAGGGGCCACACUUUGGAGG

>TP570

UAUUAUGUAAAUGUUCACUAUAGGCAGGGCGAGGUGGCUCAAACCUGUAAU

>TP571

UCCCUCAGGGUUUUGCAUCUUCUUAAUGGCAGAAACCUAGUCAAACAAAAU

>TP572

CUUGGAGCAGGGCAGGAGCAGCUGAAAGGGAGGUGGCACCUGGAGGUCUCA

>TP573

AAAGGACAUACCAAUUUUAGGAUUGAAAUAGUUCAGGCCGGGCGCAGUGGC

>TP574

GUAUAUCUUCUUUGGAUUAAUGUCUAUUGAAGUCCUUUGCCCAUUUUUGAA

>TP575

AGAGAAUUUCAGUGAUGGGAAAUAUAUUUGGUGACUAUUAUCAUUAAGAUA

>TP576

GCCAGGUGUAGCGACACACACCUGUAGUCCCAGCUGCUCAGGAGGCUAAGC

>TP577

CCUCUCUUCCUCUAGUUUUUAGCUUAGGGUGCAUCUCAACCCGGAAAGGUG

>TP578

AUGUUAUAGCUUCAUGGAUAAAGCCAGCCUGAUCACCAAACUCACCAGACC

>TP579

AUUGGAUCGCUUAGGGCUAGGAGUUAAGACCAGCCUGAGCAUUACGGUGAA

>TP580

UGGGGCAGAAAGAUUGCCUGAGCUUAGGAGUUCUAGGUUACAGUGAGCUAG

>TP581

CUACAAAAAAUUUUUUUAAUUAACCAGGCGUGAUGGCACAUGCCUGUAGUC

>TP582

UGUACCUAACAGUACUAAACUGCUUACUUAUAAAAUGGUUAAAAUGGUAAA

>TP583

AGCGGCUCCUGCUUGUAAUCCCUUUAGGAGGCUGAGGCAGAAGGAUCACUU

>TP584

GAUUCUUUCAAGCGCCAGACCUUAAAUUCAUCAGCAUCUUGAAACUCCACC

>TP585

ACUCUUUCUGUCUAAAUAACAUCCAAUGACAAAUCCCAUAGGGACAAGAAC

>TP586

CUUCCAGGUAACUUCUUUAUUGGGUAGCAAUACCCUUUUAAAUAACAUGCU

>TP587

AUUCAGUGGUUUUAUUAUAUUCACAAAGUUGUAUGGCCAUCACCAAAAUAA

>TP588

GAAAUCACUGCAGCCUCAAACUCCUAGACUCAACGAUUCUCUUGCCUCCCU

>TP589

GAGUUUCCUUCCUGUUUAUUUUUUAAGACAGCAUCUCACUCUGUCACCCAG

>TP590

UAUCCCUUUGACGCAGGUGGUGGCAAAGGAAGCCUGGGAGAAUCACAGGUU

>TP591

CAAUCCCUCCCAAGGUGGUGGAAUUACAGGUGUGAGCCACCAUGCCUGGCC

>TP592

CCCUGUCUCUUGGAAAAAAAGAGUCAGGCAUGGUGGCUUAUACCUGUAAUC

>TP593

AGCUACUCAGAAGGCUGAGGGAGGAAGCUUGCUUGAGCCCAGGAGUUCGAA

>TP594

CAGGGAUACAGUGGUAUGUGCCUAUAGUCCUAGCUACUCAGAAGGCUGAGG

>TP595

GUGGUGGGGAUUGCAGUGAGCCUAGAUCGUGCCACUGCACUCCAGCUUGGG

>TP596

UGGCUGGCCAACAUGCUGAAACCCCAUCUCUUAAAAAAUAGAAGAAAAUUA

>TP597

CUAGCACUGCUUGUCAUAGUUACAAACUGGAGCUAACCCAAAUGUCCAUCA

>TP598

GGCUGGCCUUGAGCUCCUGGGCUCAAGCUAUCUUCCUGCCUCAGCCUCCUU

>TP599

UGCUGCCUCGACCUCCUGGGCUCAAACAAUCCUCCCAUCUCAGUCCCCACC

>TP600

CACCUGUGAGUUCUUGCAGGUGGCGACUUCAUCUGCAAGGUCACUUACAUA

>TP601

CACUUGAGGAAGAAACAGAAACCUUAUUUCUUCCUGUGUGUCCCCUAGUGG

>TP602

GAUUUGGUUACUGGGGCUAGGAGGAAGGGAAAAUGGGGAGUUACUGCUUAA

>TP603

UUCACUUAUAUGAAAUAUGCAUAAUAGGCAAAUCUGUAGAGAUAGAAAGUA

>TP604

UAGGAAUUCAGAAGUGGCCCAGGCUAGAGUGAAGUGGUGAUCACAGCUUAC

>TP605

UGUAACUCCAGCUACUGGGGAGUCUAAGAUGGGAGGAUCGCUUGUGUCUGG

>TP606

AGAUAAAAUGUGGUGUAUACAUACAAGGGAACAUUACUCUGCAAUAAAAAG

>TP607

CAAAUGUUCACACAAAAACUUGUACAUGAAUGUUUAUGGCAGCAUUAUUCA

>TP608

CCAGGUGCCGUAGCUCAUGCCCAUAAUUCCAGAACUUUUGGAGGUUGAAGC

>TP609

AGUACCAGGCAGUGGGUAGAUCAUCAUUUGAGGAGACACCAAUAAAAAGAC

>TP610

UAAAAUUAUGUACCACACAGUUAUUAAGUACCAGGCAGUGGGUAGAUCAUC

>TP611

UUGCCUGAUUCCAGGCUGAGGUAGUAGUUUGUACAGUUUGAGGGUCUAUGA

>TP612

CGUGGUGGUGCAUGUCUGCAUUCCCAGCUACUUGGGACGUUGAGGCAGGAC

>TP613

UUCUUUUAUUAUUAUUCUUUUUUAUAGAGACAGGGUCUUGCUACAUUGCCC

>TP614

GAGUUUAGGGACUAGGAGAGAGUUUAGAUUAUCUAGCAUAUUCUCCCCCUG

>TP615

CUUAUGCCCAUGUGCCUGCCCUUCCAGCGGGCCAAGGUGUUUUCCCGGCAU

>TP616

UGAGGUGAAAGGAUUACUGGAGCCUAGGAGUUCGAGUCUGCAGUGAGUUAU

>TP617

AACAAAGCUGCCAUUGCAGCAGAAAAGGAAGCUCUGAACUUGAAGUUACCC

>TP618

AAUUUAAUCUAAAAAAGUAGAGACAAAGUCUCACCAUGUUACCCAGGCUGG

>TP619

CUCGGCCUCCCAGAAUGCUGGGAUUACAGGCGAAAGCCACCUCAACCAGCC

>TP620

UGUUUCUUAAAACACUUUUCCUACUAUUGUUUGACAUGGGAACUUUCGCCU

>TP621

CUCUGUCCCACUAGACCACAUCUCCAGGUGGGUGCCAUCUUCCCCUUUUUG

>TP622

GAUACCCUCCUGUCUCAUUCAGGUUACUCUGUCCCACUAGACCACAUCUCC

>TP623

ACCCCCCACCCCCACCCCCAUGGAUACCCUCCUGUCUCAUUCAGGUUACUC

>TP624

CACCUGGCUUCCCUUCUGCAUGGAUACCCUCCUUACCCUGCUCACUCUCUG

>TP625

CAUCCCCACUGGGCCCUGACAUUCUACACCUGGCUUCCCUUCUGCAUGGAU

>TP626

CUGCUGCAACUUUGUCCCCCAAGCUAAUAGUUUUCUCAUCCCCACUGGGCC

>TP627

UUCCCAGCAGUCAGAUGUGGUAACUAAGACUAGGACGGAUAAUAUAGGUUG

>TP628

CCAGUUGCUGCAGGUCUCAAUUCCCAGGAGGUCCCGUCGCUGGAGAUUCUG

>TP629

ACAACUCGGGGGUUGGGGGCAUGUUACUGGCAUCUAGCAAGCAGAGGCCAG

>TP630

CCCAUCCUCUCUCAGAUAAAAAUGGAACUUAUCUCUCAUGUCAAAUGUUUU

>TP631

GAUGUCCUGCGUGAUAAGGUCAAUGAGGAGAUGUACAUAGAAAGGUUAUUU

>TP632

CUUCCAAGUAUUAAUAGGCGGGACUACAGGCGUGCACCACCACACCAGGCU

>TP633

AGCUUGAGCAGCAGCAGCAGCAGCAACAGCAGCAGCAACAGCAGCAGCAGC

>TP634

GAACGCAGUGGUACAAUCAUGGCUCACUGCAGCCUUGAUGUCCCAGGCUCA

>TP635

ACUACAGGUACAUGCCACUGUGUCUAGCUAAUUUUUUUUUUUUGGCAGAGA

>TP636

GAUGCAGAGAAGCUGAGUCUCUCAUACAUUGUUGGUGGGAAUGUAAAAUGG

>TP637

CAGGUGUGUGCCACCAUGCCAGGCUAAUUUUUUUGCAUUUUUUAUAUAGAG

>TP638

AGCUGUCCUGUUUGUCUGUAAAAGUAGUUCUUUGUAAACAUUAGAGGAAAU

>TP639

UCACUGUGUACCCAUCUCUGAGCUAAGCAAUGCACAUAUACUAUCUCAUUU

>TP640

UAGCCUCUCUAAAGUCUCAUUUCUCAGGCCUCAGUUUUCCUCAUCUGUAAA

>TP641

AUCUCCUUAAAGACACAAAUCUUGUAUUUCAAAAGAAAUAUGGCAAAGAAA

>TP642

CUAGGAAUUUGAGCACCAGUGAAUGAAAAUGUUUCAUAAUAUAUAUUUUUC

>TP643

AUGUGCUUUUAUUACCUUUUUAUUUAGAGAUAGGGGUCUUACUGUAUCACC

>TP644

CUAUUGUGCCUCAUCCUCCCUGGUAACUGGGAUUAACAGGUACCCAACACC

>TP645

ACAAAAAAAAAUUUUUUUUUAAAUUAGCUGAAUGUGGUGUUAUGCACCUGG

>TP646

CACUUUGGGAGGCCAAGGCAAGAGAAUUGCUUGAGGCCAGGACUUCGGGAC

>TP647

AAGAAAAGAAAAAAAAGGGAAUUAUAUGAAAUUCAGAUUUCAGUGUCCACA

>TP648

UGAUCCUGCCACCUUACCCCACUUUAGGCAACAGAGUGAGACCUCAACCCU

>TP649

CAAUACCAUAAGAUUGUUUUGCAAAAAAAAAAAAAAAAAAAAAUGUCUAAA

>TP650

GAGACCACGAUAAAACCCCGUCUCUACUAAAAAUGCAAAAAAUUAGCCGGG

>TP651

AGUAAUGGGAUUACAGGCAUGAGCCACCGCACAGGGCGUGAUUCUAUUAUU

>TP652

UGCCCUUUGCUCUCCCGAUAAUCUUAAAUUACUACAAUUUAAUUGCAACUU

>TP653

CUUGGCCCCCCAGUGCCCUGGAAUUACAGGCAUAAGCCACCACGCCUGACC

>TP654

UUCUAACUCUUUCUCUCGUUGAAGAAGUUUAUCUCUGUAGUGUUCUACUUG

>TP655

GAAAAGCUAAAAAUAUAACUACCAUAUGAUCCAGCAAUCCCACUGCUAGGU

>TP656

UUAAAAUAACUUUUUUCCAAAAGACAGGCAGUAACGAUCCUGGUGAGGAUG

>TP657

AAGGUGCUCAGCAUCACUGAUUGUAAAAGAAAUGCAAAUCAAACCUACAAU

>TP658

UAUUUGCAAACUGUCCAUCUGGCAAAGGAUUAAUAACUAGAAUACAUAAGG

>TP659

AGACAAUCCACAUUAUGGGAAAAAUAUUUGCAAACUGUCCAUCUGGCAAAG

>TP660

GCCUAAACUAAGCAUAGGCAAUCAAAGCAAAAAUGUACAAAUGAGAUGAGA

>TP661

GGAAAACUGGAUAUCCUUAUGUGGAAAAAUGAAGCUAGAUCCCUAUCUCUC

>TP662

GAACUCAUUUUCAACAAAGGUGCCAAGAACAUACAAUGGGGAAAGGACAGU

>TP663

CAUAAAAACAGACACAAAUACCAGUAGAACAGAAUAUAAAACCAGAAAUCA

>TP664

AGACAUGUUGCACAAAAAGAACAAAACUGGAACAAUCACAUUACCUGACUU

>TP665

UUCAUUAUAUAAGGCCAUUAUCCUAAGUGAAUUAACGCAUGAACAGAAAAC

>TP666

CAGAAGAUACAAAAAUUGGCUGGGCAUUGUGGCACACACCUGUAGUCUCGU

>TP667

UCAUUUUAGGCACCAAAACUUUUGGAUUCAACUGAUCAAACCAAAGGAAAA

>TP668

AAGCGUUCAAGCUCAACACCCAUCGACUAAAAAAUCCCAAACAUACAACUG

>TP669

AGUUUAAAGUUCGAUGCAGCAGAUAACUUUAUACCCUGGUCAUCAGUGAAA

>TP670

AGGUAGGGUUUUUGAACACAUGUACACAAGGAGGGGAACAACACACACUGG

>TP671

GUGUUAAGCCCAACAUCUAUUAGUUAUUAUUCCUGAUGCUCUCCCUCCCCC

>TP672

CCACGCCCAGAGACACUGCAGACCAAACUAGGUAAAUAAUAUGUGGUUCCU

>TP673

UGGCCUCCCGUGUAGGUGGGACCACAGGUGCACACCACCUUGUCCAACUAA

>TP674

ACACUUGAGCCCAGGAACCUGAGUUAGAGUGAGCUAUGAUCAUACCACUAU

>TP675

CUGUUCAUAUUCUCACCAGCAUUUUAGUCACAAUCAUUUAAUCAAUCUCUA

>TP676

CCUGUGGGUCUGUAAAAUUAAAAUAAGUGAUUUACUUCCAAAAUACAAUGG

>TP677

AUUGAAUUGUGUCCCCUCAAAAUUUAUAUGUUAAACCCCUAUCAUCUAGUG

>TP678

GCCAGGCCCCGUGGCCCAUGCCUGUAAUCCCAAUCCUUUGAGAAGCCAAAG

>TP679

UGUGCCAUGGUGGUUUGCUGCACUGAUCAACCCAUCAUGUAGGUAUUAAGC

>TP680

GGCUUACCUAGGGGAUGGGUUGAUAAGUGCAGCAACCACCAUGGCACACAU

>TP681

AAGUCUCAAUUUCCAAGAACUAAUUAACAAUAUUAAUUGAGGACUUACUGU

>TP682

GGUAAAAUGGAUUUCAUUAUACAGUAAGUUGUUUCACUUAAAGUCUCAAUU

>TP683

AUGUUUUCAUUUACAGAGUGGCCUCAUUCUGAAUAGUCAGCAUUAUGAGAU

>TP684

UGUUUGAUAAUGUAUAGAUUCCCAUAGAUUUUGAUAGCUCUCAUAAUGCUG

>TP685

GGCAGGAGAAUUGCUUGCACCUGGAAGGUGGGGGUUGCAGUGAGCUGACAU

>TP686

CACUUGAGGUCAGGAAUUUGAGACUAGCCUCACUAACAUGGUGAAACCCCC

>TP687

UGAACAUAUAUACACGCAUACAUGUAUAUGUGUAUGUAUAUAUACACAUGU

>TP688

GUGUUCCUUAGACUCCGUUAUGGAUAAGAAGAUCAAGGAUGUUCUCAACAG

>TP689

AAAUAUAUUUUUAUUAAUACUUAAUAAAAAUAUUUUUUAAGUAUAUGUUAC

>TP690

CGUGAACCCUGGAAGCGGUGUUUGCAGUGAGCAGAAAUUGCACCACUGCAC

>TP691

GAGCCCGUAGGCGGAGCUUGCAGCAAGCCAAGACUGUGCUACUGCACUCUA

>TP692

GUACAGUGACUUAAGAAAAAUUAGCAGAUUCAAUAGGAUUUGAGGAACGAU

>TP693

AAAGAGAGAGAGAGAGAGAGAAAGAAAGAAAAAGAGAAAGAUAUAUAUAUC

>TP694

AGGUGUUUGACCGUGGAUUAGAAGAAUAAGCCCAGACGACCAUUAAGAGAA

>TP695

GGAACCUUAGUGGUCCUCUGGGGUUAUUCUUCUAACCUAUGGUCAAACACC

>TP696

AGAAUCUUAAACUUGGAGGAACCUUAGUGGUCCUCUGGGGUUAUUCUUCUA

>TP697

UAGACAAUUAUGCAUCCCUGUCAGAAAACAAAGAAUGAGGAUAAGGAAAAU

>TP698

CCUUUCAGCCAUGAACCUAUAGACAAUUAUGCAUCCCUGUCAGAAAACAAA

>TP699

CCUUUCUUCCUCUUCUACCUUCCUUAUCCUCAUUUUUUUGCUUUCUGACAG

>TP700

CUAUAUCAUCCCUAUUAUUUUCCCUAGAGUUGUACUAAUUUACAUUCCCAC

>TP701

UCCCACCUCAGCCUCCCAAAGUGUUAGGACUACAUACAUGAGCCACAGUGG

>TP702

AGUGUGAAAAUGGACUAAUACAGACAGGGCGUGGUUCUGUUGCCGAGGCUG

>TP703

AGCAGGAGCACAGUGGUGCCAUCUCAGCUCAUGCAACCUCUGCUUCCCAGG

>TP704

AACAACUUGAAAACAUGAUCAAAUUAGCCAAACUUCUGAUAGUUUUCAAUG

>TP705

AUAAUAAUAAAAAGCUCCUCACCAAAUUCAAGCUUGUACAUUAUAUUUUCU

>TP706

CAAGCAGCUGCUUCAACCCCCACAAAUGCCACAGCAGCGUCAGGUAAGAAC

>TP707

UUCAGUGAUUCAUAUUGCCAAAUAGAAGCUGAUCCAAAUGCACAGCCUGUA

>TP708

GGCCGGAGCCACCUUCGUUCCCUUGACCUUCUUUCCUUUCGGCAUCUUGGG

>TP709

AAGGUGGUGACGGUGUUAACUCCUGAUCGAAGGACAGGUGGUCUCUUGGUG

>TP710

GUCAGGAGUUUAAUCCAGGUCUCCCAAGAUUCAGGGGCAAAACUGGAACUG

>TP711

GUGUGUUGAAGGUCAGUGCUUCACUAGGAGGGCUCACAGGACUCAGAAUAU

>TP712

GGAGAGCUCUUACUAUGUGCCAGGAAUUGUUCCAAUUGCUUUCAUGUAUUA

>TP713

GAUGGUAGAACUGGGCUUUAAUAAUAACCAUUUGGAGAGCUCUUACUAUGU

>TP714

UCUUAAAAUGUAGUAUGAUGGUAGAACUGGGCUUUAAUAAUAACCAUUUGG

>TP715

GAAGGCAGCCAGCAAAGAUGGUCUUAAAAUGUAGUAUGAUGGUAGAACUGG

>TP716

ACAGUUGUGAACCAAAGAUAUAAGAAGGCAGCCAGCAAAGAUGGUCUUAAA

>TP717

AGCUCUACACUUAUACUACAUUUUAAGACCAUCUUGGUGGCUGCCUUCUCU

>TP718

AUAAGAGGUCUCCAAAUGGUUAUUUAAGGCCCAGCUCUACACUUAUACUAC

>TP719

UUAGAACAAUUCCUGGAGCAUGAUAAGAGGUCUCCAAAUGGUUAUUUAAGG

>TP720

AAUGAGUUAAUACAUGAAAGCAAUUAGAACAAUUCCUGGAGCAUGAUAAGA

>TP721

UUGUACAACCUAAGAUUGUUUUUUUAAGAUGCUUUUCAGACUGAUACCACC

>TP722

UGUGACUUUUCUAACUGUUCCUAUAAGUAAUAUCACUAUUGUACAACCUAA

>TP723

CCCCACGAUCCAAUCACCUCUCACCAGGCUUCUCCUCCAAUUUGACAUAAG

>TP724

CUCUGCCUUUCCUGGAAAGUUGCUCAGAUGACUCAGGCACUGUUCUCUGCA

>TP725

UGCAGAGAACAGAGCCUGAUCCCCUAAGCAACCUGCCGAGAACCAGAGGGA

>TP726

CCAGUUCCCAUGCUCAUUUGAAUUAAGGUUCUGUUUUGCUAGCUGCCUCCU

>TP727

AGGUAGAGUACUUCAAUGUCACAUCAGUUUUUCUUCUCCAGUUUGACACUU

>TP728

GGAAAUGCCUCUGAGAAAGGGGAAAAAAUGCCAGCUAAACUGGGGCUUACC

>TP729

AUCAAUUGAUGGUAUGCCCCAGUUUAGUUGGCAUUUUUUUCCCUUUCUCAG

>TP730

UUCACAUUUUAACAAUACUGAAAUAAGGUAGUGUACCUGUUAUCAAUUGAU

>TP731

AUAUUCCAGUGUAUGGAUAUAUCACAUUUUGUUUAUUCAUUCAUCAAUUGA

>TP732

GCACUUUACUCUCUUUAUGCCUGAAAAAUAUUCCAGUGUAUGGAUAUAUCA

>TP733

UCAUGCACUUAAAAUAUUAGUUUCAAGGUUCAUCCAUGUAGCAUGUAACAG

>TP734

CCUUGAAAACAUGACGCUGAGUGAAAGAAGCCAGACACAAAAGGUCACACG

>TP735

AAAAUAUAAUUGCGAAUAUCAGAAAACUCAUACUGGAAAACUAAAUUUUUU

>TP736

GGACCUUUUUAUUUAUUUUUGAGACAGGGUCUCACUCUGUCAUCCAAACCG

>TP737

AAUGAUGAGCAUUUGGAUUCUUUCCAAUCUUUUGCAAUGACAAUCAGCUCU

>TP738

AUUUGUUCAUUUUUUUCAUUGUAUAAAUGUGUUAUAAUUUAUUUCGCCAGU

>TP739

AAGAAGGAAAAAGACAAAAAAGGAAAUCUGGAUUCUGAGGACCACGUCUCA

>TP740

CAAGCCUACUGAACCAGGAACUCUGAGGAGGGGCCCAGAAGUUUGUGUUUA

>TP741

GUUUUGCAGGUGGUUUCUGUACAUCAUCUUCGUCUUCCUCCUGGGCGCCAU

>TP742

AGGGCUGUUUAAAAAAAAAAAAAAAAGGCCGGGAAACUUUAAAAGUAGGCA

>TP743

AAUCAAGAAACAGGUCAGACGUGGUAGUUCAUGUCUGUAAUCACAAUACUU

>TP744

CUACCCUCAUGUCCUAAUCUUUCCAAAGGCCCCACCUCCUAAUACCAUCAC

>TP745

ACUGAGUGGCUUAUAAACAAUAGCAAUGAAUUGCUUACAGUCUUCGAGGCU

>TP746

CUGUUGUUUACAAAUCACCCAGUCUAUGGUCAUUUGUUAUAGCAGCCUGAA

>TP747

CAGAAGUCUCCUUUUUUCCAGCCAUAUGAAGAUACAGUGAAACACAGCUGU

>TP748

CUAAUUUUAUAUUUUUAGUAGAGACAGGGUUUCUGUCUUCAUAGAUUUGCG

>TP749

AGCUAAUUUUUAUAUUUUUAGUAGAAACACGGUCUCGUCAUGUUGCCCAGA

>TP750

ACACAGCGUGACCGAUUUUAUCUUCAGUCAGCUGAUACACCUCAUGGGGUG

>TP751

UCUUGGACGCCCUCUGUCCAGUCAGAAGCAGCCCUUGGCUGGGUGAGGUGU

>TP752

UUUGUAUUCUGGGGAGUCCUGGCCCACUAUCCACUGCCAGGGAUAACCUGG

>TP753

CCCAGCCACAGCUUGAUGCAGACGUAGCUGGGGGCAGCCAUGAGAGAAGAG

>TP754

UGGGGCUGAGAUGCACCAGCCACAUAGCACUGCCAAGGCCUGGGGCCUCAG

>TP755

CUGGUGUCCACCAGGGGAAUCCAUGAGGCCCAUGGCCACCCAGGGAAGGCU

>TP756

UCUCUCCAGGCUCCUGUGCCAGCCCAGAGACCACAGGGAAGGUCAUGCUGA

>TP757

UCUCAGAGGUCCCACCUGUCUCUCCAGGCUCCUGUGCCAGCCCAGAGACCA

>TP758

ACAGGUCUGAUGGGCAAGCUUGGCAAGGGUGGCUGGCAAGGUCCGGGGAAG

>TP759

CCCAGUGCUCUGCCUGCUGGGUGGCAUGGAGUUCUGCUCCGGCCCUCUCUC

>TP760

AAGGGGGAGCAGGAGGGAGGAGAGAAGGCCUGAGAGGCAGGUAGGGGCCAG

>TP761

CCUGCAUAGCAGGGAGGAGUUGGCCAUGAAGACCCAGGGGCCCGUGUUUCU

>TP762

CGUGGCCGCUCCCUUCGGAGGAUUCAAACAGUCUGGAUUUGGCAAAGAUCU

>TP763

AAAUUAGCUGGGCACGGAGUCGAGUACUUGUAGUCUCAGCUACUCAGGGGA

>TP764

CUUUGUUACUUAGUGCAUUUUGUCCAGUUUUUUGUUCAAAACACCAAGAAC

>TP765

AUCUGCUAUGUAGUGCAAAGGGAGUAGCCUCUGAUCCUUUUGUUACUUGGG

>TP766

CCUAGCUACUCAAGAGGGUGAGGCAAGAGCUUUGCUUGAACCCUGGAGUUC

>TP767

AUUCCUGAACUCAGGCAAUCCUGCCACCUCAGUCUCCCAAGUAGUUAGGAC

>TP768

CCAAGUGAAAUUUUUUUUAGAGACAAGGUCUCUUGCCCAGGCUGUCCUCGA

>TP769

AGGAUGGGACCAGCUGUGGAGAAGCAGCCUGCUGACAGCCACAGCCUGCAG

>TP770

GUGGUGUCAGUCUACAUGCAUUGUAAAGUUGCCCAAAACUUUUCAUGUGGU

>TP771

UAAUAUUCCACUGUGUGGAUACUUCAGUUUGCUUUAUCCAUCCGGCUACUG

>TP772

UAGGAAACUUACAAUCAUGGUGGAAAGUACCUCUUUAUAGGGCAGCAGGAG

>TP773

GGGAAUUUGAUACCAGCCUGGCAACAUAAUGAGACCCCGUCUCUUAAAAAA

>TP774

UUCAGGCAGGAAAAGAAUACGUUGAACACACUGUGAAAGAGCGAAAAAAAC

>TP775

GAGACAAGGUUGCACCAUGUUGCCCAGACCAGUCUCAAAUUCCUGGGCUCA

>TP776

CUGUAGCCUCGACUGCCUGGCGUCAAGUGAUCCUCUUUUACUUCAGCCUCC

>TP777

CCGAACCAGUGGCCACCACCAUGACAAGGGACCAGAAUGGAACCUGGGAGA

>TP778

GCUCCAUUUAGUGUCCUCUUGAUAAAGUCCGUAGUGAAUCUGCAUCAUAGU

>TP779

AAGAACUGAAGAUAUUGUACAGAUAACAGAUAUAGUCUAAUUACAAAGAAA

>TP780

AGAUAACACAGAAGCAGAUGGGAGAAAAGUCUGGGGAGAAACAACAUGAGC

>TP781

GCCAACGUAGCUGAUGCUCAGGGUCAGGCCUCGCCCCUUCGCCGCAGGUCU

>TP782

AAAAUCAUUAGAAAAUACAAAAAUUAGCCGGGCGUGGUGGCACAUGCCUGU

>TP783

UGUUAGGAAACCAUGGGCUGGGUGCAGUGCCUCUCGCCUGUAAUCGUAGCA

>TP784

ACAGCCUGUACCUGAAGGUGAAGGGAAAUGUGUUCAAAAACAAGCGGAUUC

>TP785

GAAGAGGCUCGCCUCUUCUGUCCUCAGCUGUGGCAAGAAGAAGGUCUGGUU

>TP786

GGUGGCUCACGCGUGUAUUCCUUGCACUUUGAGAGACCAAUGCAGUUGGAU

>TP787

AGAAAGAGAUUCAUAUUCCUGUAAGAAAAAUCCGGUAUGUAAAGUUUAUUU

>TP788

GCCGGGCAUGGUGGUUCAUGCUUGUAAUCCCAACAUUCUGGGAGACCAAUG

>TP789

UUCCCCUGGCUGAUCUCGAACUCCUAGACUGGGGCCUUCUGCCACCUUUGC

>TP790

GGAGGCUGAUGUGAGAGGAUUGCUUAAACCUGGUAGUUGGCUACAGUGAGC

>TP791

CAGCUAAGCGUGGUAGUACCCACCUAUAUUCUCAGCUAUUCAGGAGGCUGA

>TP792

GAUAUUACUCGAAUUUUUUUUUUAAAGAGAUGGGGUGUCAUUUUGUUGCCC

>TP793

CUCAUUAAGUGAGUAAUAGCUCUGUAGUGAUGUAGUCUCAAUGAGCUAAAC

>TP794

GAUCCUGAUGAACUCUCUCUUUAGUAGACCUCAUUAAGUGAGUAAUAGCUC

>TP795

AGAAAUAGCCUCACAAGGAUCCUGAAGAACUCUCUCUUUAGUGGACCUCAU

>TP796

UCUGAUUUUCUGGAUAUGAGAAAUAACCUCACAAGGAUCCUGAUGAACUCU

>TP797

UUAAGUAACGUGAAGUUUGUGCUUCAAAGGGUACUUCCCUUGAUCUGAUUU

>TP798

GUUGCACCCUUAAUCAGGAUUUUUUAAAUUGCAUAAUACUGACCAUCUAAU

>TP799

AGGAUUACCUGAACCUGGGACGUCAAGGCAGUGGUGAGCUGUGAUUGCACC

>TP800

AGUAUUCAAUAAAUAUGUUUUUAAAAAGCAGACAAUCCUAAUGGUCAAGAG

>TP801

CCCUCUCAACUAGAUUUUCAUUAGUAUUCAAUAAAUAUGUUUUUAAAAAGC

>TP802

AAAAAGUAAGGUAUCAAAUACUAAUACACAUCUAGGUCAGGGAUCAGCAAA

>TP803

AAAACACUUGAUUUGUGUCCUCUUGACCAUUAUGAUAGUCUGCUUUUUAAA

>TP804

GCAUUAUAGACAGAGGGAACCUCACAGGUAGAAGUCCUGGGCUGGAGGGAA

>TP805

GGAAGUCUCAAAUCAACUAGUCUAUAGACGUGUGGGUGAGUGGGUUGGUGG

>TP806

ACCCAGGAAUAUAAUGUUAACGAUGACUCUAUGAAACUUGGAGGAAACAAU

>TP807

CUUAGAGUGGAAGGACUGAUUGAGAAUGUUCCAAUCCAAAUGAAUGCAUCA

>TP808

UCAAAUAAACAAUAGGUAAAUUUUUAGUUUUAAGUAUAUCCCAGAUAUUGC

>TP809

CACAGAACUUCACGCCUAUAAUUCCAGCACUUUGGGAGGCCAAGGCAGGCA

>TP810

GGAGGAACUUUUUAAAAAUGCACAGAACUUCACGCCUAUAAUUCCAGCACU

>TP811

UUCCUCAGGCAGGAAGCCACAGGAAACAAAGUAUUGUGGUGCAUAUUUCCC

>TP812

UGAAGAUACUCAUGAAGAACAGUGAAGAAACAGAGCUUAGAACCAAACAGU

>TP813

UGCUUUGUUGUAUUCUACUUUCUCUACCUCAAACAGGAACAAAAGUUCUUU

>TP814

UUAGGUAAACGAAAGCUGUGUCUUAAUUGAUUUAUUCUUUAAAAAUAAAGU

>TP815

AAAAGCUUCUAAAUGUCUAAUAUAAAGGGAGAUGCUUAUAGCCACAACAUC

>TP816

GACUGGAGUGCAGUGGCAGGACCUCAGCUCACUGCGAUUUCCACUUCUCUA

>TP817

CGGGAGAUUGUGGCUUCAGUGAGCUAUGAUCCAAUCAUUGCACCCCAGCCU

>TP818

GGCCUCUUCAACCAGCUGCCCCUAAAGCUAGCAAGCUCCACCCUUCAGUGU

>TP819

CCCCUCCAGUGUCCCCGGAGCUGAAAGAUCGCAAAGAGGAUGCGAAAGGGA

>TP820

AAAGAAGAGGCAGAGCUUAAAACAAAUACUAGGAUUCCAGCCUGUAUCUUC

>TP821

AGGCAUUUCUGAAGGAAAUCAUGGCAGUCUUCAGCGAGGCAAAGAAGAGGC

>TP822

UUAAGCUCUGCCUCUUUUUUGCCUCAAUGGAGACUGCCAUGAUUUCCUUCA

>TP823

GGGCUGGAAUCCUAACAUUUGUUUUAAGCUCUGCCUCUUUUUUGCCUCAAU

>TP824

CCUAUCAAGCCUGCAUUCACAAAGUAUUUUGGACUAAUCUUUUCAUCUCCA

>TP825

UUUAAUAACUUGCCAUUUCCUAUCAAGCCUGCAUUCACAAAGUAUUUUGGA

>TP826

AAGGAGGGAUUCUACCCAGAGUCUCAGAGGAAGCAUGGCCCUGCUGACACC

>TP827

GCUUUAAAAAGUUUUGAAAGCCUUUAGACUAAAUGAUGUUUAAGACCUCUU

>TP828

UCGUCUUUCCUCUUACAGAUUAUAUACUUCCUGAAGAGCGGAAACAUUUCA

>TP829

ACUACCUACUAUGUAAAGGUAAUGUAAAAUGUUUCUGCUGUUCAGGAAGCA

>TP830

AGCACUGAGAUAUAUAUAUAUAUAUAUGUGUGUGUAUAUAUAUAUAUAUAU

>TP831

UUAAGUGUGGUGCUGCGCUGCUGUAAUUCCAGCUUUUCAGGAGGCUGAGGC

>TP832

UACUAAAAAUAAAAAAAAUUGUUUAAGUGUGGUGCUGCGCUGCUGUAAUUC

>TP833

CAUUAAAGACCAGCCUGGGCAAAAUAGUAAGACCCGGUCUUUACUAAAAAU

>TP834

AGGCUGGGCAUGUUGGCUCAUGCCUAUAAUCCCAACACUUUGGGAGCCCAA

>TP835

ACAGCAGUAGCAACAGCCUUUAAAUAGGGCCUUGUUACUGUGAUACUUCCU

>TP836

UUUAAAAGAUAUAUGACUCUAUAUUAGUCUGUUUUCUUGCUGCUGAUAAAG

>TP837

GUUUGGUUACAUGAGUGAGUCCUUUAGUGGUGAUUUCUGAGAUUUUGGUGC

>TP838

UCAGAGGACCCUCUGCCUUUUUGCAAAGGCAGAGGGCCAAUGUGACAGCUU

>TP839

CUCCAGCUUCUGCACCUGUCCAUCUACAUGCUCCCCAUCCCAUAAGGGGUU

>TP840

GUGAAGAAUGUCAUUGGUAUGUGAAAAGGGAUUACAUUAAAUCUGUAGAUU

>TP841

GAUUGUUUUUUCUAUUUCUGUGAAGAAUGUCAUUGGUAUGUGAAUAGGGAU

>TP842

GGAAUAAUCCUAAAUUCAGAAAUAGAUACCAAUGGAGAGACUUCCGACACU

>TP843

GAAGUCAAGGAGGCCCUGAAGCAAAAAGAGGAAAUGCUCGAGAAACAUGGA

>TP844

AAAAACACGCCCACAGUAUACUGCAAUUUCAGUUUGCUGAAGUCAAGGAGG

>TP845

GCGGCAGUACGAAGAGAAAAACAAAAAAUUUGAAAGGGAAAAACACGCCCA

>TP846

CACCCUGAAAGAUAUGUUGCUGGAGAUUGAAGAACAGCUGGCUGAAUAUAG

>TP847

CCUCUAUAUCCAUCGACACCGAGGCAUCCAUCAGGGAAAUCAAGGACUCUC

>TP848

AGAGAGGCAGCGGAGACACCUCUAUAUCCAUCGACACCGAGGCGUCCAUCA

>TP849

AAGGGGUCUCGUAACAUGCUGGGCCAGUCUGCAGCCACGCUGGCCUUUCUG

>TP850

GGAGGUAGAAGAGAGACCAGAAAAAAAUUUUACUGAGAAGGGGUCUCGUAA

>TP851

AUAGCUGGACUUUCCUUUUCAUUUCAUAUUUGGAACUAAGUUUGUAGCGUA

>TP852

UCAGAGUGUAUUUUCUUUGUGUAGAACAGAAUGUACACAUUAUAGCAGCUC

>TP853

CUCAGUCUCCUAAGUAGCUGGAACUACAGCUGUGCGCCACCAGAUCUUGCU

>TP854

UUAGGCUGGAGUUAGUGAUGUGAUCAUGGCUCAUUGCAGCCUCAACUUUCU

>TP855

UAGGCGUGAGCCACUGCGCCCGGCCACUUCUGAAUUUUUGAAAGGUAGAUU

>TP856

UGAGAGAAUCAAUUGCGAUAUGAUGACUAAUCCAGGUAUUUUAAGAUGGUU

>TP857

GAGUUGUCUGGGCACAGUGUCUCACACCUGAAACUUUGGGAGGCCGAGGUU

>TP858

GAGAUAAUAGAACAACUUCACAGACAAUUUGCCAUUCUUUCAGGUAAGGGG

>TP859

CCAGUGAACCCACCACAUUUCAGAAAUAUCACCAAGUGAGUCUACUUGCAA

>TP860

AACAAAUACAGAAUAAAAACAUUUAACAAAAUUAAACACCCAUUCACAAAA

>TP861

UUCACAUGGUCAACUCAACAAAUACAGAAUAAAAACAUUUAACAAAAUUAA

>TP862

CCAAGCCCACAGACAUCCAGAAUAGAUGUUGAGCCUUGUAAUGUCCGUCUU

>TP863

GUAUAAUUCAAGAUGAGAUUUGGGUAGGGACACAGCCAAACCAUAUCACUG

>TP864

CCUGAUAAAACCAACAGAUCUCAUGAGACUUAUUCCCUACCACGGGAACAG

>TP865

AGGGACCCUGGGCCCAGCCCACAAAACCACUUUUUUCUCCUUGGCCUCCAG

>TP866

CAGCUCUACUAGGCAGUGCCCCAGUAGGGACUCUGUGUGGGGACUCCGACC

>TP867

GAGCCUGUAAAAUCAAAAGCAAGCUAGUUCCUUCCUAGAUACAGUGGGGGU

>TP868

UAACUCAUUUCAGCAUUAACCCAAAAGUCCACAGUCCAAAGUCUUAUCUGA

>TP869

AAAUCUCAUGUCCUCAAAUUUUAAAACCAAUUGUGCCUUCCCAACAGUCCC

>TP870

AUUAGCCUGUUUUCACAUUGCUGAUAAAGACAUACUCUAGACUGGGAAGAA

>TP871

UACUUAAAAGUGACAGCUUUUGGCCAGGUGCGGCGGCUUAUGCCUGUAAUC

>TP872

UAUAGUGAAACCCCAUCUCUAUAAAAGCACAACAAAUUUGCUGGGCGUGAU

>TP873

AGAUGAUAGACAAAGCAACACAACCAAGUUUGGACAUUCCAGUUUCGACCA

>TP874

UGACCUGUUUCUACAAAAAUUGAAAAGUUAGCUGGGUGUGGAGGCGUGUGU

>TP875

CCUAGAUAUGUUUAGAUACACAAAUAACAUUGUGUUAACAAUUGCCUACAG

>TP876

ACUACACAUCUAGGGUAUAUGAUAUAGCCUCUGGGUCCUAGGAUACAAACC

>TP877

UGUAUUUACACAACUUAGAUGGUAUAGCCUACUACACAUCUAGGGUAUAUG

>TP878

GCCAGGUGUGGUGGCACGCAUCUGUAGUUCCAGUUACUUGGGAGGCUGAGG

>TP879

AUCAUGGCUCACUGCAGCCUCAAGCAGUCCUCCCCCUUCAGCCUCCAAACU

>TP880

CUCUAUAUAUUUUUCUGGGCAUGGUAGUUCAUGCCUGUACUUUGGGAGGCU

>TP881

AUAUUGAGGCAGAAAGUGCAAAUUUAGUUUAUGUUAUUUUAUUUUCGAGAC

>TP882

UAGGAAACAAAAAGCUAUUUUUUGUAGAUACCCUCAAAGUUGGGUUAUGCA

>TP883

UGAGAGCCUCCUUGGCUUCCUCAUUAUCCAAACCACUGGCCACUUCAGGCC

>TP884

UCUUUCAAACUUGGGGUUUUUUUUGAGAUGGGGGUCUCACCAUGUUGCCCA

>TP885

CAGUGGCUCGCUGCUUACCCAGGUUACUGCUGUCUUAACUUCCUGGGCUCA

>TP886

UUAUAUUUACAAGAUAAAGUGAAGAAGGGAACAGAGGCCCAAGAUGGAAAA

>TP887

UGUUGAUCUCAUGGAGGUAAAGAGUAGAAUGAUAGUUACCAGAGACUGGGA

>TP888

UAUACAAAUGGUGUGGUAUGGUGGUACAUGCCUGUAGUCCCAGCUACUCAA

>TP889

UGGCUGGACGUGCCUGGCAUGCACCAGAUUCUCUGGAGGUUGUGGGCAGGG

>TP890

UUACAGCUUCCUCAAAUUCAACUUUAGCUCCUCCCACAAAACCAUCUCCUG

>TP891

GGACACGUGCCCCUGACACAGCCUUAGGAAGUCUUGAUGACGUGUGCCCAA

>TP892

AGCUGCUUGCCCUGAAGCAGUUUGUACAGGAAAGGCCGGCCAUGGGCUUGG

>TP893

CUUCAAGAACUUUUCUUGGCUGGGCACAGUGGCUCAUGUCUAUAAUCCCAA

>TP894

CACCCACAGUCCCACUGACUCUGGAAGCCGAGGCUGGUGGAUCACCUGGUC

>TP895

UUGUUGAUGAUGUUGUUUUUGAGACAGGGUCUUGGUUUGUCACCCAGGCUA

>TP896

GUGUAGACGCGCACCCACAUCCCCCAGGAGGAAGCUCACGGGAAAGCACCU

>TP897

GGCUAAGCAGUCCUUCCAUGUAGCUAGGCCUCUAGGUAUGUGCCACCAUGC

>TP898

AUAAAAGAAGGGAGUCAGGUGUGGUAGUGUGCACCCACAGUUCUAGCUACU

>TP899

ACCACUGCACUACAGCAUGGUGGCAAAGCGAGACCCUGCCUUUAAGAUCAU

>TP900

GUGUGUUUGUGGUUCCGGCCACUCCAGAGAGUGAGUGGGGGAGGAUCACUU

>TP901

CUUUUUUUUUGGGGGGGGUUUUUUGAGACAAGGUCUUGCUCUGUCACCCAG

>TP902

ACAGUCCUCCCACCUCAGCCUCCUGAGUAGCCGGAACUACAGUCACGCACU

>TP903

CUCCUGAGUAGCCGGAACUACAGUCACGCACUUCCAUGUCCAGAUAAUUUU

>TP904

ACUACAGUCACGCACUUCCAUGUCCAGAUAAUUUUUUUUUUUUUUUUAGAG

>TP905

CUCGGCUCACUGCAACCUGCAUUCAAGUGAUUCUCAUGCCUCAGCUUCCCG

>TP906

GGCUGGUCUCGAACCCCUGGCCUUAAGUAAUCUGCCCAUCUCAGCCUCCCA

>TP907

CUCAUUUUACAUUUCCACUUGUUAAACUGAAAACUGGCCCGAGAAAGCUUC

>TP908

AAAGGUUCUCUGAAAACUAAGGAAAAUAGGCAGGGUGUGGUGGCUCAUGCC

>TP909

CAGCUUCCUGUUUGGAUACCCACUAAACAUUUGAAGUUCUACAAUGAACCC

>TP910

UGAAGUUCUACAAUGAACCCAUCAGAGAUGCAAAUGAAAGUGCCUCUACAG

>TP911

CAGAGAUGCAAAUGAAAGUGCCUCUACAGAGACAGAAAACCCGCAAUCGAG

>TP912

GCACAUGUGGAGAGUCAGGGAGAGGAAGAGAAAGAAAAAGAGACAGAGAUC

>TP913

UAUGCAAAGGAAGUUAGUAAAGCACAGAAUUCGGCACACGGGGGAGCGGCC

>TP914

UUCAAAUCUCAACUUGUAUCUCCCAAAAUUCCCACGUGUUGUGUGAGGGAC

>TP915

CCUCACAAUCAUGGCAGGAGGUGAAAGGCACAUCUUACAUGGUGACAGACA

>TP916

GGAGAAGCCAACAUGGGGUACGCUAAUGCUGGGUCACCACUGUGGCCCCUG

>TP917

GUGUCCUGCUGCGGCUCUACCACCUAUAUGAAGUGGGCGAGGACCCAGUCC

>TP918

CCCUUUAGAGCAUGUGGGCUGCAUGAGGCUUGGUUUCUGUGUCAAGAAGAC

>TP919

GCAACCUACAAUUGGGAUGAGUUCUAGGUACAUGUGUUUGCUGACUUUGGG

>TP920

UAGAGACAGCCAUGGGCAUGAUCAUAGACGUCUUUUCCCGAUAUUCGGGCA

>TP921

UCCUGCCUCAGCUUCUCCAAUAGCUAGGAUUAUAGGCACACACCAUGGCAU

>TP922

AGUUCCUGGGACUUGCUGCUUCAUGACCAGCGUCUCGGGCCCCUGUCAGCU

>TP923

CUGGAGCUCCUUAAGGACAGGGCCUAGAUCUCAGUCAUCCCCAAUCCCCAG

>TP924

UCACAUUUCACCAAGCAGGGGCAGCAGAGCCUGUAUUAGACAGCAGCAGGG

>TP925

CUGGCAGUGACUACGGGCAACCGCAAGGCGGAGCUGCGGCUGUGCAACAAG

>TP926

AUGAAUAGUUCACCCCUUGUUUAGCAUAUGAUCAAGAAAUAACCAUGAAAA

>TP927

CUACAUUAGGCUAACCUCCCAUUCUAUUCCUAAAUAAGAUGGCUGCAUAGG

>TP928

CUUCCAGUCUUUGCAGCUGUCUUAUAUAGGGAUAAAAUGGGAGGCAGGUUG

>TP929

GCGGGGUAUGGUGGCACACACUUGAAAUCCCAGCUGUCUGAGAGUCUGAGG

>TP930

CUUGAGCUCCUUGAGCCUGGAAGGCAGAGGUUGCAGUGAGCCGAGAUCAUG

>TP931

GGAGCUGGGCAUUUAGCCCUGUAUUAGGCCAUUCUUGCACUGCUAUAAAGA

>TP932

UCAGGAAAUGUACAAUCAUGGCAGAAGGUGACGCGGGAGCAGGCAGUUCAC

>TP933

GUGCAGGAGGAGAGAGAGAGGCAGGAGAUGCUACACGCCUUUAAACAACCA

>TP934

UUAUAAUUCCACGUGAGAUUUGGGUAGAGGACACAGAUCCAAAAUAUAUCA

>TP935

GACAGGUGAUAUGGUUUAGAUCUGUAUCCCCACCACAAUUUCAUGCUGAAU

>TP936

AAUUGUAGUCCCCAAUGUUGGAGGUAAGCCCUGGUGGGAGGUGUUCGGGUC

>TP937

ACAAGAUCUGGUGGUUUAAACGUGUAUGGCACCUCUCCCCACCCCCACUCU

>TP938

AAAAGCUCCCUGGAGCCUCCUCAGAAGCUGAGCGAUGUUGGCGCCAUGCUU

>TP939

AAAUAGGUCCAGAACACACAGGUUUAAAUUCUGCUAGGGUCACUAGUUGUA

>TP940

CUAGUUGUAUAACCUUAAAUACAUUACUCAAACUUCCUGAGCUUUGCUUUA

>TP941

CAGAGUGAGACCUUUCCUCAAAAAAAGAAAAGAAAAAGAGGCCAGGCACGG

>TP942

AUUGCUCUGUUGCUCAGCCUGGAAUACAGUGGCAAAGUCGUACUUCACUGC

>TP943

CUGGUGGGAGUGGUGGGGAUGCUGAAGUGGGAGGAUCGCUUGAGGCUGGGG

>TP944

GAACUGAAGUGUAAAGAUUGGCAAUACAGUGUCAAAAUAAUAGCUAGAUGG

>TP945

UGCAGAUAAAGAAACUGAGGACUCAAGAGAUUACAGCCUGGGCAACAAGAC

>TP946

AAUAGCUGGACACGGUGGCGUGUGCAUGUAGUUGCAGCUACUCAGGAGGCU

>TP947

UUCAGAAGACACUGGAUUGGGGUUGAGAUUUGGGGGAGGAGCCUGAUGCAG

>TP948

CUGCAACCCCUGCUUCCCGGGGUCAAGAUAUUCUCCAGCCUCCGCCUCUUG

>TP949

CUUGAACCCGGGAGAUGUAGGUUACAGUGAGCCGAGAUAGCAGAGAUCGCA

>TP950

GAGCCCAACAGGUUGAGGCUGCAGUAAGACAUGAUCAUGCCACUGCGUCCC

>TP951

GUCUGAUCUCAGCUUACUGGGUUCAAGCAAUUCUGUCUCAGCCUCCCGAGU

>TP952

AGGAUCUGGCUCUGUUGCCCUAGCUAGAGUGCAGUGGUGCCAUCAUCACAG

>TP953

UUUCCUGGCAACUUGCAAUAGCUUAAGCCGGUAAUCCCAGCGUUUUGGACG

>TP954

UACAGAAUAUUUAAAAAAUGAGGCCAGACACGGUGGCUCACGCCUGUAAUC

>TP955

GUGGUGGUUUACGCCUCUAAUCCCAACAGUUUGGGAGACUGAGGCGGGAGA

>TP956

AGUUUUUUAUAUUAAAAUAGAGACAAGGACUUGCUAUAUUGAGCAGGCUCU

>TP957

AAAGGCUGGUUUAGAGUAAUUGGCUAGUUGUCGUGGCUCACACCUAUAAUC

>TP958

UUUAGCCUGAUUUUGGGGUCUAUAGAGAUUGCUUUAUUGGAUACUUCAAGU

>TP959

AACUUCAUCUCAAAAAAAAAAAAAAAAAAGAAAAAAGAAAAAAAAAAAGCA

>TP960

GCUUUAUUGUCUGGGCUGGUCUCAAACUCUGGACCUCAAGGAAUCCUCCCU

>TP961

AUGGUUAGUUAUCAAUCUGAGGCCAAGGGUGGUUGCUCAGGCCUGUAAUCC

>TP962

UGUGAAAAUGCUAUGAAUAUGGGUCAGGAAUAAGUCCUGUGUUCUCAUUCA

>TP963

AUCUAACCCUGAUGGGAUGUGUAUUAGUUCAUUUACAUACUGCCAUAAAGA

>TP964

CUCCCUGUCCUGACAUGUGGGGAUUACAACUUGAGAUGAGAUUUGGGUGGG

>TP965

AGAGACAAAUUUGAAGGUUAAAUUUAUUCAAUGGGCCAGGUGUGGUGGCUC

>TP966

AGUUCAAGACCAGCGUGGGCAACAUAGAGGGGCCUUGUCUCUACCAAAAAU

>TP967

CCAAAAUGCUGAUAGCUAGGACAUGAACAGAAUGCACCCAAGCACUUUGCU

>TP968

CAGAAUGCACCCAAGCACUUUGCUAAGGCAAAACAUGCGUGACCUUUGCUC

>TP969

GCAAAACAUGCGUGACCUUUGCUCUAGUUCCCAAUAAUUUCCUUAUUUCCA

>TP970

GCUGCUGAGGCCUCAGAUGGAAAUUAGGAACUUACUGGGAACUAGAGCAAA

>TP971

AUGUUAACGCCUUAGCAAAGAGCUUAGCUGCCUUCUGUUCAUGCCCUAGGA

>TP972

ACAUUUCAUUCAAAGGGUAAAACAUAGACUCCCUUUGUUCUAUAAACCUAC

>TP973

GUAAAACAUAGACUCCCUUUGUUCUAUAAACCUACAAACAUGUCUGAGCAU

>TP974

UGCUCAGACAUGUUUGUAGGUUUACAGAACAAAGGGAAUCUAUGUUUUACC

>TP975

UAGGUUUACAGAACAAAGGGAAUCUAUGUUUUACCCUUUGAAUGAAAUACA

>TP976

CUCACUGCACUCUUACAUUGGAAGAAGAUGCCAUCUAAGACUUUCAGAGCU

>TP977

CACAGAGGUCUCCAGUGCCAUAGAGAGAGUACAACUUUGAACCCCAAAGCU

>TP978

CUCUGUCAUUUUCUUUGGUCUUUUAAGGGAGGGAGGCCUUUCCCUGCUUUU

>TP979

CUCCCAAAGCCCUGGGAUUACAGGCAUGCAUCACCAUGCCUGGCCAGUGAU

>TP980

GUCUUGUUUUUAUUUUAUAUAUAUAAUAUAUAUAAAAAAUAAUAUAUAAAU

>TP981

UAUAUAUAAUAUUAUAUAUAAUAUUAAUACUGUAUUAUAUAUAAUAUAAUA

>TP982

UAUUAAUAUAAUAAUAAUAAAUAAUAUUAAUAUAAUUAAUAUAUUUAUAUU

>TP983

GUGGUGAAACCGCAUCUCUACUGAAAAUACAAAAAUUAGCUGGGCAUGGUA

>TP984

GUUUUUUCUUUUUAAGACUUGGGCCAGGUGUGGUGGCUCACGCGUUUAAUC

>TP985

CCCAGGAUGGAAUUUGUAGCAUAGUAGUGUGAUCAUAACUCACUGUAGCUU

>TP986

UCCACUAGACUUUAAAAAAUUUUUGAGAUAGGGUCUUGCUCUGUUGCCCAG

>TP987

UUGUAAAUUUGUUUGAGUUCAUUGUAGAUUCUGGAUAUUAGCCCUUUGUCA

>TP988

UCUGGAUAUUAGCCCUUUGUCAGAUAAGUAGGUUGUGAAAAUUUUCUCCCA

>TP989

UUGUGAAAAUUUUCUCCCAUUUUGUAAGUUGCCUGUUCACUCUGAUGGUAG

>TP990

AUGGUAGUUUCUUUUGCUGUGCAGAAGCUCUUUAGUUUGAUUAGAUCCCAU

>TP991

UGUGCAGAAGCUCUUUAGUUUGAUUAGAUCCCAUUUGUCAAUUUUGGCUUU

>TP992

UUUGUCAAUUUUGGCUUUUGUUGCCAUGGCUUUUGGUGUUUUAGACAUGAA

>TP993

CAUGGCUUUUGGUGUUUUAGACAUGAAGUCCUUGCCCAUGCCUAUGUCCUG

>TP994

AGUCCUUGCCCAUGCCUAUGUCCUGAAUGGUAAUGCCUAGGUUUUCUUCUA

>TP995

GCCUAGGUUUUCUUCUAGGGUUUUUAUGGUUUUAGGUCUAACGUUUAAGUC

>TP996

UUUUAUGGUUUUAGGUCUAACGUUUAAGUCUUUAAUCCAUCUUGAAUUAAU

>TP997

GAGAUGAGCUCUCACUAUGUCACCCAGGUUCGUCUCAAACUCCUGAACCCU

>TP998

UCGUCUCAAACUCCUGAACCCUAGUAAUUCUCCUAUCUCAGCCUCCCAAAG

>TP999

CUCCCAAAGUGCUAGGGUUACAGACAUGAGCCACUGUGCCUGUCUAGACUU

>TP1000

UAGCUGGGCACAGUGGCUCACACCUACGAUCACAGCACUUCGGGAGGCUGA

>TP1001

CACAUUGGUACCUUUAGUUCUCUGAAGGCCCACGUUUUUAUCAUUAAGACC

>TP1002

ACUACAGGCUAAUUUUUGUGCAUUUAGUAGAGACAGGGUUUCACCCUUUUG

>TP1003

AUUCAGCCUGUGGAGGUGGAUAAAUAUGGGAAUAUCAAUGCAGUUCACCUC

>TP1004

UUUUGUAUAUGCAUUAUUUCACUACAUAUGAAAUGUAUAUACUCUUAUUUU

>TP1005

AAAGUUUGAUAUAAGCUUAAAAAGAAGCAGCUUUUAAAGAUCAUAUUCAUU

>TP1006

ACCUAGAACUGAGUACAGUGGCACAAUCAUGCCUCACUGCAGCCUCGAUUU

>TP1007

UUUUUUUUUUUUUUUUUGGCCGGGGAGGGGGUCUCAUUGGUUGCUCAGCCU

>TP1008

UCUAAUUGUUUUGUGUGCCAGGGGCAGUAAUGUCCCUGCCUCUUCUCCCAA

>TP1009

CAGUAUUAUAGGUAGUGAACUUAAUACAGUAUGUAACUUACAAGAAGGACA

>TP1010

GUGUUUGCCAAUAAAAGUAAUGACAAAAACUGCAGUUACUUUUGCACCAGC

>TP1011

AUUGACAAUUGAAAUCUUGCAGCCGAGCGCGGUGGCUCAUGCCUGUAAUCC

>TP1012

GCAAUCCUGUGGUUCGAGCUACUUGAGAGGCUGAGGCAGGAAGAUUGCUUG

>TP1013

UUUAAAGGCUUCCAGGUGGUCGAGCACGGUGGCUUUUGCCUGUAAUCCUAG

>TP1014

UAUAUUUACCAGCUGAGCAUCCCUUAUCCAAAAAUCCAAAAUCCACAGUGC

>TP1015

CAGAUUUUGGGGCAGUUUGGAUUUCAGAUUUUUGUAUUAGGGAUACUCAAC

>TP1016

CUGAACCCCCAAGUUAAACAGCUUUACCUACCUCAUCAAAGGUCAUGUUAG

>TP1017

UGGCUUCAAGCUUUUCCUUAUUGGCACCAGAAAAUUCACCCACCUUUUGUC

>TP1018

UCAUCCACAUCUACUUCAAGGAAUAACAUGUUGGAAUACUUUUCAGAGAGG

>TP1019

UGUGCUUAGUUAAAUCUUUUCCAGGAAAAAGAACUUCCCCAUACAAAUAAG

>TP1020

AGACUAUGUAAAAAUAACCUUGCAGAAGCUGAUGGGGCAAACUCAAGCUUC

>TP1021

AUAUUCUCAGGUUUUGGACCAAAUCAGUGUUUAUUUUCCCAUUUAAUGGUG

>TP1022

UAUAUAUAUGUGUGUGUGUGUGUGUAUAUAUAUAUAUAUAUACAUUUACAA

>TP1023

CUUCUUCCAUUCCUGUCUCACAUGAAACAGCCAUCUUCCAUGCUCUCUCAC

>TP1024

UUAAUCAUCUGAGUUCAUGCUGAAUAAAUCAAUCUAUUGGCAGGAUGUAUU

>TP1025

AAUGAGUCAUUACAUUAUAUGUUUUACAUGUCAUUUUGUCUUCACAGCUAU

>TP1026

UGUCUUCACAGCUAUUCUCUGCAAUAUAUCCUGCCAAUAAAUUGAUUUAUU

>TP1027

UGAAUGGAUACAGAAAAUGUGGUAUAUACAUAUAUGGUAAAAUAUUUUGCA

>TP1028

UAUAUCCAGGGUAUCCACAGUGUCUAAAAGAAUGCAUGGUACAGAGCAGGU

>TP1029

UUGUAUUGGGUAUUGAAAGUAAUGUAGAGAUGAUUUAUAGUAUAUGGGAGG

>TP1030

ACGUGAGCCCAAGAUAAGUACAGUCAUCCCUUGGUAUCCAUGGGGGAUUGG

>TP1031

AUCCAAGGAUGCUGAAGUCCCUGAUAUAAAAUGGUGAAGUAUUUUCAUAUA

>TP1032

AGGAAAUUAGUGAUGAUGAGGCAGAAGAAGAGAAAGGUGAGAAAGAAGAGG

>TP1033

GAGGAUCCCCAGACCCACUCCAACCACAUCUACCACAUGAUCAAGCUAGGU

>TP1034

GAUGAAGUGGCAGCAGAGGAACCCAAUGAUGCAGUUCCUGAUGAGAUCCCC

>TP1035

CAGCAUCUGCUUCUGGGGAGGCCUCAGGAAGCUGUUACUCAUGGCAGAAGG

>TP1036

GCCUCAGGAAGCUGUUACUCAUGGCAGAAGGUGAAGCAGGAGCUUGCACAU

>TP1037

CUUCAGGGCAGACCCUCACAACACAAGCCUGGAGGCCUAGAAGGGAAAAAU

>TP1038

GCAUCAAACUGCCUAUCACUAGCCUAGGAAAAGAUCAAAAUUCAAAAUUCU

>TP1039

AAGAUCAAAAUUCAAAAUUCUAAGUACAGUUUCUACUGAAUGCUUAUCACU

>TP1040

GCUUAUCACUUUUGCACCAUUUUAAAGUAAAAAAAUCAGUAAGUUGAACCA

>TP1041

CUUUUCCACAAAUAAUGCUUCAACUAGUACUUGCGUGCCAGUGACUCCACG

>TP1042

ACGAUGGUUCGAUUUAUGAUUUUUCAACUUCAUGGUGAUGUGAAAGUGAUA

>TP1043

AACUUCAUGGUGAUGUGAAAGUGAUACACAUUCUAUAGAAACCACACUUUC

>TP1044

AAGUGAUACACAUUCUAUAGAAACCACACUUUCAAUUUUGAAUUUUGGUCU

>TP1045

UACUUUCUCUGUCAAAGAUUGAAAGAUACAGGGUUUUAAGUUUCCAACUGU

>TP1046

AAGAAAUCAAAGGCAGGCCAUGCACAGUGGCUCAUACCUAUAAUCCCUGCA

>TP1047

ACUUUGUUUCAUGCACAAAAUUAUUAAAAAUAUUGUUUGAAAUUACCUUCA

>TP1048

UUGUUAUAAAAAUUACAUUAGGGCCAGGUGCGGUGGCUAACGUCUGUAAUC

>TP1049

UCAUUCUGUGAAUUUUUGAAAGUCAACAGUUCAGGAGAAGAGAAGGAAUUU

>TP1050

GAACCAUUGAAAUUCAAAUGUAGGUAGGUAAGUGUUCACACCAGCGCAUGC

>TP1051

AAUGAAUUUGGUCUUUUGAUUUUUCAGGAGAACUUGCGCCUGUCAGGGGCU

>TP1052

UGCAGCAGUUGUUGCAUAUGGAUUAAACAAACUGAAGAGCAGGGGAAAUAC

>TP1053

CCCUUCUCAACAUCGGCCUUGGCAAAGAAUUUUUGGCUAAGCCCCCAAAAG

>TP1054

AAGAAUUUGAAAUCCCACCUAUCUCAUUGGAUUCUGAUCCCUCAUUGGCUG

>TP1055

GAGCAGCUGCUCACCACCAUUGCCCAAACCAUCAUCGAGGUGGAGAACCAG

>TP1056

CGGGAUAGUGAAAAGCCGGAGUUGGAACAUGGAUAGCCGCUUGCAGGAGAU

>TP1057

CCCCGCUGAAAAAGCACAAUCUAACAGCAAGGUGAGUAUGUUGUUUCCUAA

>TP1058

UAAUUUUUUGUUUUUGUUUUGAAACAAGGUCUCACACUCUGUCACCCAAGC

>TP1059

ACAUGCCAUUUCUUAUUAAUAUUUAAGCUAUUUCUUACAUUAUUUUCAAGU

>TP1060

CAGUACAUCAUUAGCCACAAUGGAAAUACAAAUUUAAUUCAUAAUGAGAUU

>TP1061

AAAAAGACCAGUAGUUGGUGAGGAUAGGAACAGCUGGAUCUCUCACACAAG

>TP1062

UCACUUUCAAAACUGUCAGUUUCUUAUAAAUUUAAACAUAUAUUUUCCAUA

>TP1063

ACUGUGUAUCUGCCUCCCUUCCUUUAGCAUAAUGUCUUUAAGAUUCAUUCA

>TP1064

AAGUGGGAUUUCUGGGUUGUGUGGUAAGUGUCUGUUUAACUUUACAAGAAA

>TP1065

AAUUUGCAUUUUAUGAGAGCAAUGUAUGAGAGUUCCUGUUGUUCCACAUUC

>TP1066

UCGUGUUGGUAUUCUUUUUAAUGUUAGCCCUUCUCGUGUAUGUAUAAUAUU

>TP1067

GCCCUUCUCGUGUAUGUAUAAUAUUAUCUCACUGUGGUUUUCAUUUGCAUU

>TP1068

UUGCAUUUCCCUUGUCAUUGGUAAUAUUUUGAGCAUCUUUUCAUGUGCUUG

>TP1069

UUUCUUGCAUUUUUUUUUUUUUGCUAAAACUGCAUCAUUUGUGUUCUGUGU

>TP1070

UAUUGUUAUUAGCAAAGAUGUGUAUAUAUUUUUAUCCUUCUUAUAUUAACC

>TP1071

UUACUGGGCUAUAGUUUCUUGGAAUAGUAAGGUAAUGAUAAAACAAAUACU

>TP1072

GCACCAGGCACUGGUUUCGUGGAAGACAGUUUUUCCAUGGACAGCGGUAGU

>TP1073

GCAGAACUGGACAAAGAGAAAAGAAAACUACUUAUGCAGAACCAGUCUUCA

>TP1074

GCUAGCAUUGCACUCUCGAGACCCUAUCUUAAUAAGGACUUCUGGGAUCAC

>TP1075

UUGCAGCAUGCUCAUGCACAUUCAUAUGGAUACUUCAUAACUCAAGACUCU

>TP1076

ACAAUCUCUGCUCACUGCAACCUCCACCUCCUGGGCUCAAGCCUCAAGCAA

>TP1077

AGAAAAAGUAGGUUUGCUGAAAAACAUUACUAAAUGACUUGGAGCUCUCCA

>TP1078

CACAUAUUAAAUACAGAUGCUCCUCAGCUUAUGAUAUGGUUACAUCCUGAU

>TP1079

CGGGAGGUGGAAGUUGUGGUGAGCCAGGACCGAGCCAUUCAGCCUGGGCAA

>TP1080

UCACUUGAAUAAAAAAGAAAAAAAUAGAAGAAAAUAAAGAAAAAUAUUUGA

>TP1081

UAAAAGGUGCCAGAAAUUUAUUAGGAGCUGCCUCAGAUACAUCAGCCUGUU

>TP1082

GGCUUGAGCCCAGGAACUUGAGACCAGCCUGGGCAACAUAGGAAGAUACUG

>TP1083

UCAAGCCUGGAAACCCAUGGCCCUAAAUGGGAACAGGCAUUCCUAUUUUUG

>TP1084

CUUGUCUGACAUACUUCUGGUGGGCACUGUGUGGAAACUUGAGGUUGAAAU

>TP1085

GUGGGCUCUGUGUGGAAACUUGAGGAUGAAAUCAGUGAGCUGCAUGCACUU

>TP1086

ACUAACAUUCUACUGGCAGAAACCUAGUGACAUGUCCACAUCUGGUAGUGA

>TP1087

AAAUGUCUGCACUCUGAUGCCUAUCACAGCAUUAUUCACAAUAGCCAAGAU

>TP1088

CCCAGGACCUGCACACAAGAGUGGUACUGAGGAUUGUCAGUGCCCACUCCU

>TP1089

UGGUACUGAGGAUUGUCAGUGCCCAAUCCUAUGCCAUCGUGUGGGUGAAUG

>TP1090

GUUUUUUUCUUCACCUUCAAUAUGAAAAUUCAGCGAAAUUGAAAGAAAAAU

>TP1091

GUACAAGAGGUUUUACUACAGAAGGAAUUCAUCUUUAAAACCUUUUAGUUG

>TP1092

GUACUGGCAUGGGUGAAAUGAGAAAAGCAUUCCUACACUGCUGGUGACAGU

>TP1093

AUAAAUGAAAUGGGCAAACAUAAGUAAAGUGUUUCCCUGAGUUCUGUGAAC

>TP1094

UAAAGUGUUUCCCUGAGUUCUGUGAACCACUCUAGCAAAGUAAUGGAACUC

>TP1095

AAAUAUGAUUAUUAUUACUAUUAUUAUUUGAGUCAGAGUCUUGCUCUGUUG

>TP1096

CUUAUUUUUUACCCUUUUUUCAAGAAAUGGGGUCUCACAGUGUUGCCCAGG

>TP1097

CUAUCAGAAAUACAAAAUUUAGCCGAGUGUGGUGGCACAUGCCUGUGGCCC

>TP1098

AGCCACUCUGAACGCUGAGAUGGGAAGGUCACUUGAGCCCAGGACCCCACC

>TP1099

GUGAAAUGCCUCCCAGGAAAAAAAUACAUAUGUAUUUUUUUGCGACAGGGU

>TP1100

UAUUGCUAUUGUAAUAUUAAUAUAUAGUAAUUAACUACACAUGACACAGCU

>TP1101

UGGUUUUCCUUGGGUGCCUUUAUGCAGCAAGGAUGCGAUAUUUCGCCAAGG

>TP1102

UGUACCUAGUGUUGCAUAAUCUAGAACACAGUAUAGAGUCCAUAUACAAAG

>TP1103

CUGUAGCCUCGAAUUACUGGGCUCUAGUGAUCAUCGUACCUCAGCCUCCUG

>TP1104

AACAUGGCGAAACUCCAUCUCUACUAAAAAUACAAAAAAAAAAAAAAGAAA

>TP1105

AGAGCGAGAUAUAUAUAUUUGGAAUAGGGUCUUGCUUUGUUGCCCGGGCUC

>TP1106

GAGAGAGAGAGGGUCUCGCUUUGUCACCGAGGCUGGAGUGCAGUGGUGUAA

>TP1107

UGAAAGAGAAGACCCUAUCUCAAAAAAUAAAAAAUUUUAAAAACCUGUCAA

>TP1108

ACUGCAACCUCUUUCUUCCAGGUUCAAGCAAUUCUCGUGCCUCAGCGGGGA

>TP1109

CCCAGCUGGUGGCAGCAGCUGAAAUAGAUGAAGAGCCAGUCAGUAAAGCAA

>TP1110

UACCUUAGUGAUGUUACUGAACAUAAAAUGGAGAUAAAAGAGGUAUCCCUU

>TP1111

GCACCAAGUAGCAGAGUUGCCUUCCAGGAGGUGCAGGGCAGGUAAGCAUGA

>TP1112

CUACAGAUAAAUCUAUAGACAAAAUACAAUCCCGGACAACAAGAAGCUCCU

>TP1113

AUGGAAUAGUCAUGGCAUACGCUGUAAAUUUUUCCACUAAUCCCUUGUCUC

>TP1114

UGGGAUUCCCUCUGUUGUCCAGGCUAAAUUUCAGUGGCCCCAUCAUGGCUC

>TP1115

CAGGCUAAAUUUCAGUGGCCCCAUCAUGGCUCACUGUAGCCCUAACCUCCC

>TP1116

GCUGGGAUUACAGGCAUGAGCCACCACACUCGGCCUAUACUUCUCUUAAUU

>TP1117

AGGAUUGUCUUGGGGAUCAUCACAAAGAAGAACAUAUUAGAGCAUCUCGAG

>TP1118

CAUUCUAGAUAUUUAAGGACUGGUAAGGUCUCUGUUUAAUCUUGGGCUUAU

>TP1119

UGGUGGGUCUCGUCAGCCAAUUCAUAGCUGGGCUUUAAGAUUAUCAUCUUA

>TP1120

AGAUGAAAAGCUGGGCUUUUUCUCUAAGAUGAUAAUCUUAAAGCCCAGCUA

>TP1121

GUGUCUCAAAAAAAAAAAAAAAAAAAAGAAAAGAAAAGAAAAGAAAAAAGA

>TP1122

CUCUCUCUCUCUCUCUCUCUGAGACAGCGUCUCACUCUGUCAUCCAGGCUA

>TP1123

GACUUCCUCCUGUUUCCCCGCGUGAAUGUGUCAAAGAUGCCGUGGCAGCAG

>TP1124

AGAUAUUUGAUAUGUAUCAGCAAUAAGCUGCAUUUAAAAGAUGGCAUUUUG

>TP1125

UGAACAAACUUGGUCUGGGUACUGAAGAAGAUGACCCUACUGCUGAUGAUA

>TP1126

AAACCGUAGCCAUAACACAAGGAGAAGAGCUGUGAGGCAUACAUAGCUGAG

>TP1127

AUGGAUGUUAACUCCUGUCAAAUAGAUCACUUGCAAUUUCUUCCUUAUGUG

>TP1128

AAUUAAAAACUUACUUUUUUGAGAUAGGGUCAUGCUGUGUUGUCCAGGCUG

>TP1129

UGAGCUGAGAUCAUGCCACAUUUGAACCCGUGAGGCAGACGUUGCAGUGAG

>TP1130

GCAGACAUGUGCCACCAGCCCGACUAAUUUUUGUAUUUUUAGUCGAUACGG

>TP1131

AGAAAUUCACCUAGGACUGGCGCAGAAGGUAUACACCAUGAGCCUGGAAAU

>TP1132

AAAUGCAAUGAGAAAUAAAGACAGAAAGGUAUCUAGAAAAUCUCCCAAAUA

>TP1133

CCAGCAUUAGGAACGUGCUGGGAUUACAGGCGUGAGCCACCGUGCCUGGCC

>TP1134

AGUUUGAAACCAGCUUGAGCAAUAUAGUGAGACCCCUGUAAGAAUUAGCCA

>TP1135

AGCCAGGCCAGGCGGAGUGGCUCACACCGUAACCCCAGUGCUCUGGGAGGC

>TP1136

CCUGGCACAGUGGCGCAUGCCCAUAAUCCCAGUAUUUGGGGAGGCAAAGAG

>TP1137

UAGUCCCAACUACUCGGGAGGCUGAAGCAGGAGGACUGCUUGAGCUGAGUC

>TP1138

UGGUCUUAGCGUUAUUCCCAGGUCUAGGUGUGAGUCCUUCCUCAGAUAUGU

>TP1139

GGUUUGCACUGGUGCACUUACAGGCAGAAGAGCUUCCUCAUUUGCUGAGGG

>TP1140

GGUGUCCAUGCGUUGACUGCCAUGUAUGACUCCAGGUAUUGAGUUCAUCUU

>TP1141

GUGUGUGACUCCAGUCGGCGUCUCAACAGUCUCCGGUGUCCAUGCGUUGAC

>TP1142

UUGAGGGGAGGCAGUUACACAAAAAACCCAGAUACCGAGUGUUAGUUCCAU

>TP1143

CUGGAAGAGGCUCGGGUCUGGCUCCAGGCGUGGUUCCCUUCCCGUGUCUGA

>TP1144

CACCGUGGCUAAACUUAUGUCCCAAAAAAGCAACCAGCAUCCCACCCUUCC

>TP1145

GGUUGCUUUCUUGGGACAUGAGUUUAGCCAUGGUGGCAGGCCAGGUGCCAA

>TP1146

AUGUCAGUUAGAAUUUGGGUCUUCUAACUUUCAGGCCCACACUGCCUCCCA

>TP1147

UAACUGAGUGCAUUCCAGCUGCUAUAAUGAAAUGCCUUAAACUGGGUAGCU

>TP1148

UAGUAGCCAGGAGCAGUGCUGGUGCACACCCGUAGUCCUAGCUACUCAGGA

>TP1149

GGUUUACCUCGUUUUGCAGGGAUGAAGGACAUCAGUGUAUUCAUCUGUUUU

>TP1150

UGCCAGUCUAGUGUUGUUUUCUGUUAUUUGCCGUUGUUAACACUAAUUACA

>TP1151

CAGUACAGUUUGAUUUAUUAAAUGUAGGUCCUCAAGCUUCUCAGUGUCAUC

>TP1152

UCUGCAUCCAUAAGUGGAGUUUAGCAUGGCAUCUGCUACAUCAAAGGCACC

>TP1153

CUGUGGGCCUUGGGCCAGCUACUUUAUCUGAGACUCAAUUUCUGCAUCCAU

>TP1154

GGAUGCAGAAACUGAGUCUCAGAUAAAGUAACGAGCCCAAGGCCUAAGGUA

>TP1155

GUGCCUUUGAUGUAGCAGAUGCCAUACUAAACUCCAUUUUAUGGAUGCAGA

>TP1156

ACAACCCUAUUCUAGAGUCCAUAUUAACAAUAGGGUUUACGACCUCGAUGU

>TP1157

CUACUGAAUUGGUGAAUUUCCUGACAGACAAUGCUAUCUAACCAUAGUUCA

>TP1158

UCAUUAUUAAAAAUCAUCAUACAGCAGAGACUGUGGCACAUUUCUUCUCAA

>TP1159

GCUGUCAACAAACCCCAGGAUUUUUAUAAACCCUAUGUGAAAAACACGCUA

>TP1160

GCCAAGCAAAAGGGGGUUUCCCCUUAUAAAACCAUCAGAUCUCAUGAGACU

>TP1161

CAAGCUUCCUCCUGUUCCACCCUCCAUAAGUGCUAAGAUUACAGUCGUGAG

>TP1162

UUUUUCUCCGUAGAGAUAGAGCCUCACUAUGUUGUCCAGGAUGCUGUCAAA

>TP1163

UUUUUUUUUUUUGAGAGGCAGUCUCAGUCUGUUUCUUAAGCUGGAGUGCAG

>TP1164

AGGGUUUCAGCAUGUUGGCCGGGCUAGUCUCCAACUCUUGAUCUCAAGUGA

>TP1165

ACUUAAUUAUAAUAUGAAUUUUAAAAAAUCACUUAGUGUAUGCUUUUUCUU

>TP1166

AUCUUCUCCAAGUAGGACUGAGCCAAGGAGGCAGAGGUUGCAGUGAGCCAA

>TP1167

UGGGCUCCAAGGAUCCUCCCACUUCAGCCUCCCAAGGUGCUAGGGUGGCAG

>TP1168

ACUGUAGCUUCUACCUCCAGGGCUCAAACAGUCCUCCUGCCUCAAUUUCUG

>TP1169

AGUCUAAAACAAGAAAACCUAAGCUAGCCAGAGUGGCUCACGCCUGCAAUC

>TP1170

AAAAAAAAAAAAAAAAAAGGAUCUCACUCUGUUUUCCAGGCUGGAGUGCAC

>TP1171

AAGAGAGGAUAAAUGAUGAAGACACAGAAUGGGCCGGGCCCAGUGGCUCCU

>TP1172

UUCUCAGUCAAUGCUAGUCUCCCUAAGUCCCUUCCCCAGCUCACACCAUCU

>TP1173

GUGUGAACAUGGAUCACUAUUACUUAUAUUCUCUGAGCCUUAGUUUCUCAG

>TP1174

AUUUAAAAAAAUAGACAGGCAUGGUAGUGUGCACCUGUGGUCACCUCUACA

>TP1175

CAACCAGCCCAGCACGGUGGCUCAUACUGGUAAUCCUAGCACAUUGGGAGG

>TP1176

UGUACAAUUCACCCUUUUAAAUUGUACAGUUCAGACUGGGCGCGGUGGCUU

>TP1177

CCGUCACCUCAAAUUUCUGGGCUCAAGCAAUCCUCCUGUGUCAUCCUCUGG

>TP1178

CAGAGUCUGUUGCCCAGGCUGGAGUACAGUGGCAUGAUCAUGGCCUGCCGU

>TP1179

AGGUGUAGGCUGCCAUGCCUGGCCAAUUUUUUGUAUUUUAGUAGAGAUGAA

>TP1180

AAUAUCCCCAUGUCACUAGUGGCUAACAUAUGGUAAAGCACAUUAUGUGUU

>TP1181

AGGAACAGAAAGCCCAACACUGCAUAUUCUCACUCAUAAGUGGGAGUUGAA

>TP1182

UGGAUCACAGUGGGCAACUUUAUAUAUAUAUAAAUACACACACAUACACAC

>TP1183

UCCUGAUAUUAGCCUGGCAUGCAUUAGCUAUUUUUCCUGAUGCUCUCCCCU

>TP1184

UUAGUAAAGAUGGGGUAUUUUUAGUAGAGGUGGGGUUCUGCCAUGUUGGCC

>TP1185

UUAAAGUCAUCUGAUUAGCAACAUUAGUUCCAUUUUCUACCUUAAUCCCCC

>TP1186

GGGUUACAUGGCAAAAGGAAAUUAAAGUUGCAGAUGGAAUUAAGGUUGUUA

>TP1187

UAAAUAAAAAAUGAAAAAACUAGCCAGGUGUGGUGACCUGUAGUCCCAGCC

>TP1188

GGCAACACAGCAAAACUCAAUCUCUACAGAAAAUUUAAAAAUUAAAUAAAA

>TP1189

AGUCAAGUAUGAUUUAGAGCCCGGCACAGUGGCUCAUGCCUGUAAUCCCAU

>TP1190

GUCAAGUAUCAAUAUAACGUAGUCAAGUAUGAUUUAGAGCCCGGCACAGUG

>TP1191

UGUAAUAUCAGCACUUUGAGAGGCCAAGGUGGACAGAUCGCUUGUGCCCAG

>TP1192

ACUGUCUUUAAUACCGGAGUGGUAAAGUGAUUACAAGAGGAAAAGCAGUCA

>TP1193

CCCUAGCACUUUGGGAGGCUGAGAUAGGAGAAUUACUAGGGUUCAGGAGUU

>TP1194

UAAGCAUAGCACAUCAGAGCAUAACACAGUGUGAGGGAAAUAAAGUGUACA

>TP1195

ACUAUAGUUCUGAGGCCAAGGAACCAUAUUUACCUUGCUCACUGCAGUAUC

>TP1196

CUGCUAUUAGGGAUUAGCAAAAUGCAGGUCUGUUAGGAUGCUGGAGGAAAG

>TP1197

GAACCUAGGUAUCACUGCUGCUAUUAGGGAUUAGCAAAAUGCAGGUCUGUU

>TP1198

UAGUAAAAAUGCUCCUGUUGAACCUAGGUAUCACUGCUGCUAUUAGGGAUU

>TP1199

CUCACUUAGGUAGAGAAUAGUAAAAAUGCUCCUGUUGAACCUAGGUAUCAC

>TP1200

UCCCUUUAUUAUACAUAAAAUGUUAAAUUUCUCACUUAGGUAGAGAAUAGU

>TP1201

GCAAACCUGGAUAUCGGGUCCCUUUAUUAUACAUAAAAUGUUAAAUUUCUC

>TP1202

ACCAGUACAACAGGCGUACAUCAGAAGUGUCCCAGGCAAACCUGGAUAUCG

>TP1203

AGACUCCAUCUUGAAAUUAUUAAUAAUUACACCAGUACAACAGGCGUACAU

>TP1204

AAUCUACGGAUGGGUGCACUGGCUCACACAUGUAAUCCCAACGCUUUGGGA

>TP1205

UUGCCCAGGCUCCCAGGCUGGUGUCAAACUCCUGGGCUCAAGUGAUCCUCC

>TP1206

UCAUUAGUCAGUCAGUAGCUGUCUCAGCUGUCAGAUUGACUGUCAUGGUAU

>TP1207

GCAAAGGCUAGGGGCUAGGGAGGGCAAGACAUGCUUGAGGAGCCACAGCAA

>TP1208

CCGUUAUCUAGGAAUGAGCUGAGGCAAAUAUUUAACCCCUGGGGCCUCUUA

>TP1209

UGCGUGAAGAGCCGCAAGAGGCGGAAGGGUAAAGCCGGGGCAGCAGCCGGC

>TP1210

UCCCUUUGAAAACCAGCACAAGACAACGAUGACCUCUCUCACCACUCCUAU

>TP1211

GAGACUGGGUAAUUUAUAAAGAAAAAGAGAUUUAAUGGAUUCCCAGUUCCA

>TP1212

CCUUUCCUCCUGCUCUAGCCACGUAAGACAUGUCUGCUUCCUCUUCACCUC

>TP1213

UGCCCAAAUCUCAUGUAGAAUGGUAAUCCUCAGUGUUUGAGGUGGGGCCUG

>TP1214

UUUUUAAUUCCUUAAAAGCAGCCAUACACUCAUCUGUUUUGACCUGCUCAC

>TP1215

UUCAGUUGCCAAUCACUAGAAGAUAACUGGCCAUCUGCUCUACCUAUACUA

>TP1216

UGCCUCUAGGUUUGUUCUUUUUUGUAGUCUUUCUUUGACUAUGCAGGUUUC

>TP1217

UGCCUUAUAAUACAUUGGGUAAUGUAAUGCCUCUAGGUUUGUUCUUUUUUG

>TP1218

GUUUUGGUAACUUUUAAUGUGCCUUAUAAUACAUUGGGUAAUGUAAUGCCU

>TP1219

AUACUGAUACCAUGUUGUUUUGGUAACUUUUAAUGUGCCUUAUAAUACAUU

>TP1220

CUAUUCUGUACAUGCCUGUUUUCAUACUGAUACCAUGUUGUUUUGGUAACU

>TP1221

CAGUUGGCUAUAAGUAUUUGUCUUUAUUUCUGGAAUCUCUAUUCUGUACAU

>TP1222

UUGAAUAGAGUGUCAUUUCCCCUUUAUGUUUUUGUUUGCUUUGUCAAAGUU

>TP1223

AUUAUUCCAGCCCCAUUUGUUGAAUAGAGUGUCAUUUCCCCUUUAUGUUUU

>TP1224

UCAUUCAUCUACAUGUGGAUUGCCAAUUAUUCCAGCCCCAUUUGUUGAAUA

>TP1225

UUGAUCCAUCUUGAGUUGAUUUUUUAUAAGGUGGGAGACAAAGGUGAAGUU

>TP1226

AGAAUUUUUUAUUGUUUCUGAUCUUAGUUUUCACUAUUUGAUCCAUCUUGA

>TP1227

AGUUUUUCCAGUGUUACCUUCUAGAAUUUUUUAUUGUUUCUGAUCUUAGUU

>TP1228

ACCCAAUGUCUAGAAGAGUUUUUCCAGUGUUACCUUCUAGAAUUUUUUAUU

>TP1229

AUUUUUGGUCAUGAGGUUUUUGCCUAACCCAAUGUCUAGAAGAGUUUUUCC

>TP1230

CCAUCUCUGCUAAAAAAAAAUACAAAGAUCUAGCCAGGUGUGAUGGUGCAC

>TP1231

UAGUGACGUGAUCCCAGCUCAUUACAGCCUCCACCUCCCAGUUUCAAGCAA

>TP1232

CAGUCUCAUCUUUUUUUCUAUAUGCAUGUCCAAGUAAGUUGUAGACAUUUU

>TP1233

UAAUCCCCGCACUUUGUGAGGCUGAAGUGGGAGGACUGCUUGAGCCUGGGA

>TP1234

GAAUAGGCUAGGUGUGGUGGCUGACAUCUGUAAUCCCCGCACUUUGUGAGG

>TP1235

AUUAUAUGAAAAUCAAGAAUAGGCUAGGUGUGGUGGCUGACAUCUGUAAUC

>TP1236

UUAAACCUGUUUAUUUGUAAGGAUUAAGUACCCUAAUAGGCUUAAACUAUG

>TP1237

UUUUCUAUAAUUUAAGAAGUCUGGAAUACAGAGUGUAACACUGUGUACUGC

>TP1238

ACAGAGGUUUCAGUGAGCUGAGAUCACCCCAUUGCACUCCAGCCUGGACAA

>TP1239

CUGCUCCUCCUCUGAGGACUCCUGCAAGAUGCCCUCCUCUUCCUCCUUCUC

>TP1240

AGGGAUAUCUGAAAGAGCAACAGUUAGGUUUCAUAUAGAUGGUUACUAACC

>TP1241

GUGAUGCUGAUGGGCAGCUUGGAUUAAGCACCACUGAAUUAAAAUAAUAAA

>TP1242

AAGCAUGCUUACCCCUAACCUUUUUAUUCUAACUCGGUGCUUAAUCUAGGC

>TP1243

UGUUGCUUUUGCAGAUAUCGCUAUAAAGCAUGCUUACCCCUAACCUUUUUA

>TP1244

AGUCUUCUCAACAAAUGAUGCUGAAACGUUUGACUGUCAACCUAUGUUUUG

>TP1245

UGCCUAUGGUGGAAGGAGUAGGUUAAAGUCCCAAGCAAUAGGUUGAUAGCC

>TP1246

GAAAAAAUAUGUAGUAUCUAGGCUUAUGAUUUAUUUCAGGUUAAUUUUUGC

>TP1247

CCAUUUGUUAUUAUAAUUUAGUUCAAGACACAAAGAUGAGGGUUUGGAGAA

>TP1248

UGAGCCAAUUAAACUUCUUUUUCUUAUAAAUUAUCCAGUUUCAGGUAUUUC

>TP1249

CCUCCCUAGAAGCCAAGCAGAUGCCAGCAUCAUGCUUCCUGUAAAGCCUAC

>TP1250

GGUGCCUGCUCCCCUUUAGCCUUCCACCAUGAUAGGAAGCUUCCUGGGGCC

>TP1251

UGUACCCACCCAAAUCUCAGGUUGAAUUGUAAUCCCCAGUGCUGGAGGUGG

>TP1252

UAUAUCCAAUUGAUAUAGUUUGGAUAUCUGUACCCACCCAAAUCUCAGGUU

>TP1253

GUGGCAGGCGCCUGUAAUCGCAGCUACUCAGGAUGCUGAGGCAGGCAGGAG

>TP1254

UGCUCUGGCUAGCAGUCUUUUGGAUAGGUUUUUAGCUACCGUAAAGGUAAG

>TP1255

GUUUUUCAAAUGGGCUAUCAUUUCCAUUAUCAUAGUUCAUUUAUAUUUCCU

>TP1256

UGCUGUGGCAUGAUCAGGGCUUACCAUAGCGUCAACCUCCCUGGCUCAAGC

>TP1257

GAGUUAUAUCCUUUCUUAGGUGACUAGGCCUGCUGCACAAUAAUAGGUUAA

>TP1258

AGAUCUUUGCUGAUCUUCUGACUUUAGUGAACCUAUUAAUGUGCUGCAGGC

>TP1259

CUUCUCUUGUCAACAAAAGGCUGUUAUUAAAAUCUUUCAGCCGGCAGCAGC

>TP1260

CCUUGCUACUCAAAGUGUGGUCCUCAGACCGCCAGCAUCAGCAUCACCUGG

>TP1261

CAAGCUGGAAUGCAGUGGCACCAUGACGGGUCACUGAAACCUUGACCUCUU

>TP1262

AGACAGGGUUUUUUCGUGUUGGUCAAGCUGGUCCCAAACUCCUGGGCUCAA

>TP1263

AGCUGAUCUUGAACUCCUGGGCUCAAGCAAUCCACUUGCUUCGGCUCCUAA

>TP1264

CUAGUUCCAAUUAGCUUCUCUAAGUAGCUGGAACUGCAGGCGUGUGCCACC

>TP1265

CCUUGGGAAUUUGUGGAAAGAAAAGAAGACAGACAAAACAAAGAAGGGUAU

>TP1266

ACGUUCAAACAGGCCAGGCUCCAGCACUUCAGUACGUCACCAGGGAUCUGG

>TP1267

UAGCUGGGUGUGGUCCUGUAGUCCCAGCUACUUGGGAGGCUGGGGCAGGAG

>TP1268

AUUGAUCCUCCCACCUUGGCCUUCCAGGUGCUGGGAUUUCUUUGGGAGUAC

>TP1269

GUGUUGGCAUGCCUGUAGUCCCCAUAACUAGGGAGGGUGAGGUGAAAGGAU

>TP1270

UGCAAUGGCUCACGCUCAUAUUCUUAGCACUUUGUGAGGCCAAGGAGGGAG

>TP1271

UGAAAAGAAAAGGAAUUUAUUUCUUAUGCUUAUGGAGACUGAAAAGUCCAA

>TP1272

AUUUUGCAGGUUUUGAUGGGCUAAAAGUCCUUGUAAACUGUACAAUAGCUU

>TP1273

CCAUGACAAUUUAGGAUUUCAAGACACUCGAGUCUUCCAUCCAGGGCCUCU

>TP1274

AUCAAAAAGUCAUAAUGUAAAUGUCAAAACAACAUUGAAAUAGUAAUGAAU

>TP1275

AGUGAUGCAGUCAUGGGUCAUGGCAACCUCCACCUCCUGGACCCAAACCCU

>TP1276

CACCCAGGCUGGAGUACAGUGAUGCAGUCAUGGGUCAUGGCAACCUCCACC

>TP1277

UUUUGAGACAAGGUCUUGCUCUGUCACCCAGGCUGGAGUACAGUGAUGCAG

>TP1278

CGUGUGUAGUAUAAGCUACGUACAUAGGCUAUAUGGUAUAGCUUGUUGUUG

>TP1279

UCCAGGGGGCUGAGGCAGGUGGAUCACCUGAGGUCAGGAGUUUGAGACCAG

>TP1280

UGGCAGGCUUGCAAUGACAAGAAGUAUUGGAGAUUUGGACCUUAAGACCAG

>TP1281

GGGAGAUAGGGUGAGACUCAAUCUCAAAAAAAAAAAAAAAGUGAAAAUUAU

>TP1282

AGUCCGGGAAUUAAGAGAUAUAAGAAAGAGGGGGCUGGGCACGGUGGCUCA

>TP1283

GAUAAUUUUUUUUCUUCAACUUUUAAGUUCUGGGGUACGUGUGCAGGAUGU

>TP1284

AUAGUAAUUGUGAUUAUGUGUGUAUAUAUAUAUUAAUAUAUACACACAUAU

>TP1285

AGAAACCUGAGAUUGCAGUCAGCCAAGAUUGUGCCACUGCACCCCAGCCUG

>TP1286

GAGCCCAUGGGUUCAAGAUCAACCUAGCCAACAGUGAGACCCCCAUCUCUA

>TP1287

UCACUAGGUGAAGUGGCUCAUGCCUAUAUAUUCCUAGCACUUUGGGAGGCA

>TP1288

GUUGGCCAUGCUAAGCAACUGGUAUAGAAAUAAGCCAUGGCGGGGCUAGAU

>TP1289

CAUCAGUGGCAUAAAUAUGUCAGUCACAGUUGGUAGCAGCUGUCCCUUUCU

>TP1290

UAUUUGAUUUGCUAGGUAUGGCAGGAUUUUAUGUUUGCCUGUGCCCUUUAU

>TP1291

UGAGCUAUCAACAGCUAUAGACUCUACACUUCCAGCAUGACCUCUGCAGCA

>TP1292

CCACUACAAAAUUUAGUUCCUGAGCAAUCAACAGCUAUAGACUCUGCACUU

>TP1293

UGGCAUGGUGGCGGGCACCUGAGGCAGGAGAAUGGCGUGAACCUGGGAGGU

>TP1294

AACCUUACCCUUCUGUGUUAUUUCCAUGUUUAUAUGAGAAAGGGGAUAUAG

>TP1295

UCUUCUGCUGUUCAAAUGGAAUCCCAAACCUUACCCUUCUGUGUUAUUUCC

>TP1296

CUCCCAGGGUGUUAGGAUUACAGGCAUGAGCCACGUACCUAGUGGUCUUCU

>TP1297

UGAGUAGCUAGGACCCCAGGCAUGCACCACCAUGCCUAGCUAACAUUUUCG

>TP1298

CGUGGCUAAUUUUUGUGAUGUUAGUAGAGAUGGGGUUUUACCAUGUUAGCA

>TP1299

GCAGUGGCUCUCCACCAGCAUGAUCAUUUAACACACAAAAACCUGAAACAC

>TP1300

AAUAGGCAUUAAACAUUGUUGUCCAAGCUAGAGUGCAGUGGCUCUCCACCA

>TP1301

CUGUACAUAGUGAUGCGUACCUGCAACUCCAGCUGCUUAAGAGGCUGAGGC

>TP1302

UCAGUUUUAACAUUGCUGGUGGCUUACAAAUACUGUCAGCUAGAAGCAAUC

>TP1303

CUUUGGAAAUUCUAACAGUGCCUCAAGGCGUAUUUCAAAUUCCACAUCCUC

>TP1304

GGCUCAAACUAAACACAUUCUCUUAAGAUUUGGUUACACGUUUUUCUAGGG

>TP1305

CAUCAGCAUCAUCUGGGAGCUUAUUAAAAAUUCAUGUUCUCAGGCCUCAGC

>TP1306

UGUAAUACACUUCCCAAAGAGUUACAGUAUACUGAUCAAGGAAAGUAUCCA

>TP1307

CUUAAGCAAGGUGGAAACACCCUAAAGCAAGGGACUUGGAGAUUAUUGUUU

>TP1308

AUCAGUAUUCCUGCUUGCUUUUUCAAGGUAACAUGGGGAAGAAAAAAACAU

>TP1309

GGUGUAAUAUUUGCAGUGUUCACUAAUACAAACAGCACUGUGAUAAACAUC

>TP1310

GUGUAAAACAGAGUGGAGGAGCAGCAAUGAAGGAUGUUGCCCGAAUCAUGU

>TP1311

CGUAUUGUUACCCAUACUGGAAUGCAGUGGUGCAAUCAUGGCUCAGUGCAG

>TP1312

AUCCUUCUACUCUAGACUGCUGAGUAGCUGAGAUUACAGGCAUGGGCCACU

>TP1313

UGGGGGGAUGGGGGAGGAAAGCAUCAGGAUAAAUAGUUAAUGCAUGUAGGG

>TP1314

GUGCCAUUGCACUCCCGCCCGGGUGACAAGAGUGAGGCUCUGUCUAAAAAA

>TP1315

GAGUUUCACUCUUGUUGCCCAGGCUAGAGCGCAAUGGUGCAGUCUGGGUUC

>TP1316

UCUCAUCUACUUGAGACGCUGAGGUAGGAGGAUCACACCCCAGGAGAUCUA

>TP1317

GCCCCCUCAGUUUUCCACUUUUGGGAUUUUUUAUUGUUAUUAAACUGAUGG

>TP1318

GGACCGCAGGAGAGGGCCCGGGCGGAGCGGACGAUGAGGGCCCAGUGAGGC

>TP1319

AUUUUUUACAUUAGCCAGGCAUGGUAGCAUAUAUUUGUAGCCCAGAGGCUG

>TP1320

UACAUAUGUUUAAAACUUUGAGGGAAGCCAAGGCAGGAGGAUUGCUUGAGC

>TP1321

UAUUUACCCUAUUAGUGUAUAAAAUAUUUAAAAUAAUAUAUUUACAUAUGU

>TP1322

AAAACUGAUAAUUAUAUUUUAAGCAACCUUUCCUUUGGUGACGUUAAUAAG

>TP1323

CAGAAAUUAAAAUAAAAAAUUAGCCAGACAUGGUGGUUCUACACACCUGUA

>TP1324

UACAUUUCCCUGAAAUACACCUGUCACACAAGAGCGAUAUGAUACCUGCGU

>TP1325

AGGUCGGGAGUUCAAGACCAGUCCAACCAACACGGAGGAACCCCAUCUCUA

>TP1326

UUGGCAGCACCGUUCACAAUAACCAAGAUUUGGAAGCAACCUAAGUAUCCA

>TP1327

GAAUUUGAGGCUACAGUGAGCUAUAAUCAUGCCCCUGCACUUUGCCUGGGC

>TP1328

AUGAAAGGCCACCAGCCACCAAGUUAGGAUGAGAGGGGCUGGAAUUCUAAA

>TP1329

CCCUGUCCCAAGCCACAGCUAUGGCAUUAAUGUCACCAGUGUUCUCACCCU

>TP1330

CCUGAAGCCAAAAAGAAGCCUCUGAAGGCUCAGCUGAGAAAGGUGUGCUGC

>TP1331

AGGGUCUCACUAUGUUGCCCCCGCUAGCCUCAAACUCUGGAACUCAAGAGA

>TP1332

CCUUCCGGAAACAAGUGAUCCUCCUACCUCAGUCUCACAAGUAGCUGCGAC

>TP1333

GAAUAUGUGUAGUCAGUGGAAACAGACUCAUUUUCUAUUGCUACUCGUCUU

>TP1334

CACAGCCAUGUAGGAGGAUCAUCUGAGUCUGGCAGGUCGAGACUGCAGUGA

>TP1335

AAGCUGAGCGUGAUGGCUUAUGCCUAUAAUCCCAGUUACUCGAGAGGCUGA

>TP1336

CAAAAAUGUUUUGGUUCUCAAAGAUACCAUUUGUUAGUGUGAUUUAUGACA

>TP1337

CUGAGACCAGCCUGGGCAACAAGGCAAGAUUCCAUCUCUAUAAAAAUUAAA

>TP1338

GUCCUCUAAAAUAGUGCUGUAAAAUAAAACUAUACUGCAAGCCGCAAGUGU

>TP1339

UGGUAUAUACGCAGAGUGGAACAUUAUCCAGCCAUGAAAACAAAUGAAAUA

>TP1340

AAAUAGAAAUUUACCCAAAGAAGACAUGCAAAUAGUCAACAAGCAUAUAAA

>TP1341

UAAUGGGUACUAGGUUAAACAUGAGAGCAAUGAAAUAAUCUGUACAACAAA

>TP1342

CAUGUUUAGCUCUUAAAAUUUGGCCAGAGUCAGGGUCUCAGAAAAGUCUUC

>TP1343

UCUCAAAAAAACAAAAAAGAAAAAGAAAAGAAAUAAGAUAUGAUGAUUGAU

>TP1344

UUACACAAACCCAUUGAAAGUUCAGACGGUUAUAAUCCUUGGGAAAGUUAU

>TP1345

AGAUCAUCACACGGAAAGAUGACCAAGGAAACCCACAGAAAGUCUUUGCAU

>TP1346

CUUUUCUACCACAUGGUCAGGCUGCAAAUUCCCUAAACUUUUAUGCUCUGC

>TP1347

UGAAGGAGAAGCAGACACGUCUCACAUGGACAAAGAAGGCAGAACAGAGAG

>TP1348

GGAUAAUUUAUAAGAAAAGAGGUUUAAUUGGCUCAUUGUUUCACAGGCUGU

>TP1349

UGGCAAAGAAUUCUGCAACUUUGUUAAGUGCCAGCGAUUAUGAAGUGGCUC

>TP1350

UUGCCCCUCCUCCGCAUGCCAACGCAGUUCAUGUACAAGGCCCCUCUGCAA

>TP1351

UCUCAGGUGAUAGGGUGGGGGCAGUAGCUCAUGCUUGUAAUCUCAGCACUU

>TP1352

CAGGCACCAUUCAGGAAGAUUACCUAAGAGAGCUGCUGACAACCGUGGGGG

>TP1353

UUGCUUGCUUUGAUGAAGAAGCAACAGGCACCAUUCAGGAAGAUUACCUGA

>TP1354

GUGGUUCUCCUACCUCGGCCUCCCAAGUGGCUGGGAUUGCAGGCACAUGCC

>TP1355

ACUGUGACACCUAGUUUGGACUGCAAUGGCGCGAUCUUGGCUCACUGAAAC

>TP1356

AUGGGUGCUAUUGUGAGGCGGUUGUAGAAGGUAUGAGGAGGCUGUGCGGUG

>TP1357

CAUACAUACAUACAUACAGGAUCUCACUCUGUCACCCAGGCUGGAGUGCAC

>TP1358

AAUUUUAUUUUAAAAAUCUUUUAAAAAAGAUUUUUAUAUAUAUAUAAAGAU

>TP1359

UAUAUAUAUAUGUUUUAUAUAUAUAAUCUUUUUAUAUAUAUAAAAAAUCUU

>TP1360

CAUGGAGAAUGGCUGUCUCUCAGCUAGGGAGGAGGGUCUUGGUGUCCUAGU

>TP1361

CAUGGUGUGUGGAUUGCCUGAGGCCAGAGGUUCAAGACUGGCCUGGCCAAC

>TP1362

ACCCAGCCAGUCCCAGCAUUUUGGGAAGCCAUGGUGUGUGGAUUGCCUGAG

>TP1363

AUUACAGGCGUGAGUCACUGCACCCAGCCAGUCCCAGCAUUUUGGGAAGCC

>TP1364

CCGGCUAAUUUUUGUAUGGUGUUUCACCACCUUGGCCAGGAUGGUCUUAAC

>TP1365

GAUAGAUAUAUCUAUCCAAUAUAUCAAUCAUUAAAAAUAUAUAUUCCUAUU

>TP1366

ACAAUGCAGAUAAUCUAUAUAGAUAAAUAAUACAGGCCAGUUUUAUUGAUA

>TP1367

UUUUCUCAUCCCCAAAAUAGCUUCUAGCUCUUCUGACUUUUUUGUCUAUCU

>TP1368

UGGCCUGCAUUAUCUAUCUACAGAUAGACAGAUGAUGAUAGAUAGAUAGAU

>TP1369

GAAUUUGUUCCUAAAGAGCUAGUCAAUAAAACUGGCCUGCAUUAUCUAUCU

>TP1370

GAAUUUGUUAGGGAUGCAGAAUUUCAGGCCCCGCCAGACCUACUGAAUCAG

>TP1371

GUCAAGAAUCACCCGGGAAUUUGUUAGGGAUGCAGAAUUUCAGGCCCCGCC

>TP1372

GCUCAGUAGGUCUGGGUGGGGCCUGAGGUUUUGCGUUUCUAACAAGAUCCU

>TP1373

GAGCCCACAGAACCAGGAGCCAAUUAAGUCUCUUUUCUUAUAAAUUAUCCA

>TP1374

AGAACUAAAAGUAGAUCUACCACUUAAUCCAGCAAAUCCCACUCUUGGGUA

>TP1375

GUCAGUUGGUAAAUGAUUAUUGACAAUGUGUGGGAAGGUGGAGUUAUUUGA

>TP1376

AGUUAUUUGAGUAGAUUUGGCCUCUAUUCUCAUGGAGCUUCCUUUCUAGAG

>TP1377

UCACUGAGCAAACUUCUGAUUCAGAAGUCGAAGAGUCACUGUGAUUUUUCU

>TP1378

CUUUCAGCGCCUCCUGGAGACUCUCAACGUCCAUGGCUUCCUGGAGUCCCU

>TP1379

GAGACUCUCAACGUCCAUGGCUUCCAGGAGUCCCUCACAGCCUCCGCCUCC

>TP1380

GCAGGUGGGGGCACAUCCCCCAGCCACUCCCAUUUCCUGACAUUGUCACUU

>TP1381

UAUCUCAAAAAAAAAAAAAGAGACAAGGUCUGAUUAGGUUACCCCGACGGG

>TP1382

CAUCUCCUGAUUCAUGCGUUGUUUCAUAGGUUUCAAUGUCUCUGUAAAUGU

>TP1383

AAGCAAUGCAGUUAGUUGCCAGGCAACGUGCUAAAGGAGAGGUUCUGAACU

>TP1384

AAUUUAAAGUACAGUUGACUCUUGAACAACAUGGGUUUUUGAACUACAUGG

>TP1385

UUUUUUUCUUCAAUAAAUAUAUUGAAAAAAAUUUUGGAGAUUUGCAGCAAU

>TP1386

ACCCCAACUCCCAAUGUGAUGCAUUAGAAGGUGGGGAUUUGGGGAAGUAAU

>TP1387

CUCCGUAUGCCCACAAAUUAGCACUAGGGGAGGAGAUGCUCUGUUGCCAUU

>TP1388

UUAAGAAUCCACAUUGUAUUUAUUUAGGUUGAUGCAAAAGUAAUUGCAGUU

>TP1389

ACUGGGGUUUUCAUGGUGAAGUCCAAGGCAAGGGCAUAGAAAGACCACUUG

>TP1390

UUAAAGGAAGCAUCUGCUUCUCUCAAUACAAGGUUGAUGGAGAUGAUUAGU

>TP1391

UGCUGAUGAUUUGGAGCAAGGAGGUAGAAAAUAGUCAUAUUAUUUGUCCCU

>TP1392

CAAAAACGGAAAUGAUAAAAACAUCAGAUCUUGUGAGAUUUAUUCACUACC

>TP1393

CAUUUCACAUGGUGGCAGAAAGAGCAGAAUGAGAGCCAAGCAAAUGGGGUU

>TP1394

GGGGUUUCCCCCUUAUAAAACCAUCAGAUCUCAUAAGACUUAUUCAUUACC

>TP1395

AUUACCACAAGAACUGUAUGGGGGAAACUGCCCCCGUAGUUCAGUUGUCUC

>TP1396

UGAGCUGAAGACUGCCAUGAGUACAACUCAUGUGGCAGAGCCUGCUGAGAA

>TP1397

UGUUUUCCAUAAUGUGGCCAUCCAGAAUGAGGAAGGAACAAGAAUACUUGA

>TP1398

AAUGUUUCUAAUUUUUUCUACCACUAGGUAAGAUUAAAUCAAUCUCUUUUU

>TP1399

AUCAAUCUCUUUUUCUUUUUUCCAUACAGAUGGGGUCUUGCUGUAUUGCAC

>TP1400

AGUGGCCAAGGCAGGAGGAUCACUUAAGCCCAGGAGUUUAAGUCUAGCCUG

>TP1401

CCAGGAGUUUAAGUCUAGCCUGUGCAAUACAGCAAGACCCCAUCUGUAUGG

>TP1402

AAGACCCCAUCUGUAUGGAAAAAAAAGGAGAAGAGGUUGAUUUAAUGUUAC

>TP1403

AAAAAAAAGGAGAAGAGGUUGAUUUAAUGUUACCUAGUGGUAGAAAAAAUU

>TP1404

GGUUGAUUUAAUGUUACCUAGUGGUAGAAAAAAUUAGAAACAUUUUGUUUC

>TP1405

GUGUUUAGGAUCCUCAGCUUCUGCAAGUCUGAAAGUAUUGUUUCUGAGAGU

>TP1406

AAGGUGUGAAUUCUGGAAGAUUGGUAGCUUGCAUAACCACUUUGUUCUUAU

>TP1407

AAUUUCACACAAAGGAGUCAGGUGCAGUGGCUCACACAUGUAAUCCCAGCA

>TP1408

AAUCAUAAACUCCUGGGCUCAAGUAAUCCUCCUACUUCAGCCACCUGAGUA

>TP1409

UUUGACGUGGUUCACAUGAAAGAUGACAAUGGCAACAGCUUUGCUACUCGA

>TP1410

CAGUUCACUGCAGCCUCGACAUCCCAAGCUCAAGUGAUUCUCCCACUUGGG

>TP1411

GUCCUCUCACCUCAGCCUUCUAACUACAAGCACAUACCACCAAGGCCAGCU

>TP1412

CCAGGUUGGGGUGCAGUGUGGUGUGAUCUUGGCUUACUGCAGUCUCCGCCU

>TP1413

GAUUUAUAUUCUCUGACACCAAAUUAGGAAAAAAAGAGGUCUCUAUUUUUG

>TP1414

CCUUGUAGGACAGCAGCCCUGUCCUAGAAGGUAUGUUUAGCAGCAUUCCUG

>TP1415

ACCUAUUAAAACUGUGAAGAGUAAAACUAAAGCCAAUUUAUUAUAGUCACA

>TP1416

AGAUACUGUAGGUCUCUACAGAGAUACUAUAGUGUCCCUAUAGUACCUCUA

>TP1417

AAGUCUUAACAAGUAAUAAGAUAUAAAAUUAAGUAUCUUAUUUUAUACAAG

>TP1418

UCAGAUCUGUUUUUCAGGGAAAAGUAGUUACUGUUUUUGCCAUUACUUCUA

>TP1419

UUCAACAACAACAACAACAACAACAACAGAGCAACCAAAGCCAAUGGUUCA

>TP1420

CAGGGUAAGGUGGCGCAUGCCUGUAAUCCUGGCUAUUUGGGAGGCUGAGAC

>TP1421

UUUUUUGGGGGGUGGUAGGAGGGACAGAGUCUCUGUUGCUCAGGCUGGAGU

>TP1422

UCCCAUCUUAGUCUCCUGAUUGUCUAGGACUACAGGUGCUUGCCACUGGGC

>TP1423

GUGGUGCAUGCGUGUAAUCCGAGCUAUUCAGAUGGCUGAGGCAUGAGAAUC

>TP1424

AUUUUUGCAAGUGCUUAACAAAACAAAACAAAACAAAACAAGGGUUUAGGG

>TP1425

AGGGUUUAGGGUGGGGAGGGGAAUAAAACAAUAAUCAAAAGUCUCCCCUAA

>TP1426

UUAGUAACCUUGAAUAUCAAACACAAACUCUCAAAGUAUAGGACAGAAACA

>TP1427

ACAAACUCUCAAAGUAUAGGACAGAAACAACAAAACAAGUAAUUAUUAAGC

>TP1428

AACUACAGUUCUAAGAAUUGAAACAAAACAAAACAAAAAGGGCAAGGAGAA

>TP1429

CAAGGAGAAACGAAAAGAUAAGACAAUGAAAGAGAAGUCGAAGUCAGGCCC

>TP1430

GUUUUAGCUCCUGGAUGACAUUUCUAGACACAUCCUAGGCCAGAAGGAAAU

>TP1431

ACAUGCUCCUGAAUGACUUCUUAGUAAAUAAUGAAAUUAAGGCAGAAAACA

>TP1432

GGAAGAAAGAACUGGAAAAACACAGAGAGAAAUUGUUAAGUGGAAGUGAGA

>TP1433

UGUUUCCGCAGAUGAAAAGAAGCAAAGAAUUGAUAACUAAAAAUCAUAGUC

>TP1434

UGGGCUGGUGUGGCAGCUUAUGUCUAUAAUCCCAGCACUUUUGAGAGGCCA

>TP1435

AACAUCGAUGGAACUGGAGAACAUUAUGCUAAGUGAAGUAAGCCAGGCACA

>TP1436

UGAUUCAGUUACUUUUGGGAAAGGCAGUACAAGUAAAGCCACUCAAUAUGG

>TP1437

UGUUUGUGCCAUUGGGCUGCAGUCUAGGUGACAGAGUGAGACCCAGUCUCU

>TP1438

GAAUUAGGAAAUAUAGGACGCUUAAAUUAGAAUUUUCAAAUAAGGAAUAAU

>TP1439

UGGUCUUGAACUCCUGGACUCAAGUAAUCAUUCUGCCUAGGCUUCCCAGGC

>TP1440

UUAAAUCCUUGUUUUUCCUAAAAACAUGGACAUUCUCUUAUAAAGCCAGCA

>TP1441

CUAAAAACAUGGACAUUCUCUUAUAAAGCCAGCAGAGUUGUCUACUUCAGG

>TP1442

AUAAGAGUUCUCGGCCAGGUGUGAUAGCUCAUGCCUGUUUUCCCAGCAGUU

>TP1443

UGCUCUCCUUUUUGGAAAUUUUUGGACACAUCCAAUAACAGACCAGCUUCG

>TP1444

ACCUACUGGGUCGGGAAGUCACUGGAAACAUGAGAGCCAUCCUAAUUGACU

>TP1445

GGAGAGGAAAGAUAUCUGGAUUCUGACCAACAGAUUCAUCAUGUCCACUGC

>TP1446

UAAUUCAUUAAUUUAGUGUAAGAAGAAAGUUAAGUCUGAAUGUAAAUUCAG

>TP1447

CCGGCAGUUUGGGAGAGUCGGGAGAACUGCUUGAAGCCAAGAGUUUGAGAC

>TP1448

AUUUUAGAUGAAAGCCAUUACAAAGAGUUGCUCACACAUCAUGUUAGUCCU

>TP1449

AAGACUUCCAGGGAAACUCAUUUCAAGAUGAAAAUGGACCAGCCGCAGUGG

>TP1450

CCAGGCUUGGUGGCACACGUCCCCUAGCAAUUCAGGAGGCUGAGGCAGGAG

>TP1451

UGUCUGAGACCAGUCUGGGCAACAUAGUGACACUCUGUCUCUACAAACAAA

>TP1452

UUUGUGUGACCAUUUUAUUUCAUUUAGCAUAAUGUUUUCACAGUUCAUCUA

>TP1453

GAGCCACCACACCUGGCCAAUUAAUACAUUCUUUUAAAAAAAAUGUAUUAA

>TP1454

UUAAAAAUACCUUAUUGCUUUUAAAAAUGCUAACAAUCAUCUGAACCUUCA

>TP1455

ACGGAGUUUCACUCUUGUCUCCCACACUAGAGUGCAAUGGCAUUAUCCCUG

>TP1456

UCCCACACUAGAGUGCAAUGGCAUUAUCCCUGCUCACUGCAACCUCCACCU

>TP1457

AUUACAGGCGUGAGUCACUGCGCCCAGCCAGUAGUAGUUUUAUAGAAUGAG

>TP1458

UCCUGGUCUCGGAGGACCCAAUCCUAGCCCGACCUGUCUCGGCCCGCAACU

>TP1459

GACAAUAGCAGAAGCAUUCUUAAGAAGAGAUACUACGGACAGCACUGCACU

>TP1460

UUAACUAGUGAAUUAAUUUUUCAAGACAGGUUCUCCCUCUGUUUUCCAGGC

>TP1461

GAAAAUAAGGCUUAUUGGCGAGGCAAGGUGGUUCACAUUUAUAAUCCUAGC

>TP1462

UGGUUGGGUGCAGUAGCUCAUGCCUAUAAUCCCAACACUUUGGGAGGCCAG

>TP1463

UGAUUCUACAAAAAAAUAAAAAAUUAGCUGGUGUACUGGCACGCACUUGUA

>TP1464

CCUUUCUCUUGGGCAUGGUGGCGGCAGCGACGGCAGCGGGACAUAGGUGCU

>TP1465

UUGUAAACAGACUUAAAAAGGCCUAAGCACAGAUCCGAGUUGGGAACCCAU

>TP1466

UCCGAUUCUGUAUGUCUUUAAAUUCAAGAAUGAAGAAGAGGUCUUUGCAUG

>TP1467

GCAUCCUCACCCUGAAGUACCCCAUAGAGCACGGCAUUGUCACCAACUGGG

>TP1468

CCAGGUACAAUGGCGUACGCCUAUAAUCCUAACACUUUGGGAGGCUGAGGC

>TP1469

UUUUUUUUGAGAUGGGGUCUUACUAAGUUACCCAGGCUAUUCUUGAACUCC

>TP1470

UGUGGUGCUGCACACCUUUAGUCCCAGCUACUUGCAGGGCUGAGAUGGGAG

>TP1471

GAGGUCAAGACAGCAGUGAGCUGUGAUUGCGACAUUGCACUCUAGUCUGGG

>TP1472

AAAAUGCUGAUGCCUGAAUCUUCUGAGAUUCUGAUUUAAUUGCUCUGGGGU

>TP1473

GAUCAUUUUGCUUCUUGAAGGUGAAAGCUUUCAUCCUGUUACAUUUGUCCU

>TP1474

AACUUCGCAUAUUUUCAUUGACACCAGUGUAUAAGUAUAAAUUUAAAUGAA

>TP1475

ACUCCUUCUCAAAAAACAAAAAAACAAAAAAACAAAAAAAACUCUCCCAUA

>TP1476

CAUUGUCUUUCUCUGCAAACUGGGGAUUAUGACUUAAGCCCCCAUGCAGCA

>TP1477

CAUGUCAUUCAAGCUUAAAAAAUAUAAACUCCUCUCUAAGCCUUUGGGAGA

>TP1478

AAAAGUCCACAAAAACAAAACAACUAACAACCACCCCAACAAAGAUAGUAU

>TP1479

AUCAAGAGAAUGAACAAGCCUUCAUAGCCAUGGCACAUUUAUAUAAAAAGA

>TP1480

AUAAUACUACUUUACAAUAGUUUUCAACAUUUCCAUAUGGUGCGACCCCUU

>TP1481

AGUAAAGUGGGGAAGCGCCACACACAUUUAAACCAUCAGAUCUUGCGGGAA

>TP1482

GCAGAUUCCAGCACCAUGCUUCCUCAAUAGUCCUGCAGAACCAUGAGCCGC

>TP1483

CGAAACCCCGUUUCUGCUAAAACUCAAAAAUUAGCCGGGUAAGAUGGCAUG

>TP1484

ACCCCGCUUGUCAUAAUCAUGAUAAAGAUAACAUGACCUUUCCAACCUUCU

>TP1485

UCCAGUCCACCAUUGGUGGGCAUCUAGGUUGAUUCCGUAUCUUUGCUAUUG

>TP1486

UACCGCUCAACCUGGCAAUCCCAUUACCAAGUAUAUACCCAGAGGAAUAUA

>TP1487

CUGCUGAAAGAAUUCAUACAUGACAAAAACAAAUGGAAAAAACAUUUCAUG

>TP1488

ACAAAAAGAGAGCUAGAAUAGCCAAAGCAAUCCUAAGCAAAAGUAACAGAC

>TP1489

CAAUCCUAAGCAAAAGUAACAGACUAGAGGUACCACAUUACCUGACUUCAA

>TP1490

GUCAAUUUUUGGUUCUGUUGCAGUUACUUUUGGUGAUUUUGUCAUAAAAUC

>TP1491

AAAUAAGAUUAGAGAAAAUUGUGAAAGAGAAAGCAUUUCUUUCAAUUCAGU

>TP1492

GAAAGGGAGGAAAGGAAAGGAAAGGAAAAAAAAAGAAGGAAAGGAAAGGGA

>TP1493

GACUUGAAUUUAUGGAACUUAUGUAAAGUCUCAGUACUCUACAUAAAUAGG

>TP1494

UUUUAAUGGCCAAAGAAUAUAAUUUAAGAAUUUUUUUUUGUUCUCCUAUUU

>TP1495

AGAAUUUUUUUUUGUUCUCCUAUUUAUGUAGAGUACUGAGACUCUACAUAA

>TP1496

UUGUAUGCAGCUUAGCAUCUCCCAGAGUAAAUACAGAAUGCACACCAUAAA

>TP1497

CCUUCCUCUCAUUUAGUUAAAUUUCAACUGGCUAAUAUAGGAAAAUAGUAU

>TP1498

UUUUGUAUGGAAAUGAGUUUAUAUUACAUUUCCAUUAUACUAAGUCAGUGU

>TP1499

UGCCACAUGAGGUGCAGAGAAUACUACCAGGUGCUAGUUUUUCCAGUAUUU

>TP1500

GUAUUUCAAUUGUGUAAUGUUAAGGAGUUUUUUCAUAGCUUCAGAAAAGAG

>TP1501

AAAACUGAGCAGGAAAGGAAACAGAAUCCAAAGUCAUUUUUCAUAUAGCUG

>TP1502

UCGAGAGGGGUCUUAGUUAUUUCAAAUCCAUGACCAAAGUGUCCAAAGACA

>TP1503

CCACUAUAGAGUAUAAUGAUGGAAGACCAGCAAAGCGCCAUGAUAUUGCAC

>TP1504

GCUCUUUUCAAUCAAGCUGCCCUCAAAGUGCCAGCUGAUGAUACUCCCAAG

>TP1505

CAGUUUAUUUAUUCAUAACACUGUUAAUGCACAUUUUGGUUGUUUCCAGUU

>TP1506

GGAAAUAAGAUGAUCAUUGAGGAGGAGAAACGAUCCCUUCACGAUGCUUUG

>TP1507

UCUGCCACUCAGGCUGGAGUGCAGUAGUGUGACAUCAGCUCACUACAACCU

>TP1508

GAUGGGGAAUAUGGCUGUACCUUUGAAUUUUGCUGUGAACUUAAAAAUACU

>TP1509

UGGCUUGAACCAGGGAGUUUGCAGUAAGCCAAGAUCACACCAGUGCACUCC

>TP1510

AAAAAAUUACGUUUGAUGAUUGAUUAGUGCCUCUUUCUGCAAACUUUUCCA

>TP1511

AUCUAAAAAAAAAAAUGUUUUUUUUAAGACAGGAUCUCAUUCUGUUGCCCA

>TP1512

AGGAUCUCAUUCUGUUGCCCAGACUAGAGUACAAUAUUGCAAUCACAGCUC

>TP1513

AACCAAAAACUAAGUAAGUUAAAAUAGCGUCUUUAAUGUCUUUUUUGAGUA

>TP1514

UAAUGUCUUUUUUGAGUAAGUCUGAAGUCUGGGCUUCCUCAGGGAAUUUCU

>TP1515

AUGGUCCAUGCACUGAUGGGGAAAAAAGCAAUAGAAAUUCCCUGAGGAAGC

>TP1516

CUGAGGAAGCCCAGACUUCACACUUACUCAAAAAAGACAUUGAAAACGCUA

>TP1517

UUGAAUCAGGGAUGGUUCAAUCCAAAGUGUUGAAAUUACAGGCAUGAGUCA

>TP1518

UCCGGGCUGCAUCGGUGGCGACAGCAGAGGCUCGGGCGGCGACUCUCCGGC

>TP1519

AAAGGGCAGUUUUUAAGGUUCUUUAAAAAUUUAUCGGCCCGGCAUGGUGGC

>TP1520

CCUUCAGCUUGCGAGGCUCUGUUUUAAGUGAGUUCUGUCAGAAAUGAGGAG

>TP1521

ACCCAAUUAAAUGAAUACAUACCAAAAGUAACUGUCUAAUGAAAUAAGACC

>TP1522

GGGCUGAACUGUGCUGAGCGCUGCGACUGCAGCCACGCAGAUGGCUGCCAC

>TP1523

GGUUUCAGCAUCACAGACAAGAAGCACAGGACAGGCUGCACUGGGGCCAAA

>TP1524

AUUCUUAUAUAGCAUAUGCUAAUUUAUUUAUUUAUUUUUUGAGAUUGAGUU

>TP1525

CAGUUAUGCCAACUAAGCCUCCCAAAUAGCUGAGACUAGAGGUAUGCGCCA

>TP1526

AGUAGCUAUUCAUAGGCACAGUCAUAGCACACUGCAGCCUAGAAUUUCUGA

>TP1527

UAGGACUACAGGUGCAUACCACCAUAACCAGCUUUAAUUAAAUGUUUUUUA

>TP1528

CUUUGGGUUUUUUUUUUUUAGAGACAAGAUCUCUCCGUGUUGUCUAGGCUG

>TP1529

GAGACAAGAUCUCUCCGUGUUGUCUAGGCUGGACUCAAACUUCUGGACUCA

>TP1530

AUCCUCGCAACAUCAUUAAUAGCUGAGAGUAGAGACUUGAGCCACCACACC

>TP1531

ACUGAUAUUAGCUGGGUGUAGUGGCACAUGCUGAUAGUCCUAGUUAUUUGG

>TP1532

AGUCCUAGUUAUUUGGGAGGCUGAUAGGGAAGGAUUGUUUGAACUCAGGAG

>TP1533

GGCUGGACUUGAAGUCGUGGGCUCAAGUAGUACUCUGGCAUCAGUCUUGUA

>TP1534

UUUUUCCAAAUCGCUGUUUUUUUUUAUUUUUUUUUUUUUUUUGCUGCUCCA

>TP1535

UACACUCCCUCCAGCAAUGUAUGAGAGUUCUGGUUGCUUCACAUUUUUGCC

>TP1536

AAAUGAAAACCAUAAUAAGUAAGAUACCACUUCACAUCUGUUAGGAUGGCU

>TP1537

AGGCUUUCCUGACUUUCUCCCCUAGAGGACUGAUGUUCUCUACUGGUACAG

>TP1538

UAAUGAACAUGGGCUAUGUGCCAGAAACCACCAGGUGCCAGAAUACAACAC

>TP1539

GUCGAGACUACACCGAGAUUGCACUAAGCCGAGAUUGCACCACUGCGCUCC

>TP1540

UUGUGCUUUAUGAGAAGGAGGGAUUAUUUUUAAUGUAGAAGAAACUAGAAA

>TP1541

AUCUAUAGAGACAGAAAGUAGAUUUAGUGAUUGCCUAGGGCUGUGGGAGUU

>TP1542

UUAAAAUUUAUUUCUUUUUGAAACAAGUUCUCACGUUGUCAUGCAGUCUGG

>TP1543

AGCUGCUGCAGUCAUCCAGGUGCAAAGUGAUGGUGGCUUGGACUGGGUGCG

>TP1544

AAAAAACAAAGGUAAUAUGUAAGCCAGGCACAGUGGUGCACACCUGUAGUC

>TP1545

AGUAAGUUGGCUUUGAAGCUGAGAGAUAGAUCUAGGCUAAGGCCAAUGAUU

>TP1546

GGUCAGGAGUUUGAGAUCAGCCUCCAGCCUGGCCAAUAUGGUGAAACCCCA

>TP1547

UUGUUGUUGUUGUUGUUUUAGAGAUAGGAUCUUGCUAUGGUGUCCAGGCUG

>TP1548

AAACUUCUAAUAACAAGACUGGGCCAGGUGCAGUGGCUCACACCUGUAAUC

>TP1549

ACGAAGAUGAUCCUGCAGAAGCUGAAAAGGAGGGAAAUGAAAUGGAGGGUG

>TP1550

UAGCUUUCAGUUUUGUAAAGUUAUCAGAAAAACAUCGGGAGGGUUUGGCCA

>TP1551

UUUGAAUUGUGGGGACAUUAAUCACAGUGAUUCUUAAAACUUUGCUGUUGA

>TP1552

CAUUGUAACCUCCAACGCCUGGACUAAAGUGAUCCUCCUACCUUGGCCUCU

>TP1553

CUGUCUCAGAAAAAAAAAAAAAGAAAAGGAAAAAGAAAAAUAUAUAUUCUA

>TP1554

UAAACAUUUUUCUAAAUCCUGAAAAAUGCAUAGACAUAUUUUAGUGCCUGU

>TP1555

UUUACUUUGCAUCUCCCUGAUUGGUAUUGCUGUAGAACAUAUUUGGAGAAG

>TP1556

UGUACAAGGCUUAGCGUGGUGGCUCAUGCCUGUAAUUCCAGGACUUUGAGA

>TP1557

CUUCUGUGACUAGCCCUUUUCACUUAGUGUAAUGUUUUAAAGUUUCAUCUG

>TP1558

AACAAAGUAUAAAGCAAGCCUUGGAAGAAGCAAACUGAUUAGCAAGUUACA

>TP1559

GGAGUCUGACUGUCUCCCAGGCUGAAGUGCAGGGGUGUGAUCUCUGCUCAC

>TP1560

CCCGCCACAAAUAUUUUUUAAAAGUAGCCAGGCAUGGUGACACAUGCCUGU

>TP1561

AAACCAAAAAGAAAUGGGACUGGCUAGGUUUGGUGGCUCACACUUGCAAUC

>TP1562

AUCCUGGUCUUUAACUUUUACGCUCAAGCGAUUCUUCCACAUUGGCCUCCC

>TP1563

GUUGGGGGUGAUGGUGCACUCCUGUAGUUCCAGCUACUUUGCAGGCUAGGG

>TP1564

UUAUGGUUUUGGCCAUUUAAAAAAAAGGUGAUGAAAACUGCAAUUACUUUU

>TP1565

UUAGGCCACGUUUUGUUUUGUUUUUAUUUGUUUGUUUUGAAACUGAGUCUU

>TP1566

ACUGUGGCCCAUGCCUGUAAUCCUAACAUUUUUGGAGGCGGAGGUGGGCAG

>TP1567

UUUAUCCACCCUUCUCUUGGAGAUUAUGGUUGCUUUCACGUUGUUACUACA

>TP1568

UCACGGCAACCUCCGCCUCCGGCUCAAACGAUCCUCAGCCCCGUCCUCCGU

>TP1569

GUAUGAUCAGUGGCUCACGGCUCACAGCAGCCUCGACCUCCCAGGCUUAAG

>TP1570

UUAAGUCUUUUUGAGUCAGAGUCUCACUCUGUCACCCAGGCUGCAGUGGCU

>TP1571

CUACCAAAACAAUGAAAACUAGCCGAGCAUGGUGGCAUGUGUCUGUAGUCC

>TP1572

UUGAACCCAGGGGGUCGAAGCUGCAAUGAGCCAUGGUCAUGCAGCUGCACU

>TP1573

AAACCUGGCUAAUUUUUUGUAUUUUAGUAGAGACGCCCUGUUGUCCCGGAU

>TP1574

GGACGAAUGUUGACGCACACAAUUUAGAUGGAUCUCAAGGGAAUUACGUGA

>TP1575

CAUUGUACUAUGUAUAUAUUUUCUGAGACGGAGUUUCUUUCUUGUUGCCCA

>TP1576

UGCUCUUUCACCACACCUGAGGUGCAGUGGAACCACCACGGCUCACUGCAG

>TP1577

UGCAGCCUCAUCCCAGAAAUGCAGAAACACACUUGUUUUGUCUGUAGAAAU

>TP1578

AAGCGAAACAAAGUGAAACAAAAUUAGCUGGGCAUGGUGAUGUGUGCCUGU

>TP1579

UAUGUCAGGGAAAAACUUUGAAAUUAGCUGGGUACAGUGGCUCAUGCCUAU

>TP1580

AUAAUCUCAGUGCUUUGGGAGGCCAAGGUGGGAGAAUCACUUGGCUGGGAG

>TP1581

UGAGGAGAGUGGAUGGCUUGAGUCCAGGAGUAUAAGGCCAGCUGGGACAGC

>TP1582

AAAGGGAAAUCUGCACAUCCCUUGAAGGAUGCCUUCCCCUCAAGGAAUUUA

>TP1583

AGCCUGAGCCUGGGUAGCAGAGUUUACAGUGAGCCAGGACCAUGCCACUGC

>TP1584

CAGCUUCUGGAAGUGUUGGGAUUACAGGUGUGAGCCACCACACCUGGCUCU

>TP1585

UGAAGAAGAAGGAUGGUUUGAACCCAGGAGGUUGAGGCAGUGAGCCGUGAU

>TP1586

UGGGUUGUAUGGUAAGGGCAUAUUUAUGAGAAAUAGCCAAACUCUUGCAAA

>TP1587

GCUAAACUUUAGGAGAAUCGGUAAUAACGUGUUGGUGACGAUGUGGAACAA

>TP1588

UGCUGGCAGGAGUGUGAACUGGAACAGUCACUUGGAAAAAAGUUUGGUGGU

>TP1589

CAUUUAUUUAUUUAUAAAUAGAGAUAGGGUCUUGCUGUAUUGCCCAGGUCU

>TP1590

AUUUAAAAAGAUGUGGUGGCCUGACACCUGCAAUCCCAACAUUUUGGGAGG

>TP1591

UGCAAUCCCAACAUUUUGGGAGGCCAAGGUGGGAGGGUCCCUUCCUUGAGC

>TP1592

AAUGUAGUGAGACUCAUCUCUGCCAAAAAAAAAAAAUUAGCCGGGCAUGGU

>TP1593

GCCUGGGACAUUGAGGCUGCAGUGAACUGUGAUGGCACAACUGCAUUUCAG

>TP1594

ACUGCAUUUCAGCCCGGGAGACAGCAAGAUUCUGUCUCAAAAAAAAAAAAA

>TP1595

CCUGUUCAAAUGAAAAUAAACCAAAAAGGAACUUUAUGCAUGUAAGAAAGA

>TP1596

CAAAAAUCAGCAUGCCUGUAAUCCCAGCUACUCAGGAGACUGAGGCAGGAG

>TP1597

UUUGGUUUUGAGAUGGGGUCAUGCUAGAGUGCAGUGGCAUGAUCAUGGCUC

>TP1598

UUUAUUCUGCCAUCCAAAUCUAACUAGGUGUCCUGUAUUUUAUCUGGCAAC

>TP1599

GAUUUCAAAUUCACUUGGCCUGCAAACAACAGAGUUAUCCGUAUCUUCCAC

>TP1600

AGCUUUACAGGUGGUUUUAAAGUUAACAGGGGUUUGUCAUGGUGAUUCACU

>TP1601

CUUAGUAGCUAAGGCUAGUGUUCAAAAGCACUCUAAAAGACAUUUUGUCCA

>TP1602

CUUUUCAUUUCCAUAGGCAGGAGGGAAGCACUACCACCCAACCUGUGCCAG

>TP1603

AUUCUGAGCUUGCAUGGUUGUCAUGAGGAGUGAGGGAUGUAGGCACAUAGA

>TP1604

ACCCUGUUGCUGCCCCGCAACAGAAAGUUUGUCAUUGGUCCCUCACAGCCA

>TP1605

CAAUUGACUGCAACCUCUGCUUCCCAGGCUCAAGUGAUCCUUCCACCUCAG

>TP1606

GCUGCCGCCUGCUAACAGCUUGUCUAGACAGGCCCCAUGGGGCUUCACCGC

>TP1607

AACUGCCCUUAUACCUUCAAAAAUAACACUUGCCAUAUAUCAAGUCCUUUC

>TP1608

GGUGAAAUGAUUAGACCCUGCCCCCAAACCAAGGCCUGGCCAAUUGGACAG

>TP1609

AAUGGAAAAUUUUUCAAGGUAGAGAAGAUGAAAACUCAGAACGCCCUCUUG

>TP1610

GGUGGCUUGGAAGAAGGCAGCAAAGAGAAAGAUCAAAGAAAAAGUAGUGAC

>TP1611

AGUGCAGUUUGAUGAUAGCUUACUGAAGCUUCCCACUCCUGGGCUCAAGUU

>TP1612

AACUUUAAAAAUGUGUCCAGGUGUUAGAUGAGUUCAUUAGACUCUUUUAAU

>TP1613

CAUUUAACCCUCACAACAACCCUAUAAGGUUAAGUACUGUUAUCACACCCC

>TP1614

CCCAGACAAGCUCUAUUUUUAUCACAAUGACCUUUAGAGAGGUCUCCCAGG

>TP1615

CCUGACUCUGGAGCCUGACCCCAUCAUCGUUCCUGGAAAUGUGACCCUCAG

>TP1616

UCCUGGAAAUGUGACCCUCAGUGUCAUGGGCAGCACCAGUGUCCCCCUGAG

>TP1617

UCGCUGCCUCUCUAAAGGGCAUAUAACAUGGCAUCUGCCACAGCAGAAUGG

>TP1618

GUUGGAAAUAUAAUUAAGGGUGGCAAGGACUGGAGUCAGUUGGAGAGUGCA

>TP1619

UGUAGAAGGAACGAUAGAAAUAGAAAAAAUGGCUGGGUGCAGUGGCUCACG

>TP1620

UUGCAUGGUUUUAAGAUCCCUUCACAAGAUACUAAUUACAAAGGGAAAAAC

>TP1621

CAUUCUUAAUUGCAUCAGCAGAAAUAGUUAAAAAGUACUUCUAAAACCACU

>TP1622

GACCUCCCAGUUCCAGUGGCUGAAUAUCAGGGCUCAGGCAUUAGAGGAUUC

>TP1623

UUAAAUUUAAUGCAUCCACUUUGGGAGGCUGAGGUGGGCAGAUCACGAGAU

>TP1624

CACCACGCCCAGCUAAUGUAGAGAUAGGGUUUCGACAUGUUGACCAGGCUG

>TP1625

AAAAAAAACCCAUUUUUUUUCAGACAGGAUCUCACACCGUUGCCCAGGCUG

>TP1626

GUAACCAGGAAAAGUUACAAGAAGAAGGGGCCAGGCACAGUGGCUCAUGCC

>TP1627

UGUAGUCCCAGCUACUUGGAAGGCUAAGGUAGGAGGAGGAUUCCUUUAGCC

>TP1628

CACGCUCAGCUCAUUUUGUAUUUUUAGUAGGUCAGGCUGCUCUUGAACUCC

>TP1629

AUUGAGUGCAUCUGUUGUCCCAGCUACUCAGGAGCUGAGGCAGGAGGAUUU

>TP1630

GAGGAUUUCCUUGAGCCAGGGAGGUAGAGGUUGCAGAUAGCCAAGAUCAAG

>TP1631

CUAUUCACAGAUGCAAUCAUAGCUCACUGCAGCCUUGAACUCCUGGGCUCA

>TP1632

ACUCCAGAAUGAGUGUUGACCACUGAAGCAUCUUUUAAGUCUGUGUUCCAU

>TP1633

GGAGGUACCCUGCCUGGAUCUAUAAAUGCAGAACCAUCAGAGAAUCUACAA

>TP1634

CCAAAUGGCAGAGAAGCAGUUAGAGAAAUCAGCCAGUGAAAAGGAACAACA

>TP1635

GGAACAACAGCUGGUGAGCACACUGAAGUGUCAGGAUGAAGAACUUGAGAA

>TP1636

AUGGCGACAGUGAUGAGGGGGAUGAAGAGGAAUGGAAGCCAACAAAAUUAG

>TP1637

GGUGUUCCUGCAAGGGCUGGUGUGGAAACAAGCAGUGUGGGUGCAGGAAGC

>TP1638

GCAGAUAGAGCUGGCCAGAUACAUUAAGACCAGUGCUCACUAUGAAGAGAA

>TP1639

UCCAGGGGUGUUACUCCUUGGAGGCAAUGUGGGCCAUGAGGUCCACCACCC

>TP1640

GAUUUCCAUUGAUGACAAGCUUCCCAUUCUCAGCCUUGACAGUGCCAUGGA

>TP1641

GGAAUUUGCCAUGGGUGGAAUCAUAAUCGAACAUGUAAACCAUGUAGUUGA

>TP1642

UUGGAAGGAUGGUGCUAUGGUUUGAAUGUUUUCCUCCAAAAUUCAUGUUGA

>TP1643

UUGGGGUCUGCUUGGCGUGUGGUGAAGCCAGGGGAAGAGAAGGGACAGUGG

>TP1644

GAUCAAAUUAAAGAUUAGCCUGGGUACCAUGGCUCACACCUAUAAUUCCAG

>TP1645

AAUCAUGGUGGAAGGGGAAGCAGGGAGGUUUUACACGGUGGCAGGAGAGAG

>TP1646

CCAUGUGUUGUGGGAGGGACCUGGUAGGAGGUAAUUGAAUCAGCCAGGCAG

>TP1647

CCGGCCCCUCUCUCACUUCUCAAAUAUCUUCUCCACAUAGCAGCCAAAGCA

>TP1648

AAUGUUAUUCCCAUCCAAAUUUUGCAAUGAUUGCUUUGGCUGCUAUGUGGA

>TP1649

GAUGUGCUGAAAUUUACUGUCAGCAACAGGAAACUCACACACCCUGCCGGG

>TP1650

AUGUGGGAUGGACCGGGCAGGGUGUAUGAGUUUCCUGUUGCUGCUGUAACA

>TP1651

GCUGGGUGUGAUGGUGGGCGCUUGCAAUCCCAGCUGCUGGGGAGGCUGAGG

>TP1652

GCCACCAGGCCCAGCGUAUUUUUGUAUUUUUGAGUAGAGAUGGGGUUUCAC

>TP1653

GACCCAGUCCCUGAGCCGCCCCUACACCCACAGGUGAGCGCCGCCACCCGC

>TP1654

AAGGUGAACUUUCAUGUUGAAUGUAAAGACUAUGUAAAAAAGGCAAAGGUA

>TP1655

GAAUCCAAGAGAUGAGAGAGGACCCAACAUGGGGCAGAAGCUUGAAAUCCU

>TP1656

AAAUCCGUAGAGACAGAAAGUAGAAAGGUGGCUGCUGGGGGCUAGAGAGGG

>TP1657

CUGGAGAUGAAGAGUGAUGACAUUUACACAACUUGUAGGUGUGCUUGGUGC

>TP1658

UACUGAGAGGUGACAGGGUGCUGGCAGCCCUCCCUCGCUCUUGGUGCCUCC

>TP1659

CUGGGCAGUGAGGGGCUUAGCACCCAUGCCAGCAGCUGCAGAGGAUGUGCC

>TP1660

GCACCCAUGCCAGCAGCUGCAGAGGAUGUGCCGGUCCCCUAGUAGUGCCGG

>TP1661

GAAGUAUGACAACAGCCUCAAGAUCAUCAGCAAUGCCUCCUGCAUCACCAA

>TP1662

GGAGACAUGCCACACUCAUCAGGGGACGUGACACACUCACCUAGAGACAUC

>TP1663

CUCUGAGCACAGAGAAGGAGGAAGUAAUGGGGCUGUGGAUAGGGGAGUUGA

>TP1664

GGGGCUGUGGAUAGGGGAGUUGAACAAUGAUACAAGGAGUGACUCCAAAUU

>TP1665

UGUUUCAUAGAAGAUAAGAACACAAAGGCUGGCCGGGUACUCUACACUUGC

>TP1666

GUACCUCAUGUCACUAUCGGGAAAGAGUGCCUUGAAUCAGCAGUAGAGCUG

>TP1667

UCCUGUGCCAGGAGGAGCAGGAUGCAUAUUGGAGGAUCCACAGCCUUACAC

>TP1668

CCAAGAAUCUGUGCAGUCAGAUGUCAGCAGUCAGCGGGCCUCUUCUACAGU

>TP1669

AGAUGUCAGCAGUCAGCGGGCCUCUACUACAGUGGUUGGAGGACAGACUGG

>TP1670

CAAGAACUUUCUUCUCUGGAAUAAAACAGGAGACAAAAUGGAGAAAGAUGA

>TP1671

UGGAAUAAAGCAGGAGACAAAAUGGAGAAAGAUGAAAAUAUCCAGUGUAAA

>TP1672

AAAGUUACUUAAGCUAAAUCAAUUUAGAAGAAGAAAAACUUGGAGGACUCA

>TP1673

GUGGUGGCUCAAACCUCUACCUCCAACACUUUGAGAGGCCAAGGAAGGUGG

>TP1674

UGCCAACUAUUACAAGAACAUACUGAUACUUAAUUGAGGGUGGCAGUUGAA

>TP1675

UUUAGCUAUAUGAUAAGCUCAAGUGAGAGCAAAAGUUCUGCAGUUUGAGCU

>TP1676

CACCUGGGUGUGCACGUAGUCAUUCAGCUUCACUUGCAGGCAGAACGGGGA

>TP1677

ACUGGGUGUGGUGGUAUAUGCCUGUAGUCUCGGUUAUUCAGGAGGCUGAGG

>TP1678

AUCAACAUGUAAACAGCUUGGCAAAACCCCAUCUCUACUAAAAAUAAAAAA

>TP1679

AAGACCAAGGAAGCAGGCAAGUGCCAUGAAGAGCGCCUCCAGGCCAAGAAG

>TP1680

UCCUGUACGCUUGGAGCGACCUUUGACCACGUGGCUGGCCUUGUUAUUUCA

>TP1681

AUGUGGCUAUACUGUUCACCCAGGAAGAGUGGGGGCAGCUGAGCCCCGCCC

>TP1682

GAAUGGCAAACACUCGGCUGGGCACAGUGGCUCACGUCUGUAAUCUUUGGG

>TP1683

CAAGCCCUCCAGGAAAUGGGCCUGCACCUCAGCCAGUAAGAACCCCACUCC

>TP1684

UAGUGCCACUGCACUCCAGCUGAGCAACAGAGUGAGACCCUGUCUCUCUCU

>TP1685

UAUUCUCAGCUCCUCGGGAGACCGAAGUAGGAGGAUUGCGUGGGACCGGGA

>TP1686

ACACCAUCCCCCACUUCCCGUGUCUACAAACACUAUGAAAAUUAGCCAGGC

>TP1687

GGACUGGUAGACUUUAAUGUGGAGAACUUCUGAAACAGCAGUUCUCAAAAU

>TP1688

UUGAUGGGGCCUUGUUCUGUUGCCUAGGCUGGAGUAUAGUGGCAUGAUCAU

>TP1689

UUCAGAACUCUAAGAAAGAAAUGGUAGCAUGGCUUGGUCUAAGUUGGAAGG

>TP1690

UCUAAGUUGGAAGGCCUGAGAAACAAGCACAUGGUGGAACUGAGUUCAAGG

>TP1691

UCAUGCCCUUGUACCACACAACUCUAGGUCCUUUGGCCUUUGGACUCUGAC

>TP1692

GCACCAGUGGCCCUCUGGGGCUCUCAAGCCUUUAGCCUUGAACUUAGUUAC

>TP1693

UACACCAUGUGCCUCCUUGUUUCUCAGGCCUUCCAACUUGGAUCAGGCCAU

>TP1694

AGGGGAGAAUAUUCAGUAUUUCAGCACUCCAGAGGGCUUCUCUCUCUUCAC

>TP1695

AAGAUCAGUGGAGCCUGGGAGGUCGAGGCUGCAGUGAGUGGAGAUGGCACC

>TP1696

GGGGCCUGGUGUCAUAAAGACAGGGAUGGGACCGGGACAGACUCUGGGGGC

>TP1697

UGACCUCAAAUGAUGCAUCCAUCUCAGCCUCCCAGAGUGGUAGGAUGUUGU

>TP1698

UUCUGCCUUUCAUCUCUGUAGGAUGAAGCCCCACCCGCCCUUCAGGAUGCC

>TP1699

CACUUUGGAAUUCUCUUUUCUACUCAUCUUAGCAACAUUUACCACCAGCAU

>TP1700

AAACCCGGAGGUGGAGGUUGCAGUGAGCUGAGAUGGUGCCAUUUUACUCCA

>TP1701

CUUGGAGUCCAAGCAUUUGCUGAUGAGUUGCUCAUUAUUCCAAAGGUUCUU

>TP1702

UGGCCAUCUGAUCUAUAAAUGCAGUAGCAUCGACAAAAGAACCAUUGAAAA

>TP1703

GAUUACAGGCGUGAGCCACUGCACCAGGCGUACAACUUGUUUUUAAAAGUG

>TP1704

AGUUGUUGUUUAUUCAUGGUGGUCAAACUGCCAAGAUACCUGAUUUCUCCU

>TP1705

GCUUCCAGUGGUACUGAUCACAGACAGAAUGUCUAGGAUUUAAGUAAAAUU

>TP1706

GAGUGAAAUUCCAUCACCAAAAAAAAAAAAAAAAAAAAAAAAAAGUGUGCA

>TP1707

GGCAGUGGGCGCCUAGCUGGAUCAGAGGCGCAGUGGACACCCUGCCGAAUC

>TP1708

GUGAAAGUAACUUUGAGUUAUAAACAUGAUACUUAAUGCACAGGGCUUCUU

>TP1709

AUACUUGUAGUCCCAGCUAAUCAGGAGGCUGGUACGAGAAGAUGGCCUGAG

>TP1710

GCCAGGCGUGAGGGUGUAUACUUGUAGUCCCAGCUAAUCAGGAGGCUGGUA

>TP1711

UAAUUAAUUACUGGCCAUGCACAAUAGCUCAGGCCUAUAAUCCCAGCACUU

>TP1712

UAUUCAGCAACAAAUACAUAAAGAAAAUGUGGUAUAUAUACACAAUAGAAU

>TP1713

UGCUCAAGACCUGGAUGUAGGCAGCAGUGCUGUUAGGUGAGAAAAAGAAAC

>TP1714

AUUUUACAUCUUUGGCAUAAGCUCGAGUGAGAUGAGGAGCCAGUACCCUGG

>TP1715

ACUCCAUCUGAAAGUUUUGCCAGCCAUUCAUUCAGUUUUUCCUUUUCAUAU

>TP1716

ACUUGUCCCACCAAACUUCAGCACAACUACUCCAUCUGAAAGUUUUGCCAG

>TP1717

AUAAUUCCUUUUUCUACCAUAUUCAAAAAAUCUCCAACCAUAGCAUCAUAA

>TP1718

GAUUCACUAUCAGACACUAAGAAUAAAUCUUGAUUUUCAUAAGCUCUGUUG

>TP1719

UCAAUGUAGCUUUCUGUUUGGAAACAGAUUUUUGCUCUUGUCGCCCAGGCU

>TP1720

UGGUCUUGAACUCCUGGCCUCCCAAAGUGCUGGGAUCACAGAUGUGAGCCG

>TP1721

GAUCUGCUGUCUAAAUGAUAUAUAUAUGAUAUAUAUGUGAUAUAUAUAUAU

>TP1722

UGCUAGAGUGCAAUGGCGCAAUCUCAGCUUACUGCAGCCAGUAAACGAUCC

>TP1723

GUCUCACUCCAUCACUCAGGGUGCUAGAGUGCAAUGGCGCAAUCUCAGCUU

>TP1724

UUUUUUGUUUUUUUGUUUGUGAAGAAAGAUGGUCUCACUCCAUCACUCAGG

>TP1725

GUGUGUCUUUUAAAAAUUUGUGGCCAGGUGCAAUGGCUCACACCUGUAAUC

>TP1726

GUUAAGCUGUACUGAGUUUUGUAUUAUAUUUUCUAUUCCCCGCCCCCCCUC

>TP1727

AAGUGCUAUAUAUAAAUGGUUGUUAAGCUGUACUGAGUUUUGUAUUAUAUU

>TP1728

UACUUACAGUACCUAAUACCAUGUAAGUGCUAUAUAUAAAUGGUUGUUAAG

>TP1729

AUGUUAAAUUAUCUCUAGAUUACUUACAGUACCUAAUACCAUGUAAGUGCU

>TP1730

CUACGUACAUCCUCUUAAAUAUGUUAAAUUAUCUCUAGAUUACUUACAGUA

>TP1731

CAAAAUUGAGCAGUUAGCUGUGUUUAGAGAACAUGGUCCAUAUACCUUUGA

>TP1732

CUGCAGCCUUGAACCCCUGAGCUCAAGUGAUCCUCCUGCAAUGGGCUUCCA

>TP1733

AUCAUUCAUGAGAAAGCCAUGUCAUAAUCCAAUCACCUCCCACCAGGCCCC

>TP1734

UCUUUGAUAAAGGGCAUAUUCUGUUAACCCUCCUCGAGGACACAGGAAGGG

>TP1735

UUGAAUUGUACUCCCAUAAUUCCCAAGUGUUGUGGGAGGGAGCUGGUGGGA

>TP1736

CUGUAUUAUUAUUAAUUUAUUUUUUAGAGGGAGUUUUGCUCUUGUCACCCA

>TP1737

UAAGGGAGAAGCUAGGCAUGUGUGCAGGUAGGGGGUAAAUGGGAAAUCUCU

>TP1738

UUGAGACUAGUCUGGCCAACAUAGCAAGACUCCAUCUCUAAAAAAAAUUUU

>TP1739

UUUGUACAUUUAAAACUAUUAUUUUAGGCCGGGAGUGGUGGCUCACACCUG

>TP1740

UUUUGAUCUCAGUUUUUUUAUUUUUAUUUUUGGAGACAAGAUCUUGCUCUG

>TP1741

AUUUUUAUUACUGUAUUUGUAAAUGAGUUUGAAGGAAUUUGUAAAUGCCAC

>TP1742

UAAAUAGCUUCUGCACAGCAAAAGAAACUAUCAUCAGAGUGAACAGACAAC

>TP1743

UUGACGAAUGGGAUCUAAUUGAACUAAAUAGCUUCUGCACAGCAAAAGAAA

>TP1744

GAUUUCACAAUGAAAAUGCUAAAGCACAGCAACAAAAGCAAAAGUUGACGA

>TP1745

UUUCCAGUAUACCCCGAUCCUUAAAAGUCUGGUAUAGCUUUUUGCGCAGUU

>TP1746

GGUUUCGAAGUUGUAUCUUGAGUGUAUCCAGUAUACCCCGAUCCUUAAAUG

>TP1747

CUUUUCUUUUGCCAAACCACUUUCUAGAAAGAAAACAGAAAGUGAAUAUUC

>TP1748

CAGCUCCUGAAACCAGUGAUUUGUAAAGACUGGAAGUAGGGUUGAUUUUAA

>TP1749

AAACCUUUUUGGUUUUCUUUAUCAGAUCCUGAAACCAGUGAUUUGUACAGA

>TP1750

CUCAAAACUCUUUAUAUUCUGCUCCAGUCUCCUAAGUGCCUCAGUCAUACU

>TP1751

UUCAUAUAUACAUAUGUAUACAUAUAUACACACAUAUACAUAUACAUACAC

>TP1752

UAUACAUAUGUGUAUAUUCAUAUAUACAUAUGUAUACAUAUAUACACACAU

>TP1753

AUUCACAUGUACACAUGUAUACAUAAGUGUAUUCACAUGUACACAUGUAUA

>TP1754

CAUGCAUACAUAUAUGUGUAUUCAUAUGUACAAUGCAUGCAUAUGUGUAUU

>TP1755

UAUAUUCAUAUACACGCAUAUGCAUACAUAUAUGUAUAUUCAUAUACACGC

>TP1756

ACAGUUUACAUGUAUGUAUAUAUGUACACAUGUAUUCCAGUGCGUGUAUAU

>TP1757

UAUAUAUACAUGCAUGUGUACAUGUAUACAUUGUAUACAGUUUACAUGUAU

>TP1758

UAUUCAUGUGUAUAUACAUGUAUAUAGGUGUACAGAUGUAUAUAGUAUGUA

>TP1759

UAUGUAUAUACUUGUAUAUGUAUUCAUGUGUAUAUACAUGUAUAUAGGUGU

>TP1760

GUUGUCACUGCUCACAUAUACACGUAUAUACACGUGUAUACAUAUACAUAU

>TP1761

AUUAACUAGACACAGAGCGCUGAUUAGUGCGUUUAUAAACCUUUAGCUAGA

>TP1762

GAUUGGUCCAUUUUACUGUGAGCUCAUUGGUCCAUUUUAUAGAGAGUUGAU

>TP1763

UAUACACACGUACAUAUCGAUAUGUAUAUGUAUACAUAUGUGUCCGGAAUU

>TP1764

CACAUGUGUAUACAGGCAUGUAUAUAUGUGUGCUUACCUACGAAUAUACAU

>TP1765

UAUGAAUACAUGUGUAUCUACAUGUACACAUGCACACAUGUAUAUGCACAU

>TP1766

CAUGUAUGUAUAGAUGUAUACAUGUACACACUUAUGAAUACAUGUGUAUCU

>TP1767

ACCAUGUAUGUAUGCACACGUAUACACAUGUAUGUAUAGAUGUAUACAUGU

>TP1768

CACGUACCCAUGUAUGUACACGUAUACACAUGUACCAUGUAUGUAUGCACA

>TP1769

CACGCGUAUACACACGUACCCAGGUAUGUAUGCACGUGUAUACACACGUAC

>TP1770

UGAGUAUUAUUCCAGUGUGUGCAGUAUGUAUACACACGUAUACACAUGUAC

>TP1771

CAGGCGUCAGCCACUGCGCCCGGCCAAAAAUGCCAUCCAAUCUUUACCGUG

>TP1772

UCCUAGUCUCAAAAUUCUGGCCUCAAGCAGUCCUCCCACCUUGGCUUCCCA

>TP1773

UAGUGGCACAAUCACAUCUCACUGCAGCCUGGACCUCCCGCACUCAAUCGA

>TP1774

UGAAUCUGUGGCGGCCGGGUACGGUAGCUCACGUCCUAUAGUCGCAGCACU

>TP1775

AGCUGAGAUCAUGCCACUGUGCUCCAGCCUGGUGGCACAGUGAGACUCCAC

>TP1776

GCGUGAGCCACCACUCCCGGCUGUCAUCAUCAAAUUUUCAAGUGAAGAUAG

>TP1777

UUUUGUAGAGAUGGAGAUUCACUCGACUAAUUCUUUUUGUAUUCUUAGUAG

>TP1778

GCCUUUCUUGUUUACAGAUAGUGCAAUGGCGCAAUCCUGGCUCACUGCAGC

>TP1779

AACCCAAGAGGGCAGCUGUUGCAGCAUCCAGUUCAUCUUAAGAAUGUCAAC

>TP1780

UGUGAUCAUACUUUAUUUUAGAGACAUCAUUCUGGCUGCAUUAUGAAUGGA

>TP1781

UACCACCUCAGCACUCGGAAUAGCUAGGACUUUAGGUCUGUGUGCUACCAC

>TP1782

CCUGUAACAGUUGGGAUGCAGUGGCACAAUUGUAGCUCACUGUAAUCUCUA

>TP1783

UGCGCCAGUACGCCUGGCUAGUUUUAGUAUUUUUUGUUACAGACGGGGUUU

>TP1784

GGCUGGAGGGCAGUGGCAGGAUCUCAGUUCACCCCAACCUUUGCCUCACGG

>TP1785

CAUUUCUCCUAUUAAGCUGCCUUUUAUCAGUUGAUUUUUCAGUGGACCUUC

>TP1786

GGUUCCCCUGUUGAAGCUUGCUAGGAAUUACAGUGGGUUUGACUCAAUUCA

>TP1787

UUUAUCACCUUGCCUGGCGUGCAGUAGUGCCACCUUAGCUCACUGCAGCCU

>TP1788

AUAUCACUCUGUUGCCCAUGGGGAAAUGCAGUGGUGCAGCCUUGGCUCACU

>TP1789

ACUGCAGCCUUCAACUCCCAGGCUGAAGUGACCCUUCUGCCUCAUCCUCUG

>TP1790

GACAGGCAGACCAGGAAGGCUGGGAAAAGGCUGCUGGUACAGGAGAUUCAA

>TP1791

UGCUGCACUGUACUCAUGUGAAUUUAUCUAUUUUAACUUCUGUACCAGCAG

>TP1792

AGGUUUUCAAUUCAUUUGUCCAAAUACGAAGGAGUAAGGAUUACAGGAUAG

>TP1793

UUCCCCCUCCUCCCUAACAACUUCUAGCAGCCAUUGAUCUUUUUAUUGUCU

>TP1794

UAAAAGCUAAUGGACUUUAGUUUUUAGAGCAGUUUCAGGUUUACAGCAAAG

>TP1795

AAUCCUGUUCCUUGGCAUUUACCCAAAUGCGUUGAAAACUUAUGUCCACAC

>TP1796

GAACCCGGGAGGCAGAGGUGAGAUCAUGCCACUGUACUCCAACCUGGGUGA

>TP1797

UUUAUUAUAAAGUAGGUUUUGUGUUAGAUGAUUUUGCCCAACUGUAGGCUA

>TP1798

AAGAAAAGUAAGGGCAAAAUCAACUACUGAGAACAACGAAGGGGCAGGAUA

>TP1799

AGUAAAUUACAUAAAUAUUUCAUGUAAUUUAUUGAAUACUGUACUGAAAGU

>TP1800

ACUUUGUAUGGUGUGAACAUGGCUAAUUCAGCUUUAUUCUGGGGUAGUGCC

>TP1801

CUCCCAAGUAGCUGGGACUGCAGGUACACACCAUCAUGCCCAGCUAAUUAA

>TP1802

UGUCCAGGCUGGAGUACAGUGGCACAAUCAUGGCUCAUUGCAGCCCCGAUC

>TP1803

UGGGGUUUUUUUGAGAUGGAGUCUUACUCUUUUGCCAGGCUAGAGUGGUGU

>TP1804

AGUGCUGGGAUUACAGGCAUAAACCACAACUCAAUAAAAAAGUUUUUAAAA

>TP1805

UCCUCCCAACUCAGUCUCCCACGUAACUGGGACUCCGGCACAUCACCACAU

>TP1806

AAGUCCCAAACAGGCUACGUCUGGUAGCUGAUGCCUAUAAUCCCAGCAUUU

>TP1807

CAGUUUUUAAAUCUUUAUAAGUUAAAGCAAAUAGGCCAGGUGGGUGGCUCA

>TP1808

ACCUAGCCUCCCAAGUUUUGGGACUACAGACGUGUGCUACCAUGCACAGCU

>TP1809

GCUGGGUAUUCAAUGGGCAUUUUCAAUCUGAGCUCUUGGGUCUCCCAUCAA

>TP1810

GCACAUGCCUGUAGUCCCAGCCACUAGGGCGGCUGAGACGCAAGACUUGCU

>TP1811

AUGACACAUGAGCAGACAUGACAUGACGGGAUUGAUGGAGCAAUGACCUCA

>TP1812

UAGAAUGAGGACUAAACUACGAGUCAGCAAACUUUUGUUCAAAUCUAAAGU

>TP1813

UCAUGUUUACCUGGCAGUGAAUGCAAGUUUUGAUUACAAGAAUCUGCCUCA

>TP1814

CAAGCUGAGGACUGUGGAGCGAAUCAGCUUUUUAAUGUGCUUGCUCACCCA

>TP1815

CAGAAGUUCAAGAUUACAGUGAGUUAUGAUUGUGCCGCUGCACUCCAACCU

>TP1816

ACACACUAAAGACUAGAAAGUUCUUAAAUAGAGAAAGUUGUUUAUCAACAA

>TP1817

AUAGCAAGGUUGUAGCGUCCUCUCCAGUGGGGGGAGAAAAGGAACUGUGCC

>TP1818

UAAAUGAAUGUGAAAGGCUAUUUAGACCUCUGCCUUUUCACCGUCCUCCCA

>TP1819

CUUUUUGGGAGUUACUGCCUUAGAAAACUAUCCAGAUAGUUGUGACCAAAG

>TP1820

GAAUACUUUGGAUGUCUGGUGGGCAACUUUUUGGGAGUUACUGCCUUAGAA

>TP1821

AGGCUUAUCUGGAACUCCUGACCUCAAGUGAUCUGCCCCCGCUUGAUCUCC

>TP1822

UGGGCUUAAGCAGUCUUCCUCCCUCAGUCUCCCAAAUAGCUGGGACUAUAC

>TP1823

UUAUCCUUGGCUCUCUGUUCUUUGUAGUUGAAAUAUCUUCAGCCACAAAGU

>TP1824

GUUAGAAAUCUUCCACCCCGUGGCCAGGCGUGAUGGCUCACGCCUGUAAUC

>TP1825

AGAUAAAAAAAAAUGUUUUAAGGCAAGUUCUCACUAUGUUACCCAGGCUGG

>TP1826

UUAGACAGGGUCUUACUCUUGCCCAAACUGGAGUGCAGUAGUACAGUCAUG

>TP1827

AAAAAAUAAAAUAAAAAAUUUAGACAGGGUCUUACUCUUGCCCAAACUGGA

>TP1828

AAAUGUAACAAUGUGUAACAUUUUGAGAUGCAGUCUUGCUCUGUCAUUUAG

>TP1829

UAAGGCAGGCAGAUCACUUGAGACCAGCUUGGGCAACAUGGCAAAGCCCCA

>TP1830

GAAACAAAACAUUAAAAUGUGACUCACUCCAAACAGAAAGAUGGAAAUGUA

>TP1831

AUCAUCCUCCAGACCCCACAAGGGUAGAUCCACUGACAGCUUGCACUGUGU

>TP1832

UAAGUCUUCUGAGGCCUCUCCAGCCAUGACUCCUGUACAGCCUACAGAACU

>TP1833

UUCCCGCAAGAUCUGAUGGUUUAAAAGUGUGUGGCGCUUCCCCACUUUACU

>TP1834

UGUUGUGGUGUAAGAGCAGAGGAAAAACAAUUUGGGAGUUUUUCCUAAACA

>TP1835

CACCUUUAUGAUCCAAACACCCACCAGACCCCACUUCCAACAAUGGAGGUC

>TP1836

UUUCAGGAUGAAACUGUUUCAGCUCAGAUCAUCAACCAUUAGAUUCUCAUA

>TP1837

GCGGCUCAUGGUUCUGCAGGACUAUAGAGGAAGCAUGGUGCUGGAAUCUGC

>TP1838

UGAGACUGGGUAAUUGGUAAAGAAAAGUGGUUUAUUGCGGCUCAUGGUUCU

>TP1839

CCUCAACCUCCAGGACUCAGGUGAUACUCCCGCUGCAGCCUCCCAAGUAGC

>TP1840

AUGUCUUUAUCAGCAGCGUGAAAACAGACUAAUACAAUUUCUUUCUCUACU

>TP1841

CUUCUCCAGCCAUUGUGGAACUGUAAGUCCAUUAAACUCUUUUUCCUGUAU

>TP1842

GUGAAUAAGUCUCAUGAGAUCUGACAGUUUUAGAAGAGGGAAUUUCCCUGC

>TP1843

UGGAAUGUUAUGAUAGGACAUAGUAAUAGCGGUGGUCAGACAUGGAAAUGG

>TP1844

UGGGAUUACAGGCAUGAGCCGCCACAUCCGGCCCAUUUUAACCACUUUUAA

>TP1845

AAAUCGACUGUUGUAAUAUCAUAGUACUUGUGUUCAAGUAAUCCUUAUUUU

>TP1846

UGGUAGCCAUCUUGGGCCAUUAUUAAGUGCAGUAAGGGUUACCUGGACACA

>TP1847

CUGAAGGUGGGGCCUAGGCACUCAUAUUCUAUAUGGCCCUAAUGAUUCUAA

>TP1848

AUAAAAGUGGAAUUGGAAUUUUUAUACUUAUUUGUUGUAGUGAAUGGUUUA

>TP1849

GUACUACUUGGUUCCUGAUAUGGAUAAUGAAGAAGGAGAAGGAGAAGAAGA

>TP1850

AAGUGCUGGGGGGAAUUUUUUUUUUAUUUUUAAUGCAGAAACAAGAAAAAA

>TP1851

CCUUAAGGGGGAGAUGGGGAAGGAAAGAGGGAAAUAUCCCAUAAAGCAAAA

>TP1852

CUAUACCUGUACUGUCCAGUGUGGUAACCACUAGCCACAUGUGGCUAUUUG

>TP1853

AAAAUUCAGAAUGACAUGUAAAAACACAAUGAAGUUUGCACUGUUCACAUC

>TP1854

GAGCCUGGGAAGUUGAGGCUGCAGUAAGCCCUGAUUGCACCCCUGCAUUCC

>TP1855

UUUUGCCAUUUAAAAGUAAUGGCAAAAACCACAAUUAAUAUUUGUAUACAU

>TP1856

GUUCCAUCAAUAUUAACUGGGAGUAAUGAUAGUGAUGAUCUUCAUGACUAA

>TP1857

GAUUUUUUUUGUUUUAAACAGAGACAGCGUCUUGCUAUUUUGUCCAGGAUG

>TP1858

CGUUGCUAAAGAUUUCUCUAUAAUAAGCCACACAUUAUAUUUAGACUAUAU

>TP1859

AGCAACCACUCCUCAUGUAAUCUUUACCUCUCUCUUUCCAAUAGCUUCUCU

>TP1860

AUUCUUCUUGUGCCUUCUGCUCCCUAAGCAACCACUCCUCAUGUAAUCUUU

>TP1861

CUUUAACUUUCUCUCUUGUUCUUCUAGCCAUUUUUUAGCCGCCUCUUCCUU

>TP1862

UUUGCCAUGUGGUACUAUUUUCUAAAUCAUUUUCAGCCUGAUCCAGCAUCU

>TP1863

GGAAAUUAUGUUUACGUGAACAUCUAUCUCCAAAUCUGCAAGCUCCUGUUU

>TP1864

ACCGGGUCACUGGGCAGAAUUCACAAUGCAGCUGUCGUCCUGCAUACCAUC

>TP1865

UGAACACAUGAAGAAAGUUGCAGUGAUUUCCUCUUGGACAUUGUUGUAUUU

>TP1866

GCAUGUUUCUUUAAAUUUAUAGAUAAGGAAUUACUGGGGCAAAAAGUGUGC

>TP1867

CUCAAUACUAUGCAACUGUUAAAGUAUGGAUAUUGGAAGGCAUUCGUAAUG

>TP1868

UGAUACAUUUAUGUAACUCAAUACUAUGCAACUGUUAAAGUAUGGAUAUUG

>TP1869

AGGAUCUCACAGCCACAUUUUUGUAAUUGUGAAAAAUAAGACACAUUAUGA

>TP1870

UUUGGCCCAGAUACUCUCUAUCUAUAGAUUUAGCAUAAAGAAACAUGCAAA

>TP1871

UAUCAGAAUUUUUUAAAGUGUGCACACUUUUUGGCCCAGAUACUCUCUAUC

>TP1872

GCCAGAUGUAUGUUUGUUUUGUUUUAGCAAAUAACUUUAUGUCUGAGAGAC

>TP1873

GUGCUGAUCACUUGACUCCUGUCUUAGUCCAUUCUGGCUGCUCUCUCAGAA

>TP1874

GUAGAUGUAUUUCUUUAAAAUAGGUAUGGAUGGAUAUUUUUAUGAAUAAAU

>TP1875

UUAUUUAUUUAUGUAUUUUAGAGACAGGGCCUCCUUCUGUCACCCAGUUUG

>TP1876

AAAAUUAAAAAAAAAAUAAUUAGCCAGAUGUGGUGGUGUGCACUUGUAGUC

>TP1877

AUGAUUAUUUCUCACAGUUUUCAUGAUCAUUUGUUUAUUAUUUACUUGGAU

>TP1878

AUGUUUGAAAUGUUUGUUUUUGGGUACUGUAAAGAAAUAGUACUUGAACAU

>TP1879

UAGUUUGGCAGUUCCUCAAAAAGCUAAACAAUAGUCACCAUAUGACCUAGC

>TP1880

ACAAGUAACUAUUCCCAAAGAUUUGACUGGAUCUAUUAUUGGCAAAGGUGG

>TP1881

UGAUCUUGGUGGACCUAUUACUACUACACAAGUAACUAUUCCCAAAGAUUU

>TP1882

UCUUUUUUUUAUAUAGAUGUAUUUUAUUAUACUUUAAGUUCUAGGGUACAU

>TP1883

UAAGCAGUCUGCUUUCCAAAGUGCUAGGAUUACAGGCAUGAGCCACCACAC

>TP1884

UGUAAUCCCAGCACUUUGGAAGUUAAGGUGGGCAGAUUGCCCAAGCGCAGG

>TP1885

CAUAGUGCCUUGUGCCUGUAAUCCCAGCACUUUGGAAGUUAAGGUGGGCAG

>TP1886

UUAAGUUUUUUUUUUGGUUGGGCAUAGUGCCUUGUGCCUGUAAUCCCAGCA

>TP1887

CCAGCUAAUUUUUAAAUUUGUAGUAAAGAUGGGGUUUUGCCAUGUUUCUCA

>TP1888

UUGUUGGAUCCUACUGUUGGAUGGUAGCUCUAUUUUUAGUUUUUUGAGGAA

>TP1889

UCCAUUCAUCUGUUGAUGGACACUUAGGUUGCUUCCAAAUCUUAGCUUUUA

>TP1890

AUUGUAUGUACUAUUUAUAUGUCUUAUUUGGUUUUUACUAAGCUUCUUCAU

>TP1891

UCCACCUUGGAUCUACAACAGGAUCAGAAAAUGAACGUUAUAAAUAUACCU

>TP1892

CCCCUGGGCUCAAGUAGCUAAGACUACAGGCACGUGCCACCAUGCUCGGCU

>TP1893

UUGUAACUUUUGUCACCCAGGCUGAAUUGCAGUAGUAAUCAUGGCUCAAUG

>TP1894

CCAGGCGUGUUGGCAUGUGCCUGUAAUCCCAGUACUUUGGGAGGCCAAGGU

>TP1895

CAGGUGUGAGCCACUGCACCCGGCCAACAUAGUCUUUUAUUAUCAAGUAUU

>TP1896

AAGUAGCUGAGACUACAGUACAGGCACAUGCCACCACACCUGGCUAAUAGA

>TP1897

GUCUUAAAAGAAGCAUGACCUGGCCAGGUGCGUGGCUCAUGCUUGUAAUCC

>TP1898

UUACGUGUUUACAAUGAGUCUUAAAAGAAGCAUGACCUGGCCAGGUGCGUG

>TP1899

CUAGGAGGUCAGGAGAUUGAGGCUGAGGCUGCAGUGAGCUAUGAUUGCCCC

>TP1900

GCCUCGCCUAUAAAAUUUUUUUAAAAUUAGCUGAAUGUGGUGGCAUGCACC

>TP1901

UGCAAUCCCAACACUCUAGGAGACCAAGCUGGGCGGAUCACCUGAGGUCAG

>TP1902

UGGCUGAUUUUUAGAAUUUUUUUAGAGAUGCUGUCUUGCUAUGUUUUCCAG

>TP1903

CUCUCAACUAGGUGGCACUACAGGCAUGUGUCACUGCACCAGGCUUAGUAU

>TP1904

UAGUGUACCAUGUUUGCUUGUCUUUAGAGAUGGAAUCUUACUAUGUUGCCC

>TP1905

GCUGAGGCAGGAGUCUCUGGGAGGCAGAGGUUGCAGUCAGCUAAGAUGAUG

>TP1906

UAUAUGGUAGGUGUCUAUGCCUCACACCUGUAGUCCUAGUACUUUGAGAGG

>TP1907

GCUAACACGUCUCUACUAAAAGAAUACAAAAAAUUAGCUGGGUGUGGUGGC

>TP1908

CCUUUGGUUUAAGAAAAGCGCUCCUACUGGCCAGGCGCAGUGGCUUAUGCC

>TP1909

CUAAAGACUGCCUAAAGCUAACCUGACCUGUUACCUUGCUGCUUUUUCCUU

>TP1910

GGCUUGAAACCAGGAGGUCGAGGCUACAGUGAGCUAUGUUUGUGCUACUGC

>TP1911

UGUCAACAAAGACUCAUUGAGUGUCACAAGCUCUGGCCACAAAAUGAAGGA

>TP1912

GGAGGCUGAGGUGGGAGGAUCGCUUAAGCACAGGAGGUCGAGUGAGCCAUG

>TP1913

GGAGUUUGCAACAUGAUGAAACCUUAUCUCUACAAAAACAAAAUACAAAAA

>TP1914

UCAAGUACAGUGAUUGCGCCACUGCACUCCAGGCUGGGCAAUAGAGCAAAA

>TP1915

AUUGCUUGAGCCCAGGAGGUCAAGUACAGUGAUUGCGCCACUGCACUCCAG

>TP1916

AAAAAAAAAAAAAAAAGAAAAGAAAAUGGGAUGAAAUUCCACUUUCUUCAC

>TP1917

AUGAGACUCCAUCUUAAAAAAAAAAAAAAAAAAGAAAAGAAAAUGGGAUGA

>TP1918

UUAAUGAAAAUGGGAUGAAGUGGCCAGCGGCUGUAAUCACGCCUGUAAUCC

>TP1919

CAUCACCUGAGGCUACUUAAUGAAAAUGGGAUGAAGUGGCCAGCGGCUGUA

>TP1920

UCUACAAAAAGUGCAAAAUAGGCAUAGUGGUGCACACCUGUGGUCGCAAUU

>TP1921

GUAUGCUUAUUGUCGAGGAGCUAUGAAUCUAGUUUGAAAGAAAAGCAUGGU

>TP1922

GAGAUGGGACUAUGACUUGAGCUCAAGAGUCUGAGGUUGCAGUGAACUAGC

>TP1923

AUGGGGCAUAGUGAUAGACACUGAUAGUCCUACCUACUCAGGAGGCUGAGA

>TP1924

UGGCUGGGUGUGGGGUCUCAGGCCUAUAAUCCCAGUGCUUUGAGAGGCUGA

>TP1925

AUUUUCAAGAAUAUAAUACAUUGUUAUUAACUAUAGUCACCACGUUGUACU

>TP1926

AAGAACACUUAAAAUCCAUUCUCUUAACUAUUUUCAAGAAUAUAAUACAUU

>TP1927

CAUCAUAUACUUAUUUUUUUGUGGUAAGAACACUUAAAAUCCAUUCUCUUA

>TP1928

CCACCAAUUGCCUGGGCAUCCGUGCAUUUGCAGAUGUACACACCUGCACUG

>TP1929

ACCACACACGGGGUUUCAAUUUGUUAGCCAUGCUGGUCUUGAGCUCCUGAC

>TP1930

AGCGAGUGAAGCUUCAUUUGUAUUUACAGCUGCUCCCCAUGGCUCACAUUA

>TP1931

GAAAUCCUGUCAUUUGCAACAGUAUAGAUGAACCUGGAGGACAUUAUGCUA

>TP1932

CUCAUCAGAAACUUUGAAGACUACAAGGCAGUGCAAUGAUAUAUUCAAAGU

>TP1933

AUUUCGGCCCAUAAAUUAUUUUAAAAGCUAUUUAUUCGCUUAUGAACAUUU

>TP1934

UAUAGAUCAGGGCUGACCGGGCAUAAUGGCUCACGCCUGCAGUCCCAGCAC

>TP1935

UUUUUUUUUUUUGGAGACAGUCUCUAUAGCCCAUGGUGGAGUGCAGUGGCA

>TP1936

UGUGUCUUCCAUAGAACUUAACACAAAGCUUUAUACAGAGAGUGACCAUAU

>TP1937

GAAAAUAUUUGCAAACUACUGAUCUAAUAGGGAAUUACUAUCCAGAAUAUA

>TP1938

CAAUAAGCAUGUGAAAGUAUGUUCAACAUCAUUAGUCAUUAGGGAAAUGCA

>TP1939

GGCUGGUUGGUUUGUUUUGUUUUUGAGACAGAGUCUCGCUCUGUCUCUCAG

>TP1940

UGGGAGGUCCAGGCUGCCAUGAGCUAUGACUGCACUGCUGCACUCUAGCUU

>TP1941

CUGUCUCUACAAAACAUAAAAUACUAGCUGAGCACAGUGGCGUGUGCCAGU

>TP1942

GAGUGAGUAGAUGAUACAUACAUGCACUCUGGUUCUGCUCCUAGCUGGCUG

>TP1943

AAUUUCCAAUUUCUGGCCGGGCGCUAUGGCUCACGCCUGUAAUUCCAGCAC

>TP1944

AAUCAAUAGGUGGGACUACUACACUAGCCAAAGCUGGCAUCCUUGUGGUCU

>TP1945

GGAUGGCAGCUUUGGCCAGUGUAGUAGUCCCACCUAUUGAUUAGAGUCAAA

>TP1946

UGAAUGAGGUCUUCCAAGCAAAUGAAGCCAAACUUCCCCAGGUGCUCCUCA

>TP1947

CAAUAAUUAAAACCUGGCUAUGGCCAGGCACUGUGGCUCACGCCUGUAAAC

>TP1948

GGCUGCAGUGUGAAGUAGAAGGGUCACAUGAGCCAGGAGGUCGAGGUUGUA

>TP1949

CAUGUGCCUGUAGUCCCAGCUACUCAGGAGGCUGCAGUGUGAAGUAGAAGG

>TP1950

UUUCUAUAAAAAAAUUAAAAAAUUAACUGGGUGGGUUGACAUGUGCCUGUA

>TP1951

AAAGCAGAAUCCGGUCAAGUCCAAAAGGUGAGCACAGUAGCUAAAGCAGAA

>TP1952

GAGUGUGGUGGCCAGUGGGAGAGUCAGUGUCACCCGCCAGCAACUUCUCCU

>TP1953

CUAUAAUCCCAGCAUUUUUGAGGCUAGAUGGGAAGAUCACUUGAUCUCAGG

>TP1954

AGAUAAUCAAACUAAAUAGACGUCUACAACUUCCGGACGAGGAGAACAAAG

>TP1955

GACAGCUGGUCAGAAAUGAUUCUCUAUAUGGCAUUUCAAAUACAGAUACAA

>TP1956

AGUACAAUUCACUUAGUGGUUAGUUAGGCCAAUCCUCCCCGCCAUUAUCCU

>TP1957

AAUAUCCCAGAUAAGGAUGUCUCAUAAAUCAAAGCUGCAGUACAAUUCACU

>TP1958

UCAAAAAUGAGUAAAGGAUCUGAAUAGACAUUUCUCCAAAGGUGUACAAAU

>TP1959

AAAAGGCAAAGAAUCCAAUUCAAAAAUGAGUAAAGGAUCUGAAUAGACAUU

>TP1960

GACUUAUACCUAUUGCAAAAACCACAAUUACUUUUGCACCAACCUAAAAGA

>TP1961

AAUGGAUUCCUGGAUUUGACACCAAAAGCAUGAACAACAAAAGAAAAAAUU

>TP1962

AAAUCUUCAUGACCUUGGAUUUGGAAAUGGAUUCCUGGAUUUGACACCAAA

>TP1963

GAAAAAAAAAAAACCUACAGGGGUAAAUCUUCAUGACCUUGGAUUUGGAAA

>TP1964

UAAGAGCUAAAGUUAUAAAGCUCUUAGGAAAAAAAAAAAACCUACAGGGGU

>TP1965

GAUCAAAGACUAAAUGUAAGAGCUAAAGUUAUAAAGCUCUUAGGAAAAAAA

>TP1966

UAAAAAUUAACUUAAAAUGGAUCAAAGACUAAAUGUAAGAGCUAAAGUUAU

>TP1967

GCUACAUGCAAAAGAAGUUAGUUGGAACUUACACCAUAGGUAAAAAUUAAC

>TP1968

AUGGUGCUGAAAUAACUGGAUAGCUACAUGCAAAAGAAGUUAGUUGGAACU

>TP1969

AAAAGAAAAGAAUAGUCUUCAAUAAAUGGUGCUGAAAUAACUGGAUAGCUA

>TP1970

CCAAAAGUAUGAGCAACAAAAGAAAAGAAUAGUCUUCAAUAAAUGGUGCUG

>TP1971

CUAUCUCUCUGAUUUUCACACCAAAAGUAUGAGCAACAAAAGAAAAGAAUA

>TP1972

UCUCAGCUGCUAGGGAGGCUGAGGCAGGAGGAAUACUUGAGCCCAGGAAGU

>TP1973

AGUAACUCUUCAUAGGUGUGAUCAUAGUGCACUAUAGCCUUUAACUCCUGG

>TP1974

CUUUAUUUGUUCAUUGGUUUGAGACAGUGUUUCACUAUAUGGCCCAGGCUG

>TP1975

AUUGAAACCUACAUAUAUUGCUGGUAGGAAUGCAAAAUGGUAAAACACUUU

>TP1976

AGGGUUUGAAUAGACAUUUCUCCAAAGAAGAUAAACAAAUGGCCAAUAAGC

>TP1977

GCCUAUCAGAACAUCUCCUUUAUUAACUGCCGGAUCAGUGUUGUGUGAACU

>TP1978

AUACGCCUUUCCAAUCUUUAUUGGAAGUCUCUCCCUGCCUACUUCCUAAUU

>TP1979

CAUAGUCCCUUCCCCCUGAAGCAAUAGCCCCUCCCCACCUCCUGCAAUACG

>TP1980

UUGGAGUGUUUUUUUGUAUUGCCAUAGUCCCUUCCCCCUGAAGCAAUAGCC

>TP1981

AAAGCCUUUUUCUGCCCCAGCUCUGAGACACUUGCAGAUCUUAAGGUCUGA

>TP1982

AGCCUUGCUGACUUCUCCCUAUAAAAGUAAAGCCUUUUUCUGCCCCAGCUC

>TP1983

UGCCUGCUAGCCAUAGAUUCCUUUCAGCCUUGCUGACUUCUCCCUAUAAAA

>TP1984

CCAGGCAGAAAAGGACUUUUCUUUUAUUGGAAGAAGUAAACAUGGCUAGAA

>TP1985

GCAAGCGUCUCAAAGCCCAGGCAGAAAAGGACUUUUCUUUUAUUGGAAGAA

>TP1986

UGUUUUGAGGUGUCAAAUACAAAUAAAUCUGGGCUUAGGGAAGGAGAGACC

>TP1987

GCAUGCUGUCUUGCCCAAGUAGGGUACAUGAAGAUUCCCCAUGAGGAUACU

>TP1988

UUGGGCACGGUGGCUCACGCCUGUAAUCCAAAGCCAAGGUGGGUGGAUCAU

>TP1989

GGGGUGUCUGUGCUAUGGCAGCCCUAGCACACAGAUACGCCCAGAGAAAGC

>TP1990

CUGUAAUCAAGAAGAUAGAUAGGCCAGGCAUGGUGGCUCAUACGUGUAAUC

>TP1991

UCACAGUGGUGGGAAAAGCUGUGUGAACAUGUGGGUACACAGGCACCCAUG

>TP1992

GAAGCAGGACUGGGUAAGGAGGGAAAUCAAUUUGCAACACAGGAUGGCAGC

>TP1993

CUUGACCAGACACCCUUGGAUUCCAAUCCCAUGUUCCUGCAGACCUGUGCU

>TP1994

CUGCAACUCCUGCCUCCUGGGUUCAAGCGAUCCUCAGCCUCCUGAAUAUCU

>TP1995

UUUCCCUGAGUUGUUGGGGGCAAGAAGUGGUGCCAAGCCGGGAAGAAGGCC

>TP1996

CGACCUGGAGGACAAGAACGAGUGGAAGAACUGCAUUGACAUCACGGGAGU

>TP1997

GUAUAGACCAAGAGGUAUGGAGGCCAAGGCGAGCAGAUUACUUGAGGUCAG

>TP1998

UCACAACUCUGUAAAAUAUGGUUUUAUUCCCAUUUCAUAAAUGAGGAAAUU

>TP1999

UCUGUCACCCAGGCUGGAGCUCACUAGCACAAUUAUAGCUUACUGAAACCU

>TP2000

ACAGCCACAUCCUCUAAAUGGGCGCAAUUUGUCCUGCCACCUAGAAAAAGU

>TP2001

AUCCACCUGAGCCCAGGGAGGUUGAAGCUGCAACUUAUCUCCAUUGUCAUG

>TP2002

UCAGUGCUUACCCUUUAAUGUGUGUAUGUGAGUGUGUUUAUAUAUAAAUUA

>TP2003

CGUUUGGUUUGGUUUGUUUUGCCUGAGACAAGGUCUCACUCUGUCAUCCAG

>TP2004

CAUUAGAAUCACCUUGGGAGCUUUCAAAACAUACCCGUGCCUGCACUGAAC

>TP2005

UGGGGGUGGAGUGUGGGCACCAGUAAGUUUUAAAAGCUCCCCAGAUGCUUC

>TP2006

UCACACUCUCCCCUAGGUGGUUCUGAUUCCAUUUGUCCAGGGUUCAGUGCA

>TP2007

GUACCACACUUUCUUUAUCCAGUCCACUGUUGAUGGGCACCUGGGUUGAUU

>TP2008

UUAUUAUUUUUAUAGAUGAGGUCUCACUCUGUUGCCCCAGCUGGGUGCAGU

>TP2009

UGUUGCCCCAGCUGGGUGCAGUGGCAUGAUCAUACCUCACUAGGCUCAAGU

>TP2010

GCAUGAUCAUACCUCACUAGGCUCAAGUGAUCCUCCUGCCUCAGCAUCCCU

>TP2011

CCCACCUCAGCUUCCCCAAGUGCUGAGAUUACAGGCAUGAGCUGCCUGCAC

>TP2012

ACAGGCAUGAGCUGCCUGCACCUGGACUAAUACAGUAAUUUUACUGUACCU

>TP2013

UGCACUCCAGCCUGGAUGACAGAGCAAGACUCUUUUUAAAAAUAGCCAGGC

>TP2014

AGUUUGAGACCAGCCUGGGCAACAUAGUGAGAUCCCCCGGCCCCCCCAUCU

>TP2015

UACUCUCCUCUGUCUGGACCACAGAAGGGAAGGAAGGGCCUCUUUCUGCAG

>TP2016

ACUUGUGGCCUUGUUACUGUCUGUAACUCCCUGAGUUACAGAUGAAAGUUC

>TP2017

ACUCCAGCUUGAACUCAAGUGAUCCACCCACCUUGGCCUCUCAAAGCAUUG

>TP2018

AAGUUCUCACUUAACGUCAUCAAUAAGUUCUUGGAAACUGCUACUUUAAGC

>TP2019

AAAAUCUGUUGUACCACCUUAUGUAAAGCAGAAGAAUCUUGUAACAGUAUA

>TP2020

GUACCCCUAUGUUAACAUCAUCAAUAGCUAAAAGGUGGAAGCAACCCAAGU

>TP2021

UUGGAAAAGUACUUUGCAGUUUUCAAAGCACUUUGUUAUUCACAGUAACUG

>TP2022

AGCCACCAGGCACCAGGUGUGAUCUACCACACCUGUCUAAAAAGUAUGUAU

>TP2023

CGCUUGAGCACGGGAGGUCGGGGCUACAGUGAGGGGUGGUCGUGACAUUGC

>TP2024

GGUAUUUAAAGAUCAAAAUCUGGGAAGCUGAAACAGGAGGAUCACUUGAGC

>TP2025

UGUGGUGACAUGCUCCCAGCUACUCAAGAGGCGGAGGUGGGAAGGAUGGCU

>TP2026

AAUCUGAGCUACUAAGGAGACUGAGACAGGAGAAUUGCUUGAACCCUGGAG

>TP2027

CAAUGAAGCGACAUUCCGUCUCAAAAAGAAAAAAAAGAAAAAAAAUGUUUC

>TP2028

CUAAAGUAAGAUUUGGUUUCAAAAAAGUCACCAAGAACCAUGGAAAAUAAA

>TP2029

AGACAAGGCCUUGUUCUGUCAUCAUAGCUCACCGUGGCCUCAGCCUCCCAG

>TP2030

AAUUUUUAUGUUUUUAAUUUUUUGUAGGGACAAGAUUUCAAUACAUUGCCC

>TP2031

CCCUUAAGUGGGUGAUUUGCACCAAAGGCUGACACAGUGAAGGUGGCUCCA

>TP2032

CACUUCAGCAUCCUGAGUGGCUGGAACCAUUGGUGUGCGCCACCAUUCCCA

>TP2033

CUCGCUCUGUCACCUAAGCUGCAGUAGAGUGGCACAACCUCGGUUUACUGC

>TP2034

UAGUUUAAACACUGUUUGUCCUAUUAGAUUGAAUUUUGAAUGUAAAGAAUC

>TP2035

CUAGCCGCGCUUGGUUGUGCCGUCUAUAGUCCAUCUCUGUGGAGGCUGACA

>TP2036

GCCUACUUCAGCCUCCCAGAGCCCCAGGAUUAUAGGCAUGGGCCACUGCGC

>TP2037

UUGGAAUAUUUGCAUUAUACUUACUAGUCUACCAUCUCUAAUCUGAAAAUC

>TP2038

UUGAGCACCAUAUUGGUGCUUAAAAAGUUUUGGAUUUUGGAGCAUUUCAGA

>TP2039

CUGACAGCUGACAGUUUUAACUGACAACUUUGAUAACAGAGGCUGCUAUUU

>TP2040

UGCAGUGGCUCAUACCCAUAAUCCCAGUGCUUUGGGAAGCUGAGGUGGGCU

>TP2041

ACAGGGCAUGGUGGUGCACGCCUGUAGUCUUAGUUACUCGGGAGAGCGAGA

>TP2042

GGCGAGUGGUUUCGGAAAAAAAAAAAGAAAAAAAGAAAAAAAAAGAAAAAA

>TP2043

AAAAAAAAGAAAAAAAGAAAAAAAAAGAAAAAAAAAAGAUUUUUUUCUUCU

>TP2044

UAAUUCUGGUAUAAGGUUUGUCAUAACCAAAUGGAAAUGUAGGAAACAUUU

>TP2045

CUGCUAGAAUGGCGUGAACCCGGGAAGCGGAGCUUGCCGUGAGCCGAGAUU

>TP2046

CAGAGCCACAUUCUUUUAUGGAAAUAUUUAGGUUUGUGCAAAAGUAAUUGC

>TP2047

AUUAAUUUUAUUAGCCCCGUGUGCUAUGCCUGUAGUUCCAGCUACUGGGGA

>TP2048

UGAGUUGCACAGCUAUGAAGGCUGUACACUGCACGAAUGGAAGAGGCACCU

>TP2049

CGUGAUCUAAACACUUCGUUGUGAUAACUUUAAAAAUGUAAAUUGAAGGCA

>TP2050

CUUCCUUCAAUCAAGGUUUCCAGGCAGAGCAAAUACCCUAGAGAUUCUCUG

>TP2051

UUUCCAGGCAGAGCAAAUACCCUAGAGAUUCUCUGUGAUAUAGGAAAUUUG

>TP2052

AAUUUGGAUGAAGGGAGCUAGAAGAAAUACAGGGAUUUUUUUUUUUUUUUA

>TP2053

CUGAUAUGGGUAUAGAUAUCUCUAUAGAUAUAAUAAUAUGAAUACAGGUAU

>TP2054

CACACAGCAGUUUUGUUUAGGUAUAAGGAAGAUGACUUAGGGCUAGAAAAU

>TP2055

AGACAUCUUCUGGCUCAGCAGAGAGAGAAACUGAGGCCCAGGGAGGGAAGG

>TP2056

CUCUGUCUCAAAAAAAAAAAAAAAAAAAAAAAGAGGAACUAAAGCCUCUGA

>TP2057

CUUAGUAAAGGCAGCUUAACUGCCAAAACAGCAGGAAUUAGGGCUUUCUGU

>TP2058

GGGGCGGAGGUCGUGCGGUGAGGCAAGAACAUGCCAUUGCAUUCCAGCCUG

>TP2059

GCAUGCGCCACCACCGCUAAUUUUUAGUAGAGCCAGGGUUUCGCCAUGUUG

>TP2060

UCCUGGCCUCAAGCAAUCCCACCUCAACCUCCCAUGUAGCUGGGGUCACAG

>TP2061

AGUGAGCUAUGUUCAUGCCAUUGCAAUCCUGCCUGGAUAAGCAGAGCAAGA

>TP2062

GAGGAGGAGGAGGAGUCUUGCUUUUAUCGCCCAGGCUGGAGUGCAAUGGCA

>TP2063

UGCUUUUUUGUUUUUGUGGUUUUUGAGACAAAGUCUCCUUCUGUUGCCCAG

>TP2064

AGCUGGAACGGGAGUACUGCGACGCAGCCCGGAGUCGGCCUUGUAGGGGCG

>TP2065

AAGAAAGCAAAAGCAGACUGGGCACAGUGGCUCACAUUUGUCAUCACAGCA

>TP2066

UUUAAAUUACUUGUUUAUUGAGACAAGGUCUCGCUCUGUUGCCCAGGCUGA

>TP2067

UGUUGCCCAGGCUGAAGUGCAGCUCAGUGCAGCCUCGACCUCCCAGGCUCA

>TP2068

GGAAAAUGAGAUUAUUGGGAAUAGUACAUGUAUUUCUGGAAUAGUAGGAAG

>TP2069

UUGCUCUGUCAUGGGUUGGAGUGCAAUGGCAUGAUAAUGGGUUACUACAGC

>TP2070

AGCCUCCUGAGCAGCUGGGACAACAAGUGUACACCAUCACACACCAGCUAA

>TP2071

UAGUUGGCCUCGGCAAAAAGGCAGCAAGAAUCGACGAACAGGAAAACUGGC

>TP2072

AGGCCUGGCUGGCUGCGAGCUCAGGAGGCCGCCUGAGGACUGCACACCGGG

>TP2073

GGCUGGUCUCAAACUCCCAGCCUCAAGCGAUGCUCCCACCUCCACCUCCCA

>TP2074

ACUUGUCUCUGGGAGGAGGGACAGAAGGGACUUGAUCGGGAGGACAGAGGC

>TP2075

GCUAAGGGAGAUUUGGGACGGCCCCAGUGGUGGGAUCCCAGUCCAUUCCAG

>TP2076

GGUGGGAUCCCAGUCCAUUCCAGAAACUGCUUCUGAAUCUUCUCUCUGACU

>TP2077

UUGGACCGUUGCCCUGGUCCUUGGCAGAGAGACACUCCCCUUUCUGGGCCU

>TP2078

AGACACUCCCCUUUCUGGGCCUGAAAGACCCACCCCCUGGCUUUAGGCUGU

>TP2079

CUGUUCUGCAGCCUUUUCGGGAGGAAGCAGAGGAUGGGCCUCUGUCCUCCC

>TP2080

CAGAGGAUGGGCCUCUGUCCUCCCAAUCAAGUCCCUUCUGUCCCUCCUCCC

>TP2081

GCUGUAGAGAUGGAGGUCUCACUAUAUUGUCCCGGCUGGUCUCAAAUUCUU

>TP2082

UAAUUCUUUUGUGAAUGAAUGAGACAGGGUCUUGUUCUAUUGCUCAGGCUA

>TP2083

AAGGAAAGUGUAGAUAUGGGAAUGUAUGUAUGUGAAUGAGUCAGUGAAUUU

>TP2084

AAUCCCAGCAAUCCAGCACUUGGAAAGGUCAAGGCGGGAGGAAUGCUUGAA

>TP2085

CGCUACUGGGGAGGCUGAAGCAGGAAGACCUCUUGAGCCCAGAAGGUUGAG

>TP2086

UAAUUUCCUUUUGCGACGGAAUCUCACUCUAUUGCCUGGGCUGGCUGGAGU

>TP2087

GUCCUAUAAGUGUUGCGCUGACUCUAGGACUGAAAAGUCCCAAACUCAGUG

>TP2088

GGUGCCACUGCAUGCCAACCUGGGCAACAGAGGUAGACUCUCUCUCAAAAA

>TP2089

AUUUGAUUCCUUUGAAACUCAAGAGACCAGGGAAAGUGACAGGAAGGAAAG

>TP2090

CAACCUCCACUCCCCGGGUUCAAGCAAUUCUCAGAGUAGCUGGGAUUACAG

>TP2091

UGAAAUGCAUUUGUUGUUAUCUGGUAGAGUAUGCUUUACUACAGAAUCCAA

>TP2092

UUGGCUUUUUUUCAGAUGUUCACCAAGCUUAAGUUUAAAAUAAUAGGUAUU

>TP2093

UCGGCCGCCAUGGUCUUCAGCGCCCAGCGCGCUGCCUAUGGCACGGUCAAG

>TP2094

GUGUGCACUUAUAUUCCCAGCUACUAGGAAGGCUGAGGCUAGAGGAUUCCU

>TP2095

CAACCAGGUUACAAAACUAAAAAAAAGAGUCCAGGACAUUGUCUGUCUGCC

>TP2096

UUAAAAAAUGUGAUCCCUGAGGUCAAGUGAUUGAGACCAUCCUGGCCAACA

>TP2097

GAGGUGGCUUAUGCCUGUAAUCCUAACACUUUGUGUGGCGGAGGUGGCGGA

>TP2098

AAGAAUACCCACGUGACAGCUGGUUAUGGUGGCACAUACCUGUAGGCCCAG

>TP2099

UGGGCUCAAGCAAUUCUCGUGCCUCAGCCAACCAAGUAGCUAGGAUUACAA

>TP2100

CAGAAAAAAAAAAGAACAAGGAGUGAGGUCUUGUGUUACCCAGGCUAGAGU

>TP2101

GAUUUUUCCCUUCAUAGUGGCAGAUAGUGUUAACCCCUGCACCAUCUGUAA

>TP2102

CAAUUGGCAAGGAUGUGCCAGGCCUAGGUUAAACUUGGAAACAUGCUUGAC

>TP2103

AUGCUAGGAGUUCGAGACCAGCCUGACCAACAUGACAAAACCCCAACUCUA

>TP2104

CCUUCUUUUUGUGUUUCCCCCCCGAAACAGAGUCUCGCUCUGUCACCCAGG

>TP2105

CCCCGAAACAGAGUCUCGCUCUGUCACCCAGGCUAGAGUACAGUGGUGCAA

>TP2106

UUUCUUUCGUAUUUUUAGUAGAGAUAGAGUUUUGCCAUGUUGGCUAGUUUG

>TP2107

UGAGUACAACUUUGUUAAAAGAAAUAGCAAAGUGGCCUGGGCACAGUGGCU

>TP2108

ACUCAGGAGGUUGAGGUGGGAGGAUAGCUUGAGUCCAGGAGAUUGAGGCUG

>TP2109

CUUGAAUCGCUUGAACCCAGGAGGCAGAGGUUGUGGUGCGCCAAGAUUGCA

>TP2110

ACUAGAGAUGGAGUUUCACUGUGUUACACAGGCUGGUCUUGAACUCCUGGC

>TP2111

UUUUUUUGGUGUCACCCAGGCUGGAAUGCAGUUGUGAGAUCUUGGCACACU

>TP2112

GCGGAGGUUGCAUUGAGCUGAGAUCAUGCUAGUGCGCUCCAGCCUGGGCAA

>TP2113

GUCGUGGUGGAGCUUUUGGCUUAAGAAUUCUUUGUCCGGAUUUAAUUGCUC

>TP2114

GGGCUGGGUUUUCAGAAAAUAAUAUAUUCUGUAUUUGCAUAGCAUACAAAG

>TP2115

GGGUAAGAUUCUGUCUCAAAAAAAAAAAAAAAAAAAAAAACCAAGAAAUAA

>TP2116

UGUGUGUGUGUGUGUGUGUGUAUAUAUAUAUAUAUAUUUUUACUUUUAUCC

>TP2117

GCCCUGAGUUGGAAAAAAAAAAAAAAAAGAGAGAGAGAGAGAGAAGCUGGC

>TP2118

AUAGACACUGGGGACUCCAAAAGGAAGAAGUAAGGGAGAGGGACAAGGGUU

>TP2119

GUGCCUGUAGUCCCAGCCACUCGGGAGGCUUAAAUGGGAGAAUCACUUGAA

>TP2120

UCCUGCCUCAGCCUUCUGAGUAGCUAGGCCUGUAGUCUCAGAGGGAACUGU

>TP2121

UGUAUUUUGUAUCUAGAAUGCAAAAAGAACUUCCAAAACACAAUAUUAAAA

>TP2122

GGGGCUGGGAGUGUAGACUUCAGUUACACCCCCAGCCCCCAGGGGAAAGCA

>TP2123

UCAUCAGUCAUAGAGCCAACACUUGAAGGUUCAACAAUAACAUCAUAAAUU

>TP2124

CCAGUGGUGAGGAAGUAAGACACCAACACCAGAGCAUACACAGUCAUGGUC

>TP2125

AAAUCAGAAUUAACAAGAGUUUUUAAAAACAAUUUUAGAAGGAACUAAGGU

>TP2126

AGGAACUAAGGUUGUAAGUUUGGCCAGAUGUGAAAUAGCUGAGAUUCUUUU

>TP2127

CUCUCAACUAGGUGUGGUGGCUCACACUUGUAAUUCCUACAUUUUGGGAGG

>TP2128

CAGGAAUUUGAAACUAACCUGGGCAACAUGGCAAAACCCCAUAACUACCAA

>TP2129

GCCUGUAGUCCUAGUGACUCCAGAGACUGAGGUGGGAGGAUUGCUUGAGCC

>TP2130

UGGUAACCAAAGCAUUAGUCACAAUAACAAAGACAAGCAGUCAACCUAGGU

>TP2131

ACUACAUAGCCAUAAAAAGAAUGAAAUCAUGUCCCUUGUAGCAAUGUGGAU

>TP2132

CCCUUGUAGCAAUGUGGAUGCAGCUAGAGGCCACUAUCCUAAGUAAAUUAA

>TP2133

UAGAGGCCACUAUCCUAAGUAAAUUAACACAGGAAAAGAAAACCAAAUACU

>TP2134

CUCAUUUUCUUUCCAAUCUGACAUUACUCAUAUGCCCAUUCUGUAACCAUA

>TP2135

CCACUACUGCUUUGCGCCUGCGCGGAGGCUCGGGGAGUCGGCGCCAUGACC

>TP2136

UGCUGUAAAUUAGGAAAGGGAGACCAGCCUGACCAAUAUGGAGAAAUCUCG

>TP2137

UUUGUAUGUUGAUCUUUUAUGUACAAGGCUCAUGCCAUGGAGUGUUGUACA

>TP2138

UGUUACAUAGGUAAACGUGUGCCACAGUGGUUUGCUGCACAGAUCAACCCU

>TP2139

UACCUGGGUAACAAAAUAAAUCUGUACAACAAACCCCUGUGACAAGAGUUU

>TP2140

CAACAAACCCCUGUGACAAGAGUUUACCUAAAUAACAAACCUGCACAUGUA

>TP2141

CUCCUAAGGUGCUGCUGUCAACACUAUUGCAUUGGAGAUUAAGUUUUCAAC

>TP2142

GUGCGCAUGGCUAGCCCCAGGGGCCAGAGUAUUAAGAGCCGACAAGCCUCA

>TP2143

CGUCGGGGUUUCACCAUGUUGUCUAAGCUGGUCUCGAACUGCUGACCUUGU

>TP2144

CUUAGGUGUUUUGUUAUAUAGAGACAGGGUCUCAGUGUUGCCCAGGCUAGU

>TP2145

AAAUUUUUGUAUUUUUAGUAGGGAAAGGGUUUCAUCGUGUUGGCCAGGCUG

>TP2146

AGGUAAAUUGGACCUUCUUAGUGAUACCAACAUGGUAGACUUUGCUAUGGA

>TP2147

AACAUGGUAGACUUUGCUAUGGAUGAAUACAAAAACCUUUAUUCUGAUGAU

>TP2148

AUAUUCCUCAUGCUUUGAAAGAGAAAAGAACCACAGUUGUUGCACAACUGA

>TP2149

UGAAAAUGUUUGAAGAUCCAGAAACAACAAGGCAAAUGCGGUCAACCAGGG

>TP2150

UCUACACAUAUGCAAAAUUCCAGUAAGAAUGUGGGAAUUACUCAGGAGCAG

>TP2151

AGAAUAUCUUUAUUUUUUCAGAGUGAUGGUUCCAGCAACAGACAGAAAUGC

>TP2152

UCAGAGUGUUGGUUCCAGCAACAGAAAGAAAUGCUGUAAGUUCACUCUGGG

>TP2153

GGAAAGCUGGCCUCUGAAAUCUUAAAGCAGAAUUGGGAUGCAGACAUGGAA

>TP2154

UUAUAAAUGAUAAACACAGGUGUUUACCCAGACAGAAGUUAAGCAAAGUGU

>TP2155

UUUGACUUUUUAUUAAGUAUAGAUAAAAAGACUUAUAAGAACCUUAUAAGA

>TP2156

AUAAGAAAUUCUUGUAAUUCUUAUAAGGUUCUUAGAAGUCUUUUUAUCAUC

>TP2157

UGGCCCUAAAGAGUCCUACUGCGUCAGCCUCCCAAUGUGUUGGGCUUACAG

>TP2158

AUUGGACACGGUGAUGCUUACCUGUAGUCCUAGCUACUCAGGCGGCUGAGG

>TP2159

CUUCUUGCUUCUGCUCUCACCAUGUAACACCACCCACUCUCCCUUUGCGUU

>TP2160

CCCUUUGCGUUGCACUAUGAUUGUAAGCUUCAUGAGGCCCUUUCCAGAAGC

>TP2161

UUCAUGAGGCCCUUUCCAGAAGCAGAUGCUGCAGCCCUGCUUAUACAGCCU

>TP2162

CUUUUCUUUCUUUUUUUUUUGAAACAGGGUCUCACUGUGUCACCUGGGCUA

>TP2163

CAGUGCAGUGGCGCAAUUUCAGUUCACUGCAACUUUGACUUCCUGGGCUCA

>TP2164

UUUUUUUUAAUUUAAAAAUUGAAACAGAGUCUUGCUGUAUUGCCCAAGCUG

>TP2165

GGUGAAAAAACACAAAAGAUUAGCUAGGUGUGGUGGUGGGUGCUUGUGGUC

>TP2166

UGGGCUCAGGUGAUUCACCUGUCUUAGCCUCUCAAAUGGUUGGGAUUACAG

>TP2167

UGGCUCGAAUAAAAAUACACAAAUUAGCCCAGCAUGGUGUGUGCCUGUAGU

>TP2168

GAGUUGCCUUCAUGCUUUUUCUAUUAGGUUGGUGCGAAAAUAAUUGCGGUU

>TP2169

UUUAAUUCUCAAAGCCCAACUCACAAAAGCAUGAGGAACAAAUAUAAACAA

>TP2170

UCCACAGUAACAUAUGUUGUUUUUUAACUUUGCAAUAAUAGCCACCCUGAC

>TP2171

AGAUGUAAGCGGUUUUCCCACCUCAACCUCCCCAGUAGCUGGAACUACAGG

>TP2172

AGAAUAUAAGAAUUCUAUCUAAAAGAAGUGAAUGAAAGGUAUAUUCUGGUA

>TP2173

AUCUUACCUUUAAAGAUUCUUGGCCAGGUGCUGUGGCUCAUGCCUAUAAUC

>TP2174

GCAAUUUAAUCCCCAAAGAAGUCCUAGACAUGGAUGGUGGUGGUGCUUGUG

>TP2175

GUGGAUCUUUAUAGAAGGAAGCCCAACCCUAGCAAUUACUGAGUUACCUCA

>TP2176

CAUUUUUUUCUUGAAUGUCUUCAUAACUUUGUCUUCAUUGAAUCAUAAGCC

>TP2177

UUGGGGCACUUAGAAAAGUUGGUGGAGUCUUCCACUUUUUAAAAUCUAAAC

>TP2178

UACAAAUGAAAGAGCUGAUGAAAAAAUUUAAAGAAAUACAGACACAGGUAG

>TP2179

UGCUUUUCGUGUUUACAUUUCCCCAAUGAUCUCAAAGGUAUCUACCAACAG

>TP2180

GGGAAACUGCCUACAAAAAAAUACAAAAAUUAGCUGGGUGUGGUGGCCCAG

>TP2181

AUACAAGUUGGCUCUUUCCUUGUGUACAUGCAGCCUAGGAUAGUGGAAUGA

>TP2182

GACAAGGACUGUGUGUUUUAUACCAAGGCCUACCCAGGAACUUGGCAUAUG

>TP2183

UAACUUUUGGGGGAGGGCUUUAACUAAAAUAACUUCAUGUAGUGUUUGAGC

>TP2184

AUUGCAGGCCUGUGAGAUUGCUUAUAUUCUUUCUGUAAUUGGAUGUCUGUG

>TP2185

AAAUUUGACAUUCUGAGGUUAAAAGAACACUCUCACCAAUGAAUUCCCUAG

>TP2186

AGCUGAAUGAAACUCUAAACCACUUAAAACACACUGGGCUCAGUAGCAUAC

>TP2187

UCUUCGUGGUCUUUUCCAUUAUGGUAAGCCAUAGAGGCUUUCCCGGGCUCA

>TP2188

UCUAUGUGAGAAGAUCUGAGGGGUAAGCAGGUUUUAAUGGACUAAGAUUUU

>TP2189

GACUAAGAUUUUUUUUAUAUGUAUAAGGAGGGUGGGAGGAGGAUUUUAGAA

>TP2190

CCUGCAGUUAGAAGUCGAACACUGAACUUGGGAAAGCUUCUGUGGCUGACC

>TP2191

AAUGGUGGUGGGAGCCCAAGUGCUAACUAUCUGGGAGACUGAGGGUGGAGG

>TP2192

AGGGUUUCUCUUUCCUGAGUCUGAAAUACCUGGGAAACCAGUUAGAAGGUG

>TP2193

UAUUAGGGGUGGGGGAUGGUGUCUCACUCUGUCACUCAGGCUGGAGUGCAA

>TP2194

GUAAUGUCUGUACAGGCUGUACUGAAGCGGCCUGUACAGGGCCGCUUCAGU

>TP2195

AAGUUCUAAAAACUGAGCUAAGAGCAGGACAUGAUGGCUCACACCUGUAAU

>TP2196

CACCUGUAAUCCCAGCUACUCAGGAAGCUGGGGUGGAAGUAUCUCCUGAGG

>TP2197

UUUAUUUUUUAAGUGGCCUGAUCGCAGCUCACUGCAGCCCUGACCUCCCAG

>TP2198

CUCCCAAACUGCUGGGAUUACAGGUAUGAGGUACUGUGUCCAGCCAGAAAA

>TP2199

UUUCUUUUCUGAUGGUGAUUGAUAUAGUUUGGCUGUGUCCCCACCCAAAUC

>TP2200

AUUACCUAAUCUCAGGUCUGUCUUUAUCAACAGCAUGAAAACAGACUAAUA

>TP2201

UUUGCUCACUGCCAGAGAACAGCACAGGAAAGACCUGCCCCCAUGAUUCAG

>TP2202

AUUAUUCUUUUUGAGACAGAGUGAGACUCUGUCACCCAGGCUGGGGUGCAG

>TP2203

AUCAGAAGCUCUGAGAGUAAGGCCUACCAAUCUGGUUUAACAAGCCCGUCA

>TP2204

CAGAGAUAAAAAAGGAGACAUUACUACUAAUACCAGAAAAACUCAAAGGAU

>TP2205

UUAGUAGCUUCUAAUGAUCCUUUGAAUUUUUGUGAUAUUAGUUGUAAUGUG

>TP2206

UCUUAUUGCUCUGCAGGGGCUGUGAAAUGGCUUGGAUUUGUGUCCCCACCC

>TP2207

UGGUUAAAGUAGUGGUUAACAAGGAAACAAUAAGCAACAUGAAACAGCAGC

>TP2208

ACUCAUACAACUCAAGAGUGAAGAAACAAAUCACCUGAUUAUAAAAUGGGC

>TP2209

AAAGUGGUGCCUGGCACAUACAUUUAUAUUUUAUGUGCCACAUAGGUGGCA

>TP2210

UGUGCCACAUAGGUGGCAUUGAACAAAUGUCAUCUAUUAGUAUUAUUAGGU

>TP2211

UAUAUUUAUUUGGUAAUGAAGAUUUAUUUCUAAGUGAAGGAUAGAGUCAGU

>TP2212

UCACAAGGCUGCAGGAGAGAGAAUGAGUGUAAGCAGGGUAAAUGCCAGAUG

>TP2213

UGAGAACUCACUUACUAACAUGAGAACAGCAUGGGGAAACUGCCCCUAUGA

>TP2214

CCCUAUGAUCCAAUCAUUUCCUGCCAGGUCCCUUCCAUGACAUGUGGGGAU

>TP2215

AUUGCAGUAGAGAAAGAGUUUAAUUAAUGCAGGGUUAGCCAAGUAGAAGAC

>TP2216

AAUCCUUAUCUUGUGGCCUUUCAUUAGUUUUACACAGACAGUUUCAGUCCU

>TP2217

UAUAUACUACAUUCCUCCCAUGGUUAGCUUGGCUUACACUCAGGAAUGAAC

>TP2218

UGCUUGGGGACUAGAUUGCCUUUGUAGGACUAACAAAGUAGCCACAAGAUU

>TP2219

UCAGAUUGAUAAACUGGCUCAUCCAAUCUUGUGGACCCCACCCAGGAACUG

>TP2220

GACCCCACCCAGGAACUGAUACAGCACAAGUAGACAGCUUCGACUCCCUAU

>TP2221

UGAGGUGGGAGGACUGCUUGAGCCCAGGAGGUCAAGGAUGCUCUGCCACCA

>TP2222

GUGUUAUUUCCAAUUUUGUCAGUUAAGUGUGUAAAGUUGCCACACGAAGAU

>TP2223

CAGUUAUGUGUGUAAAGUUGCCACAAGAAGAUAUGGACCAUCACUUGGUGC

>TP2224

AGGGAUAUGGAUUUGGCUUAAUAAAACCUGAUUUGAAAACAAAAUCUGAGA

>TP2225

ACAAAUUUGACUAUAUUAAACUAUUAAUAAAAAGUUUCUGCUCAGCAAAGG

>TP2226

GCAAAGGAAGCAGUCAACAGAGUGAAGAGACAAUCUGUGGAAUGGGAGAAA

>TP2227

GCUUCAAUACUUGGAAUUGCAAUAGAGUUGUAAGUACUGAUUCUUUUACAA

>TP2228

UUUUUUCUUGGUUUAGUAGGAGAAUAUCUACUCAAAGAAAUUUACUUUCAC

>TP2229

GUGUAGUGGCACAAUCUUGGCUCACAGUGACUUUCGCCUCCCAGGUUCAAG

>TP2230

GGUCAAAGGCAAAUUAUGGUUCAAAAGUGUCUGGCAGGCCAGGCACAGCGG

>TP2231

AGUGAUAUAGAAGAUAUAGUGAUAUAGUUUGGCUCUGUGUCCCCACCCAAA

>TP2232

CAGCAUUGUUUGUAAUAGAAAAUAAAAGCAAAAUAGGCUGGGCACGGUGGC

>TP2233

CAUGUGGUGUUGAGGAAAGCAGACAAUGACCUCACCAAGAGGGCGGGAGAA

>TP2234

AGUUUUGAGUCAUGCUGUGUCACCCAGGCUAGAAUGCAAUGGUGCAAUCUC

>TP2235

ACCCAGGCUAGAAUGCAAUGGUGCAAUCUCGGCUCACUCACUGCACCUCUG

>TP2236

AGGCGAGUUUAAUGAGGAGGAUGAAAGUGCUAUUGAAAUGUACAGACAGCA

>TP2237

UUAUGUCAAAAUUAAGCGUAACUGGAGGAAACCCAGAGGCAUUGACAACAG

>TP2238

GUCCCAUGCAAUAUUAUACUAAAAUAUUAUUUGUUGUUUAUCUGAAAUUCA

>TP2239

AAGCCUUUGCAAUGAAACUUUUUAAAAGAAAGUCAUGGCCGGGUGCGGUGG

>TP2240

UCUCAUUGUUCAGCUCCCACUUAUGAGCGAGAACAUGCAGUGUUUGGUUUU

>TP2241

UACUUCACCUUAAAAUAUUUAAAAUAGGCUGGGCUCGAUGGGUCAUGCCUG

>TP2242

UUGUUGUUGUUAUUGUUUAAGACUGAGUUUCUCUUGCCCAGGCUAGAGUGC

>TP2243

AGGCAUGUGCCACUAUGCACACCUAAUUUUUAAAUUUUUUGUAGGGAAGGG

>TP2244

CACUGCUUCCUGUAGCUUCCAAACCACCUCCUCAGAAGUGUCAUCUCCAGC

>TP2245

GCCACUGCACUCCACAGUGGGCAACAGAGCAAGAUUCUGUCUUAAAAAACA

>TP2246

CAAGGUUAGAGGAUCGUGUGAAGUCAAGAGUUUGAGACCAGUCUGGACAAC

>TP2247

UGAGGCGGGAGGAUCUUAUAAACCCAAGAGGCAGAGACUACAGUGAGCCGU

>TP2248

UCAGAAUCACCAUCCGCGGCGCGGGAGACGAGCCGGCCGUCCCGGGCCGGG

>TP2249

AUAAGGGGUUCUUCCACCUUCACUCAGUACUUCUCCUUCCUGCCACCUUGU

>TP2250

CUUUAUUUUAUUUUAUUUUUUUUGAAACAAGAUCUCGCUCUGUUGCUCAGG

>TP2251

UCACUCUUUGGGUAAUGUGUACAAUAGAAGCCCAAUCCCCACCUAUACCCA

>TP2252

CCUUUCACGUGCAACAGGGUUCUUCAUAGUGGCCUAGGGAAUUUGAGUGUG

>TP2253

CUUCAUCAGACUUAGCAGCGUAAAAAUACACAGGUGUCGGCCAGACACAGU

>TP2254

GACAGGAGGAACCCGGGGCAGGGUAACACUGAGGGCCGUAAGGUCACAUAG

>TP2255

GAACCUAGGAGGCAGAGAUUGCAGUAAGCCGGGAUCGUACUGUUAUACGCU

>TP2256

GUUUAAGAAAUUUGUGUGCAUAGUUACUCAGUUUUUAUGAACUGUUGUAUC

>TP2257

UAGCUUCCCCUCCUCAGGGUCACUUAGAGAAGGAAAUACUUCUGCCUCUUU

>TP2258

AUAGAAAUAAGUGGACAUACAGGCUAGACAUGGUGGCUCACACCUGUAAUU

>TP2259

AGUUUUCAGGUGUCUGUUUCUUUUUAGAGACAAGGUCUCACUCUGUCACCU

>TP2260

AAAGUAAGGAAAGCAGAAGGCUGUCAGGAAGUCCGGGCCAAAGAUGACUGG

>TP2261

CUGUUCUGCCCCAGGAUUGUGCUGGAAGUGCUGGUUGUGCUCCGAAGCAUC

>TP2262

AGUGCUGGUUGUGCUCCGAAGCAUCAGCGAACAGUGCCGCCGUGUGUCCAG

>TP2263

GCAGAAAUAUGUUCUUCAUCCCCUCAGGGCUAGCUGUGUUUACACGAUUUG

>TP2264

UGCUGACCAUUUGUGUACUGUCUUUAGAGAAAUGUCUAUUCGUGUCCUUUG

>TP2265

GGAAACAUUUUUAAUUCAACAUUUUAAAUUCAAAAUUAAAAAUGUCGCUCU

>TP2266

GUCGUCUGCAAGGCCUGCAUGAAGGAGAACAGACGCAUCACUGGCCGAGCC

>TP2267

AUAUUUCAGUUUCCAAUUAUAAGACAAUAAGAUGGAUAAUUCUGUAUUUAG

>TP2268

AGCCCGAAUAUUACUGUUUAUGUUAAGCAAUGCAUGGAAUCUAUUCUCUGA

>TP2269

AGAAAUCAGAAAAACAGUGCAGGAUAUGAAUGAAAAAUUUGCCAAAGAGAU

>TP2270

UACAUUACUUGUGGUAAUGUACUACAGGUGUACAUUACCAUGCCUGGCUAA

>TP2271

AAGUGUGGAGGGAGAGGAGAGGUGUAGAGGGAGAGGCGCAGGUGGGAACCG

>TP2272

AAACUACACCUGUUACAUACCAUUCAGUUAAAUGAUUUAAGGGGGGAAAUG

>TP2273

UCCAAAAAUGCUGGGAUUCCAGGUGAGAGCCAUCACGCCUGGCUAUUUUUA

>TP2274

AAUCUGCCCAUCUUAGCCUCCAAAAAUGCUGGGAUUCCAGGUGAGAGCCAU

>TP2275

UUCUAUGGAUCUAACAUUGAUCAAUAACGAAUUCCUAAGCAUCUAUAUUAG

>TP2276

GUGUCAUUUACAAAACAAACAAACAAAAUUAUUGCCAAGACUAAUGUCAUG

>TP2277

GUACCCUGGAAACUUAUCCGUGACCACAUUAAUUGUUCUCAUCCUAAGGUC

>TP2278

AGUGCAGCUUGGUUUUAUAGAUUUUAGGGAGGCAUGAGACAUCAAUCAAAU

>TP2279

AUGUACAGACAUAGGUUCUUUCAUAAACUGUUGGUGGAAAUGUAAUUUGGU

>TP2280

AAAGAACUCCAACCCAGUGAACCCAAGUCAGCAUGGCAUGGAGUGGUGAGA

>TP2281

UAGGUUGAAAUGAUCCUCCCACCUCAGCCUCCUGAGUUAGUUGGGACUAUA

>TP2282

UUGGCAUAGUUUGGUGACAUUCUCUAGUUAGUCUUGGCCAGAUGGGGUAAA

>TP2283

GGCCAGCACUUUGGGAGGCUGAGGCAGGAGGAUUGUUUUGAGACCAGGCUG

>TP2284

UUUAUGCUGAAGAGGUGGCCACUAGAGGUCUGUGUGCCAUUGCCCAGGCAG

>TP2285

GUUUCUACUGGCAUUCUGAUUGGAUAUUUAAAGAAGGGUGCCACAUUUUUC

>TP2286

GAUGUUCACCUCUCUCCUGAAAACUAUUCCCACCAGACCGUUUAGCCUCUG

>TP2287

AUAAAUCUGAGAGCCACCAUCACCUAGAUGGUGUGUCAAGUCCAGGAAGAC

>TP2288

GGAUGUCAAUGUGCAGUUGUCAAAUAGACAGUUGGAUCCAAAGUCUGUAGC

>TP2289

CAGCAUUAACAUCACCUGAGAACUUAAUAGAAAUGCAGAAUCUUAGGCCCU

>TP2290

AACUGGCCAGGUCCUUGAUCAUGGAACUAUAGAGCUACCAGGACAUAUCCU

>TP2291

UUCCCUGAUGUAUUAGUCAGUUCUCACACUGCUAUAAAGAAAUACCUGAGA

>TP2292

AGCAGAAGCCUCUGAGCUUUCUGUAAAGCCUGCAGAACCGUGAGCCAAUUU

>TP2293

UCCAGUGCUGAAGGUGGGGCCUGGUAGGAGGUGUUUGGAUCAUGGUGGUGC

>TP2294

UUGUUUUGUUUUGUUGAUUAAAGGUAGAACAGCAAUGGUAUCUCCACAUAG

>TP2295

UUUAUCUUCCCCCACCCUAACAUGCAAUCUUCUAAGAAUUUUGCAAAAUUC

>TP2296

ACGAAAUGCUCUCGCUACUAAAAAUAGAAAAAUUAGCCAGGCGUGGUGGCA

>TP2297

CCUACAGCACUCAGCACCUUACUGUACUGAAUACUGUAGGCACUUGUAACA

>TP2298

AGGAUACAAACUUGGACAUAGGGUUACGAUUGCCUACAGCACUCAGCACCU

>TP2299

GCAAAGCAUGAUGGCUCAUCCAUGUAGUCCCAGCUACUCAGCAGACUGAGA

>TP2300

CAAUAGAGACUCAGGUUUGAUUACUAUCAUGACUCAGAGACUUUAUAAUUU

>TP2301

UCCAGAUAAAAUAUGUCAAAUUAUAAAGCCUCUGAGUCAUGACAGUAAUCA

>TP2302

AUAUUUGUCAUACUGGCCCAGAUCCAGAAAGUAGUAUGUUCAGUGUCUAAU

>TP2303

GUUGCCUGGGCUGGAGUGCAAUGGCACGAUCUCAGCUCACUGCAACCUUUU

>TP2304

AAGUGGCAAAUUGUUAUAAUGCAAAACUGCAAUUACGUUUGCACCAACCUA

>TP2305

UGCUGUGCUGGUGCCAGCCCCAGGCAGUGGGAUUCCUGGGGCACCUGCCGA

>TP2306

UGCUUGAGCCCAGGAGUUUGAGGUUACAGUGAACUGUGAUUGCCUCAAGCC

>TP2307

UCGAUACUAACUUGGGCAAUGUUGCAAGACCCCAUCUUUGCAACAACAGCA

>TP2308

AUGUGUUAAGUCCUAACUAAUUAAAAAGUUUGUCAUAAAAAAUUCUAACUA

>TP2309

UACUAGGAAAAUCCCAGCUACUGUAAUCUCAGCUACUCAGAAGGCUGAGGC

>TP2310

GUGAUCUUGGGUCACUGCAAACUCCAACUUCCAGGCCCAAAUGAUCCUCCC

>TP2311

CAGAAAAGUUACAAGGAUAUUAAUUAUAAUUAAUACAAUAUUAAUUAUAAU

>TP2312

UAGUCAUACAACAGACUACUACUCAAGAAUAAGAACUGCUGCAUACAACAG

>TP2313

GUAGGCACACAUGAGCACAAUGUAUACAUGUACACGUGCAUACAUGUGCAU

>TP2314

ACAAAUAUAAGGCAAAGAGGAACUGAAGGCCACGAGUACGGGGUGUGGCCA

>TP2315

AGAUAUGAAUCUUUUUGCCUCCACCAAUUUCAAGAACAAGGUAAGGAUUUU

>TP2316

AAAAUUAGGGUGGUAGUGCACACCUAUGGUCCCAGCUACUUGGGUGUCUUA

>TP2317

CAUAGUAAGACCUCAUCUCUACAAAAAAUUUUUUAAAAUUAGGGUGGUAGU

>TP2318

UUAUUUUUUGUAGAGACAGGGUCUCAAACUCCUGUAUGCAAGCAAUCCUCU

>TP2319

AGUAGAAAAAUGUUAACUGCCUAGAAGGUGUUACUUUCUCCAAGCUCCUCA

>TP2320

AUUAAAGCAUUUUCAGAAAGGGCCAACAUCAGCCUUGGAAAAAAGGAGGAA

>TP2321

GGGAGUGCAGGGGGCAGCAACAGGGAGGCUGUCUUUUCUGAGAUGGGGUCU

>TP2322

GGUCACCAGCUGGUAAGUGGAGCCAAGACUGGCACUGAAGCUGUCCUGACU

>TP2323

AACUGAGGCUCAGAGUACUUAAGUGACAUAUCCAGGGUCACCAGCUGGUAA

>TP2324

GCUGAGAUUUGAACCCAGGCAGUCUAGCUCCUAAGCAUGAUGCCGUGCUGC

>TP2325

ACAGAGAGGUCCAGUAACUGACCUAAGGUCACACAGCAGUAAGUGGCAGAG

>TP2326

UUUAUAGGUGAUGUAUGUUCAUCACAGAGAGGUCCAGUAACUGACCUAAGG

>TP2327

GCAGGUAACUGUGUGGCUUUGGGUAAGCCACUUCUCUUCUCUGAGCUUCAG

>TP2328

CCAUGCCCACCCAGGCCGUGGGGCCAGGGGCCUGCCAGGGCUAGGAGUGGG

>TP2329

UGCUUGAGCUCAAGAGUUUGAGACAAGGCUGAGCAAUGUAGUGGGACCUCG

>TP2330

AUUUAGGCCGGGUGCAGUGACUCACACCUGCAAUCUUAGCCCUUUGGGAAG

>TP2331

CUUGCCCUCCCGAAGUGUUGGGAUUACGUGCACGAGCCACCACAUCUGGCC

>TP2332

CCCCCAUCCAGCUGGGUGGUGGGUGAGGGUGUGAGACAGGCAGGGAGACCA

>TP2333

GCUUGCCUCAGCCUUCUAGAGUGCUAGGAUUACAGGCAUGACUCAACACAC

>TP2334

AGGUCGAGGUGGUAGUAUCUCUUAAACCCAGGAGUUCAAAAUAAGCCUGAU

>TP2335

AAUGCAGAAAAUGUUUCUCUUGGUCAGGUGCGGUGGCUCACACCUGUAAUC

>TP2336

CUACUAAAAAUACAAAAAAUUAGCCAGGUGUGGUUCAUCUCAAAAAAAAAA

>TP2337

UCUUUCUGAACAUAGUCUUUUCAGUAUGUGCUUUCUUUAGAGGUGAAGUAA

>TP2338

GAGUUGUAUUUUGAUUUGCCGUUUUAAGGUCUAGGCAGUGACAUACAUGCU

>TP2339

AGAAGGAAAAAGGGCCGGGUGCGGUAGCCCAUGCCUGUAAUCCCUAGCAAU

>TP2340

UCGUGCCAGCCAGCAGGGCUGAGCCAGUUUCACCCACACUGAGAGGGAACU

>TP2341

GACUCCUUCUCUGAAAAAAAAAAAAAGAGAGGGACAAUAAACAAGUAAGCA

>TP2342

CAUCCAUCACUUCAAGCACUCUAUUACCCUCAACACAUGUUGUGCCCAGUU

>TP2343

UUUUCUGAUUUCAGAUUUCAGUGCAAAUAUCCAUAAGCAAAAUCUGUUUAU

>TP2344

ACUUCUGUAAGCACUGCAAAUUGUAACUUGUUUACUCCUCAAUGGCAAUCC

>TP2345

UUAAGCAAGUGUUCAAGGUUACUAGAUGUAUUUAGCAAUUUAACAGAGGAU

>TP2346

GCUUUGAGCACAACUUUAAGAGGACAUAAUGGGGUCCUGGCCAUCCCACAA

>TP2347

UCUCUUCAAUAAAUGGUUUUGGGAAAACUGCAUAUCCACAUGCAGAAGAAU

>TP2348

GGAACAGAAUAGGGAACCCAGAAAUAAGUCCACACAUUUAUGGUCAAUUGA

>TP2349

ACUGUAAAGCUAUAGCAAUCAAAAUAGCAUGGUACUGGCAUAACAAUAGAC

>TP2350

AUAGCCAAAACAAUCUUGAGCAAAAAGAACAAAGCUGGAGGCAUCUCACUA

>TP2351

AACCACAAAAGAUCAUGAAUAGCCAAAACAAUCUUGAGCAAAAAGAACAAA

>TP2352

CCUGAAAUUCAUAUGAAACCACAAAAGAUCAUGAAUAGCCAAAACAAUCUU

>TP2353

CUAUAAAAUAUUGAUGAAAUAAAUUAAAGAAGACACAAAUAAAUGGAAUGA

>TP2354

UUAAUCAAGGAGGUAAAGGACUUGUACAUAGAAAACUAUAAAAUAUUGAUG

>TP2355

CAACAAACUAUCCCCCAAAGAAAUCAAAACAAUAAUCCCAUUUAUAAUAGC

>TP2356

UAGAGCUAAUAAAUUUAGUUAAAUUAUGGAAUACAAAAUCAUCAUACCAAA

>TP2357

AUGGCAUCCAAAUUGGAAAAGAAGAAGUAACAUUGUCUCUGCAGAUGACAU

>TP2358

GCUAGAGCAAUUGGGUAAGACAAAUAAAUGGCAUCCAAAUUGGAAAAGAAG

>TP2359

UUUAUUUUAUUAUUAUUAUUAUUUUAGACGGAACCUUGCUCUUUCACCAGG

>TP2360

UACUAAAAAAUUAACUGGACGUGGUAGUGCACGCCUGUAGUCCCCACUAUU

>TP2361

AGACAAGAAGAAAAAUUCACCUGUCACCCAGGCUGGAGUGCAGGGGCAUGA

>TP2362

GAGAUAUUCAUUUUUUAUUUUUAUUAUUUUAAAUUGUUUAUUAUUAUUUUU

>TP2363

UUUCCAGAGGCAGGGGUUGGGAGGAAGUGGCCCCUGUUGAGAAGCCCGGGA

>TP2364

CAAUAAGAAAUGCUGGCAAGGAUGUAGAGAAAAGGGAACACUCAUACACUG

>TP2365

GAGUUUGAGAUCAUUUCAGGCAACAAAGCAAAACUUCACCUCUACCAAAAA

>TP2366

CUACACCCUCAGCCUAGGCUCAAGCAAUCUUCCCACCUCAGUCCCCCAAGC

>TP2367

CUCAUGCCUGUAAUCCUAGCACUUUAGGAGGCCAACAUGGAUGAAUCUCCU

>TP2368

UUCAAAUGGCCUCCUUUUUCCUUCCAGCCAGACUUCAUAAGCAUGCUGAAG

>TP2369

AAGGUUCGUGGCAUCUUAUUUUGUCAAGUAAGGACACAGGAUAGGUAAAAA

>TP2370

CCUCUGAGCUUUGGUUUUCUCAUCUACUAAAUGGGGAUAAUGCUCAUAUUU

>TP2371

AUCUCAGCUUGGCUACUCACCAGCUAUGCGACCCUGGGCAAGUUACUUAAC

>TP2372

UCCUCGUUGCCCAGAAAUUCAAUGUAGACAUCCUUGGUAGGCAUUCAGGGU

>TP2373

ACCAUUGCACUCCAGCCUGGGCAACAAGAGUGAAACUCCUUCUCAGUCUUG

>TP2374

GAGUGCAGUGAGAGUCUGCAUUUCAAGCAAGCUCCCGAGUGACACUGAUGG

>TP2375

CUUCUCAUACUCUAAUAAUCAUCUAAGAAUCUUGUUAAAAAUGCAGAUUCU

>TP2376

GGCAGGCUGAGGUUGCAGUGAGCCAAGACUGCAUCACUGUAUAGCCUGGAC

>TP2377

GGUGUGGUGGUACACACCUGUGGACACAGCUACUUGAGAGGCAGAGGUAGA

>TP2378

CCUACUGAUCAGAAUCUGCAUUUCAACAAGCUCCCCAGAUGAUUAAUAUGC

>TP2379

GGAAAAUUUUUAUAUGUAUUUUUUUAGCCCUCAAAUUCAUUUUGCUUUCUC

>TP2380

GUUCUUUUAGCUUCUUAGUAUGGAAAUUUUCAAAUACCAAUAAAUGUAGAG

>TP2381

GGCCCUAACCUAAAAUCGGAGAGUAAUUUAUGCUUUGGAGAAUUUGACUCA

>TP2382

UACAACAACAGGAAAUAAUGAAGAUAAAGUUGGACUAAAUUAUCCCUGAGC

>TP2383

AGAAUUGGGGUGCAGACUCUACAUCACAGCCCUGCAGUUAUCACGGGCCCA

>TP2384

UAGUUCCUUGGUUCCCUCCUGGGGUAGAAAGAAGGGCAGCAAAAAGAGCAC

>TP2385

GAAAUCGCCCAGUGACAUAUUUCUAAGAAUGUAUUGCCAUUGUUAAGUGAC

>TP2386

UGUGUUUCUGUCUUAGUGUUUAAGGAAAAAAAAAGUUUUAAAAGAAAAAUU

>TP2387

GAGAUGGGGUCCCACUGUGUUGCCCAGGCAGAUCUUCAACUCCUGGGCUCC

>TP2388

AAAAUUUUUAUUUAUUUAUUUUGGAAAUGGGGUCUCGCUCUGUCAUUCAUG

>TP2389

ACCUGACCAACAUGGUCAAACAAAUACAAAAAGUAGCUGGUGGUGCACACC

>TP2390

UGAGUUGCCCAGACUGGAGGGCAAUAGUGUAAUCUAGGCUCACUGCAACCU

>TP2391

CGCAGUGGCAUGACCUUGCCUCACUACAGACUCCACUUCCCGGGCUCAGGU

>TP2392

AUCUAAUUUUGAAUAUAAUCUAAGAAUUGAAAACAGGGUCAUGAAGAGCUA

>TP2393

CAUCUAGUGAUCAGAUAAAAAGGGGAGGAAAUAAAAAUUAACACUCUUGUU

>TP2394

CAAGAAAAAUUUGAAAAGGGGCCAGACGCUGUGGCUCACACCUGUAAUCCC

>TP2395

UGUGUUUUGUUCGUUUGUAUUUUGUAGAGAUGGGGUCUCGUUUUGUCGCCC

>TP2396

CUCAGACUCUUAACCAGCUGGAAUUACUGCUGCGCACCACCACACCCGGCU

>TP2397

CCUUGCAGUUUCCCUUAGUUCCCCUAAUUUCCAAGUGAUCUGAACUAAAAU

>TP2398

UAAAGAUUUGGGAGCUUUUUGACAAAGGUACUCUGCAUGUCACAAAGGAAA

>TP2399

UGGCACAAAACAGGAGGAGUGAGGCAUCUCACAUAGUGGAAGCAGUAGCAA

>TP2400

CAGCUUCUGGUUUGGGGGAGGCCUUAGGAAACUUACAAUCAUGGCACAAAA

>TP2401

AGGCCAUGCCAGUGAGCUUCCCGUUAAGCUCAGGGAUGACCUUCCUCACAG

>TP2402

CGGCAGGUCAGGUCCACCACUGACAAGUUGGCAGUGGGGACACGGAAGGCC

>TP2403

AUUGAAGAAGGUAUUGUUGUAAUGGAAGAUGAUUCUCCAGUGGAGGCUGUG

>TP2404

GUAGACAUAAAAUAUAUGAAUAAAUAUAUGUUUUAAGUCACUUAAUAGCAA

>TP2405

AAUUGAUACAGACAUCAAAUCUAUAAAUCUAUUCUGUAGAUUUAUAGAUUU

>TP2406

CAGCCCCCCAAAUAGCUGGGAAUACAGGUACACGUCACUACACCCAGCUUA

>TP2407

CCAGGCAGCGUGCACCUGUGGUCCCAGGAGGUCAAGGCUGCAAUGACCUGU

>TP2408

AGGCGCGUACCACCACACCUGACUAAUUUUUGUAUAUUUGGUAGAGAUGGG

>TP2409

UGAGAAAAAUCUGAUGGUAAAUGCAAAACUCUUUCACAUCAUUCAGAAUUU

>TP2410

GUUGCCCAUGCUGGAGUGCAGUGGCAUGAUCCCAGCAUACCAUAACCUCAA

>TP2411

UGCACUCUAACCUGGGUGACAGAGCAAGACCUUAUCUCGAAAAGAGAAAAA

>TP2412

AUGCAGAUAUCCUACCUGCCAAAUAAGUCAGUGUGAAAGCUAAAUCACCUC

>TP2413

UAUUCAUCCUCAUUGUCGUCAUGUUAAAUAGACUAAGAGGGAGGAAGAGGA

>TP2414

GGGAUAUUGUAGCAAAUUACCACAAACUGGCUGAUUUCAAACAACAGAAAU

>TP2415

GUGAGGAAAUAAAUUUCUGUUGCUUAAGCCACCCAGUCUUUGGUACUUUGU

>TP2416

AAAUCAACCCCAUAGAGCUUACACUAUACGCCUCCAGAACAGUGAGGAAAU

>TP2417

UAGAUACCCAUACUUGCUAUGAGUCAGUCACAGUGCUGACACUAUUUUAAC

>TP2418

GUUAUCAUGGAGAUCUCAGUACCAGAGCAGUGAAUUUUUUGAAAAAUAUUG

>TP2419

AAAAUUAAAGAAAAACUAACUGGGCAUGGUGGCACACACCUGUAGUCCUAG

>TP2420

UCCAGUGAUCCUUUUACACUCUCUCAUAUAUAAAGUUCAGGAUUUUGGAAA

>TP2421

GAGGAUCUUUGUCUCUAUUGUACAGAUGUUCUAACUUCUUCCUUUCCAGUG

>TP2422

UUCCAAUUUUAUUCUCAUUUUGAGGAUCUUUGUCUCUAUUGUACAGUUGUU

>TP2423

CUCCUGUUUGGAGAACUCACACUGUAUUGCUGCUCUGAACAUCCAUGCAAU

>TP2424

AGUAAACUGGUAAAAAUCCUUAAAUAGUCCUGGUUCUUUCAAUUCUUGUUA

>TP2425

AUUUCUAGAUCUAAUCCUUUUUGUCAUGGAUUCUUUGCAAAAUUAAAAGUA

>TP2426

CAUAAGUCUAAGAAUUUAAAUCUUCAAUUAAGCACUAAGUUCCAGUAGGCA

>TP2427

CACUGUUGUACUUUUUGUCCCAGCAAUUUGAGGGCGUGCAAAUUCCACAAA

>TP2428

CCCUGAUCUGAUCACUAUACAUUAUAUGUAUCAAAACAUCACUAUGUAUCC

>TP2429

AGUGAUUACUACAGUCAAGCUAAUUAACAUACCUAUCACUUCAAAUAGUUA

>TP2430

AGUUUCCCGCCGCCCCAACCCUGCUAGAGUGCAGUGGUAUAAUCAUGGCUC

>TP2431

AAUAUGUUAAUUUUUACAUGAAAGCAUUAUUGUUGGUUUUAAGGUUCUUAA

>TP2432

GCUGGGAUUACAGGUGUGAGCCACCAUGCCCAGCCCAGGAUGGGGGCAUGG

>TP2433

AAUGAGAUACCAUCUCACGCCAGUUAGAACAGCAGUCAUUAAAAAGUCAGG

>TP2434

UCUUAUACAAAAAAAUUUUUUUUUUAGUUAUACUUUAAGUUUUAGGGUACA

>TP2435

UGGAGAUCCAGGUGAACGGAGGCACAGUGGCUGAGAAGCUGGACUGGGUCU

>TP2436

GCUGGACACAGUGUCUCAUACCUGUAUUUGCAGCACUUUGGGAGGCUGAAG

>TP2437

AAUCAAGAGCAGGAUGGAUGGAAGUAUGUACAACAAUGCUUGAAGUAAGAG

>TP2438

UCAUUCUCCAUGCAGUGAACAUCUUACAUGGAAUUGCAUUUGUAUUUAUCU

>TP2439

UAUUUUCUCUCUUAAUGUAAGCAUUAUGGUAUACACUUUACCCAUCCUGCU

>TP2440

GCCCAGGUGGUCCCUGCAAAGCAGCAGCAGCCUCAGGACAGGAGCAGCGUG

>TP2441

CCAGUGAUUAAUACUUUUAAUAAAUAGAAAGUAUGAGUUUUGUGGCUUGCU

>TP2442

GAGUAGUUUGUUUUUUAAUGAAAUGAAACAUGUACUGACAUGCGCUCUGUG

>TP2443

CAACCCUUCAUCAAGACCUUUUCUAAACAUUCGUAUCUCUAUAAUCCAGUU

>TP2444

UCAGAUAACAUCACCCUCCUAUUUAAAUACCUCACUAGUUUUCUACCAGGU

>TP2445

AGCAAAGGGUGGUGGGAUUAUCAUUAGUUCUUACAGGUUUUGGGAUAGGCA

>TP2446

CCGCCUGCACUCAGGUGAAAUAAACAGCCAUGUUGCUCACAGAAAGCCUGU

>TP2447

ACCCUAACUGAUCAAUGUUCUUUAUAAUCUCUCCGACCCUUAAGAAGUUUC

>TP2448

UAACUGAUGACAUUCCACCAUUGUGAUUUGUUUCUGCCCUACCCUAACUGA

>TP2449

GCAACUGAAGAUCCACAGAAGUGAAAAUAUCCUUAACUGAUGACAUUCCAC

>TP2450

UCUCUUUCAGAUAGUUUUAUGAUUCACACAGGUUUGAGGAUGCUGGGGAGA

>TP2451

GCAACUUUCCUGUUUAGCUGGGAUUACAGGUGCCUGCUACCAAGUCUGGCU

>TP2452

AGCAGGCUUUUUUUUGAUGAAUCAUAGUCAUAAUAUAGGUCAGCUUUUUCC

>TP2453

UCCAUCACCUGGAGCGCACUGGUGCAGUCUUGGCUCCCUGCAACCUCCACA

>TP2454

UCAGAUACCCAACUCAACAUGACCCAUACUAAGCUCAGUGACUGCACCCUA

>TP2455

UAUUUAUUUAUUUAUUUAUUUAUUGAGAUGGAGUCUUACCCAGACUAGAGU

>TP2456

CUUUGUAAAUUAUCCAGUCUCAGGUAUUUAUAGCAACACAAAACGGACUAA

>TP2457

AUGGCUUGGUACUCCUCUUAUGGUAAUGAGUGAGUUCUCAUUCUGUUCGUU

>TP2458

GACAUCCAAUGUUAGAGGCAGACCUAGUGGGAUAUGUCUGGGCCAUGGGAG

>TP2459

CUAACUGAGGGAGAACUUACUUAUCACCACGGGGAAGGAGCUAAACUAUUC

>TP2460

AAACAACCAGACCUUAUGUAAACUAACUGAGGGAGAACUUACUUAUCACCA

>TP2461

UGUUAGGCCUUUUUUUGUGUCACUUAAACACCUGAGGCUAGGCAAUUUAUA

>TP2462

CUUGCUAUGUUGCCCAGGCCAGUCUAGUACUUCUGAGUUCAAGCAAUCCUC

>TP2463

UACUUUUCCUUCCUCUUUCAGAGAUACUGUAGUUCAUCCCGUUGAUGAAAU

>TP2464

UAUAUAUAUAAAGGGGAGUUUAUUUAGUAUUAUUUAGUAUUAACUCACACG

>TP2465

CAGGCACAGGCCUCUUCACCUGGCUAAUUUUUUAAUUAUUUGCGGAGACAG

>TP2466

CUUAGGCACAGACUAGUGGAAAGCAAGGCGGGGUGCUUGUCUCUUUUAGGG

>TP2467

AGGCUGGGCUCCCCCGUUACUCCCCAGUGUUCAAAUCCUGGAUUCACUGCU

>TP2468

GCGACCUCAUGGCUGUGAACAACUGAGGGCUGCAGGCUGGGCUCCCCCGUU

>TP2469

UUUUGACUUGGGAUAUUUUCAGCUUAGGUUGGGUUUAUCGGGAUGUGACCC

>TP2470

GCGAUACACAUUCAGUACACUCCUCAACUUGUGAUGGAAUCACAUUCCAGU

>TP2471

UAAAAGGGUGAAUUUAAAGGUAUGUAAACUUUAGCUCAAUAAAGCUGUUAA

>TP2472

AAAGAUCCUAGUCUCCAGACUUGCCAGACAAUAAAUUUCUGUUGUUUUAAG

>TP2473

GUGGUUUCCCCCAUACUGUUCUCAUAGUAGUAAAUAAGUCUCAUGAGAGCU

>TP2474

GUGAAACCAUGUCCUUUGUUGCGACAUGAAUGCAGCUGGAGUCCAUUAUUC

>TP2475

AAUAGCAAAGACAUGGAAUCAACCUAGGUGCCCAUCAAUGAUGCACUGGAU

>TP2476

AUGGCGUAUAGAUGUGUAUAUAGAUAUCUGUGUAUGUACAUUUGUACAUAU

>TP2477

GUAAUAGAUCUGGUACAUGGCGUAUAGAUGUGUAUAUAGAUAUCUGUGUAU

>TP2478

CUGCCAUACUGUUGAAGAGAUGCGUAGGGUAAUAGAUCUGGUACAUGGCGU

>TP2479

CUCAUCUCUUCAACAGUGUGGCAGUAAGGAGCUCUAGACACAAAUGAUGAG

>TP2480

AUCUAUAUACCAGUUCUAUUACCCCACUCAUCUCUUCAACAGUGUGGCAGU

>TP2481

UAUACACAUAUGUACAAAUGUACCUACACAGACAUCUAUAUAUACAUCUAU

>TP2482

UUGUGUUAAGUGUAUGUGCAUAUAUAUGUAUGCACACAUAUACACAUAUGU

>TP2483

UCUCUUUAGCUUUUUCUCCACUCUGAGACUCCAUUCAGUCUUGAAAAUUCU

>TP2484

UGGGUCAUGACCACAUUAGAGGAUUAGUUAAUUGGCUUCUCCUGUUAACAU

>TP2485

UCCAGACAUACUCGUCCUUACCUCUAUUGUAGAAUAGCAGUCCGAUUUCUC

>TP2486

GAGUAACCCAAAUGUCUAUUCCUGUAGGAUCUCAGCAAUUUGCAGCCCUGC

>TP2487

AAAAAAAAAAAAAAGACAGAGUCUCACUCUGUCACUCUGUCUGGAGUAUAG

>TP2488

GGUUUUAGUGUGUGAGUGGGAUCCAAAACACUCAAUCCUGUAAAAGGGUAC

>TP2489

CUAUACUUAAAAACAUAUGGUGGCCAGGCAUGGUGGCUCACGCCUGUAAUG

>TP2490

UUUGCUUUUCUGUCCUAAUAAAUAUAUAUAUUUUUUGAGAUGGAGUUUUGC

>TP2491

CAUGCAAUUAGCCUUUUGAAAUCCAACUUCUGUGCAAAAUUUUAGUAUCAG

>TP2492

GCCUCACUUCUCUUUUUUUUUUUUGAGAUAGGCCCUUACACUGUCACCCAG

>TP2493

GAGCUCACUGAAGCCUCUACCUCCCAGACUCAAGUGAUCCUUCUGCUUUAG

>TP2494

AGACAGGAUCUUACUUUGCCACCCAAGCUGGAGUACAGUUUAAGAGCUCAC

>TP2495

UUCAGUCUUUUUUUUUUUUUUUUUUAAGACAGGAUCUUACUUUGCCACCCA

>TP2496

AUUAUAGGCAUUAGCCACUGCGCCCAGCCUCCACCUGUUUUUUAAGUGUAU

>TP2497

CUGUGUUUUCUCAAGAUUGAACAAAAGCUUGUCCAGGCCCAGAACUUAACU

>TP2498

AAAUGACAAAAGUCUGUGAUUGGACACACUUUUUUUUUUUUUUUUGAGACG

>TP2499

GCACACACAGUGGCUCACACCUGUAAUUGCAUUGUUUUGGGAGGUCCAGGC

>TP2500

CGCCUCCAGGGUUCAAGCAAUUCUCAUGCCUCUGCCUCCUGAGUAGCUGGG

>TP2501

CUGGGCUGGUCUUUGCCAACAUGGCAAAACCCCGUCUCCACCAAAAGUACA

>TP2502

UUCCCUGGUGUAUCUGGGAUUACCAAUGUGCACCACCACACCCGGCUAAUA

>TP2503

GAACAUAAAACUCUUCCAGCAUAGAAGAAAAUGGGUACUAUUAACAUUCAG

>TP2504

UUUCUUUUUUUGAAAUUAAGAAUUUAGCUGAAUGCUGUUGCUCACACCUGU

>TP2505

GGAAUGGGUUCCUUAUAAAAAGAUGAGUUCAGUCCCCUCUUGCUCUCCUCU

>TP2506

UGGGUCAUGGGGGCACCGCCUUCAUAAAUGGAUUAAUGCCAUUAUUGUGGG

>TP2507

CAGUGGUGUUGGGAAGUGAGGCCUAAUGGAAGGUGUUUGGGUCAUGGGGGC

>TP2508

GAUUUGAAUGUGUCCCCCAACGUUCAUGUGUUGCAAAUUUGAUUCCCAAUG

>TP2509

UGCCAUUCAUGAAGAAUCUGCCCCCAUGACCCACACACCUCCCACCAGGCC

>TP2510

AGAAGGGGAUGACACCUUGCCAUUCAUGAAGAAUCUGCCCCCAUGACCCAC

>TP2511

GGCUCACAAUCAUGGCGGAAGGCAAAGGGGGAAGCCCGGCAUAUCCAUGGU

>TP2512

UCGUGUAUUAUAAUGUAACAUCAAUACAUCAUUUCUUAUGUUAAUUUGUCU

>TP2513

AUUCGGAUUCAAGACUAUGCCAUUAAAUGACCACAUUAACGAGCUCUCCGA

>TP2514

AGCUUGGGCAUCAUAGCAAGACCCUAUCUCUAUUAAAAAAAAAAAAAAAAA

>TP2515

AUAACAGUUUUGGAAGAAAAUAUGAAAGGUCACACUUCAAUCUUCUUUUAA

>TP2516

UGGGUUCAAGCAAUCCGCCUGCCUCAGCUUCCCAAAGUGCCAGGAUUACAG

>TP2517

UGUGAUUUAUAAACAACAGAAAUUUAUUUCUUACGGGUUUUGAGGCCGGAA

>TP2518

UCAGCCUGCUGUAAUAAAAUACCUUAGACUUUGUGAUUUAUAAACAACAGA

>TP2519

GGUCCUAAAAUUAGGACUAAAAUUAAGACCAAUUCUAGCUAUCGCAGUCUC

>TP2520

GAUUAUGCGUAAGGAAACUCAAGAUAUAAAAUUCACAGAAGAGAUCCCCUU

>TP2521

UGUAAGUCUAUUCUGGAGAUUAUGCAUAAGGAAACUCAAGAUGUAAAAUUC

>TP2522

UCGAUGUCCACCGUAAAGAAAAUGCAGGGGCUGCUGAGAAGUCGAUUACUA

>TP2523

ACGAGAUCUGGGCCCUCAAGGCCAUAGAGGCGCUUUCAGGUAAAAUAGAAC

>TP2524

GCGUUCGUGGACUGCCCAGACGAGAACUGGGCCCUCAAGGCCAUUGAGGCG

>TP2525

AAAGACGGAAACUCUGGAAGUCUGUAGGUCUUUGCCGCAGAACUCCUGGAA

>TP2526

AUUUGGCCCUGAGAGAUGCCACGUGAUCGUCAUUUUAAAUCCUGUACGUGU

>TP2527

CUCAUAUCUUUGCCAAGUCAAACCAAACUACCUCCUUCUAGAAAUUUGUCU

>TP2528

CUCGGGACCCAUCUGUGGUGGACUCACCGGGCAGCCUUUUGGGGUGAUGUC

>TP2529

CCCUCUGAGUCAGAUAAACUGAAAUAAUCACUUUGAGGUGAUUGAGAAGGC

>TP2530

UGUCAAGAGCUGGGUAUAAAACUGUAUGGGUUUUUUUGUUUUUUGUUUUUU

>TP2531

ACACAAAAUGAGAACAAAAGCAUUAAGAGUUGGGCACGGUGGCUCACGCCU

>TP2532

AUGCCAUUGAUAGGUUGAACAUAACAUUUUUCUUAGAAUAAAAGCACAUUC

>TP2533

ACAAGGCAAAGUCCCAUGACAGGCCAUCUGCAAGCUGAGGAGCGAGGAAGC

>TP2534

AGACAGAGCCUCGAGUCAUUCUGCCAAGAGGAUCCAGAAACACAGACUUUU

>TP2535

UAGGGGACACCGGCCACCCACCCUCAGCUUCUUCCCCCACUUCUUGGAGAA

>TP2536

CUAUAGGCAUGCGCUACCAGGCCUGACUUUUUUUUUUUUUUUUUUUUUGUA

>TP2537

UUUUUUUUUGUAGAAAUGGGGUUUUACUGUGUUGUCCAGGCUGGUCUUAGG

>TP2538

CAUUUAUUCCAAAUGUAUGAGCAGGAAGGAAAGAGGGAGGGAAGCUGCAAG

>TP2539

CCCCGUUUCCAUGUCUCCCCACGUCAGGGUGAACACUUUUUAUGCGCUGUG

>TP2540

UUGAGAUUCGAGACUGACAGAUGCAACAGAGAUUUUAAAUUUUGCAUAGGC

>TP2541

UGGGAGGAUCACCUGAGCCCAGGGAAGUAGUGGCUGCAGUGAGCUGUGAUC

>TP2542

GUAAAACCUCGUUUCUACUAAAAACACAAAAAUUAGUCGGGCGUAGUGGGG

>TP2543

CGCCAUCACGCCUGGGGGUUUCACCAUGUUCAACAGGUUGGUCUCGAACUC

>TP2544

CACACCUCAAAUUAUUUUAAGGGCCAGGCAUGGUGGCUCACACUUGUAACC

>TP2545

AAUUUUAAACAUUUCUUAAUUUUUUAUUUUUGUAGAGAUGGGGUUUCACUU

>TP2546

GAACCAGAAGGACCCCGGGGUCCUUAACCGCAUGAUGAAGAAACUGGGCAC

>TP2547

CCCGUCACUGAAAAAUAUAAAAAUUAGCUGAGCAUGCUGGUGAUUUUCUGU

>TP2548

CGUGCCUCAGCCCCCCAAGUAGCUGAGACUACUGCUGCACACCGCCACACC

>TP2549

AGAAAAAAAAAAUAUGUGUUUUUGUAGAGCUAUGUUUUGCUAUAUUGCCCA

>TP2550

UUUCUGGAGGCAGUAGCUUGAUCAUAGCUCACUGCAGCCUUGAUCUCCUGC

>TP2551

CCAAAUAUGGUGGCGUGCAUCUGUAAUCCCAGCUAUUCAUUCGGGCGGCUG

>TP2552

UUUUUAGUUUAUUUUCUGUGGAGAUAGGGUCUCACUGUAUUGCUCCGUCUG

>TP2553

UGGUUUGGGUGUUUGUCCAGGUGUCAGGCUGUUCUCGCAUUGCUGUAAAGA

>TP2554

UGUCAGGCUGUUCUCGCAUUGCUGUAAAGAAAUACCUGAGACUGGGUCAUU

>TP2555

GCCUGGCUUCUAGGGAGCCUCAGGAAGCCUUUAAUCAUGGUGGACGGUGAG

>TP2556

UGUAAGGAAUGUAGGAAAUCCUUCCAUGUGAAACCAAACCUCACUAAACAU

>TP2557

AUUCUCCCUAGUAGCUGGGAUUAUAAGUGCAUUCCACUAUGCCCUGCUGAU

>TP2558

UGUUAAUCCUCAAAAUUUGGGUGUUAUACCCCUAUUAUAGAUAAGGAAACU

>TP2559

UCCCAAGAAUUAAGCACAAGAAGAAAGGCACAAUGUCCAUGGUGAAUAAUG

>TP2560

GGCACAAUGUCCAUGGUGAAUAAUGACAGUGAUCAACAUGGAUCUCAGUUU

>TP2561

AAGGCAUUGACAUAAUUAAGAAAAUAAAUGAGACCUUUGUUGACAAGGACU

>TP2562

UAGAAGAAAUAAAGGCAGAAAAAGAAGCUAAAACUCAGGCUUUACUUUUAG

>TP2563

AGAAAAAGAAGCUAAAACUCAGGCUAUACUUUUAGAGAUGGUGGGAGACCU

>TP2564

ACAGAAAAUGCUACAUAUAUUACAUAGAUAUACAUAGAGAUACACACAAAC

>TP2565

CACACAAGGUCUUGCUAAUUUGCCCAGGCUUGCCUCAAACUCCAGAGCUCA

>TP2566

CAUGGCAACCCGUUCUCUAGUAAAAAUGAAAAGAUUAGCCAGGUGUGGUGG

>TP2567

CCUUUGCCUUCUGCCAUGACUGUAAAUUUUCUAAGGCCUAUUCAGCCACGC

>TP2568

AAGGCCUAUUCAGCCACGCAGAACUAUGAGUCAGUUAAACCUCUUUUGUUU

>TP2569

GUUUAUAAAUUACCCAGCCUCUGGUAGUAGCUUUAUAGUACUGUGAAAACA

>TP2570

GGAAUGCAGAAAACCCUCAGAAACUAGACGAAGCAAGGAAACAGAUUCUCC

>TP2571

UGACCAACACAGCCUCCAGAACUGUAAGGCAAUACAUUUGUGUUAUUUUAA

>TP2572

UAAGGCAAUACAUUUGUGUUAUUUUAAGCCACUACACUUGUGUUAAUUUGC

>TP2573

UUUUCUAGGACUGCUGUAGCAAAUUACCACGAACAUGGUGACUCAAAAUAA

>TP2574

CACGAACAUGGUGACUCAAAAUAACAGAAAUUUAUCCCCUCACAGUCCUGG

>TP2575

AAUAGACCGAGACUCCGUCUCCAAAAAAAAAAAAAAUACAAUUUUUAUUUC

>TP2576

CUUUAGAGAACAGGAUAUAAAGUAUAAUAUUGAUGUAUAGUUCAAGCUAUA

>TP2577

AAUAGUACUAAUUACUAAGAUACUUAAACUUACAUUGGUAUCAUUACCUAC

>TP2578

AUCCUUUUUGGAAGGGAUCUUCCAAAAUUAAAUCAAUCAAGCAAUCAAUAU

>TP2579

AGAUGGGGGUCUCGCUGUGUUGCUGAGGCUGGUCCCAGACUCAUAGGCUCA

>TP2580

UUGCUGAGGCUGGUCCCAGACUCAUAGGCUCAAGCCCUUCACCCUCCUCAG

>TP2581

UGUCCAUAAAGUGUAUAGAAAAGAUACUGGAGCACUAUUCAUCAAGCACUU

>TP2582

AAUGUGUUAUUCACACCCAGAGAUAAACUAACAGUAGAAGAACUGGAACAA

>TP2583

AAAAAAAGUCCAUAGAAUAUUUACCAAAGUAGACCUUGUUCUGGGCCAUAA

>TP2584

AAAGAACUUGUCCUUUAAUAGGGUCAAAAAAAAGGGACAAACUUAUGAUAA

>TP2585

CGGUAGUAUGAUUAUAGCUCACCGCAGCCUUGAACUCCUGGGCUUAAGCAG

>TP2586

AUCUUCCUGCUUUACCCUUCCAAGUAGGUAGGGCUACAGGCACACACCACC

>TP2587

GACUUAGAUUAGGCAGUAGUUUCUUAGAUAUGACACCAACAGCAUAAGCAA

>TP2588

AUAUAGGUUGACUAUCCCUUAUCCAAAAUGCUUGGGACCAGAAGUAUUUCA

>TP2589

UUCAAAUUUGGGAUGUUCAGCUGGCAAGCGUAAUGAAGUAUUACAAAAUCU

>TP2590

GAAAUGGACAGGCUGUGGUUAAGUAAAUAUGUAAAUACAUACUAGUAGUAC

>TP2591

GUAAAACUGUCCCGGGUUUUGUCUGAUGAAUCUGUUGAGUUUGGUUGUAGU

>TP2592

GCGUAAUUUUGAAAUAUAUCCUCACACCUGGAAUGCCAGCACUUUUAGAGA

>TP2593

ACACUUUCAAAAACCAGAUCUUGUGAGAACUCACUAUCACAAGAACAGCAA

>TP2594

AGAACUCACUAUCACAAGAACAGCAAGGGGAAAACCACCCCCACGAUCCAG

>TP2595

CUGCAUUCCACCCCUCGUGGAGGGAAUGCACAGGUGAGUGGGUACAGGAGC

>TP2596

AGCAGGUUCUUGGCUCUCAUGCAGGAAAGAAUUCAGGGCAAGUCACAGAGU

>TP2597

UGCCUAUAGUCCCAGCUACUUGGGCAGCUGAGGCAGCAGAAACGCUUGAAC

>TP2598

UGGGCAACGAGCGAAACUCCAUCUCAAAAAAAAAGAAAAGGAAAAGAAAAG

>TP2599

GGAUUGCUUGAAACCAGGAUCUAGAAACCAGCCUGUGCAACAAAGCAAGAC

>TP2600

UCAACGCCGGCUACUAGCAAGAUUCAAAGAUAAAACUCCUGCCCCUCUUCA

>TP2601

CCCAGUAGUGGGGUUUCUGGAUCAUAUGAUGGUAGUUCUAUUUUUAACUUU

>TP2602

AACAGGAGUGAGGUGAUGUCUCAUUAUGGUUUUGAUUCUCAUUUCCCUGAU

>TP2603

GAUUGUUUCUAAAACAAAUGCUUUCACAAAGUAGAGGUAAAUCUUUUUUCU

>TP2604

UGUCCAUGAUUUACAGUGAGGGGUCAUUACAGUGAGCACAGCUUUAGGAAC

>TP2605

GGCCAAACAGCCACAUGGUGAGGCCAGUCAUUGACUUAAGAGAAUCCACCU

>TP2606

UCAUUGACUUAAGAGAAUCCACCUUAAAUUAGCGCCUUUACCAUUUUCCAG

>TP2607

UAAAUUAGCGCCUUUACCAUUUUCCAGGUAAUGCAAGGAGCUCACAACUCU

>TP2608

AUUUUCCAGGUAAUGCAAGGAGCUCACAACUCUUUGAUCCUUUCUGACAUU

>TP2609

CCUUUCUGACAUUUUGUGCUAUUAUAGUCAUGUCUUUUAAUUCUACAUUUA

>TP2610

AGUCAUGUCUUUUAAUUCUACAUUUAUUUAAGCCCUCACAAGAUAUUAUUA

>TP2611

UUUAUUUAAGCCCUCACAAGAUAUUAUUAUUAUUAUCACUGAAAAUAUAUU

>TP2612

UAUUAUUAUCACUGAAAAUAUAUUUAUUCAAUCUGCCCACGUUCCUCCUGU

>TP2613

UGCCCACGUUCCUCCUGUGCUUGUGACACCCAUUCCAUUCUGCCUUAGCUU

>TP2614

UCCAUUCUGCCUUAGCUUGCUUGAUAAGCUCCUUUUAAUAUUUCUUUUGGU

>TP2615

CAACCAGCCCCAGCUCCUCCUCCAUAGUGGUCUCGAAACUUGUAUUCUUCU

>TP2616

CAGGGCCUGUUUUGCACAGCCCUGCAAUAUCUGGGCACAGAUCACUGUGGG

>TP2617

UGGGGGUGACGUUUGCCCAGAGAAGACUUUCUACUGUCAGAGAUGUUGGCG

>TP2618

UUGGCCCUUUUUGCUUUUGUCAGCAACAGAAGAACCUUGAAGGCUAUGUGG

>TP2619

ACAUUUAUUUAUUUAUUUAAAGACAAGAUAUUGUUCUGUCACUCAGCUGCA

>TP2620

AGUUUAAAUUUUUCCAGCAUCAUUUAUUUGAAAGAAUUUCCUUUCCCUGUU

>TP2621

CACCUCCUGCCUCCCGGUGGCCUGUACAGGCCCAGCUCUGGCUGGAGAACA

>TP2622

UGGCCCCGGGAGGCCGCGGUGGGGCAAGAGCUUGGCCUGGAGACGCCCCUG

>TP2623

CUUCUCCAGCCAGAGCUGGGACUGUACAGGCCACUGGGAGGCAGGAUGUGG

>TP2624

AAACGCCGGCGAGAGCUGAGCCGAAAGAGCUUGCUUGCUGGGAGGCAGGAG

>TP2625

AGGAGCUGGGCCGGGAGAUGCAGCCAGGAGGAACAGCUGGGCCUGCAGAGG

>TP2626

AGUUUUCCCACCCCUGUUUAUUUUUAAGACACAGGGUCUUGCUGUGUUGCC

>TP2627

UUUUGAAUGGAGCCCUCCGCCUCCCAGGUUCAAGUAAUUCUCCUGCCCUAG

>TP2628

AUUGGAGGGUGAGCAUGUGGUCCGUACUACUCGUGAAGCUGAGGUGAGAGG

>TP2629

AAGAGACUUGAAUAAUAAUAAUUCAACAACAGCUUUAUUUUUAUGUGGAGA

>TP2630

CUUCCAAAAUCAUUGGAAUAAAUGGAGAUUUCUUUGCUAACAUGGUAGUAG

>TP2631

GUGCUACGGCAGUUAGAAGAGUUUUAAAAAGGGACCUUAAACGCAUUCCGA

>TP2632

UAAACCUCAAGACAACAAACAAGCAAGGGUGUUUGAACCAACCAUAGUUAA

>TP2633

UAGUGCCUUUGUGACAGGGGAGCUUAGUUCCUGACAAUGUCCUCUUGAGUC

>TP2634

GCCUCUGCAGAAUGAAGGUUGGGGCACGGGGGGCGCUCUACUUCUUAGGGA

>TP2635

AGGUUAUUCAAACUCAGAUCUUGAAAGCAUAAUGAUGAUAGGCCAUGGUCU

>TP2636

AUACCGCCAACCACCGCUGGCUGGGAGGAGUCGGAGACUGAGACCUACACA

>TP2637

CUGGGCCUGGUGGUGCACACCUAUAACACCAGCUACACAGAAGGCUGGCAU

>TP2638

AGAUCGCGCCACUGCACUCCAGCCUAGGUGACUCUGUCUCGGGCAAAAAAA

>TP2639

UAAAGCCGAGCCCAGCCAUGGGUUUAUACCAUCAAGUACCUAUGAGUUGGG

>TP2640

CAGGAGUUCAAAGCUGCAACGACCUAUGACAGCACCACUGCACUCCAGCCA

>TP2641

CAAUGUUUGUUGAGUGGAAUAUUGAAAAUGUAGGCAGCAACUGGGCAUGGU

>TP2642

GACGCUGUCUCUACAAAAAAUAAUUAGCCUGGCCUGGUGGUGCAUGCCUAG

>TP2643

UGACGUGGGAGGAUUGCUUGAGCCUAGAGUGAGCUAUUAUCAUGCCACUGU

>TP2644

GAACACUGUUGAUGUUCUUGAGGGAAGCAUAUUGGGCUUUAGGCUGUAGGU

>TP2645

UACUGUUGCCAUGUUGUAAGUUGUAAGAUUGGGAUGUGUGAGUCCUACAAC

>TP2646

AGGCUGGUGGCUUGGAAGACACUGGAUCUCACUGGCUCAGCUGGGCACGGU

>TP2647

CCAUGUUAACAUUUCUCUUGUGAAAACUGUGCAGAAAUUUUGGCAUAAGAU

>TP2648

CCCCACCACGUACCAGAUGGAUGUGAACCCCGAGGGCAAAUACAGCUUUGG

>TP2649

AAAUUACAAGUGUACCCAAUAACUGAAAAUGUUUUAACUCACUCUCAUUUG

>TP2650

UUGACUUCAGAAUUGUUUGGGGGCUAGGUGUGGUGGCUUAUGCUUGUAAUC

>TP2651

CAAAGCUGCGGCGAGCUAUGAUCGCACCACUGCUGUCUGGCCUGAGUGACA

>TP2652

GAUCGCACCACUGCUGUCUGGCCUGAGUGACAGAACAAGACCCUGUCUCAA

>TP2653

GAAACCCAUCCAGGCUCACACAUGUACAAUAAGUAGCUUUGAGAAUCUUAA

>TP2654

GCAGAGAACUAGUUCUGAGCUAGCUAUGAAGUUGUAUUGCAUAUUGGAGUG

>TP2655

AAACUUCACUGAUCACCUGAGUGUAAAGGAGAUUGUAGGCAGGUUACUUAA

>TP2656

UCCUUGUGAUAGUUUGCUGAGAAUGAUGGUUUCCAGUUUCAUCAUGUUUUA

>TP2657

UUUGUCAAAGAUCAGAUGGUUGUAGAUAUGUGGUGUUAUUUCUGAGGACUC

>TP2658

UGAUUCUUCCAACCCAUGAGCAUGGAAUGUUGUUCCAUUUGUUUGUAUUCU

>TP2659

GGAGACCCUGUCUCUGCAAAAAAUUAAGAAGUUAGCUGGGUGUGGUGGUUC

>TP2660

UUAGGUCCUGCGAAUAUUGCUGAAAAUCGGAUUUUCCACAUUCACUUUCUU

>TP2661

CUUAGCCACCCAAAGUGCUGGAAUUACAUGGGUGAGCUACCGUGUCCAGCC

>TP2662

CUUAUUCACGGUAGCAAAAAAUGGAAACAGUCUGGGCGCAGUGGCUCACAC

>TP2663

GUAAAGAUGGAUCAAUAGAUCACAAAGGAAUAUUAUUCAGCCUUAAAAAGG

>TP2664

AAGACCAGCCUGAGCAACAGAGCGAAACCUCAUCUCUGCAAAAAAUUAGAA

>TP2665

ACCUCAUCUCUGCAAAAAAUUAGAAAAUGAGCUGGGUGUGGUGGCACAUUC

>TP2666

CGUAGUGAAACCCCACCUCUACCUAAAAUACAAAAAUCAGCUGGUUGUGGU

>TP2667

GUGUUACCCUCCUGUGGUUCCAGCUACUUGGGAGGCUGAGGUGGGAGGAUC

>TP2668

AAGUAACAGGGAGGCUGGGCACAGUAGCUCAGGCCUGUAAUCUCCACGCUU

>TP2669

CUCUACUAAAAAUACGAAAUUAGCCAGGCAUCUUGUAAUCCCACCUACUCA

>TP2670

GGCCAAGACAGGAGGAUCACCUGUAAUCCCACCAUUUUGGGAGGCCAAGAC

>TP2671

AGAUGGGUCUCGGCUGUGUUACCUAAGCUGGAGUGCAGUGGCUAUUCCCAG

>TP2672

CAGUGGCUAUUCCCAGCACAAUCAUAGUGCACUACAGUCUCCAACUCCUGG

>TP2673

CUCCUUGUGCCUCCGCCUCCUGAAUAGCUGGGAUUGCAGGCAUGUGCCAUC

>TP2674

UGCAGUGGUGCAAUCUCGGUUCACCACAAGCUCUGUCUCCCAGGCUCAAGU

>TP2675

CCAGGCAUGGGGGCUUAUACCCGUAAUCCCAGCACUUUGGAAGGCCACAGU

>TP2676

CAGGCCAGGAGUUAAAGAGCAGCCUAGGCAACGUGGCGCGACCCCAUCUCU

>TP2677

AUGUUUCACGAUGUUUGACUGUGUUAGCCAGGAUGGUCUCGAUCUCCUGAC

>TP2678

CCAUUCCCAAGGCUUAGGUAUCCAGAGCAGGGCAUCUGGUGGGGAGGCCUG

>TP2679

GAAUUGCCUGAAUGUGGGAGGCAGAAGUUGCAGUGAGCCGAGAUCACAGCA

>TP2680

UGGGACUUUAGGCAUAUACCACCAUACUUGGCUAAUUUCUUGAUUGUUGUU

>TP2681

UCCCACCACUGCACUCCAGCCUGGAAAGCAGAGUGAGGGAGACUCCGUCAC

>TP2682

CAAUUCUCCUGCCUUGACCUUCCAAAGUGGUAGAAUUAUAGGCGUAAGCCA

>TP2683

UCCAAAGUGGUAGAAUUAUAGGCGUAAGCCACCACACCGUGGCCUGGUUUC

>TP2684

AGGGAGGAAGGAAGGAAGGAAGGAAAGAAAGAAGGAAAGAAAACAGUGUUG

>TP2685

UUGCUCUCCUGCAGGGGUUGGAUGGACUUCUGGGAAAAGCACCUGUAUUUG

>TP2686

GGAGUGGGGCCUCCAGCGUGAAAACAGAAGUCACCGUCAGCCUUGCACCCC

>TP2687

CUAAGACACCUGUGAAGGGUCCCACAAGGCCAGGCACAGUGGCUCUUGUCU

>TP2688

GCAGAGCUGGGGGAGCGAGGCUGACAAUGACUCCUGUUUUCACGCUGAGAG

>TP2689

AAAAAAAAUAUAUAUAUAUAUAUAUAUGUAUGUAUGUAUAUACACAUAUAU

>TP2690

AAAACCCAGCUGCGUGUGGUGGCACAGCCUCAAACUCCUGGGCUCAAGCGA

>TP2691

AGAUAACCACUUCUAAAAUUUUUUUAGAGAUAGGGUCUUCGUCUUACCCAG

>TP2692

AUGGGAGCUUUGGAGCUCAGUCCCAAGGUCCUCAAAUAUUUGAGGGACCCA

>TP2693

GCUGUAGCAAGAUACUCCCAUGUUCAAGGCGGAGCAUGCAGUGAGCCGAGA

>TP2694

AAUUCUGAAAGAGGAGAAUUGCCUGAACCUAGGAGGUGGAGGCUGCAGCGA

>TP2695

AAAGGCGACAGGGAACCCCAGAGCUAGGUCCCAAGAGCCUCUUGGUGAUGG

>TP2696

GGCUUGUCUCAGACAGUCUGGCACUAGCCCUCAAAGCUCAGAAUCCCUGAU

>TP2697

UAGCUUCACGUCAAAAUUUUUCUAUAAUUUUCUGAAGUUAGAGUCUUAAUU

>TP2698

CUUCAUGGUUCUCUUGAAGAAAAAUAGGAAUGGAGGCCACCUCUGUGUCAA

>TP2699

AGUGUUUAUUAGGACUGGGCAACAUAGUGAGACUCUGUUUCUAUGAAAAAU

>TP2700

AAGCAAUUCUCAUGCCUCAACCCUCAGCCUCAUGAGUAACUGGGACUACAG

>TP2701

CCUACAAAAAAUAGUAAUAAUAAUUAGCUGGGCAUGGUGACAUGAGCCUGU

>TP2702

AGGUAUCAUUUCACUCUAAAUUAAUAUAAAGGUUAUUUGUUUCACUCAUAG

>TP2703

AUGCUGAGGAACUUUGCCUUGAUGAAAAUGUGGUUUUUUAAAAUACCCAGG

>TP2704

UGAGGUGGCAUGAUGGCCUUAGCCCAAGAGGCGGAGGUUGCAGAGAGGCAA

>TP2705

GCAUGUCUUACAUGGUCAGAGCAGAAGGAAGAGAGAGAGAGAAGGGGGAGA

>TP2706

GACAGAGAAGUAGGUACUUAGACCAAUGGAACAGAAUAGAGAACCCAGAAA

>TP2707

AGUCUGCAAGGAACUCAGAUCAGCAAGGAAAAAACAAUUCCGUUAAAAAGU

>TP2708

UCCCACUUCAGUCUCUGAAAGUGCUAGGAUUAUAGGCAUGAGCCAUUUAUG

>TP2709

AAUGCAAAUCAAAACCACAACAAUCAGAAUGGCUGUUUUUAAAAAGUCAAA

>TP2710

CAACAAUCAGAAUGGCUGUUUUUAAAAAGUCAAAAAACAACAAAUGCUUGC

>TP2711

UGUUUUUAAAAAGUCAAAAAACAACAAAUGCUUGCAAGGCUGUGGAAAAAG

>TP2712

AGGCUGUGGAAAAAGGGGAAUGCUUAUACACUGUUGGUGGGAAUGUAAAUU

>TP2713

GGAAAGCAGUUUGGAGAUUUCUCAAAGAACUCAAAACAGAGCUUCCAUUCA

>TP2714

AAACAGAGCUUCCAUUCAACCCAUUACUGGGUCUAUAUCCAAAGGAAAAUA

>TP2715

CAACCCAUUACUGGGUCUAUAUCCAAAGGAAAAUAAAUCAUUCUACCAGAU

>TP2716

GAUAGACACAUGCAUUUUUAUGUUCAUUGCGGCAUUUUUCACAGUAGCAAG

>TP2717

AAAUCAAUCUAGAUGCCCAUCAAUGAUGGAUUGUAUAAAGAAAAUGUGUUG

>TP2718

AUUGUAUAAAGAAAAUGUGUUGCAUAUACACAAGGAAUAUUACACAGCCAU

>TP2719

AGAAAACCAAAUAUUGCAUGUUCUCACUUAUAAGUUGGAGCUAAACACUGG

>TP2720

AUGUUCUCACUUAUAAGUUGGAGCUAAACACUGGGUACACAUGGAUGUAAA

>TP2721

GUCCCUUGGUCAUCCAGUUUGGAGUACAGUGGUGCCAUCGCGGCUCACUGC

>TP2722

CAGGUGUGAUGUGUGUCUGUCAUGUAGUCCUAGCUGCUCCACAGGUUGAGG

>TP2723

AGGCAUUAUAUUAUGUAGUGCUGAAAAACAGGCGGUGACAUGGUACCAAAA

>TP2724

UACAAAAACAAUACAUAAAUUGCAAAGACUCUCUAAGUGAACACAUUCAGU

>TP2725

GUAAGAUUUCAUCAUGCCACUUAGAACAGUGCACAAUUUAAAACUUAUGAA

>TP2726

GAUGAGCUAUCUUACCGUGUGGAAUAAACAGUGAUUUAGAAGAUUACAUUG

>TP2727

AAUGUAAUCUUCUAAGUCACUGUUUAUUCCACACGGUAAGAUAGCUCAUCA

>TP2728

AUCAGCCAGAUGUCUGCAGUGAGCUAUGAUUGCACCAUUGUACUUCAGCCU

>TP2729

CCCUUUUUGUUUGUUUUUUUGAGACAGGGUCUCACUGUCACUGAGGCUGGA

>TP2730

GAAAUAAAAGGGAAUAGCCAGGCAUAGUGGCACAUGUCUGUAGUCCUAGCU

>TP2731

UCUGCUAUUUAUUAGUUGUUGAUUUAGGUAAUUGGUAGCCUGCCUGUUCCU

>TP2732

AAGUUCAUAUUAUUAUCUCCAUUUUACAUAUGGUGAAACUGAGGCUUAGAG

>TP2733

AACCCUGCUUUUAGCUGGACAUGGUAGUACAUGCGUGUAAUCCCAGCUACU

>TP2734

AUGGUUACUUCUAGGGAGAUCCCAAAGGGGGUUUUGAUUUGGAAAGAUGGA

>TP2735

GGGGGUUUUGAUUUGGAAAGAUGGAAGAAUGUUUGAAAACAUGAUAGGAGA

>TP2736

UUCAUAUCCAGUUUCAAUGGCUCACACCUGUAAUCCCAGCUACUUAGGAGG

>TP2737

GCUCACUGCAUCCUGGAUUUACUCAAGCGAUCCUCCUGCCACAGCCUCUCC

>TP2738

UGAUUUGGUCUCAAACUAGGCCCAAAUGAUCCUCCUGCCUUGGCCUGCCAA

>TP2739

CAAGGAGAUUCUGAGAAGUGGCAGUAAAAGGAGUAGGAAGAAAACCAAGGG

>TP2740

GUAAUUGGGAUUACCAAUAUGUACUACCACACCAUUUUUGUACUUUUAGUA

>TP2741

CCAUUGCAACCUGGGCAAUAUAGCAAGACCUUGUAAUCCCAGUACUUUGGG

>TP2742

UGUCUAAGUGCUUGCAUAGAGCAGAAAGCUGAGUUGGUGACAGCAAUUUGC

>TP2743

UAUCUGUUAUGAAUAUAGAUGUAAAAAUCCUCAACAAAAUAUUAGCAAAUU

>TP2744

UAGGGCAUGUUUCCGCAAUUGGCUUAAAAAGUUCAACCCCUGCUGGGUGCA

>TP2745

GUUUUGAGACCAGUUUGGGCAACAUAGCGUGACCCCAUCUCUAUAAAAAAG

>TP2746

GGAGACUGAGGCAGGAGCAUUGCUUAAGCCCAGGAGUUGGAAGCUACAGUG

>TP2747

CAGCUGAGACUUCCCAGGAGCAGGAAGACCUUUUCAUAGUGAAGGUGGAAG

>TP2748

UGAGUCCAGGGACAAUAUGGAGCUCAUAGUGAAGCAGAUUUCUGAUGACUC

>TP2749

UCAAUCCACCACUUCUCUGUGAAACACUCUACCUUGUUUUUGGUUUGAUUC

>TP2750

CUGGCUUACAAUAAAUGCCCAAUAAAUAUUUGUUGACCAUAUGUGUUGUAC

>TP2751

UGUAUGUAUACACACACACACACAUAUGUAUAUACACACACACAGACAUAG

>TP2752

AUCAUGCUAAUUUUUUUCUAUUUUUAGUAAAGAAGGUGUUUCGCUAUGUUA

>TP2753

CUCAUAACUUACUUAGGCUGGGUGCAGUAGCUCGCGUCUGUAAUACCAGCA

>TP2754

UCCAAAUUUUUGUUGUUCCAAAACUAGGACAACAGAUCUUCCUGUUGUCCC

>TP2755

UUCUAUUUAUUAUGUCAGGUGGUGAAAACAGGAAUGAGAACGAGGAGUCAA

>TP2756

CCCUUUCCAAACUCCAGCCUGUCUCAGGAGCUGAGCAGCCCAGAGCAGAAG

>TP2757

CCAUCAAACAAAAUACGGUGUCUGUAGUCCAAGCUACUCGGGAAGCUGAGG

>TP2758

GCUGUAAUGUAGGGGGAGGCGGGGGAGGCUUCAAACAUCCGGCCUUCAAGA

>TP2759

AGUUUUUGUGGAGCUGGGGUUUCACAAUGUUGCCCAGGUUAGUUUUAAACU

>TP2760

GUAGUGCCAGACACUCGGGAAGCUGAGGGAGAACUGAUUGAACCCGGGAGG

>TP2761

UUUUGGAAUAUAGUGGCACAAUCAUAGCUUACUGUGGCCUCGAUGUCCUGA

>TP2762

UUUUUUUCUUUUUUGAAAUUUUUUUAGAGUCAGGGUCUCACUGUGUUGCCC

>TP2763

CCAUGCCCACCGUGUCCAGCUACCCACCUCUCGCCAUCUCCCUGAAGAAGC

>TP2764

AAAAUAAAAAAUAAGGCUGGGCGCAAUGGCUUACACCUAUAAUCGCAGCAC

>TP2765

CCAGCCCCCACCAGGGGUUGUACAUACUGAACACAGAAAUGUGUUUAAGAG

>TP2766

UCAUCGAAGGGUAGACUGGCAGAGGAACUAGAAGAUGCAGACAAGGCCCUC

>TP2767

UGAGAGGACAACAUGAUGAAACUCCAUCUCUACUGAAACACAAAAAUUAGC

>TP2768

GGAGGAUUGCUUGAGGCUGAGAGGCAGAAGUUACAGUGAGCAGAGAUCGAG

>TP2769

CAGCUUAUGUUUAUAGAUUUAUUUUAGAGACAGGGUUGGGCUCUAUUGCCC

>TP2770

CAGCUCGCUGCAACCUCCACUUCCCAGGUUCAAGCAAUUCUUCUGCCAAGC

>TP2771

ACCCAUGUAGAACCUGCCAACCUGUAGCUCACACCUAUAAUCCCAGCACUU

>TP2772

ACUGGAUAAAGACAGGGAUGAUCCUACUUUUGGCCGUGAUAGAAAUCGGGA

>TP2773

GUCUCUUGAACCCAGGAGACAGGUUACAGUCAGCCGAAAUCACACUACUGC

>TP2774

AAGGUCGCACAGACUGGAGUACAGUAGUGUGAUCAUGGGUCACUGAAGCCU

>TP2775

CAGCACCCCAAGUAGCUGGAACUACAGGUACACACCACCACACUUGGCUAA

>TP2776

GAACAAACUUCUCUUUUUUUUUUUUAAGACAGGAUCUCACAUUGUCGAACA

>TP2777

AGGUUACAUACAGCCUGGGCAACAUAGUGAGACCUUAUUUCUAUUUAAAGA

>TP2778

GCCAGGUGUAGUGGUGCAUAUGUGUAGUCCCAACUACUGGGGAGGUUGAGG

>TP2779

UAUCAUUUUCUAUAGAGAAACUAGAAACAUAAUUUUCUCCAUAAUUGUGGU

>TP2780

AGUGAACCACAUUAUGGAGAAAAUUAUGUUUCUAGUUUCUCCAUAGAAAAU

>TP2781

AGUAAAUGGGUACCACACAGCUGCCAGGUGUGGUACCCAUUCCAGACGUAU

>TP2782

UCCUCUAGGUCUCCAUCCAAAAUGGAGUAAUGACACCUACUUUCGUGUUUU

>TP2783

UUAUCUGUGUUGAAGGAAUGGCUGGAGGUGCUGUGCACAUUGACUACAAUU

>TP2784

UAAAAACAAAAUUUAAAAAUUAUCUAGGAUCAAUGGCAUGCACCUGUAGUU

>TP2785

AAAUUAUCUAGGAUCAAUGGCAUGCACCUGUAGUUCCAGCUACUUGGGAGG

>TP2786

CAUCUGUUGCCAUUACUUCAGUCAUAAAAACGAGGAAGAUAGAUCCAGAAA

>TP2787

UGGAAGGCAGUGUAUUAGGGGAUUUACACUAUAGGCAGAUACACAGAUAUA

>TP2788

UCCUGGAAGCCACCUGCCUUCUUUCACAUGUAUUCCCGUCCAUCUUCAAAG

>TP2789

GAUACUAUCCGUGGUAUACAUCACAAGGGGUCCCCAUUAAUGGAGGUCUCC

>TP2790

UUUUUGCAAACUAUAUGUGUGACAAAGGUCUAAUAUCCAGCAGCUAUAAGG

>TP2791

AAAGGUCUAAUAUCCAGCAGCUAUAAGGAACUUAAACAAAUUUACAAGAAA

>TP2792

AAGGAACUUAAACAAAUUUACAAGAAAAAAGCAACCCCUAAAAAUGUGGGC

>TP2793

ACAUGUGGCCAAAAAUCAUAUGAAAAAGAGCUCAAUAUCACUGAUCAUUAG

>TP2794

AGAAUGGCUAUUAUUAAAAAGUUAAAAAAUAAUAGGUGCUGGCAAGUUGUU

>TP2795

GUCUUAUUACUGGGUAUAUACCUAAAGGAAUACAAUCAUUCUAUUAUAAAG

>TP2796

CCACAUAUAUGUUCCUUGCAGCACUAUACACAAUAGCAAAGUUAUGGAAUC

>TP2797

CUAUACACAAUAGCAAAGUUAUGGAAUCAACCUAAAUGUCCAUCAAUGACA

>TP2798

UGUCCUGUGCCAAGAUAUGGAUGGAACUGGGGGACAUUAAUCUUAGAAAAC

>TP2799

GGAAGUUAGGAGCAGGGAGAGGGUCAGGAAAAAUAACUAAUGGGUACUAGC

>TP2800

UCAGGAAAAAUAACUAAUGGGUACUAGCUUAAUACCUCAGUGAUUAAAUAA

>TP2801

GGAAGCACAUGUUUAUUUAUUCUGCAUUUUAUUCUGGAUGGAUUUGAAGCA

>TP2802

UGCAUUUUAUUCUGGAUGGAUUUGAAGCAAAGCACCAGCUUCUCCAGGCUC

>TP2803

UGGCUGACCCCAAAGAGCCUGGAGAAGCUGAUGCUUUGCUUCAAAUCCAUC

>TP2804

UGAGGCCUGCCCUCAGGGAUCUUGCAUUCCCAGUGGUCAAACCGCACUCAC

>TP2805

AUUUUUUUAAGCGUUAGCUGGGCAUAGUGGCGCACACCUUUAGUCCUAGCU

>TP2806

UUGUGUGCGUGUGUGUUUGUUUAGAAACAGGGUUUCACUCUGUCACCCAGG

>TP2807

CCAGGCACAAUGGUUCAAGCCCAUAAUUUCACUAUUUUGGGAGGCCGAGGU

>TP2808

AUGAUGCUUAUUACUGGAUCGCUUCAGCCCAUGGAACGGAGGGAGCAGUGA

>TP2809

UCCUGUCUCAGCCUCCUGAGUAGCUAGGUGCUACUAUGCCUGGCUAAUUUU

>TP2810

GCGGGGUCUGCAGACACCCUUCUCCAGCUGGAGCUGGGACUGUUCAGUCAC

>TP2811

GCCUCCCAGCGUCCUCUCCGGGCCCAGCUCUUCCUCCCGGCUGCGUCUCCA

>TP2812

CCGGUUGGUGGGCUUCUCUAGGCCCAGCUUGGGCCUCCCGGUGGCCUCUGC

>TP2813

UCUCCAGGCCCAGCUCCGGCCUCCCAGCGGCCUCUGCAGGCCCAAGUCGUC

>TP2814

AAGAAGGGGUGCAUGAACUCCCCUUAGUCCACAGGCGCCUCCCUGUGGCCC

>TP2815

CACCUGGUUAAUUUUUUGCAUUUUUAGAAGGCGUGGGUUUUUGCCAUGUUG

>TP2816

ACCAUCUUGGCUCAUCACAACCUCCACCUCCCAGAUUCAAGCGAUCGCCCU

>TP2817

UCUUUUUUAAAAAGACAAGAGUCUCAUUCUGUUGCCCAUGUUGGUGUGCAG

>TP2818

UGGUGUGCAGUGGCACAAUCACAGCAGCCUCGAUCUCCAAGGUGGGGAGAU

>TP2819

AUCAGCAGCCAAGAAGCCAGGCCCAACAUGCCCAGAGGCCAGAUUCAGAAU

>TP2820

CACGGCUGGUGGAUUGCUCGAGUCCAGGAGUUUGAGAACAGUCCGGGCAAC

>TP2821

UCCUUUUCACUUUCAAUGGUUCUAUAGUCUUCUGCCCAAUUUUUAAGUGGU

>TP2822

ACAGAAGAAAGAGAAUGAAGGGGGAAGUGCUACACACUUUUAAACAAGCAC

>TP2823

AUAGCUGGGAUUACAGGUGCCCACCACCAUGCUGGGCUACUUUUUGUAUUU

>TP2824

GUUAAGAACUUUUACCUUUUCACUUAGCAGAAAAGCCGGGUAGAUACUAUA

>TP2825

ACCAAAGUGCCCUUCAGUGGCCACUACUGGUCAGGUCAAAGAGCUAGUGGA

>TP2826

GCAGGUUGACCUACCAUGGGGUCUCACCAUGUAAGGUGACUGCAGAGAAAU

>TP2827

GUAAGGUGACUGCAGAGAAAUUAAUAGACUUACUAAAUGAAUACCUGCAAG

>TP2828

CAAGUUACUGUUACCACUUCGCCUUAUACUUUUGAGAAAGAGUCUGUGCCU

>TP2829

AUGCAUACUGAGACAUUAAUUACCUAUUAAAAAAUGGAAGCAUCCUCUAUA

>TP2830

CGAGCUACAGCUUUUCUUGCCAAUUAAUGCUGUAAUCUACGAUUUUCAUCA

>TP2831

GAUGGGUACCAUAGCUUGUGUCUGUAGUCCUGGCUACUUGGGAGUGUGAGG

>TP2832

GUGAUUGAAUCACAUCCUACACGAAAGUUUUUGGAGUGCUCCAGGCAUAUC

>TP2833

CUAAUUUUAAAAUUUCUGUAGAGAGAGACUCUCGCCAUGCUGUCCAGGCUC

>TP2834

AACCCUGUCUCUACACAAAAUGCAAAAAGUAGCCGGGUCUAGUAAUGCGUG

>TP2835

CCUUAAAUAGGGACUUGUAUGAAUGACCCCACGAGGGUUCAGCUGUCUCUU

>TP2836

CAGCUCACUGCAGCCUCAACUUCCUAGGCUCCAGUGAUCCCUCCACCGUAG

>TP2837

UGCAGUGGCACAAUCAUAGCUUACUAUGUCCUUUAACUCCUAGGCUCAAGC

>TP2838

UUCCACCUCAGUCUCUCAAUUAAUUAGGACUAUAGGCACAUGCCACCACAC

>TP2839

AUUUAUUUUUAUUCUUUGUAGUGACAGGGUCUUGCUUUGUUGCCGAGGCUG

>TP2840

AUUAUUCACCUGUGUGCUAGCCAGCAGAGAGCUGCCUCCUGGAGGGCCUGC

>TP2841

AGGCUUGGGGCAGCAGGGGCUGGAGAGGAAGGAGGGUGAGGGCACCAGGUC

>TP2842

GCUGGAGAGGAAGGAGGGUGAGGGCACCAGGUCUGCUCUUUCAUUCUGGCU

>TP2843

UCUGGCUCAUCCUCCCCUGGGCCUCAGCUUUUCCCCAUCUGUGACACUAGG

>TP2844

UCAGCUUUUCCCCAUCUGUGACACUAGGAGGCCUUAUAGACAACUGUGGCC

>TP2845

UGUGACACUAGGAGGCCUUAUAGACAACUGUGGCCCCUCCCUGUGAGUUCU

>TP2846

GCCCCUCCCUGUGAGUUCUGAUGGAAGUUAUUCAGAACCUGGCCGGACUGU

>TP2847

UCUACCGAACAUCAAAUGAAACCAAAACCAAACCCAGAAGUGCCAUACCAA

>TP2848

CCAAAACCAAACCCAGAAGUGCCAUACCAACCCCAUGUGCCUGCCGGAGUG

>TP2849

CCCUGGGCCCUUCUUCACCCUGGGAACACAGGCCCUGCCAUCUGCUGGGUU

>TP2850

CAUCUGCUGGGUUGGCCGCAUCCAAAAUAAUAUGACCGAGGCCUCCCCUGG

>TP2851

CCUUAAGAGGCUUGCUUUCUCAAAAAGUGGCCAAGGAUGUGGAGUGGGCAU

>TP2852

CUGAUCUCCAGAAAACUUUUCUUCUAGAGAUCCAGGACUGAGUGAUUCAGG

>TP2853

CUGUAAUGGAUUCACAAUUUGAUUCAACAACUGGAUUUCUAGGUAAAACUA

>TP2854

CAGCAAUUCCAUAUAUACCCAAGAGAAAUGAGCAUUAUUCCUAAUAGACAA

>TP2855

UGUGUGUGUGUGUAUGUGUGUGAGUAUAUAUAUAUUCCCAUAGGCUAGCAA

>TP2856

GCAUUGGGCAUUUCCUCCUGGCAUAAACAACUCUAAAGCUGGUAAAAUGUG

>TP2857

GGUAUGACCAAAAAGAUCAGUUGGUAAUUUCCCUGCUGCCAAAACAAAACU

>TP2858

CUACUGAGGUGGCUGAGGCACAAGAAUUGCUUGGACCCUGGAGGUAGAGGC

>TP2859

ACCGCAGCAUCAAAUUCCUGGACUGAAGCGGUCCUUUUGCCUCAGCCUCGC

>TP2860

UGGACUGAAGCGGUCCUUUUGCCUCAGCCUCGCGGAUAGCUAGGACCACAA

>TP2861

UUUUGCCUCAGCCUCGCGGAUAGCUAGGACCACAAGCAGUAGAGACAAGAU

>TP2862

CCGUAUGUUAUACAGGCUGGCCUCAAGUGAUCCUCUUACCUUGGCCUCCCA

>TP2863

AAGUCUGUUCAUUUUAACAACAUACAGGGCUGGGCACGGUGGCUCACGCCU

>TP2864

AAGUCAAGAGCUGCAAAUUCAGGACAAGACUGUACAAUAAAGUUCCGGACA

>TP2865

CAGGGGGAUCUCUCCUCUUUUAACAAACUCCAGAAAUUGAGCAUUUGUUGG

>TP2866

ACAUCUUGUAGCUGAUUUUCUCCAUACAUAUAUUCAGACCGCUUACAAAAG

>TP2867

UUAAAAAAAAAAAAUUCUAUGGGCCAGGCGUGGUGGCUCUCGCCUGUAAUC

>TP2868

UAAAAAAAUUUUCUUCGUAGAGGCAAGGUCUCUCUGUGAUGCUCAGUUUCU

>TP2869

GAUCAAGUUAUGUGUUCGGGGAGCUACUGUGCUGAUCCUGCACUGUGCAUG

>TP2870

UAAUAUCGAAAAAUAGGUUUGCUUUAGUGUGACUUGUGAAAGUUUUUAGUG

>TP2871

GAGGCUGCACGGCACGCUCGGUGCCAGCUGGGUACCCUCGGCGCUGGCUCA

>TP2872

UUCUGAGAUGUGCUGGACAAGGCCAAGAUCCCCCGGACGGACCUGACAGGG

>TP2873

CAAGUCUGGAAUUCCAGGGAAAGAAAGUUCAUUCCGGCAGGGUCCUGUGUG

>TP2874

AGGAAGGCUGAGCUUGAUGGCCCACACCUGUAAUCCCAGCAUUUGGGGAGG

>TP2875

GCUGGGAAAUAGGGAGGAAGCCUGCAAAGAUAGUAGACUUGGGUGCUGUUG

>TP2876

AUCCACCAAGAAGGCAGAGCCGCCAACUUGGGCACAACUAAAGAAGCUGAC

>TP2877

CGAGCACCGUUGACUCACAAGAUGAACAAAAUGGUGACGUCAGAAGAACAG

>TP2878

UGAACCCAUCAGAGAUGCAAAGAAAAGCACCUCCGCGGAGACGGAGACAUC

>TP2879

GGCCACCUCCUGAAGUGCUGCAAUUACAGGCGGGAGCCACUGCGCCCUGCC

>TP2880

CGACCUCCCAAAGUGCUGGGAUUACAGGUGUAUACCACCACGUCUGACUAA

>TP2881

AGACUCUGUCUCAAAAAAAAAAAAAAGGCGGCGGCGGGGAAGGUGGUCUCA

>TP2882

AGAUGAGAUUGGCAUGGCUUUAUUUAUUUUUUUUGUUUUGUUUUGGUUUUU

>TP2883

UGUUUAAAAAUUAGCUGGGUGUGGUAGUUCACACCUCUAGUCCCACCUACU

>TP2884

GCACCAGCAACUGCUGCAGCUCACAACCCCUGCCCCAGACGACUUGGUGAA

>TP2885

AACCUCUGGGCUCAGUCUCCCAAGUAGCUCAGACUACAGGUGGGCACUACC

>TP2886

AAAAGUUGCUAGAAAUUAAUAAUGCAACUUUUUUUUCCUUUUACCAUGUAA

>TP2887

CAUGUUAUGAAAUGACAGAAAUGAAAAGGAACCAAAUCCAGAAACUUGAGA

>TP2888

CAAAAAAAAAAAAAAAAAAAAAAAGAGAGAUCCAGAGGUAACUUUACAGUU

>TP2889

UACGUAUAUACACACAUAUAUGUAUAUACGUACGUAUAUAUGUAUUUUUAU

>TP2890

CAUACACACAUAUAUGUAUAUACGUACGCAUACACACAUAUAUGUAUAUAC

>TP2891

UAUGUACAUGCAUGUAUGUAUGUAUAUACGUACGCAUACACACAUAUAUUU

>TP2892

AUUCACAGCAUUGGGAAAAAAAAAUACAUACAUACAUAUACAUGUAUGUAU

>TP2893

GAAGACAAACUGCAUAUGCUUCCUUAAAACAAAAUCUACAUCGAGCUUCAA

>TP2894

GAAGAAACCAGGGAAGAUGAAGACAAACUGCAUAUGCUUCCUUAAAACAAA

>TP2895

AAUUAAGACAUUAAUUAAUUACUUUAGAAAAUUAUCACUCUCCUUAUAGAA

>TP2896

ACUGUGUUCAUAACCCACUUGCAUUAGAUUUGCUUGGUUGAAUGUCAUUUU

>TP2897

UCUGGUUUCUUCACAUACAGUUGUAAAUUUCCUCACAAUUACUUGGGUUGA

>TP2898

AUGGUUGUUUUGAGAAUUAAUGGUAAGAAGCAUGUGAAGAUAAUUUUAAAC

>TP2899

ACCACCUUGUCCGGGUAAUUUUUGUAUUUUUAGUGGAGACAAGGUUUUACC

>TP2900

AAAGAUUCUUCGUGUUUUUUCAGUUAGCCCCUGUAUUUCCUUUCCCCAAUA

>TP2901

AGGAGUGUGUGGGAAAAACAGUUUUACUGGGGAAAGGAAAUACAGGGGCCA

>TP2902

UCAUAAUAAAAUAUUUAUUCACACUAAACACCUAGGAGUGUGUGGGAAAAA

>TP2903

CAUUUGAACCCAGAUAGCUUUUACAAAUGAAGAUAAAUAAAAUUCAUAAUA

>TP2904

GUCGCCUGUGAAAAGUGUGUUUGUUACAGCUCACCUAACCAAAAAAUAUAA

>TP2905

GGAGAAUAUUAUUUCAAAUGAGUUUAUAUUUUUUGGUAACUAGGUGAGCUG

>TP2906

AAUCACUUGUGUCCAAGAGUUUAGGAGUUUAAGACUACCCUGGGCAACAUA

>TP2907

CCACAGGGUAGAGUGGCUCAGAAAGAGGAAAUAAUCUGCUGCUAUUUAUCC

>TP2908

CAAUAUGGAGUUUCCUCAAAAAACUAGAAAUGUAAUUACCAUAUGACCUAG

>TP2909

CUUUUUAUUGACAAGAAAUAGAAAUAUACCAUUUGACUCUCAAUUUGGAUA

>TP2910

UACCCAUGGAAAAUAUAGACUUUUUAUUGACAAGAAAUAGAAAUAUACCAU

>TP2911

UGGUAGGCUUGCUUUAAGUUUUUGCAGGGAGUCUUGCUGUGUUGCUUAGGC

>TP2912

UAUUAAGAAAUGCCCCUUUGGCGCCAUAUCAAUUGACAAUUUACCAAGCAA

>TP2913

GGCAGGAGGACUGCUUGUGCCCAGAAGUUCGAGGCUGCAGUGUAGCUGUGA

>TP2914

UCUUUACUCUAUUAAAUCUAGAAUUAGCCGGGCAUGGUCGCUCAUGCCUGU

>TP2915

ACAUCCCAGUUUAGUGACAUAAUUUAUUUCGUUCUUACGUGAAACCAGACA

>TP2916

UGGGACCUCAACACUCUCAAGUAUGAGGUUUCUAGGGCAACCUCUGGACAC

>TP2917

ACAUAAAAAAUUAACCAGGUGUGGUAGCACAUGCCUGUGGUCUCAGCAACU

>TP2918

UCCCCCAAAAAGAGAAAGUCACCCCAGGGUAUGACCUAAUUCAGGAAAGGU

>TP2919

CCAGGUCCACUCACUGAAAUUCCAUAAAGACUGGAAUUUGUUGCCUUGGAG

>TP2920

UUGAAUAUCCAAAAUACUGAAUACUAGAUAUUCAAUAUUUCUGGGUUGGGU

>TP2921

AGGUUAUAUGUCCAUAUCAGGCCCAACCCAGAAAUACUGAAUAUCCAGUAU

>TP2922

GCGAGAAAUUCAAAGAAGACCUGAAACUCAUUCUUUCACGCCCUCCUUAGA

>TP2923

UCUCUGGGAAGAGUUGGUAAAUAUUAGGUUGGUGCAAACGUAAUUGUGGUU

>TP2924

UCCGAAGUGUUAGGAUUACAGGCGUAAGUCACCAUGCCCAGCCAAGAAAGC

>TP2925

GUUAGUCAAUUAUUUUUCAUGUGUGACUAGCCCAAUUUAAAAUAUAGGUAA

>TP2926

UCCCAGGGCUUUGGGAGGCCUUGGCAGGUGGAUCACCUGAGAUCGCACGUU

>TP2927

AUAUAAUAUGUAACAGGUUGUUUAUAGAAGCCAUAGCUGGUUGACUUCAAA

>TP2928

UGUUUUGCUUUUGGUUGGGAUUAUUAUCAAUGAUGAUGAACUGCAGAGAAA

>TP2929

UACAAUCCAUUUUCUUUGUAGGUGCAGGUAUUUGGACUGUAUUCUGGAGAA

>TP2930

AAAGAGAAACCCUGUAAAAAAAAUUAGCCAGGCUUGGUGCCACACACCUAG

>TP2931

UUGGCUUUAACCCAUCCUGGCUAACACAGUGAAACCCCGUCUCUACUAAAA

>TP2932

CUGCUGUAAGAAACAGUUCUUUGUCAGUUUCAAGGACAUCGGCUGGAAUGA

>TP2933

AAAUUUAUAGUACCGUAAACAUCUCAGGGCCUCGAGUGAUAAGUAUGGCUG

>TP2934

UUCAGCGGACUUUCUCUUCUGACAUAUUCCUCUUCUACUAUCCAGGAACUG

>TP2935

AAUUUUAGUUUUCGUAUGUUAAUAAAAUACUAUAGUACAACAAAAGGGCUG

>TP2936

UCUCACCUCAGGCACCCAAGUAGUUAGGACUACAGACAUGCAUUAGCAUAC

>TP2937

UACUCAUCCCUCCUCAUGUCCCUGUAUGGGCCACCAAAAUGAUGCAGGAUU

>TP2938

AGCUGCAUGGGGCCCUUGGGCCAUGAGCCCCAGCCCCAGCUGGGAGGGGCA

>TP2939

UCCUGGCCUGGUGGAGAAAAGGAAAAGAAAAACAGCGUAAAUGCAGGGCUU

>TP2940

UCCUCGCCGUUCUAAGUAAUAGCAAAAACUGCAACUACUUUUGCACCAACC

>TP2941

GAAGGCAUUUAUUUAAAGGAGCCCCAGGACUCAGGAUGCAUUUGAAAGAUG

>TP2942

CCCUUGGGGCUGAUACCAUGCUCAGAGCACAGCUGUUUCCAGAACUCGAAC

>TP2943

UUCUUCCUGGCCCUCGGGUUCAUGCAACGGCGUAACUGGAACCGACGCUUA

>TP2944

CCAAGCCCUGCAGCUCAUGGAACAGAAGAAGUGGAACCGACGUUUCUGGAA

>TP2945

UAAUUUUGUAAAGGUGUGAUAAGAUACUGCACUUGGCUGGGCACAGUGGCA

>TP2946

AACAGGGACUGUGGAUCAUAUUGGUAGAAUAACUACUGGCCCUACAUAGUC

>TP2947

CAGGUUUCUGACAGGCAGUCCGGUUAGGGAGCCCUACAGCAACCCGCCGGU

>TP2948

ACUGGACGUGGUGGUGCGUGCCUGUAGUCUUAGCUUUCCAGGAGACUGAGG

>TP2949

UACGUACAUACAUACAUACACGCUAACUGGACGUGGUGGUGCGUGCCUGUA

>TP2950

UCAAAACAAACAAAAAAAAGUCUCUACUAAAAAUACAGAAAUUAGCCAGGC

>TP2951

AGCCACCGCGCCUAGCCGAAACCCCAUCUCUACUAAAAAUACAAAACUUAC

>TP2952

GCUGGUUGGAAGAAUCUUUAUAUUUAGAAAUAGGAUGGUGAGUGGUUUGUU

>TP2953

GGUGCAACCGAUCGCACUGUCGCGCAGAAGCUCCUCAAUGGCCAGCGCCAG

>TP2954

GCCCCGUGGGGUAGGAGGGACAGAGAGACAGGGAGAGUCAGCCUCCACAUU

>TP2955

CCAAGGACAUGACCAGCAGCUGGCUACAGCCUCGAUUUAUAUUUCUGUUUG

>TP2956

CAGUGUCGCCCUUCCAAAGGCAGGAAGCGGGGCUUCUGCUGGUGUGUGGAU

>TP2957

GCACACUCAGCGGGCCUAGGCACGCAUGGCUCUUGUGUUGCCUUAGCUGAA

>TP2958

UCCUCCGACUCACUGGCAUUUCCCUAGAAAGUCCAAGUGAGAAGAAGGCAU

>TP2959

AAACUGGCCAUGUGGUCUUCCUCCGACUCACUGGCAUUUCCCUAGAAAGUC

>TP2960

CACUCGGUCCAUGCUGGGGAUGGCCAGUCUCUCCAAACUGGCCAUGUGGUC

>TP2961

UGUCUCCCUUGGGCUUUUCAAGGAAAUGCUAGUGAGUGGGGGGAUGACUGC

>TP2962

AGGUUGCAAUAUUAAGAUUUUGAAAAGGCUGUUGCUAGAUGUUGGUGACUC

>TP2963

CCACUAUGGUCAGAAAUGAUACUUGAGGCCAGGCGCGGUGGCUCAUGCCUG

>TP2964

AACGCACUUCACAUGGUUUAACCAAAUUGCUUUGGAAAAAGGCGGUCCAGC

>TP2965

UUGGUAGGCUAUUAAUUGCUGCCUCAGUUUCAGAACUUGUUAUAGGUCUAU

>TP2966

UUGGUAUCAGGAUGAUGCUGGCUUCAUAAAAUGAGUUAGGGAGGAUUCCCU

>TP2967

UGUUGUGUCUCUGCCAGGUUUUGGUAUCAGGAUGAUGCUGGCUUCAUAAAA

>TP2968

GAAUUUUGUCGAAGACCUUUUCUGCAUCUAUUGAGAUAAUCAUGUGGUUUU

>TP2969

AAUACCUAGUUUAUUGAGAGUUUUUAUCAUGAAGGGCUAUUGAAUUUUGUC

>TP2970

UUUGAGAUAUGUUCCAUCAAUACCUAGUUUAUUGAGAGUUUUUAUCAUGAA

>TP2971

CAUAAAUAGCUCUUAUUAUUUUGAGAUAUGUUCCAUCAAUACCUAGUUUAU

>TP2972

UAUUGGCUGUGGGUUUGUCAUAAAUAGCUCUUAUUAUUUUGAGAUAUGUUC

>TP2973

CCAGCUUUUGCCCAUUCAGUAUGAUAUUGGCUGUGGGUUUGUCAUAAAUAG

>TP2974

CAUCCUUGUCUUGUGCUGGUUUUCAAGGGAAAUGCUUCCAGCUUUUGCCCA

>TP2975

CUGUGUUGAAUAGGAGUGAUGAGAGAGGGCAUCCUUGUCUUGUGCUGGUUU

>TP2976

GAUUGCCUUGGCCAGAACUUCCAAUACUGUGUUGAAUAGGAGUGAUGAGAG

>TP2977

CUAAUUGAAUACGCUUUAUUUCUUUAUUUCUUUCUCUUGCCUGAUUGCCUU

>TP2978

AUCAUGCUGUCUGCAAACAGAGACAAUUUGACUUCCUCUUUUCCUAAUUGA

>TP2979

GACAAUGGGGUUUUCUAAAUAUACAAUCAUGCUGUCUGCAAACAGAGACAA

>TP2980

UUCCUAGGUAUUUUAUUUUCUUUGUAGCAAUUGUGAAUGGGAGUUCACUCA

>TP2981

CUUAUUUCCUUGAGCAGUGGUUUGUAGUUCUCCUUGAAGAGAUCCUUCACU

>TP2982

UGAUAGGGACUGCAUUGAAUCUAUAAAUUACUUUGGGCAGUAUGGAUUUUC

>TP2983

CAAUUCUGUGAAGAAAGUCAAUGGUAGCUUGAUAGGGACUGCAUUGAAUCU

>TP2984

UUUUUUGGUUCCAUAUGAAAUUGAAAGUAGUUUUUUUCCAAUUCUGUGAAG

>TP2985

CUUUUUGCUUAGAAUUGUCUUGGCUAUGUGGGCUCUUUUUUGGUUCCAUAU

>TP2986

AUAUAUCUGUUUUGGUACCAGUACCAUGCUGUUUUUGUUACUGUAGCCUUG

>TP2987

UCUGUUCUGUUCCAUUUGUCUAUAUAUCUGUUUUGGUACCAGUACCAUGCU

>TP2988

UUGUAGAUGUGUGGUGUUAUUUCUGAGGUCUCUGUUCUGUUCCAUUUGUCU

>TP2989

CUUAUUUUUGUCAGAUUUGUGAAAGAUCAGAUGGUUGUAGAUGUGUGGUGU

>TP2990

AUAGGGAAUCCUUUCCCCAUUGCUUAUUUUUGUCAGAUUUGUGAAAGAUCA

>TP2991

CAGUUUUCUCAACACCAUUUAUUAAAUAGGGAAUCCUUUCCCCAUUGCUUA

>TP2992

CAGUUUCAGCUUUCUGCAUAUGGCUAGCCAGUUUUCUCAACACCAUUUAUU

>TP2993

UGUAUAAGGUGUAAGGAAAGGACCCAGUUUCAGCUUUCUGCAUAUGGCUAG

>TP2994

UCCAUCUUGAGUUAAUUUUUGUAUAAGGUGUAAGGAAAGGACCCAGUUUCA

>TP2995

CUCCCGUUCUGUAGGUUGCCUGUUCACUCUGAUGAUAGUUUCUUUUGCUGU

>TP2996

AAGUUCUUUGUAGAUUCUGGAUAUUAGCCCUUUGUCAGAUGGAUAGAUUGU

>TP2997

UAUCAAUGAAUGAAUGAAGAAAGAAAAUGAGACAUACUUACACAAUGGAAU

>TP2998

UGUUUACUGAAGCACUAUUCACAAUAGCCAACAUACGGAAUCAUCUAAGUG

>TP2999

AAUGAGAUAUCACCUUACCCCACUUAGAAUGACUAUUAUUAAAAAGACCAA

>TP3000

UCAGGGAAAUGCAUAUCAAAACCACAAUGAGAUAUCACCUUACCCCACUUA

>TP3001

AUAUACAAGGAAUUCAACAGCAAAAAAAAAAUCCCUUUUAAAAAGUAGGCA

>TP3002

AAACUAUUUAUCCAACAAGGGACUAAUAUUGAGAAUAUACAAGGAAUUCAA

>TP3003

UGAAGAGACAAUCUGUAGAACAGGAAAAUAUGUGCAAACUAUUUAUCCAAC

>TP3004

UUCUGCAAAACAAAGGAAACAGUCAACAGAGUGAAGAGACAAUCUGUAGAA

>TP3005

UAUUAAGCUAAAAAGAUUCUGCAAAACAAAGGAAACAGUCAACAGAGUGAA

>TP3006

UAGACAAAUGGGACUAUAUUAAGCUAAAAAGAUUCUGCAAAACAAAGGAAA

>TP3007

GACCUGAAAUUAUGAAACUACUGGAAGAAACCACAGGGGAAAUGUUUCAGG

>TP3008

ACCAUACACAAAAAUUAAAAUGAUUAAAGACAUAAAUAUAAGACCUGAAAU

>TP3009

UAUUCAUCUGCAAAAUAAUGAAACUAGACCCCUAAUGUCUCACCAUACACA

>TP3010

UCAGGGAAGGGAUACCCUCUUCGAUAAAGGGUACUGAGAAAACUGAAUAUU

>TP3011

GCAUAGUAUUGAUAUAAACAGACACACAGACCAAUGGAACAGAAUAGAAAA

>TP3012

AUUUGUCACAGAAAUUGAAAACAAAAAUACCAAAGUUCAUAUGAAAUCAAA

>TP3013

UUUACCCAAAGUAAACUUGAUUCAAAGCAAUCUCUAUCAAAAUACUAUUGU

>TP3014

UAAUGAAAGAAAUAAACAAAUGGAAAGACAUUCCAUGUGCAUGAAUCAGAA

>TP3015

CCUCGGAUCUAACCAAAAAGGUGAAAGGUCUCUACAAGGGAAACUACAAAA

>TP3016

AAAUAAAAAGCAUCUAAAUUGGAAAAGAGUAAGUCAAAUUGUCUCUUUCUG

>TP3017

CACUCUUAUUCAACAUAGCACUAGAAGUUCUAGCCAGAGCAAUCAGGCAAG

>TP3018

CAUACCUCAACAUAAUAAAGGCCAUAUAUGACAGAUUCACAGCUGACAUCA

>TP3019

UUCAUGAUAAAAAAAAACUCUCAACAAAUUAGACAUACAAGGAACAUACCU

>TP3020

UGACAUCAGUGGAAUGAAGGAAAAAACUUUAUGAUUAUCUCAAUAGAUGCA

>TP3021

UGCAAGGAUGGUUUAACACAUGCAAAUCAAUAAAUGUGAUGACAUCAGUGG

>TP3022

CAAGUGUGAUUUACCCAAAGAUGCAAGGAUGGUUUAACACAUGCAAAUCAA

>TP3023

CAAGCUGAAUUCAACAGUACACCAAAAAGAUAAUAUACUAUGGUCAAGUGU

>TP3024

AAAAAUCAUCAACAAAAUGCUAGCAAGCUGAAUUCAACAGUACACCAAAAA

>TP3025

CAGAGAAGGACACAACGAAAAAGAAAGCUACAGGCCAAUAUCCCUGAUGAA

>TP3026

GGAAUUUUUCCUAACUCAUUCUACAAGGCCAGCACAACCCUGACACCAAAA

>TP3027

UCACAAACUGUUUAAAAACAAUUGAAGAGGAGGGAAUUUUUCCUAACUCAU

>TP3028

AAUGGAUAUUUGGGCUCAGGCCUGUAAUCUCAGCACUGUGGGAGGACAAGU

>TP3029

AGAGAUUGUAGUGUGUUGAGAUUACACCACGGAACUCCAGCCUGGACAGAG

>TP3030

CAAAAUCCCAUCGCUUGAACCCAGAAGGCAGAGAUUGUAGUGUGUUGAGAU

>TP3031

UGCUGGCCAAAACAGAAUGUUGGAGACACACACUGAAUUUACUUGGAAGGC

>TP3032

UAAUUUUUUGAUGUUUUGUAGAGACAGUGCUUCAGUUUGUUGCCUGUGCUG

>TP3033

UGGCGUACACCACCACGUCCAGCUUAUUUUUUGUAUUUUUAGUAAAUACGG

>TP3034

UCAAACUUAUGACCUCAGGCAAUCUACAUGCCUUGGCCUAUGAAAGUGCUG

>TP3035

ACUGCUGCACUCCAGCCUGGGUGACAGAGCAAGACCCUGUCUCAGACAUGC

>TP3036

UUUGGCCUUCCAGAGUGCUGAAAUUACAGGUUUGAGUCACUGUGCCCGGCU

>TP3037

CUGCAGCUUCCACCCCUGGGGCCCAAGCCGUCCUCCCACCUUAGCCUCCCA

>TP3038

GGUAUAAAAACAAUAGAUUUCAGCCAGGCGUAGUGGCUCAUGUCUAUAAUC

>TP3039

AUUUUACUUCAUUUUUUCUUUUUAUAGACACACGGCCUCACCAUGUUGCCC

>TP3040

UUCCCGAGUAGCUGGGAUUAUGAGUAUGCACCACCACACGCAGCUAAUUUU

>TP3041

CACCCAGGCUUGACUAUAGUGCCAUAAUCAUGACUCACUGUAGCCUUGACC

>TP3042

GGCCACAAAUUCCUGGGCCCAAGCAAUCCUCCCACCGCAGCCUCUUUGAAG

>TP3043

CCCACCACAAGGAUGACCCUCCUCCACCUGAAGAUGAUGAGAACAAAGAAA

>TP3044

UCCUCUACCAAAUGUGAAUGCAGCAAUAUUAAAAAAGGUCAUUCAGUGGUG

>TP3045

UUGCUGUGGGCCAUGGACAGGCAGAAGGAAGCGCCUCCUCAUGGCAGAGGC

>TP3046

UUAAAAAGGGUCAGGAGGCCGGGCAAGGUGGCUCAUGCCUGUCAUCCCAGC

>TP3047

AGGGACUGGCCUUGGCCUAGGGAACACUAGCUGUCCAGGGAGGGGGAGCUU

>TP3048

ACAGGCUAAGAGGCUGUGUGUGAAAAGCCCGCAGCACAGGAUCUGGCACGC

>TP3049

CAUUCCGGCAGCAUUUCUGGGGGCUAUUCUCCUUCCCCCCACUGCACAACC

>TP3050

CCCUGCUGUUGCUUCUGAGGUUUGAAAACCGCUGCCUCAAUGCUCCUGAUU

>TP3051

AUUCUUGUGCCUCAGCCUCCAGAGUAGCCAGGAUUACAGGCACCCGCCACC

>TP3052

CAGCCUGGCUCCUGUUUUUUGAGACAGGGUUUCACCCUGUUUCCCAGGUUG

>TP3053

AGCUGGUUUUGAGCUUCUGGCCUUAAGCAAUCCUCCUUACUCAGCUUCCCA

>TP3054

AAAGAAACCUUUUUAAAUAGAGACAAGGUCUCACUGUGUUGCCCAAGCUGG

>TP3055

GAAUAAUUAAGGCAGCUUUAAGGCUAAAGUCAAGAGGGGGGUUGGCUAGAU

>TP3056

GAGAUGCGUCCCACAUCGCCUGGCUAAUUUUUGUAUUUUUGUGGAGAGGGG

>TP3057

CUCAGCCUCCUGAGUAGUUGGGAGUAGAGAUGCGUCCCACAUCGCCUGGCU

>TP3058

UGUAUUUCAGACUGGGAGACAGAGUAAGACCCUGUCUGGAAAAAUAUAUAA

>TP3059

GUAUUUUUAGUAGAGGCAGAGUUUCACCAUGCUGGUCUCAAACUCUUGACC

>TP3060

UCUGAAUUUACCCUCUCCAAGUUUCAUUAGCCGAGGAGUUUUCCUAAAGUA

>TP3061

CUUGGAACUCCUCAAGAACCUGAAGAUUCCAGUGGUCAGUGUCGGUGGGGG

>TP3062

CUUCAGCCUGGGCGACAGGAACGAAAAAAAAAAAAAAAGAAAAAACAACCU

>TP3063

AACAAAAUAAAAUAAAAUAAAUACAAAUUGGCCGGGUGUGGUGGCAGACAC

>TP3064

AGUUCAAUAUACCUAUACCAAAUCUAAUGUCAUAAAAUUUUCCCCCAUUUG

>TP3065

CCGGGCUUGGUGGCUCAAUCCUAUAAUACCAGCACCUUAGGAGGCCAAGGC

>TP3066

CCCUUCCUUCCUUCCAUUUUGAGAUAGUGUCUCGCUCUUGUCACCCAGGCU

>TP3067

GUCCAACAUGUUUAAAUCCGUCUCUACUAAAAAUACAGAAAAAAUGAGCUG

>TP3068

CCAGGCGCGGUGGCUCAUGCCUGUAAUCCUACCACUUUAAGAAGCAGAGGC

>TP3069

AAAAAACUUUUUUUUUUCACCAGAUAGUGCUGGGAAAACUGGAUAUCCACA

>TP3070

UUCCUGAUUUCAAAACUUAUUAUAAAACUAUAGUAAUUAAAACAGUGUGGU

>TP3071

CAAGGGACUCUGAGUAGCCAAAACCAUCCAGAAAAAGAAAAACAUAGCUGG

>TP3072

UAAAAUUAUCCUCGUGUGGUGGCAUACACCUAUAGUCCCAGCUGUUUGAGA

>TP3073

AAAGUACGAAAAUUCUCUGGGCGUGAUGGUGUGUGCUUGUAGUCCCAGCUA

>TP3074

UGUCUCACUAUCUCACCUAGGCUGAAGUGCAGUGGUGUAAUCAGGGCUCAC

>TP3075

GAUCUUGUCUCUGCUAAAAAUAAAAAAAAAAUAGCCAGGUAUGGUGGUGCA

>TP3076

GGCUCACAGUUCUGCAGACUAUACAAGUAGCAUGGUGCCAGGCUGGGAGCA

>TP3077

GAAUUCCUGUGCACUAGGGAUCCUCAGCUUCCGAAAGUGUUGGGAUUUUAG

>TP3078

CUUGUUGUUUAUUUCAAUGGAAACAAGAUGUUUCUGUAAAGAUUCCUGAAU

>TP3079

CAUUUCAACUCUCUGGAAGGUGCCAAGCCAGAUCUCUGAUUUGUGUGCUAC

>TP3080

GCACACAAAUUCAGAGAUCAGUCUUAGCACUUUGUAAGGAGAUAACAUUUC

>TP3081

AUUCGGUUUGCCAGUAUUUUAUUGAAGAUUUUUGCAUCGCUGUUCAUCAAG

>TP3082

UGAAGCCCACUUGAUCAUGGUGGAUAAGCUUUUUGAUGUGUUGCUGGAUUC

>TP3083

GGGUGAUGUUUAUUGAUUUGCGUAUAUUGAACCAGCCUUGCAUCCCAGGGA

>TP3084

UGGCCUUUUCUGCAUCUAUUGAGAUAAUCGUGUGGUUUUUGUUGUUGGUUC

>TP3085

GCAUGAAGCGUUGUUGAAUUUUGUCAAUGGCCUUUUCUGCAUCUAUUGAGA

>TP3086

CAUCCCAUCAAUACCUAAUUUAUUGAGGGCUUUUAGCAUGAAGCGUUGUUG

>TP3087

CUGACAGCUCUACCUAGUGCUAUCUACAAUAUUUUUUCACUGAAGGAGAUA

>TP3088

UUGGCAGGCCAAGGCAGGAGGAUCAAUUGGGCCUAGUUUGAGACCAAAUCA

>TP3089

UUUUUAAAGGCUAUAGUCCUCCGUUAUUGGACAUUUAGAAUAUUUCCCAAA

>TP3090

CAGGAUGUGUACACUCCCUAGGGAUAUUGUUCCUAAUAUCCAGGGACGGAG

>TP3091

AAUAUCCAGGCGGCGAGAGGAUGAUAUUAGUCUGAAUAUUGCAGGAUGUGU

>TP3092

ACACCCUUGUGAUAUUGAUCCUAAUAUCCAGGCGGCGAGAGGAUGAUAUUA

>TP3093

AACAUCCAAAGGUGGAGAGGAUGAUAUUUCUUCCAAUUUCGCCGGGGGUGC

>TP3094

CUGUGAUACUGUCCCUAACAUCCAAAGGUGGAGAGGAUGAUAUUUCUUCCA

>TP3095

GGGAGCGGAUGGUAUGACUCCCAAUAUCGCAGGGGGCAGAGACCUCCCCUG

>TP3096

UUAUUGAAAAUAGCGCAGUGGGUGUACAUCCCUUCGGUCAUCUUGUUCCUA

>TP3097

UAAUAGCCGGGGGGUAGAGGAUGACAUUAUUGAAAAUAGCGCAGUGGGUGU

>TP3098

ACACCCCUCUAUGAUGUUACUCCUAAUAGCCGGGGGGUAGAGGAUGACAUU

>TP3099

UUACUCCCAAUAUCGCAGAAAGUGUACACCCCUCUAUGAUGUUACUCCUAA

>TP3100

CGUGGGAGAGAGGAUAUUACUCCCAAUAUCGCAGAAAGUGUACACCCCUCU

>TP3101

AUCCAGCUAGCAUAUUUUCAAUAAUAUCACAGAAGGAACACACGUGUGACA

>TP3102

AAUAGGAAUAAUAUACUUCUACGAUAUGACAAAUAAUAUCACAGUUUGUAC

>TP3103

CCUACAACAUCGGGAAUAAUACCACAGGGUGUACACCACCUGUGAUAUUAG

>TP3104

AGGAGAAACCUCCCCCGAGGAUAUAACGAACAAAUACAGAGGAUGUACACG

>TP3105

AUCACAGAAUGUACACGCAUGGUGUACACCCACUGCGACAUUAAAAGCAAU

>TP3106

AGCGAGACAGGGCACUGUGAAUCACACCUGUAAUUUCAGCAUUCUGGGAGG

>TP3107

CCCUGCCUUGGCCUCUCAAAGUGCUAGGAUGACUGGCCUCACCCAGCCCAG

>TP3108

CUUCCAAAAGAUGACCGUGACCUUGAACUUCCUGGGGAGGUAAGUGAGCUC

>TP3109

UCCACUUAUUGUGUACUUUAUUAUCACAUUGUAAUGGAUAAUGAAACAAUU

>TP3110

UGCAACCGCCGCCUCCCGGGUAGCUAGGAUUACAGGCGUGUGCCACCACGG

>TP3111

CGGGAGGUGUCGGUGGCAGCCUCUGACUAGGCUGUUUCUGACAGUGUAGUG

>TP3112

CACUGGGGAAAAGCCCUAUGAAUGUAAUGAGUGUGGGAAGGCCUUCAGCCA

>TP3113

UCCCACCUCUGCCUCCUGAGUAGCUAGAACAACAGCCCUGUGCCUGCAUAC

>TP3114

AGUUCAAGACCAGCCUGGACAACAUAGUGAGACUCUGUCUCUGCCCCUGCC

>TP3115

CCCCAUCUCAGAUACUGUCAACUCCAUCCUUCUGUCAGGCCAAAAUCCUUG

>TP3116

CUUUGAGGACCCCUGAGAGUUUCUCAGACACCCGGGGUGGGGGAAGUGGGG

>TP3117

CUGGACUAUUGAUCCUCCUGUCUCCACCUCCCGAGUUGCUGGAACUACAGG

>TP3118

UUAAGAGACACGUGUUUACUAUGUUACCCAGGCUGGACUCCAACUCCUGGA

>TP3119

AAUUGUUGAUUUUUUUUUAAGAGACACGUGUUUACUAUGUUACCCAGGCUG

>TP3120

UUCUCCUCCAGGUGAGGAUACAACUAGAAGCCAGCAGUCUACAGGCUGGAA

>TP3121

CUCCCUCCAGAGCUGCCCUCCUCGGAGCACCUGAGUGUGGCGGAUGCCACC

>TP3122

AAAGCACCCCUCCCUUCAUGGUUUCAACCUGGCAUGCAGGCACUUAAAUUU

>TP3123

CAGGCUGGAAGACAGAGCGAGACUCAAUCUCUUAAAAAUAAAAAAAAUAAA

>TP3124

UGACAUCAGGCGAUCCACCUGCCUCAGCCUCCCGAAGGCUUCUGUCCUUCA

>TP3125

UAAAAAAAAGAUAAUAAAAUACUUUAGGAGGCCAAGGCAGGCAGAUCACUU

>TP3126

UGAACUGGUUUCAGGUGAUCCACCCACCUAACCUUGCCAAAGUGCUAGGAU

>TP3127

UACGCCUGGUGCCUGGUGCGGAGCAAGUACAAUGAUGACAUCCGUAAAGGC

>TP3128

GAGAAAAUCCUAUUAUCAAAUUUGGAUUUCCUGGCCCCAGAACUUCCCAAA

>TP3129

AUCACAGGUGCACACCAUCAUGCCCAGCUAAUUCUUUUAUUUUUAGUGUAG

>TP3130

GCCAGGUGUGUUGGCAUGUGUCUGUAGUCUCAGCUACAUGUUAGGUUGAAG

>TP3131

GGAGACCCCGUCUCAACUAAAAAUAAAACAUUAGCCAGGUGUGUUGGCAUG

>TP3132

GGAUCGCCUGAGGCCUGGGCAAUAUAGGGAGACCCCGUCUCAACUAAAAAU

>TP3133

UAAUCCCAGUGCUCUGGGAGGUCAAAGCGGGAGGAUCGCCUGAGGCCUGGG

>TP3134

CAGGGAGGCUCAUUCCUCUAAUCCCAGUGCUCUGGGAGGUCAAAGCGGGAG

>TP3135

ACGGUGCACACCUCUAGUCCCAGCUACUCGGGAGGCCAAGACAGAAGGAUC

>TP3136

UGGCCUCCGAAAGUGCUGAGAUUACAGUUGCGAGCCACUGUGCGUGGCCAG

>TP3137

ACUUUAGCUGAAUGUGGUGGCUUACACCUGGAAUCCUAGCACUUUGAGAGG

>TP3138

GGUGCGGGUUUCACCGUGUGGCCCAAGCUGGUCUUAAACUCCUGACCUCAA

>TP3139

UGAGGUAGGAGGAUGACUUGAGCCUAGGAGUUCGAGGCUGCACAAUUAUUG

>TP3140

GUGGAUCGUUAGAGGUCAGGGGUUCAAGACCAUCCUGGCCAACAUGGUGAC

>TP3141

UGCCUGUAAUCCCAACAUUUUGGGAAACCAAUUCAGGUGGAUCGUUAGAGG

>TP3142

GGGAGUCUGGAGCCCAUUCCCUCACACUGGUACUCACUGCAGCUGGGGACA

>TP3143

UGGAUUCCUGUAAUCGCAGCUACUCAGGAGAGGGCGCUGGAGAAUCACUUG

>TP3144

AUCCUUUAUCAGAAAGACAAAAAAUAACAUGCUUAUGAGGAUGCAGAGAAA

>TP3145

GUCUCAUUAAAAAAAAAAAAAAGAAAGAAAGAAAGAAAAGAAAUUCAGCUU

>TP3146

GUGUUUGCAAAGUUUUACUAUACGAAAGAAUAGCAUUGGCCGGGCGUGGUG

>TP3147

CACGGUCUCCCCUGCCUUCAGAAUCAGAAGAAAGCAAUCCAUUUUACAUAU

>TP3148

UCCCUUGUUAUCUCUUCUCCCUAGUAGGAAGCUGAGCCCACUAGGGAAAGU

>TP3149

UCCACCGUCAUCGUCUCCAGUAGCUAGGAAAGGCAUGUGCCACCAAGCCUG

>TP3150

CGUAUCUCACUCUGUGGCCCAGGCUAGAGUUCAGUGGCAGGGCAGUCAUAG

>TP3151

CUACCAAAAAUGCAAAAACUAGCUGAGUGUGCUGGUGGACGUCUGUAAUCC

>TP3152

UCUUGCUUUGUUGGCUGACAGUGGCACAAUCAUAGCUCACUGCCACCUCAA

>TP3153

UUUGGCCAUUUUGAGUAAUGCCACUAUGAACAUUGGUGUCAUAUAUUUUUA

>TP3154

UCCAUCUCAGGGGAAAAAAAAAAAAAGGUUUUUUUUGAGAUACCCAAAAUU

>TP3155

AAUAACUGAUGUCAUGCAGCUAGUAAGGAGUGAAGUGGGAAUCCAAACAUA

>TP3156

CUGUUCAUUGAAACAUAAAGAGGUUAAAUAACUGAUGUCAUGCAGCUAGUA

>TP3157

CUCAUCCUCUUGAAUGGGUGGGAUUACAGGAGCACGCCACCGCACCCAGCU

>TP3158

CUCAUUCUGUUGCCCAGGCUGGAGUACAGUGACGCAAUUUUAGGUCACUGC

>TP3159

CUGCUCGAAGGCCCUAAGAUUGGAUAGCUCUGGAGAGAAGUGAAUUAGGGC

>TP3160

AUUGAGGGUUAUGGAACAGUUUCUAAGACAGUGAUGUGUAUUCUGAGAUCU

>TP3161

UCAGAGUACACAUCACUGUCUUAGAAGCUGUUCUCUAUCCCUCAGCCUAAU

>TP3162

CUUUUUGAACAUCGACAUGAUGCUCAAAAGAAAAUGCUCUUUGGAGCAUUU

>TP3163

GGGGUCUCGUUUAGUUGCCUACGCUAGUCUUGAACUCCUAGCCUCAAGUGA

>TP3164

UAAUGUCAUAGGAAUGAGAUCUAAAAAUCUCAAGCUUUGUUUAGAGGUUGU

>TP3165

UGCCUGAAUUUUUUAUGUCAUAGUAAUGUCAUAGGAAUGAGAUCUAAAAAU

>TP3166

AACAGUGAAAAUUACCUAAAUUCUUAGCCCUAAUCCUCACAGAUGAGGUAA

>TP3167

UGUAAGGAUUAGGGAUAAGAAUUUCAGUAAUUUUUACUGUUUCUAGAGUUU

>TP3168

AAAUCGGGCCAUUUACCUCAUCUGUAAGGAUUAGGGAUAAGAAUUUCAGUA

>TP3169

UAGACUUCCUAGCAAACAACCUCUAAACAAAGCUUGAGAUUUUUAGAUCUC

>TP3170

AUGGUUUAAAAAUCCCACAGUGUGCAGGCACCUGUAGUCCCAGCUACCAGG

>TP3171

CAACAGGUGGGGCACAGAGUCUCAUACCUAUGAUUGCAGCACUUCGGGAGG

>TP3172

UAAUCCCUUCAAUAUGCUGUUGAAAAGCCAGGCGUGAUGGCUCAUGCCUGU

>TP3173

UAGCUGGGUAUGGUGGCACAUGCCUAUAGUCCAAUUUACUUGGAAGACUGA

>TP3174

AUUUUCCAUUUAUUUCAAAGGAAAUAGAGGAAAAAGAAAUAACUUGUGGUC

>TP3175

GUGAUCUUGGCUUACCGCAGCCUCCACCUCCCGAGUUCAAGCAGUUCUCCA

>TP3176

GAUAACCUAGAACUUAUUAUUUUUUAAGAGACAGGGUGUCACUCUAUUGCC

>TP3177

AAAAGAAAAAUCUCAGAUACAAGCCAGGCACUGUGUGUGCACUUGUAGUCC

>TP3178

GCAGUCGUGGCCACUUGCCUGGCUAAUUUUAUUUUUUUUGUAGAGAUGAGG

>TP3179

GAGACCGAGGUCUGACUAGGUUACCAGGCUGGCCUCCCAACUCCUGGCCUC

>TP3180

GACAUUCAUUCCAUUUAAUUGGGGGAACAAAAGGCCUCAGGUAAGGAUGAG

>TP3181

GCUACUUCCACUUCUGGAGUGCAGUAGCAUGAUCUCGACUCACUGCAACCU

>TP3182

GCUACCUCUUGUGUUUAAGAAUUAAAGGAAGAUGAGCUAGGAAUCAAGAAA

>TP3183

GUUUAUCCUAAAUAAACUGAAGUCUAGGCAAGUGGCUGAGCUAGGUUUGCC

>TP3184

ACUUUGCUGUAUUUACUAUACAGAUAGUUGACUGACAGCUGAGCCCGGACC

>TP3185

AGGUAACAAUAUGAUGUUUCAGGAAAUUAGAGAAGUUAAGAGACUUUGCUG

>TP3186

ACUCACCUAUCUCUGUGACAUAGCAAAGUCUGUUAAUUUCUCUAAUUUUCU

>TP3187

CUAGCCUGGGAAAGUCCAAAACCUUAGGUCUGGUUUCAGUUCACUCACCUA

>TP3188

CUGCAACCUCCGUAUCCUGGGUUCAAGCAACUCUCCUGCUGCAGCCACGUG

>TP3189

UUUACACAUAUUUGAAGAGUCAUAUAUAGCCAUAAAAUAGAAAGAGGAAGC

>TP3190

CCAGAGUUUCUGACUCAGAAGGUCUAGGGUGGGCCCACUAAUGUAUAUUUC

>TP3191

GAAACUGCGGAGGGAAAGCUGGAAAAGAGAGGUUAAGCAGCUGCAGGAGGA

>TP3192

UACAGUGUGCCGUGUUUGUGCUGCUAGGCUCCAGCCUGGGUGGCAAAGUGA

>TP3193

ACCCAUUUUUCCUUCUUACAGUUGCAGUUUCUUCAUUGCAAGUGAUUCACC

>TP3194

GAUUGCUUGAGCUCAAGAGCUCAAGAAGAGCCUGGGCAACAUGGUGAAACC

>TP3195

ACCAUCUGGGCAGCUCAGUGUUUAAAAAAAAAAAAAAAAAAGGCAGACCUC

>TP3196

CACGUCAGCAGAGAUGAGUAGAUGCAGCAGAGAGGAGUUGCGGUGUAUAGA

>TP3197

AAACAAGAGGUUUGUUGUUUUACUUAAAAUUGCAGUUUCCAAGAACCUAUC

>TP3198

CUUUUUUUUUUUUCCCCCAAGAUGGAGUCUCACUCUGUUGCCUAGGCUGGA

>TP3199

AUUUUGGAUUUUGGAUUUUCAGAUUAGGGAUGCUCAGCUGGUAAGUAUGUA

>TP3200

ACAGGUUGAGCAUCCCUAAUGCAAAAGUCCAAAAUCUGAAAUGCUCCAAAA

>TP3201

AGCACCCCUGCAGGGCUCAGUGACCAAGGAAGAAUCAGAGAAAGAGCAAAC

>TP3202

AACUGUUAUGCCUCAGCCUCUCCUGAGUAGCUGGUAUUACAGGUGUGCGCC

>TP3203

AGGGAGUUACUUAAUCUGAAAAGUAAGGCUUUGGGCCUGGGCGCGGUGGCU

>TP3204

CCCAGGGAGGUUAAGGGCGCAGUGAAUUGUGAUUGUGCCACUGCUCUCCAG

>TP3205

UAAGAUUCCUCUGCUUUCAUUAAAAAAAAAAAAAACAAAACUUGUAAAUUA

>TP3206

UCAGUUCCAGUAUCAUGAACAGGAUACUCAGUUACUGAGUAUCCUGAGCAG

>TP3207

AGGCGUGAGCCACCGCACCCGGUCUAUGAUCCUUAUCGUUCACUUUGAGCU

>TP3208

GAGGGAAAGACCUUGGCUCCAUGGAAUGUGAGCAGAGGUGCUGUGUUCAAC

>TP3209

GUUCCAAGUCUUUGCUAUUGUGAAUAGUGCCGCAAUAAACAUACGUGUGCA

>TP3210

UUCCAGUUUUAUCCACGUCCCUACAAAGGACACGAACUCAUCAUUUUUUAU

>TP3211

UGCAGCCAUAAAAAAGGAUGAGUUCAUGUCCUUUGUAGGGACAUGGAUGAA

>TP3212

UAUAGCUCACUUCAGCUUCCCAAGUAGGUGGGACCACAGGUGUGUACCAUC

>TP3213

ACUAUAUUGAAUAUUCAAUAUUCUAAGACUAUAUUGAAUAUUCAAUAUUCU

>TP3214

UACAAGUCCCCCGUGUCACACAAAAAUAGACGGGGUAGCUCUGACAGCAUG

>TP3215

GUGGUGCAUACCUGUAACCCCAGCUACUCAAGAAGUGGAGGCGGGAGAAUU

>TP3216

UCAACACGAGAUUUGGUGGGGGCAUAUCAAAACUACAUUAACCUUAAAUUA

>TP3217

UGCUCUGCCAGCUUCAGCUUCCCAAAGUGCUGAGAUUACAGGCGUGAGCCA

>TP3218

GCCUUAGGAUUUGUCUGCAGAGAAGAGAAAAUCCAGGUAGCUGUGGUUCCA

>TP3219

CUCAUCCUCCCCAGUAGCUGGUACCACAAGAGCACACCACCACACCUGGCU

>TP3220

GAGGUACUUUGUUAUAAAGUUUGAGACUUUGUUGUAUAGAUGUAAUGGCUC

>TP3221

AUUGGUUUGAAAGAUUCAAAGUCAAAGUUGACUUUCUUUUCCUUUCCUUCU

>TP3222

AUAUAUCAACUAAUUUUAAAACUUUAGAUUGUAACCUCUGAUCAAACAUAU

>TP3223

UUCUUUUCUUGAAUGAAUUUCACUUAGGAGAGGACUUCAGUAGAUAACUCA

>TP3224

CAAAGUAUCGUUCUAUUGAUUUCUUAUCUUGAAUGAAUUUCACUUCGGAGA

>TP3225

CCCUGGAAAUCUACUCGGUACUGCAAGAUACCUCCAAUUCCACCAAAUCCU

>TP3226

CUCAGCCUCUCAAGUAGAUGACACCACAGUUCUGUGCCACCGUGCCCAGCU

>TP3227

CUCAGCUCUGAGUGGGAGACCAUCCAUGGAAGAAUUUUCCUCGUUUCUCCA

>TP3228

UAAACUGUUGGCAUUCCUCUGGUAUAAGUGUGUCUGCUUUGGCAAUAAGUU

>TP3229

GAGUCCUUUAGUUUUUGAACUUUUUAUUUGACCUUCAUCUCUAACACCUGC

>TP3230

ACUGGAGCUCAGCUUCAGAGUCCUUAAGUUUUUGAACUUUUUAUUUGACCU

>TP3231

UUCCAAAUUCUUUUUCAUUAGCUCAAGGCGCCACUGGAGCUCAGCUUCAGA

>TP3232

GCACAUUCUUGUGUGAUCCCAGCUAAUCGGGAGGCUGAGGUGGAAGGAUCA

>TP3233

CCGCAGACCCAAAGCCUAAGGAGCCAACAAGCAGUUCGUCCAUGGAGCCUG

>TP3234

UUUCUCGCCCUGCAGGAGCAGUUUGAGUUCGCGCUGACAGCCGUGGCUGAG

>TP3235

CAAAAAAAACCUUUGACAUGGUGAAACCCCGUCUCCACUAAAAAUAUGGUG

>TP3236

GCAGCAUCUGGCCCCAGACAGGGGGAGCUGGGGAGUCCUCAGGGCCCUUCG

>TP3237

UGGGGUAUUCGAUAUUUAAAACAAAAUGCAGUUCCAACAAUAUUUUCUUUG

>TP3238

UAUGAAGCGAGAUUCAUGGGUUCCCAGUAAAUACCAGUUUCUGUGUAGUGA

>TP3239

GAUUUGUUGUAAGAACUGCCGGGGAAGAAACAAUAAAGACCGGAAGCUGAG

>TP3240

CUCCCAAGCAGCUAGAGCUACUGGCAUGUACCACCACAUCUGGCUUUUUUU

>TP3241

GUCCUCCUGCCUCAGCCUCCCAAGCAGCUAGAGCUACUGGCAUGUACCACC

>TP3242

UGAAGGUUUAAAAAAUUUUAAAGACAGUGUCUUGCUCUGUCACCCAGGCUC

>TP3243

CAGGCGUGCACUACCUCGUCCGACUAACUUGUAUUUUUAGUAGACACGGGG

**(2) List of 3,243 non-A-to-I editing-site samples**

>TN1

GAATGAGTCCGAACTGGATCTGGACAAGACATGTGAAGAGAGCTCCAGGCT

>TN2

TTGATTCCAAATAAAACAAATATTTAAAAAATTTAATGAATAAACACTGGG

>TN3

AAGGGATTTTTTTTAAAAAGCAAACAACAACAACAAAAACCCCACAGAAAA

>TN4

TGGCCTCCCGACCCTGATGGCAGAGAAGCAAACACCAGTCGGAGAGCTGGG

>TN5

GTGCTCTGTAGACACTGGTTGTAGGAAGGAATCTACAGGTTGAAATAAGGA

>TN6

ACTGCTAATATTGAGCACTATCAGTAAAATACATAAAACCCTTTGCCAATC

>TN7

TTTTAATTTCATTCACGATGTTTTTAATGAATAATTTTAATTTTTATGAAT

>TN8

GCTTAAGTCACTAAGATTTGGGCAAAAGCTGAGCATTTATCCCAATCCCAA

>TN9

CTAATCATCAGAGAAACGCAAATAAAAAACTGCAATGAGGTCTTCTCTCAC

>TN10

AGATGGAACTAGGGATCATTATGTTAAGTGAAATAAGCCAAGCACAGAAAG

>TN11

TATTATGCATCAGTGAAAAAGAAAAAAGAAACAAGAACTTAGATTTTAAAC

>TN12

TAATATAAATATATATTTATGGAATAAATAAATGAATAAAATATCTTTGCA

>TN13

CAGCCTAGCTGTGGGCAACCCCGGGAAGGCTGAGCCCCGCAGGCTGTATGG

>TN14

CAGGTCACAGGTGGGCAGGAGGTCTAACACCTGTAGCGAAAGAACGCAGGA

>TN15

CACAATCCCAAGCACCATAATGTGGAATGTTGAAATCCCTAAAGATCAAAA

>TN16

TCAAATACATACCTTTTAAAAATGTAAATAAATAACATCGACATTATTTTT

>TN17

CCATCTCAGCAGCTCACGGTGTGGAAACTGCGACACTCACACGGGTGCCAT

>TN18

TCTTGTATTTTCTCTCCCACATCCCAATTATCAGCCATTTCTCCAGGGAGA

>TN19

GCCCATGGCCTTGGGAAGCAGGCATAAGTTGGGGGGGGTGGGCATACAGGG

>TN20

ACTCCTCAAGAGTCGATTGAAGCACAATTTATTAGAGCCCAGAAATCATGG

>TN21

AAATGAAAAGCTGGGGATTTTAAGCAAAGGAATGCCTTGACCCAACCCTCA

>TN22

TATCTAGAATACATAAAGAACTCTCAAAATCCAACAGTAAAAACACCAAAT

>TN23

TGGGCATCAGAGGCGCAGAGGTGGGAAGGGCTGGCTTCAGGTGGGAGTTAT

>TN24

TAGGGTGCCTGGGTAATAGCAGAGGAAGAAAAAGGCTTAGAGTTGGAGGGA

>TN25

ACTGGCAAGGGAGTGCCGACTGGAGAATCTTATCCTCTGTTCCTTAGCTGT

>TN26

CCAGTCTCTGCGGTGAGGCAGTGGCAAGAGCTCCGTGGGCGGGAAGAATAT

>TN27

GATCCCCAGCAGCAACAGACTTTACAAAGTACTCAGTATCTAATATTTAAC

>TN28

TTCTACTGACAACCAGTGAGCTTGCAAAGAGGACTTGGAGCCCCAGATGAG

>TN29

CAAGTCACCCACTGAACACAGAGTGAAATTTGCAGAGGTTTCCCCTCTGCT

>TN30

AAAAGTGAAATTTAGAAGCCCTATGAATGAAACAACAATCCAACAAAAAAT

>TN31

GGAGTGGGGATGATGGACTGTGGGAAACCTGCATGATGGGCATGAAACAAT

>TN32

AACCGTTGTTTAGACAGTTATATGAAATGGGGTATTTTCTAGAGAAAAGGA

>TN33

GAGCCAAATAGATTTTCAAATAAGAAAATGAGAGGACATGAGCTTGAGGAA

>TN34

TTAGTGAGTACTTATTATCTCATTGAATCCTGAGGACTACCTCACAAGGTA

>TN35

TCAAGTTGGAGGACAGCAAATACAGAAATATAGAGTCTAACATAACAACAG

>TN36

TTCCTGCCAGGACTCTGACAGATACAAAGAACCCACAGCCGACTGCTGGGT

>TN37

CAGCCTGACTGGCTTGGTGTCCTTGAACAAGTTACCTAACCTCTCCATACC

>TN38

TCAGTCCCTCAGCTGTAAAATTTAAAAAAAAAAAAAAGAAGAAGAGTACCT

>TN39

CAGACAGAGGTCAGTATCAAACTAGAAAATTTAATAAATGCTGTCAGATTT

>TN40

AGATTATATTCTCTGATTATGACACAACAAAACTAGAAATTACAGCATGGA

>TN41

ACAACAACTATCCTTAGGAAAGTGGAAATAATGTGTTAATAAATATGAAAG

>TN42

CACTATGCCTTGAAAAGAGGGAGAAAAATTGTGAATTAAGGAAGGGAAGAG

>TN43

AGGGCGAGATTCCGTCTCAAAAAATAAAATAAAATAAAATAAAAAATAAAA

>TN44

GCCAGCTGGCATGTCAACCATTCGAAAACTCAGGGTGTTCGGGATAAAGAA

>TN45

TAGATTATTTTGACCAATGAAGTCTAATCTAAATGTTCTGAGCATGTTCAA

>TN46

TACTCAAAAGTGCTTACATACAATGAAAAATTTGGAAATCCATAAGCATGG

>TN47

AGGAACCGAGCCGCCTTCAAACATCAAACATAAAAAAGAATGCAGATATTA

>TN48

CAAAACCAGTTTACAAAAGTTACTAAACAAATAAAAACTACATCCCACAGT

>TN49

CTTAAATAAACTTAAAGAGCTAAAGAAACCCAAGAGAATGACATATAAATA

>TN50

GAAGAGAGAGAAAGAAAGGGAGAGAAAGAAAAAGAAAGAAAGAAAGAAAGA

>TN51

AAAAGAAAGAAAGAAAGAAAGAAAGAAAGAAAGAAAGAAAGAAAGAAAGAA

>TN52

ATCAGACAAAATAGACTTTAAGTCAAAAACTGTTACAAAATACAAAGAACA

>TN53

TGTACACCAACTAACAGGGCTCCAAAATATATAATGTAACCATTGAGAGAA

>TN54

CAGATGATATGTTAGGCCATAAGATAAGCTCAATAAACTTAAAAAGATTGA

>TN55

AAATCCAGAGCTAGCAGAAGGAAGGAAATAAAGATTAGAGCAGAGATAAAT

>TN56

TACACCAAAAATACAAGCATGAAACAAACAAATGTAGCCAAAATGTACCAG

>TN57

AGTGAATCCAAATATATTTCAAATGAATGAATGACATAATCAAACTTAAGG

>TN58

GTATAAATATAACAATTCTGAAACAAATGTATGTGCATTGTAAGATTAAGC

>TN59

GAGAGTCACATGCTTCAGGGCTGGCAAAGTCCTATATCTTGACTTATGTGA

>TN60

CAATGCATTCATATTTTTAACACTGAAACACAGTAAACAGGGAAAATTTTG

>TN61

TAACAAGGGAAGCAAATAATCATTGAATAAAAATAGCAGAAAGAAAAAGCT

>TN62

ATGGAATGAATAAAATGATAGCCACAAAAATCAAGGTGGGAGAAATACTTA

>TN63

TAGCTCAACACTGAGTGACTATAGGAAACCAGAAACCAGGCTGGGCGCTAA

>TN64

TCAATGAGGCCTCAAATGAATCTTGAAAGTGTGCAAGGATTAACCAAATGA

>TN65

AGAAATGTGTAAGTTTTTCAAACAAAAAGGAACAGCATGAGCAAATGCAAG

>TN66

ACTACAATGCAGATGAGTGATTATCAAGGTCTGAACTGAATAGTGGAAATA

>TN67

ATATGTAAGCTTTTCTATATTTCAGAAACTATATGACGTGACGAAAAGTAA

>TN68

ATTCAATAATAAGTTTGCATATTACAACCTTGTTGAATGTTGGTGTAATTC

>TN69

ACTGCTGCATATATACCCCCCAAAAAAGAAATCAGTATATCGAAGAGATAT

>TN70

TGGATTGTTTATAACACAAATAATAAATGCTTGAGGGGATGAATATCCAAT

>TN71

TGAATTCATTCCTCCTGTACAATGCAATTTTGTACCCTTTGACCAACATCT

>TN72

TCTTCCTAGAAGTATTTCAATTTCAAAAAGTAGCAACAACTGTGGGAGTTC

>TN73

ACCATTGCACTCCAGCCTGGGCAACAAGAGCAAAACTCCATCTCAAAAAAA

>TN74

GGGGTAGGAGACCATCAGGACAAACAAGTGGGTACATGGAGGGGAACAACA

>TN75

CAGGGGGTAGGAGATCATCAGGACAAACACGTGGGTACATGGAGGGGAACA

>TN76

AACACGTGGATACATGGAGGGGAACAACACACACCAGGGCCTCTCAGGGGG

>TN77

ACAGGGGGTAGGAGACCATCAGGACAAACACGTGGGTACATGGAGGGGAAC

>TN78

ACAAACACGTGGGTACATGGAGGGGAACAACACACACCAGGGCCTCTCAGG

>TN79

ACAAACACGTGGGTACATGGAGGGGAACAACACACACCAGGGCCTCTCAGC

>TN80

AGGAAGTTGGTTCTAACGTTTCTCTAACAACTGGCTTCAGTGAAACACTCC

>TN81

CACCTTGTGGATTTTTAGGTTATTGAAATTAACCAGTCTTCTGGGTGCAGC

>TN82

TTGAATGGCTTGGTGGCTGAATTCCAACAAACACTTAGATGACTAACACCA

>TN83

CACTATAAACAATTACAAGCCAACAAATTGGATAACCTAGAAAAAGCAGAT

>TN84

AAGTCCTAGCCAGAGCAATTAAGCCAAATAAAGAAATAAAAGATTCAAATT

>TN85

TCACAACAAACTATATGACAAAAATAAAGAAATCAATCTCATTCACAGTAG

>TN86

ACAGAAATAGAAAAAACAATTTGAAAATTTATATGGAACCACAAAGGATCC

>TN87

ATCTGACTTCAAAACATATTACAGGAAAAGAACAAAAGAAGGAAGAAGAGG

>TN88

GAAAAAAATCTCAGAATTTGCTGGCAACATGGCCAAATAGGAACAGCTCCA

>TN89

CACAACTCCTCACCAGCAAGGGAACAAAAGAAAACTGGACAGAGAATGAGT

>TN90

AACAACTGGTATCAGCCACTGCAAAAACATACCAAATTGTAAAGACCATTG

>TN91

ACACTATGAAGAAACTGCATTAACTAACAGCAAAATAACCAGCTAGCATCG

>TN92

TAATGACAGGATCAAATTCACACATAACAATATCAACCTTAAATGTAAATG

>TN93

ACAATTATAGCAGTGTGTAGAGGGAAATTTATAGCACTAAATGCCCACAAG

>TN94

ACCACTAGCCAGACTCATAAAGAAGAAAACAGAGAAGAATCAAACAGATGC

>TN95

TTCCCTTTGAAAACCAGCACAAGACAAGGATGCCCTATCTCACCACTCCTA

>TN96

GAAGGAGAACTACAAACCACTGCTCAAGGAAATAAGAGAGGACACAAACAA

>TN97

CCACTGACTTTGTTCACAGAATTGGAAAAAACTACTTTAAATTTCATATGG

>TN98

TCCACATTTGAATTCCATTAAACCAAAATCTATGTTGAACGAAGTGAAGTC

>TN99

CTAACCACTGTGAGAAACCCAAATAAAAATCGATCCCCCCCCAAAACAAAT

>TN100

ACGTATCACAAAACCACAGTAATCAAAACAACATGACACTTGCACAAAAAC

>TN101

ATTTATGACCAAGAAATTTTTGACAAAGGTGCCCAGAAAACGTAATGAAGA

>TN102

TATGTGACAACATGAATTAACCCAGAAGATATCACGCTAAGTGAAATAAGC

>TN103

AGGGCCTTGGATGAACCACCTGAGGAATGTTTCCAAACAGTAAACAGGATG

>TN104

TTCCTATTCCAGAAAGACAAATTGCAATAATAATCAAATAATATGAGCAAT

>TN105

CATCCAGTAAAAATAATCTGGTAAAAACAGCAAAACTCAAAAGAGTGATTT

>TN106

ATATATATCAAATGAGTATTAAGATAAACATTCAAAGAGTTTTAAAGAAAA

>TN107

AAGTGTTATAGATATTGGAGGGCAGAAGATACAATTGCCATTAAGAACAGG

>TN108

TAACCATATTAGATACCCACAAACAAATAGAAAGTGGATAGTTCTTGATTG

>TN109

GGTTGACCAGGCCTGATTTTTGAATAAATTCACAAACTTACCTCCATACAT

>TN110

TATCAAAAATATCACATGTACCCCAAAACATGTACAACTATGATACATCAA

>TN111

TAAAAAACAACAAAAAAACCAAAAGAATAGAAATCAAAAATAAATACATAA

>TN112

GTTGACCAGCCAAGGCAAAGCAGTCAAACCATACAATACCTTATCCTCAGG

>TN113

GATGTATCAAATGGAGGAAAAGAAAAACGGAGGGAAGGAGTTCCCTTAAGA

>TN114

CACAGTTCCAGCTTTAAGGCTATAGAAACAGAAATAGATATACTGGTAAGT

>TN115

ATTATAAATGTCAAATGTATTAATGAATGAATGAATGGATAATATAATGAA

>TN116

ATGAAATCATATAATATTCCATGGAAAAAACAGAATGTATAAGGCAAAGAG

>TN117

AGCCACAACCTTACAAAAGCTAGAAAATATTTACAATTCCACACAACAACA

>TN118

CATGAAGAAAACCTTCTGGACATCAAAAGTTTAAACTAGTCAAGACTGAAC

>TN119

ATAAAAATACTAGAAGAAAAAGGGAAAATTACATAAGGTACAGAACATACA

>TN120

ACACAAGAAGGTGAAATGTAACATTAAAGTCAGGTCCAACAAATGAGAAAT

>TN121

TGAATTTACCGGACTTCACAAACACAAAATAAACAACCCACAAAAAAAAAA

>TN122

TAGAAAAATTAAAAATAACAAAATCAAATAAAGTTGAATGACGAAGGTGGG

>TN123

GGAGAGCGCCCTTGGAAATAACACGAAAATTAAAGAACTAAATATAATTTA

>TN124

AATTGCAACCATAAAAATTACTCTGAAATACAATGAAAATTACTGTAAGAG

>TN125

AATCCAGTGGGACAGTCAAATCTTAAAGCTCCAAAATGATCTCCTTTGACT

>TN126

GCCTCACAATCATGGTGGAAGGCAAAAGGCACATCTCTACATGATGGCAGA

>TN127

GGATAGAAGCCAGAAGGATTCCACAAAGACTGTTAGTGAAAAGTGAACAGA

>TN128

GTCTACTGGAAAGGGCTTTAAGGATAATGAGAAAAAATCATCAGTGGAGGC

>TN129

GCCTGGAATGATATCAAATATAGGAAAAATACCGAAAAAGTTTGTGGATCT

>TN130

GGTGATCCACCTGCCTCGGCCTCCCAAAGTGCTGGGATTACAGGCGTAAGC

>TN131

TGCCAGCTTCCCTTCCCCTTCTGCCATGAGTGGAAACAGACTAAAGCCCTC

>TN132

ACCAAATAAACCTCTCTTCTTTAAAATTATTCAGCCTCTGGTATTCCTTTA

>TN133

AAGGTAGAGAATCTGGTGGGGAAGCAAGCAAATGCCCATCACATGCACTTT

>TN134

TACTCTCAGTTATAAGTGGGAGCTAAATGATGAGAACTAATGAACACAAAG

>TN135

GCCATTGCACTCCAGCCTGGGCAACAAGAGCAAGACTCTGTCGGGGAAAAA

>TN136

CAGTGTACATAAAAATAATTTCAAGAAATTTATAAAATACCGAGATTATGG

>TN137

AGAGCCAAGTATGTGCTTACTGAATAAGCTGCTAAGGTTTGGTGGTTACAT

>TN138

AAGGTTCATTTCCATTAGACCAGAAAAGACAGCACATTTGAAGGCCTGAAT

>TN139

TGTACTAGAAATTTAAAAAATAAATAAAATAAACCTTCAAAGTGAGCTAGC

>TN140

CATGAGCTTCTAACACACACACAAAAATCACACACACAAAATGGGGGTAGC

>TN141

GTGTAGCAAAGGTCAGAATTCTGTGAAGCTTGAGATGTTTATTATAATGAA

>TN142

TTAATGTGCAGATTATACTACAGTGAAAGTTGCCAATGACAAGGCAAAGTC

>TN143

TGTCTTATTTTCTTGTCTCCGGAAGAATTTCTGTAACAGTGCAATTAAACG

>TN144

TGTGTATAGAAGACCAAAGGGCCAAAAGAGTCAACTTCTGAAGAAGCGCAA

>TN145

GAGTCCAGAGAGAGATGTGACAATGAAAGAATGGTCAGAGAAATGTGACAT

>TN146

ATATTACATTATAGAATAAAGTTGTAAGTATGTATATGCAGTGACTCAGCA

>TN147

TGCAAAATAAAGAAATGTGTTTAGAAATAGATTCACATGTGAGAAAACTAG

>TN148

AAAATGAGAAAACAAAAAAGTAGAAAATGATAAATTACAATAAAGAAATGG

>TN149

AGAAAAAATTATAATCTAGTTGAGTAATGGTATATTACATAGCTATTTTCT

>TN150

TCCTTTAATAAAAATATAAAGGAATAATATAATAATTTTCTTTAATAAAAT

>TN151

ATAATCCTTGAGTGGGGGAAAGGTTAAAAACCCCCCTGGATAAGTGTTACT

>TN152

TAGATAAATATCAAACTTTCTCTGAAACTGTAACTGTAAAATGTAAAAAAC

>TN153

GGGGAACATTAAAAGCTTTCTTCCCAAGCCACTAAATCAACTTGACTAACA

>TN154

AAATTACCACTTGATTTAGTATTAGAAAATTACATTACATATCAAACATAA

>TN155

TAAATACCTCTTAGGTCAGAAAAAAAAAGTCAAAAGCTAGAGTATAGAGAA

>TN156

AATATTTTAATGGAAAATAGAACACAACTAATTATTGAAGAAATTATAGAA

>TN157

TAAAAGGAAAGATGCATAAATATATAAATAAATGATAAAAAATGTTGCATA

>TN158

CATATATGACTTTTTCAGAATCAAAAAATTTAAATTTCTGTAATAAAATTT

>TN159

GGATTATATATTTAAAATAATGAAGAAACCTGTCAATTGAGAAATATATAG

>TN160

TAAATCTGTTAGCTGGTATACAATGAATAAAGATATAATTTGTCACATCAA

>TN161

GTTATATGTAATTCTCATAGTAACCAAAAATGAAATATACATAGAATATAA

>TN162

ACAAAAGGAAATGAGACTAGAAACAAAATGTGTCACTACAAAAAAATCAAC

>TN163

TAAAGATAAAAAAGAAATAATTGAGAAAATGATTGGCAAAAATCAGTAACT

>TN164

AAAAGTCACTACTGGATCCACAAGCAAGACTATGGTAAATAAATTTCTCCA

>TN165

GCTTTAGTTGGGGAAAATATACAATAAGCAAGCCAGTTTTTAAAATGAGAA

>TN166

CAAAACAAAAGAAAACAAAACAAAAAACCACAAAAAAAAAGACTCCATTTC

>TN167

AAAACCCCACAGAAAAGCAAACAACAAACAAACAAAAAACAGAGGAAGAAG

>TN168

TTAGCATAAAGATTTTCCTTAAAATAAGAAGAAATGCCTTGAGTAGGCTTT

>TN169

TGTCCCCCAGGTCTGTGCTAAGCAGAACGCAGCTCCGCCCTCGCGGTGCCC

>TN170

CACGCCGCCGGGCGGGGAGCGCGGGAATGGCGAGGTGCAGGCGCAGAGACA

>TN171

TCACCAAGAGGCAGTACAAAGATGGAAGATAACTTCATTGAAAAGAAATAC

>TN172

TTCAAAATACATAAAAAATTGTTAAAACTAAACAATAAGTTAAACAGCCCA

>TN173

TTAACAAATGACAAATTGCTGGAGGAAAAACAAGAACTCTTTTCATTGCCG

>TN174

TCCAAGCTAATTATTTTGAAAAAAAAAAATGCTAACAAAGGAAGTTGTGAA

>TN175

TGTCCAATTAAAGCAGAGCTCCCAGAAAAATACAGCTGCCATTAACCCCAT

>TN176

CTCTGTCTCAAAAAAAATAAAAATAAACATAAAAATGAAGAAATGTCTCCT

>TN177

GCCTCACAATCATGGCAGAAGGGGAAACAAAGACGTCCTTCTTCACATGGC

>TN178

AGCAACAAGAAGAAGTGCTGAGCCAAAGGGGAACAGCCCCTTATGAAACCA

>TN179

TGGCTTTAACATCCACGAAGAACACAAGCATGTTGCTTTCTTCTATCTTCT

>TN180

GGAAAATAAAAGATTACTAAGTATTAAAATTATAATCAGTATATGTAAATA

>TN181

TCAGGAGATCCAGACCATCCTAGCTAATTTCTTACTTTGAGATTGCTATCC

>TN182

TGAAAACTTCCAGCAAAGAAACTTGAAAGCACCTATGTGGTCATCTCCTGT

>TN183

TCTTGCTGCACTTATGTAAATAATCAAGCAAAATCTAACAAAACTAGACTT

>TN184

ACTACAGAGAGAAATTTTATGTTTCAATGGAAAACTATAATTTAGCCGGGC

>TN185

TGGTGTTGACCTAAGTCACTTTGAAAATGAATAGAATCTGTAAGCTGAAGG

>TN186

CAGAAGCAACTGTGAACAACTGTAAAACCACAGTGTCTGTATCTGGAATAA

>TN187

TATAGATACACACAGTTCTACATAGAAAACTTTATAATTAGGTGTGTATAG

>TN188

GCTCACTGGGAAAAAATATTCAAAGAAAGAAAGAATGTGGACAGAACTTAT

>TN189

GGTATGGCTTCACATATGAAGTTCTAATACTTTGCAAGACATAAAATGTTT

>TN190

GTTCAAGACCATCCTGACCAAGATAAACAAACCCCATCTCAAACAAAAATA

>TN191

CCATTAAATACTGTATGTGGAATATAAACGGTGCAGCCTATTTGTAACAGT

>TN192

TCAAGACCAGCCAGGATGACATAGAAAAACCCCATCTCTCCTAATAATATA

>TN193

CAATACCAACATGACCAACGTAAACAAACCCCATTTCAACTAAAAATACAA

>TN194

AAAAAAAAAAAGTAAGTGAAAAGACAACCCACAGAAAGGAAGAAAATATTT

>TN195

ACACACACTTTGCCAAAAAACATGCAAATGGAAAACAAGCACATGAGAAGG

>TN196

GAGGCAGAGGCAGGTGGATCCCTTGAATTCGGGAGCTCAAGACCACCAAGA

>TN197

ATAGCAAAACCACGTATCTACTAAAAATACAAAAATTAGCCGGGTATGGTG

>TN198

ACTGAAAAGACAACCTACAGAAAAGAAGAAAATATTTGTGCATTATATATC

>TN199

AGAAAGGCTATAATCCAACAAATGTAAAATAACAAGTGTTGGCAAGCTCAG

>TN200

TCTTTACATCTGTTAAGGCTGTGAAAACTGAGAGTCGGCCGGATGCAGTGG

>TN201

GCTGAAAACAGGAAGGATTTTACTCAACCATGGACGCCGCCGGCTCAAGGT

>TN202

GAAGCCCAGTCTCTACCAAAAACACAAAAAATTAGCCGGGTATATTGGCAC

>TN203

GGAATTTGAACAAACACCGACAATGAAGGAGAGTGACCTGAGCAAGTAGTA

>TN204

AATTCAGTGTGCACTTCGTAAGGATAATGATGATTTGAGTTAGTTTAGTAT

>TN205

TGAATCAGTATTGATCTGGCCACAAAATATTAATGATTTGAATTAAAAAGA

>TN206

GTTTAATTATATGTACGCACACACAAACACACACTTCGGTGTCTTATTATG

>TN207

TTTTTATATCCTCATTAAGTTTTAAAATTTATTTACATGGGTCTACAAATT

>TN208

TTTAATCTTTAAAATTACTTTGTGAAAATGATAGGTTTTCTAGTATGGAAT

>TN209

CAAGATGTGACCCTACTGAATGAACAAAATAAATCTCCAATAATTGACCAT

>TN210

AGTAAAATCTGGAAAAGGATACATGAACAACATGAGAATATCAACAAAGAA

>TN211

AGCAAGTAGAAAAAGAATTTTTTAAAAGTGAAGAAACTGTCTGTCTTTGCT

>TN212

ACAGAAGACACTCTTATCACCTATGAAGTTCCAAGGCATTACGAGCTCTGT

>TN213

TGGAAGAGACAGAATCATTCAGTACAAGTGTATCCAGAGGACCAGCAGAAG

>TN214

TTTTTAACCTTAGACAACATGAGAGAACTCACACTAAAAAAGCAATGAATA

>TN215

TGTAAGAATCATCAGCTGTAGCGTTAACACTAAATACACCAAGGACAAACA

>TN216

TTTACCACTTTGCTCAACCTAAATGAATTCAAGGTAGAGAGAATCCAGATG

>TN217

ATATTATATGATTTAACATTGCAAAAATAAAACTAATTACAAATGAATCTA

>TN218

TCAGATTAAGAAGTGGTTCCTGCAGAAATTTTTAGATAAGGGCTAGTAAGT

>TN219

AGATTAGATATTCCTCCTTAATCTTAAGGGTGCTGTAGAAGGGCAGAAGTC

>TN220

TCTCAAAAAAAAAAAAAAAAGAAAAAAGAAAAATCCCTGGGTACCTCAACT

>TN221

CAAGGACAAAACAGGTGTTTTGCAGAAGTTGGCTAAAGCAACATAAAAGAA

>TN222

GAGCACAGATCTCAGAGGAACAGCTAAAAGGGGGTCATCTAGGGTATCTGG

>TN223

CAATTCCTCAAGGATCTAAAACACGAAATACCATTTGACCCAGTGATCCCA

>TN224

AAAATACTTCTAACTTATAGGGCAGAAAAGGGCAAGACCAATACTCCCCTA

>TN225

TAATGGAACAACACAATGGAGTCCGAAGAGAAAGTACTCAGGGAAGGGGTG

>TN226

ACTGGCAGTCTCTTTGATAAAACATAACAAAAGCCACCTTTGCTCCAGTTC

>TN227

TTCAAGTAACCTTTTCATTAGGTTTAACTTCCAAGAAATCTAAATGGGTTT

>TN228

AGAGCTGGACTAAAATCTTATATATAAAACCATTCTAGGCTTAAAATACAT

>TN229

TATGACCCAATTGGTCACTACGAGTAAGCGCTTTGAAAGACTTTTAGAATC

>TN230

ATAAAAAATGCTAAAAGAAAGAATAAACAGGCTAATAAACTAATATCTTTA

>TN231

CAATTACACTCTTCTAATTACTTTTAAGTGTACAATTAAGTTATTATTGAC

>TN232

GGAATTTCCGGATTCTCCAGAACACAAAATTATTAATTAAATTATTAAAAG

>TN233

GAAACCCCATCTCTACTGAAAACACAAAAAATTAGCCGGGCGTGGTGGCGG

>TN234

AAATGGAGACGACAGTAGTTACGACAAATACTTGAGAAAAGCCTATGAAAT

>TN235

GTGTGGTGATTCCTCAAAGACCTAGAACCAGAAATACCATTTGACCCAGCA

>TN236

CATGGCACACGTTTACCTGTGTAACAAACCTGCACATCTTATACATGTATC

>TN237

CCATAACTTAAAATAAAATAAAATAAAAATAAAATTCAGAAATAACCACCC

>TN238

TATAATGCTTCGTTGAAGTGAGGAGAAATCACACTTACATAGCTTAAAAAT

>TN239

AACTGATCACTACATGCAATGAGAGAAATGTCGCACTGGGGAACACAACAG

>TN240

TTCTTGTTCATCAAAGCCATGACATAACAGGTCCACAGATCAGGAAAACAG

>TN241

CCCCACAGTGAGGAAGAAGGAAAGAAACCCGGGAGCCAGAAGTGGGAGGCA

>TN242

GGGAGTAGAGGGTGGGGTTGGGGTGAAGATTGTGGGGTGGGGGTAGGAAGT

>TN243

TTATATGAGTGGCAAGAAAGACCACAAATGTTGACTTGGTGACTATATAGA

>TN244

TTCAGTTGAGAGATGACTTTGGCAGAAGAATCAACAGGACTGCTTTGAGAC

>TN245

AAAAATTTTGATTCTCAATGATGCGAAAGTAAAATACTCACTTAAACTACC

>TN246

GGGTTAGTTCCTGCAGCTGCTGTAAAAAGTCGCTACACACTCATAGTTTAA

>TN247

AGCAAAAAAAGAAAGAAAAGAAAAAAAGAACGCACCCCCAAGGTGAATTCC

>TN248

CACCCCTAGCACACCAGTCTCATGAAAAGAGGGGGGTCCCTGAAGAGCACC

>TN249

GCGCTGTTTTGCATTCCCACTCTGGAATGGATGAGAGTTCCTGCTGCTCCA

>TN250

ACCAGATGTCAGGAGGTGTAGGTTTAAAGAAAGTTCCTAAGTCTGGGCCGT

>TN251

GAGAGAAGTCAAAAGTTCTGATTTGAACCAGACTTCCAAGTGGAGATGTCC

>TN252

ATGTTGGTTTCTAAAAGGTTAGCAAAAGTGTCCAAGTGACACTTCGTCTCT

>TN253

GCAGTGAGCTATGATCACACCATTGAACTTCAGCCTCGGTTTTAGAGCAAG

>TN254

ACCCTGTCTCAAAAAAAAAAAAAAAAAAAAAAAATGTCCAAACACTGTTGA

>TN255

ATCCATGGTAATGATAACGAGGGCTAATACTAATCCCTCCTTCTTCCCTCT

>TN256

GCATTTTCAGGGGACTGTGTAGTAAAACCTAAGTACAGCTGTCCTAGGGAA

>TN257

GCTGTGCAGAGAAAACCATCTTAGAAATGAAGCCAACACAGAGACAGCAGA

>TN258

AAGGGAGTCACCAAACAGCAGAAGCAAGAAAGCATCGTATGCCTTTGCCTT

>TN259

GAAACTCTTACAGAGGGCTGAGAAGAAAAGTCAGCTTAAAACGTCCACATT

>TN260

GACCCTGTCTCCATTAAAAAAAAAAAAAAAAAAAGATGGTAGTGATGATTC

>TN261

AACTCAGGGTTGCAATTCTGAAGTGAAATTTAGATACTTCCAATAAGAGTC

>TN262

TTTATAAAAGAGTAGATATGGAGACAAGGCGTCTGCGGCCCATAGTATTTA

>TN263

GATTTTTCAGATAAATATGACTCAGAAGACCTCACTGTTTCTGTGTGATTT

>TN264

CTAAAAAATGAAATGAAATAAAAATAAAGAAAGAAAAAACTTGAGAACAAG

>TN265

GCTTCTCTGCCTACTGGGCACATAGAAACGAGTTTAAAAGCTGCAGACAAT

>TN266

TAATCACCATATACATTTGTTTTTGAATATTCACTTTTCTGCAAAAACGTG

>TN267

TTTCTGGGGCCAACTAATTATGGGAAACATGGCTCTCTGTATCTTCCTCTT

>TN268

GTGCTGTACATTCTATAGGTTTGGGAAAATATATAGTGGCATGAACTCATC

>TN269

TTGTAATTTAAATTAATTAAAATAGAATAAAATGAAAACTTCAGTTCCCCA

>TN270

TAGGGTTCATTTGTTCAAATGCTGGAACATTCTTCAAACCCTGTCTTAGAA

>TN271

AGGAAAGAAACGACAGAGGCCATCAAATGCACAGATGAAATTTTTAAGTGA

>TN272

ATGCAGTTGCTTTTGAAAACAGGAGAAAATATATGCATTTATACTTTTTTT

>TN273

CTCATAAAGCAGTTCAGCTAGCAAGAAATTCTCTCCGTTTTTGCTTATCTT

>TN274

GATGGGGCTCCCACCCTCTGAGGACAAGCACAGGCTGAAGCAGGGGAGCCC

>TN275

CTCATTTGTTAATTCAAAAAAGTGTAACTACTTATATGCTGGGATAAACCA

>TN276

TGTTAAGTCAGGAACAATCCCCTGGAAAGTAAGTTAGGAATGTAGGAAATT

>TN277

ATCTCTTAAAATCAATTAGAATAATAAAGTCATATATGTTTCTGGCAAGTT

>TN278

CTACCCCGAGGGAGAAAAGCAGCTAAAACCAACAATGGAAGCATTTTATTC

>TN279

CTCCTCCAATGAATAAATGTAAGTGAATAGAAATAAAGTTGCTTTCTAATT

>TN280

AAATCAACAACAAAAAGAGATCTAAAAGGTGAAAAATAAGAGAAAAAAGTG

>TN281

TAAGAAAATTGGAGAAGTGGTCTGGAACGTTCAACACCAGAAATAATGGGT

>TN282

CTGCAATTTCAGAAAGCCAAAAATGAAGAAAAGACCCTGAATGCTTCAAGA

>TN283

ATTCTCCTGGAGGAATTTAACAAACAAACAAAAAGAAGTGATCCAGGAAAC

>TN284

CACGGACAACTAAACAACAACAAAAAAGATAATTATTGACTACCCATCAAG

>TN285

TAACAACAAAGAAAGAAGAAGCGATAAAAAAGATAATCCTTAGAACTTGGA

>TN286

AAGGCTTCGTTATAGAGCAATTGAAAAAGTTACCTCACAGCGTGCCAGGAA

>TN287

CAGCTGGTCTCGGGAGCCACAGAAGAAGATTCGAGTAGGAGCGAGAGAGCT

>TN288

TCACAGCCTTTCCTGGGTGTAATGGAAACACATTTCACTGAAGCATTCGGT

>TN289

TTTATTTATTTTTAATTTTTTAATTAAATTTTTTATTCCTTAATAAGCATT

>TN290

AAGCAGTGAAACTTTTAAAGAGTGCAAGCCTCACGTCAAATGGAGTCAACT

>TN291

GTCAAGAAACAAGAGAAAAATGTGTAAAGCATAAGCATAGGTTATTTAAAG

>TN292

CTACCTGGAATTTAGGGAAAACTCAAATTAAAATATTATCTATCAGGTTGA

>TN293

CATCGGGGCTCCATTCTCAGGGCGTAATCACCTCCCAAAGCCCCTGGTCCT

>TN294

AAGAAGAAGAAGAATTCAGCAAAACAACACAGGCACAGCCCCTGAAGGTGA

>TN295

TTATAGCCAGACATCTTTTAAAATGAAAATAAATATTTCTTATATAATTCA

>TN296

TATGATTCTGGTAAATCTGTTAGAAAATTATTCTAGAAAGAAAAAAAAGAC

>TN297

CATATTTGAAAATTGAAGCATTTGAAAGAAATACAGAAAACTTGAATACCA

>TN298

ATTCCATATGAAAACCAAGATTTAAAATGTGAAAAGTAGTAATCACAATTA

>TN299

TGTGTCTGCCAATCTCATCAGGAGGAATTGAACCCAGAGCCCAGCCCAGGG

>TN300

AATACTACAGGTGAACTTTCTGTGTAAGTAGAGGTTCTTTGCAGAAACATT

>TN301

TTCTTAGAAGGTAAGTTACACTCAGAAAAGTCTTATCTGAAAAATCGTGTT

>TN302

AACAAAACAAAGGCATGTACTGGGGAATAGGGGATTTCAATGGGAATATGT

>TN303

GCAGGTGAATCACTTCATATTTCACAACGGTGAGGTGGGATTTGCTCACAG

>TN304

AACAAACTCAAACAAATTAGCAAGAAAAAAGCAAACAATCCCATCAAAAAG

>TN305

TTTACCTCATATATATATGTCTCAAAAAATATATATATATATAACCTCATA

>TN306

TATATATATAACCTCATATATATATAACCTCATATATATATAACCTCATAT

>TN307

CTACCATTTGCTAAAAGAAATGTGGAAGGAAAATACAGTCATGAAAATCTC

>TN308

TTCCTAACCCTGGACTCTGACGCCTAAAGAACAGATCCAGGCTTTCACCAT

>TN309

TTTGCTTTAAAACTGTCACCGCGTTAATAAATACAGCATCTGAAAATGTTC

>TN310

GACTTCATAGGAAACCAAAAATTCTAACCTTATTAATGACAATAATACATA

>TN311

AGTAGTTTTTTCCAATTCTGTGAAGAAAGTCATTGGTAGCTTGATGGAGAT

>TN312

TAGATATACAATCATGTCATCTGCAAACAGGGCAATTTGACTTCCTCTCTT

>TN313

TCAAACTGCTGTGCTAGCGGTGAGTAAGGCTCTGTGGGCGTAGGAGCCTCT

>TN314

AGATCTCACTTTTAAGAATGTATGAAATGGCTAAAAGAAACCTTTAGCCTC

>TN315

GCTGTCTAGGGTCCAGACCAAGATGAAAATGGGACTTGGCATCGTTGATAC

>TN316

GAGGCTCTGGGAGCCCCTCACAGCCAAGCTGAACACTGGTTTATCCATCTC

>TN317

ACCGAGCCCAACTGCCATAAACCAGAACATGATTCTGTTCCTGTCCTCCCC

>TN318

TCCTCAAGTGGAGGCAGTGACAGCAAAAGCAAACATTTTGGATCACACACA

>TN319

TGTCACTCCACCGATGACATTCATGAAGGAAATATTAGGGCCCAAATATTC

>TN320

AGAGACAGGGACAGAGAGAGACCAAAAGAGAGGCAGAGAGAGATAGAGGGA

>TN321

ACTAGTGAATATGGAGGAAAGCATGAATGTTCAGGCTTGCACATACATTAT

>TN322

CGTTACTGCAATACTGTGTAGAAGAAAGGCCATAGATGAGAAAACACAAAG

>TN323

CACACAGAGTGGACGCAGTCCCAACAAGGCTGCATAGAGTCTGGCTCTCTC

>TN324

ATACTTCTTGAACAAAGATGATCTGAAAAAAAAGAAAACGAAAAGTCACCC

>TN325

AAATACGCCAAGAAAAAGGGTGGCAAAGTTAAAATTTACTTCAACAGGCTG

>TN326

GTAGTCCCAGCTACTCGGGAGGCTGAAGCAGGAGAATCGCTTGAACCCGAG

>TN327

AACGAGCGAAACTCCGTCTCAAAAAAAAAAAAATTTCACTTCAACAGAGAA

>TN328

ACTGTTTTTTCTCCTCAAACCTTTTAAACGTTATGTTATTTTTAAAAATGT

>TN329

TGTATAGAATCATAGCATCTGCAAGAAAGTCAATCTTTTGCTTTACTTTTT

>TN330

GGGTAACCCTGCGTAACTCCCACCCAAGGGCCCTGCCGGGAGCCTGAGTCC

>TN331

TCAGAGCCAGTAATGAATTAATCAAAACGCACAGAGCTGCTTCTGCAGGCG

>TN332

AGTCACCAGGCAAAGCACGGCCCCCAAATCTGGAGACACAGACAAGCGGGT

>TN333

GACAGAGGGAGATCCTGTCTCAAAAAAAAAAAAAAAAAAAAAAGGGAGAGA

>TN334

CTGGAACAAAGAGTTTGAGCGTTTCAAAGTCAGGTAGGAATGCACTGATCT

>TN335

CATCATTTGTACAACCAGTGGCCCTAACTGGAAACTTGGAAAATTCCCACT

>TN336

ATTTCATGACGAACACACCAAAAGCAATAGCAACAAAAGCAAAAATTGACA

>TN337

AAAAAAACCAAACAGCCCCATTAAAAAGTGAACAAAGGACACGAACATATG

>TN338

GCAAACCTGTACATGTAGGGTGATGAAATAATCTGTACAACAAACCCCCAG

>TN339

CAAAAGCTTACTTTATCTTATGTGAAATGTAGATCTACTGAGTATGAGACA

>TN340

TCTAGCAATTCACATTTTTTAGCTGAACAAATACATTCCCATTGAAAGCCG

>TN341

CCCAGATCCAGGGGCTACAAAACAAAATCTAGATAACTTCCTTGATTTCCT

>TN342

GTTAAGAACTTTAATCTTTAAAATTAATAAATGTTCTAAGTGAAAAGAAAC

>TN343

TTTTGTCCGTTCCTACCAAAATGGAAATCCAAACCTGTGGGTAGAGGCTGT

>TN344

TTAGATGGATTTTAGAATGAACTGTAAAAGGCTAAAACTGTAAAGTTTTAA

>TN345

GAAGAAAACAGTATTTTCACAATGTAAAGTAGGCAGATTGCTTACCACACA

>TN346

TGAACTCAAAATGGATCATATACCTAAATGTAGAATGTAAAACTGTAGAAC

>TN347

CTCAACAATAAGAAAACAACCCAGTAAAAAATTGGGCAATAGTTTCGGTGA

>TN348

TATGCTCTCCAACCCTGCTACTGGGAATGCAGAATGGCACAGCCACTCTGG

>TN349

TTATTTTGCAACAATTGGGGAAAATAATTTAGGGAATTACTGTCTATCGTA

>TN350

AACTAGTCCAGCTTCCAAGAAACTGAAGGATTAGATTCTGAGAAAGAAAAC

>TN351

TCCTGTTGTGCTGCATGCTTCACTGAAGCCCAGCCACCACATGCAGGCTGG

>TN352

ATGGGCTGAAGGTCACAATGGAGTCAAGGACAGAGACCAAAAACGTAGCTG

>TN353

CAAAACAGCATGATACTGGTACCAAAACAGATATATAGACCAATGGAACAG

>TN354

TAAACTCCTTCATAAGTTCTACTTGAAGTAGGAACTATTAAGATACTCTAA

>TN355

AAGCAGAGAAAAGGGACAACCCCAAAAATAGAATGACTTCTCACAGACCAC

>TN356

GATGATAGGAGGGGAGGTAGTCGGGAAGACCTTATCTCCCAGGGAACACAT

>TN357

AAACCTGTCTCTACTAAAAATACAGAACTTAGCCAGGCATACAGAAAACTA

>TN358

GCCATTGCACTCCAGCCTGGGGGACAAGAGTGAAACTCCGTCTCACAAAAC

>TN359

TAACGAATGTGAGGATGGAAAAGGAAAATCATATCTTAGTGAAACTGTGGA

>TN360

AATAGTTTAGACCTCGGGCTCCCCTAACAGGCCCCTAGACGATGCTTTGGG

>TN361

GGAAAAGGGGTGCTATTCAACACTAAAACAAGGAATTTAATAACAATATTC

>TN362

GTTCATCATAAATGAAAAAAACAGGAAACTCATGAAGGAGTTCTGTGGCAG

>TN363

GAGATCCCAGTGCAGGAATGCGACAAAACAGAAAAGAGATTTTAATGAGGT

>TN364

TGAGTTTAAAAGGAACTAGAAAGAAAATTACAGCTGAAGAGAACAGGGAGA

>TN365

GGGGTTCCAGCATATTTATCACTGGAATCTTCAAACAGGAAAACAAAACAA

>TN366

AAGGACAGAACAAATATTTAAAGATAATGTTCAGGAAAATTCTGCTCAGGT

>TN367

GATTTCAGATTGAATCAAAAAGTGAAACCCAACTCCAAGTTGTACGTAAGA

>TN368

AGCACACCTAAAACAGAGATTTACAAAAGTTGAAAATAAAAGAATGAAAAG

>TN369

ATACACTAGGGAAAAGTAAAAAATAAAGTGGGACAAAAAGCATTAAAGGAA

>TN370

TTGTTACTGTGGCTTCCCAGAATACAAATTCTACAATTTTGTATTTTAGTG

>TN371

ACCCAGTACACATACTGTCAAAAACAATTGTGTAAATAATGTGATAACATC

>TN372

TTTTTTAAAGTTGCTTTTGCATACAAATGGATATATTAGGAAAAATAACTA

>TN373

TGAAATTACATCTTTTTATTGTGGTAAAATACACATTACATAAAATTTACC

>TN374

ATGGTGAAACGCTGTCTTTACTAAAAATACAAAAATTGGCCAGGTGTGGTG

>TN375

AGCTAATCAGTGGAGGAAGTGCAGCAAAAAACGCAGCAGGATTTCTGACAT

>TN376

GCGGGGACCATGCTGGCTGCTGGGGAAGTGATGTCTCTTGGACTGTGCAGC

>TN377

CAGTCCATGAAAGCTTCATGTCCAGAATGTTAGAGCACTTCACAAACTGTC

>TN378

ACCTCAATAAAGAAAAAAACCCCACAAAGCTAGAAGGAGCTTTGCAGACGA

>TN379

CCATGGACAGAGAGATTAATAGGACAAAGAAAAGCCTGTGAGATTTAGCCG

>TN380

ATGTCCTTACGGCTCCATGTTGTAGAATAAATCAGAGCTTCATTCAAGACT

>TN381

TTGATTGACATTTAGTTTGTTTGATAAAAGTTTGTGGCTTTTACAAATAAT

>TN382

TCGGCACTGCAGCATTCTCTTTTGGAAGGTACTGATTTAAATGCACTCTTG

>TN383

GAATTTCAGGATTAAATTTTGGTGAAACAAACTAATCACCTATATTTTAAG

>TN384

AAAATACCATTCATAGAAGGGATAGAAGGCTTTCAGTTTTGTATGTGGTAG

>TN385

TGAATCCATGCATTGGAAGTATTCAAAAAGAGCCATCTCTCCTAATTTAAC

>TN386

GAGGGAGGCTGCTAGATGTGCGCATAAACACTTCACCACACAGCACCTGGT

>TN387

TCCAGCTGAGAAAAGAAATTGTTCTAATCTAGGAAAGGGAGTAAGTACATT

>TN388

TTAATGGAGGGTATAAGGACAGAATAAAGACAATTTTGAGGCACTGATCAG

>TN389

CCAATGGCAAAGCCAAGGGATTAGCAACAGGTAGGAGGAAAAAGAAGGACC

>TN390

GGCCGTTTATATGTCGTCTTTGGAGAAATGTCTATTTGGGCTCTTTGTCCA

>TN391

TTGATTTAAAAAAATCTTGATCAGGAATACTGATGTGTGGTGTTTTTTTCT

>TN392

CTGCAGTCTCCTACTCTTACTGTAGAACTATCCATTTCTTCCTTTGATTCT

>TN393

AGACCTCGTCGCTACAAAAAAAAAAAAAAAAAAAAAAAGAAAAAGGAAAAA

>TN394

CCCAGTGGATGGAGATTTGCCATGAAACAGCAGGTCTTGGAGATACAGTAA

>TN395

AAAAGGTACATTCCAGACAATTGGGAACATATATATATGTATTAGATGATA

>TN396

TGAAATACACCTGGTTAATTATGAGAATTAACCACTATCTGAGGATTAAGT

>TN397

CAAGAGAAGAGCAGAAAATGAACAAAATGAACAGGAGGGGACCACAATGGA

>TN398

TGTCTAGGAAGCACCACAGGTATACAAGCACTGTGTTTGTGAATAGTCAGT

>TN399

CAACCCTCACAGTCCTTAAAACCACAAAACCCCCATGAAGCCTGTGCGGCT

>TN400

GGTAACAGAGTAGTGCTTTTTCTTAAATAGGTGCAGTCAACTGGCTGGACT

>TN401

AATAAATCACTTAAATCTGAGGCTGAACTGAGCATAATTTCATCTGATTCA

>TN402

AGCTTAAAGACAGAACTCATGAAATAAAGAACCAAAAACAGCAGCCGGGGT

>TN403

TCAGTTATTCCACAGGAACAAACGGAAGGAACATCCACATGCATGAAACAG

>TN404

AGAACAAAGTTTAATTTGTCAAGTTAAACAAAATTTAACATAACTTTGGAA

>TN405

CATTAAATAAAGAAACAGAGGTCCAAATATCAAATATAGGCAAAAAGTAGA

>TN406

ACATAAGCAAAGAACTAGTTCTGACAAGAAAGAAGTATTATCACACAAGAG

>TN407

TCTATGAGAATCCAGGGTCTAGATTAAGAAACAAGGGAAATCTATTCAATT

>TN408

TAGCAAGGTCAGTTTAAATGTATTCAATTCTCTAAAATCTTTTACAGTTTA

>TN409

TTTTCCACTTTTCCATAACCTTTCAAAATGCCAACAGAGACCCTCAGGACC

>TN410

TAATAAAAGGTTAAGAAAAATGTCAAATCTGATGTCATGAATCAACAGAAT

>TN411

GCAACAGAGCAAGACCCCATCTCAAAAAACAAACAACAGCAACAACAACAA

>TN412

AACACATAGGAGACTGACCTTTGAGAAGGTTGTTACAAGAGGTCCCCTAGA

>TN413

TCACAAAGAGATGGTCTGAAATTGGAACTTATGTTTAAAAGGCAAGCAGAG

>TN414

GGACATCCAAGCAGAAATCTGCTGCAAGGGTAGAGCCCTCAAGAAGAACCT

>TN415

TACATTTGGGAGGGGCCAGGAGCAGAATGATGTGGTTTGTCTCTGTGTCCC

>TN416

CACCAGCTTCCTGAGCCCCTGCTCTAACATGCACCTCCATGGAGAACAAGT

>TN417

TAATGACCAAGAAGTTAGGCCACAGAAATGAGAAAGTAAGTTCCTGCACAA

>TN418

TTTTCTCCTATTTTTCATTAAATGTAAAATTCAAGCATTAAGTTCTGAATT

>TN419

GTACCTGTGAAGGACAGAATCCAGCAAGAGGGTTAACAAGAACATGAGCAG

>TN420

GGGCGACAGAGTGAGACTCCATCTCAAAAAAACAAACAAACAAAAAAAACA

>TN421

TATTGAGAAACTGGCCATTCTGGAAAACATTGGTTGATGAACCCAGTCCTG

>TN422

CCCACCTCAGCATGCAACAACACGGAATCCAGAGCCACACATCAGAACCTG

>TN423

TGTTTGGTGAACTGGCAATTCTTGTAAGAATCACCAGGTTACAATTTGGGT

>TN424

TTACCACATTTGGAATTACTTATGTAATCTAAAACCAAAAATTCTCAACAC

>TN425

AGAAACATAATCCATGACTACTGCGAACCGAAATCCAGACTGCAGGTGCAG

>TN426

GAATCCACTCAGATCAGCCACAGCCAACAGCACAGGCTCCCTCTCACTCCA

>TN427

CAGGGTGGAGAATTGACTTCTCTAAAATAATCCAGCCAATCACTAAGCAAA

>TN428

TAAAGAGGCAAATAACAACAGCAACAATGGGAAAGGGGGAACAGTCAGTAC

>TN429

CCAGAGTGGCTACAATGTATCATCCAAAATGTCCAGTTTCTAACCAGTGGG

>TN430

GTTAAAAAATAAAAATAAAATATATAAAGTGTCCTGTTTCAACAGAAAGTT

>TN431

CCATTATAAATATGTTAACAAAGCTAAAGGAAAGTATGATAAAGATGTAAA

>TN432

TATTAGTTCATTTTCACATTGCTATAAAGAACTGCCCAAGACTGGGTAATT

>TN433

AACATAAGTTCTGTGAAAGGAAAATAAATGTTGGGACTCCAAAATCACTAA

>TN434

GTGGAATAACACAATCAGAGCACTCAATGAAGAAAAAAAAATTCTCAACCA

>TN435

GTAATATATTTGCCAATAACACCACAAAAGATGAGGGTAGAAGCAAAGCTA

>TN436

GAATTTAAAAATATACACAATGCAAAAGAAAGCAGCAAAGGAACAAAATGA

>TN437

AGGCATGAGACATATGGAAAACAAAAAGGGAAATGGCAGATGTAAACCCAA

>TN438

CCCTACTAATTCAATATTAAATGTAAATGGATTAAAACTCCAAGCAAAGGC

>TN439

AGGAAACAGAGATATGCAAACAGCAAACACAACAAAGCTAAAGTGACTATG

>TN440

CATAAAGGGAGAACTAGAAAAATCCAATATTCACAGAGATTTCACCATGCC

>TN441

CCTTTCTTTGACCATAATGGAATGAAATTAGAAATGAGGCAGGAAAAACTC

>TN442

ATGTCTATAAAGAAAAAAAGATATCAAATCAATAACTTAAACTTCTATCTT

>TN443

AAGGCAGTGGAAAAAGAGCAAACTAAACCTAAAGGAAGCAAAAGGTAGGAA

>TN444

AATATAAAGAAACATTGTCCATCAAAAAAAAAAAGGCAACTTAGAAGAAAT

>TN445

CACCAAACTGAATCCAAAAACATTAAAAAATGAAAAGAATTATGTACCATA

>TN446

CCATTAATATAGCAAATGAATAAAAAAAATTATATAAGCATCTCAATATAT

>TN447

GCAGAAAAAACATTTGGCAAAATCCAATACTCTTTCATGATAAAAACATTC

>TN448

ATTGCGTTCTTACACACCAGCAAGGAACAATCCAAAAGTGAAATTAAGAAA

>TN449

GCAATTCCAGTTACAATTACATCAAAATAATAAAATACTTAGGAATATGCT

>TN450

TGAGCTGACTCTAAGATGCATACAAAATAACAGGGGATTCAAGCCAAAATA

>TN451

TGTGCACAGCCAGAGATCCAAGTACAAGCACACTGATAGGTTCTCTTCCCC

>TN452

CACTTAGCAATCATTCAGACAGAAAAAAATGTAAAATTAGGAAATCTCTGA

>TN453

ATCAAAGAACGATTAGACCTAACAAAAAATGGCTGAATATAAAAGAAATAA

>TN454

CAAGAACCATAGTTAAAATGTCCAAAAGAACAAGTTACAAGTTTCCTTAAA

>TN455

CCTCAGGAATGTGGAATACAAATAAAACATTCAAGAGAATGAGAGGAAAAG

>TN456

CCATAGACTTGGAGAAAATATTTGGAAAAGGCACATCTGATAAAGGATTGT

>TN457

TGTCCAAAATATACAAGGAACTCTTAAAACCCAACAATAAGAAAACAGCCC

>TN458

CGGTTCTATTTATCCAAATGACTTGAAACCAAATGACTTGAAAACTTATGT

>TN459

CCCCACTTTAGAGTCAGCTGAGTAGAAGCAAGGGTAGCATGGATACCTATG

>TN460

TCGTGACCACCATTCTGCTTTCTCTAAGAGTTTGCTGTAACCCTCAAAGGG

>TN461

TTTAAAGACAGGGTTTGGAATGCAGAAGAGGCAGTGGGGCGAGGCATTGTC

>TN462

GTGCTTTACCGGCATCCAGCTAAATAAATGCGTGTAAGGCACAGAATGGGA

>TN463

AATCATCCTCCCTGAAGGAACACCAAATTTAACAACTATCTACACAAAAAT

>TN464

AGCTAAAAAATTAAACTGACATATGAAGAATGCATCAGTCTCAAAAGTAGA

>TN465

AAGGGAATTCTTCAATTTGAAAGAAAAGGATGTTAATTAGAAAGAAGAAAT

>TN466

CTCCTCTATTTTTCATACTGAAGGGAAAAATGAAATCCTTTCCTCTCAGAT

>TN467

ATGGACTGGAAGAGTCACTATTGTTAAAATATCCATATTGCCCAAAGCAAT

>TN468

CAAATCAAAATGGATTAAAGACTTAAATCTAAGACCTCACACTACGAAACT

>TN469

TGGAAAGGCAACACACAGAATGGGAAAAAATATCTGCAAACAACCCACCTG

>TN470

TTCCAAAAGAAGACATATAATTGGTAAATGGGTATATGAAAAGGTATTCAA

>TN471

TATATACATATATTTATTTATATATAACTATGTGTGTGTGTATATGTGTGT

>TN472

CACGAAAATTAAAAATTAAAAAACCAAATCGAGTATTTTATATTTTTATCT

>TN473

GCCAAATATCTGGCAAAGAATAGCTAAAAGGAGATGTGAGTAGTTACAAGA

>TN474

TAAGACCATTTATGCAAAAGCGCTTAAAACTATACAAATGCACACTTGCTA

>TN475

CATAGTAAGACTCTTTAATACAAGAAACTCTTTAATACAAAACTCAAGCAT

>TN476

AAACACAATTTTATCAGTGCTTTCAAAAGGCACTGATTGTTGAAGCCCTAG

>TN477

TTATTTAATATTTTTAAAAATTGGAAAATGAAGAAAAATGGTATCAGAAAT

>TN478

GGTTGTAACTTTACACGAGTTATTTAACTTCTCTAAGATCATCGAATATAA

>TN479

CTGTTTTTTCACCTGTACATTTTATAAACTCTAAATACTGATCAAGTATTT

>TN480

AGAAAACCAAATCCTAAGTCAGTAAAAAGCATTAACTATTTCTATTATTTA

>TN481

TCCATGAGACACATATTATGAAGGGAACTCAAGGTACAGAAAAATGTTAAC

>TN482

TCTTGAAGAAATTAACTAAATTTAGAATAAGACTAATCTGTCATAATTAAC

>TN483

TCTGGAAAACTATGGCGACAGTACAAAGATCAATGTTTGCCAGGGGTTCCG

>TN484

CAACACACACTTTAGATGTACAGATAACAGGCCAGGCGCGGTGGCTCACGC

>TN485

GAGGCTGAGGCAGGAGAATCACTTGAACCCGGGAGGGTGCAGTGAGCTGAG

>TN486

TTCAGAAAAATCAAGAATTTAAGAAAATACTTGTTATGCAAACAAACATTT

>TN487

CAAAAACTATCAAACAGAGGTTGCAAAACAAAGTCAGGATCCAAACACAGC

>TN488

TCATAAAAATACAATTGTTACCACTAAAAATCCATTTTGGGATCGAGAAAG

>TN489

CCTTTCTAGCTATTTTGGACTATACAATGCATTGTTACTAACTATAGCTAC

>TN490

AATTGTTATATCCTCTTGCTGAATGAATCCCTTTATCATTATATAATGACC

>TN491

GCAAATTGGAAGGGTGAAAAAGCTCAATAAGTGAGGGCTCATGAGCTGAGT

>TN492

GACTATACCATGTCTGCTGTAGTGTAAGAACAAAGTATTTGCCAAATAAAT

>TN493

GGACTGCAATTTTAGCTCTGCTAAGAAAGCACAATCTGAAAGGATTTCCAC

>TN494

CACACACATTTTGTATAAGCCTTATAAATCTAGTATGTGTTATGCAGGCAA

>TN495

CCCAGCCCCTAGGGCCCCCTCCCAGAAGAGTGCCCCCATCCAAGGTGCCTC

>TN496

TAATGAAGGATGTTTCTGTGAAAATAAAAGAAAAGCCAGCAGGGCACAGTG

>TN497

ATAACTGTGTTGCCATGAAAATAAAAATACTCAATAAGAGTTTTCCAATCC

>TN498

AGTACAATAACTTAAGAGAAAAGAGAAAGGGGTTTCTTATGTCCAGAAAAG

>TN499

ATACAACATTTAATAAAAACAATTGAAATTGTGACCGATAATATGGAACTG

>TN500

ATTCAAACCATAGCAAAAGTGAAAGAAGTCCTTTGGAATTATCTGGGACCT

>TN501

TATGGGAAGTTCCAGGTCCGTTCAAAAACAAGATTTCATTTAGGATTTGAT

>TN502

CTAAAGAACTAAAGAAGCAAAAGCAAACCAATCCAAAAGCTAGCGGAAGAC

>TN503

CATTTTATGAGGCCAGCATCATCCTAACACCAAAACCTGGCAGAGACACAA

>TN504

TCATCACTGATCATTAGAGAAATGCAAATCAAAATCAAATGAGATACCATC

>TN505

GGGCTACAGGGAGGGGAAGCAGGGAAAAAGAAGGAGCGGCCAGAACCTGGG

>TN506

AGGGGGTCTCACAGAGAGCTGGGAGAAAGAAGCTTCCAGCCCAAGAGGCTG

>TN507

CTAAATAGGATGGCTACAAGGTGAAAAGCTACACATTTCCCACAAGGAAAT

>TN508

GCCTCGGAACCTGTGACTATGTGAAAAAGGGGAAAGGGGGATTGTGGTTGC

>TN509

TGCTCAGAGCTCAGGACCTTTCAGGAAGCAGGTGCTCAAAACAGACAGGCT

>TN510

GACTCCTATCTCTACAAAAAATTAAAAAATGAACTGGGCCTGGTGGAGCAC

>TN511

GAAACCCCATCTCTACTAAAAATACAAAAAATTAGCTGGGTGTGGTGGTGC

>TN512

CTATGAGCAGGGACAAAAGTCCAGGAAGGGTCACGTGAGGCCATTGGGGCT

>TN513

GTGGGGGGTGTCACGTGGAGTCAGTAAAAGATTACAGAGGCACCGAAGATG

>TN514

TGGCGGAATGCAAAAAAAAAAAAAAAAAAAAAAAAAAATCCCCCAAAATAA

>TN515

CCTCAAACTGGCAAAATTAGAAGCTAATTCAAACATAATTATAAAACAGTT

>TN516

TATTATTTTGGAAGAAACCATACCCAAGTTAGGCCCCATGAATACAGACTC

>TN517

AACCTGAAAACCTGAAAAGCCCATGAAGGAAAGAACAAAGTAGGCTGGACG

>TN518

GTTTGAAACACTTATAGCAATAAAAAAGAATGAGTGAAAATTGATTAATTA

>TN519

AGCATCAAACTCAACAATTAGAAACAACACAGCAAAATTAACTGAAGGAAA

>TN520

TTGTAACAAACTTTAAATAGCGGATAATTCCAATAATTTAAAAAACTCTTC

>TN521

GTCTGTTATGGAGAATGGTCTAAGCAATAAATAAATACATAAAGAGAAAAA

>TN522

GCTTAAATACAAGTGCATACATCATAAAACCTTTAGAAGAAAACATAGAAG

>TN523

TGTGCATCAAAGAACACAACGAAGAAAGTGAAAATACAGCCCATAGAAAGG

>TN524

TTTTATGGTTTTAGGTCTAACATTTAAGTCTTTAATCCATCTTGAATTAAT

>TN525

ATGCGGGCTCATTTTGGTTCCATATAAACTTTAAAGTAGTTTTTTTCAATT

>TN526

TGGACTTAGGAGTCCCCAGTGGAAGAACTGGGCCATCTCCACTGCTGTGAG

>TN527

GAGCCCCAAAATAAACATGAGACACAAGCATGTTCTTTCACAGGAGGCAAA

>TN528

CACCAAGAGACAGAGTGACAGCCGCAAGGACCGAGGGTGGAATAAGGAAGC

>TN529

GTAACCCATGGCAGATGAACAAGAGAAAAACAGAAGTTTATTAATCCACAC

>TN530

CTCAGATGGGACCCTCTAGTTGCAGAAAAACAAGCTCAGAGTTCCCACTGA

>TN531

TCTTTTGACCACAAAGCACACCAGGAACATTGCTTTACGTCAGGACTCAGC

>TN532

CAAACATTCCAGGATACTTCCAGTCAAAGTTTCTCACGAACGAGGGCCCAG

>TN533

GAGTTTGAGCGTTCTCTGGGTATTCAAGGTGCCAGTCTGCTGTTGGATTTG

>TN534

TCACAGAGCAAGTTTTACATTTCGCAAAAGTCCAATTTGTAATTATTTTCC

>TN535

CCACTTGTGCCTTCCTAGTATACAGAAATGCAATGGGTTTTTGTGTGCTTA

>TN536

TGAGGACTTTTTAAATTGCAAATTTAATGTCCTTAATAGATACAGGGATAG

>TN537

CAGTGGCTCATGCCAGTAATCCCGGAACTTTGGGAGGCTGGGGTCAGAGGA

>TN538

TTTAAAGGAACTGACACTGACAAGGAAAAGGCTCGTTAGTAAAGATCTTGT

>TN539

AAGAATAATATTTTCATTGCATAAAAATAAGAAAATTCAGTAAACTATAAA

>TN540

TGCAATACTTTTGAATATAGCATTTAAAAGGGACTATTTTTTAGAGAAGTT

>TN541

TAGATTTATATAAAAATTGAGCAGAAAGTACAAAGTTTCCTCATACCTCCT

>TN542

TATGATTTAATATCTGATGGGGTGTAATAAATATTTAGTAGCACATTTTTC

>TN543

TGGTTTGGTTCAGAAAGACTTGGGAAAATGAGTCTTATTTTGAGAGATAAA

>TN544

AATTACCCCACATCCTTCACTCTAAAATGCAGTTTGCTGCCAGAATTATGC

>TN545

TAAAGAACATCTTCCTTCCCATCTGAAATGGATCCATTTTGATTGGAGCTA

>TN546

TGAAGTGGAAAATATTCCAAACATGAAAGAGAATTTTCATTAGGTAGAGTT

>TN547

AGACAGATCCAAATTTTTCAAAATGAAAATTCAATATTCATGAGACTCAAT

>TN548

GGCACTGCAGAAAAGTGATTGCAACAAGGACAGCCTTGAAGAAACAACCCT

>TN549

TTGAGACTTGGGGCTGACCTTTCAGAAATGCAAAAGGAACACCAACATACG

>TN550

CCCTGCTTTCCTCAGTCATAAACAGAACAATGATCAAAGCAATGGTCACAA

>TN551

ACAGAGAACCTAGGGAAATTATCCTAATTAATTACTAAGTTACAATTTTCC

>TN552

CCGCACACCCCATGCTCTCCTGGCTAAGCGGATTCAGCGCTGGCCAACAAA

>TN553

TAGGGAAGCGGGAGAGAGAGGGACAAAGGGAGAGGAAGACAGAGAGAGGGA

>TN554

ATATTACATTATAAAATAGCCACTTAATATGAAAAAGCAATAAAAGAAAAT

>TN555

GGAGGAATAATAAGGACATATACAAAACAAAAAGTGGGCCAGGCGTGGTGG

>TN556

ACTTAGATTTAACCAGCATCTACAGAACACTTCACCAAAAACAGCAGAATA

>TN557

TATATTGTCTGAGCACAATAGACTTAAATTAAAAATCAGTAATAGGAAGAA

>TN558

ACTTGGGAAATTCACAAATATGTGAAAATTAAACAATACACTCCTAAATAA

>TN559

AAACAATAGAGAAATCAACAAGACCAATGGTTAGTTCTTTGAAAAGACTGA

>TN560

TAGACACCATATGAATAAAGTACAAAAACCATGTGGCTTCCCAATAGACGC

>TN561

AATAAATAAATAACTTTTACAACTCAATAATAAAAAACCCAACTGCAGATT

>TN562

CGAAACCACAATGAGACATCAGCTCAAACCTGTGAGGATGGCTAGATGAGA

>TN563

GCAACCATGTGGAACACAGTCCAGCAATTTCCCAAAAAGGTGAACACAGAG

>TN564

AGGCTTCCTGAGAACTCACTGCCACAAGAACAGCGAGGTGGATTCCACGCC

>TN565

AGCACCAGACAGAGCTCCCCGGGACAAAAACTGAACACAGTTCAGAGACAC

>TN566

ATAACAGGCCAGGGGCTGGCAGACGAAGGGCTTGAGTGAATGACAGCTCCT

>TN567

GTTTTAAAAAAATGTATGCAGCTCTAAAATTCAATGTTTTTTATTTCTCAT

>TN568

GTTCCTGATTACCCAAAACAATCCTAAAAAGAACGAAGCTGGAGACTCACA

>TN569

AAATAAACCCTCAAACTCAAGGTCGAATGGGCTTTGGCACAGGTGCCAGGA

>TN570

GAAACAGGACATCCACACGCAAAGGAATGAAATTGGCCCCTACTTTACAGC

>TN571

TAAAACGGCAGAGCACACAATGGGGAAAATATTTACAAATCATGCATCTAG

>TN572

CTGAGCAGCCCAGGTCAAGCATCAGAAAATCAGGGAAAGGTTCTTTTTTGT

>TN573

GGATCCAAAACGCAGCAGGTCCTTCAAAGGTTCACCCCATTTAACTTGCAA

>TN574

CAGGTAAGATTCTCAAGAGACAAAGAAGAATAATGCTCCCTAAAAGCTGCC

>TN575

TGAGAATGTAATGTGATATGAGGCTAACCTCAAGCTGCAGATGTAGAGAAT

>TN576

TGTGTGTAATTAATAATGCCACAGCAAACAATCTTCATACTTTAAAAAGAA

>TN577

GGCCTCATTTTCAAAATTAAAAATTAAAAGAGAAAAAGAAATCCCCAAGTC

>TN578

GGGAACTGCTGCAAAGAACATCCCTAAAAAGAATATCATGGTCTGGACTTT

>TN579

AGCTCTCACAAAGACTAAAATTGGGAAATTTGCCTAAACTGGTGTTTAATC

>TN580

AGGCTGGACGCTGATGGAGCCACACAAGCCGCAGCCCCACTCTGAGGGCCA

>TN581

CCACATTCATACAGGGTCCTCATTTAATCCTTCTCAAAATCCCGTGAGGGT

>TN582

AATTTAAAAAGGACTTGAAATTTGAAAAGAAATTTAAATTTTAAAAGAACT

>TN583

TCAGTTACTGAAAGGAGGAGGGCGGAATGGACATGAGGACAACTCGTTTCT

>TN584

TTTGCATTTTGAAAACATTGGAAAGAAAGCTCCTTTGCAAGTGATTGAAAA

>TN585

GAAATAGATCTGGGGTCCTGTCACCAAGGCGGTGTATAAACAGTTTACAAA

>TN586

GGTAATAAATGTGGCAGGAATGGTGAAATTAGAAGATCAACTATTTTGCAA

>TN587

TTATAAAAACTGTGATAAACAAAACAACCCAAAAGTAGGCACGGGACCGGA

>TN588

AACAAATGGAATATGATCCAGCCTTAAAAAAGAAGGGAGTCTGGACACAGG

>TN589

AAGCCCCAGTTCTTTCAATCTAAATAAGAAAATAATGCAAAATACGCCAAA

>TN590

TTAAACATTATGGATTAATTCATTTAAGATAAACAATACACTCCTTATATG

>TN591

GCCATGTTAAATTAAAGGACTCAGTAATGTGCAGGAATTCCTTGCGATAAT

>TN592

CCCGGGCTGTACTGAATCCCCACCCAAAGACACTGCGAGGCGGCAAATGGT

>TN593

AACTGAATATTTTGCACTATTGTAGAAGATAAATTGATCTAATTATTATCT

>TN594

ATCTCAAAAAGAAAATAAAAATAAAAAATAAAAAAAGAAGCATTTAAACGG

>TN595

CGTCCTGTCCTCGGGTGACGCTGGGAAGAGTGGCGTCCGCTGCCACATGCC

>TN596

GCAAAAAACACAAAACAAAAAATAAAACAAAGACAAAAAGAAAAATCATAA

>TN597

AGACCTGGAAGAAAACCCACATATAAACCGAAATGAAAATGAAATAGAAGT

>TN598

AAAGCCCCAACAGTCAGGATGATAAAAATCTAATTCCCATACAGAGAATTA

>TN599

TGAGAAACAGAGCAAAAATTAAAAAAATTAAAAAGTTAAAATCGCTTACTT

>TN600

GAATCAGACTGTGGCTGTTTAGAAGAAATCAAGGAGAACTGAAAACAAGCC

>TN601

AACCTGAGAGGCAGAGGTTGCAGTGAGCCAAGATCATGCCACTGCACTCCA

>TN602

GAAACTAATAAATTAGAAAACAGAAACATAGAACTAATTTATAAATCAAAG

>TN603

CCACCGGCCCCAGCTCCAACCTCTGAACCCCCTGGCCCCAGGTCCAACATC

>TN604

AGTATTAAAAAAAATTAAAGAGAAGAAAGCCAGGAAGCCAGCGCACGGTGA

>TN605

TCACTATGCAAGAGAAAAACAGTAAAAAACATACCGTCATAAAAATACATT

>TN606

AAGCCCCGCCCAGACACTGATGGGTAAAGTCTATACCACGGGCGTCTGTGC

>TN607

CAAACTGCCTCAAAGGAAGAAATCTAAATCGGATACCGGATACCTACAAGA

>TN608

CCTGTGAACACAGAAAGTCCAAGAGAATCCATAGCTGCCACCCCTGTGAAC

>TN609

ATCCACAGCTGCCACCCCTCCCCCGAAAACACGGAAAGTCCAAGAGAATCC

>TN610

ACAGCTGCCACCCCTGTGAACACAGAAAGTCCAAGAGAATCCACAGCTGCC

>TN611

GCAGACTCATGAACTGCACACTTTGAACATCAGTGATGCTGCCCAAAGCCA

>TN612

TTCAGAACCACTCCAGAGACTCCAGAATGTCCACATCTCCAGGGCATGTGG

>TN613

AAGGGAGGCACAGCCTTGGAGCTGGAAATGTGGCCTCCTCCCCCGGCTCCA

>TN614

CCATACGGGACCTCAACGTTATAAAAAACTGAGTTGGGCTAAAACACAACA

>TN615

TGAGTCTTAAAAACTGACTTAGTGGAAAAGTAGTTAGTGTCTCACCATAAA

>TN616

ACGGGCAATAAAATATCACAAGGCAAATGAGCAGGGCAAGGTCACAAGGCC

>TN617

GGAAATCGAGTAACTATGTTTTACGAAAATAAATGGCAATAAGAACAAATG

>TN618

GAGCTTGGACACAGCTGAATGTGTGAACTCTCATATCAATAAATCAGCGTG

>TN619

CCACAAACTGGAGGTCAAAGGAAACAACTGCTACTGAATAAAAACAATGAA

>TN620

TTTACAAAAACAAGGAAAAAAGGCCAACACTCTCCAGGTCTTTTGATTAAT

>TN621

GACTTGATGTAAGGTAAGCATTTCCAAAACCTAACGGACTGATCAATTCCT

>TN622

TCCTTCTTGGACAGATTAAGTACGCAATGAAGCATATTCACCCAGAGGAAT

>TN623

TGATGTACAATGTCAGAAATTCTGAAAAACTTGGAAAATCAAGAAAATAAT

>TN624

CATGTGGTTCATTATACAGTGTAACAACATGCTCACCCACCCCCCAAGTAT

>TN625

ACTCAATGACTCATGCAGAGCAAACAAGGATGCAGCTAAGAGGACGGGTCC

>TN626

CAGCAACTTCAAGTGAAATCAGTGTAAAATCCAAGGACAAGCACGAGCACA

>TN627

CAATGCACAGCCCAGCTCTCAAGACAAGGGCAATCTGGCCCAAAAATGACA

>TN628

ATCACGGACAATGTGAATTGTCCCCAAATCATCCACCAGTGACACAAATGC

>TN629

AGTGGGTTTGCAACACACAGAGGACAAATGAAGGACAGATGAAAAAAATAC

>TN630

TCCAACTCTGAGACGCAGTGACAAAAACGAAGGGTGCAGCCGGAAGGGTGG

>TN631

AATGTCTGAATGACAGGAATTCTGTAAACCGAAGATTGAAAGCAAAGAGGA

>TN632

TACACCCCGAAAAACTTTTAAGTACAAGAATTAAGGTATACTTGACCTTCA

>TN633

CATTTCCAAGGCACAGCACTTCAAAAATATGTTGTTCATGATTAAAAAAAA

>TN634

ATAAAAATAAATGTATTAAGTATGAACAACAAAAAAGCTAGTAAAGGTTGA

>TN635

TTTCCCTACTCTATATAAAGCCACCATTTATCAAATGCCTACATGGACCAA

>TN636

TCAGGTTTTATAGATAAAGATCTATACCTTATAGATAAAGGTATCTATAAG

>TN637

ATGTGTGACTGTACATAGAAAATCTAAGAAATCCACAACAAAAATGTAAGA

>TN638

TGGAATACACTTTTTAAAAAACGGTAAAACTTTTCTACGTCCTGTAAGTAC

>TN639

TGGAAGGAAAAATATAAAAAAAGATAAGGCCAGGCGCAGTGGCTCACGCCT

>TN640

GGATGGACATACATCAATGGAATAGAATTGAGAGTCCAGAAATAAACCCCA

>TN641

AGCAACAGAAAGACAATCCAATTAAAACTGGGCAAAGGACGTAAGCAGATA

>TN642

CATCAACTGATGAATGGAGAAACAAAATGTGGTCTATTCATACAGTGGAAT

>TN643

GCCTCCCAAAGTGCTGGGTGTTTTTAATTTCAAATTCCACTTGTTCTTTGC

>TN644

AAGCAAGAAAATTTCTAACAGGCACAATGGACAGTGCATTAGCTTTCTAGG

>TN645

AAAGACCACGGGACAAGAACCTCAGAAGCCTGTCCCTGAGTGGGCACAAGC

>TN646

CTTGGCCACTTGGAACAGGAGGAAAAAGTCTCTGCAGCACCTTCTCCACAA

>TN647

GGCCTCTGGGACAAACACGTGATAGAAATAACCCTCCTACAGGCCCCGCTG

>TN648

AAGCATCAGATGACAGAGTTTTCTGAAACAAGGCAGCTGATCCCAAAGAAA

>TN649

TCAGGGAGGCCGTTTCAACCGTGAAAACTTGGGTTTCTGCCCCACCCCAGC

>TN650

ACAAAAGAGGACTTGGAGGAAGAGGAATGTCCTGGACACCCTCAAAGACAA

>TN651

CCTGCAGATATGGACACGATTCTGTAAACACAGCCTGTCTTCCAACGGGCC

>TN652

AAACAGAAAAGTGATTAGATTTTCAAACAAGAAATAATCATTAGTAGAAAT

>TN653

TTGAGGAGTTTCGTAACAGAATGACAAATGGAAAAATAATTGGCTAATACT

>TN654

TAAGCAGAAGTGGGCAAGCTTTCTGAAATATCAAAAAGAAAAAACAAAAAA

>TN655

ACCAGGGAGGAGGCTCCGCCAGCCCAACAGCAAGACTCCGCCCAGGCAGCA

>TN656

TCTCAGAGGCTTGCAAAGTAGGCCCAAGAAATATCCTCCGGTGCTATACTT

>TN657

AGCTTTCATTCACTTTAATTTGGTGAATGGAGAAAGGTTTTTTTTTTAAGT

>TN658

TGATTAGGTACATTACAGATGCTATAATAATTTCTTGCATAGAGAAGAGCA

>TN659

GATTGATCAAACTGGGCTGTGACAGAAAAGGAGAAATGTAGAATGGCTGTA

>TN660

GTGGTAGAAGCAGAAACTTGATGGAAATTCATAGAATTTCGAGGAAAAGTT

>TN661

CATACCTGTAGTCTCAGCTACTCAGAAAGCTGAGGTGGGAGAATCACCTGA

>TN662

CTCTACTAAAAATACAAATACAAATAATAATAATAATAATGATAATAATAA

>TN663

GAGTGAGACTCCGTCAAAAAAAAAAAAAAAAAGGCATCAAGTCCTTCTATC

>TN664

TCCCGCGTTCCGATCCCGGAAGGGAAAACCCATGATCCTGGCGAGGGTCCG

>TN665

GGCGCTACCCAGCGCAGGGTTTTGTAACTAAGCCTCCCCCGGCAGCGGCTG

>TN666

CAAGGCCAAAAATGGCGAAATGATGAAAAAATGGTAATTCTTGGCAGGTCA

>TN667

AAATTCCAAATTAAATATTAAAAGCAAAAATAGGCTTCGTGGAATTTAAAT

>TN668

AAGGAAATAATCATGAATTTACAATAAGTTAGCTAAGTGGATGTTCCCTCC

>TN669

ATTACTTAGACGTCCAAGGCCACAGAATAGGGATTGAAAAGCAGTGTGCCT

>TN670

GACCCTGTCTCTAAAAAAGAAAGAAAAAAAGCAAGTAAGGAAGCAAGCAGT

>TN671

GAGCAAGACCCTGTCTCAAAAAGAAAAAAAATTAATTGCCAAGCAAGTGAA

>TN672

AAGCCCATCTGTGAAAAATGCTAAGAAGGCCCCCATGAGATTTATTGAAAA

>TN673

TAGGGAACATCTTCTTTCATGTTTGAAAGCAGAGACATCAGCAACCATGGA

>TN674

AGTTTCTCATGCTCACGTCTAGAAAAACAAAACATAATCCTAGAAGTAATG

>TN675

AAGAATGAGGCAAACGATGAAATAGAAAATCCTTCTAACTGCAGTCTTGAC

>TN676

CACACACACGCGATGATGCACACACAAATGCACAAACACACCCCCACACAC

>TN677

CGAGACTCTGTCTCAAAAAAACAAAAAACAAGAAACAAAACCAATCCACAC

>TN678

GCTAGCAAGGAAATAAAAACACAGGAACACCAGCCTGGTGAGCCACAGTTG

>TN679

CACTCCAGCATCAAAAAACACTTAAAAATGGTGCGCTTCCATCGGAAAACC

>TN680

TATGGAAGTAATTTACACTAACCATAAAAATTGAAGTGGCACATATAGAAT

>TN681

CTTTTAGGGCAGGCCTGGTGGTGACAAAATCTCTCAGCATTTGCTTGTCTG

>TN682

AAATTTGAATGTTTCTAGCATAAAGAAAAGATGAATGAGGTGAAAGATATC

>TN683

CCAGTTATCCTGATTTTATTATATGAATGTAACAAATTATTGTGTGCACCT

>TN684

TAGAATTCCATAGATAACCTCTAATAACTGGTAGTATCTTGATAGAATTTT

>TN685

TGATGCAATTGACTAAAGTTACAACAAAGATTCTGCCTTTTCTAGGACATC

>TN686

AGAGATGTATTTCCAATTCAGTTTTAATGGTAATATTTAGTGTTCATCAAA

>TN687

GATATGGTTCTGTAGGAGGAGATGGAATGGAAATAAGAATTTAAGGACAAA

>TN688

TAGTTAGATTACAGTTGATATGTTTAAAAGGGTGATATAACAGTTTTATCT

>TN689

AACCCCCTCTCTACTAAAAATACAAAACAATACAAAACATTAGCCGGGTGT

>TN690

CTTAAGGTGAAGTATCTCAGCTTGGAAAAAAGTGAAGTTTGGTTTTGCGAG

>TN691

GGCAATTTATGAAGAAAGGAGGTTTAATTGGCTCACGGTTCCTCAGGATTT

>TN692

AAGGGGGAGGTGCTACACACTTTTAAACTACCAGCTCTTCTGAGAACTCAC

>TN693

TCACTATCACAAGAACAGCAAGGGGAAATCCACCCCTGTGATCCAGTTACC

>TN694

AAGCAGCTGGAGACATGAAGCCAGGAAGTCAGAATCTTAGCTGCACCCCTG

>TN695

CCATGCCCCAGTGTTATCAGGAGAAAACAATTTAAACAGGGATATGTTTTT

>TN696

GGTGGGAAAGCTCTTAGAATCCTTGAATGTCTAAAAATATGTTTATTCCCA

>TN697

TATTTTGTTTTACAGAACCAAGGACAACATCTTCCTTGCACTTCCTGTGGC

>TN698

TCCCTTGTGGTGTTGTAGATTAGATAAACTACATCTATTACAACCATATGA

>TN699

GAGTGGCAAGCAGCCGCAAAGCAATAATTATGATCAATACAACTTTGTTGG

>TN700

GTGCCCTGGAAGCAGGTGCCCCCCGAACAGGTGTACCCGGAACAGGCTTCC

>TN701

TTATATAAGTTAGAGAACAGTATATAAAAGAAGAAGCATTAAATTATCACC

>TN702

GCAGCCGGAGGAACATAAAGGGGAGAAGGTTGGCGGGGAGCTAAGAACCGG

>TN703

TCCCATCAACCTTGGAATGAGCAGGAACTATCTGGGGGCCCAGCCAGGGCT

>TN704

GCATTTGGTGTGCTGAGATTTAATCAAAGCTTTGATAAGGACGGGGATAAC

>TN705

TGGCTGTGTGTCGTGTAAGTGACAAAACGTGATAATAGAACACATGTAATT

>TN706

TGGTTTATATTTCCTTTTCAAAACGAAAAAAAAACCAAAATGCTGAGACAT

>TN707

TTGATAAAGTGTTTATATTCATATAAACACTTGTATTTTGTATTACAGAAA

>TN708

AACCCCGTCTCTACTAAAAATACAAAAAAAAAAAATTAGCTGGGCGTGGTG

>TN709

CGGAGCGGATATATCTGAAAAGCAAAAAAGTAGATCATTAGAAGCTAAAGA

>TN710

ACAGCAAAATCCTAAACAACTTGGTAAAGAAAATATAAATTGGTTATAGAT

>TN711

AATGATTTGTTTTCTTGGTGACTATAAATGATATAAATTGATTCAACAAAA

>TN712

GTGGAAAAGTAGGCGCATCCTCAGAAAGAATAAGCCTCACAAAATATATTG

>TN713

ATGAAAAATTTATCCCCCAAAAGAAAAAAACTAAGAAACAGTTTACACAGA

>TN714

AATTTGTTTGCTCAATAATCACATTAAAAGGTCCCCAGACTCTAGGAAAAC

>TN715

GGAGAAATGTTAATATATTAATAGGAAGTGGATAAGTCTTGAAGAGCAGAA

>TN716

AGGCAATTTAGACTGACAGTCACATAAAATGTACGTTAACTGCTTACAGGA

>TN717

CATTTCAAATCCTGCTTTAGAATGGAAATAGTAGTTTCCATGTTCATACAT

>TN718

CCACAGCCAAGAATCATCCAGACCAAAATGCTGAAAAAGGCTTCATTTGTA

>TN719

AGCAAGTCTTTATTAGGGATTTTCAAAAGGGGAGGGAGTATATGAATAGGT

>TN720

TAACAAACAAAATGAAGAAAAGTTGAAAGGTTCAGAGATGGTTGTTATTGA

>TN721

ATAATCCTTTGGGTATATACCCAGTAATTAGATCACTGGGTCAAATGGTAT

>TN722

TGTCAGATTATCTTTGTATTTTGGGAAAATGTTAATTGTGGTCATGGGATA

>TN723

AAGTTTAAGCCATTATAGTTCCATGAAGTTCATAATAGGCTCTGAGCAAAT

>TN724

ACACCGAATCCTGCAGTGTCAGGAAAAGCTGGAGAATGCAGCTGCGGGCCG

>TN725

GATCCTGTAGTGTGGACGATGGTGGAAATTGAATTAAATTTATGCAGACTC

>TN726

TAGGATGGCTGTTACTGAAAACCAAAAGAAAACAAGTGTTGGTGAGGAGGG

>TN727

TTGGTCCCATATCTTGGATATGATGAATAGTGCTGCAGTGAACATGATAGT

>TN728

AGGACAGCTGTGAGTTGTTACAGAGAAAGGACGCGGAGCATGGTCATCAGA

>TN729

GTGACCACGCATGTCGTCCAACAGGAAGCTTGTGAGAGACACGGTGCCCAG

>TN730

TTTGTGGAAAGGTGGTGCTGGCAGCAACCGAGGAGCGGTTTGGGCAAAGTG

>TN731

GGTGCCAAGAGAAAACAATTTCAAGAAGGAGGGTGTTTAACAGCACCACGT

>TN732

AAAAAATCCAATCCCATTAATGATAAAAACTTTCAGTGAGCTAGGGATGGG

>TN733

GGAGAACTTCCTGAACTCAATAAAGAAAATCTAACCACCCCCCAACAGCTA

>TN734

AAGTAAAAGTTGAACAAAATAGCATAAGATCTATATGAGGAAAACTACAAA

>TN735

ACAAGTTAACACAGTATTTAAGGAGAACAAAGTCACAGAACTGACAGTTGA

>TN736

AACAAATAGACAAATAGATCAATGGAACAGAACAGAGGGTCCAGAGACAGA

>TN737

ACAACCAACGTGCCTTTAAAAGATGAATGAATAACCAAACTGGCCTATCTG

>TN738

GCTCAACTTTTCTGTAAGCCTATAAAAAATAGTATTTTAATTAAAAAATAC

>TN739

TTCAAGTAGCAGGAGTGAAGGGGTTAACTGGCAACCCTAATTAGAGTGAGG

>TN740

AAATGAAAACAGCCCTGGGCTCCAGAAACACTTTCAAGGCTCTCCATTATC

>TN741

AAGTTTTTACCTTTAATGTGAAATCAAATATGGAATTCCAAACAACCCAGG

>TN742

ATAAGTAAATGCAAATTGTCCTCTGAATTCATTAGGAAATTATTTTAAAAG

>TN743

TCCGGGCCTCGTCCAGAACAAAAATAATCCACGGGCTGAGTCGCACCCAGA

>TN744

ATTAACATACATAATTGATCCACAAAATCAGGAAAATAAATCATAGTACTT

>TN745

TCGGGGAGATACAAATCAAAACCACAACGAGCTGTCCCCTGGCCCCTGTTC

>TN746

GGATAAACCCAGAGGACGCTTTGCTAAGTAAAGTGAGCCAGGCAGAGAAAG

>TN747

GCTGCTGAATGCATTCACTTGAAATAAGAACATCTTTTTCTGGCCCTCCCT

>TN748

TTTGTTCTTCTTTTTCCAGTATCTTAAAGTGGAAGTTTAGCTTCTTCATTT

>TN749

GAACCCTGCTTTGGCTGCATCTCATAAGGTTTGATGTGTTGAGTCTTCATT

>TN750

GATGAATGCTTTGCTCTTCCTTGTGAAGGTGGTGGTGGGGTTGTGGGTCAG

>TN751

ACATACGCATGTGTCTTTGCAGTAGAATGATTTATAATTCTTTGGGAATAT

>TN752

AGGATTTCAATAGAAAAGCAGAAGAAAGGCCAGGGGTGGGGGCTGAGAAAC

>TN753

AGAGAATGTGGCATGTACACAATGGAATACTATTTAGCCTTAAAAAAGAAT

>TN754

AATAACACAGAGCTGCATATTTCAGAACAGCCAGAAGAGGGGATTTAAAAT

>TN755

GGGACCAGGCTTCCTCCCACATCCCAAAGCCCTGCACGTGCAGCGAGTTGC

>TN756

TGTGAAGGCAAGAGAATGTCCCAGGAAGTAGAACAAAAACACAGAGAAACA

>TN757

GAAAACAGAAATGAGATGTTAAAAGAAAATGGGCTCCGTAGGTCCGACAGC

>TN758

GGTCAGGGGAACCCAGGGGGGTTCCAAGCCATGGTCATCGGAACCGAGGAA

>TN759

GAGAGCAGGGAGGGGCGGTAGAAATAACTGCAGAGCCCCCCGGGCAGCACC

>TN760

CATTTTACTACTGAAGTATGAATGTAAATACAGCCCTTACTTTCACATTTT

>TN761

TGTTTTTAGTGCAAGATTGCGTATTAACTAACTGTAAGACCTGAAAGTAGG

>TN762

CAAAGTCAGCATCACACAATTTTTTAAAAGAAATTAATTTTCCTGATTCAG

>TN763

TCAACGCAGAGGACATTAGCCGGGCAATTTCCTCAAAATTATTTTTATAGT

>TN764

AATGTATGTTGGAAACATACTGGAGAATGTTGGAAGGATGGTTGTATAATG

>TN765

GGAAGGATGCTGGAGAACACATCAGAAGCCACCTTCCATTGAATCCACATT

>TN766

AAGTATAATAAAAAAAATTAAAGAAAATAAATTTAAAAAAAAGAAACTCAA

>TN767

TCTGCACTAAAGGGGAAAGGTTTTTAAAACTATGACTTACTTCATCTTTGA

>TN768

TGATGCACAAATAATGAAAGAAAATAAAATGTTTCTTGAGGGGAAATGAGA

>TN769

TGCTTAACCCTGCATTCAGAATGAAAAAGCTACAGTTCCTGAAGTTGACAT

>TN770

TGTCTCAGACGCTGGCTTTTAAAATAATTAAACAATCAAAGACTGCCAAAG

>TN771

GGAAAAAATGTGGTGTTCCAGTTCAAAGGCAGGCAGGGGCGTTGCATAATA

>TN772

ACTTGAACATGTTGATTCAATGACTAAAAATGACCGTGACTGTAGCACTTT

>TN773

CTGCCCTCGATGCAGGAAGAACCAGAAGATTCACAGAGCCTTCGAGTTAGA

>TN774

CCAGGCACACACAGCATGATGTAACAACACTGCATCAGAAGCCAGGGGGTG

>TN775

TGTCAGGGTTTGGGAAATCAGGGTAAAGAGTATGTAGGAATTCCCTGTACT

>TN776

GCTGCGGATCTCACACGAGCCTGGGAAGGCCACAAAGCTCAATGACAAGTT

>TN777

AGTTCTCAAGGGGACGGAGGTGAAGAAGAAAGACCAGGCCTGGACCCTGAG

>TN778

TCACCTTTAAAATTGGGAGAACAATAATGGCGACATTCTGAGCTGGTCAGG

>TN779

GAGAAAAGATCACCACCTTTCTGGGAAGAGTTATTGGTGGTTGTCCACCCA

>TN780

CATAGAGCTAAGCCAGCAAAGGGAAAAGGTGCACAGGTTGGAGTCTGGAGG

>TN781

GCCATCCCGCCACAAGCACAGCAAGAACCGCTGTGTACAAGATCATGTGGA

>TN782

TTTTTCCATTATCTGACTGGTTTATAAAAAGTGTGAGCAGTGTTTTAAATC

>TN783

TCCGGGTGCGCCCGCATCTGCCAGGAAGACTGTCTTTCAATTGAACACAGC

>TN784

CGTGGGACAACTTGGGCCGGGAGAGAAGATCTTTCACTTTGATTTGGGGCA

>TN785

CCTTTTAATTATTCAAAGAATACATAAAATAATGCCTCTTACCAAGCGCTT

>TN786

TGACAGTGTGATACATGATCTCAAAAAAATAAAAATAAAATAAAAATAATC

>TN787

TGCCTAGAAGAGAACGAGGATTTTTAAAAAGACAAGCAAATAGCATTCCAC

>TN788

CTTTAGTGCACATGTGTCACTGTTTAAATGGTATTTAAGTGGGAACAGGAT

>TN789

ATTTTCAAACTTTTTAAAAAATGACAATGGATGAATAAGTATTTTTGGTAA

>TN790

TCCTAAAGTTTGTTCTAGTTTGAAGAAAATGGGAATGAGCCCAGCTTAAGC

>TN791

GGGATGGGCTCACGCAATCCCCTCAAAGACCTGACTAGAATGAAGGACACC

>TN792

AAGAACCAACTGTTAAGTTTTTAGGAATCTTGTGTGCCAGTTAGAAAAAAA

>TN793

ATGAGTGATATAAGCAAAGAGATGGAAACTCTAAGAAAATCAAAGATAAAA

>TN794

TTAAAAATGGAACAGAATATTGAAGAACTGTAGGAGAAATACAAAAGAAGT

>TN795

AACATATGTGGAATGGTAATACCAGAAAGAGAAGAAAGAAAGAAAAGAAAA

>TN796

AAGTTTCCAAAATATATAGATACTAAACAACATACTTCTAAATAACACATT

>TN797

AAATCCAAAATAAGCAGAAGAAAAGAAATACTATGAATGAGAAAAGAAATC

>TN798

ATCAGTCCTGATTCCAGGAACATTAAAAAGATAATAAAAGAATACTATGAA

>TN799

CGTTGTCTGTGGAAAAAATCCAAAGAATCAACAAAAATTACCCTGAAACCA

>TN800

ACAAGATAAAATTAACACCAAAAATAAAGAAATACTTAGGTATAAATCCAA

>TN801

GTACCAAAAGCACTGTTCATGAAAGAAATAATTGATAAGTTGAACTTGCAT

>TN802

TGTATATCATCTGGTAAGGCATTTCAATGGTGGGATTTTCCCCAGAAGTGA

>TN803

GCTCAACATTGTTAAGATTCTTCAAAAGCAATCACACTGACTTCACAAAGG

>TN804

GGATTCCGGGAGTCAGGAGTGACTGAAGAGGAGACCTCTCAGGAGACAAAT

>TN805

ATTATATCTATAATGTTATGTGACCAACATTACCATCTATTTCCGGAAAAT

>TN806

CCCCTGCCCCAGACCCTGCACCTGTAACCTACTGTCTGTCTCTCGGAATTC

>TN807

CCCAGTCAATACGCCGACCTCACTTAAAGACAGGACGTGATTAGCTGAGAT

>TN808

GGTATTCATGTGCCAGGGTGGTTGCAAATTAAACTTTTCTCTAGACATTTT

>TN809

TAAGTATAATTTCTTACTTAGAGTTAAAAATTACAGCTAAAACAAAGTTTC

>TN810

AAGGACTGGAGGAGAAAAGGAGACTAATAATGTATTTACTGGGCACCCACT

>TN811

TTGGGGAGTTTTGCGAAGTAGATAGAAATTATTTTGACCTAAACATCCTTA

>TN812

AGATGCATTTACACACAGGGGCTGGAACTCAGCTCTGTAGGAGATGGCGTG

>TN813

TATAAAACAACTGTTTTGTGGGAGGAAGTGGGTGAGGTTGGAAGTGTGTTG

>TN814

AAGTGGCATGCACATATTTCTATTCAAGATCTAAGATGGCTAACACACCAG

>TN815

CATGGGAAATGAATTAAAATATTGAAATTAATTCATGAAAATTACAAAATA

>TN816

TGTGGTATTTTAAAATAAAGCATAAAATGTAACTATTTGATTTTAATAAAA

>TN817

ACAAACACACATACACACATGCATAAACACCCATGCACATGCATGCATACA

>TN818

ACACACAAATGTGCATGCACACATAAATACACACATACACATGCATGCATG

>TN819

ATTCAGTAAAATATTATTCTGCAGTAACTTGTGGAATGACTGCTGTACACA

>TN820

GGCAGCATCCCTGATAGTACTTCTGAAAGGGACAAGATAGAAAAGCAAACC

>TN821

CTTTTGGCTTTTATAGTATTAAAACAAAAATTTAATTTCCTAAAGCTTTTT

>TN822

TATTACGTAGAACAAATGCTAAGACAAAAACAGGCCATTTGAAAACTGGCT

>TN823

TTCCGATGGCTGTTCCTGAACAAGAAAAAGTTAAGACTTGCATGAAAACTT

>TN824

CCTCAGTCTACACAGTGAGCTCTGGAATGAGGGGTCTTAGTCTACACAGTG

>TN825

TCTCAGTCTACACAGTGAGCCCTAGAATGAGGAGTCTCAGTCTACACAGTG

>TN826

TCTCAGTCTGCACAGTGAGCCCTGGAATGAGGGGTCTCAGTCTACACAGTG

>TN827

TCTCAGTCTACACAGTGAGCCCTGGAATGGGGGGTCTCAGTCTGCACAGTG

>TN828

TCTCAGTCTACACAGTGAGCCCTGGAATGAGGAGTCTCAGTCTACACAGTG

>TN829

TGAAGCGTATTAGAAAGTGACCGTTAATTGCTAACCTGAGAGAACGTTTGT

>TN830

TTAAATCATAGCCTGAGCAACTTCCAAACAAGCAAACACGGTGCCGCCCTC

>TN831

TGGGATATTAGGGCGAGAAGAGTACAACCTGGGACAAATTTCCAAGGAATA

>TN832

TTGTCTGGGAGGGGCAGGTGGGGTCAAAGGAGAGATGGGTTACTGTTCGAG

>TN833

TACTCTTGATATCCTCAGATGCCAGAATGCTTCCTAGAGACCCAGGTTATT

>TN834

CCAGTTGTTTTCATTGTATTTAGAAAATAATATTTTTTAGAAAAGGTTGTT

>TN835

CTTATCTCTGTCTCAAGAAGGGAGAAAAATGGGATGAATGAAGAATAAAGG

>TN836

GAAAAACACCTGCAGCTGTGGGTAAAATGATGGTGTTGGTATCCCCTGGAG

>TN837

GAGCAACATTTTATGGGCTAGAAATAAATATTAAACGTTAGGAGTAATTCT

>TN838

AAAATATGACAGTAGGTGAGGGTCAAAATTAATATGTGGTTGGGAGTTATG

>TN839

TGTGCCTGTTTCTCCTGTTTCTGAGAAAAGTGTTCTCTGAGCCCCTTGCCG

>TN840

CCTGTTGAGAGGAACTGGTGGTCATAAAACCCAAAATACGGCAAGTTGTCA

>TN841

CCTGAACCACAGAGGTTGAACGCAGAAACCCTGGTCTAGACTTCCCTGCAA

>TN842

CCTAATAGTTTGGTTGTCAGCATTAAATGAGTGAGTGTATATAAAGCACTT

>TN843

TATGATCTCTCTTCAGCTCTTGATTAACAGAGATGCTTGTGGAATGAGGAA

>TN844

TTTTATGCAAACGGGGGAAATGTATAATAAAATGGCGAGTTTTATGCAAAT

>TN845

ATGTTATAAAAGGTAAGGCGTATACAAAAAGAAAATATTCAGTTATTCCAT

>TN846

GTGGATACAGACACAGGCACATATGAATGGAGGCACAGACATACATGAACA

>TN847

AGAGGTTAAAATGTGTTACCAAGAGAACAAAAGGGAGATAATCTTTAGTTC

>TN848

GGAAGAGTGATACGGACTCTGGGCAAATATTTGTGGAGAACGAATTGGGCC

>TN849

GCCTCACGTCTGCGGAAGAGGCCAGAAGCCAAGCTAGAGAGGCCTCAGTTT

>TN850

TCAGCGCCGGCACCTCACTGAGCAGAATGAGGGTCCTGTGTTTCGGGGGCG

>TN851

ATTCTGTGGCAGAGTCCAGGGGAGTAAGTTCTGATGTTTTTTGTACATTTA

>TN852

ACTTCATAATATCAACTGGTGTGCTAACAAGAAGGTGAGATGGTGTATGCT

>TN853

GGTGGCACATTGGTTACCGTTGAGGAACCAGTATTGATCCATGATTATTGA

>TN854

CTTCCAAGTTTTGTCAGCTAGAAATAAAGCTACTGTAAACACTTGTGTGCA

>TN855

CACTAAAATATTTTTGAAAATGGAGAAGCCATCGATATCTTGATAGTTTAA

>TN856

GATCTAGCTAAGGGTGGGGGCAGCCAACACGGGATACTAATGGAGAAAATC

>TN857

AATAATGGAAAGATCATACAGACAGAAAATTAATAAGAAAACATTGGACTT

>TN858

GTTCATATTGATTTTCAGGCCAAGGAATGCTGTTCTTATAAATGCTGTGTT

>TN859

GAAGAAGTGACTTGGGGGGCTGTGGAAACTCACACCCTGAGGACCTGTTTC

>TN860

ACCTTCACACCAACAGCCAGACAGAAACACCTTCACACCCGCAGCCAGAAA

>TN861

GCTAAATGAAGAAAATATTCAGTGTAAACATTGTTCAAAACGTTTGTTTGT

>TN862

TCCTCACTTGCTTTGAACATCACACAAATCACAGAACGACTTTGCTATTGC

>TN863

TCCACAGTGGACAGCGTGTGTAGGGAAAACCTTTGTCCAGCATGTCCACAG

>TN864

ACTCCTCAGGGGCTAGAAGGGCCTGAACGGCATCTTCACTCTGATGTTAGC

>TN865

AAAAGGAAAAAAGATTTATGAACTTAAAAAAGCAGCCAGCATATTGTATTA

>TN866

GTGCCTGAAAATGAAAAGTATTCTGAAAGTGAAGACTCTACCACAGTTGTG

>TN867

CTGAATGTTTCCCCAATCTATGGGGAAATGAAAAGAAGACAGTGCAGTCAA

>TN868

TGGGTTGAATGGCAGTTCTGCTTGTAACTCTTTGAGGAATCACCACACACT

>TN869

TACAATAGTTTTTTTCTAGTTCTGTAAAGAGTGTCATTGGTATTTTGATAG

>TN870

AATACCTAGTATATTGAGAATTTTTAACATGAAGAGGTATTGGATTTTATT

>TN871

ACACCATAGATGCTGTAGAAATTTAAATGATCTAAATACAGTGGTGGCATC

>TN872

GTGACAATATTCACCTCACATTGAGAATGGGGCAGATAATATATTTGAAAA

>TN873

ACAGGGCAAAACCCTGTCTCAAAAAAAAGAGAAAAAAAAAGAGAATATTCA

>TN874

CTGAACACAGTTGATGGGAAGACAGAAACCAAAGCAAAACCTCTTCCGCAG

>TN875

AAGTGCTCATTTTAGCTGCCAAGAAAAGCCTAATTTATTTTCAGGGCAAAA

>TN876

ACATAGCATTATGCTTACATAGAATAATTTATCGTTTGATGTTCAATATCA

>TN877

ATCCAGAGCTGAGGGGAACGTAGAGAAAGATCACATTTTTGATCCCACAAT

>TN878

TTATCTTTCCTTTATTCCTAAGGGAAAAAAAGAAATAGTTCTATGCACAAC

>TN879

CATCACAAATGGATGTTTTCTTACTAATGAGCTTTGATTTATTTGTGCATT

>TN880

CCTTGAAAGCTTCCTCCATATATAAAAATATATTTCCATGTAGCCAGAAAC

>TN881

CATGCTGTGTGGGTGTGTAGCCCAGAAGCAACAAGCTGCACCACACAGCCT

>TN882

TAACAAGAAATCAAAACAAACTCACAATTCCACTTCATGGTCCTCTGTCGC

>TN883

TTTTGTGTACAGACATGGACATTGGAATCACATTTTAAATCTTACTTAGGG

>TN884

GAGATCTTCTCATTTGCAACTGGTTAAAGAAGAGAAGCTTTGTTTAAAAAT

>TN885

CTGTAAAACCAGGAAGTTATCATAAAATTATATTGATCATTAATAGGAGTC

>TN886

ACACAGTAGTATTTAATTTGTAATGAACATCACTCAGTTGTTTTATACAAT

>TN887

CTAAAACATGATTTCAAATTGAATAAACTTGTATAAGTTCGTTTTCACACT

>TN888

AAGCCAAACCTCTCCATGGATCCCTAAGCTTACCGATGCAGAGGATGGCCA

>TN889

AACACGAGAAGACACTGAGAAGTTCAAGTGCGAATAAAGTGCCGAGTGACA

>TN890

TGCTTTTTTTCTATGTAAGCATCATAACAGTGTGGCCATCAGCCCAGTAAG

>TN891

CAACAAAATTGAATTTGGGCTAAAGAAACCCAATTGGACCTGATTTATTTT

>TN892

TATATTATTAAAAAAAGAAAAATGTAATCCAGAATGTTGGTGTTGTGGTCA

>TN893

GACTGAAAAAAATGACTCAAGGCTGAATACTAATGATATTTATACAAAAGC

>TN894

AGTCAGACCGCAGACGGCCTGTATGAACAGTCAAACCACAGACGGCCTGTA

>TN895

ATGGCCTGTATTCACAATGCAAAGGAAGGAAAAGCAAAAGCAAAAGTTAAT

>TN896

TTGGGCTTTGATTAGGAATTTATGGAATCCGTAAATTAAAATTTGGACATT

>TN897

TATTGTAAACTTTATGTGCGTCAAAAAAATGATGCTTTTGTAAAAATGAAA

>TN898

TAAGGGTCCAATACCATAAACAGCGAAGCGTCCTGATATGTAAAGGCTGAT

>TN899

CATTCACACTGAGAGAGGTTGGTAGAAAGGTTCTGATTTCCACGCGAAGAT

>TN900

AATGTACTGTAGGAAGATAATTCTAAAACTCTAATACAAATAATAATAGGC

>TN901

CCTATGCGTGGAGCTTCGTCATTTGAAAAGCCTTTTCAACACAAGGTCATC

>TN902

CCTTCAAGAGTATCAGGAGTTATACAAGAGACAGATTCCAGTTTTACAGAG

>TN903

GAGAATCCTGGGCACCAAAGTGGTTAAATCATGGCCCACCCTAGCTTATGG

>TN904

GTTTTTACTAAGATGTGTTCTAAACAACTCACGAAGCAGCACACTCTTCTG

>TN905

TGTATTTTTCTATCTAGATTTGTACAACAGAGAAAGAATACCTGCATCTAT

>TN906

ATACGATTTCAGTCATACATTGAAAAATACTAGTGAAGTATTCAAGGAACG

>TN907

CAGCATGGTGCAGACCACAAAGTGTAAAGCCACCCCGAGCCTGGCTCCTTC

>TN908

GAGAAAGAATATTGTTTTCAATAGGAAAATGCATCAGTTAAAGCAGTCTGT

>TN909

TTCTACTCGTGGAAGAGAAATATGCAACTCACTGCTAGCATCACTAGGAAC

>TN910

AGAGACCATAAATACAGAGAAAATTAAGCAGGTTTTAAAATGATCAAAATA

>TN911

TTTGGGGCAGAGAGCTGCGTGAGCCAAGAACGCGGTTGTGTAACTGACCCC

>TN912

CCTCGTCTCCTGTAAAAACACATGCAAGATGCAATCGTGGAAAATGATAAA

>TN913

CAGTTTACCTCTCAATAAATTATACAATTATTATTTGTCAATTAAAATAAA

>TN914

AGGCCCCCAAAGGTCTGACTCCATGAAATTTATCACAAACCCTCAACCACA

>TN915

GAGATGAACCTGGTACCTCAGATGGAAATGCAGAAATCACCGTCTTCTGTG

>TN916

AAAAAAAAAACCTAAGAAGTGTGTTAAATTTAGCCCAATAGTGCATTTCAT

>TN917

CGGTGGCCAGGCTCTGGAAACTGGGAAAAGTCAGTCCTTGAGATCATCGTT

>TN918

GACTGCCTGAAGGCAGGGAAATGTTAAATTCAGGAAGATAGTAGTTAAATG

>TN919

CTATAATAATAGACGTCCTTATTAAAACAGCTGAACATAAATATCAAGTAA

>TN920

GCTAAGGAGCTGATCTTCAGCTCACAACAGCGTCTCCTCCGTGTTCTGTTC

>TN921

AGTCACGGTGCTCTAAGCAGGGCAGAAACCACTGTGCGTTCCCAACAGGGG

>TN922

TTCACGGAAGGGGAGACTCCAGAATAATATCAGTTCACACAATAAATAAAA

>TN923

CAGTGTGTTTGCGTTGTGTTTGGGGAAGCAGCGTGTTTGCATTGTGTTTAA

>TN924

AGTTGCACTAAAGAGAACATATACTAAAATATGAAGCATTTGTGATTTATT

>TN925

ACTGGCTACATATTGACTGCAATTTAACAATGGGGGCAATAGTCACAACAC

>TN926

TTTACCTTGAAATTCAATTGTATAGAAATAAACCGTATTATATAAGATATG

>TN927

GCTTTGCATTGTGAATACCTTGCGGAATAGCTCGATCAAGCTGATTAACAG

>TN928

CTTTCCGGGGAACCCAAACCAAGACAAAGGCCCTGGATAGAAAATGCTGTA

>TN929

TGCAGACTGGCAGACCCCAAAAGGCAAGGGGAGAAAGGCCGAGCCGCTCAG

>TN930

CATCCCCGGGGGTCCCATCCTGTGGAAAAGTGTGGGGAGGCTGGGGGTGAC

>TN931

AAGAAGGAGATGTCAGACATACAAAAACATGTTTTAAGGAATGTATTCCAA

>TN932

TGTGAGATCCTTGACATCTGCCTGGAAATGAGGGAGGGATGGAGATGGATG

>TN933

GAGGTTCTTAGGGGCAGTAGAGTGCAAAGAGAGAGAAGCTTAGATTTATGA

>TN934

CTATTTGAGAAAAGGAAGGGTCCAGAAGTGCAGGCTCTTCCTGAAAAGGCT

>TN935

CACGCAAGCCATGTACACTGTACCCAACGTGTAGTCTTTTATCCTCCACCC

>TN936

CATCATCATTCTTCACAGAAGTAGAAAAAACAATCCTAAAATTCATATGGA

>TN937

TGTGAGGGCTTTTGCAGGGGTAATGAACACCACCAGCCAACGATCTTGTTT

>TN938

AAAAAATAAAATAAAATAAAAAAATAAAAATAATAATTTGCTTCCATCGCT

>TN939

AGAAAAAGAAATGAAATCACAAGAAAAGGGCAAAATCCCCACATTCCCAAG

>TN940

GAATGAGGAGGCAGACGTGGTCATCAAGGAAGGCTGCCTGGAGGAAGTGAC

>TN941

GTAGGACACCACAGCTCCAGGACAAAAGCTCACTACCTGCGGCCCCGCCAC

>TN942

CATGAAAGGATGTTGAATTCTATCGAAAGCCTTTTCTGCATCTATTAAGAT

>TN943

TTGTTTCAGCCATTTCAATCTGGTGAAGAAACATTGCCGGGGAGCTAGAGC

>TN944

CTATATGTTCTGTTCTCTAGCAAACAATGTGTGAGATGGAGAAAAACAGAA

>TN945

ATATGAAGGCAGATTCAAGTTAAGCAATGAATCAAAACGATATTCTTTGAG

>TN946

TTAAAATTCAACAACACAAAAGCCTAATTTTAAAATGAGCAAGGGGACTGG

>TN947

TGTTTTTATTTTTTTTTTCTGAAATAAGAATAGCAACTCTTCCACTTGTTT

>TN948

TGGAGGCCGTTATGCTAAATGAAATAAGGCAGACACAGGAAGAAAAATATT

>TN949

ATGGTCTCACTGGTGAATTCTGCCAAACATTTAAAGGATAAATTAATAACA

>TN950

TATAGAAAATATTTAGAAATTTCCTAACAAATTATTAGAAATAATAACTGA

>TN951

CATGTATACTGCCAATGTACAATACAAAAATGACCAAGATGTTATAAAACT

>TN952

TAAATGCAGTTCACCACATAAACAGAATTACAAACAAAAACCATACGTTCA

>TN953

AAAGACATCCAAATAATAAAACAGGAAGTCAAACTATTTGTCTTCACAGAT

>TN954

GAACTGATAAAGAAAATTTCAGTAAAATTTCAGCATACAAAATCAATGTAC

>TN955

CAAAAACAGACACATAAACCAACAGAACAGAATATACAACCCAGAAATGAA

>TN956

ACATGTAGTCAAAAAACCCACGAAAAAATGCTCCATATCTCTAATTGTTAT

>TN957

AGAGAGGAAATCAGCCTAGGCATGCAACACAGGTGGACTGGATAAAGAAAA

>TN958

GTTCTTTGCAGCAATGTGGATGCAGAAGGTCATTATACTAAAAGAACTAAC

>TN959

AACCTGCACATGTACCCCTGAATGTAAAATCAAAGTTGAAAAAATTAAAAA

>TN960

ATGTAAATCTTCAAAATAAAATAATAAATGCCAGTAGCCAAAAGAAAAATT

>TN961

TGTAAAAAAGGTTTCTATTTAGAAGAACTCCTAAAGCACATAAAAAGACAA

>TN962

GTGATGGTCCATGTCTGTGGCCGGGAAGACCTTTGAGATCAGTGTGTGAGT

>TN963

CTGATAGTCCACGTCGGTGGCTCGGAAGACCTTTGAGATCAGTGTAGGAGT

>TN964

ATATTTTGCAAATTGTTAAAAACGAAACTAGAATAATTCCTTATACGACTG

>TN965

GGTAGATGTTCAGTCATGTTCGCTGAAGGAAGAAGGGAGTGAATGAATGTC

>TN966

GGTGAGGGTTCAGGTTCACGTACACAAAACCATCAGACAGATTTCTCCATC

>TN967

AAGGCAGGTCAGCTTCCAGATCTTTAAAACGAGACAGACCGGGAGGGCTGG

>TN968

CTTTGCAGAGACCTCTCCTGATAATAATAATAAATTTACTCAGAACAATCT

>TN969

CGTGTGCGATATAATCTGTCAATTTAATATCGGAAAATAATTTAAGAGTAC

>TN970

GATTGCTTCTTGAAATTGCATATTGAAGAGCTTAGAAATAACCCCAGCCCT

>TN971

CCAGAATCAGTTATGATTCTGTCAGAACCTAAGGGCTCTGGCAGAATCAAA

>TN972

CATGCCTTCTAAAGTGAAACCACGGAATAGGAGACAAAGAATCAATAAAGC

>TN973

TCATCTCAAAATAAATAAATAAATAAAATAAAATAAAATAAAATAAAATAA

>TN974

ATTCACAAATAAACCCTAGTTTGCAAATTTCTTTCTTTTTAAGAAATAATA

>TN975

AGTAATTCATAATAAACTCATTTGCAATATTGAAATAATTGGCCAAAGAGA

>TN976

GCTGAGAGTCAAGTATCCTTAAGAGAATTCTTATCATCTTCCTTAGCTCCA

>TN977

ATCAGTAGTTGCAATAAAGAGTTGTAAATACAGCAATGGTTGTACTCTAAT

>TN978

CAGTGGTGCAACCTCAGCTCACTGCAACCTCCACCGGGTTCAAGTCATTCT

>TN979

GCCGTAATTGTGGTCAAGCTGAACTAAAGCTAAGAATTACATACCAAACAT

>TN980

AGCTCGAGCAGGGAATTTGGAATGGAAAAAAAAAAAAACACAATAGATTTG

>TN981

ATATATATAATATATAAATATATATAATATATAAATAAATATATAATATAT

>TN982

ATATAAATAAATATATAATATATATAAATATAATATATAAATAAATGTAAT

>TN983

TTGAGGAGTAAGGGGAATTTTGTTCAATGAAGAATGGGAAGACCCGCTGCC

>TN984

TGTCACCTGTAATGGGGAGATTCTTAAATGGGACACATAGGCCATCCTAGG

>TN985

CGAGGAGAAAACCAATTCTCAGTAGAATGAGAACGTTAAATACAATGAGGG

>TN986

AGTGTTCATTCCTCCATAGGCTTCAAATGAATTCAGTTGCTCGGCTTGCTT

>TN987

ATGTCATTTTCAGTGCAGCCTTGGGAATATCTGTCCCTGGGGGCTGGCCTG

>TN988

GCTGTCAACATAAACAATACTGTGTAAGGTGGTGTTTTAAAGAAATGGCAT

>TN989

AACCACCTCCCCCATCCAGCCACAGAACTTTTTCTAGTAGACCAGCCCTAC

>TN990

CCAGTTAAAAGTTACAGATTCACAGAATGGATGGAAAATCACAAACCAAAC

>TN991

AGCAAAAACCCTGGACACCCTCCAGAATCGCTGGCTTTGTGGGGAAGCATG

>TN992

TTCCCCATTCTTTATAATACAAACAAACGATAATTAACATTTCGGAGCTGG

>TN993

TTTTATTATCTGTTACCAAATGTTTAAAATATAAAGCCATCCCATTGAAAT

>TN994

CCGAGCGCGTTAATGAAATCCCCGGAAGTGTGTCTGCCAACAATCCTTTTA

>TN995

TTTAAACTAAAGACTTCTATTTGCAAAGATCGTCTTGAGAGGCAATATAAC

>TN996

ATGGTACAAAGAAGCAGGCAATATTAAGTATGATTTTGACCAAATGCAAGA

>TN997

CCATAAAATTGTGATGATGTTTAATAATACTCTTTGTGATAATCTTAGGAA

>TN998

TAGCTGGGAAGGGAAAGGGAAGCAGAAGCTCTATAAGTTCCTGAGCCTTGC

>TN999

ACAAAAAAAAAAAAAGAAAAAAAGGAAGGAGAAGAGGAGTGTTTTCCTCCA

>TN1000

TCAGCAAAAGCAGGAGATGTAAGGAAAACGGCTTCAGTGATGATTCATGTT

>TN1001

TAGACAGAGAAATTAGGCAAAAAAAAAAAAAAAAAAAAAAAAAAAAAGAGA

>TN1002

CCCACACACACCTACACAAGGGTATAAAAAAGGATTCAGCAAAGTGTCTGC

>TN1003

ACCCAAAATTCTTAGAAATAAGCTTAACCAAGGAGGAGAGAGGCTGTACAC

>TN1004

ATTAAGGGACACTTTCAGCTGAATGAAAGGGATGGAATGGGAGTAGATATC

>TN1005

ACATCATTAATTATTAAGGGAATACAAAGCTAAACCACAAGGAAATGCCAA

>TN1006

GTCCAGTTTTCAACAAAATGACTAAAAGACATATAAAAAACAGGAAAGTAT

>TN1007

AAAAGAGAACGAAATGAAAAGAAAGAATATTTGGAGAAATAAAAGCCAAAA

>TN1008

CTATGTTACCGTGCACTCAATGTGTAAAGGTGAAACCTGTGACAGGCCGGG

>TN1009

AAAAGAGATCATTAAATTAAATTCAAAAAATAGAGTATCAACAAAACTGAA

>TN1010

AAAGAAAAAAAAAAGAGAACACATAAACAACTAAAATTGGAAAGGAAAATG

>TN1011

AGTTGACACACGAAAGGTGTCTGACAAAATCTCATAACTGTTCATGATAAA

>TN1012

ATATAAAACACAGGGTCTAGAATTTAACATCAGAGGGAACCCAGGTGACTT

>TN1013

CATAATTACACACTTAAAATGAGTGAAATGGCAAATTTTATGTCATCTGTA

>TN1014

TTGAGAAGAGTAAAGCTTCCTGGAGAAAGTCAAGAAATAACTTGCGATAAT

>TN1015

GCGTGTGCAAGTACAGCGTCTCTCCAACAGTGGTCTACACTCGAGAAACTC

>TN1016

TCCCCAACAGTGGTCTACACTCGAGAAACTCAGCAGCGTTTAAAAATAGAG

>TN1017

ACTCGAGAAACTCGGCAGCTTTTAAAAATAGAGCGTGTGTGAGTGCAGCGT

>TN1018

GCGTGTGCGAGTGCAGCGTCTCTCCAACAGTGGTCTACACTCGAGACACTC

>TN1019

CTGTGGCTATAAGTTAAATACTGCCAAGCAACATACTAAGTTACCACTTTC

>TN1020

TAGAATTCTAAAGGGGAAAATGTGTAATTAGAATTCTAAGGCAAAGAATGT

>TN1021

TAGTTAAAAGCCAAGCATACTGAAAAAACTTCGAAATCTCAAACTTAAAAT

>TN1022

GTTCATTCTTTACCACAATAAATACAACAAAGGCAAATCAGGACATGTATT

>TN1023

TTTTAAAAACTCGTAGTAAAGACAGAACAACAACCACAGCAAAATGAACTA

>TN1024

AATGGCCGTCACAGTGCACTGGGGGAAGTGGATTCTTAGTCGACAGGTAGA

>TN1025

CTCACCTCTCACGCCCCAGACCTGGAAAAGCCATTGAGGCTGCAGGTAGCT

>TN1026

GGCTAAAAGGAAACTAAGGGAAGCAAAGTGTGGCAGAGCCGGGGCTGCGCG

>TN1027

AGAGCTCCAGGGGGGAAAACATCACAATATAAAGAAACTCTGAGCGGTGCT

>TN1028

AGAAAGGAAGGAAAGAGCTGGAGGAAAAGTGGCGAGGAGGAGGAGGAAAGG

>TN1029

CACCGTGCATCCACAGGCTGTCAAGAACCGGCCGGTAGAAGTCTGGGCTCT

>TN1030

CAGTGGGCTCCTCTCACCCTCTTTTAATAAACTACTTAAGAGCTGTTGTTC

>TN1031

TCAAAAGACACAATCTAGAAAGTGAAAATACAACCGATAAAATGGTAGAAA

>TN1032

TACTTGAATAAACAGTTCTCCAAAGAAGGCCTGCAAATGTTCCACTAAGTA

>TN1033

GTCCACAGCAGCATTGCTTAGCCAGAAAATGGAAACAGCAGATGTTCCCCA

>TN1034

TTTTAAAAATCTAAAACAAACCAAAAACTAAAGACACGATCTGTACAGTAT

>TN1035

ACACATTTGTAAGAAAAAAACCATTAAAAAGCGGACAAACAACAGGAACAG

>TN1036

CCAAATACTGCATGTTCTCACCTGTAAGTAGGAGCTAAATGATGAGAACTC

>TN1037

CACCTAAACAGACACAGCTTTACAAAACACAGCTGTGTCGTACGCTCACAT

>TN1038

TCTATTTTTTTTCAGCAACCAAAACAACACATATAACCTTACAACTAATAT

>TN1039

CTGTGCGGGTCCTGACTGTGTGTGGAAAGGCCGTGTGGGTCCTGACTGTGT

>TN1040

CTATAGGTCAAAGAACCTTGTAAACAAAATTAGAAGGCAAAAACACGACAG

>TN1041

TCAGAAAAAAAGAAAGAACGAAAAGAAAATACCTTGGAATGAAAGACTGTG

>TN1042

CGCAGGAATGGCAAAGTACGAGGGGAAAACAGACAGTGGGATATGGTGAAA

>TN1043

CGGGAATTCTCGGTGCAGAGGCCCCAAAGGGAGAGAGTCTAGCTGGTCAGC

>TN1044

TTTTCTGTCCATAAATTTGTTAAAGAAAATCTCTTGGAATTTTGCTAAGGC

>TN1045

ACACGGTTGCCCAGAGGCACGAGGAAACAGTCGCCCTGAGGCAGGGCGTGG

>TN1046

CAGAAGCACAGTGCCGAGTGTCTAAAAGGGAAGTGGGTGATTACAGAAAAA

>TN1047

CATTCTTAACTCACTGCATCGTTTAAACGGAGCATGGGAGCCCCCCTGGGT

>TN1048

AACCCCGCAAGGACACGTTTTTATAAAGTAGATGCTCTACGAAGCGGGAGG

>TN1049

CAAGGCATCTCTGCAGCATGTAGGTAAACTTTTACTTAGCCTGGAACCATC

>TN1050

AGCTGTCACACCCAATGAGGATTAGAAGCCATCTGAAGGCACCTGTTCCTG

>TN1051

ATTTGAAGATCTTTTATGTTTTCTTAAATTTGATGGTAATGAAACTGGATG

>TN1052

CTGGGACGCTGGTCACCGTGGTGCCAACGCTGGGACGCTGGTCACCGTGCT

>TN1053

CACATCAAATTCATTTCAATGCCAGAATTCCTTCAGCTGTGTTTCATTCTT

>TN1054

GGCCACAGCGGCGCTTTCTCTACCCAAACCTCGCTTCCTCTCGCTGAAGGG

>TN1055

GCAGGGCACCGAGGCACCTAACGTCAAGGCTTCAGTCCGAATCGGTCCAAG

>TN1056

GATGCACAGGTCTTCATATCCCAGGAAAGGTGAAGCTGGGAGGCAGAGGAC

>TN1057

TTCCTTCATTTCCCCAGATTCTTTCAAATCCTGATTCTCTTCTCTGAGGAG

>TN1058

TAGTGATAAAATATGTGACCCCAACAAGAAAGGGGTTTTCGTTATTCACTG

>TN1059

CTCCCAGAAAACAGGAAAATGACAAAATAATGGAAAATAAGAGTAAAAAGA

>TN1060

ATTCTAGAAAAATCACCAATTCTAGAAAAATCACCAGACCCAAAGTCGGTG

>TN1061

GTAAAGTAATAACTCCAGGGAAGGTAAAATGTTGTATGAGGATAGATAATA

>TN1062

GGAAGGGGTGACTCAGTGCCAAGCAAAGTGAGGGATGATGAAAAATGATAA

>TN1063

GAGGATACCAGTAATAGTGACACGGAAGGTAACATTCACGGGCATGGCTTC

>TN1064

TAGAAGGATGGGAAGCAGAATGAATAAATGGATAGATATTTTCAAGAACAG

>TN1065

TGAGAAGAGGCACGTGCAGATGCACAATGTCAATGGCATACTTATTAAATG

>TN1066

AAAATGCCCTCCACTCCAACCCAGTAAAGCCACTGCTGTTTCCCAAATCTA

>TN1067

TAATATACAGGCCAACACAATCTGAAAATGCATAGAATCACTTTCATGCTA

>TN1068

TACTCAAGAGGCTCTGAGGCAGGAGAATCACTTGAACCCGGGAGGTGGAGG

>TN1069

AGACTCCATCTCAAAAAAAAGAAAAAAGAAAAAAGAGAAAAAGTGGCGGGT

>TN1070

AAATAATGAATGAACCACAACAGCTAAAGCCTAGGTGGCAAGTTAATATAA

>TN1071

TTTAACTCGTTTCAATGATAGTGAAAATATCTGTCTGTATTTTAAAGAATA

>TN1072

TAAAAGACTTAGCCATGCCCATGGTAAATGGGGGGTTCATAAAAGCATGTG

>TN1073

GAGTGCATCCCTGCAGGGGCCCCAGAAGGCGTTCCTCTGGATCCCTTGGAG

>TN1074

CAGAACATGCTACACCTGCAAGGTGAATGCGTGGGTGTGGGGTCCTCAGCA

>TN1075

TGAGAATGAGACCAAACTCTGAACCAAGGGAGAGCATCCCCTAAGGCCGGG

>TN1076

TGTCCACCCAATACCCACATCAGCAAATTCAAAATGATGAGAACAGAGGCA

>TN1077

AACCTGGACAGATGATGTGAAACTCAAGACCATGCATGACTAAGAGAAACT

>TN1078

GTCTAAGTAAATTAGTCGTAAAAAAAAATTAGTGTCAGATATACAAAAACT

>TN1079

ATTTTAATAAACTTTAGTTAAGGCAAAATACAAAATAAAAGATAAGAAACA

>TN1080

TAAACAAACATCCTCATATACTTGAAAATAATCAAATGTCAACAGAAAGCA

>TN1081

GGCCTCCCCAGCTACATGGAACCGTAAGTCCAATAAGTCTCTTTCTTTTGT

>TN1082

TACAGTACTCTTCTCCATAATTCATAACTTAACAGTGCCGCAATGAATATT

>TN1083

TTATTGGAAAATGACAAGGAAAATAAAAATAAATGACTTACATAGCAAAAT

>TN1084

TAAATATAAAATAAAAAATAATCAAAATAGAAAACAAAATGCTAGTTAGTT

>TN1085

GCCTATTAGAAATATAATTTTTTGAAAAAATATAACACAATGAGCAATTTT

>TN1086

CATGTAAGGTTTATCTCAGGAATGCAAGGATGGCTCAGTGCCAGCAAATGT

>TN1087

GTCAAGTGGAAGCTTTGCAGGCAGGAAGAGGCCATGTGGCTCATATCAGTG

>TN1088

TTTTGTGAAACATTCTTACAATAACAACTTACAAGAACTAATCCTAAAGAC

>TN1089

GAAAGAAGAAGAGAAAGGAGAAAGGAAGAGGCAGCCAGCTGCCTGGCGCTC

>TN1090

GGGACTTGGGTTCCATGAACTCTTGAAGGAGCTTACCTTGAAAATAAGTCA

>TN1091

CTTGATCTTGTGGATCCAGATGTACAACATTCAGTACTCTGTGTTATTCTT

>TN1092

GTAAATTTAAAAATTAAAGATTAAAAATAGTAAAAGGGTTATAGAATAAGA

>TN1093

AGCAAAACTCCATCTCAAAAAAATAAAATGAATAAAATAAAAACCAAAGTA

>TN1094

TTAGTGAAACTAGCAGTGAGGAAAGAACCGAGAACTATTTTATACATGGAG

>TN1095

TCTGGTTGGAAGTGGAATGGGCTGTAATACCAATATCGTAATATTCACCTT

>TN1096

AAAAGTTTTATGTTATGAAAGTCATAATCATTACAGAAAAACTTAGAAAAC

>TN1097

ATTTGCTGAGGAGAGCTTTACTTCCAACTATGTGGTCAATTTTGGAATAGG

>TN1098

CTGTCATCCCGGCCTAATGATTATTAATTCCTGTTCTCTTTCCTCTCATCT

>TN1099

TTAGTCCAGCCCAGGCAGCTGCAGGAATTCTCTCAGAGTTGTCTTGGGGCT

>TN1100

ATCGACCAATGTGAGTGTTCATTGCAAGGAAAGCCAAGATTCATCTCCATT

>TN1101

AGGGGTCAAACCGACAGTAAAATAAAATATTAGACTCTTTACTAATCCACA

>TN1102

TAGAAGTGATTTTAAATTTTTGTAAAAAGTGGCTGAAAGGCTGAACACTTT

>TN1103

TCGCTGCCTTACTGAGCTTCCTCAGAAGAACCTCCCACGTATTCGTATTTT

>TN1104

AATTGCTTCCACTTTGTTTCTTTTGAAATCACTAAACAAGGACAAGGGTAG

>TN1105

TGAAGATCAGTCATTAGCCAGGTACAAAGGAACAAGAAAGTAGCTTGGAAT

>TN1106

TCACCAGCCCCTGTAAGACAGGTGCAATTAGTATCTCTCTTGCAGAGAGAG

>TN1107

GCCATTGCACTCCAGCCTGGGAAACAAGAACGAAACTCCATATAAAAAAAA

>TN1108

AAAAAAAAATTTTAAAAACCATCAAAAATGAAAGAAGGTTGAAGGAGGCAT

>TN1109

CCCAGAACAGCCATCCAGACACTCGAAGGAGGTTCACGCTGATTCAGAGTC

>TN1110

AAAAATTATAATAATTTTAAAAAGGAAGATCAGTCATCAGCATGGTCTTCA

>TN1111

CTGTAGGACAACTGAATGCAGGTTTAAAACAGTGCTACTGAAGTGAAGGAT

>TN1112

AGTGGTGCCATTATCTGTGTGAGGGAACAGCAGTCACTCGGGGCTGACCCA

>TN1113

ACAGGTTGGTGGGAGAAAATTTGGAAAGCACAAAAAATTTAAAGAGAAAAT

>TN1114

TAAATGGGCATCACAACTTACCCTGAAGTCATTTGCACAATACCCAGAACA

>TN1115

ATAATAAAATTTAAAAAAATAAAATAAAATAAATAAAAATAAACTGGAAAC

>TN1116

CAAAAAAAAAAAAAAACCCAAAAATAAAATCACTTTTCATGAACAGAGCAA

>TN1117

TTTGACATGGAGCTATGCTTACAAGAACAAGGCCCAATGCTTCTGTTAAAG

>TN1118

GGGAGGCCTTTTAGCCCATTCGTGCAAATATTAGGTCTTGACCCTGGTTCT

>TN1119

TGTTGGCTGTGGTGAACAGCATCTTAACGAACACGAGAGAGCAGATACCCC

>TN1120

AATTCTACTGGGTCAAATTAAAATTAAAAACACTTCATAATGATCTTGATG

>TN1121

ACAGCACAGTATAGGTTAAAGTCATAAAGCAATGAAGTGACATTTGCTTCT

>TN1122

TTTTGAAATACCTGAATATTAAAATAATACTTAGAGCCTTCCACCTGGCAG

>TN1123

AGTTGGAAGGAAAAATGGAATCCACAACTGCTGCGCACAGGAGGGAAATGA

>TN1124

AAAACCGTATAGACTAGAAAATACCAAAGTACACTCTGGATAATAAGAGCA

>TN1125

GTTTGGAGGTCAGCCTGTGGCTGTGAAGTGTGGCTTCCAGGCTCATAGGAA

>TN1126

CAGCTCCAGGGAGGCTCGGTGGGATAAGCGTCTGCCTTTCCCAGTGCAAGG

>TN1127

GGATGGATGCATCCAACCAAAGAGAAAACAAGGAAGGAGGAGACATGGAAG

>TN1128

CAGAAGGGAAGAATATTTCAGCTGCAATTGGGAAGAAACAGAAGGGGCTTC

>TN1129

GAACCCTTGTCTACTTGGAAATCTTAAAAAAAAAATCTAAACATGTGCTTT

>TN1130

CATCTCTGGTTTGCACACTTGTTTTAATTTGGAATACTTGGGGGCAGGTGA

>TN1131

CGGAACGGAAATATTCAGAATGTAAAACACTCCTGCCTGTGTCTATGCAGC

>TN1132

TAAGGAGCCTGGCCTTCCTGTTTGGAAACAGGAGGATGAGCCAAGGAGGAG

>TN1133

TGGAGTGTTCATCATATGCTGAGAAAAATTCATGTTTATAGAAAGCACCAA

>TN1134

CATCAAGTACTTTACAAGGTAATAGAATACCACAAGGCAAGTGGAGGCAGG

>TN1135

GAATACAAGTCCAGCCTAACTTTTTAAAGTCCTCATGAGCGTGGCTGCCTT

>TN1136

GCAGCAGAGCAAGACTTTATCTAAAAAAATAAAATACTATGAATACATATT

>TN1137

TCAAAATTACATTTGCTTATGAAATAATTATTAAAAGTCAAATGTTTTAAA

>TN1138

GCAGGTTATAAATTTGTGTCAAAGCAAAAATATAATCACCCAAAACCGTAT

>TN1139

GATGGATGGATGGATGAAAGGATGGAAGGAAGGAAGGAAGGAAGGAAGGAA

>TN1140

CATTTTAAAACATGTTCTCTCCACAAACACAGAATTCCCTGAGCACCTAAG

>TN1141

CAAGTCCATCTGGGTGAGGACTCCGAAGAGGAAAACAGGTTACCACGTGGT

>TN1142

TAATTCTATGACCTCATGAGAAAAGAAAAAAAAACACCCACCTAGTGACCA

>TN1143

TGGGGATATAAGTCACACACCATAAAAGTCACGCTTTAGGATGTACAATTC

>TN1144

ATGAAGGAAGATTAAAATTGTATTAAATTACACGTTAATGGAAAAACAAAG

>TN1145

TATGTGATACATGAGCTTACTAGACAATATCACCCATCAAGTACCAGATGC

>TN1146

TGTCTGTGGGTCCTAGTGTACGGATAAGATTTAAAAGAATAAGAAGAGAGA

>TN1147

AACACAGAGGGGAGGGGGTGCGATGAAAACGGGGAGCCCTGAGCATTCCCT

>TN1148

GAGGGCTGCAGGGTTCGAATGCTGTAACATAACGGAGAGAGATTTGTTTGC

>TN1149

TGGAAACATGTTTTTAATTCACAAAAAAAATCCCATAAACCTGCCAAAGTG

>TN1150

CAGCGTGAGCTCCCCCGTGCCCAGGAACTGGGTGGCCGGGCCTCCTTCTGC

>TN1151

CATATAGGGTTCATGTAGACAATTTAAAAATATAAAGAAAAGTCATTGAAA

>TN1152

CCTCTTGGAATCTGGGTGGAGGGAGAATCTGGAACTTCCCATCCTGTCCAA

>TN1153

TCCCCTCAGAGAGACACGTAACGCAAACACACAGCAGGCAGCCTGTGGGGC

>TN1154

CTTATACCAGAGGGAAATAAAACTAAATTTTAGCCAGTGCTTTGTCCCAGC

>TN1155

TTTTGTTATTTATCAATTACATAATAATTAAGGAAGTGTGCTTGGGCAACT

>TN1156

AGATAAAACTCTTTGGGAAATCAGCAATTCCTATGAACCATGAAAATATGA

>TN1157

GGGGAGCAGGTGTTTGTAAGCCAGGAAATAACCAGGAAAGATGATTACCAG

>TN1158

TCTATATGTTTGAAAGCAAAACATGAAAAAACCAAAGCCTTTTTTAGACCA

>TN1159

ATTTCTTGTTTTTGTCAGGTTTGTCAAAGATAAGATGGTTGTAGATGTGTG

>TN1160

CAATGCCTTTCTTCACAGAATTGGAAAAAACTACTTTAAAGTTCTTATGGA

>TN1161

TTCAAAATGTTCCACCTCTTGGAACAATGCCAGAATTATTCTGTTAAAAAC

>TN1162

AAGACTATTCATCTACCTTATCAATAACTAAAACGTTTGGAGAGCATGCCA

>TN1163

ACATCACAGACTGAAGAACCTGAGAAAATCTCAAAGACAGATCAACACCAG

>TN1164

ACTACCAGAAGTGAAGGATACAGACAAAACCCCACACATCTATACACCACA

>TN1165

TACCAGAAGAGAAGGATACAGACAAAACTCCACACATATACACACACCACA

>TN1166

CACCACAGTCAAACTACCAGAAGTGAAGGATACAGACAAAACTCCACACAT

>TN1167

ATATACACCACAGTCAAACTACCAGAAGTGAAGGATACAGACAAAACTCCA

>TN1168

CACACATATACACACACCACAGTCAAACTACCAGAAGTGAAGGATACAGAC

>TN1169

TCCACACATATACACACCGCAGTCAAACTACCAGAAGTGAAGGATACAGAT

>TN1170

AGGTGATCAAGTGAGTAACCAAGAGAACACCAGCTATTGATGGACGCATCT

>TN1171

TAAGCTCCTTTAGGTTCTATTAGTCAAAACGGGAGCAGACGCCAGACTCCT

>TN1172

CTCATTGTAAAGAGCTAACACACGTAATTGTGTGAAGTGGAGTGTCACTCT

>TN1173

GAGCCAACACGGGTTTCTCTGGGCTAAGGCCGAGGTGTCGGCAGCCTGCCC

>TN1174

TGAGAATCTCCTAATTTTAGGCAGCAAAGCCTTATCCCAAATACTGGTCTC

>TN1175

GCACTAAAATAAGAAAAGATAACTGAAAAACATTAGTTTCGTTGTCTTCTA

>TN1176

CACCCGCCCAAGGCTGTTTTTAAAGAAGATGTACAGTAAGGGAATACGTAG

>TN1177

GCCCTCGCTTGGCCTCTGCGTCAATAATTCTGACATGAACTACAAACTCCC

>TN1178

CCCATTGCATTACTGGACAACTTCTAACTATTGAAAATTTTCCATTGGGAG

>TN1179

AATAATAAAATAAAAATAAATAAATAAATAAATGACACTAATTGAAAATCT

>TN1180

AACAGAAGAAAGCATCGGTGAACTCAAAGACAACATCGTTACAAATAATTC

>TN1181

AATCAGGACAGCAAAAAATAGCAATAAAAAGAGTGAAGAAAGCTTACAGAA

>TN1182

GCTTAAGGAGATTTAGGGCTGAGACAATGGGGTTTTCTACATATACAATCA

>TN1183

ATGCACTGGAGTTTGCTGAAAATGTAAATACGAATTTTATCTCCTAACACC

>TN1184

TTACCTTCCCTGTTATAAGGAAGAAAACAGAGAATGCAGCGTTTGCTAACT

>TN1185

GCACCAAATCAGAGGCGAAGAATGAAAGTATTTGTGGAAAATGAATTATCG

>TN1186

AAGGCAAAGAAGTGGAAAACTTTGAAAAAAATTTAGAAGAATGTATAACTA

>TN1187

GAATAACCAATACAGAGAAGTGCTTAAAGGAGCTGATGGAGCTGAAAACCA

>TN1188

GAGAAGGGAAGGTTAGAGAAAAAAGAATAAAAAGAAATGAGCAAAGCCTCC

>TN1189

GACACATAATTGTCAGATTCACCAAAATGGAAATGAAGGAAAAAATGTTAA

>TN1190

TCCTCAGCAAATGTGAAAGAACAGAAATTATAACAAACTATCTCTCAGACC

>TN1191

CAAAAGCTAGCAGAAGGCAAGAAATAACTAAAATCAGAGCAGAACTGAAGG

>TN1192

AAATAGAGACACAAAAAACCCTTCAAAAAATCAATGAATCCAGGAGCTGGT

>TN1193

ACTTCAGCAAAGTCTCAGGATACAAAATCAATGTACAAAAATCACAAGCAT

>TN1194

CATTCACAATTGCTTCAAAGAGAATAAAATACCTAGGAATCCAACTTACAA

>TN1195

TATACTACAAGGCTACAGTAACCAAAACAGCATGGTACTGGTACCAAAACA

>TN1196

CAAAAGAAGACATTTATGCAGCCAAAAAACACATGAAAAAATGCTCATCAT

>TN1197

TATTCTCACTCATAGGTGGGAACTGAACAATTAGAACACGTGGACACAGGA

>TN1198

GTTAGAATTCACCGGAAAGATCTTTAAACAGCCATTGTAGTCTTATGAATG

>TN1199

ATATAAAAATGTTAGAACTCACCGGAAAGATCTTTAAACAGCCATTGTAAG

>TN1200

TATAAAAATGTTAGAACTCACCACAAAGATCTTTAAAGCAGCCGTTGTAAG

>TN1201

TGAAGGGACACACATATAATTAGAGAACCAGGGAGAAAACTGAGAGAATTT

>TN1202

CCAGACTGAGGTATGGATTCAGTGCAACCTCAGTAGAATCAATGTCATTCA

>TN1203

ATGTCAAGAACTCCTACAAAACAAGAACGTAAAGACAACCCCAGAAAAATG

>TN1204

TAAGAATTAGTCATCCAGGAGGTGAAAGTGAAAACTTTATTGGGATACACT

>TN1205

TAACATTCTTACCTTATATTCACAGAACTCCATAAGTTATGTCCTGTGACT

>TN1206

GACCTGTAAAGCACATTTCACGGCCAAGCACACTGTTGGCCTCAGCATAGC

>TN1207

AAGGGGAGCAACATCTGAAAGGCAAAACTAGAACAAAAACATTTCTGTGTT

>TN1208

TTTTTTGGTCTAAGTCAGGGAGGGGAAGGTAGGTAGGAGCTGCTATATGTT

>TN1209

ACTGCACTGTGGAAAGATTGTCAATAAAACTGGAAAGTGGTCCAGTTAGCC

>TN1210

AGTTCTATGGCATTACTTTAAATTGAACTCAGTTTTGAATTCTCAGGAAAA

>TN1211

GGAGGGTGGTGCCCAGGACCCACCCAAGGGAAATCCACAGGCTGACAACAC

>TN1212

AAGAAATGAAAACTCCCAGTCTGGGAAAACAAAATTATGGAGGAAAACTAT

>TN1213

GATTATTTCACCTTGTATAACTACAAATGTATAATATTCATATAATAATAA

>TN1214

AGAGAATTTAAAGGAAAAGAAACTGAAAATGGATGTCAGCTGGGCTGATCA

>TN1215

TAAAAAATGAACTAGAATAAAGAACAATCATCTTATATGCCAATTGAAGAT

>TN1216

TCCTGAGCATTGGCCCCGAAGGCCTAAAGTCTGGAAACAGTTGCAGTTGAA

>TN1217

GCCTGAGCACAGCACCAAGACCGTCAAACGCTAAAAACAGGCACAGGTGGA

>TN1218

GACGAAATGCAGTCCAGATACCTAAAAGATGAAAACTAATCTTTGTGATTT

>TN1219

AATGAATGAATCAAATGAATGAATCAAATGAATGAATCAGATGAATGAATG

>TN1220

CTTTTTGTTTCTTCAAAGCTACCAGAACACTCAATACCCAGTGTCTAAATT

>TN1221

CTTTTTGCTTCTTCAAAGCTACCAGAATACTCAATACCCAGTGTGTAAATT

>TN1222

CTTTTTGCTTCTTCAAAGCTACCAGAACACTCAACACCCAGTGTCTAGTCA

>TN1223

TGGGTGCTTTTCAAAAGATCCTCAGAATCTGCATCTGCATTTCTTGACCAA

>TN1224

AATCAAATATTGCCAATATAAGGAAAATATAAGTGAAACAGCTTCGTTGTC

>TN1225

GTGTTGGATTTCTCATGGGGTCATTAACAAAGGGACTTACATGGACAAGTG

>TN1226

GACATGCTTTCATGTCTGAAGCAAGAAAGATTGATTTAATAGAACCAGAAA

>TN1227

CCACTCAGTCCTGCCAATAGCCTAGAATAAGCCAAAACCCAACCAGCGGCC

>TN1228

CAGACTTTACCATCCAGCAATGGAGAACTTCATTTTACTTAGAAAGATTTC

>TN1229

TCAAATTCACTATAAAAAATAACAAAAATATATTAGAGGAAATTTGGAGAC

>TN1230

TCCCGTCTGGTGAATATATTCAGAGAAGTTTTCACACAAGTACAGACATGT

>TN1231

ATTTTTAAGAAGGGAGAGATTTTATAACTTAGCTATGACACTTCAGAGAGG

>TN1232

ATGCAGCCATCAGTATAAGTCTTTTAAATCGCTTATTTGTTGTCATTAGCT

>TN1233

AAGTCAGAGTCCTTGATCCAAAGGAAAGAAGGACGGGCTGAGGGTGACGCC

>TN1234

TGAAACACAATTTGGAAGCCACTACAACTCAGTATGATGTTACTATTGTTT

>TN1235

TCTCATTGTATCATTCAATAAACTTAAATCCCAAGTGGGTGTTATTTAGGC

>TN1236

CTCCTTTTGCAGACACGGACGTTTTAAACGTTCTAACAGCGTCACGGCCGC

>TN1237

GACAAAGAGGATGGAAGGCTGGTGCAAAGAGATGGAGAGAGAGGCGGAGGA

>TN1238

AGGTCTCACTCCTGGGTGGAATGGGAAGGCCGTGTCATTCCTCTACGGGAG

>TN1239

AAAAAAAAAAAAAAAAAGAGCTCAGAAGCAGCACCCATTATAAATTGCTGT

>TN1240

ACATAATTGCACAATACCTACAACTAATGCTTATGTTTAACACAGTACAAT

>TN1241

TCACTGCAGAACAAGACCCTGTCTCAAAAAAGAAAGAAAGAAAGAATATCT

>TN1242

ATTTTTGCCCAAAATATAATTTCATAACACAGTTCCCCGAACTGATTGAAT

>TN1243

CAAGGCAAACACTAATGCTGGGAGGAACATAGGGATTTGCTCACTAAGTTC

>TN1244

ACATGGATGCTTGTCAGCACAGGAAAAGGAAACAGCAGAGTGAAAAGACAA

>TN1245

AGAACCAGAATATACAAGGAGCTCAAATAACAGAAAAAAAAAAAAATTCAA

>TN1246

TGTCCTGCTGGGCATAGACCCAAACAAAAGGAAATTCATATGTCAAAGGGA

>TN1247

CCGGAACAGGAGAGAGGAGGAAGAGAAGTTGATTCATACTAATGGGTACAA

>TN1248

GAGACTCCATCTCAAGAAAATAAATAAATAAAACAAAATAAACACACACAC

>TN1249

TCTCAAAAAAAAAAAAAAAAAAAAAAAAAATTAGTATTTGATTAACCAAAT

>TN1250

CATGTCTTTTCCTTAGGATCATTAAAAAGTATGCCTTCTCTAATACTTTCC

>TN1251

CCCAGCATGTGGTCTGGGTGAGCCCAAAGGACGGGTGTGTTGTTGCCGAGG

>TN1252

CAACACAGCCAGACCCCATTGCTACAAAATTAAAGAAATTAGCTGGGTGTA

>TN1253

AGCCACACATGGGACAGGTCCATGTAAGCCATGCATGGGACTAGACAGGTC

>TN1254

TGTTTCCCCGCACACCCATTCACGTAAGATGGTTTTCATGAACATACTGGA

>TN1255

GAAATTGCCCCACCTGTGAGCACAGAAACACGCTTCTCCAACACGGGCCGA

>TN1256

CCTTGCCACATTGAAAAATGTCCACAATTTTATGAATTGCTGTCCTAACCC

>TN1257

TGGCGTTTTTATCCAGTGTTAAAATAAATTATTTCAAGTTTCAATGTAAAC

>TN1258

TGTGGAATTCATTTTAAATTATAACAAGATAATTTATAGAATCTTTTAATT

>TN1259

AAAAACCAAGAGACCAGTGCATGAGAATTTCTGGTTTCTCATACAGCATTC

>TN1260

CAAACCCAGAAGCTCAACGTGGACAAACCTGAGGACTGAAAACTCTAGGGG

>TN1261

TTACCAGAAAAGCGCTTTAAATATAAAGACACTGATTAAAAGTAACACCAT

>TN1262

AATGCTCAATGTGCCTGTGCCTAAAAACAGTGTCAAGTGACACGAAAACCA

>TN1263

ACCCTACTTCGTCCAACTGCAGCAGAACACACAGGCTTCCCAAGCTACACT

>TN1264

CTGCAGCAGAACACACATGCTTCCCAAGCTACACTCTGGGCTATGAAACAC

>TN1265

GAGTTAAACTAGAAATCAGTAACAGAAAGATAGCTGGAAAATCCCCAAACA

>TN1266

AAAAATCTTAAGGGCAGCCAGAGAGAAAGGTCAGACTACCCACAAAGGGAA

>TN1267

GAGACCAACCTGGCTAACACAGTGAAACCCCATCTCTACTAAAAATACAAA

>TN1268

AAGACACAGACTGGCAAATTGGATAAAGAGTCAAGACCCATCAGTGTGCTG

>TN1269

TATTCAGGAGACCCATCTCATGTGCAAAGACACACATAGGCTCAAAATAAA

>TN1270

AACTATCCTAAATATATAAGCACCCAATACAGGAGCACGCAGATTCATAAA

>TN1271

CCCAAGTCTAAACCAGGAAGAAGTCAAATCCCTGAATAGACCAATAACAAG

>TN1272

AACTTTCTGTCTCATTGATCTGTCTAATGTTGACAGTGGGGTGTTAAAGTC

>TN1273

TCTTTTCAGCACCACACCTATTCCAAAATTGACCACATAGTTGGAAGTAAA

>TN1274

ATTGACACCCTAACATCACAATTAAAAGAACTAGAGAAGCAAGAGAAAACA

>TN1275

CATTCAAAAGCTAGCAGAAGGCGAGAAATAACTAAAATCAGAGCAGAACTG

>TN1276

ATAAAGAAGAAAAGAGAGAAGAATCAAATAGACACAATAAAAAATGATAAA

>TN1277

CGGGATATCACCACCGATCCCACAGAAATACAAACTACCATCAGAGAATAC

>TN1278

TTGAACAGACCAATAACAGGGTCTGAAATTGAAGCAATAATTAATAGCCTA

>TN1279

TACCAAAGCCTGGCAGAGACACAACAAAAAAAGAGAATTTTAGACCAATAT

>TN1280

GAAAAGGCCTTTGACAAAATTCAACAACCCTTCGTGCTAAAAGCTCTCAAT

>TN1281

TCAAACTATACTACAAGGCTACAGTAACCAAAACAGCATGGTACTGGTACC

>TN1282

AGAAAAAAAACAAACAACCCCATCAAAAAGTGGGCAAAGGATATGAACAGA

>TN1283

AAGCTGGAAACCATCATTCTGAGCAAACTATTGCAAGGATAAAAAAACAAA

>TN1284

TTGTGCACATATACCCTAAAACTTAAAGTATAATTTAAAAAAAAAGAAAAA

>TN1285

CAAAATTAACAAATGGGATCTAATTAAACTAAAGAGCTTCTGCACAGCAAA

>TN1286

TGAGGTACCATCTCACGCCAGTTAGAACGGTGATCATTAAAAAGGCAGGAA

>TN1287

TGTTTATTGCGGCGCTATTCACAATAACGAAGACTTGGAACTAACCAAAAT

>TN1288

TTCCTAGGAAGATACAATCCAACAAAACACACCTAAGTGGAAATATGTATT

>TN1289

CTGAATAGGACTGTATTTATTAAAGAAATTAAATAACCTTCTTCCCAAACA

>TN1290

AGGAACATAGATGCAAAATCCTCAAAAAAAGAAAAACCCAAAAATAACAAA

>TN1291

TATCCCCAGTACACAAGACTGTTATAAACATTTGAAAATCAATTAATGTAA

>TN1292

TCACCACATCAACAGGCTAAAGAAAAAAATCATAAATTTTATCAGTAGATA

>TN1293

AGAGAGCGAGACCCCGTCTCAAAAAAAAAAAAAAAAAAAAAAAAAAAAAAA

>TN1294

AAAATAGATCATAGACCTAAATGTAAAATGCAAAGCTATAAAACTTCCAGA

>TN1295

GTAAGAAAACAAATAACCGAATTTAAAAATTGGCAAAGAGGCATGGTCGCT

>TN1296

ACCTAAGGAGACACCTCATCAAGAAAATATCCAGAAGGCAAATAAGGCATA

>TN1297

TAGCCATATGCAGCAAACAAACTGGAACCATTCCTTGCACCTTATACAAAA

>TN1298

ATTAGAGTGAACAGGCAACGTACAGAATGGCAGAAAATTTTTGCAATCTAT

>TN1299

CCCAAAAGAAGACATTTATGCAGCCAACAAACATATGAAAAAAAGCTCATC

>TN1300

CATGCCAGTTAGACTGATGATTATTAAAAAATCAGGGAACAACAGATGCTG

>TN1301

TACCACACCAATGCAAGATATTAACAAGGGGGGAAATGGAGGTCAGAGTGA

>TN1302

AGCAATGCACAGATGTTGGGCTGGGAAAGAAAAGATTTCGGAGAGGACCGC

>TN1303

GTATTTAAACAAACAAACAAACCACAATGGGTGCGGCTTCCCAGAGGCAGT

>TN1304

GCCTGGACTTCCACCAATTTTAAATAATATAGCTGCACCAACAATATCCTA

>TN1305

AGCAATTTCCATGTGGAAAAAATTTAAAATTAAATTATCAGCCCTGAACTT

>TN1306

TGTCTCAAAAAAACAAAACAACAACAACAAAAAACCTAATTAGAGAGGCTC

>TN1307

CAGTGTTCAAGGATGTTGAATAAACAACGATTAGTAAAACAACATTGTATT

>TN1308

GTCGTGCACAACATCAGAAAACTTTAACACCTATTTCCTATTTAACCCTAA

>TN1309

GTCACCTCCCACCAGGCCCACCTCCAACACCGGGCGTCACATTTCCACAGG

>TN1310

AGTTTAGGGGAGGAAAAACCCCTGAAACTCAGCCCCCAAACAGCACAAACC

>TN1311

AAAGTGAAAATTATAATAATAAAAGAATCAAGTAGAAGTTTTAGAAATGTG

>TN1312

CAAGACCATCCTGTCTAACACTGTGAAACCCCGTCTCTACTAAAAATTCAA

>TN1313

AAAAAACAAAACAACAACAACAAAAAACACAAAACAAAATATGACAGGTGC

>TN1314

TAGGCAGCCTAAAACTACAACCTTTAAAACTCTCCCATAAAGAAACCTGTG

>TN1315

TGCTCTGCTCAGCTTCCGTAATTCCAAATGTTTTATAAGGGTGACTGCAGC

>TN1316

TGGAAATAGGGTCTTGTCTATTTCCAAAGAGGGAATGAAATTAAATCAAAT

>TN1317

ACACACAGCTGAGCCCTTTCAGAATAAACCACCATACTGTCACTCGAGCCC

>TN1318

CAATAAGAGGGAAACGTCATTTCAAAAAAAAAGAAAGTCAGCAATAAGTCA

>TN1319

CATGGAGAAACCCTATCTCTGCTAAAAATACAAAATTAGCCAGGCATGGTG

>TN1320

AAATACATAAACAGATAAATACATAAATACATAAATAAATAAATAATCTGG

>TN1321

GGCAACAAGAGTGAAACTCCGTCTCAAAAAAAAAAAAAAATCTGATGTTAA

>TN1322

TAGACATGTTGAGATGACAATAAATAAGGAACAGAAACATGCTCTTATCTT

>TN1323

GCATCTCTACACATCTCTAAAAAATAAGAACCTTTTCCTGTAGAACCACAA

>TN1324

AAGAGTGAAACTGTTTCAAAAAAAAAAAAAAAAGAAAGAAAAAGAAAAAAC

>TN1325

CTATTATCTCGGGGTTATCATTTTCAACAGGAAGAACCCTGCCAAATGTTG

>TN1326

TAAAAGAGGTGCCAGACTCTTAGTTAAATCTCTCCTAGATCAGGGAAAAGA

>TN1327

TCGTGCCACAGGCAGGCCAGGTGATAAGATACCGAGGGTGAGGGATGAGGA

>TN1328

AGCAGTGCCCAAGCTGAGACGGAGCAAGCTGGGAATCCGCCCGGGTTTCAT

>TN1329

TACAAAGGCAGTCTAGTCTCCAGACAAGAAAAGGATTTGTTTCGGGAAACC

>TN1330

GTAGGAGCGGGGAGGGACGCTGGGTAAGGTCACCAAAGTGTGCGGGGGAGA

>TN1331

GTATACATTTTCTCCAAAGAAAATGAAGATCTGCAGCGTCAAGAGATGGTG

>TN1332

GACCCACAGTAACGAGTTAGACTAGAACAGCACCAGCTTACCCAGAAAGTC

>TN1333

TTTGCAGACAGGTTCAGGCTTAGGTAAAGTGGACCCTTTCAGCATGATTCA

>TN1334

ATGCAGTAAGAAAACACTATGTCTAAAATGTTGAAATTCTTTGAAATTCTT

>TN1335

TGAATACTGTAGGCAAGTGTAACACAACTGTAAATATTTGTGTATCTAAAC

>TN1336

ATACCTAAACATAGAAAGGTATAGTAAAAATACCATGTTAAAGATGAAAAA

>TN1337

AGTGTGTATCATCTTTGCACCATAAAATCAAACAATTGTAAGTCAAACCAT

>TN1338

GTGGACTGGAACTTCGCACAGCCAGAAGCCAAGAGCAGGCCAGAAGGCAAC

>TN1339

GTTGGAGAGAATTGGGCTAAGATAGAAAATAACATGATTTGTTCCTTATTA

>TN1340

AGCTCACCTCAGAGTAAATCAGAATAAACTGCACCCAGACTTTCACGAATG

>TN1341

TTCAAAAATCATATGGGAAATGCAAAATCCCAATTGGAACACAGTGCATTT

>TN1342

GAAAGCACGCAGCCCTTAAAAAAATAAAGCTCAGGAAAAATCTCATTTGCT

>TN1343

AAGTGCTAATTTAACATTGTTTCACAAAGTGTGACATTTTTGTTTTTATTT

>TN1344

GGCGACAGAGCAAGATTTGTCTCAAAAAATAAAAATAAAATAAATAAATAA

>TN1345

GTTGGCGCACTGGGGCTGCCGTGGAAACTGCCCTGGATGTTTCCCAGTCAT

>TN1346

TTCACAAAATTGACGTCTCAACTCTAATATTTATGTTCTTGTCACAGACAA

>TN1347

GGAAGATATTTATAGTTTAAACGATAATAGCCCTTCTCCCGAACTGAACTG

>TN1348

ATGCCCAGATGGCCTGAAGTAACTGAAGAATCACAAAAGAAGTGAATATGC

>TN1349

TCTCGAGACCCCAAGTCACTAAGCCAAAGGGAAGAATCAAGCTGGGAACTG

>TN1350

TAATAAAACCTCCTAACATATTAGAAATGGAAGGGAACTCCCTTAATCTGA

>TN1351

GAATGTTCACATCAGTATTGCTTATAATAGCCCTAAACCAGAAGTGACAGA

>TN1352

AAAGAAGGAACGTATTGGGAACGACAAAGAAAGCCTCAGAAAAACACATGT

>TN1353

CTGCCTCAAAAACAAACAAACAAACAAAAAAACATGCAAATCTAAACATAT

>TN1354

TGTTTAATTATGCCTGTGATAGAAGAACGGAATGCACCGGTATGAAAAGCA

>TN1355

AATATAATTGAATAAAAAATCATTTAAAATTTCTATTAAACATTGGTGATC

>TN1356

TTTGGAATGATAAGTAATACAAAACAAGGCTTCTATACCCAAAGTTAAACC

>TN1357

GGAGTCATCCCAGCACGGACCCCGAAACCTAGGCAGGGTGGCAAATGGCAG

>TN1358

ACAGCCCAAGGACAGACCCCAGACCAAAGATGAGGAGGGCGGCTTTCAGGA

>TN1359

GCAAGAAAACAGGCTTAGTTGTTGCAAGAGCTCTGGAAACAAAAACATCAC

>TN1360

CACCTCCAACACGGGATACAATTTCAACATGAGATTTGGAGCGGACACACA

>TN1361

GGGGTATGACCCTCATGGATGGATAAATGCCATGACTGAGGCGGTGGGCTC

>TN1362

GTAGATAAAAAGTACAAGTAGATAAAAAGTACAAGTAGATAAAAAAATCAG

>TN1363

GTTGGCTATGATTTTTACATAGTCCAAAATTTTCAGGGAAATAACTTATTC

>TN1364

AGTCACTAACTTTGGCTGCAAGTAAAAATTCTCATTGACTCAAGATGTAAC

>TN1365

GGGCCCTTGTGAGGGAGCGATGGGGAAGCATGCAGGTTGTTAACAGGCACA

>TN1366

CAGGTCAAGGGCCTGTGTGGCTGGGAAGTGCTGGGCTGGGGCAGATGCAGA

>TN1367

TTCTGAGGCCTCCCCAGCCATGTGGAACTATAAGTCCAATTAAAGCTCTTT

>TN1368

CGGCGGGAACTGGGGCCGCGGCGGGAACTGGGGCCGCGGCGGGAACTGGGG

>TN1369

GGACGGGGTACTTCTGGAACCACTGAATTGCTGGTCACTGGGGTATGGGGT

>TN1370

AATAATAATTAGTTGTAATAGTTGTAATAATTAGACCCCATCTCTGCAAAA

>TN1371

AAAAGAGCACTTAAAACTAACTTCAAATTAGAATAGCCAAAATCCAGAACA

>TN1372

CGTGAAAAGACATGGAGGAGCCTTAAATGCATCTTACTGAGCGAGAGAAGC

>TN1373

ACCAGACATTGTCAAGCTTTTTTAAAAAACAATATTTATAGACTCATGTCT

>TN1374

TGTTCATATTTGACACTGATTTTCTAACCATCAGAGATATTTAATTAAAAT

>TN1375

TGGTTCCCTCCTCTCCACGCCCCCGAAGTGGCCTGTGGTTCCCTCCTCTGC

>TN1376

TCCTGCCTACCGAAACCAGCTCTCAAAGACGTCCACAGGCAGGTCCTGCTC

>TN1377

TAAGATAAGCCTCTGATTGATAGGAAAGTGTGAAACCATTTTTAAGGGTAA

>TN1378

TGAGGAAAACCTTAAATGATCTTCAAAAGGATGAAATTTTTCAAACATAGA

>TN1379

ACTCTAAAAAGATGTTTATTTACTGAAAATAGTAACATGGGCTGACTGTGC

>TN1380

GCTAACAGTTTTATTTCCGAGGTAAAATTTGTCTGATTTTTTTCTCATTAC

>TN1381

ATGATCTCTAAACTAGCAGTCTCTGAACATTATCTAAGAGGAGTAGAAATC

>TN1382

AGTTAATGATGCACTCGCTGCAGGGAAGAGCACACTGAACCACGGAGGAGG

>TN1383

TCATGCCGTGTGTTCTGAAGTTCTGAAGTCGGCATTTTTCAGGGGGAGGAT

>TN1384

ATAAGAGTACAGAAGATTTGTAAGTAAATGGATGGAAAAAGAAATATAATG

>TN1385

ACCCCATCTCTACTAAAAATACAAAAATTAGACAGGCGTGGTGGCGGGCGC

>TN1386

AAATCAGTAAAAAAAAATCAATACAAACATTTAGAAATTAAAAATGATATA

>TN1387

TTGAATAATAATAAAAACATTACATAAACTATATGGGATAGATTTTAAAGT

>TN1388

TTTTATCAAAAAGTTAGATGAAATTAAAAATTGCCTTGAAGAATCAACTAT

>TN1389

ACAGAAAATGCAGCTCCAGCTCTCAAAGGGCTGAAAATCTAATTGAGGTGA

>TN1390

GGTGATCATTGTCCGTGGCACACAGAACACACTGCAGCTTCTGCAAAGTGA

>TN1391

GGTCCTTCCTGCGCAGCAACCACAAAAAGCAAGTACGTGTTCCCTGCACAT

>TN1392

AAAGGGTCACATTTTAAAAACGACCAAAATAAAACTATATGTTGTTCAAAA

>TN1393

TTTTTATTCCACCAAAATGTAAACGAATGATTTCACAGATATTTTGTGTGC

>TN1394

TGTTTGGGAATCTGAATTTCAGAAGAAAGACTGGCCTGGGTTTGATGATGG

>TN1395

GAAAGATCTTTGCAAAAAAAAAAAAAAAAAAAAGATTCCTCCACATTCCAC

>TN1396

AAACTTTGTTTTCTTAGAACTTTGGAATAAATATTTTAGATAAGTGTTGCT

>TN1397

GTGACAGACTGAGACTGTCTCAAAAAAAAAAAAAAGAAAAATGTCTGAAGC

>TN1398

GATTTGCTGAAGCTTTTTGTTGTTGAACAAGGATGCCGTGTGTTTGGCAAT

>TN1399

GACTATGATGCTATCAAAGGGATATAAGCTTGCAAGTTTTGAAAAAAGTGT

>TN1400

AGTCATAATAAAGTTTATCTTATGAAAACATCACAGTGACCGAAAGAGTAT

>TN1401

AGTTACATGTCAAAGCTTGACTCTGAATGCAGTTATTCGTTATTAAGATTT

>TN1402

ATGGTATTAGGAATCAAATAAACTAAACATCTCAATTCCAGGCCTCTTTGG

>TN1403

GACTCCCCCCAGGTCAACATTGCCCAACAGCCTTGGTAGCAAATGGCCCCA

>TN1404

GGATGGACAAGCTTGGAAAGCTAAGAATAGATTTAAACAAAACATACCCAT

>TN1405

AGATGCTGATCAACGCTGTGAAGTGAAGCAAATAGCCAAAGCCATAAACGA

>TN1406

GCTTGGTCTCAGAACAAACAAACAAAAAAGGTAGTGTGACTTCTACATCGC

>TN1407

TGTAGTGAGATGAGGATATGTGAGAAACTAAAAGGTCTTCAAATACAGGTA

>TN1408

GCACATCCACCTTCTCTGAACTCTGAAAATTACCCTCTCTGACTTTGCCTC

>TN1409

TGGAGACTAAAGGTGTTTATCACTTAACAGTATGGCTTCCAAGTTTAACTG

>TN1410

CCAGACGTGGTGTACTTTAAAGATGAATTAAAATGGATACTTTAAAAGTAA

>TN1411

GTATATGTTCTATTTATGATAAAAGAAGAAAATAATCTGTATAAGTCATAG

>TN1412

TTTTTTTTTTATCACGTCTCTATGGAAAAGCACCTAGTTAACCTCAGTGAA

>TN1413

TGGCCCATTCCGCTTTCTGTCCCGGAAGCGTGTCCTGAGTGCCAGGAGTGC

>TN1414

TGCCTCTGTGCTCCATTGCTGTGGGAAGGGCTGCTTCTGCGTCATCTGAAC

>TN1415

TTTGATGAAAATGGCATCTGAAGTGAATGGAGCAAAACAGTCACATCACAG

>TN1416

TTTAAAGAGTATTTCATACTTTCACAAAATGAGAGCCCATTAAGGAAGCCC

>TN1417

ACACTTACCATTTGTTCATGGTGAGAACCTTAAAGTCTACTCTTAGTGATT

>TN1418

TAATCTGAAATGTTAACGGAGTATAAAATACAGAAGAGAAATGCAGAGCGT

>TN1419

ACAGCCCCTCTCTCTCTTGCTGAATAATTTGGTCATTTTTCAGGTCAGGTT

>TN1420

TGTATGTAGCGACAACAAATACAAGAATAAGGAGAGTTTAACAACATTCTC

>TN1421

CACTCCCAAAGTCATTGAGTGCTTCAACGTGGAATCTCGCATCCTGTGCAT

>TN1422

TTTTAGGGTCATTGCACTTTGAAATAACTAGTGAATTGGGCCCATGGAAAT

>TN1423

GTGAAGTTCCAAGATACACAGTAGGAAGAACTAAGTTTTTTCATATATCCC

>TN1424

AAGTCGACGGCATTGATGGATTAATAATTTAGTTACACTTTTAAGGTCGAT

>TN1425

CGACTGTCTAGGCTTCAAGAAAGGGAACAAATTCATCAAAAGTAAGGAATT

>TN1426

CCTTTTACCTCAATTTTGATTTCTTAAAGTATTCTGTGAAGATATGGCCTC

>TN1427

CAACAAAGTGAGATCCTGTCCCTACAAAAAAATTAGCTGGGTAGGGCAGTG

>TN1428

CTCCAAAAAAAAAAAAAAAAAAGGAAAGGGAAAGAAAATTCCTTCTAGATT

>TN1429

GCACGTGCAGGGAAGCAGGCTGCGGAACACTGTCCGAGCTCACTCCGAGGG

>TN1430

GAGTAAGACCCTGTCTCAAAAAAAAAATTCCTTTGACCCTCACACAAAATG

>TN1431

CTGCATCCTCTGTCATCTCAGAGGGAAAGTGGCTCCTGAGCTGCCACGTGG

>TN1432

CTCTTGACTGAAGACAGATGGAAGGAAGGAAAGAGAGGAAAAAGGTTTCTG

>TN1433

GCCCTCATCCTCCACTGAAGGGAAAAACAATGAAATAATAAAATAGGCGAG

>TN1434

ATATTAAAGATGACCTAAATAGTGCAACATAGTGATTTGCTGCAGCAATGC

>TN1435

ATCTGTCCTCCAGAAGAAACATAGCAAACATTTACCAAGTACCTAGCACTG

>TN1436

AGTACTCTTTCCCAAACTCTCCAAAAATATTGATGAGGACGGAACAACACT

>TN1437

GAAGAAAAGTTTTCCCCTGAAGATCAAGAACAAGACAAAGATGCCTTCTGC

>TN1438

GAAAAAGAGTGGAGGACTCAGATGAAAGGAAGAAGTAAAGTTGCATTTGCA

>TN1439

ATAGATTGGAAGATGTACTATTGTTAAGAAGAAAACAGTCCCCAATTGGAT

>TN1440

CATACAAAATTATCTCAAATGAATTAAAACATAAATGTAAAAGCTAAAACT

>TN1441

GCTGCGGGGCTGCAGCTGAGCCTGGAAGCTCCAGAAGGACCAAGTTGCTTG

>TN1442

TTCAGAGTTCCGAGAGCAGCCACGGAAATGCAGCGGCCCTGCAGGATCCTT

>TN1443

GCTATGAAAAGCAAAGCAGGATAGGAAAGCCTGCGCTCTGGGGACTCACAG

>TN1444

GTCTAGTCGTCTTTGCCTTTCAGCTAATATTTCATATATTTCATAGAAATA

>TN1445

GTTCCTTTCTGTATTTTATGTTGTGAAGAAGGAAGCCGGGATCTCCGTCTT

>TN1446

TCTCAGCACTTTACAGATGACTAAAAATGTTAATTTTATGACTTAGCCAAA

>TN1447

TCCAGGAAGGCAGGATGGATTTACTAATTTGGTAGAAAAGGGGAGGGGGGT

>TN1448

CTTGGTGTTTCATCACAGCAGCCCGAACGGAGACAGGAAGGCACCAAGAGG

>TN1449

CTGTGGAGGACACGCCTCTGCACACAACGGCTGCACACAGGGCACGCAGTC

>TN1450

AGGGCTGGAAGCAGAAACCAAACAAAAGCTGCCCACAGGAATCCGTGCCCT

>TN1451

TCTATTTTACATGTTTCAGATTTAAAACATCTATTTTACTTGTTTCAGATT

>TN1452

AGATTTAAAACATAAATTTATATATAAAATATATATTTATAAATTTATATA

>TN1453

TATATTTATAGATTTTATAAATAATAAAATCTATAAATCTATAAATCTATA

>TN1454

TCAGGACAATTAGGGTATCATCTTAAACCTGGGACTGTCTGTAATTTAAAA

>TN1455

GTTCATCAGAGCTGAGGAAGCACTGAAGTAGGTAAGGTTCACCTAAGGGAG

>TN1456

TGCCAGGGCCGGGTAAGGTTCACCTAAGGGAGTGCCAGGGCCGGGTAAGGT

>TN1457

CTAAAAAAAAACAAAAGAGAAAAAGAAGTATTATTCTGCAGCCGAAAGGAA

>TN1458

GTGGGCCGCGGTCCTGGAGGGGGAGAAGCGCCCGGCGGGCAGGCGCGGGGC

>TN1459

GCAGATTTATGGTAACTGGCATTGCAATGCTCCCATCAGGGCATAACTAAG

>TN1460

GAGGGGCGCTGGAGCAGGGTGTAGTAAAAGTGTAATGTCAGCCGATTTTAT

>TN1461

GCATGAATGGGTTAAAAAAACATATAAGACCCCACTATATGCTGCCTACAA

>TN1462

CTATCTCTCCAAAAAAAAAAAAAAAAAAAAAAAAAACCACGCACACACACA

>TN1463

TATTTCTACTTCTAGATCCCTGAGGAATCGCCAAACTGTCTTCCACAATGG

>TN1464

GCTGGCGCGCTGTCTGGCACATTGTAAATACTTCATATATGTCTGCTAAGT

>TN1465

AACTTTGATTTCAGTTTTAAAATGAAACAGATTTATTTTAAAAGAGTCAAG

>TN1466

CGAGTTTCAGTAATTTCACAGTAACAAGGATGGACGCTCCTCAGGGAGCCT

>TN1467

AAATTTGAAACTGAAAATGCTCCAAAATCTGAACTGTTTTGAGTGCTGACA

>TN1468

TGAAATAATTAACTGGGAAACACTGAAAGAAAACTTGTGATCTGTTAATAA

>TN1469

CATGCTACTTACACTTTTTATGTTGAAAACAGTTAATTGTTGATACTCGAC

>TN1470

GTGGTAGACTCCGCTGACACGAAATAACCTTACTGAAAAACGCACAAATAT

>TN1471

CCTCTCTTTTTGTCCTATAAACAGGAACAAGAGTGTGGTGAGAGGACGCGG

>TN1472

TAAATGTGACCAAACAGCACATCATAAGTAGGAAAAACTTACCAGGGTGCT

>TN1473

AGAAACGTAAAGAATAAGAGGAATTAATACCCACCATTAAAGGATGTCCGG

>TN1474

CGATTCTCAAATGTTACCATTTATTAAAGGTAAACTACACCTGTTGAAGGC

>TN1475

AGAGGCGATGAGCTCATAGTAAAATAAAGCGGAGGGGAGTCCAGCCTGCAA

>TN1476

GCCAGTGACAGGAGGAGAGGGAAGGAAAATGGCAGCTGGGGGTCCGGAACG

>TN1477

TCAGACAAGTTTTAGGCTCACAGCTAAATTTAGAGGAAGGTTCAGAGTTGC

>TN1478

CAAACCATCCTTGCATCCCTGGGATAAATCCCCTTTAGTCATGTTGAATGT

>TN1479

GAGGTCGAAGAGGGTGGATCATTTGAAGTCAGGAGTTCGAGAAGAGCCTGG

>TN1480

CTAACCAACAAACAAAGAGAAAACTAATAAAAACCCTACACTTTAACTTTG

>TN1481

GATATTACCATTTTATGTATTTTAGAAATGTTAAATATATTCTTCCACATA

>TN1482

TCCAGGGTCTCGAAAATTTCCGCGTAACCAGGAACCGCAGGTCGAAGGCCA

>TN1483

GGAGTAGACTGTCGGGGGTCAAAAGAAACACAGGGCTTACTCCCTGCTCCG

>TN1484

AAAAATAAACCTTATATAGTTCAGAAATGTTTAAGAAAGAAAAAAACTGAA

>TN1485

TTTGAGAAATTAATAAGGAAAACAGAAAAGGAGTCTGAGAAGGTGCTACTA

>TN1486

AGTACCTATCCAGCCATCTTATCTGAAGCCAGACATTAAAGAGATTTGCAA

>TN1487

AAACCCCCTCTGTACTAAAAATAGGAAAATTGGCCGGGCACTATAATCCCA

>TN1488

TGTGTATTATCTGTCAGTTCCCAGGAACAGTTTTTAACAATAAAAAAAGAA

>TN1489

CAAGAAGTTCATCTTTTAATTTATGAAGAACTGAGTACTCTGAGGCCATAA

>TN1490

GCACAAGCCTGTGCATTTTATATATAATTGAGCAAAATTAAAATTTTATGT

>TN1491

ACATGCCACCGCCTCTGAAGATAGAAACCCATCAACAGTCGCTACAGGACG

>TN1492

GTTGATAAAATAAAATATTAAGAGAAAAAACACTGAATGAATAAAAAATAA

>TN1493

ACCCACTACATAGAGTTATAGTATTAAGACTTGCTGGTACTCTGAGACATA

>TN1494

GTAAATGCTTGATAAATATTAATTTAAATGTATTTAAAAGTATATATATTT

>TN1495

GATAATTTCTAAAATAATTTCTAAAAAACAGAAAATTTGTTTTCTGAGACG

>TN1496

GCTAAAGCAGCCTGAATAGACTAAGAAATCACTCAACAAACACTAATTAAA

>TN1497

CAGGCATGATGCAGATTCAATATGGAACGCCAGAGCTAGAGGAGCTAGAAG

>TN1498

TAGTGTGGCAGTGTGAAAGGCCCAGAATAAGGATGGCCTTGGGGCAGGGGT

>TN1499

TGCAAGTTCCCAGAGAAAAAGATGGAACTCACTCATCTCCTGTTTGGGGAG

>TN1500

TTAAAATTATTTCCAGATGACGGAGAAACAGGTGGTGATATGGCTTTGATC

>TN1501

ATATCTTCAAAGCATTTAAAACCTGAAGGAAAAATAAGATTTGAGTAAACA

>TN1502

CTCTGAGGGAGAGACCCCTGTGCTGAAAGCAGGAGGAGCAGAAGCCAGCCC

>TN1503

GAATGACAAGTCCTGAATTCCTAACAAAGGTCAGGCAGGTGTTTGATCTGC

>TN1504

AAATGTAGACAAATAAAAGAAAAACAATCATCTATACATCCAGCACCCAGG

>TN1505

TGTTTGTATAAATAAGGTAAAAAATAAGTGAGACATTGGAACATACGATTT

>TN1506

GAAGACTCTCTCCATCTATTCTGGGAACCTGTCAGAACAGGAAATAAAACC

>TN1507

GAAAAGGTGAGACAGCGGGAACCATAAACAGAAGGCCTAACTTACCCGTGC

>TN1508

GTCTACACTGACACCACAGTTTAAAAACGGCTAAAACACACGGAGGAAGAA

>TN1509

CATGGCTGTGCCTGTGGTGATCTGGAAATAGGACAGCAGCGTAGCTTCTAA

>TN1510

GACAGAGCTGGCATTGTTCTTGCACAAATCACTCATGAACAAATAAACCAC

>TN1511

AAACATCCATGTATATATAAGCTGGAATTCTTACAGTGATAGCAAAAGGAA

>TN1512

AGCCCCATAAAAAGGCAATAAATGCAACACGGCTCAAATCGCCTTGCTCAC

>TN1513

TCAGCACCAGCAACAGGAGGAAAATAAGGCAAAGGTGGGGAGTTTAACCAT

>TN1514

AGGGTCATCAATAGACTGAGAAACGAATGATGCAATATTGTCCCAAATATT

>TN1515

TTATAGTTGAAGAAAATGAATTTAGAAATTAGACAGAGAGTATAAGAGGAT

>TN1516

ATGGTGAAACCCTGTCTCTACTAAAAATACAAAAATTAGCTGGGTGTGCTG

>TN1517

AATGCAAAATTTTATATGTATATTAAACAGAAAAATAAGGTGATTATCTTA

>TN1518

GGTAAACTCCGGGCCCGTGTCCTGGAAAAGTAGATCTCTGCATGGCCCCCC

>TN1519

GCTCTGTCGCCAAGGTTTTTAAAGTAATTACAAAAGATAAAAATTAAACAA

>TN1520

AATGAGCAATAATTCCACCATATAAAATAGCCACATTAACGTGTGGTACAT

>TN1521

TTAGATGTTCTTCACAGCCCACAGCAAATGCAGAAAGATCGATCAGAATAA

>TN1522

GAGGAGGCCCAGAAAGTACAAAAGCAAAGGTGAACTCACAGAACAAGTGGC

>TN1523

TGACAGTGTGAGAGGGAGAAATGATAATACACTAATAAAACGCAAATTAAA

>TN1524

TAAAAAATAGCATCATGAGATCATGAAGTTTTGGAGCTCAAAAGATCCTAG

>TN1525

GAATCATATTCTTTTTCTTTCTGAGAAGCAGTGAAGAAGGGAGCTCAGTGA

>TN1526

TCCCTTACAATGTCTAAATGTTCTCAACTTAAAAATTATTTTATTTTTAAT

>TN1527

CTGTAATCCCAGCACTTTGGGAGGTAAAGGCGGGCCAATCACTTGAGGTCA

>TN1528

GCTACTCGGGAAGCTGAGTCAGGAGAATCGCTTGAACCCGGGAGGCGGAGG

>TN1529

TGTATGTACAAGTAGAGATGGATTTAATTCTGATGCCCCAATGATGACTTT

>TN1530

TAAAATATGCAGAGATAAAAAAATGAAAGAGGTTCCATCAGATTGGGAGCG

>TN1531

TAAAAAAAATTAGCAACAACAAAACAACCAAGCCCCTCCAGGTCCTCCCTA

>TN1532

TATCATGCAGAGGCTGTGATCTGAGAACATCCGACCCAGGATCGGTGCTGT

>TN1533

AGATCACCATCTTCTTTCTGATTCTAATTTTAAGAAGGAAGGAGAGAAAAT

>TN1534

TCCCCCCACTGACAGCTTCCCATGGAAGGCATCCGTGGGGAATAAGTTTCT

>TN1535

AGAAGCACAAAAAGGTAAAAATATCAAGCCCCCAAATAAGGCAACCAGCAA

>TN1536

AAAAAAAAACAAAAAACAAACAAACACAACATAAAAAAGAGAAATAAGCAT

>TN1537

ACATCACAAAATCAGAGGGAAACAAAAGATCAAATTTGCTCAATTCATCTG

>TN1538

ATGCAATTGTTAAATGTTGCAAGGGAAAACTAGGAGAGATTTCCTTGTATA

>TN1539

GGAGTCCAGCAATTTTAATGAAAAGAATAAAGAATGGGGGTAGAGGAGGTG

>TN1540

ATTAACATTTTTTAAAAACATATATAAACCTGTCTTTGATTTTGAATGCTA

>TN1541

CTGCACAGAAAGGTATGTTTCTTCCAAATTTTAGACATCAGCACTGCAAAA

>TN1542

CCAGCAATGTAAAATTTTTTTGAAGAAGAGATGAAGAGAAAACAGTATTGA

>TN1543

AATTTTGAAATGTTAAAGTAAAGGGAATCGTCTGCTAGAGTCGTGGAGCCT

>TN1544

GAACATCGAATTGTCAGACTTGTATAATCTCATGGGATTCAAACATTCTGC

>TN1545

TGTTAATGACATTTCATATAGACCAAAAGATCTTTATGCCTAGTCTAATTT

>TN1546

AAAACGGCACTTTCAAATAACTCGTAAATTCTACAATCCTCTAATTTGCAT

>TN1547

CCTGAATTTCGGTGGTTTGCAAAGAAAATGTATTAGAGTTTATTACGTTTT

>TN1548

ACTCCTAGAAGCACAATAACTATGTAAAAACTAAAAGTAATTTTAAGATTC

>TN1549

TAATGAATGGAGACAGCATGGGAATAAAGTATAAGAATTTGTGGCTGGCCG

>TN1550

TGATTAAAAGTACCAAAACAAGCCCAATCATTAAAATTAAAAACAGAAGAA

>TN1551

CTGGGTCGGTACGGATCACTGAGAAAACTACCAGAGACTTCTCATGCAACC

>TN1552

CTGGTTTGCTTAGGTGACTGTAAGTAAATTGAAGAGTTGAAAGCATGTATG

>TN1553

TTTATCGGGATTTATTTTGTGGGACAATGAGTAAATCTATGTTTCTAGCTG

>TN1554

TGTTCTTTAGTTAAAAATGCTGAGTAATACATCAGAGGCTATAGAACATGT

>TN1555

AGAGGAATTCAGTAAAAATGTTCTTAAGAGAAAAGATTTTGTTCTCTTACT

>TN1556

TATGTCTCTAAACAACAATAATAATAATTTTAATATCCCTATATGTCCAAG

>TN1557

CCCAGATTGTGTGGCTGTTGTAAAGAATCTCTCGCTGAAGTAGAGGGTCAC

>TN1558

AGTATCCAGGCTTTGCAGGGCAGGGAAGCAGGGTGGGGCCAGGCAGGACTG

>TN1559

TTCTCTGTCCGGGATCCCAGAGGCTAAGTCTCAGGGTGAAGCCAATCCCAC

>TN1560

GCTGGAGAATTCCCTTTCCCGATGGAACATAGGCATTCTTGGTTGCCGGCA

>TN1561

TCGGAAATCATTATTGTGAGAGTTGAAGAAAGAGAAAAGAAACACGCAAAG

>TN1562

TGACCCGAACATGTTCTTCTAAGAGAATGCAAACGTATCTCCTTCTTCTTT

>TN1563

CCTGTGGCCATTCACCCAGAGGAGCAACTGCAGGATGAGGCAGCCACACTC

>TN1564

TGTACATAAAGGCTTGTTTCTAAACAAACGATTGAAGGACCAATAAATGTA

>TN1565

TTTTTAGATTCCACATATGAATGAGAAGATGTGGTATTTGTCTTTCTCAGC

>TN1566

TGATTCCCATATAAAGTAAATCACCAACACCCAGGTTTACCTTTGTCTATC

>TN1567

CTCAGGCAGTCCTCATAGCAGTATGAAAACAGACAAATACCACACCCTTGG

>TN1568

AGACTGGCTCATTGTCATGGCCAATAATAGTTAAAATTTACTGAGAAATTG

>TN1569

TTGCTGACAAAACCAAAACTGAAACAACAACGGTAAGAATTTTGACATGCT

>TN1570

CAGATAGGGCAGAAAATACTGTCAGAACAAACAATATCATCTAAGCAGCAA

>TN1571

GAATCAAACCAAATTAATATGAGGCAAAAGACATTACACTCTCTATCTTTG

>TN1572

AAAACACAGCAAAAGGTTCTGACAGAATAGAAAGATATTCCAGATTCTAGC

>TN1573

TCCAAAATTGCTGAGAAAGAGTTAAAATGTCAGTAGCTGAAGTAGGGAGGG

>TN1574

AAACAAAAAACCCCATAAAAACCAAAAATTAGCCCACAGTGGTTTGTGCAC

>TN1575

GAAGGCACAGATACAGTGTTGCCGGAAATAGAAACTGTTACAACATGTTTT

>TN1576

CCTTCTGGGTGAGTTAGACAAAGTCAAAAGAGCTGGGTTCCAGCTTGCCAT

>TN1577

AGCTTACATGTCACCAGTGAGTCTGAAAACAGACCCACCTGGTCTTTTTAT

>TN1578

TTTCACCTTACCTTAAAGATGGGACAAAAGAAGTCTCTGGTTAGTTGAAAC

>TN1579

ATGGTGATTTAGAGAATTCAATTATAATCCTAACCATAGAGAAACTAGGGT

>TN1580

CTGGTCTCAGTTCTCCCTTCTTCCAAAGGGCAATGGCAGGCTCTGCACAGG

>TN1581

CGTGTGTGCCTGTACTGTCAAAATGAAGGCCCGCGATAACCCAGGTTCCTG

>TN1582

CGGATCCCGGTCCTCTTCCTCGAAGAATCCATGGAAGGCTCTCGAATGGCT

>TN1583

TAGAAATGAACAGATTTGAATCATCAATGCAAAAAGATCTCCCAATGAATA

>TN1584

GCACAGACATTCCCATCGCAGAGCAAATGCAAACATTGAGAGAAGCCACAA

>TN1585

ATGTGGCATTCCCTCTATTGTGTGTAAGGTCAACACCATTCTATTTTGAAT

>TN1586

GCACCCCTGACCTTAAAAGTTAAATAAGAAAACCAAAACAGCCACAACAGC

>TN1587

CTTGTGTACACGTGAGCCTGTGCCGAATCGCAGGGGTGGTTTCCGTGCTGT

>TN1588

AGAAAAAAGTTATTTGTGTAGGAGTAAACTCCTGGAAAGTTCCCTGAAATC

>TN1589

CAGTGCTTCTGCAGATGTGCTTTCTAAGCATCTAAAATAGAGACATATAGG

>TN1590

AGGAAATAGAATGGATAAAGAAGAAAAGTGTAGGAATAAAGAGCTAAGGGA

>TN1591

TACTTATTCTTATGAAAACCATTCCAATTTGCTGATATATTGTAACTTGTG

>TN1592

AGGACTGAAGGAGACAATATTGGTCAAACAACAGGACCTTGCCAGGTGAAC

>TN1593

CTTGATATACGTTTTTGAATGAATTAATGAGTAACTGATCCTAATTAATAG

>TN1594

TTTCTTTAATATATTGGAGACATAAAAGATACAGTAGAAGTTTTATAAATT

>TN1595

AGGCAATAACACTTACCAATTTGTAAAATATGGAAAAAAATTCCATTTCAT

>TN1596

GTTATAAAAATAAACATTTAAATAAAACAAGATTAAATTCCTGCAGGAAAA

>TN1597

ATTTTCACCCTGGCTTTAGTCTTATAAAGGTGCTCATTCTCGCATTGGACC

>TN1598

AAGCTGAGGAAACAGAAAAAAAAAAAATGGAAAAGAAGAAGTGGTCAAGAA

>TN1599

TTCGCAGCCACACATAAACTCACAGAATAGCTTCTGATGGAGCACAGTAAA

>TN1600

TCTTCTTCCTGCTCCACAACGATTGAAGAGACAGGAAACAAACTTTTAAAA

>TN1601

TTACGTAAATAAACGGGCCAAGGGGAAATACAAGAGGACAAGCAAAGACAG

>TN1602

GACAAGCCAATCAAAATTGAAGGAAAAATAGAATCAGCACCTAGTGTAAAT

>TN1603

GGTTTAGTCCCACTTAGCAGGTAACAATTGCCAGCCAAGCCGCTGGATTAC

>TN1604

TTCCTAGTCAGTAAGATAAATGAAGAAGGTAGTTTTATGTCCATAAAGGCA

>TN1605

GAAATGTTGAAATCCTGTCAGAGAGAAAGAGCTAATTCAGGATATGGATGG

>TN1606

TAAAAACCAAAACACAGGTTTAAAGAAAAAGATGAAAATTGGATTATTGAA

>TN1607

GGCATCCAGGACAGAACTGAAGTTGAACAGGAGCAGATGGGAAGACAGGAT

>TN1608

CAGGGTTCCTCTCACAACACCTGGGAATTCAAGATGAGATTTGGGTGGGGA

>TN1609

ACCTGAAGACAGAAATCTGCTAAGGAAATTTTCATTAATCACACATACCAA

>TN1610

CAGTGCTGCCTGCCCTGCCTCCAAGAACAGCCTTGCGTGCTGGGTGTTCAC

>TN1611

CCTTCAAAGTTGCAGGAAGAACTGGAAAAGTTGATCACGTATGAAGTTGCT

>TN1612

CAGAGCAGGAGCAGCAGCACCTCAGAACCCGGCTCAGGAACCCGATGTGAC

>TN1613

ATTACAAAACATGAATAATTGAGAAAAAGAAAAGAATTTATTAATACATCC

>TN1614

CTAAACGGGCTTCTGAGCGGGAGTAAAAGACCACCCCAGAAAAGAGCTCCT

>TN1615

CTAGGAAAATATAAAAAATACAACAAAGCTCAGAACTGAAAAAGAGAGATG

>TN1616

ACTCCTTACACTATAGCTATGGGGGAATAGAAAACTTTAGGCACAGCGCCT

>TN1617

GAATACAGGCCATCTGTTTGGTAAGAACACAGTTTATGATCACAGCATAAG

>TN1618

AAGAGGAGTTTAGGACACAGACACGAAGAGACGGAGGCTGTGAGGACACAG

>TN1619

AAGCATTTGCATCGGTAAGTTGTTTAACTAGAAACAGAATATTTGTGAGAT

>TN1620

GTAGCATTTCTCAAACACTATTTTAAATGGAAATTTAAGTTCTTTAATTCC

>TN1621

AAGGGTGGTATCCCAAAACCATCCGAAATGCCTGAAACAGACCCAAATCTA

>TN1622

AACCTATAAAAAAGATAAATAAGTGAAGAGTTAACCAAAAAAAAACCAAAA

>TN1623

AACTTTCGTAAGGTCTTTGTGATGTAATGTTATAATTTGCTCTTTGAAATG

>TN1624

CAAAAAAAAAAAAAAAAAAAAAAAAAAAAGAAATTAGGCAAATCCCTCAGG

>TN1625

CTTCCCTAATAACTGAGGCCACTTCAATTAAACTCTACATGGATAAAATAT

>TN1626

GGCAGGAGAATGGCGTGAACCCGGGAAGCGGAGCTTGCAGTGAGCCGAGAT

>TN1627

TCAAAAAAAAAAAAAAAAAAAAAAAAAGAAGATCTAAAGCAGGCAGTGAAG

>TN1628

TGTTTTGCTTCCCTGTGCTAAGTTTAAAGCTGCCATTCTCTAATGTCCATC

>TN1629

CACCATGGCACATGTTTACCTGTGTAACAAACCTGCACATCCTGCACATGT

>TN1630

TGTCCTGAATTGGGCCAGGTCACCTAATCTCTCAGCCTTCCTTCCTCATCT

>TN1631

AGAGCTATGTGCAAAATTAGGGCAGAAGAAGAGGGTCTGGGAGGGCTGGGG

>TN1632

TCTGTTGCCAGGGCTAGGAGACCTGAATGTTGGGAGAACCACAGAAGACCC

>TN1633

AGGAGGCTAAGCCTGGAAGCGTGGGAAGCTCAGGCGGGGGTGCTGGGTCGC

>TN1634

TGTTATTATTTTATCCCCAAGGTCCAATGTGTTATTAGATATTCAATACTT

>TN1635

TAAATGCATCTTTTGAAGGATATAAAAAATAGATAAAATTATGTATACAAT

>TN1636

TATTTGTGACATACACATGCTCATTAACAGTAACATATAAGCATGCATCAA

>TN1637

CACTGTCACGCAGGCACACGTGCGCAAACATACATGCACACATCCACAGAT

>TN1638

CCTAATATTGGGGCATTAAAAGAATAAGTTGTGGTATAAGAATCAGGAACG

>TN1639

AAAAAATCTTGGAGACAGGGAAAGAAAAGAGGAGACGAGGAACAAAAAGAG

>TN1640

CCAAAATCATCGGCAGGACACATGGAAGGGGAGTGTGAATTAGGGTAAAAT

>TN1641

AATTATTAAGAATTTCTAGATGGGGAAAGCAGAACATTAAATCAAGTATGG

>TN1642

CTCAAAAATAAATAAATAAATACATAAAAATAAAAAAACCAAACTCTTCTA

>TN1643

CCACCCAGGTCTCATGTTGTACTGTAAACACCAACACTGATGGGGGACCTG

>TN1644

ACTCTGTCTCTACTAAAAATACAAAAAAAAAAAAAAATTAGCGAGGCATGG

>TN1645

CCAATAGTGACCACAGTCATGAAAGAACATTCAGCCTGAGACAAAAGGGGT

>TN1646

GTGGGGCTGCATTAACGATGGAAAGAAAGGGGCTGCCACAGGGTGAGGCGG

>TN1647

AGTATTAGAACAGCTCAATCCAACTAAAAACAAACAAACAAACAAACAAAA

>TN1648

CCTAACCCCATCCTGGACCTGGAGGAAGCGTGATTCGCCACCTCCATTGTT

>TN1649

TATGCCAGCCATGCACCTTTGATTCAATATTCATGTATTCTCCCACATACG

>TN1650

GAATTTCAAGGCCATGTTTAAGGGGAATGAAACGAGGCCTCATAAAGAATG

>TN1651

TTTCAAACTTCCAAATTTTAACCCCAAATCCACTAGAGCCCAAATACCCAC

>TN1652

ATGCAAATTTAATTTCTGAAACCTGAAGATAGATAAAACTCATTTTAATTG

>TN1653

GGGGCCAGAAGCGTCCGGGCTGTACAAATAGTACCACCATCTTCCTGGAGT

>TN1654

AATGAGAAGCCGTTCAAGCATGGGGAACTTTTCCAGAATGAGAAGCTGTTC

>TN1655

TAAAAAGGGGTGTACAGAGAGAAAGAAAATTTCAGCCAGAGAGAATTCTTT

>TN1656

TGGAAATGAGCAGGAGGATGACTGCAATGGCCCTTCAGTTAGGTTCAGGCT

>TN1657

ATTAATTCTGACCAGAGTAACAGAGAAGGCTTCATGGATGACACAGAACTT

>TN1658

CAGAGGGAGGATTAAAGCACAAGTAAAGCAGGATTTTTTATTTTAGCAAAA

>TN1659

CCCCAGCCTCAAGTACAGAGTCCCCAAGAATTTGAAACAGGAAGACCCAGT

>TN1660

TACCTAGGGGTGGGGATAAGGAGCAAACGTGGAGATTGTGATTTTCTAAGA

>TN1661

AATCGGGATAGGAGGATTCATCAATAAACTCCAGAACTATACAGGACTCCC

>TN1662

GATCTACCAGGCAAAGCGCCTCCAGAAGGCAGCAGAGGGCTTGCTGTAGGT

>TN1663

GACAGAGTGAGACACTGTCTCAAAAAAAAACAAAAAAAACCCAAAAAAAAC

>TN1664

ATTTTTATGTGTAACAAAAAGTAAAAATGTGCCTAACAAGTATAATTAACC

>TN1665

CATGGATCATTTTGAGAGTATGATTAAACATCTAATCTCCAATTAAAAAGA

>TN1666

TTAAATAATTAATTGTAAAACATTTAAATTACGTATACTGGCTGGGAGCAG

>TN1667

ACACCTGTAATCCCAGCTACTCGGGAAGCTGAGGCATGATAATCGTTTGAA

>TN1668

CTTGGTGACAAGCAACACTCTGTCAAAAAAAAAAAAAAAAAAAACACACTT

>TN1669

CTCCACCTCAAAAAAGACAAAAAAAAAAAAAAAACTATAACAAATCATGAG

>TN1670

ACCCCATCTCTACAAACAAACAAACAAACAAAAATTAGCTGGATGTGGTGG

>TN1671

CAACGGGGGGAGGATTTAAATGAGTAAACATGCACACATGTACATGCACCT

>TN1672

GAAAAATAAAACTACTAAACAGATAAAATGTTAATTGTAATTTATGGAAAT

>TN1673

AACCCCTCCCACCCCTGTCAGCTTCAAGACATTTTTCCTTTCTGGACCAAA

>TN1674

TTCAGAGAATCAGGAACATTGTATTAATGAGGTAGAGGGAATCAAAAATTA

>TN1675

GAAGGTGGAGGTTGCAGTGAGCCAAAATTTCACCATTGCACTCCAGCCTGA

>TN1676

GCAACAAGAGCACAAGTCCATCTCAAAAATCAAAAAAGAAAAAAAACAAAA

>TN1677

GATACAATGTCCAAACAGTTTCATGAATTTAGATAAGTGAAAAAGCATAAA

>TN1678

ATCCTCAAACCTCAAACCTCTCTGGAAAAAAAAAAAAAGTCCTGTACCTTT

>TN1679

ATTTAATGAGAAGCCCTTAAAAAACAAAACTTGGAATAATTGAAAATGGCA

>TN1680

GTGTCCCATAAATCTGAGGACATTTAATCCCAATAATCCCTCTCCCAGTTC

>TN1681

CGTCTCTGCCTGGTACTCTCTGTCTAAGAGACGACATCACCAAGGGCCTAG

>TN1682

CTCATCCTTCGGAACCCAGCTCAAAAACATCACCTCCTCCAGGAAGTCCTT

>TN1683

CATGTTACAACATGGCAGAACCTTGAAGACATTGTGTTAAGTCAAATAAGC

>TN1684

TGTTAGCAACACTTGTTAAAAACACAAGAAAATATCTCATGAAAATGTAAC

>TN1685

ACATTTGTTTCTGGAGATTTTCCACAAGCCTGTTATCTGTGTTACCGTAAT

>TN1686

GAAAGGCTAAATAGAAAGACACTTTAAACAGAACATGTGCCATGAACGTGC

>TN1687

ATTCTAATTTACATATTTAATATTTAAGTAGAAAAATATTCTTTGAATGGA

>TN1688

AAACATACAGATCATTATTTTCTTGAAAAAGGGTACACACGTGGAGGAAAA

>TN1689

ATTTTGCCTAAAGATGTAAATATATAAAAATAGCCAAATCAGAGACAGGGA

>TN1690

CAGCACAGGGCACTGGAACCCCAGGAACAGTGCTCAGTTCCTTCCACGGGA

>TN1691

AACACAAAGCAGAAACAACTTCCTAAACAAATCTCAGCTACTTGAGACGTC

>TN1692

GAAAGGAAAATTGGTGATATTAGAGAATAACATGTTGGCATAATCGCAATT

>TN1693

CCTCAGGAAACTTACAATCATGGTGAAAGGCACATCTTCACAGGGTGGCAG

>TN1694

GAGAGAGAAGGAGTGCCAGCAGGGGAAATGCCAGATGCTTATAAAACCATC

>TN1695

GTCACAGGACAGTAGTAGAAAAGAAAATGGCAGGACAATCTCAATGATATG

>TN1696

CAGCTTTGTTCCGTTGCTGGTGAGGAACTGCGTTTCTTTGGAGGAGGAGAG

>TN1697

TATTCCACATCCACGGGTAGTACTCAATATTAAAGTGCTTTCAAGTGTACC

>TN1698

TAATCAAGATAATATTGATAAGGAGAATAAAGCAGAGAGAGTCGCAACACC

>TN1699

ATCTGTGCTATGTTGGATAACCCTGAAAAGATCATGTTAGTGAAAAAGCCA

>TN1700

CACTGTGCATCCATTAATAAACTGTAAACTCTTTCAGGGCCGACGTTTTAG

>TN1701

AAATATACAAGGCTTGGGTATTTACAAATGCTAGTAACTTTTGTGTGTGTT

>TN1702

CAATCAAGCAATGAAAAAAAAAAGGAATTCTACATCTCAAGAATAAAGACG

>TN1703

GTATGTGTATAGACATACACATCTGAATAATGTTTATTATGTGTACACACA

>TN1704

CATCCGTATAGTAAACACTGCATAGAAATATTAGTGCCAACCTTGTGGGGT

>TN1705

TGCAATGGGAAGACCACCTACAGAGAAAAGAAGACATCACTATTTATTAAA

>TN1706

AATAATAATGATATAATACTTACACAAAAACATCCAGTCCTTTTTTTTAGA

>TN1707

ACAAAAATTGCCATATGTAGTATGGAAGAATAGCAATACCGTTTTTAAAAA

>TN1708

GAGGATTCTCAGCTGATTTTAACAAAATGACTTTAAGCAGTTCCTGTTGCA

>TN1709

TCTTTGCTATTGTGAATAATGCCGCAATAAACATACGTGTGCACGTGTCTT

>TN1710

ATGCACGTTGCAAAGTTAGAAGCAAAACTTACAAGAGAAATGAAGCAAGAC

>TN1711

CTAGAGAAACGATAAAGGGTATACTAAGGCACTGATACTCTGCAGAAACAG

>TN1712

AAAGACGAGTAAGTTTTGTGTAATAAAGAAAAGTATCATTTTATATAAAAG

>TN1713

TATGAAGAAACTTTGGCGAAACTATAATTTTGTGTTTATAGATGCTCCTCC

>TN1714

GATGATTTCCTTTAGCTATTCTTTAAAATAGGCCTTCTAGAGACATATTCT

>TN1715

GAGGAATATCTGAGATAAAGGAGGAAATGGCAAGCAAACAAACTTATAAAC

>TN1716

ACCCAACAAGTCGTAACTCAAGAGAAAGAAATAAGAATTAAAATACCTAAG

>TN1717

AACCCCTAATTATACTAGAAAGAGAAAAGGAAAGACAGAAAATAGAAAGAA

>TN1718

TTAGGAGAAAATCATTAAGAGGGATAAAAAGCTATTAAGAGGCTGGGCGTG

>TN1719

TAATTGTAATTTTTTGTTTTTTAATAATTGTAGTCTTAATTTAGGAAATAT

>TN1720

TTGTCAAACCATAAAATAAATAATTAATATTTAGCAACAAAGAGGAAAATA

>TN1721

AGATCTAGGAGAAAAAAGGAAGAGAAATTCCAGAATAAATAATTGTAAAAA

>TN1722

TGAAAAAGTGACATACCATCTATTCAACAGAGATTGACAGGGTAATAAAAG

>TN1723

GAATATTTTACCAAAATTGAATAAAAAAGGAATAGAAAACCAAAATATCAC

>TN1724

TAACACTAGGAAAATTCAGATGCCAAAACCATACAAGACAGAAAAGGGAAT

>TN1725

TGAAGCAAAATGTTGCCTAGATTAAAAGATATGGAAACTGGGGATTCCCAG

>TN1726

AGATTCCCTCGGGTACCTACACCACAATGGACCTGAGTTTCAAGCACAAAA

>TN1727

CCCAGCACAGCATTCAAACTCTGCTAAGGGTCAGACTGCCTCCTCAAGTGG

>TN1728

ACAAACAGAAAGCAATAACATCAACAAAAAGGATTCGAAGGTCACCAACAG

>TN1729

GAGCATGTTCTAACCCAATGCAAGGAAGCTAAGAACCTTAATAAAAGGTTA

>TN1730

AGGAAAAAATGTTAAGGGCAGCCAGAAACAGAGGTTGGGTTACCCACAAAA

>TN1731

CCAGAATTTCATATCCAGCCAAACTAAACTTCATAAGTGAAGGAGAAATAA

>TN1732

ACCAGCCACTGCAAAAACAAACCAAAATGTAAAGACCATCAACACTATGAA

>TN1733

GATCAAATTCACACATAACAATATTAAACTTAAATGTAAACGGGCTAAATG

>TN1734

GAACTCAGGATTAAGATACTCACTCAAAACTGCACAACTAAATGGAAAGTG

>TN1735

ACAAATAAGTTATTTGAAACCAATGAAAACAAAGACAAAACATACCAGAAT

>TN1736

AAGAGAAAGAAATAAAGGGTATTCGAATAGGAAGTGAGGAAGTCAAATTAT

>TN1737

TAAAATACCTAGGAATACAACTTACAAGGGATATGAAGGACCTCTTCAAGG

>TN1738

AGAACTACACACCACTGCTCAAGGAAATAAGAGAGGACACAAACAAATGGG

>TN1739

AAAACATTCCATGCTCGTGGATAGGAAGAATCAATATCATGAAAGTGTCCA

>TN1740

AAAAAAGAGCCTGTATAGCCAAGACAATCCTAAGCAAAAATAACAAAGCTG

>TN1741

AACCTGAGAAAAACAAGCAATGGGGAAAGGATTCCCTATTTAACAAATGGT

>TN1742

CAGCCAAGAAACTATCATCAGAGTGAATAGGCAACCTACAGAATGGGAGAA

>TN1743

TTATTGTAGCACTATTTACAATAGCAAAGACTTGGAACCAACCCACATGCC

>TN1744

CATCAGTGCTAGGCTGGATAAAGAAAATGTGGCACATATACACCATGGAAT

>TN1745

CTCAAAAACACAAAAACAAAAACAAAAAAGAATGAGTTCATGTCCTTTGCA

>TN1746

CAGCTACTGCCATCCTTGCTAAGTGAAAGAAAACATTAAAATAAAAATTCA

>TN1747

AGAAAATATCTGGAAATACAGAAGCAACTGAAGTCAATAATTGTGTAATAC

>TN1748

GCCATTTGGCAGGGAAATAAAGGTGAATAAGGCTGTATCCTGTCCTCAAGA

>TN1749

ATATCTTAAAAGGAAAAGGACATCCAACTCATGAATATGTTTTCTGTCTGA

>TN1750

CAGGACTGCCCCTGGAACTTTCATTAATGTGTTATAAAGGATGTTGATGAA

>TN1751

TTGCTCTTGCTTTTCTAGTTCTTTTAATTGTGATGTTAGGGTGTCAATTTT

>TN1752

CTTCTGGAGTAAAATAATATATGAAAAGTATGAAAAACAGGAATCAAATAG

>TN1753

TTTGATCAGTTATAGCAATGGGAAAAACACACAAACACCCAAACAGTAATG

>TN1754

AAAAAAAAGAAATAGAAGAAAAGGGAAAGGGGATGTCAGAAAGAGCCTGAT

>TN1755

ATTTTATTTATTTGTCCCTACAGGGAAGAACAAACAAGTTTTTTCTACTGA

>TN1756

TCAACTAAAGAGCTTCTGTGCAGCAAAAGAAACTGTCAGCAGAGTAAATAG

>TN1757

GCAAGACTCCATCTCAAAAAAACAAAAAAAAAAAGAAAGAAACAAAGAAAA

>TN1758

AGAAAAAAAAAAAGATCTGAAGTTCAAAATTTCTTCAAATTACTTATTTAC

>TN1759

GGATCCTCAGTTTCCTCAGATGTGAAATAAGCTCTGTAATTAAAAGTTGCT

>TN1760

AAATGTCCTTCCCTAACACAAGGCTAATGATATCATTTTATGCCTAACTAT

>TN1761

AAGCTGCACTGAAATATTCATATAAAAAGAAAATTTCCATATGAAGCAGAA

>TN1762

TAAATAATTGGTTTCTCAAAAGTGAAAAAAAAAATGGGTCTCCCTTTGTCC

>TN1763

GCAACAAAAGCTGTACTTTTGGCATAAAACTAGTTCAAACTTAAACCAATC

>TN1764

CACCAGAGGTGCGAAGGCGGGAGGGAAATTCTCTGTACTCACAGGAAGCCT

>TN1765

ACTGGCTGCCTGCAAGATTCACAGCAAATGCAGACATGCACCGTGTAAGAC

>TN1766

ACAGAAGCACTGTGCTCAGAAAGCCAAGTCCTAAAGCAAGGCACATATGGG

>TN1767

AAGTACGAGTAGACAACTTACTTGGAATTTTGTTGAGAAAGGGCTGGAAAT

>TN1768

AGTAGCAAGGACTTCATCCTTCAATAATCAAACTATGATATCATAATAAAT

>TN1769

TGATTACAGTTAGAAAATGTGATGGAAAGCCTTTATTTTCTTCTGTAAGTA

>TN1770

GTGCAGGACCACATCCCATTTCTATAAAACTTCCGAATCTTTCTGAAGATG

>TN1771

TGGCTCTCTCTGGGGCCTCTTTTAAAAGGGCACAAACCCCATCCTGAGGGC

>TN1772

TGAGCAAAATCTTAGTGAAAATAGTAAGTCATTATACATATATTATTTCAA

>TN1773

TTTGGTTTCAGAAAAAAATGGCAACAAAATGGATCAGTATATTTTAAAAAT

>TN1774

ATTGCTTGTTTTTCTCAGGTTTGTCAAAGATCAGATGGTTGTAGATTTGTG

>TN1775

GTGGTAGACTATTAATTACTGCCTCAATTTCAAAACTTGTTATTGGTCTAT

>TN1776

CTTGTAAGACAGGCCTGCTGGTGACAAAACCCCTCAGCATTTTCTGGTCTG

>TN1777

GTAAACATAAAAGGAATCCCCATGGAAGCATCCACCGAAAAGTTGGGGCTG

>TN1778

GAATGGCAATGATTCAAATGTCAGAAACATTGGTATTCTAATAGCGTTTCT

>TN1779

AAGATATGTGAAGAAAATCATCTGGAATCTTTAAAGGTGCACCTTGCTCTC

>TN1780

GATACATCATCTGACAATGTAAAGAAAGGTGCTCACCTCATTTTTAAAGTC

>TN1781

TACGCTGTGAGTAACAGGTGAGCACAATGACGGTGATACTCAGCATTAGCT

>TN1782

GTAAAGACCCCAGTGATATTCGGGGAATAAACACGTCCACCCCATAGCTTT

>TN1783

AGAGCTGTCTCTCTCCCACCTGTAAAAAGTCCACAGATGTGGATTTTTTTC

>TN1784

GAAAGGAAGAAAGAAAAATGAAAGAAAGAAAGAAAAAGAAAAAGAGGCCGG

>TN1785

AACAGAGCGAGACTCCATCTCAAAAAAAAAAAAAAAGAAAGAGAAAGAAAA

>TN1786

CATAGATTGACATTGGATTATTAATAACTGAGGTAAGTAAGAAGGGGTGTA

>TN1787

TACTTTCCTCCTGCATTTCTGTGTAAAAACTGATAAGGAAATCATCTGACC

>TN1788

CTCAGCAGCAGCCCCAGAAGGTGACAATATCAGCCTATCCCAGGCACGACG

>TN1789

AGTCTGTTCTCACACTGCTGTAAGAAATACCTGAAATAGGGTAATTCATTT

>TN1790

ATGTGAGCAATGGCTGATTAACAATAAAATTTTAGAAAGCGGGGGTCATTC

>TN1791

TATCTAAAGACCCGGAATCAATATAAAAGAATGTCTGGGTTAAGATAAGGG

>TN1792

TGTAATGTCTTTTCTCCAAACTTTTAATCTGTACATGGGCAATTCTAATTT

>TN1793

TCTATTTGGTCCCCTATGAAAACCCAAAGTAAAAAATTAATAAAGCAATTT

>TN1794

AAACAAAGTCAATCACACTTAAATTAAGAATCAAAGGAAAAGATCTCAATT

>TN1795

TACTCCAAAAATTATTATGCGTAGGAAAATATTTACATTATTAGGATTAAG

>TN1796

AAAGCATCAAATATTTGAAAGATTTAATAGAAGACAAAGGACGTGCCTTGT

>TN1797

CATAAAGACACATCGCACAGAAGTCAACACAGGACTTTTCTGAGAGCCGCT

>TN1798

TTCTCTTTCATATTCAGGAAAACGTAATGGACATTACATTCTTTAGAAAAA

>TN1799

AAGCAAAATGAAACAACAAAAACAAAAATGACAAAGAAGAGCAAAAAAAGA

>TN1800

GTGTGCAATGATAATGAAAACATTAAAAAGTAAAATTAAATGTTATATTTT

>TN1801

AAAAAATAAAGAAAAAGAAAAAGAAAATATGTTAACCATATTCTTAAATTT

>TN1802

TTTAAAAAATGAATTATAGAAATAAAAGGAGCATGTAGTGATTTTTGTTTC

>TN1803

ACACAGACAATCTGCAAAAACTAATAATGAAAGCTCCGCCCAGGAAAATTT

>TN1804

ATGGTGAAACCCCGTCTCTACTAAAAATACAAAAACTAGCCAGGCATGGTG

>TN1805

CATTTTTACATAGCCTGCAATCTTAAAAGTATAATGGCAATACAAATTAAT

>TN1806

CTTCTCAGGTCTAGCTGTTTAAGGAAAATAAACATTGCATCTCTTGTGATA

>TN1807

ATGAATGAAAAGTTGTACTAACGAGAAAATCTGTGTCTTTAAAACCTCAGT

>TN1808

CCACTGGGAGCACAAAGTCCACGGGAAGGATTTTCTGTTACAGAACAGCGT

>TN1809

TCCTGGACTGCAAAAATAAATAAATAAATAAATAAATAAATAAATAAACAA

>TN1810

CTGCTCAAAATTATATTGGTGACTTAAAAGAAGTACCGGATGATGAGAAAT

>TN1811

AAATTATGATTTAGGATACCAGTTCAACTTCATTGTTGATTCAATAAATGT

>TN1812

AATTAGTCAGAGCGTTGTGTGAGATAAAGCCTGTGAATGCATTTTGTAAAC

>TN1813

TGACTAATCCACTGTTCCTTATTAGAAAGACGGACCTTGCTCCTAAGGGTG

>TN1814

GTACAATTGAACAGGATAAATGCTTAACTATACTTTCAAATGTTGTGAACG

>TN1815

TAAACATCAAAAACAGAATGTTTGAAAATAACATTAAGAAATATTAGATAT

>TN1816

AATTCTCAAAAGACTAGAAAGTGTGAAACAAGGCCCAGGAAAGTATCTGTA

>TN1817

GGAGCAGAGCAATTGCTGTGATTGGAAATCTCAACCACAAGATATACATTA

>TN1818

TAGTTGATGGATACATAAATGTAGCAACTGTAACTAAAGCTAGGAAAATAA

>TN1819

TGTACTTCCAGAGATAGGATTAAAAAAAATTTGGAACTGGTTTTATTGAAT

>TN1820

CAGTGGCACAATCTCAGCTCACTGCAAGCTCTGCCTCCCGGGTTCATGCCA

>TN1821

ATAAAAATAGGTTTTCTCCACTTGTAAACCTCAAAGAGTCTATTCCCCTAA

>TN1822

GCTACTCAGAAGGCTGAGGCAGGAGAAGTGCTTGAACCCAAGAGGCGGAGG

>TN1823

GCTGACAAAAGAATATTAAGAAAGAAATCTTTATTAAAACATAAAACTTTA

>TN1824

TGTCGACGTGCCAAACACCATGTTAAACACAACAAATGCTTTATCTTATTT

>TN1825

ACATTGGTGCAAAGATTCCATAAAGAAGACGGCTGTCATCCACGCTTTCTG

>TN1826

ATAAAAGATACAGACAGAGTTCCCAAACTCTCCAAAAGTTGTCAGTGACTG

>TN1827

CAAAGGGAGGTGATAGAGAAAAAAAAATGTGCGGAATACATAGTACTGTCT

>TN1828

AAATGCTGAAAAATTTTCATCATATAAGAGAGGACCCGGGGTATAGGGTAA

>TN1829

CTTTAAAAAGTATGACTTTGAAGAGAATGAAAGGAGTTTTGACTCTGCAGA

>TN1830

CGAGGGCATCCTGTCTCCATGGCTCAACGGAGGCGCCTCCAGCTAAAAGGA

>TN1831

AAGGAGCACAGTAAGTTTCGGGCACAACCCCTTTGTGCCCCATTATAATCT

>TN1832

GAGCCACAACCATATCACCAAATGCAAACAGAGCGGGCATGGCTGTGGTAA

>TN1833

AAGAAAGGAAGAAAAAGGAACTTCCAATTTGTTAATAGGTTTATTTCAGTA

>TN1834

TCTGTATCTTCAGAATATGCAAAATAAATAAATAAATAAATAAATAAATAA

>TN1835

CACCATTTGAAAAATACTGATTTACAATACCTTTTATTTTTAATTTTTTTT

>TN1836

GGAGATGCCTGGTTAATGCTGAATGAATGTATCTGGCAATGCAATTACCTT

>TN1837

AGTAAGATAAGGCATTAATTTCCAAAATATATATGGAGCTCCTGCAACTCA

>TN1838

AACCTTGAGGTTATCGTATTAAGTGAAATAAGCCAGTCACAGACAAAGGCT

>TN1839

TCATGCTAAGTGTTCTTATCATGATAAAATAAAAAAGAAAGAAAGAAAAAG

>TN1840

CACATTTCAAGGAGTTATACAAAATAAAAAAGAGAAAACTTCTGGAGTCAT

>TN1841

TCTTGAAGTTGGACAAAGAATAGGCAATGGCACCACTATGTACAAAAGATG

>TN1842

TGACTGTATACAGAGAAAATACAAGAAAATCTACCTTTCAGGACCTCCAGG

>TN1843

AACATTGGAATTAGGAATCGGAATTAAAAATAAGGAATTAAGAGTTGGAAC

>TN1844

GACTGCTACAGGCAAGTCACTATGCAAGTTATTTCGAGACAAAAATATAGA

>TN1845

CCTGTCTCAACCCCTGCTGGAAAAGAAAATAATGGAAACACGATTTTTTTC

>TN1846

AATGGAAATGAACCACCAAAATAGTAATAATCATAATGACCACAGTAACAG

>TN1847

GGTTTAAAAAAACAGTTCAATATTTAAAAAAAAAAACAATTCAATGGACTG

>TN1848

AGCTACCCCATAATGGTTGCACCTGAAGAAACACGTCTGTTTGCCAGAAAC

>TN1849

AGGGAAGTTAAGTTCTCAACAAACAAATCAATATGATTAAGAAAACTAACA

>TN1850

AAAGAAAGAGAGAGAAAAAGAAAGAAAGAGAAAGAGAGAGAGAAAGAGAGA

>TN1851

TCACAAGCACACAGCTGTAAGCACCAAAGATCAGATTAAAACCCAGACCTT

>TN1852

TTTGCTTTTCTGTTTTTGATAATTTAAAGGCTCATTTAATTCTAATTTTAA

>TN1853

TCTCTACACAGGCAATGGGTAGATCAAGAAATATTAATGAGAGAAAATCCA

>TN1854

CACTTCCAGTTTCCAGAATTGTGAGAAGTCGGTGTCTGTCATTAGGCCACC

>TN1855

TATTTATGCTTTTGTATTTAAAAAGAAAGGAGCCTCTTTGTTCTTTAATGT

>TN1856

GCAGTAGAACAGGTGTCCTAAAAGAAATGCTAACGTGCATTGACTAAAAGT

>TN1857

TTGATAATGCCCAACACACAGCAGAAATAAAATAAATGTATGTTAAATAAA

>TN1858

CCACCTGGACATTCCGATAACACTCAAGTAATCTGAATAAAAAGTGAACAA

>TN1859

TGGTGCAATCAGAGAGAAGAAAAAAAAAAGAAAGAAAGAAAGATGCGTGCT

>TN1860

GGCAGGAAACCTGGAAGTGTTGTAGAATGCTTCTTAATGCCTCCCATAGGA

>TN1861

TAAGGTGGTATAAATATAAAGTAAAAACTTTCCCACTTCAATAAATAGCTA

>TN1862

TAAAAAAACAAGATATCCACATGCAAAAAATGTGAATCTAGAGACAGACGT

>TN1863

TTATTTCTACCAAATGGTAAGGGCGAAAGTTAGATTTAGATCATCCACATT

>TN1864

AAGAGCTTTCAAGTTAACACAGGAGAAACTAACGATCTTTTCCTATCTTTA

>TN1865

GACTAGCACTCAACAGATATTTTTAAATTAATGAATAGATAAAAGTATCAA

>TN1866

AAAAATTCCCAACATCGGAATGATGAAAAGAGTCTACAGTGTGAAGAAAAA

>TN1867

AAGAAATTTTTATAAAACATCATAGAAATAACAAAGACAGTTTCCTGGTAT

>TN1868

CTTCCAGCGAGGCAGCTTTAGTTCCAACAATAATCTACAGCTTAAAAAGCA

>TN1869

GTGCATTAAGAAAAATGAAATTATTAATAAGAAGTTTTAGGAATTAATAAA

>TN1870

TGGAACGTTGTTTCACAACATTTAGAATTGTTCTTTAAGAAACATGCAAGA

>TN1871

GAGAATTTGTTCAGACTAATTATTAAATTTTAATTAGGAGTTCAGTAAAAT

>TN1872

CATTTAAATTGGTGGTTATCAAAACAAAGACTCCATTCACAATAAACCCAG

>TN1873

ACCAAGAATATTCGGTACTAACTTGAAAATGAAGAATAAGGATAGAGATTG

>TN1874

ATGAAAGAAATTAATATATGCAGAAAACAATAAAACAATATAAGAACCGAC

>TN1875

TTTTATTTTACACAGTACACAAAACAACTATAAGAAGGTGATTAAAAATAC

>TN1876

ATTTTAGATTCCAAATTCATAAAATAATGCTGTTGATAGAGTTGCCCTGGA

>TN1877

CTAGCACTACTAATAATAATATTGTAAAGAAGCCAAACACTTATAGAGTGT

>TN1878

AGATAGAAAAGGTTTTCATAGAAATAAATTGCTTAAAACTTTCCAAACAAT

>TN1879

TGGTAGAGTCTGTGAAACTAGATTTAATGAAACTTATTGTATCCTTTTCAT

>TN1880

CAAAAAGAAGAAGTCAAAAGTTTGAAAATTTACGCTGAAACTATTTGCCGT

>TN1881

ATAGAAACCAAAATAGTTCAATTTTAACATCCTAGTATACATAGTAAAAGT

>TN1882

GGCTGATACCTTCACAGATGTTCTCAAAGCCCCCTCCCACTGCTGACCCCA

>TN1883

TTGCCATTGTAATTGCAAAAACCGTAATAATTTTTGCACCAATCTACTACT

>TN1884

TAAGACACCAACATATATCACTGTGAATTCAAACAAGAACACGCACTTTCT

>TN1885

AACCAAGCCCATCAGAGACATTTTGAAAATTGTCCCATAACTGCCTTGAAT

>TN1886

TCAAATTTAAAGATGGGTTATGTAAAAGTCAAAATCTTCCAGATTGCTGTA

>TN1887

CAATAATTGTTGCAGGTTTAATAATAATTAAGTATAAACATTTTTCTAATG

>TN1888

CCTACATTCTTTAACATACTCTCAAAAGCAAAAACACACACTGATGTCTAC

>TN1889

CCCTTAACTTAAGATAAAAGTTAACAAAAACAGAGAAAGCTGCTGCTAGAT

>TN1890

TAAAACCGATGGTAATACCCAATGTAACAGATGAAGAAAAGAAGAGAGACT

>TN1891

GTACTTTGTTATGGAAGCCCCAGGAAATTAACACACTAGGCTTAGACCAAG

>TN1892

ATGCAAAATTTAGAAATAATTTTAAAAACTTGAACAGAAGGCTAAAAAATA

>TN1893

TTAAGCTAAGTAGCTTCTACACCGCAAAAGAAACAATCAACAGAATAAAGA

>TN1894

TATAGAAATGGCCAACAAACATGAAAAAATGCTGAATATCACTAATCAGGT

>TN1895

TATCAGTTTATAAAACGATTCAAAGAAGCATTGTCTTTGCAAAATACAGAT

>TN1896

AGAGCAGCACCCAGAGAAAAAATAGAACATATGTAACGTTACATCTCAGCA

>TN1897

AACAGAGCAAGATGCCCTTTCTAAAAACTAAATGAATAAATAAACAAGCAA

>TN1898

GAAAAATGAAGTTAGTAAATAGATTAATAGAAAACTAAGGAAGAAAAAAAC

>TN1899

AACAGGGAATAAGACAAGGAAGAACAATATTGTATCTACAAAATTTACTAC

>TN1900

GAAAGGGAAGCTTCATTAAACACCCAATGAGATTCACCAGGACTCCTGAAG

>TN1901

ATGTGGCACCTTCTAGCATATAGAGAAACCCTCTGGATGGCAACTGATAGG

>TN1902

ACTTAGAATAATATCCCCAATATTTAAGCATGTTGTTACAGATCATAAATT

>TN1903

TACTAGGATTCATAGCAGTTTATAAAATGCAAAAGAAAAAATCAGTGAAAT

>TN1904

AATCTTTAAAAAAAAAAAAAAAAAAAAAAACAGAAAAACCAAAACAGTTAT

>TN1905

GCGGCGCAGCCGCAGTCTTAATGGGAAGATTCTAGAAGTAAGAGAACCATG

>TN1906

GACTTAAAAACTGTATGCAAATTCAAATGTGTTTCTCAACCCGTGAACAAC

>TN1907

TAGTCCAACAGCCCTGAGGAAAAAAAAAAAAAAAAAAAAAACTATGTCTTC

>TN1908

ATTGCCGTCCGCTCCATATTTGTAGAATCTCATGACGAGGATGGTAAAATT

>TN1909

AAGAGCGAAACTCAGTCTAAAAAAAAAAAAAAAAAAAGAGAGAGAGAGAAC

>TN1910

AGAGGGACACAGACATTCAAACCCTAACCCTGTTTTTTGTTGATAGTTTGT

>TN1911

GCCTAAAAGATCTAAGAGATCTGAGAAACCCCACAGCTAACTCGTCACCAC

>TN1912

ATTGTTTTGAACTTGAGTAAATTACAATCTCTGTAAATTTTGTCCTTTTCC

>TN1913

CATTCTGATGATAATTAATATAGAAAAAGAAAAAGGGAAACAATTGTGAAG

>TN1914

ATGATAAAAAAAATTCTAAGACTTTAAAGTCAGGATCAGTTTTCTACCAGA

>TN1915

AAAAAAAAAAAAATCCATACTCTTCAAGATCGATGTCCGTGGAGATTTATG

>TN1916

CAGATAGGGTACAGAAGTCCCCTGTAAGGCTTTCTCTTTAAGGAAAAGCAG

>TN1917

AGGAACCAATCTGTGAGCCCTACGTAAATCAGACACCACCTTCACAAACTG

>TN1918

CTATAAATAAATAAATAAATAAATAAATAAATAAATAAATAAATAGAAAGG

>TN1919

AACATTAAGTATGGGAGGAAAATTAAAGACAATATGAAACTAAGAGGGTGC

>TN1920

AATGCTTGTCAGAGTAGAATAGGGGAATTCTAGTTGAAGGAAACAGGTATT

>TN1921

AAACTGTTATTGTTTAAGAGAAGGAAACGTTGAAAGAAAGGAGACATTTGA

>TN1922

ATTATTAAAATGAAACAAGAATAGGAAATTAATGAACGCAAAGTTGCTGAA

>TN1923

TAGCATCACAAGACAGCAGTTCCTGAAAGAAATAAAAATATTTTGCCCCAA

>TN1924

GAAAGAGCGAGAATTAAATATAGAGAAGAGGAAGGTAACTAATGTTAGCTG

>TN1925

AACAAGTCATTTCAACTGTAATTTTAAGATAATAGCTTTTGAAATATGGTC

>TN1926

AAGGCATGAAGTAAGCAAGCAGGAGAATTCTTTCACAGCTTCCACTTAAGG

>TN1927

TAACGCTCAAGTTCTGCAACTAGACAATGGAGAGATTCTTAACTCCACAAC

>TN1928

TATCAAGTCTATGTTTCTATCCATTAACCGACCTCGTCATTCCTCCTCCCC

>TN1929

GACAAATCATTTTTTCTGACCACAGAATTTAAAGCAAGTGACTAAGTTCGA

>TN1930

GCATTTGTTCTGAAAAATATTAAAGAATATGAGAAAAAACACCCTGTAATA

>TN1931

GAAAAGATCACCATTAGAATCCTCTAATGTAATTATCTATGAGGAAAGAAG

>TN1932

ACTTAATGGTGTTGCAATATGGTTTAATTGAGATAACATATTAGAGTAATC

>TN1933

TTCTTTCTATCAGACATTATTTCAGAAGTATTAGTATAAGTAGGGTAGAAA

>TN1934

TGTTGGAATGACCTGAAGTGCAACAAAACTCCATACAGAAAAACCAGATCT

>TN1935

TGCATCTGGTGAGATAAACCCCAAGAAATCCTGGCTTGAAGACATCAGGCT

>TN1936

AATTGCACAACTAAAACTTGCCGAAAAATACAGCTGTTTTGTAATAAGTTG

>TN1937

CAAGGAGACCCGGTTTGGCGGAAGAAACTAAAGGGAAGCAACATAGTGAGG

>TN1938

GACGACAATGCTGCAGATCAGGAGGAACCTGGGAGCGTCTGGGGAAATCTT

>TN1939

AAACATGAATTCGTGAAATAAGTTGAAGACAAACATTAAATAAAATTTCAT

>TN1940

AATATATAATAAACACATGTGGTTTAACAAATGTCTGCAAAATGATATAGC

>TN1941

AAAATCTAATAAAAATACAAATATAAAAATAAAAAACAACCTTAGCATCTG

>TN1942

AAAAGGTATTGAGCACAGTAATGCCAAAACAGAAAATATAATTAGAAATGA

>TN1943

CCCAAACGACTGCAGTCTTCCAGCCAAGGGAGGCTTGTCTCCCTCAGGAGA

>TN1944

CGGGCCACTCCAACCAAGCAACCGCAAGGAAAGCTACATGGTTACGAGGCC

>TN1945

TCCTGAGATTTTTAATCTGACTTATAAATTGTCAAGTCTCAACAAGTATCA

>TN1946

GTTCACCTGTCTGTCACACACATTGAAAAGTTACGCGGACTTCGTAACACA

>TN1947

TTACCTGCAAGAAAACAAAAATTATAATGTTCTCCTCATCGTCTGTGAAGT

>TN1948

CCAACCAGCCAACCAAACAATCTAAAACAGAAGAAAAGTTGGAGGATGGCA

>TN1949

AAATAGGGAATAAGTGTACGATTCTAATTAAAAAGGTAAATTCTGCCATAT

>TN1950

AAAGAAAGTCATTTAAGGTTTTTATAACTTGGAATCATTGAATTAAATAGT

>TN1951

GTTTCCGTTATGACAAGACACAGTGAAAATTACTACAGAATAAGGATGCTT

>TN1952

GATTGTATTTGCTGATTTTTCAAAGAATGCTAAAACATATATATATGTATA

>TN1953

AAGAAGCAGGGAAAACAGCACACCCAAATCAGCTTTCATATGCTAAGGCTT

>TN1954

TTTGTAATGTACATTAATAATGGGAAAAATATCTTTGGTTATCTAAACAGA

>TN1955

TCAAGCGAACTTGAAATGACTCATGAATAGCAGCAGTTATTCCCCTTAGAA

>TN1956

ACAACAACTACAAAAAACAGTTAAGAAAATTATACAAGAAGCATTAAAAAG

>TN1957

AATAGTAAGACCTATTCCCAGAGATAATAATTGCCAAGAAAAGAGTGAAGA

>TN1958

TGCAGACAGGGCAGGTTGGCAACACAAGTCATCCAGCAGGTATGGCCACGT

>TN1959

ACTTTTAAAGCTGTGTTATCAGCAAAAGGCCAATTAACTAATTTTGAATAT

>TN1960

AACTTCAAAGACCTTTACAGGTAGCAACACAGTGACAAGAGGACCAGATCC

>TN1961

AATGAAAAAAATCAGCCTTTTAAATAATTAAAATTAGTTTTGGCTGGGTGT

>TN1962

TTAAAATAAACATCCATGAAGCCAGAAACCAGATCACAAAACGCAACATTA

>TN1963

TAATAATTATATTAATTGGGCCCATAAAGTTGTCATAAATGCTATTTTTTT

>TN1964

CAGTTGTTAGGTGGCTTTAAAAGAAAACAGGCACAGAAATAACCTCCAAGG

>TN1965

AAAAAAAAAAAAAAAAAAAGAAAGAAAAGAAGAAAGAAAAGTCAGAGAACC

>TN1966

TCTAAAATGCATTGAAATAGGATTGAAACTCGAGTATTTAATAGAAATTTC

>TN1967

TGCCCCACATATTCCTTTACAGTGGAAAGCTGTAGTTTTTGTTTTGCTTTA

>TN1968

CCTAAAAGCTGGTTGTTTAATGATGAAATTTGGCCAACGATTAAGCTTAAT

>TN1969

TTTCTCCACTCTGGAATAAGATTTTAATAGTGCACTTTTCTCTATAAGATA

>TN1970

ACATAGATTAGTTTATAAACACTTGAAACACTGCTTTGAAAGATAAAATAG

>TN1971

GAGCAGGGCTGAGTGTAAAGGCTGAAAAGTGTTCCTTCCATGCCTCCAGCC

>TN1972

CTCCTACGTCTTAGAGGAAGTACAGAAGATACAATGGTAAAAGTGATTGCT

>TN1973

GGTTCAAAAGAAAACATAAATAAATAAATAAATATAAATCTGTTTCTGAAA

>TN1974

CTTAATGAATTAACAATGACAGACAAACGGGCAAAATGCTTTTTCTTACTC

>TN1975

ACCTGGAGCCCTGTGGAGAACAAGGAAGGGAAAGTTTTTGATGCCAACTTG

>TN1976

CTGTATCTCTATCTCATCTATTTGTAAATCCATATATCTCCTAATGTGCAT

>TN1977

TGAATCCGCTATTCAATAAAAAGCCAAAAACATATGGTAATATATTTGAAT

>TN1978

ACAAGGAATGCTTGCCTGATACTCTAACAACATATTCTAACAAGTTAAATC

>TN1979

AGGCGGTAGTCCTTTGTAAATAAACAAGTGCTGCAGGAGACAAAAGTTGAG

>TN1980

TGGAAACAAAAAAATATAAAGGCCAAAGACTGCTTTGTCGAAGCTCAATAT

>TN1981

TTGAAAAGAATGAAAAGCTGTGAAAAACACTCTGAATTACACATGGCTACT

>TN1982

TTTTTAAATTTACAATAGCCACAATAAGTTCATAAATTTGGGAGATAATCA

>TN1983

TTCATAGAATTTCTCTGAGTCATAGAAGCTGAATGTGTGTATATAACTGAA

>TN1984

AGGAAAATAAAAGCAGATAATGATTAACGAAGAGGGCTTGGTATTGAGTTT

>TN1985

TAGCACCATAGTAAGTAATCCTGAGAAAATATTCTCAAGTTAACTCTCAAG

>TN1986

TAGCTTGATCGAAGTACCAGAAAGTAAACATTCACATAACCACCCCAAAGA

>TN1987

CACAGTTAACAATAAGCTTTAGTGAAAATCGACAAAGTTAATGTCCAACGT

>TN1988

ATTCCAATCTCTCCATTTTCCCAAGAACAGCATATGAGAGTTCCAGTTACC

>TN1989

ATTTTCACTTATATTAGCCAGCCATAATGATATATATATCATAAATATATA

>TN1990

GCGTTGATCCTTTGTGTAGCCAAAGAAGCTCTAGAAGGGCTACACTTCTTT

>TN1991

ACAAAAGAAATGGAAAATATTTTAAAATCCAATCTACGGAGATAAAAATGA

>TN1992

GCAGACTCCCCAGGGAAATGCAGCTAATTTATTTATTCTTTTTATAATTTT

>TN1993

CACAGTTGATAATATTCAAACTGAGAAAGGAAAAACCACCCTGAAACTTTA

>TN1994

ATTACCATCAATATTGATATTAAATAAAAATCTATCGTGGTAGAAAGTTGA

>TN1995

TTAAATTAGTCTCCTTCTATGTGAGAATCGGCAGTGGTTCATCTTGATTGG

>TN1996

CTGTATTAGTCAAGGTTCTCCAGAGAAACTGAACCAATGGGAGAGAGAAAG

>TN1997

GAATGCAAAATATGTTGCTACTGGAAAAACTATGGGAGCAGAAGAAAATGG

>TN1998

TAAGAAACACAGGTAAGAAATATTTAATGTTGCTCATGGAACAATAAAAGA

>TN1999

TTGAGATATAACTTTTAATTAATAGAAAAACATACTCCTTCTGCTACATGA

>TN2000

CTTCAGCCAAATTTGAAAACAATTTAAGGAGCATTAGGTAGAATATAAAAA

>TN2001

ACAATCTTTAAGTGAATCTTACAATAAAGCTCAACATTTTAATAGAAATAC

>TN2002

CAAAATATGCACCAAACAACAAAGTAAAAATCACAATGATTGGCATGCAAT

>TN2003

AAGACGGACATAAAAATTATACAACAAAAAACCCAAGTCACATTTCAGGAG

>TN2004

GTAAAAACTAAAAAGTCTGATATGAAAATATGGTGGTTAGCATTTGTGAAA

>TN2005

TGAGTCAGTCTGGAGAGCTGACTGAAATACAGGGATAGAGGAGGCAGCAGC

>TN2006

CTGAGGAGATTTCAAAAGATGCATGAAAAACGTAATGTAAAGATAAAAGTA

>TN2007

CCATTCTTTGGCCCTCCAGAAAATCAACAATCAAAATGCCTTGGGCATCCC

>TN2008

AAATAATTTTGAAATAGAAAGGAGAAAACAGAAAAGTGAACAACTGCAAAT

>TN2009

AGGGGAAAAGAGGAAGAATAATAAGAAGAAAAAGGAGGATAAAGAGAAAGA

>TN2010

CAAGAGTAGGATGTTTTGAGACAAGAAAACAGCAACTCGAAAGGCAAAGAA

>TN2011

GTGACTTATTATTATTATTTGAGACAATGTCAACTGGCATATTCAAAGTAA

>TN2012

CTAACACTAATAATACAATACATATAAAAATATATGTTTACAAGACAGCTA

>TN2013

GATTCTCAGTCAAAATGTACAAGTGAAGACTCCGGATGTTTGAATTTGTTC

>TN2014

AAAGAAGTTAAGAGCCCCAGAATAAAATCAAGTAAATGTAATATTTTTTCA

>TN2015

GAGATAGACTATTTCTTTACCAGAGAATATAATATTCAACAGAAATACTAG

>TN2016

TCAAACTCTGTCAGCACATCATGAGAAAATTATTTTAGAATGCATATGCAG

>TN2017

AGGAATTAGAGAATAAGGTTGATGTAATGTGGAAACTAACAAAATTCAGGA

>TN2018

GTGTAGGACAAGGTTCAAGTATCTTAAAGTGATGCCTGTATTGTCCAAATA

>TN2019

ACTATTAAACATTCACTTAACACACAAGAAGGCTGTGAGGGGGGGACGAAA

>TN2020

TATTACATTTATATTATAAACTGGAAATCATTATAAACATTTCACTACTAT

>TN2021

TCTCTCTGTTCAAGTCAGCTGCTCTAAAAACTACCAACCAGTCACTGGAAA

>TN2022

GGAGGAAGTGGCAGGACATGCATTTAAATTCCCTTCTTAGTGGCCTACCTC

>TN2023

TCAGAAAAATTTTAATTCTACCTATAAGCTGGAAGCCCCCACTTTGAGTTG

>TN2024

GGGCACATGTTCTCAGGACCTCCTGAAGGCTGTGTCATGGGCCATGGACAC

>TN2025

GTCTGTAATAACAGTGTAAACAAGTAAATCACATCATATCTCCCCTATGCT

>TN2026

GCTCTCAACAAACAAGCTCAAAGAAAATCTCCAGTCAGAGGAGAAGAAAGA

>TN2027

GACAAAATTTTTTGAGATAAATTATAAAGAAAACACAATATTTCCAAGCCA

>TN2028

GAAACTAGAATAACAAAAGCAAAACAAACTCAAAATAAGTAGAAGAACTAA

>TN2029

TTAAGATCAGAGTAGGAATAAATAAAATAAGGGCTAAAAACTATTAAAGAT

>TN2030

AGACCAACAATTAACAACAAAATGGAATCCGTAATAGAAAAGTCTTCCATT

>TN2031

TCAATAGACAGATAATATTCTTCACAAAAAGAGTAAAAAGCAATTCTAAAA

>TN2032

CACTCCAGTCAGAATGGCTACTAACAAAAAAATCAAAAGATAACAAGTGTT

>TN2033

ATGGAACATATTATGAAGGTTTCTAAAAAATTAAAAACAGATCTAGCAATG

>TN2034

TGACATTTTGAATAAATGCTTATCCAACATTCTTTCACATTTTAGTATCTG

>TN2035

AGACTGGGCTGGCTGGCTTCGGTGGAAGCGCAGATTCTGACTGTGGAGAGG

>TN2036

TTGAAGAGTTTCTGAAAACTAAAGCAAAACTCATCAGAATAGAAGACACCA

>TN2037

GACAGAGTGAGACTCCATCTCAAAAAAAAAAAATGAAAATAAAAAATAAAA

>TN2038

TGTAGTTCTAGCCCCTAATCCCTCAAAATGTGACCTTATTTGGAAACAGGG

>TN2039

CCAATATGACTGGTGTCCTTATTAAAAGGAGAGAGACTTAGACACAGAGAC

>TN2040

CTTAAACTCGTTTACATTTGTATACAACCTGCTTCATTGTAAACATATAGC

>TN2041

AAAACCTCAGTAATACATTTAAATGAACCCAGGGCCCAGTAGGGAGGCAGT

>TN2042

GGAAAGTATGAGAACTAGCATGCAAAAGCCATATGCAAAACAGTTACAATC

>TN2043

ACAAAATGTTTAAAAAAAAATCAGAAAGAATCTTAGGTGGAAGACAATGGA

>TN2044

TAACTAACACACCACATAAAACAGGAAGGAGAGCAGTAAGGAGTTTTAGGA

>TN2045

TTGCTGTTAACTTAAAAGTGCTCTAAAAAAAATAAAGTATGTTAAAAAATG

>TN2046

AGACTATCAAAAGTAAGAATAGCAGAATTCTGAGAAAGATGAGAAAATTGA

>TN2047

GCAAACAATGCAGTTTGCATTACAGAATTCCAAAAGCTACACAGAAAGTTA

>TN2048

AAATATGCAAAAATATAAACATGCAAAAAATAAAAATATGCAAAATATGCA

>TN2049

TAAATTACAAAGCAATAACAGGAAGAACTGTGGTACATTCCCACATATTTA

>TN2050

GAAACTGATCAGCACATTTGTAAATAATCCATAGATCAAAGAAAAAATCAA

>TN2051

TGTGTGGGATAACCCTAAGGCAGCGAAGTAAGAGCTGCAGTCCTAAGTGCT

>TN2052

TACTCATAATCACCGCGAAAAGCATAACAGAAAACAATAGAAAACATCAAT

>TN2053

TTAGCCTGATCTGAAAATAGAGAAGAAACAAAATGTGAGAAAGGTGACATC

>TN2054

TTATACTGAAGGTTTGATCCAGTGCAATAATAAAAGAATATAAAACAAAAG

>TN2055

ACACCTCAATTCTTTCCATAGTGACAATGATAAGCAAAAATTTTGGTATAC

>TN2056

AAAAAACAGAGAAAGTACCTTAGCCAAAACTCTTTTGGGAAATAACAAATT

>TN2057

TATGAAGACCAAGAGGAACATTTTGAACACCTCTAATAGTCTTTGCCACTC

>TN2058

AAAAAAAAAAAAAAAAAAAAAAAAAAAAAAAAAAAAAAGAACTAGGTTCAT

>TN2059

CAGAGCCCAGTTAGCTAAAATGCTGAATAACTGGAAAAAAACACAGCGTCT

>TN2060

GAATTTCAGCATATGCTTCCTACAAAATGTAGCATATTAAATAATTCAAAG

>TN2061

TTACTAAAAAAAAAAAAAGGCTAAAAATTAACAAAAACATGTCACTAAATA

>TN2062

GGCAGCTGCCTTGGTCAATGTCTCTAATGTCCAGCAGGTTGAAAGTGAACT

>TN2063

TGCCCTACCATGGGGTGCAGTGTAGAACCCCAAAAGGGAAACAAACTGAGA

>TN2064

AATAGAAATGTACAGCATTGCAGTGAATAATCTTATTAATATCTAACATTT

>TN2065

AAATTAATAAAAAATCATAGCATTGAATTCTATAGCTAAAAGAAACTTTAG

>TN2066

ACATCAATTTATTGCATTTATAATGAAACCAAAACCCAGAGAATTTAGATG

>TN2067

ATAACAGGTTTTCGTCTTGCACTTTAAAAATAAATACTTTAAGTTGTCGGT

>TN2068

GGTGGAACTTAAAATCGCTTATAGGAACTTGTGAAGGCAAGGTGTGTTGTT

>TN2069

CATTTATCCTATTTTCCTATATAGAAAATGTGACTTTGTTTAAACACTTAA

>TN2070

ATGTGTGAGAATAAAGATTATTTTGAACCTATACTCAGTGCAACATTAATT

>TN2071

GACTTTTGCTTTATGAGAATGTCACAAAAGGATCCAGGTGGCATGTGTCCG

>TN2072

TTGGTACAAGTTCAAAGTACATTGGAAAAAAAAAAGTGTATTCTGCTCTTG

>TN2073

CTTACTTTTCTAAGTGTAACTGTGCAAGACATAGAGAAGTGAATTGATTCT

>TN2074

GAAAATAAACATTCTCTATTCCTTGAAATAAGTCTAAAAGCCACACCAGAA

>TN2075

TTAATGCCCCAGCTAAAGATTCACAAAGACATATAGCAGCAGACAATGTGA

>TN2076

GAGGGTGCATTCAGTTACAGTGTAGAAGATATATTTAAAATACAATTATCA

>TN2077

AGCTTGAACTAGTGAAAAGTATTAGAATCCCTCTACCAATAATTAGAGACT

>TN2078

AACAGAAAATAAAACTCAGGAAAGTAAAAGAAAAAATACTGCTGGGAGAAG

>TN2079

TCAGTTGTTTCTTTGAACGACTCACAAAATATATGAATCTCTCATGGGGCT

>TN2080

CAAGAAGATAATATCTTTCACTAATAAAAATGATCTACTAAGAATGACATC

>TN2081

GTACACATAATCAGTGGCAAGAAATAAATGTAACAAAAATTTCATAAACTT

>TN2082

CAAGGATGCCAATTCTTCCCAATACAAATCAAAACAATTTGGTGTCCATTT

>TN2083

ATCAATGAGCAAGTGGACAAATAACAAGAATGAGCAGTCTAGCGATGAGCA

>TN2084

AAACACGTCTTACTTATATGTGAGCAAGAGTGAACAAGCACAAATAAAACC

>TN2085

GAGAAAAACTGAAATCCAATATTTAAAATAAGCTAAAATATATTTTAAAAG

>TN2086

ATATCATAACTCCTCTAACAAAACCAACCAAAACATGGCAGTCTTGTATGC

>TN2087

AGTTATGGTGGAGCAAATTTGAACCAATATGAAACAAAGGACAGGAGAGGA

>TN2088

GCATTAAGAACCCACTGCTGGTTATAAATGAGATGTTAATAAGCTTTGCCA

>TN2089

GAGTAGCAGAGCTGAAAGGACACTAAATGCATACATGCAATAAGTTTACCT

>TN2090

GGGACATTTGAATAGAAAAAAACAGAATTTTAAACTTCCAAATTTCCTTAA

>TN2091

TAGCCTATATTGTGATATAGAACGGAAGAATTGGTACTGTTCTAAGCAGAA

>TN2092

TAAAGACATATGAGGCAGCAACTGCAATGACACAGAAGTAACCCTTGCTTT

>TN2093

AAAAAACACAAAGGAAAAAAAAAAAAAAGGAAAAGAAGAAGACCTAGAACA

>TN2094

GTGGAAAAAGTCAACATCACTATGTAATAATTGAATGTGAATGAAAAGTAC

>TN2095

TAAACTCACTCACTGGTGTTTTTGGAAATATACATCAAAGCCTAGGGAAAA

>TN2096

AAAATTTGCCATCTCTACACAACAGAATACTACTCGGTCATTTAAGAATTC

>TN2097

TTTTGAAGTTAGATCCCCAAAACACAAAATAATAGGCAGAAACAGTAACAT

>TN2098

TATTTTGAACAATTAAGAGGCAGTGAAGAGATGTATTCTTGAAGACAGTGT

>TN2099

TTGTAAATATACACATGCTCACAGGAAAAAAACACAGAAGGTAGAAAATGG

>TN2100

TTTGTTGTTAATGTGCCCCACTGGGAATGGCATGGGTCACTTCCTCCCTCA

>TN2101

ATAAAGACATGAATACATGTGGTCCAAGGAGGCCACAGGTGAAAGGGAAGT

>TN2102

GATTCCGTCTCAAAAAAAAAAAAAAAAAAAGACTTGGTTAATGAAAGAGAA

>TN2103

CTATAAAAGCTAAACTCAGGAACTGAATAGAAGAGCAGTTGACAGAGCCTG

>TN2104

TAGGGAATCCGGAGGAAGATCTACCAAGCAAATGGAAAACAGAAAAAGGCA

>TN2105

ACTCAAAACCGCTCAACTACATGGAAACTGAACAACCTGATGTATGTACTT

>TN2106

CTTTGGATAAATATTCATTAGTGGGAATGCTGGATCATATGGTAGTTCTGT

>TN2107

ATTGAACTTTGACAAGAGGGCCAGGAATGCACAATGAGGAAAGGAAAGTCT

>TN2108

GATGAATGCATAGTCAACTTTTTAAAATGTTATTTTATATCTGTTGGCAAC

>TN2109

AGTTAATGAAAAAGAACTCCTGAATAAAAACAGTTAGTGAAAGCCAATTAC

>TN2110

GTTTTTGCTCTCTTGCCTATAGGTTAATATCCAGAGTCACACGTGGAGAGG

>TN2111

CTACCAATTAATGCCGATTATCCTCAAACTCTTCCAAAAACTGACGAAAAG

>TN2112

CAGTAAAACTAAATCAGAACAGAGGAATGATCAGAAATAATGAAGAGATGA

>TN2113

TATGAAGAGTACACTTAGGAATAAGAACAGAAGTTGTTGTTTTTCTCTAAT

>TN2114

ACAGTGAACACAGCCATGGGAGCGTAACCCTCCCCACCAATGCCTTCCTGG

>TN2115

CATTAAACACCGACACCTGGTGGCGAAGCCAGGAACAGCACCAGCAGATTG

>TN2116

ACAGCCCAGTTCTTGACAGGTTTGTAATAGAAGGACCTTGCTCTGGGGACA

>TN2117

TCTATGCATATTGATTACAGAGTTAAATGTTAGAAGTCAACCGTCAGGTTA

>TN2118

TAGTGCAATCTGCATGCCTATCCAGAAGGGACACGCTGACAATATTTCCAA

>TN2119

CTGCTTCCCCTGTGTGCAGCTCTGTAACGAATGACTGAGCTAAGTGCTTGT

>TN2120

AAATTTCTGCATCTAGATGTAAAAGAAAGTCTGTTTTTTTAACCAGCTTCA

>TN2121

TATGGACTTTTTAAGATACATAACCAAAATGAAATTATCACGCTTGATAAA

>TN2122

CAAGACCAGCCTGGCCGACATGGTGAAACCCCATCTCTACTAAAAAACTAC

>TN2123

ACATATATTTTACCATGTATGCTAGAAACAGTATTAATATCATCAGGAAAA

>TN2124

TTGTGACCATGTGCTCACGTAAGAGAAAGGACAAACTATGTAATATTAATG

>TN2125

GGGAGATAGATGTTTCCTAAATAAGAAATAAAGAAACCTGAAATGTCTTCC

>TN2126

AATGAGAAAAACCATGATGGTCGAGAAGGCATGGCCATTGCAAGTCAGAAC

>TN2127

TAGACGTGCTTGCCTGATTTATCAAAAAAAGTCTAAATGATTCCACAATAC

>TN2128

CTGGGAGACAGCGAGACTCCGTCTCAAAAAAAAAAAAAAACAAAAGAAAAG

>TN2129

CTCAAAAATAAATAAATAAATGAATAAAATATAAAATAATAAGATAAGTAG

>TN2130

TCCTGGCTTCCCTGGGCCACACTGGAAGAAGAAGAATTCACTGGGCCACAC

>TN2131

ACCATAAAAAAAAAAAAAAAAAAAAAACTGCTGTTACCGTGTCGACAAAAA

>TN2132

TTTCCTCCTTAGGAAGGTGGTAGTAAAGATGAAAATAGTAAGTAGTAAATA

>TN2133

GAAAAGACACCAGATGTTTCCGATCAAGAGACAAGAGAAACATTCAGCGTG

>TN2134

GGCACAAGCACACCTTACCATAGCAAAGCAAAAGAGAGAGCAAGAGAGAAA

>TN2135

TCACTATCACGAGAACAGCACAGGGAAATCCACCCCCATAACCTACCACCT

>TN2136

AATATAGAGGAGTAGCTAACATTGCAAAGGTTATGACTGAAGAATCATATA

>TN2137

AAAAGTGTTTGGTTGTCAAATGATCAAAAGTAAATGATAGCTGAAAGTGAT

>TN2138

CTAAATTATAAAACTCATTTTAGATAAAGGTGTGTGAAACTGCATGTCAAA

>TN2139

TAAATATCTCCTTTAATAATCCAATAAAATTGTATAAGTTCCAACATATGG

>TN2140

GACTCTGGGCCACTTCTGTATCACCAACGTTTACAATGATTGAATGTCACT

>TN2141

CTAGGTTCATTTACTTCTTATTCTAAAGCCAAATATTTAGGTCCATTAGAG

>TN2142

TGGAATGCCCTGGATAATGAGAGAAAAAAGGGGAAAAGAAGTCCTTTAGAT

>TN2143

AAATACTTTTAACCCTATGATGTATAAATTCCTACTCTGCACAGTTTTTAC

>TN2144

GAGAGATGCAGAGAACCAAGGAATCAATGGTGATTAAGACCCTGATTGCAA

>TN2145

CAGAGAAATCTAATAGAAACGCGGGAAGCAATCATAAAAGTCTTTAAAGGC

>TN2146

ACACACACACACACAGAATATAGAAAATGTATATACACATATAATACAAGA

>TN2147

CATCAAAAATTGAGTTACCAGCTGAAATAATGTGATCATCTTGGTCTTTTC

>TN2148

GTCTCACGAGATCTGAGAGTTTTATAAGGGGAAACCCTTTTCGCTTGGTTC

>TN2149

TTTATCAGCAACGTGAAAACGGACTAATACACATGCCATCCATTTTTCCTT

>TN2150

ATATTTTATTGTCCTCAAAATTGCTAAGAATGAATTAATCCTCATTTGCTA

>TN2151

TATTAGTTATCTACTTCTGAGAAACAAGTTACACTAAAATATCTCAATCTA

>TN2152

ATTAGCCATGAGACTGTAAAATAAGAAAAAGAAAAATAATCGACAGAAGAA

>TN2153

CAACACAGTAAAACTCTGGGAAATGAAGAGACAAAAAAAAAAAAAAAAATG

>TN2154

TACCCCAAAACAAAACAAAAATCACAATAGATTTTAAAACATTTTCATACG

>TN2155

CCTCCCACTTAGGAAGAGGCCGGCGAACTAGCAAACACCTCTAGTTATACA

>TN2156

TTAATGGAAACAAGGTGCCAAAAGCAATATCACTGGGCTGAACAGAATTCA

>TN2157

GTCTCAAGAGATCTGATGGTTTTATAAGTGTTTGGAAGTTGCTCCTTTATT

>TN2158

GATAGGATTCTTATTTGAGTAAATTAAAACTAATAGGAACAACATGACTGA

>TN2159

AAGAATATTAAGAAATGTAAACTTAAAAAAGTTCTTATTATTCATAAATAC

>TN2160

AGAGTGAAACCCCGTCTCAAAAAAAAAATAAAAATAAAAAAAGAAGAAGAA

>TN2161

TATTTTACCTGGATGTTGGCTCCATAATCCACAGATATTTTATTTTTCTCT

>TN2162

CCATTGCTGTATTCCCAGCAAAGAAAAAATAGTAGTTACTAAAAAAAATTT

>TN2163

CAACAAACATTATCAAAGTCCTTAAAACGCACCAACCCTCAGTGGACACTC

>TN2164

CCCTCAGTGGAAACTTCAACCAACAAACATTATCAAAGTCCTTAAAACGCA

>TN2165

CCAGCCCTCAGCGGAAACTCCAACCAACAAACATTATCAAAGTCCTTAAAA

>TN2166

CGCACCAACCCTCAGCGGACACTCCAACCAACAAACATTATCAAAGTCCTT

>TN2167

CCAACAAACATTATCAAAGTCCTTAAAACGCACCAACCCTCAGTGGAAACT

>TN2168

TCAAATTAATACGCAATAATCCAATAATCAACAGCAGCTCAAAACTTGTAT

>TN2169

AGGAAGGAACTGCATTTCTCCTAAAAAAGAATTCTTAGGTATCAAGGAAGA

>TN2170

TTTTGACATGATCCAAAAATAACCAAAGTAAAAGGAGTTTGTCAGCCAGTG

>TN2171

TCTACTTATATTAAGTGAATAATAGAAACATATCACCTTCAGCATAAAATA

>TN2172

GCAGAGATCCTCAAGGAGTTCACACAAGAGACAAAGACTCTTTGCCATCCT

>TN2173

AACAAGGGAAATGCCCCAAACATAGAATGTTCAGGACCCATGTCACAGGAG

>TN2174

TCCAATGTGTCCATCAATACGAGGAAAGAAGGCCATTTCACTGGGCATGGA

>TN2175

AGGCATCACTCCTAAGTGAGCAGTGAAGCTTAAGCAAAGACAAGAAGGATA

>TN2176

TCCCACAGGAGGCATTTGGCCCTGGAATGACAGACTCAACACAATAGAAAG

>TN2177

GAAGAGAGTCTCTGGAACATTGGATAAACCCACTTTCAACGTAATGCCTAC

>TN2178

TGGTAGAAATTATTTAAATATTCAGAATACAAGACTTCTGTCAGAAACGTT

>TN2179

ATTATTATACTTTCAGGAGAAATAAAAACAGGATAACGATTTTCATACACT

>TN2180

TCAATGATGAGAACAATCACAGCCTAATACCAGACAGGTGTGCTGAGGCTG

>TN2181

CACTGAGAAAAAATTATTAACACAGAAAACTATCTTTTCTTGGACTGATTT

>TN2182

AGTATAAATTTAAAATCAGAGCAGTAAATTTTCAAAGGCTGTGTGAACAGT

>TN2183

TAATTTTATTCCAGTGTTTAAATACAACAAATTCGACAAATGTGCATGGAG

>TN2184

CTCGGTAGGTTTCCGTGTAATTTACAATATACGTGACTATTCATACATGAG

>TN2185

TAAAGAAAGGAAACGTAAGACATTGAACGGTAAGACTTTAAAACCACCAAG

>TN2186

CAGAGAGAGTAAGTTCAACACACACAAATTAAGAGAATCCAGAAATGAAGA

>TN2187

TGTGTATATAGCTGTAAATTTAGGGAAAAAATATTTTTTTCAATATAAATA

>TN2188

TTTCAGGGAAAATATATAATGACTCAATATTTAGGCCATGGTACATATAAT

>TN2189

ACAGACGAAAGAAAGAGAAAGAGAGAAAAAGAGAAAGAAAGAAAAAAGGAA

>TN2190

GGAAGGAAGGAGAGGGAGAGAAAGAAAGAAAGAGAGAAAGAAAGAGGGAAA

>TN2191

AAGTCCTGTGACCTTTGGAATTCAGAACTTACATGAGAGGCCTTGCAGACT

>TN2192

ATTTTTAATGATGATTGTGCTTAACAACTGTTTCACAAAATTCCTGCAAAT

>TN2193

GAAAAAACAAACAAACAAACAAACAAACAATTGTCCACCCACCCCGTCCTC

>TN2194

GCTCATTACCTTTATCTTGCCTTAGAAATCTATTCTTAAAAGCAGTAACTA

>TN2195

ATAGTTCAAGACCATGGCCATCATGAAAGGCCTGTTTGATGACATGACATT

>TN2196

ATTAACATACATTATTAACCTCTAAAACAAATTAATCAACTTACTTTTACA

>TN2197

TCAATAGAAATCAGTCGGTTTTCCCAATTTGATTATAATAGATTATATATG

>TN2198

TGATAACTGCAGTTGATAGACCCACAAGAAGCAGATACAGGTGTGGGAAAG

>TN2199

TGCACACATCCCAACACAACACAACAAACGCGTAGCACTGTTTTTCAGGGG

>TN2200

TTGGATGTCTTGACATATGTCACATAATACATATACATATACCCGCACACA

>TN2201

AGACAAGGAAAAACTATCAAAACAGAAAAAGGAGTGGATGCAAAGGAGTTT

>TN2202

CAGAAATACTCACTCCTAGAGCTAGAAATGTCTGGCAAGGTAGCTGGGCAG

>TN2203

AGAAAATGAGAAAATATCCTAACTGAATGATAATAAAAATAATACATATCA

>TN2204

AGCCTTAATTACTTACATTAGAAGGAACAAGTGCTGTGGTAACATAAGAAT

>TN2205

AAAATATAGAAGATCAATAAAGCCAAAACTTTAAGATGATCTCGTATATAA

>TN2206

ACGCAATTTTAACAAGCTTAAAGAAAAGGAGAAATAATATTAGGAATAAAA

>TN2207

ATGGACTGAAGAATGAGAACATATAAATCATTTTAAAACCCTAAAAGAAAT

>TN2208

GTTTATCTAATGAAGAATAAACATGAAAAAATCTAGTAATCATGATAATTA

>TN2209

AAAGATATGGTTATTGTAAAGATAGAAGGCATTATTTGAAAAAATACACTT

>TN2210

CAATAATATTTTTAAAGCTTCAAGTAATAATTTTGACTTTTGGTTGACAGC

>TN2211

AAATAACCAGGAAATTACAAAACAAAAGCCCACATGATTTACAACAAAGTT

>TN2212

AACAAAACATATATTTAGTATTCTTAAAAAGTCAAAAGTTCAGAGTTCGGT

>TN2213

TGAAGTCACAAAATTGTGAATATTCAAAAAAAAACCCACTGAATTTTATAT

>TN2214

CAGTAAAATTATATGAATTTATGTGAATTTTGCATTAATAAAAGCTGATTA

>TN2215

ACATGGTGCTCATAAATGAGAAGAAAAAAGATATTTAAATTCAGTAAAGAT

>TN2216

TAAGTCTAAAAGTCCAATGAATTTAAAATAAACATTAGAAAATATTTTAAA

>TN2217

GTAACTTCAAAGAAAGCTGAAGAGAAAAAAAGCATAAAGATAAGACCAAAA

>TN2218

TTTTTTGAAAGGATTATTAAATCTGAACCTGTAGGAATATTTATATAAAAA

>TN2219

AAGAGAAAGTAAGGGCACAACTAACAATCAAGAATTGGCAAAGATGACATC

>TN2220

AGTGTAAATCCTGCATACATTCAATAATTCATCAAATTTATTGTGAAAGAT

>TN2221

TATAATCTCTTTAGAAAACACCTTAAAATTATCTATTAAAGCCCAACATAC

>TN2222

GCATGTCCTTGGTCCTGAAATTTTTAATCTTGAGTCTATGTCCAATAGACC

>TN2223

GGATGGATGGATGGATGGATGGACGAATAGTTAAATGGATGAATGGATACT

>TN2224

CAATCATCTTTTGATGTCCCATTCTAAAATATGAATGGAGAGAGAAAGTTC

>TN2225

TATGGAAAAAGAATACATTCAGTAGAATAGTTCAAGTTCTAATATTTGAAA

>TN2226

TGATACATTAAAGACAATTTTGGATAAGCAAGGCCTCAAAAATGTAACCCC

>TN2227

TAAATTCTAAAAAATAATTAATTAAAAGAATTGAACATGCGAGCCTGTGGG

>TN2228

TATAGGCAAGTATAACTCTACGATAAATGAAAGCCAAATTAAAAATAAAAC

>TN2229

GCAGTCAGGGCATCTGAAAACACAGAATCTTGAAGGCAGACTACATAGAGT

>TN2230

TTTACAGCTAATAAGCGCACAATGAAATAAAAAGGCCAATTGATGGATAAT

>TN2231

CGTCGGTTAACATGTAAGGAGCTTAAATACTTTACAACACATCATAAATGC

>TN2232

GATGGTGACTTCAGTGCCTGTGCACAACAGAGCTATAAAAGTCTGTGTGTC

>TN2233

GACCAGAGTCAGACTCTGTCTCAAAAAAAAAAAAAAAAAAAAAGGTTTGTG

>TN2234

TCTGCTACATGTCGGAGCAGAGACCAAGATACAATTAAGATAAAAGATCTC

>TN2235

TCCTTCGGAAACTGCTAATGTCCCCAACTAACAAATCTTTCACAGAATAGG

>TN2236

TACGGGAAGAAAGCAAAGACACAATAAGACATAGTTAATTGCAACTCCCAA

>TN2237

AAAAAAAACCGACAAAATTGGAACAAAAATTTTTAGTGGTGGCTCACGCCT

>TN2238

ACTCAAAATTTCTCTCTAAGACAGAAATAAATAGAGGTCTTCACTTCAGCT

>TN2239

GACTCAGGCCACAGAAAACTTACAGAAGGAGAGACTTTTCCAAGAATCTAA

>TN2240

GATTCGCGAATAAGGCAGCTCCCAGAATCACAGCAGATTCAGAGAGACTCC

>TN2241

CTTTGAAAAACATGACTAGATGCAAAATTTGAGTATTCGTCTTGACGGCTT

>TN2242

TGCCCAAGCCATATTTAGTTCCTTTAACACTTACATAGAATGGACTAAAGG

>TN2243

AACTCCACCACCTGTCCGAAGATCTAAGAGAACACCAGCTGTCTGACAGTG

>TN2244

TCAAGACATTACAGAAAACCTGAAGAAACATGAAATAAAGAAGGAAATAAA

>TN2245

ACCCATTCATAACCTGCTACCCCTGAATAAATCCAGCAAGCTTCTTGTCCA

>TN2246

ATGAGATAAGATGGTCTGGAAATAAAAGCTGGGACACCAGGTGGAAACAGG

>TN2247

TAATGTGCGAAACACAAAATAGTGCAAAACAGTTAGCATACTGTCTCGCGT

>TN2248

AACCAAATATCACCTGTCCCCCAATAACCTATGGAAATAAAAAAAAAATCA

>TN2249

ACATGTAGGTACTTGTACAACTTGTAAAAGTTAAAACTTTTTTCTACTTTC

>TN2250

CTAAAAGTTGCTGTGATATCCACAAAAGAATAACGCCTTAAAACAAGCAAA

>TN2251

AGAAAAAAAATTGGATAAGAAAGGGAATATTGGATGCATTTCATAATGTGA

>TN2252

ACGGGGGACCGCTGGTTCTGAGTTTAAAAAGCGGTTTGATTTTTTTGAGCC

>TN2253

AGGTGCGCATATTAAAGTGAAAATAAATTCACACAGAAAAATAGAAATCTA

>TN2254

CCAATCTAAAATGTGAAACAGAGAAAATCTGCCACAATTGAATAGGAACTG

>TN2255

GAATTTAAACTTGAAAAGTCACTGAAATTACCTTTCCATCTGGAATCATAA

>TN2256

CTTATTTCTTAGATGTATTCTGCTTAAAAAGTAGGGCAGAAAACAACAGAA

>TN2257

AGGACAACAAAAAATCTTGACAAATAAGCATTGTTAGTCCAAGAAAGAGCA

>TN2258

TCCGGCACGTGTACCCCGGAACTCAAAATAAAAGTTGATTTAAAAAAAAAA

>TN2259

AAATTAGAGCAAAGAAAGGAACCAGAATGGCAGCCGCCACAGAGCCACTGC

>TN2260

TAGTCACAAAAATGTAACTTTCCTGAAGCCAGGGATTTTGTTTTGTTTTGT

>TN2261

ACATGAGAATAAAAATCCCTTGTTAAACCAAGCTGTTATTTTGTTAGATTA

>TN2262

AAAAGAAAAGGGGTAATGCAAAGAAAACGTGTAATAGCAAACTGATAGTTC

>TN2263

TTTATAGTCGCAAATAATGAGGACAAAAGTATATTGTTCCTATACCCATGC

>TN2264

CCTATAAAACTTGGCCTAGCAAGAGAAGGGTTAAAGAGGAACAGAAGATAA

>TN2265

AAAATTAATAGCCTTTAAAGTACATAAAAAATATCTTCCCTAATTAGAATT

>TN2266

TAGATATTTCAATATCTTAAACACAAAATAAGCAAATAAAAAACAAAACAC

>TN2267

TATTATCAAGCTCCATATTATATATAAACATGCATATATATATGTGTATGT

>TN2268

CTGTTATAGCAAGAATAATTTTTTAAACACTTCTACTTAGACTCTGACATA

>TN2269

GAACCAAACTTTGAGGACATCCGAAAATTTAGGATTAGGGAATTAAAAACA

>TN2270

ATTTCTCATTATTTCCCTTAGTGGTAAACACGTCTCAGACATTCACAATAA

>TN2271

AGTCAGTATATTATTAGCTCCCTGTAAGGACTTCCCCTGTGGGATGCAAGA

>TN2272

GTGCAAGATGTTTTTCAGGAAAGTTAAAAATTAAATTGCTACTTTTATCTT

>TN2273

TGACAGCAAATATTCTTCTAATTAGAAAAAATGCTTCAATGACAATATTTC

>TN2274

TTTTTACGCTCTTTAGTCAGTAAACAAAAGCAAGCTCGGCTGCCGCTGGGT

>TN2275

TGTTAGTTTGCTAATATTGTTAATGAATGAGTACCAGCATAATGCTATCCT

>TN2276

CCAGCCCTCTTTGTAACTATTGAGGAAATATTTTAAATTTCAATTAAAATA

>TN2277

GCTAAGGTAGCTTTCCTTATGGTAGAAATTTACATGTGGATTTTCAAGGAG

>TN2278

CATGGACTGTACCAGGAATGCTCACAAAACCCAATCAGGTCTCACAGCACT

>TN2279

CCCAGCAAGTAAAATCCCTTCTTGCAATGGTACAGGATACTCATTCCATAC

>TN2280

AATGACAATTATTCCATCAACACCTAACAGAAATGCAAATCAAGCCAGCCT

>TN2281

CATTCAGCCCAGCAGGGAGAAGTTCAAATGAACACACAGGCTGTGTTCTGT

>TN2282

TGTTGTTCTCAGAAAAACTTTTTATAATCAGTTCTTATATTAAACAGAATC

>TN2283

CTTTGGCATCAGCACTCTTTCCTACAAGCACAATAGTTTTTAAAGAAAGTA

>TN2284

TAATGCAAAGGAAAAACATTGCAGGAAATATTAATTTGGTGCAAAAGTAAT

>TN2285

TGTATACATCATATATATAAATAAGAAATATATAAGTATATAAGAAACAGA

>TN2286

CATGTGTCTAAACACCCAATCCTATAAAATTATGAAAGAAAAGAAAAAACA

>TN2287

CATACAAAAAAAGCTTTTCATACTTAAAAGCCCACTCTTTTGAGTTCAGTT

>TN2288

CCCAGAGCTATGCAAGGTATTGAGCAAATGAGATAGTTTGAAACTTCGCAT

>TN2289

AAATCGCAATGCTAAAATACTTCTTAATACACATGGCACTCTGATAAATTT

>TN2290

TTGATTAATGAAACATACCCTTTTTAAAAAAAAGCAAAAATGCGAGTGAAT

>TN2291

CATGGTCATGGCGAAAGCGCCCCTGAACTGCACACTTTAGAACGGTTAACT

>TN2292

AAAACTCCATCTCAAAAAAAAAAAAAAAAAAAAAAAAAAGCAAGAAGAAGA

>TN2293

AGAGGAAAACGGATGCTTTTTGACTAAAAGACTAAAACTTTTATGAAAAAC

>TN2294

AGACTTTATACAAAAATTAACACAAAATGGATCACAGACTTTAAAATATAT

>TN2295

TTCACTGAAGAGTACAGATGGGAATAATCACCAGATAAGGTACTCACGTCG

>TN2296

TTCTCGTGATTGTGAAACTAAAGTGAAAGAGATCATTTCAACCCTAATTCT

>TN2297

CCCAAATGCTAACTATACAGGGTAGAAATAAATGTGTAAATACATGAATAA

>TN2298

TACAAAACTCCCTATATTCTTGACGAAGCAATGACAAAAATGTATGCAACA

>TN2299

TAACAAAAGACAATGTGGCAAAAGGAAGCATCGATGAATTCGTCCACAAAG

>TN2300

TATTATCAAGTCAATAACAGTATTGAAGGCGCAACATATTGAAATCTTTAT

>TN2301

CATTACATATATATGTATACTTTTAAATATAGTTTTTCTTTTTTTTATTAT

>TN2302

TAGCTAGTTTCTAGAAACACACAAGAAAATGACACCAGCAATTCCTTTTGG

>TN2303

TGGGGCTGGGGATTAAACAAGGAATAAGACACAATCGCAGAGGGTAAACGC

>TN2304

TACTCTTAAATCAATCTTCTGCGTGAATTTATTAGTCTACTAGTAATCTGA

>TN2305

TAAATATATCCTCATGGAATTGCAGAATTTGCTTTTAGCTCAAGCACGAAT

>TN2306

GAGTTACAAAGAGTTTCCGTACCATAAGCTTTGAAAATCACTAGCTTTTCA

>TN2307

GCCAGGGCAATTAGGCAGGAGAAGGAAATAAAGGGTATTCAATTAGGAAAA

>TN2308

ACCAACAACAGACAAACAGAGAGCCAAATCATGAGTGAACTCCCATTCACA

>TN2309

TATCTGATCTTTGACAAACCTGAGAAAAACAAGCAATGGGGAAAGGATTCC

>TN2310

CCAAAAGCAATGGCAACAAAAGACAAAATTGACAAATGGGATCTAATTAAA

>TN2311

CTAAAGAGCTTCTGCACAGCAAAAGAAACTACCATCAGAGTGAACGGGCAA

>TN2312

ATATCCAGAATCTACAATGAACTCAAACAAATTTACAAGAAAAAAACAAAC

>TN2313

AATGGGTTCCCTGATTTGGAAATGTAATAAAGTTCCACTGTTTCACTTCTC

>TN2314

TACTGGTACTTATTGCTGAAAATAAAATACATTACAAATATCCTGGGCCTG

>TN2315

TTACTCCTTAGAGTATAGATTTCATAAAAGAAGAACCAATCTTATTAACCT

>TN2316

CCCCAGCCACTGACAATGCATTTGGAAAATCTGAGAACTGGTATAGGGAGT

>TN2317

CCAGGATGGAAAACATATTGGACCTAAGGTATACAGCCCAAGGGAAGTTAA

>TN2318

CCCACCTGTCAAGGATGGAGACAGGAAGAGATAATTGAATCATGGGGGCGG

>TN2319

CCTCAAGAGCCCACTTAAACTATTAAAAAACTGAAGTCATGATTCTGGATA

>TN2320

TATGTATCCATAAAAATTAAAATTAAAAAAGTTTTTAAAAGCTTCTTAATG

>TN2321

AGAAGGACTTGTGACTCTCTACATGAAGAAGCATACATAGTCCAAGGAAAT

>TN2322

ATTTAAAAAAAAAAAAAAAAAAAAAAAAAAAAAGGAAGAACGCGTGGAGAA

>TN2323

ATTATTGTAGACATATAGCATTTTAAATAATTCACTGTACTTATGTCAATT

>TN2324

CAGAAGCCTCAGGAGCCTATCAGGAAATAAGGAATACCAATTTTCTTCACC

>TN2325

CAAAACTATTTTAAGTTTGTCCTCTAACAAGATTTACTCAATTGTAAAGAA

>TN2326

ACACCCGCAATCCCACAGCATCCGGAATACACCCGCAATCCCACAGCATCC

>TN2327

GCACCCGCAATCCCACAGCATCCGGAATGCACCCGCAATCCCACAGCGTCC

>TN2328

CATAGGAAGCCTGGCACAAAGTAAAAATGTTCAAACATGAAATCAGCTGTC

>TN2329

GAGTCAGCTGACTTTGAGTTAATAAAAAATGAGATGATTTGGATGGGAACA

>TN2330

TAACTAACACAGGTCCTCAAATTTTAAAAATAAAAATTTTATGAAAACCCC

>TN2331

GAGAAATACAATTATTAACAAGGAAAAGACAACATCAAATGCCTAATCGAG

>TN2332

TTTTTGATATCATCACAACAGTAACAATGAAGTCTAAAATGGGAAGAAAAA

>TN2333

CCTGCACGTTGTGCACATGTACCCTAAAACTTAAAGTCTAACAATAATAAA

>TN2334

CCAAGAAAATACATTGCCACAACAAAAGAAAGAGAGGAAAGAAACGTGTAT

>TN2335

GAGTGGAAATTAGGAGTAAAAAGAAAAGTGAGGACTGCAATAAATATTAAT

>TN2336

TCGCGAGAGGGTACTGAATTCAAATAAAAACAGAACATTTTACAACCCCCA

>TN2337

GGGAGAGCATTAGGACAAATACTTAAACCACGCGGGATTTAAAACCTAGAT

>TN2338

TAAAATAAACTTAAAGTAAAATAAAAATAAATAAACAAATTTGGCTTCGAG

>TN2339

AAGAAAAACTGGAAATAGTTCAACTAAGTCCCAGTGAAAAAATCTTCTGTC

>TN2340

TTGAGGTACAAATATATATGTATACAAACACACATTACATATATAAACACA

>TN2341

AGAATATCCTTAGAGCTCTTTGCTGAAAAAAAAATTCGCTTAAGTATTCTT

>TN2342

TTGAAATCTATCTATCTATATATATAAACATATAAACATATATATGTATAT

>TN2343

CTCAAGTATACATTTAGAACTGTTAAATGAAAGATGAGGATGCCAATCCAC

>TN2344

CATGTAGCCGACAGTCTGTGGGTGTAAAGTGCTTTAAAGCAGTACAGTACA

>TN2345

GAAGCAATATATCCTAGAAATTAGGAAGACAGATTCTCTTCTCCGATTTTC

>TN2346

TTCCTTTACCCCCAACCAACGACTTAAAACCTACATTGTGTTTCTTTTCCT

>TN2347

ACAGGCACATGTGCGAGAAATCCTTAAAGAAATCTAAGGGTAACACATCAT

>TN2348

GGTTTGGTAATGACTTTGTAGATACAAAAACAGAGGCAGGATGCATTTTAA

>TN2349

TTTGCAAAAGACATATGGGATAAAGAACTGGTATCTAAACTATATAAAGAA

>TN2350

CTCTTAAAAAAAAAAAAAGGAAGTTAACAACCTGGCTAAAAATGGGCAAAA

>TN2351

CTAGAGAACTTAAAAAAAAAAAAAAAACTGATGATTGGTCTCCACCTAAGA

>TN2352

TCCCATACTATTACTTAATTGCAATAAAATAGTACCGTCTTAAATAGAAAA

>TN2353

CAATAAAAATGATGAAATAAAGAAAAATAAAAGCTTTTGGGAAGAACTCAA

>TN2354

TAAGTATACACATTTTCAAAATCTTAAGTATATTAAAGGTTAAATGAGGAA

>TN2355

ATAAAAGAAAAGTTCTATGGTAACTAAATAAAGAGCCTTCTTTTACTCAAT

>TN2356

AGATGACATTCTACAAACACAGATGAAGCCCCATCACAAAACAATTCATAC

>TN2357

TTATGTTGCCTGATCAACAACCGGGAATCTTTTTATGGTTTTGAACCACAG

>TN2358

TGTTTAGCCCCTTAAGCGTCTTAACAAAAGGCTATTTGGTAGATTTAAAAT

>TN2359

AAATTATATATGTATATAGAAGTATAAATATATGTACACATATAAATGCTA

>TN2360

AAAACACCTTAGGAAACACTAACTTAAGGGAATCATCAACTATATGTGTAT

>TN2361

GCTATATGTCTGAGAAAACATATATAAGTACATATGTGTAATTATGTCTTA

>TN2362

AGTCATTAAAAGGCTGAGGCCTCAAAACGGTTCTGCTGAATTCAGGTAGAC

>TN2363

GAGTTTGAAGATGGGCAGTGACACCAACTCATTTGTGACCTGATAAAACCC

>TN2364

GAGAAGGAAGGGCAGGCAGCAAGTGAAGACCATGAAGTATTTCTAACATTT

>TN2365

TTATAGAAACAGAATAGGATGCTTGAAAGAACATAGATTTAGACATAGCAA

>TN2366

TGTAGCCATCATCCAATATACCATTAATACCATGCTGAAGGCTGAGATAGA

>TN2367

TTGAAACAGACAAAAAAGCTATGAAAAAGAAGGCAGCAGAATAGGGTTGGC

>TN2368

AAATAAACAGCTAATTATTACAGTGAAAGGCTCTGGCTTGGCAAATAAAGC

>TN2369

TTCAATAAAAAGAAAATTCCTAGAGAAAAAAAGCTGGAGGAACGTAAAGTG

>TN2370

AAAATAAGAATAAAAACGATAGCAAAAACTAGGAAATAATGAGTGCTGACA

>TN2371

GAACATACACAGAGAGGAAGACTGGAATATGATACATAGATAGATAGATAG

>TN2372

TAAAAGGAAAATTAGGCATAAACTAAAATGCATGGATAAATCAAAAGTATT

>TN2373

CTAAATAAACTATAAGCACAATGGCAAATGACTGTTCAGTCATTTAAATGA

>TN2374

AAAAATTGTCTATTGGCAATTAGAGAAATTGCCAAAGGACAATTCAAAGGA

>TN2375

AATTCTGATAATTGCATGGTAATGGAAACTCCACGGAGAACAGAAAACTGA

>TN2376

TCTGGCCTCAAAAATGCATGGAATGAAGTGTCAGAGTTATTAACAGTTAAT

>TN2377

TTAAGAGAAGTTGATGGGTGTAATTAAATTGCTAATAAAGAGGTACAGACT

>TN2378

CACAGACATTTAACAGTAAACTGAGAATCAGAATTTAAACCCCAGTACTCC

>TN2379

CTCATGTTTCTGCAGGCTGTACGGGAAACAAGGTGCTGGCATCTGCTTGGC

>TN2380

CAAGGCCTTTTCCCCATTGTTTTGGAATATTAGCACTTAGCTCCCTTTTAG

>TN2381

TAGTTGGAATTTTAAATCAGCAAAAAAAAATTAGTGGAGAAAATAATGAAA

>TN2382

ATAATTACCCAGCAAAGTATTTAATAAATGACACTCAGAAAACTGGGTAGC

>TN2383

ATAAAATTGAATAAAACCAAAGAAAAATGAGAACATGAACTTTAAAAAAAT

>TN2384

CCACTCAAATCTCATGTTTAATTGTAATTCTCAGTGTTGGAGGTGGGGCCT

>TN2385

TCCGATTGCTGGAAGATATTCCCATAACTGCTCTCACTATTTTTAAAAGTT

>TN2386

TGGCATACTCATCACCTGAGTAGCGAACATTGTATCCAACAGGCAACCCTC

>TN2387

AGCTGCACAAATAGGAAATCAAAGTAAGAAATGCCAAGACATGGACAAAAG

>TN2388

TCTGAAAGATGCCCTCTCTGTGGGCAAACATCATGTTTCGGGGGTGAATGC

>TN2389

AGAACCAACAGAGATTTTCAGAGAGAAATAATTTAAAAGGTCAAGCAAAGT

>TN2390

GAGAAAAACAACCTGGAGTTCCCTGAAACCAATAAAATACATTTTGCCGGC

>TN2391

AAGACAACAAAAGCCAATTCTAAATAATCTTTAAAAGGAAAAATATTCTCC

>TN2392

TTACAAAGTCAAATATGATAAAGCAAATGAAGCATCCAGTATCTTAAACTA

>TN2393

TTTTAAAAAGACAGAACATGTCTTCAATTTTCTTAGAACTGAAAATAAAAT

>TN2394

GCAGTGTGCACTATTTGTATTTCTGAATCATTTAACTTAGCATTAAATAGT

>TN2395

CATAAATAATACAGGCTGTCTGATCAACTTCTTACAGTCAAAGGCACCTAC

>TN2396

TGTTTTTTACTTCAAGAGGAAGTACAACTGTTAGAATAACTTGACCCAGGA

>TN2397

CTGTGGGCAGACAGACACACACAGAAATAAGTCAACAAGCAAACAAATGGC

>TN2398

CAACAGAGCCAGACTGTATTTCAAAAAAAAAAAAAAAAAAAAAAAAAGAGT

>TN2399

TAGTAATTTGGAGTTTTAAAAAAAGAATTGAAGATTCCATGAAAAAGAATT

>TN2400

AGATTCATACAGCATTAAAATTAACAAACTAGATATACACATATCCACATA

>TN2401

ATATAATAGGATAACAATTATACAAAAAATAAAAAATGCAACATATAACAT

>TN2402

GTGTATCTGTGGACGATAGATACACAAATAATTATAAAACATGCATAACCA

>TN2403

TCTTTATAAGATGAGGGCCTAAAGTAAATTTGACAAAGCCCTGAAATTCAT

>TN2404

ACAAACCAAAACTAGCATATTAAATAATCGGGCAATGCTGACTTTGGTCAG

>TN2405

AAAAGGATAATGTATATTCTGAGATAAAATGCCACTGGTAAATTAATTAAC

>TN2406

AAGAGCCCGTCTTACAAAAAAAAAAAAAAAAAAAAGAGAGAGAGAAAGAGA

>TN2407

CAATTCAACTACTGTGAAACAAAAGAACATGCATAAGTTCAAAATAATTAA

>TN2408

ATTTCCTAACTCTACTACAAAGAAGAACCTCACCATTTCAAAAGAGTATTA

>TN2409

GTTCAAAAATAAAAAATGTATATCAAAATTACTTGTAGAGTGTTAAGAGTT

>TN2410

TAAGCCTTATCCAAGCGCTGCAGTGAAGCCAGCAGACTTCACTCATCAGAC

>TN2411

CACTTGAGCACAATCGAATCAATCAAATAGTAGAAAAGTACTTAAAGAAAG

>TN2412

AAGCCTGGAACGGAGTGTTTCCGTTAAATATTAAAAGCTGTAAGAGCCAGC

>TN2413

CCATTTCTACAAAAAAAAAAAAAAAAATCCAAAAATTAGCCAGGCATGGTG

>TN2414

AACAATACAATTTTCCATGTTGAATAAGTTTATGTAGTTTTAGTATCGAAC

>TN2415

ACTGGTACTTCACAAAATCTCCTAGAAGAGTCAATGCAACAAACCAGGAAA

>TN2416

TGGCAAAACAAAAAAAAATCATGGTAATGAAGAGGGACAGACATTTTCTAT

>TN2417

GAGACAGGGATGAGTAAATCTATAAAAGGTACCAAATCTCTTTCAATGGTC

>TN2418

CCCTTCCTGTTGGGAAAACAAAACAAAAAGCCATCGTTCCCACCTCTTAAA

>TN2419

GTGGTCCTAGCCACTTGGGAGGCTGAAGTAGGAGGATTGCTTGAGCCTGGC

>TN2420

TTTTCTGAAATGCAAAATCAATATGAAATTATTATAAAAGCTAAACAAAAA

>TN2421

ATCTGATATAACCACTGTAAAGCTGAACGCTGCTGACTTCGCGGAGATTTC

>TN2422

AGTCTTTTTCTAAAAAAAAAAAAAAAAAAAAAAAAAAAACACTGTATAATC

>TN2423

TATCCATAGCTTTCTCCTATTTAATAAACTAATGAAATGGCATTGTTGCCT

>TN2424

GAAAAATGAGTCCTACTGTACAGCAAATATGAACGCTAATAAAACGTTAAG

>TN2425

AGTAAAGAAAATATAAAACCTGGTTAAGGTTATGCAGTAGTGGTCAAAATT

>TN2426

TTGGATTCACTGTCACACTTCACACAATAAGGTATAAAGGACTATATTAGG

>TN2427

AGTCTGAATGGAAATAACTAAAAATAAGTCATGAAGATAATTCCTGAGTAT

>TN2428

TTAGAAGCATATCAACTTTATGTACAAATTATGGTTTGTATACAAGCAACA

>TN2429

CCGCTCACAACCTAAACATCACCCCAATCATTTCCTCCACAGCAGGCACCA

>TN2430

TCTGGGAAGTCTAATAAGTTTCTCAAACTAATGTCTTACTGTATAAAATAA

>TN2431

AAAAATAAATAAATAAAATAAAATAAAAAAAGAAATATCTGCACTAAATGA

>TN2432

TGCCATGTTGGTCAGGCTGGTCTTGAACTCCTGACCTCAGGTGATCCACCC

>TN2433

TGTTGGGGCTCAGGACACCACCCCAAAATATGACTCTAGAAGACCAGAATA

>TN2434

TCAGGAGATGGAGACCAGCCTGGCCAATGTGGTGAAACCCTGTCTCCACTA

>TN2435

CAAAAAAACAAGAAAAAAAAGAGAAAAAGAAAAACAAAAGAAAGAAAAGAA

>TN2436

AGAAAGTGTTCCCAAAATGTTTAGCAATTGCAGTAGGAGAAGCAGGGTCCT

>TN2437

GGAATGCTCGTGACCAGCACACAAGAAGAAGCACATCAGAATTCAAGCTTC

>TN2438

GTCAGTTAGAAGTGGTCAAGGAAGCAAAGAAAATAACAACTTAGCCAGAGA

>TN2439

AGTAGCTGAAAATGCAGAAATAGAAAATATTCTCTAAATAATGTCTCTGAA

>TN2440

AACACAGAAAGAGTGTTTCATAGAAAAATGCTGGCAATGTCTAGAGACATA

>TN2441

CAAAATTGATCATGGCCCTTTCCCGAAAAGACCCCCTTCTTGCCTGGGGAC

>TN2442

CTTCGGTAGAAAAATTCAGAAACTGAATATGTGCACCTATGGAAAAACATG

>TN2443

CAAACTTAATGAAAGTTCATATCAAAATGTATAAAATCAATGCTCATAAAA

>TN2444

CGTCCAGAAGTCAATTTGTTTGTGAAATATGAATATTACCAGCACCGTACC

>TN2445

AAAAAGGAAGAAAAAAGTCCAAGTGAAAAATTGTAGGGAGGAAACACCTAA

>TN2446

ATATTGTGAGCAAAATTACCATTTTAAGCAGGATAAATTATACAGCCACAT

>TN2447

ATCAAACTACCTAATACATATCAGAAAATAGAACAGAGCCCAGCTCTAATC

>TN2448

GTTTGACATTGTAAAGCCCAAATAAAAGGATTAAAATAAGCATAACCTGGT

>TN2449

GAAAACAGCTGTTTCTGGAATGAGTAATTAGGAAAGCCAGGTAAGATGATT

>TN2450

TAAATCTGACACTCTCTTAGGGTCTAAGGCAATCAAGTTTCCCATAACGTC

>TN2451

ATGAGCTGTGCAGTGCTGTGGGTATAAATTAGGAATTGAACAGGAGAATCT

>TN2452

CGAAAATGATTTAAGCCCTAGAAAGAACTCCTACCAGGGGCATCATAATTC

>TN2453

ATATACTAAATGATACCCTTCTCAGAAATGATAAAATAGTAACATTGGAAA

>TN2454

CAGAATAAGGCATTTTATTCTGCTTAATTCTTTCCAAGATGGAAATGAAAA

>TN2455

GAAGAAACCAAGCCTGGAAGAGATGAAGAAATTTGCCTAAAGTGACATGCC

>TN2456

CCAACTGTCAGAGGTGTTAAACCAGAACAACTCCACCTGGAATAGGAGCTG

>TN2457

GGACCTCAGCGTAGACAGCATCATCAACACGGATGTCTGTGTCCCACACAG

>TN2458

AAAGCCTATTATATTTTGCAAATATAAGTTAATTCTTTGAATAAAACATCC

>TN2459

TAGGGAAGGCTCTTCATTTAAAAATAAATAGGCAGATTGATGTTCTGTATG

>TN2460

GCTCAATGTTTTTAGCTGACATGTTAATCTAGACATTTATTTTTACCAAGA

>TN2461

CAAGAGGGTGCCTAAACCCTCCAATAAATATTTTAATGCAATATTTGGAAA

>TN2462

TTTGTTGTTTAGACAAAAAAAAAAAAAAAAAAAAAAGCAGGATACTGCTAA

>TN2463

ACATATAACTAAAAACAGAGTATTTAAGGATTAAATGTTTGAGAATATCTG

>TN2464

GTGATTAAAAGGCCAAAATGTCAGCAATACAAAGTCTGCTTATGGAGAGGT

>TN2465

TTATGTTTAAAAAGACCATTAAAACAAGTTGAGAGAAGATGGAAGTCATAC

>TN2466

ACCTATATATTTCTCCTGACTTTAGAATATACATGTTTTGATTATCAATTT

>TN2467

TTATTGCTGGCAGAGAGAATTATATAATAATTATGCTAAGTGATAGACACT

>TN2468

TATTTTGAAATAAAACAATTGTGAAAAATACAATGCTTAGCTTTGAAAGAG

>TN2469

AAGAACTTCAAAACCAGCGGCCACCAAGCACGAGACCCAGTGTGACTCACG

>TN2470

AACAGAGCGAGACTCCATCTCAAAAAAATAAAATAAAAAATAAAAAGAGAA

>TN2471

ATATAAATAATGGGCAAACCTATAGAAATAGACATTTATAAGTATTTATAT

>TN2472

AAGTCAAGGACAGAAAGATCACTTGAAATTTATGATTATAGTTCATGAGAA

>TN2473

TAGTTACTTACCATGAAACATTTGCAAGACCTACTGTAGAACAGAGAATTC

>TN2474

TTTCAAGTTTTAACATCCAAGACATAAATTTGGTAGAAAAACATCATGAAT

>TN2475

TCTAAAGTGAGTAAGAATATCCTGAAACCAAAGGAATTATTTAAAAAAATA

>TN2476

TTTCTAATTGTGCCCCCACCTCAAGAATTTAAATTTGAAATTAGCAGAGAC

>TN2477

CAAATAAATATTGAATGCCTAAAGTAATATTGTAGAAGTGACTTCACATTC

>TN2478

ACTCATGTTCACCAAAATTCCAATTAAGAACTCTCCTCAGTAGTGTTAATG

>TN2479

TTATGATTTTATTGTCAGAACATTTAAAGCTAAATGTAAAGCCTAATAATA

>TN2480

ATATAAATACATGCAAGAATGACTTAATCAGGTATAAGTTTTGAACTCAGT

>TN2481

AGAATGTGGGAAGAAATAATAAAAAAACAGATGGGGTAAATAGAAACACTC

>TN2482

GCATTCATCAATACTAACTCCCCCAAAACTATTAGTGAATTTTTAAATACT

>TN2483

GCCCTGTGGTTTGTGCCTGGCTTAGAATATTTATCATCAGCTGGCAATCTG

>TN2484

GACCTTACTTTTGTATTCCAGGTGGAAACCAGCAGCTGCCACACTAATGTC

>TN2485

TAATGATGCTGGCAGGTGCTGAGGAAAAGAAAATCGTTCATCACTCAGAAG

>TN2486

CGGCATCCATCCACGTCACAAACTGAACGCCTAATCTATCTATCCCTGAAA

>TN2487

TTTAAAAAAAACAAAACAAACCATGAAAGAACAATTTATTTTTCTTTTTCT

>TN2488

AACTATGATCTTCCATATACAGTTCAAGTTGTTTCCGTATGGCTCAGGGTA

>TN2489

AGTTATAGATCTTGAGTCTATAAATAAATCTATCCAAGTTCTCTAAGAAGG

>TN2490

GATGATGTCCAATGATGGCATCTCCAAAGTGAATTCTGTTTAGAATAATTT

>TN2491

GGCGCTTAAAATGTACAAATGATTCAAGTGCTAATCATGATTGCGCTGGAG

>TN2492

TACGTAGCCTGGATAGAAACATGAGAAGTAACTCTTTGGCACCACCACAGA

>TN2493

AGCTGGTGCAGATAGAGAAGTTAATAAAGCCTCCAACAATCGCTATAGAGA

>TN2494

TCTCCTCCACTGTGAACACTCGAGGAATTCCCGGGAGCTAAGAAACTGGTA

>TN2495

CCTTCTGCTCTCAAGAAGTGTGGAAAACACACAAAAACCCAGCAATTGCAT

>TN2496

AGATCTCAACTATTTCAAAATACGTAATTAGATAAAATACTGTATTTGAAG

>TN2497

AGTGAGTGAGACTTTGTCTCAAAAAAAAATAATAATAATAATAATAAAATA

>TN2498

TACCCAGTACCAAACTGATTTAGTAAATGATGAAAATGTATCAGTACAAAT

>TN2499

AAAAAGCAAAATGGTGGCGGGGACAAATGCAGCAAATAGTCAACTTCAGCT

>TN2500

GATATGAGTATGACTTCTTTAGCTGAAAATTTTTAATTGTGTAATGATGAC

>TN2501

TCATTCAAATTCAGAATTAAGGCTAAAGTTAAATGAGGGACTTTAAAGAAT

>TN2502

CATTTATCATAACAAAGGGAACAGCAAGGTTTGGGGAGGGTGTGCATTACA

>TN2503

AACCCTAGAGGGGACTGTGAGCATTAATATCACATTGGTAAAAGGACTGTA

>TN2504

GACAAGAAGTATAAATAAAAAGTGGAAACGTCATCACTGGGCCAAAATTTT

>TN2505

AAGAAGCTTGCTTATCCATTTGGCTAATTAGAATATATACATCTCGTACAT

>TN2506

CATATATACGTGTGTATATATGTATAATTCCTATACATATGTGTATAAATT

>TN2507

ATCATTTTCTGGGATACAAGAAAGAAAATTAGAATTTTTACCCAAAATTAC

>TN2508

CTTTGTTACATCAAAATGGTTTTGGAAACCAGTTACATACAGAATTAAGAT

>TN2509

ACCTGGCCTATGATCACGATTTTAAAACACAAAAAATAACGTTCTTCCTCT

>TN2510

TCATCTTAAAATAACCCTAGAAATGAATGTCTCTGCAACAAGCCATGAAGT

>TN2511

TATGGAGAGAGAGAGAGAGAGAAGTAACTAGAGAAGGCTAATTAGCACCTC

>TN2512

TTAAGTATTTCTTCTTCCCAAGTAAAAATACAAAACCTTTTTTCTTCAGTC

>TN2513

AATTTTCTGTTTCAATAGACCATCTAAAATATCTCTAAATGAAAAAGTGTA

>TN2514

CATTATTTTAAGCAAATACAGATCAAATAAGTAAACCTGTAAGTACTATGA

>TN2515

ATGGCCATACCGGTTACCCAAACAAAAATTAAGGAAGTTCTTTCTTCATGC

>TN2516

TGTACATTCTCACAATTATATGAGGAATTGTTTTGTTTACTTGCCTGCATG

>TN2517

ACCAATTTTCTACTTAAAACTATATAAACAGTTCTGGTAATGAGGTAAGAG

>TN2518

AAGACTCCATCTCAAAAATAAAAAAAAAAAGAAATTATTTTTGGCTGGGCA

>TN2519

TTAAACAGACAAGCAAGATGTTACAAAGCAATGCCTACAATAAAAACAGTC

>TN2520

GAAGTCATAGCTGGGCTCCATGTTGAAACTAAAGAAAAGAATAGTAATTAT

>TN2521

CAAAAAGTTAAATGTAGTATGAATCAAAATATTAATAAAATAGCAATAGTA

>TN2522

TTCAAAATATTGGAATAGTCAGTAGAATTACTGACTATTCTTACTTTTATT

>TN2523

ATGTCTAAGATCTTGATATGACCAGAATAAACTGATAAAGTAGAAGTATTA

>TN2524

TATCTTGAAACCAGAGGTGCCTCCTAATTTTAAGGAAGACGCTACTAGAAT

>TN2525

GGTATGTAAACACATTCAGGCCTTGAATACTGACATAAAGATCTGCAGAAA

>TN2526

CTTGGATAGCTGAAAGGTCAATAGGAAAGTCTACAGAATGACTCACCTTCA

>TN2527

TAAACAACACATTCATTTAGTTCAAAAGTCAAAGTCTAAAGTTAGAAAACA

>TN2528

CCTCCACAAGCAGCTGTCACAGAACAAAAGTTACAGAGAAAAAGTAAGGAC

>TN2529

CACACATGCACTAAAAAATATGTAGAAAAGAAAATGTCTTAATTGCTGATT

>TN2530

TCAAACAGCCAATAAGCATATGAAAAAAAAAAGTTCGACCTTATTATTAAT

>TN2531

CACGGAAGGAGGTTGCAGTGAGTCGAAATCATGCCCCTGCACTCCAGCCTC

>TN2532

GCTAATTATAATTAACTCATGCCCTAATGGGCAAGCTTCTTACAGAGGACA

>TN2533

TAAACCTCATTCCTAAAAAACAGAAAATTACAAAGTAATAAAAATATAATG

>TN2534

AAGTAACTAAGAGATGATCTAACAGAAGATCGATTTGTTTATAGATTCATT

>TN2535

CACTTTCTCTCTTCACACATCAATTAAATTTTACATTTTTCAGACAGATTA

>TN2536

CTAATAAGTCATTTGTCTACACATGAACATACGAAAATAGTTTCTTTTCCC

>TN2537

CACTTAAGCACACTTATCATCAGCTAACAAATCAGTAAAAATTAGACTAAT

>TN2538

TATAAATTTTCAGAGTCCTCACTGTAATACTGCAAACGGAAGCCACTCAAT

>TN2539

GATGGCCTGGATACAGTTTTGTATGAATTGAAAAACTAAACGGAATAAGAG

>TN2540

GTCTGGAATGAGACTGGGGCCTAAGAAAAAGGAACATCTATACAGGAGTTT

>TN2541

AAGGTTTCCTTTTGTGAGTTTATATAATGGTTTTGTTAGGATGGCAAAACC

>TN2542

AAAGCAGGTTTGAGATCTAGAACAGAATAATGGGTTGTGGAGGGAGGTATC

>TN2543

ATATAAGAGGTCAGGGCGCAGAAATAAGGGATCGGGGTACAGAGATAAGAG

>TN2544

ACCACCAAACAGGCTTTGTGTGAGCAATAAAGCTGTTTATTTCACCTGGGT

>TN2545

CCTGTCTTCTGAGTTGCTCAAATCAAAACCTTAGTAGTTGTCCTTGAGTCC

>TN2546

GGCACAGAATGAAATGTTACACTAAAAAAACAAAACCAACAACAAAAAACC

>TN2547

GCACCCGGCCTGTGAGATTTAAGAAAATAAAAGCAAGTAACTCAAATGATC

>TN2548

TTCTGGGTTGAAAGTTCTTTTCTTTAAGAATGTGGAATATTGGCCCCCACT

>TN2549

AGAGAAAATAAAATATTTGTAAGAGAATTTTCATTACTATTCATTCATTTA

>TN2550

ATGAGAATGAACAGGGTAAACAGACAACCTAAAGAAAGGGAGAAAATTTTT

>TN2551

TGAGTATTACACAAAACAAAATGAGAACCTCGACCTACCTCAAGAGTTAGG

>TN2552

AACAACAACAACAAACAAATACCAAAAAAAACCTTTCACTGTAGAGGAAAC

>TN2553

ACAGGTGAGAACTATAAAACATGAAAAGATGCTCTAAATTAGTAGCCATTA

>TN2554

AAAATTTCTACTATATATGTTTTCTAAATGATGTAAATACCATAGACAATT

>TN2555

ACCCCAAAGTAAAACAAGTTCAATTAAAAAAGTAAAGGTTATGTATGTCTG

>TN2556

AAAGATGAGGCTAAACCCAGGCCAGAAACAGTGCCTCTGTTTTGCCTACTG

>TN2557

CCAATAACTAAAAAATAAGAATCAGAAAATAAGCACAGACACATAACGAAG

>TN2558

AAATAATGAAAAAACAAGGGAAGACAAAATGCTACTAAGTGTCAAATGTGA

>TN2559

CATCTCCTTGGAGGCCACAAGCAGGAAATTCAGCACTTGGCCAGGGAAAGG

>TN2560

AAGAAGCAAGAAGCCAGAGAGAGGGAAGGGTGAAAATCAGGAGAGAAAGAG

>TN2561

CATGGGGTACTCAGGGCACAATAGGAAGAGACGGGATATTTGTAGGTGAAT

>TN2562

GTATGAACTGATATCTGAATGAATGAATGAATGAATGAGTGAATATCGAAT

>TN2563

GTTAGAGAAAATGAATCAGAGCTAAAATCCTGTCATCCCCCAGTATACAAG

>TN2564

TGTTTGTCTGTTGTTGGTGTATAAGAATGCTTGTGATTTTTGTACATTGAT

>TN2565

TATGAAAGGTTGTTGAATTTTGTCAAAGGCCTTTTCTGCGTCTATTGAGAT

>TN2566

TGCTGGGAGAACCACTGCTCTCTTCAAAGCTCAGATGGAAATGCAGAAATC

>TN2567

GGTATTCATTATGCAAAGTCATACTAATATTTTCAACATTTATGCACGTGG

>TN2568

CTGTATTAATAAAATGGTAACCCACAATGGGCTAAGCACTATTATAGAAGT

>TN2569

AAGGGAAAAAATATATGAATCAGCCAAAGAGCCAATGATAGAGTGACCCTT

>TN2570

GGAATAAGGAGGAACAAGAGAGAATAATAAGATGAAAAACAACTGAAGAAA

>TN2571

GAGTTTCCAGAAGAGAGAGTCAAATAAGTGTTTAAAAAGCTCCAATGTAAC

>TN2572

TATGATTCAAACTAGTACACATGGAAAACTGAGTAAAAAATAGTAATTGTC

>TN2573

AAGAGAGAATCATGAATATATAGAAAAAAAAGAAGTGATGGCCAGGTTTCA

>TN2574

GAAAAAAAATTCACGGACCGTCCTGAACCGGGAAGTTGCAAGCAGAGAATC

>TN2575

GGAATGTGTGGTGTATTTTTAAAATAATCAAGATAAATCCTTAAATATAGA

>TN2576

GTGTCTTTATAGCAGCATGATTTATAATCCTTTGGGTATATACCCAGTAAT

>TN2577

AATCTCTCAGCATTTGCTTGTGTGTAAAGTATTTTATTTCTCCTTCACTTA

>TN2578

TGTCTTCAACAAAGAAATAGTTTATAAACTATAGGACTGGATGAAATCTTC

>TN2579

TCACATATGTTTGCCATTAAGCAGAAAGCATCCTAGAGCTGGAAATAATTC

>TN2580

AAGAGTGTTTCTGAAATTCATATGGAAGTGCAGAAGGCCAAGAATAGTCAC

>TN2581

TGGAATTAAAAACCTGTGTTTATCAAAAGAAATCATTTCAGTATAAAAAAA

>TN2582

TGTTGACCAGGCTGGAGTGCAATGGAATGATCGTGGCTCACTGCAACCTCT

>TN2583

ATATGCTAATGAGAAGCTTACACAGAAAAACAAACATGCACCCTGGTACCA

>TN2584

ATGGTAGCATTACTTACAACAGTGAAAAAATTAAAAACAACCTGAATATTC

>TN2585

ATCCGGTAACTAAATAGGATGAGGCAAAATTACAAATTTGGATGTAAACAT

>TN2586

TTGGCCATGTTTATTAACTCAAAATAAATGTTCAGAATAACATGGCGTAAA

>TN2587

GACTACTTGTCACATCATTATGTACAACATGAAAAATTAGAAATGGCCAGG

>TN2588

CAACAGAGTGAGACTCTGTCTCAAAAAAAAAAAAAAAAAAAAAAAGAGAAA

>TN2589

AGAAGGAAGGTAACTGATGAAGAGAAATGATGAGATCAGGGAACAACACAG

>TN2590

ATGGGAATTCATAATGGTGGATAAGAAGAAAGGAAACATAAAGAGATGGAG

>TN2591

GAGGGAGACAGTAACACAAATAGGGAATGATGACAATAATGATGAAATAAT

>TN2592

TTGTATTGTCTATGAAAACTAAGGGAAGGGTCTATAGTGATGTTGATAGGA

>TN2593

TAAAAATATTACTTTTTGTTGGTTTAAATAACTCATCATCATAACTCAGAA

>TN2594

CTTTACCTTTTCTTCTTTAGTTCAGAAACTTTAACCATGGATATTTGGTGT

>TN2595

ACCACTATCTCTGCTACAAGAATAGAAGGCATATCTCTTTTCTCAAATCAC

>TN2596

TGGAGTCCACCTCCCTATTAATAACAAACAATCTTCCAAAATCTTGCGCTA

>TN2597

GTATTGAATACTGTACAGGAAATGAAAAACAGATGGTTGCATGGGTGCCCA

>TN2598

TTATTTTAAAATTAGTTTAAGAAGAAACATTTTCCTCTACCCTAAAACTTG

>TN2599

GCAATCAATCATTAAAAAGTCAGGAAACAACAGGTGCTGGAGAGGATGTGG

>TN2600

AATAGCAAAGATTTGGAACCAACCCAAATGTCCAACAATGATAGACTGGAT

>TN2601

TCTCAGCAAACTATCACAAGGACAAAAAACCAAACACCTCATGTTCTCACT

>TN2602

TGATATATTTTCTCATGAGCAACTGAAAAAAGCTTGAAGTATTTACAAAAA

>TN2603

CACGATGAATGCTATGGTTTGCGTGAAGGTTGAGAGGGACAAAGGAAACTA

>TN2604

GGTTCCTTCTGCAGGGTTTCTGTGGAAGCCAGATCCGAGCACAGGGCAGCC

>TN2605

TCCCCCATGCTGTAGCGATTTTTACAAGAAAAACTAAAGGATTTATGAAAA

>TN2606

CAGCACAGATTTCCAGCAGGAGCGGAACATTTTCCTTCACCTCCTGATGAA

>TN2607

ACTCACACCATTTTTAGAAGGAGTTAAAAGGGAAACTTGCCTAAAACCATC

>TN2608

TCATTGTTAAAATAGACACACACCCAACCCCAGAGACCCAGGTGGGGGAGT

>TN2609

TCCTACAAATGAACCTTGCAAATGCAATGCATCCATGACTGACTAATACAA

>TN2610

AAAAACAAAAACAAAACAAAAACCCAAAAATGAAAGTAAATAACAATGTGA

>TN2611

TGCTGTTGGCATGGAATGGAGGTGGAAGATATGTAGTTGAGGGAGAGAGAA

>TN2612

AATAAAATATTTCAAATATAAAAGAAAAGCAACAGTGCCGGGCGCGGTGGC

>TN2613

AAGCTGAAATATTTCCTGAAACTTTAAAAGTACATGTAAGCAATGAATTGT

>TN2614

TCTGAGATGGAAGGAAAACATTCCTAAATAGGAAACTCTGACGGGCAGATG

>TN2615

AATTTACAAACTACACAGATACTGCAATGCCAGGAGTAGATATTAGAGCAG

>TN2616

CAACTCTTGTAATAATGCTCCCCTGAAAAAAGTGAGTTAATTCTAGGAGAT

>TN2617

AGAGCAGAAGGTAGAAACCTGATCCAAGGCTTCTCTGTTCTCAAAAGTCTG

>TN2618

TCACACATCTGTGTAAGGTGCGAAGAAGTACAAACAAGTGGACACTCATTG

>TN2619

TACAAAAGTCTTTTGAAAATAGCTGAAAGTGTCTTTACACAGTCTTAAGCA

>TN2620

GAAGCGGGGATGAAGACATCCAAGTAAGAAAACAAAATACATTGAACATCT

>TN2621

AAAACACAGTAGGCAGCCAGAAAAAAATCAATATTTAAAACTTAATATAAG

>TN2622

GATGCAGTTTTAAACAATACTTAAAAAGGCATATAGTTTACAGAAAGATAA

>TN2623

AGTTATAAAAGTAAAAAGGATTTATAATATTTTAGTCTTGAATATCTCCTC

>TN2624

ATCTCATCATGCAATAGTAATACAGAAGTGTGATGTTGCTTAAAATGCTCT

>TN2625

AATGTGAGAAGCACGTTTTTTTAAAAACAACTTTTTGTTATAATCAACTTT

>TN2626

CACTAAAGATAACTGATAAGCTTAAAAAAATCACAGAACATCTCATAATGT

>TN2627

TTATAATTCTGTAAAAATCATTTTAAAAGAGAAATTTATATGTATATATAC

>TN2628

TGTGAAGAAAAAAAATCTAAACCAAAAGAAATAATTTTACATGTTTTTATT

>TN2629

TGAATATACATGACCAAAACACTTAAATGTACAAAGGTAGGATTTCATTAC

>TN2630

TTTCAAAAAAAAAAAAAAAGAAAAGAAAAGAAAGGAAAGTATACAAACTTG

>TN2631

GCTTGTTAACTAGAAAAATTATTTTAATACATATACATTAAATGATATACG

>TN2632

AAGATTTTAAAGACGATTAACATAGAATCACATTTTTAAAAATTTAAATTA

>TN2633

TTTTATGATTTTATCAACATGACTGAAGTGGAGCATCACATGAAGGAACTA

>TN2634

AGTAGGTGAAAATATATAACCTCTCAATCTGCCTATTCAATACAGTTAAAT

>TN2635

AAGATAATGACTCTGGATGAAATAGAATAACATTTAATGGGTCTTAATCAT

>TN2636

GAAAATATATAGATACATGTTTTGGAAACAGACACAGATGATTTGATTCAT

>TN2637

ATGGTAAAATAAAACAAGTTATGCTAAACAAGTTATGCTAAGTGAACATGT

>TN2638

CCATCTCAAAAAAAAAAAAAAAAAAAAACAAACAAAAACATTCTTGCCAAA

>TN2639

ACCAACAAAACAAACTAACAACACCAATCTCTGGACTTCACAGTCACCCAG

>TN2640

GTAGAAACCAATGCAATCAGGTGACAACAGACCCAGAGGATGCCTCCCAAT

>TN2641

TGGATGTAGCCTCCACATAGAGCTTAAAATAATAAAGAGAGATGATTACAG

>TN2642

ACAACATAAAAGGCAGATGATATACAATATTGGCAGATGACACATGACTGT

>TN2643

GTGCCGTACTTAGGAAGTGGTCTATAAATAAGGACTTGAGAGAGACAAAGG

>TN2644

CAGTTATCATGAAGACTTTACATAAAAGTAATTCTGTCCACAAATCCTATA

>TN2645

GAACAAGAATATTAAGTTGCTCTCTAAACTCAAAGGAGGTCTAGATGATTA

>TN2646

TTGAAATATTAAGAAATAAAGAGTAAACTAATTGCCTGCAAGTATGGATGA

>TN2647

AAAGGGTAACCAATAATACTTTTTCAAATGCCAAGAACAACAAATTTGTCT

>TN2648

GAAAACATATTTGAATTGGGAAGGAAAGCATTGATTATATCTCATGGTGAC

>TN2649

TCTAACTTTATGGAGCTGAATATTAAAAGAATTGAGGTCAGAAAAGTCAGT

>TN2650

AGAAAATAGTCTTGGCTAAACCAGGAAGAAGTCGAATCCCTGAATAGACCA

>TN2651

GCTAGTTCCATATATGCAAATCAATAAACGTAATCCATCACTTAAACAGAA

>TN2652

ATCATGCTGAATGGGCAAAAGCTGGAAGCATTCCCTTTGAAAACTGGCAAA

>TN2653

CTGGCCAGGGTACTCAGGCAAGAGAAAGAAAGAAAACGCATTCAAATAGGA

>TN2654

ATTAAAGACTTAAACATAAGACCTAAAACCATAAAAACCCTAGAAGAAAAC

>TN2655

ATGCAGCCAACAAACATAAGAAAAAAAGCTCATCATCACTGGTCATTAGAG

>TN2656

GTGTTTGCATGGCACATAAACATTCAAGTAAAATTATTGATACAATTAACT

>TN2657

TTCAGTTACACATGAAGAAAACTTGAATATTTTAAGGGTTATCATTCTTAT

>TN2658

TGCAAATATTTTTAATATAGAACCCAAAAATATGACAAATATTTGCATCGT

>TN2659

TTACTAGAGTTCCAATTACAAAAATAAGACTCAGTAACATAAATTCAAATA

>TN2660

TATATATATCTATAATAGATATATAAATATCTCTATATAATATATAATAAT

>TN2661

AAAATTAAAAATTTCTATTCATCGAAATGATGCCAATAAGAGAAGGAGAAG

>TN2662

AACAACTCCAGAATAAAAGACTTACAATGTGTAGATCGAAGGAACTGTATC

>TN2663

ATAGTATAACGTTGAGCAATACAATAACACAAAATAATCAGGAATGTTATT

>TN2664

CATACACGGTGGTAGGTAAACTTCAAATGATCGGGAATGATACGATGATGG

>TN2665

TAGTCTCAGAAATAATAAGACCTGAAACATTATGCAAGTGAACCCAACAAT

>TN2666

CTTTTCCCACACAAAATTTACAGTGAAAAGAGACAAACTGCGGTAGAAATA

>TN2667

AGAGATGAGCAATATTCCTGTTTTTAAGAGGCATAAAGAGTTTGATATAAA

>TN2668

CCTCCTGAGACTTCCAACAAGGTGGAAAGAAGAGGGAATGCTAGACTCTCC

>TN2669

ATATATATATATATAGAAAAAAAGAAAAATGGCAAAGAGCTTTACTCTCTG

>TN2670

TTTTTATCATTTCCTGATTTTCCAGAAAGAGAAATAAGAAGAAGACATTTA

>TN2671

CAGCTACAGAAATACAGATGAAAAGAAAGGAAAAGTGTTCTAATATTTGTC

>TN2672

TCCTGATATTGGAATATTTTTATCTAAAAGTAGACAAAATCTTAAGATATA

>TN2673

TTATTATAAAATAATACAATTACTTAATCTCATCATTTCAGAAGCTAAAAC

>TN2674

CCATCTCTGACCTCCAAGGTGTCATAATTGACCTCTGTCTGAAATCTGCAA

>TN2675

GATCATGGATACACTGATTAGTGCAAATGCACATGATTACCTATTCCTGCA

>TN2676

CTTTGTGGATTTATAGATATCAAATAAAGTACGTGAAAATACTCCAAGGTG

>TN2677

TTACGTCAGGAAACAAATACAGAAAAAATAAAAGCAAAAATTTATGGAAAC

>TN2678

TCAGTGAACACGGCAGTGGAAATTAAAAACATGTTAATAATGGATAGCACT

>TN2679

ATGGCAGGTGGAAAAAGGTCTTAGGAAAGCAATCAAAGACGCTGACCCAGA

>TN2680

GACCTATAACTTATTTTAATTTGTTAAGGTTCACTTTGTGGTCAGTTATAT

>TN2681

AATTGATTTAAACAAAACACTTTTCAAGAAAAACAATAAATAAGAGTATTC

>TN2682

TCCTCTCTATATTTATTAGATAAGTAACCAGAACAGAAATATGAAGAAGAA

>TN2683

AAAGAAGAAAACAGAACAAGCTTATAAAGCATTCATATAATGTATATAATT

>TN2684

TGGATGGACAGATGGAAGGATGGATAATGGAAGGATATGGATGAATGGATG

>TN2685

TGCAACTTCACATACATATAAGCAAAATGGGAACATGTAAGTAAAATGAGA

>TN2686

AATGTTTTCTCCTTTCCTAATTTGTAAGTCGAATATTTCATTATCATATAG

>TN2687

TAGTTGGATAATATAAAGTATTTTGAAAAATTTACTTTTAAGAATGTGTAC

>TN2688

GAATATTTTTATCTCTACAACTTAGAAAGTAAATACGTGGCTTCTTTTTGC

>TN2689

TTAAATTTAATTTCAAATACCAGAAAAAGAATGTCATTTCAAATGAAGGGA

>TN2690

CAACTATGTGCTTTTTGCTATAAACAATACAAATAATATTCTTCATTCCCT

>TN2691

GGCAATGTTTGTAGAAGTGACTCAGAACTGAATTTCCAGATGGTTTGAAGG

>TN2692

CATTAGGGGTTGACAAGTAACTGTCAAAGTAGCACGGTCCAAACTCAGTAG

>TN2693

AAAGAAAAAAAAAAAAAAAAGAAGCAAACACTATGATTTGGTATCAATTCA

>TN2694

AAGACTGATGCACCTAAAGCAAAAAAAAAAAAAAAAAAAAAAAAAAATGCT

>TN2695

AAGGGGCCTACGGAAAATATTGTTTAAGGATCATTCCGAAGAGCTTACATA

>TN2696

AAACACTACAAGAGAAATAGAGAAAAAGAACTCTGAAGGTAGAAATAAATG

>TN2697

TGGATGTCAGAGAGCACTTACTGGGAAAGCAACAATTTTATTCAAGATATT

>TN2698

CAGACCATAGTAGGAAATGAGAAGGAAGAATGAGTCATTTTAAAGGATAGA

>TN2699

GTTCTCTAATTCTTAACTCAGCAGGAACTGAACTTTTTGCCTTATCAAGTA

>TN2700

CACTGTAAACTTCAAAAATAAAACTAACTCCACTGCCGTTTCCAAAATGAA

>TN2701

CAAAATTATTTGATAAAAGTCCTTAAAAGATACAACTACTTTATGATATTC

>TN2702

TAAAGATGGTACTTGTTCACGCTCCAATATATTAAAAATAATAAGTAAAAT

>TN2703

GTATACTACTGAAGAAATCTATTGTAAGACACAAACAGAAGACAGAAGACA

>TN2704

GAGAGCAAGATTCGGTCTCAAAAAAAAAAAAAAAAAGAAGAAAGAAAAAAG

>TN2705

CAGAGCATGTGAGGAGCTGTATTAGAATCAGGATTTCATTCTCAGTAATAT

>TN2706

TTTCAAGGCCAGCCTACTTCATCGCAAACCACAAAATGACACAAATAAAAT

>TN2707

TAATCATAAAAATTTAACAATATGTAATGATTCCACAATTCCTATATATAT

>TN2708

CATGGAAATGTAAAAGTAACTATTTAATTTAATCAGGCTAATAATAACCTA

>TN2709

TCTTAATGAAATTCAGAGAGGAAACAAGAGACTACTTTAAAAACCCTCTCT

>TN2710

TAACAATATTGCATGGTATATTTCAAAATAGCTAGAAGAGAGGATTTTCAA

>TN2711

TGGGAAACAATTTGGTAACTTTTTAAAAAGTTAAACATGTAGTTAATATAT

>TN2712

ACTGATATGTAGGACTATCTGCTATAAGGGAGATGTTATTACATCATTATT

>TN2713

GGTAGAGAACTGAATATGACTATGGAAAAATAAAACACCTTTTAAATAAAT

>TN2714

AGTTGTTACTCCTTCCCATTACTGAAAGTTAAAAATTGTTTTCAATATGGG

>TN2715

CGTATGTTTTATTATTGATCATAATAAACATCACACCAACATAGTGATTAC

>TN2716

AAATGCAATTGTGTGAATTTATCATAATACTGTTCAAGGGGAAAACTCATA

>TN2717

ATTTCTAAAATAGAGGTTTTTGTACAAATAAGAATTAAGTATCCATAGAAT

>TN2718

CATCACCACCAGTATACTGACGTTGAAACAGGTGAGATACAGAACATGTCC

>TN2719

AAAAAAAAAAAAAAACCAAGAAAAAAAAAAGAAAAAGAAAAAAATTGCCAA

>TN2720

GTGAAAGGTCTTCTGAACATATCCTAACTCTGTGACTCTGTGTGTGTATCT

>TN2721

GACACCAAGAGAAACATGTGATATGAAGACAGAGGTAGGAGTTCCAGCGGT

>TN2722

AAAATTTTAAACAAAAAATTTTAAAAAGTAAAAAAAAAAATACAAAATTAA

>TN2723

AATCATAACACAGTACACTTTTAATAATTCTTTTATCACCTCATCATGAAG

>TN2724

GGAACTAGAGGGGACGAGAGGAAACAAGGCCATGTAGCAAATGAGGAGTTC

>TN2725

CCGGATTGATAATTACATTTTTGACAAGAAGGTGAACAGTCTGGAAAGATA

>TN2726

GAGGATGAGGGAGAAAAAAATCGTGAATGCACTTTCTGAAGCACTCCTAGT

>TN2727

CCTTGGAAGAGGGACAGACTCTCAGAAATCGGAAACGTGTGCTGCATGGGC

>TN2728

TAGGATGAGGTTATCTAGACTTCGGAATCCTGACGTCGATGAAATGGAACC

>TN2729

AGCCACGGGCACTGATGAAAAGATGAAAAGAACTCTATGGCAAAGAAAAAT

>TN2730

TCAAGAAAAGAAAGCAGAGCTGAGGAAATGAGAGTCCTGATACCACTGGGA

>TN2731

AAAAGAGGGACACACATCTTTCTGAAACTGGTGGGAAGGAAGGAAGGAAGG

>TN2732

GAGCAGTGGTTTGTAGTTCTCCTCGAAGAGGTCCTTCACATCCCTTGTAAG

>TN2733

GGATTTTATTTCTCCTTCACTTAAGAAACTTAGTTTGGCTGGATATGAAAT

>TN2734

TCCCATCTATAACTTTTAAATCAGTAATGCAAAATATCATCCATTTCTAAG

>TN2735

ACAACACAATTCAGAAATTTTAACAAAGACTCTTGTTGAGATCATAAAAAC

>TN2736

TAATAATGGCTTATCTCACATTATTAAGAGGAATCTGTGCTTATAATTCAT

>TN2737

CAAACTCTCCAACCCTGTCAACAAAAAAGGGTGATGACTGGAAACTCAATA

>TN2738

GTATAAAAACTCAACTAAGACTTAAAAAAAAACTGGAGTGGTCCTAAGATT

>TN2739

GGTGAGAGATGAAGATGGAATCTGGAAACCACTTTACAGAATATTCTGTGG

>TN2740

GTTTGTTCTTTAGCATATTAAATGGAATGGCAGTCCTCAGGAAAGCCCGTT

>TN2741

ACATTACATTGTAATAATAGAAATAAAGTGCACAGTAAATGCAATGCACTT

>TN2742

TCATAGACTTAAGTCAAGCCCTTGGAAAAAAATGTTAAATCCACCTTAGAT

>TN2743

TCAACTCACTTTACTATAGACAGAGAAGCCATAAAAACGTGGGACCACTAC

>TN2744

AATAGCAAACATGTAATTGCTTATAAAAATATCCAGGGATCAAGTTTGCTT

>TN2745

GGGCACACACTGAAAATTTCTGGGGAAACTATGAGAAAATGTCGAGAGCTG

>TN2746

GTATACGCATAAAGGTCACTTTTATAAGTGATTTGCACTTTGTCTTCAACT

>TN2747

GAACTCAGTTACCAATGAAGGAACCAATAAGATATTTTAAAAACTATTTCA

>TN2748

TGTTACAACTGGGACAGTCCTGGAAAAAATATGGCACAGTTGTTCATGTGA

>TN2749

AAAGACAAAGCATCTCTATCACAACAAATTGTATGGGTTCATTCACAAAAG

>TN2750

AAAAGTGTAAGAAGAAAATCAATTCAACCAAAACAAATGTCCCTAATCAAA

>TN2751

CAAGCCAAAAAGCGCACAGATCGTGAATGTCTAACTCTATGAATTTTTACA

>TN2752

TGTTGTCGGCATTTTTCCACACTTTAACAGCTACGTGCATTTAACTAATGG

>TN2753

CATTCCCTAAGATAAGTGAATATATAAAAGAAAAATATGTATCTTAAGTCA

>TN2754

TACTAAAAACGTAATTGTCATGTAAAAAATTCTTACACATTGAAAAGACTT

>TN2755

TATTATAGTTATTTTAAAGCTGATTAACTTACAAAAATTTCATACAGTAAA

>TN2756

GGCCACTTTATCCCTAGTCTCTAGTAAATAACATAAACATTTTTAAAATAA

>TN2757

CAATGCAAATGTGTCAGTGTTCTTGAAAGCTTAATGACAACCATAAGATAC

>TN2758

CAACCAAAATACAAATGCAAAAACAAAACAAGAGATGGATAACACCTATCT

>TN2759

GGTGGCACAGAAGATGATGTGACTTAACATCAATCATTTACAATCGCTATT

>TN2760

TGATAATGGCACAAGAAGCTACCAGAAGACACTGCTAAAATGTGATAGGAG

>TN2761

CTTGAATGATGAAAGACAATCAACCAACCCCAACAGAAATGAATCAGAGGG

>TN2762

TGGATTTTAAAGCCAGGTCGCTTAAAAATGCTTCAGTGATCATTTACAAAT

>TN2763

GAATCAGTGAACTTGAGATCAATGGAATTCTCCTACACTAAACAACAGAGA

>TN2764

CCTGTGGGACAAAACAAAACAAAACAAATACACATAGAAAAAAGAATCCAA

>TN2765

CAGAAAAAACAACCAAAAAGAAACAAAATGTTACCTACAGGATAAAACTAC

>TN2766

TTCTTCTTGAAAAGAAATGAAGGAGAAAAGAAAAGAAACTTTTACCTGTGT

>TN2767

AATCAGTTATTAAAAGGCAGTAGAAAAATCTCTAAACATATGGAAGTTAAA

>TN2768

AAAGAAATACATAGAATGAAGTTAAAATAAAAACACAACATATAAAAATAT

>TN2769

AAAACTCAACCAAGATAAAACAGACAATCTAAATAGTCCTTTATTATAGAA

>TN2770

GCAAATATCCTCAACAAAATATTAGAAAACTGAATGCAACAATGTACAAAT

>TN2771

CAGATAGTAAATAAGCATCTGACAAAATGTTCAGTATCATTAGCCATCAGG

>TN2772

CAGTTTCCTACAAAACTAAACATACAACCAACATACAACTCAAGCAATTAC

>TN2773

GCTTCTAATTATATAACATTCTTGCAATGACAAATTTTTAGAGCTGGAGTG

>TN2774

TGGTGTATAAATATTTAGAATTCTTAACGTTTATCTACTTAGAATAATGGA

>TN2775

GCCTGACATTCTATAAATATGACCCAAGTTCCTTCAACCACCTCTCAGGGA

>TN2776

TCTCTTAAAATCGAGATGTAACTAGAATTCCATATTTCCCACAAAATGGAA

>TN2777

AGAAAAAAATTTTTTTTTACCTAGTAATTTGTCAACAAAAAAGAGTCATTA

>TN2778

GCTACCCGGGGCAAGATTCCTCACAAATGAAGAGTGTTAGGGAAACCAGCC

>TN2779

CAGACCAGCTCATCTGGCACCACAGAAACTCTCTTTCCCACAAAGAGGCAT

>TN2780

GTCGAAACAGATAACACATACTCCTAAATTATACTCATAATGTGTTCTCAT

>TN2781

AGTGCTTAACTAAATCAATATTAGCAAAAGAAATGTTGGTTATAGTTAATG

>TN2782

TACTCCACGCTGCTGATGCTCAGTAAAAACAGAAAATGCAGCCTTGGTCTT

>TN2783

GAGAGGTTCACGACCAGACACACAGAAATGGGGGTCCCTAACCACAGATGC

>TN2784

TATCTTAACTAACATGGATGAAGAGAAGTAAGAATGTGTCAGAGAGGATTT

>TN2785

TGAATTTCCTCATAAAAAACATGATAACAAGTTTATGTCTGGAAATTATTA

>TN2786

TAAGGGCCAATCATAAAACAACTAAAAATGTAATTAATGATAAGGAGACAA

>TN2787

ACAGTCACTTTGGAAGATACTTTGGAAGCTTCTTACATAACCAACTATATG

>TN2788

GACCGAGACTTCATCTCAAAAAAAAAAAAAAAAAGAAGTATTTCCACACAA

>TN2789

CCATGAGAAAACATGGGGGAAAACTAAATATATTTTACCAAGTGAAACAAG

>TN2790

TGATGGAGAAACAGTTAAAAAAAAAAAAAGTTTTGCTGCATAAGATGGGAG

>TN2791

AATAAGCTAGTGAGAATTTCAGGAGAATTTCTGTAACCCCTGAAATAAAGT

>TN2792

GTGACCGCTGAAACTTTGAAGGAGCAAAAGATGAAAAGAAGATGAGAGAAA

>TN2793

ATTATAGATCACTACATGAAAGCAAAAGATATTGAAGTTTCTTGAGAAATA

>TN2794

TGTAAATGAAATAAAGCTGTATGACAATAATAGGAATACGTCACCTCACAG

>TN2795

CTATTTTATGTGAGAAGGAAGAGAAAATATTTACAGATTTTTAAATGTCTC

>TN2796

AATACACTAAAAAGAATGATCTCCAAAAAGTTATGTAAACCAGAAAGGAAG

>TN2797

GCCCTTTGTCAGATGAGTAGGTTGCAAAAAGTTTCTCCCATTCTGTAGGTT

>TN2798

TTTCAGCTTCCTCATCTTTAAAATGAAGAAGGTAGGCTATTCTACCAAGAT

>TN2799

CTTGGAATGAACCCAAAGGCCCATCAATGATAGACCAAACAAAGAAAACGT

>TN2800

CGTTTTTGTTAGAAAGTCAAATTAAAAAAAAAAAAACGGTTGTTATATGAT

>TN2801

AAACACACAAAGGTTATAACCACAGAATAATTCAGGCCAGAACATAAGAAG

>TN2802

AACAACTACTTTCTGAACATGTAGGAAGTGTCAGTCATGACACTGGGCAAT

>TN2803

AATTAACTTCTCATAGCATATCTTGAAATAGTGTTTGGTTACAGCAGAAGG

>TN2804

TGCCAGTCAATGTTCTAAAGGTTTAAAATTAGTAAGTCTACTCAATTAACT

>TN2805

TCCACTAATATTGTCTCCAAATTTGAATATAACTGTCCTGGAAAGGGACAT

>TN2806

CAAGCAAAACTATAGTGGTGTTATTAAGTGTTTTAAGGAAGCTGATAAACA

>TN2807

CCAAAATGTTGTATGGTTATTAGCGAACTATGGACCAATGATTACTTATTT

>TN2808

AAAGTCCTTGAGGAATGTGGAGAAGAACAGTTCATTACATGCTCTGTTTGT

>TN2809

GAAGTTTCTTGATCATCTGAGAGGAAACACAGGCTCACAATTGAGACTACA

>TN2810

CTAATAATTATGATCCTCAAAAATAAACACTTCAACCATAAAAAGTATATC

>TN2811

TAGGAAACATCAACACCTCAAATACAACCTCTCCCAGCCTGAACCCCACTT

>TN2812

GGAAAACCATGTGCCCATCAAAGTCAAAGCCCTCTCTGGAGCATCCATTCC

>TN2813

CACACCGATACTGATTCTGCACCTTAATATGTGGCATTCGGGAACTACAAA

>TN2814

CATACTCACATATGCACACACATGCAACCCAGACAATATGCACACACATAC

>TN2815

ATGTGCACACACACAGACCCATGCAAAAACACATGTACACACATGCACCAA

>TN2816

AATAATTTACCCAAATTAAACAATTAATAGCCTGGGTATATGTAGCTTCAA

>TN2817

CCTTCCTGAACCTTCTATAGAATATAAATTGTCTTAAAATATAAAGCTACC

>TN2818

ATGCCAGAAGGAGCCCAGAACCCAGAAGAAGACAACATATGAGCCCTCGGA

>TN2819

CATGGTTAACTCCCCTGGTAGAATCAAAGTTTGATTTTGTTGCACAAATAT

>TN2820

TACATTTATACTGACATATTTTCAGAAGCCCAAAATATTATCAATCTCAAC

>TN2821

AAATATCCAATTGTGTAGTAAAGACAATACATACGCTACTGATTTAGGTCC

>TN2822

ACACTTAAGTGTACTAAATCAATTCAAGAAAGAGTAACTCCCTCTTAAAAT

>TN2823

TATCTGATTCACATTTGAAGTCATTAAAGCACACAGCACAATGACCTACAG

>TN2824

CCGAGATTGTACAGCAGATCTCTAGAACTTATTCATCTTACATAAGAGAAC

>TN2825

CCAATCACATGAACTCTCTAAGGATAAGAGGGCACATGAAACAAAATTTTG

>TN2826

CCTGAGTCCAGAGGACAGAAATGGAAATAAAAGTGTGAACCCTGACACCTG

>TN2827

GTTCACACCTTCCCACATGCTTTGAAAATTCCCAAGGGCTGGATTCCTTGC

>TN2828

TGTGGGATAACAGTGTTTAAAATTAAAAACATATCACACATTGAAAACAAG

>TN2829

TCTTTTACACTGTTGGTGGGAATGTAACTTAGTTCAACCATTGTGGAGGAC

>TN2830

ACAAGTTGACAACCAGTGCAAAGGGAACCTGCTTCATGCATCGTGAGCAAA

>TN2831

GATGGCTGGATCCCCAACATGATGGAAAATGAACAGAGTGTATGGGTCTGC

>TN2832

TGATGAATGTGGGGTATGAGAACAAAAGAAGGATTAAATATAAGTATAATA

>TN2833

ATTTTCAGCCTAAATAACTGAAAAAAAAAAAGAATTGCCATTTTCTAAGAA

>TN2834

ACAAATGAATCCTGTCTTTACCATGAAGGCTACTTATCATGGGCTCTCCAC

>TN2835

TCAGGAGATCGAGACCATCTTGGCAAACATGATGAAATCCCCATCTCTACT

>TN2836

CTCCTCACAGTACTGAGCAATTGTCAAAAGTTTTGAATAATACTGCAAAAT

>TN2837

ATTACTGAAATGAAAGGGAAGGTGAAATATTGGAAAAACGATGTTGCAAGG

>TN2838

TCCTGGACAAGCTCTGGGCTTGGCAAAGATGAATACCTTGTCTTACACAAG

>TN2839

GAAAATAAAATGATTAAAGACATGCAAACAAAGTTTTTTAAAAGGAATTTC

>TN2840

AAATTCATTCATGGATGAATGGATTAATGCATTAATTTGTTAATGAATTAC

>TN2841

TAGCATGATGGGAGAAATCTAGGAGAAATCTAGCTAGCCCCCTTCAACACT

>TN2842

TTCATATTGAAAATCATTCTAAGTAAAAGGCAAGAAAAAAGAAAGCAAAGC

>TN2843

TATTTTCCACTTTGCACCTCCTTGAAAAGAAGCTTCATGGCCAACCAGGCT

>TN2844

CAGAAAACATTGCTAAAGAGACTCAAAAGTTTTTTTGAATTGGATTAGCCA

>TN2845

CTGTCAATTACTGGTAGTTTTGCAGAAGAATACTGAAGAGTTCAATATATT

>TN2846

AAATTGTGGACAAGGAGAAAAAAAAAAAGAAAGGAAAGACCTACTCTGAAT

>TN2847

ATGTTTTATTAAGTGAAAAATAGGCAAATAATTTCACAGAATATTTATAAT

>TN2848

CCTTTTATACAAGTGAAACATACACAAAAAGAAGGTAGACAAGAAGAAGTT

>TN2849

TTTAATGGATATAGCCATTTAAGGCAAAAAAGATTGTGTTTTTCTTTATAA

>TN2850

GGGTGGAAGGGAAGCAAGGCCACATAAATGTGGAATTGAGATAATTGGTAC

>TN2851

ACAAACTTATTTTACATATAAATTTAAAGTAAATATGGAAGTACATGTACA

>TN2852

AAGCCAAAATTATCAAACAAAATACAAACTTCTTTTTCACACGCACACACA

>TN2853

CTCTACTGTAAGCACCTTTAAATGAAAAGTATGACTTAACATTTAACTTCT

>TN2854

GTCTCATTATGCACCATTAAGAAACAAACAAACACTCTTTAAAAAGAGGCA

>TN2855

TAGGCATGGTCTGAACAGAGGTTTAAAGGAGTAAGCTGATGGGAAGAAATA

>TN2856

CAAAGAAATTCAAGGTCCAAAGAAGAAATTATATTAATAGAAGGATAATAT

>TN2857

CGGCAGGCAGTGAATACACACATGAAAAAATGCCCAGCATCACTAATCATC

>TN2858

CAGTATGGAAGTTTCTCAAAGAACTAAAACTGGAACTCCCACTTCACTCAG

>TN2859

CAATTCTACTACTAGGTATCTACTCAAAGGAAAGGAAATCATTCTACGAAA

>TN2860

AGTAAAATTAAAAACAGAAAAAGAAAAAAAAGTAGTATCTATAAAGTTAGT

>TN2861

AAAAAATCACAAAGAGTTACATGTTAAACAACTGATTGGTTAAAAATAATA

>TN2862

TACCCATGAACTTAAAAGTTAAAAAAAAAACCAAACCATCCATTTAGTAAT

>TN2863

ATGCATAAAATAGGTAACGTATTAAAAGGTAAAAAAAAAAAAAAAAAAAAA

>TN2864

AAAAAAACAAAACAAAAACACAAGTAAAAACATTTTTTAACCTGGCCTGGC

>TN2865

GAACTAGACTCCGTCTCAAAAAAACAAAAACAGACAAACACACCAACATAT

>TN2866

ATACTAGAATTTTTTCCTTTTCCCTAACTTAAGCAACTTTTATTAAGAAAA

>TN2867

GATCAACGCCATAAGATTAATGATGAATAGCTAAGGAATGCTGGGCTTAAT

>TN2868

TGTAAAAACACAAATTGCTGTGACTAATTACATAACCCTGAGATATCTCTC

>TN2869

AGGAGAGGGAAAAGATGGAGGAGAGAAAGAGGAGAGGAGGCAAACAACAAA

>TN2870

ATAAAAATATAAATGAAGACATAAAAAGTAAATAAAAAACAACAAAAGGAA

>TN2871

AAGGGAAGGAAAGGGAAGGGAAGGAAAGGGAAGGGAAAGGAAGGAAAGGGA

>TN2872

TTATTTCCCCTCTCTGAATTTCTGGAACGTCCAACCTTCCTCCACATCCTC

>TN2873

TAAATATATAATAAAACAAACTTATAATTAAATACTTGAAAATTCATATGA

>TN2874

AAACAAAGAAAAAGAAAACTAAACAAAGAACAAATCAGTAAGATGTACTAA

>TN2875

GGATTGTCAAATGTGTCTACAATAAAACATATTTATACATATCATATAATA

>TN2876

AAGAGAGGATTAAATGTTTCAAAAAAAGAGAAGTTTGTATTGACTAAGAGA

>TN2877

CAGACCAGGTCAAACACACAAATTGAAGTATGCAAATTCTTCACATCCAAA

>TN2878

CACGGAGCTACTGAATACAACCTGGAAGCTCCCTGACTCTGCCCCTTTGTT

>TN2879

TTCAATTATTCTAGTATGTGTAGGAAAATAATAGAAACTAAAATGTGCTAT

>TN2880

TGAGATGGCTTAGTAAATAGTTAACAAATTGTTAATAAGTGTTTTTTTCTA

>TN2881

GAAAAATAATAGTTTCTTGGTAAAAAACTGAGTAACAAACCATTGAGTAGC

>TN2882

TGTGATGAACACTTTACGGAATCTAAAATCAGAACATTTCTCTTTTAGAAC

>TN2883

TGGCAACCCAACAGCTTTGAGAGGAAACTATATAAATAAGATGGATATTTT

>TN2884

AAATATAATTATTTTAATATTACATAATTTAGTGAAATCCATACCTATAAT

>TN2885

AAAAACGTATATAGCTTATGAGAAGAAACACATGAACTGTAGAAAACAATT

>TN2886

ATCCTATAAGAGTGCTGTGTGCATTAACCACAGGGGTTGGAGGCGGAGCCT

>TN2887

ATCAATACACATATTTTAGAAATATAATGTCAGCAGAGACTCTGGTTTTAA

>TN2888

AAATAATAATAATAATAATAATAATAATAATAATACAAATCATGGCTATTC

>TN2889

TGGGTAAAACATAACTGGGTTTCCGAAACTCAGTAGGTGTGTCATTGGTCT

>TN2890

CAATTAGTCAACGAAGTATGTGATTAACCAGCATAATTAAGTAACTACAGT

>TN2891

AGTAAAATTCACCGACATGCTTGATAAAGGTACTTGGATAATAACTACGAC

>TN2892

CCAGATTGTGAACCATAATGAAGATAATATATCACAGAGGCTAAGAACTTC

>TN2893

AACTAAGTTTCTTGCCTTCAGCAGGAAACTAACAAACACATGGACATATAA

>TN2894

GGGAGGCGGATACTCCAGAGAGGGGAAACCAAATGCAAGAATCCTAGGAGA

>TN2895

TTTCTCCCAGGTGACCTTCAGGTAAAAAGTATAAAGCTTCAGGAGTCAGGA

>TN2896

GAAGCAAATTGAGACGCTACTTAAAAAAGGGAATGGTGACATACTCAGGGT

>TN2897

AGGCAGAGTCTTGGCGATTACTAGTAACGTTCCAGAAAACTAAACCAAACA

>TN2898

CACCAAACAAGAGAGACCCTGCGAAAAAAGTGTTCTCTTCCAAAATATGAT

>TN2899

CAAGTGGGACACTTGAATTGACGAGAACAGGAAACCACAAAGAATCCTCTT

>TN2900

GTTTACATAAATCATTTTATGGCCAAAATTCAAAAACAAAACCTCCAAACC

>TN2901

CGTTAAAAAAGAAAAAAAAAAACCCAAATTATTTACAGAAGACTTCTTTAG

>TN2902

AGCCACAATCTCATATAGATAACATAATAGTTAAGAAAGAAAAAAATAACC

>TN2903

AAAAACAGACAAACGAGATGACATCAAACTAAAACCCTTACGCACAGTAAA

>TN2904

GACCTGAATAGACATTTCTTTAAAGAAGACATAAAAACGACCAACAGGTAT

>TN2905

TGTTGTTAGAAATGTAAAATATGTAAATTAGCATACTCATTATGGAAAATA

>TN2906

GTATAGGGGTTGCTGAAAAAATTAAAAATAGAACTACTACATGATCTAGAA

>TN2907

AGCCGAGGAGGAGACTCCACGTGGGAATAAATCAAGTTGAGGCAGAAACTA

>TN2908

CAGCCACACTTTATCAAAGGTTTAGAAATGTTGCTTGAATGAAAGTATTTG

>TN2909

AATAAAGTCAAAGCCCACAGTGTGGAATCGATGAGTTGCCCAAACAGCAAA

>TN2910

AATAAGCAAGACCAGGAAGGCTGGGAAGACCATTTGCAAGCAAGCCAGCCA

>TN2911

ACTAGGGGAGGGATAGCATTAGCAGAAATACCTAATGTAAATGATGAGTTG

>TN2912

CTGGGACCGACCGCCCCATAGGCTCAAGTGGGGACATCCTTTCCTTCCCAT

>TN2913

ACTGCAGGTGGCATCAGCAAGCTGAAAACTGACAAACCATTGCTTCCCGAA

>TN2914

CTCCCTAATGTTTCCAATTCGTTTAAAAACTCTTCAGAATCTCAAATGATT

>TN2915

AAGAAACAGCAGCTTTTTTATTGGGAATAATTATGTTCTTACAAGTTTACC

>TN2916

GAATAATGGCTGAATGCTTCCTTCGAAGCAGGCTCTGTACTGAACACTGGA

>TN2917

TCTATAAAGTATATAAATGATGACAAATAAAATAGTAAGCAAGAAAAGGAA

>TN2918

TCAAAGGAGCCAAAAAGCAGGTGGAAATACAGGAGGGGAAGAGCTAGAGGT

>TN2919

TCAGTGCCCTTGGACACATGGGCATAACCTGAGTGTGCAGTTTTATTGCTT

>TN2920

AACAGATGTTATTAGCATGAATAGTAACATGCACATACCAAGGACATACAT

>TN2921

CCTTTAGAGCCTAGGTTATGACTCTAATTTTAATTGTTAATCTATTCTCTG

>TN2922

GTGTTCGTATATCTAAGTATACCTAAACATAGAAAAGGAGCAGAAAAGATA

>TN2923

CAACAGAGCAAGAAGCCATCTCAAAAAAAAAAAAAAAAAAAGTAAAGGAAG

>TN2924

CAACACAGCGAGACCTTGTCTTTACAAAAATCTTTTAACAAATTAATTGAG

>TN2925

CCCTAAAAACTTAAAGTATAATAATAATAAAGTTAAATTAAAAAAAAAAAG

>TN2926

TATTTTTGTAAATAATGTATAGGACAATGTATAAATATTTGAAAGAACTCA

>TN2927

ACAGATTGAATATAGAAACATATATAACAATCCATATATTATAGACTAACA

>TN2928

GCAATTATGACACAAAAAATTAAACAGTGCAGACTGATATATAAATCAAAA

>TN2929

TTTCAAGTCATAGCTGAATATTTTCAAAAGAGTGACTTTGTAAAAACATGT

>TN2930

TCCAATGGCAAATTGATTCATTGTGATGGGATCAATTATTCCAAAGACTTC

>TN2931

TTGTCTTTATTTTGTTCCCATGCCTACCTTTTAGCCATAATACAACAGAAT

>TN2932

CAAATATTGGCCACTGGGAAAAAATATTCAAAGAAAGAAAGAATGTGAACG

>TN2933

AAAATGAGGAAACTAGAAAAACAAAAATGGCAGGACATTCTACGGGTGATT

>TN2934

TTACATGTTGCTATGTTTTATGGGAAAAAATACTTTACCTTTTAAAGAATC

>TN2935

TCATAGGAACACTTACGAAGGGAAAATAAATCTTGGGGACTCAAAATCACT

>TN2936

CATTCTATCCAAAGACACCCGTCTGATCACCTAGATAAATGCATACCTGAT

>TN2937

TGGACTGAACCAATGTACATCTTACACGTATTGATTGATCTCTCGTGTCTC

>TN2938

TCAGCATCTCCTGAGGAGGAATCACAGGTGCACATCCTCAAGATTGGCAAA

>TN2939

AATATGCCTACTTTTCAATTTTTCAATACTATCTTTACTAATTTAACACTG

>TN2940

CATGTTTCCTCCAGCCTCTGCCTATACCCAACTTTCATCCCAACTGTCCTG

>TN2941

CCCAAGACTTTGACACTGAACCTAAATCCTGATCCCTATCCTGGTCCCTAA

>TN2942

CCCTAACCCTATTATTATCTTTACAATCTATGTCTAATCTTACCCTCTAGT

>TN2943

CCTCTGAGTGGTATGGCTTCAGATAAGAAGTTCTAATACTTTGCAAGACAT

>TN2944

TTCAGGAGAAATCTCCAAGAAACAGAGTGCTATTCCACATACTTTTTTATC

>TN2945

CCTGTGTTAGGATGACACAGCACAGAGCTACCTCTCACCTGACCCATGATG

>TN2946

GAGTTAAATTCAAGTGTTTTCTTAAAGATAACAGTGAGCACGATATGTTAT

>TN2947

TGCTTAGCATTTGCGATTGTGATGGATGAACTAATTAAGAGCCCAAAATGA

>TN2948

GACCTGCATTTCTTCAACAACCCACATAGAGAGACTTTCCTGCACTTTTGA

>TN2949

ACACCTTTAACTCTCTACGATTTACAGGTTATTAAGTGGCGCTTACAATTC

>TN2950

AATACGATGTTCTACAACATTGCTTAACACAAGGGGAGACGCTCCTGACTT

>TN2951

AATGAAAAGAACCCTGGGGTGATAAAGTGAGTCAAAGGGGTACCAGGTGCA

>TN2952

TCACAGCAAAATAGATTCCTAAAAAATCCCTGGCCTAAGATGATACCCTTG

>TN2953

GCTGGATACGTTTGAATGTGCTGATAGTGGACATGGTAGAGTGAAGGTGGT

>TN2954

ATTTAAACATTTCTCCCTTATATTAATACAAATACTAAAATTACAAATACT

>TN2955

TTGAACGATGGATACGGCAGAAGAAAGCATGAGGATTTCACAGATTTAAGG

>TN2956

ATGTAATGTTTGCTGCAAAATTAATACATGCTAGAAACAGAAGCATCTGGG

>TN2957

TTCAGACATGTTGCATTTTAGTTGAAAGGTTGATATAATTTTTTTTAAAGA

>TN2958

ACACTTGCGGTGTTTGAAGTGACAAAGGCTGCTGTGACAAAAAAGCAGGGA

>TN2959

GTGACTACTTCCAGGAAGGGGCTACAAGAGGCAGTTGGAAATTCTATTTGC

>TN2960

AACTACCATGCATGGACCAGGTCTGACACAGTCTGCGTTTGTAAGTAAAGT

>TN2961

CATGGTATAATGGAAGACCTGAGTCATTAAGAGAGAGACCATATGGCTTGG

>TN2962

CTTGTTTTAAGAGATCTCTGTTTAGAATGCTACCTATTGCCTTCTGGATAG

>TN2963

TTCTGATGCTCTCCCTCTGCCTCTCAAGAACTTCCTGCCCCATCTCTCATG

>TN2964

ACAAATCCCTTCTTCATTCTTTAAGATGCAGCCCCTTTGCTCCTTCCTTAA

>TN2965

GAGCCACCCTGTGTGTGTCAGCTACAGCATTTCTTTGCATCTCTGTGTCAT

>TN2966

ATATCACCAAATCTGCCTAACCTTGAGTGAGTCACTGCATGACAACTTCAG

>TN2967

ATAATTAATTTGCAGATGTCCACAAAAAAGCCTATTAGAATTTTAATGGGA

>TN2968

CAGCTAATGCTAGAAGGGTAATTTTAGGAGAAACAAGAGATGCTTATAAGT

>TN2969

GAGACAGAGAGAGAGAGAGACAGACAGAGACAGAGAGAGACAGGGTTTCAC

>TN2970

AGGAGCTGCCCCATAATACCAGTAAAGTGAGAAGCAGAGATAAACTAGTCC

>TN2971

TAGACAGCCGACTCATGTTGGGGGCAGCCCACTCACAGTGGCCCTGACCCA

>TN2972

CCTGGTACTCTGCTCTTCTCTCTCCACCTTCGCTTTCCTGCAGTCTATGCA

>TN2973

TTGAAGTCATCTCCTTCTCCAGGAAATCGTATTGGGGGAGCTACAAATATC

>TN2974

CAGACCAGGTTACTCCTCTACTCTCATAGCATTTGGAGGAAAACCCAGAGT

>TN2975

GACCTCCCACCTCTCTCCCTCAGCTAGTCCTCGAACATGTCTGATGTGGTC

>TN2976

GTCTTTTCCTGACACATACATTGTAAATAATTTTCTGGCTTACATTTTGAC

>TN2977

GGGTCTTCTCCTCCAAGAGCACAGAAATATTTGCCAATACTGTCCTTAAAA

>TN2978

TCGGTCACAGTTTCATTTTTTATATATGCATTTTACTTCAATTGGGGCTTC

>TN2979

AAAGTGCTAAGTATGGTAGATTGCAAACATAAGTGGCCACATAATACTCCC

>TN2980

GTCTGATGTGCACAGAGGCTGTAGAATGTGCACTGGGGCTTGGTCTCTCTT

>TN2981

AACCTGCTGCTTCCTGGAGGAAGACAGTCACTCTGTCTCTGCCAACCCAGT

>TN2982

TGACCGCAGACATGCAGGTCTGCTCAGGTAAGACCAGCACAGTCCCTGCCC

>TN2983

AACACGGTGAAACCCCGTCTCTACTAAAAATACAAAAAAATTAGCCGGGTT

>TN2984

AGCACCCCCAAGGGCATCTTCTCAAAGTTGGATGTGTGCATTTTCCTGAGA

>TN2985

GCTTCATCAGGAAAAGCTTTGGATCACAATTCCCAGTGCTGAAGAAAAGGC

>TN2986

GAAAAGCCAAGCTGGGAACTGGTTTATGCAAACCTGCTTCCCATCTGGTTC

>TN2987

ACACTCACCTTGGTTGTGTTCTTTGATCAGCGCCTGTGACGCAGCTTCAGG

>TN2988

GAAAATTCAAAGTAATGGGGTTTACAGGTCATAGATAGATTCAAAGATTTT

>TN2989

TGTCCCCCACAAGAGACAGCTTGGCAGGGCCATTTCAAAGTATGTCAAAGA

>TN2990

ATTCCAAAGGGAGGAGGGTACAATGAGGCCTGTCCAGCCCCCACTCCTCCT

>TN2991

ACAGACACAGACGACCCTGAAGGTGAGACTGTCTGCTGGTGGGATGCTGGG

>TN2992

TTCTAGCTGCCTCCTCTGCCTGCCCAAACAGGACAGGCAGGAAAAACTGGC

>TN2993

ACTTTCCTTCCGGCTGCATGTGGAGAGGCTCGAGCGGGGCACAGTCCATGA

>TN2994

CAGACACAGACGTGATGGTGGAGACAGGGGCAGGAAGACAGAGCAGCTGAC

>TN2995

AACCCCCTACTTAATTACCTATAAAATGAGTCATTGCAAGGATGACAAAGA

>TN2996

CGCTCTCCTTGACCAAACTCCACTCAGGCTCCTTTGAGCCTTCTCCTTGAT

>TN2997

TATAAAACTACTGGAAGAAAACATTAGGGAAGTGCTCCAGGACATTGTTCT

>TN2998

AAGCTACCCACCTGACAAGGGATTCATAACCAGGAGCTCAAACAATAGCAA

>TN2999

CTCAGTTAAAATGGCTTTCGTCAAAAACGCAGGGAATAAGGGATGCTGGCG

>TN3000

AGGATGTGGAGAAAGGGGGACCCTCACACACTGTTGTGGGAACGTTGATTA

>TN3001

ACCCCATCTCTACTAAAAATATAAAAAATTAGCCAGGCGTGGTGGTGCGAC

>TN3002

AGGAGGTGGAGATTGCGGTGAGCGGAGAGCGCACCATTGCACTCCAGCCTG

>TN3003

GGTGACAGAGCAAGACTCCTCCTTAAAAAAATAAATAAATAAATAAAAGTT

>TN3004

AAATCCCGTCTCTACTAAAATACAAAAAATTAGCTGGGCATGGTGGTGCGC

>TN3005

TGGGCCTATAGTCAAAAGAAACAACATCAATATATCGTAAAGACATCTGCA

>TN3006

CTCTGAGTTCATCAGCGGATGATGGATAAACAGAACGTGGTGTGTATACAC

>TN3007

AGTGGAATATTATTCAGCCATACAGAGGAACGACAGCCTGTTATTTGTACA

>TN3008

AATTATATGAATGCATTAAACTATCACATGTACCCTGAAACTATGTACATC

>TN3009

TCAGCACTCTCCTAGTGGGCTCCTTAAAAATATTTTTGTTTGGGAGGACAA

>TN3010

AGTAGGAGGATTCCTTGAGCCCGGGAGCTTGAGGCTGCAGTGAGATAGTGC

>TN3011

CACTGCACTCCAGCCTGAGCAACAGAGAGATACACTGGCTCTAAATATAAA

>TN3012

TAGGAAAGCAGGTGAGAAAGGTGTAAGTTCCCAGGCTTGGGGGTCCTGGCC

>TN3013

AAGGAACTGAGGCTCTGCTACCAGCAGATGCTCCAAGCACATCCCACGGGG

>TN3014

AGGACCATGCACAACTCAGCTGGCCACAACCAGGAAAAGGGTGCCCAGGGC

>TN3015

GTGCCCTCTCAAGAACAGAAGCGGAACAGCCTAACGCTAATGTTTGGGAAG

>TN3016

TGCATCTGTGCACACACGCTCACACACACATATGTCCATCAATCCACTCAC

>TN3017

GCTGGATAGGTTGCCTTTGGCCCACATGGAGTCAGCCCCCTGCTCACGCCA

>TN3018

GAATCTGCAGAGCAGTGGCTTCCAAACTGTGTGATGCAACCCCAGCCAGAA

>TN3019

GGAGCTGGAAAGGTGGGCATTTGATATCATGAGGTATAAAGAGAGCTCCTA

>TN3020

GGGGTCCCAGACATCAACTAATAAAAGCACCTCAGAAGTTCATAGATGGGG

>TN3021

CTCGAACCCATGGCTACTACTCCCCACCCCCGTCCTCCTCCTCCTCTTTCC

>TN3022

ATTAAGTTTTTGTGATTATGAAAGTAGCTTACATTTGGTGTAGAAAATATG

>TN3023

TCTCTGTGCATGAATCTTTTTGTATATGTTGGAATATCCTTAAGATAAGGC

>TN3024

CCCAGAACTAAAAGTACCCTGTCAAAGGGTGAGCATTTCCGGTTCCCCTGC

>TN3025

TTTTATTGTGTAAAGTGGCCTATGAAATGTTCTGTTGTGTTTTTATGTTTC

>TN3026

GAAAGCTCCCAGTGCACCTGCACAAACACACCCACACATGCACCCATATCA

>TN3027

TATACACACGTGCAAACATGTTCACATTCACACTCACTCCTACATACTCGG

>TN3028

ACTCATACCCAGATCATACACACTTATGCACACATTCACACTCACTTATAC

>TN3029

ACACACCCAGATCATACACACATATACACACTTGTGCATACACATTCATGC

>TN3030

TCACTCCTACACACCCAGATCATATATACACTCGTGCGCACATGTTCACAT

>TN3031

TCATACACACCCAGATCATATATACACTCGTGCACACATGTTCACATTCAC

>TN3032

ACTCATACACAGCCCAAAATAATATACACATTAATGCACACAATACATATT

>TN3033

CATACCCCCAAATCATACGCACACTAGTGTATACATGTACACACTCACACA

>TN3034

AAATGTTTTATTTAAAAAAAAAAAAAAAGATGTCCAGAAGAGTTGCAAAGA

>TN3035

ACACTCACGCGGGTGCCGTCTCAGCAGCTCACGGTGTGGAAACTGCGACAC

>TN3036

CGTCTCAGCAGCTCACGGTGTGGAAACTGCGACACTCACGCGGGTGCCGTC

>TN3037

CAGCTCACGGTGTGGAAACTGCGACACTCACGCGGGTGCCGTCTCAGCAGC

>TN3038

TCACGGTGTGGAAACTGCGACACTCACGCGGGTGCCGTCTCAGCAGCTCAC

>TN3039

GCGACACTCACGCGGGTGCCGTCTCAGCAGCTCACGGTGTGGAAACTGCGA

>TN3040

CACGCGGGTGCCGTCTCAGCAGCTCACGTCCAGGACCCCAGGCTGCACTGG

>TN3041

CCCTCACGCCTCCTTAGTCCCCTGCACCTGTGACCCTTTCCTGGCCTGTCT

>TN3042

TGCTCTCAGGGGCCACGATGTCAACATGCCTCATCACTGGTGGTCTGGACC

>TN3043

ATGAGCTCCAACTCCTACAGGAAGGAGCATCAAAGAATTTGGGCACCGCGG

>TN3044

TAATTACTGAACATTTAGGGGAGACACTTTGACAGTATACAAATATCTTCT

>TN3045

TTAATTTATTCAATAATTTATTTATATGCTTACGAATCCATGGACATTCAT

>TN3046

ACATTTTGGGTTCCATGCCCTTTTGAAATGCCCACAGCTTTTTAAAAATTT

>TN3047

CTTGGCTCCTTTTATTGAAGATGAAATTTAGAAACTAACATCTGGGCATGG

>TN3048

ATATCTGACTATAACTGACCACCTCAGGGTCCATTCTGATCTGTATATATG

>TN3049

TCAGGGTCCATTCTGATCTGTATATATGTATCATGTAAACATGATTTCCTA

>TN3050

CTGGCATATCTGACTATAACTGACCACCTCAGGGTTCATTCCGATCTGTAT

>TN3051

TGTATATATGTATCATGTAAACATGAGTTCCTACTGGCATATCTGACTATA

>TN3052

ACTGACCACCTCAGGGTCCATTCTGATCTGTATGTATGTATCATGTAAACA

>TN3053

CGAGTTCCTACTGGCATATCTGACTATAACTGACCACCTCAGGGTCCATTC

>TN3054

CGATCTGTATATAAGTATCATGTAAACACGAGTTCCTGCTGGCATATCTGA

>TN3055

CCATTCTGATCTGCATATATGTATAATATATATTATATATGGACCTCAGGG

>TN3056

ATAATATATATTATATATGGTCCTCAGGGTCCATTCTGATCTGTATATATG

>TN3057

TTAGGGTCCATTCTGATCTGTATATATGTATAATATATATTATATATGGAC

>TN3058

ATTGGAGTTTAGTGCTTAGATATGCAGAGTTATTTGATTCTTTCCAGAATC

>TN3059

TAATCAAAACACTGTTTTTGGACTTACCCAGGTCAGCTCCTTTCTGCCCAC

>TN3060

ACACAGAAGACTTTCATCCCTCAAAAATGGCCCCAGTTCGGCCCCTCGGTA

>TN3061

TCCCGATAATTGTGTCTTTCCATATACACAAAAGTGAAGTCTGAGGGTGAG

>TN3062

ATGGCTCTTTCCCTTTCCTGCCACAACTGAACTGAGTGCAGGTGATTTTCG

>TN3063

CTGCCTGCTGGCTTTATTCAGCTTCAACTTCTTGACTTTAAAGGTGGATGC

>TN3064

AGGAAATGTGTGTCTTGTGTCACACATGGAAATGTTGCTGAAATAAGTTAC

>TN3065

AGCAAAGCAGACCCTCACCCGACTGACCTCCTGGCTGTGACGATGTGTGTT

>TN3066

TCTATCCCACACAGGGAGGGTGTTTATGGTCTGAAGTGAGGCCTCTCATTA

>TN3067

CAATCCATTCCCACAAGCACACAGCACAGCTAAACCAGCTCCAAGGAGGGT

>TN3068

CCCTCTCTGGAATGACAGTCTCATGACCTACAGTCAAACAAGGGATATTGG

>TN3069

TGTGACCGGCCTTGGGGAAAAAGAGAGTCTGGTTTCTACAGCGCCTTCGGG

>TN3070

AGGGCACCCTCGAAGCAGGCAGCTCAGCTCACAGAGCTCCCCTCGGCCATG

>TN3071

TCCTCCACCTGCCCTTCCTTGGTCCAGCACCTCACCTGCACACACCTGTCT

>TN3072

GCGCAATACTTCCTTGTGCCTCCTAACCAGGATGGGCGACACCAGCCCATT

>TN3073

CACATTCTGGTCATGTCATCAGGCCAACTTGGCTGAGCTCTGCCCTCCTTA

>TN3074

AAAAAAAAAAAAAAGACCAGCCTGAAGCAGAGATTGGGTCCCAGCCTGGCT

>TN3075

CTGCCTGGCCCTCTGCTCCCGCTTCACCTCACAGACAGAACGCTGCCTTGT

>TN3076

CAACTTACATAATAATTAACTTAAAAGGAATCATAAGTTTAAACAGAAAAT

>TN3077

GGATATGGATGGGAAATAAGCTTCCATCAGCCACCAGGGAGATGCAAATTA

>TN3078

CAGCCACTAGGAAACCTTTTCATTCATTCCGGGATGGCTGAAATGTAAGCA

>TN3079

CGGAAAATGCTGGGTGCCCGCAAGAACGCGGAGCAGCAGGCACTCATTCCC

>TN3080

TAAACTCATAGAACTGTACACCAAAAAATAGCAGTTTTGCTGAATGTTAAT

>TN3081

AAAAAAAAAACCTCATTTCCTCCCCACAAAGCCACCTCATGAGCCTGGGTG

>TN3082

GACTCGAGGCTGGGAGCCAAGGGCCAACCACAGGACAGGCAGCAAGACCCG

>TN3083

GTAAAGTGCAAGATTTGGGGTAGAAAAATGACAAGTCAGAGGACTGGAAGG

>TN3084

CAAGTTTCCCTGCATCAATCCAGGCAGGCAGCCCCGAACAGTGCACTCCAA

>TN3085

GCAACCCCCGTGCTGAGAGTTCCCCAGGGTCACGACTACCCAGAGTCAGAG

>TN3086

CCCAAGGTCAGAGCTGTCCTGGGTCAGAGCTGCCCATGGTCAGAGCTGTCC

>TN3087

TCTCACTCCAGCTCGGGCAATACTCAACTACACGGACGTGGATGCTCTCAA

>TN3088

AGTTAATTCCATAAAGAGGAGGATGAGGGGACAGAAAGGCAGGAGGAAGAG

>TN3089

AGCTGTCAGTCAGAGCTCAGGCCAAACACTGGGGCTAATAGGGGTGAGAGC

>TN3090

TTCTGCTCCATCACGTGTATGGGGCAGGGGGCAGCAGAGCTGGGGGAGAAG

>TN3091

GAGTGAGAATGACAAGTTGCGTCTGATTGTCCTGGAGACCTGGCTGAGAAC

>TN3092

CTGCCCTTCTTTCTCTCTACAAATTAGAGGCCACATCCCTGGCCGCTGAAG

>TN3093

CCTTGTACCCTGACCCACTGTCTGAACCTGATGGAGTCTAAATGCAGTGAG

>TN3094

GATGCCCAGGCCTGGCGGCCGGCGCACGCGGGTTCTCTGTGGCCAGCAGGC

>TN3095

CTCTGTGGCCAGCAGGCGGCGCTGCAGGAGAGGAGATGCCCAGGCCTGGCG

>TN3096

GATGCCCAGGCCTGGCGGCCGGCGCACGCGGGTTCTCTGTGGCCAGCAGGC

>TN3097

CTCTGTGGCCAGCAGGCGGCGCTGCAGGAGAGCTCAGGAGCAGGGGCCTGG

>TN3098

GAGCTCAGGCCCACTCTGCACACCCAGCCCGCCACCTCCCCCGGCTCTCTC

>TN3099

TTCCTTCGTGCACATTCTGGGGCTCATGCTTCTGCTGTGGTCCCATTTAGC

>TN3100

TTCTCGGTGTGAGTTCATGGGTGTGATGGGGTGTGCTGTGTGAGAACGTGT

>TN3101

TTCTCGGTGTGAGTTCATGGGTGTGACGGGGCGTGTGCTGTGTGAGAACGT

>TN3102

GTGTGTGTAGTGTTCATATGTTCTCAGTGTGAGTTCATGTGTGTGACGGGG

>TN3103

TAGTGAGACCCTGTCTCTACAAAAAAATTTTAAAAATTAGCCGAATGTGGT

>TN3104

AAAATTTTTAAATCTTTAAAAATTAAAAATCTTAAATTTTTCTTTAAGATT

>TN3105

CTTAATGATTGACTTAATTACTTAGATGTCAGTCTGTATGTTTTTGGTGTC

>TN3106

TCAGGAGTTTGAAACCAGCCTGCCCAACATGGTGAAACCCCGTCTCTACTA

>TN3107

CTCCGTCTCAAAAAAGAAAAAAAAAATTACTTTTAAAACCAAGTACTAGCT

>TN3108

CATCTCTAAGTAATACTATTTAAGTAGGCAAAAGCCCCTGGAGGTCGCCTG

>TN3109

GTTCAAGAAAACAAATTATTTCCCTAATGGACAGTGTAGGTCAGGACTAGA

>TN3110

AGCAAACAAGGATCTTCTATCCCTCACACATCTCAACACTCAAGCAAAAAC

>TN3111

TCAGAAACATGGATGTCTTAGTCTGATAGGCATCCTCTGCTAATGCATTTT

>TN3112

ATCACCTCTCCACTTCCTCCATCCCAGAGTTAAATTAAAAATCCCATCTGC

>TN3113

TGAGGTGGGCAGGTCACCTGAGGTCAGGAGTTCAAGACCAGCCTGGTCAAC

>TN3114

ATGGTGAAACCCCGTCTCTATTAAAAACACAAAATATTAGCCAGGCATGGT

>TN3115

AGGTGAGCAGCTCCTCTCCCCACCAATGCGGTGAGATGCTGAGCCAGGCCC

>TN3116

TAACCTGCAGCTTTCTCCTTCGGGAACAAAGCGCAGCCTCTTAGCAGCTAG

>TN3117

CCGACAGAGGGAAATGGGTTTTCATAAAAGACGTTTCCTCACCTTCGGTTG

>TN3118

TCACCACAGTCTCTGTGCAGAGTCCAGAGTTCACAAATGAGATAAATGGAC

>TN3119

TTGTTTATTTTAAAGCAGATCCAGAAACCTTTCTTGTCTAACAGGAGAGCG

>TN3120

TGGCTGGCAACATGGCCGCCCCCACATATCCCCACGTGTGTAGAACATCAT

>TN3121

TCCTTCCTTCTTTCTTGTATATTAAACTTTTCGCTCCTTAAAACCACTCCA

>TN3122

ACTAACCACCAGCATACCCTGCTCCATCAGAACACTCATCCTCCTGAGCTC

>TN3123

GGCAGAGAGAAACTGCCTCCTGGTAACACAGTGTGCACCCCTGGATCAAGC

>TN3124

CACGCCTGAAGGCAGTTTATTCTGAACTTTTACAATAAGTGCTCTTTTTGT

>TN3125

TGATAAAAGGGGATACAAGACAAGTATCACAAATCCAGAAATGAGAAGGGC

>TN3126

AAGATGCAGGAGATCCCTGTGGGGAACTTCACAACAGCGTAGATTCCCACT

>TN3127

ATCTGGTGATCTGTGACATACAACGAGCCTGCAGATCACAGGCAATGATTT

>TN3128

AATAATTCTCCCTTCCACCGGGGGAAGGGCTGGGTCTCAGGAAAGGTCTAA

>TN3129

GGGACACACAGTGCGGGGATCAGTGAGACCCTACTCAGCAGGCCCAGATGA

>TN3130

ATGGAGAGTGGAGGAACACAGGGGCACCCTTGCAAGTGAAGACTAAGCTCT

>TN3131

GATTTTTTTTTTTTATCTTACCCAAATTCCCTATCTAAAGGGTCTGGGGAG

>TN3132

AAAGAATCTCTATTAACATAGCTAGATATTTTTCTTCCAGGCCCTCCCAAT

>TN3133

CGTAGGATACTAAGATGAAATAGAGACAGTTCCTGCCTTTGAAGGGTGGTG

>TN3134

TTCAGCCTGCCGAGAGTTCCCAGGAATTTCCTGGAGGATCAGTCCCCAAAC

>TN3135

GCCTCACTCCCTTGCTGAGACCAATAGCAATCCCTGATGATCTCGCCACAG

>TN3136

ACCTCACTTTTCAGTGTCTCTTCACACTGACTAGACGGTTGGCTAATACTT

>TN3137

CAGAGCTTGCAGAGCATCTTTATACACTTTTAATCCTCCTAACAACCGTGA

>TN3138

TTGAGTGATTTCTGTAAGGTCTCAGAGCCAATAGACACTGGTAATGAAATA

>TN3139

TTCTCCACCCAGACTGCCCAGGAGAATGTGGAATCTCCAGCAGTTCTGACT

>TN3140

TGTCAACAACTTGAGAGTGGCCTTGAGTTGCTGGGAGCACCCCAGACTGAC

>TN3141

AAGCAAACCTGGCTACCCCGATCTCAACCCTGTGAGATCCTAAACAGACAA

>TN3142

TCCGTGAGCATCTGGTGATGGATGGACACACACACATAAAGGTAGACTGTG

>TN3143

TTTACAAGACCACTGTTAGATGACCACTGACAAGAAGACTGTGAGCTGGTC

>TN3144

GTCTTTCCATATGCATCAGGAAGCCATGGAAAATTCACAGACTGGAGTGAC

>TN3145

TTTGGCAAAGGTGGGATTTAGGAAGACTGATCAAGGCACGGGATAGACCCA

>TN3146

CTTCCACCTTAGCACTAGTGGGCTAAATGATGACTGCCTTGCTGTCCCAAC

>TN3147

CAGACCACCACCAAGATCAGAGTTAATATTATATATCAAAGTCCTGCCATC

>TN3148

CAGAGGCAGGGTACTCTCTCCCCTGAATGCCTTGCTATTGATGCATCACCA

>TN3149

GTAAGGCATGCATTCATTGCACAGAAACTTAGGCATATGCCTACCAACATG

>TN3150

GAATGGATTCATGAGACCAGCCCTCACTCTCAGGACTGAGGGCATTGGTGC

>TN3151

GTACACATGTGAATGCTGATTTCTTAGCATGAATTTTAACTCAGCTGCTGC

>TN3152

GGCTGCCAGTAGCCTGACAGCTTCCAGCAGCTTTGTCTGCCACCGTGTCCA

>TN3153

CAGAAAGGGAGGGAAGGAAGGAGCAAGGAAGGAAGGAAGGAAGGAAGGGAG

>TN3154

AGCATCATTTTCAAAAAGTGAAAACATGATTCTTATATAAGTCTATAGGGA

>TN3155

GTGTGTATAAAGATCTGTTTACTTCACAAAAGAAAGAACCAGAGGATGGTA

>TN3156

TCCTGTGGAGCAATAAAATCTTTTTACAGGACAAAAATCATTTGTATCTCT

>TN3157

TTTTGCTCTTGTTGGCCAGGCTGGAATGCAGTGGCATGATCTCACCTCACT

>TN3158

CAAGAAGGGGATAGGCTGGAATGGAAGAAAAGAGTCCAAAGAGGAAGGAAC

>TN3159

TGCCTCTTTTCCACATTTGCCCAGCAGAGGAAATGTTTGGGCCGAATGACA

>TN3160

CTGTCGCCTAGGCTGGAGTACAGCCATGCTCCCAAGCCATGCTTCCTGTAT

>TN3161

GGCCTGTGGAACATAGAGTCAATTAAACATCTTTCTTTTTTTTTTTTTTTT

>TN3162

GTGATCTTGGCTCAATGCAACCTCCACCTCCAGGGTTCAAGCAATCCTCCT

>TN3163

GTGTGTGTGTGTGTGTGTTTAGTAGACACGGGGTTTCACCATGTTGGCCAG

>TN3164

TATGGTGAAGATTCAATACAATTAAATGATATAACATATGTAAAGGGCTTA

>TN3165

AAGAATCTGAGAGCTCCTGAGAGACAGAGACGGTGTCTTACTCATCTGCTC

>TN3166

CTGCTATTAGGCCCATCAGAATGTAAGCACAAATGCCTAGAGAATGACAAG

>TN3167

GCCTGGCTGCCTCTCTCCCTTCCTTATTTCCAAGATTGGTCACCTCCTGCT

>TN3168

CTGAAATAGCAATTGATTTTCAGACATGAGTTGATAGAACCATGATTTTCT

>TN3169

AATACTGTTCCAAAGAGGCTAAAGTACCTCTGAGGAGCAGTGCAGGCTAGG

>TN3170

CATTTTCTTTTCTAACCACTGCCCTAGAAGAATAACACCTCACATTCTCTG

>TN3171

GAGGTAGGCACAAAAATCCCCATTTACAAGTGAGAAAACCAAGGCAAAAGA

>TN3172

CAGCTAGTGTTTGAACCCAGGTCTGAAACAAATAGGATTGACAAGGGGAGG

>TN3173

CCTGGCTTAGCCTGGATCGTTTGAAATGGTCATCCATCCTTTGGCCCCAAT

>TN3174

ACCTAAACTAAGGTCTATGAACAATAAGATGATTTTCTTCAGTGGGACTTT

>TN3175

ATTCAAGAAAATTCTGGAGCATGAAAGCTATTAACGATAAACCCATTCACA

>TN3176

AGTATAGCTCTTTTCATTACACATGACAAACAAGGCTCAGAGAGGTTAAGT

>TN3177

AACTTGCTCAAGATCACAAAACTGCAGAGTGACAAAACTAGAATTTTGAAT

>TN3178

CTAAGCTCAAAGGGTCACCAAACCAAATTTGGGTCCACCCACCCAGCCTAT

>TN3179

TTGCAGGCAGCAAGCAAGGAGTATCAGGGAGCTAATCCTTAAGGCCTAATC

>TN3180

TTCAAAGATTTTCTGATTTGAGACCAGGTGCAGTGGTTCACACCTGTAATC

>TN3181

AGACCAGCCAGACCAACATGGCAAAACACCATCTCTACTAAAAATACAAAA

>TN3182

GTGACTTCCTCCAGGTCCCTCAGGAAGAAATTTAGAACAAAGAACAGTTAT

>TN3183

TCTTGAGGCTAGCCAGGTGGCTGGAATTTTCCTTGGAGGGACTCAAGAGTT

>TN3184

AGCTCTTAAGGACTATGCTATTGTTAGATTTTTCGTATTGGAGCAGTCCTT

>TN3185

AGAGCTGCTGAATTCTTTTACTCTCACTGCACTCATGCTAAAGTTTCTTTC

>TN3186

TGGGACAAGTGAATGTGACCAGGAAAGTCCCTCCCCTCTCAGAGGTTCTTT

>TN3187

GGAGTAATAAACACTTTGAAGAAAAATAAAGTGGAATAAGAGGGTAGGGTT

>TN3188

ACAGTATGTACAATATTACTGCAACAGACACTGAGACTGACTTAGCATTGT

>TN3189

GGGAGTCTGAGGCAGTCAGATCACAAGGTCAGGAGTTCAAGACCAGCTTGG

>TN3190

TTGCTTGAACAGAGACCCGGGAGGCAGAGGTTGCAGTGAGCCAAGATCGCA

>TN3191

AGCAGAAATGTTGAAAGTAAGAGCAAATAGATAGTTAACCACTCTTTAAGA

>TN3192

ACTGACCCTTCACTAAATTCTTCCTAGTTAACCCTTTGAGAATGAAACCAT

>TN3193

GGCAGGGAAGATTAGAGGGAAGCTTACAGTGGAATTCAGGGCTGAGGCTGC

>TN3194

TCTCTGTGGGGTTGGTCTCAGAGCCAGGTTACCTTGTCTTAGGTCCAGTGG

>TN3195

GTGGGAGAAGGTAAAGAGATGGGCAAATGATCTCTAAAGTCTCTCTGGCAC

>TN3196

GAAGCTCTCTTTATTTGCTTCTGCTAATTAAAAAATCAGAGCTAAAGATAC

>TN3197

CTTATGAAATCAACTCTAAAATGTTATCCATCATAAATCATGAAACACAAT

>TN3198

CACTCTCTTCCGTAGGTTGTACAATAACCTTTGGCGAGAAAAAATAAATGT

>TN3199

TCACCATATTGGCCCAGATGATCTCAATCTCTTGACCTCATGATCTGCCCA

>TN3200

AGTTCATTCTCCACATGGATGTCAGAGTAACTTTCTAAAATGAAAATCTGA

>TN3201

GCTTCTTACAGTGGTTTCAGGCAGCATCTGAAGACAGTAAATGCAGAAGCT

>TN3202

TTTTTTTTTGTATTTTTTGGTAGAGACTGTGTTTCACTGTGTTAGGCAGGA

>TN3203

ATAAACTAAATGTTTTCCAAAGGGAATAGGGCAAAACAAAAAGGACCTTGA

>TN3204

TGAGGGTTAAGATGTAAGAATGCTTATCAAGGTAAATGCTGTTCACACTGC

>TN3205

TATCGATACATGTCCGTTGCAGAAAATCCAGGTGAATCCAAAGAAGAAATA

>TN3206

AATGTCTTCCACAATCCCATAGCCCAGAGCTAACTAACCACTATAAAGAAC

>TN3207

CCAGCGTGGTTTTAACTAATGGATCAAAAGATGCTCATCAAAAGCTCTGAG

>TN3208

GGAGCCAAGCCCAGGAATCAGGATGACCAAGACATACTGGACAAGGAGGGA

>TN3209

TGAGGAAATCTACAAAATTAATTTCACAATACACTTTACAGGATAGGTGGA

>TN3210

CACCAAAGCCTCTGGTAGCCACCACAGGACGCCCAGAGCACGTTTTAAAGC

>TN3211

TGAACACCAAACTGCGGACTTCGGGAGTAAGTGAACTGACTGGTTTTTATT

>TN3212

AGTTGGTAGACTAATACCTAATAAAAGCAAAGCTGCCAACAACTGAAATTG

>TN3213

GGGAAATAATAAGAATGTTCAGTCCATAGCCCTTCATTATAAAAAGGTGAG

>TN3214

AGACCACATGGTGGCTTAATGCTGCATTGATTTGGCTATCAATTTGTTTTC

>TN3215

TTGTAAAATAGATCTTTTAAAGGAAATTTACTGTGATTTTTTTCTATTTAA

>TN3216

GAGCTTCCTCTCCAGTTGAGCATGTAAGAAAATTATACCAGGAGAATACAG

>TN3217

GTCACTTCCAGCAGAAGCTTTAAGAATCTGAGAGACATTCATACGTTTTCC

>TN3218

TGACTGACTAACTAATGTGGTCATTAATCTTCATAAAGAAAGGCTCTCTAC

>TN3219

GTCTATTTGTGTGTGTGCATGTGGTAGTGGGGAGGGAAGAAAAAAGGAGGG

>TN3220

ATATGGATAATGACTAATATTCAACACAGATATTCTCAGCTCAGAAGAGCA

>TN3221

GCCAGGCACCCAGATCCAGAGTTTCACATGATCATGAGTGTTGGTTAATAA

>TN3222

TATCAAATTATACTGAGACTCTTGCAGTCACACGGGCTGACATGTAAGCAT

>TN3223

ACTAGGAATAACCTCTGTACTTTGGACAGTAGACCTGCATAGCCCATTAGG

>TN3224

CCTCAATGAAGTCTTATGCAAGACCAGAAGCCAATTTGCCATTTTAAGGTG

>TN3225

TCAGGATGTTTCCTGGAGTGCAGGGAGCTGTCCGTGTTACTGAGCAGTTCT

>TN3226

ATTTTACACCTGGGCTGTTAACACCAGGCCAGGTCAAATTCAAAGGAGAGA

>TN3227

GCAGGTCACTTGAGGTCAGGAGTTCAAGACCAGCCTCGTCAACATGGCGAC

>TN3228

ACCCTGTCTCTACTAAAAATACAAAAATTAGCTGGGCCTGGTGGCGCATGC

>TN3229

GTGACACAGCAAGACTCTGTCAAAAAAAAAAAAAAAAGAAATCCAAATAAA

>TN3230

GTATAGATAAAGGTAAGGTATCTATACCTTATAGATAAAGAAATTGAAGCT

>TN3231

TATAGAGTTTAAGTAATGTTCCCAAAGCCTCGTGGCTAGTAATTCAAACCT

>TN3232

CAAATTGTTGAAAGGGAAGGCTAAGATGATTAATAAAATCAAGAGCCAGAT

>TN3233

GTGCAGCGGCTCACGCCTGTAATCCAAGCACTTAGGGAAGCCAAGGTGGGT

>TN3234

GTTTGATATATTCAGAATCAGGGAGATCTGTTGGGTGCAGTTCATTTGAAA

>TN3235

TAGCAGGAAATTGTTGCACTCTCAAAGGATTAAGCAGAAAGAGTTTAATGA

>TN3236
[truncated: 486 more chars]
